# Single-cell landscape analysis unravels molecular programming of the human B cell compartment in chronic GVHD

**DOI:** 10.1172/jci.insight.169732

**Published:** 2023-06-08

**Authors:** Jonathan C. Poe, Jiyuan Fang, Dadong Zhang, Marissa R. Lee, Rachel A. DiCioccio, Hsuan Su, Xiaodi Qin, Jennifer Y. Zhang, Jonathan Visentin, Sonali J. Bracken, Vincent T. Ho, Kathy S. Wang, Jeremy J. Rose, Steven Z. Pavletic, Frances T. Hakim, Wei Jia, Amy N. Suthers, Itaevia M. Curry-Chisolm, Mitchell E. Horwitz, David A. Rizzieri, William C. McManigle, Nelson J. Chao, Adela R. Cardones, Jichun Xie, Kouros Owzar, Stefanie Sarantopoulos

**Affiliations:** 1 Department of Medicine, Division of Hematologic Malignancies and Cellular Therapy, and; 2 Department of Biostatistics and Bioinformatics, Duke University Medical Center, Durham, North Carolina, USA.; 3 Duke Cancer Institute, Durham, North Carolina, USA.; 4 Department of Dermatology, Duke University Medical Center, Durham, North Carolina, USA.; 5 Department of Immunology and Immunogenetics, Bordeaux University Hospital, Bordeaux, France.; 6 UMR CNRS 5164 ImmunoConcEpT, Bordeaux University, Bordeaux, France.; 7 Department of Medicine, Division of Rheumatology and Immunology, Duke University Medical Center, Durham, North Carolina, USA.; 8 Division of Hematologic Malignancies and Department of Medical Oncology, Dana-Farber Cancer Institute, Boston, Massachusetts, USA.; 9 Experimental Transplantation and Immunology Branch, National Cancer Institute, NIH, Bethesda, Maryland, USA.; 10 Department of Immunology, Duke University Medical Center, Durham, North Carolina, USA.

**Keywords:** Immunology, Autoimmune diseases, Bone marrow transplantation, Tolerance

## Abstract

Alloreactivity can drive autoimmune syndromes. After allogeneic hematopoietic stem cell transplantation (allo-HCT), chronic graft-versus-host disease (cGVHD), a B cell–associated autoimmune-like syndrome, commonly occurs. Because donor-derived B cells continually develop under selective pressure from host alloantigens, aberrant B cell receptor (BCR) activation and IgG production can emerge and contribute to cGVHD pathobiology. To better understand molecular programing of B cells in allo-HCT, we performed scRNA-Seq analysis on high numbers of purified B cells from patients. An unsupervised analysis revealed 10 clusters, distinguishable by signature genes for maturation, activation, and memory. Within the memory B cell compartment, we found striking transcriptional differences in allo-HCT patients compared with healthy or infected individuals, including potentially pathogenic atypical B cells (ABCs) that were expanded in active cGVHD. To identify intrinsic alterations in potentially pathological B cells, we interrogated all clusters for differentially expressed genes (DEGs) in active cGVHD versus patients who never had signs of immune tolerance loss (no cGVHD). Active cGVHD DEGs occurred in both naive and BCR-activated B cell clusters. Remarkably, some DEGs occurred across most clusters, suggesting common molecular programs that may promote B cell plasticity. Our study of human allo-HCT and cGVHD provides understanding of altered B cell memory during chronic alloantigen stimulation.

## Graphical abstract

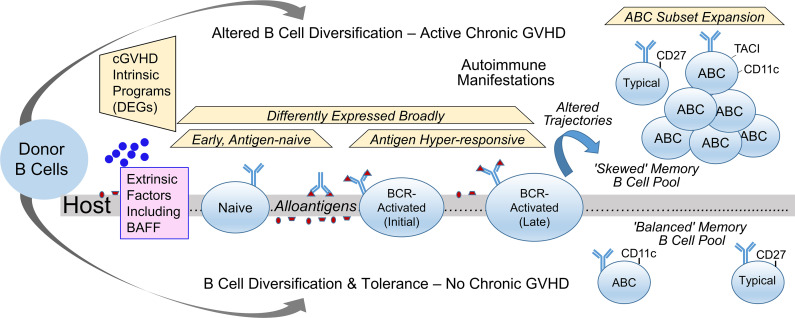

## Introduction

B cell tolerance checkpoints silence the high proportion of potentially self-reactive peripheral B cells that recirculate and can mediate autoimmunity (1). Chronic graft-versus-host disease (cGVHD) is a T cell–incited autoimmune-like syndrome some patients acquire months after allogeneic hematopoietic stem cell transplantation (allo-HCT). B cells also have a substantiated role in cGVHD (2, 3). Since blocking T cells potentially attenuates antitumor effects after allo-HCT, aberrant B cell activation, survival, and maturation pathways have become promising therapeutic targets for patients with cGVHD (4–6). B cell recovery and homeostasis are altered in patients with active cGVHD, whereby extrinsic factors including B cell–activating factor (BAFF) may contribute to a break in B cell tolerance (7–12).

B cells continually regenerate, yet following allo-HCT B cell reconstitution is often delayed (13). This “space” in the B cell compartment may allow excess BAFF to promote aberrant B cell activation, altering memory B cell development (11, 14, 15). Once donor-derived stem cells replace the recipient (host) immune system, T cells recognize alloantigens in a coordinated T cell–B cell response (16, 17). Follicular Th cells are required for B cell antihost reactivity and cGVHD development (18), and alloantigen and BAFF operate together to promote B cell tolerance loss (8). The peripheral B cell compartment in patients with active cGVHD manifestations becomes enriched for B cell receptor–stimulated (BCR-stimulated) and IgG-secreting populations (2, 3, 7, 9, 16, 19–21). Yet, how allo-HCT and cGVHD alter intrinsic B cell pathways at the molecular level remains largely unknown.

The advent of single-cell RNA-Seq (scRNA-Seq) enables identification of intrinsic B cell pathways that potentially underpin pathological functions. We used scRNA-Seq to delineate transcriptional programs within 10 peripheral B cell clusters resolved in allo-HCT patients and identified differentially expressed genes (DEGs) within these clusters associated with cGVHD. Some DEGs were widespread across these B cell populations, while others were more restricted. Homing and cell cycle genes including *GPR183* (*EBI2*) and *CKS2*, respectively, were overexpressed in circulating transitional, activated, and memory subsets, suggesting potential roles in B cell dysfunction from the time B cells first emerge in the periphery, then after alloantigen BCR stimulation. In patients with active cGVHD, we found an expanded population of potentially pathogenic atypical/age-related B cells (ABCs), which are emerging as an important subset in both normal and pathogenic humoral immune responses (22–29), and known drivers of ABCs, including *TBX21* (*TBET*) and *ZEB2* (30, 31), that were among the increased DEGs. By comparing our allo-HCT scRNA-Seq data with a public data set on blood B cells from healthy donors (HDs) and non-HCT patient groups (HIV, malaria), we found signature gene profiles unique to allo-HCT within these ABC and other memory subsets. Thus, we hypothesize that scRNA-Seq will provide insight into B cell transcriptional programs when tolerance is maintained or lost after allo-HCT and identify potential molecular targets for future study in alleviating cGVHD and possibly other B cell–associated autoimmune diseases.

## Results

### The circulating B cell compartment after allo-HCT comprises transitional, naive, antigen-stimulated, and memory single-cell signature gene profiles.

**Figure 1:**
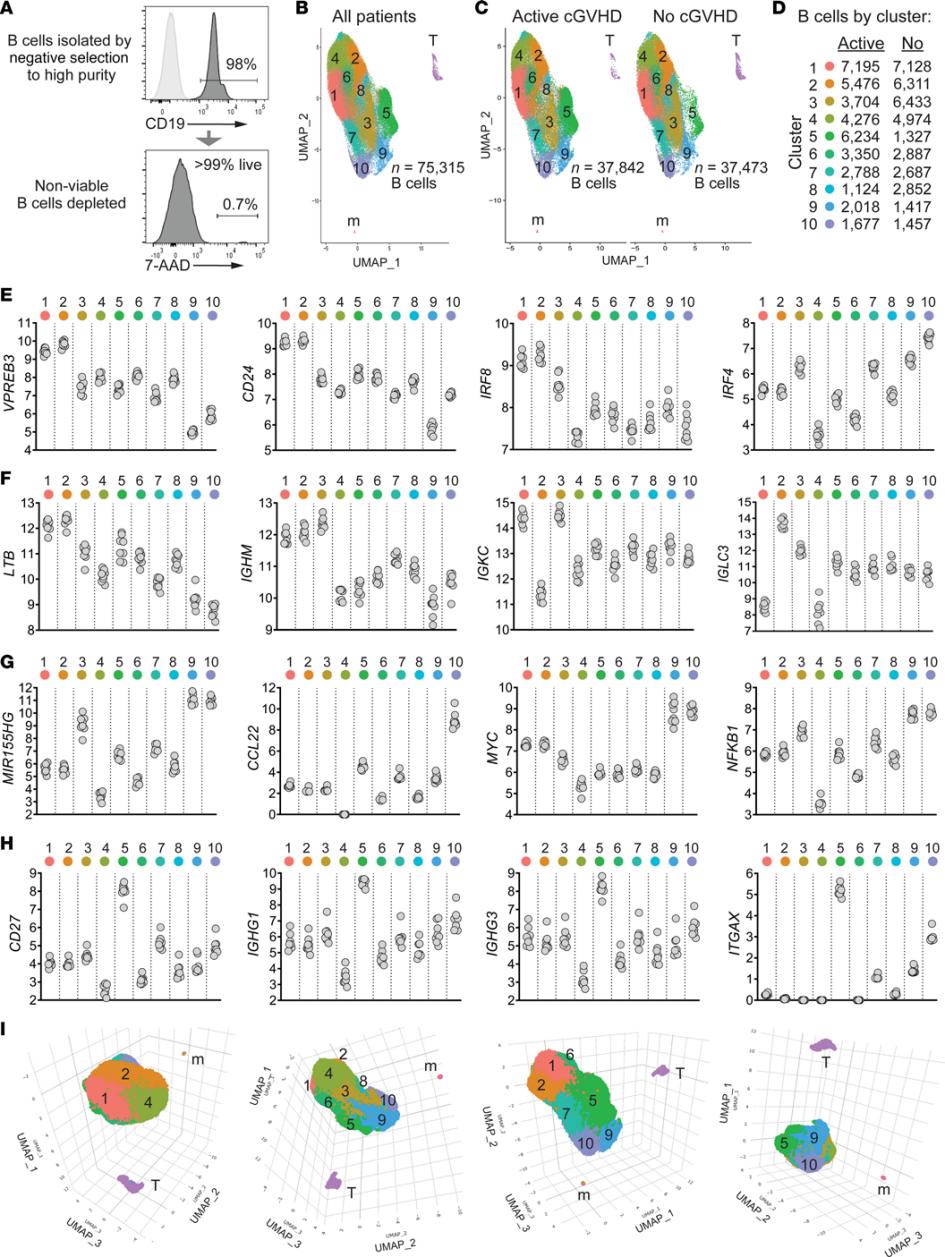
Unsupervised clustering analysis of scRNA-Seq data reveals multiple B cell subsets characterized by signature genes in allo-HCT patients. (**A**) Representative flow cytometry histograms showing B cell purity and viability from an allo-HCT patient; 10,000 high-quality B cells per patient sample isolated in the same manner were targeted for 10x Genomics single-cell library construction (*n* = 4 no cGVHD, *n* = 4 active cGVHD). (**B**) Two-dimensional uniform manifold approximation and projection (UMAP) expression profiles of all untreated cells from the 8 allo-HCT patients. Numbers indicate each of the 10 major clusters identified as B cells, which were distinct spatially from residual cells identified as T cells (T) and monocytes (m). As indicated, 75,315 high-quality B cells were analyzed. (**C**) UMAP as in **B**, with B cells partitioned by patient group. Total high-quality B cells per group are indicated. (**D**) Number of B cells mapping to each of the 10 B cell clusters by patient group. (**E**–**H**) Log_2_-normalized expression of genes indicative of B cell maturity (**E** and **F**), along with activation, antibody production potential, and memory markers (**G** and **H**), by B cell cluster. Each symbol (gray circle) represents regularized log-transformed gene counts (95) (*y* axes) from 1 of the 8 allo-HCT patients. (**I**) 3D UMAPs of B cells from all patients viewed at different angles of rotation.

Because allo-HCT affords an opportunity to better understand the intrinsic programming of B cell subsets when B cell tolerance is lost or maintained, we analyzed molecular features of a high number of highly purified, viable human B cells (Figure 1A) from 8 allo-HCT patients more than 9 months after engraftment (Supplemental Table 1; supplemental material available online with this article; https://doi.org/10.1172/jci.insight.169732DS1). To confirm cell types, inferences were made by examining the relative expression of genes representing B cells (*PAX5*, *CD22*) and rare residual T cells (*CD3E*) and monocytes (*LYZ*) (Supplemental Figure 1). Unsupervised clustering analysis of the 8-patient scRNA-Seq data set identified 10 clusters of B cells (Figure 1B). All 10 B cell clusters were represented in both the 4 active cGVHD and 4 no cGVHD patients (Figure 1C) and were numbered 1–10 based on largest to smallest total B cell number in both patient groups combined (Figure 1D).

To validate known and potentially novel B cell subsets in allo-HCT patients, we identified hallmark signature (marker) genes for all 10 clusters. We then plotted log_2_-normalized expression values for 16 major B cell development and function genes from all 8 patients (Figure 1, E–H), each validated using findMarkers in the R package Seurat (32). These signature genes were remarkably homogeneous within clusters among all 8 allo-HCT patients. Levels of the transitional B cell molecules *VPREB3*, *CD24*, and interferon regulatory factor 8 (*IRF8*) were highest in clusters 1 and 2 and lowest in clusters 9 and 10 (Figure 1E). Comparatively, cluster 3 had lower expression of *CD24* and *IRF8*, and higher *IRF4*, suggesting a more mature B cell stage. Elevated *LTB* further supported that clusters 1 and 2 represent recent bone marrow (BM) emigrants because lymphotoxin-β is required for follicle development in the spleen (33) and expressed highest in late transitional B cells (Immunological Genome Project [ImmGen], https://www.immgen.org). Clusters 1, 2, and 3 expressed the highest levels of IgM heavy chain (*IGHM*). Clusters 1 and 3 primarily expressed κ Ig light chain, and cluster 2 primarily expressed λ Ig light chain (*IGKC* and *IGLC3*, respectively, Figure 1F). Clusters 9 and 10 expressed similarly high levels of microRNA-155 (*MIR155HG*, Figure 1G), essential for B cell antibody isotype switching (34), and similarly high expression of *MYC* and *NFKB1*, suggesting these B cells are poised to enter the cell cycle. Cluster 10 had particularly high levels of the chemokine *CCL22*, indicating B cells primed to interact with follicular Th cells within secondary lymphoid organs (SLOs). By contrast, cluster 5 expressed high levels of *CD27*, along with IgG1 and IgG3 heavy chain genes (*IGHG1* and *IGHG3*, respectively), indicating an enrichment for isotype-switched memory B cells (Figure 1H). Cluster 5 also expressed the highest *ITGAX* (*CD11C*), a distinguishing marker of ABCs (35, 36). Since cluster proximity can reflect signature program relatedness, we also depicted 3D UMAP plots to further highlight spatial relationships among neighboring B cell clusters (Figure 1I). Together, these data allowed us to define molecular signatures of B cell subsets that regenerate and circulate in an alloantigen-rich environment.

### Allo-HCT B cell clusters can be further delineated via established signature pathways.

**Figure 2:**
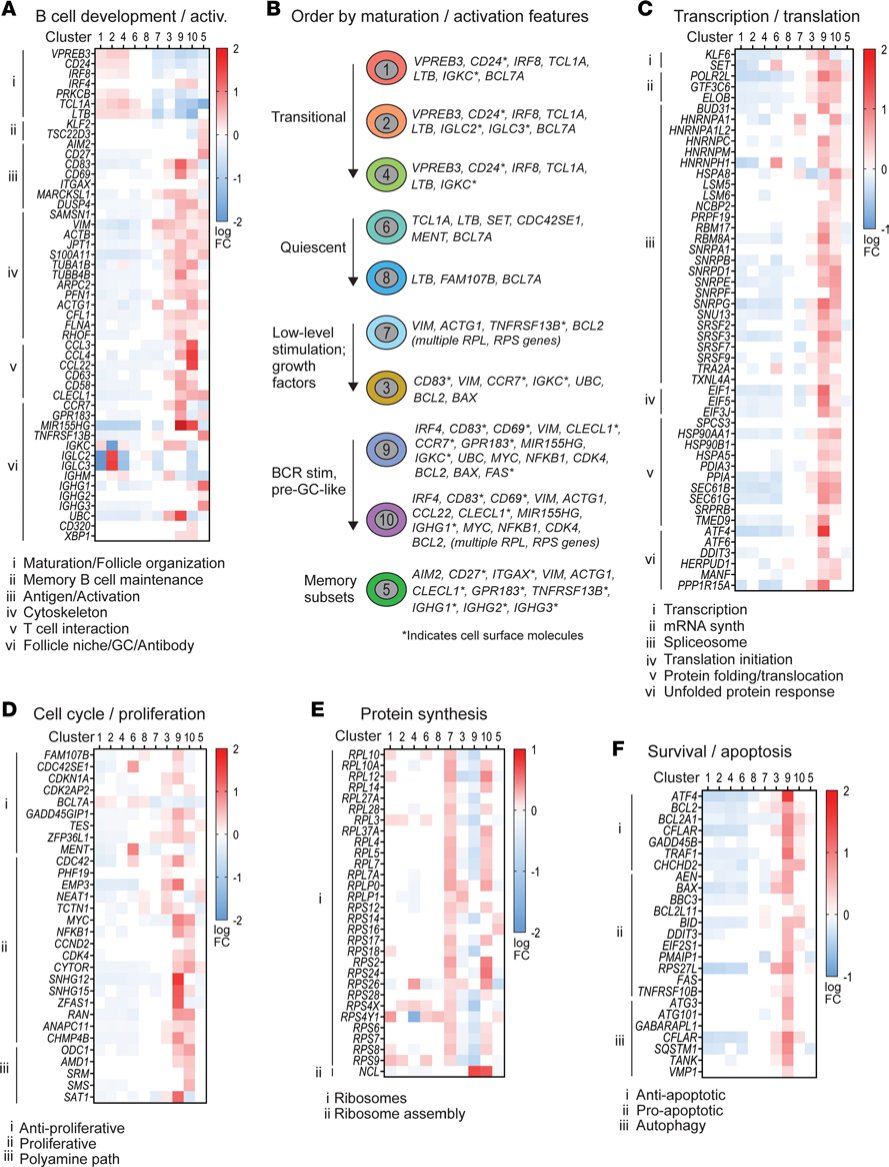
Signature genes empirically assigned to biological pathways corroborate the interrelatedness and features of B cell development and activation among the 10 B cell clusters. (**A**) Signature (marker) genes related to B cell development and activation assessed in the 8 allo-HCT patients in the scRNA-Seq data set by manually interrogating the Kyoto Encyclopedia of Genes and Genomes (KEGG) and Gene Ontology (GO) databases. Each colored square represents a significant (*P_adj_* < 0.05) log fold-change (log FC) value for the gene indicated (rows), in the B cell cluster indicated (columns). Red shading indicates increased signature gene expression, while blue shading indicates decreased signature gene expression, as weighted against all other clusters. Genes were subdivided according to specific pathways as indicated by the Roman numerals at left and corresponding key below each heatmap. GC, germinal center. (**B**) Cartoon depiction of B cell clusters and their associated signature genes that may help categorize these subsets (as labeled at left). Signature genes encoding surface proteins are indicated. (**C**–**F**) Signature genes assessed as described in **A** within the major biological pathways indicated above each heatmap and subdivided by the more specific pathways indicated by the Roman numerals at left and corresponding keys below.

We first reordered the 10 clusters based on the unsupervised clustering in Figure 1 and published literature of known B cell subsets that enter the periphery and those that eventually encounter antigen/costimulatory signals. We henceforth ordered from least mature/activated first and ending with memory signature gene profiles as follows: cluster 1, 2, 4, 6, 8, 7, 3, 9, 10, and 5. In Figure 2 and Supplemental Figure 2, colored squares in the heatmaps indicate signature genes of interest (rows) reaching significance (*P_adj_* < 0.05) in the cluster indicated (columns), as weighted against all other clusters. Red hues indicate increased signature gene expression, and blue hues indicate decreased signature gene expression (log FC). Notably, clusters 1, 2, 4, 6, and 8 retained 1 or more hallmark genes of B cell immaturity, while clusters 7, 3, 9, 10, and 5 expressed markers of antigen activation, cytoskeletal activity, or the capacity to produce antibodies (Figure 2, A and B). We further validated this ordering of the 10 B cell clusters by interrogating the KEGG (37) and GO (38) databases using signature genes of interest for major molecular pathways. Across pathways, clusters 9 and 10 showed striking increases in signature genes involved in B cell–T cell interactions, transcription, proliferation, survival/apoptosis, and metabolic processes (Figure 2, A and C–F, and Supplemental Figure 2, A and B). Interestingly, clusters 7 and 10 shared multiple ribosomal signature genes (Figure 2E), suggesting increased protein synthesis potential. Cluster 6 had strong expression of actin cytoskeleton organizer *CDC42SE1* and the proto-oncogene *MENT* (*C1orf56*), Figure 2D, but overall clusters 6 and 8 had relatively few signature gene increases, suggesting quiescence. In total, the less mature/less activated subsets (clusters 1, 2, 4, and 6) tended to have more decreased signature genes (down, Supplemental Figure 2C), while the activated/memory subsets (clusters 3, 9, 10, and 5) tended to have more increased signature genes (up, Supplemental Figure 2C). Thus, our data elucidate a hierarchy and relatedness among peripheral B cell subsets in human allo-HCT.

### The circulating memory B cell compartment in allo-HCT patients is distinct from HDs and non-HCT patients with chronic infections.

**Figure 3:**
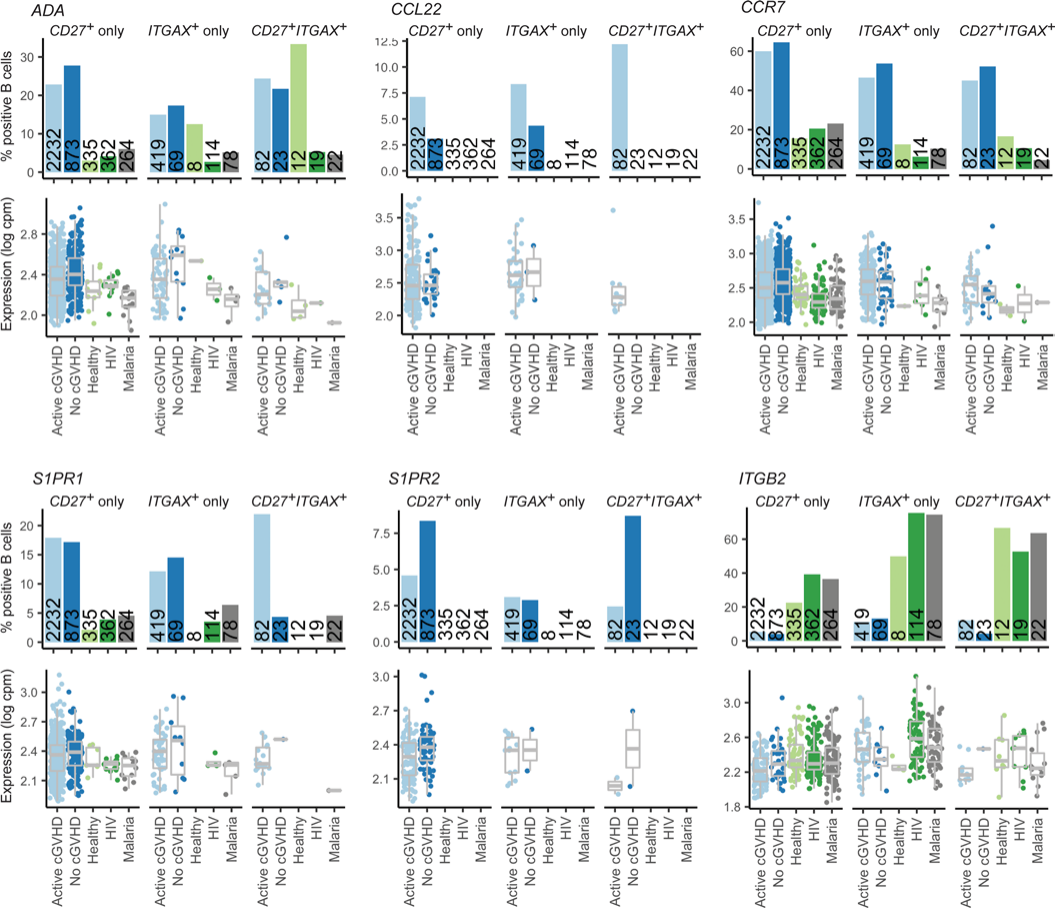
*CD27* and *ITGAX* memory B cell subsets from allo-HCT patients are altered in tolerance and homing genes compared with HDs and non-HCT patients with chronic infections. Shown are results for 6 memory B cell genes of interest displaying differences in the proportion of positive B cells between allo-HCT patients compared with HD (healthy) and non-HCT patient groups (HIV, malaria). Bar graphs represent the percentage of positive B cells for the population indicated. Numbers embedded within the bars indicate the total number of B cells identified within the subset indicated for the patient or HD group. In the gene expression graphs below each bar graph, each point represents an individual B cell, with the data normalized to show expression values for the gene of interest per million total mapped reads in the same B cell, expressed on a log scale (log counts per million). Box plots show the interquartile range (box), median (line), and minimum and maximum (whiskers).

**Figure 4:**
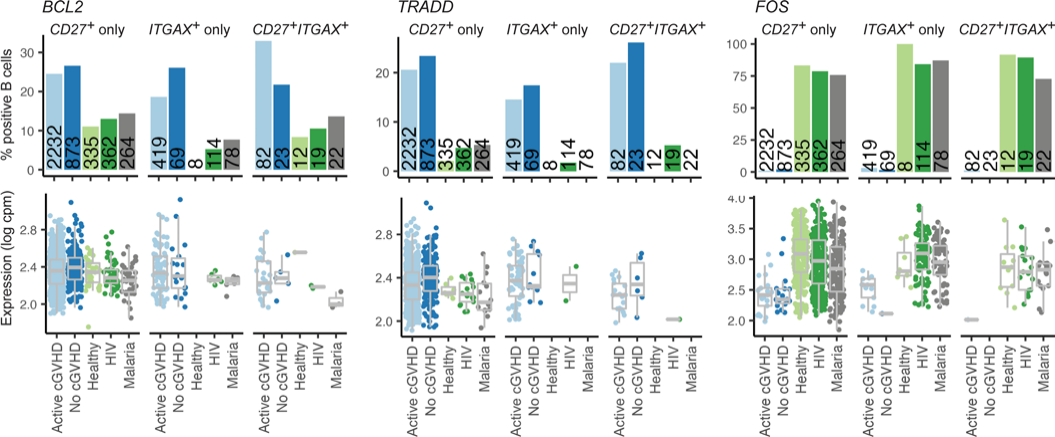
*CD27* and *ITGAX* memory B cell subsets from allo-HCT patients are altered in survival genes compared with HDs and non-HCT patients with chronic infections. Results for the 3 survival regulatory genes shown were obtained as described for the genes depicted in Figure 3.

To begin to address whether the allo-HCT setting itself causes intrinsic alterations in the B cell pool, we compared allo-HCT signature gene profiles with HDs and individuals with chronic infectious antigen exposure, namely HIV and malaria. Since functional memory B cell recovery is delayed after allo-HCT (13), we assessed memory B cell signatures. Leveraging a similar publicly available scRNA-Seq data set generated from blood B cells of HDs (*n* = 3), patients with HIV (*n* = 3), and patients with malaria (*n* = 3), published by Holla et al. (35), we first identified total *CD27^+^ITGAX^–^*, *CD27^–^ITGAX^+^*, and *CD27^+^ITGAX^+^* B cells from all groups, which should collectively represent BCR-experienced memory subsets. We then assessed the frequency of B cells within these cluster 5 subsets expressing known genes associated with typical memory B cells or ABCs (39–42). For most memory signature genes, the proportion of positive B cells in the 3 subsets was similar among all 5 groups (Supplemental Figure 3). Remarkably, B cells expressing *ADA*, associated with a form of SCID with disrupted B cell tolerance (43); the T cell–attracting chemokine *CCL22* (44); and 3 G protein–coupled receptors (GPCRs) important for B cell homing to lymphoid niches, *CCR7*, *S1PR1*, and *S1PR2* (45–49), were increased in proportion across *CD27*/*ITGAX* subsets in allo-HCT patients, irrespective of cGVHD status (Figure 3). By contrast, *ITGB2*, which pairs with various integrin α chains to mediate migration, was decreased in allo-HCT (35). Allo-HCT patients also had increased proportions of B cells across these subsets expressing survival regulators with roles in memory B cell maintenance including *BCL2* (50) and *TRADD* (51) (Figure 4). By contrast, allo-HCT patients had a paucity in B cells expressing *FOS*, important for apoptosis during the GC response (52). Together, these data reveal that the memory B cell pool in allo-HCT possesses intrinsic molecular hallmarks of altered tolerance, migration, SLO homing, and survival.

### BCR-experienced cluster 5 harbors distinct CD27^+^ B cell and ABC populations, with the latter expanded in active cGVHD.

**Figure 5:**
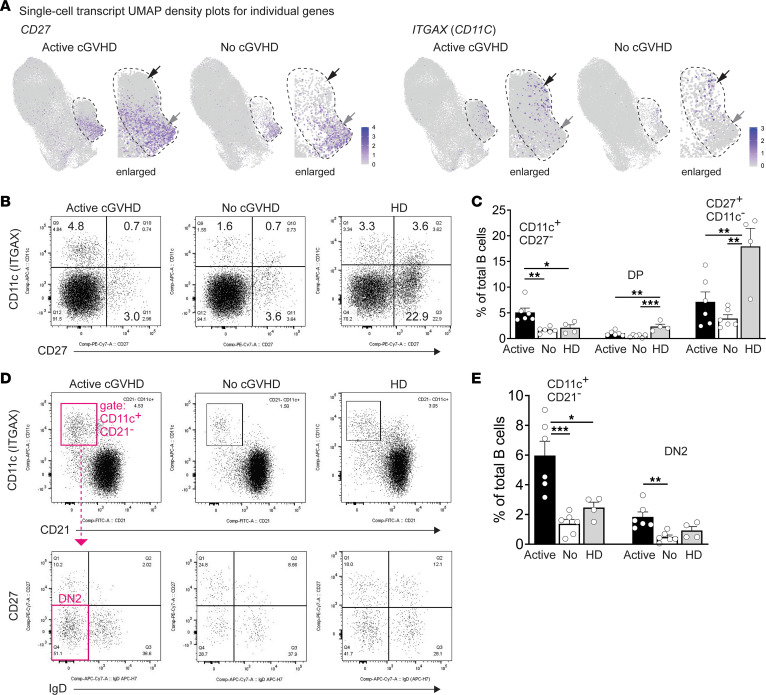
Cluster 5 contains both typical CD27^+^ memory B cells and ABC subsets, with the latter expanded in active cGVHD. (**A**) B cells expressing *CD27* or *ITGAX* transcripts are enriched in cluster 5 in 2 separate regions. Shown are UMAP density plots from the scRNA-Seq data set displaying relative transcript density for the gene indicated, at the single-cell level. The dashed line approximates the perimeter of cluster 5 (enlarged for clarity). Gray arrows indicate a region enriched for *CD27*-expressing B cells, and black arrows indicate a region enriched for *ITGAX*-expressing B cells. (**B** and **C**) Flow cytometric analysis to identify ABCs, CD27^+^ memory B cells, and CD11c^+^CD27^+^ memory B cells in PBMC samples from allo-HCT patients with active cGVHD (n = 6) or no cGVHD (*n* = 6) or from HDs (*n* = 4). PBMCs were pregated on live (7-AAD^–^) CD19^+^ B cells. Dot plots in **B** show representative individuals from each group. In **C**, results from all groups for the CD11c^+^CD27^–^ population, CD11c^+^CD27^+^ (DP) population, and CD11c^–^CD27^+^ population (as gated in **B**) are represented. Statistical comparison: 1-way ANOVA with Tukey’s multiple comparisons test (GraphPad Prism 9 software; *, *P* < 0.05; **, *P* < 0.01; ***, *P* < 0.001; error bars represent mean ± SEM). (**D** and **E**) DN2 B cells are expanded in allo-HCT patients with active cGVHD. (**D**) Representative FACS plots from PBMCs of allo-HCT patients with active or no cGVHD, and from an HD, generated after first gating on viable (7-AAD^–^), CD19^+^ B cells, then CD11c^+^CD21^–^ cells (upper panel), followed by DN2 B cell identification in all groups (CD11c^+^CD21^–^CD27^–^IgD^–^), as depicted in magenta for active cGVHD. (**E**) Statistical comparison between groups (active cGVHD, *n* = 6; no cGVHD *n* = 6, HDs, *n* = 4) was performed using a 1-way ANOVA with Tukey’s multiple comparisons test (GraphPad Prism 9 software; *, *P* < 0.05; **, *P* < 0.01; ***, *P* < 0.001; error bars represent mean ± SEM).

Since antigen-experienced B cells likely comprise the pathogenic B cells that emerge in cGVHD, as occurs in other diseases such as lupus (1, 53), we further interrogated “memory” cluster 5 for the presence of CD27^+^ typical memory B cells and for the presence of ABCs, which express CD11c (encoded by the *ITGAX* gene). ABCs have a pathogenic role in both autoimmunity and chronic inflammation, revealing a functional complexity of these memory B cells (28, 35). ABCs are often termed either “atypical” or “age-related” B cells, the latter term reflecting observed accumulation in aged mice (54). ABCs are considered atypical because they can undergo activation and differentiation outside the GC, homing to the outer follicle as extrafollicular B cells (23, 55, 56). To elucidate these memory subpopulations in cGVHD, we examined the pattern of *CD27* and *ITGAX* expression in cluster 5. As shown in Figure 5A, B cells expressing either *CD27* or *ITGAX* (UMAPs) were largely spatially segregated, suggesting mostly independent subsets. To validate this finding, we performed flow cytometry analysis on blood B cells from an independent cohort of allo-HCT patients. As shown in Figure 5B, allo-HCT memory B cells primarily expressed either CD27 or CD11c alone, with a smaller subset expressing both markers (double-positive, DP). Importantly, CD11c^+^CD27^–^ ABCs were significantly expanded in patients with active cGVHD compared with patients with no cGVHD and HDs (Figure 5, B and C). Interestingly, the CD11c^+^CD27^+^ DP subset was proportionally lower in the allo-HCT environment compared with HDs (Figure 5, B and C). Overall, these data support previous work showing that some CD11c^+^CD27^–^ ABC subsets are expanded in cGVHD and other diseases, sometimes exhibiting an exhausted phenotype (22, 57–59). We also examined a subset of CD11c^+^CD21^–^ ABCs that lacks both CD27 and IgD expression (CD11c^+^CD21^–^CD27^–^IgD^–^), called “DN2,” that is prevalent in lupus and can produce disease-associated antibodies (23, 25, 29). The emergence of pathogenic ABCs (including DN2) can be driven by one or more costimulatory signals that cooperate with BCR stimulation (23, 60). Indeed, DN2 ABCs were markedly expanded in patients with active cGVHD relative to no cGVHD (Figure 5, D and E). These observations affirm our scRNA-Seq data and advance previous work (23, 25, 29, 58, 59) suggesting that potentially pathologic ABC subsets arise under constant alloantigen exposure.

**Figure 6:**
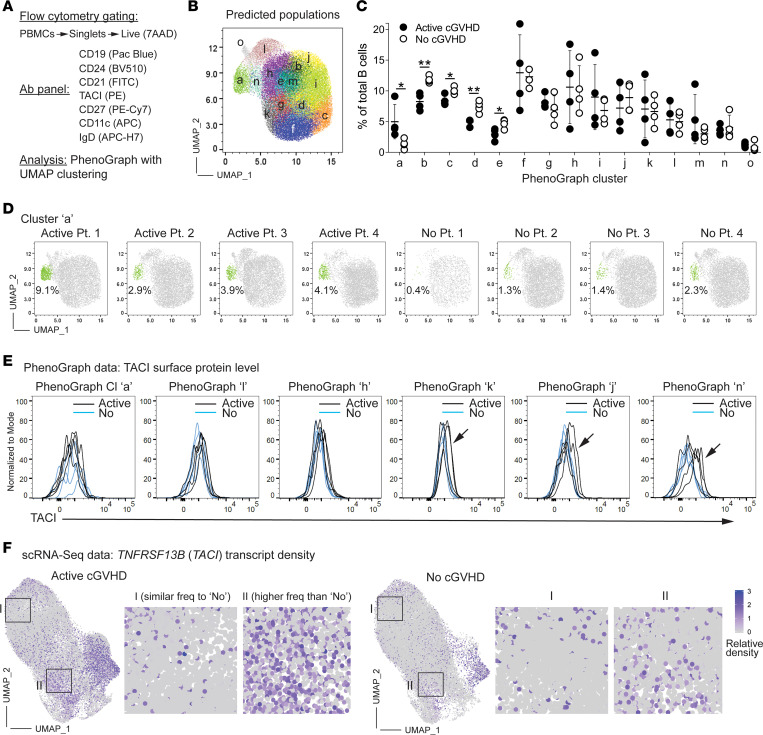
High-dimensional flow cytometry further delineates naive and memory B cell subset differences among allo-HCT patient groups. (**A**–**E**) Flow cytometry plus PhenoGraph analysis performed on blood B cells from active cGVHD (*n* = 4) or no cGVHD (*n* = 4) patients. (**A**) The gating strategy to distinguish B cells (top line), the B cell antibody panel used (middle), and the analysis platform (bottom). (**B**) The concatenated PhenoGraph UMAP plot from all 8 patients, with 15 clusters identified (letters). (**C**) B cell frequency within each of the 15 clusters by patient group. Each symbol represents results from 1 of the 8 patients assessed. Statistical comparisons were performed using a 2-tailed, unpaired *t* test (GraphPad Prism 9 software; *, *P* < 0.05; **, *P* < 0.01; error bars represent mean ± SD). (**D**) The PhenoGraph UMAP plots show the position (green) and frequency (%) of B cells for cluster A, which represents a population of ABCs (Supplemental Figure 4). (**E**) Histogram overlays for TACI surface protein expression on B cells in the 6 clusters from the PhenoGraph assessment described as TACI bright (TACI^br^, Supplemental Figure 4), from the 4 allo-HCT patients in each group. Arrows for clusters k, j, and n highlight elevated TACI expression levels observed in some patients with active cGVHD. (**F**) B cells positive for *TNFRSF13B*/*TACI* transcripts are elevated in patients with active cGVHD. Normalized expression of *TNFRSF13B* in UMAP space are shown, separated by patient group. Boxed areas in representative regions are enlarged to show detail.

To assess B cell surface phenotypes more broadly and elucidate subsets potentially responsive to excess BAFF (11), we examined cell surface CD24 and TACI (TNFRSF13B) expression in the context of CD27, IgD, CD11c, and CD21, using high-dimensional flow cytometry (PhenoGraph; refs. 61, 62; Figure 6A). We identified discrete B cell populations in an unbiased manner from concatenated allo-HCT patient samples, whereby PhenoGraph analysis resolved 15 clusters, designated by lowercase letters a–o (Figure 6B). CD11c^+^ ABCs were primarily resolved by cluster a (Supplemental Figure 4), with cluster a significantly expanded in active cGVHD (Figure 6, C and D). By contrast, 4 clusters consisting of IgD^+^CD27^–^ naive-like B cells (clusters b–e, Supplemental Figure 4) were decreased in patients with active cGVHD (Figure 6C). ABC cluster a was also TACI^+^, with a mix of IgD^+/–^ B cells (Supplemental Figure 4). Six clusters expressed high levels of surface TACI (clusters a, h, j, k, l, and n, Figure 6E and Supplemental Figure 4). Cluster n expressed substantially higher surface TACI in some patients with active cGVHD (Figure 6E, arrow) and mapped adjacent to ABC cluster a in the PhenoGraph UMAP (Figure 6B). Clusters j and k likewise had elevated TACI in some patients with active cGVHD (Figure 6E, arrows). Accordingly, regions of B cells in the scRNA-Seq data having increased *TNFRSF13B/TACI* transcripts were readily evident in active cGVHD (Figure 6F). These results support studies implicating BAFF as a potential driver of B cell hyperresponsiveness and alloantibody production in cGVHD (7, 8, 10, 11).

To compare allo-HCT with the non-HCT setting, we also performed the PhenoGraph analysis on HD blood B cells. The HD analysis also predicted 15 clusters, but HDs had a greater number of B cell clusters marking with CD27 (Supplemental Figure 5, clusters m, n, o) compared with allo-HCT patients (Supplemental Figure 4, clusters l, o only). Furthermore, in allo-HCT patients, cluster o represented mostly CD27^bright^ B cells that lacked CD21, CD24, IgD, and CD11c expression, suggestive of isotype-switched plasmablasts (PBs, Supplemental Figure 4). These were among the rarest cells in both allo-HCT groups (Figure 6C), and this cluster was notably absent in HDs (Supplemental Figure 5). These data reveal that CD27^+^ B cell subsets after allo-HCT are abnormal or lacking, extending previous observations (22). The relative reduction in CD27^+^ B cells in allo-HCT with reciprocally expanded ABCs in active cGVHD is consistent with recent PhenoGraph findings in pediatric patients with severe autoimmune syndromes (63). Together these data further elucidate the allo-HCT memory B cell pool and begin to validate our scRNA-Seq data by showing TACI expression increases in ABCs or their precursors in cGVHD.

### Genes critical for B cell fate and function are differentially expressed in active cGVHD.

**Figure 7:**
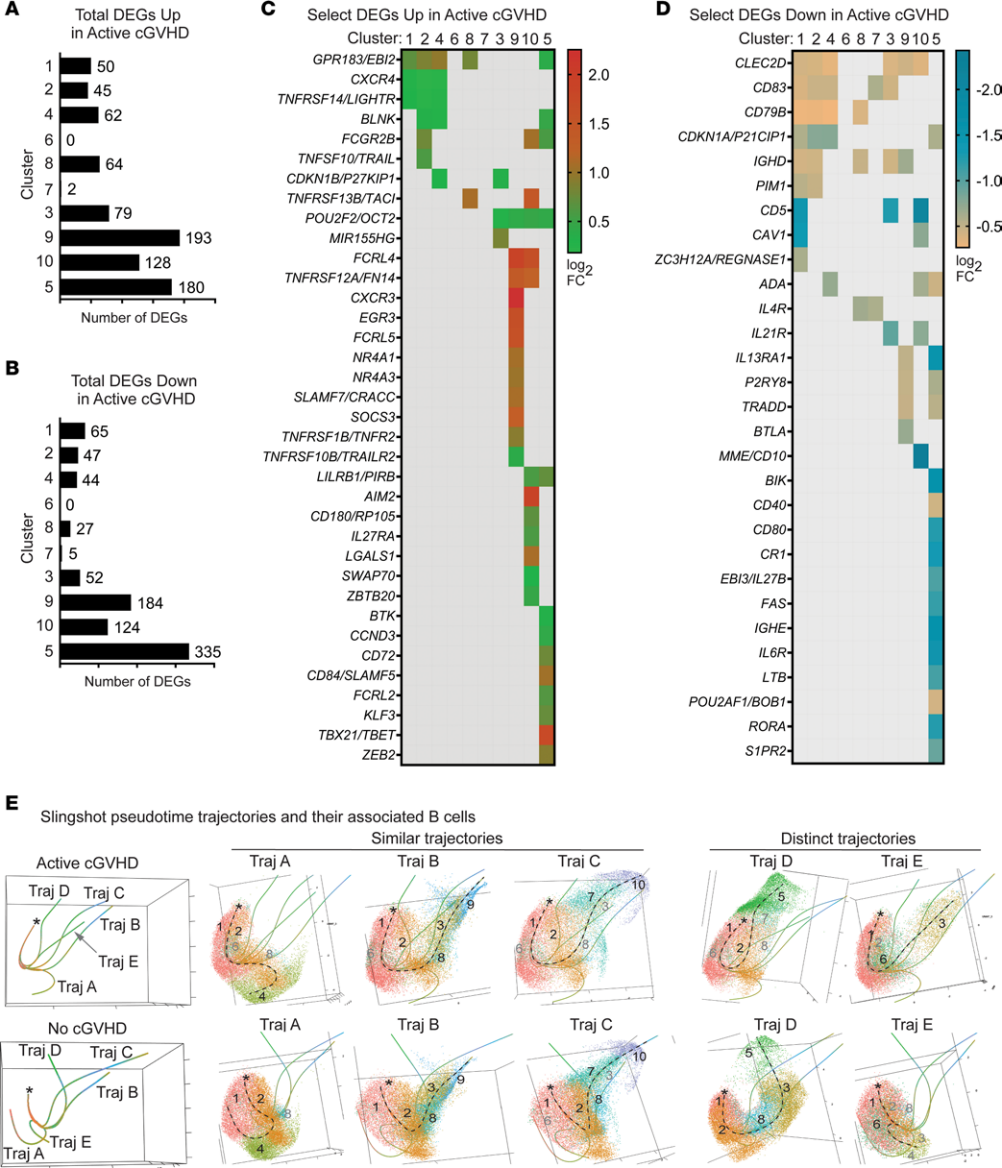
Molecules critical to B cell function are altered within clusters in active cGVHD. (**A**–**D**) DEG analysis within the 10 B cell clusters in the scRNA-Seq data set based on disease status (as described in Figure 1: active cGVHD, *n* = 4; no cGVHD, *n* = 4). Bar graphs indicate total DEG number by cluster, up (**A**) or down (**B**), in active cGVHD. The heatmaps in **C** and **D** depict DEGs selected from the entire data set (Supplemental Table 3), critical for various aspects of B cell function (select DEGs). Colored squares represent significant (*P_adj_* < 0.05) log_2_ fold change (log_2_ FC) values for DEGs shown (rows) within the cluster(s) indicated (columns), either up (**C**) or down (**D**), in active cGVHD B cells. (**E**) Panels at left show Slingshot pseudotime trajectory predictions (Traj A–E) for untreated B cells from patients with active cGVHD (top) and no cGVHD (bottom). The origin of the pseudotime analysis (asterisks) was set as cluster 1 based on our knowledge of its transitional like *IGKC* signature gene profile (Figure 1, E and F, and Figure 2, A and B), suggesting it is the earliest peripheral B cell population emerging from the BM. Panels at right represent each trajectory in isolation (dashed line for reference), along with its associated B cells. B cell clusters are colored and numbered per original unbiased clustering (Figure 1, B, C, and I). Black numbers indicate major clusters that lie along each trajectory, while clusters present but having only a small number of B cells represented are indicated in gray.

We next asked whether intrinsic B cell programs were altered in patients with active cGVHD by examining DEGs between allo-HCT patient groups in the scRNA-Seq data set. Bar graphs in Figure 7 represent total DEGs by cluster, either increased (up, Figure 7A) or decreased (down, Figure 7B), in active cGVHD compared with no cGVHD. BCR-activated clusters 9 and 10, and memory cluster 5, collectively had the most DEGs (up or down) compared with the more naive, resting subsets (Figure 7, A and B). From the total list of DEGs (Supplemental Table 3), those depicted in Figure 7, C and D, and Supplemental Table 4 represent genes with known functional roles in B cells (select DEGs). Regulators of upstream BCR signaling, *BTK* and *BLNK*, were up DEGs in active cGVHD, including memory cluster 5 (Figure 7C and Supplemental Table 4). This is consistent with work showing BAFF-dependent increases in BLNK protein in B cells from active cGVHD patients and mice (8, 19). The transcription factor *ZBTB20* was increased in pre-GC–like cluster 10 and is essential for the conversion of BCR-activated B cells to antibody-secreting cells (ASCs) and maintenance of long-lived ASCs (64). *CAV1* (*CAVEOLIN-1*), which controls BCR compartmentalization on the plasma membrane (65), was decreased in clusters 1 and 10 (Figure 7D and Supplemental Table 4). Accordingly, *Cav1* deficiency in mice leads to altered nanoscale organization of IgM-BCRs and a skewed Ig repertoire with features of poly-reactivity and autoreactivity (65). Molecules functionally important in ABC expansion or function were also significantly affected. *ZC3H12A*, encoding the RNA-binding protein REGNASE-1, was decreased in cluster 1 (Figure 7D and Supplemental Table 4), and Regnase-1 deficiency at earlier stages of B cell development in mice produces severe autoimmune immunopathology, accompanied by ABC expansion (66).

*TBX21*, *ZEB2*, and *EGR3* are important and potential master regulators of ABC development and/or function in mice and humans (24, 26, 30, 60), and all were up DEGs in either memory cluster 5 (*TBX21*, *ZEB2*) or activated cluster 9 (*EGR3*) (Figure 7C and Supplemental Table 4). Cluster 5 was enriched for ABCs (Figure 5A), suggesting that dysregulated *TBET*, *ZEB2*, and *EGR3* are potential drivers of the observed ABC expansion in active cGVHD (Figure 5, B–E). Thus, key DEGs observed in active cGVHD suggest potential functional diversity before and after BCR engagement.

Given a potential role for BAFF in the genesis and progression of cGVHD (7, 8, 10, 11, 67), it was notable that BAFF receptor *TNFRSF13B*/*TACI* was increased in clusters 8 and 10 (Figure 7C and Supplemental Table 4), also supported by our PhenoGraph observations in some subsets (Figure 6E). Other members of the TNF superfamily (TNFSF) or TNF receptor superfamily (TNFRSF) were likewise up DEGs in active cGVHD B cells. These included *TNFRSF14* (*LIGHTR*, *CD270*), *TNFSF10* (*TRAIL*, *CD253*), *TNFRSF12A* (*FN14*, *TWEAKR*, *CD266*), *TNFRSF1B* (*TNFR2*, *CD120b*), and *TNFRSF10B* (*TRAILR2*, *CD262*) (Figure 7C and Supplemental Table 4). Interestingly, *GPR183*/*EBI2* was overexpressed in cluster 8 along with *TNFRSF13B/TACI* (Figure 7C, Supplemental Table 4, and Supplemental Figure 6A), possibly reflecting a positive influence by BAFF on EBI2 expression, as reported (68). Coexpression of *GPR183* and *TNFRSF13B* indeed occurred in some B cells, which were expanded 4-fold in patients with active cGVHD (Supplemental Figure 6, B and C). Accordingly, EBI2 and TACI was each expressed robustly on the surface of CD11c^+^ ABCs (Supplemental Figure 6D), while follicular like naive B cell subsets were generally low/negative for both proteins (Supplemental Figure 6E). These data support the notion that EBI2 and TACI, when dysregulated together, represent a developmental branch point important for ABC expansion (35).

Along with *GPR183/EBI2*, other GPCRs known to regulate B cells within SLO niches, including *P2RY8*, *S1PR2*, and *CXCR3* (47, 69–72), were also DEGs. *GPR183/EBI2* was notably increased across 5 B cell clusters in active cGVHD (Figure 7C and Supplemental Table 4), including transitional-like clusters 1 and 2 and memory cluster 5, which is remarkable because GPR183/EBI2 regulates B cell movement within the follicle before and following the GC reaction (69, 72). *P2RY8* and *S1PR2* were down DEGs in activated cluster 9 and/or the memory cluster 5 (Figure 7D and Supplemental Table 4), which is intriguing given their pivotal roles in B cell homing and confinement to follicular niches or the GC reaction (47, 71, 73). Decreased *P2RY8* expression is also linked to pathogenic antibody production and expansion of plasma cells and ABCs in humans and mice (74). These DEG data thus suggest altered follicle and GC movement of B cells in human cGVHD, consistent with findings in cGVHD mouse studies (3, 20, 75). Finally, genes associated with cell cycle were among the observed DEGs in active cGVHD. These included *CDKN1B* (*P27KIP1*) and *CCND3* (up, Figure 7C and Supplemental Table 4) and *CDKN1A* (*P21CIP1*) (down, Figure 7D and Supplemental Table 4). This is consistent with our previous observations that active cGVHD B cells demonstrate enhanced proliferation to some important stimuli (9).

Numerous DEGs described above influence B cell migration and homing, which compelled analysis of cGVHD diseased tissue sites. We performed scRNA-Seq on a punch biopsy of lesional dermal skin from a patient with sclerodermatous cGVHD and compared it with a biopsy of dermal skin from an HD. Dermal B cells were detected in both samples, distinguishable by a single UMAP cluster signature gene profile (Supplemental Table 5), indicating the presence of isotype-switched (*IGHG1*, *IGHG3*, *IGHG4*, *IGHA1*) B cells. Other notable signature genes included *GPR183*/*EBI2* and BAFF receptors including *TNFRSF13B*/*TACI* and *TNFRSF13C*/*BAFFR*. Multiple up DEGs in blood B cell clusters (Figure 7C and Supplemental Table 4) were also up DEGs in active cGVHD skin B cells (Supplemental Table 6). *POU2F2*/*OCT2*, important for generation of ASCs (76); *SWAP70*, shown to influence ABC expansion (77, 78); and *AIM2*, which positively regulated expansion of autoreactive B cells in a mouse lupus model (79), were increased in both blood and skin B cells in active cGVHD. Thus, lesional skin B cells in active cGVHD share characteristics of hyperactivity with circulating B cell subsets.

### Trajectories for B cell subset diversification in patients with active cGVHD reflect potentially reversible maturation defects.

Our past findings suggest a maturation block is linked to aberrant activation of circulating active cGVHD B cells (9). Thus, we performed pseudotime trajectory analysis on our scRNA-Seq data set using Slingshot (80). As shown in Figure 7E, Slingshot predicted 5 pseudotime trajectories (designated Traj A–E) for each allo-HCT patient group. Asterisks indicate the origin for the trajectories, empirically assigned to cluster 1 because of its transitional like, *IGKC* (Igκ^+^) phenotype. Traj A, B, and C were remarkably similar between patient groups (Figure 7E, similar trajectories). Traj A consisted primarily of B cell clusters 1, 2, and 4 only, possibly indicating termination at cluster 4 that had generally low transcripts (Figure 1, E–H), consistent with anergy. Traj B proceeded through clusters 1, 2, 8, 3, and 9. Traj C proceeded through clusters 1, 2, 8, 7, and 10, although in active cGVHD there were notably fewer B cells representing cluster 8. Traj D and E were markedly different between patient groups (Figure 7E, distinct trajectories). In no cGVHD, Traj D progressed through clusters 1, 2, 8, 3, and 5. By contrast, in active cGVHD, Traj D lacked cluster 3 and had minimal cluster 8 B cells, proceeding primarily through clusters 1 and 2, then directly to cluster 5. Traj E had major contributions from clusters 1 and 6 in both groups, while cluster 3 was uniquely prominent in active cGVHD. These observations suggest that the generation of cluster 5 memory B cells in patients with active cGVHD may occur along a diversification pathway that bypasses tolerance checkpoints, represented by cluster 8 and cluster 3 in no cGVHD.

**Figure 8:**
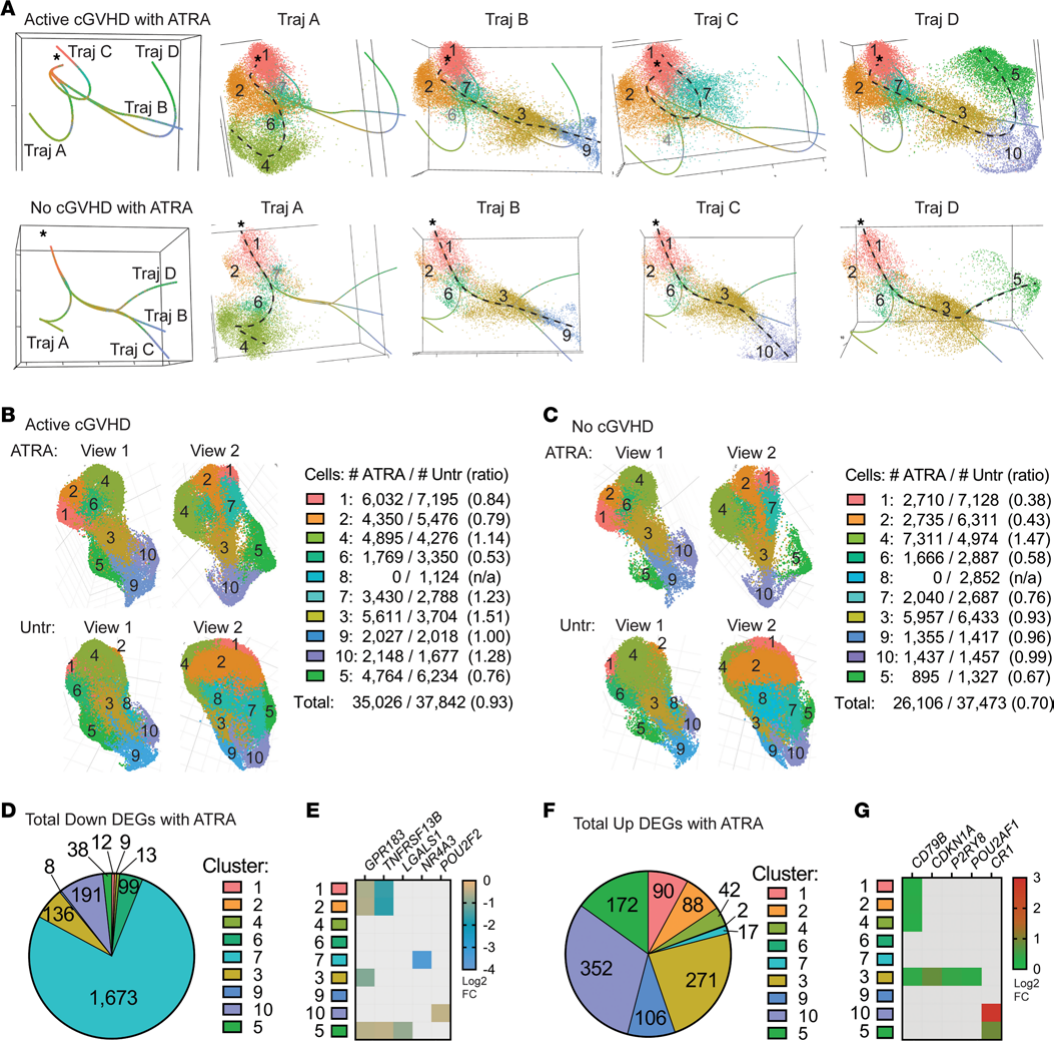
An inducer of cell differentiation, ATRA, differentially influences B cell pseudotime trajectories and cluster distribution in active cGVHD. (**A**) Panels at left show trajectory estimates (Traj A–D) for B cells from patients with active cGVHD (top) and no cGVHD (bottom) after ATRA treatment. The origin (asterisks) was set as cluster 1, as described in Figure 7E. Panels at right for each patient group represent each trajectory in isolation (dashed line for reference), along with all its associated B cells (colored and numbered by the cluster to which they belong, as in Figure 7E). Black numbers indicate major clusters that lie along each trajectory, while clusters present but having a small number of B cells are indicated in gray. (**B** and **C**) ATRA effects on B cell distribution among clusters relative to untreated B cells. UMAP cluster projection and cell distribution per cluster among ATRA-treated and untreated (Untr) active cGVHD samples (**B**) and no cGVHD samples (**C**). Total B cell numbers within each cluster for each treatment group and patient group are indicated. For ATRA-treated B cells, the 9 clusters shown were identified as corresponding to the same 9 clusters in the untreated groups based on signature gene profiles. Ratios represent the number of B cells with ATRA treatment divided by untreated cells (ATRA/Untr). (**D**–**G**) DEGs induced by ATRA in B cells from all 8 allo-HCT patients in the scRNA-Seq data set, compared with untreated B cells from all 8 patients. Pie charts indicate the total number of DEGs significantly decreased (**D**) or increased (**F**) after ATRA treatment in the cluster indicated. Heatmaps (**E** and **G**) represent some key DEGs observed in untreated B cells alone (active vs. no cGVHD, Figure 7, C and D), that were also altered by ATRA treatment. Colored squares represent significant (*P_adj_* < 0.05) log_2_ FC value for the gene and cluster indicated, for ATRA-treated B cells (all 8 allo-HCT patient samples) compared with untreated B cells (all 8 allo-HCT patient samples).

Previously, we utilized all-*trans* retinoic acid (ATRA) as a tool in vitro to “mature” active cGVHD B cells by restoring a normal IRF4/IRF8 ratio, attenuating hyperresponsiveness to BCR and NOTCH costimulation (9). Thus, we assessed the effects of ATRA on B cell clustering, DEGs, and trajectory inferences. A total of 10,000 ATRA-treated B cells per sample were targeted for scRNA-Seq analysis, from the same 8 allo-HCT donors described in Figure 1 and Supplemental Table 1. ATRA efficacy was validated by the upregulation of known ATRA-responsive genes across multiple B cell clusters, including *PLAAT4* (i.e., retinoid-inducible gene 1) and *ASB2* (81) (Supplemental Table 7). ATRA treatment resulted in 4 predicted pseudotime trajectories (designated A–D) in both allo-HCT patient groups (Figure 8A), compared with the 5 trajectories for untreated B cells (Figure 7E), suggesting a narrowing of transcriptional diversity. Supporting this, cluster 8 was notably absent from the post-ATRA UMAP clusters, while the 9 remaining clusters were present (Figure 8, B and C). Remarkably, the trajectory leading to cluster 5 in ATRA-treated active cGVHD B cells (Traj D, Figure 8A) included clusters 1, 2, 7, 3, 10, and 5, which was a markedly different profile from the trajectory leading to cluster 5 in untreated active cGVHD B cells (Traj D, Figure 7E). This suggests that ATRA, to some extent, normalized the trajectory leading to memory B cells in active cGVHD. In all scenarios with ATRA treatment, cluster 3 preceded clusters 9, 10, and 5, further implicating cluster 3 as a pivotal precursor memory checkpoint population.

Interestingly, ATRA affected the distribution of some B cell populations differently between patient groups. The ratio of B cells mapping to clusters 1 and 2 with ATRA (vs. untreated) was much lower for no cGVHD B cells (0.38 and 0.43, respectively) compared with active cGVHD B cells (0.84 and 0.79, respectively) (Figure 8, B and C). The ratios of B cells mapping to clusters 7 and 3 were reciprocally changed between groups by ATRA, decreasing in no cGVHD (0.76 and 0.93, respectively) and increasing in active cGVHD (1.23 and 1.51, respectively). These differences may be explained, at least in part, by apparent reduced viability following ATRA treatment in no cGVHD compared with active cGVHD B cells (ATRA/untreated total B cell ratio of 0.70 for no cGVHD; ATRA/untreated total B cell ratio of 0.93 for active cGVHD). These data affirm important maturation potential of the allo-HCT B cell compartment.

Finally, we assessed DEGs following ATRA treatment and found altered expression of a multitude of genes compared with untreated B cells from all 8 allo-HCT patients (Supplemental Table 7), although these DEG changes were statistically indistinguishable based on cGVHD disease status. Total DEGs decreased or increased by ATRA are represented numerically in Figure 8, D and F, respectively. Cluster 7 was most affected by ATRA, with 1,673 down DEGs compared with untreated B cells (Figure 8D and Supplemental Table 7). ATRA influenced some key genes (Figure 8, E and G) that were also DEGs in the untreated only disease group comparison (Figure 7, C and D). Notably, *GPR183*/*EBI2* and *TNFRSF13B*/*TACI* were decreased by ATRA across multiple clusters (Figure 8E), and these were up DEGs in untreated active cGVHD B cells (Figure 7C). Thus, ATRA both reshaped the distribution of B cells among clusters and reciprocally influenced some DEGs observed in active cGVHD.

### Broadly dysregulated genes suggest a common program underpins B cell diversification in patients with active cGVHD.

**Figure 9:**
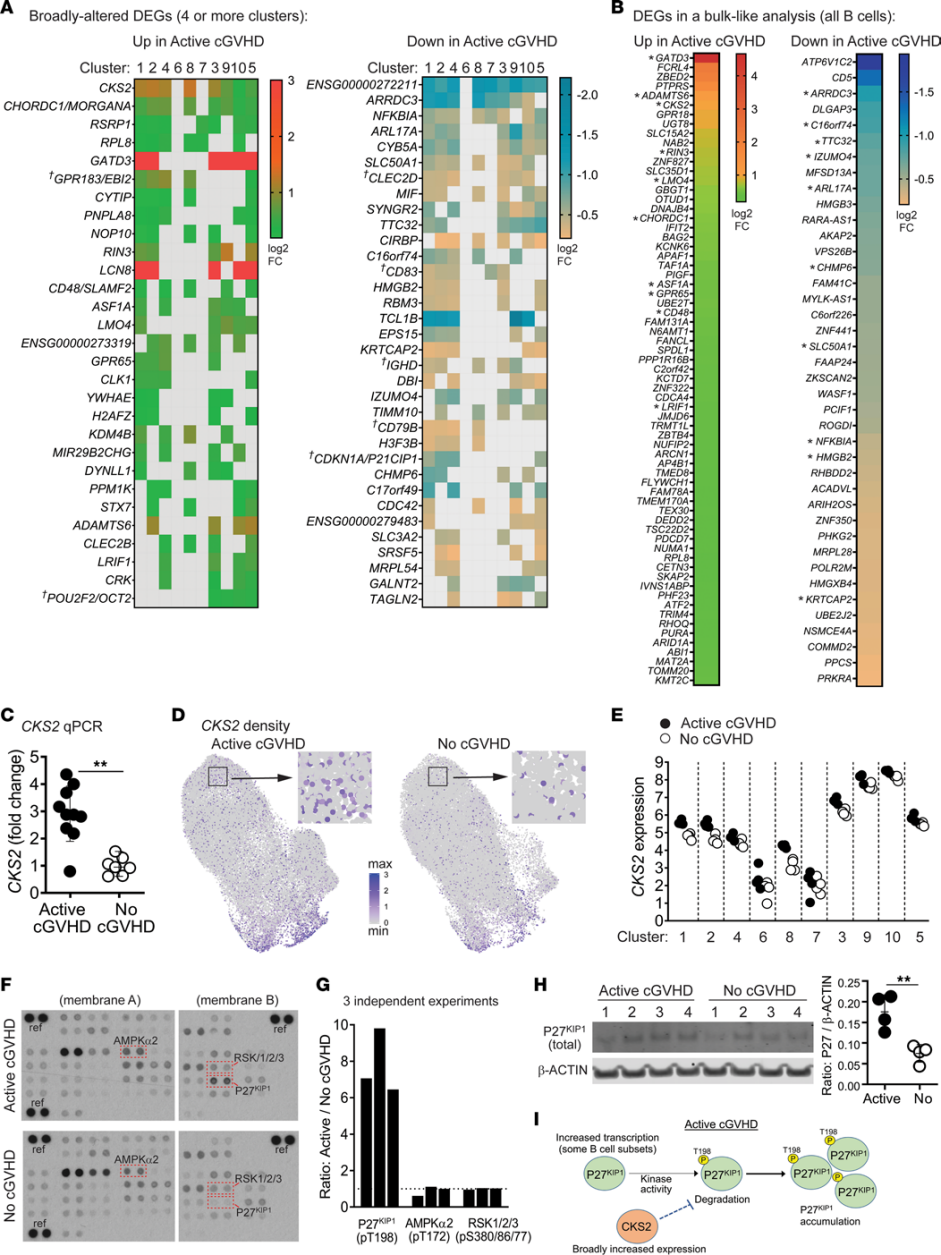
Assessment of DEGs occurring across many clusters provides additional insight into altered B cell functions in active cGVHD. (**A**) scRNA-Seq DEGs (*P_adj_* < 0.05) occurring in 4 or more B cell clusters, either up or down in active cGVHD. Colored squares indicate significance. Crosses indicate DEGs also depicted in Figure 7, C and D. (**B**) scRNA-Seq DEGs (*P_adj_* < 0.05) in total untreated B cells, up or down in active cGVHD. Asterisks indicate genes also represented in **A**. (**C**) qPCR analysis of CKS2 on B cells from patients with active cGVHD (*n* = 10) or no cGVHD (*n* = 7). Results indicate fold-change *CKS2* expression with the mean value in no cGVHD normalized to 1. *ACTB* was the housekeeping gene. Statistical comparison: 2-tailed Mann-Whitney test (GraphPad Prism 9; **, *P* < 0.01; error bars, mean ± SD). (**D**) *CKS2* UMAP transcript density plots between active cGVHD and no cGVHD. Representative regions (boxes) are enlarged to visualize single B cells. (**E**) Representative phosphoprotein arrays on whole-cell lysates of untreated B cells from active cGVHD (*n* = 3) and no cGVHD (*n* = 3) patients. Boxes and protein IDs indicate the location (duplicate spots) of P27^KIP1^ (phospho-T198), AMPKα2 (phospho-T172), and RSK1/2/3 (phospho-S380/S386/S377, respectively). Reference control spots are indicated as “ref.” (**G**) Combined results from 3 independent phosphoprotein arrays shown in **F** and Supplemental Figure 7. Each bar indicates results from 1 experiment, representing average dual spot intensity for active cGVHD over no cGVHD B cells (dashed line = ratio of 1 as a guide). (**H**) Western blot of total P27KIP1 relative to β-ACTIN in blood B cell lysates from patients with active cGVHD (*n* = 4) and no cGVHD (*n* = 4). Statistical comparison: 2-tailed, unpaired *t* test (GraphPad Prism 9 software; **, *P* < 0.01; error bars represent mean ± SD). (**I**) Model depicting heightened P27^KIP1^ accumulation in active cGVHD B cells.

Since B cell hyperresponsiveness to surrogate antigen is found in a high proportion of B cells from patients with active cGVHD (9, 19), we assessed whether some DEGs occur broadly, which might affect most B cell subsets. As shown in Figure 9A, we identified up DEGs and down DEGs in the scRNA-Seq data set occurring in at least 4 clusters in untreated active cGVHD B cells, ranked based on occurrence in the most clusters. Twenty-nine DEGs were increased and 34 DEGs were decreased in 4 or more clusters in active cGVHD. DEGs with a known role in B cell function (depicted in Figure 7, C and D) are indicated by crosses in Figure 9A. To corroborate broad dysregulation, we also subjected the scRNA-Seq data set to a “bulk-like” DEG analysis (all B cells, Figure 9B). Indeed, numerous DEGs from Figure 9A were also significant in the bulk-like analysis (Figure 9B, asterisks). We grouped the DEGs from Figure 9B empirically into GO-annotated cellular pathways to further highlight potential function (Supplemental Table 8). *ARRDC3* was among the most broadly decreased DEGs in active cGVHD B cells (Figure 9, A and B). Although the function of this α-arrestin in B cells is unknown, this finding further highlights the dysregulation of multiple GPCRs in active cGVHD. Also notable, *NFKBIA* (encoding IκBα, the inhibitor of NF-κB signaling) was decreased in 8 clusters.

*CKS2* stood out among the most broadly increased DEGs in active cGVHD B cells (Figure 9A) and in the bulk-like analysis (Figure 9B). We further validated this *CKS2* finding by quantitative PCR (qPCR) analysis on purified B cells from an independent cohort of allo-HCT patients (Figure 9C and Supplemental Table 2). CKS2 serves as a coactivator of cyclin-dependent kinases (CDKs) (82). Alternatively, CKS2 can delay premature cell cycle entry by directly protecting phosphorylated CDK inhibitor P27^KIP1^ (CDKN1B) from proteasomal degradation (83), which prevents apoptosis (84). UMAP plot comparisons depict the widespread increase in *CKS2*-positive B cells in active cGVHD (Figure 9D), and when displayed as normalized expression values (Figure 9E), *CKS2* was uniformly increased in the 8 significant DEG clusters from Figure 9A.

Broadly increased *CKS2* in active cGVHD suggested P27^KIP1^ may be more protected from degradation, a mechanism described in neurons that relies on P27^KIP1^ phosphorylation at T198 (83). Employing protein phosphoarrays, we tested purified B cell lysates from an independent cohort of allo-HCT patients. P27^KIP1^ phosphorylated T198 (phospho-T198) was detected at a 6- to 10-fold greater level in active cGVHD B cells compared to no cGVHD B cells (Figure 9, F and G and Supplemental Figure 7). Active site phosphorylation on AMPKα2 and RSKs, kinases known to phosphorylate P27^KIP1^ T198 (85, 86), were similar between allo-HCT patient B cells, suggesting increased kinase activity was not responsible for enhanced phospho-T198 in active cGVHD. Western blot analysis of total protein supported the concept of P27^KIP1^ being protected in active cGVHD B cells, with significantly higher levels compared with no cGVHD B cells (Figure 9H). These data enable formulation of a model for potential P27^KIP1^ regulation in active cGVHD B cells (Figure 9I), whereby broadly increased CKS2 expression, combined with increased *CDKN1B*/*P27KIP1* transcription in some subsets (Figure 7, C and E), may lead to P27^KIP1^ accumulation. Thus, our data extend potential functional contributors to the enhanced B cell survival described in active cGVHD (7, 9, 19).

## Discussion

Our scRNA-Seq analysis reveals and details the breadth of B cell subset and gene expression abnormalities in human cGVHD. While immediate BCR hyperresponsiveness and aberrant upstream signaling in B cells from patients who manifest cGVHD has been shown (7, 9, 19), comprehensive analyses of the peripheral B cell compartment have been lacking. The clarity provided herein identifying signature genes that define individual B cell subpopulations, and DEGs associated with active cGVHD, may support the development of improved targeted therapies for patients by exploiting the identified molecular pathways to further understand tolerance loss in B cells, in allo-HCT and beyond.

**Figure 10:**
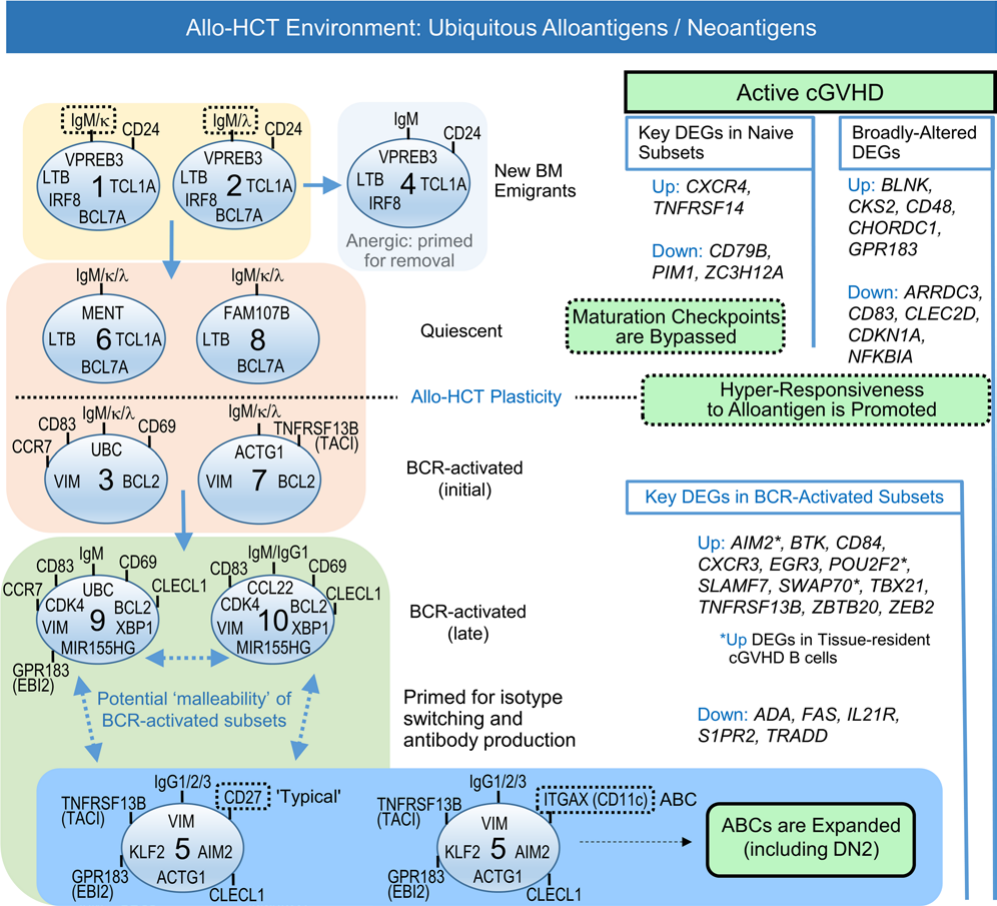
Working model for extrinsic activation signals and intrinsic gene changes influencing B cell maturation, survival, and proliferative capacity that lead to the expansion of pathologic memory B cells in active cGVHD. Cluster-defining signature genes and trajectory analyses led to assignment of the 10 clusters along a logical pathway of B cell diversification in the periphery of allo-HCT patients (left side), from newly emigrated populations, through proposed checkpoint steps where survival decisions occur, and finally to BCR-activated and memory states. Together, data suggest that extrinsic activation signals, including ubiquitous alloantigens and cytokines like BAFF, enable gene changes generally in the allo-HCT environment relative to the non-HCT setting that leads to plasticity, and potential therapeutic malleability, of the B cell compartment. DEGs that influence diversification checkpoints and BCR hyperresponsiveness can occur early, broadly, or exclusively after BCR stimulation, leading to pathogenesis characterized by ABC expansion in active cGVHD (right side).

DEGs in active cGVHD could be subdivided into major categories (Figure 10) that comprise intrinsic differences from a B cell–tolerant state (no cGVHD). The first category of DEGs emerge in B cells in early maturation states (DEGs in naive subsets, clusters 1, 2, 4, 6, and 8), with some of these DEGs maintained throughout B cell diversification (broadly altered DEGs). The second category of DEGs emerge only after some degree of BCR stimulation (DEGs in BCR-activated subsets, clusters 3, 7, 9, 10, and 5). Examples of unique transcriptional changes included *CKS2* and *GPR183*/*EBI2*, each overexpressed in active cGVHD from the earliest transitional/naive B cell subsets to the most differentiated subset, cluster 5, enriched for ABCs and CD27^+^ memory B cells. Such DEGs occurring from (at least) the time B cells first enter the circulation may cause epigenetic changes that alter subsequent tolerance checkpoints and potentially affect clonal diversity. Extrinsic factors in microenvironmental niches may be the initial drivers of such DEGs that lead to early loss in peripheral B cell tolerance.

The observed expansion of potentially pathogenic ABCs in patients with active cGVHD (Figures 5 and 6, and as modeled in Figure 10) is likely a consequence of gene alterations before and after BCR engagement occurring in a cumulative way. ABCs have emerged as important in both normal immunity and autoimmunity (24, 26, 35, 36) and may contribute to pathogenesis by producing alloantibodies after migrating to tissue sites where cGVHD manifests (11). The expansion of ABCs (including DN2) is driven by one or more costimulatory signals that cooperate with BCR stimulation (27, 87–89). Indeed, B cells isolated from patients with active cGVHD are hyperresponsive to minimal BCR ligation in synergy with costimulatory molecules including NOTCH2 and BAFF (9), processes likely driven in part by the increased BAFF/B cell ratio that occurs in patients with cGVHD (10, 11, 67, 90) and by niche production of BAFF in the SLO microenvironment (8). BAFF influence may additionally propagate by increases in BAFF receptors such as TACI/TNFRSF13B, as published (11) and herein described for B cell subsets. It is also noteworthy that multiple other TNF superfamily and TNF receptor superfamily members were increased DEGs in cGVHD B cell clusters, including the metabolically active clusters 9 and 10. Therefore, ongoing studies of these molecules as potential drivers of ABC expansion in cGVHD is warranted.

Plasticity or malleability has been demonstrated in newly formed B cells and during the generation of memory (91, 92). Our scRNA-Seq data set and supporting work provide insight into these processes (Figure 10, allo-HCT plasticity). Signature gene profiles defined 10 distinct B cell subsets (Figure 10) that continually developed and circulated under constant exposure to alloantigens. Allo-HCT patient memory B cell subsets had some remarkably distinct gene features, regardless of cGVHD status, compared with the same subsets from healthy individuals or patients chronically exposed to microbial antigens (Figures 3 and 4). In allo-HCT, striking differences in the proportion of B cells expressing some molecules that orchestrate exit from the BM and homing to/establishment in SLO niches, along with molecules affecting survival, suggest potential impacts on the clinical manifestations of cGVHD. These observations may additionally reflect the apparent maturation block associated with B cell hyperresponsiveness in patients with cGVHD (9) and may partially explain the paucity of CD27^+^ memory B cells in allo-HCT patients compared with HDs (Figure 5, B and C). The peripheral B cell pool in allo-HCT patients is thus potentially malleable (Figure 10, potential malleability of BCR-activated subsets), and therefore, possibly amenable to novel treatment strategies to prevent or treat cGVHD, while maintaining immunity against pathogens and possibly malignant cells (9). This unique plasticity of the B cell compartment in an allo-HCT environment can now be modeled based on our scRNA-Seq data.

Identification herein of certain molecules dysregulated in subsets of active cGVHD B cells is potentially novel, particularly those that regulate B cell movement and positioning within SLO. The identification of *GPR183*/*EBI2*, *P2RY8*, and *S1PR2* as DEGs is particularly remarkable, as each molecule is important for follicular movement and GC positioning of B cells (47, 69–72). The increase in *GPR183* across 5 B cell clusters, including transitional like subsets, suggests that strictly regulated tolerance mechanisms initiated as B cells first recirculate and enter the SLO microenvironment may be disrupted in patients with cGVHD. This is consistent with the notion that altered intrinsic functions of lymphocytes and stromal cells within SLOs disrupt normal architecture during follicle organization and GC development, potentially leading to premature displacement of B cells to extrafollicular spaces and altered tolerance (75). Remarkably, *GPR183*, *P2RY8*, and *S1PR2* were each DEGs in cluster 5, which was enriched for ABCs and DN2. *P2RY8* expression was additionally decreased in metabolically active cluster 9, which is intriguing given recent evidence that decreased B cell *P2RY8* propagates pathogenic antibodies and ABCs in humans with lupus or antiphospholipid syndrome (74). The ligands for each of these molecules are known, and in some cases inhibitors and agonists have been described, permitting future studies in mouse models of cGVHD and eventually clinical trials. This concept is supported by an observation in a mouse lung transplant model of bronchiolitis obliterans, a condition that frequently causes lung damage in patients with cGVHD, where inhibition of GPR183/EBI2 with a highly selective inhibitor reduces pulmonary lymphoid lesions and lung damage (93). Elucidating B cell transcriptional changes that incite cGVHD development, versus transcriptional changes that occur after cGVHD genesis and nevertheless contribute to ongoing pathogenesis in an unrelenting cycle, will remain an area of investigation.

While the implications of the findings herein remain to be fully realized, our data provide a foundation for future assessment of novel, potentially targetable pathways that alter B cell homeostasis during the diversification of B cells in the periphery when alloantigens or neo-autoantigens are prevalent. Mouse studies can now be launched that evaluate the hierarchy of these molecules in initiating or propagating loss of B cell tolerance. The ultimate benefit of this research will be the ability to design tailored therapies that eliminate or prevent the emergence of pathogenic B cells while retaining humoral immune responses required for long-term health.

## Methods

### Experimental procedures.

See Supplemental Methods for information on B cell isolation and culture, scRNA-Seq sample preparation, scRNA-Seq data analysis, dermal skin B cell isolation and scRNA-Seq analysis, qPCR, flow cytometry and PhenoGraph analyses, phosphoarray sample preparation and analysis, and Western blot analysis. Original, uncropped array and blot images are included in the online supplemental materials.

### Statistics.

For scRNA-Seq data, clustering analysis was conducted using R statistical environment and extension package Seurat v3.1.4 (32). DEG analysis with respect to disease status (active cGVHD vs. no cGVHD) within each cluster was performed based on the read counts across all cells within each sample using the Bioconductor package DESeq2 (version 1.26.0). Global interaction between disease status and the B cell clusters also was analyzed. Multiple testing was accounted for within the framework of control of the false discovery rate using the qvalue approach (version 2.14.1) (https://github.com/StoreyLab/qvalue; commit ID 9b3f9a8). Statistical tests used in supporting experiments are described in the figure legends. For all comparisons in the scRNA-Seq data set, an adjusted *P* value of less than 0.05 was considered statistically significant. For all other statistical comparisons, standard *P* values were used to determine significance, as follows: *, *P* < 0.05; **, *P* < 0.01; ***, *P* < 0.001.

### Study approval.

Deidentified whole blood samples or apheresis samples were obtained from allo-HCT patients under IRB protocols from Duke University, the National Cancer Institute (NIH), or the Dana-Farber Cancer Institute. Informed written consent was obtained from all patients, with the scope of the research and the minimal risks fully explained. Beyond some basic inclusion and exclusion criteria given to clinicians for obtaining PBMC samples for study (including cGVHD status), samples were obtained and chosen for use in a blinded fashion. A skin punch biopsy was collected from an adult allo-HCT patient with active cGVHD, and from surgically discarded abdominoplasty tissue from an HD, in accordance with an IRB protocol approved by Duke University.

### Data availability.

All blood B cell scRNA-Seq library data from the 8 allo-HCT patients (16 samples) are available through the Gene Expression Omnibus database (https://www.ncbi.nlm.nih.gov/geo/) under accession code GSE161343. The R scripts to reproduce the analyses of these scRNA-Seq data are available at this site: https://gitlab.oit.duke.edu/dcibioinformatics/pubs/sarantopoulos-10x-cgvhd/; commit ID 93fac70a. The Supporting Data Values file is included in the online supplemental materials.

## Author contributions

JCP and SS conceived the study; JF, MRL, and KO performed scRNA-Seq data curation; JF, DZ, MRL, XQ, JX, and KO performed scRNA-Seq formal analysis; SS, SJB, and JV acquired funding; JCP, RAD, HS, JYZ, ANS, and IMCC investigated; JF, DZ, MRL, RAD, XQ, JV, JX, and KO developed methodology; VTH, KSW, JJR, SZP, FTH, MEH, DAR, and ARC provided resources; JF, DZ, MRL, XQ, JX, and KO developed software; SS and KO supervised; JF, MRL, XQ, and KO performed validation; JCP, JF, JV, KO, and SS performed visualization; JCP, SS, and KO wrote the original draft; and JF, DZ, MRL, RAD, XQ, JYZ, JV, SJB, WJ, WCM, NJC, ARC, and JX reviewed and edited the manuscript.

## Supplementary Material

Supplemental data

Supplemental table 3

Supplemental table 7

Supporting data values

## Version 1 05/02/2023

In-Press Preview

## Version 2 06/08/2023

Electronic publication
