# Supplementary material for: Single-cell landscape analysis unravels molecular programming of the human B cell compartment in chronic GVHD
Source: JCI Insight. 2023 Jun 8;8(11):e169732. doi: 10.1172/jci.insight.169732 (PMC10393230; doi:10.1172/jci.insight.169732)

Supplemental Methods

Additional Information on Patient and HD blood samples

Single-cell RNA-Seq was performed on peripheral B cells from allo-HCT patients chosen at random, with the desire to gain new knowledge about the molecular mechanisms that emerge in the allo-HCT setting to drive B cell hyper-responsiveness and cGVHD development in some patients (loss of tolerance). Anonymous HD samples were buffy coats purchased from Gulf Coast Regional Blood Center (Houston, TX). Viably frozen PBMCs from allo-HCT patients or HDs were prepared by Ficoll-Paque® PLUS (GE Healthcare) gradient separation prior to storage in a liquid nitrogen freezer. For some experiments, purified B cells were additionally isolated immediately following PBMC gradient separation using a Human B Cell Isolation Kit II (Miltenyi Biotech), washed in cold Dulbecco's phosphate-buffered saline, and stored as B cell pellets at -80°C. The characteristics of allo-HCT patients who provided the samples used for single-cell RNA-Seq analysis and the samples used for additional experiments are described in Supplemental Tables 1 and 2, respectively.

B cell culture, single-cell RNA-Seq library preparation, and library sequencing

B cells were purified from viably cryopreserved allo-HCT patient PBMCs using the Human B Cell Isolation Kit II (Miltenyi Biotech). B cells were then cultured for 18 h at 5% CO_2 in RPMI-1640 medium containing 10% fetal bovine serum (FBS) and 55 µM 2-Mercaptoethanol (Gibco). In some wells, cells were treated with 100 nM ATRA (MP Biomedicals, cat# 190269) for the entire culture period. The cells were then harvested, and dead cells were removed using an EasySep™ Dead Cell Removal (Annexin V) Kit (STEMCELL Technologies, Inc.). Live B cells (in HBSS containing

2% FBS) were immediately transferred on ice to the Duke Molecular Genomics Core for 10X Genomics Chromium™ Single-Cell 3' library generation according to the manufacturer's instructions and the Core Facility's standard methods, targeting 10,000 B cells per sample. 10X Genomics libraries were then transferred directly to the Duke Center for Genomic and Computational Biology, Sequencing and Genomic Technologies Shared Resource, for sequencing. All individual 10X Genomics libraries (16 total) were pooled and then applied evenly across all 4 lanes of a NovaSeq S4 Flow Cell (Illumina) with 100-bp paired-end sequencing at a depth of ~700 million reads per sample (~70,000 reads per cell).

Single-cell RNA-Seq data analysis on purified B cells from allo-HCT patients

The raw data in BCL format were de-multiplexed into FASTQs by Illumina's bcl2fastq (https://support.illumina.com). The extraction of cellular barcodes and unique molecular identifiers (UMI sequences) and genome alignment of the biological sequences were performed using the Cell Ranger pipeline (version 2.1.1, https://support.10xgenomics.com) to generate a filtered sparse matrix of gene read counts per cell. The 16 individual matrices were integrated using Stuart et al.'s method to remove batch effects (1). Any cells in which fewer than 500 genes were detected and those with greater than 5% mitochondrial gene counts were considered poor quality and excluded from the analysis. Any genes detected in fewer than 150 cells were likewise excluded. Visualizations of single-cell-level transcription profiles in UMAP space using color was performed using R package Seurat v3.1.4 (1). Trajectory analysis was performed to infer the relative relationship among cell clusters over 'pseudotime' using the package Slingshot (2).

Comparison of allo-HCT patient single-cell RNA-Seq data to a publicly available dataset on purified B cells from HDs, HIV patients, and malaria-exposed individuals

To compare scRNA data from this study to data published by Holla *et al.* (3), both datasets were filtered for cell quality using the same thresholds (cells with more than 5% mitochondrial gene counts, counts from fewer than 200 genes, or counts from more than 2,500 genes were excluded). High-quality cells were then filtered to retain only B cells, with cell type inferred based on signature genes from CellAssign (4) and expression of the union of those signature genes and the top 2000 most variable genes (following variance stabilizing transformation (5)) using the R package scSorter (6). To normalize the datasets, gene counts per cell were transformed to counts per million reads mapped (CPM) using the Seurat R package. To compare gene expression, data from the present study and Holla *et al.* (3) were integrated following steps recommended by the developers of Seurat. Five cohorts were integrated to account for batch effects, as follows: Holla *et al*. data (3) were split by sample type (HD vs. HIV vs. Malaria), and allo-HCT data was split by the two sequencing runs. To investigate biologically-relevant genes across studies and sample types, we generated a heatmap with the following attributes: We identified each subset based on a detection threshold of 50 CPM for *ITGAX* and *CD27* expression. To illustrate distribution of gene expression, values were plotted lowest to highest for each gene of interest and within each B cell subset (*CD27*$^{+}$*ITGAX*$^{-}$, *CD27*$^{-}$*ITGAX*$^{+}$, and *CD27*$^{+}$*ITGAX*$^{+}$) and sample type. Populations with more than 100 B cells were randomly down-sampled to 100 cells. Data analyses and plot generation were carried out in the R statistical environment with packages from the comprehensive R archive network (CRAN; https://cran.r-project.org/) and the Bioconductor project (7). Code used to replicate the analyses and figures followed principles of reproducible analysis, leveraging knitr dynamic reports and git source code management (8).

Dermal skin cell isolation and single-cell RNA-Seq analysis

The skin biopsies were washed with cold DPBS and digested with 1X dispase (ThermoFisher Scientific) overnight at 4ºC. The dermis was mechanically separated from the epidermis and then digested at 37ºC and 5% CO2 with 0.25% trypsin/EDTA for 1.5 hr. Dermal cell suspensions were then passed through 70 µm and 40 µm cell strainers, then pelleted by centrifugation and resuspended in Keratinocyte-SFM (ThermoFisher Scientific) containing 0.4% BSA. Single-cell suspensions were then subjected to 10X Genomics Chromium library generation and Illumina sequencing as described above. Signature gene analysis was performed using the R package Seurat to identify B cells within dermal cell clusters. DEG analysis was performed using the R package DESeq2, as described for blood B cells above. The skin cell scRNA-Seq library data is available through the Gene Expression Omnibus (GEO) database (https://www.ncbi.nlm.nih.gov/geo/), accession ID GSE190338.

Quantitative PCR (qPCR)

Frozen (-80°C) B cell pellets were thawed on ice, and total RNA was isolated using the RNeasy Plus Mini Kit (Qiagen) and quantified with a Qubit® 3.0 Fluorometer (ThermoFisher). RNA (500 ng) was reverse-transcribed with an iScript cDNA Synthesis Kit (BioRad). *CKS2* gene expression was assessed by qPCR using *ACTB* (*β-ACTIN*) as the housekeeping gene. Primers were designed with the NCBI primer BLAST program: *CKS2*, forward 5'-ACGAGTACCGGCATGTTATGT-3' and reverse 5'-GCCTAGACTCTGTTGGACACC-3'; *ACTB*: forward, 5'-GCTGTGCTACGTCGCCCT-3' and reverse, 5'-AAGGTAGTTTCGTGGATGCC-3'.

Amplification was performed using the iTaq Universal SYBR® Green Supermix (Bio-Rad) with an annealing temperature of 60°C on an ABI StepOne Plus™ Real-time PCR System (ThermoFisher Scientific). Each 20-μl reaction contained 2.5 ng of cDNA, with each primer used at a concentration of 250 nM. Data analysis was performed using Step One Software v2.3 software, and relative quantitation of gene expression was achieved using the standard-curve method. Fold change in expression in each sample was calculated using the average gene expression in patients with No cGVHD.

Flow cytometry and PhenoGraph analysis

Viably frozen PBMCs from allo-HCT patients were surface stained using antibodies against CD19, CD11c (ITGAX), CD21, CD24, CD27, EBI2 (GPR183), and TACI, along with their corresponding isotype control antibodies. 7-AAD (BioLegend) was used to assess cell viability. PhenoGraph cluster analysis was then performed on the remaining 6 markers after first pre-gating on viable B cells ($CD19^{+}$ $7AAD^{-}$), using this available function in Flowjo in combination with R. A detailed summary of all antibodies used is as follows: CD19 Pacific blue (clone J3-119, Beckman Coulter, cat#A86355); CD24 BV510 (clone ML5, BioLegend, cat#311126); CD21 FITC (Bu32, BioLegend, cat#354910); TACI PE (clone 1A1, BioLegend, cat#311906), EBI2 (GPR183) PE (clone SA313E4, BioLegend, cat#368912); CD11c APC (clone 3.9, BioLegend, cat#301614); CD27 PE-Cy7 (clone O323, eBioscience, cat#25-0279-42); IgD APC-H7 (clone IA6-2, BD Biosciences, cat#561305). Isotype control antibodies used for flow cytometry were: Mouse IgG2a BV510 (clone MOPC-173, BioLegend, cat#400268); Mouse IgG1 FITC (clone MOPC-21, BioLegend, cat#400110); Rat IgG2a PE (clone R35-95, BD Biosciences, cat#553930); Mouse IgG2a PE (clone MOPC-173, BioLegend, cat#400214); Mouse IgG1 APC (clone MOPC-21,

BioLegend, cat#400120); Mouse IgG1 PE-Cy7 (clone MOPC-21, BioLegend, cat#400126); Mouse IgG2a APC-H7 (clone G155-178, BD Biosciences, cat#560897).

Phosphoarray analysis

Proteome Profiler™ Human Phospho-Kinase Array Kits (R&D Systems) were utilized according to the manufacturer's instructions to analyze whole cell lysates of purified, untreated B cell samples from patients with Active cGVHD or No cGVHD (n=3 each). Spot densities on the arrays were quantified using ImageJ software (https://imagej.nih.gov/ij/).

Western blot analysis

Frozen (-80°C) pellets of purified B cells from allo-HCT patients with Active cGVHD (n=4) or No cGVHD (n=4) were lysed using a commercially available whole cell lysis buffer (Roche Complete Lysis-M) with freshly added protease inhibitors (Roche). Protein lysates were then denatured and loaded into wells of a 4–12% Bis-Tris gel (ThermoFisher) for electrophoresis under reducing conditions. Proteins were transferred from the gels to a nitrocellulose membrane using dry electroblotting (20 V for 9 min). Membranes were blocked in 2% fish gelatin buffer for 75 min and then incubated with rabbit anti-human $P27^{KIP1}$ polyclonal antibody (C-19, Santa Cruz Biotechnology, cat#sc-528, lot#KO413) overnight at 4°C. The membrane was thoroughly washed in Tris-buffered saline containing Tween 20 (TBST) before incubation for 1 h with donkey anti-rabbit polyclonal secondary antibody labeled with a near-infrared fluorescent dye (IRDye® 680, LI-COR, cat#926-68073, lot#C50821-05). The membrane was then washed thoroughly in TBST,

and fluorescently labeled proteins were detected using an LI-COR Odyssey CLX Imaging System. Band densities were quantified using ImageJ software (https://imagej.nih.gov/ij/).

References for Supplemental Methods

1. Stuart T, Butler A, Hoffman P, Hafemeister C, Papalexi E, Mauck WM, 3rd, et al. Comprehensive Integration of Single-Cell Data. *Cell.* 2019;177(7):1888-902 e21.
2. Street K, Risso D, Fletcher RB, Das D, Ngai J, Yosef N, et al. Slingshot: cell lineage and pseudotime inference for single-cell transcriptomics. *BMC Genomics.* 2018;19(1):477.
3. Holla P, Dizon B, Ambegaonkar AA, Rogel N, Goldschmidt E, Boddapati AK, et al. Shared transcriptional profiles of atypical B cells suggest common drivers of expansion and function in malaria, HIV, and autoimmunity. *Sci Adv.* 2021;7(22).
4. Zhang AW, O'Flanagan C, Chavez EA, Lim JLP, Ceglia N, McPherson A, et al. Probabilistic cell-type assignment of single-cell RNA-seq for tumor microenvironment profiling. *Nat Methods.* 2019;16(10):1007-15.
5. Anders S, and Huber W. Differential expression analysis for sequence count data. *Genome Biol.* 2010;11:R106.
6. Guo H, and Li J. scSorter: assigning cells to known cell types according to marker genes. *Genome Biol.* 2021;22(1):69.
7. Gentleman RC, Carey VJ, Bates DM, Bolstad B, Dettling M, Dudoit S, et al. Bioconductor: open software development for computational biology and bioinformatics. *Genome Biol.* 2004;5(10):R80.
8. Ram K. Git can facilitate greater reproducibility and increased transparency in science. *Source Code Biol Med.* 2013;8(1):7.

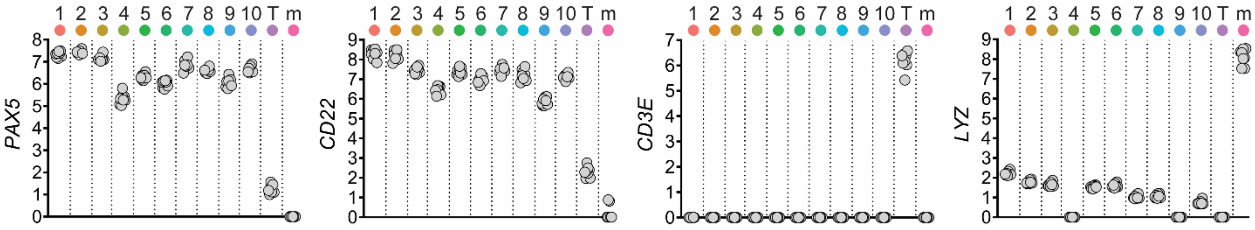

Supplemental Figure 1. Expression of genes with known specificity for B cells (*PAX5*, *CD22*), T cells (*CD3E*), or monocytes (*LYZ*) confirms cluster lineage identity. Log2 normalized expression values (Y-axis) for the lineage-specific genes of interest indicated. Numbers and colored dots at top represent the corresponding B cell cluster, with letters and colored dots representing clusters for residual T cells (T) and monocytes (m). Each symbol (gray circle) represents the results from one of the 8 total allo-HCT patients assessed.

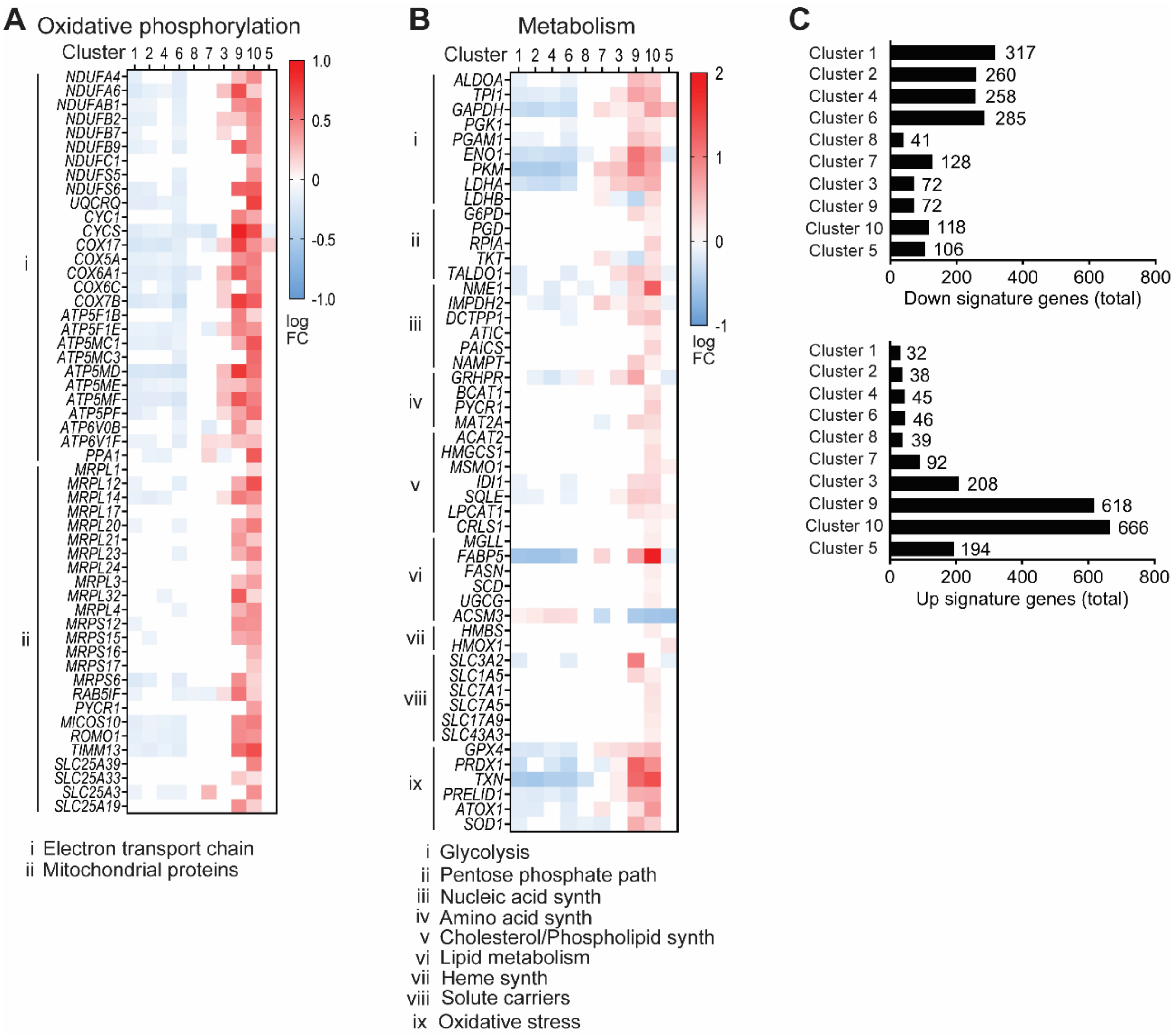

Supplemental Figure 2. Additional signature genes empirically assigned to biological pathways further corroborate the inter-relatedness and features of B cell development and activation among the 10 B cell clusters. (**A,B**) Signature genes were assessed in the biological pathways indicated above each heat map, and subdivided by the more specific pathways indicated by the roman numerals at left and corresponding keys below, as also described in Figure 2. (**C**) Total numbers of signature genes reaching significance within each cluster, being either decreased ('Down signature genes', top graph) or increased ('Up signature genes', bottom graph).

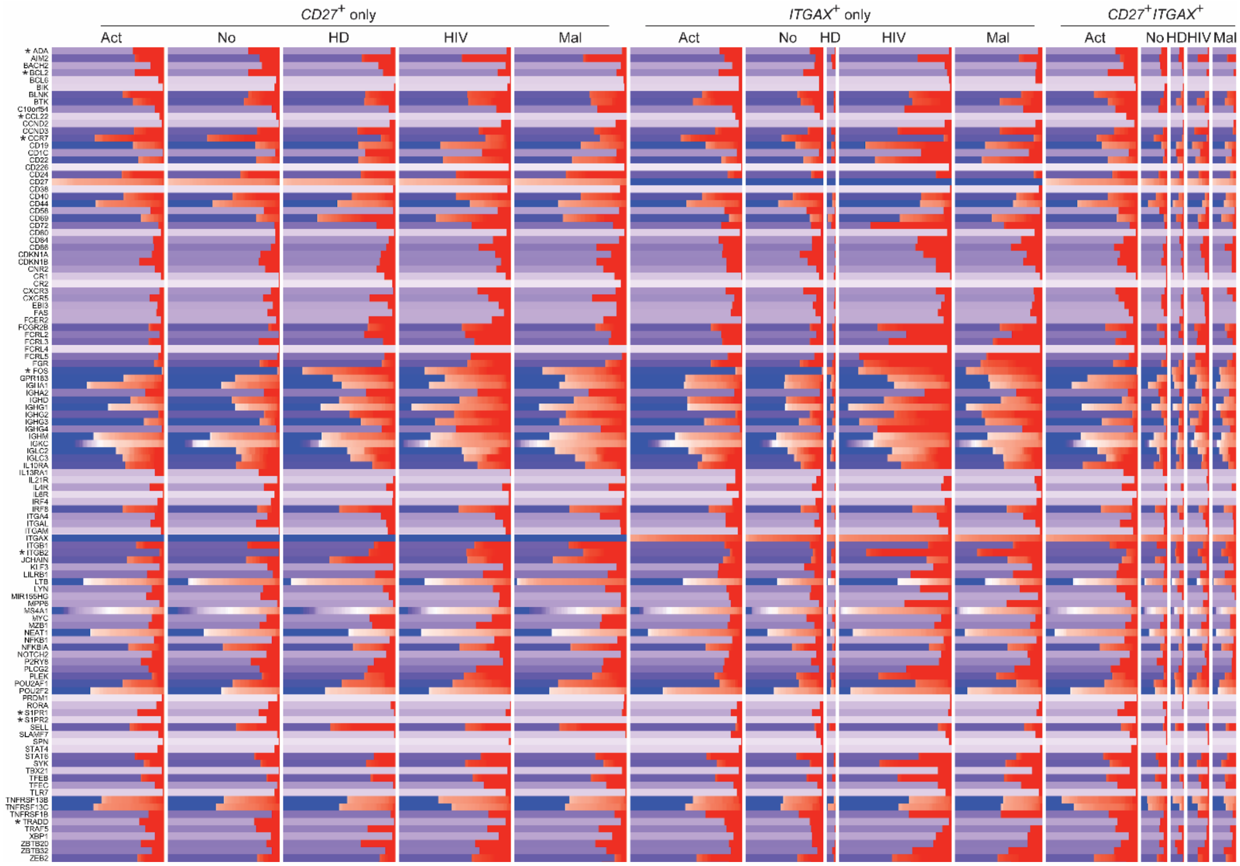

Supplemental Figure 3. The proportion of B cells expressing signature genes that define memory B cells subsets in allo-HCT patients compared to healthy donors or patients with infectious disease (HIV, Malaria). Genes on the Y-axis were chosen based on a thorough search of the literature for those known to be associated with memory B cell subsets. Memory B cell subsets were defined by *CD27* expression only (*CD27*$^{+}$*ITGAX*$^{-}$, left 5 columns), by *ITGAX* (*CD11C*) expression only (*CD27*$^{-}$*ITGAX*$^{+}$, middle 5 columns), or co-expression of both (*CD27*$^{+}$*ITGAX*$^{+}$, right 5 columns). Individual columns represent the patient or healthy donor group indicated (Act, Active cGVHD; No, No cGVHD; HD, healthy donors; Mal, Malaria). Colors represent log counts for each gene per million total reads in the same B cell, reported as log counts per million (log CPM). Red hues indicate that a B cell was positive for the gene of interest, and so the relative amount of red to blue is representative of the % positive B cells for that gene within the corresponding subset and group. Each column represents a maximum of 100 B cells, with fewer B cells shown in groups where < 100 B cells mapped to the subset of interest (indicated by a narrower column width). Where > 100 B cells were identified, 'downsampling' was performed to reduce the maximum number B cells shown in the column to 100 for visual purposes, as described in the Methods section. Asterisks indicate the genes also depicted in Figures 3 and 4.

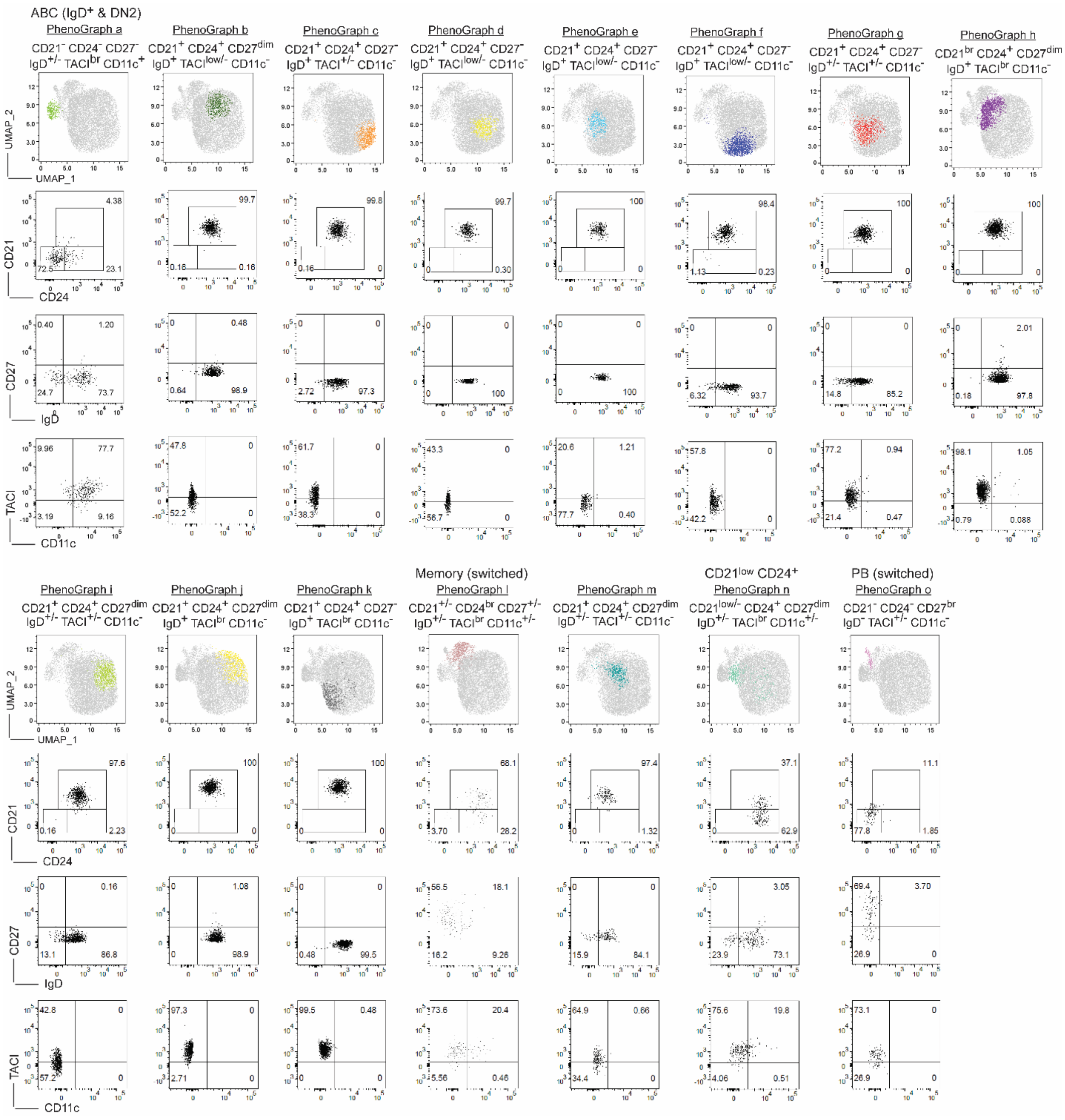

Supplemental Figure 4. Surface protein analysis by flow cytometry and PhenoGraph provides novel resolution of B cell subpopulations in allo-HCT patients. PhenoGraph results as described in Figure 6, A-E for the 15 identified B cell clusters from a representative allo-HCT patient in the 8-allo-HCT patient cohort. PhenoGraph UMAP projections are shown in the top panels, with the cluster of focus shown in color. The corresponding flow cytometric results for that cluster are shown in the dot plots below each UMAP. Numbers in the dot plots represent the frequency of B cells within the corresponding gate. ABC, memory, and other populations of interest including plasmablasts (PB) are labeled above their corresponding PhenoGraphs.

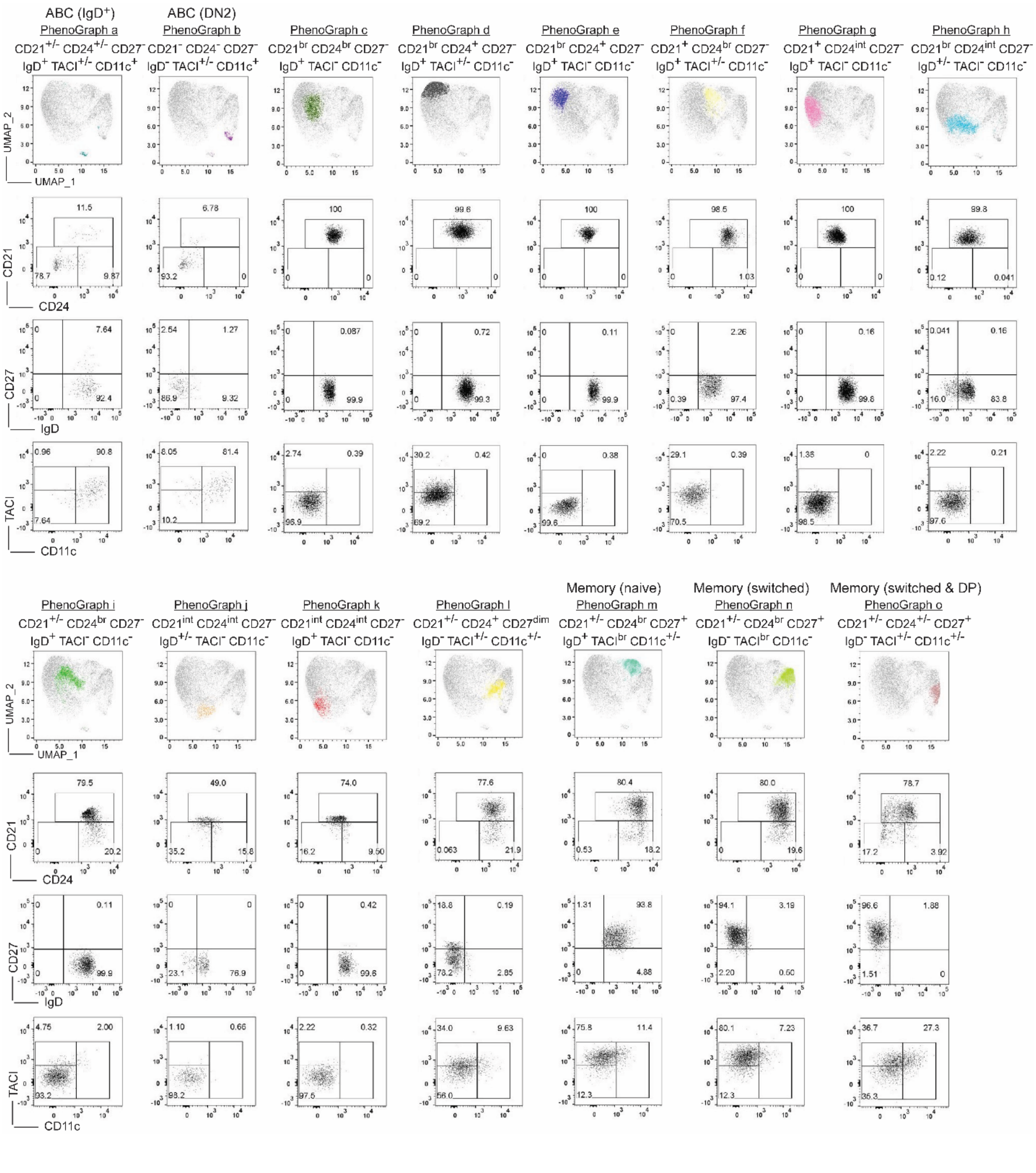

Supplemental Figure 5. Surface protein analysis by flow cytometry and PhenoGraph on HD peripheral B cells reveals subset distinctions with allo-HCT patients. PhenoGraph results as described in Figure 6, A-E and Supplemental Figure 4 showing representative results from 1 of 4 healthy donor (HD) PBMC samples assessed. ABC and memory populations of interest are labeled above corresponding PhenoGraphs.

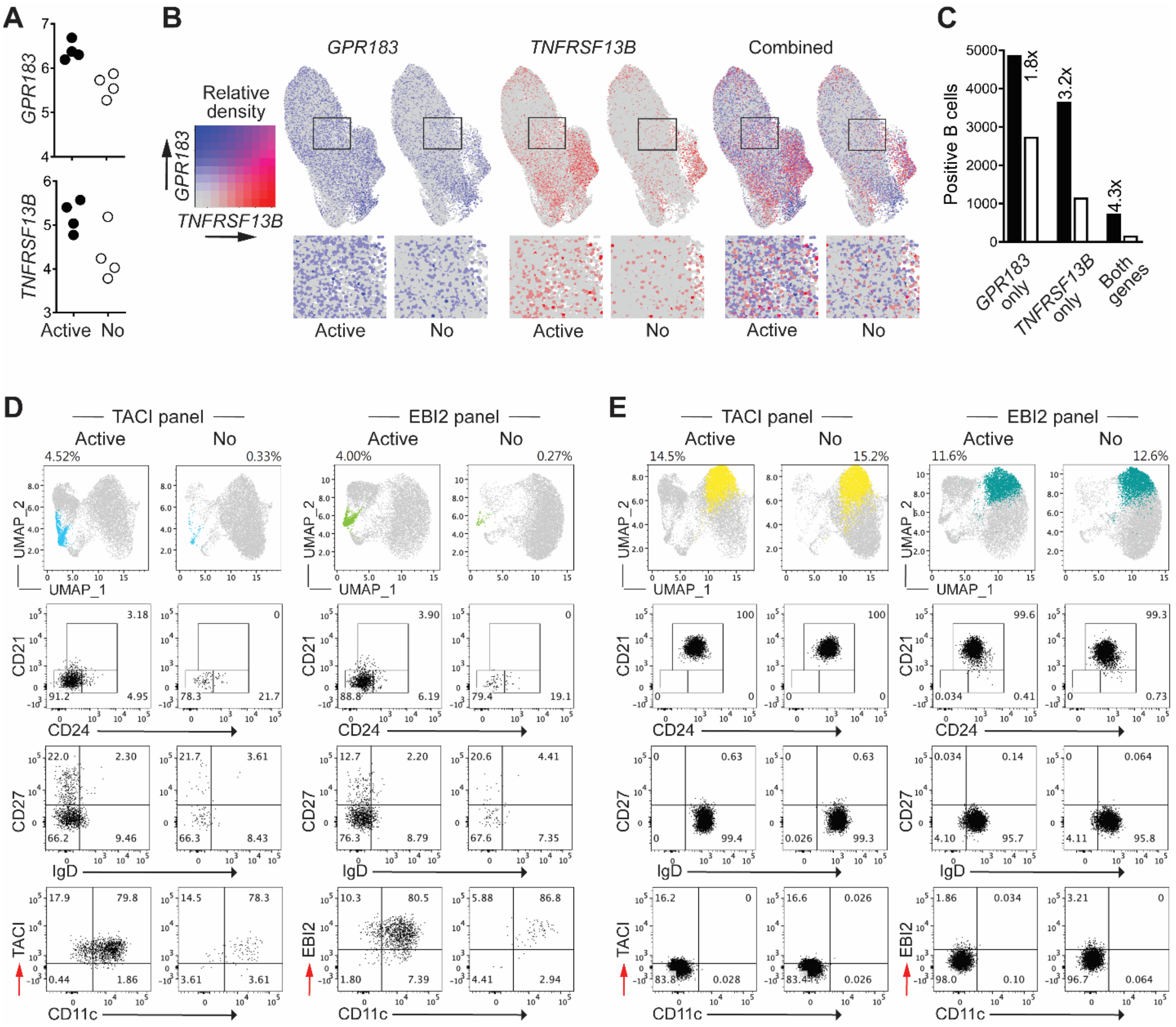

Supplemental Figure 6. B cells expressing *GPR183, TNFRSF13B* or both genes are increased in Active cGVHD. (**A**) Log2 normalized expression values for *GPR183* (*EBI2*) and *TNFRSF13B* (*TACI*) within Cluster 8 are shown, each correlating with the observed significant difference between allo-HCT patient groups within this cluster (Figure 7C and Supplemental Table 4). Each symbol represents one patient within the group indicated (Active, Active cGVHD; No, No cGVHD). (**B,C**) Co-expression analysis of *GPR183* and *TNFRSF13B* using Seurat provides insight into numbers of B cells separately expressing and co-expressing these genes. In (**B**), normalized expression density UMAP plots are shown for *GPR183* only (left panels), *TNFRSF13B* only (middle panels), and both genes in combination (right panels). The relative expression level of each gene in the density plots is based on the minimum to maximum color threshold range shown at the left. B cells co-expressing the genes at a high threshold level in the combination plots are depicted by magenta color. The boxed areas in the density plots approximate the B cells in Cluster 8 and are enlarged at the bottom to more easily visualize single B cells individually expressing or co-expressing the two genes. Active, Active cGVHD; No, No cGVHD. In (**C**), all B cells in the scRNA-Seq dataset individually expressing or co-expressing *TNFRSF13B* and *GPR183* were quantitated using this available function in Seurat and separated by patient group. Bars

represent total B cells expressing one or both genes as indicated by the X-axis labels, with the ratio of Active cGVHD B cells (filled bars) to No cGVHD B cells (open bars) indicated by the number above each condition, highlighting the increase in Active cGVHD. (**D,E**) PhenoGraph analyses of representative allo-HCT patients as described in Figure 6, A-E and Supplemental Figure 4 for identical flow cytometry panels with the exception of the inclusion of antibodies to either TACI (left plots) or EBI2 (right plots), as indicated by the red arrows. Cluster prediction was similar for the 2 panels, with 15 clusters predicted in both cases (UMAP plots at top, and not shown), enabling the comparison of similar clusters of B cells between panels. In (**D**), a similarly positioned and shaped cluster representing ABCs is present for both the TACI panel (blue) and EBI2 panel (green) that is otherwise phenotypically similar based on the other markers used, and is increased in frequency in Active cGVHD B cells (percentages indicated above each UMAP plot, and as with ABCs described in Figure 6, B and D and Supplemental Figure 4). For comparison, (**E**) shows a different cluster in the PhenoGraph projections (yellow, TACI panel; aqua, EBI2 panel) that has a naïve follicular B cell phenotype, lacks expression of TACI and EBI2, and is similar in frequency between patient groups.

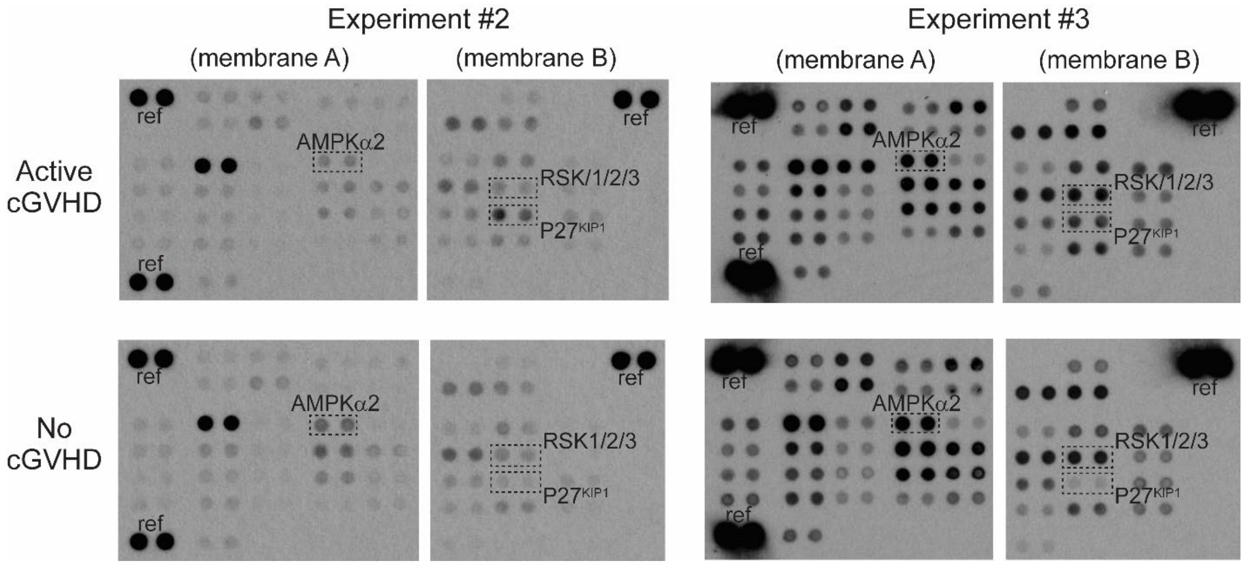

Supplemental Figure 7. Additional experiments represented in the bar graph in Figure 9G (along with the experiment shown in Figure 9F) demonstrating P27^{Kip1} phosphorylation at a major regulatory site is enhanced in Active cGVHD patient B cells. Phosphoprotein capture arrays (Proteome Profiler™ Human Phospho-Kinase Array) for detection of various intracellular signaling molecules phosphorylated on key sites involved in their regulatory activity, performed on whole cell lysates of purified, unstimulated B cells isolated from Active cGVHD and No cGVHD patient PBMC samples. Dashed boxes and protein IDs indicate the location and assay results for duplicate spots of capture antibodies against P27^{KIP1} (phospho-T198), AMPKα2 (phospho-T172), and RSK1/2/3 (phospho-S380/S386/S377, respectively). 'ref' indicates duplicate reference control spots on the arrays.

Supplemental Table 1. Characteristics of patients whose samples were used to generate the single-cell RNA-Seq dataset.

Characteristic	No cGVHD (*n*=4)	Active cGVHD (*n*=4)	*p*
Median age, years (range)	37 (30-48)	47 (22-61)	0.54
Sex, no. (%) of males	2 (50)	4 (100)	0.43
Median time after transplant, mos (range)	11.5 (10.0-12.3)	12.6 (11.1-13.9)	0.16
Conditioning regimen (%)			1
Non-myeloablative	4 (100)	4 (100)	
Source of graft (%)			1
Peripheral blood	4 (100)	4 (100)	
HLA matching (%)			1
Matched (MRD, MUD)	4 (100)	4 (100)	
Mismatched	0 (0)	0 (0)	
Immunosuppressive treatment (%)			
Steroidal (Prednisone)	0 (0)	2 (50)	0.43
Other IST	1 (25)	4 (100)	0.14
Initial disease (%)			
HD	3 (75)	1 (25)	
Thalassemia	1 (25)	0 (0)	
CML	0 (0)	1 (25)	
NHL	0 (0)	1 (25)	
Diamond Blackfan Anemia	0 (0)	1 (25)	

All patients provided consent, and all studies were approved under IRB protocols of Duke University, The National Institutes of Health, and The Dana-Farber Cancer Institute. Statistical comparisons between groups were performed using two-tailed, unpaired Student's t-test (age, time after transplant) or Fisher's exact test (other comparisons). cGVHD, chronic graft versus host disease; HLA, human leukocyte antigen; MRD, matched related donor; MUD, matched unrelated donor; IST, immunosuppressive therapy; HD, Hodgkin's disease; CML, chronic myeloid leukemia; NHL, non-Hodgkin lymphoma.

Supplemental Table 2. Characteristics of patients who provided samples for supporting experiments.

Characteristic	No cGVHD (*n*=19)	Active cGVHD (*n*=14)	*p*
Median age, year (range)	53 (24-73)	52 (22-69)	0.88
Sex, no. (%) of males	10 (58)	9 (64)	0.72
Median time after transplant, mos (range)	19 (11-73)	48 (13-112)	0.013
Conditioning regimen (%)			0.73
Myeloablative	11 (58)	7 (50)	
Non-myeloablative/reduced	8 (42)	7 (50)	
Source of graft (%)			1
Peripheral blood	17 (89)	12 (86)	
Bone marrow	2 (11)	2 (14)	
HLA matching (%)			0.42
Matched (MRD, MUD)	19 (100)	13 (93)	
Mismatched	0 (0)	1 (7)	
Immunosuppressive treatment (%)			
Steroidal (Prednisone)	2 (11)	8 (57)	0.007
Other IST	1 (5)	7 (50)	0.005
Initial disease			
ALL	2	1	
AML/AML from MDS	6	3	
Burkitt's lymphoma	0	1	
CLL	1	0	
CML	1	1	
DLBCL	1	0	
MDS/MF	4	3	
MM	1	0	
T-Cell lymphoma	1	1	
Other	2	4	

All patients provided consent, and all studies were approved under IRB protocols of Duke University, The National Institutes of Health, and The Dana-Farber Cancer Institute. Statistical comparisons between groups using two-tailed, unpaired Student's t-test (age, time after transplant) or Fisher's exact test (other comparisons). cGVHD, chronic graft versus host disease; HLA, human leukocyte antigen; MRD, matched related donor; MUD, matched unrelated donor; IST, immunosuppressive therapy; ALL, acute lymphoblastic leukemia; AML, acute myeloid leukemia; MDS, myelodysplastic syndrome; CLL, chronic lymphocytic leukemia; CML, chronic myeloid leukemia; DLBCL, diffuse large B-cell lymphoma; MF, myelofibrosis; MM, multiple myeloma.

Supplemental Table 4. DEGs with known function in B cells from Figure 7, C and D, categorized into their respective functional pathways. The direction of change in Active cGVHD compared to No cGVHD is indicated (Up DEG or Down DEG), along with the cluster(s) in which the DEG reached significance ($P_{adj} < 0.05$).

Follicle, GC organization				
Up DEG	Cluster(s)		Down DEG	Cluster(s)
GPR183	1 2 4 8 5		*P2RY8*	9 5
CXCR4	1 2 4		*LTB*	5
CXCR3	9		*S1PR2*	5
LGALS1	10			
SWAP70	10			
CD84	5			
Tolerance / Survival				
Up DEG	Cluster(s)		Down DEG	Cluster(s)
TNFSF10	2		*CLEC2D*	1 2 4 3 9 10
SLAMF7	9		*ZC3H12A*	1
TNFRSF1B	9		*TRADD*	9 5
TNFRSF10B	9		*FAS*	5
BCR signaling and activation				
Up DEG	Cluster(s)		Down DEG	Cluster(s)
BLNK	2 4 5		*CD79B*	1 2 4 8
FCGR2B	2 10 5		*PIM1*	1 2
FCRL4	9 10		*CD5*	1 3 10
FCRL5	9		*CAV1*	1 10
NR4A1	9		*MME*	10
NR4A3	9		*BIK*	5
LILRB1	10 5		*CR1*	5
AIM2	10			
CD180	10			
BTK	5			
CD72	5			
FCRL2	5			
KLF3	5			
Cell cycle / Proliferation				
Up DEG	Cluster(s)		Down DEG	Cluster(s)
CDKN1B	4 3		*CDKN1A*	1 2 4 5
CCND3	5			
Isotype switching / Antibodies				
Up DEG	Cluster(s)		Down DEG	Cluster(s)
TNFRSF14	1 2 4		*CD83*	1 2 4 7 3
TNFRSF13B	8 10		*IGHD*	1 2 8 3 9
POU2F2	3 9 10 5		*ADA*	4 10 5
MIR155HG	3		*IL4R*	8 7
TNFRSF12A	9 10		*IL21R*	3 10
SOCS3	9		*IL13RA1*	9 5
IL27RA	10		*BTLA*	9
ZBTB20	10		*CD40*	5
			CD80	5
			EBI3	5
			IGHE	5
			IL6R	5
			POU2AF1	5
			RORA	5
ABC development, function				
Up DEG	Cluster(s)		Down DEG	Cluster(s)
EGR3	9			
TBX21	5			
ZEB2	5			

Supplemental Table 5. Signature genes reaching significance in the skin cell scRNA-Seq dataset for the cluster identified as B cells. LogFC values represent the expression level of the gene of interest in the B cell cluster relative to clusters for all other skin cell lineages. Some genes with particular relevance to the B cell lineage are highlighted in yellow.

Gene	avg_logFC	pct.1	pct.2	p_val	p_val_adj	Additional description
IGKC	5.840324113	0.5	0.041	5.91E-69	1.35E-64	Ig kappa light chain
IGHG1	4.48271604	0.167	0.002	1.01E-127	2.31E-123	IgG1 heavy chain
IGHG3	3.890657725	0.241	0.003	6.89E-187	1.57E-182	IgG3 heavy chain
GZMB	2.908180123	0.556	0.049	6.89E-70	1.57E-65	
PLAC8	2.838899102	0.556	0.023	5.90E-146	1.35E-141	
IGHG4	2.568618359	0.167	0.004	2.98E-82	6.81E-78	IgG4 heavy chain
TCL1A	2.384288612	0.222	0.002	6.96E-224	1.59E-219	
LTB	2.31135052	0.537	0.097	3.62E-32	8.26E-28	
IRF8	2.065864406	0.37	0.071	2.23E-19	5.08E-15	
RGS2	1.968658624	0.426	0.175	8.35E-09	0.000190494	
ALOX5AP	1.923027434	0.407	0.098	1.78E-15	4.07E-11	
IGHM	1.877102155	0.204	0.005	4.60E-91	1.05E-86	IgM heavy chain
SPIB	1.844424329	0.296	0.012	1.58E-77	3.61E-73	
IRF7	1.835136805	0.352	0.12	6.22E-09	0.000141869	
PLD4	1.828996003	0.204	0.018	2.76E-24	6.30E-20	
GPR183	1.728443737	0.407	0.121	1.66E-11	3.79E-07	a.k.a. EBI2
MZB1	1.690247628	0.241	0.012	4.90E-54	1.12E-49	Marginal Zone B and B1 Cell-Specific Protein
ITM2C	1.655431722	0.481	0.14	1.21E-14	2.77E-10	
LILRA4	1.632328535	0.167	0.007	1.81E-46	4.13E-42	
AREG	1.587745467	0.315	0.089	1.18E-09	2.70E-05	
UCP2	1.483850276	0.407	0.125	9.11E-11	2.08E-06	
CYBA	1.446793637	0.611	0.334	2.11E-10	4.82E-06	
NCF1	1.393078587	0.37	0.059	1.48E-22	3.38E-18	
FCHSD2	1.382376321	0.278	0.09	4.37E-07	0.009977268	
BCL11A	1.355831393	0.241	0.024	6.30E-26	1.44E-21	
MPEG1	1.270548263	0.167	0.041	2.11E-06	0.048204372	
GAPT	1.242264259	0.204	0.009	1.19E-50	2.71E-46	
SELL	1.223975793	0.204	0.044	6.14E-09	0.000140149	
TYROBP	1.199836161	0.296	0.111	2.13E-06	0.048492729	
MS4A1	1.158058622	0.185	0.004	2.23E-87	5.08E-83	a.k.a. CD20
DERL3	1.094782318	0.222	0.005	1.49E-105	3.40E-101	
MARCHF1	1.094421244	0.241	0.046	4.01E-12	9.16E-08	
CD79A	1.057818646	0.259	0.002	1.26E-295	2.88E-291	Immunoglobulin-Associated Alpha
PRKCB	1.041053875	0.222	0.063	6.85E-07	0.015628941	
SIDT1	1.034585484	0.111	0.018	1.36E-07	0.003106374	
IRF4	1.032109579	0.185	0.028	3.30E-12	7.53E-08	
BANK1	1.023041358	0.204	0.016	1.68E-28	3.83E-24	B-Cell Scaffold Protein with Ankyrin Repeats
ADAM19	1.017732552	0.204	0.033	1.79E-12	4.08E-08	
NEK8	0.977455409	0.111	0.019	4.18E-07	0.009546737	
NUP210	0.89893479	0.148	0.026	1.45E-08	0.000331422	
CYBB	0.873497434	0.185	0.044	4.22E-07	0.009631	
FAM129C	0.859258535	0.167	0.003	1.15E-101	2.63E-97	
ARHGAP24	0.805663503	0.185	0.045	3.13E-07	0.007149293	
LY9	0.799524896	0.185	0.025	4.51E-14	1.03E-09	
TRAF4	0.792841267	0.204	0.04	8.04E-10	1.83E-05	
EVI2B	0.787922656	0.278	0.092	1.02E-06	0.023281725	
CD74	0.777773836	0.833	0.555	2.82E-09	6.42E-05	
RALGPS2	0.769373394	0.259	0.03	8.14E-23	1.86E-18	
HLA-B	0.684407312	0.907	0.753	5.73E-08	0.001307338	
BTK	0.639524637	0.185	0.034	6.32E-10	1.44E-05	Bruton Tyrosine Kinase
TLR10	0.61921292	0.167	0.004	9.48E-69	2.16E-64	

BLK	0.616529536	0.148	0.002	1.12E-109	2.55E-105	B Lymphocyte Kinase
LY86	0.58109155	0.167	0.025	1.60E-11	3.66E-07	
BLNK	0.574324462	0.167	0.019	7.72E-16	1.76E-11	B Cell Linker
DOK3	0.539394416	0.111	0.014	2.42E-09	5.53E-05	
ATP2A3	0.528046235	0.222	0.049	4.35E-09	9.94E-05	
ITGB7	0.462564875	0.185	0.046	1.20E-06	0.02727237	
GPR18	0.420638934	0.13	0.011	3.96E-16	9.04E-12	
LINC01781	0.411192206	0.111	0	2.61E-305	5.95E-301	
IGHA1	0.384504615	0.111	0.001	3.75E-159	8.57E-155	IgA heavy chain
MIR29B2CHG	0.37431651	0.185	0.041	4.87E-08	0.001110869	
HSH2D	0.366454394	0.241	0.031	2.65E-19	6.05E-15	
CD22	0.345489373	0.148	0.003	5.25E-75	1.20E-70	
TNFRSF13C	0.344446007	0.167	0.002	3.69E-113	8.43E-109	a.k.a. BAFFR
TNFRSF13B	0.335683275	0.222	0.006	8.11E-82	1.85E-77	a.k.a. TACI
FAM30A	0.313279998	0.148	0.001	9.17E-191	2.09E-186	
LINC00926	0.308727601	0.167	0.011	2.74E-26	6.26E-22	
ADAM28	0.306994007	0.185	0.019	3.27E-19	7.45E-15	
POU2AF1	0.268814264	0.204	0.001	1.23E-254	2.81E-250	a.k.a. BOB.1
TMEM156	0.266320283	0.204	0.016	1.56E-27	3.55E-23	

Supplemental Table 6. All DEGs reaching significance in the skin B cell scRNA-Seq dataset. LogFC values represent the change in Active cGVHD skin B cells compared to healthy donor skin B cells. Skin DEGs that were also DEGs in the blood B cell scRNA-Seq dataset are highlighted yellow, with the clusters reaching significance as indicated: 'Finding in Active cGVHD Blood B cells'.

Gene	avg_logFC	pct.1	pct.2	p_val	p_val_adj	Finding in Active cGVHD Blood B cells	Additional description
PLEKHO1	1.731832406	0.833	0	1.25E-10	2.85E-06		
MS4A1	1.970212715	0.75	0.024	2.29E-08	0.000522363		a.k.a. CD20
AES	1.886489815	0.75	0.071	8.76E-07	0.019996573		
SH3BGRL3	1.804945432	0.833	0.095	6.16E-08	0.001404755		
POU2F2	1.748266571	0.75	0.024	9.35E-09	0.000213346	Up in Clusters 3, 9, 10 and 5	a.k.a. OCT2
COTL1	1.693307692	0.75	0.048	5.78E-08	0.001317887		
CD69	1.606726935	0.75	0.071	2.07E-06	0.047341368		
GMFG	1.594499772	0.75	0.024	9.35E-09	0.000213346		
COMMD6	1.587647041	0.917	0.119	2.23E-08	0.00050919	Up in Cluster 8	
GGA2	1.573872227	0.5	0	1.71E-06	0.038925645		
HLA-DRB5	1.547592835	0.917	0.048	7.27E-09	0.000165891		
NOP14	1.536044844	0.5	0	1.71E-06	0.038925645		
PRR13	1.502403175	0.667	0	1.71E-08	0.000389903		
CD79B	1.500873877	0.75	0.119	1.53E-06	0.035026517		Ig-Beta
HNRNPDL	1.479502198	0.833	0.095	6.16E-08	0.001404755		
UIMC1	1.441756297	0.583	0	1.77E-07	0.004041391		
STX12	1.432046578	0.667	0	1.71E-08	0.000389903		
RPL36A	1.425296202	0.833	0.095	3.90E-08	0.000890461		
BASP1	1.401746836	0.75	0.048	1.11E-07	0.002538172		
ATAD1	1.375997106	0.5	0	1.71E-06	0.038925645		
CRIP1	1.323870643	0.75	0.024	1.60E-08	0.000366112		
ATP2B1	1.286889212	0.583	0.024	1.25E-06	0.028457219		
HIST1H4C	1.283036004	0.75	0.071	2.66E-07	0.006063075	Up in Clusters 9 and 10	
GPR18	1.277021074	0.583	0	1.77E-07	0.004041391	Up in Clusters 1 and 5	
RPL23A	1.271659998	1	0.405	2.08E-06	0.047378048	Up in Cluster 8	
BTF3	1.220760448	0.917	0.167	7.05E-07	0.016078078		
PNISR	1.215349733	0.833	0.095	1.30E-07	0.00296091		
CD40	1.215322917	0.833	0.095	1.30E-07	0.00296091		
SIPA1	1.185170632	0.667	0	1.71E-08	0.000389903		
CAPZA1	1.182468362	0.667	0.048	5.15E-07	0.011758898		
EIF4A1	1.178192543	0.75	0.071	3.09E-07	0.007057947		
CD24	1.156117001	0.667	0.024	2.26E-07	0.005161888		
BANK1	1.146682611	0.75	0.048	2.48E-07	0.005649835		B-Cell Scaffold Protein with Ankyrin Repeats
UQCRB	1.138665328	0.917	0.143	6.92E-07	0.015788036		
PDCD4	1.121603609	0.833	0.071	9.26E-08	0.002113134		
EZR	1.112715616	0.833	0.119	1.97E-06	0.044950962		
NDUFC2	1.105321475	0.75	0.071	2.28E-07	0.005204314		
C4orf48	1.091497768	0.583	0.024	1.25E-06	0.028457219		
IRF1	1.081771718	0.833	0.119	1.33E-06	0.030299525		
TRAF3IP3	1.061680721	0.667	0.024	1.13E-07	0.002576638		
U2SURP	1.056527054	0.833	0.095	1.12E-07	0.002554318		
RSRC1	1.045776177	0.583	0.024	1.25E-06	0.028457219		
GRK2	1.032408611	0.583	0.024	2.06E-06	0.047109747		
ZFAND6	1.027058439	0.583	0.024	2.06E-06	0.047109747		
MARCKSL1	1.006574335	0.667	0.024	3.77E-07	0.008601702		
TNFRSF13C	1.003644959	0.667	0.024	1.60E-07	0.003654234		a.k.a BAFFR
TOMM22	0.995554389	0.75	0.024	4.61E-08	0.001051747		
RPL17	0.980823901	0.75	0.119	1.34E-06	0.030592196		
MYCBP2	0.977666984	0.75	0.095	1.70E-06	0.038863573		
CMTM6	0.969105333	0.75	0.071	1.56E-06	0.035632785		
ADAM28	0.969034967	0.667	0.048	6.04E-07	0.0137911		
FCRL2	0.960641873	0.583	0	1.77E-07	0.004041391	Up in Cluster 5	
LINC00926	0.95768142	0.667	0.024	1.90E-07	0.00434529		
TELO2	0.934612879	0.5	0	1.71E-06	0.038925645		
ARF6	0.901562563	0.667	0.048	5.15E-07	0.011758898		
PCSK7	0.900022264	0.583	0	1.77E-07	0.004041391		
BDP1	0.899996906	0.75	0.071	6.53E-07	0.014909069	Up in Cluster 9	
ACTR3	0.891973675	0.833	0.095	6.29E-07	0.014348891		
TRAF5	0.882415435	0.583	0	1.77E-07	0.004041391		
SP100	0.881179509	0.75	0.048	2.11E-07	0.004822465		
FAM107B	0.880310967	0.667	0.048	7.08E-07	0.016159769		
SP140	0.873552264	0.583	0	1.77E-07	0.004041391		
GRK6	0.865769615	0.667	0.071	2.08E-06	0.047445504		
DRAM2	0.851895555	0.667	0.048	5.15E-07	0.011758898		
TMBIM4	0.849998071	0.75	0.071	3.60E-07	0.008209564		

CAPG	0.848095752	0.75	0.071	5.63E-07	0.012858288		
LIMD2	0.843313942	0.75	0.071	5.63E-07	0.012858288		
COX8A	0.841398255	0.833	0.095	4.75E-07	0.010838304		
ZNF655	0.84068727	0.667	0.024	4.46E-07	0.010177604		
LAT2	0.832692064	0.583	0.024	1.25E-06	0.028457219		
PHF20	0.795379869	0.75	0.048	1.80E-07	0.004112769		
SNX5	0.792810403	0.5	0	1.71E-06	0.038925645		
PSMA3-AS1	0.786434561	0.583	0	1.77E-07	0.004041391		
RAP1B	0.764140085	0.75	0.071	1.01E-06	0.023130948		
IFT57	0.761529181	0.5	0	1.71E-06	0.038925645	Up in Cluster 3	
SNX2	0.736198813	0.75	0.071	7.57E-07	0.017273273		
TCP1	0.72581675	0.667	0.048	1.13E-06	0.025856449		
DGKD	0.664724383	0.5	0	1.71E-06	0.038925645		
TSC22D4	0.664253013	0.5	0	1.71E-06	0.038925645		
IRF2	0.655922604	0.5	0	1.71E-06	0.038925645		
TMC6	0.644492191	0.583	0	1.77E-07	0.004041391		
PXK	0.64226044	0.5	0	1.71E-06	0.038925645		
SSBP1	0.636605768	0.833	0.048	2.28E-08	0.000519848		
CCDC88B	0.629988365	0.583	0.024	1.25E-06	0.028457219		
HIGD2A	0.629139979	0.833	0.143	1.74E-06	0.039774971		
ARHGAP25	0.624924796	0.5	0	1.71E-06	0.038925645		
CMTM7	0.605603609	0.5	0	1.71E-06	0.038925645		
BRD4	0.602992705	0.5	0	1.71E-06	0.038925645		
ANKRD10	0.602691643	0.667	0	1.71E-08	0.000389903		
SWAP70	0.590166501	0.75	0.024	4.61E-08	0.001051747	Up in Cluster 10	Switching B-Cell Complex Subunit 70
PLEKHB2	0.578750717	0.5	0	1.71E-06	0.038925645		
FCRLA	0.576012988	0.5	0	1.71E-06	0.038925645		
TERF2	0.57600478	0.583	0	1.77E-07	0.004041391		
ARFGAP2	0.572533511	0.583	0	1.77E-07	0.004041391		
LINC00513	0.571454147	0.583	0	1.77E-07	0.004041391		
IL16	0.563591483	0.5	0	1.71E-06	0.038925645	Up in Cluster 4	Interleukin 16
REL	0.548459613	0.75	0.048	7.33E-07	0.016715073		
AIM2	0.543726595	0.583	0.024	1.25E-06	0.028457219	Up in Cluster 10	Absent In Melanoma 2
PHTF2	0.517004237	0.5	0	1.71E-06	0.038925645		
SFSWAP	0.514465126	0.583	0	1.77E-07	0.004041391		
MED28	0.508644339	0.583	0	1.77E-07	0.004041391		
PTPN11	0.494422297	0.5	0	1.71E-06	0.038925645		
RAB5IF	0.47342014	0.583	0.024	1.25E-06	0.028457219		
PLEKHG1	0.467787339	0.5	0	1.71E-06	0.038925645		
GABARAPL2	0.44397426	0.75	0.071	5.63E-07	0.012858288		
PTBP3	0.427655806	0.5	0	1.71E-06	0.038925645		
DAZAP2	0.413067189	0.75	0.071	6.53E-07	0.014909069		
SELL	0.35536355	0.75	0.048	7.33E-07	0.016715073		
GGA1	0.35047393	0.667	0.048	1.80E-06	0.041033934		
LY86	0.322450642	0.667	0.024	4.46E-07	0.010177604		

Supplemental Table 8. DEGs identified in the bulk-like analysis shown in Figure 9B, assigned to their respective biological pathways after interrogation of the GO database. *Typical direction of impact on cell activation, differentiation or survival based on GO annotation. '?' indicates potential direction based on a thorough search of the available literature. DEGs from Figure 9B without an assigned classification to at least one GO Qualified Term/pathway were omitted from the table.

	Up in Active cGVHD		Down in Active cGVHD	
General Pathway	Gene ID	Impact*	Gene ID	Impact*
Cell Cycle	*CDCA4*	Positive	(no genes)	
	CETN3	Positive?		
	CKS2	Positive		
	NUMA1	Positive		
	SPDL1	Positive		
Cytoskeletal Reorganization	*ABI1*	Positive	*DLGAP3*	Negative?
	RHOQ	Positive	*WASF1*	Positive
	SKAP2	Positive		
	UGT8	Positive		
GPCR / GTPase	*GPR18*	Positive	*ARRDC3*	Negative
	GPR65	Negative?		
	RIN3	Positive?		
Immune Response	*CD48*	Positive	*CD5*	Negative
	FCRL4	Positive		
Kinase/Phosphatase Activity	*PPP1R16B*	Positive?	*PHKG2*	Positive?
	PTPRS	Negative	*PRKRA*	Negative
Metabolic processes	*GBGT1*	Positive?	*ACADVL*	Positive?
	KCNK6	Positive	*ATP6V1C2*	Positive?
			KRTCAP2	Positive?
			SLC50A1	Positive?
mRNA Splicing; Translation	*IVNS1ABP*	Positive	*MRPL28*	Positive?
	PDCD7	Negative?		
	RPL8	Positive?		
Protein/Amino Acid Transport	*ARCN1*	Positive?	*ARL17A*	Positive?
	AP4B1	Positive?	*CHMP6*	Positive?
	KCTD7	Positive?	*VPS26B*	Positive?
	PIGF	Positive		
	SLC15A2	Positive		
	SLC35D1	Positive?		
	TOMM20	Positive		
	TRMT1L	Positive?		
Protein Stability; Proteolysis	*ADAMTS6*	Negative?	*RHBDD2*	Positive?
	BAG2	Positive?	*UBE2J2*	Positive?
	CHORDC1	Negative?		
	DNAJB4	Negative?		
	FANCL	Positive		
	OTUD1	Positive?		
	PHF23	Positive?		
	TRIM4	Negative?		
	UBE2T	Positive		
Survival	*APAF1*	Negative	(no genes)	
	DEDD2	Negative		
	IFIT2	Negative		
Transcriptional Regulation	*ARID1A*	Positive	*FAAP24*	Negative?
	ASF1A	Positive	*HMGB2*	Positive?
	ATF2	Positive	*HMGB3*	Positive?
	FLYWCH1	Positive	*HMGXB4*	Negative?
	JMJD6	Positive	*NFKBIA*	Negative
	KMT2C	Positive	*NSMCE4A*	Negative?
	LMO4	Positive	*PCIF1*	Negative
	LRIF1	Positive	*POLR2M*	Negative
	MAT2A	Positive	*ZKSCAN2*	Negative?
	N6AMT1	Positive	*ZNF350*	Negative
	NAB2	Negative	*ZNF441*	Negative?
	NUFIP2	Positive		
	PURA	Positive?		
	TAF1A	Positive		
	TSC22D2	Negative?		
	ZBED2	Positive		
	ZBTB4	Negative		
	ZNF322	Positive		
	ZNF827	Positive		

Phosphoarray original film: Figure 9F

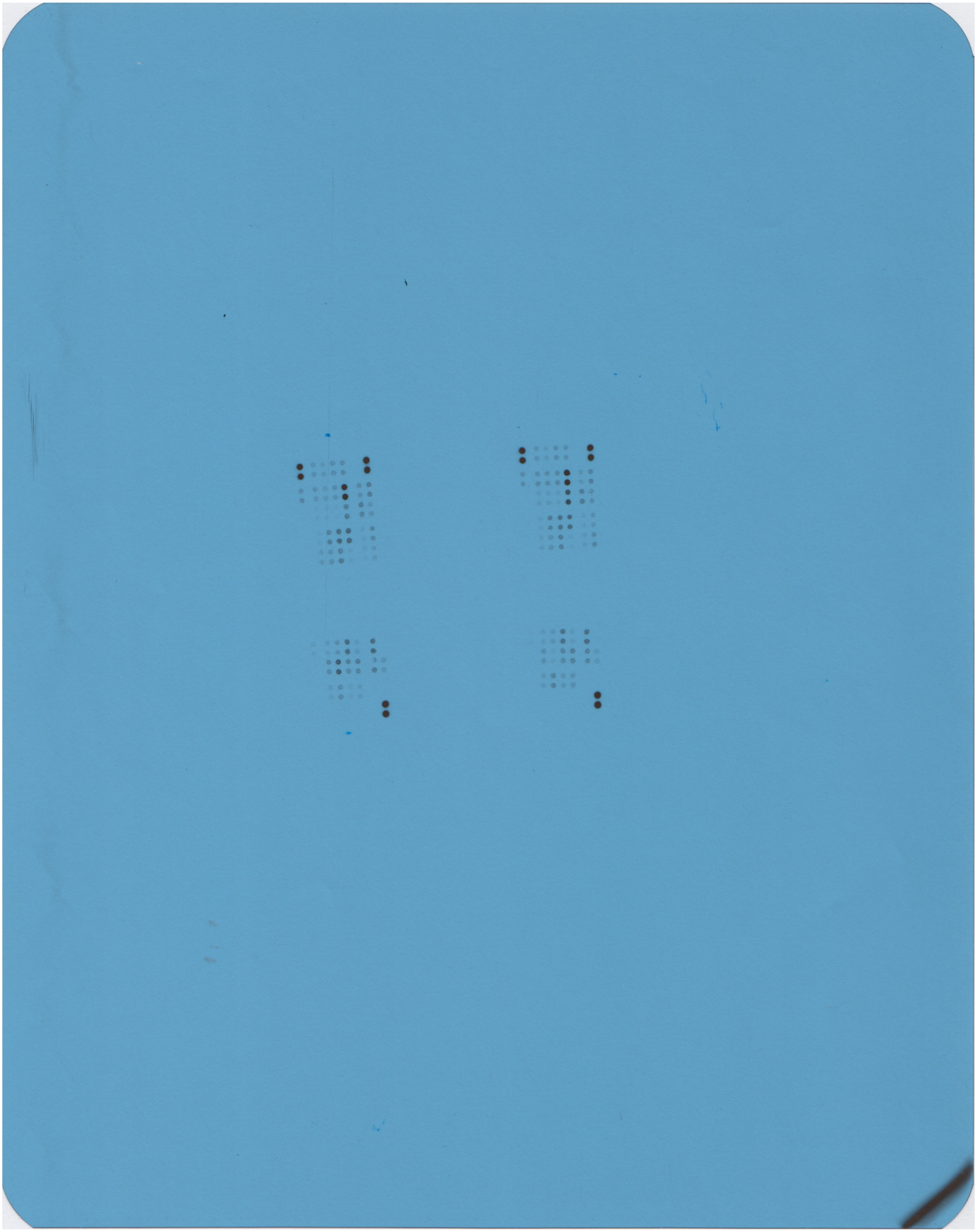

Phosphoarray original film: Supplemental Figure 7, Experiment 2

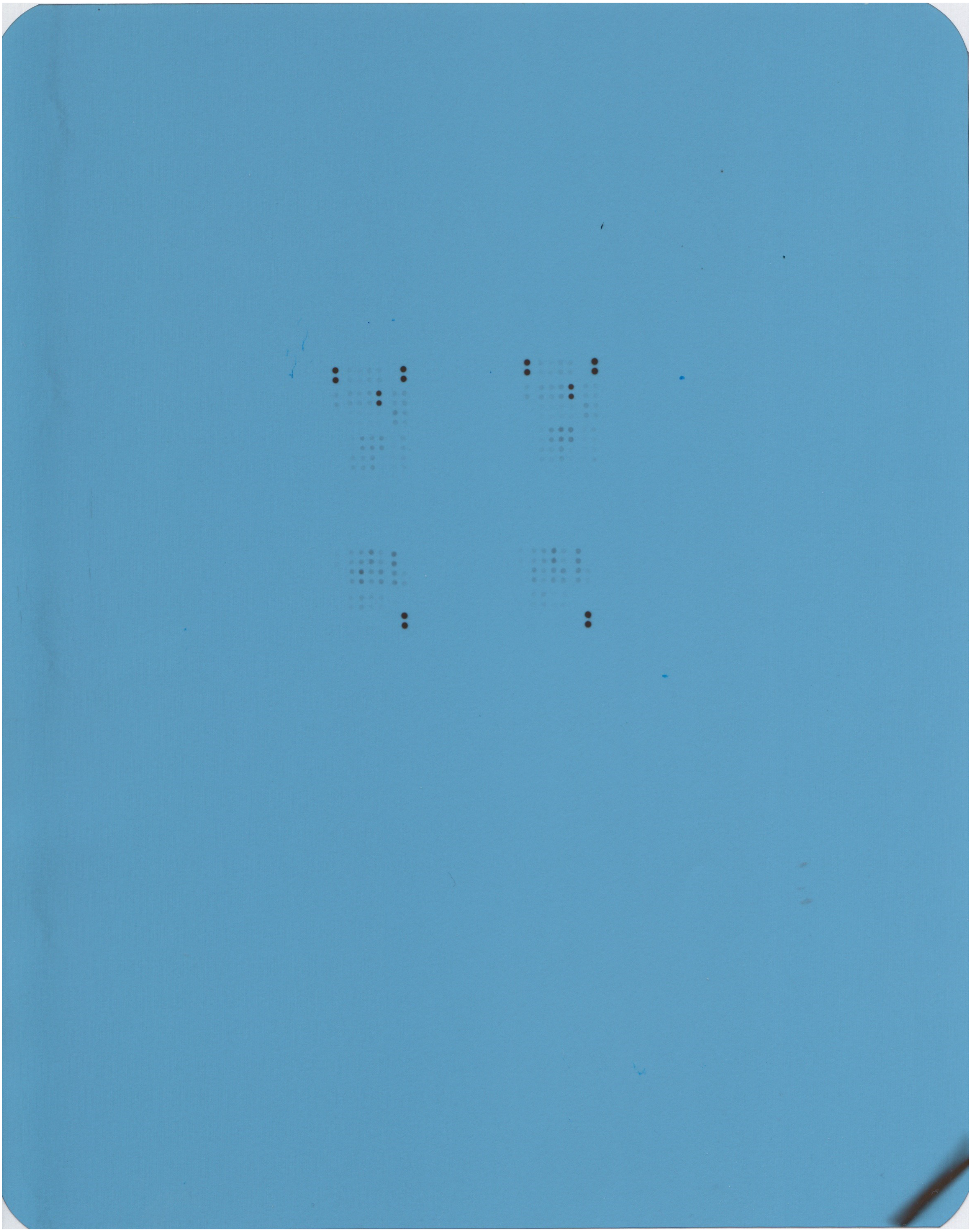

Phosphoarray original film: Supplemental Figure 7, Experiment 3

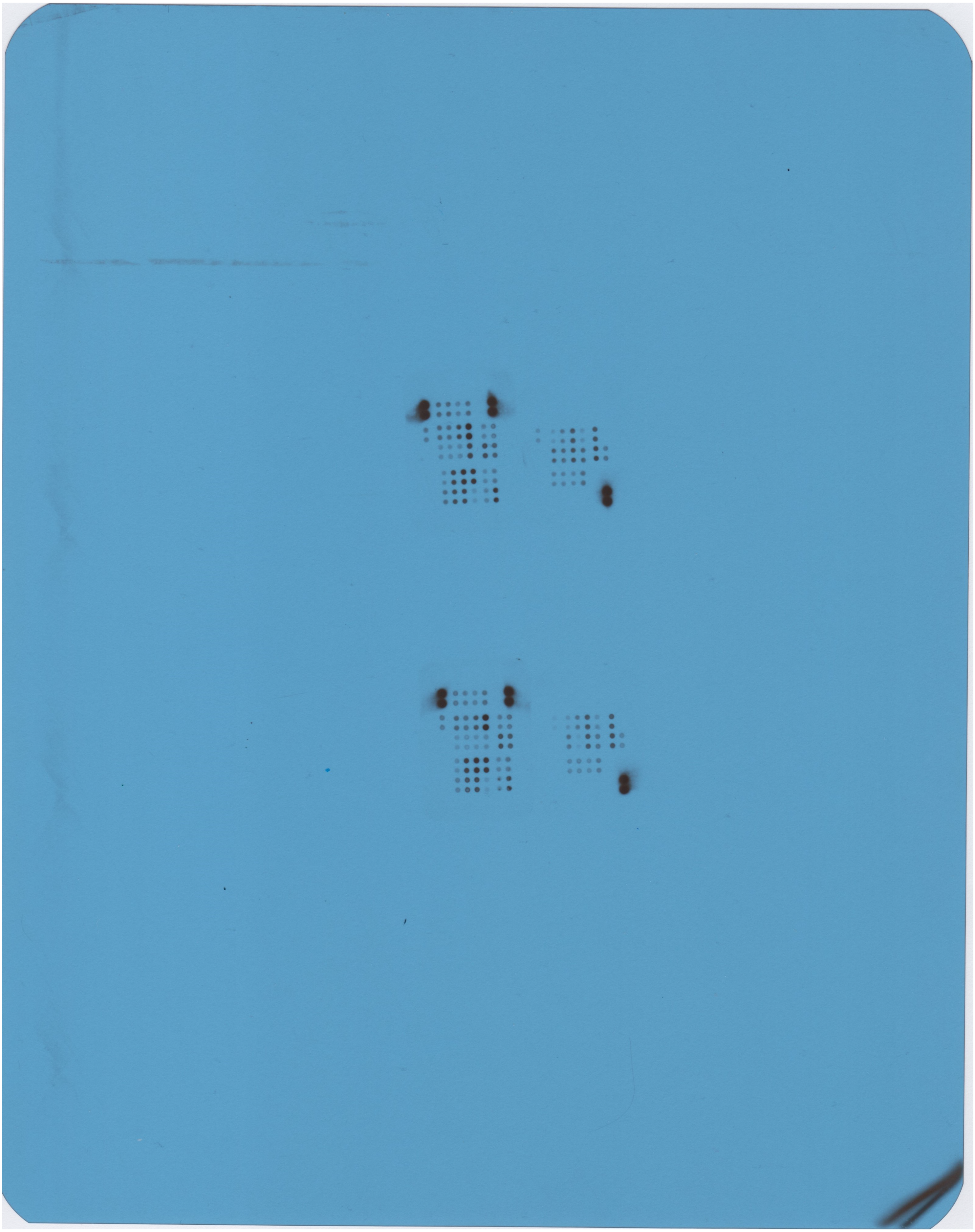

Western blot uncropped scans (converted to B/W): Figure 9H

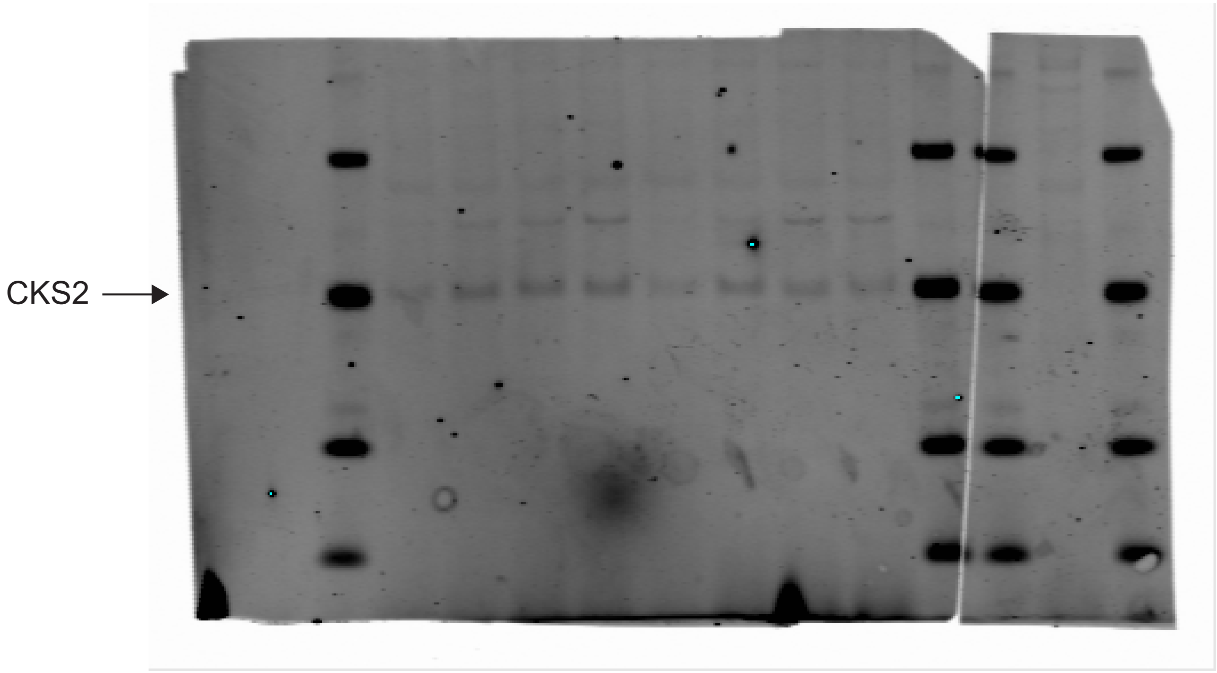

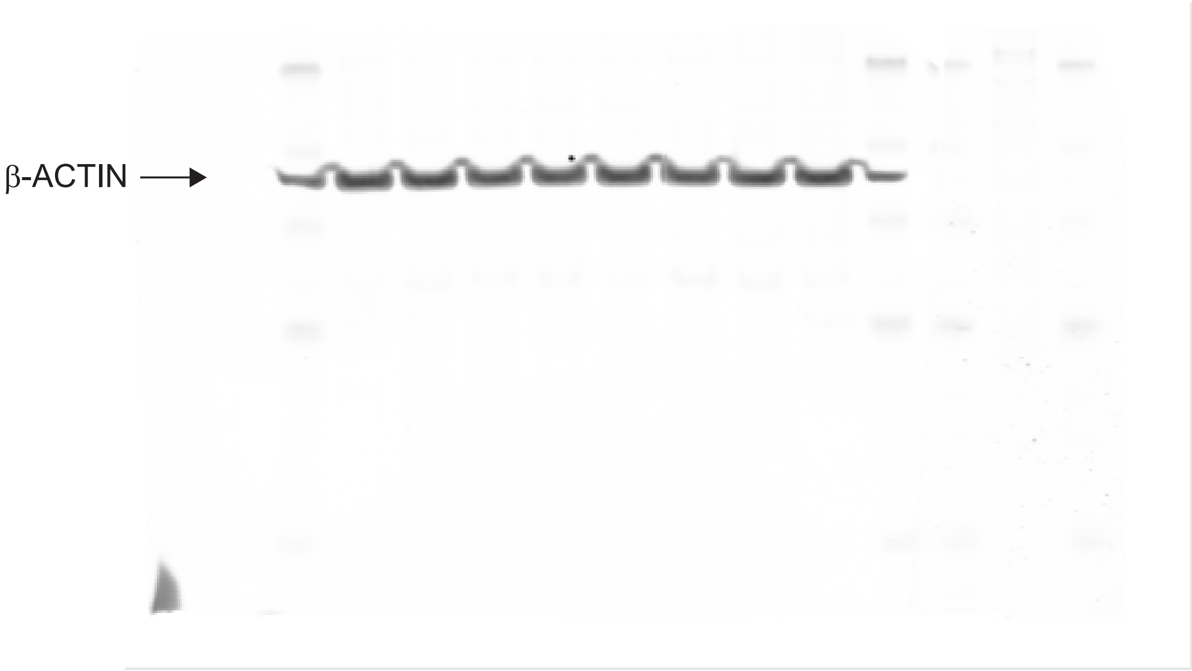

Western blot uncropped scans (original color): Figure 9H

CKS2 →

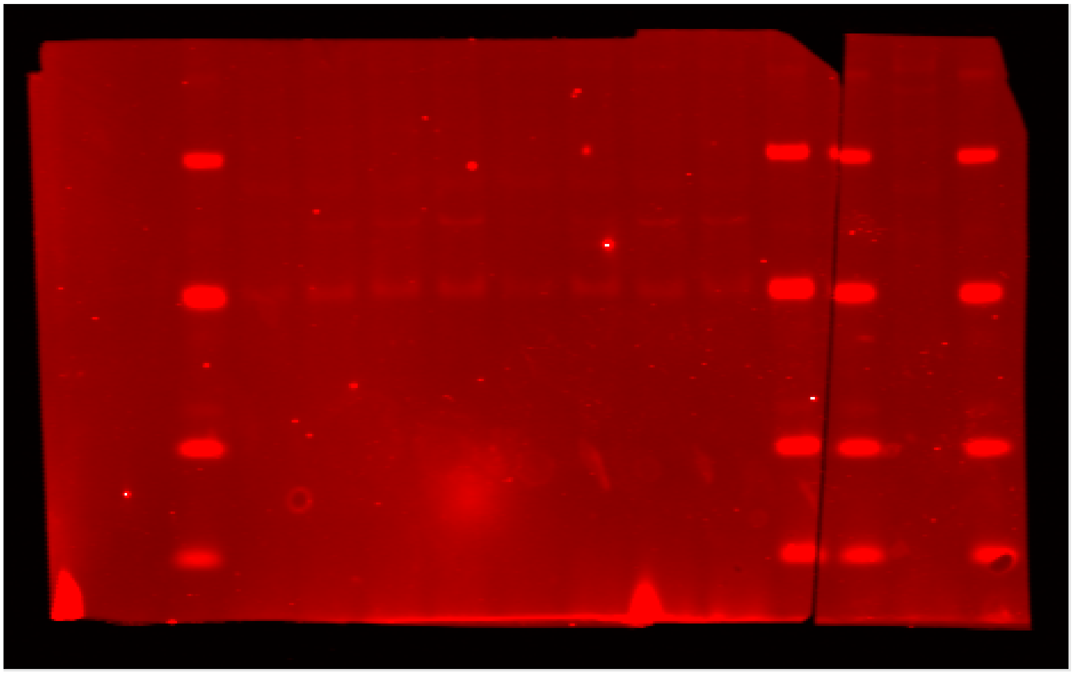

β-ACTIN →

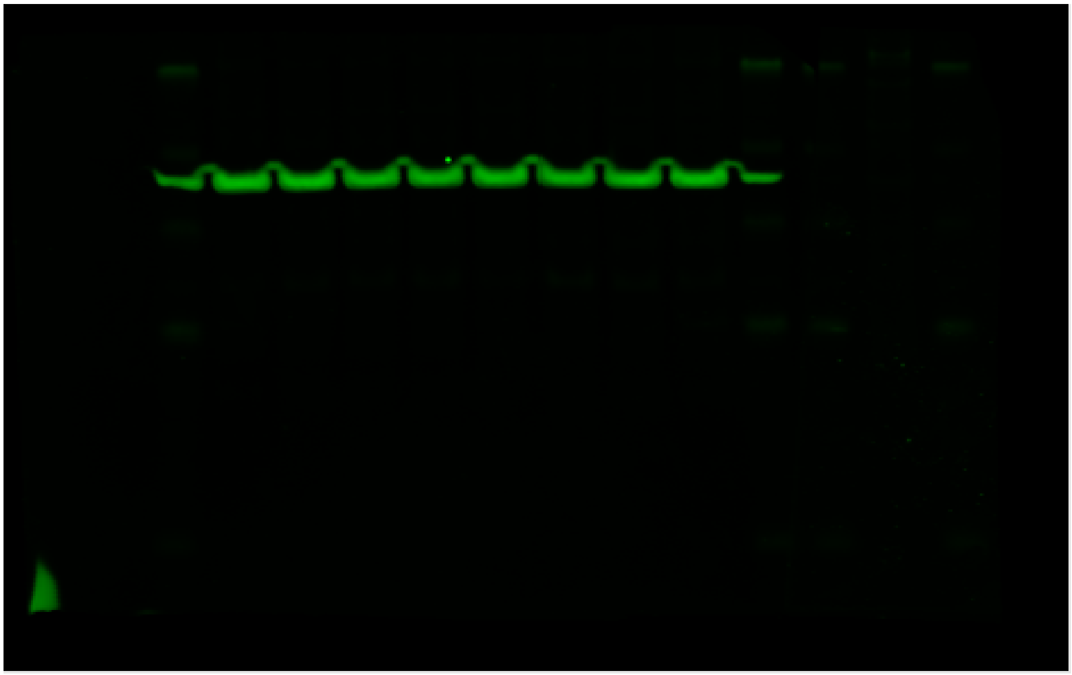

Supplement: Supplemental data [file jciinsight-8-169732-s112.pdf]
