# Supplementary material for: Single-cell landscape analysis unravels molecular programming of the human B cell compartment in chronic GVHD
Source: JCI Insight. 2023 Jun 8;8(11):e169732. doi: 10.1172/jci.insight.169732 (PMC10393230; doi:10.1172/jci.insight.169732)
Supplement: Supplemental table 3 [file jciinsight-8-169732-s113.pdf]

Supplemental Table 3. All DEGs by cluster in the blood B cell scRNA-Seq dataset for untreated B cells. Log2FC values represent the change in expression for the gene of interest in untreated B cells from patients with Active cGVHD compared to No cGVHD, along with associated statistical data.

Cluster 1	log2FC	lfcSE	stat	pvalue	padj
ARRDC3	-1.16089	0.124256	-9.342741	< 2.22e-16	< 2.22e-16
CKS2	1.150264	0.145341	7.914258	2.49E-15	1.17E-11
SMIM10	-4.719045	0.714476	-6.604905	3.98E-11	NA
NFKBIA	-0.633411	0.10013	-6.325907	2.52E-10	7.88E-07
GATD3	4.647591	0.850573	5.464067	4.65E-08	0.00010924
CXCR4	0.256004	0.048661	5.260934	1.43E-07	0.000269166
RSRP1	0.390152	0.074935	5.206521	1.92E-07	0.000281759
C7orf50	-0.470123	0.090579	-5.190221	2.10E-07	0.000281759
SLC50A1	-0.579278	0.113403	-5.108134	3.25E-07	0.000381886
C16orf74	-0.70939	0.141126	-5.026657	4.99E-07	0.000520732
ACADVL	-0.343337	0.068627	-5.002962	5.65E-07	0.000530124
SYNGR2	-0.729816	0.146819	-4.970843	6.67E-07	0.000539799
ZBED2	2.186817	0.440846	4.9605	7.03E-07	0.000539799
CYTIP	0.273594	0.055287	4.948643	7.47E-07	0.000539799
GPR183	0.68349	0.138905	4.920551	8.63E-07	0.000550377
C17orf49	-0.94241	0.191667	-4.916913	8.79E-07	0.000550377
CLK1	0.541952	0.111031	4.881087	1.06E-06	0.000612724
LMO4	0.689222	0.14149	4.871187	1.11E-06	0.000612724
ENSG00000272211	-1.16566	0.240687	-4.843062	1.28E-06	0.000632555
RIN3	0.762537	0.157466	4.842555	1.28E-06	0.000632555
CD5	-1.485943	0.30748	-4.832647	1.35E-06	0.000632555
ENSG00000274265	-0.550603	0.116453	-4.728101	2.27E-06	0.001013358
ENSG00000275964	-1.161019	0.246299	-4.713867	2.43E-06	0.00103742
CHMP6	-0.829528	0.177124	-4.683326	2.82E-06	0.001152347
CD79B	-0.290299	0.062331	-4.657387	3.20E-06	0.001252968
HMGB2	-0.382977	0.0825	-4.642141	3.45E-06	0.001255193
H3F3B	-0.299	0.064433	-4.64051	3.48E-06	0.001255193
GLRX	0.413958	0.090174	4.590641	4.42E-06	0.001536785
CHORDC1	0.587313	0.128611	4.566594	4.96E-06	0.001633973
MIF	-0.415094	0.090973	-4.562852	5.05E-06	0.001633973
HINT2	-0.434598	0.095437	-4.553756	5.27E-06	0.001649399
CD48	0.394216	0.087627	4.49882	6.83E-06	0.002011967
IGHD	-0.420601	0.093507	-4.498094	6.86E-06	0.002011967
CAV1	-1.331937	0.300417	-4.433623	9.27E-06	0.002636676
HSPD1	0.432091	0.098449	4.388966	1.14E-05	0.003087995
VMAC	-0.831149	0.189472	-4.386667	1.15E-05	0.003087995
RBM3	-0.42132	0.096375	-4.371671	1.23E-05	0.003216054
KRTCAP2	-0.258614	0.059542	-4.343415	1.40E-05	0.0035602
ZC3H12A	-0.618369	0.142572	-4.337239	1.44E-05	0.003565332
GTF3C6	-0.396704	0.09224	-4.300789	1.70E-05	0.004097685
GPR18	0.895258	0.210281	4.257429	2.07E-05	0.004854436

KRT10	-0.337307	0.079644	-4.235192	2.28E-05	0.00488225
RPL8	0.357522	0.084461	4.232968	2.31E-05	0.00488225
ARF5	-0.425702	0.100766	-4.224671	2.39E-05	0.00488225
SUSD3	0.576231	0.136416	4.224075	2.40E-05	0.00488225
UGT8	0.87686	0.207602	4.223757	2.40E-05	0.00488225
NUP210	0.444684	0.105367	4.22032	2.44E-05	0.00488225
DBI	-0.322718	0.076475	-4.219935	2.44E-05	0.00488225
LCN8	3.644744	0.870617	4.18639	2.83E-05	0.005544514
CYB5A	-0.767566	0.184549	-4.159138	3.19E-05	0.006121721
NINJ1	-1.082314	0.26119	-4.143781	3.42E-05	0.006415748
CLEC2D	-0.42138	0.101973	-4.132261	3.59E-05	0.006613734
SLC5A3	-0.534456	0.131281	-4.071085	4.68E-05	0.008450031
GPR65	0.579118	0.142868	4.053528	5.05E-05	0.008938394
INKA1	0.604139	0.149323	4.045864	5.21E-05	0.00906491
ZBTB25	0.524798	0.130158	4.032025	5.53E-05	0.009440941
ENSG00000279483	-0.350686	0.087464	-4.009477	6.09E-05	0.010203814
TNFRSF14	0.290898	0.072707	4.000988	6.31E-05	0.010391356
NSUN7	-1.03129	0.260893	-3.952917	7.72E-05	0.012499074
DYNLL1	0.411037	0.104805	3.921903	8.79E-05	0.013981949
ASF1A	0.571849	0.146569	3.901559	9.56E-05	0.014795111
PIM1	-0.54679	0.140195	-3.9002	9.61E-05	0.014795111
TCL1B	-1.290914	0.331564	-3.89341	9.88E-05	0.014970205
GTF2IRD2	-0.504434	0.130221	-3.873685	0.000107202	0.015978195
TMEM170A	0.448666	0.116308	3.857573	0.000114518	0.01647536
NUFIP2	0.422939	0.109695	3.855601	0.000115446	0.01647536
ZNF350	-0.45989	0.119302	-3.854849	0.000115801	0.01647536
ZFAND2A	-0.53185	0.138426	-3.842116	0.000121978	0.017095139
GRAMD1C	-0.771036	0.201458	-3.827275	0.00012957	0.01789209
NELFE	-0.374344	0.098337	-3.806738	0.000140812	0.019032576
ZNF564	-0.797105	0.209496	-3.804863	0.000141883	0.019032576
EPS15	-0.678831	0.178621	-3.800395	0.000144466	0.019106088
H2AFZ	0.266131	0.070243	3.788698	0.000151439	0.019750181
NOP10	0.199173	0.052859	3.76803	0.000164541	0.021164922
MIR29B2CHG	0.540707	0.144595	3.739462	0.000184415	0.023400737
APOBEC3C	-0.29942	0.080151	-3.735707	0.000187188	0.023435964
KDM4B	0.682874	0.184351	3.704215	0.000212046	0.026160816
YWHAE	0.343801	0.092887	3.701269	0.000214524	0.026160816
DEDD2	0.448125	0.122142	3.668885	0.000243611	0.029327001
FRG1	-0.27148	0.07433	-3.652339	0.000259862	0.030839417
EVI2A	0.596672	0.163494	3.649508	0.000262743	0.030839417
ENSG00000260879	-1.428487	0.39218	-3.642427	0.00027008	NA
TRMT112	0.262871	0.07225	3.638378	0.00027436	0.031805473
DNAJB4	0.912539	0.251043	3.634993	0.000277988	0.031832997
ATF7IP2	-0.543517	0.149881	-3.626315	0.000287494	0.032524966
TNFAIP8L2	0.517053	0.142818	3.620355	0.000294199	0.032887273
ENSG00000254397	1.73244	0.479891	3.610068	0.000306116	0.033816855
RPS9	0.291026	0.080799	3.601869	0.000315938	0.034496028

CMTM7	-0.444415	0.123653	-3.594058	0.000325568	0.035138871
SKAP2	0.312678	0.08711	3.589457	0.000331367	0.035358398
ODF3B	-0.704025	0.196646	-3.580167	0.000343375	0.035826235
RPS5	0.228828	0.06393	3.579355	0.000344443	0.035826235
TXNL4A	-0.438732	0.122644	-3.577273	0.000347198	0.035826235
ORMDL2	-0.381928	0.106964	-3.57061	0.000356151	0.036309812
MRPL28	-0.333947	0.093634	-3.566514	0.000361762	0.036309812
DDB2	-0.486155	0.136359	-3.565268	0.000363485	0.036309812
CD83	-0.390139	0.109527	-3.562039	0.000367985	0.036372415
METTL26	-0.287434	0.081341	-3.533709	0.000409772	0.040080791
THEMIS2	1.146557	0.324875	3.529229	0.000416772	0.040345243
GAPT	0.38751	0.110015	3.522343	0.00042775	0.040468656
MICOS13	-0.256183	0.072784	-3.519775	0.000431913	0.040468656
BCL7C	-0.536615	0.152504	-3.518699	0.000433669	0.040468656
PNPLA8	0.352473	0.1002	3.517711	0.000435286	0.040468656
INTS13	0.65668	0.188391	3.485727	0.000490802	0.044849206
SGK1	-0.956973	0.274652	-3.484314	0.0004934	0.044849206
ZNF688	-0.464641	0.133421	-3.482512	0.000496732	0.044849206
ARL17A	-0.574469	0.165372	-3.473793	0.000513156	0.045890795
YARS	-0.974461	0.280885	-3.469255	0.000521903	0.046232726
GBGT1	0.701681	0.202602	3.463339	0.000533517	0.046411383
THAP9.AS1	-0.613626	0.177185	-3.463193	0.000533805	0.046411383
LINC00638	1.573401	0.45509	3.457337	0.000545542	NA
LPAR5	0.748113	0.216636	3.453321	0.000553731	0.047009118
TMEM141	-0.376051	0.108913	-3.452763	0.000554876	0.047009118
PRDX3	0.379255	0.109854	3.452363	0.000555699	0.047009118
TTC32	-0.632841	0.183627	-3.446337	0.000568241	0.047269271
CDKN1A	-0.581922	0.168963	-3.44409	0.000572986	0.047269271
ZNF91	0.394216	0.114476	3.44367	0.000573876	0.047269271
CDC42	-0.410693	0.119491	-3.437033	0.000588125	0.048021683
WNT10A	-0.970705	0.283539	-3.423537	0.000618117	0.050035525
ENSG00000254614	-0.753886	0.220615	-3.417195	0.000632699	0.050736973
KRCC1	0.347691	0.101863	3.413326	0.000641751	0.050736973
MT1X	-1.355045	0.397048	-3.412799	0.000642993	0.050736973
ENSG00000267002	0.419032	0.122934	3.408591	0.000652993	0.051096713
TAGLN2	-0.334047	0.098089	-3.405563	0.000660278	0.051239728
STARD10	-0.533181	0.156871	-3.398851	0.000676697	0.051894883
ATF2	0.52874	0.155621	3.39761	0.000679773	0.051894883
SOAT1	0.662492	0.195159	3.394624	0.000687229	0.052040985
NDUFB7	-0.273641	0.080731	-3.389543	0.000700093	0.052590966
ZKSCAN1	-0.376423	0.111438	-3.377865	0.000730508	0.053362415
ZNF860	0.645387	0.191179	3.375817	0.000735968	0.053362415
TSC22D1	-0.646647	0.191589	-3.375176	0.000737684	0.053362415
CRK	0.445801	0.132207	3.371982	0.000746292	0.053362415
ENSG00000224505	-0.389297	0.115471	-3.371386	0.00074791	0.053362415
FKBP5	1.225567	0.363773	3.369044	0.000754293	0.053362415
CDCA4	0.589569	0.175057	3.367867	0.000757522	0.053362415

AKAP13	0.225168	0.066866	3.367451	0.000758666	0.053362415
UROD	-0.377402	0.112108	-3.366419	0.000761508	0.053362415
RENBP	0.758214	0.225797	3.357946	0.000785239	0.054617722
FANCL	0.743954	0.221754	3.354855	0.000794066	0.054825597
EGLN2	0.407101	0.12175	3.343738	0.000826579	0.056653844
ROCK1	0.27931	0.083615	3.340449	0.00083643	0.056913626
ENSG00000237596	-0.929469	0.279221	-3.328788	0.000872246	0.058546903
IL23A	-1.068503	0.321089	-3.327751	0.0008755	0.058546903
ENSG00000273319	0.580923	0.174659	3.326042	0.000880886	0.058546903
MICAL1	-0.646335	0.194408	-3.324625	0.000885374	0.058546903
NFKBIE	-0.436676	0.131566	-3.319074	0.000903165	0.059236291
BLNK	0.257367	0.077646	3.31463	0.000917646	0.059236291
MYBL2	-1.852426	0.558952	-3.314105	0.00091937	NA
RPL30	0.153139	0.046209	3.314045	0.000919569	0.059236291
HLA.A	-0.463547	0.139892	-3.3136	0.000921033	0.059236291
HHIP.AS1	-2.030877	0.614239	-3.306327	0.000945276	NA
MFSD13A	-0.961639	0.291074	-3.303764	0.000953961	0.060936709
SPINT2	-0.280067	0.084863	-3.300236	0.000966036	0.061291046
LIMD2	-0.236235	0.071842	-3.288253	0.001008114	0.063531451
FGL2	-1.625232	0.494597	-3.285975	0.001016301	NA
EYA3	0.756162	0.230172	3.285209	0.001019066	0.063793522
PILRB	0.721077	0.219838	3.280029	0.001037964	0.064546227
GPR137	-0.59461	0.181538	-3.275396	0.00105514	0.064871871
PYROXD1	-0.56969	0.174027	-3.273581	0.001061941	0.064871871
RAF1	0.37146	0.11349	3.273051	0.001063934	0.064871871
TCP1	0.297048	0.090806	3.271223	0.001070835	0.064871871
HMGB3	-1.141141	0.348875	-3.270915	0.001072001	NA
COMMD3	-0.344209	0.105288	-3.269206	0.001078499	0.064917329
ENSG00000254837	1.194193	0.365596	3.266425	0.001089147	NA
CRIPT	0.417244	0.127995	3.259845	0.001114731	0.066670854
ZC3H12D	-0.647001	0.198631	-3.257304	0.00112476	0.066844912
CASP4	0.243324	0.074754	3.255009	0.001133887	0.066963505
NAB2	1.055125	0.325418	3.242363	0.00118543	0.069500561
ZFYVE26	0.924577	0.285381	3.239798	0.001196144	0.069500561
CHP1	-0.339326	0.104814	-3.237403	0.00120623	0.069500561
TSN	-0.340065	0.105104	-3.235495	0.001214322	0.069500561
ENSG00000278834	-1.04798	0.32401	-3.234401	0.001218984	NA
RHOBTB2	0.965666	0.298631	3.23364	0.001222235	0.069500561
SERPINI1	1.024205	0.316875	3.232202	0.001228401	0.069500561
ADAMTS6	0.972409	0.300856	3.232143	0.001228657	0.069500561
RHOH	0.246444	0.076412	3.225203	0.001258833	0.070525326
CCT8	0.456192	0.141476	3.22453	0.001261795	0.070525326
MTPN	0.256935	0.079766	3.221124	0.001276889	0.070946669
ENSG00000166927	-1.03188	0.322399	-3.200628	0.001371285	0.075743323
ENSG00000268858	-0.878811	0.275171	-3.193692	0.00140466	0.076852121
NRL	-1.502583	0.470491	-3.193651	0.001404856	NA
PTGES2	-0.47533	0.148863	-3.193062	0.001407728	0.076852121

ENSG00000239636	-0.804073	0.252183	-3.188455	0.001430353	0.077600443
DNAJB1	0.524837	0.164685	3.18692	0.001437963	0.077600443
DAP	-0.650767	0.204534	-3.181714	0.001464065	0.07855755
C6orf203	0.546628	0.172073	3.176724	0.001489487	0.079016803
CUTC	-0.397087	0.125	-3.1767	0.001489612	0.079016803
SCRN1	0.926439	0.291783	3.175096	0.001497869	0.079016803
EIF4A2	0.178043	0.056194	3.168354	0.00153305	0.080420886
RFTN1	-0.468597	0.148154	-3.16291	0.001562008	0.081484733
IZUMO4	-0.566745	0.179396	-3.159177	0.001582154	0.082079726
CFAP410	-0.374775	0.11879	-3.15495	0.001605256	0.082820627
PTPMT1	-0.340336	0.107957	-3.152527	0.001618637	0.083054652
POGZ	0.363958	0.115919	3.13976	0.001690861	0.085895694
ROGDI	-0.612072	0.195131	-3.136717	0.001708507	0.085895694
SYNJ2BP	0.339509	0.108268	3.135821	0.001713736	0.085895694
ENSG00000237491	-0.670294	0.213783	-3.135387	0.001716273	0.085895694
MAML3	-1.034544	0.33002	-3.134795	0.001719743	0.085895694
RBBP8	-0.770184	0.246901	-3.119408	0.001812148	0.089963196
ATP6V1C2	-2.14975	0.689292	-3.118777	0.001816033	NA
SMCR8	0.610329	0.195739	3.118079	0.001820342	0.089963196
JADE1	0.35726	0.114949	3.107975	0.001883742	0.091571792
JMJD6	0.654045	0.210452	3.107816	0.001884753	0.091571792
TNFSF10	0.582584	0.187465	3.107699	0.001885501	0.091571792
PPCS	-0.241844	0.077846	-3.106698	0.001891899	0.091571792
CTNNBIP1	-1.011588	0.325967	-3.103348	0.001913445	0.092049805
BOLA2B	-0.611408	0.197093	-3.102123	0.00192138	0.092049805
FGFBP3	-1.060911	0.342838	-3.094493	0.001971496	NA
PXMP2	-0.680085	0.219864	-3.093201	0.001980102	0.094381518
TCOF1	-0.406225	0.13149	-3.089393	0.002005657	0.094649807
BBX	0.242398	0.078462	3.089359	0.00200589	0.094649807
MCRIP1	-0.223996	0.072687	-3.081656	0.002058528	0.096647869
ZNF253	0.521255	0.169349	3.077994	0.002083992	0.097356644
CYB561D2	-0.37341	0.121481	-3.073813	0.002113416	0.098242473
ZNF442	-1.370983	0.446494	-3.070549	0.002136657	NA
TMEM179B	-0.317544	0.103449	-3.069581	0.002143595	0.099078874
SRSF5	-0.173124	0.056423	-3.06834	0.002152512	0.099078874
UTRN	0.396235	0.129255	3.065539	0.002172783	0.099524074
PCIF1	-0.494578	0.161724	-3.058156	0.002227038	0.101514031
FGFR1OP2	0.337413	0.110454	3.054776	0.002252284	0.102065614
CD180	0.60242	0.19728	3.053634	0.002260878	0.102065614
METTL7A	0.358239	0.117513	3.048501	0.002299864	0.10273556
FMNL2	-1.869366	0.613231	-3.048387	0.002300733	NA
ENSG00000282988	0.803505	0.263897	3.044768	0.002328598	0.10273556
PFN1	-0.189443	0.062225	-3.044463	0.002330959	0.10273556
YIPF6	-0.43366	0.142462	-3.044046	0.002334191	0.10273556
IGHG1	1.111677	0.365289	3.043277	0.002340167	0.10273556
POLR2E	-0.248482	0.081654	-3.043123	0.002341364	0.10273556
SLC3A2	-0.408761	0.134551	-3.037966	0.002381811	0.104024218

RMND5A	0.536121	0.177	3.028926	0.002454244	0.106659345
SETD1B	0.690387	0.228029	3.027622	0.002464865	0.106659345
ZBTB20	0.31137	0.10309	3.020378	0.002524593	0.108287638
ADO	-0.734679	0.24325	-3.020262	0.002525558	0.108287638
ICA1L	-0.474282	0.15751	-3.011118	0.002602879	0.110822514
RAB11A	-0.199029	0.066112	-3.010488	0.002608283	0.110822514
POLH	0.524935	0.175031	2.999092	0.002707857	0.113973575
CHTOP	0.247792	0.082648	2.998179	0.002715984	0.113973575
AKAP2	-0.704735	0.23508	-2.997856	0.002718858	0.113973575
DUSP12	-0.3299	0.110253	-2.992208	0.002769675	0.115587751
LRIF1	0.346047	0.115728	2.990174	0.002788188	0.11584551
ZC3H6	0.425917	0.142588	2.98705	0.002816835	0.116520191
PTMS	-1.661519	0.556945	-2.983272	0.002851847	NA
ADA	-0.572731	0.192011	-2.982805	0.002856203	0.117630466
MRPL32	0.39143	0.131713	2.97184	0.002960206	0.120475892
TBC1D10A	0.473991	0.159511	2.971531	0.002963187	0.120475892
COL19A1	-0.483238	0.162783	-2.968605	0.002991553	0.120475892
MAP7D1	-0.42946	0.144672	-2.968521	0.002992367	0.120475892
ASPSCR1	-0.462098	0.15567	-2.968452	0.002993043	0.120475892
FAM41C	-0.810986	0.273307	-2.967308	0.0030042	0.120475892
TMEM208	-0.324331	0.109361	-2.96569	0.003020051	0.120475892
CLECL1	1.516478	0.512001	2.961869	0.003057781	0.120475892
SYNC	-0.502881	0.169819	-2.961275	0.003063685	0.120475892
FXYD2	-1.035424	0.349836	-2.959736	0.003079027	0.120475892
LRRC61	-0.667859	0.2257	-2.959056	0.003085833	0.120475892
BRK1	-0.195204	0.065973	-2.958862	0.003087776	0.120475892
MTHFR	0.581089	0.196481	2.957478	0.003101665	0.120475892
PDE6D	-0.418857	0.141642	-2.957155	0.003104916	0.120475892
RPS6KB2	-0.271591	0.092002	-2.952023	0.003156998	0.121768294
DUSP22	-0.355981	0.120618	-2.951323	0.00316416	0.121768294
TSEN15	-0.507926	0.172267	-2.948473	0.003193478	0.121987831
NDUFS8	-0.231341	0.078467	-2.948244	0.003195847	0.121987831
TUBB4B	0.373356	0.126958	2.940785	0.003273816	0.124050513
FCRL3	-1.024275	0.348328	-2.940549	0.003276307	0.124050513
ETS2	-1.290828	0.438984	-2.940493	0.003276906	NA
IL21R	-0.623476	0.212223	-2.937831	0.00330517	0.124640764
C1orf43	0.242806	0.082825	2.931542	0.003372838	0.12543106
LRIG1	-0.754339	0.257342	-2.931266	0.003375835	0.12543106
FNTA	0.250265	0.085381	2.931163	0.003376958	0.12543106
ALDH9A1	0.388372	0.132508	2.930924	0.003379559	0.12543106
DNAAF4	-1.619068	0.553301	-2.926198	0.003431325	0.126386548
FXYD7	0.703499	0.240465	2.925578	0.003438169	0.126386548
PRICKLE1	0.595171	0.203535	2.924164	0.003453827	0.126386548
ENSG00000225963	0.92584	0.316896	2.921584	0.003482563	0.126386548
STAM2	0.679355	0.232566	2.921126	0.003487689	0.126386548
RPL24	0.173892	0.059538	2.920676	0.003492725	0.126386548
GDF11	0.450888	0.15441	2.920071	0.003499521	0.126386548

VPREB1	-1.814691	0.622183	-2.916651	0.003538109	0.127290575
FBXO4	-0.485592	0.166634	-2.914124	0.003566886	0.127836097
IER3	-1.127319	0.387392	-2.910022	0.003614029	NA
FAM177B	0.623079	0.21412	2.90995	0.003614866	0.12804914
XIAP	0.427322	0.146857	2.909788	0.003616737	0.12804914
DFFB	0.895908	0.307937	2.90939	0.003621348	NA
ORMDL3	-0.449711	0.154599	-2.908879	0.003627274	0.12804914
TMEM243	0.236022	0.081139	2.90887	0.003627377	0.12804914
SIGLEC5	1.555229	0.53558	2.903822	0.003686377	NA
CHMP5	0.267365	0.092107	2.902757	0.003698938	0.130086256
DCAF13	0.368919	0.12725	2.899167	0.003741551	0.131093911
TAPBP	-0.299784	0.103476	-2.897133	0.003765895	0.131456344
RNF43	-0.801607	0.276826	-2.895709	0.00378303	0.131565361
LY9	0.458417	0.158422	2.893642	0.003808016	0.13194565
H1FX	-0.888601	0.307293	-2.891704	0.003831588	0.132274298
POLE3	-0.306222	0.105942	-2.890461	0.003846771	0.13231201
RPS28	0.183031	0.06341	2.886489	0.003895659	0.133504528
PIGX	-0.552344	0.191528	-2.883888	0.00392798	0.134122646
TPM4	0.314647	0.109189	2.881684	0.003955564	0.134575166
POR	-0.464993	0.161732	-2.875081	0.004039241	0.136568436
GDI1	0.311317	0.108293	2.874768	0.00404324	0.136568436
PTGIR	-1.387579	0.48322	-2.871528	0.004084922	0.137481772
LINC01991	-1.107371	0.385675	-2.871256	0.004088437	NA
EIF5	0.184059	0.064222	2.865981	0.004157195	0.139269814
LYRM2	-0.277301	0.096783	-2.865181	0.004167712	0.139269814
RPL19	0.118167	0.041297	2.861415	0.00421754	0.140435103
AIM2	1.751814	0.613558	2.855174	0.004301326	NA
IFIT2	0.533366	0.186818	2.854997	0.00430373	0.142798664
TDG	0.323483	0.113422	2.852035	0.004344033	0.143529531
RBM43	-0.632853	0.221965	-2.851136	0.004356328	0.143529531
ENSG00000272277	-1.05399	0.369818	-2.850019	0.004371657	NA
PIGF	0.53759	0.188804	2.847344	0.004408572	0.144572504
LINC00662	0.298325	0.1048	2.846608	0.004418776	0.144572504
TTC21A	0.337012	0.118777	2.837341	0.004549103	0.148003859
HLA.C	-0.485777	0.171234	-2.836915	0.004555177	0.148003859
ENSG00000280433	-0.894275	0.315624	-2.833357	0.004606196	0.149145451
ENSG00000273145	-1.583334	0.559347	-2.830684	0.004644856	NA
SMIM2.AS1	-2.109612	0.745598	-2.829422	0.004663222	NA
PDCL3	-0.50087	0.177037	-2.829185	0.004666677	0.150584541
GALNT2	-0.415248	0.147154	-2.82185	0.004774757	0.153278685
LAMC1	1.018833	0.361121	2.821309	0.004782817	0.153278685
CREBRF	0.287077	0.101849	2.818651	0.004822591	0.154027667
BIN1	0.317453	0.112798	2.814354	0.004887532	0.155344062
ST13	0.134099	0.047659	2.813739	0.004896895	0.155344062
IL16	0.243047	0.08642	2.812392	0.004917455	0.155471063
TRMT13	0.341244	0.121396	2.810989	0.004938948	0.155626591
SLC16A7	0.420343	0.149638	2.809067	0.004968526	0.156034965

SAFB	0.312617	0.111345	2.807648	0.004990476	0.156201886
TAGAP	-0.396166	0.141493	-2.799898	0.005111882	0.159470348
YWHAG	0.39984	0.143094	2.794255	0.005201947	0.160496171
PPM1K	0.197311	0.07062	2.794004	0.00520599	0.160496171
EFCAB11	-0.694267	0.248488	-2.793971	0.005206511	0.160496171
CFAP73	-1.323146	0.473663	-2.793435	0.005215155	0.160496171
SCN3A	1.542525	0.552389	2.792461	0.005230875	NA
PPP1R15A	-0.581857	0.208493	-2.790768	0.005258307	0.160496171
NDUFS7	-0.208876	0.074848	-2.790673	0.005259853	0.160496171
LINC02422	0.65955	0.236448	2.789406	0.005280481	0.160496171
OCLN	-0.817582	0.293171	-2.788753	0.005291143	0.160496171
SNRPA1	-0.301484	0.108125	-2.788297	0.005298596	0.160496171
CHL1	1.339659	0.480531	2.787873	0.005305528	NA
SNAP23	-0.230134	0.082667	-2.78386	0.005371628	0.162185163
NDUFB11	0.280297	0.100732	2.782586	0.005392753	0.162301123
SLAMF1	0.468927	0.168596	2.781372	0.005412967	0.162389003
COL9A3	-1.00434	0.361582	-2.777629	0.005475705	0.163246405
TMEM99	0.609994	0.219613	2.777593	0.005476317	0.163246405
TMEM205	-0.348325	0.125555	-2.774279	0.005532424	0.164397032
DHPS	0.232991	0.084027	2.772821	0.005557261	0.164489914
ENSG00000259943	-0.619443	0.223461	-2.772042	0.005570585	0.164489914
KLHDC7B	-0.921422	0.333223	-2.765184	0.005689065	0.166407883
SREBF2.AS1	-0.692699	0.250569	-2.764501	0.005700996	0.166407883
CEP104	0.389294	0.140822	2.764452	0.005701853	0.166407883
SMIM4	-0.41057	0.148559	-2.763693	0.005715121	0.166407883
PODXL2	-1.312078	0.474882	-2.762957	0.005728028	NA
FAAP24	-0.749711	0.271374	-2.762652	0.005733388	0.166407883
TAX1BP3	-0.360475	0.130504	-2.762169	0.005741869	0.166407883
FCGR2B	0.804798	0.291929	2.756825	0.005836552	0.16821394
RETREG1	-0.659536	0.239382	-2.755157	0.005866391	0.16821394
POLR2B	-0.274625	0.099727	-2.753771	0.005891296	0.16821394
PPP1R35	-0.549201	0.199467	-2.753344	0.005898985	0.16821394
DMAC2L	-0.260385	0.094571	-2.753333	0.005899194	0.16821394
ENSG00000272606	-0.505394	0.183603	-2.752641	0.005911672	0.16821394
MRPL50	0.294232	0.106988	2.750146	0.005956875	0.168988095
MRPL23	-0.281482	0.102476	-2.746807	0.006017852	0.170203696
ZBTB16	1.855098	0.676434	2.742466	0.006097971	0.171553146
ADAL	-0.516844	0.188475	-2.742244	0.006102103	0.171553146
PPP1R16B	0.371481	0.135543	2.740693	0.00613098	0.171850446
ENSG00000262580	1.188904	0.433851	2.740349	0.006137398	NA
MPND	-1.084342	0.395719	-2.740179	0.006140568	NA
C1GALT1	0.368354	0.134645	2.735747	0.006223882	0.173935261
SELENOW	-0.372056	0.136133	-2.733036	0.006275346	0.174117038
SRSF10	0.190367	0.069664	2.732647	0.006282761	0.174117038
DUSP14	0.64163	0.234835	2.732255	0.00629025	0.174117038
HMGXB4	-0.40231	0.147301	-2.731219	0.006310061	0.174117038
VOPP1	-0.328164	0.120183	-2.730538	0.0063231	0.174117038

CIRBP	-0.175971	0.064559	-2.725738	0.006415795	0.176152962
RWDD2A	-0.873893	0.321161	-2.721039	0.0065077	NA
ZNF721	-0.322834	0.118717	-2.719352	0.006541003	0.178631109
HERC2	-0.319741	0.117587	-2.719195	0.0065441	0.178631109
C9orf72	-0.449174	0.165301	-2.717309	0.006581522	0.179131849
TNFRSF17	1.265323	0.465922	2.715739	0.006612811	0.179291827
TMEM140	0.413952	0.15247	2.714981	0.00662796	0.179291827
ZNF557	0.474963	0.174995	2.714146	0.006644681	0.179291827
AGAP9	1.524093	0.561719	2.713267	0.006662339	NA
ATP6V0E2	-0.673254	0.248239	-2.712121	0.006685427	0.179874381
ZNF628	-1.364092	0.503248	-2.710576	0.00671664	NA
RPL9	0.15455	0.057057	2.708717	0.006754389	0.181210599
OPTN	-0.736532	0.272036	-2.707474	0.006779731	0.181372292
XXYLT1.AS2	1.133009	0.418782	2.705485	0.006820481	0.18194407
RAB11FIP2	-0.457953	0.169364	-2.703959	0.006851875	0.182263754
TCTN1	-0.798005	0.296151	-2.694588	0.007047575	0.186919045
ENSG00000262292	0.711962	0.264535	2.691373	0.007115869	0.186919045
RPS23	0.139149	0.051706	2.691125	0.007121148	0.186919045
PFKP	-0.763444	0.283779	-2.690277	0.007139276	0.186919045
NT5E	0.668207	0.248467	2.689315	0.00715989	0.186919045
AGMAT	0.729028	0.271131	2.688842	0.007170028	0.186919045
PARP9	0.561489	0.208945	2.68725	0.007204308	0.186919045
GLS	0.407385	0.151604	2.687164	0.007206162	0.186919045
PHKG1	-0.524366	0.195146	-2.687038	0.007208882	0.186919045
RPL35A	0.15235	0.056743	2.68492	0.007254726	0.186919045
PFDN2	0.222657	0.082933	2.684795	0.007257424	0.186919045
EMP3	-0.344669	0.128397	-2.684412	0.007265756	0.186919045
MED23	-0.482284	0.17982	-2.682037	0.007317539	0.187736858
AAK1	-0.913817	0.341002	-2.679797	0.007366692	0.187826387
TMEM60	0.330271	0.123274	2.679162	0.007380657	0.187826387
ZNF830	0.281757	0.105196	2.678408	0.007397305	0.187826387
LILRB1	0.414567	0.154827	2.67761	0.007414945	0.187826387
XRRA1	-0.65964	0.246379	-2.677335	0.007421043	0.187826387
CENPS	-0.494336	0.184813	-2.67479	0.007477606	0.188455976
SYNE2	-0.37654	0.140794	-2.674411	0.007486057	0.188455976
CLCF1	0.531298	0.19874	2.673329	0.007510254	0.188559576
IFT57	0.448623	0.168013	2.670165	0.007581398	0.189576885
TNFAIP8	0.300739	0.112654	2.669578	0.00759466	0.189576885
ZNF816	0.618176	0.231627	2.668841	0.00761134	0.189576885
LONP2	-0.260358	0.097594	-2.66777	0.00763566	0.189679492
ZC3H7A	-0.467025	0.175312	-2.663961	0.007722649	0.191334235
PDPK1	0.372638	0.139957	2.662519	0.007755812	0.191380149
TMEM14B	-0.30359	0.114071	-2.661402	0.00778159	0.191380149
BAG2	0.672967	0.252885	2.661154	0.007787341	0.191380149
COG3	-0.481723	0.18108	-2.66027	0.007807795	0.191380149
PTP4A1	-0.382614	0.143869	-2.659468	0.007826409	0.191380149
GSTO1	0.295132	0.111172	2.654724	0.007937331	0.193588409

IL18BP	-1.472422	0.555331	-2.651432	0.008015117	NA
NAA50	-0.258821	0.097628	-2.651091	0.008023231	0.195141217
ZNRD2	-0.281016	0.106033	-2.650278	0.008042561	0.195141217
CCDC191	-0.817398	0.308659	-2.648222	0.008091639	0.195826
SUGCT	-2.789715	1.055027	-2.644213	0.008188119	NA
RDH5	0.497628	0.188221	2.643855	0.008196778	0.197860524
ZNF322	0.474622	0.179611	2.642502	0.008229588	0.198107503
BCL6	-0.491069	0.185892	-2.641696	0.008249205	0.198107503
PSMB10	-0.343732	0.130202	-2.640003	0.008290534	0.198586326
TPI1	-0.171074	0.064855	-2.637797	0.00834466	0.198586326
ZNF568	0.468811	0.177746	2.637524	0.008351367	0.198586326
PSMA4	-0.190331	0.072165	-2.637428	0.008353738	0.198586326
ENSG00000216895	-0.528582	0.200521	-2.636038	0.008388044	0.198898306
ENSG00000271746	-1.420261	0.539478	-2.63266	0.008471916	NA
ZNF595	0.829007	0.314926	2.632385	0.008478775	0.200543308
GID8	-0.272751	0.103674	-2.630866	0.00851677	0.20093586
CLTA	0.188596	0.071713	2.629872	0.008541706	0.201019106
HAUS3	0.517933	0.197188	2.62659	0.00862453	0.201088846
TMX4	-0.324487	0.123569	-2.625954	0.00864065	0.201088846
TSPAN33	-0.421031	0.16035	-2.6257	0.008647111	0.201088846
OTUD1	0.59483	0.226624	2.624737	0.008671582	0.201088846
RHBDD2	-0.354183	0.135	-2.623582	0.008701038	0.201088846
SNHG9	-0.333345	0.127091	-2.622893	0.008718673	0.201088846
NUAK2	-0.290662	0.110839	-2.62237	0.008732061	0.201088846
LINC01215	-0.326311	0.124455	-2.621925	0.008743464	0.201088846
GPX1	-0.24121	0.092023	-2.621196	0.008762199	0.201088846
MAGEH1	-0.497388	0.189823	-2.620271	0.008785995	0.201088846
SMAP2	-0.298928	0.114122	-2.619371	0.008809209	0.201088846
ELOB	-0.167336	0.063906	-2.618498	0.00883178	0.201088846
ZNF213.AS1	-0.62556	0.238914	-2.618351	0.00883558	0.201088846
LETMD1	0.270743	0.103416	2.618008	0.008844483	0.201088846
VSIR	-0.774977	0.296121	-2.617095	0.008868159	0.201140117
DDX6	-0.348896	0.133408	-2.615256	0.00891605	0.201628635
ZNF267	-0.436942	0.167138	-2.614258	0.008942155	0.201628635
SNHG32	0.193928	0.07421	2.613222	0.008969295	0.201628635
HSPH1	0.395268	0.151308	2.612338	0.008992535	0.201628635
TPMT	-0.580867	0.222426	-2.611503	0.009014509	0.201628635
TTC9C	0.301035	0.115325	2.610325	0.009045619	0.201628635
LDHA	-0.32796	0.12566	-2.609904	0.009056773	0.201628635
CYC1	-0.239816	0.091893	-2.609726	0.009061479	0.201628635
SORD	-0.546811	0.209621	-2.608569	0.009092158	0.201833014
MVP	-0.420726	0.16137	-2.607211	0.00912832	0.202099302
LIPT1	0.496987	0.190679	2.606411	0.009149669	0.202099302
HAUS8	0.490332	0.188177	2.605698	0.009168722	0.202099302
SF1	-0.201569	0.077442	-2.60285	0.009245241	0.203050879
ELF1	0.244474	0.094003	2.600702	0.009303324	0.203050879
BET1	0.345708	0.132979	2.599709	0.009330286	0.203050879

RPL7L1	0.194288	0.074746	2.599316	0.009340981	0.203050879
C2orf42	0.531729	0.204565	2.599311	0.009341113	0.203050879
NME2	-0.885503	0.340671	-2.599292	0.009341638	0.203050879
RFX1	0.330303	0.127124	2.598269	0.009369507	0.203186316
ENSG00000257354	-0.714341	0.275084	-2.596809	0.009409434	0.203581997
FRY.AS1	-1.437212	0.553805	-2.595157	0.009454761	NA
MFNG	0.350123	0.134983	2.593837	0.009491134	0.204877577
RPL38	0.150979	0.058283	2.590456	0.009584899	0.206427069
EIPR1	-0.391308	0.151129	-2.58924	0.009618804	0.206683218
PTPRS	1.54415	0.597224	2.585544	0.009722537	0.208017312
ENSG00000267458	-1.419959	0.549212	-2.58545	0.009725197	0.208017312
GLUL	-0.563213	0.21791	-2.584611	0.00974889	0.208050169
CCT5	0.335435	0.129866	2.582934	0.009796415	0.208079446
CTH	1.411147	0.546348	2.582871	0.009798204	NA
RCC1	0.409393	0.158526	2.5825	0.009808743	0.208079446
ASB16.AS1	0.396384	0.153572	2.581099	0.009848626	0.208079446
PHF21A	-0.316506	0.122646	-2.580647	0.009861543	0.208079446
HSPBAP1	-0.783509	0.303669	-2.580147	0.009875837	0.208079446
TIMM10	-0.365184	0.141569	-2.579545	0.00989306	0.208079446
RNF34	0.327712	0.127083	2.578734	0.009916308	0.208079446
NDUFB10	-0.196159	0.076091	-2.57796	0.00993856	0.208079446
SLC37A3	0.653456	0.253589	2.576835	0.009970958	0.208079446
MAVS	0.340464	0.132127	2.576804	0.009971858	0.208079446
AP1S1	-0.355763	0.138254	-2.57326	0.010074557	0.209756306
POLR2J	-0.179786	0.069895	-2.572241	0.010104249	0.209909066
PRMT7	0.5307	0.20649	2.570096	0.010167026	0.210746954
BLOC1S1	-0.17318	0.067506	-2.565394	0.010305858	0.213154189
PRDM8	-0.770938	0.300827	-2.562726	0.010385394	0.213924413
ERP29	0.152301	0.059442	2.562178	0.010401814	0.213924413
CUX1	-0.349117	0.136333	-2.560769	0.010444071	0.213924413
MRPS15	-0.242209	0.094593	-2.560547	0.010450762	0.213924413
TAF10	-0.550206	0.214896	-2.560339	0.010457008	0.213924413
SAFB2	0.244349	0.095469	2.559464	0.010483382	0.213997733
TLR7	0.78002	0.304912	2.558184	0.010522047	0.214056319
MED6	-0.314018	0.122766	-2.55786	0.010531844	0.214056319
ZNF714	0.599237	0.234595	2.554341	0.010638897	0.215765096
GLMN	-0.393196	0.15413	-2.551077	0.010739049	0.217215091
MPHOSPH8	0.156331	0.061294	2.550506	0.010756658	0.217215091
TRIM69	0.402412	0.158003	2.546864	0.010869577	0.219024307
PHF23	0.331322	0.130311	2.542546	0.011004814	0.2212158
MAP3K8	-0.544835	0.214346	-2.54185	0.011026753	0.2212158
TOMM20	0.175173	0.068945	2.540749	0.011061522	0.2212158
WASHC1	-0.461397	0.181641	-2.540154	0.011080366	0.2212158
MT.CO1	0.224598	0.088436	2.539657	0.011096128	0.2212158
HLA.G	-1.937256	0.763262	-2.538126	0.011144792	NA
GIMAP2	0.489658	0.192924	2.538095	0.011145774	0.221734785
KCTD7	0.496738	0.195813	2.536796	0.011187224	0.222088856

PRKRA	-0.255192	0.100626	-2.536053	0.011210987	0.222091066
KCTD3	-1.225784	0.483854	-2.533377	0.011296931	NA
BZW1	-0.226015	0.089232	-2.532887	0.011312743	0.223220306
UQCRH	0.158358	0.062527	2.532627	0.011321147	0.223220306
SPATA2L	0.559054	0.22079	2.532065	0.011339306	0.223220306
MED14OS	-0.787357	0.311081	-2.531035	0.011372639	0.22340812
KPNB1	0.215964	0.085393	2.529051	0.011437143	0.223543046
CCDC138	-0.693338	0.274161	-2.528941	0.01144073	0.223543046
FBXO28	0.596044	0.235718	2.528628	0.011450927	0.223543046
FAM184B	-1.590337	0.629145	-2.527777	0.011478734	0.223620985
TNFRSF13B	0.931101	0.368493	2.526783	0.011511276	0.223790638
HHEX	0.242992	0.096251	2.524558	0.011584386	0.224267304
RIOX1	-0.72167	0.28592	-2.524025	0.011601969	0.224267304
CYSTM1	-0.321824	0.127514	-2.523836	0.011608223	0.224267304
PGGHG	-0.460717	0.182597	-2.523136	0.011631329	0.224267304
KMT2E.AS1	-0.513062	0.203521	-2.520932	0.011704435	0.224737411
ORAI3	-0.235793	0.093536	-2.52087	0.011706493	0.224737411
CBLN3	-0.568582	0.225607	-2.520239	0.011727511	0.224737411
PPP1CB	-0.309529	0.122886	-2.518828	0.011774607	0.225180374
IGLC7	-2.060806	0.818588	-2.517514	0.011818625	0.225515616
HSD17B11	0.139016	0.055243	2.516469	0.011853739	0.225515616
SMPD4	-0.416541	0.165546	-2.516158	0.011864187	0.225515616
ENSG00000187186	-0.979523	0.389313	-2.516029	0.011868538	NA
NPRL2	0.391221	0.155665	2.513229	0.011963158	0.226707989
MYC	0.270865	0.107791	2.512874	0.011975204	0.226707989
ENSG00000257839	-1.291571	0.514145	-2.512078	0.012002254	NA
RALGPS2	0.315108	0.12555	2.509831	0.012078886	0.228062454
CD40	-0.153249	0.061071	-2.509351	0.012095325	0.228062454
MYCBP2	0.168394	0.067193	2.506136	0.012205867	0.228841308
HSBP1	0.204431	0.081586	2.505697	0.012221016	0.228841308
PPP4C	-0.183206	0.073133	-2.505094	0.012241883	0.228841308
RAB34	-0.643444	0.256882	-2.504823	0.012251252	0.228841308
DLGAP3	-0.866635	0.346073	-2.504201	0.012272832	NA
SIDT1.AS1	-0.592577	0.236644	-2.504086	0.012276804	0.228841308
MARCHF1	0.404454	0.161529	2.503912	0.012282856	0.228841308
FIS1	-0.267853	0.107041	-2.502326	0.012338013	0.229413747
DPM1	0.281586	0.112595	2.500874	0.01238871	0.22951289
UBE2L3	-0.179493	0.071775	-2.500774	0.01239223	0.22951289
LINC00680	-0.461139	0.184489	-2.499547	0.012435206	0.229855474
H6PD	-0.464914	0.186083	-2.498424	0.012474671	0.230131953
MRPL41	-0.257272	0.103073	-2.496009	0.012559959	0.231251002
ENSG00000260349	-0.578667	0.231967	-2.494607	0.012609671	0.231711961
PKNOX1	-0.433922	0.174084	-2.492603	0.012681072	0.232568881
HRK	-1.252097	0.502537	-2.491554	0.012718556	0.232801648
NCBP2AS2	-0.277258	0.111317	-2.490718	0.012748513	0.232895993
RPL13A	0.119521	0.048043	2.487772	0.012854623	0.234239177
RASAL1	-1.320389	0.530772	-2.487674	0.012858141	NA

SHARPIN	-0.263292	0.105855	-2.487293	0.012871929	0.234239177
ZNF835	0.769677	0.309675	2.485436	0.012939282	NA
CARMIL1	-0.940716	0.378945	-2.482462	0.013047814	0.236980598
OSTF1	0.34996	0.141028	2.481484	0.013083645	0.237172634
LIMD1	-0.816246	0.329592	-2.476536	0.013266408	0.238923469
SLC9A9	-0.502482	0.202928	-2.476162	0.013280321	0.238923469
KIAA0040	0.46311	0.187187	2.474044	0.013359319	0.238923469
BHLHE41	1.756752	0.710126	2.473859	0.013366261	NA
MGAT1	0.25301	0.102287	2.473532	0.013378494	0.238923469
SHF	-0.751675	0.304032	-2.472354	0.013422649	0.238923469
ELMSAN1	0.318229	0.128723	2.472205	0.013428254	0.238923469
LINC02201	-1.237143	0.500463	-2.471998	0.013436017	0.238923469
MAN1B1.DT	-0.621304	0.2514	-2.471375	0.013459468	0.238923469
LPIN2	0.488001	0.197466	2.47132	0.013461538	0.238923469
FAM117A	0.302043	0.122233	2.471037	0.013472196	0.238923469
EEA1	0.404775	0.16383	2.470696	0.01348503	0.238923469
DDX5	-0.289184	0.117046	-2.470682	0.013485563	0.238923469
SLC2A14	-3.695871	1.496675	-2.469388	0.013534429	NA
NEK1	0.432185	0.175079	2.468513	0.013567576	0.239923794
ACAP1	0.284352	0.115228	2.467734	0.013597114	0.239994172
JRK	0.470737	0.190826	2.466834	0.013631357	0.240147163
ZNF605	-0.402652	0.163312	-2.465544	0.013680538	0.240562274
CCDC126	0.436936	0.177301	2.464369	0.013725485	0.240901501
DENND2D	0.390674	0.158639	2.462659	0.013791116	0.241194735
CD58	-0.569048	0.231119	-2.462141	0.013811051	0.241194735
JOSD2	-0.417872	0.169734	-2.461928	0.013819251	0.241194735
FAM204A	-0.225313	0.091657	-2.458233	0.013962265	0.24270801
GCNT1	0.795572	0.323643	2.458178	0.013964376	0.24270801
ENSG00000268400	-1.209155	0.492	-2.457634	0.01398557	NA
PLEKHO2	0.883051	0.359375	2.457187	0.014002992	0.24270801
ABTB1	-0.269904	0.10985	-2.457024	0.014009344	0.24270801
SLC38A11	1.622383	0.66075	2.455368	0.014074058	NA
CELF2	0.286704	0.116788	2.454916	0.014091759	0.243686212
PDE4B	0.226047	0.092192	2.451905	0.014210199	0.2448464
LAMTOR1	-0.191297	0.078028	-2.451626	0.014221242	0.2448464
PSPC1	-0.308529	0.125867	-2.451225	0.014237075	0.2448464
RBMX	0.129714	0.052976	2.448528	0.014344123	0.246236405
ZNF580	-0.264121	0.107899	-2.447864	0.014370596	0.246240689
MX1	0.702208	0.287038	2.446394	0.014429304	0.246516039
MINDY2	0.33607	0.137387	2.446148	0.014439172	0.246516039
ANKRD28	-0.593943	0.242932	-2.444893	0.014489526	0.24692676
UFD1	-0.324147	0.132716	-2.442405	0.014589774	0.248184738
MPP7	-0.768844	0.314984	-2.440898	0.014650791	0.248772015
CNOT6L	-0.609132	0.249759	-2.43888	0.014732849	0.24971382
JUND	-0.320256	0.131418	-2.436929	0.014812601	0.250222592
FAM131A	0.553611	0.227184	2.436842	0.014816162	0.250222592
NDUFB8	-0.167939	0.069094	-2.430584	0.01507453	0.254128977

OR2A1.AS1	-0.915744	0.377083	-2.428491	0.015161789	NA
MRPL54	-0.272029	0.112049	-2.427778	0.015191646	0.255644366
SNAP29	0.296079	0.121994	2.426996	0.015224406	0.255737343
PCF11	0.199098	0.082129	2.424206	0.015341894	0.257250694
COX16	-0.348454	0.14378	-2.423525	0.015370716	0.257274546
SLC35D1	0.579094	0.23918	2.421162	0.015470999	0.25849232
FBXO2	-0.868107	0.358694	-2.42019	0.015512397	0.258723631
ENSG00000276509	0.549238	0.227041	2.419116	0.015558267	0.258754598
ENSG00000271204	0.446237	0.184483	2.418857	0.015569366	0.258754598
PFDN1	-0.268706	0.111151	-2.417483	0.015628257	0.259005292
OSTM1	0.342269	0.141596	2.417219	0.015639617	0.259005292
USP53	0.951023	0.393614	2.416128	0.015686564	0.25917512
CAPN12	0.520111	0.215339	2.415315	0.01572159	0.25917512
IGHG3	1.031544	0.427422	2.413408	0.015804118	0.25917512
PPM1D	0.515373	0.21356	2.413248	0.015811045	0.25917512
CYP4V2	0.647396	0.268272	2.413205	0.015812925	0.25917512
NECAP2	-0.224156	0.092912	-2.412575	0.015840279	0.25917512
SLC25A26	-0.281432	0.116682	-2.41195	0.015867457	0.25917512
ENSG00000228463	0.475214	0.197031	2.411876	0.015870681	0.25917512
PCNX4	0.366652	0.15213	2.41013	0.015946849	0.259966853
ZSCAN18	0.234655	0.0974	2.409186	0.015988136	0.259997185
C9orf16	-0.177	0.07348	-2.408822	0.016004087	0.259997185
TBC1D8	-1.141503	0.474211	-2.407162	0.016077021	NA
ISCA2	-0.231081	0.095998	-2.407139	0.016078055	0.260010225
UBE2E1	0.330835	0.137468	2.406638	0.0161001	0.260010225
ENSG00000247373	0.828262	0.344283	2.405762	0.016138775	0.260010225
SCP2	-0.237532	0.098768	-2.404946	0.016174852	0.260010225
RAB5C	-0.186187	0.077423	-2.404805	0.016181119	0.260010225
ANP32B	0.159874	0.066488	2.404559	0.01619201	0.260010225
SENP3	-0.527752	0.219494	-2.404407	0.01619872	0.260010225
APOBEC3G	-0.221621	0.092247	-2.402466	0.016284945	0.26051831
NOTCH2	0.316716	0.131834	2.402393	0.016288191	0.26051831
SPOP	0.230537	0.095984	2.401823	0.016313607	0.26051831
FIBP	-0.206006	0.085922	-2.397583	0.016503629	0.262789448
IRF3	0.190959	0.079652	2.397402	0.016511797	0.262789448
DSCAML1	-1.257975	0.5253	-2.394773	0.016630648	NA
SMIM14	-0.261254	0.109127	-2.394033	0.016664259	0.264611317
ZBTB8OS	-0.258481	0.107987	-2.393629	0.01668263	0.264611317
POLR2M	-0.27314	0.114172	-2.392363	0.016740277	0.265077915
WASH9P	-0.436919	0.182695	-2.391521	0.016778721	0.265239374
PSMD8	-0.1616	0.067636	-2.389283	0.016881292	0.26566487
TRA2B	-0.169214	0.070852	-2.388264	0.016928187	0.26566487
SLA	0.395691	0.165689	2.388146	0.016933632	0.26566487
DRG2	-0.374524	0.156849	-2.387801	0.016949526	0.26566487
MME	-1.99894	0.837224	-2.387582	0.016959634	NA
STIP1	0.337602	0.14141	2.387394	0.016968297	0.26566487
SLC2A1	-0.317901	0.133172	-2.387137	0.016980174	0.26566487

COMMD2	-0.249164	0.1044	-2.386628	0.017003683	0.26566487
ERV3.1	-0.308685	0.129504	-2.383605	0.017143971	0.266991678
ENSG00000125726	-1.513501	0.634972	-2.383573	0.017145472	0.266991678
TMEM165	-0.33557	0.140836	-2.382706	0.017185928	0.267178577
RPRD1A	-0.253143	0.106299	-2.381423	0.017245898	0.267667743
UNC119	-0.320824	0.134824	-2.379565	0.017333103	0.267670508
DEAF1	-0.559263	0.235061	-2.379227	0.017349005	0.267670508
CPT1A	-0.508516	0.213751	-2.379011	0.017359154	0.267670508
FH	0.399364	0.167871	2.378991	0.0173601	0.267670508
RNF144B	0.740134	0.311525	2.375841	0.017508987	0.269523588
VDAC2	0.191583	0.080751	2.372531	0.01766669	0.271506087
LINC02035	0.853765	0.359949	2.371907	0.017696552	NA
GPS2	-0.252839	0.106614	-2.37153	0.017714626	0.271579817
SENP2	-0.476655	0.201042	-2.370915	0.01774409	0.271579817
ZNF776	0.483814	0.204095	2.370535	0.017762351	0.271579817
PET100	-0.237262	0.10011	-2.370019	0.017787177	0.271579817
ADGRB2	-1.314415	0.555073	-2.368005	0.017884304	NA
PLXNB2	-1.140005	0.481665	-2.366798	0.017942718	NA
CEP170	-0.321295	0.135904	-2.364123	0.018072807	0.275444519
ABHD3	0.379083	0.160436	2.362833	0.018135847	0.275444519
RPLP2	0.183504	0.077674	2.362488	0.018152716	0.275444519
WDR33	0.255383	0.108104	2.362388	0.018157631	0.275444519
ZKSCAN2	-0.714877	0.302723	-2.361487	0.018201827	NA
S100PBP	-0.346852	0.146939	-2.360516	0.018249543	0.276392277
ENSG00000226571	1.153364	0.488752	2.359815	0.018284059	NA
BCAP31	-0.217763	0.092295	-2.359418	0.018303611	0.276764745
COA6.AS1	-0.507295	0.215083	-2.358601	0.018343975	0.27692914
CALM3	-0.275938	0.117195	-2.354518	0.018546757	0.279541012
DDX55	0.398966	0.169857	2.348835	0.01883226	0.283389304
HEBP2	-0.517344	0.220341	-2.347926	0.018878282	0.283627308
MPLKIP	0.214016	0.091265	2.344994	0.0190274	0.285088417
SOX4	-0.63265	0.269834	-2.344591	0.019047978	0.285088417
ATP5ME	-0.156031	0.06656	-2.344226	0.019066616	0.285088417
RPL32	0.126919	0.054157	2.343551	0.019101136	0.285150498
MFAP1	0.382812	0.163438	2.342249	0.019167902	0.285693013
APOL6	0.745414	0.318619	2.339516	0.019308758	0.286759656
BUD13	0.394329	0.168588	2.339007	0.019335048	0.286759656
PHYH	-0.725462	0.310159	-2.339002	0.019335316	0.286759656
ARCN1	0.421139	0.18009	2.338494	0.019361621	0.286759656
ZFYVE27	0.325612	0.139387	2.336023	0.019490049	0.288207178
HSD17B4	0.418195	0.179128	2.334611	0.019563744	0.288394435
ZNF680	0.304522	0.130445	2.33448	0.019570626	0.288394435
CCL5	-1.362285	0.583795	-2.333497	0.019622064	0.288394435
ZDHHC23	-0.450347	0.192998	-2.33343	0.019625564	0.288394435
ATP6V1C1	0.347022	0.14888	2.330885	0.019759418	0.289266442
ENSG00000224746	-1.046286	0.449189	-2.329279	0.019844304	NA
HSH2D	-0.294635	0.126541	-2.328374	0.019892251	0.289266442

NPM1	0.127144	0.054626	2.327564	0.019935279	0.289266442
ENSG00000267390	0.472164	0.202874	2.327372	0.01994548	0.289266442
IDNK	-0.486209	0.208932	-2.327119	0.019958928	0.289266442
YY1	0.177093	0.076113	2.326715	0.019980414	0.289266442
RALA	-0.290484	0.12486	-2.326468	0.019993593	0.289266442
ACYP2	-0.300867	0.129329	-2.326366	0.019999008	0.289266442
DUS2	0.340592	0.146414	2.326236	0.020005974	0.289266442
C12orf49	-0.531167	0.228368	-2.325921	0.020022764	0.289266442
XYLT2	-0.707896	0.304353	-2.325902	0.020023769	0.289266442
KIAA0930	0.523746	0.225397	2.323659	0.020143784	0.290553203
PPP4R1	-0.524484	0.226113	-2.319569	0.020364217	0.293187165
CFL2	-0.380287	0.16398	-2.319114	0.020388841	0.293187165
ARL11	0.561761	0.242338	2.318087	0.020444599	0.29353943
PEX13	0.590358	0.254844	2.316544	0.020528594	0.294295422
FXYD5	0.161398	0.069749	2.314007	0.020667361	0.295833113
ENSG00000274400	1.581173	0.683324	2.313942	0.0206709	NA
RER1	-0.224169	0.096954	-2.312121	0.020770994	0.296711637
SMARCD1	-0.273623	0.118362	-2.311741	0.020791934	0.296711637
GSTP1	-0.19264	0.083357	-2.311011	0.020832223	0.296835464
GUK1	-0.200605	0.086842	-2.310007	0.020887758	0.297175825
DUS4L	-0.495216	0.214529	-2.308381	0.020977949	0.297900264
CCNQ	-0.316439	0.137187	-2.306625	0.021075746	0.297900264
FHL3	-0.814912	0.353332	-2.30636	0.021090507	0.297900264
CLNS1A	0.329814	0.143022	2.306033	0.021108768	0.297900264
NUP160	0.256503	0.111236	2.305925	0.021114847	0.297900264
LENG1	0.277682	0.120441	2.305556	0.021135451	0.297900264
A4GALT	1.441525	0.625285	2.305387	0.021144911	NA
RTCA	0.374968	0.162696	2.304719	0.021182295	0.297900264
HGSNAT	-0.411296	0.178484	-2.304387	0.021200956	0.297900264
POU2AF1	-0.19466	0.084489	-2.303972	0.021224204	0.297900264
ENSG00000275441	-0.663706	0.288347	-2.301759	0.021348747	NA
ZNF526	0.388417	0.16878	2.301315	0.021373832	0.299552659
PCMTD2	-0.265623	0.115619	-2.297403	0.021595809	0.302212589
ENSG00000170846	-0.832788	0.362727	-2.29591	0.021681039	NA
CEP152	0.420129	0.183025	2.295471	0.02170615	0.302890942
LAMTOR4	-0.156069	0.067992	-2.295425	0.021708797	0.302890942
FAM111A.DT	0.323071	0.140842	2.293852	0.02179899	0.303698096
ZNF579	-0.932055	0.406461	-2.2931	0.021842273	NA
ING3	-0.330455	0.144174	-2.292064	0.021901975	0.304191004
FOXN2	0.395889	0.172728	2.291985	0.021906482	0.304191004
LDHB	0.173779	0.075852	2.291035	0.021961417	0.304191004
PTK2B	0.23838	0.104051	2.290991	0.021963951	0.304191004
POLA1	-0.790528	0.34525	-2.289727	0.022037176	NA
PSPH	-0.749264	0.327712	-2.286353	0.022233632	NA
PFKFB3	-0.314353	0.137499	-2.286218	0.022241515	0.306542512
PCYT2	-0.470029	0.205632	-2.285773	0.022267538	0.306542512
MSL3	0.25067	0.109699	2.285062	0.022309167	0.306542512

ENSG00000272941	0.956558	0.418614	2.285058	0.022309431	NA
OPN3	-0.489965	0.214443	-2.28482	0.022323372	0.306542512
HCK	-0.463672	0.202992	-2.284191	0.02236033	0.306542512
MAP4	-0.234469	0.102653	-2.284089	0.022366326	0.306542512
LMF1	-0.607699	0.266065	-2.284028	0.022369889	0.306542512
STK16	-0.242341	0.10615	-2.283009	0.02242985	0.306542512
ABCG1	-0.628384	0.275523	-2.280694	0.022566551	0.306542512
SLC15A2	0.868573	0.380848	2.28063	0.022570371	0.306542512
WDR11	-0.330921	0.145144	-2.279952	0.022610557	0.306542512
LDLRAP1	0.481656	0.211263	2.279888	0.022614316	0.306542512
CYBC1	-0.169237	0.074238	-2.279638	0.022629158	0.306542512
RPSA	0.191754	0.084117	2.279626	0.022629903	0.306542512
ENSG00000251661	-1.525007	0.668974	-2.279621	0.022630155	NA
TMEM242	-0.290094	0.12728	-2.279175	0.02265664	0.306542512
SNHG25	-0.332722	0.146011	-2.278744	0.02268227	0.306542512
TBCB	-0.20982	0.092089	-2.278433	0.022700804	0.306542512
BARD1	0.370916	0.162819	2.278088	0.022721362	0.306542512
LZTS3	-1.388416	0.609656	-2.277377	0.022763702	NA
PDCD4	0.43204	0.189776	2.276583	0.022811143	0.307093248
RPL3	0.116615	0.05123	2.276308	0.022827592	0.307093248
MIR210HG	-1.389388	0.610397	-2.276204	0.022833796	NA
STK38L	-0.496584	0.218409	-2.273646	0.02298728	0.308799089
AAAS	-0.417062	0.183618	-2.271355	0.023125473	0.310054802
PHF7	-0.471557	0.207643	-2.271003	0.023146796	0.310054802
WDFY2	0.288527	0.127196	2.268369	0.023306753	0.311665409
HNRNPD	-0.185858	0.08195	-2.267931	0.023333417	0.311665409
ZNF827	1.028256	0.453654	2.26661	0.023414041	NA
PPWD1	0.304475	0.134392	2.265576	0.023477372	0.31314279
KIAA1324L	1.020768	0.450816	2.264268	0.02355761	NA
SUMO1	-0.163519	0.07224	-2.263552	0.023601656	0.314353976
ICMT	-0.677418	0.299376	-2.262767	0.023650032	0.314552125
NMI	0.249371	0.110241	2.262051	0.023694234	0.314613259
STX7	0.219384	0.09702	2.261225	0.023745335	0.314613259
DNAAF2	0.299748	0.132569	2.261066	0.023755144	0.314613259
ACSM1	-0.805007	0.35607	-2.260811	0.023770933	NA
TLE1	-0.693686	0.306972	-2.259769	0.023835613	0.315234377
VPS9D1	-1.096947	0.486001	-2.257086	0.024002674	NA
SORL1	-0.295079	0.130786	-2.256201	0.024058036	0.317728492
USPL1	0.503223	0.223132	2.255271	0.024116298	0.31805061
TOLLIP.AS1	-0.898371	0.39844	-2.254719	0.024150981	NA
ARRDC4	-1.91203	0.848174	-2.254289	0.024177995	NA
NAT14	-0.629125	0.279285	-2.252626	0.024282751	0.319796678
ENSG00000253948	-0.563772	0.250357	-2.251878	0.024329957	0.319969603
HFE	-1.034696	0.459758	-2.250524	0.024415711	NA
SLC30A1	-1.149455	0.510771	-2.250431	0.02442158	NA
DHRS4L2	0.452477	0.201067	2.25038	0.024424852	0.320768331
KMT2C	0.243814	0.108383	2.249567	0.02447643	0.320996754

GM2A	0.5507	0.244887	2.248797	0.02452539	0.321190254
RHOC	-0.581917	0.258912	-2.247547	0.024605072	0.321355972
STX5	-0.240923	0.107195	-2.247525	0.02460649	0.321355972
ADRB2	0.882184	0.392541	2.247367	0.024616572	NA
ZNF90	-0.331622	0.147592	-2.246886	0.024647303	0.321441907
OAS2	0.496481	0.221024	2.246278	0.024686198	0.32150263
UBA52	0.124058	0.05534	2.24175	0.024977528	0.324846243
GATM	0.953598	0.425551	2.240854	0.025035539	0.325048523
TOPORS	0.237629	0.106063	2.240441	0.025062314	0.325048523
ENSG00000228106	-0.348156	0.155527	-2.238559	0.025184599	0.326183978
MED11	0.243303	0.108719	2.237911	0.025226847	0.326281121
MXD4	0.419217	0.187411	2.236889	0.025293564	0.326609567
CNN3	-0.633211	0.283131	-2.236458	0.025321807	0.326609567
MRPS36	0.18978	0.084896	2.235447	0.025388029	0.326810273
IFT88	-0.379353	0.169721	-2.235158	0.025406975	0.326810273
PPP1CA	-0.136524	0.061106	-2.234228	0.025468081	0.327148129
IGHV3.15	-1.135136	0.508247	-2.233432	0.025520472	NA
STAC3	-0.993426	0.444856	-2.233138	0.025539858	NA
C1orf21	-1.810576	0.810999	-2.232526	0.025580243	NA
SPAG7	-0.170155	0.076279	-2.230677	0.025702501	0.329708306
KLHDC10	-0.483652	0.216928	-2.229556	0.025776932	0.33021199
APH1A	-0.158532	0.071126	-2.228907	0.025820125	0.3302735
FBXO22	0.275965	0.123866	2.22793	0.02588518	0.3302735
SLC25A5	-0.152667	0.068529	-2.227766	0.025896151	0.3302735
HDDC2	-0.212119	0.095247	-2.227031	0.025945221	0.3302735
ZNF718	0.804765	0.361425	2.226646	0.025970953	0.3302735
PELI2	-0.608531	0.273344	-2.22625	0.025997471	0.3302735
IFNLR1	-0.490539	0.220388	-2.225795	0.026027944	0.3302735
ENSG00000270820	0.764293	0.343462	2.225265	0.026063458	0.330277831
PLAAT3	-1.543901	0.693936	-2.224846	0.026091608	NA
TRIP11	0.274642	0.123558	2.222767	0.026231492	0.331846604
PDE4D	-0.79384	0.35722	-2.222273	0.026264858	0.331846604
C3orf33	-0.813403	0.36608	-2.221928	0.0262882	NA
JUP	-0.694638	0.312639	-2.221852	0.026293277	0.331846604
PIGZ	-1.225145	0.551469	-2.221605	0.026310031	NA
RPS16	0.151937	0.068453	2.219569	0.026448067	0.333304107
TXNL1	0.174406	0.078593	2.219103	0.026479751	0.333304107
CMTM3	-0.82268	0.370826	-2.218507	0.026520262	0.33336715
PTPN20	-1.69566	0.764934	-2.216739	0.026640919	NA
MAPKAPK2	-0.355317	0.160304	-2.216518	0.026656025	0.334625124
CYP3A5	0.468584	0.211519	2.215329	0.026737479	0.334625124
RAB31	0.640004	0.288923	2.215135	0.02675082	0.334625124
RPS8	0.170743	0.077086	2.214959	0.026762883	0.334625124
WIPF2	0.280429	0.12667	2.213844	0.026839494	0.3348799
IMPACT	-0.459489	0.20758	-2.213554	0.026859485	0.3348799
CUL2	0.373228	0.168644	2.213107	0.02689025	0.3348799
GNAI3	-0.210352	0.095082	-2.212322	0.026944447	0.335110404

UCP2	-0.280931	0.127087	-2.210532	0.027068292	0.336205379
CD69	-0.305024	0.138049	-2.209531	0.027137711	0.336622333
BASP1	-0.325277	0.14748	-2.205569	0.027414195	0.339603283
ATP6V0A1	0.430274	0.195139	2.204956	0.027457194	0.33968781
FGR	-0.524923	0.238211	-2.20361	0.027551795	0.340409672
LINC00926	-0.215194	0.097722	-2.202105	0.027657864	0.340605158
NDUFA6	-0.175399	0.079657	-2.201933	0.027670058	0.340605158
SERPINF1	-0.873992	0.397	-2.20149	0.027701351	0.340605158
YTHDC1	0.177826	0.080781	2.201329	0.027712709	0.340605158
FDXR	-0.730491	0.332017	-2.200162	0.027795368	NA
ALAS1	0.48397	0.220036	2.199501	0.027842283	0.341364533
POLR3K	0.253452	0.115249	2.199171	0.02786575	0.341364533
EPB41L2	-0.569273	0.258887	-2.198921	0.027883557	0.341364533
TRG.AS1	1.510739	0.687268	2.198181	0.02793621	NA
CCDC130	0.268664	0.122283	2.197058	0.028016291	0.342542929
ROR1	-0.878881	0.400053	-2.196912	0.028026751	NA
RAB13	-0.823281	0.375187	-2.194321	0.028212334	0.343634365
METTL15	-0.312593	0.142473	-2.194059	0.028231195	0.343634365
NFKB2	-0.336243	0.153253	-2.194042	0.028232418	0.343634365
NBPF9	0.513445	0.234063	2.193618	0.028262895	0.343634365
CARM1	0.489756	0.2233	2.193262	0.028288537	0.343634365
SWAP70	0.209191	0.095406	2.192631	0.02833397	0.343741576
NLRC5	-0.44029	0.200875	-2.191867	0.028389107	0.343966085
HIBCH	0.301183	0.137508	2.190294	0.028502952	0.344900408
THRAP3	-0.230928	0.105489	-2.189117	0.028588341	0.345264161
SINHCAF	0.24091	0.11009	2.188297	0.028647997	0.345264161
LMBRD1	-0.188708	0.08624	-2.188185	0.028656114	0.345264161
DAZAP1	-0.303799	0.13888	-2.187489	0.028706873	0.345264161
RPL34	0.119191	0.054491	2.187352	0.028716859	0.345264161
ENSG00000258302	1.439461	0.658133	2.187188	0.028728827	NA
NDUFAF4	0.294009	0.134481	2.186246	0.028797642	0.345353679
SHLD1	-0.450191	0.20592	-2.186243	0.028797863	0.345353679
APP	0.32251	0.147582	2.185293	0.028867346	0.345636404
RPS15A	0.152591	0.069839	2.184915	0.028895056	0.345636404
CRYM	-1.951094	0.893163	-2.184476	0.028927321	NA
EXOSC3	0.284212	0.130111	2.184374	0.028934751	0.345670884
METRNL	1.174133	0.537878	2.1829	0.02904317	NA
CTSH	-0.299355	0.137228	-2.181435	0.029151264	0.347814955
ACAA2	-0.307573	0.141038	-2.180779	0.029199748	0.347932515
PSMG4	-0.315321	0.144623	-2.1803	0.029235224	0.347932515
SAP18	-0.124049	0.056964	-2.17767	0.029430644	0.349802547
MGME1	0.268137	0.123158	2.177184	0.02946686	0.349802547
ZNF345	0.573071	0.263378	2.175851	0.029566382	0.349940697
CERS4	-0.287415	0.13212	-2.175407	0.029599604	0.349940697
ASCL2	-1.215169	0.558738	-2.174847	0.029641566	NA
RPS12	0.136187	0.062631	2.174449	0.029671439	0.349940697
FNBP1	-0.289253	0.133032	-2.174322	0.029680949	0.349940697

DCXR	-0.27333	0.12571	-2.174289	0.029683438	0.349940697
CABLES1	-0.62706	0.288431	-2.17404	0.029702102	0.349940697
ZNF358	-0.444606	0.204617	-2.172871	0.029790005	0.350536526
MED17	0.254988	0.117502	2.170082	0.030000611	0.352013862
MRPL43	-0.192535	0.088762	-2.169123	0.030073324	0.352013862
SNX5	0.225561	0.103995	2.16896	0.030085715	0.352013862
ST3GAL1	0.42875	0.197694	2.168756	0.030101211	0.352013862
ELOVL5	0.186207	0.085872	2.168437	0.030125482	0.352013862
IKZF3	0.165099	0.076144	2.168239	0.030140484	0.352013862
ENSG00000271895	-0.896038	0.414089	-2.163876	0.030473838	NA
KIF9.AS1	1.160133	0.536293	2.163245	0.030522378	NA
UQCRFS1	-0.192724	0.089138	-2.162092	0.030611063	0.35706569
ODC1	-0.736137	0.340751	-2.160335	0.030746733	0.358203253
PREX1	0.4147	0.192473	2.154593	0.031193688	0.362960012
CTSB	0.261021	0.121259	2.152599	0.031350225	0.364329971
MBP	-0.228751	0.1064	-2.149927	0.031561023	0.365704186
PPID	0.361163	0.167991	2.149892	0.031563795	0.365704186
ENSG00000253982	-0.336155	0.156379	-2.14962	0.031585314	0.365704186
CBY1	0.578267	0.269151	2.148487	0.031675116	0.36629229
LRRC47	-0.555334	0.258637	-2.147153	0.031781104	0.367065884
PRR5L	-1.608997	0.749366	-2.147143	0.031781881	NA
GNG7	-0.166578	0.077633	-2.145704	0.031896632	0.367947636
ERICH1	0.183139	0.085385	2.144861	0.031963923	0.368271461
KLB	-1.568704	0.731463	-2.144613	0.031983811	NA
POLR3D	0.376195	0.175485	2.143747	0.032053163	0.368847055
L1CAM	-0.832631	0.388655	-2.14234	0.032166153	NA
COG8	0.334498	0.156174	2.141824	0.032207637	0.36949918
MATR3	-0.710117	0.331551	-2.141806	0.032209061	0.36949918
THRB	1.551006	0.724241	2.141559	0.032228961	NA
ABRACL	0.336099	0.156981	2.141015	0.032272789	0.36949918
MIEN1	-0.187822	0.087756	-2.14027	0.032332928	0.36949918
PCYT1A	0.407309	0.190336	2.139942	0.03235947	0.36949918
BCL2L13	-0.396896	0.185512	-2.13946	0.032398424	0.36949918
SCAMP4	-0.400914	0.1874	-2.139346	0.03240765	0.36949918
RPL35	0.137769	0.064422	2.138549	0.032472213	0.36949918
RELB	-0.337908	0.158017	-2.138423	0.032482428	0.36949918
DDX23	0.339865	0.158996	2.137574	0.032551369	0.36949918
CYTH3	-0.568964	0.26629	-2.136633	0.032627884	0.36949918
ST6GALNAC6	-0.240997	0.112817	-2.136186	0.032664235	0.36949918
ZNF500	0.439966	0.205963	2.13614	0.032667985	0.36949918
ENSG00000228434	0.34828	0.163055	2.135969	0.032681918	0.36949918
SERPINB1	-0.2132	0.099825	-2.135746	0.032700087	0.36949918
TNKS1BP1	-1.716027	0.803574	-2.135493	0.032720768	NA
JTB	-0.105467	0.049395	-2.135188	0.032745667	0.369569482
CCDC121	1.336731	0.626266	2.134447	0.03280618	NA
MYO18A	1.010107	0.473288	2.134231	0.032823873	NA
HCST	0.852019	0.399592	2.132224	0.032988468	0.371721707

PEX16	-0.187587	0.087991	-2.131894	0.033015538	0.371721707
ENSG00000235501	-0.9729	0.456605	-2.130725	0.033111803	NA
NR6A1	-0.789484	0.37053	-2.130689	0.033114804	NA
NR2F6	0.974482	0.457364	2.130648	0.033118171	NA
ENSG00000267519	-0.502407	0.23599	-2.128934	0.033259735	0.374022651
CGAS	0.414205	0.194658	2.127864	0.033348372	0.37403956
DNAJA1	0.30746	0.144539	2.127173	0.033405718	0.37403956
SLC7A6	0.448629	0.210978	2.126426	0.033467791	0.37403956
GLTP	-0.229345	0.107894	-2.125657	0.033531808	0.37403956
ENSG00000272221	0.719362	0.338429	2.125593	0.033537169	0.37403956
PARP15	0.53892	0.253559	2.125417	0.033551796	0.37403956
LRRC59	0.351973	0.165622	2.125161	0.033573196	0.37403956
ARPC1A	0.229952	0.108208	2.12508	0.033579909	0.37403956
SMG9	-0.297196	0.140003	-2.122777	0.03377251	0.375657565
ENSG00000283013	-0.265646	0.125185	-2.122035	0.033834824	0.375657565
GATD1	0.33595	0.158324	2.121911	0.033845186	0.375657565
FAM149B1	-0.692567	0.32651	-2.121119	0.033911813	NA
ZBED5.AS1	-0.339829	0.160216	-2.121069	0.03391601	0.375724302
HELZ2	0.487976	0.230081	2.120888	0.033931226	0.375724302
FAM114A2	0.420066	0.198174	2.119684	0.034032664	0.3760526
CNR1	0.677918	0.319835	2.119586	0.03404097	0.3760526
IFRD2	-0.231184	0.109103	-2.118938	0.034095677	0.376214346
ABI1	0.22921	0.108286	2.116713	0.03428423	0.377095867
SIPA1L3	-0.300803	0.142152	-2.116064	0.034339356	0.377095867
SCNM1	-0.170765	0.080714	-2.115663	0.034373438	0.377095867
ENSG00000277654	-0.473586	0.223871	-2.115442	0.03439226	0.377095867
ZNF234	0.35334	0.167135	2.114093	0.034507365	0.377095867
HMGA1	-0.274342	0.129781	-2.113882	0.034525349	0.377095867
PTPRA	-0.558446	0.264214	-2.113613	0.03454834	0.377095867
CLMN	0.477013	0.225713	2.113362	0.034569793	0.377095867
PDE3B	-0.414958	0.196354	-2.113317	0.034573684	0.377095867
AGTRAP	-0.380857	0.180221	-2.113276	0.034577161	0.377095867
MPP6	0.36802	0.174232	2.112247	0.034665232	0.377357199
DPP3	-0.471278	0.223137	-2.112058	0.034681498	0.377357199
MDFIC	0.791488	0.374761	2.111981	0.034688095	NA
DYNC1LI2	0.299869	0.142031	2.111282	0.034748095	0.377644224
APOBEC3B	-2.108066	0.99951	-2.109099	0.034935996	NA
IL13RA1	-0.614724	0.291512	-2.108745	0.03496659	0.37944706
SNX8	-0.401353	0.190363	-2.108355	0.035000299	0.37944706
PXK	0.220265	0.104509	2.107616	0.03506423	0.37944706
APLP2	-0.419192	0.198906	-2.107484	0.035075617	0.37944706
ECI1	-0.51488	0.244404	-2.106678	0.035145497	0.379574887
INAFM1	-0.525864	0.249649	-2.106415	0.03516828	0.379574887
AFF2	-1.131643	0.537358	-2.105941	0.035209519	NA
ENSG00000273748	-0.836944	0.397433	-2.105874	0.035215341	0.379646441
PHF19	-0.655375	0.311409	-2.104547	0.035330746	0.380453793
ZFP82	0.365894	0.173942	2.103547	0.035417996	0.380956457

ENSG00000263080	-1.273838	0.606006	-2.102021	0.035551448	NA
IGLC2	-0.665612	0.31672	-2.10158	0.035590085	0.382016851
MIS18BP1	0.172707	0.082201	2.10105	0.035636545	0.382016851
ZNF622	0.251015	0.119473	2.101027	0.035638633	0.382016851
RPL5	0.135118	0.064373	2.098996	0.035817253	0.38349374
RP2	0.537969	0.256435	2.097877	0.035916027	0.384113322
INAVA	-2.001256	0.95432	-2.097048	0.035989299	NA
RNF26	-0.339252	0.16179	-2.096868	0.036005241	0.384629364
UBE2T	0.487243	0.232478	2.095868	0.03609394	0.385138743
TCL1A	-0.353778	0.168852	-2.095191	0.036154026	0.385237027
TP73.AS1	-0.451535	0.215558	-2.094726	0.036195315	0.385237027
SMIM27	-0.257464	0.122931	-2.094379	0.036226229	0.385237027
WDR66	-0.819242	0.391374	-2.093246	0.036327192	0.38566652
NUMA1	0.358348	0.171266	2.092352	0.036407015	0.38566652
RAC1	-0.353913	0.169148	-2.092324	0.036409499	0.38566652
ARPC5	-0.151219	0.072295	-2.091708	0.036464617	0.38566652
FADS3	-0.264256	0.126362	-2.091252	0.036505495	0.38566652
KHDRBS1	-0.348965	0.166969	-2.090003	0.036617572	0.38566652
CFL1	-0.180319	0.086296	-2.08955	0.036658274	0.38566652
SMARCE1	-0.203028	0.097168	-2.089442	0.036667957	0.38566652
NUPL2	0.254747	0.121923	2.089419	0.036669989	0.38566652
GCC2	0.228526	0.109377	2.089338	0.036677338	0.38566652
INTS3	0.346863	0.166103	2.088244	0.03677581	0.386269416
ZDHHC3	-0.261446	0.125306	-2.086462	0.036936796	0.387151784
PCNA	0.486717	0.233281	2.086401	0.036942279	0.387151784
RNF181	-0.17129	0.082179	-2.084344	0.037128865	0.388673404
ZBTB44	0.190833	0.091599	2.083351	0.03721921	0.38918528
LAPTM5	-0.177142	0.085071	-2.082277	0.037317198	0.389775845
ADPRHL2	0.279609	0.134351	2.08119	0.037416487	0.390152468
SPPL2A	-0.20385	0.097959	-2.080973	0.037436355	0.390152468
ACAP3	0.541	0.26008	2.080127	0.037513911	0.390279819
RBM25	0.164788	0.079235	2.079745	0.037548927	0.390279819
AUP1	-0.163006	0.078419	-2.078659	0.037648721	0.390279819
TRIB3	0.443367	0.213299	2.078619	0.037652387	0.390279819
PDE7B	-0.472164	0.227158	-2.078575	0.037656392	0.390279819
SLC5A6	-0.409269	0.19697	-2.077827	0.037725287	0.390562787
LACTB2	0.403507	0.194282	2.076917	0.037809183	0.391000247
ARID5B	-0.183721	0.088501	-2.075935	0.037899976	0.391280365
ABHD15	-0.307562	0.148171	-2.075723	0.037919609	0.391280365
GOSR2	-0.322543	0.155482	-2.074475	0.038035202	0.392042309
ZNHIT3	0.170507	0.082225	2.07367	0.038109936	0.392381904
ATRAID	-0.17861	0.086208	-2.071854	0.038279081	0.393487111
PFDN5	0.103412	0.049924	2.071402	0.038321252	0.393487111
GPSM3	-0.098943	0.047772	-2.071169	0.038342993	0.393487111
HINFP	0.342721	0.16551	2.070692	0.038387621	0.39351502
RAP2A	0.762256	0.368301	2.069657	0.038484445	NA
ENSG00000270189	0.55138	0.266682	2.067555	0.038681901	0.395219312

HSPA5	0.334863	0.161983	2.067276	0.038708155	0.395219312
SRPK1	-0.273897	0.132493	-2.067256	0.038710068	0.395219312
ERGIC2	0.221042	0.106932	2.067127	0.038722233	0.395219312
HNRNPA0	-0.112035	0.054214	-2.066553	0.038776274	0.395341161
POLDIP2	-0.242816	0.117594	-2.064865	0.03893576	0.396536646
RAB11FIP5	-1.070835	0.518828	-2.06395	0.039022479	NA
ABHD12	-0.439992	0.213318	-2.062612	0.03914948	0.39815827
SLC25A1	-0.316675	0.153555	-2.062294	0.039179791	0.39815827
GKAP1	0.288437	0.139913	2.061551	0.039250492	0.39827542
AHCYL1	0.270482	0.131224	2.061225	0.039281537	0.39827542
BRWD1	-0.219949	0.106728	-2.060837	0.039318564	0.39827542
CRNDE	-1.620745	0.787312	-2.058579	0.039534576	NA
ENSG00000268516	0.355781	0.172881	2.057952	0.039594724	0.400640579
ENSG00000262222	-0.600937	0.292107	-2.057246	0.039662613	0.400895523
DLG2	1.15147	0.559793	2.056955	0.039690545	NA
DTX3L	0.516388	0.251104	2.056468	0.039737448	0.401100313
UBQLN1	0.265586	0.129167	2.056147	0.039768306	0.401100313
CHCHD3	-0.220864	0.107462	-2.055275	0.039852417	0.401435006
RBM12B	0.376819	0.183386	2.054791	0.039899162	0.401435006
DHX57	-0.377611	0.183907	-2.05327	0.040046439	0.401435006
MRPL14	-0.211942	0.103237	-2.052972	0.040075316	0.401435006
TERF2IP	0.221451	0.107874	2.052861	0.040086033	0.401435006
BAG1	-0.190218	0.092665	-2.052758	0.040096102	0.401435006
CDK5RAP2	-0.321694	0.156717	-2.05271	0.040100749	0.401435006
DOCK8	0.284733	0.138764	2.051927	0.040176786	0.4015242
UBE2J2	-0.210887	0.102784	-2.051737	0.040195181	0.4015242
LPCAT4	0.345506	0.168736	2.047608	0.040598428	0.405121402
NUDT12	0.8107	0.396047	2.04698	0.040660086	NA
CHCHD5	-0.294622	0.14393	-2.046973	0.040660719	0.405312264
TMEM231	-1.153115	0.563584	-2.046039	0.040752513	NA
PRR13	-0.145965	0.071372	-2.045114	0.040843586	0.4053796
CYB5R3	0.24117	0.117943	2.044796	0.040874939	0.4053796
KLC4	0.428557	0.209721	2.043461	0.041006792	0.4053796
HIST1H2BN	0.537565	0.263091	2.043267	0.04102599	0.4053796
CHMP2B	0.224817	0.11004	2.043058	0.04104666	0.4053796
RSBN1L	0.180861	0.088525	2.043049	0.041047562	0.4053796
MZF1	0.409398	0.200526	2.041622	0.041189088	0.4053796
PF4	3.158337	1.546978	2.041617	0.0411895	NA
RACK1	0.116613	0.057123	2.041433	0.041207838	0.4053796
CEP57	0.185106	0.090686	2.041179	0.041233018	0.4053796
ZNF865	-0.548254	0.268644	-2.040817	0.041269055	0.4053796
AZIN1	-0.260124	0.127463	-2.040782	0.041272465	0.4053796
APH1B	-0.40471	0.198318	-2.040716	0.041279068	0.4053796
RAB9A	0.342886	0.168023	2.040704	0.041280241	0.4053796
ZBTB1	0.296125	0.145139	2.040286	0.041321809	0.4053796
DAD1	-0.169094	0.082881	-2.04019	0.041331437	0.4053796
ENSG00000224521	-1.733902	0.849903	-2.040118	0.041338565	NA

TMED4	-0.208415	0.102168	-2.039921	0.041358217	0.4053796
ZMIZ2	-0.396226	0.194288	-2.03937	0.041413147	0.405494737
EFHB	1.581973	0.775981	2.038674	0.041482624	NA
FUT7	1.506134	0.739024	2.038004	0.041549517	NA
NUP188	0.430951	0.2115	2.037595	0.041590454	0.406298289
RAB30	0.176617	0.086684	2.037496	0.041600387	0.406298289
DRAM1	-0.518963	0.254737	-2.03725	0.041625022	0.406298289
USP44	0.681509	0.334635	2.036576	0.041692524	NA
HNRNPA1L2	-0.321812	0.158031	-2.036385	0.04171167	0.406721273
CENPF	-0.748229	0.367497	-2.036015	0.041748876	NA
TUNAR	-1.372752	0.674335	-2.035713	0.041779171	NA
RUBCNL	-0.21954	0.107853	-2.035547	0.04179588	0.407119618
SERGEF	0.37133	0.182601	2.033562	0.041995823	0.408643297
MED21	0.356508	0.17551	2.031275	0.042227128	0.409558319
CD2	-2.600764	1.28053	-2.031006	0.042254393	NA
HNRNPL	-0.266722	0.131377	-2.030197	0.042336471	0.409558319
MYL12A	0.1294	0.06374	2.030111	0.042345287	0.409558319
EEFSEC	-0.323095	0.159164	-2.029953	0.042361326	0.409558319
KLC1	0.301492	0.148545	2.029633	0.04239388	0.409558319
RPF2	0.307267	0.151415	2.029312	0.042426495	0.409558319
ENSG00000246790	-0.623532	0.307271	-2.029255	0.042432326	0.409558319
MRPL21	0.23687	0.116737	2.029095	0.042448581	0.409558319
HSPE1	0.160059	0.078903	2.028542	0.042504932	0.409558319
MAU2	-0.362268	0.178618	-2.028176	0.04254227	0.409558319
CNBD2	-0.451097	0.222445	-2.027908	0.04256964	0.409558319
ENSG00000259038	-0.675706	0.333315	-2.027229	0.042639016	NA
NACA	0.12199	0.060214	2.025926	0.042772392	0.410323033
DHX15	0.257623	0.127165	2.025885	0.042776532	0.410323033
ZBED1	-0.368215	0.181758	-2.025849	0.042780218	0.410323033
VPS37C	-0.66734	0.32955	-2.025005	0.042866847	NA
MEX3C	0.294154	0.145345	2.023838	0.042986827	0.410428198
ENSG00000283103	0.311725	0.154046	2.023591	0.043012232	0.410428198
ENSG00000273270	-0.471284	0.232908	-2.023481	0.043023581	0.410428198
INPP5B	0.346979	0.171485	2.02338	0.043034027	0.410428198
KCNK6	0.458079	0.2264	2.023315	0.043040724	0.410428198
COPG2	-0.585705	0.289496	-2.023191	0.043053437	0.410428198
ITGB2.AS1	-1.8836	0.931132	-2.022914	0.043082027	NA
C1orf35	0.202304	0.100044	2.022151	0.043160803	0.410545591
ANKLE2	-0.330305	0.163349	-2.022079	0.043168238	0.410545591
PMF1	-0.222601	0.1101	-2.021801	0.043196916	0.410545591
MIB1	0.542843	0.268606	2.020965	0.043283426	0.410659524
HIP1R	-0.269899	0.133558	-2.02084	0.043296372	0.410659524
ZNF74	0.855972	0.423813	2.019695	0.043415008	NA
ENSG00000261770	0.890543	0.4411	2.018914	0.043496146	NA
SPN	-0.868668	0.430413	-2.018221	0.043568221	NA
TMUB1	-0.223652	0.110824	-2.018096	0.043581316	0.412928502
ENSG00000271133	-1.70875	0.846858	-2.017754	0.043616939	NA

ATP5IF1	-0.124159	0.06155	-2.017208	0.043673761	0.412928502
CBX6	-0.173391	0.085967	-2.016961	0.043699625	0.412928502
ZBTB43	-0.347655	0.172376	-2.016847	0.043711494	0.412928502
HSPA8	0.257992	0.127962	2.016158	0.043783442	0.41319248
ARL2BP	-0.257589	0.127824	-2.015185	0.043885239	0.413737344
IGHA2	-0.968913	0.480942	-2.014615	0.043945053	0.413752165
ZNF174	0.491474	0.243989	2.01433	0.043974937	0.413752165
ENSG00000234936	-0.847418	0.421195	-2.011939	0.044226389	NA
SIGIRR	-0.217889	0.108312	-2.01169	0.044252668	0.415948503
CEP295	0.32575	0.162008	2.010709	0.044356173	0.416103904
FGD2	0.201837	0.100382	2.010694	0.044357828	0.416103904
CEP95	0.304247	0.151403	2.009516	0.044482441	0.416603229
MANF	0.240253	0.119567	2.009352	0.044499791	0.416603229
ZNF138	0.370673	0.184644	2.0075	0.04469647	0.417650493
THOC6	-0.277715	0.138342	-2.007461	0.044700612	0.417650493
INPP5K	-0.388239	0.19352	-2.006198	0.044835093	0.418021336
VDAC1	0.313071	0.156088	2.005726	0.0448855	0.418021336
FNIP1	-0.265683	0.132469	-2.005629	0.044895855	0.418021336
ZNF785	0.340177	0.169651	2.005164	0.044945528	0.418021336
TXLNG	0.349464	0.174296	2.005001	0.044962891	0.418021336
COLCA2	-0.888989	0.443481	-2.004571	0.045008941	NA
ANKRD12	-0.178774	0.089232	-2.00347	0.045126864	0.418624462
TEX264	-0.214608	0.107157	-2.002751	0.045204007	0.418624462
ACSF3	0.276478	0.138055	2.002664	0.045213386	0.418624462
ALG13	-0.231193	0.115445	-2.002628	0.045217218	0.418624462
CLSPN	-1.136596	0.567576	-2.002544	0.045226309	NA
BCR	0.451057	0.225281	2.002198	0.045263466	0.418624462
DTD2	-0.473358	0.236454	-2.001902	0.045295256	0.418624462
AEBP2	0.349603	0.174708	2.001074	0.04538438	0.419035718
TAF6L	-0.682853	0.341438	-1.999931	0.045507764	0.419762188
GINM1	0.305392	0.152792	1.998745	0.045635906	0.420230332
MGAT5B	-0.594044	0.297225	-1.998633	0.045648023	0.420230332
ZPBP2	-1.135336	0.569019	-1.995253	0.046015245	NA
NINL	-1.296684	0.65003	-1.994805	0.046064104	NA
DPH5	0.293308	0.14711	1.993798	0.046174082	0.42436526
MIS12	0.322968	0.161996	1.993675	0.046187571	0.42436526
SOCS4	0.423835	0.212727	1.992389	0.04632841	0.425243177
CDKN1B	0.180564	0.090681	1.991198	0.046459149	0.425903445
CTDP1	-0.648776	0.325897	-1.990737	0.046509789	0.425903445
NOP9	0.638462	0.320787	1.990301	0.046557817	NA
SURF4	-0.252835	0.127044	-1.990133	0.046576309	0.425903445
RPL21	0.121054	0.060838	1.989762	0.046617126	0.425903445
ARHGAP30	0.204894	0.102979	1.989672	0.046627129	0.425903445
LDB1	0.767572	0.38578	1.98966	0.046628353	NA
TTC7A	-0.374097	0.188098	-1.988845	0.046718282	0.426321352
P2RX1	-0.350855	0.176514	-1.98769	0.046846	0.42707179
GATC	0.338515	0.170347	1.98721	0.046899162	0.42714174

BTBD3	-0.709347	0.35709	-1.986467	0.046981447	NA
ELMO3	-0.759993	0.382653	-1.986117	0.047020336	NA
KMT2A	0.198344	0.099866	1.986102	0.047021946	0.427845034
TAF9	-0.213637	0.107623	-1.985039	0.047140136	0.428505207
HMGCL	0.319202	0.160865	1.984287	0.047223874	0.428851236
TCF25	0.152731	0.076999	1.983543	0.047306848	0.429078598
PRADC1	-0.476493	0.240288	-1.983007	0.047366667	0.429078598
IGIP	0.918936	0.463447	1.982829	0.047386521	NA
INSR	-0.590533	0.29788	-1.982451	0.047428746	0.429078598
TRAF3IP1	-0.683633	0.34485	-1.982408	0.04743358	NA
SYK	0.173307	0.087427	1.982304	0.047445246	0.429078598
NIPAL2	0.784683	0.395861	1.982218	0.047454874	NA
BSDC1	0.199557	0.100684	1.982017	0.047477387	0.429078598
IER2	-0.177019	0.089357	-1.981042	0.047586522	0.429647581
EXOSC7	-0.27955	0.141141	-1.980638	0.047631856	0.429647581
NREP	-0.40684	0.205528	-1.979489	0.047761032	0.430399316
NDC80	1.199463	0.60616	1.978789	0.047839804	NA
PSME3	-0.359801	0.181907	-1.977939	0.047935604	0.430913836
SLTM	0.149808	0.07574	1.977925	0.04793712	0.430913836
SNHG30	-0.262755	0.132855	-1.97776	0.0479558	0.430913836
P4HB	-0.193851	0.098045	-1.977163	0.048023217	0.431107081
CDK6	-0.533176	0.269922	-1.975296	0.048234592	0.432455343
VAMP2	-0.153303	0.077621	-1.975023	0.048265516	0.432455343
KLHL5	-0.372646	0.188746	-1.974321	0.048345284	0.432757122
TOMM34	0.329322	0.166912	1.97303	0.048492097	0.433590982
ZNF28	0.655319	0.332195	1.972691	0.04853079	0.433590982
SAR1B	0.307308	0.155892	1.971285	0.048691247	0.433851034
TMEM187	0.510546	0.259	1.97122	0.048698742	0.433851034
RND1	-0.577673	0.293069	-1.971118	0.048710405	0.433851034
BET1L	0.298595	0.151508	1.970818	0.048744711	0.433851034
NIPSNAP2	-0.188966	0.095934	-1.969752	0.048866781	0.434525635
CLK3	0.182957	0.092935	1.968656	0.04899264	0.435232632
SCAF11	0.121093	0.061589	1.966159	0.049280288	0.437374202
PAQR4	-1.475493	0.750484	-1.966054	0.04929243	NA
NAGK	0.245674	0.125043	1.964717	0.049446995	0.438037442
IARS2	0.303225	0.154336	1.964706	0.049448316	0.438037442
MREG	-0.733682	0.373648	-1.963565	0.049580566	NA
MYL6	-0.135468	0.069009	-1.963049	0.049640463	0.438342841
SMIM12	-0.188649	0.096102	-1.963015	0.049644406	0.438342841
ARRDC2	-0.258979	0.131937	-1.962889	0.049659089	0.438342841
RAN	0.130253	0.066361	1.962799	0.049669519	0.438342841
ZFP62	-0.296203	0.150986	-1.961785	0.049787473	0.438391688
ADAMTS7	-0.740964	0.377717	-1.961692	0.049798326	0.438391688
TSGA10	0.485954	0.247759	1.961398	0.049832555	0.438391688
CLOCK	-0.288394	0.147054	-1.961148	0.049861802	0.438391688

Cluster 2	log2FC	lfcSE	stat	pvalue	padj
ARRDC3	-1.177752	0.110552	-10.65342	< 2.22e-16	< 2.22e-16
NFKBIA	-0.519261	0.069716	-7.448257	9.46E-14	3.14E-10
H3F3B	-0.3961	0.054845	-7.222115	5.12E-13	1.13E-09
CD83	-0.465405	0.065285	-7.128808	1.01E-12	1.68E-09
CKS2	1.220771	0.183998	6.634679	3.25E-11	4.32E-08
GPR183	0.855737	0.146652	5.835136	5.37E-09	5.95E-06
CHORDC1	0.751185	0.135081	5.560986	2.68E-08	2.54E-05
CHMP6	-0.798552	0.148419	-5.380405	7.43E-08	6.17E-05
RSRP1	0.398926	0.074696	5.340668	9.26E-08	6.83E-05
SMIM10	-3.791334	0.710869	-5.333376	9.64E-08	NA
GATD3	4.608235	0.895634	5.145222	2.67E-07	0.00017737
CDKN1A	-0.777446	0.152389	-5.101708	3.37E-07	0.000203124
CD79B	-0.30552	0.061409	-4.9752	6.52E-07	0.000336284
CLK1	0.536565	0.107891	4.973194	6.59E-07	0.000336284
NOP10	0.268179	0.055204	4.857916	1.19E-06	0.000562466
HMGB2	-0.404119	0.086356	-4.679673	2.87E-06	0.001271543
ARL17A	-0.719897	0.154759	-4.651723	3.29E-06	0.001365657
TAGAP	-0.52709	0.113688	-4.636295	3.55E-06	0.001385039
BLNK	0.327873	0.072122	4.546109	5.46E-06	0.001997552
ERP29	0.28176	0.062109	4.536572	5.72E-06	0.001997552
ATF7IP2	-0.688388	0.152688	-4.508469	6.53E-06	0.002167211
CXCR4	0.180207	0.040347	4.466365	7.96E-06	0.002514843
KRTCAP2	-0.298987	0.067355	-4.438965	9.04E-06	0.002633078
IGHD	-0.443708	0.100003	-4.436971	9.12E-06	0.002633078
MIF	-0.405706	0.092884	-4.367875	1.25E-05	0.003470044
SLC3A2	-0.485364	0.112259	-4.323628	1.53E-05	0.003894959
TTC32	-0.70869	0.16416	-4.31706	1.58E-05	0.003894959
TIMM10	-0.522847	0.121124	-4.316633	1.58E-05	0.003894959
SNHG9	-0.479621	0.112649	-4.257672	2.07E-05	0.004897097
EMP3	-0.395834	0.09465	-4.182092	2.89E-05	0.006265733
HSBP1	0.355446	0.085127	4.175497	2.97E-05	0.006265733
RPL8	0.378487	0.090702	4.172846	3.01E-05	0.006265733
IGLC2	-0.417639	0.100107	-4.171912	3.02E-05	0.006265733
LCN8	3.779297	0.91018	4.15225	3.29E-05	0.006622356
C16orf74	-0.626001	0.151076	-4.143606	3.42E-05	0.00662472
C7orf50	-0.46358	0.112011	-4.138687	3.49E-05	0.00662472
ENSG00000273319	0.595867	0.144949	4.110884	3.94E-05	0.007118565
PIM1	-0.484403	0.117878	-4.109343	3.97E-05	0.007118565
JMJD6	0.803095	0.196072	4.095909	4.21E-05	0.007345732
SRSF5	-0.310027	0.075984	-4.080149	4.50E-05	0.007660381
ENSG00000272211	-1.000086	0.247674	-4.03792	5.39E-05	0.008949206
DNAJA1	0.449953	0.11191	4.020684	5.80E-05	0.009364755
TCL1B	-1.397902	0.348103	-4.015767	5.93E-05	0.009364755
RIN3	0.699153	0.174454	4.00766	6.13E-05	0.009466618
CLEC2D	-0.4074	0.102081	-3.99093	6.58E-05	0.009929051
GPR18	0.915276	0.230187	3.976223	7.00E-05	NA

SLC50A1	-0.471271	0.118949	-3.961948	7.43E-05	0.010966079
CCT8	0.604433	0.152816	3.955306	7.64E-05	0.011030136
ENSG00000235078	0.897166	0.227923	3.936271	8.28E-05	NA
RBM3	-0.381252	0.097665	-3.903668	9.47E-05	0.013381326
H2AFZ	0.270115	0.069346	3.895181	9.81E-05	0.01356992
ENSG00000224505	-0.518755	0.134323	-3.862001	0.00011246	0.015156338
CIRBP	-0.20865	0.054078	-3.858332	0.00011416	0.015156338
ADAMTS6	1.038987	0.270359	3.842992	0.00012154	0.015819682
GPR65	0.582748	0.1529	3.811294	0.00013824	0.017402028
TCTN1	-1.118132	0.293763	-3.806232	0.0001411	0.017402028
PNPLA8	0.372045	0.097767	3.805418	0.00014157	0.017402028
YWHAE	0.332198	0.087523	3.795569	0.00014731	0.01753509
C6orf226	-0.657625	0.173309	-3.794517	0.00014793	0.01753509
SRSF11	0.243417	0.06425	3.788566	0.00015152	0.017645355
FCGR2B	0.781868	0.207361	3.770556	0.00016288	0.018635642
GAPT	0.454182	0.120589	3.766371	0.00016564	0.018635642
MED21	0.590251	0.157641	3.744268	0.00018092	0.019823043
GLRX	0.411067	0.109836	3.742547	0.00018216	0.019823043
ATP6V0E2	-0.748252	0.202734	-3.690805	0.00022355	0.023933828
TDG	0.414617	0.113062	3.667155	0.00024526	0.025842274
CYB5A	-0.615565	0.168883	-3.644924	0.00026747	0.027691383
SCRN1	1.100879	0.30231	3.641552	0.000271	NA
MRPL32	0.368212	0.101178	3.639261	0.00027342	0.027691383
ZBED2	1.836315	0.505329	3.633903	0.00027917	NA
EPS15	-0.598349	0.164815	-3.630421	0.00028296	0.027691383
ZFAND2A	-0.550626	0.151758	-3.628322	0.00028527	0.027691383
PPM1K	0.261652	0.072149	3.626555	0.00028723	0.027691383
PMF1	-0.388777	0.107219	-3.626002	0.00028784	0.027691383
SMIM4	-0.430031	0.119555	-3.596922	0.00032201	0.030535312
PTGIR	-1.757506	0.490371	-3.584032	0.00033833	NA
TNFSF10	0.593193	0.16605	3.572367	0.00035377	0.033074967
JADE1	0.430241	0.1214	3.543992	0.00039412	0.035693292
TMEM9B	-0.392535	0.110957	-3.537736	0.00040357	0.035693292
MRPL54	-0.395262	0.111741	-3.537295	0.00040425	0.035693292
UBE2L3	-0.254563	0.072021	-3.534546	0.00040848	0.035693292
SLC16A7	0.448153	0.126796	3.534427	0.00040866	0.035693292
BOLA2B	-0.656056	0.186723	-3.513531	0.00044219	0.038057031
PSMA4	-0.262787	0.074857	-3.510544	0.00044719	0.038057031
STX7	0.275863	0.07883	3.499448	0.00046622	0.039174463
EXOSC3	0.511944	0.146527	3.493869	0.00047608	0.039502285
CREBRF	0.307288	0.088038	3.490393	0.00048231	0.03952566
PIGF	0.670396	0.19241	3.484197	0.00049362	0.03995878
HSPE1	0.251856	0.072368	3.480204	0.00050103	0.040070533
ZCCHC9	0.472834	0.136043	3.475622	0.00050967	0.040276106
TNFRSF14	0.216856	0.062655	3.461119	0.00053793	0.041575926
SNAPC3	0.435739	0.125908	3.460764	0.00053865	0.041575926
AP4B1	0.930623	0.269796	3.449359	0.00056192	NA

PDE4DIP	-0.730859	0.212573	-3.438153	0.0005857	0.044440773
IL21R	-0.841727	0.244958	-3.436215	0.0005899	NA
COMMD3	-0.391135	0.113848	-3.435582	0.00059128	0.044440773
IFIT2	0.696895	0.203159	3.430294	0.00060293	0.044440773
CYTIP	0.236633	0.06899	3.429976	0.00060363	0.044440773
ELMSAN1	0.389727	0.113707	3.427469	0.00060924	0.044440773
ROR1	-1.299246	0.379567	-3.422969	0.00061941	NA
IZUMO4	-0.707578	0.207504	-3.409944	0.00064976	0.046881794
RPL30	0.155733	0.046092	3.378734	0.0007282	0.051161528
RHOH	0.241595	0.07151	3.378473	0.0007289	0.051161528
DMAC2L	-0.333608	0.098861	-3.374534	0.00073941	0.051161528
ZNF441	-0.663633	0.196749	-3.372991	0.00074356	0.051161528
UCP2	-0.344525	0.102237	-3.369861	0.00075206	0.051161528
CD48	0.333025	0.09886	3.368668	0.00075532	0.051161528
WDR13	-0.358117	0.106677	-3.357012	0.0007879	0.052828943
SGK1	-1.622911	0.484192	-3.351795	0.0008029	NA
EIF4A2	0.220356	0.066073	3.335052	0.00085284	0.056611173
ENSG00000237596	-1.005042	0.30171	-3.331156	0.00086486	NA
IL23A	-1.016229	0.305228	-3.329407	0.00087031	0.056663465
IL2RG	-0.410417	0.123275	-3.329284	0.0008707	0.056663465
CRIPT	0.406671	0.122514	3.319372	0.0009022	0.058143904
POU2AF1	-0.343366	0.103625	-3.313531	0.00092126	0.058292334
UBE2J2	-0.331044	0.099914	-3.313285	0.00092207	0.058292334
ENSG00000237491	-0.854833	0.258775	-3.303381	0.00095527	NA
PRICKLE1	0.844305	0.255656	3.302497	0.00095828	0.059695566
LIMD2	-0.235713	0.071399	-3.301337	0.00096225	0.059695566
NFX1	0.386971	0.117462	3.294432	0.00098621	0.060406824
LRIF1	0.481814	0.146412	3.290818	0.00099897	0.060406824
TMEM243	0.245528	0.074623	3.290241	0.00100102	0.060406824
RPS9	0.296649	0.090295	3.285353	0.00101855	0.060910845
ZNF564	-0.9478	0.290249	-3.265477	0.0010928	NA
MSMP	-0.692931	0.212248	-3.264732	0.00109568	0.0643658
CYTH1	-0.319651	0.097911	-3.264723	0.00109571	0.0643658
MME	-2.329112	0.717147	-3.247748	0.00116322	NA
TPI1	-0.177085	0.054558	-3.245794	0.00117123	0.068198679
SAYSD1	0.441649	0.136225	3.242062	0.00118668	0.068497346
ANAPC15	-0.519052	0.160439	-3.235196	0.00121559	0.069561277
TAGLN2	-0.282729	0.087631	-3.226362	0.00125375	0.071131305
PTPMT1	-0.407196	0.126389	-3.221766	0.00127403	0.071669699
SCN3A	1.319171	0.410129	3.216475	0.00129776	NA
ENSG00000259001	1.439227	0.448179	3.211276	0.00132147	NA
DBI	-0.256013	0.079723	-3.211269	0.0013215	0.07371532
NRROS	-0.546232	0.170326	-3.206977	0.00134138	0.074200514
DTX3L	0.548116	0.171045	3.204511	0.00135292	0.074220598
KRCC1	0.274702	0.085806	3.201446	0.0013674	0.074399878
KIAA0040	0.594552	0.186143	3.194059	0.00140287	0.075709556
MRNIP	0.362635	0.113625	3.19151	0.00141531	0.075764782

ZC3H12A	-0.503614	0.158215	-3.183092	0.00145711	0.077378534
DYNLL1	0.403626	0.126941	3.179623	0.00147467	0.07768936
DOCK9.DT	-1.802569	0.56736	-3.177116	0.00148748	NA
FADS1	-0.571351	0.180151	-3.171514	0.00151647	0.079262185
ING3	-0.339441	0.10733	-3.162604	0.00156365	0.081089727
ABTB1	-0.280605	0.088842	-3.158466	0.00158602	0.081612388
ZBTB16	1.920903	0.608894	3.154742	0.0016064	NA
TMEM60	0.398212	0.126299	3.152917	0.00161648	0.082360607
IGHG1	1.33429	0.423433	3.15112	0.00162646	0.082360607
THAP5	0.456929	0.145122	3.148577	0.00164067	0.082360607
TNFAIP8L2	0.587545	0.186707	3.146887	0.00165019	0.082360607
MARCHF7	-0.312542	0.099685	-3.135289	0.00171685	0.085048152
SIGLEC5	1.883248	0.600694	3.135122	0.00171783	NA
HHEX	0.388525	0.124006	3.133102	0.0017297	0.0850498
TLE1	-0.793759	0.2537	-3.128734	0.00175561	0.085643246
UBE2I	-0.165457	0.052917	-3.126739	0.00176757	0.085643246
SYNGR2	-0.553756	0.177597	-3.118047	0.00182054	0.087215452
LMO4	0.438017	0.140574	3.115929	0.00183366	0.087215452
NAGK	0.403179	0.129431	3.115002	0.00183943	0.087215452
SAP18	-0.209103	0.067292	-3.107399	0.00188741	0.088087234
CDC42	-0.282256	0.090835	-3.107333	0.00188784	0.088087234
UROD	-0.426598	0.137385	-3.105126	0.00190198	0.088087234
UQCRH	0.196568	0.063333	3.103742	0.0019109	0.088087234
KLHDC7B	-0.903374	0.291816	-3.095695	0.00196352	NA
RPL35A	0.189463	0.061345	3.088475	0.00201187	0.092101847
SYNCRIP	0.391192	0.126746	3.086426	0.00202579	0.09210388
EVI2A	0.503732	0.16355	3.079991	0.00207007	0.093476862
MEGF6	-1.059687	0.344472	-3.076263	0.00209613	NA
CLEC2B	0.417045	0.135657	3.07426	0.00211025	0.094336515
LYRM7	0.311791	0.101485	3.072298	0.00212417	0.094336515
MAVS	0.403705	0.131447	3.071237	0.00213174	0.094336515
THEMIS2	0.971331	0.316823	3.065851	0.00217052	0.09541645
PHKG1	-0.512084	0.167215	-3.062425	0.00219552	0.09588051
NREP	-0.876796	0.286688	-3.058364	0.00222549	NA
IGHG3	1.527124	0.499509	3.057251	0.00223377	0.096582783
MARCHF1	0.401624	0.131408	3.056323	0.0022407	0.096582783
AUP1	-0.243508	0.079827	-3.050461	0.0022849	0.097295085
NDUFS8	-0.248862	0.081588	-3.050246	0.00228654	0.097295085
ENSG00000239636	-0.751415	0.246867	-3.043809	0.00233604	0.097914563
HLA.A	-0.463217	0.152185	-3.043781	0.00233625	0.097914563
HIPK2	0.943667	0.310042	3.043672	0.0023371	NA
TTC7A	-0.5576	0.183264	-3.042612	0.00234535	0.097914563
GINS2	-1.102848	0.363504	-3.033936	0.00241386	NA
ZNF557	0.565987	0.186713	3.031324	0.00243484	0.10101523
ENSG00000279483	-0.285339	0.094472	-3.020356	0.00252478	0.104096145
MT.CO1	0.222812	0.073816	3.018474	0.00254052	0.104098419
ENSG00000263394	0.631845	0.209598	3.014551	0.0025736	0.104807086

COLCA2	-1.381221	0.458436	-3.0129	0.00258764	NA
ARL6IP6	0.369167	0.122548	3.012426	0.00259169	0.104900226
BLOC1S1	-0.211451	0.070291	-3.008202	0.00262798	0.105724474
CAMTA1	-0.306899	0.102261	-3.001143	0.00268968	0.107554905
ENSG00000275964	-1.020822	0.34018	-3.000831	0.00269244	NA
BLVRB	0.661982	0.22106	2.994584	0.0027482	0.108673679
ACAP1	0.369048	0.123333	2.992299	0.00276885	0.108673679
C17orf49	-0.819006	0.273953	-2.98959	0.00279353	0.108673679
ARHGAP30	0.274711	0.091903	2.989135	0.00279768	0.108673679
TMEM138	-0.352863	0.118056	-2.988935	0.00279952	0.108673679
PARVB	0.646323	0.21641	2.986565	0.0028213	0.108882652
EBLN2	0.594972	0.199451	2.983051	0.0028539	0.109066659
ARID5B	-0.325892	0.109268	-2.982512	0.00285893	0.109066659
FXYD7	0.816187	0.274116	2.977521	0.0029059	0.110224898
ZNHIT3	0.261632	0.088185	2.966872	0.00300846	0.111288028
HNRNPA0	-0.141821	0.047827	-2.965296	0.00302392	0.111288028
PILRB	0.694089	0.234122	2.964648	0.00303029	0.111288028
IFIT5	0.529993	0.178795	2.964253	0.00303418	0.111288028
RUBCNL	-0.262173	0.088473	-2.963328	0.00304332	0.111288028
TMC6	-0.339092	0.11443	-2.963306	0.00304354	0.111288028
METTL26	-0.272233	0.091892	-2.962524	0.00305128	0.111288028
RPS5	0.229804	0.077673	2.958613	0.00309027	0.112093934
ENSG00000271204	0.524355	0.17746	2.954779	0.00312893	0.112879456
SNRPA1	-0.287459	0.097347	-2.952928	0.00314776	0.112944981
PPP1R16A	-0.673571	0.228767	-2.94435	0.00323634	NA
MAPK14	0.601882	0.204535	2.942679	0.00325386	0.116095692
HRK	-1.430781	0.486479	-2.941094	0.00327055	0.116095692
FIS1	-0.29289	0.099731	-2.936808	0.00331609	0.116279999
SP140	0.287727	0.097986	2.936405	0.0033204	0.116279999
ST6GALNAC6	-0.355432	0.121074	-2.935669	0.00332829	0.116279999
CERS4	-0.324136	0.110501	-2.93333	0.00335347	0.116546196
DDB2	-0.517366	0.176624	-2.929196	0.0033984	0.117492558
PTPRA	-0.788848	0.26933	-2.928922	0.0034014	NA
GLRX2	0.512648	0.175477	2.921449	0.00348408	0.119830523
NBPF9	0.876661	0.300284	2.919444	0.00350656	NA
RPL19	0.142523	0.048872	2.916232	0.00354287	0.121160197
PHF23	0.395438	0.135666	2.914793	0.00355924	0.121160197
RTL10	0.732556	0.251731	2.91007	0.00361348	NA
LIMD1	-0.907512	0.312354	-2.9054	0.00366784	0.12386072
SRSF6	-0.342588	0.117942	-2.904714	0.00367589	0.12386072
DNAAF2	0.459293	0.158231	2.902675	0.0036999	0.12404008
SAP25	0.730464	0.252072	2.897844	0.00375738	NA
RACK1	0.159888	0.055175	2.897813	0.00375774	0.125346221
ENSG00000266844	-1.715952	0.592891	-2.89421	0.00380114	NA
ZNF426	0.407027	0.140649	2.893927	0.00380457	0.125763709
CFL1	-0.215241	0.074384	-2.893631	0.00380815	0.125763709
SLC2A14	-3.605707	1.248446	-2.888156	0.00387507	NA

ACTN1	2.844679	0.985118	2.887652	0.00388129	NA
GPS2	-0.367503	0.127399	-2.884668	0.00391826	0.128672843
ENSG00000256092	-0.550564	0.190948	-2.883325	0.00393501	0.128672843
C6orf203	0.499207	0.17331	2.880423	0.00397143	0.129227039
UXT	0.209451	0.072955	2.87096	0.00409228	0.132509952
JUND	-0.31461	0.109662	-2.868914	0.00411884	0.132722622
ENSG00000254614	-0.76304	0.266145	-2.867012	0.00414367	NA
ADA	-0.602061	0.210216	-2.864013	0.00418311	0.133465057
SLC5A3	-0.418507	0.146194	-2.862684	0.0042007	0.133465057
MAML3	-1.107093	0.386748	-2.86257	0.0042022	0.133465057
PEX10	-0.586348	0.204967	-2.860699	0.00422708	0.133615873
THEM6	0.789358	0.276362	2.856247	0.00428682	NA
EPB41L2	-0.590689	0.206834	-2.855857	0.00429208	0.135027707
ENSG00000089127	0.482521	0.169054	2.854244	0.00431394	0.135075254
RPL38	0.169596	0.059546	2.848156	0.00439734	0.13704021
SCAND1	-0.223233	0.07842	-2.846619	0.00441863	0.13706001
MED23	-0.578377	0.203351	-2.844227	0.00445193	0.137450794
SLC38A2	0.364225	0.128349	2.837764	0.00454307	0.139506764
EIF3E	0.208432	0.073481	2.836534	0.00456062	0.139506764
CAB39L	-1.175181	0.414606	-2.834452	0.00459043	NA
ASPSCR1	-0.48013	0.169424	-2.833898	0.0045984	0.139506764
MUC20.OT1	-0.372565	0.131481	-2.833607	0.00460259	0.139506764
DRG2	-0.420148	0.148409	-2.831014	0.00464006	0.139856574
PDE4B	0.25732	0.090929	2.829899	0.00465627	0.139856574
ATP6V1C1	0.453115	0.16038	2.825252	0.00472435	0.14126237
CHM	0.631983	0.223919	2.822373	0.00476698	0.141897675
MYCBP2	0.241982	0.085831	2.819274	0.00481324	0.142635222
NAE1	-0.365645	0.129831	-2.816309	0.00485789	0.143318471
PTPN20	-2.618712	0.930067	-2.815619	0.00486834	NA
VAV3.AS1	-1.212294	0.430636	-2.815124	0.00487584	NA
FAM111A.DT	0.526128	0.187411	2.807351	0.00499508	0.146388181
GM2A	0.828694	0.295388	2.805438	0.00502483	0.146388181
SNHG30	-0.374087	0.133353	-2.805228	0.0050281	0.146388181
HLA.C	-0.473834	0.169468	-2.796013	0.00517373	0.14940857
SPATC1L	-0.576267	0.206118	-2.795818	0.00517686	0.14940857
MGST2	-0.93523	0.334741	-2.79389	0.00520782	NA
SNHG32	0.217864	0.078053	2.791226	0.00525088	0.150384931
ST13	0.137263	0.049182	2.790911	0.005256	0.150384931
FAM184B	-1.703321	0.610989	-2.78781	0.00530657	0.151180355
TOMM20	0.231839	0.083218	2.785939	0.00533728	0.151405511
PDE6A	-1.360102	0.488534	-2.784048	0.0053685	NA
NKIRAS2	-0.335691	0.120585	-2.783859	0.00537164	0.151731799
CLECL1	1.392572	0.500736	2.781049	0.00541835	NA
HSPD1	0.293641	0.105789	2.775714	0.00550807	0.154926122
CREG1	0.891341	0.321585	2.77171	0.00557627	NA
EIF5	0.220205	0.079486	2.770369	0.00559928	0.156827039
KCP	-2.303337	0.83183	-2.768998	0.00562289	NA

ASF1A	0.446854	0.161519	2.766574	0.00566487	0.157997421
MRPL35	0.465375	0.168536	2.761274	0.00575764	0.159383877
TIAL1	0.27525	0.099729	2.759986	0.00578038	0.159383877
ICA1L	-0.456645	0.165473	-2.759634	0.00578661	0.159383877
PRMT7	0.552167	0.200281	2.756961	0.00583413	0.160028659
ENSG00000225342	-0.695043	0.252229	-2.755598	0.0058585	NA
IL27RA	0.419602	0.152427	2.752797	0.00590885	0.160755563
PRPF38A	0.256866	0.093316	2.752661	0.00591131	0.160755563
CDCA4	0.495646	0.180253	2.749724	0.00596455	0.160755563
CFAP410	-0.346298	0.125973	-2.74899	0.00597792	0.160755563
MTHFR	0.465091	0.169199	2.748782	0.00598172	0.160755563
INSR	-1.042519	0.379873	-2.744386	0.00606242	NA
GPS1	-0.391351	0.143236	-2.732221	0.00629089	0.16838263
LINC00926	-0.217827	0.079778	-2.730403	0.00632569	0.168634229
ENSG00000224746	-1.240809	0.455704	-2.722839	0.00647235	NA
ISG20L2	0.346455	0.127337	2.72077	0.006513	0.172933276
GATM	0.932994	0.343118	2.719162	0.00654475	NA
KLK1	1.288407	0.474082	2.717687	0.00657399	NA
MRPL17	0.464257	0.170914	2.716324	0.00660112	0.174574687
COL9A3	-1.250024	0.460569	-2.714083	0.00664595	0.175062854
TNFAIP1	0.677419	0.249686	2.713089	0.00666591	NA
APOOL	0.630127	0.232283	2.712748	0.00667277	NA
SUSD3	0.359441	0.132563	2.711466	0.00669865	0.175142349
ORMDL3	-0.407972	0.150504	-2.710702	0.0067141	0.175142349
RPS28	0.176311	0.065059	2.71001	0.00672813	0.175142349
CHL1	1.830075	0.675749	2.708216	0.0067646	NA
SLC23A3	1.006803	0.372102	2.705719	0.00681566	NA
ENSG00000267002	0.337488	0.124895	2.702178	0.00688869	0.177541415
POLR2M	-0.320334	0.118553	-2.702019	0.00689198	0.177541415
LTBP3	0.335759	0.124281	2.701607	0.00690053	0.177541415
IGLV6.57	-0.994622	0.368324	-2.700401	0.0069256	NA
FKBP5	1.031473	0.382043	2.69989	0.00693625	NA
FNIP1	-0.40962	0.151736	-2.699558	0.00694317	0.177948853
ENSG00000274265	-0.276371	0.102437	-2.69797	0.00697637	0.178111979
IFI44	1.015015	0.37812	2.68437	0.00726666	NA
LHFPL4	1.162362	0.433869	2.679063	0.00738286	NA
RAB9A	0.457246	0.170753	2.677822	0.00741027	0.18844412
MAP3K9	0.762881	0.284943	2.677316	0.00742146	NA
CCDC66	0.386815	0.144519	2.676577	0.00743784	0.18844412
NAB2	0.847115	0.317023	2.672096	0.00753792	NA
GRAP2	1.087227	0.407108	2.670612	0.00757132	NA
TNFAIP8	0.320975	0.120221	2.669878	0.00758788	0.191514586
LILRB1	0.467936	0.175652	2.663989	0.00772201	0.194161632
SERP1	0.14527	0.054579	2.661655	0.00777574	0.194775026
CAV1	-1.120652	0.421164	-2.660842	0.00779455	NA
LDHA	-0.343209	0.129074	-2.659009	0.00783708	0.19557356
CD180	0.42468	0.159837	2.656963	0.00788481	0.196027543

NPM1	0.183985	0.069302	2.654816	0.00793516	0.196416569
RNASEK	-0.416085	0.15679	-2.653777	0.00795964	0.196416569
RING1	-0.328167	0.123832	-2.650103	0.00804673	0.1978303
POLE3	-0.256062	0.096679	-2.64857	0.0080833	0.197996135
LETMD1	0.284733	0.107587	2.646539	0.00813202	0.198457271
DHPS	0.223618	0.084591	2.643519	0.00820492	0.199022222
TRIM69	0.435499	0.164787	2.642804	0.00822225	0.199022222
PFKP	-0.942396	0.356716	-2.641864	0.00824512	0.199022222
IFIT3	0.711901	0.269514	2.64142	0.00825592	NA
LY9	0.449578	0.170317	2.639656	0.00829903	0.19918574
ENSG00000253948	-0.793616	0.300801	-2.638342	0.00833126	NA
COL19A1	-0.483302	0.183213	-2.637916	0.00834173	0.19918574
RSBN1L	0.242608	0.09197	2.637908	0.00834192	0.19918574
NEIL1	-0.362759	0.137666	-2.63506	0.00841224	0.200144919
NSMCE1	-0.267368	0.101546	-2.632964	0.00846434	0.200312071
ARF5	-0.300007	0.113974	-2.632244	0.0084823	0.200312071
C15orf61	0.429294	0.163158	2.631144	0.00850979	0.200312071
WIPF2	0.371925	0.141549	2.627526	0.00860082	0.201574678
RNF144B	0.767639	0.29219	2.627193	0.00860925	NA
RHOC	-0.67186	0.25579	-2.626604	0.00862417	0.201574678
RP9	0.51712	0.197189	2.622459	0.00872977	0.20332695
PODXL2	-1.428765	0.54505	-2.621347	0.00875831	NA
OTUD1	0.666187	0.254198	2.620737	0.00877399	NA
HMG20B	-0.252825	0.096491	-2.620206	0.00878768	0.203960128
PPP4R2	0.31663	0.121068	2.615314	0.00891456	0.206184049
NDUFB5	0.222033	0.08504	2.610924	0.0090298	0.20812444
XKR6	1.134278	0.434976	2.607677	0.00911589	NA
ENSG00000272994	-0.402656	0.154419	-2.607552	0.00911923	0.208973808
HCK	-0.630644	0.241889	-2.607162	0.00912962	0.208973808
PSMG3	-0.401087	0.154017	-2.604174	0.00920961	0.209697096
PHF20	0.214318	0.082315	2.603624	0.0092244	0.209697096
BSG	-0.209967	0.08075	-2.60022	0.00931639	0.210434413
ZBTB25	0.453066	0.174251	2.600079	0.00932024	0.210434413
POU2F2	0.275913	0.106266	2.596436	0.00941965	0.211958171
CCDC120	0.982708	0.378604	2.595608	0.00944237	NA
MORC3	0.341039	0.131579	2.5919	0.00954476	0.213837884
DDX6	-0.283449	0.109394	-2.591077	0.00956762	0.213837884
NAA35	0.580176	0.223926	2.590935	0.00957157	NA
XPO4	-0.420877	0.162759	-2.585887	0.00971287	0.21613234
ASNSD1	0.309805	0.119843	2.585089	0.0097354	0.21613234
USP49	-0.866485	0.336194	-2.577338	0.00995646	NA
NFE2L2	0.279894	0.108661	2.575853	0.00999933	0.221251798
NACA	0.142626	0.055409	2.574069	0.01005104	0.221657066
LINC01250	-2.722735	1.057815	-2.573925	0.01005522	NA
MRPL28	-0.241128	0.093748	-2.572088	0.01010873	0.222191214
MINPP1	0.721592	0.280701	2.570672	0.01015013	NA
PCMTD2	-0.366568	0.142913	-2.564976	0.01031831	0.225225992

LINC01215	-0.311626	0.121517	-2.564463	0.01033357	0.225225992
MTPN	0.221086	0.086229	2.563959	0.01034859	0.225225992
MGAT3	1.228248	0.479077	2.563777	0.01035402	NA
LILRA4	1.941654	0.757507	2.563216	0.01037076	NA
SLC25A45	0.567909	0.221708	2.561521	0.01042149	0.225868897
SKAP2	0.245428	0.095844	2.560699	0.01044618	0.225868897
VMAC	-0.558851	0.218247	-2.560639	0.01044798	NA
GALNT2	-0.537012	0.209981	-2.557435	0.01054472	0.226632175
CCDC191	-0.757304	0.296246	-2.556336	0.01057809	0.226632175
CGGBP1	-0.244446	0.095631	-2.556145	0.01058391	0.226632175
ENSG00000261087	0.868756	0.340427	2.55196	0.01071187	NA
FGFR1OP2	0.283897	0.111335	2.549929	0.01077448	0.22997095
ALOX5	-0.274426	0.107723	-2.547506	0.0108496	0.230832149
CHPF2	-0.543453	0.213641	-2.543766	0.01096644	0.232572694
GNG11	1.179621	0.463731	2.543763	0.01096654	NA
SDHAF4	0.957667	0.376538	2.543345	0.01097968	NA
REST	0.282099	0.110976	2.541996	0.01102216	0.232610752
AGTRAP	-0.459464	0.180786	-2.541483	0.01103832	0.232610752
RNF43	-0.739766	0.291237	-2.540087	0.01108249	NA
ZEB1	-0.310664	0.122449	-2.537096	0.01117765	0.234131411
GTF2E2	0.344685	0.135864	2.53699	0.01118103	0.234131411
ENSG00000278834	-1.030856	0.407344	-2.530677	0.01138426	NA
RPL9	0.155734	0.061601	2.528113	0.01146774	0.238632913
TUT7	0.325736	0.128846	2.528108	0.0114679	0.238632913
CHTF18	-0.70264	0.278506	-2.522887	0.0116396	NA
RHBDD2	-0.394263	0.15628	-2.522792	0.01164273	0.240353431
IL13RA1	-0.85679	0.339659	-2.522503	0.0116523	0.240353431
SORL1	-0.493487	0.19565	-2.522294	0.01165921	0.240353431
JUNB	-0.29076	0.115477	-2.517901	0.01180566	0.242462605
GSDMD	-0.346503	0.137678	-2.516761	0.01184392	0.242462605
ENSG00000166685	-0.461943	0.183606	-2.515953	0.0118711	0.242462605
SNAP23	-0.21855	0.086907	-2.514759	0.01191138	0.242536696
MRTO4	-0.387307	0.154148	-2.512571	0.0119855	0.242536696
SPESP1	1.527076	0.607803	2.512455	0.01198945	NA
MREG	-0.863258	0.343685	-2.51177	0.01201273	NA
ENSG00000258757	0.584023	0.232524	2.511662	0.01201643	0.242536696
MED11	0.242545	0.096573	2.511531	0.01202088	0.242536696
FHL3	-0.786236	0.313554	-2.507498	0.01215892	NA
TAF9	-0.285624	0.113952	-2.506515	0.01219277	0.245259486
MINDY2	0.381266	0.152302	2.503346	0.01230251	0.246080967
CD69	-0.390544	0.156018	-2.503195	0.01230776	0.246080967
UGT8	0.738404	0.29499	2.503148	0.0123094	NA
PPARD	-0.493075	0.197322	-2.498832	0.01246033	0.248383381
BCAS4	0.36509	0.146208	2.49706	0.01252277	0.248636132
MRPS14	0.251776	0.100886	2.495656	0.01257244	0.248636132
RPSA	0.23382	0.093705	2.495291	0.01258538	0.248636132
OPTN	-0.753233	0.302442	-2.490501	0.01275632	0.250413166

RPS15A	0.172929	0.069436	2.490485	0.0127569	0.250413166
CD40	-0.206197	0.082823	-2.489605	0.0127885	0.250413166
SLC2A1	-0.313428	0.126122	-2.485116	0.01295093	0.252847765
EMC9	-0.486767	0.195902	-2.484743	0.0129645	NA
IFIT1	2.018428	0.81241	2.484492	0.01297363	NA
RAB30	0.212917	0.085742	2.483242	0.01301927	0.253409359
ENSG00000237773	-0.913858	0.368054	-2.482946	0.01303007	NA
FASTK	-0.245613	0.098948	-2.482237	0.01305604	0.253409359
FLYWCH1	0.54623	0.220291	2.479587	0.01315346	NA
DOCK8	0.340323	0.137279	2.479058	0.013173	0.254933974
USF1	-0.667133	0.269478	-2.475652	0.01329932	NA
CFAP73	-1.047877	0.42334	-2.475259	0.01331396	NA
GCNT2	0.684373	0.276925	2.471335	0.01346097	0.259539909
CETN3	0.439238	0.177838	2.46988	0.01351585	0.259539909
JAM3	0.718412	0.290901	2.469608	0.01352612	NA
RANGRF	-0.280088	0.113417	-2.46955	0.01352829	0.259539909
GGCX	-0.499102	0.202299	-2.467147	0.01361943	0.260535326
HSPA8	0.245918	0.099747	2.465403	0.01368593	0.261055248
ADHFE1	1.471291	0.597106	2.464038	0.01373816	NA
RND1	-0.884731	0.359115	-2.463639	0.01375344	NA
ATP6V1D	0.457626	0.185789	2.463147	0.01377234	0.261333908
POLR3K	0.2964	0.120372	2.462377	0.01380194	0.261333908
RPL24	0.160687	0.065276	2.461667	0.01382931	0.261333908
GCNT1	0.837723	0.34057	2.459765	0.0139028	NA
C2orf69	0.385022	0.156529	2.459749	0.01390342	0.261333908
ARPC3	-0.156184	0.063503	-2.459456	0.01391476	0.261333908
ACADVL	-0.212839	0.086559	-2.458889	0.01393676	0.261333908
RAB33A	1.575588	0.641604	2.455702	0.01406098	NA
TRMT13	0.290659	0.118365	2.455618	0.01406424	0.262981451
RNF181	-0.19372	0.078929	-2.454359	0.01411362	0.263163409
ABHD12	-0.601078	0.245126	-2.452114	0.01420197	NA
ZNF860	0.492524	0.200894	2.451654	0.01422012	NA
PURA	0.356434	0.145464	2.450323	0.01427282	0.264829124
TTC19	-0.26316	0.107409	-2.450073	0.01428274	0.264829124
ATP5F1B	-0.230273	0.094166	-2.445401	0.01446911	0.266398949
LINC00662	0.296198	0.121131	2.445274	0.01447422	0.266398949
SLC25A37	0.330138	0.135029	2.444936	0.0144878	0.266398949
ENSG00000271646	-0.577288	0.236195	-2.444113	0.01452089	NA
RPRML	-1.961666	0.806212	-2.43319	0.01496646	NA
VPS26B	-0.807113	0.332356	-2.428458	0.01516319	NA
DPY19L3	-0.754895	0.311465	-2.423687	0.01536385	NA
ENSG00000223478	-0.58899	0.243223	-2.421605	0.01545212	NA
ZNF260	0.473219	0.195753	2.417423	0.01563084	NA
SYNJ2BP	0.277652	0.114862	2.417265	0.01563763	0.2863145
SIDT1.AS1	-0.621963	0.257349	-2.416811	0.01565715	0.2863145
ENSG00000235501	-1.158859	0.479514	-2.416736	0.01566038	NA
LAPTM5	-0.20122	0.083305	-2.415463	0.0157152	0.286586553

BAX	-0.312829	0.129653	-2.41281	0.01583007	0.286993728
PIGS	0.441336	0.18297	2.412073	0.01586213	0.286993728
HIRA	-0.552858	0.229216	-2.411955	0.01586723	0.286993728
CDK7	0.554761	0.230136	2.410577	0.01592732	NA
ALAS1	0.518025	0.214938	2.410115	0.0159475	NA
ENSG00000204802	-1.014382	0.420999	-2.409464	0.01597597	NA
PURB	0.450359	0.187004	2.408288	0.01602752	0.287192474
ERH	-0.218321	0.090664	-2.408018	0.0160394	0.287192474
LPAR5	0.469961	0.195167	2.40799	0.01604061	0.287192474
CLMN	0.645887	0.268253	2.407747	0.01605128	0.287192474
CD5	-0.85502	0.355695	-2.403802	0.01622557	NA
PYGO2	0.371257	0.154463	2.403534	0.01623744	0.28951641
KMT2A	0.211491	0.088017	2.402838	0.0162684	0.28951641
RHOBTB3	1.759852	0.732812	2.401503	0.01632786	NA
HIST1H2BN	0.871351	0.362849	2.401413	0.0163319	NA
ARL11	0.626704	0.261087	2.400364	0.01637878	0.290701388
PIAS2	-0.45379	0.189358	-2.396465	0.01655406	0.292553132
YDJC	-0.219516	0.091614	-2.396085	0.01657125	0.292553132
IRF5	0.613587	0.256123	2.395673	0.01658988	NA
FANCL	0.589617	0.246167	2.395195	0.01661153	NA
MORF4L2	0.254457	0.106241	2.395107	0.01661551	0.292556413
NINJ1	-0.882172	0.368325	-2.39509	0.01661627	NA
DNAJB4	0.613456	0.256273	2.39376	0.01667664	NA
AKAP12	-1.938961	0.810069	-2.393575	0.01668505	NA
CCDC51	0.603294	0.252257	2.391581	0.01677598	NA
FBXL6	-1.259591	0.526687	-2.391537	0.01677797	NA
APBB2	-0.739353	0.309188	-2.391271	0.01679015	0.294849165
TRMT112	0.206043	0.086248	2.388959	0.01689621	0.295350411
ITGB7	0.524978	0.219775	2.388709	0.01690768	0.295350411
ZNF827	0.734557	0.307704	2.387221	0.01697629	NA
TMX2	0.268946	0.112667	2.387096	0.01698206	0.29587112
ENSG00000258017	-1.234596	0.517453	-2.385908	0.01703701	NA
SLC15A2	0.833893	0.349513	2.385873	0.01703866	NA
GPR137	-0.355168	0.148973	-2.384118	0.01712012	0.297433914
TIPARP	-0.414776	0.174064	-2.382894	0.01717712	0.297433914
TLR7	0.77367	0.324741	2.382419	0.0171993	NA
COA5	0.269944	0.113314	2.382272	0.01720618	0.297433914
CYB5RL	0.956372	0.401465	2.382203	0.01720942	NA
INKA1	0.419128	0.176217	2.378472	0.01738456	0.299100679
MCM7	-0.353132	0.148481	-2.378299	0.01739272	0.299100679
DDX5	-0.290524	0.122282	-2.37585	0.01750856	0.300314765
ARL4D	1.062334	0.447179	2.375636	0.01751874	NA
VCPIP1	0.390673	0.164581	2.373742	0.01760887	0.300501029
RPL5	0.167745	0.070681	2.373289	0.01763047	0.300501029
RPL37	0.129717	0.054669	2.372771	0.01765523	0.300501029
ECHDC2	1.158543	0.488589	2.371201	0.01773038	NA
INTS13	0.505084	0.213072	2.370486	0.01776472	NA

SWAP70	0.245541	0.103603	2.370011	0.01778756	0.300710814
EMC8	0.349106	0.147329	2.369565	0.01780901	0.300710814
ATP5IF1	-0.166884	0.07047	-2.368145	0.01787753	0.300710814
TNFRSF13B	1.080443	0.456265	2.368018	0.01788368	0.300710814
PPP1CA	-0.16852	0.071171	-2.367803	0.01789406	0.300710814
RPS6KB2.AS1	-0.910507	0.385323	-2.362971	0.01812907	NA
MAD2L2	-0.289822	0.122717	-2.361706	0.01819105	0.303966517
RENBP	0.596236	0.252622	2.360189	0.01826561	0.303966517
RNF19B	0.949342	0.40234	2.359554	0.01829693	NA
LMBRD1	-0.258254	0.109461	-2.359312	0.01830888	0.303966517
BCAP31	-0.255473	0.108287	-2.359223	0.01831324	0.303966517
PEX13	0.46454	0.19691	2.359152	0.01831675	0.303966517
FAXDC2	1.519125	0.644405	2.357407	0.01840309	NA
ENSG00000272630	1.104355	0.468514	2.357147	0.01841598	NA
POLD3	-0.528557	0.224274	-2.35675	0.01843568	NA
SLC35D1	0.741048	0.314585	2.355635	0.01849109	NA
PPOX	-0.412682	0.175212	-2.355335	0.01850599	0.306341118
SKI	0.576502	0.244936	2.353684	0.01858841	NA
ZNF667.AS1	0.491333	0.208761	2.353566	0.0185943	0.307037184
SAMHD1	-0.95498	0.406065	-2.351792	0.01868321	NA
BATF	1.120456	0.476749	2.350202	0.01876324	NA
ARHGEF3	-0.447753	0.190548	-2.34981	0.01878302	0.309383806
CCDC157	-0.892233	0.379859	-2.348852	0.01883141	NA
SNHG25	-0.336021	0.143075	-2.348564	0.01884595	0.30965201
NPHP3	0.442287	0.188426	2.347279	0.01891109	0.30995506
NDUFB3	0.194162	0.082779	2.345554	0.01899882	0.310625941
CCDC144A	1.423199	0.607817	2.341493	0.01920676	NA
CRIP3	0.698797	0.298453	2.341401	0.01921151	NA
HELZ	0.358712	0.153213	2.341263	0.01921864	0.313447925
KLC4	0.482429	0.206175	2.339902	0.01928879	NA
BIN1	0.260565	0.111393	2.339141	0.01932814	0.313701161
MHENCR	-0.208641	0.089196	-2.339131	0.01932868	0.313701161
IGHV3.30	1.295754	0.554235	2.337916	0.01939159	NA
HNRNPDL	-0.177524	0.076004	-2.335708	0.01950646	0.314370415
TRADD	-0.30522	0.130741	-2.334538	0.01956758	0.314370415
PFKFB3	-0.387987	0.166214	-2.334257	0.01958229	0.314370415
TMX4	-0.277785	0.11905	-2.333349	0.01962981	0.314370415
RPL3	0.133846	0.057402	2.331723	0.01971529	0.314370415
LPIN1	0.56204	0.241186	2.330321	0.01978919	0.314370415
ENSG00000260409	-2.001854	0.859048	-2.330318	0.01978935	NA
LDHB	0.186209	0.079962	2.328703	0.01987479	0.314370415
LSM6	0.277774	0.119286	2.328639	0.01987819	0.314370415
ZC3H12D	-0.625287	0.268559	-2.328301	0.0198961	0.314370415
MRPL50	0.274045	0.117712	2.328101	0.01990674	0.314370415
MYC	0.288218	0.123892	2.326363	0.01999922	0.314370415
FANCF	0.353307	0.151912	2.325733	0.02003281	0.314370415
TOMM7	0.20183	0.086801	2.3252	0.0200613	0.314370415

RAF1	0.297629	0.12801	2.325035	0.02007008	0.314370415
SFR1	-0.329746	0.141836	-2.324844	0.0200803	0.314370415
SGPP1	1.040489	0.447668	2.324242	0.02011252	NA
LACTB2	0.409416	0.176294	2.322348	0.02021419	0.315721794
OAS2	0.398018	0.171525	2.320476	0.02031516	0.316554081
EIF4EBP1	-0.298688	0.128795	-2.31909	0.02039016	0.316978607
DOK3	0.829356	0.357718	2.318461	0.02042426	NA
WRB	0.375994	0.162209	2.317956	0.02045173	0.317192954
TSC22D1	-0.599387	0.25865	-2.317367	0.02048377	NA
RPL26	0.146212	0.063172	2.314514	0.02063957	0.318640168
DEDD2	0.284471	0.122909	2.314487	0.02064105	0.318640168
KBTBD8	0.870275	0.376142	2.313691	0.02068467	NA
WASH9P	-0.419451	0.181349	-2.312948	0.02072548	0.319201251
ENSG00000272106	-0.459681	0.198849	-2.311708	0.02079378	0.31951177
MPEG1	-0.490849	0.212573	-2.309084	0.02093894	0.320999336
GATD3B	-1.275884	0.552572	-2.308992	0.02094403	NA
SPN	-1.036413	0.449064	-2.307939	0.02100253	NA
CLCF1	0.40249	0.174416	2.307638	0.02101927	0.321488235
NR2F6	1.099224	0.476779	2.30552	0.02113745	NA
POLDIP2	-0.26453	0.114791	-2.304451	0.02119734	0.323466525
IFT22	-0.361579	0.157068	-2.302046	0.02133258	0.324409994
TOR3A	-0.28128	0.12221	-2.301615	0.02135691	0.324409994
TPM4	0.237969	0.103544	2.29824	0.02154812	0.326567185
FAM13A.AS1	-0.791225	0.344429	-2.29721	0.02160682	NA
H6PD	-0.430523	0.187481	-2.296354	0.02165562	0.326762007
CFLAR	0.284277	0.123799	2.296288	0.02165943	0.326762007
LY6E.DT	1.308133	0.569716	2.296114	0.02166938	NA
IGHV3.15	-1.437448	0.626262	-2.29528	0.02171707	NA
AASDH	0.406424	0.17707	2.295268	0.02171778	0.326899294
C22orf34	0.808	0.352154	2.294452	0.02176457	NA
SEC24A	0.845711	0.368713	2.293683	0.02180869	NA
KRT10	-0.263607	0.114999	-2.292262	0.02189055	0.328754427
MAT2A	0.246138	0.10745	2.29072	0.02197959	0.329346518
ENSG00000254397	1.082559	0.47268	2.290258	0.02200634	NA
HMGB3	-1.106553	0.483661	-2.287871	0.02214503	NA
INPP5K	-0.472332	0.206667	-2.285476	0.02228495	0.332620607
PRDM8	-0.719139	0.314712	-2.285068	0.02230884	NA
MED10	-0.157061	0.068743	-2.284752	0.02232737	0.332620607
LTBP4	0.518644	0.227038	2.284393	0.02234842	0.332620607
ASAP1	0.567859	0.248948	2.28104	0.02254606	NA
FXYD5	0.202793	0.089043	2.277468	0.02275828	0.337963014
STARD9	0.64671	0.283996	2.277182	0.02277534	NA
MAP3K8	-0.484887	0.213069	-2.275728	0.02286229	0.338043062
P2RY10	0.200851	0.08826	2.275674	0.02286552	0.338043062
SRA1	-0.307967	0.135384	-2.274759	0.02292036	0.338100837
RPL13A	0.118537	0.052143	2.273299	0.02300816	0.338643379
SERPINB6	0.426027	0.187837	2.268069	0.02332502	0.342547585

TRIM32	0.901458	0.397723	2.266547	0.02341794	NA
ENSA	-0.163036	0.071956	-2.265786	0.0234645	0.343238718
TTC21A	0.312417	0.137895	2.265606	0.0234755	0.343238718
EXD3	-0.425364	0.187854	-2.264325	0.02355412	0.34363135
UTP14C	1.01663	0.449	2.264211	0.02356111	NA
ENSG00000274922	-1.438869	0.635843	-2.26293	0.02363998	NA
CERS6	0.956849	0.422891	2.262638	0.02365801	NA
FNTA	0.267567	0.118271	2.262323	0.02367745	0.344431669
NSMCE4A	-0.277282	0.122596	-2.261752	0.02371276	0.344431669
TCL1A	-0.37924	0.167845	-2.25947	0.02385414	0.344648239
NSMAF	0.601224	0.266148	2.258985	0.02388431	0.344648239
ZC3H7A	-0.353375	0.156445	-2.258785	0.02389677	0.344648239
COX17	0.234878	0.104013	2.258165	0.02393535	0.344648239
ENSG00000272758	0.590359	0.261488	2.257694	0.02396472	NA
ZNF747	-0.521229	0.230993	-2.256464	0.02404156	0.345428318
ENSG00000275799	0.417137	0.185033	2.254389	0.02417171	0.345485311
TMEM9	-0.414781	0.18409	-2.253143	0.02425015	0.345485311
BNIP3	0.369202	0.16392	2.252329	0.02430148	0.345485311
PDHA1	-0.370658	0.164622	-2.251572	0.02434932	0.345485311
TPCN1	-0.337874	0.150061	-2.251572	0.0243493	0.345485311
ALDH9A1	0.386823	0.171812	2.251438	0.02435781	0.345485311
KLF8	-0.327316	0.14561	-2.247896	0.02458281	0.346749515
NOTCH2	0.319627	0.142204	2.247667	0.0245974	0.346749515
CDKN1B	0.2471	0.109941	2.24757	0.02460365	0.346749515
GRAMD1C	-0.600071	0.267232	-2.245505	0.02473575	0.347872692
FXYD2	-0.886314	0.394896	-2.244423	0.02480518	0.348111562
BANK1	0.225442	0.100497	2.243268	0.02487955	0.348313878
AFG3L2	-0.391107	0.174534	-2.240872	0.02503437	0.348313878
PTRH1	-0.472092	0.210699	-2.2406	0.02505199	0.348313878
DDX21	0.199816	0.089188	2.240399	0.02506502	0.348313878
IER2	-0.210297	0.093877	-2.240138	0.02508196	0.348313878
RPS8	0.194387	0.086836	2.238565	0.02518421	0.349003781
RAB11FIP1	0.317289	0.141832	2.237076	0.02528138	0.349539074
FRG1	-0.220349	0.09853	-2.236361	0.02532816	0.349539074
RFX7	0.565215	0.25284	2.23546	0.02538714	NA
HSPBAP1	-0.888631	0.397576	-2.235122	0.02540933	0.349548696
TDP1	0.496407	0.222105	2.235011	0.02541663	NA
EIF4B	0.148473	0.066439	2.234744	0.02543417	0.349548696
FAM177B	0.569952	0.255266	2.232779	0.02556352	0.350600448
THRB	1.757718	0.787868	2.23098	0.02568243	NA
CBL	0.417025	0.186934	2.230868	0.02568988	0.350995918
PRNP	-0.375732	0.168433	-2.230744	0.0256981	0.350995918
MANF	0.296335	0.132913	2.229546	0.0257776	0.351358738
OAZ2	-0.178658	0.080232	-2.226782	0.02596188	0.352901902
MIR762HG	-0.727742	0.327033	-2.225283	0.02606222	NA
ZNF844	-0.477927	0.214796	-2.225025	0.02607953	0.352901902
PCIF1	-0.325859	0.146461	-2.224892	0.02608851	0.352901902

POLR3C	-0.455669	0.204826	-2.224669	0.02610347	0.352901902
FALEC	-0.478433	0.215199	-2.223209	0.02620173	0.35329158
ENSG00000272990	-0.890217	0.400567	-2.222391	0.02625689	NA
C9orf72	-0.365745	0.164596	-2.222081	0.02627781	0.35329158
VTA1	0.401166	0.180609	2.221187	0.02633829	0.35329158
KMT2E.AS1	-0.420725	0.189423	-2.221085	0.02634518	0.35329158
TMSB10	-0.173529	0.078164	-2.220053	0.02641518	0.35351607
NFU1	-0.272249	0.12268	-2.219186	0.0264741	0.353591689
DUSP12	-0.31519	0.142129	-2.217628	0.02658021	0.354177454
APAF1	0.558833	0.252099	2.216722	0.02664207	NA
WASF1	-0.580722	0.261996	-2.216527	0.02665545	NA
SNX8	-0.408588	0.18434	-2.216492	0.02665785	0.354177454
HES6	-0.680461	0.307029	-2.216273	0.02667279	NA
TRMT1L	0.414968	0.187243	2.216197	0.02667803	0.354177454
LONP2	-0.253145	0.114265	-2.215418	0.02673138	0.354177454
MOAP1	-0.30311	0.136868	-2.214623	0.02678596	0.354193649
ZNF257	0.615014	0.277874	2.213283	0.02687811	NA
CRY1	-0.584073	0.263931	-2.212977	0.02689924	NA
CRK	0.270578	0.122276	2.212836	0.02690894	0.354307089
TCP1	0.239004	0.108018	2.212621	0.0269238	0.354307089
HMGN2	-0.146321	0.066148	-2.212027	0.02696482	0.354307089
DYRK2	0.395976	0.179204	2.209634	0.02713056	0.354307089
OVCH1.AS1	-1.670878	0.756456	-2.208823	0.02718693	NA
EPB42	-0.435922	0.197428	-2.208004	0.02724403	0.354307089
HGSNAT	-0.44682	0.202367	-2.207969	0.02724642	0.354307089
TMEM208	-0.244037	0.110608	-2.206319	0.02736169	0.354307089
P4HB	-0.206133	0.093436	-2.206128	0.02737506	0.354307089
AKAP9	0.14973	0.067879	2.205849	0.02739461	0.354307089
ICAM3	0.22577	0.102417	2.204421	0.02749475	0.354307089
LEAP2	0.363248	0.164799	2.20419	0.02751097	0.354307089
LASP1	0.246169	0.111714	2.203571	0.02755455	0.354307089
MYO1C	-0.430923	0.195579	-2.20332	0.02757224	0.354307089
TSC22D2	0.348475	0.15816	2.203304	0.02757333	0.354307089
CD72	0.30898	0.140346	2.201569	0.02769575	0.354307089
EYA3	0.783415	0.355865	2.201435	0.02770527	0.354307089
ATP1A1	-0.288533	0.131084	-2.201141	0.02772606	0.354307089
TSEN15	-0.488987	0.22226	-2.200066	0.02780222	0.354307089
C12orf49	-0.577584	0.26255	-2.199901	0.02781392	0.354307089
SUMO1	-0.179856	0.081793	-2.198911	0.02788428	0.354307089
GTF2IRD2	-0.438613	0.199508	-2.198473	0.02791543	0.354307089
NCBP2	-0.213097	0.096996	-2.196968	0.02802273	0.354665457
PARL	-0.275675	0.125559	-2.195587	0.02812153	0.354665457
KLHL2	-0.574913	0.261888	-2.195265	0.02814462	0.354665457
CD82	-0.282619	0.128751	-2.195087	0.02815738	0.354665457
MAP11	-0.547699	0.249589	-2.194402	0.02820654	NA
SLC16A1.AS1	0.329744	0.150288	2.194085	0.02822928	0.354897646
DHFR2	0.547235	0.249593	2.192512	0.02834253	NA

RBM8A	-0.135569	0.061844	-2.192109	0.0283716	0.356012668
INO80	-0.322349	0.147206	-2.189788	0.02853964	0.356529296
EPHB6	-0.417276	0.190587	-2.189419	0.0285664	0.356529296
ATG16L2	-0.322414	0.147267	-2.189316	0.02857391	0.356529296
MYL6	-0.138589	0.063404	-2.185796	0.02883056	0.358427579
DENR	-0.185675	0.084948	-2.185748	0.02883404	0.358427579
BBX	0.182217	0.083399	2.184892	0.0288968	0.358536352
ZNF506	0.343448	0.157301	2.183377	0.02900803	0.359244953
PPP1R15A	-0.524557	0.240361	-2.182376	0.02908178	0.359487572
WAC	0.178485	0.081841	2.180872	0.02919286	0.359590228
TMED2	-0.207501	0.095149	-2.180797	0.02919842	0.359590228
ENSG00000275418	-1.465782	0.672512	-2.179563	0.0292899	NA
MMP28	1.609095	0.738813	2.177946	0.02941006	NA
RPL36	0.107235	0.049272	2.176397	0.02952556	0.362140364
RIMS3	-1.560775	0.717173	-2.176287	0.02953379	NA
ENSG00000273748	-0.903295	0.415119	-2.175992	0.02955584	0.362140364
POLM	-0.266141	0.122318	-2.175814	0.02956916	0.362140364
PF4	2.393039	1.100456	2.174588	0.02966104	NA
TTC9C	0.250948	0.115519	2.172347	0.02982952	0.36465622
C9orf66	-1.382959	0.63668	-2.172143	0.02984491	NA
PPP1CB	-0.222237	0.10241	-2.170058	0.03000242	0.365672932
ODF3B	-0.550128	0.25354	-2.169789	0.03002286	0.365672932
RPH3AL	-1.042184	0.480456	-2.169155	0.03007096	NA
ENSG00000274015	-1.705455	0.786423	-2.168621	0.03011146	NA
RBM26.AS1	-0.693014	0.319775	-2.167191	0.03022032	NA
GOLGA6L9	-1.112481	0.513553	-2.166244	0.03029254	NA
PPWD1	0.292306	0.135048	2.16446	0.03042907	0.368088243
YWHAG	0.318446	0.147129	2.164395	0.03043401	0.368088243
DAZAP1	-0.363682	0.168032	-2.164363	0.03043651	0.368088243
MIIP	-0.325787	0.150529	-2.164279	0.03044297	0.368088243
GSTP1	-0.19029	0.087982	-2.162824	0.03055474	0.368767939
DUSP2	-1.898357	0.878374	-2.161217	0.03067855	NA
CNOT6L	-0.391928	0.181368	-2.160957	0.03069869	0.369832861
MPP5	0.60044	0.278043	2.159522	0.03080969	NA
ST20.AS1	-0.884263	0.409559	-2.159059	0.03084556	NA
THRAP3	-0.206011	0.095431	-2.158742	0.03087016	0.370817737
APOBEC3H	-1.825928	0.845867	-2.158648	0.0308775	NA
C15orf62	-0.677962	0.314095	-2.15846	0.03089207	NA
TRIM23	0.519078	0.240486	2.158459	0.03089217	0.370817737
LRP5L	-0.594752	0.275611	-2.157936	0.03093279	NA
ENSG00000261353	-1.040004	0.482125	-2.157124	0.03099598	NA
TMEM14B	-0.23109	0.107154	-2.156625	0.03103489	0.37185853
CAPN12	0.370543	0.171891	2.155684	0.03110831	0.372066617
FAM117B	-0.542172	0.251686	-2.154164	0.03122728	0.372216546
TRIM4	0.264445	0.122792	2.153602	0.03127136	0.372216546
PET100	-0.182837	0.084928	-2.152851	0.03133035	0.372216546
ELF1	0.213387	0.099127	2.152663	0.03134514	0.372216546

RPS23	0.131221	0.061006	2.150935	0.03148131	0.373165934
CR1	0.962701	0.44762	2.150709	0.03149917	NA
SPTLC1	-0.64257	0.298854	-2.150109	0.03154656	NA
CALHM6	0.263532	0.122609	2.14937	0.03160509	0.373965361
TRIM21	0.585018	0.272488	2.146952	0.0317971	NA
GFM2	0.457375	0.213192	2.145363	0.03192387	NA
UNC50	0.313216	0.146167	2.14286	0.03212432	0.377867817
ENSG00000261840	-0.627235	0.292831	-2.141969	0.03219595	NA
CUL4B	0.451479	0.210845	2.141288	0.03225086	0.377867817
DCXR	-0.312946	0.146155	-2.141194	0.03225836	0.377867817
PEX16	-0.227036	0.106038	-2.141082	0.03226745	0.377867817
BCL7A	-0.476373	0.222531	-2.140705	0.03229786	0.377867817
LINC00324	0.448688	0.209729	2.13937	0.03240571	0.377867817
ZNF667	0.527666	0.246701	2.138887	0.03244485	NA
MFAP1	0.410441	0.191917	2.138633	0.03246537	0.377867817
TBCC	-0.214757	0.100425	-2.138475	0.03247818	0.377867817
SHMT2	0.205667	0.096179	2.138383	0.03248565	0.377867817
COASY	-0.300235	0.140418	-2.138155	0.03250415	0.377867817
ASCC1	-0.429156	0.200875	-2.136439	0.03264361	0.378825674
PSMC5	-0.174202	0.081671	-2.132966	0.03292751	0.381453425
HADHA	0.229774	0.107872	2.130056	0.03316697	0.38335407
C1GALT1	0.307636	0.144459	2.129571	0.03320708	0.38335407
ABHD17A	0.380972	0.179091	2.127249	0.03339937	0.384904499
RANBP1	-0.230047	0.108179	-2.126541	0.03345823	0.384914624
IMPACT	-0.489426	0.230288	-2.125273	0.03356386	NA
ENSG00000282988	0.703786	0.331256	2.124597	0.03362028	0.385636007
TMED8	0.322393	0.151758	2.124395	0.03363713	0.385636007
DNAJB1	0.403781	0.190308	2.121722	0.03386105	0.387185545
EEA1	0.385714	0.181821	2.121391	0.03388894	0.387185545
ENSG00000125726	-1.396224	0.658449	-2.120473	0.0339662	NA
CD24	-0.171514	0.080971	-2.118223	0.03415621	0.388701076
PHF21A	-0.302211	0.142688	-2.117991	0.03417579	0.388701076
FADS3	-0.223492	0.105554	-2.11733	0.03423188	0.388701076
NDUFA13	-0.186902	0.088284	-2.117047	0.03425582	0.388701076
ZNF48	0.554701	0.262053	2.116753	0.03428081	NA
ENSG00000250790	1.248255	0.589934	2.115925	0.03435122	NA
NR6A1	-1.209176	0.571484	-2.115852	0.03435739	NA
PTPRS	1.333231	0.630141	2.115766	0.03436469	NA
MDK	-0.675252	0.319182	-2.115568	0.03438154	NA
PDCD7	0.439589	0.207838	2.115055	0.03442527	0.389957217
TNFRSF13C	-0.124297	0.058791	-2.114223	0.03449624	0.390095491
SOX4	-0.510952	0.241857	-2.112618	0.03463351	0.390494268
RSPRY1	0.377127	0.178527	2.112435	0.03464916	0.390494268
ANKDD1A	-1.444866	0.684181	-2.111816	0.0347022	NA
PPCS	-0.195168	0.092426	-2.111622	0.03471891	0.390617131
RAB1B	-0.295767	0.140165	-2.110124	0.03484767	0.391402417
RMDN3	0.508325	0.240915	2.109978	0.03486023	NA

ERV3.1	-0.246754	0.117055	-2.108018	0.03502942	0.392239895
SSU72	-0.207258	0.098325	-2.107891	0.03504041	0.392239895
MAP4K3.DT	-0.523107	0.248215	-2.107473	0.03507664	NA
LINC02201	-1.567934	0.744408	-2.106283	0.03517978	NA
PUS3	0.413404	0.19646	2.10427	0.03535489	0.395093883
RPL18	0.137837	0.065567	2.10223	0.0355331	0.396418041
ZNF646	0.687893	0.327434	2.100856	0.03565362	NA
GCFC2	-0.449286	0.213863	-2.100813	0.03565742	NA
CEP97	0.524226	0.249695	2.099467	0.03577579	NA
FAM41C	-0.57364	0.27333	-2.098711	0.0358424	NA
PIN4	-0.187102	0.089187	-2.097865	0.03591704	0.400029037
ENSG00000274605	0.742668	0.35419	2.096809	0.03601053	NA
FAM200A	0.59494	0.283872	2.095804	0.03609956	NA
SEC11C	-0.258311	0.123267	-2.095547	0.03612238	0.401642148
CLPX	-0.313276	0.149636	-2.093591	0.03629641	0.402902325
ENSG00000261505	-0.636747	0.304543	-2.090826	0.03654365	NA
ATP2A3	-0.287922	0.137768	-2.089898	0.03662697	0.405892841
IFI44L	1.039675	0.497806	2.088515	0.0367514	0.406490785
ACP1	-0.151095	0.072377	-2.087609	0.03683315	0.406490785
ELF2	-0.191329	0.091665	-2.08726	0.03686464	0.406490785
RXRA	-0.876269	0.41993	-2.086701	0.03691518	NA
ENSG00000272335	0.971244	0.465553	2.086216	0.03695908	NA
TUBD1	0.34803	0.166864	2.085715	0.03700442	0.406929587
ERICH1	0.190517	0.09136	2.085338	0.03703865	0.406929587
MRPL14	-0.245325	0.117674	-2.08479	0.03708834	0.406929587
FKBP3	-0.185465	0.089083	-2.081951	0.03734697	0.409091029
AKAP13	0.181504	0.087252	2.080234	0.03750403	0.409831096
IKZF2	-0.825451	0.396863	-2.079941	0.03753092	NA
ZNF138	0.434833	0.209068	2.079864	0.03753801	0.409831096
EVA1B	-0.783345	0.376716	-2.079407	0.03757992	NA
TM4SF19.AS1	-1.282044	0.61686	-2.078337	0.03767834	NA
ATF4	-0.169699	0.081666	-2.077967	0.0377124	0.410213859
ZC3H6	0.329175	0.158424	2.077807	0.03772713	0.410213859
NAA10	-0.215631	0.103795	-2.077467	0.03775846	0.410213859
FUT2	1.548763	0.745556	2.077325	0.03777154	NA
PLA2G6	-0.48034	0.231276	-2.076908	0.03781004	NA
ENSG00000270562	0.578285	0.27859	2.075761	0.03791609	NA
FAU	0.113768	0.054827	2.075061	0.03798089	0.411956075
TMEM99	0.48254	0.232634	2.07425	0.0380561	NA
POLB	0.51607	0.248896	2.07344	0.03813132	NA
ENSG00000224790	-1.273637	0.614278	-2.073389	0.03813611	NA
ENSG00000237357	1.113593	0.537102	2.073336	0.03814104	NA
SLC15A3	-0.651355	0.314178	-2.0732	0.03815365	NA
WDR46	-0.279631	0.134883	-2.073139	0.03815932	0.412997172
ENSG00000257275	-0.335579	0.161905	-2.072688	0.03820131	0.412997172
MAP1LC3A	-0.693302	0.33456	-2.072276	0.03823973	NA
TCOF1	-0.2803	0.135312	-2.071507	0.03831143	0.413514221

IGLV3.21	-1.187244	0.573458	-2.070325	0.03842197	NA
CHRAC1	-0.236516	0.114261	-2.069966	0.03845553	0.414395841
ENSG00000227468	-1.508145	0.729175	-2.06829	0.03861273	NA
CACYBP	0.161629	0.078162	2.067859	0.03865326	0.415851437
TOMM40L	-0.884936	0.427985	-2.06768	0.03867015	NA
SYNC	-0.260785	0.126228	-2.065988	0.03882965	0.415984225
ISOC2	-0.398447	0.192873	-2.065851	0.03884252	0.415984225
RARA.AS1	-0.624547	0.302336	-2.065734	0.0388536	0.415984225
NACC1	0.550731	0.266721	2.064824	0.03893966	NA
XRRA1	-0.631194	0.305709	-2.064691	0.03895225	0.416368826
CHMP5	0.209471	0.101521	2.063332	0.03908109	0.417074363
DCUN1D5	0.43368	0.21033	2.061906	0.03921668	0.417849671
FUT4	-1.384376	0.671783	-2.060749	0.03932696	NA
NEMP1	0.676984	0.328566	2.060424	0.03935802	NA
THOC3	-0.422708	0.20523	-2.059684	0.03942881	0.419436546
CCZ1B	-0.63728	0.30955	-2.058727	0.03952036	0.41973784
SPEF2	-1.410971	0.685649	-2.057862	0.03960341	NA
FAM86C1	-0.843412	0.410289	-2.055654	0.03981585	NA
CHCHD5	-0.254126	0.123623	-2.055646	0.03981666	0.42220925
LINC01970	-0.890605	0.433269	-2.055548	0.03982612	NA
IL10RB	0.377243	0.183593	2.054779	0.03990032	0.422209674
TMEM170A	0.240952	0.11731	2.053979	0.03997777	0.422209674
ENSG00000262420	-1.235971	0.601753	-2.053951	0.03998048	NA
PSMB10	-0.21918	0.106726	-2.053671	0.04000752	0.422209674
DOK1	0.328708	0.160277	2.050876	0.04027902	0.423919206
GMFG	-0.1261	0.061491	-2.050689	0.04029723	0.423919206
NBDY	-0.179734	0.087683	-2.049808	0.04038313	0.424006027
TTLL12	-0.643269	0.313854	-2.049582	0.04040525	NA
TELO2	-0.424981	0.207379	-2.049295	0.04043324	0.424006027
NSUN7	-0.678539	0.331114	-2.04926	0.04043674	NA
RAB3A	1.062736	0.518812	2.048403	0.04052056	NA
STARD10	-0.378432	0.184751	-2.048338	0.04052692	0.424318103
CD99L2	-0.631275	0.308269	-2.047806	0.040579	NA
GTPBP6	-0.574254	0.280515	-2.047143	0.04064403	0.424590754
TAF1C	-0.388739	0.189993	-2.046069	0.04074957	0.424590754
TAPBP	-0.233335	0.114063	-2.045672	0.04078863	0.424590754
TMEM179B	-0.223179	0.109109	-2.045467	0.04080881	0.424590754
SNAI3	0.716255	0.350209	2.045221	0.04083307	NA
UBAP2	-0.421248	0.206011	-2.04479	0.04087556	0.424619668
ENSG00000260572	-1.579772	0.772673	-2.044554	0.04089881	NA
COMMD1	-0.3176	0.155493	-2.04253	0.04109903	0.425382421
RYBP	0.405155	0.198387	2.04225	0.04112672	0.425382421
EFHB	1.573414	0.770622	2.041744	0.04117695	NA
MSRB1	-0.307995	0.15091	-2.040919	0.04125888	0.425382421
MAPRE2	0.26686	0.13077	2.040684	0.04128227	0.425382421
KIAA1191	0.327508	0.160554	2.03986	0.04136431	0.425382421
HSD17B7	0.309794	0.151902	2.039437	0.04140644	0.425382421

ZNF397	0.191922	0.09415	2.038465	0.04150343	0.425382421
CD2BP2	-0.220013	0.107949	-2.038128	0.04153714	0.425382421
NFKB2	-0.31114	0.152715	-2.03739	0.04161102	0.425382421
FAM161B	-0.953978	0.46825	-2.037327	0.04161725	NA
ING1	0.370143	0.181686	2.037268	0.04162314	0.425382421
NOC2L	0.21945	0.107766	2.036348	0.04171539	0.425382421
FAM107B	-0.277558	0.136304	-2.036323	0.04171798	0.425382421
LINC02413	1.321044	0.648742	2.036316	0.04171861	NA
LINC02550	1.360633	0.668589	2.035082	0.04184266	NA
CD8B	-2.908313	1.429836	-2.034018	0.04194976	NA
PSPC1	-0.302681	0.148815	-2.033944	0.04195728	0.426975521
MYO5C	-1.382105	0.679624	-2.033632	0.04198868	NA
QDPR	0.689701	0.339281	2.032831	0.04206957	NA
BOLA2.SMG1P6	-0.532937	0.262205	-2.03252	0.04210104	NA
CIAO2B	0.171633	0.084453	2.032279	0.04212539	0.426975521
ARID5A	-0.376573	0.185306	-2.032168	0.04213664	0.426975521
PNRC2	0.151481	0.074556	2.031758	0.04217817	0.426975521
PIGT	-0.279894	0.137771	-2.031584	0.04219583	0.426975521
C15orf40	-0.230515	0.113552	-2.030033	0.04235317	0.427556799
CPM	-0.721817	0.355594	-2.029889	0.04236784	NA
GSTO1	0.237936	0.117225	2.029749	0.0423821	0.427556799
IGLV4.69	-1.244647	0.613425	-2.029012	0.04245712	NA
SERPINI1	0.582509	0.287294	2.027575	0.04260367	NA
NDUFV2.AS1	-0.386991	0.190865	-2.027565	0.04260462	0.429149379
PRRT3	0.588855	0.290536	2.026791	0.04268381	NA
GLUL	-0.489441	0.241526	-2.026451	0.04271856	0.429645136
AMIGO2	0.950752	0.469377	2.025563	0.0428096	NA
HSD17B11	0.135569	0.066932	2.025493	0.04281677	0.429819387
DNAJC7	-0.196225	0.0969	-2.025019	0.04286539	0.429819387
WNT10A	-0.650252	0.321152	-2.02475	0.04289309	NA
NDUFA4	-0.109192	0.053956	-2.023731	0.04299784	0.430029262
XG	1.851903	0.91519	2.023518	0.04301981	NA
ENSG00000247765	-1.459896	0.721593	-2.023158	0.04305685	NA
GEMIN6	0.257083	0.127077	2.02305	0.043068	0.430029262
CLASRP	-0.251755	0.124451	-2.022927	0.04308067	0.430029262
CNR1	0.716045	0.354001	2.022719	0.04310213	NA
CLOCK	-0.342908	0.169688	-2.020817	0.04329867	0.431556447
C12orf76	0.247853	0.122762	2.018969	0.04349048	0.432247825
TP53INP1	0.39232	0.194324	2.018894	0.04349828	0.432247825
APH1A	-0.178357	0.088399	-2.017645	0.0436282	0.432279608
ATF7	-0.396479	0.196509	-2.017612	0.04363172	0.432279608
EHD3	-0.614361	0.304513	-2.017522	0.04364108	NA
TOMM5	-0.240312	0.119152	-2.016857	0.04371049	0.432414614
CXorf65	-0.690161	0.342415	-2.015571	0.04384489	NA
BCL6	-0.359361	0.178358	-2.014824	0.04392308	0.432984833
ERGIC2	0.208406	0.103442	2.0147	0.04393612	0.432984833
N6AMT1	0.557884	0.276923	2.014582	0.04394851	NA

HIBCH	0.243815	0.121034	2.014436	0.04396381	0.432984833
CYHR1	0.238549	0.118514	2.012838	0.04413171	0.433994483
ANKMY1	-0.456868	0.227045	-2.012236	0.04419506	NA
ZNF786	0.549369	0.273143	2.011287	0.04429511	NA
ENSG00000197302	0.245058	0.121897	2.010373	0.04439175	0.434957504
POLR2I	-0.225578	0.112223	-2.010085	0.04442217	0.434957504
PRDX6	-0.144163	0.071741	-2.009504	0.04448374	0.434957504
KDM4B	0.336336	0.167379	2.009428	0.04449174	0.434957504
ARMC10	-0.705312	0.351496	-2.0066	0.04479223	NA
TSEN54	-0.530212	0.26425	-2.00648	0.04480511	NA
LTA4H	0.190418	0.094932	2.005841	0.04487319	0.438041487
HCG11	-0.43486	0.216839	-2.005445	0.04491546	NA
TAF6L	-0.659766	0.329128	-2.00459	0.04500692	NA
PRR4	0.364545	0.181925	2.003819	0.04508944	0.439506197
MAGED1	-0.582391	0.290831	-2.002509	0.04523004	NA
G6PC3	-0.490079	0.244808	-2.001894	0.04529609	NA
ATP11A	0.542068	0.270896	2.00102	0.04539023	NA
NUP210	0.204825	0.102401	2.000218	0.0454767	0.442331376
MRPS36	0.180506	0.090258	1.999886	0.04551255	0.442331376
SMIM20	0.333107	0.166624	1.999157	0.04559133	0.442449213
POLE2	0.87002	0.435503	1.997736	0.04574526	NA
ERF	0.475606	0.23808	1.997673	0.04575215	NA
ZNF628	-0.931539	0.466626	-1.996326	0.04589845	NA
PCID2	0.25292	0.126739	1.9956	0.04597748	0.443733612
RALY	-0.154601	0.077473	-1.995535	0.04598453	0.443733612
NELFE	-0.173316	0.086864	-1.995246	0.04601601	0.443733612
LAMC1	0.806688	0.404358	1.994983	0.04604475	NA
CTNNBIP1	-0.688917	0.345339	-1.994899	0.04605384	NA
IL6	-0.780578	0.391386	-1.994396	0.04610885	NA
ENSG00000267040	0.238568	0.119622	1.994348	0.04611402	0.443733612
SLA	0.380222	0.190655	1.994287	0.04612073	0.443733612
ZPR1	0.2415	0.121132	1.993694	0.04618546	0.443733612
FLCN	0.328253	0.16465	1.993638	0.04619161	0.443733612
DPH3	-0.305803	0.153443	-1.992942	0.0462678	0.443823168
CEMIP2	0.505914	0.254027	1.991575	0.0464177	0.44455636
CCL5	-1.14281	0.573874	-1.991396	0.04643732	NA
GPR160	0.57876	0.290684	1.991025	0.04647817	0.44455636
GTF3C6	-0.205495	0.103252	-1.990222	0.04656646	0.444759915
SENP6	0.208346	0.104726	1.98945	0.04665154	0.444932378
CCNG2	0.376171	0.189158	1.988656	0.0467392	0.445128842
TFDP2	-0.277722	0.139812	-1.986391	0.04698997	0.44687599
HIST1H3H	1.286066	0.647511	1.986167	0.04701474	NA
GTF2H2C	-0.380469	0.191925	-1.98238	0.04743675	0.449014872
RBAK.RBAKDN	0.345426	0.174249	1.982373	0.0474375	0.449014872
USP53	1.123284	0.566678	1.982225	0.04745401	0.449014872
CPQ	-0.641696	0.323737	-1.982155	0.04746195	NA
MKNK1	0.283301	0.142976	1.981467	0.0475389	0.449014872

PSPH	-0.829097	0.41843	-1.981446	0.04754123	NA
HMGXB4	-0.241921	0.1221	-1.981341	0.0475531	0.449014872
UBA6	0.27138	0.137014	1.980675	0.04762771	0.44908061
DAPK2	-0.77251	0.390102	-1.980275	0.04767258	NA
DLGAP3	-0.921743	0.465591	-1.979728	0.04773415	NA
TTC9	0.412173	0.20825	1.979219	0.04779137	NA
FXYD1	0.530461	0.2681	1.978591	0.0478621	0.450315583
PPM1N	-0.438653	0.221732	-1.978304	0.04789437	0.450315583
PTPRC	0.134667	0.068104	1.977365	0.04800039	0.450674131
NPEPL1	-0.523817	0.264926	-1.977217	0.04801708	NA
LINC00663	0.696683	0.352402	1.976953	0.04804695	NA
C1GALT1C1	0.423372	0.214188	1.976639	0.04808241	NA
IRAK3	1.184596	0.599576	1.975722	0.04818622	NA
MRPL39	0.318243	0.161108	1.975344	0.04822916	0.452182477
CCDC28A	0.27493	0.139236	1.974566	0.04831738	0.452370592
MEX3A	1.040362	0.527247	1.973197	0.0484731	NA
MDFIC	0.749214	0.379794	1.972684	0.04853158	NA
ZNF346	0.544996	0.276313	1.972387	0.04856542	NA
FNDC3B	-1.180799	0.598745	-1.972122	0.04859564	NA
GDF11	0.295078	0.149662	1.971633	0.04865148	0.453961136
NMT2	-0.269505	0.136719	-1.971224	0.0486983	0.453961136
MKLN1	0.292251	0.148306	1.9706	0.0487697	0.453961136
NDUFB11	0.170917	0.086796	1.969172	0.04893332	0.453961136
CARD8	-0.231938	0.117803	-1.968862	0.04896889	0.453961136
SIKE1	0.268053	0.136175	1.968452	0.04901604	0.453961136
SPAG1	-0.585204	0.297343	-1.968112	0.04905513	NA
SPCS1	-0.16638	0.084549	-1.967861	0.04908408	0.453961136
NARS	0.213928	0.108729	1.967526	0.04912256	0.453961136
SOCS4	0.466069	0.236885	1.967491	0.04912658	NA
RPA2	0.192852	0.098031	1.967247	0.04915474	0.453961136
STMN1	-0.218426	0.111039	-1.967105	0.04917114	0.453961136
LRRC8B	0.618861	0.314659	1.966768	0.04920992	NA
SENP3	-0.518271	0.263675	-1.965569	0.04934846	NA
ENSG00000105705	-0.392993	0.199969	-1.965269	0.0493831	0.455284759
YJEFN3	0.833037	0.423913	1.965111	0.04940144	NA
MFSD6	0.759613	0.386652	1.964589	0.04946188	NA
PSMB3	-0.175919	0.089623	-1.96287	0.04966132	0.456832518
ENSG00000231760	-1.121619	0.571722	-1.961826	0.04978277	NA
NPC2	0.29125	0.148519	1.96103	0.04987553	0.456832518
RPLP2	0.160744	0.08197	1.961008	0.04987805	0.456832518
CREBL2	0.359342	0.18328	1.960616	0.04992384	0.456832518
CLNS1A	0.306302	0.156234	1.960534	0.04993337	0.456832518

Cluster 3	log2FC	lfcSE	stat	pvalue	padj
ARRDC3	-0.906905	0.105263	-8.615607	< 2.22e-16	6.71E-14
CKS2	1.060892	0.156998	6.757345	1.41E-11	6.77E-08
ZBED2	2.066521	0.311917	6.625237	3.47E-11	1.11E-07
SMIM10	-4.230547	0.66977	-6.316416	2.68E-10	NA
LRIF1	0.666533	0.107266	6.213866	5.17E-10	1.25E-06
CD83	-0.444947	0.073151	-6.08258	1.18E-09	2.28E-06
RIN3	0.695222	0.122011	5.698023	1.21E-08	1.95E-05
GATD3	5.237077	0.938273	5.581615	2.38E-08	3.28E-05
ASF1A	0.675453	0.132032	5.115829	3.12E-07	0.000348589
NFKBIA	-0.405485	0.079381	-5.108082	3.25E-07	0.000348589
SCRN1	1.075203	0.214137	5.021097	5.14E-07	0.000495276
AIM2	1.736807	0.347411	4.999281	5.75E-07	NA
GPR65	0.543458	0.109702	4.953934	7.27E-07	0.000637361
DYNLL1	0.411612	0.083675	4.919141	8.69E-07	0.000698298
ARL17A	-0.614261	0.126289	-4.863922	1.15E-06	0.000734787
CIRBP	-0.333865	0.068691	-4.860373	1.17E-06	0.000734787
ENSG00000273319	0.612141	0.126004	4.858106	1.19E-06	0.000734787
CHORDC1	0.556131	0.114826	4.843251	1.28E-06	0.000734787
ATF7IP2	-0.651997	0.134699	-4.840399	1.30E-06	0.000734787
LMO4	0.60854	0.127267	4.781616	1.74E-06	0.000931285
ZNF441	-0.722512	0.151552	-4.767413	1.87E-06	0.000946783
TTC32	-0.676371	0.145857	-4.637225	3.53E-06	0.001640089
MS4A1	-0.402119	0.086864	-4.629271	3.67E-06	0.001640089
KRCC1	0.380676	0.082357	4.62226	3.80E-06	0.001640089
RBM3	-0.430066	0.093325	-4.608245	4.06E-06	0.001640089
GALNT2	-0.6758	0.146687	-4.607101	4.08E-06	0.001640089
RHOH	0.373675	0.081743	4.571317	4.85E-06	0.001868877
DNAAF4	-1.166947	0.258729	-4.510303	6.47E-06	0.002367313
SLC3A2	-0.499769	0.110931	-4.505219	6.63E-06	0.002367313
ENSG00000226571	1.235661	0.278802	4.432042	9.33E-06	0.003213735
SIGLEC5	1.782323	0.402959	4.423083	9.73E-06	0.003234457
SNAP23	-0.293072	0.067151	-4.36434	1.28E-05	0.004084577
FAM131A	0.782884	0.180143	4.345911	1.39E-05	0.004084577
CFLAR	0.389848	0.08978	4.342266	1.41E-05	0.004084577
CLECL1	1.747045	0.402629	4.339094	1.43E-05	0.004084577
ARID5A	-0.621658	0.143319	-4.337577	1.44E-05	0.004084577
ENSG00000254397	1.389237	0.321424	4.322136	1.55E-05	0.004223669
GLUL	-0.641415	0.148558	-4.317606	1.58E-05	0.004223669
TNFAIP8L2	0.679909	0.157704	4.311298	1.62E-05	0.004228543
PCIF1	-0.455296	0.106268	-4.284405	1.83E-05	0.004648238
HHEX	0.360067	0.084353	4.268581	1.97E-05	0.004727466
CDKN1B	0.332463	0.077921	4.266657	1.98E-05	0.004727466
HMGB2	-0.378623	0.088801	-4.263706	2.01E-05	0.004727466
NOP10	0.294742	0.069322	4.251756	2.12E-05	0.004795266
DUSP14	0.66526	0.156537	4.249868	2.14E-05	0.004795266
CLEC2D	-0.386421	0.092384	-4.182786	2.88E-05	0.006308894

PIGF	0.688922	0.165275	4.168329	3.07E-05	0.006444678
IZUMO4	-0.629073	0.150936	-4.16782	3.08E-05	0.006444678
SNAI3	0.997271	0.240037	4.154662	3.26E-05	0.00668171
RNF34	0.435951	0.105574	4.129333	3.64E-05	0.007306662
JADE1	0.445268	0.108046	4.121105	3.77E-05	0.007418079
MIR29B2CHG	0.465665	0.11402	4.084077	4.43E-05	0.008531866
KIAA0040	0.686825	0.168627	4.073045	4.64E-05	0.008770964
MDFIC	0.984743	0.243154	4.049878	5.12E-05	0.009499929
MRPS36	0.255238	0.063632	4.011149	6.04E-05	0.010990331
ARL6IP6	0.42088	0.10511	4.004196	6.22E-05	0.011108984
RFTN1	-0.479819	0.120415	-3.984725	6.76E-05	0.011841146
ADAMTS6	0.986226	0.247795	3.980015	6.89E-05	0.01186254
SORD	-0.844154	0.213829	-3.947806	7.89E-05	0.013204865
POU2F2	0.285831	0.072434	3.946058	7.94E-05	0.013204865
NT5E	1.02071	0.259458	3.934011	8.35E-05	0.013649571
TXN	0.486873	0.123957	3.927768	8.57E-05	0.013660976
ZBTB25	0.461119	0.117563	3.922304	8.77E-05	0.013660976
NAB2	0.929734	0.237064	3.921879	8.79E-05	0.013660976
MTMR6	-0.600899	0.154273	-3.895035	9.82E-05	0.014663406
ERV3.1	-0.38838	0.099768	-3.892825	9.91E-05	0.014663406
MIF	-0.347729	0.089397	-3.889719	0.00010036	0.014663406
CCDC117	0.557054	0.143215	3.889641	0.000100393	0.014663406
YWHAE	0.355407	0.091824	3.870519	0.000108604	0.015625973
HLA.G	-1.71986	0.445191	-3.863198	0.000111912	NA
NUFIP2	0.462702	0.120234	3.848356	0.000118913	0.016857684
CYTOR	1.331962	0.347611	3.831758	0.000127231	0.017775459
IGHD	-0.442154	0.115836	-3.817062	0.00013505	0.018350263
KCNK6	0.59021	0.154712	3.814893	0.000136242	0.018350263
SYNGR2	-0.564226	0.148074	-3.810442	0.000138718	0.018350263
APBB2	-1.03418	0.27157	-3.808158	0.000140006	0.018350263
H2AFZ	0.309938	0.08142	3.806648	0.000140863	0.018350263
TMEM170A	0.354453	0.093204	3.802985	0.000142963	0.01837554
SMIM20	0.454421	0.119613	3.799095	0.000145226	0.018407057
OCLN	-1.057143	0.278486	-3.796037	0.000147027	0.018407057
SLC50A1	-0.350839	0.092632	-3.787454	0.000152199	0.018587949
CRIPT	0.395735	0.104515	3.786377	0.00015286	0.018587949
TM2D3	0.396752	0.104847	3.784114	0.000154257	0.018587949
ATP6V1C2	-2.678555	0.708674	-3.779672	0.000157035	NA
LCN8	3.906921	1.033802	3.779178	0.000157347	0.01872625
SKAP2	0.273097	0.072546	3.764445	0.000166919	0.01960483
IL21R	-0.963932	0.256253	-3.761649	0.000168797	0.01960483
SLC2A14	-3.80731	1.012741	-3.759412	0.000170313	NA
GLS	0.421884	0.11264	3.745437	0.00018008	0.020666332
ENSG00000272221	0.858746	0.230673	3.722779	0.000197042	0.022346868
CLEC2B	0.463362	0.124583	3.719303	0.000199773	0.022393175
RAB31	0.970033	0.261755	3.705882	0.000210657	0.023341733
EGLN2	0.312647	0.084478	3.700947	0.000214796	0.023529961

HLX	1.210844	0.328047	3.691072	0.000223311	0.024100005
HSBP1	0.321188	0.087063	3.689155	0.000225	0.024100005
MIR155HG	0.830094	0.225835	3.675673	0.000237223	0.025130023
CRK	0.377479	0.102893	3.668673	0.000243813	0.025547364
ACADVL	-0.270675	0.07417	-3.649383	0.000262871	0.027248098
SCN3A	1.352025	0.370853	3.645716	0.000266649	NA
ABTB1	-0.29489	0.080896	-3.645284	0.000267097	0.02726419
CYB5A	-0.49586	0.136085	-3.643762	0.000268682	0.02726419
PCNX4	0.413661	0.113789	3.635331	0.000277624	0.027878105
MPLKIP	0.231161	0.063715	3.628079	0.000285538	0.028377211
GLRX2	0.564294	0.155782	3.622327	0.000291965	0.028719828
TAGLN2	-0.252827	0.069977	-3.612983	0.000302695	0.029474552
TNFAIP1	0.595717	0.165602	3.597274	0.00032157	0.030733531
TRIR	0.147309	0.040954	3.596925	0.000322001	0.030733531
TAF1D	-0.182812	0.050932	-3.589331	0.000331528	0.031332605
RSRP1	0.283335	0.079185	3.578134	0.000346056	0.032388164
RAF1	0.322477	0.090209	3.574763	0.000350545	0.03249284
YJEFN3	0.927618	0.261254	3.550633	0.000384306	0.035282935
MT1X	-0.879434	0.247879	-3.54784	0.000388404	0.035322756
LHFPL4	1.11981	0.31593	3.544483	0.000393384	NA
SFR1	-0.410136	0.115795	-3.541921	0.000397224	0.035787254
XIAP	0.388941	0.109911	3.5387	0.000402103	0.035891399
TIMM10	-0.497742	0.140926	-3.531939	0.000412524	0.035939054
TTC17	-0.319771	0.090552	-3.531368	0.000413417	0.035939054
CNFN	-0.429206	0.121585	-3.530097	0.000415407	0.035939054
MME	-2.306357	0.653879	-3.527192	0.000419992	NA
CHMP6	-0.496426	0.140764	-3.526658	0.000420841	0.035939054
SESN3	0.309394	0.087782	3.524573	0.000424165	0.035939054
ENSG00000272211	-1.021569	0.289885	-3.524049	0.000425005	0.035939054
PTPRS	2.08247	0.591799	3.518879	0.000433374	NA
SNRPN	0.260136	0.074103	3.510469	0.000447317	0.03749685
MYBL2	-1.938921	0.552882	-3.506933	0.000453303	NA
TMEM243	0.338596	0.096971	3.491723	0.000479916	0.039882676
MAT2A	0.283832	0.081397	3.487015	0.000488444	0.040226173
ENSG00000282988	0.854927	0.245347	3.484568	0.000492932	0.040226173
IFIT5	0.473834	0.13609	3.481769	0.000498114	0.040226173
CHRAC1	-0.321039	0.092243	-3.48036	0.000500741	0.040226173
COL9A3	-1.723667	0.495742	-3.476946	0.00050716	0.040405121
GPR146	-0.931862	0.268656	-3.468609	0.000523159	0.04130651
IFT57	0.52268	0.150775	3.466621	0.000527044	0.04130651
FNBP1	-0.345967	0.100181	-3.453404	0.000553559	0.043034768
GTF3C5	-0.395877	0.114785	-3.448845	0.000562991	0.043417837
MRPL28	-0.287606	0.083553	-3.442193	0.000577018	0.044146471
INKA1	0.485292	0.141294	3.434636	0.00059335	0.045038523
CHP1	-0.296851	0.086627	-3.426761	0.000610828	0.046002948
GPAA1	-0.415578	0.121522	-3.419764	0.000626756	0.046253119
ZCCHC9	0.404566	0.118308	3.419601	0.00062713	0.046253119

CD5	-1.268106	0.370901	-3.418989	0.000628543	0.046253119
AZIN1.AS1	-1.860333	0.547489	-3.397937	0.00067896	NA
A4GALT	1.296368	0.38187	3.394786	0.000686822	0.049948755
CDCA4	0.4629	0.13641	3.393437	0.000690214	0.049948755
TAPBP	-0.249669	0.073609	-3.391817	0.000694308	0.049948755
NR6A1	-1.036896	0.306379	-3.384359	0.000713448	0.050945455
LINC02422	0.665729	0.197012	3.37913	0.000727156	0.051542512
LINC00525	-2.81289	0.834013	-3.372718	0.000744302	NA
BHLHE40	-0.488228	0.14477	-3.372431	0.000745078	0.052086733
ENSG00000173727	0.296929	0.088051	3.372223	0.00074564	0.052086733
RCC1	0.475178	0.141189	3.365539	0.000763942	0.052981283
PFKL	-0.330825	0.098442	-3.36061	0.000777705	0.053550518
DSCAML1	-1.500081	0.446759	-3.357695	0.000785954	NA
HSPE1	0.232143	0.069277	3.350959	0.000805321	0.054759772
MIR181A1HG	-0.573768	0.171248	-3.350511	0.000806627	0.054759772
TEX30	0.492295	0.147091	3.346862	0.000817319	0.055097555
ENSG00000257275	-0.606902	0.181441	-3.344906	0.000823104	0.055102233
ENSG00000263394	0.715972	0.214804	3.333141	0.000858714	0.057089679
PNRC2	0.258484	0.07767	3.328001	0.000874716	0.05775521
MANF	0.374779	0.11285	3.321036	0.00089684	0.058813208
ODF3B	-0.731436	0.220995	-3.30974	0.000933826	0.060824852
PIM1	-0.361973	0.10956	-3.303873	0.00095359	0.061695357
ENSG00000185527	-0.997815	0.30221	-3.301733	0.000960896	NA
LARP4B	0.452635	0.137148	3.300344	0.000965663	0.062059912
DPP7	-0.353173	0.107489	-3.285661	0.001017433	0.064697222
HIPK2	0.589507	0.179459	3.284918	0.001020122	0.064697222
EHD1	-0.370326	0.112806	-3.282847	0.001027645	0.064748329
NUAK2	-0.335724	0.102562	-3.273388	0.001062666	0.06618916
LAPTM5	-0.335023	0.102361	-3.272968	0.001064245	0.06618916
ENSG00000259976	0.656715	0.200787	3.2707	0.001072815	0.066294474
TMEM138	-0.295569	0.090478	-3.266734	0.001087959	0.066301985
IL4R	-0.475145	0.145487	-3.265901	0.001091163	0.066301985
ROCK1	0.234812	0.071912	3.265277	0.00109357	0.066301985
ABHD17A	0.437069	0.134055	3.260384	0.001112615	0.06703507
ENSG00000254614	-0.853697	0.262221	-3.255638	0.001131379	0.06732656
CD48	0.354668	0.10894	3.255627	0.001131421	0.06732656
MCCC2	-0.485503	0.149513	-3.247231	0.001165336	0.068919278
MYO1C	-0.404901	0.124832	-3.243563	0.001180449	0.069280545
MAP3K9	0.776167	0.239471	3.241173	0.001190388	0.069280545
FAM184B	-1.726672	0.532834	-3.240547	0.001193005	0.069280545
LSM6	0.344573	0.106648	3.230945	0.001233817	0.071221538
IFIT2	0.615786	0.190877	3.226088	0.001254949	0.072010193
ZNF350	-0.306739	0.095222	-3.221302	0.001276098	0.072431836
ADA	-0.551165	0.171115	-3.221026	0.001277325	0.072431836
ZNF267	-0.269864	0.084063	-3.210256	0.001326168	0.074761735
KIAA0930	0.857553	0.26737	3.207363	0.001339579	0.075078709
UBAP1L	1.048304	0.327239	3.203487	0.00135774	NA

PRICKLE1	0.606399	0.189304	3.203311	0.001358571	0.07570304
HCK	-0.748083	0.233762	-3.200183	0.001373403	0.076089706
TBC1D9	0.538531	0.168391	3.198094	0.001383389	0.076204995
RUBCNL	-0.305857	0.095794	-3.192875	0.001408639	0.077155005
SSPN	2.283536	0.71591	3.189698	0.001424214	NA
ROR1	-1.163179	0.365244	-3.184662	0.001449232	0.078929939
CDKN1A	-0.362959	0.114067	-3.181968	0.001462782	0.079099654
INPP5K	-0.435088	0.136786	-3.180786	0.001468759	0.079099654
GLRX	0.287717	0.090509	3.178872	0.001478491	0.079181424
ENSG00000225302	2.371706	0.746567	3.176815	0.001489021	NA
C7orf50	-0.5609	0.176716	-3.174025	0.001503406	0.080012062
UGT8	0.813983	0.256563	3.172639	0.001510601	0.080012062
ZNF726	-1.094163	0.345157	-3.170043	0.001524166	NA
GPS2	-0.339433	0.107123	-3.168633	0.001531575	0.08067971
IL23A	-0.669339	0.211622	-3.1629	0.001562061	0.081838415
MED21	0.416483	0.131915	3.157196	0.001592945	0.083005336
FAM41C	-0.705204	0.223537	-3.154757	0.00160632	0.083252289
ITGB7	0.371445	0.118449	3.135913	0.001713201	0.086683873
APOL6	0.86288	0.275165	3.135862	0.001713497	0.086683873
ZNF580	-0.317757	0.101373	-3.134528	0.001721306	0.086683873
GPATCH11	0.466294	0.148782	3.134069	0.001724001	0.086683873
DDX5	-0.371748	0.118643	-3.133326	0.001728372	0.086683873
SPINT2	-0.227372	0.072611	-3.131369	0.001739931	0.086683873
TSC22D2	0.385875	0.123275	3.130192	0.001746921	0.086683873
ENSG00000166927	-0.762691	0.243658	-3.130165	0.001747084	0.086683873
SUSD3	0.612465	0.195732	3.129094	0.00175346	0.086683873
LYRM7	0.292592	0.093577	3.126742	0.001767551	0.086934662
HMGXB4	-0.300102	0.096115	-3.122313	0.001794359	0.087805176
PRCP	-0.396809	0.12773	-3.106619	0.001892401	0.092135083
TMEM187	0.755501	0.243515	3.102482	0.001919055	0.092587706
MAGEE1	1.023203	0.329909	3.101471	0.001925618	NA
ZNF486	0.937018	0.302243	3.100209	0.001933841	0.092587706
ENSG00000258757	0.735415	0.237259	3.099625	0.00193766	0.092587706
FAM200A	0.57884	0.186794	3.09882	0.001942931	0.092587706
TMEM64	0.666633	0.215223	3.097402	0.001952251	0.092587706
NSUN7	-1.029441	0.332472	-3.09633	0.001959325	0.092587706
IFI16	0.194621	0.062941	3.092105	0.001987426	0.093457485
YTHDC1	0.21842	0.07073	3.088103	0.002014387	0.093629524
CLK1	0.259635	0.084148	3.08545	0.002032441	0.093629524
PPP1R9A	1.528429	0.495517	3.084515	0.002038841	NA
CHTOP	0.260678	0.08453	3.083866	0.002043296	0.093629524
IGHG1	1.273043	0.412811	3.083838	0.002043487	0.093629524
DDAH2	0.366539	0.11886	3.083773	0.002043936	0.093629524
EPS15	-0.428854	0.139104	-3.082984	0.00204936	0.093629524
USP28	-0.359864	0.116801	-3.081007	0.002063021	0.093809079
TNFRSF13B	1.121829	0.364598	3.076896	0.002091686	0.094069044
DDX19B	-0.330851	0.107535	-3.076679	0.002093209	0.094069044

SF3B4	-0.398188	0.129498	-3.074844	0.002106127	0.094069044
RRM2B	-0.383902	0.124862	-3.074611	0.002107771	0.094069044
ALOX5	-0.300147	0.097738	-3.070925	0.002133968	0.094799334
NSMAF	0.464512	0.151868	3.058661	0.002223288	0.098314225
ENSG00000272172	-1.522937	0.498043	-3.057843	0.002229361	NA
INAVA	-2.995991	0.980522	-3.055507	0.002246801	NA
PDE4B	0.211589	0.069285	3.053884	0.002258996	0.09847402
CABLES1	-0.693697	0.227169	-3.053662	0.002260663	0.09847402
KPNA2	-0.44671	0.146368	-3.051958	0.002273537	0.09847402
ELP3	-0.563254	0.184604	-3.051148	0.002279684	0.09847402
MFSD13A	-0.70715	0.231806	-3.050618	0.002283711	0.09847402
TNFAIP3	-0.63185	0.207162	-3.050029	0.002288193	0.09847402
RAB9A	0.407206	0.133711	3.045423	0.002323533	0.099204193
AASDH	0.391809	0.128667	3.045137	0.002325741	0.099204193
TAF11	0.312455	0.102692	3.042659	0.002344981	0.099584215
PFKFB3	-0.490162	0.161224	-3.040247	0.002363844	0.099868223
STX7	0.237964	0.078299	3.03916	0.002372388	0.099868223
TMC6	-0.288386	0.094932	-3.037808	0.002383058	0.099881219
SPPL2A	-0.3066	0.10104	-3.034443	0.0024098	0.099921447
ZNF860	0.519527	0.17121	3.034437	0.002409853	0.099921447
PNPLA8	0.268386	0.088466	3.033779	0.002415114	0.099921447
SARS2	-0.697553	0.230052	-3.032155	0.002428146	0.100031305
TAGAP	-0.492943	0.16273	-3.029217	0.002451886	0.100087824
ENSG00000237491	-0.671006	0.221545	-3.028755	0.002455634	0.100087824
NOP16	0.466188	0.153969	3.027796	0.00246344	0.100087824
ADRB2	0.823436	0.27207	3.026559	0.002473549	0.100087824
GPR18	0.629552	0.208075	3.025597	0.00248143	0.100087824
SERPINB6	0.501514	0.166032	3.020582	0.002522896	0.101336335
HLA.C	-0.485151	0.160839	-3.016376	0.002558159	0.101919252
ACAP1	0.293331	0.097248	3.016329	0.002558554	0.101919252
PTPN20	-1.630377	0.541231	-3.01235	0.002592333	NA
MREG	-0.624325	0.2077	-3.005905	0.002647915	0.105044848
MINDY2	0.378593	0.126093	3.002486	0.002677843	0.105796765
PPM1K	0.22176	0.073958	2.998476	0.002713335	0.106761407
RBM15	0.402534	0.134337	2.996445	0.002731474	0.107038268
REEP3	0.452765	0.151178	2.994911	0.002745249	0.107142494
THG1L	0.570651	0.190701	2.992388	0.002768045	0.107596593
CD27	1.127606	0.377183	2.989546	0.002793923	0.108166345
CCDC78	-1.867102	0.625101	-2.986881	0.002818397	NA
DUSP12	-0.310487	0.104004	-2.985341	0.002832624	0.109091027
CARD8	-0.276231	0.092591	-2.983338	0.002851232	0.109091027
P2RY8	-0.336082	0.112655	-2.983281	0.002851757	0.109091027
SPOP	0.259784	0.087273	2.976699	0.002913699	0.111019975
NMT2	-0.382223	0.128459	-2.975449	0.002925599	0.111034548
LILRB1	0.393952	0.132513	2.972941	0.002949607	0.111304639
SLC25A26	-0.264806	0.089138	-2.970753	0.002970705	0.111304639
STN1	0.44127	0.148579	2.969943	0.002978551	0.111304639

TRAF3	-0.261284	0.087977	-2.969907	0.0029789	0.111304639
LY6E	0.349634	0.117799	2.968055	0.002996906	0.111545056
POLE3	-0.271571	0.091552	-2.966306	0.003014005	0.111750015
MRPL54	-0.268261	0.09048	-2.96487	0.003028105	0.11184267
ENSG00000259001	1.094851	0.369705	2.961419	0.003062252	NA
HVCN1	-0.396961	0.134054	-2.961197	0.00306446	0.112451453
EIF2AK4	-0.44711	0.15105	-2.96001	0.003076291	0.112451453
SERTAD2	0.351269	0.118685	2.95968	0.003079583	0.112451453
MED11	0.223049	0.075395	2.958423	0.003092175	0.112485168
HLA.A	-0.469464	0.158929	-2.953931	0.003137545	0.11370652
ARCN1	0.413952	0.140445	2.947435	0.003204226	0.115688147
TMEM120A	-0.470106	0.159661	-2.944412	0.003235694	0.116388398
TMEM70	0.349462	0.118898	2.939169	0.003290933	0.117824309
ARHGEF3	-0.342433	0.116541	-2.938311	0.003300058	0.117824309
ENSG00000278384	1.814486	0.617706	2.937459	0.003309138	NA
ENSG00000267002	0.31354	0.106783	2.936251	0.003322056	0.117866197
LTBP3	0.310247	0.105697	2.935257	0.003332711	0.117866197
DHRS4L2	0.450895	0.153639	2.934774	0.003337912	0.117866197
TOE1	-0.430269	0.147113	-2.924748	0.003447357	0.120829305
SLA	0.375959	0.128603	2.923413	0.003462167	0.120829305
SNX8	-0.357695	0.122398	-2.922396	0.003473492	0.120829305
ARSD	0.711152	0.243472	2.920876	0.003490489	0.120829305
C21orf62.AS1	1.057755	0.362201	2.920354	0.003496341	NA
SMIM4	-0.406814	0.139398	-2.918367	0.003518701	0.120829305
TRIM56	0.225729	0.077362	2.917835	0.003524706	0.120829305
AK6	0.355372	0.12185	2.91648	0.003540057	0.120829305
FAM120A	0.336977	0.115592	2.915224	0.003554339	0.120829305
FAM102A	0.592718	0.203328	2.915086	0.003555901	0.120829305
FNIP1	-0.382095	0.1311	-2.91453	0.00356225	0.120829305
HIST1H3E	0.754127	0.258977	2.911946	0.003591851	0.120829305
GATM	0.938007	0.322181	2.911428	0.003597809	0.120829305
FGR	-0.735434	0.25274	-2.909841	0.003616123	0.120829305
NBPF9	0.608436	0.20912	2.909505	0.003620012	0.120829305
RABEP2	0.250293	0.08603	2.909379	0.00362147	0.120829305
PARP9	0.387314	0.133166	2.908507	0.003631593	0.120829305
RBAK.RBAKDN	0.35122	0.120768	2.908222	0.003634906	0.120829305
ENSG00000265218	-2.08549	0.717473	-2.906715	0.00365246	NA
RAB3GAP1	-0.312244	0.107533	-2.903702	0.003687794	0.121428324
TACC1	0.361836	0.124622	2.903472	0.003690498	0.121428324
TYSND1	-0.514334	0.177169	-2.903069	0.003695253	0.121428324
TPMT	-0.477976	0.164781	-2.900684	0.003723488	0.121428324
GM2A	0.543719	0.187464	2.900385	0.003727047	0.121428324
BAG2	0.527044	0.181723	2.900262	0.003728505	0.121428324
RMND5A	0.453612	0.156587	2.896867	0.003769096	0.122202518
USP53	0.768912	0.265494	2.896157	0.00377763	0.122202518
ZNF667.AS1	0.422151	0.145846	2.894491	0.003797735	0.122442026
FADS3	-0.26965	0.093264	-2.891248	0.003837147	0.123300334

CALM3	-0.284303	0.098426	-2.888485	0.003871022	0.1239756
FCRL4	1.750294	0.606847	2.88424	0.00392359	NA
CHCHD5	-0.307109	0.106546	-2.882412	0.003946439	0.125581419
SUN1	-0.310729	0.107804	-2.882349	0.003947217	0.125581419
SLC25A43	0.424961	0.147492	2.881253	0.003960978	0.125604682
KLB	-2.062507	0.715961	-2.880753	0.003967258	NA
CCDC51	0.50831	0.176623	2.877944	0.00400276	0.12596178
DEDD2	0.290403	0.100917	2.877642	0.004006593	0.12596178
METTL7A	0.298127	0.103615	2.877261	0.004011438	0.12596178
CRTC2	-0.32478	0.11297	-2.874927	0.004041209	0.126484603
FAM122A	0.301002	0.104818	2.871672	0.00408306	0.12738091
AMD1	0.345662	0.120452	2.869705	0.00410855	0.127762667
PILRB	0.481528	0.167941	2.867238	0.004140713	0.128348791
THRB	1.669661	0.58237	2.867013	0.00414366	NA
MAPRE2	0.397558	0.138745	2.865385	0.00416502	0.128688426
CREB3L2	-0.395342	0.138107	-2.862579	0.004202091	0.129419042
RBIS	0.239542	0.083716	2.86137	0.00421814	0.129499579
PEX16	-0.231391	0.080922	-2.859419	0.004244181	0.129785168
ENSG00000280433	-0.838318	0.293181	-2.859384	0.004244646	NA
CCDC126	0.484819	0.169597	2.858658	0.004254369	0.129785168
C9orf72	-0.513412	0.18012	-2.850396	0.004366479	0.13240725
ZBTB3	-0.732261	0.256988	-2.849401	0.004380162	0.13240725
WDR55	-0.324152	0.113765	-2.849302	0.004381526	0.13240725
PHF23	0.296513	0.104144	2.847154	0.004411208	0.13288765
ZNF330	0.363477	0.127755	2.845117	0.004439505	0.133323439
CNTRL	0.210615	0.074067	2.84359	0.004460842	0.133341068
SPATA24	-0.667122	0.234646	-2.843096	0.004467756	0.133341068
ALOX12.AS1	0.358796	0.126298	2.84086	0.004499204	0.133865192
ZNF835	0.889371	0.313136	2.840208	0.004508408	NA
ATP6V0A1	0.432192	0.152457	2.83484	0.004584871	0.135994324
BCL2L11	-0.615994	0.21762	-2.830595	0.00464615	0.137185549
CD79B	-0.233717	0.082613	-2.829057	0.004668536	0.137185549
POLR2L	0.289306	0.102304	2.827896	0.004685509	0.137185549
MTHFR	0.403748	0.142791	2.827539	0.00469073	0.137185549
HRK	-1.662404	0.588011	-2.827167	0.004696186	0.137185549
ZNF827	0.796675	0.281891	2.826177	0.004710719	0.137194357
TMEM205	-0.316397	0.112002	-2.824914	0.004729325	0.137321376
CAV1	-0.639229	0.226838	-2.818	0.004832386	0.139332861
AK1	-0.580421	0.206047	-2.816937	0.004848398	0.139332861
TMEM19	0.238673	0.084792	2.814809	0.004880617	0.139332861
DBNL	-0.252878	0.089842	-2.81471	0.004882125	0.139332861
ENSG00000275464	-0.898276	0.31916	-2.814502	0.00488529	0.139332861
LIMD2	-0.229136	0.081413	-2.8145	0.004885322	0.139332861
MACF1	0.297323	0.105831	2.809415	0.004963163	0.141135363
ENSG00000279483	-0.30216	0.107605	-2.808062	0.004984062	0.141312825
UPB1	-1.49338	0.532397	-2.805013	0.005031453	NA
ZNF391	1.351697	0.48246	2.801676	0.005083794	NA

ENSG00000261786	2.142904	0.766699	2.794975	0.005190376	NA
GRN	-0.285108	0.10209	-2.79271	0.005226847	0.147761898
TTC39B	-1.214583	0.435049	-2.791831	0.005241073	NA
NDUFV3	0.29424	0.105436	2.790686	0.005259652	0.148254514
FRY.AS1	-1.11033	0.398511	-2.786196	0.005333066	NA
AAK1	-0.665325	0.238825	-2.785828	0.005339126	0.14968864
ENSG00000270562	0.63918	0.229452	2.785678	0.005341586	0.14968864
TAF1A	0.480443	0.17253	2.784689	0.005357902	0.149710648
ENSG00000277369	-2.602331	0.934549	-2.784584	0.005359636	NA
RHBDD2	-0.354405	0.127361	-2.782689	0.005391038	0.150201165
CARMIL1	-1.004674	0.361598	-2.778427	0.005462276	0.150764885
RPL8	0.30357	0.109287	2.777734	0.005473946	0.150764885
CMTM7	-0.395453	0.142388	-2.77728	0.005481602	0.150764885
CKS1B	0.233624	0.084133	2.776843	0.005488961	0.150764885
FOXO3	0.473644	0.170647	2.775585	0.00551025	0.150764885
ERGIC2	0.232212	0.083678	2.775074	0.005518907	0.150764885
ENSG00000268400	-1.32119	0.476102	-2.775013	0.005519944	NA
MED13	0.342148	0.123298	2.774966	0.005520747	0.150764885
MYL12A	0.199378	0.071985	2.769706	0.005610697	0.151881812
BNIP3	0.298852	0.107904	2.769613	0.005612295	0.151881812
PMVK	0.297232	0.107322	2.769544	0.005613481	0.151881812
VPREB1	-1.621823	0.585841	-2.768369	0.005633758	0.151881812
ZNF470	0.772951	0.279252	2.767936	0.005641259	0.151881812
UBC	-0.366361	0.132418	-2.766711	0.005662497	0.151881812
GNG11	1.085592	0.392394	2.766589	0.005664604	NA
DDB2	-0.283271	0.102416	-2.765895	0.005676684	0.151881812
EIF5	0.192797	0.069721	2.765263	0.00568769	0.151881812
PROC	1.354931	0.489999	2.765169	0.00568933	NA
ZC3H7A	-0.413084	0.149472	-2.763629	0.005716257	0.152222973
SGMS1.AS1	-0.686578	0.248719	-2.760457	0.005772061	0.153285594
C3orf14	1.848453	0.66969	2.760162	0.005777278	NA
ZNF628	-0.862717	0.31274	-2.758579	0.005805333	0.153441072
ARL4C	-0.632539	0.229319	-2.75833	0.00580975	0.153441072
GOPC	0.338593	0.122893	2.755183	0.005865935	0.154501665
CNOT1	-0.261178	0.094876	-2.75283	0.005908254	0.155122606
NELFE	-0.212901	0.07736	-2.752086	0.005921693	0.155122606
ENSG00000277476	0.964681	0.350569	2.751755	0.005927678	NA
NIPAL3	0.400066	0.145436	2.750807	0.005944872	0.155304347
BLOC1S3	0.407396	0.148148	2.749927	0.005960852	0.155304347
FGFR1OP2	0.268088	0.097523	2.748982	0.00597806	0.155332886
PM20D2	0.520528	0.189872	2.741464	0.006116615	0.158505822
MED23	-0.382389	0.139704	-2.73714	0.006197593	0.159948626
ELOA	0.297589	0.108739	2.736722	0.006205476	0.159948626
APOBEC3H	-0.973374	0.355874	-2.735169	0.006234825	0.160276579
CTDSP2	0.281041	0.10285	2.732549	0.006284639	0.160817091
SNHG9	-0.365774	0.133886	-2.731975	0.006295587	0.160817091
UPP1	0.655629	0.240031	2.731436	0.006305898	0.160817091

ENSG00000007237	-0.791293	0.289908	-2.729459	0.006343836	0.16132299
ENSG00000228463	0.413991	0.151719	2.728661	0.006359205	0.16132299
ENSG00000245904	0.289023	0.105958	2.727705	0.006377659	0.161366498
ELMSAN1	0.322584	0.118312	2.726555	0.006399931	0.161506109
CHID1	-0.374345	0.137476	-2.722976	0.00646967	0.162839735
ERRFI1	1.42442	0.523333	2.721822	0.006492319	NA
ENSG00000273748	-0.934016	0.343283	-2.720833	0.00651176	0.163325071
ENSG00000244701	-0.563501	0.207149	-2.720271	0.006522837	0.163325071
GBP2	0.351092	0.12911	2.719322	0.006541584	0.163370117
LINC00638	1.186595	0.436454	2.718717	0.006553575	NA
SEL1L3	0.354316	0.130521	2.714628	0.00663503	0.165275683
GCNT1	0.701622	0.258602	2.713139	0.006664906	0.165591997
SF1	-0.137746	0.050819	-2.710519	0.006717791	0.166372407
FCGR2B	0.713676	0.263528	2.708157	0.006765809	0.166372407
TMED8	0.355321	0.131225	2.707728	0.006774553	0.166372407
GSTO1	0.261215	0.096472	2.707666	0.006775813	0.166372407
TNFRSF12A	1.066839	0.394055	2.707333	0.00678261	0.166372407
BBX	0.201828	0.074594	2.705692	0.006816214	0.166772342
ZNF419	0.427381	0.158098	2.703273	0.006866026	0.167565802
DNAJA1	0.268408	0.099328	2.702249	0.006887206	0.167658254
TBPL1	0.232037	0.086031	2.697136	0.006993877	0.169354717
PTGIR	-1.054023	0.390854	-2.696721	0.007002587	0.169354717
PHKG1	-0.432647	0.160475	-2.696044	0.007016835	0.169354717
TOB2	0.267048	0.09907	2.695554	0.007027167	0.169354717
FBXW9	-0.89046	0.330713	-2.692547	0.007090844	NA
GPM6B	0.304518	0.113204	2.689998	0.007145242	0.171770916
EIF3J.DT	0.257369	0.095724	2.688668	0.007173775	0.171940186
LINC01355	0.5143	0.191331	2.688009	0.007187956	0.171940186
FAM78A	0.390355	0.145323	2.686127	0.007228566	0.172218935
PRDX3	0.241097	0.089799	2.684861	0.007256004	0.172218935
BYSL	0.684816	0.255122	2.684265	0.007268941	0.172218935
SDE2	-0.507355	0.189065	-2.683502	0.007285561	0.172218935
ENSG00000272831	-0.429619	0.160106	-2.683347	0.007288934	0.172218935
DDX49	-0.382366	0.142589	-2.681598	0.007327135	0.17269825
GTF2IRD2	-0.384834	0.143592	-2.680051	0.0073611	0.173075624
CDC26	0.238739	0.089379	2.671099	0.007560341	0.177327716
ZRANB3	-0.634184	0.237575	-2.669402	0.007598646	0.177703698
FXYD2	-0.772116	0.289317	-2.668758	0.007613239	0.177703698
UNC45A	-0.282765	0.106014	-2.667233	0.007647863	0.178080686
PPBP	2.854967	1.071457	2.664564	NA	NA
DHX30	-0.26267	0.098718	-2.66082	0.007795052	0.181070595
DDX6	-0.230462	0.086654	-2.659576	0.007823908	0.18130403
TERF2IP	0.22002	0.08286	2.655332	0.007923045	0.182819627
YTHDF3	0.412165	0.155232	2.655153	0.007927241	0.182819627
PIGT	-0.329406	0.124161	-2.65306	0.00797657	0.183518229
RTP4	0.609309	0.229788	2.65161	0.008010899	0.183869208
NCBP2	-0.210297	0.079348	-2.650317	0.008041635	0.184136245

UPF2	-0.138622	0.052323	-2.649365	0.008064314	0.184217987
HDDC2	-0.253771	0.095854	-2.647463	0.008109829	0.184423313
GOLGA7	-0.247379	0.093443	-2.64739	0.008111565	0.184423313
DPH5	0.300297	0.113551	2.64461	0.008178521	0.185508094
ZNF473	0.680745	0.257575	2.642903	0.008219867	0.186008262
TRMT112	0.218873	0.0829	2.640207	0.008285546	0.186570187
GID8	-0.227424	0.086161	-2.639523	0.008302291	0.186570187
TSHZ1	0.676657	0.256429	2.638772	0.008320696	0.186570187
NOL12	-0.393043	0.149021	-2.637496	0.008352062	0.186570187
IGHA1	-0.452862	0.171704	-2.637449	0.008353209	0.186570187
C16orf74	-0.406055	0.153976	-2.63714	0.008360822	0.186570187
RNF19A	0.348502	0.13225	2.635182	0.008409212	0.186855397
UCP2	-0.259322	0.098412	-2.635055	0.00841237	0.186855397
ZNF784	-0.6876	0.26122	-2.632263	0.008481809	0.187559523
BARD1	0.367529	0.139647	2.631833	0.008492556	0.187559523
CCDC191	-0.713952	0.271316	-2.631438	0.008502439	0.187559523
ENSG00000272379	1.410762	0.536303	2.630531	0.008525169	NA
CLHC1	0.74967	0.285154	2.629002	0.008563594	0.188400341
ACO2	-0.219637	0.083564	-2.628356	0.008579871	0.188400341
C6orf47	0.247582	0.09423	2.62741	0.008603762	0.188400341
MOB4	0.258686	0.098479	2.626818	0.008618729	0.188400341
FNBP1L	0.586361	0.223386	2.624877	0.008668016	0.188791429
LRP5L	-0.661872	0.252183	-2.624567	0.008675931	0.188791429
TNFRSF17	0.958871	0.365505	2.623412	0.00870539	0.188791429
FAM111A.DT	0.361052	0.137646	2.623038	0.008714957	0.188791429
DNASE1	0.360106	0.137345	2.621913	0.008743764	0.188990778
PDE4DIP	-0.425812	0.16272	-2.616836	0.008874888	0.1913958
LDOC1	0.832412	0.318239	2.615685	0.008904855	0.191613406
PDE6D	-0.307802	0.117794	-2.613049	0.008973851	0.192667987
MALSU1	0.29483	0.112974	2.609728	0.009061428	0.194115934
NRDC	-0.256242	0.098284	-2.607169	0.009129434	0.195139123
MGAT5B	-0.736478	0.282587	-2.606202	0.009155235	0.195257659
TBC1D8	-0.964129	0.370294	-2.603685	0.009222741	NA
DPY30	0.196672	0.075575	2.602332	0.009259225	0.197039568
CYBC1	-0.220977	0.085104	-2.596541	0.009416757	0.199733478
GNPDA1	1.002097	0.385992	2.596159	0.009427254	0.199733478
GATC	0.341663	0.131705	2.594163	0.009482148	0.200455945
C2orf42	0.50367	0.194282	2.592467	0.009529043	0.200984148
ENSG00000254837	0.85048	0.328127	2.591925	0.009544063	NA
ENSG00000266918	-1.067828	0.412064	-2.591414	0.009558235	NA
IL2RA	-0.828927	0.319916	-2.591079	0.009567557	0.200984148
STMN1	-0.317673	0.122642	-2.590241	0.009590875	0.200984148
AGTRAP	-0.379794	0.14664	-2.589969	0.009598461	0.200984148
NRP2	-0.604964	0.233679	-2.588872	0.009629083	0.200984148
C9orf64	0.665563	0.257097	2.58876	0.009632228	0.200984148
ZNF442	-1.361937	0.526219	-2.588158	0.009649079	NA
SORL1	-0.308906	0.11939	-2.587367	0.009671254	0.201362614

PSMA8	1.558479	0.602757	2.585586	0.009721367	NA
PIGX	-0.441953	0.170935	-2.585498	0.009723848	0.202021325
RDX	0.324749	0.125723	2.583049	0.009793134	0.202878717
XRRA1	-0.882864	0.341857	-2.582554	0.009807208	0.202878717
DBI	-0.205654	0.079657	-2.58176	0.009829805	0.202910753
C9orf16	-0.198219	0.076817	-2.580393	0.009868781	0.203280024
ZNF747	-0.390453	0.151578	-2.575917	0.009997454	0.205491385
PURB	0.37356	0.14517	2.573258	0.0100746	0.206354794
LRRC61	-0.610744	0.23738	-2.572849	0.010086513	0.206354794
LPAR5	0.412034	0.160201	2.571978	0.010111923	0.206354794
TRIM4	0.261697	0.101767	2.571528	0.010125085	0.206354794
ENSG00000244459	0.530167	0.206399	2.568645	0.010209695	0.207399409
HSPA1A	-1.423805	0.554373	-2.568317	0.010219369	0.207399409
FKBP5	0.620247	0.241574	2.56753	0.010242586	0.207433885
MIS12	0.286223	0.111559	2.565674	0.010297558	0.208109977
JADE3	0.535892	0.208999	2.564091	0.010344653	0.208370527
TMEM60	0.311244	0.1214	2.563788	0.010353681	0.208370527
S100A13	-0.699847	0.273071	-2.562881	0.010380777	0.208480606
TPM4	0.241441	0.094294	2.560506	0.010451995	0.209474485
UQCRC2	-0.2253	0.088035	-2.559205	0.010491191	0.209771825
PLXNA1	-0.734738	0.287223	-2.558071	0.010525467	NA
APEH	-0.384043	0.15013	-2.55807	0.010525485	0.209771825
MSL3	0.219271	0.085725	2.557851	0.010532112	0.209771825
GCFC2	-0.420248	0.164351	-2.557011	0.01055759	0.209845716
TPI1	-0.150772	0.058987	-2.55603	0.010587394	0.210005101
LUCAT1	1.151097	0.450596	2.554612	0.010630613	NA
RAB23	0.915486	0.35877	2.551738	0.010718724	NA
MLST8	-0.318165	0.12469	-2.551641	0.010721683	0.212232086
POLR3K	0.235166	0.092203	2.550525	0.010756089	0.212418654
MARCHF1	0.45882	0.179936	2.549907	0.010775179	0.212418654
CASP4	0.171507	0.067302	2.548309	0.010824646	0.212958337
S1PR4	0.332309	0.130475	2.546905	0.010868312	0.213381934
ENSG00000272426	0.899759	0.353559	2.54486	0.010932168	NA
BET1	0.255193	0.100279	2.544843	0.01093269	0.214209609
KLK4	1.932528	0.759725	2.543721	0.010967851	NA
MOB3B	0.659278	0.259186	2.543648	0.010970172	0.214439137
CERS4	-0.314954	0.123849	-2.543052	0.010988894	0.214439137
SCAMP4	-0.462194	0.181921	-2.540626	0.011065413	0.215496133
UACA	1.588208	0.625325	2.539814	0.011091157	NA
NDUFAF4	0.273037	0.107581	2.537963	0.011149982	0.216705291
ENSG00000271204	0.434991	0.171604	2.534845	0.01124972	0.217784411
PHF20	0.178383	0.070375	2.534742	0.011253005	0.217784411
CSDE1	-0.151986	0.059993	-2.53338	0.011296842	0.217784411
PARP15	0.327502	0.129309	2.532706	0.011318592	0.217784411
ARHGAP17	0.292886	0.115699	2.531461	0.011358861	0.217784411
NFKB2	-0.395099	0.156118	-2.530763	0.011381457	0.217784411
ILK	0.200973	0.079414	2.530684	0.011384035	0.217784411

CCDC71	-0.366438	0.144802	-2.530616	0.011386239	0.217784411
ZKSCAN1	-0.232638	0.091967	-2.529587	0.011419687	0.217991657
DHTKD1	-0.346789	0.137171	-2.528143	0.011466753	0.218311765
TMF1	0.202155	0.079986	2.527396	0.011491182	0.218311765
ENSG00000223881	-1.074169	0.425048	-2.527172	0.011498503	NA
ZNF557	0.461746	0.182726	2.526993	0.011504396	0.218311765
WBP1L	-0.298134	0.118082	-2.524798	0.011576495	0.219041528
TRIM69	0.340218	0.13477	2.524439	0.011588297	0.219041528
DCUN1D5	0.275544	0.109208	2.523098	0.011632592	0.21944851
CREBRF	0.205851	0.081615	2.522232	0.011661284	0.219529162
CD44	-0.23864	0.094638	-2.521595	0.011682413	0.219529162
SPINK2	-1.885229	0.747874	-2.520784	0.011709382	NA
ENSG00000267042	1.739171	0.690157	2.519964	0.011736688	NA
IGLV4.69	-2.097872	0.832677	-2.519431	0.011754468	NA
TRADD	-0.243154	0.0966	-2.517132	0.011831445	0.220628521
RALGPS2	0.300132	0.119245	2.516939	0.011837917	0.220628521
TCTN1	-0.763839	0.303542	-2.516419	0.011855413	0.220628521
GBGT1	0.581525	0.231151	2.515784	0.01187678	0.220628521
VPS26C	0.277213	0.110193	2.515706	0.011879429	0.220628521
DIP2B	0.368645	0.146579	2.514995	0.011903409	0.220628521
CXorf21	0.374027	0.148752	2.514433	0.011922403	0.220628521
RBBP8	-0.472804	0.188039	-2.514385	0.01192401	0.220628521
SLC38A2	0.24901	0.099074	2.513375	0.011958212	0.220837472
AP4M1	-0.317901	0.12652	-2.512659	0.011982516	0.220863207
LZTS3	-1.217939	0.485052	-2.510943	0.012040926	NA
SAMD9L	0.323284	0.128811	2.509761	0.012081284	0.221508603
GPATCH2	-0.402455	0.160364	-2.509624	0.012085985	0.221508603
PAFAH2	0.686438	0.273524	2.50961	0.012086465	0.221508603
ZNRD1ASP	-0.441665	0.176213	-2.506427	0.012195814	0.222587568
CPLANE1	-0.443497	0.176957	-2.506233	0.012202504	0.222587568
NDUFAF1	0.357688	0.142739	2.505883	0.012214608	0.222587568
FYCO1	0.937562	0.374401	2.504165	0.012274086	NA
FMNL2	-1.466461	0.585966	-2.50264	0.012327089	NA
ENSG00000275964	-0.652133	0.260591	-2.502512	0.012331528	0.223958297
ENSG00000268516	0.387623	0.154902	2.502376	0.012336292	0.223958297
C6orf226	-0.406671	0.162563	-2.501625	0.012362462	0.224011529
CD37	-0.201285	0.080515	-2.499962	0.012420665	0.224529212
PDCL3	-0.332311	0.132959	-2.49935	0.012442126	0.224529212
JMJD6	0.31584	0.126396	2.498816	0.012460905	0.224529212
HSH2D	-0.328509	0.131521	-2.497762	0.012497997	0.224777413
PLAAT3	-0.903806	0.362394	-2.493986	0.012631769	0.226404768
NUCB2	-0.540416	0.216729	-2.493508	0.012648786	0.226404768
HNRNPDL	-0.184246	0.073903	-2.493088	0.012663762	0.226404768
TSPAN33	-0.336321	0.13493	-2.492555	0.012682758	0.226404768
RHOT2	-0.2119	0.085035	-2.491908	0.012705911	0.226404768
CLNS1A	0.259339	0.104151	2.490029	0.012773278	0.226877522
SCP2	-0.290299	0.116593	-2.489855	0.012779512	0.226877522

COX17	0.314517	0.126393	2.488412	0.012831512	0.227369764
TPRA1	0.406747	0.163498	2.487778	0.012854411	0.227369764
C17orf49	-0.462936	0.186175	-2.486559	0.012898512	0.227731976
LINC00662	0.294875	0.118802	2.482062	0.013062439	0.229859586
ENSG00000255240	-0.549757	0.22154	-2.481523	0.01308221	0.229859586
SNAPC1	0.318273	0.128269	2.481296	0.013090551	0.229859586
CNOT6L	-0.450012	0.181575	-2.478388	0.013197764	0.230861454
GADD45B	-0.344762	0.139107	-2.478386	0.013197826	0.230861454
EIF4E	0.197487	0.079711	2.477529	0.013229569	0.230861454
GINS4	-0.552733	0.223132	-2.477156	0.013243401	0.230861454
IKZF2	-0.611851	0.247179	-2.475335	0.013311124	0.231615204
OPTN	-0.522105	0.210977	-2.474703	0.013334693	0.231615204
RHEB	0.226147	0.091431	2.473409	0.013383079	0.231847429
UNC50	0.291963	0.118082	2.47254	0.013415667	0.231847429
ENSG00000258056	0.579013	0.234225	2.472034	0.01343467	0.231847429
NAA10	-0.158491	0.06412	-2.471779	0.013444265	0.231847429
C19orf12	0.336793	0.136318	2.470638	0.013487213	0.232172742
BHLHE41	1.149076	0.465155	2.470308	0.013499662	NA
NFE2L3	1.184714	0.479868	2.468833	0.01355546	NA
ZNF506	0.331767	0.134403	2.468445	0.013570156	0.233184137
IRF2BPL	0.36505	0.147964	2.467156	0.01361911	0.233608934
ETS2	-1.043487	0.423533	-2.463769	0.013748457	NA
C12orf65	0.24897	0.101067	2.46341	0.01376223	0.235285613
TOB1	0.347784	0.141185	2.463321	0.013765673	0.235285613
DTX3L	0.341194	0.138673	2.460415	0.013877626	0.236779312
DHX9	-0.223124	0.090772	-2.458082	0.013968143	0.23786215
BMI1	0.30248	0.123084	2.457509	0.01399044	0.23786215
IGHV3.30	1.111375	0.453197	2.452299	0.014194672	NA
CYTIP	0.183134	0.074685	2.452074	0.014203564	0.240221556
S100A11	0.336669	0.137311	2.451878	0.014211275	0.240221556
LCLAT1	0.642027	0.261878	2.451624	0.014221329	0.240221556
PPP2R2A	0.225114	0.091844	2.451045	0.014244211	0.240221556
CA2	0.720318	0.29389	2.450978	0.014246865	NA
TCL1A	-0.430437	0.175631	-2.450803	0.01425381	0.240221556
TMEM101	0.283717	0.115796	2.450138	0.014280139	0.240232301
CDC42EP3	0.684629	0.279536	2.449164	0.014318812	0.240232301
CEP135	-0.21056	0.085981	-2.448903	0.014329209	0.240232301
EEFSEC	-0.339293	0.138601	-2.447982	0.0143659	0.240429296
RHBDF2	-0.231711	0.094709	-2.44656	0.014422664	0.240661341
NDUFB11	0.207247	0.084729	2.445992	0.014445401	0.240661341
MRPL32	0.224443	0.091768	2.445761	0.014454659	0.240661341
PRDX2	-0.407855	0.166988	-2.442414	0.014589402	0.241349731
NRROS	-0.270755	0.110887	-2.441712	0.014617823	0.241349731
LDHA	-0.298754	0.122397	-2.440864	0.014652169	0.241349731
ENSG00000166685	-0.377964	0.154861	-2.440669	0.014660091	0.241349731
EXOSC3	0.242486	0.099353	2.440661	0.014660421	0.241349731
NDUFA8	0.211023	0.086482	2.440069	0.01468446	0.241349731

TAOK3	0.154402	0.063287	2.439727	0.014698356	0.241349731
ENSG00000278869	-1.372303	0.562529	-2.439522	0.014706694	NA
RAB14	0.201863	0.082758	2.439191	0.014720187	0.241349731
TMEM99	0.505968	0.207435	2.439163	0.014721332	0.241349731
TMEM44	1.176217	0.482455	2.437981	0.014769565	NA
TPP1	0.220439	0.090531	2.434945	0.014894063	0.24291553
ACTN1	2.025524	0.832182	2.433992	0.014933329	NA
ATP6V0E2	-0.546584	0.224584	-2.433756	0.014943063	0.24291553
CLPTM1	-0.225675	0.092732	-2.433631	0.014948227	0.24291553
CTSH	-0.183722	0.075496	-2.433539	0.014952041	0.24291553
RAB10	0.251984	0.103577	2.432818	0.014981819	0.24291553
HLA.DMB	-0.257051	0.105663	-2.43275	0.014984655	0.24291553
HMGN2	-0.173934	0.071503	-2.432543	0.01499323	0.24291553
POR	-0.32243	0.13264	-2.430865	0.015062806	0.243633305
ENSG00000228172	-1.550922	0.63839	-2.429427	0.015122704	NA
GPSM2	-0.444644	0.183043	-2.429179	0.015133065	0.244255482
RRP1B	0.375036	0.154458	2.428079	0.01517904	0.244255482
NRDE2	-0.27923	0.115026	-2.427534	0.015201861	0.244255482
RNF38	0.365068	0.150388	2.427516	0.015202623	0.244255482
SERTAD1	0.408418	0.168326	2.426347	0.015251677	0.244568455
PPIL4	0.257809	0.106308	2.425116	0.015303487	0.244568455
LSR	0.68907	0.28417	2.424855	0.015314523	0.244568455
SLTM	0.181095	0.07469	2.42464	0.015323584	0.244568455
ZNF547	0.822169	0.339424	2.422247	0.015424852	NA
ASB16.AS1	0.399857	0.165083	2.422155	0.015428773	0.245659567
ENSG00000235078	0.473279	0.195461	2.421352	0.015462902	0.245659567
USF1	-0.573339	0.236797	-2.421223	0.015468398	0.245659567
TNRC6C	0.344169	0.142303	2.418568	0.015581723	0.247052322
LANCL2	-0.713504	0.295266	-2.41648	0.015671393	NA
CDC42SE2	0.206113	0.085305	2.416191	0.01568382	0.247573742
PSMA5	0.177786	0.073596	2.415692	0.015705323	0.247573742
PFDN2	0.166644	0.068991	2.415455	0.015715551	0.247573742
XKR8	0.43758	0.181187	2.415074	0.015732015	0.247573742
SRSF6	-0.285443	0.118205	-2.414819	0.015743019	0.247573742
GPR183	0.393364	0.163176	2.410671	0.01592319	0.249867998
C8orf37	0.674546	0.279863	2.410273	0.015940592	NA
TMC8	-0.345587	0.143396	-2.410021	0.015951589	0.249867998
KIF1BP	0.477009	0.197956	2.409677	0.015966669	0.249867998
WDR92	0.416019	0.172823	2.407196	0.016075547	0.251164146
ATP13A3	0.399144	0.165858	2.406533	0.016104758	0.25121338
LIMD1	-0.74012	0.307796	-2.404576	0.016191239	0.251656337
ZNF608	-0.591373	0.245988	-2.40407	0.016213661	0.251656337
APAF1	0.454102	0.188889	2.404065	0.016213899	0.251656337
YARS	-0.387903	0.161426	-2.402979	0.016262105	0.251656337
PFKP	-0.559377	0.232788	-2.402944	0.016263682	0.251656337
ENSG00000228242	1.294571	0.538786	2.402755	0.016272081	NA
TNFRSF14	0.173708	0.072338	2.401326	0.016335784	0.252080189

MBP	-0.230101	0.09585	-2.400647	0.016366127	0.252080189
ZC3H6	0.487046	0.202887	2.400571	0.016369523	0.252080189
MAP3K4	0.333038	0.138771	2.399902	0.016399481	0.252138751
HIP1R	-0.334217	0.139311	-2.39907	0.016436772	0.252309685
ENSG00000276449	0.928079	0.387069	2.397711	0.016497887	NA
MICAL1	-0.346141	0.144457	-2.396148	0.016568421	0.253650009
NEIL1	-0.629627	0.262899	-2.394942	0.016622975	0.253650009
GATD1	0.276208	0.115338	2.394774	0.016630615	0.253650009
BAG1	-0.194403	0.081196	-2.394241	0.016654806	0.253650009
JARID2	-0.291537	0.121782	-2.393921	0.016669355	0.253650009
POLR2J	-0.178853	0.074751	-2.392657	0.016726877	0.253650009
BIN1	0.359564	0.150354	2.391451	0.016781943	0.253650009
C4orf36	0.593021	0.248005	2.39117	0.016794784	0.253650009
ZFP37	1.171485	0.490119	2.390204	0.016839027	NA
ZNF138	0.335426	0.140363	2.389704	0.016861978	0.253650009
PCYT1A	0.364373	0.152478	2.389675	0.016863285	0.253650009
HSPBAP1	-1.060638	0.443857	-2.389597	0.016866881	0.253650009
DIPK1A	-0.447733	0.18746	-2.388418	0.01692109	0.253650009
ASCC2	-0.25646	0.107406	-2.387765	0.016951169	0.253650009
LLGL1	-0.676866	0.283549	-2.387123	0.016980787	0.253650009
ZNF594	0.609196	0.255225	2.3869	0.0169911	0.253650009
ENSG00000188206	0.302909	0.126907	2.386862	0.016992878	0.253650009
MED25	-0.385915	0.161701	-2.386599	0.017005033	0.253650009
BCS1L	0.394452	0.165295	2.386355	0.017016328	0.253650009
ZBTB43	-0.317587	0.133094	-2.386189	0.01702402	0.253650009
TUNAR	-1.520323	0.637413	-2.385145	0.017072391	NA
RASSF1	0.220894	0.09262	2.38496	0.017080979	0.253716467
SDAD1	-0.261782	0.109764	-2.384957	0.017081119	0.253716467
ADD2	0.841739	0.353134	2.383626	0.017143034	NA
RPRD1A	-0.243313	0.102119	-2.38265	0.017188513	0.254550782
GPN3	0.36739	0.154262	2.381598	0.017237691	0.254550782
ENSG00000272758	0.613999	0.257818	2.381524	0.017241187	0.254550782
ENSG00000261669	0.768193	0.32262	2.381107	0.017260701	0.254550782
PSMA4	-0.130101	0.054643	-2.380923	0.017269317	0.254550782
ZBTB20.AS2	-1.076662	0.452221	-2.380829	0.017273713	NA
RNF43	-0.662986	0.278576	-2.379915	0.017316623	0.254587651
LTBP4	0.380352	0.159829	2.379745	0.017324637	0.254587651
TMA16	0.283821	0.119366	2.377739	0.017419133	0.254922136
RBM28	0.272153	0.114482	2.377252	0.017442177	0.254922136
RPL24	0.203397	0.085567	2.377042	0.01745209	0.254922136
ATG101	0.26315	0.110706	2.377019	0.017453175	0.254922136
UBE2J2	-0.181608	0.076421	-2.376398	0.017482584	0.254965375
SNRPG	0.160576	0.06763	2.374335	0.017580576	0.255669147
ZBTB20	0.248874	0.104821	2.374266	0.017583884	0.255669147
TSFM	0.339043	0.142973	2.371383	0.017721649	0.25728418
PLTP	-1.234125	0.52096	-2.368943	0.017839006	NA
TOX4	-0.209834	0.088633	-2.367449	0.017911167	0.259644587

ZDHHC16	0.297357	0.125658	2.366398	0.01796212	0.259733531
BDP1	0.145741	0.061592	2.366211	0.017971189	0.259733531
RGMB	0.810702	0.342719	2.365503	0.018005583	NA
TCL1B	-1.242108	0.525821	-2.362224	0.018165646	0.261976473
HNRNPH3	0.180874	0.076593	2.361504	0.018200989	0.261976473
ECE1	-0.389026	0.164754	-2.361249	0.018213511	0.261976473
PSMC3	-0.207839	0.088045	-2.360592	0.018245773	0.261976473
AGMAT	0.576338	0.244185	2.360257	0.01826226	0.261976473
OTUD6B.AS1	0.215057	0.091145	2.359495	0.018299804	0.262124025
BCLAF1	-0.162901	0.069057	-2.358946	0.018326929	0.262124025
YY1	0.152605	0.064759	2.356512	0.018447479	0.262741848
OTUD1	0.504246	0.213987	2.356436	0.018451268	0.262741848
UBALD2	-0.249223	0.105763	-2.356423	0.018451891	0.262741848
BEND4	0.516975	0.219631	2.353837	0.018580775	0.264186824
NPHP4	-1.115287	0.474093	-2.352464	0.018649495	NA
PGPEP1	0.430555	0.183035	2.352301	0.018657646	0.264889119
PRDX6	-0.174361	0.074186	-2.350337	0.018756438	0.265900088
DTNBP1	-0.200815	0.085589	-2.346281	0.018961814	0.268378487
ENSG00000239636	-0.59951	0.255569	-2.345787	0.018986943	0.268378487
ENSG00000277007	0.900116	0.383833	2.34507	0.019023521	NA
DDX23	0.30078	0.128267	2.344958	0.019029209	0.268582097
ENSG00000276075	-1.077995	0.460021	-2.343359	0.019110978	NA
WAC	0.155672	0.066475	2.341806	0.019190696	0.270076051
BOLA2B	-0.360762	0.154053	-2.341798	0.019191089	0.270076051
LINC01250	-2.012553	0.859833	-2.340633	0.01925107	NA
UBE2L3	-0.146382	0.062579	-2.339145	0.019327932	0.270989764
RAE1	-0.336519	0.143893	-2.338676	0.01935221	0.270989764
DPYD	-0.410374	0.175505	-2.338239	0.019374846	0.270989764
OGT	0.272605	0.116593	2.338098	0.019382155	0.270989764
GNG3	-1.343126	0.574478	-2.337994	0.019387557	NA
RBM18	0.289409	0.123794	2.33782	0.01939657	0.270989764
ZSCAN29	0.457447	0.195803	2.336269	0.019477218	0.27165467
CDC37	-0.166878	0.071443	-2.335822	0.019500522	0.27165467
ENSG00000267169	-0.639668	0.273919	-2.335243	0.019530744	NA
ZBTB16	1.469758	0.629481	2.33487	0.019550198	NA
LPP.AS2	-0.849675	0.36395	-2.334594	0.019564615	0.272154239
ARID1A	0.238832	0.102327	2.334014	0.019594986	0.272183953
AKAP12	-1.735568	0.743802	-2.333373	0.019628559	NA
HSPA8	0.300879	0.128959	2.333129	0.019641355	0.272435492
KPNA6	0.219499	0.094122	2.332081	0.019696417	0.272758104
ING1	0.299385	0.128437	2.330985	0.019754125	0.272758104
ENSG00000272106	-0.391122	0.167819	-2.330615	0.019773689	0.272758104
SDF2L1	0.24965	0.107143	2.330073	0.019802318	0.272758104
LPIN1	0.50362	0.216146	2.330001	0.019806086	0.272758104
DDI2	0.334699	0.143827	2.327091	0.019960419	0.274491361
PSMB10	-0.211002	0.090702	-2.326322	0.020001393	0.274663006
JUP	-1.006515	0.433331	-2.32274	0.020193105	0.276478586

ARHGAP30	0.182219	0.078475	2.322007	0.020232537	0.276478586
ZNF2	0.592888	0.255348	2.321885	0.020239133	0.276478586
ENSG00000224086	-0.387217	0.166787	-2.321634	0.020252658	0.276478586
SLC25A38	0.231989	0.099959	2.320838	0.020295595	0.276478586
GRAMD1C	-0.33736	0.145373	-2.320651	0.020305689	0.276478586
DLGAP3	-0.789879	0.340678	-2.318547	0.020419634	NA
SP2	-0.302041	0.130292	-2.318183	0.020439358	0.277557786
ENSG00000229539	0.361154	0.155819	2.317786	0.020460963	0.277557786
ZNF284	0.501504	0.21639	2.317595	0.020471326	0.277557786
UBE2E3	-0.214999	0.09281	-2.316562	0.020527594	0.277929781
OAS2	0.392717	0.169574	2.315905	0.020563471	0.278025045
NAA60	-0.259469	0.112146	-2.313664	0.020686177	0.278806703
SFXN3	0.549334	0.237445	2.31352	0.020694089	0.278806703
CCDC88B	0.320902	0.138722	2.313265	0.02070805	0.278806703
ZNF395	0.264439	0.114474	2.310035	0.020886235	0.280792593
ARPC3	-0.132395	0.057325	-2.309537	0.020913805	0.280792593
TYW3	0.301001	0.130384	2.308579	0.020966935	0.281114406
ZNF420	0.348108	0.150861	2.307471	0.021028591	0.281147259
PSAP	-0.153908	0.066713	-2.307023	0.021053565	0.281147259
APIP	0.237781	0.103071	2.306963	0.02105688	0.281147259
ATP6V0A2	-0.35007	0.151809	-2.305993	0.021110997	0.281479954
CTBP1.AS	-1.276876	0.554036	-2.30468	0.021184476	NA
CIPC	0.385058	0.167084	2.304574	0.021190455	0.282052038
ZC3H12D	-0.351046	0.15238	-2.303756	0.021236317	0.282052038
UBLCP1	-0.316544	0.137409	-2.303661	0.021241678	0.282052038
CNR1	0.711396	0.309004	2.302224	0.021322528	0.282531849
ZNF184	0.440309	0.191288	2.301813	0.021345735	0.282531849
GINM1	0.303166	0.131728	2.301458	0.021365738	0.282531849
PJA1	0.365318	0.158791	2.300625	0.021412836	0.28276676
CEMIP2	0.543599	0.23637	2.299783	0.021460501	0.283008528
AP1S3	-0.509132	0.221461	-2.298968	0.021506738	0.28323081
NPHP3	0.343873	0.1497	2.297072	0.02161469	0.283966938
PPP4R2	0.237276	0.103301	2.296951	0.021621549	0.283966938
POLR2B	-0.23662	0.103068	-2.295762	0.021689499	0.284471791
ENSG00000277654	-0.464981	0.202597	-2.2951	0.021727413	0.284581871
DNAJA2	-0.204152	0.088976	-2.294452	0.021764561	0.284681644
AGO2	-0.242292	0.105656	-2.293211	0.021835865	0.284872375
HINT1	0.233137	0.101668	2.293119	0.021841178	0.284872375
MSL2	0.282448	0.12328	2.291119	0.021956546	0.284872375
FRG1	-0.210053	0.091707	-2.290486	0.021993131	0.284872375
HLA.E	-0.260839	0.11388	-2.290466	0.021994343	0.284872375
SETDB1	-0.360352	0.157335	-2.290355	0.022000719	0.284872375
ELP6	-0.320438	0.139967	-2.289387	0.022056886	0.284872375
FAM214B	0.5619	0.24553	2.288522	0.022107138	0.284872375
CDC42	-0.159678	0.069774	-2.288494	0.022108772	0.284872375
TAOK1	0.274947	0.120144	2.28848	0.022109562	0.284872375
NUDCD2	0.232226	0.101487	2.288239	0.022123615	0.284872375

CNIH4	0.218412	0.095457	2.288065	0.022133756	0.284872375
HIST1H2BN	0.652152	0.285194	2.286699	0.02221338	0.285515979
PRPF18	-0.28806	0.126005	-2.286094	0.022248737	0.285589644
RNASEL	0.534892	0.23403	2.28557	0.022279407	0.285603038
ENSG00000267213	-1.820087	0.796399	-2.285396	0.022289628	NA
NOTCH2	0.281947	0.123438	2.284117	0.022364656	0.286315116
TMUB1	-0.227589	0.099677	-2.283261	0.022415009	0.286579165
PCBD1	0.413756	0.181327	2.281825	0.022499647	0.28728026
ABI1	0.192018	0.084217	2.280047	0.022604891	0.288242262
TSPO	0.19151	0.084036	2.278891	0.022673533	0.288546732
CISD1	0.255499	0.112128	2.278637	0.022688633	0.288546732
FAM89A	1.187535	0.521219	2.27838	0.022703957	NA
PAK1IP1	0.341597	0.150008	2.277195	0.022774584	0.289258221
DGAT2	0.681154	0.299129	2.277123	0.022778854	NA
ARHGAP20	1.291142	0.567362	2.275694	0.022864352	NA
NCBP2AS2	-0.241269	0.106165	-2.272594	0.023050653	0.291859637
MRNIP	0.23175	0.101994	2.272191	0.023074987	0.291859637
THEMIS2	0.708246	0.311718	2.272069	0.023082319	0.291859637
KLF6	0.304123	0.133871	2.271768	0.023100509	0.291859637
HIPK1	0.355692	0.156641	2.270745	0.023162403	0.292258596
BATF2	1.14306	0.503455	2.27043	0.023181527	NA
ZNF613	0.725898	0.31975	2.270203	0.023195269	NA
TTC21A	0.312017	0.137544	2.268493	0.023299188	0.29360023
ATP6V1C1	0.268665	0.118523	2.26677	0.023404261	0.294539269
STT3B	-0.329412	0.145408	-2.265426	0.02348653	0.294654164
NUP107	-0.229407	0.101297	-2.26469	0.023531687	0.294654164
JRKL	0.402949	0.177928	2.264678	0.02353247	0.294654164
KLHL21	0.50577	0.223375	2.264216	0.023560819	0.294654164
NAALADL1	0.442018	0.195227	2.264128	0.02356622	0.294654164
HIST1H2BH	0.830854	0.367453	2.261117	0.023752043	NA
SLC15A2	0.790525	0.349701	2.260577	0.023785441	NA
CBFA2T3	0.571699	0.25323	2.257631	0.023968687	0.297451548
MED30	0.235782	0.104444	2.257493	0.02397727	0.297451548
REL	-0.271584	0.120304	-2.257483	0.023977917	0.297451548
VPS11	-0.364878	0.161681	-2.256779	0.024021884	0.297451548
SUB1	0.13462	0.059662	2.256385	0.024046516	0.297451548
MIATNB	0.490883	0.217558	2.25633	0.024049979	0.297451548
TSNAX	-0.261451	0.115891	-2.256009	0.024070052	0.297451548
CYC1	-0.170618	0.075671	-2.254741	0.024149567	0.297451548
IGKV3.20	-0.82903	0.367708	-2.254585	0.024159371	NA
UGDH	0.280876	0.124592	2.254367	0.02417307	0.297451548
LINC02362	-1.4649	0.649837	-2.254258	0.024179917	NA
TNFRSF10D	0.591574	0.262528	2.253374	0.024235552	0.297451548
COMMD6	0.126199	0.056005	2.253337	0.024237938	0.297451548
FLYWCH1	0.353324	0.156803	2.253293	0.02424066	0.297451548
GIT2	0.194001	0.0861	2.253199	0.024246621	0.297451548
PRMT1	-0.189263	0.084026	-2.252442	0.024294377	0.297451548

TSC22D1	-0.424737	0.188574	-2.252369	0.02429899	0.297451548
ELOC	0.192633	0.085529	2.252252	0.024306336	0.297451548
MAP3K8	-0.452597	0.201016	-2.251546	0.024351004	0.297451548
PYROXD1	-0.363854	0.161652	-2.25084	0.024395684	0.297451548
ATP1A1	-0.197422	0.087711	-2.250834	0.024396055	0.297451548
ENSG00000267390	0.462101	0.205318	2.25066	0.024407072	0.297451548
HIRA	-0.451131	0.200504	-2.249979	0.024450296	0.297602093
ENSG00000232611	0.643721	0.286158	2.249528	0.024478895	NA
PCGF1	-0.428319	0.190459	-2.24888	0.024520163	0.298076125
TAT.AS1	0.951655	0.423227	2.248568	0.024540007	NA
ENSG00000258308	-1.509776	0.671605	-2.24801	0.024575521	NA
EYA3	0.559103	0.24876	2.247559	0.024604345	0.298165384
THAP2	0.331562	0.147537	2.247324	0.024619328	0.298165384
IKZF5	0.333449	0.148412	2.246781	0.024653995	0.298165384
TRA2B	-0.155683	0.069307	-2.246262	0.024687246	0.298165384
CD82	-0.216672	0.096469	-2.24602	0.024702756	0.298165384
RNH1	-0.230852	0.10279	-2.245858	0.024713085	0.298165384
SSBP4	-0.289651	0.129012	-2.245149	0.024758573	0.2983408
NEDD8	0.119982	0.05347	2.243918	0.024837679	0.298571996
ACTL6A	0.28044	0.124991	2.24367	0.024853623	0.298571996
RYK	0.376165	0.167676	2.243406	0.024870676	0.298571996
THNSL1	0.816568	0.364098	2.242717	0.024915038	NA
CD38	-0.512464	0.228621	-2.241549	0.024990555	0.299027299
IL2RG	-0.249508	0.111313	-2.2415	0.024993683	0.299027299
LACTB2	0.33353	0.148806	2.241377	0.02500166	0.299027299
ZNF709	-1.397995	0.623948	-2.240564	0.02505432	NA
BAZ2B	-0.341969	0.152678	-2.239808	0.025103394	0.299872019
RASGEF1A	-0.834402	0.372668	-2.238992	0.025156447	NA
HHIP.AS1	-1.451788	0.648716	-2.237941	0.025224935	NA
PRRT3	0.550644	0.246075	2.23771	0.025239977	0.301130422
HES6	-0.537488	0.240289	-2.236841	0.025296739	0.301434562
ZBTB4	0.304131	0.136013	2.236045	0.025348799	0.301682
SRSF11	0.15124	0.067701	2.233939	0.025487108	0.30233239
ST3GAL1	0.3436	0.15383	2.23364	0.025506766	0.30233239
SPDL1	0.518211	0.232029	2.233386	0.025523495	0.30233239
PRR4	0.376771	0.168706	2.233304	0.025528897	0.30233239
CENPM	-0.379531	0.170032	-2.232113	0.02560749	0.302626271
POLH	0.40591	0.181861	2.231977	0.025616498	0.302626271
DCXR.DT	-1.107495	0.496595	-2.230175	0.025735846	NA
RCSD1	-0.287432	0.128919	-2.229559	0.025776758	0.30360294
MTHFD2L	-0.341373	0.153131	-2.229294	0.025794335	0.30360294
PDCD4	0.426457	0.191347	2.228714	0.025832921	0.30360294
RAD23A	0.134139	0.060196	2.228386	0.025854817	0.30360294
UBB	-0.208779	0.093692	-2.228358	0.02585664	0.30360294
ENSG00000271971	1.008736	0.452708	2.228226	0.025865456	NA
TBC1D12	1.86663	0.837923	2.227688	0.025901352	NA
HMOX1	0.773206	0.347168	2.227183	0.025935055	0.303929723

FZR1	-0.307976	0.138315	-2.226627	0.025972226	0.303929723
PSMC1	-0.162944	0.073183	-2.226525	0.025979055	0.303929723
ENSG00000264443	0.55707	0.250298	2.225628	0.026039126	0.304052359
SLC16A1.AS1	0.323992	0.145591	2.225362	0.026056928	0.304052359
ENSG00000279927	-1.17333	0.527316	-2.2251	0.026074504	NA
QTRT1	-0.330967	0.148752	-2.224956	0.02608416	0.304052359
ZNF146	0.257077	0.115677	2.222358	0.026259113	0.305722036
FGL2	-1.169023	0.526216	-2.221564	0.026312777	NA
NUP43	0.386396	0.173931	2.221554	0.026313463	0.305985267
VSIR	-0.65088	0.293198	-2.219932	0.02642335	0.306892888
ZNF322	0.314464	0.141729	2.218777	0.026501868	0.306992141
CDK5R1	1.98572	0.895079	2.218486	0.026521736	NA
CEP85L	0.354008	0.159591	2.218223	0.026539625	0.306992141
UBA6	0.27765	0.125176	2.218071	0.026550012	0.306992141
EPB41L2	-0.423495	0.190945	-2.217894	0.026562064	0.306992141
FCGRT	-0.291407	0.131419	-2.217387	0.02659667	0.306992141
DR1	0.200082	0.090249	2.217002	0.02662297	0.306992141
RIPK2	0.267373	0.120661	2.215909	0.026697751	0.307279515
ENSG00000261087	0.643231	0.290305	2.215706	0.026711642	0.307279515
ENSG00000274213	-0.783953	0.354057	-2.214202	0.026814854	NA
ZHX2	-0.279018	0.126014	-2.214187	0.026815924	0.308010351
DYNC1LI2	0.244152	0.110284	2.21385	0.026839076	0.308010351
PELI3	-0.708255	0.320038	-2.213031	0.026895536	NA
CHD1	0.194021	0.087686	2.212681	0.026919675	0.308432203
MRPL18	0.201664	0.091161	2.212181	0.026954134	0.308432203
EP300	0.207195	0.093674	2.211869	0.026975696	0.308432203
TMEM140	0.408961	0.184955	2.211138	0.027026285	0.308432203
PHF13	0.495761	0.224225	2.211	0.02703581	0.308432203
GTF2IRD2B	-0.433868	0.196447	-2.208572	0.027204417	0.309988868
CFAP73	-1.35794	0.61562	-2.205808	0.027397455	NA
RPL36	0.12709	0.057622	2.205589	0.027412797	0.311693955
CLMN	0.486069	0.220389	2.205504	0.027418721	0.311693955
WDR11	-0.220339	0.099999	-2.203412	0.027565724	0.312995972
AVP	-2.254481	1.023476	-2.202769	0.027611059	NA
KARS	-0.192369	0.087337	-2.202609	0.027622348	0.313089243
ENSG00000125726	-0.921826	0.41856	-2.202374	0.027638895	0.313089243
FBXL8	-0.822997	0.374051	-2.200226	0.027790871	NA
ZXDA	-0.710118	0.322774	-2.200046	0.027803609	NA
TWISTNB	-0.328444	0.149301	-2.199883	0.027815221	0.313917916
HMGB3	-0.51531	0.234346	-2.198929	0.027882968	0.313917916
SPHK1	0.716971	0.326061	2.198887	0.027885941	0.313917916
RBX1	0.168303	0.076554	2.198503	0.027913296	0.313917916
HDHD3	0.239255	0.108846	2.198098	0.027942145	0.313917916
MAVS	0.230229	0.10474	2.198098	0.02794213	0.313917916
MYO1F	0.374427	0.170359	2.197878	0.027957799	0.313917916
DLGAP1.AS2	0.671586	0.30559	2.197671	0.027972561	0.313917916
C1GALT1	0.259914	0.118321	2.196696	0.028042178	0.31411531

CD55	-0.265692	0.120961	-2.196512	0.02805532	0.31411531
MINK1	-0.430959	0.196483	-2.193367	0.028280921	0.316273868
CBLN3	-0.451568	0.20615	-2.190483	0.028489202	0.318233959
MEGF11	-0.888699	0.405771	-2.190146	0.028513638	NA
MED6	-0.208103	0.095027	-2.18993	0.028529331	0.318313373
C8orf82	-0.709761	0.324131	-2.189734	0.02854355	NA
PPP2R5A	0.396011	0.180896	2.189162	0.028585067	0.318566531
SUGCT	-2.539144	1.160063	-2.188799	0.028611477	NA
TNFRSF21	-2.074996	0.948378	-2.187941	0.028673934	NA
WASF1	-0.432681	0.197836	-2.187068	0.028737524	0.319895764
DIRAS1	-0.545263	0.249381	-2.186463	0.028781761	0.320018658
MGST3	0.351708	0.160893	2.185976	0.028817394	0.320045709
DUSP22	-0.187738	0.085928	-2.184838	0.028900762	0.320236933
SRPRA	-0.274886	0.125815	-2.184834	0.028901051	0.320236933
CYTH3	-0.634104	0.290363	-2.183833	0.028974494	0.320682115
TTC9	0.34512	0.158134	2.182453	0.029076103	0.321102803
P4HB	-0.16099	0.073767	-2.182412	0.029079123	0.321102803
PCED1B	0.531251	0.243586	2.180963	0.029186142	0.321222433
JAK3	-0.310337	0.1423	-2.180868	0.0291932	0.321222433
ASPSCR1	-0.314458	0.144194	-2.1808	0.029198206	0.321222433
NDUFC1	0.190264	0.087258	2.180462	0.029223244	0.321222433
ZNF362	-0.584645	0.268197	-2.179907	0.029264386	0.321308292
IFI27L2	0.235215	0.107977	2.178392	0.029376845	0.322081135
HINFP	0.265841	0.122054	2.17806	0.029401597	0.322081135
HSPD1	0.219025	0.10068	2.175448	0.029596526	0.323536399
PHF12	0.28942	0.133055	2.175191	0.029615818	0.323536399
ZNF614	0.494253	0.22725	2.174933	0.029635129	0.323536399
RHOBTB1	-1.194767	0.549635	-2.173745	0.029724322	NA
NDUFB3	0.168528	0.077612	2.17142	0.029899459	0.326052921
MCM6	0.604955	0.278714	2.170522	0.029967317	0.326179362
RUVBL2	-0.281551	0.129748	-2.169989	0.030007679	0.326179362
CTDP1	-0.645131	0.297306	-2.169925	0.030012562	0.326179362
ENSG00000261386	0.491195	0.226496	2.168673	0.030107496	0.326842631
MDM2	-0.253873	0.117131	-2.167434	0.030201805	0.327497638
KLHDC2	-0.202737	0.093572	-2.166656	0.030261119	0.327772116
APH1A	-0.123338	0.056942	-2.166041	0.030308019	0.327911672
SNHG25	-0.327898	0.151433	-2.165305	0.030364299	0.328022832
CASP10	0.495096	0.228686	2.164966	0.030390327	0.328022832
FBXO44	-0.301516	0.139297	-2.16455	0.030422141	0.328022832
ZNF43	-0.290612	0.134324	-2.163521	0.03050115	0.328022832
UQCRH	0.142912	0.06606	2.163371	0.030512623	0.328022832
LENG8	-0.232076	0.107282	-2.163243	0.030522457	0.328022832
JHY	-0.639168	0.295508	-2.162944	0.030545483	NA
RMDN2	-0.496016	0.229383	-2.162396	0.030587646	0.328165501
WDR81	-0.416668	0.192707	-2.162186	0.030603816	0.328165501
TSTD1	-0.233083	0.107843	-2.161306	0.030671698	0.328500971
ENSG00000268352	1.133751	0.524806	2.160325	0.030747489	NA

WNT10A	-0.564208	0.261191	-2.160139	0.030761908	0.328500971
SEMA4F	0.688794	0.318923	2.159748	0.030792158	0.328500971
MIR22HG	0.440212	0.203829	2.159712	0.030794943	0.328500971
PDCD6IP	-0.17373	0.080459	-2.159241	0.03083147	0.328500971
ENSG00000262873	-1.359977	0.629854	-2.159193	0.030835195	NA
PLAGL1	-0.351309	0.162708	-2.159137	0.030839562	0.328500971
SLC38A11	1.453132	0.673026	2.159102	0.030842251	NA
NDUFB5	0.151574	0.070255	2.157489	0.03096756	0.328995326
PTPRA	-0.498999	0.231299	-2.157376	0.030976378	0.328995326
COG3	-0.415359	0.192543	-2.157222	0.030988356	0.328995326
BMPR2	0.311729	0.144741	2.153704	0.031263397	0.331550222
PNPLA4	0.467974	0.217361	2.152976	0.031320598	0.331791826
RBL1	0.421783	0.196139	2.150429	0.031521267	0.33250536
N6AMT1	0.510091	0.2373	2.149556	0.031590336	0.33250536
NUDCD3	-0.2412	0.112228	-2.149203	0.031618341	0.33250536
ENSG00000131408	0.148525	0.069108	2.149173	0.031620649	0.33250536
DUSP11	0.166521	0.077488	2.149009	0.031633695	0.33250536
BABAM2	-0.322384	0.150029	-2.148817	0.031648902	0.33250536
TMEM123	-0.229511	0.106841	-2.14816	0.03170103	0.33250536
GRAMD1A	-0.472477	0.219955	-2.148063	0.031708723	0.33250536
VPS16	-0.322287	0.150055	-2.1478	0.03172965	0.33250536
SCYL2	-0.287665	0.133949	-2.147576	0.031747423	0.33250536
GCSAM	-0.569786	0.265347	-2.147326	0.031767369	0.33250536
ENSG00000260572	-1.731671	0.80655	-2.147011	0.03179242	NA
ZFAND2A	-0.390162	0.181784	-2.146296	0.03184935	0.333001879
C3orf52	-0.719095	0.33516	-2.145525	0.031910913	NA
ACAT1	-0.36852	0.171796	-2.145104	0.031944554	0.333631524
SWAP70	0.181967	0.084846	2.144676	0.031978789	0.333631524
CCL4	-0.87465	0.408022	-2.143635	0.032062137	0.334139457
CHAF1A	0.583891	0.272439	2.143197	0.032097269	0.334144359
KIAA1324L	0.863807	0.403119	2.142809	0.032128464	NA
PEA15	-0.33333	0.155612	-2.142062	0.032188525	0.334478349
IL13RA1	-0.51055	0.238359	-2.141935	0.032198746	0.334478349
ENSG00000274184	0.486035	0.226965	2.141451	0.032237693	0.334511165
BRF2	-0.431648	0.201607	-2.141034	0.032271305	0.334511165
RIF1	0.230304	0.107631	2.139765	0.032373768	0.335133245
APMAP	-0.327311	0.15299	-2.13943	0.032400849	0.335133245
CROCC	-0.620521	0.290353	-2.137126	0.032587737	NA
TOMM20	0.17154	0.080294	2.136408	0.032646168	0.337308745
TLR4	-1.950021	0.912977	-2.135893	0.032688167	NA
NFE2L2	0.202254	0.094742	2.134799	0.032777432	0.338093876
AKIRIN2	0.224006	0.10494	2.134617	0.0327923	0.338093876
IGHG3	1.169622	0.548233	2.133439	0.032888704	0.338725544
ENSG00000268205	0.2713	0.127204	2.132797	0.032941375	0.338905931
IRGQ	0.496552	0.233017	2.13097	0.033091582	0.340088324
RIMKLB	-0.321313	0.15092	-2.12903	0.033251785	0.340730747
NOL10	-0.257202	0.12083	-2.128635	0.033284506	0.340730747

CLN5	-0.275445	0.129401	-2.128615	0.033286093	0.340730747
STRBP	-0.151441	0.071162	-2.128112	0.03332782	0.340730747
AGO1	-0.224371	0.105435	-2.128053	0.033332659	0.340730747
ADGRG5	1.028748	0.483504	2.127691	0.033362678	NA
ZNHIT3	0.180843	0.084996	2.127649	0.033366164	0.340730747
ITPR3	0.469352	0.220695	2.126699	0.033445117	0.341066389
NR1D2	0.316516	0.14885	2.126402	0.033469793	0.341066389
MT.ATP8	-0.431748	0.203106	-2.125728	0.033525947	0.341277851
CCDC82	0.232357	0.109361	2.124671	0.033614068	0.34135726
PCYOX1	0.328305	0.154531	2.124524	0.033626334	0.34135726
ISG15	0.229931	0.108236	2.124354	0.033640547	0.34135726
ITGA6.AS1	-0.495875	0.233503	-2.123634	0.033700764	0.34135726
EML3	-0.272171	0.128189	-2.123198	0.03373728	0.34135726
HMOX2	0.255038	0.120126	2.123091	0.03374621	0.34135726
ENSG00000237499	-0.523609	0.246705	-2.122407	0.033803606	0.341579415
IFT22	-0.244022	0.115025	-2.121464	0.033882738	0.34165741
C12orf49	-0.490729	0.231354	-2.121118	0.03391191	0.34165741
DCXR	-0.247531	0.116712	-2.120883	0.033931666	0.34165741
SRD5A1	-0.32284	0.15227	-2.120187	0.033990239	0.34165741
AGPAT4	-0.753415	0.355384	-2.120002	0.034005842	NA
ADAL	-0.285006	0.134447	-2.119837	0.034019815	0.34165741
ZEB1.AS1	-0.463689	0.21878	-2.119433	0.034053864	0.34165741
COMMD2	-0.171732	0.08103	-2.119368	0.034059416	0.34165741
ANKS6	-1.068145	0.504234	-2.118349	0.034145514	NA
PPIL3	0.36322	0.171525	2.117598	0.034209148	0.342802688
LYAR	0.418968	0.198021	2.115777	0.034363801	0.343994852
ZFAS1	0.246542	0.11655	2.115331	0.034401704	0.344017042
HTR3A	-0.790885	0.374104	-2.114077	0.034508678	NA
SH3RF1	-1.817151	0.860022	-2.112912	0.034608307	NA
ENSG00000251661	-1.520032	0.720442	-2.10986	0.034870382	NA
UTP6	0.232646	0.11027	2.109787	0.034876721	0.348037981
SNX2	-0.20963	0.09937	-2.109595	0.034893258	0.348037981
OTUD6B	-0.399278	0.189287	-2.109376	0.034912109	0.348037981
FGFBP2	-2.663971	1.263113	-2.109052	0.034940057	NA
MED18	0.435071	0.206392	2.107983	0.03503243	0.348876681
ZNF33A	0.182803	0.086747	2.107303	0.035091355	0.349102852
GLMN	-0.323939	0.153794	-2.106321	0.035176519	0.349589326
SPRYD7	0.368192	0.175008	2.103862	0.035390477	0.351353448
CD52	0.245508	0.116819	2.101615	0.035586984	0.352940869
TBCA	0.153523	0.073072	2.100996	0.035641284	0.353116116
AEBP1	-0.441841	0.210384	-2.100163	0.035714506	0.353354196
SVBP	-0.154407	0.073531	-2.099889	0.035738625	0.353354196
CD3D	-2.240022	1.067439	-2.098502	0.035860853	NA
GTF2E2	0.226854	0.108118	2.098203	0.035887191	0.354459552
RHEBL1	0.399921	0.190718	2.096922	0.036000449	0.355023388
CETN3	0.264037	0.125928	2.096725	0.036017933	0.355023388
FOXN2	0.368216	0.175699	2.095717	0.036107271	0.355540438

DNAAF2	0.221248	0.105684	2.093499	0.036304609	0.357118805
ZBTB11.AS1	-0.541334	0.258638	-2.093017	0.036347644	0.357177668
GNMT	-1.446775	0.691298	-2.092838	0.036363628	NA
ABCG1	-0.606045	0.289621	-2.092546	0.036389738	0.357227161
FXYD7	0.551685	0.263748	2.091715	0.036464013	0.357248238
LINC02193	-0.657285	0.314268	-2.091475	0.036485482	0.357248238
VPS52	-0.440399	0.210588	-2.091279	0.036503062	0.357248238
LAMP1	-0.366507	0.175348	-2.090168	0.03660274	0.357860457
IRAK3	0.712035	0.340891	2.088747	0.036730473	NA
CD40	-0.1748	0.083698	-2.088466	0.036755841	0.358212414
GATD3B	-1.252563	0.599814	-2.088254	0.036774911	NA
XRN2	-0.174252	0.083469	-2.087616	0.036832523	0.358212414
PLEKHG1	-0.259257	0.124216	-2.08715	0.036874572	0.358212414
IKBKE	-0.516027	0.247294	-2.0867	0.036915298	0.358212414
SIPA1L3	-0.240402	0.115209	-2.086669	0.036918073	0.358212414
SLC35D1	0.494818	0.237195	2.086121	0.036967638	0.358212414
DNAJC18	-0.395655	0.18967	-2.086022	0.036976639	0.358212414
DEGS2	0.94486	0.452961	2.085963	0.036981996	NA
AVPI1	0.753446	0.361215	2.085866	0.036990784	NA
GART	-0.30725	0.14733	-2.085453	0.037028244	0.358212414
PIK3CD.AS2	0.947144	0.454213	2.085243	0.03704727	NA
FAM177B	0.453161	0.217371	2.08473	0.037093774	0.358212414
ARMH3	-0.371897	0.178398	-2.084653	0.037100808	0.358212414
METTL8	0.461096	0.221219	2.084344	0.037128857	0.358212414
BORCS6	0.242596	0.116396	2.084222	0.037139943	0.358212414
RTL8C	-0.312748	0.150065	-2.084081	0.037152811	0.358212414
SEC61A2	-0.402739	0.193252	-2.084013	0.037158964	0.358212414
SMAD5	0.25151	0.12083	2.081522	0.03738613	0.359651966
EXOG	-0.243059	0.116775	-2.081437	0.037393882	0.359651966
LYSMD3	0.333254	0.160145	2.080952	0.037438271	0.359651966
LDLRAP1	0.360362	0.173189	2.080742	0.037457528	0.359651966
DOK7	1.245203	0.598643	2.080041	0.037521788	NA
FAIM	0.346979	0.166828	2.079861	0.037538319	0.360010554
ORMDL3	-0.3354	0.161359	-2.078599	0.037654234	0.360010554
MAP3K5	0.489627	0.235604	2.078181	0.037692693	0.360010554
ZFAND6	0.253796	0.122136	2.077986	0.03771064	0.360010554
IRF2BP2	-0.269366	0.129639	-2.077817	0.037726179	0.360010554
COL19A1	-0.341599	0.16442	-2.077597	0.03774651	0.360010554
TBC1D20	0.240144	0.115609	2.077212	0.037781985	0.360010554
DYNLT3	0.218709	0.105315	2.076712	0.037828174	0.360010554
PEX6	0.593562	0.285877	2.076283	0.03786779	0.360010554
ZNF100	-0.424713	0.204555	-2.076277	0.03786833	0.360010554
WIPF2	0.227145	0.109456	2.07521	0.037967092	0.360292921
NUP50	0.22539	0.108614	2.075148	0.037972781	0.360292921
TNRC6B	0.126742	0.061135	2.073168	0.038156681	0.361302189
RGS14	0.317448	0.153147	2.072823	0.038188714	0.361302189
EMC1	0.374244	0.180564	2.07264	0.038205824	0.361302189

LPCAT4	0.26755	0.129102	2.07239	0.03822907	0.361302189
CGAS	0.255697	0.123428	2.071625	0.038300421	0.361621999
ZKSCAN3	0.668229	0.322599	2.071392	0.03832219	NA
PHOSPHO2	0.40115	0.193859	2.069292	0.038518716	0.363327221
ENSG00000258017	0.776977	0.375602	2.068618	0.03858198	NA
STRIP1	-0.316818	0.153232	-2.067578	0.038679683	0.364360472
PIP4K2A	0.372118	0.180022	2.067066	0.038727923	0.364360472
LINC00663	0.66765	0.323035	2.066801	0.038752876	NA
BMT2	-0.404984	0.195966	-2.066604	0.038771443	0.364360472
UBE2E1	0.211873	0.102527	2.06652	0.038779444	0.364360472
ENSG00000080031	-1.663115	0.805292	-2.065232	0.038901086	NA
ATG16L2	-0.219452	0.10626	-2.065228	0.038901413	0.365150558
TMED4	-0.203024	0.098333	-2.064656	0.038955546	0.365302981
ZNF79	0.76227	0.36948	2.063088	0.039104261	NA
NFIX	0.439114	0.212898	2.062556	0.039154826	0.366814894
HIST1H2BJ	0.498825	0.241957	2.061628	0.039243172	0.36728561
HIST1H3H	1.261566	0.6121	2.061046	0.039298658	NA
PPL	1.098883	0.533565	2.059513	0.039445164	NA
DGCR6	-0.997567	0.484372	-2.059506	0.039445775	NA
SYNGAP1.AS1	0.675878	0.328217	2.059242	0.039471051	NA
ZNF627	-0.497227	0.241558	-2.058417	0.039550093	0.369434699
BCAT1	-1.482462	0.720279	-2.058178	0.03957308	NA
MRPS18B	0.145754	0.070831	2.057783	0.039610934	0.369434699
ABHD6	0.46755	0.227253	2.057397	0.039648092	0.369434699
CARD11	0.365937	0.177865	2.057381	0.039649632	0.369434699
PITPNA	0.332484	0.161618	2.057227	0.03966441	0.369434699
CAPN12	0.400382	0.194839	2.054935	0.039885236	0.371132893
ITGA10	0.549348	0.267499	2.053647	0.040009909	0.371605004
GGCT	0.24197	0.117839	2.053399	0.040033856	0.371605004
ARID5B	-0.195328	0.095133	-2.053216	0.040051618	0.371605004
CALHM2	0.618803	0.301454	2.052729	0.040098858	0.37168557
PNO1	-0.33366	0.162622	-2.05175	0.040193924	0.37211293
LSM10	0.14234	0.069385	2.05146	0.040222165	0.37211293
R3HDM2	-0.327076	0.159471	-2.050999	0.040267061	0.372171115
DGLUCY	0.247904	0.120963	2.049412	0.040421816	0.373243592
AIDA	0.216109	0.105555	2.047354	0.040623352	0.374422661
GMNN	0.481236	0.235087	2.047059	0.040652316	0.374422661
TRIB3	0.409721	0.200165	2.046919	0.04066603	0.374422661
KLF12	0.294009	0.143787	2.044753	0.040879179	0.376026033
ZKSCAN2	-0.60906	0.29805	-2.043483	0.041004684	NA
ZNRD2	-0.206959	0.101301	-2.04301	0.041051476	0.377250934
IGHV3.15	-1.016542	0.498042	-2.041075	0.041243408	NA
MXD1	0.425268	0.208379	2.040839	0.041266851	0.378516777
ARL2	0.328917	0.161168	2.04083	0.041267752	0.378516777
AMIGO2	0.920519	0.451139	2.040434	0.041307098	NA
ATP5PD	-0.127802	0.062642	-2.040192	0.041331187	0.378738257
CFP	-1.647387	0.807652	-2.039722	0.041377992	NA

MTPN	0.156917	0.076933	2.039661	0.041384063	0.378862645
STYX	0.296963	0.145687	2.038365	0.041513474	0.379441949
STX5	-0.182042	0.089313	-2.038239	0.041526064	0.379441949
MRPS21	-0.156072	0.076612	-2.037184	0.041631624	0.380046262
ACTR1B	-0.193178	0.094873	-2.036171	0.041733165	0.380330394
GSE1	0.447144	0.219673	2.035502	0.041800339	0.380330394
VPS25	-0.240741	0.118278	-2.035375	0.041813116	0.380330394
PLA2G6	-0.463567	0.227767	-2.035266	0.041824122	0.380330394
PLOD3	0.334642	0.16445	2.034909	0.041860015	0.380330394
TMEM167B	-0.189311	0.093065	-2.034171	0.041934349	0.380647006
TNFSF10	0.3564	0.175266	2.033483	0.042003761	0.380818631
ARF5	-0.242767	0.119403	-2.033167	0.042035656	0.380818631
CLTC	0.233343	0.114788	2.03281	0.042071768	0.380818631
POGLUT3	0.900104	0.442831	2.032614	0.042091527	NA
AKIRIN1	0.164488	0.080935	2.032359	0.042117346	0.380873559
MYL6B	-0.211233	0.104042	-2.030265	0.042329592	0.381482826
SRSF2	-0.121404	0.059802	-2.030084	0.042348022	0.381482826
MAX	0.176452	0.086925	2.029932	0.042363455	0.381482826
SF3B2	-0.13961	0.068779	-2.029824	0.042374406	0.381482826
GABPB2	0.344738	0.169843	2.029739	0.042383084	0.381482826
LINC00954	-0.326522	0.1609	-2.029355	0.042422157	0.381482826
SEPHS1	0.416583	0.205362	2.028534	0.042505733	0.381878162
SLFN12	0.724823	0.357548	2.027205	0.042641422	NA
POLM	-0.221996	0.109524	-2.02691	0.042671652	0.382657319
ENSG00000245869	0.466044	0.229949	2.026726	0.042690466	0.382657319
BAD	-0.192507	0.095003	-2.026336	0.042730315	0.382657319
MAP3K2	0.206896	0.102114	2.026132	0.042751238	0.382657319
AKAP10	0.196495	0.097061	2.024448	0.042924079	0.383669117
VCPIP1	0.260831	0.128853	2.024255	0.042943877	0.383669117
ENSG00000257194	-1.412965	0.698482	-2.02291	0.043082435	NA
NKG7	-1.799232	0.889561	-2.022607	0.043113729	NA
ERLIN1	0.506621	0.25053	2.022192	0.043156495	0.384793427
RIPOR1	-0.190794	0.094387	-2.021403	0.043238086	0.384793427
SQOR	-0.257544	0.127419	-2.021239	0.043254988	0.384793427
FAM71D	-0.598012	0.295874	-2.021173	0.04326183	0.384793427
KCTD7	0.317491	0.157088	2.021101	0.043269302	0.384793427
MYLK.AS1	-0.541303	0.267974	-2.019986	0.043384825	0.385465171
ESYT2	0.434154	0.214972	2.019587	0.043426248	0.385477927
ACSM3	-0.410769	0.203452	-2.019001	0.043487097	0.385662937
MIR4435.2HG	1.141831	0.565865	2.017851	0.043606798	NA
ENSG00000273002	0.554073	0.274661	2.017296	0.043664591	0.385952631
HLA.DMA	-0.262497	0.130157	-2.016771	0.043719396	0.385952631
UBL4A	0.219545	0.10886	2.016768	0.043719686	0.385952631
FAM8A1	0.571606	0.283427	2.016765	0.043720075	NA
IDH1	0.257305	0.127583	2.016764	0.043720115	0.385952631
SEMA4A	-1.040722	0.516097	-2.016523	0.04374532	NA
TIPARP	-0.233262	0.115711	-2.015911	0.043809267	0.385952631

PCF11	0.162865	0.080813	2.015338	0.043869231	0.385952631
PWWP2B	0.59519	0.295333	2.015314	0.043871735	0.385952631
VPS37B	0.196684	0.097596	2.015282	0.043875119	0.385952631
ANXA6	-0.241566	0.11987	-2.015234	0.043880092	0.385952631
CISD2	0.213511	0.106075	2.012819	0.044133717	0.387829566
TCOF1	-0.240661	0.11963	-2.011711	0.044250368	0.388500498
EIF2B5	-0.226047	0.11243	-2.010552	0.044372759	0.389220558
MBOAT2	-1.046021	0.520485	-2.009706	0.044462273	NA
FUOM	-0.390634	0.194461	-2.008805	0.044557868	0.39048895
SAMD10	0.579118	0.288325	2.008563	0.044583475	NA
DRG2	-0.230472	0.114765	-2.008201	0.044621947	0.390570353
PPP2R5B	0.255491	0.127239	2.007954	0.044648188	0.390570353
PLXNC1	0.813193	0.405154	2.007121	0.044736832	NA
DAZAP1	-0.275083	0.137084	-2.006673	0.044784472	0.391057623
ENSG00000186019	0.950143	0.473598	2.006221	0.044832631	NA
ZNF417	0.545855	0.272149	2.005721	0.044886006	0.391057623
MORC3	0.207808	0.103612	2.005637	0.044895037	0.391057623
SRSF5	-0.182219	0.090872	-2.005221	0.044939414	0.391057623
TCEAL4	0.243137	0.121256	2.005151	0.044946876	0.391057623
SYNE2	-0.288137	0.143706	-2.005046	0.044958121	0.391057623
EVI2A	0.314154	0.156703	2.004768	0.044987853	0.391057623
RPSA	0.221315	0.110418	2.004343	0.045033306	0.391100066
ENSG00000236723	1.027524	0.512734	2.004011	0.045068887	NA
KMT2A	0.197977	0.098835	2.003109	0.04516557	0.391782285
NINJ1	-0.435747	0.217563	-2.002852	0.045193143	0.391782285
GLB1L	0.582102	0.290667	2.002641	0.045215866	NA
LAGE3	0.177089	0.088453	2.00206	0.045278325	0.392168059
AP1B1	-0.344892	0.172356	-2.001046	0.045387436	0.392760215
ERGIC1	-0.206242	0.103088	-2.000637	0.045431563	0.392789476
ECI2	0.220699	0.110356	1.999881	0.04551311	0.393141917
ENSG00000204802	-0.710427	0.355302	-1.9995	0.045554241	NA
LINC00667	0.222098	0.111156	1.998078	0.045708153	0.394473224
ENSG00000271869	0.358652	0.179622	1.996708	0.045856938	0.39540329
MAMLD1	-1.021688	0.512056	-1.995268	0.046013712	NA
ENSG00000256690	0.353092	0.17697	1.99521	0.046019959	0.395906872
ERO1A	0.310329	0.155565	1.994857	0.04605852	0.395906872
CXCR5	0.196094	0.098302	1.994816	0.046062988	0.395906872
ZEB1	-0.270537	0.13563	-1.994663	0.046079617	0.395906872
LRMP	0.271657	0.136286	1.993292	0.046229507	0.396640625
WASIR2	0.660196	0.331288	1.992814	0.046281782	0.396640625
CENPS.CORT	-0.998298	0.500956	-1.992785	0.046285048	NA
DPP3	-0.353491	0.177388	-1.992753	0.046288455	0.396640625
NMNAT3	-1.011631	0.507856	-1.991966	0.046374821	NA
GTF3C6	-0.134324	0.067538	-1.988874	0.046715099	0.399940993
AGAP3	1.647152	0.828234	1.988752	0.046728623	NA
POLR2H	-0.232122	0.116739	-1.988388	0.046768755	0.400045075
IP6K2	-0.202639	0.102009	-1.986479	0.046980149	0.400082168

MGME1	0.254364	0.128072	1.986106	0.047021568	0.400082168
ETFRF1	0.236662	0.119213	1.985204	0.047121758	0.400082168
DHRS3	2.263718	1.14047	1.984899	0.047155739	0.400082168
PAFAH1B1	0.14299	0.07205	1.984584	0.04719075	0.400082168
REEP4	0.309959	0.156187	1.984538	0.047195865	0.400082168
GDI2	-0.168399	0.084872	-1.984151	0.047239025	0.400082168
RAB11B	-0.158072	0.079674	-1.983982	0.047257833	0.400082168
FAAP24	-0.493202	0.248631	-1.98367	0.04729261	0.400082168
IRF5	0.316422	0.159554	1.98316	0.04734954	0.400082168
UBE4B	0.241019	0.121543	1.982997	0.047367796	0.400082168
XRCC4	-0.353502	0.178274	-1.982915	0.047376963	0.400082168
PLD4	-0.587347	0.296209	-1.982881	0.047380699	0.400082168
CAMTA1	-0.163788	0.082613	-1.982591	0.047413125	0.400082168
C1orf35	0.193305	0.097503	1.982563	0.047416246	0.400082168
ZBTB1	0.210769	0.106321	1.982376	0.047437128	0.400082168
ENSG00000257354	-0.503089	0.253865	-1.981721	0.047510479	0.400350543
IL27RA	0.213954	0.108007	1.980931	0.04759901	0.40074625
PITPNM2	-0.709471	0.358294	-1.980136	0.047688288	NA
ITPR1.DT	1.349532	0.682147	1.97836	0.047888136	NA
XXYLT1.AS2	0.800203	0.404714	1.977208	0.048018106	0.403921939
PPTC7	0.2526	0.127788	1.976714	0.048073963	0.403953132
USP40	0.576646	0.291794	1.976207	0.048131339	0.403953132
MAD2L2	-0.181604	0.091902	-1.976064	0.048147526	0.403953132
NDUFS6	0.12979	0.065717	1.974962	0.048272432	0.404626884
LINC02285	-1.006182	0.509547	-1.97466	0.048306707	NA
EEA1	0.260421	0.131898	1.974411	0.048335067	0.404626884
HIST4H4	0.548887	0.278005	1.974378	0.048338828	NA
C22orf46	0.393635	0.199418	1.973917	0.048391143	0.404626884
TMEM179B	-0.174632	0.088485	-1.973572	0.048430462	0.404626884
ATG5	0.181015	0.091736	1.973217	0.04847086	0.404626884
UBE2W	0.19221	0.097413	1.973139	0.048479673	0.404626884
CETP	-0.812432	0.411995	-1.971945	0.048615901	NA
CCDC152	0.381457	0.193442	1.97194	0.048616476	0.405417674
RPS6KB2.AS1	-0.64505	0.327321	-1.970699	0.048758353	NA
ENSG00000256591	-0.716611	0.363722	-1.970219	0.048813312	NA
PATJ	0.373495	0.189573	1.970191	0.048816479	0.406391041
KANK2	-0.987851	0.5014	-1.970184	0.048817282	NA
FYN	0.415787	0.21104	1.970182	0.048817513	0.406391041
TOLLIP.AS1	-1.071128	0.543839	-1.969569	0.04888778	NA
C1orf216	0.509095	0.258611	1.968571	0.049002377	0.407578013
ENSG00000267939	-1.76805	0.898636	-1.967482	0.049127677	NA
HIST2H2BE	0.413122	0.209987	1.967367	0.049140874	0.408138074
RPAP2	0.210956	0.107238	1.967171	0.049163504	0.408138074
ENSG00000272870	0.628355	0.319451	1.966987	0.049184663	NA
SEC13	-0.255711	0.130008	-1.966883	0.049196726	0.408138074
FAHD2B	-0.853381	0.433929	-1.966638	0.049225008	NA
HCCS	0.248304	0.126303	1.965935	0.049306076	0.40826996

E2F6	0.32729	0.166504	1.965659	0.049338002	0.40826996
INO80	-0.271897	0.138327	-1.96561	0.049343716	0.40826996
DUSP18	0.323468	0.164592	1.965278	0.04938203	0.40826996
C7orf61	0.847245	0.431158	1.965044	0.049409114	NA
ORAI2	0.136245	0.069343	1.964798	0.049437568	0.408378883
LPCAT2	-1.153615	0.587204	-1.96459	0.049461711	NA
RMND1	-0.332451	0.169296	-1.96372	0.04956259	0.409061104
PIGC	-0.21394	0.109	-1.962748	0.049675521	0.409563033
TRIM52	0.274312	0.139779	1.962465	0.049708376	0.409563033
CLIC1	0.185901	0.0948	1.960976	0.049881821	0.410385651
ADH5	-0.19598	0.099945	-1.960877	0.049893359	0.410385651

Cluster 4	log2FC	lfcSE	stat	pvalue	padj
ARRDC3	-1.093058	0.159098	-6.870345	6.40E-12	3.13E-08
GPR183	0.96683	0.165013	5.859129	4.65E-09	8.55E-06
C7orf50	-0.510511	0.087428	-5.839218	5.24E-09	8.55E-06
SMIM10	-3.760857	0.646237	-5.819627	5.90E-09	NA
MT1X	-1.838662	0.323822	-5.678001	1.36E-08	NA
SYNGR2	-0.76872	0.138034	-5.569053	2.56E-08	3.13E-05
CLK1	0.558842	0.104823	5.331317	9.75E-08	9.54E-05
ENSG00000273319	0.725078	0.139862	5.184235	2.17E-07	0.000176885
LRIF1	0.552042	0.10815	5.104394	3.32E-07	0.000231967
ZBTB1	0.638495	0.127926	4.991123	6.00E-07	0.000332472
CYTIP	0.267219	0.053577	4.987538	6.12E-07	0.000332472
CD83	-0.470277	0.095109	-4.94463	7.63E-07	0.00037328
NFKBIA	-0.499838	0.101575	-4.920864	8.62E-07	0.000383268
CKS2	1.120976	0.229744	4.879249	1.06E-06	0.00041957
ZC3H6	0.698181	0.143357	4.87022	1.11E-06	0.00041957
CDKN1A	-0.787616	0.165683	-4.753741	2.00E-06	0.000697906
GATD3	4.371743	0.928638	4.707696	2.51E-06	NA
CHMP6	-0.771983	0.165038	-4.677617	2.90E-06	NA
RPS5	0.328483	0.071666	4.583509	4.57E-06	0.001455455
CRIPT	0.523886	0.114507	4.575127	4.76E-06	0.001455455
CHORDC1	0.772773	0.171623	4.502747	6.71E-06	0.001930744
DBI	-0.329261	0.073967	-4.45147	8.53E-06	0.002293783
RPL8	0.467311	0.1052	4.442137	8.91E-06	0.002293783
PDE4B	0.388308	0.088625	4.381483	1.18E-05	0.002668076
PNPLA8	0.483716	0.110466	4.378877	1.19E-05	0.002668076
IZUMO4	-0.787927	0.179988	-4.377656	1.20E-05	0.002668076
IL16	0.325289	0.075833	4.289552	1.79E-05	0.003719356
H3F3B	-0.381837	0.089241	-4.278705	1.88E-05	0.003719356
GPR65	0.735149	0.171913	4.276289	1.90E-05	0.003719356
TTC32	-0.737522	0.17261	-4.272771	1.93E-05	NA
HHEX	0.299358	0.070253	4.261117	2.03E-05	0.003827968
CXCR4	0.233225	0.054846	4.252374	2.12E-05	0.003833151
MRPL54	-0.374501	0.088664	-4.223842	2.40E-05	0.004197022
TCTN1	-0.818617	0.194231	-4.214647	2.50E-05	0.004220955
TAGLN2	-0.352291	0.084397	-4.174201	2.99E-05	0.004877244
CD5	-1.539502	0.370699	-4.152975	3.28E-05	NA
ENSG00000272211	-1.215828	0.293575	-4.141449	3.45E-05	0.005447301
CRK	0.524754	0.12822	4.092613	4.27E-05	0.006377082
ZC3H12D	-0.885557	0.216481	-4.09069	4.30E-05	0.006377082
RPS9	0.367874	0.090905	4.046809	5.19E-05	0.007471984
TMEM243	0.368468	0.091278	4.036785	5.42E-05	0.007575573
HHIP.AS1	-2.757367	0.684093	-4.030691	5.56E-05	NA
SGK1	-1.312415	0.326232	-4.022949	5.75E-05	NA
NPM1	0.251972	0.062941	4.00333	6.25E-05	0.008488956
RHOH	0.313857	0.078709	3.987579	6.68E-05	0.008827378
ASF1A	0.516179	0.130493	3.955596	7.63E-05	0.009667011

CIRBP	-0.221754	0.056092	-3.95339	7.71E-05	0.009667011
CD79B	-0.267773	0.068073	-3.933594	8.37E-05	0.009909599
RPS23	0.201496	0.051273	3.929904	8.50E-05	0.009909599
LETMD1	0.396342	0.100899	3.928091	8.56E-05	0.009909599
RPL13A	0.189182	0.048211	3.924013	8.71E-05	0.009909599
SLC3A2	-0.466697	0.11978	-3.896275	9.77E-05	0.010862825
LINC01215	-0.476098	0.124651	-3.819456	0.00013375	0.014542706
LRRC61	-0.99072	0.259955	-3.811123	0.00013834	NA
RPL7A	0.183581	0.04829	3.801651	0.00014374	0.015044042
SNAI3	1.347743	0.3548	3.798596	0.00014552	NA
RPL30	0.208312	0.054928	3.79249	0.00014914	0.015044042
KDM4B	0.555616	0.146581	3.790499	0.00015035	0.015044042
CCDC191	-1.283915	0.338765	-3.789986	0.00015066	0.015044042
RPS8	0.262593	0.069939	3.754612	0.00017361	0.016989515
RSRP1	0.391818	0.105313	3.7205	0.00019883	0.019075832
ZBTB25	0.485386	0.1309	3.708074	0.00020884	0.019651197
ENSG00000261386	0.711354	0.19227	3.699765	0.0002158	0.019797206
CHCHD5	-0.40761	0.110331	-3.694435	0.00022038	0.019797206
TCL1B	-1.245556	0.337736	-3.687953	0.00022607	0.019797206
SLC50A1	-0.497085	0.134807	-3.687377	0.00022658	0.019797206
TNFRSF14	0.265524	0.072173	3.679004	0.00023415	0.020099615
KRTCAP2	-0.320078	0.088092	-3.633442	0.00027967	0.023153585
MIR29B2CHG	0.497632	0.136968	3.633198	0.00027993	0.023153585
FGFR1OP2	0.417302	0.114974	3.629547	0.00028392	0.023153585
RPL36	0.151161	0.041698	3.62516	0.00028878	0.023164186
ARPC3	-0.207215	0.057507	-3.603321	0.00031418	0.024794632
ZBED2	2.042977	0.571681	3.573633	0.00035206	NA
C16orf74	-0.494382	0.138619	-3.566491	0.00036179	0.028099316
ENSG00000261505	-0.912922	0.256074	-3.565066	0.00036377	NA
PPM1K	0.261894	0.074005	3.53886	0.00040186	0.030043256
PNRC2	0.280407	0.079258	3.5379	0.00040332	0.030043256
LPAR5	0.667879	0.188845	3.536646	0.00040524	0.030043256
C17orf49	-0.973275	0.276885	-3.515092	0.0004396	0.032104083
RPSA	0.302235	0.086137	3.508774	0.00045018	0.03239285
CD48	0.334635	0.095525	3.503137	0.00045981	0.032606736
NUP210	0.460743	0.132151	3.486482	0.00048942	0.033158573
PPP1CA	-0.169175	0.048537	-3.485508	0.0004912	0.033158573
RPL38	0.221852	0.063673	3.484257	0.00049351	0.033158573
CLEC2B	0.392925	0.112826	3.482572	0.00049662	0.033158573
RPL9	0.224606	0.064543	3.479965	0.00050148	0.033158573
PRICKLE1	0.71638	0.205928	3.478789	0.00050368	NA
FGR	-0.939853	0.270302	-3.477044	0.00050697	NA
TPI1	-0.232038	0.06684	-3.471533	0.0005175	0.033529992
RBM3	-0.34656	0.099878	-3.469823	0.0005208	0.033529992
EPS15	-0.591244	0.171341	-3.45069	0.00055916	0.035107659
RPL32	0.163843	0.047562	3.44486	0.00057136	0.035107659
ADA	-0.701694	0.203741	-3.444048	0.00057307	0.035107659

NDUFB5	0.297633	0.08643	3.443608	0.00057401	0.035107659
HMGB2	-0.377199	0.109819	-3.434741	0.00059312	0.035828879
PSMD8	-0.245944	0.071851	-3.422963	0.00061943	0.036727913
DYNLT1	0.503514	0.147166	3.421391	0.00062302	0.036727913
SCRN1	1.094996	0.320881	3.412465	0.00064378	NA
BLNK	0.269009	0.078995	3.405379	0.00066072	0.038262808
APH1A	-0.27726	0.081472	-3.40311	0.00066624	0.038262808
MCRIP1	-0.267807	0.078754	-3.400547	0.00067251	0.038262808
SCN3A	1.614904	0.475378	3.397093	0.00068106	NA
RPL3	0.191296	0.056317	3.39677	0.00068186	0.038348887
PFDN5	0.136899	0.040385	3.389816	0.0006994	0.038887962
GALNT2	-0.573459	0.169338	-3.386483	0.00070795	0.038921093
EIF4A2	0.271207	0.080172	3.382832	0.00071743	0.039003995
CLEC2D	-0.324356	0.096008	-3.378428	0.00072902	0.03919858
ENSG00000266844	-1.940438	0.576394	-3.366511	0.00076126	NA
NEIL1	-0.495229	0.147137	-3.365767	0.00076331	0.040582092
RPL24	0.224521	0.066764	3.362882	0.00077133	0.040582092
CELF1	0.264683	0.078986	3.350993	0.00080522	0.041914451
RNF43	-1.01973	0.30488	-3.344693	0.00082374	NA
CYB5A	-0.795223	0.237863	-3.343194	0.0008282	0.042656694
FAU	0.163075	0.04884	3.33896	0.00084093	0.042860988
ODF3B	-0.700625	0.210202	-3.333111	0.00085881	NA
TAGAP	-0.414947	0.124867	-3.323112	0.00089019	0.044904126
HHLA3	0.773717	0.232877	3.322436	0.00089235	NA
GPR18	0.607875	0.183046	3.320887	0.00089732	NA
CDKN1B	0.288132	0.087012	3.311391	0.00092833	0.046212037
NUFIP2	0.362653	0.109583	3.309386	0.00093501	0.046212037
WRB	0.554147	0.167931	3.299844	0.00096739	NA
ARL17A	-0.642947	0.195193	-3.293904	0.00098806	0.048345877
FXYD2	-1.132432	0.344316	-3.288932	0.00100568	NA
RMND5A	0.539333	0.164063	3.287352	0.00101134	0.048403941
RNF34	0.500672	0.152344	3.286454	0.00101457	0.048403941
TRAF4	-0.361903	0.11016	-3.285248	0.00101893	0.048403941
SRSF5	-0.214314	0.06541	-3.276474	0.00105112	0.049453151
PTPMT1	-0.326816	0.099849	-3.273094	0.00106377	0.049499404
TRMT13	0.40174	0.122825	3.270827	0.00107234	0.049499404
DNAJB4	0.795668	0.243279	3.270596	0.00107321	NA
ZNF250	0.744533	0.227668	3.27026	0.00107449	NA
LINC02201	-1.671062	0.511111	-3.269472	0.00107749	NA
ORMDL3	-0.657683	0.201815	-3.258837	0.0011187	0.051156917
SNX15	-1.439564	0.443814	-3.243623	0.0011802	NA
RHOC	-0.671051	0.207118	-3.239944	0.00119553	NA
CDC42	-0.274011	0.084799	-3.231305	0.00123226	0.055828425
ENSG00000256092	-0.622792	0.192845	-3.2295	0.00124007	NA
TNFSF10	0.750956	0.233104	3.22155	0.00127499	0.057234314
LCN8	3.047177	0.951215	3.203457	0.00135788	0.060094407
H2AFZ	0.285445	0.089137	3.202317	0.00136327	0.060094407

TSPAN33	-0.533479	0.166663	-3.200951	0.00136975	NA
ACADVL	-0.283078	0.088489	-3.199017	0.00137897	0.060240479
RPS28	0.194913	0.060978	3.196469	0.00139121	0.060240479
PLAAT3	-0.879527	0.275932	-3.187479	0.00143519	NA
RPS6	0.198205	0.062226	3.185257	0.00144626	0.062074775
MAML3	-1.127562	0.354737	-3.178587	0.00147995	NA
IGLV6.57	-1.292565	0.406772	-3.177617	0.00148491	NA
ARF5	-0.34252	0.107889	-3.174743	0.00149969	0.063463521
HSH2D	-0.316101	0.099625	-3.172895	0.00150927	0.063463521
HSPD1	0.325575	0.102706	3.169981	0.00152449	0.063463521
IGHD	-0.311222	0.098272	-3.166954	0.00154045	0.063463521
ELF1	0.284993	0.090006	3.166385	0.00154346	0.063463521
H1FX	-0.841793	0.266156	-3.162773	0.00156274	NA
TIMM10	-0.466356	0.147899	-3.1532	0.00161491	0.065847903
HLA.A	-0.48811	0.155191	-3.145216	0.00165964	0.066734957
DEDD2	0.373975	0.118986	3.143012	0.00167219	0.066734957
RPL11	0.17566	0.055922	3.141162	0.00168279	0.066734957
RPL19	0.174982	0.055732	3.139698	0.00169122	0.066734957
TOE1	-0.525842	0.16765	-3.13654	0.00170954	NA
HSD17B11	0.225734	0.071969	3.13654	0.00170954	0.066918293
ENSG00000280433	-1.035115	0.330889	-3.128285	0.00175829	NA
S1PR4	0.334145	0.106935	3.12474	0.00177962	0.069108695
CD82	-0.453641	0.145515	-3.11748	0.00182404	0.069941038
CFAP410	-0.353813	0.113536	-3.116309	0.0018313	0.069941038
PRPF38A	0.267702	0.08596	3.114281	0.00184394	0.069941038
ENSG00000275964	-0.777304	0.249614	-3.114021	0.00184556	NA
XXYLT1.AS2	1.363731	0.438451	3.110336	0.00186875	NA
PHF23	0.402011	0.129262	3.110054	0.00187053	0.070403929
STX7	0.242498	0.078181	3.101743	0.00192385	0.071857913
LYRM7	0.38125	0.123316	3.091657	0.00199042	0.073781386
PILRB	0.788754	0.255835	3.083062	0.00204882	0.075315587
RPL23A	0.201632	0.065442	3.081068	0.0020626	0.075315587
ENSG00000254397	1.823559	0.592292	3.078818	0.00207824	NA
SPINT2	-0.295527	0.096286	-3.069263	0.00214588	0.077776047
NFKB2	-0.583608	0.190647	-3.061202	0.0022045	0.079313463
TAPBP	-0.323273	0.10571	-3.058101	0.00222745	0.079553971
ENSG00000166927	-1.052166	0.344448	-3.05464	0.00225331	0.079686918
IL23A	-0.942947	0.308746	-3.054122	0.0022572	NA
HSBP1	0.262303	0.085909	3.053254	0.00226374	0.079686918
PCMTD2	-0.384406	0.126111	-3.048158	0.00230249	0.080019533
LDHA	-0.326486	0.107125	-3.047713	0.0023059	0.080019533
ROR1	-1.186801	0.391034	-3.035032	0.0024051	NA
RPL35A	0.205765	0.067807	3.034584	0.00240868	0.082407157
RPL10	0.231465	0.076286	3.034188	0.00241185	0.082407157
ENSG00000282988	0.739522	0.243864	3.032518	0.00242523	0.082407157
NIPAL3	0.476726	0.15732	3.0303	0.00244311	0.082442266
RPS18	0.2255	0.074541	3.025194	0.00248474	0.083272686

SRA1	-0.430777	0.14273	-3.018135	0.00254335	0.084657294
IL21R	-1.125275	0.372887	-3.017737	0.0025467	NA
ENSG00000273748	-1.525363	0.50591	-3.015087	0.00256905	0.08493499
TCOF1	-0.419291	0.139407	-3.007673	0.00263256	0.086450426
DDB2	-0.496002	0.165111	-3.004043	0.00266417	0.086905343
ELMSAN1	0.380013	0.126616	3.001288	0.0026884	0.087074039
TNRC6C	0.5103	0.170071	3.000504	0.00269533	NA
TDG	0.305235	0.101855	2.996769	0.00272857	0.087074039
PCIF1	-0.393408	0.131431	-2.993279	0.00275997	0.087074039
GCC2	0.263848	0.088149	2.993201	0.00276068	0.087074039
RPLP2	0.24274	0.081112	2.992641	0.00276575	0.087074039
MRPL28	-0.286764	0.09586	-2.991499	0.00277612	0.087074039
RHBDD2	-0.500153	0.167411	-2.987582	0.00281194	0.087635666
AASDH	0.382776	0.128361	2.982025	0.00286349	0.088677528
RPS13	0.149323	0.050206	2.974185	0.00293768	0.090402951
CRLF3	0.270125	0.090932	2.970624	0.00297196	0.090886182
TMEM170A	0.384487	0.129542	2.968052	0.00299694	0.091080836
CMTM7	-0.37344	0.126134	-2.960668	0.00306973	0.092554195
RIN3	0.716283	0.242034	2.959429	0.0030821	NA
SRP72	-0.35572	0.120203	-2.959314	0.00308325	0.092554195
CCL4	-1.705615	0.57698	-2.956108	0.00311548	NA
RPL34	0.15883	0.05384	2.950051	0.00317722	0.094793416
RPL21	0.191804	0.065263	2.938915	0.00329364	0.097671311
MME	-2.060243	0.701642	-2.936317	0.00332135	NA
COLCA2	-1.373355	0.467992	-2.934569	0.00334012	NA
PURA	0.493217	0.168154	2.93313	0.00335563	0.097868847
PRDX3	0.428249	0.146052	2.932166	0.00336606	0.097868847
MAPKAPK2	-0.453663	0.154867	-2.929369	0.0033965	0.097868847
PSPC1	-0.406329	0.138719	-2.929142	0.003399	0.097868847
POLR2J	-0.270736	0.092432	-2.929022	0.00340031	0.097868847
ZNF16	-0.651843	0.222714	-2.926816	0.00342451	NA
LIMD2	-0.255781	0.087633	-2.918778	0.00351406	0.099441379
PCGF5	-0.287392	0.098463	-2.918774	0.00351411	0.099441379
RPL37	0.142625	0.048867	2.918614	0.00351591	0.099441379
ENSG00000130313	-0.230551	0.079121	-2.913899	0.00356945	0.100278419
FAM184B	-1.755632	0.60281	-2.912412	0.0035865	0.100278419
ZC3H7A	-0.374955	0.129031	-2.905923	0.00366172	0.101235053
MIF	-0.356909	0.122823	-2.905891	0.00366209	0.101235053
ADAMTS6	0.901601	0.310914	2.899836	0.00373358	NA
TNFAIP8L2	0.551683	0.190483	2.896234	0.00377671	NA
RPL35	0.169675	0.058619	2.894539	0.00379715	0.104379059
SVBP	-0.243834	0.084301	-2.892421	0.00382285	0.104498456
TM2D3	0.501328	0.173548	2.888691	0.00386849	0.105158555
VOPP1	-0.382596	0.13265	-2.884248	0.00392349	0.106064304
SLC25A25	0.636256	0.220833	2.881164	0.0039621	NA
LILRB1	0.496608	0.17276	2.874558	0.00404594	0.10877356
SEC61B	-0.207005	0.072317	-2.862458	0.00420369	0.112228979

MRPL41	-0.291403	0.101846	-2.861205	0.00422034	0.112228979
ATF7IP2	-0.437288	0.152942	-2.85917	0.00424751	0.112340831
CAV1	-1.194325	0.41795	-2.857577	0.00426889	NA
ENSG00000272754	-0.743623	0.260326	-2.856502	0.00428337	NA
SESN3	0.306248	0.10733	2.85334	0.00432623	0.11380773
GOPC	0.394866	0.138553	2.849933	0.00437284	0.114418726
SETD5	0.254646	0.089505	2.845045	0.00444051	0.115312441
FAM98A	0.497794	0.175039	2.843904	0.00445645	NA
RPS15A	0.205888	0.072402	2.843665	0.00445979	0.115312441
ACTR1B	-0.283076	0.099591	-2.842388	0.0044777	0.115312441
LNPEP	0.366092	0.128977	2.838425	0.00453367	0.116142742
NOP10	0.19167	0.067576	2.836369	0.00456297	0.116213014
AEN	-0.57529	0.20291	-2.8352	0.00457969	NA
PTGES2	-0.437927	0.15455	-2.833558	0.0046033	0.116213014
TIAL1	0.237631	0.083933	2.831198	0.0046374	0.116213014
INKA1	0.475206	0.167912	2.830087	0.00465353	0.116213014
SUMO3	-0.422274	0.149237	-2.829557	0.00466125	0.116213014
ZNF253	0.551765	0.195023	2.829235	0.00466594	NA
CYBC1	-0.189521	0.067008	-2.828346	0.00467892	0.116213014
CLECL1	1.554259	0.549767	2.827121	0.00469685	NA
RPL39	0.18079	0.063997	2.82499	0.00472821	0.116844021
RPS12	0.189623	0.067167	2.823153	0.00475539	0.116925297
UQCRH	0.152193	0.054	2.818374	0.00482676	0.118086557
ZNF557	0.433904	0.15411	2.815549	0.00486939	0.11853699
EIF3E	0.256169	0.091168	2.809868	0.00495618	0.1196862
YWHAE	0.34407	0.122477	2.809262	0.00496552	0.1196862
ENSG00000245904	0.246239	0.087772	2.805448	0.00502468	0.120518344
CPM	-1.058451	0.377783	-2.801742	0.00508275	NA
AKAP13	0.242941	0.086908	2.795369	0.00518404	0.123692773
CYTH1	-0.245946	0.08803	-2.793905	0.00520759	0.123692773
SLC15A2	0.786214	0.281523	2.792714	0.00522678	NA
MLST8	-0.394124	0.141246	-2.790328	0.00526546	0.124463333
PTP4A1	-0.34734	0.124874	-2.781521	0.00541048	0.127276274
LINC02256	-0.537581	0.193402	-2.779603	0.00544255	NA
ADRB2	1.090669	0.393052	2.774874	0.0055223	NA
RPL5	0.178347	0.064296	2.773852	0.00553969	0.129474663
RPL26	0.160762	0.05801	2.77131	0.00558313	0.129474663
APOBEC3C	-0.276212	0.099669	-2.771299	0.00558331	0.129474663
HCK	-0.816004	0.294459	-2.771199	0.00558503	NA
C6orf226	-0.52381	0.189086	-2.770215	0.00560193	NA
TTC9C	0.320794	0.115897	2.767925	0.00564144	0.130183803
RPS27L	-0.326341	0.117964	-2.766445	0.00566711	0.130183803
RYK	0.538182	0.194859	2.761908	0.00574647	NA
POU2F2	0.221034	0.080064	2.760725	0.00576732	0.1309594
SLC25A5	-0.178823	0.064788	-2.760103	0.00577831	0.1309594
VPREB1	-1.983685	0.71898	-2.759027	0.00579738	0.1309594
GAPT	0.317165	0.115066	2.756373	0.00584463	0.1309594

ABTB1	-0.273906	0.099476	-2.753481	0.00589653	0.1309594
HRK	-1.300865	0.472458	-2.753397	0.00589803	0.1309594
MFAP1	0.427017	0.155097	2.75322	0.00590122	0.1309594
PSMB10	-0.318096	0.115733	-2.748524	0.00598643	0.1309594
EGLN2	0.274869	0.100019	2.748168	0.00599294	0.1309594
BET1	0.325685	0.118557	2.747086	0.00601274	0.1309594
TRAM1	-0.185794	0.067656	-2.746158	0.00602978	0.1309594
SYNE2	-0.422918	0.154009	-2.746061	0.00603155	0.1309594
SCAND1	-0.222559	0.081074	-2.745124	0.00604881	0.1309594
NT5E	0.899723	0.327816	2.744596	0.00605854	NA
ATP6V0A2	-0.770433	0.280977	-2.741978	0.00610705	NA
CRIP1	-0.563519	0.205696	-2.739571	0.00615194	0.131908708
ITGB7	0.445843	0.162795	2.738677	0.00616869	0.131908708
RANGRF	-0.29113	0.106313	-2.738419	0.00617353	0.131908708
BIN1	0.397057	0.145105	2.73635	0.0062125	0.132164144
GTF3C6	-0.260674	0.095321	-2.734688	0.00624394	0.132258025
PDPK1	0.383146	0.140236	2.732153	0.0062922	0.132705683
RPL13	0.193309	0.070909	2.726172	0.00640736	0.134554481
ENSA	-0.167654	0.061575	-2.722777	0.00647357	0.135364026
RPS15	0.142783	0.052471	2.721161	0.00650531	0.135448812
ENSG00000263004	-1.062851	0.391107	-2.717544	0.00657684	NA
UBE2L3	-0.212085	0.078046	-2.717448	0.00657874	0.136038619
YTHDF3	0.53961	0.198618	2.716819	0.00659126	NA
EEF1B2	0.286283	0.105401	2.716133	0.00660495	0.136038619
UBA52	0.125479	0.046208	2.715527	0.00661704	0.136038619
VPS25	-0.384442	0.141728	-2.712529	0.00667719	0.136700872
FBXO4	-0.489094	0.18045	-2.710417	0.00671986	NA
INTS5	0.593392	0.219082	2.708535	0.0067581	NA
ATP6V1C2	-1.757281	0.649031	-2.707545	0.00677829	NA
PFKFB3	-0.42051	0.155317	-2.707427	0.00678071	0.138241642
SLCO5A1	-1.131897	0.418556	-2.704288	0.00684509	NA
RACK1	0.179328	0.066359	2.702394	0.00688421	0.139474999
DENND2D	0.494061	0.182869	2.701719	0.00689821	0.139474999
ENSG00000260879	-1.134608	0.420333	-2.699308	0.00694839	NA
UCP2	-0.298811	0.110771	-2.697552	0.00698514	0.140651475
MYL12A	0.174549	0.064795	2.693868	0.00706282	0.141292516
UBE2E3	-0.310966	0.115459	-2.693306	0.00707473	0.141292516
LACC1	-0.831987	0.30913	-2.691377	0.00711577	NA
FCRL2	0.367777	0.136673	2.690914	0.00712566	0.141731174
ENSG00000278158	0.280179	0.10437	2.684487	0.00726411	0.143899991
ALOX5	-0.306124	0.114164	-2.681448	0.00733044	0.144628401
RPL18	0.187302	0.069909	2.679234	0.00737909	0.144952071
NDUFS8	-0.279515	0.104374	-2.67801	0.00740609	0.144952071
PIP4K2A	0.335144	0.125522	2.67	0.00758514	0.147864808
SPHK2	-0.31189	0.117137	-2.662617	0.00775357	0.149994386
ID3	-0.317455	0.119248	-2.662152	0.00776429	0.149994386
HSPE1	0.163839	0.061566	2.661197	0.00778634	0.149994386

FNTA	0.311237	0.117106	2.657749	0.00786644	0.150943179
NFKBIB	-0.398174	0.149909	-2.656117	0.00790463	NA
ENSG00000275799	0.600337	0.226095	2.655239	0.00792523	0.151477045
CCNG1	0.238444	0.089906	2.652145	0.00799822	0.152277394
ENSG00000239636	-0.611627	0.230822	-2.649785	0.0080543	NA
THAP2	0.418559	0.15798	2.649438	0.00806258	NA
CLCF1	0.548929	0.207306	2.64792	0.00809887	0.153244288
PLTP	-1.516412	0.572743	-2.647632	0.00810578	NA
PF4	2.595135	0.980214	2.647517	0.00810852	NA
ENSG00000271204	0.544914	0.205831	2.647387	0.00811164	0.153244288
C11orf24	-0.416076	0.157279	-2.645465	0.00815786	NA
PPP4C	-0.181597	0.068761	-2.640997	0.00826623	0.155564172
ATP6V1C1	0.376196	0.142712	2.636051	0.00838772	NA
PFKP	-0.85598	0.325288	-2.631451	0.00850211	NA
ZNRD2	-0.342515	0.130443	-2.625785	0.00864495	0.162064417
RPS27	0.157738	0.06012	2.623714	0.00869768	0.162064417
NDUFB8	-0.151631	0.057804	-2.623193	0.008711	0.162064417
PIM1	-0.377791	0.144357	-2.617052	0.00886929	0.164384309
TOMM20	0.240327	0.091908	2.614873	0.00892606	0.164812027
DNAAF4	-1.198195	0.458246	-2.614741	0.00892951	NA
RBBP8	-0.645056	0.246755	-2.614156	0.00894481	NA
SPACA9	-0.57916	0.221558	-2.61403	0.00894811	NA
UBE2J2	-0.265526	0.101645	-2.612293	0.0089937	0.165436778
NUAK2	-0.243456	0.093275	-2.610094	0.00905172	0.165880454
CHRAC1	-0.394821	0.151412	-2.607587	0.00911829	0.16647674
ENSG00000231856	-0.935858	0.359122	-2.605961	0.0091617	NA
DNAAF5	-0.91531	0.351264	-2.605764	0.00916696	NA
MYC	0.379556	0.145798	2.603309	0.00923288	0.167929264
HSPH1	0.43613	0.167721	2.600334	0.0093133	0.167929264
BBC3	-1.118981	0.430397	-2.59988	0.00932563	NA
CAPN12	0.440776	0.16954	2.599839	0.00932674	0.167929264
SLC16A1.AS1	0.445365	0.171325	2.599531	0.00933512	0.167929264
FUOM	-0.543878	0.209222	-2.599526	0.00933527	NA
ENSG00000185527	-0.963706	0.371097	-2.596908	0.0094067	NA
XIAP	0.370507	0.142697	2.596455	0.00941912	0.16881961
FAM217B	0.393687	0.151827	2.593005	0.00951414	0.169900311
ENSG00000179094	-0.784403	0.302812	-2.590394	0.0095866	NA
ENSG00000262089	0.643232	0.24832	2.590333	0.0095883	NA
PTPN20	-1.27365	0.491973	-2.58886	0.00962942	NA
HSPA4	-0.359719	0.139095	-2.586129	0.00970607	0.172697399
LINC02422	0.787421	0.304825	2.583188	0.0097892	NA
OTUB1	-0.222	0.085975	-2.58216	0.0098184	0.173495571
RPS16	0.172607	0.066849	2.582039	0.00982184	0.173495571
LHFPL2	-0.464941	0.180134	-2.581083	0.00984909	NA
NEMP1	0.741633	0.287391	2.580575	0.00986361	NA
LINC02397	0.281732	0.109292	2.577777	0.00994383	0.174082295
FCGR2B	0.672499	0.260887	2.57774	0.00994488	0.174082295

PDE6D	-0.381198	0.147914	-2.577153	0.00996179	0.174082295
UNC50	0.318851	0.123849	2.574509	0.01003826	0.174794303
RPS3A	0.167783	0.065258	2.571072	0.01013842	0.175812233
FZD3	1.107262	0.430665	2.571052	0.010139	NA
ERGIC2	0.282611	0.110004	2.569093	0.01019651	0.175812233
DYNLL1	0.315994	0.123055	2.567914	0.01023126	0.175812233
BBX	0.231082	0.089999	2.567603	0.01024044	0.175812233
ZNF350	-0.260766	0.101683	-2.564492	0.01033268	0.176634813
MAT2A	0.243615	0.095049	2.563038	0.01037605	0.176634813
GLRX	0.306484	0.119612	2.562324	0.01039742	0.176634813
DLGAP3	-1.01477	0.39612	-2.561772	0.01041397	NA
RPL29	0.169651	0.066259	2.560415	0.01045474	0.176634813
MAD2L2	-0.353588	0.138264	-2.557342	0.01054753	0.176634813
CCNG2	0.571025	0.223312	2.557076	0.01055563	0.176634813
NUP50	0.352357	0.137812	2.556802	0.01056394	0.176634813
ATRAID	-0.20057	0.078459	-2.556367	0.01057715	0.176634813
ST13	0.185407	0.072644	2.552262	0.01070262	0.178122098
FHL3	-0.914414	0.358412	-2.551292	0.01073243	NA
IGHV1.2	1.61346	0.632743	2.549946	0.01077395	NA
KRCC1	0.250183	0.098177	2.548279	0.01082559	0.179558034
RPN1	-0.453929	0.178354	-2.545098	0.01092469	0.180268041
IFIT2	0.479433	0.188384	2.544975	0.01092855	NA
STK17A	0.268257	0.105439	2.544192	0.01095308	0.180268041
HINT2	-0.219144	0.086163	-2.543369	0.01097892	0.180268041
BATF	1.104642	0.434625	2.541597	0.01103473	NA
CD40	-0.233378	0.091867	-2.540385	0.01107304	0.181205321
FAM43A	0.710271	0.279846	2.538077	0.01114636	0.1817971
DTX3L	0.466191	0.183842	2.53583	0.01121811	NA
TMEM205	-0.331599	0.130965	-2.531972	0.01134232	0.184378613
FBXO8	0.5057	0.199768	2.531436	0.01135964	NA
SOX4	-0.873724	0.345358	-2.529911	0.01140914	NA
NGLY1	0.199051	0.078686	2.529688	0.01141639	0.184660001
PPP1R15A	-0.556058	0.219905	-2.528629	0.0114509	0.184660001
ECI2	0.323018	0.127785	2.527824	0.01147719	0.184660001
SLK	-0.489084	0.193505	-2.527501	0.01148775	NA
SKAP2	0.297638	0.117792	2.526804	0.01151059	0.184660001
MED21	0.397974	0.157584	2.525474	0.01155423	NA
IKBIP	0.293966	0.116638	2.520326	0.01172461	0.187478867
APBB2	-0.846972	0.336169	-2.519485	0.01175266	NA
GRAMD1C	-0.571206	0.22692	-2.517208	0.01182891	0.188530403
YARS	-0.566445	0.225029	-2.517204	0.01182902	NA
CYSTM1	-0.321657	0.127958	-2.51378	0.01194451	0.189523685
CFL1	-0.169002	0.067254	-2.512884	0.01197487	0.189523685
RRP8	0.358214	0.142565	2.512634	0.01198337	NA
RPL27A	0.139133	0.055389	2.511926	0.01200743	0.189523685
FNBP1	-0.331856	0.132229	-2.509698	0.01208345	0.190110347
LINC00638	0.976702	0.3894	2.508226	0.01213392	NA

MIS18BP1	0.182819	0.072906	2.507597	0.01215552	0.190631231
ENSG00000270562	0.618622	0.247183	2.502686	0.01232548	NA
ZBTB20	0.258248	0.10326	2.500939	0.01238645	0.193632305
KLHL2	-0.727038	0.29076	-2.500474	0.01240273	NA
ENSG00000268516	0.456051	0.182396	2.500337	0.01240753	NA
EXOG	-0.432704	0.17346	-2.494542	0.01261198	0.196530062
MICOS13	-0.213222	0.085535	-2.492805	0.01267385	0.196867091
CFAP73	-1.104437	0.44357	-2.489883	0.01277851	NA
HIP1R	-0.289572	0.116436	-2.486968	0.01288371	0.19895543
ZNF138	0.414299	0.166704	2.485235	0.0129466	0.19895543
CCT8	0.440732	0.177377	2.484713	0.01296558	0.19895543
FLII	-0.35755	0.143961	-2.483667	0.01300372	0.19895543
MT.CO1	0.211661	0.085229	2.483452	0.0130116	0.19895543
SCAMP4	-0.606467	0.244284	-2.482632	0.01304158	NA
SSR2	0.148378	0.059794	2.481469	0.0130842	0.199442258
LSM6	0.19613	0.079093	2.479752	0.01314738	0.199782989
ENSG00000260349	-0.795281	0.320771	-2.479276	0.01316496	NA
HSP90AB1	0.223881	0.090354	2.477813	0.01321906	0.200250286
PIP4P2	0.50201	0.202765	2.47582	0.01329305	NA
PDCL3	-0.345681	0.139626	-2.475757	0.01329539	0.200785026
ENSG00000235560	0.510569	0.206589	2.47143	0.01345739	NA
ZBTB16	2.010131	0.813463	2.47108	0.01347058	NA
CAST	-0.309502	0.125292	-2.47025	0.01350187	0.202514433
PHPT1	-0.466565	0.18889	-2.470041	0.01350974	0.202514433
KMT2A	0.232561	0.094177	2.469398	0.01353407	0.202514433
UNC119	-0.335012	0.135785	-2.46722	0.01361668	0.202532344
PTTG1IP	0.396481	0.160714	2.46699	0.01362541	0.202532344
RNF146	0.283151	0.114817	2.466097	0.01365945	0.202532344
RND1	-0.839869	0.34071	-2.465051	0.01369936	NA
MTHFR	0.332436	0.134886	2.464565	0.01371795	0.202785345
ENSG00000274922	-1.550244	0.629042	-2.464453	0.01372226	NA
SAP18	-0.166721	0.067806	-2.458789	0.01394066	0.205456695
THNSL1	0.846126	0.344461	2.456378	0.01403455	NA
EBLN2	0.453626	0.184717	2.455783	0.01405779	NA
NDUFB7	-0.160348	0.065295	-2.455735	0.0140597	0.206508251
RPL37A	0.129089	0.052602	2.454074	0.01412481	0.206508251
CHMP2B	0.240972	0.098207	2.453722	0.01413862	0.206508251
KCTD5	0.379322	0.154662	2.452585	0.01418338	NA
TBPL1	0.314131	0.128207	2.450194	0.01427794	0.207922462
ENSG00000227495	-1.145911	0.467994	-2.448557	0.01434297	NA
ZNF593	-0.328721	0.134281	-2.448	0.01436518	0.208572193
PTPN7	-0.511668	0.209259	-2.445136	0.01447974	0.209012677
GOLGA3	-0.45082	0.184377	-2.445106	0.01448095	0.209012677
CREBL2	0.38466	0.157571	2.441188	0.01463903	NA
NHSL2	-0.519081	0.212773	-2.439602	0.01470345	0.2115999
ORMDL2	-0.290892	0.119514	-2.433956	0.01493482	0.214299339
YJEFN3	0.976485	0.401583	2.431587	0.01503284	NA

CARD8	-0.311804	0.128235	-2.431506	0.01503621	0.215123365
ENSG00000183308	-1.429081	0.588032	-2.430277	0.01508727	NA
SRD5A3	0.905191	0.372491	2.430103	0.01509455	NA
RDH5	0.463849	0.190885	2.429986	0.01509941	0.215218649
FRY.AS1	-1.566976	0.645049	-2.429236	0.01513069	NA
GPSM3	-0.119232	0.049091	-2.4288	0.0151489	0.215218649
DBF4B	0.763726	0.31456	2.427915	0.01518589	NA
FXYD7	0.683703	0.281642	2.427559	0.01520083	0.215218649
PRKRA	-0.230909	0.095141	-2.427015	0.01522364	0.215218649
UFD1	-0.35994	0.148363	-2.426083	0.0152628	0.215218649
TM9SF4	-0.432016	0.178132	-2.425255	0.01529763	NA
PRDX2	-0.329769	0.136163	-2.421876	0.0154406	0.217100147
MAP1LC3B2	-0.639375	0.264072	-2.421215	0.01546874	NA
PTPRS	1.571937	0.649256	2.421136	0.0154721	NA
ZNF827	0.833749	0.344484	2.420286	0.0155083	NA
PTGIR	-1.230154	0.508733	-2.418073	0.01560295	NA
ENSG00000233178	-0.615145	0.254418	-2.417858	0.01561218	NA
LANCL2	-0.858447	0.35536	-2.415713	0.01570443	NA
TNFAIP8	0.216674	0.089723	2.414913	0.01573898	0.220082337
KIAA0040	0.538618	0.223046	2.414828	0.01574266	0.220082337
GNG11	0.903484	0.374196	2.41447	0.01575812	NA
CNOT1	-0.259623	0.107588	-2.413135	0.01581596	0.220477139
STARD10	-0.385568	0.15995	-2.410552	0.0159284	NA
CHURC1	0.175808	0.072977	2.409091	0.01599231	0.22194853
WDR18	-0.361166	0.150015	-2.407536	0.0160606	0.22194853
TOR3A	-0.308968	0.128372	-2.406822	0.016092	0.22194853
MICAL1	-0.325634	0.135318	-2.406426	0.01610947	NA
SAYSD1	0.333674	0.1387	2.405729	0.01614022	0.22194853
WDR83	-0.281258	0.11692	-2.405546	0.01614831	0.22194853
RAB33B	0.620829	0.258085	2.405519	0.0161495	NA
FAM161A	0.498276	0.207158	2.405291	0.01615959	NA
KRT10	-0.340039	0.141472	-2.403581	0.01623538	0.222520185
TAF8	-0.3502	0.145941	-2.399593	0.0164133	0.224330318
CD53	0.193268	0.080611	2.397526	0.01650621	0.224408682
ARID1A	0.331538	0.138289	2.397425	0.01651076	0.224408682
ZNF345	0.43757	0.182628	2.395962	0.01657682	NA
DUSP12	-0.286062	0.119436	-2.395109	0.0166154	0.225205463
TSPAN17	-0.454327	0.189719	-2.394735	0.01663237	NA
CCDC122	-0.555979	0.232249	-2.393891	0.0166707	NA
CXorf40B	0.495943	0.207249	2.39298	0.01671213	NA
PHKG2	-0.328147	0.137191	-2.391904	0.01676122	0.226554276
NUCB2	-0.544639	0.227708	-2.391831	0.01676458	NA
CBLN3	-0.713134	0.298305	-2.390622	0.01681986	NA
COMMD3	-0.25651	0.107391	-2.388559	0.0169146	0.227582354
SRGAP2B	0.652928	0.273396	2.388218	0.0169303	0.227582354
TXLNB	1.174999	0.492096	2.387742	0.01695224	NA
DNMT1	-0.351861	0.147542	-2.38481	0.01708794	0.229072012

TXNDC11	0.396606	0.166352	2.384134	0.01711938	NA
SNRPB	-0.184089	0.077262	-2.382673	0.01718745	0.22946159
PIK3CD.AS1	-0.87956	0.369179	-2.382477	0.01719661	NA
HLA.C	-0.463901	0.194788	-2.381563	0.01723937	0.22946159
EVI2A	0.473635	0.198908	2.381171	0.01725769	0.22946159
OSGEPL1	0.469026	0.196995	2.380898	0.01727048	NA
ENSG00000268400	-1.498302	0.629528	-2.380039	0.01731082	NA
XPO5	-0.719274	0.302277	-2.379515	0.01733542	NA
TCTN2	-0.883452	0.371296	-2.379374	0.01734205	NA
RBAK.RBAKDN	0.68464	0.287818	2.378729	0.01737245	NA
SLBP	0.485784	0.204244	2.378447	0.01738574	0.230537765
RPS4X	0.262034	0.11032	2.375218	0.01753858	0.231548941
MOB4	0.21705	0.091396	2.374838	0.01755664	0.231548941
SEZ6	-0.62298	0.262478	-2.373452	0.0176227	NA
ENSG00000198106	-0.33641	0.141802	-2.372397	0.01767311	0.232458438
MPND	-1.03488	0.436724	-2.369642	0.01780529	NA
SUSD3	0.334266	0.141122	2.368633	0.01785398	0.234207785
TPRA1	0.642237	0.271297	2.367286	0.01791908	NA
MYBL2	-1.412934	0.596871	-2.367236	0.01792152	NA
SLTM	0.173806	0.073447	2.366406	0.01796171	0.234991059
ZNF232	0.476996	0.201852	2.3631	0.01812278	NA
SRSF11	0.203342	0.086127	2.360957	0.01822785	0.237837043
MBLAC1	-1.210656	0.512967	-2.360104	0.01826983	NA
DUS3L	-0.448706	0.190377	-2.356929	0.01842677	NA
METTL8	0.726364	0.308189	2.356878	0.01842929	0.239825825
ACAP1	0.300548	0.127708	2.353392	0.01860303	0.241296592
IGHA1	-0.552741	0.234946	-2.352635	0.01864094	0.241296592
DOCK8	0.292542	0.124726	2.345467	0.01900326	0.245337584
DRG2	-0.291938	0.124598	-2.34304	0.01912735	0.246289816
ICA1L	-0.450706	0.192484	-2.341527	0.01920503	0.246640996
NAA60	-0.383607	0.1639	-2.340495	0.01925818	0.246676132
MUC20.OT1	-0.293479	0.125565	-2.337273	0.01942498	0.248162998
CLPP	-0.211328	0.09048	-2.335634	0.01951033	0.248604245
PTBP1	-0.279724	0.11984	-2.334137	0.01958856	0.248952839
LACTB2.AS1	-0.800263	0.343216	-2.331661	0.01971855	NA
UBE2T	0.519094	0.222756	2.330326	0.01978893	NA
FOXK1	-0.352347	0.151267	-2.329303	0.01984304	0.251185624
ZNF564	-0.489022	0.209974	-2.328965	0.01986094	NA
KRIT1	0.338284	0.145303	2.328129	0.01990524	0.251185624
NDUFA1	-0.144179	0.061936	-2.327884	0.01991826	0.251185624
RPS27A	0.155948	0.067051	2.325811	0.02002863	0.251928234
FBXL22	0.805855	0.346575	2.325194	0.02006156	NA
RPS14	0.212395	0.091366	2.324657	0.02009029	0.252055916
CYP3A5	0.521592	0.22458	2.322517	0.02020511	NA
LRRC8A	0.71105	0.306187	2.322274	0.02021818	NA
LTA4H	0.310699	0.133827	2.321646	0.02025202	0.252897875
GYPC	0.181567	0.078215	2.321378	0.02026644	0.252897875

DHX40	-0.536479	0.231115	-2.321267	0.02027243	NA
PSMB3	-0.186209	0.080268	-2.319845	0.02034927	0.252897875
NKAPL	-1.088834	0.469404	-2.319609	0.02036206	NA
DDX21	0.188854	0.081417	2.31957	0.02036415	0.252897875
ENSG00000270659	-0.674386	0.290772	-2.319294	0.02037908	NA
FRG1	-0.183293	0.079105	-2.31709	0.02049883	0.25392604
CABLES1	-0.897406	0.387923	-2.313363	0.0207027	NA
NPIPB11	-1.145564	0.495286	-2.312936	0.02072614	NA
ARIH2OS	-0.425634	0.184083	-2.312181	0.02076771	NA
MYL6	-0.148029	0.064032	-2.311803	0.02078853	0.256838118
RPL22	0.124476	0.053896	2.309568	0.02091208	0.256838118
RPS3	0.167087	0.072354	2.309297	0.02092713	0.256838118
SEC13	-0.333994	0.144649	-2.308995	0.02094388	0.256838118
TMEM140	0.444785	0.192677	2.308452	0.020974	NA
ENSG00000226571	1.196915	0.518634	2.307822	0.02100904	NA
MARCHF1	0.290273	0.125793	2.307549	0.02102421	0.257178696
MSL3	0.286326	0.124159	2.306124	0.02110372	0.257499905
RPL7	0.179694	0.077952	2.305194	0.02115573	0.257499905
ST3GAL6	-1.521413	0.660082	-2.304883	0.02117312	NA
BHLHE40	-0.465646	0.202114	-2.303879	0.02122942	0.257755692
RARA	-0.355178	0.154287	-2.302056	0.02133204	0.258360597
KCTD17	-0.433665	0.188599	-2.299406	0.02148189	NA
POLR2B	-0.246923	0.107426	-2.298536	0.02153129	0.259454462
PFKL	-0.235724	0.102556	-2.298492	0.02153381	0.259454462
COL9A3	-0.960622	0.418088	-2.297655	0.02158144	0.259454462
RTL10	0.678327	0.295234	2.297589	0.02158518	NA
B3GNT7	-0.816925	0.356391	-2.292219	0.02189304	NA
HAGHL	0.355292	0.155036	2.291674	0.02192446	0.262932262
RTL8C	-0.508968	0.222147	-2.291135	0.02195561	NA
MMP7	-1.844977	0.80539	-2.290787	0.02197573	NA
TMEM60	0.343031	0.149786	2.290143	0.02201304	0.263132239
ENSG00000235078	0.45469	0.19858	2.28971	0.02203815	NA
MRPS15	-0.206158	0.090044	-2.289528	0.02204869	0.263132239
CLIC4	-0.36264	0.158495	-2.288025	0.02213606	0.26353222
TNFAIP1	0.472304	0.206446	2.287785	0.02215004	NA
KLHL8	0.410014	0.179294	2.286826	0.02220601	NA
ENSG00000267390	0.434521	0.190177	2.284826	0.02232305	NA
PDE4D	-1.042291	0.456879	-2.28133	0.02252893	NA
STK38L	-0.476664	0.209065	-2.279976	0.0226091	NA
DHX9	-0.242077	0.106184	-2.279783	0.02262056	0.26864665
HLA.F	-0.357597	0.156989	-2.277842	0.02273601	0.269267721
DHX15	0.313154	0.137535	2.276906	0.02279186	0.269267721
FDX2	-0.344718	0.151438	-2.276293	0.02282846	NA
IFT57	0.463853	0.20379	2.276135	0.02283795	0.269267721
PIGF	0.390657	0.171642	2.275992	0.02284651	NA
HSD17B7	0.334744	0.147133	2.275117	0.02289891	0.269337356
ZNF585A	0.482038	0.211967	2.274123	0.02295858	NA

ENOSF1	1.10238	0.48515	2.272247	0.02307157	NA
LINC01250	-2.270184	0.999293	-2.27179	0.02309922	NA
ZNF704	-1.167461	0.514078	-2.27098	0.02314822	NA
NUP43	0.330958	0.14577	2.270415	0.02318244	NA
HDAC9	-0.516153	0.227345	-2.270345	0.02318666	NA
APPL2	-0.54794	0.241625	-2.267733	0.02334546	NA
G6PC3	-0.533845	0.23543	-2.26753	0.02335787	NA
ADAM28	0.319272	0.140846	2.266819	0.02340128	0.274501693
SORL1	-0.345991	0.152687	-2.26602	0.02345018	0.274501693
DOK1	0.429448	0.189612	2.264882	0.02351994	0.274661282
THAP9.AS1	-0.443426	0.195998	-2.262396	0.02367296	0.275359084
LMO4	0.362146	0.160094	2.262083	0.02369225	0.275359084
ZNF707	-0.530556	0.23477	-2.259895	0.02382778	NA
GPM6B	0.301194	0.133327	2.259062	0.02387955	0.276878244
UGT8	0.605173	0.268747	2.251827	0.02433319	NA
CIZ1	-0.25951	0.115287	-2.250997	0.0243857	0.282078587
CLNS1A	0.386815	0.171962	2.249419	0.02448586	0.282569158
ZNF100	-0.558243	0.248298	-2.24828	0.02455837	NA
CSE1L	0.389061	0.173074	2.247952	0.02457925	NA
IFIT5	0.384217	0.170986	2.247069	0.02463562	NA
APOBEC3H	-1.064776	0.473937	-2.246659	0.02466186	NA
SPATC1L	-0.553716	0.246502	-2.24629	0.02468547	0.283400481
FNIP1	-0.336788	0.149948	-2.246027	0.02470229	0.283400481
RFC4	0.468067	0.208403	2.245969	0.024706	NA
SNHG32	0.197083	0.087765	2.245569	0.02473166	0.283400481
ABHD5	-0.833997	0.371713	-2.243658	0.02485444	NA
FLYWCH2	-0.270263	0.120571	-2.241528	0.0249919	0.285237149
EXOSC8	0.228365	0.101891	2.241271	0.02500853	0.285237149
COCH	-0.797252	0.355866	-2.240317	0.02507032	NA
FADS2	-1.343051	0.599647	-2.239737	0.02510797	NA
CYB561D2	-0.272959	0.121945	-2.238384	0.02519603	0.28670734
ZDHHC19	1.12934	0.505013	2.236259	0.02533483	NA
PTPRJ	-0.889542	0.397792	-2.236198	0.02533883	NA
UBE2I	-0.126909	0.056771	-2.23545	0.02538783	0.28821965
DDX23	0.363832	0.162756	2.235449	0.0253879	NA
UBC	-0.197142	0.088234	-2.234315	0.02546238	0.288396818
ENSG00000272758	0.780669	0.349446	2.234021	0.02548171	NA
LINC01473	0.440806	0.197337	2.233774	0.02549792	NA
LINC00662	0.323213	0.144876	2.23097	0.02568311	0.29022505
ATP6V0A1	0.444768	0.200004	2.223793	0.0261624	0.294806271
CDC42SE1	-0.183925	0.082734	-2.223101	0.02620902	0.294806271
TERF2IP	0.335644	0.151043	2.222183	0.02627091	0.294824684
BARD1	0.375982	0.169276	2.221109	0.02634357	NA
ENSG00000246528	-0.910668	0.410901	-2.216271	0.02667292	NA
DBNL	-0.245929	0.110986	-2.215865	0.02670076	0.298221121
THUMPD3.AS1	-0.221265	0.099858	-2.215805	0.02670486	0.298221121
CEBPZOS	0.251411	0.113501	2.215053	0.0267564	0.298221121

NUDT9	0.391853	0.176984	2.214066	0.02682424	NA
HNRNPDL	-0.1963	0.088693	-2.213255	0.02688006	0.298918461
JAK3	-0.447233	0.202231	-2.211493	0.02700172	0.299590519
CEMIP2	0.626002	0.283166	2.210726	0.0270548	NA
ZNF202	-0.597289	0.270448	-2.208522	0.0272079	NA
RIMKLB	-0.433035	0.196092	-2.208323	0.0272218	NA
LAPTM5	-0.193666	0.087748	-2.207066	0.02730942	0.30163334
CDC37L1.DT	-0.766702	0.347538	-2.206095	0.02737734	NA
UROD	-0.323647	0.146724	-2.205816	0.02739687	0.30163334
WDR45	-0.248931	0.112886	-2.205157	0.0274431	0.30163334
ATF4	-0.224464	0.1018	-2.204952	0.0274575	0.30163334
GM2A	0.588649	0.26703	2.204431	0.02749407	0.30163334
ENSG00000229043	-1.046519	0.475002	-2.203185	0.02758168	NA
GID8	-0.208247	0.094578	-2.20186	0.02767523	0.302941574
TIGD7	0.620404	0.281895	2.200832	0.02774789	NA
PIGX	-0.442382	0.201094	-2.199872	0.02781595	NA
NACA	0.123785	0.056272	2.199757	0.02782413	0.303755203
SMARCE1	-0.162353	0.073855	-2.198284	0.02792888	0.303755203
AAK1	-0.774193	0.352276	-2.197691	0.02797115	NA
ZNF441	-0.400555	0.182268	-2.197618	0.02797637	0.303755203
GLS	0.322473	0.146803	2.196642	0.02804602	0.303755203
GNB5	0.21374	0.097355	2.195471	0.0281298	0.303755203
MS4A1	-0.22316	0.101683	-2.194677	0.02818678	0.303755203
RNF26	-0.288738	0.131593	-2.19418	0.02822249	0.303755203
DEF6	0.292517	0.133335	2.19385	0.02824619	0.303755203
APOBEC3D	-0.315532	0.143874	-2.193108	0.02829963	NA
FKBP5	0.816423	0.372422	2.192201	0.02836503	NA
DHFR2	0.485969	0.221753	2.191487	0.02841659	NA
VPS8	0.315313	0.14389	2.191344	0.02842693	0.305028478
GPR137	-0.399115	0.182239	-2.190062	0.02851977	0.305355017
RGS3	0.875666	0.400127	2.18847	0.02863537	NA
CHCHD4	0.417232	0.190671	2.188233	0.02865264	NA
LINC00513	0.397351	0.181706	2.186782	0.02875843	0.306493047
NOP53	0.162702	0.074492	2.184145	0.02895158	0.306493047
PPP1CB	-0.25084	0.114846	-2.18414	0.02895198	0.306493047
NFKBID	-0.239415	0.109631	-2.183818	0.02897559	0.306493047
INTS13	0.457411	0.209482	2.183539	0.02899612	NA
CTNS	-0.572562	0.2623	-2.18285	0.02904688	NA
RBM5	0.202909	0.092969	2.182534	0.02907017	0.306493047
ZNF580	-0.227377	0.104184	-2.182461	0.0290755	0.306493047
XPO4	-0.389606	0.178599	-2.181451	0.02915008	NA
NECAP2	-0.291372	0.133576	-2.181325	0.02915939	0.306493047
TGIF1	0.322906	0.148051	2.18105	0.02917973	0.306493047
ACSF3	0.394953	0.181095	2.180914	0.02918981	0.306493047
TFAP4	0.338841	0.155546	2.178399	0.02937636	NA
PFN1	-0.113685	0.052193	-2.178163	0.02939391	0.306835784
RPLP1	0.194731	0.089417	2.177775	0.02942281	0.306835784

FAM91A1	0.348292	0.159976	2.177145	0.02946974	0.306835784
WDR13	-0.313403	0.143954	-2.177098	0.02947329	0.306835784
SYNGAP1.AS1	0.747139	0.343272	2.176519	0.02951644	NA
UBE3A	0.243435	0.111894	2.175593	0.02958572	0.307352291
EXOC3.AS1	-0.475859	0.218915	-2.173718	0.02972633	NA
AGMAT	0.577239	0.266013	2.169967	0.03000932	NA
PSMC3	-0.262537	0.121005	-2.169646	0.03003364	0.311344458
FANCL	0.486886	0.224458	2.169163	0.03007033	NA
ATXN7L2	-0.881663	0.406456	-2.169145	0.0300717	NA
C14orf119	0.209356	0.09657	2.167919	0.03016485	0.312043549
MRPL35	0.328154	0.151539	2.165476	0.03035126	NA
C1GALT1	0.339615	0.156834	2.165441	0.03035393	0.31333709
SIVA1	0.171268	0.079123	2.164569	0.0304207	0.313365211
PAN3	0.196186	0.090725	2.162428	0.03058518	0.314118511
SLC4A7	0.408706	0.189045	2.161948	0.03062222	0.314118511
MICU3	0.655942	0.303574	2.160732	0.03071602	NA
BLOC1S1	-0.187987	0.087022	-2.160235	0.03075448	0.314815187
ALDH3A2	0.903694	0.418366	2.160059	0.03076811	NA
CRYZ	0.641427	0.297035	2.159431	0.03081673	NA
VTI1A	-0.330815	0.153198	-2.159392	0.03081981	NA
BAD	-0.280777	0.130108	-2.158025	0.03092592	0.315909234
LINC00672	0.60809	0.281817	2.157751	0.03094716	NA
PYHIN1	1.014773	0.470429	2.157122	0.03099616	NA
NPHP3	0.408426	0.189413	2.156272	0.03106242	0.316233297
TTC21A	0.280964	0.13032	2.155959	0.0310869	0.316233297
MIR34AHG	-1.868089	0.86687	-2.154983	0.03116316	NA
AUP1	-0.198407	0.092244	-2.150904	0.03148374	0.318971913
ICAM3	0.23375	0.108718	2.150063	0.03155023	0.318971913
MANF	0.280768	0.130625	2.149416	0.03160142	0.318971913
DAAM1	-0.325029	0.151269	-2.148677	0.03166	0.318971913
TMEM138	-0.308735	0.143705	-2.148399	0.03168207	0.318971913
OAT	0.421171	0.19604	2.148391	0.03168272	NA
GRAP	0.393	0.183012	2.147396	0.03176173	0.31911738
L1TD1	-0.767604	0.357539	-2.146911	0.0318004	NA
NFS1	0.898964	0.418836	2.146339	0.03184598	NA
TMSB4Y	-1.259929	0.587045	-2.146223	0.03185517	NA
SSU72	-0.215261	0.100372	-2.144645	0.03198122	0.320664146
USP53	0.990879	0.462225	2.143713	0.03205587	NA
NFU1	-0.229121	0.106919	-2.14293	0.03211868	0.32138387
PDE3B	-0.465589	0.217781	-2.137882	0.03252631	0.324672224
TEX30	0.335917	0.157175	2.137217	0.03258038	NA
ARID4B	0.141121	0.06606	2.136267	0.03265766	0.324672224
ZMAT3	-0.306232	0.143374	-2.135893	0.03268813	0.324672224
LYRM2	-0.184382	0.086338	-2.135591	0.03271273	0.324672224
C12orf76	0.283776	0.132961	2.134275	0.03282032	0.325080567
FYN	0.541641	0.253856	2.133653	0.03287119	NA
DCTN4	-0.467705	0.219244	-2.133257	0.03290367	NA

OGT	0.361733	0.169584	2.133058	0.03291998	0.325408992
SNHG9	-0.335347	0.157346	-2.131268	0.03306704	0.325777956
MARCHF6	0.162642	0.076322	2.130984	0.03309047	0.325777956
ITM2A	0.903738	0.424297	2.129969	0.03317421	NA
ZC3H18	0.266024	0.124934	2.129319	0.03322785	0.325850903
PLPBP	0.237014	0.111312	2.12928	0.03323107	0.325850903
BAG2	0.415234	0.195204	2.127177	0.0334054	NA
CDCA4	0.455255	0.214039	2.126974	0.03342225	NA
ZMIZ2	-0.387629	0.182345	-2.125798	0.0335201	0.327630066
ABCB4	0.249472	0.117372	2.125482	0.03354643	0.327630066
GTF2IRD2	-0.407263	0.191798	-2.12339	0.03372121	0.328680999
ENSG00000273466	1.453839	0.684786	2.123054	0.03374934	NA
C6orf203	0.396694	0.186902	2.122477	0.03379774	NA
PSMG3	-0.431164	0.203437	-2.119402	0.03405651	0.331218411
GABARAP	-0.301618	0.142361	-2.118687	0.03411692	0.331218411
SHKBP1	-0.321995	0.152074	-2.117358	0.03422944	0.331652777
MAP3K8	-0.446497	0.211197	-2.114123	0.03450478	0.332962665
SYNC	-0.416678	0.197196	-2.113013	0.03459964	0.332962665
COL19A1	-0.362375	0.171503	-2.112931	0.03460665	0.332962665
ATP5ME	-0.135501	0.064146	-2.112393	0.03465271	0.332962665
TMEM99	0.477762	0.226228	2.111855	0.03469888	NA
FCRLA	0.333993	0.158157	2.111785	0.03470488	0.332962665
NKILA	0.811093	0.384564	2.109126	0.03493368	NA
TRMT112	0.187818	0.089066	2.108745	0.03496664	0.334817561
ARHGAP18	0.801295	0.380135	2.107925	0.03503752	NA
FADS1	-0.462327	0.21937	-2.107519	0.03507264	NA
NDUFA3	-0.256226	0.121599	-2.107142	0.03510529	0.335302133
FARSA	0.254352	0.120742	2.106577	0.0351543	0.335302133
SRRM2	-0.122441	0.058215	-2.103238	0.03544494	0.336055695
YAE1	0.605166	0.287763	2.102999	0.0354659	NA
JUNB	-0.249411	0.118602	-2.102927	0.03547212	0.336055695
FAM216A	0.383834	0.182535	2.102797	0.03548348	NA
RPL10A	0.199442	0.094869	2.102281	0.03552864	0.336055695
EPB41L2	-0.405236	0.192767	-2.102204	0.03553541	0.336055695
ATG4C	0.303164	0.144295	2.101005	0.03564051	0.336055695
LTBP3	0.268363	0.127734	2.10095	0.03564539	0.336055695
CCL5	-1.23367	0.587379	-2.100294	0.03570295	NA
ENSG00000270820	0.613259	0.292182	2.098889	0.03582665	NA
CRY1	-0.526017	0.250629	-2.098787	0.03583565	NA
PRDM8	-0.588434	0.280392	-2.098614	0.03585095	NA
SAR1B	0.392969	0.187261	2.098511	0.03586005	0.337141063
SLC38A11	1.210203	0.576858	2.097923	0.03591194	NA
CFLAR	0.263405	0.125578	2.097544	0.03594545	0.337141063
IDH2	-0.254533	0.121363	-2.097298	0.03596723	0.337141063
MRPS11	-0.207918	0.099215	-2.095621	0.03611583	0.337419276
DDX6	-0.271711	0.12967	-2.095407	0.03613483	0.337419276
HIST1H2BN	0.723943	0.345629	2.094566	0.03620962	NA

STK16	-0.229098	0.109468	-2.092832	0.0363642	0.337790199
SLC2A1	-0.27136	0.129728	-2.091755	0.03646046	0.337790199
CNIH4	0.275036	0.131552	2.090705	0.03655455	0.337790199
AQR	0.260539	0.124642	2.090295	0.03659131	0.337790199
TRIM44	0.20624	0.098668	2.09023	0.03659711	0.337790199
ERP29	0.13541	0.064784	2.090168	0.03660275	0.337790199
CCDC107	-0.200192	0.095808	-2.089515	0.03666135	0.337790199
MAPRE2	0.339325	0.162468	2.088563	0.03674708	0.337790199
TFDP2	-0.261709	0.125338	-2.088022	0.03679587	0.337790199
SRP19	-0.172345	0.082585	-2.086884	0.03689864	0.338099296
RASSF1	0.218847	0.10492	2.085833	0.03699374	0.338337138
NCBP1	-0.504233	0.241808	-2.085261	0.03704557	NA
FBXO36	-1.145428	0.549595	-2.084131	0.0371483	NA
ENSG00000277007	0.760875	0.365099	2.084023	0.03715808	NA
PCNX4	0.322904	0.154948	2.083948	0.03716492	0.338755039
SERP1	0.112403	0.053941	2.083805	0.0371779	0.338755039
WSB2	-0.60955	0.29268	-2.082654	0.03728278	NA
PNPLA7	0.556353	0.26729	2.081459	0.03739195	NA
GGCX	-0.368852	0.177306	-2.080305	0.03749753	NA
WDR11	-0.277185	0.133407	-2.077741	0.03773319	0.342774417
MED25	-0.38109	0.183494	-2.076855	0.03781496	0.342774417
ZNF667.AS1	0.400422	0.192816	2.076701	0.03782918	0.342774417
RPL27	0.133333	0.064259	2.074934	0.03799267	0.343619444
SNED1	-1.123316	0.541385	-2.074894	0.03799636	NA
AKTIP	0.521581	0.251456	2.074244	0.03805669	NA
NAB2	0.79764	0.384972	2.071941	0.0382709	NA
PTGES3	0.171657	0.082849	2.071937	0.03827134	0.345501236
THRB	2.002988	0.966801	2.07177	0.03828691	NA
YTHDC1	0.175121	0.084628	2.069289	0.038519	0.347096649
CCDC157	-0.7678	0.371183	-2.068518	0.03859134	NA
WASF1	-0.544075	0.263085	-2.068055	0.03863482	NA
TUBB4B	0.427345	0.206764	2.066824	0.03875073	0.347673644
ATP8A1	-0.310724	0.150357	-2.066574	0.03877433	0.347673644
ENSG00000258297	-0.334702	0.161963	-2.066529	0.03877852	NA
SLC43A2	-0.214103	0.103614	-2.066342	0.0387962	0.347673644
IRAK1	-0.461991	0.223597	-2.066173	0.03881217	NA
N6AMT1	0.593487	0.287371	2.06523	0.03890129	NA
EIF5	0.128413	0.062195	2.064669	0.03895433	0.348175087
PTRH1	-0.359785	0.174269	-2.064545	0.03896607	NA
ARL11	0.486967	0.235905	2.064245	0.03899447	0.348175087
WBP1L	-0.326554	0.158211	-2.064045	0.03901348	NA
TMEM177	-0.608039	0.294746	-2.062928	0.03911951	NA
SYNJ2BP	0.225364	0.10932	2.061506	0.03925479	0.349860969
WDR89	0.368041	0.178542	2.061373	0.03926751	NA
LINGO3	-0.488141	0.236809	-2.061333	0.03927131	NA
WDR34	-0.307586	0.149274	-2.060554	0.03934562	0.350032955
LINC00562	-0.876489	0.42553	-2.059758	0.03942167	NA

CHD1	0.199472	0.096873	2.059102	0.03948451	0.350537667
LINC01184	0.30932	0.150308	2.057907	0.03959902	NA
FAM117B	-0.390653	0.189837	-2.05784	0.03960545	0.350537667
AMD1	0.219065	0.106464	2.057643	0.03962438	0.350537667
OR2A1.AS1	-0.903568	0.439145	-2.05756	0.03963242	NA
ENSG00000244459	0.454549	0.220972	2.057042	0.03968215	NA
MRNIP	0.249678	0.121386	2.056903	0.0396956	0.350537667
PCGF1	-0.619752	0.301409	-2.056184	0.03976477	NA
MYNN	0.342994	0.166841	2.055813	0.03980058	NA
ACCS	0.704202	0.342662	2.05509	0.03987029	NA
MFSD13A	-0.595192	0.289625	-2.055046	0.03987457	NA
ZNF318	0.424584	0.206615	2.054948	0.03988402	0.350537667
PRCD	1.005907	0.489512	2.054917	0.03988701	NA
ENSG00000267939	-1.514017	0.736942	-2.05446	0.03993119	NA
PCF11	0.173448	0.084427	2.054408	0.03993623	0.350537667
CISD2	0.226925	0.110466	2.054257	0.0399508	0.350537667
CIAO2B	0.135959	0.066205	2.053605	0.04001391	0.350537667
PSMA4	-0.153836	0.074923	-2.053263	0.04004712	0.350537667
MTMR6	-0.357768	0.174268	-2.05297	0.04007547	NA
MBNL1	-0.155684	0.075887	-2.051531	0.04021529	0.351191722
MED28	0.150678	0.073465	2.051016	0.04026539	0.351191722
TMX2	0.453229	0.221065	2.050211	0.04034386	0.351250049
OSGIN2	0.556272	0.27141	2.049563	0.04040713	NA
SNAP23	-0.185228	0.090423	-2.048462	0.04051478	0.352111603
CCDC71	-0.336216	0.16432	-2.046107	0.04074586	NA
LAMTOR3	0.301822	0.147561	2.045407	0.04081478	0.353803136
ENSG00000274265	-0.291366	0.142476	-2.045008	0.04085403	0.353803136
ENSG00000226644	-1.948206	0.953322	-2.043596	0.04099346	NA
SNRPA1	-0.193406	0.094693	-2.042456	0.04110637	0.355274877
SETDB2	-0.373755	0.183112	-2.041126	0.04123831	0.355274877
STMN1	-0.300985	0.147463	-2.041091	0.0412418	0.355274877
GPR137B	0.724951	0.355292	2.040434	0.04130708	NA
ENSG00000276509	0.438521	0.215074	2.038931	0.04145688	NA
ADCK1	-0.918081	0.450328	-2.038695	0.0414805	NA
ASAH1	0.255154	0.125184	2.038236	0.04152637	0.357097544
SWAP70	0.187523	0.092085	2.036417	0.0417085	0.357779075
ANAPC15	-0.322941	0.158617	-2.035985	0.04175186	0.357779075
ZKSCAN4	0.58723	0.288507	2.035412	0.04180939	NA
CLEC16A	-0.483199	0.237403	-2.035348	0.04181585	NA
PPP5C	-0.513872	0.252595	-2.034373	0.04191404	NA
TMSB10	-0.169801	0.083503	-2.033466	0.04200549	0.359323192
ENSG00000261669	0.789415	0.388459	2.032174	0.04213604	NA
S100A13	-0.719974	0.354324	-2.031963	0.0421574	NA
KLHDC7B	-0.68298	0.336208	-2.031421	0.04221227	NA
LSR	0.774424	0.381431	2.030312	0.04232483	NA
VASH1	-1.561613	0.769268	-2.029998	0.04235672	NA
ASB16.AS1	0.324484	0.159889	2.029427	0.04241479	NA

DDX55	0.352222	0.173683	2.027953	0.04256507	NA
RASA2	0.285232	0.14068	2.02752	0.04260929	0.362790343
JTB	-0.100343	0.049505	-2.02695	0.04266748	0.362790343
EIF3J.DT	0.222961	0.110011	2.026714	0.04269162	0.362790343
UNC45A	-0.302401	0.149219	-2.02656	0.04270739	0.362790343
TAPBPL	0.394635	0.194955	2.024241	0.04294541	0.363968582
TENT4B	0.426684	0.210818	2.023949	0.0429754	NA
MPV17	0.284802	0.140798	2.022771	0.04309678	0.363968582
HIF1A	0.212589	0.105106	2.02261	0.04311333	0.363968582
USF1	-0.655907	0.324307	-2.022485	0.04312623	NA
SLC2A14	-3.245464	1.604725	-2.022442	0.04313069	NA
TMX4	-0.307133	0.15194	-2.021409	0.04323746	0.363968582
CD3G	-2.604577	1.288512	-2.021385	0.04323998	NA
RPS6KB2	-0.209756	0.103778	-2.021194	0.04325969	0.363968582
XRRA1	-0.587078	0.290507	-2.020878	0.0432924	0.363968582
GRK2	0.240396	0.119003	2.02009	0.04337402	0.364029253
MRPS36	0.249804	0.123859	2.016843	0.04371187	0.366236594
PSMD9	-0.236012	0.117099	-2.015497	0.04385265	0.366788069
RNF144B	0.705911	0.350565	2.013638	0.04404757	NA
IGHA2	-0.877752	0.435938	-2.013482	0.04406398	NA
DNASE1	0.288262	0.14317	2.013429	0.04406952	0.367972987
C15orf41	-0.748959	0.37202	-2.013223	0.04409114	NA
THG1L	0.428212	0.212839	2.011903	0.04423013	0.368684879
TMBIM6	-0.127894	0.063604	-2.010791	0.04434752	0.369034717
ZNF610	1.261166	0.627364	2.010261	0.04440358	NA
CEP85L	0.639366	0.318148	2.009649	0.0444684	NA
CLDN11	-1.201339	0.598005	-2.008911	0.04454654	NA
IDH1	0.237243	0.118102	2.008803	0.04455803	0.370156968
PRRT3	0.553075	0.275667	2.006314	0.04482279	NA
KYAT3	0.435636	0.217196	2.005726	0.04488553	0.372245622
H3F3C	-0.979094	0.488413	-2.004645	0.04500105	NA
HINT1	0.184085	0.091861	2.003942	0.04507624	0.372521123
AKAP9	0.159574	0.079639	2.003722	0.04509981	0.372521123
PLAC8	0.198523	0.099129	2.002675	0.04521213	0.372521123
PLEKHF1	0.523083	0.261284	2.001973	0.04528764	NA
RALGPS2	0.275388	0.137567	2.001845	0.04530146	0.372521123
ERLIN1	0.571408	0.285494	2.001472	0.04534158	NA
BAX	-0.339753	0.169805	-2.000845	0.04540905	0.372521123
PHF14	0.191864	0.095923	2.000194	0.04547928	0.372521123
SF1	-0.15519	0.077605	-1.999741	0.0455282	0.372521123
CEBPZ	0.213787	0.107002	1.997962	0.04572081	0.372521123
IGKC	-0.318196	0.159269	-1.997848	0.04573311	0.372521123
CERS4	-0.291214	0.145811	-1.997197	0.04580376	0.372521123
ZNF562	-0.236458	0.118396	-1.997188	0.04580478	0.372521123
RPF2	0.271361	0.135919	1.996493	0.04588028	0.372521123
CXorf21	0.288648	0.144596	1.996234	0.04590849	0.372521123
USP5	-0.383487	0.192188	-1.99537	0.04600253	NA

SELENOW	-0.277616	0.139145	-1.995163	0.04602506	0.372610949
HVCN1	-0.201188	0.100869	-1.994549	0.04609209	0.372610949
PLSCR1	-0.297184	0.149084	-1.993406	0.04621705	0.372610949
TACC3	0.312183	0.156641	1.992987	0.04626282	0.372610949
C1orf52	0.224118	0.112472	1.992645	0.04630032	0.372610949
SBNO2	-0.621366	0.311922	-1.992054	0.04636511	NA
FCRL3	-1.05357	0.529041	-1.991471	0.04642917	0.37303437
SLC7A6	0.355313	0.178457	1.991029	0.04647771	NA
WAC	0.1818	0.091381	1.989472	0.04664918	0.374187581
LRRC7	-0.626442	0.314908	-1.989287	0.04666958	NA
ENSG00000258634	-1.012905	0.509289	-1.988863	0.04671633	NA
GPAA1	-0.247995	0.124737	-1.988141	0.0467961	0.374518013
GUK1	-0.187762	0.094518	-1.986531	0.04697437	0.374518013
FBXO25	-0.329246	0.165742	-1.986499	0.04697799	0.374518013
MIB2	0.286571	0.144272	1.986331	0.04699654	0.374518013
ZNF584	0.55274	0.278427	1.985227	0.04711925	NA
SLC2A5	-0.43702	0.220161	-1.985005	0.04714393	0.37500056
KXD1	-0.219631	0.110678	-1.984408	0.04721037	0.37500056
C12orf49	-0.508407	0.25629	-1.983716	0.04728752	0.375004586
CLTA	0.151105	0.076236	1.982064	0.04747204	0.375858695
MRPL38	-0.488016	0.246318	-1.981245	0.0475638	NA
ZNF830	0.229563	0.115879	1.98106	0.04758452	0.376140607
ENTPD1	0.368491	0.186099	1.980075	0.04769515	NA
LY9	0.361945	0.182832	1.979657	0.0477421	0.376228172
PSMD13	-0.215961	0.109094	-1.979592	0.04774938	0.376228172
C17orf58	-0.828588	0.418616	-1.97935	0.04777663	NA
ATP6V0E2	-0.471573	0.238454	-1.977628	0.04797066	NA
GORASP1	0.359941	0.182258	1.974897	0.04827979	NA
MTRF1L	0.45072	0.228277	1.974444	0.04833132	0.379491388
NCF1	-0.15496	0.07853	-1.973248	0.04846736	0.379491388
MOAP1	-0.306584	0.155462	-1.972075	0.04860109	0.379491388
FKBP3	-0.195745	0.099341	-1.970434	0.04878866	0.379491388
SPOCK2	-0.185321	0.094077	-1.969889	0.04885109	0.379491388
ENSG00000272927	-0.575274	0.292074	-1.969613	0.04888276	NA
PTPRC	0.164171	0.083377	1.969021	0.04895071	0.379491388
NFE2L2	0.246302	0.125106	1.968746	0.04898221	0.379491388
C10orf88	0.527299	0.267947	1.967921	0.04907707	NA
TMED2	-0.194692	0.098941	-1.967753	0.04909643	0.379491388
PMF1	-0.233724	0.118784	-1.967644	0.04910899	0.379491388
NSMCE1	-0.266333	0.135418	-1.966742	0.04921302	0.379491388
RHOBTB2	0.823117	0.418569	1.966504	0.04924037	NA
IRAK4	-0.235962	0.119998	-1.966384	0.04925426	0.379491388
PRDX1	0.163638	0.083238	1.965916	0.04930827	0.379491388
PLEKHG1	-0.327123	0.166446	-1.965333	0.04937576	0.379491388
TIMM23	-0.426228	0.216883	-1.965241	0.0493864	NA
PSMB4	-0.153177	0.077946	-1.965166	0.04939501	0.379491388
NRAS	-0.322054	0.163923	-1.964672	0.04945225	0.379491388

MGAT4A	-0.473059	0.240792	-1.964597	0.04946088	NA
FAM222B	0.635691	0.323577	1.964574	0.04946362	NA
SNAPC5	-0.326219	0.166129	-1.963642	0.04957167	0.379491388
TNFRSF13B	0.837147	0.426341	1.963563	0.04958077	0.379491388
IER3	-0.675373	0.344026	-1.963146	0.04962922	NA
MDH2	-0.169608	0.086426	-1.962459	0.04970908	0.379491388
FCMR	0.220933	0.112603	1.962045	0.04975726	0.379491388
RSRC2	0.237868	0.121265	1.961553	0.04981453	0.379491388
MTPN	0.169356	0.086342	1.961454	0.04982603	0.379491388
CPT1A	-0.445588	0.22718	-1.961385	0.04983417	NA
PEX13	0.363368	0.185266	1.961337	0.04983968	NA
SBNO1	0.205894	0.105003	1.960846	0.04989694	0.379491388
TMC6	-0.194462	0.099195	-1.960414	0.04994736	0.379491388

Cluster 5	log2FC	lfcSE	stat	pvalue	padj
NFKBIA	-0.71892	0.066472	-10.815443	< 2.22e-16	< 2.22e-16
LTB	-1.092821	0.119951	-9.110537	< 2.22e-16	4.67E-16
IL13RA1	-1.515699	0.167368	-9.056083	< 2.22e-16	5.13E-16
NCR3	-1.651037	0.186618	-8.847142	< 2.22e-16	2.56E-15
MARCKSL1	-1.022467	0.13046	-7.83741	4.60E-15	1.05E-11
NCF4	-0.707989	0.091842	-7.708787	1.27E-14	2.41E-11
ENSG00000266844	-1.79307	0.233662	-7.673767	1.67E-14	2.72E-11
NT5C	-0.624744	0.089057	-7.015135	2.30E-12	3.27E-09
COCH	-1.311671	0.189993	-6.903799	5.06E-12	5.85E-09
FNBP1	-0.690901	0.100104	-6.901803	5.13E-12	5.85E-09
CTSH	-0.596976	0.089192	-6.69319	2.18E-11	2.26E-08
COL18A1	-2.216711	0.345756	-6.411198	1.44E-10	1.37E-07
SEC62	0.36247	0.05667	6.396209	1.59E-10	1.40E-07
TEX9	-1.715713	0.268807	-6.382699	1.74E-10	1.40E-07
TAGLN2	-0.566962	0.088958	-6.373399	1.85E-10	1.40E-07
SYNGR2	-0.681794	0.108771	-6.268169	3.65E-10	2.46E-07
MAP3K7CL	-1.180419	0.188346	-6.267298	3.67E-10	2.46E-07
BLNK	0.453643	0.074061	6.125238	9.05E-10	5.73E-07
JPT1	-0.733566	0.120115	-6.10718	1.01E-09	6.08E-07
GLIPR1	0.525834	0.086481	6.08031	1.20E-09	6.83E-07
MARCKS	-0.936965	0.155214	-6.036604	1.57E-09	8.53E-07
IL23A	-1.374582	0.228167	-6.024458	1.70E-09	8.78E-07
PDE4D	-1.415602	0.236425	-5.987523	2.13E-09	1.05E-06
CR1	-1.349354	0.231741	-5.822691	5.79E-09	2.61E-06
CYTIP	0.428279	0.073625	5.817029	5.99E-09	2.61E-06
TCTN1	-1.000957	0.172158	-5.814185	6.09E-09	2.61E-06
CPNE5	-0.878492	0.151155	-5.811852	6.18E-09	2.61E-06
CD40	-0.44205	0.076466	-5.780992	7.43E-09	3.02E-06
GPSM3	-0.271399	0.047247	-5.744309	9.23E-09	3.62E-06
PDE4B	0.597773	0.104822	5.702758	1.18E-08	4.47E-06
MFSD10	-0.679551	0.120057	-5.660214	1.51E-08	5.55E-06
LMO4	0.658842	0.116833	5.63918	1.71E-08	6.02E-06
MAL	-2.549903	0.452477	-5.635434	1.75E-08	6.02E-06
PSMB10	-0.488912	0.086967	-5.62182	1.89E-08	6.33E-06
RSRP1	0.463248	0.08278	5.596138	2.19E-08	7.13E-06
ZEB2	0.909023	0.163524	5.558962	2.71E-08	8.58E-06
KLF3	0.735405	0.132995	5.52957	3.21E-08	9.88E-06
AK8	-1.940642	0.354341	-5.476766	4.33E-08	1.30E-05
SAMD12	-2.164757	0.39563	-5.471666	4.46E-08	1.30E-05
RNGTT	-1.123604	0.206787	-5.433616	5.52E-08	1.57E-05
GDPD5	-1.60711	0.296093	-5.427726	5.71E-08	1.58E-05
ENSG00000167414	-2.354116	0.434019	-5.423992	5.83E-08	1.58E-05
LINC00926	-0.569602	0.105633	-5.392254	6.96E-08	1.84E-05
ATF5	-1.472486	0.274234	-5.369446	7.90E-08	2.04E-05
LYPLAL1	-0.841422	0.157202	-5.352487	8.68E-08	2.20E-05
ENSG00000125726	-1.319104	0.246981	-5.340903	9.25E-08	2.25E-05

PPM1K	0.438424	0.082098	5.340248	9.28E-08	2.25E-05
C9orf16	-0.34711	0.065287	-5.316661	1.06E-07	2.51E-05
MIF	-0.464604	0.087502	-5.30965	1.10E-07	2.55E-05
IGHE	-1.649871	0.31125	-5.300791	1.15E-07	2.63E-05
RNF130	1.336643	0.25236	5.296573	1.18E-07	2.63E-05
P2RY8	-0.60655	0.114982	-5.275156	1.33E-07	2.90E-05
PASK	-1.079442	0.205172	-5.261158	1.43E-07	3.04E-05
ATXN1	1.194377	0.227221	5.256467	1.47E-07	3.04E-05
FAM177B	1.398392	0.266041	5.256303	1.47E-07	3.04E-05
RABAC1	0.301912	0.05756	5.245185	1.56E-07	3.17E-05
TENT5A	1.638367	0.312694	5.239527	1.61E-07	3.22E-05
ITGAL	-0.619498	0.119484	-5.184784	2.16E-07	4.25E-05
MRPL54	-0.4646	0.089773	-5.175268	2.28E-07	4.39E-05
DUSP10	1.031837	0.200699	5.141221	2.73E-07	5.18E-05
PFN1	-0.242586	0.047381	-5.119873	3.06E-07	5.71E-05
MYL12A	-0.28048	0.054951	-5.104216	3.32E-07	6.10E-05
MSC	-1.828122	0.358846	-5.094445	3.50E-07	6.32E-05
ETV7	-1.89343	0.372694	-5.080388	3.77E-07	6.70E-05
ZBED2	1.789543	0.354388	5.049672	4.43E-07	7.75E-05
HMGB2	-0.437343	0.086687	-5.045097	4.53E-07	7.82E-05
CD84	1.04783	0.208393	5.028146	4.95E-07	8.39E-05
ATP6V0E2	-0.861012	0.171313	-5.025964	5.01E-07	8.39E-05
TSC22D3	0.46229	0.092505	4.997438	5.81E-07	9.59E-05
CLK1	0.490127	0.098466	4.977616	6.44E-07	0.000104706
PDE3B	-1.263605	0.255067	-4.954014	7.27E-07	0.000115178
GATD3	5.051733	1.0198	4.953651	7.28E-07	0.000115178
CAPN2	-0.779804	0.158235	-4.928151	8.30E-07	0.000129475
SQOR	-0.647609	0.131992	-4.906427	9.28E-07	0.000141929
LINC02397	0.407962	0.083175	4.90487	9.35E-07	0.000141929
CYCS	-0.349476	0.071587	-4.881852	1.05E-06	0.000157447
EPS15	-0.541886	0.111121	-4.876557	1.08E-06	0.000159631
TAGAP	-0.601184	0.124245	-4.838689	1.31E-06	0.000190786
TENT5C	0.897257	0.185677	4.832345	1.35E-06	0.000193421
FAS	-1.17531	0.243289	-4.830924	1.36E-06	0.000193421
UBE2I	-0.281176	0.058348	-4.818924	1.44E-06	0.000202888
DAP	-0.620915	0.12912	-4.808813	1.52E-06	0.000210821
TFEC	1.090121	0.227233	4.797378	1.61E-06	0.000220527
BIK	-1.666123	0.347762	-4.790987	1.66E-06	0.000224959
STX7	0.609687	0.127858	4.768482	1.86E-06	0.000244199
MIIP	-0.483322	0.101365	-4.768128	1.86E-06	0.000244199
NECAP2	-0.42313	0.088754	-4.767429	1.87E-06	0.000244199
TGIF1	0.737375	0.155423	4.744311	2.09E-06	0.000270698
MIR3142HG	-2.031154	0.429	-4.734629	2.19E-06	0.000280756
TTC32	-0.950531	0.200956	-4.730046	2.24E-06	0.000283979
BBX	0.403946	0.08565	4.716256	2.40E-06	0.000300571
BPTF	0.36769	0.078193	4.70234	2.57E-06	0.000318309
OPTN	-0.830357	0.177035	-4.690354	2.73E-06	0.000333196

EHD1	-0.550067	0.11732	-4.688602	2.75E-06	0.000333196
MVP	-0.635706	0.135665	-4.68585	2.79E-06	0.000334149
LCN8	4.670297	0.999921	4.670665	3.00E-06	0.000356081
EBI3	-1.048703	0.225459	-4.651419	3.30E-06	0.000384235
NSF	1.28705	0.27674	4.65076	3.31E-06	0.000384235
MRPL36	-0.441451	0.095013	-4.646196	3.38E-06	0.000388862
CDC42	-0.3975	0.085799	-4.632905	3.61E-06	0.000410544
IL6R	-1.572316	0.341134	-4.609082	4.04E-06	0.000450328
C12orf75	-1.095261	0.237659	-4.608532	4.06E-06	0.000450328
SLC38A2	0.499819	0.108477	4.607584	4.07E-06	0.000450328
PRKCB	-0.435596	0.094686	-4.600411	4.22E-06	0.000461635
TESC	-1.903574	0.41514	-4.585375	4.53E-06	0.000491411
ANXA7	-0.531131	0.116476	-4.559994	5.12E-06	0.000549482
CDKN1A	-0.605111	0.132857	-4.554592	5.25E-06	0.000558524
PLAAT3	-1.588497	0.349143	-4.549707	5.37E-06	0.000566355
CIRBP	-0.20639	0.045417	-4.544288	5.51E-06	0.00057579
NMB	1.413277	0.311416	4.53823	5.67E-06	0.000587191
LRIF1	0.620261	0.136837	4.532836	5.82E-06	0.000596966
HRK	-0.962769	0.212544	-4.529732	5.91E-06	0.000600395
TIMM10	-0.506074	0.111813	-4.526085	6.01E-06	0.000605436
TRADD	-0.545352	0.120647	-4.520236	6.18E-06	0.000616948
JAM3	2.20131	0.487352	4.516881	6.28E-06	0.000621351
IER3	-1.676992	0.372002	-4.508016	6.54E-06	0.000641362
PRELID1	-0.401076	0.088999	-4.506502	6.59E-06	0.000641362
NDUFB8	-0.275559	0.061319	-4.493899	6.99E-06	0.000674772
SUB1	-0.516588	0.116001	-4.453326	8.46E-06	0.000808983
UBE2N	-0.480593	0.108116	-4.445176	8.78E-06	0.00083183
PRDX2	-0.529166	0.119115	-4.442485	8.89E-06	0.00083183
CD48	0.441313	0.09935	4.441992	8.91E-06	0.00083183
TELO2	-0.948111	0.214533	-4.419408	9.90E-06	0.000909001
LGALS3	-0.705779	0.159713	-4.419054	9.91E-06	0.000909001
CERS6	1.83683	0.415796	4.41762	9.98E-06	0.000909001
ENSG00000070423	-0.471663	0.10684	-4.414664	1.01E-05	0.000914198
ISCU	-0.351577	0.079695	-4.411513	1.03E-05	0.000920299
BIN2	-0.695872	0.157885	-4.407465	1.05E-05	0.000928049
ITGB1	-0.845121	0.191863	-4.404812	1.06E-05	0.000928049
LINC02384	-1.561015	0.354402	-4.404639	1.06E-05	0.000928049
RGS2	1.30378	0.296885	4.391533	1.13E-05	0.000978278
RNF207	-1.108342	0.253097	-4.379129	1.19E-05	0.001027798
RORA	-1.253165	0.286475	-4.37443	1.22E-05	0.00104229
DHTKD1	-0.539133	0.123935	-4.350122	1.36E-05	0.001155896
REL	-0.372591	0.085682	-4.348534	1.37E-05	0.001155896
SRSF5	-0.277474	0.063988	-4.336367	1.45E-05	0.001211658
RPP25	1.088914	0.251194	4.334954	1.46E-05	0.001211658
HNRNPDL	-0.247814	0.057206	-4.331965	1.48E-05	0.001219326
PCDH9	1.132925	0.262148	4.321706	1.55E-05	0.001268251
AEN	-0.561603	0.130215	-4.3129	1.61E-05	0.00131042

SLC25A39	-0.355271	0.082594	-4.301436	1.70E-05	0.001370317
DOK2	-1.648405	0.383593	-4.297273	1.73E-05	0.001376875
RAB24	-0.743928	0.173117	-4.297256	1.73E-05	0.001376875
LY86	-0.406567	0.094985	-4.280315	1.87E-05	0.001473152
ENSG00000279483	-0.390649	0.091291	-4.279154	1.88E-05	0.001473152
GBGT1	1.213508	0.28372	4.277137	1.89E-05	0.001476377
ENSG00000227468	-2.268529	0.530628	-4.275174	1.91E-05	0.001479314
CD72	0.772487	0.180785	4.272959	1.93E-05	0.001483994
HLA.C	-0.489519	0.114809	-4.263762	2.01E-05	0.001536067
GPR183	0.380819	0.089458	4.256963	2.07E-05	0.001572961
SPART	0.755645	0.178577	4.231489	2.32E-05	0.001750499
JCHAIN	-0.993386	0.23489	-4.229163	2.35E-05	0.001757054
ENSG00000254397	1.959934	0.465181	4.213275	2.52E-05	0.001870693
RUNX1	1.255564	0.298125	4.211536	2.54E-05	0.001870693
RAC2	-0.386493	0.091803	-4.210016	2.55E-05	0.001870693
NME1	-0.640687	0.152212	-4.209175	2.56E-05	0.001870693
LTA	-1.038379	0.2468	-4.207372	2.58E-05	0.00187367
SLC2A1	-0.610823	0.145305	-4.203714	2.63E-05	0.001892167
MME	-2.93398	0.701418	-4.182924	2.88E-05	NA
CCDC167	-0.587973	0.140669	-4.179833	2.92E-05	0.002089029
CTSB	0.483123	0.116286	4.154622	3.26E-05	0.002318657
PCNX4	0.505502	0.121714	4.153187	3.28E-05	0.002318758
MGAT4A	-0.653717	0.157736	-4.144375	3.41E-05	0.002394872
IL15	-0.730688	0.176544	-4.138835	3.49E-05	0.002438377
BTG2	0.320984	0.077654	4.133534	3.57E-05	0.002480112
ATAD3C	-1.492718	0.362228	-4.120936	3.77E-05	0.00260193
INPP5K	-0.584151	0.141794	-4.119714	3.79E-05	0.00260193
MTERF4	-0.397817	0.096646	-4.116223	3.85E-05	0.002625812
CEMIP2	1.335617	0.325398	4.104567	4.05E-05	0.002740642
PRKCE	0.806417	0.196515	4.10359	4.07E-05	0.002740642
MSMO1	-0.449146	0.109586	-4.098581	4.16E-05	0.002771349
TCAF2	-0.913114	0.222804	-4.09829	4.16E-05	0.002771349
PSME2	-0.39363	0.096446	-4.081339	4.48E-05	0.00296413
SNED1	-1.73388	0.425159	-4.078188	4.54E-05	0.002972762
ENSG00000254614	-1.274299	0.312483	-4.077976	4.54E-05	0.002972762
BAIAP3	-1.864171	0.457757	-4.072402	4.65E-05	0.003018214
AKT3	0.822682	0.202045	4.071785	4.67E-05	0.003018214
CLEC2B	0.66769	0.164375	4.061981	4.87E-05	0.003130062
LCK	-1.067728	0.263622	-4.050217	5.12E-05	0.003273159
TRIM4	0.574367	0.14199	4.04512	5.23E-05	0.003326516
ALDH3A2	1.552086	0.383882	4.043132	5.27E-05	0.003336222
TRAF1	-0.848377	0.210254	-4.035001	5.46E-05	0.003434797
IFNG.AS1	-1.743474	0.433316	-4.023564	5.73E-05	0.00358622
RASGEF1B	0.646113	0.160652	4.021824	5.77E-05	0.003593069
TPI1	-0.195976	0.048749	-4.020068	5.82E-05	0.003600281
MRPL20	-0.354438	0.088215	-4.017885	5.87E-05	0.003614145
NDUFA1	-0.214758	0.053543	-4.010911	6.05E-05	0.003702589

DHCR24	-1.035768	0.25853	-4.00637	6.17E-05	0.003753542
BEX3	-1.603684	0.400405	-4.005156	6.20E-05	0.003753542
VCPKMT	-0.742482	0.186039	-3.991003	6.58E-05	0.003963681
UNC119	-0.502939	0.126081	-3.989002	6.64E-05	0.003976228
ARPC1B	-0.308308	0.077318	-3.987531	6.68E-05	0.003980005
ARID5A	-0.532797	0.133995	-3.976259	7.00E-05	0.004151605
PARM1	-1.706577	0.429904	-3.969666	7.20E-05	0.004246057
PPIA	-0.204724	0.051666	-3.962419	7.42E-05	0.004343208
LYRM2	-0.323613	0.081683	-3.961812	7.44E-05	0.004343208
SMAGP	-0.547338	0.138442	-3.953564	7.70E-05	0.004459315
LILRB1	0.722806	0.182847	3.95307	7.72E-05	0.004459315
ADAMTS6	1.113715	0.282015	3.949132	7.84E-05	0.004489947
PLEKHB1	-1.113053	0.281856	-3.949015	7.85E-05	0.004489947
RIN3	1.063832	0.270059	3.939262	8.17E-05	0.004653036
KYNU	-0.523665	0.133077	-3.935041	8.32E-05	0.004712024
DGKG	2.023743	0.514488	3.933511	8.37E-05	0.004718652
HSD17B11	0.323231	0.08231	3.926971	8.60E-05	0.004814067
UQCRC1	-0.393809	0.1003	-3.926328	8.63E-05	0.004814067
CLDND1	-0.467855	0.11923	-3.923965	8.71E-05	0.004824222
CCDC88C	-0.461745	0.117688	-3.923468	8.73E-05	0.004824222
TRAF4	-0.759875	0.193732	-3.922306	8.77E-05	0.004824222
CKS2	0.610199	0.156108	3.908817	9.27E-05	0.005077127
BMPR2	0.872665	0.223853	3.898387	9.68E-05	0.005275452
ARHGAP24	0.496399	0.127716	3.886756	0.00010159	0.005508253
PSMB3	-0.337931	0.087067	-3.881275	0.00010391	0.005607212
GSTK1	-0.344393	0.088799	-3.878332	0.00010518	0.005643686
ENSG00000273748	-1.197174	0.308757	-3.877403	0.00010558	0.005643686
ATP5MG	-0.184278	0.047551	-3.8754	0.00010645	0.00566372
ENSG00000180953	-0.570995	0.14754	-3.87011	0.00010879	0.005761126
ENSG00000272211	-0.989803	0.25609	-3.865064	0.00011106	0.005854296
RAB30	0.38834	0.100516	3.863478	0.00011178	0.005865311
HTR3A	-0.948673	0.245704	-3.861043	0.0001129	0.005896911
SH2B3	0.858501	0.222596	3.856773	0.00011489	0.005973423
JSRP1	-1.187316	0.308355	-3.850485	0.00011788	0.00610103
KRCC1	0.369182	0.095964	3.84709	0.00011953	0.006158193
CHCHD5	-0.421246	0.109591	-3.84381	0.00012114	0.006210258
TIAM2	-0.896584	0.233357	-3.842115	0.00012198	0.006210258
TSPAN5	-1.020831	0.265723	-3.841718	0.00012218	0.006210258
SEPTIN7	0.33402	0.086994	3.839595	0.00012324	0.006236354
ENSG00000237773	-1.286031	0.335887	-3.828766	0.00012879	0.00648838
FARP2	0.943484	0.246557	3.826635	0.00012991	0.006515959
ARL17A	-0.627796	0.164263	-3.821903	0.00013243	0.006613151
NCBP3	0.993391	0.260259	3.816934	0.00013512	0.006718258
H2AFZ	0.364179	0.09549	3.813814	0.00013684	0.006774108
PSME1	-0.203441	0.053565	-3.79804	0.00014585	0.007188703
FCGR2B	0.580801	0.153105	3.793481	0.00014855	0.007290466
TUBB6	1.021008	0.269288	3.791505	0.00014974	0.00731721

SESN3	0.409815	0.108323	3.783275	0.00015478	0.007531211
C9orf139	-0.773585	0.204571	-3.781496	0.00015589	0.007552987
IFNLR1	1.012013	0.268006	3.776081	0.00015932	0.007686278
POU2AF1	-0.429136	0.114032	-3.763289	0.00016769	0.00805634
CD80	-1.253557	0.333409	-3.759814	0.00017004	0.008134645
NUPL2	-0.451331	0.120135	-3.756849	0.00017207	0.008134645
UROS	-0.448588	0.119409	-3.756742	0.00017214	0.008134645
THAP2	0.706335	0.188021	3.756683	0.00017218	0.008134645
LDLR	-1.468282	0.390974	-3.75545	0.00017303	0.008140992
RBM3	-0.418467	0.111536	-3.751854	0.00017553	0.008221095
HLA.A	-0.401759	0.107109	-3.750935	0.00017618	0.008221095
AKAP9	0.293421	0.078263	3.749181	0.00017741	0.008244991
HLA.DOB	-0.56457	0.150696	-3.746413	0.00017938	0.008302565
DUSP4	1.530337	0.409203	3.739796	0.00018417	0.008489702
TAPBP	-0.322112	0.086181	-3.737642	0.00018575	0.008528211
ZNF593	-0.469603	0.126333	-3.717194	0.00020145	0.009198088
CDC42SE1	-0.34082	0.091703	-3.716552	0.00020196	0.009198088
MYO1G	-0.466258	0.125596	-3.712353	0.00020534	0.009313513
PITPNC1	-0.77814	0.209663	-3.711382	0.00020613	0.009313513
C17orf49	-0.734792	0.198045	-3.71022	0.00020708	0.009319386
RFX5	-0.386112	0.104284	-3.702504	0.00021348	0.009569697
AP1S2	-0.325022	0.087819	-3.701029	0.00021473	0.009587785
FASTK	-0.404668	0.109532	-3.694528	0.0002203	0.009776019
PROCA1	0.940262	0.254563	3.69363	0.00022108	0.009776019
CDC42EP3	-0.401857	0.108812	-3.693121	0.00022152	0.009776019
ENSA	-0.241307	0.065431	-3.687958	0.00022606	0.009904687
TBX21	1.705429	0.462448	3.687831	0.00022617	0.009904687
PSAP	0.453544	0.123301	3.678359	0.00023474	0.010240385
GTF3C6	-0.336782	0.091611	-3.676223	0.00023671	0.01028708
SRGN	0.835118	0.227646	3.668487	0.00024399	0.010563022
IZUMO4	-0.64371	0.175542	-3.666976	0.00024544	0.010585359
SMIM4	-0.504616	0.137788	-3.662253	0.00025001	0.010741791
HLA.DQB2	-2.740139	0.749243	-3.65721	0.00025498	0.010914091
JAZF1	0.526904	0.144158	3.655048	0.00025713	0.010922764
PNPLA8	0.388121	0.106191	3.654954	0.00025723	0.010922764
PPP1R15A	-0.548325	0.150089	-3.653339	0.00025885	0.010922764
IGHEP1	-1.803207	0.493871	-3.651173	0.00026105	0.010922764
FDFT1	-0.409269	0.112099	-3.650959	0.00026126	0.010922764
S1PR2	-0.933203	0.255645	-3.650389	0.00026184	0.010922764
HSBP1	0.303662	0.083187	3.65034	0.00026189	0.010922764
HSD17B10	-0.394918	0.108263	-3.647765	0.00026453	0.010992533
SNX11	-0.54135	0.149281	-3.626387	0.00028741	0.011899993
UBC	-0.363209	0.100212	-3.624415	0.00028962	0.011947729
XYLT1	0.904889	0.249954	3.620221	0.00029435	0.012099217
NFKB2	-0.622232	0.17235	-3.610292	0.00030585	0.012442281
PPIB	-0.228191	0.063206	-3.610241	0.00030591	0.012442281
IER2	-0.324222	0.089817	-3.609817	0.00030641	0.012442281

SLA	0.954793	0.26454	3.609263	0.00030707	0.012442281
SLC25A29	1.465537	0.406432	3.605863	0.00031112	0.012561622
ZBTB38	-0.465897	0.129257	-3.604426	0.00031284	0.0125867
GPR137	-0.461154	0.128109	-3.599705	0.00031858	0.012772318
PLPP5	-0.521692	0.145216	-3.592525	0.00032749	0.013083462
BBC3	-0.900152	0.250709	-3.590419	0.00033015	0.013143361
FRG1	-0.340532	0.094868	-3.589513	0.0003313	0.013143361
POU2F2	0.419557	0.116931	3.588068	0.00033314	0.01317051
CDCA7	-1.062675	0.296639	-3.582386	0.00034047	0.013413833
GMFG	-0.276363	0.07726	-3.577036	0.00034751	0.013644073
S100A11	0.27984	0.078346	3.571842	0.00035448	0.013869743
MKNK2	-0.367468	0.102974	-3.568537	0.00035898	0.013997784
NOP10	0.191403	0.053745	3.561286	0.00036904	0.014341053
CYBC1	-0.282816	0.079436	-3.560304	0.00037043	0.014345789
SPIB	-0.312259	0.087733	-3.559177	0.00037202	0.014348554
MRPL32	0.373592	0.104987	3.558473	0.00037302	0.014348554
GDAP1	-1.097204	0.308418	-3.557515	0.00037438	0.01435246
JUNB	-0.328895	0.092565	-3.55311	0.00038071	0.014546006
PRAG1	1.039112	0.293124	3.544956	0.00039268	0.014953326
DBI	-0.272757	0.076967	-3.543821	0.00039437	0.014967743
ARHGDIA	-0.261219	0.073755	-3.541689	0.00039757	0.015039133
XRN2	-0.324589	0.091984	-3.528739	0.00041755	0.015742283
SP100	-0.210048	0.059607	-3.523883	0.00042527	0.015980657
SNX9	0.504959	0.143775	3.512144	0.00044451	0.016648566
COPS3	-0.438486	0.125042	-3.506716	0.00045367	0.016936113
ETFDH	-0.686295	0.195832	-3.504511	0.00045745	0.017021221
ZNF92	-0.418735	0.119718	-3.497688	0.00046931	0.017405724
DHRS4L2	0.469958	0.134427	3.496011	0.00047227	0.017405841
SMC6	0.406123	0.116189	3.495363	0.00047342	0.017405841
ARHGAP11A	-1.324187	0.378948	-3.494373	0.00047518	0.017405841
SF1	-0.201141	0.057564	-3.494232	0.00047543	0.017405841
CSGALNACT2	-0.592531	0.169657	-3.492518	0.00047849	0.017461779
SRGAP2B	0.800993	0.229587	3.488849	0.00048511	0.017646671
TMEM131	0.548611	0.157307	3.487515	0.00048753	0.017678473
RSBN1L	0.294878	0.084577	3.486501	0.00048938	0.017689286
LPP	0.516929	0.148315	3.485352	0.00049149	0.017709217
CYB5A	-0.510126	0.14665	-3.478517	0.0005042	0.018097196
ANXA6	-0.455104	0.130858	-3.477858	0.00050544	0.018097196
ELOB	-0.164296	0.047302	-3.473362	0.00051398	0.018299125
PYHIN1	0.91661	0.263909	3.4732	0.00051429	0.018299125
OSBPL8	0.359701	0.10363	3.471009	0.00051851	0.018309299
GBP2	0.567638	0.163539	3.470958	0.00051861	0.018309299
MRPL23	-0.276036	0.079537	-3.470546	0.0005194	0.018309299
EPG5	0.897097	0.258633	3.468615	0.00052315	0.018368884
VPREB3	-0.377534	0.108883	-3.467324	0.00052567	0.018368884
CBLB	0.471282	0.135933	3.467003	0.0005263	0.018368884
RNH1	-0.458489	0.132268	-3.466366	0.00052755	0.018368884

SLCO5A1	-1.501682	0.433405	-3.464847	0.00053053	0.018416594
AKAP2	-0.844598	0.24445	-3.455089	0.00055011	0.019001571
TBC1D9	0.437045	0.126504	3.45479	0.00055072	0.019001571
UBE2J2	-0.3264	0.094519	-3.453254	0.00055387	0.019052363
ETF1	-0.519935	0.150822	-3.44735	0.00056611	0.019405843
TMSB10	-0.33219	0.09638	-3.446665	0.00056755	0.019405843
MLST8	-0.456734	0.13257	-3.445235	0.00057056	0.019450406
SIPA1L1	0.391054	0.113936	3.432216	0.00059867	0.020347616
ICAM1	-0.964375	0.281056	-3.431252	0.0006008	0.020359328
B4GALT1	0.448101	0.130639	3.430075	0.00060342	0.020387202
TOMM5	-0.341442	0.099658	-3.426135	0.00061224	0.020579794
CHORDC1	0.519388	0.151614	3.425732	0.00061314	0.020579794
PLGRKT	-0.606904	0.177192	-3.425116	0.00061454	0.020579794
ENSG00000265218	-1.892601	0.553822	-3.417347	0.00063235	0.02111406
IKBKE	-0.678227	0.198657	-3.414055	0.00064004	0.021308339
LRP5	-1.142803	0.335676	-3.404488	0.00066288	0.022004607
ANXA2	-0.440372	0.129495	-3.400687	0.00067217	0.022114415
ENSG00000263264	-0.92534	0.272148	-3.400131	0.00067354	0.022114415
ST13	0.204294	0.060085	3.400093	0.00067363	0.022114415
PSMA4	-0.256203	0.075355	-3.399959	0.00067396	0.022114415
SLC9A3R1	-0.49888	0.146787	-3.398658	0.00067717	0.022156028
ZNF107	0.89779	0.264276	3.397167	0.00068087	0.022213246
COX7B	-0.214087	0.06319	-3.387977	0.0007041	0.022905404
GSAP	0.528	0.155893	3.38694	0.00070677	0.022926695
ECH1	-0.321083	0.09494	-3.381965	0.00071969	0.023238509
NDUFS6	-0.269139	0.079588	-3.381671	0.00072046	0.023238509
ENSG00000225885	-0.765194	0.226331	-3.380859	0.0007226	0.023241437
CRK	0.455281	0.134755	3.378579	0.00072862	0.023369039
PHF23	0.475035	0.140706	3.376088	0.00073524	0.023506135
ZNF667.AS1	1.160232	0.343729	3.375425	0.00073702	0.023506135
CCND3	0.433057	0.128696	3.36496	0.00076555	0.024347873
PIK3R5	-0.650399	0.193354	-3.363778	0.00076883	0.024384231
ARL3	0.52282	0.155575	3.360561	0.00077784	0.024601464
LNPEP	0.399213	0.119061	3.353023	0.00079934	0.025211341
IL2RG	-0.378966	0.113136	-3.349656	0.00080912	0.02537616
BEX2	-0.53007	0.158265	-3.349254	0.0008103	0.02537616
ENSG00000225205	-1.188039	0.354752	-3.348927	0.00081125	0.02537616
IQGAP2	1.244571	0.371821	3.347233	0.00081623	0.025461803
MAP1LC3A	-0.902166	0.269606	-3.346239	0.00081916	0.025466083
SLC2A14	-4.275329	1.277715	-3.346074	0.00081964	NA
BMS1	-0.473971	0.141667	-3.345671	0.00082084	0.025466083
PHF20L1	0.426236	0.127438	3.344644	0.00082388	0.02549109
ZMIZ2	-0.487947	0.145943	-3.343412	0.00082755	0.025535163
E2F5	0.522862	0.156691	3.336895	0.0008472	0.02605688
VPREB1	-1.733113	0.519473	-3.336294	0.00084903	0.02605688
RNF144B	1.166389	0.350067	3.331899	0.00086256	0.026400685
GSN	-0.661515	0.198725	-3.328791	0.00087224	0.026625509

COX7A2	-0.195227	0.058689	-3.326473	0.00087953	0.026776121
ARHGDIB	-0.256681	0.077208	-3.32453	0.00088568	0.026891548
TNFAIP8L2	0.791866	0.238307	3.322884	0.00089092	0.026978754
GPX1	-0.309029	0.093034	-3.321661	0.00089483	0.027017656
MGME1	0.493475	0.148592	3.321002	0.00089695	0.027017656
CD247	-1.377388	0.414854	-3.320175	0.00089961	0.027026334
LARP4B	0.684876	0.206651	3.314175	0.00091914	0.027540365
LINC01215	-0.614931	0.185652	-3.312271	0.00092542	0.0275413
LILRA4	2.046829	0.618135	3.311297	0.00092865	0.0275413
ACCS	1.931609	0.583368	3.311133	0.00092919	0.0275413
SPN	-1.28746	0.388879	-3.310696	0.00093064	0.0275413
PILRB	0.763414	0.230603	3.310508	0.00093127	0.0275413
MCRIP2	-0.488978	0.147761	-3.309236	0.00093551	0.027595114
CALM1	-0.228988	0.069218	-3.308229	0.00093888	0.027622951
BRI3BP	0.750604	0.227153	3.304398	0.00095181	0.0279311
APPBP2	0.625292	0.189389	3.301626	0.00096126	0.028136033
ACSF3	-0.476407	0.144358	-3.300163	0.00096629	0.028210572
IMPDH2	-0.473379	0.143541	-3.297864	0.00097423	0.028369891
CDC37	-0.242775	0.073733	-3.292608	0.00099263	0.028831751
NCF2	-0.903088	0.274462	-3.290394	0.00100047	0.02891928
NEK6	-0.566778	0.172256	-3.290324	0.00100072	0.02891928
TRMT1L	0.689834	0.209749	3.288849	0.00100598	0.028941489
RAB3GAP2	-0.426221	0.129602	-3.288683	0.00100657	0.028941489
TOP1	0.350661	0.106731	3.285459	0.00101816	0.029200984
SLC11A1	1.645782	0.501606	3.281025	0.00103431	0.029537407
HMGB3	-1.157207	0.352744	-3.280587	0.00103591	0.029537407
TRAK1	0.540801	0.164873	3.280108	0.00103768	0.029537407
PECAM1	0.95561	0.2914	3.279372	0.00104039	0.029540693
CYTH1	-0.326908	0.099812	-3.27525	0.00105569	0.029900601
LPAR5	0.474235	0.145007	3.270422	0.00107387	0.030279412
PPBP	3.518736	1.076368	3.269082	0.00107897	0.030279412
UBE2F	0.437476	0.133823	3.26906	0.00107905	0.030279412
TNRC6B	0.228769	0.069984	3.268891	0.0010797	0.030279412
COX6C	-0.20603	0.063053	-3.267562	0.00108478	0.030347193
VAV2	-0.472982	0.144785	-3.266786	0.00108776	0.030355946
ABI2	0.503394	0.154252	3.263447	0.00110066	0.030640813
TIGIT	-1.440586	0.441662	-3.261742	0.0011073	0.030715445
TTC39C	-0.544721	0.167022	-3.261375	0.00110873	0.030715445
ENSG00000228037	-0.86523	0.265862	-3.254433	0.00113619	0.031376644
ERG28	-0.334099	0.102675	-3.253953	0.00113811	0.031376644
ENSG00000266088	-2.765873	0.850836	-3.25077	0.00115093	0.031653298
HIPK2	-0.69548	0.214074	-3.248789	0.00115897	0.031797775
SEPTIN9	-0.206281	0.063552	-3.245875	0.0011709	0.032047849
VARS	-0.7707	0.237528	-3.244665	0.00117589	0.032107118
DICER1	0.47189	0.145505	3.243119	0.00118229	0.032204678
GCC2	0.263941	0.081584	3.235187	0.00121563	0.033033846
HECA	0.52866	0.163499	3.233423	0.00122316	0.03315938

NUDT3	0.410734	0.127065	3.232463	0.00122728	0.033191945
ARPC3	-0.223343	0.069117	-3.23137	0.00123199	0.03324025
GFOD1	0.642739	0.199025	3.229435	0.00124035	0.033386835
HSPB1	0.644217	0.199795	3.224397	0.00126238	0.03389974
MIAT	-0.946602	0.293811	-3.221806	0.00127385	0.034127236
C1GALT1	0.463656	0.144112	3.217327	0.00129391	0.03458322
CSF2RB	-0.779514	0.242349	-3.216498	0.00129765	0.034602045
ENO1	-0.268627	0.08367	-3.210559	0.00132477	0.035159365
AFF3	0.35331	0.110053	3.210356	0.00132571	0.035159365
ENSG00000256092	-0.725202	0.225927	-3.209899	0.00132782	0.035159365
BHLHE41	0.876037	0.273015	3.208754	0.00133312	0.035217772
TERF2IP	0.289011	0.090155	3.205706	0.00134732	0.035510489
ZSCAN2	-0.822131	0.256933	-3.199782	0.00137532	0.036164762
ZNF397	0.367951	0.115085	3.197216	0.00138761	0.036403988
KRTCAP2	-0.25686	0.080371	-3.195915	0.00139388	0.036484404
GGCX	-0.552087	0.172815	-3.194668	0.00139992	0.036558373
APOBEC3H	-1.612023	0.504789	-3.193459	0.00140579	0.036572935
TIMM22	-0.423203	0.132535	-3.193136	0.00140737	0.036572935
ODF3B	-0.577629	0.180929	-3.192574	0.00141011	0.036572935
ENSG00000089127	0.815878	0.255743	3.190222	0.00142164	0.036788092
ZNF568	0.632978	0.19859	3.187368	0.00143574	0.037068839
TCF7	-0.731877	0.229794	-3.184932	0.00144788	0.037238047
BTNL9	-1.368689	0.429765	-3.184741	0.00144884	0.037238047
ZBTB24	-0.399384	0.125471	-3.183092	0.00145711	0.037366376
GRAMD1C	-0.730446	0.229819	-3.178347	0.00148117	0.037842992
ATP5MC3	-0.215307	0.06775	-3.177934	0.00148329	0.037842992
RHOH	0.270367	0.085089	3.177469	0.00148567	0.037842992
GALM	1.009233	0.317747	3.176216	0.0014921	0.037903715
ATP5PD	-0.204509	0.064398	-3.175709	0.00149471	0.037903715
BTK	0.325831	0.102645	3.174334	0.00150181	0.037999086
S100PBP	0.554882	0.17516	3.167861	0.00153565	0.038769207
RASSF6	-1.35162	0.426887	-3.166223	0.00154432	0.038901936
FBXO21	-0.364587	0.115284	-3.162506	0.00156418	0.039315004
HDAC1	-0.335387	0.106125	-3.160301	0.00157606	0.039357016
EIF4E3	-1.778117	0.56269	-3.160026	0.00157755	0.039357016
PEX6	0.983579	0.311264	3.159947	0.00157798	0.039357016
TMEM138	-0.359424	0.113755	-3.159634	0.00157967	0.039357016
ADA	-0.461264	0.146165	-3.15577	0.00160075	0.039795011
FCRL2	0.629622	0.199653	3.15359	0.00161276	0.040006199
CCDC137	0.438063	0.139016	3.151165	0.00162621	0.040219553
NOP14.AS1	-0.847462	0.26897	-3.150767	0.00162842	0.040219553
MINDY2	0.496228	0.15754	3.149849	0.00163355	0.040258786
ENSG00000282988	1.153982	0.366578	3.147989	0.00164398	0.040422313
PSMD12	-0.372519	0.118358	-3.147402	0.00164728	0.040422313
ABI1	0.34905	0.110982	3.145097	0.00166032	0.040575571
ITGB3BP	-0.474249	0.150822	-3.144421	0.00166416	0.040575571
KLHL8	0.855985	0.272224	3.14441	0.00166422	0.040575571

PPCS	-0.281687	0.089653	-3.141954	0.00167824	0.040830061
SPTLC2	0.58733	0.187033	3.14024	0.00168809	0.040904716
TLE3	-0.332787	0.105977	-3.14017	0.0016885	0.040904716
IGLC2	-0.591701	0.188616	-3.137058	0.00170652	0.041253614
ATP5MGL	-0.727537	0.232167	-3.133681	0.00172629	0.041642982
KIAA0040	0.520626	0.166203	3.132468	0.00173343	0.04172702
DHRS4	-0.75256	0.24034	-3.131239	0.00174071	0.041740335
TRIP10	0.845749	0.270109	3.131135	0.00174132	0.041740335
ENSG00000267892	1.375582	0.43971	3.128385	0.0017577	0.042044429
ATP1B3	-0.338561	0.108375	-3.123973	0.00178427	0.042590497
NDUFA6	-0.289766	0.092857	-3.120546	0.00180516	0.042976725
GPR18	0.731535	0.234473	3.119907	0.00180908	0.042976725
GRK6	-0.571606	0.183254	-3.119203	0.00181341	0.042976725
PSMB1	-0.219841	0.070498	-3.118381	0.00181848	0.042976725
INSIG1	-0.299885	0.096171	-3.118244	0.00181932	0.042976725
RHOF	-0.334095	0.10717	-3.117429	0.00182436	0.043006593
UGT8	0.822748	0.264019	3.116246	0.00183169	0.043090166
PRICKLE1	1.081873	0.347291	3.115178	0.00183834	0.043157417
PRDM2	-0.23076	0.074137	-3.112608	0.00185442	0.04344541
SPOP	0.353455	0.113648	3.110076	0.00187039	0.043627046
CD82	-0.294294	0.094637	-3.10971	0.00187271	0.043627046
CEP104	0.585137	0.188179	3.109466	0.00187426	0.043627046
ZNF557	0.648745	0.20867	3.108955	0.0018775	0.043627046
TBC1D5	0.396361	0.127634	3.105457	0.00189985	0.044056503
PRKAG1	-0.348097	0.112122	-3.104618	0.00190525	0.04407319
NAA10	-0.297051	0.095695	-3.104142	0.00190832	0.04407319
JHY	-1.440905	0.464432	-3.102508	0.00191888	0.044227506
MXD4	0.587444	0.189399	3.101619	0.00192466	0.044270961
CCSER2	-0.379902	0.122975	-3.089269	0.0020065	0.04596086
ARID4A	0.380401	0.123148	3.088959	0.00200859	0.04596086
HLA.DRA	-0.278408	0.090137	-3.088716	0.00201023	0.04596086
PRDX6	-0.242524	0.078535	-3.088104	0.00201438	0.045963413
MPP6	0.987857	0.320037	3.086695	0.00202395	0.046089365
ANO9	-0.732422	0.237331	-3.086085	0.00202811	0.046091833
ORMDL3	-0.46271	0.150016	-3.084393	0.00203968	0.046262606
PRDM8	-1.004486	0.326211	-3.079251	0.00207522	0.046975054
HIKESHI	-0.291716	0.094784	-3.077703	0.00208603	0.047125973
SNX17	-0.249171	0.081039	-3.074716	0.00210703	0.047506214
TMEM123	-0.344383	0.112123	-3.07147	0.00213007	0.047930861
PSME3	-0.45979	0.149789	-3.069577	0.00214362	0.048140598
NEAT1	0.405704	0.132205	3.068761	0.00214948	0.048177177
ENSG00000272316	0.665408	0.216949	3.067126	0.00216128	0.048277013
ABI3	0.378228	0.123323	3.066968	0.00216242	0.048277013
SLC3A2	-0.329094	0.107344	-3.065791	0.00217095	0.048372662
GCSAM	-0.866975	0.282989	-3.06364	0.00218662	0.048626681
ACAT1	-0.578819	0.189	-3.062534	0.00219471	0.048654094
SLC38A7	-0.797824	0.260531	-3.062304	0.0021964	0.048654094

ENSG00000130313	-0.214832	0.070193	-3.060583	0.00220906	0.048839567
TMEM159	0.518281	0.169631	3.055337	0.00224808	0.049543481
SMS	-0.338219	0.110711	-3.054975	0.00225079	0.049543481
SIDT1	0.684582	0.224119	3.054554	0.00225395	0.049543481
PWWP3A	0.426623	0.1397	3.053847	0.00225928	0.049564768
PRR11	-1.184217	0.387931	-3.052648	0.00226832	0.049667439
TNS3	0.923576	0.302722	3.050907	0.00228151	0.049775167
SQLE	-0.380744	0.1248	-3.050845	0.00228198	0.049775167
PPP1R7	-0.281687	0.092361	-3.049863	0.00228946	0.049842808
FBXL8	-1.027488	0.336971	-3.049191	0.00229459	0.049859113
SPINK2	-1.844774	0.605083	-3.048796	0.00229761	NA
TBC1D4	-2.118487	0.695017	-3.048111	0.00230285	0.049943366
TYROBP	1.486227	0.48788	3.046297	0.00231679	0.05011008
MSH6	-0.589508	0.193537	-3.045966	0.00231934	0.05011008
DNAJB11	-0.398755	0.130955	-3.044976	0.00232699	0.050180045
HIST1H4C	0.309291	0.101688	3.041562	0.00235354	0.050592485
BARD1	0.658194	0.216413	3.041376	0.002355	0.050592485
ATP5IF1	-0.212162	0.069777	-3.040583	0.00236121	0.050630363
COA6.AS1	-0.780178	0.256944	-3.036367	0.00239448	0.051247195
CLIP2	-0.678081	0.22355	-3.033239	0.00241944	0.051684223
ATP5ME	-0.168229	0.055502	-3.031019	0.0024373	0.051968398
GADD45GIP1	-0.19177	0.063294	-3.029846	0.00244679	0.05203339
ARNTL2	-1.247441	0.411763	-3.029512	0.00244949	0.05203339
FLNA	-0.425828	0.140617	-3.028286	0.00245946	0.052080578
THEMIS2	0.471911	0.155843	3.028113	0.00246086	0.052080578
SNHG15	-0.360456	0.119065	-3.027388	0.00246677	0.052108858
CWC15	-0.288097	0.095257	-3.024432	0.002491	0.052523216
FCRLA	0.419826	0.138908	3.022322	0.00250843	0.052690508
EBP	-0.510441	0.168902	-3.022112	0.00251017	0.052690508
CSNK1G3	-0.321272	0.106318	-3.021794	0.00251282	0.052690508
ENSG00000268400	-1.566919	0.51868	-3.020976	0.00251961	0.052735815
DUSP18	0.503377	0.16674	3.01894	0.00253661	0.052965177
PTPRS	1.28675	0.426281	3.01855	0.00253987	0.052965177
DDX6	-0.335708	0.111327	-3.01551	0.00256547	0.053401206
KLF9	0.939208	0.31194	3.010864	0.00260506	0.054126247
IL4I1	-2.298578	0.76377	-3.009515	0.00261665	NA
MARCHF1	0.244377	0.081286	3.006383	0.00264376	0.054701061
ALDH2	1.171226	0.389603	3.006201	0.00264534	0.054701061
TCOF1	-0.319405	0.106256	-3.005995	0.00264714	0.054701061
ZNF322	0.613197	0.20406	3.004988	0.00265591	0.054782938
KDM4B	0.545755	0.181734	3.003036	0.002673	0.055029185
CLCF1	0.624362	0.207946	3.002523	0.00267751	0.055029185
CBR1	0.478737	0.159509	3.001321	0.00268811	0.055049355
FAM53C	0.496628	0.16547	3.001315	0.00268817	0.055049355
THOC3	-0.472203	0.157434	-2.999377	0.00270532	0.055197168
NFE2L1	-0.440317	0.146821	-2.998997	0.0027087	0.055197168
LST1	-1.043603	0.348	-2.998859	0.00270993	0.055197168

PHPT1	-0.400367	0.133559	-2.997672	0.0027205	0.055289573
ROCK1	0.236526	0.078914	2.997261	0.00272417	0.055289573
UQCRFS1	-0.206894	0.069044	-2.996552	0.00273052	0.055319654
PWP1	-0.303678	0.101418	-2.99432	0.00275058	0.055568655
CRYBG1	-0.440193	0.14702	-2.994099	0.00275257	0.055568655
CCDC15	-1.513863	0.505808	-2.992958	0.00276288	0.05567816
MRPL47	-0.31048	0.10376	-2.992296	0.00276888	0.055700369
PFKP	-0.889787	0.297563	-2.990244	0.00278754	0.055977004
CHMP6	-0.463733	0.155111	-2.989694	0.00279257	0.055979281
TRAC	-1.063878	0.356124	-2.987385	0.00281375	0.056304707
JDP2	0.795309	0.266737	2.981627	0.00286721	0.057273733
IDH2	-0.374895	0.125799	-2.980115	0.0028814	0.057456462
PLIN3	-0.418571	0.140527	-2.978579	0.00289588	0.057644216
APOBEC3B	-1.890287	0.634768	-2.977918	0.00290214	0.057655006
ADD3	0.333771	0.112099	2.977452	0.00290655	0.057655006
DMAC2L	-0.344806	0.115988	-2.972765	0.0029513	0.058440871
SHLD2	-0.505355	0.170053	-2.971748	0.00296109	0.05853302
CD200R1	1.302202	0.438373	2.970532	0.00297285	0.058663498
ENSG00000271204	0.470375	0.158381	2.969899	0.00297898	0.058682718
SIGLEC5	1.38168	0.465603	2.967508	0.00300224	0.058992494
NOL4L	-1.221195	0.411562	-2.96722	0.00300506	0.058992494
SH3PXD2A	0.875675	0.29529	2.965475	0.00302216	0.059225906
GHITM	-0.269114	0.090793	-2.964053	0.00303616	0.059398214
ZNF487	-1.133078	0.382412	-2.962978	0.00304678	0.059461606
CARD6	1.627546	0.549442	2.962181	0.00305468	0.059461606
SARS	-0.297979	0.100604	-2.961899	0.00305748	0.059461606
ACP1	-0.227657	0.076869	-2.961616	0.00306029	0.059461606
BMERB1	1.329343	0.448973	2.960853	0.00306789	0.059507599
TUBGCP5	-0.743719	0.25128	-2.959728	0.00307911	0.059623749
NDUFA13	-0.253407	0.085655	-2.958472	0.00309169	0.059692493
IGHM	0.388805	0.13143	2.95826	0.00309381	0.059692493
ZC3H7A	-0.401955	0.135897	-2.957804	0.00309839	0.059692493
GLS	0.379668	0.128426	2.956324	0.0031133	0.059878347
RAB37	-0.445779	0.150818	-2.955747	0.00311913	0.059889444
NUMA1	0.482943	0.163555	2.952792	0.00314914	0.060363869
MYL9	-1.078571	0.365363	-2.952056	0.00315666	0.060406177
ALG14	-0.747938	0.25342	-2.95138	0.00316357	0.060436973
SURF2	-0.350091	0.118686	-2.949731	0.00318051	0.060603768
MORN3	1.611547	0.546422	2.949272	0.00318523	0.060603768
RFTN1	-0.547239	0.185569	-2.948978	0.00318827	0.060603768
SMAP2	-0.300108	0.101838	-2.946927	0.00320949	0.060905369
JUP	-0.980592	0.333205	-2.942913	0.00325139	0.061569496
RHOB	0.688492	0.234074	2.941346	0.00326789	0.061569496
CBL	0.517882	0.176072	2.941312	0.00326825	0.061569496
FLNB	-0.770206	0.261873	-2.941146	0.00327001	0.061569496
CD27	-0.650417	0.221155	-2.941002	0.00327152	0.061569496
LINC00662	0.461305	0.156886	2.940381	0.00327809	0.061591256

TGFBR2	0.354239	0.120599	2.937339	0.00331043	0.062096372
RANBP1	-0.26969	0.091927	-2.933741	0.00334903	0.062717239
KIF20B	0.40725	0.13886	2.932808	0.00335912	0.062802844
NT5E	-0.738595	0.251917	-2.931896	0.003369	0.06287666
CPTP	-0.478205	0.163131	-2.931425	0.00337411	0.06287666
ENSG00000256950	-1.659156	0.566319	-2.929721	0.00339267	0.062999382
BACH2	0.402904	0.137538	2.929401	0.00339616	0.062999382
ELP3	-0.548845	0.187364	-2.929297	0.0033973	0.062999382
TNFRSF25	-1.269731	0.433729	-2.927479	0.00341722	0.063265797
VASP	-0.270589	0.092467	-2.926328	0.0034299	0.063397389
YPEL2	0.465132	0.159013	2.925128	0.00344315	0.063539155
EID1	-0.285194	0.097577	-2.922763	0.0034694	0.063878302
EIF3J.DT	0.424513	0.145259	2.922463	0.00347274	0.063878302
SELL	-0.706237	0.241737	-2.921509	0.0034834	0.063933475
DND1	1.633138	0.559066	2.92119	0.00348697	0.063933475
KPNB1	0.21022	0.072127	2.914571	0.00356178	0.065200029
ATP5F1EP2	-0.332282	0.114033	-2.91392	0.00356922	0.065231343
ZNF761	-0.676865	0.232377	-2.912787	0.00358219	0.065358413
HACD4	-0.525487	0.180436	-2.912311	0.00358765	0.065358413
CDK6	-1.1203	0.384875	-2.910816	0.00360486	0.065566968
SCLT1	-0.810927	0.27874	-2.909261	0.00362284	0.065788908
DEF8	-0.547669	0.188315	-2.908261	0.00363445	0.065894717
FAM120A	0.336518	0.115747	2.907369	0.00364483	0.06597784
ZNF829	0.772097	0.265852	2.904242	0.00368143	0.066534612
PGM3	0.861649	0.296755	2.903574	0.0036893	0.066571097
NAPA	-0.348671	0.120191	-2.90098	0.00371998	0.067018455
NME4	0.831776	0.286822	2.899976	0.00373191	0.067127294
ITPRIPL2	-0.79593	0.275407	-2.890016	0.00385223	0.069182076
ZNF318	0.599624	0.207595	2.888429	0.00387172	0.069422617
CCSAP	1.15084	0.398558	2.88751	0.00388305	0.069516319
PTP4A3	-1.023515	0.354577	-2.886583	0.0038945	0.069611928
GGA2	0.334712	0.116154	2.881624	0.00395632	0.070605783
NDUFS8	-0.244765	0.084958	-2.881025	0.00396384	0.070605783
TMEM179B	-0.249462	0.0866	-2.880638	0.00396871	0.070605783
MYBL2	-1.489436	0.517182	-2.87991	0.00397789	0.070658782
ADAP2	1.015507	0.352998	2.87681	0.00401717	0.071245304
BDP1	0.292432	0.101731	2.874562	0.00404589	0.071643014
ANXA5	-0.46511	0.161872	-2.873312	0.00406193	0.071781437
ENSG00000235078	0.896536	0.312059	2.872971	0.00406631	0.071781437
EPHA4	-0.976376	0.340005	-2.871648	0.00408337	0.071970983
MKKS	-0.326334	0.113717	-2.869707	0.00410853	0.07230243
AUP1	-0.233903	0.081583	-2.867057	0.00414309	0.072798179
POLE3	-0.264611	0.092483	-2.861173	0.00422076	0.07396129
EMC9	0.694872	0.242872	2.86106	0.00422228	0.07396129
BLCAP	-0.297613	0.104074	-2.859634	0.00424131	0.074180543
RUNX3	-0.317306	0.11101	-2.858363	0.00425833	0.07426587
SERPINB9	-0.299445	0.104763	-2.858296	0.00425923	0.07426587

PFDN4	-0.208099	0.072836	-2.857097	0.00427535	0.074432993
PXMP2	-0.661705	0.231829	-2.854281	0.00431344	0.074981422
NFE2L2	0.293004	0.102713	2.85265	0.00433563	0.075252237
RAB40B	1.239021	0.434735	2.85006	0.0043711	0.075644013
DUSP14	0.679096	0.238277	2.850032	0.00437149	0.075644013
ABCB4	0.519646	0.182436	2.848366	0.00439444	0.075824917
DGCR6	-1.482061	0.520331	-2.848306	0.00439526	0.075824917
NOC3L	0.414398	0.14567	2.844782	0.00444418	0.076552833
TUBA1C	-0.597921	0.21024	-2.843995	0.00445517	0.076626248
RPL8	0.270633	0.095288	2.84017	0.00450895	0.077434275
LEPROT	0.417224	0.14695	2.839224	0.00452234	0.077487244
PIGC	-0.366835	0.129213	-2.838991	0.00452565	0.077487244
CDC40	0.267333	0.09419	2.838236	0.00453637	0.077546963
PURA	0.442038	0.155769	2.837786	0.00454276	0.077546963
CD58	-0.361033	0.12729	-2.8363	0.00456396	0.077792298
KCNK6	0.477573	0.168457	2.834987	0.00458275	0.077833143
MAPRE2	0.423115	0.149248	2.834972	0.00458297	0.077833143
SOX5	1.079063	0.380662	2.834701	0.00458687	0.077833143
ASF1A	0.378515	0.133596	2.833286	0.00460722	0.077952786
N4BP3	-0.668229	0.235852	-2.833259	0.00460761	0.077952786
PPP2R5C	-0.261105	0.092224	-2.831216	0.00463713	0.078229583
RENBP	0.730978	0.25821	2.830942	0.00464111	0.078229583
LY6E	0.378336	0.133654	2.830703	0.00464458	0.078229583
S1PR1	0.426963	0.15087	2.830015	0.00465459	0.078282292
EIF3A	-0.209387	0.074005	-2.829343	0.00466437	0.078331167
PTPN18	0.502422	0.17767	2.827829	0.00468648	0.078586581
ERP29	0.158063	0.05591	2.827112	0.00469699	0.078647005
CYP3A5	0.738678	0.261374	2.826136	0.00471133	0.078771198
TRA2B	-0.200956	0.07112	-2.82558	0.00471951	0.078792346
SLC19A1	-1.281506	0.454236	-2.821234	0.00478393	0.079750793
ROR1	1.204433	0.42699	2.820752	0.00479112	0.079753901
KCNC3	0.979689	0.347521	2.81908	0.00481615	0.08005357
EIF4G3	0.528955	0.187743	2.817439	0.00484083	0.080346447
CORO2B	-2.23774	0.794929	-2.81502	0.00487742	NA
CAPZB	-0.25638	0.091134	-2.813231	0.00490464	0.081287135
CXorf21	0.445883	0.158623	2.810956	0.00493945	0.08174501
PPIL4	0.431917	0.153757	2.809095	0.0049681	0.081977063
GATAD1	0.437772	0.155866	2.808647	0.00497501	0.081977063
C12orf74.PLEKHG7	-0.654418	0.233002	-2.808644	0.00497507	0.081977063
CCL4	-1.301935	0.463658	-2.807961	0.00498563	0.082032299
FUT11	0.604936	0.215496	2.807182	0.0049977	0.082112202
TAOK3	0.237267	0.084559	2.805922	0.00501729	0.082242478
IFIT5	0.545451	0.194405	2.805743	0.00502007	0.082242478
FAHD2B	-0.823504	0.293674	-2.804143	0.00504505	0.082446893
ENSG00000237513	0.917276	0.327129	2.804017	0.00504703	0.082446893
ENSG00000213865	0.703823	0.251072	2.803269	0.00505874	0.082499164
FBRS	-0.382159	0.136345	-2.802888	0.00506472	0.082499164

ENSG00000180448	-0.319561	0.114058	-2.801755	0.00508255	0.082576588
PRKCH	2.404249	0.85815	2.801664	0.00508398	0.082576588
ENSG00000272948	-2.418471	0.863395	-2.801117	0.0050926	NA
PDIK1L	-0.819898	0.292754	-2.800636	0.00510021	0.082722141
DUSP5	-1.538144	0.549351	-2.799929	0.00511138	0.082785504
ZNF22	0.285182	0.102064	2.794141	0.00520377	0.084095142
DRG2	-0.369571	0.132276	-2.79394	0.00520702	0.084095142
RNFT1	0.478531	0.171355	2.792621	0.0052283	0.08420548
GTF2I	0.25347	0.090765	2.7926	0.00522864	0.08420548
COX5B	-0.169738	0.060849	-2.789476	0.00527934	0.084805431
RFX2	0.817857	0.293203	2.789387	0.00528079	0.084805431
CFL1	-0.138171	0.049547	-2.788701	0.00529199	0.084865555
MSMP	-1.024571	0.367502	-2.787933	0.00530454	0.084947309
GRHPR	-0.376078	0.134974	-2.786292	0.00533148	0.085258722
PACSIN2	0.679518	0.244232	2.782264	0.00539812	0.086203333
HSP90B1	-0.27065	0.097298	-2.781672	0.00540797	0.086239697
CST7	2.35645	0.848701	2.776538	0.00549412	0.087457233
CREM	0.56478	0.203436	2.776209	0.00549968	0.087457233
CORO7	-0.48968	0.176564	-2.773381	0.00554772	0.088098032
ATP5MF	-0.176374	0.063642	-2.771361	0.00558225	0.08852296
FBLN2	2.142063	0.773242	2.770238	0.00560154	NA
NMT2	0.535575	0.193371	2.769672	0.00561128	0.088859515
PIK3C2A	0.561815	0.203004	2.767501	0.00564878	0.089226146
LAMTOR3	0.462681	0.167188	2.767425	0.0056501	0.089226146
YBX3	0.901049	0.325982	2.764104	0.00570794	0.090014609
TSNARE1	-1.40518	0.508566	-2.763024	0.00572685	0.090166741
PIGF	0.525227	0.190117	2.76265	0.00573342	0.090166741
NDUFB7	-0.199012	0.072094	-2.760456	0.00577208	0.090649528
TTC3	0.256506	0.092959	2.759339	0.00579184	0.090834475
RPS9	0.319199	0.11579	2.756716	0.0058385	0.091440391
TMEM141	-0.344733	0.125177	-2.753951	0.00588806	0.091983152
PTPN12	0.58	0.210612	2.753881	0.00588931	0.091983152
DVL3	0.694076	0.252656	2.747126	0.00601201	0.093571053
ZFYVE21	0.547785	0.19942	2.746895	0.00601625	0.093571053
GUK1	-0.235536	0.08575	-2.746769	0.00601854	0.093571053
ENSG00000179094	-0.76847	0.279802	-2.74648	0.00602385	0.093571053
BACH1.IT2	-1.871399	0.681466	-2.746138	0.00603014	NA
CBWD3	-0.36329	0.132463	-2.742589	0.00609569	0.094557875
DCP1B	-0.554224	0.202213	-2.740794	0.00612908	0.09475783
TXK	-1.473584	0.537797	-2.740039	0.00614319	0.09475783
WAC	0.246755	0.090056	2.740008	0.00614376	0.09475783
DHCR7	-0.66866	0.244066	-2.739667	0.00615015	0.09475783
AP4M1	-0.519086	0.189471	-2.739665	0.00615019	0.09475783
ENSG00000260267	-1.066361	0.389331	-2.738954	0.0061635	0.094779485
C12orf49	-0.563148	0.205626	-2.738701	0.00616824	0.094779485
PSMD7	-0.259057	0.094649	-2.737047	0.00619935	0.095010493
FAM131A	0.941206	0.343892	2.736922	0.0062017	0.095010493

FKBP11	-0.360427	0.131707	-2.736572	0.00620831	0.095010493
PNP	0.687393	0.251228	2.736127	0.0062167	0.095011252
ENSG00000278158	0.507077	0.18544	2.734457	0.00624832	0.095366431
HERC4	0.319266	0.116908	2.730914	0.0063159	0.096268778
BBS7	-0.753597	0.276137	-2.729074	0.00635125	0.096678305
PKM	-0.264695	0.097078	-2.726606	0.00639894	0.097151693
SINHCAF	-0.291957	0.107078	-2.726581	0.00639942	0.097151693
FBXO8	0.573721	0.210734	2.722494	0.00647913	0.098230806
CALM3	-0.228814	0.084076	-2.721519	0.00649827	0.098389973
CCL3L1.CCL3L3	-2.532956	0.93181	-2.718319	0.00656145	NA
TRIM27	0.385003	0.141655	2.717904	0.00656969	0.0993393
NOTCH2	0.431454	0.158799	2.716977	0.00658811	0.099485692
TMEM255B	1.06475	0.391973	2.716386	0.00659988	0.099531476
TULP3	0.742892	0.273559	2.715651	0.00661455	0.099612713
ADRB2	0.819895	0.30196	2.71524	0.00662277	0.099612713
ENSG00000274422	0.385719	0.142099	2.714446	0.00663867	0.099720229
ENSG00000273319	0.439711	0.162084	2.712848	0.00667076	0.100070188
VNN2	0.418644	0.15435	2.712301	0.00668179	0.100103749
SECISBP2	0.300997	0.111	2.711698	0.00669396	0.100120443
SSBP4	-0.310863	0.114651	-2.711375	0.00670049	0.100120443
GCNT1	1.11086	0.409851	2.7104	0.00672022	0.100283587
TSPAN13	-0.532371	0.196501	-2.709257	0.0067434	0.100497898
ENSG00000261079	-1.857196	0.686175	-2.706593	0.00679776	NA
TUNAR	-3.452219	1.275772	-2.705983	0.00681025	NA
TIMM8B	-0.242499	0.089659	-2.704673	0.00683717	0.101762178
TNFAIP3	-0.507204	0.187611	-2.703492	0.0068615	0.101990949
ATP5F1E	-0.105418	0.03904	-2.700286	0.00692798	0.102813694
ST6GAL1	0.336509	0.124635	2.699954	0.00693491	0.102813694
CHML	0.655343	0.242834	2.698727	0.00696052	0.102893298
NLN	1.294372	0.479678	2.698415	0.00696705	0.102893298
PEX13	0.537678	0.199264	2.698324	0.00696895	0.102893298
CENPU	-1.367091	0.506712	-2.697967	0.00697643	0.102893298
HOXB2	1.048754	0.388978	2.696178	0.00701403	0.103224726
ZNF48	0.804788	0.298515	2.695975	0.00701829	0.103224726
ABLIM1	0.409096	0.151764	2.695605	0.0070261	0.103224726
DDX17	0.189026	0.070137	2.695116	0.00703642	0.103243076
CMIP	0.448417	0.166441	2.694144	0.00705697	0.10341134
SMIM10	-2.798896	1.039366	-2.692889	0.00708359	NA
SLAMF6	0.528455	0.196282	2.692321	0.00709566	0.103844714
RAP1B	-0.308644	0.114673	-2.691525	0.00711262	0.103959248
CLEC11A	-1.107656	0.412151	-2.687498	0.00719896	0.105086288
PNO1	-0.47615	0.177207	-2.686971	0.00721032	0.10511746
PELO	0.727722	0.270961	2.685711	0.00723756	0.105379624
SDHAF2	-0.277749	0.103456	-2.684719	0.00725908	0.105558026
UBE2L3	-0.192982	0.071905	-2.68383	0.00727842	0.105657906
PABPC4	0.463426	0.172697	2.683466	0.00728633	0.105657906
CDK19	0.501663	0.18697	2.683124	0.00729379	0.105657906

STK17B	-0.242186	0.090367	-2.680022	0.00736174	0.106506639
RNF149	0.341267	0.127396	2.678796	0.00738874	0.10676172
DNASE1	0.517607	0.193319	2.677469	0.00741806	0.107044876
ABCA2	1.441407	0.538438	2.677015	0.00742813	0.107044876
ERICH1	0.236719	0.088439	2.676636	0.00743655	0.107044876
ZNF667	1.308454	0.488923	2.676196	0.0074463	0.107049932
MARCHF2	-0.939616	0.351351	-2.674299	0.00748856	0.107521692
TRIM8	0.368379	0.137812	2.673057	0.00751636	0.10778492
RABGGTA	-0.379138	0.141917	-2.67155	0.00755019	0.108133926
ADORA2A	-1.971242	0.738127	-2.670601	0.00757156	NA
FAM129A	-1.000973	0.374838	-2.670414	0.00757577	0.108363952
HACE1	1.675857	0.627893	2.669015	0.0076074	0.108555958
EEA1	0.41094	0.153969	2.668977	0.00760826	0.108555958
DEK	0.19408	0.072749	2.667815	0.00763462	0.108795692
ENSG00000256325	-1.399703	0.524894	-2.666641	0.00766135	0.109040196
CMPK1	-0.389707	0.146176	-2.66602	0.00767551	0.109105316
SH3KBP1	0.199329	0.074825	2.663957	0.00772275	0.109428175
APOOL	0.639128	0.239944	2.663658	0.00772961	0.109428175
RCC1	0.395603	0.148531	2.663444	0.00773454	0.109428175
SRP19	-0.290333	0.10901	-2.663351	0.00773667	0.109428175
TRIM5	0.639091	0.240016	2.662701	0.00775163	0.109474182
PPP1R10	0.371091	0.139383	2.662375	0.00775915	0.109474182
PCYOX1	0.582378	0.218843	2.661173	0.0077869	0.109633441
DNMT1	-0.322282	0.121122	-2.660799	0.00779555	0.109633441
TOMM22	-0.208136	0.078228	-2.660636	0.00779932	0.109633441
TBC1D8B	1.432247	0.538539	2.659505	0.00782555	0.109843189
CCDC50	-0.333078	0.125257	-2.659162	0.00783354	0.109843189
RANGRF	-0.276407	0.103966	-2.658624	0.00784604	0.109883219
BCL11A	0.344791	0.129775	2.65683	0.00788793	0.11033411
FBXL16	-1.481948	0.55848	-2.653538	0.00796527	0.111279241
GMCL1	0.361447	0.136317	2.651522	0.00801299	0.111808746
LINC01869	0.802164	0.302622	2.650712	0.00803222	0.111939873
PMEPA1	0.495276	0.186879	2.650251	0.0080432	0.111955859
LINC00539	-1.49929	0.565975	-2.649037	0.00807215	0.112221539
SLC25A30	1.135296	0.428677	2.648371	0.00808808	0.112235292
KSR1	-0.754238	0.284815	-2.648171	0.00809285	0.112235292
ABHD5	-0.610194	0.230573	-2.646427	0.0081347	0.112678418
EVI2A	0.441211	0.166791	2.645286	0.0081622	0.112921971
NARF	-0.234858	0.088847	-2.643398	0.00820785	0.113415763
SLC1A5	-0.516191	0.195363	-2.642218	0.00823649	0.11367357
FADS2	-1.576383	0.597338	-2.639014	0.00831476	0.114614871
DTX1	1.160814	0.440631	2.634436	0.00842773	0.115934978
MAP3K1	0.267826	0.101668	2.634308	0.00843089	0.115934978
P2RX4	-0.462592	0.17572	-2.632557	0.00847447	0.116390005
ENSG00000261386	0.575547	0.218663	2.632121	0.00848536	0.116390005
TCF4	0.187653	0.071316	2.631276	0.0085065	0.116390005
LINC02422	0.735465	0.279523	2.631145	0.00850978	0.116390005

ARHGEF10	1.409668	0.535805	2.630932	0.0085151	0.116390005
HLA.G	-1.32891	0.505293	-2.629977	0.00853907	0.116517999
P3H4	1.720472	0.654269	2.629611	0.00854827	0.116517999
ZNF90	-0.390805	0.148646	-2.629107	0.00856095	0.116517999
TTC33	0.706755	0.268872	2.628596	0.00857381	0.116517999
ENSG00000258168	-0.941235	0.358085	-2.628524	0.00857563	0.116517999
RDX	-0.416159	0.158379	-2.627611	0.00859867	0.116691834
DDX39A	-0.224438	0.085471	-2.625896	0.00864212	0.117141823
DDI2	0.510449	0.19446	2.624964	0.00866582	0.117323479
ZNF283	0.951637	0.362675	2.623943	0.00869184	0.117536035
MYO15B	0.892875	0.340746	2.620355	0.00878382	0.118638934
CTSC	0.38143	0.145711	2.617718	0.008852	0.119218884
RNF41	0.278123	0.106248	2.617688	0.00885277	0.119218884
GLMN	-0.428749	0.163809	-2.617365	0.00886116	0.119218884
CRTC3	0.369411	0.141156	2.617052	0.00886927	0.119218884
NUFIP2	0.363108	0.138767	2.616674	0.00887912	0.119218884
SCRN3	-0.725137	0.277226	-2.615686	0.00890485	0.119423596
MPST	0.282447	0.10805	2.614032	0.00894808	0.119862098
ZSCAN18	0.579553	0.221774	2.613262	0.00896826	0.119991357
TRIM13	0.266487	0.102006	2.612468	0.00898912	0.120129284
PHF2	0.985914	0.377447	2.612062	0.0089998	0.120131027
ENSG00000251661	-1.499405	0.574249	-2.61107	0.00902595	0.120338984
TRIO	-0.496699	0.19032	-2.609805	0.00905939	0.120496473
SMARCB1	-0.321647	0.123257	-2.60957	0.00906561	0.120496473
TNFAIP8	-0.289231	0.110841	-2.609423	0.00906951	0.120496473
HS3ST1	1.059962	0.406344	2.608537	0.00909302	0.120625135
ATP5PF	-0.15767	0.06045	-2.60826	0.00910039	0.120625135
TIPRL	-0.414066	0.158823	-2.607092	0.00913149	0.120896653
LRRC37B	0.580232	0.222636	2.606194	0.00915547	0.120963985
NEMP1	0.774818	0.297323	2.605982	0.00916113	0.120963985
APAF1	0.561014	0.215328	2.605398	0.00917677	0.120963985
YTHDC1	0.24269	0.093161	2.605056	0.00918594	0.120963985
MYO1D	-1.15546	0.443569	-2.604915	0.00918969	0.120963985
RGS1	0.724749	0.278294	2.604259	0.00920731	0.121055947
TRIAP1	-0.287115	0.110273	-2.603664	0.00922331	0.121126351
FCGR2A	1.165545	0.448014	2.601583	0.00927947	0.121723491
SERINC1	0.344523	0.132493	2.600303	0.00931415	0.121947627
TOPORS	0.312494	0.120183	2.600162	0.00931799	0.121947627
NDUFS7	-0.211248	0.081256	-2.599768	0.00932868	0.121947627
DPH7	-0.380471	0.146384	-2.599127	0.00934611	0.121957932
ENSG00000167807	0.879136	0.338341	2.59837	0.00936674	0.121957932
CBX6	-0.263882	0.101572	-2.597968	0.00937772	0.121957932
RGCC	0.975829	0.37564	2.597776	0.00938296	0.121957932
C1orf174	0.358216	0.137894	2.597764	0.00938331	0.121957932
SIAH1	0.392431	0.151087	2.597382	0.00939374	0.121957932
ARMCX2	0.674595	0.259835	2.596241	0.00942498	0.122224214
ATP2B4	-0.857822	0.330636	-2.594463	0.0094739	0.122580402

GM2A	0.487714	0.187984	2.594448	0.00947428	0.122580402
DEDD2	0.356817	0.137551	2.594069	0.00948475	0.122580402
CLMN	0.83361	0.321617	2.591937	0.00954373	0.123202867
CLN8	0.367003	0.141703	2.589943	0.0095992	0.123755465
WSB1	0.42969	0.165928	2.589617	0.00960828	0.123755465
CXCR5	-0.318655	0.123139	-2.58777	0.00965995	0.12428044
CD99	-0.268465	0.103786	-2.58672	0.00968944	0.124519181
RHBDD2	-0.388854	0.15039	-2.585641	0.00971981	0.12476857
SH2D3C	0.49743	0.192485	2.584251	0.00975908	0.125131575
BOLA2B	-0.439858	0.170252	-2.583567	0.00977844	0.125238787
IL12A	0.956266	0.3704	2.58171	0.00983121	0.125773226
DAP3	-0.234434	0.090893	-2.579244	0.00990168	0.126477082
UQCRC2	-0.205572	0.07971	-2.579008	0.00990845	0.126477082
CCPG1	0.399349	0.154878	2.578475	0.00992374	0.126530416
SNRPA1	-0.295757	0.114755	-2.577291	0.00995781	0.126822863
RHOBTB3	1.643786	0.638544	2.574273	0.01004509	0.127791525
RASGRP3	-0.430791	0.167394	-2.57351	0.01006726	0.127930615
CAMK2D	0.54122	0.21034	2.573073	0.01008	0.127949653
IFT57	0.431118	0.167623	2.571953	0.01011268	0.128221513
SPPL2A	-0.315104	0.122557	-2.571076	0.0101383	0.128228008
TMED8	0.332389	0.129293	2.570828	0.01014556	0.128228008
ENSG00000238142	0.662041	0.257557	2.570469	0.01015611	0.128228008
HADHA	0.175487	0.068272	2.570396	0.01015824	0.128228008
GNAS	0.190397	0.074085	2.569964	0.0101709	0.128245669
ACBD3	-0.45862	0.178482	-2.56955	0.01018308	0.128257241
SKIL	0.355162	0.138263	2.568733	0.01020711	0.128337902
DNAJC9	-0.342619	0.133389	-2.568566	0.01021203	0.128337902
BZW1	-0.227494	0.088594	-2.567829	0.01023375	0.128469153
CCDC28B	-0.552706	0.215288	-2.567283	0.01024989	0.12853006
ARPC5	-0.147067	0.057299	-2.566663	0.01026823	0.128618364
ENSG00000272933	-0.964709	0.375973	-2.565898	0.0102909	0.128760702
THAP9.AS1	-0.484961	0.189095	-2.564649	0.01032804	0.129037918
PSMD3	-0.326374	0.127272	-2.56439	0.01033573	0.129037918
ARID4B	0.252234	0.098389	2.563647	0.01035789	0.129172919
VSIG10L	1.053735	0.411097	2.563226	0.01037045	0.129188099
ZNRD2	-0.321223	0.125344	-2.562735	0.01038512	0.12922943
TANK	-0.224398	0.087688	-2.559048	0.01049593	0.130373672
ENSG00000271553	-1.12411	0.439292	-2.558914	0.01049997	0.130373672
PLEKHA2	0.347948	0.136034	2.557793	0.01053387	0.130548493
GDF11	0.473946	0.185302	2.557691	0.01053698	0.130548493
MIR210HG	-1.221008	0.477591	-2.556597	0.01057015	0.130694639
COBLL1	-0.236052	0.092333	-2.556545	0.01057173	0.130694639
ICAM2	-0.481076	0.188243	-2.555608	0.01060024	0.130812519
POLR2G	-0.226152	0.088524	-2.554695	0.01062809	0.130812519
TMC6	-0.252843	0.098976	-2.554596	0.0106311	0.130812519
CD53	-0.270667	0.105953	-2.554594	0.01063117	0.130812519
GORASP2	-0.357618	0.140004	-2.554347	0.01063871	0.130812519

LCP1	-0.243043	0.095177	-2.553589	0.01066191	0.1309563
PAFAH1B2	0.231839	0.090809	2.553051	0.01067838	0.131017229
ENSG00000249249	-0.815392	0.319656	-2.55084	0.01074638	0.131709658
ENSG00000273669	-1.480241	0.580659	-2.549241	0.01079576	NA
PLEKHB2	0.346566	0.136026	2.547782	0.01084102	0.13236327
LDLRAD4	0.441163	0.173168	2.547599	0.0108467	0.13236327
PRMT1	-0.263529	0.103447	-2.54748	0.01085042	0.13236327
SLC2A5	-0.828871	0.32539	-2.54732	0.01085539	0.13236327
METTL15	-0.411096	0.161392	-2.547182	0.01085967	0.13236327
ARRDC5	0.815549	0.320246	2.546635	0.01087671	0.13236327
ORAI2	0.215828	0.084766	2.546159	0.01089156	0.13236327
HYOU1	-0.648803	0.25482	-2.546122	0.01089271	0.13236327
TCL1B	-1.367421	0.537501	-2.544032	0.0109581	0.132918173
ENSG00000246528	-1.402147	0.551177	-2.543917	0.01096172	0.132918173
ENSG00000260708	-0.706028	0.277634	-2.543018	0.01098997	0.133118881
ZFP1	-0.756616	0.297703	-2.541511	0.01103744	0.133551852
ENSG00000227070	-1.954816	0.769294	-2.541051	0.01105199	NA
ATXN3	0.261252	0.102877	2.539472	0.01110201	0.134190507
ENSG00000272462	2.029764	0.799679	2.538222	0.01114172	NA
CNNM4	1.675273	0.66012	2.537829	0.01115424	0.134678849
H2AFV	-0.199594	0.078693	-2.536374	0.01120071	0.135096637
ARF1	-0.202138	0.079712	-2.535867	0.01121693	0.135149178
ENSG00000260000	1.291269	0.509401	2.534879	0.0112486	0.135348235
ENSG00000253948	-0.72045	0.284261	-2.534471	0.01126173	0.135348235
VAT1	0.491344	0.193897	2.534047	0.01127535	0.135348235
NINJ1	-0.507495	0.200284	-2.533872	0.011281	0.135348235
ARPC5L	-0.228321	0.090154	-2.532559	0.01132333	0.13564763
SELENOT	-0.182295	0.071988	-2.532279	0.01133237	0.13564763
SAP18	-0.128338	0.050686	-2.531991	0.0113417	0.13564763
PUF60	-0.316794	0.125238	-2.529529	0.01142156	0.136396464
BFAR	-0.259771	0.102704	-2.529323	0.01142827	0.136396464
SLC15A3	0.75757	0.299614	2.528489	0.01145546	0.136453361
COMMD2	-0.3126	0.123649	-2.528124	0.01146738	0.136453361
PSMC1	-0.215822	0.08537	-2.528075	0.01146899	0.136453361
PLTP	-1.071969	0.424094	-2.527666	0.01148235	0.136469779
FOXK1	-0.323861	0.128195	-2.526323	0.01152634	0.13668222
ZNF552	0.613265	0.242777	2.526037	0.01153575	0.13668222
KLHL28	0.427284	0.169153	2.526022	0.01153624	0.13668222
CCDC117	0.678214	0.268638	2.524635	0.01158185	0.137079995
SNRNP35	-0.321174	0.127239	-2.52417	0.01159718	0.137118906
ENSG00000259804	-1.517804	0.601632	-2.52281	0.01164212	0.137507431
CD63	0.414322	0.164324	2.52138	0.01168955	0.137924541
NEK7	0.557774	0.221352	2.519849	0.0117405	0.138382365
POLR3K	0.243603	0.096696	2.519273	0.01175974	0.13846572
SARAF	0.214722	0.085271	2.518122	0.01179825	0.138775656
MRPL28	-0.259968	0.103284	-2.517027	0.01183498	0.139043187
SNX5	0.280093	0.111293	2.516716	0.01184542	0.139043187

KIDINS220	0.591536	0.235113	2.515965	0.01187071	0.139196649
C2orf15	-1.074437	0.427228	-2.514901	0.0119066	0.139469387
ADRM1	-0.267624	0.106444	-2.514219	0.01192964	0.139469387
PGK1	-0.27207	0.108221	-2.514023	0.01193626	0.139469387
ARL4D	0.795118	0.316298	2.513825	0.01194297	0.139469387
FGF9	-1.367257	0.544195	-2.512439	0.01198998	0.139874859
RIPOR1	0.330336	0.131514	2.511791	0.01201201	0.139988435
CCDC66	0.343794	0.136934	2.510644	0.01205113	0.140300724
DDB2	-0.28235	0.112481	-2.510209	0.01206597	0.140330004
PCNP	-0.191736	0.076429	-2.50869	0.01211798	0.140791192
C17orf51	1.61038	0.642079	2.508072	0.0121392	0.1408939
RTL10	1.143342	0.456019	2.507223	0.01216838	0.14108878
GTF2F1	0.270745	0.108018	2.506488	0.01219373	0.141238842
ETNK1	0.355822	0.142032	2.505223	0.01223741	0.141600753
ENSG00000233461	-0.451357	0.180215	-2.504544	0.01226093	0.141728842
COMMD8	-0.292786	0.116975	-2.502975	0.01231544	0.142194018
TP53TG1	-0.275218	0.10997	-2.502667	0.01232615	0.142194018
HIGD1A	-0.327919	0.131106	-2.501181	0.012378	0.14249983
BAK1	-0.442414	0.176907	-2.500836	0.01239006	0.14249983
DDX5	-0.260237	0.104082	-2.500312	0.01240838	0.14249983
ISOC2	-0.400832	0.160318	-2.500233	0.01241116	0.14249983
CXCR4	0.279198	0.111674	2.500117	0.01241523	0.14249983
GABARAPL1	1.143316	0.457479	2.499167	0.01244857	0.142738545
SDSL	2.264314	0.906227	2.498617	0.0124679	0.142816367
FAM234B	1.367705	0.547985	2.495879	0.01256453	0.14374506
ZNF91	0.320561	0.12845	2.495606	0.01257422	0.14374506
ALOX5AP	0.395889	0.158699	2.494588	0.01261033	0.143940115
WDR74	0.279534	0.112068	2.494334	0.01261938	0.143940115
BOP1	-0.469722	0.188336	-2.494057	0.01262921	0.143940115
SUDS3	0.27288	0.109453	2.493121	0.01266258	0.144176158
CXorf56	-0.494044	0.198193	-2.49274	0.01267616	0.144186587
KIF22	-0.444467	0.17843	-2.490984	0.01273899	0.144756643
PSMD6.AS2	1.687168	0.677472	2.49039	0.0127603	0.144854193
SH3TC1	0.421906	0.169595	2.487729	0.01285615	0.145796978
ENSG00000257275	-1.147503	0.461617	-2.485833	0.01292487	0.146430421
ENSG00000259888	2.325056	0.935727	2.484759	0.01296392	NA
TNKS1BP1	-1.538451	0.619315	-2.484116	0.01298734	NA
GGT1	-1.101101	0.443284	-2.483964	0.0129929	0.146784332
PTAFR	-0.885852	0.356676	-2.483632	0.013005	0.146784332
IDH3G	-0.268289	0.108026	-2.483558	0.01300771	0.146784332
ESF1	0.251714	0.101353	2.483536	0.01300853	0.146784332
LRRK2	0.504547	0.203184	2.483206	0.01302057	0.146784332
RP2	0.654292	0.263539	2.482718	0.01303843	0.146815235
NBPF9	0.809397	0.326093	2.482104	0.01306093	0.146815235
COX14	-0.176766	0.071217	-2.482075	0.01306199	0.146815235
RGL1	-2.725948	1.098491	-2.481538	0.01308166	NA
ENSG00000092330	-0.224482	0.090489	-2.480762	0.01311017	0.14704952

NDUFAF3	-0.202375	0.081579	-2.480712	0.01311204	0.14704952
LIMD2	-0.22391	0.09027	-2.480452	0.01312158	0.14704952
SFT2D2	0.424082	0.171094	2.478653	0.01318794	0.14764783
SLC27A4	-1.028986	0.415266	-2.4779	0.01321583	0.147814759
KLK2	1.76354	0.712005	2.476864	0.01325423	0.148098744
TMC8	-0.272341	0.110034	-2.475062	0.01332131	0.148337646
ZNF628	-0.751506	0.30364	-2.47499	0.01332401	0.148337646
TMED2	-0.179019	0.072332	-2.474957	0.01332523	0.148337646
CERS4	-0.265973	0.107469	-2.47489	0.01332772	0.148337646
BLZF1	0.588301	0.237898	2.472908	0.01340186	0.149017184
TUBB4B	-0.328808	0.133097	-2.470437	0.01349483	0.149904487
SCAF11	0.146606	0.059358	2.469864	0.01351642	0.149998058
NUP50.DT	-0.813324	0.329397	-2.469126	0.01354434	0.150161442
C1orf109	0.512984	0.207917	2.467249	0.01361556	0.150804258
DNAJB1	0.440152	0.178426	2.466863	0.01363025	0.150820232
AHCYL1	0.443945	0.180099	2.465	0.01370133	0.151328987
ENSG00000131408	0.213464	0.086599	2.464961	0.01370281	0.151328987
RBM39	0.134528	0.054586	2.464534	0.01371916	0.151362702
METRNL	1.217334	0.494956	2.459478	0.01391391	0.153362848
TLR4	-2.163997	0.879934	-2.459273	0.01392188	NA
ENSG00000268403	-0.623928	0.253778	-2.458552	0.01394986	0.153504085
UPP1	0.708443	0.288166	2.458453	0.01395369	0.153504085
RGS10	0.298272	0.121392	2.457108	0.01400607	0.15393162
SMAD4	0.503826	0.205477	2.451986	0.01420701	0.155927653
SNRPG	-0.143429	0.0585	-2.451782	0.01421508	0.155927653
GKAP1	0.463908	0.189275	2.450972	0.01424708	0.156128293
MTHFR	0.446973	0.182405	2.450448	0.01426786	0.156160096
ZC3H6	0.457652	0.186781	2.450207	0.01427742	0.156160096
PDK2	-0.457412	0.186758	-2.449219	0.01431665	0.15643899
BROX	0.260914	0.10662	2.447126	0.01440006	0.157199502
ENSG00000237976	-0.926679	0.378781	-2.44648	0.01442588	0.157330544
ENSG00000226571	1.030504	0.421294	2.446045	0.0144433	0.157369776
PTPRJ	1.245054	0.509611	2.443146	0.01455985	0.158487975
SPATA13	0.707886	0.289879	2.442004	0.01460598	0.158838281
GSDME	-1.199555	0.491323	-2.441478	0.01462729	0.158918269
POLH	0.759813	0.311351	2.44037	0.01467224	0.159244057
CNIH1	0.282838	0.115921	2.439915	0.01469071	0.159244057
GPR146	0.783709	0.321241	2.43963	0.01470231	0.159244057
CD55	-0.274463	0.112514	-2.439362	0.01471322	0.159244057
TMEM109	0.34701	0.142335	2.437977	0.0147697	0.159703478
REV1	0.390671	0.160286	2.43734	0.01479578	0.159752584
PCOLCE	1.042704	0.427832	2.43718	0.0148023	0.159752584
FABP5	0.502648	0.206321	2.436244	0.01484066	0.159913057
NOTCH1	0.739119	0.303542	2.434982	0.01489252	0.159913057
FANCB	-1.014061	0.416505	-2.434691	0.0149045	0.159913057
SOS1	0.49467	0.203187	2.434558	0.01490997	0.159913057
ENSG00000132481	-0.90414	0.371428	-2.434226	0.01492368	0.159913057

ZDHHC4	0.301876	0.124032	2.433852	0.01493909	0.159913057
MID1IP1	0.492142	0.202222	2.433676	0.01494636	0.159913057
IGFBP4	-0.963446	0.395913	-2.433479	0.01495449	0.159913057
RAPGEF2	0.976854	0.401431	2.433429	0.01495656	0.159913057
RGS17	-1.355582	0.557072	-2.433404	0.01495762	0.159913057
GTSF1	-0.625603	0.25745	-2.429996	0.01509899	0.161273089
HSPA1A	1.312088	0.540033	2.429642	0.01511376	0.1612795
MPZL1	0.528297	0.217516	2.428773	0.01515001	0.161503723
SLF1	0.409225	0.168512	2.428459	0.01516314	0.161503723
MDH2	-0.207088	0.0853	-2.427769	0.01519203	0.161660208
BABAM2	-0.393799	0.16228	-2.426664	0.01523836	0.162001862
BET1	0.35046	0.144469	2.425853	0.01527247	0.162006405
CCDC180	-0.974523	0.401736	-2.425782	0.01527545	0.162006405
ENSG00000257354	-0.748517	0.308585	-2.425639	0.01528148	0.162006405
RNF24	0.643748	0.265587	2.423864	0.01535634	0.162405383
BCR	0.785793	0.324199	2.423797	0.01535917	0.162405383
PDK4	1.598314	0.659443	2.423733	0.0153619	0.162405383
IGHA2	-1.263211	0.521442	-2.422534	0.01541267	0.162790923
THOC7	-0.204978	0.084666	-2.421029	0.01547663	0.163230182
FAM184B	-1.640939	0.677827	-2.420881	0.01548293	0.163230182
ABTB2	1.988283	0.821869	2.419222	0.01555376	NA
H2AFJ	-0.416085	0.172092	-2.417806	0.01561439	0.164256333
MAP4K3.DT	0.546221	0.225927	2.417682	0.0156197	0.164256333
CCNT2	0.385008	0.159253	2.417593	0.01562354	0.164256333
POLR2B	-0.227998	0.094344	-2.416666	0.0156634	0.164405064
PHF6	0.367796	0.152217	2.416255	0.01568107	0.164405064
UPF2	-0.158293	0.065519	-2.415985	0.01569271	0.164405064
WIPF2	0.336398	0.139268	2.415477	0.01571464	0.164405064
MAP3K2	0.320777	0.132805	2.415403	0.01571781	0.164405064
KCTD2	1.056069	0.43725	2.415252	0.01572432	0.164405064
BACH1	0.383233	0.158845	2.412622	0.01583822	0.165398483
SLC35B2	-0.342115	0.141816	-2.412388	0.01584839	0.165398483
BCL11B	2.162671	0.896872	2.411349	0.01589365	0.16569344
ENSG00000077463	-0.374809	0.155466	-2.410874	0.01591435	0.16569344
GPX7	0.389983	0.161769	2.410737	0.01592031	0.16569344
IL4R	-0.456911	0.189717	-2.408387	0.0160232	0.166612061
BCDIN3D	0.543507	0.225814	2.406881	0.01608941	0.167147795
ENSG00000271133	-1.471358	0.612158	-2.403561	0.01623627	0.168519798
CCNG2	0.43342	0.180389	2.402692	0.0162749	0.1687272
ZNF250	0.719861	0.299668	2.402196	0.01629695	0.1687272
EIF2AK3	-0.542762	0.225952	-2.402112	0.01630071	0.1687272
TNFRSF14	0.207249	0.086314	2.401097	0.01634599	0.169042135
ADAP1	1.006077	0.419223	2.39986	0.01640134	0.169455031
SNAP23	-0.209907	0.087478	-2.39954	0.01641568	0.169455031
ELK3	0.224984	0.093789	2.398831	0.0164475	0.16962974
FAIM	0.35266	0.147078	2.39778	0.01649476	0.169785964
MRPL12	-0.268821	0.112119	-2.397639	0.01650111	0.169785964

RASGRP2	-0.225822	0.0942	-2.397272	0.01651764	0.169785964
ISCA2	-0.218416	0.091123	-2.396944	0.01653247	0.169785964
NDUFA4	-0.119837	0.049998	-2.396839	0.01653721	0.169785964
TMF1	0.268353	0.112002	2.395972	0.01657635	0.170034479
CCNG1	-0.216404	0.090343	-2.395358	0.01660416	0.170166494
CMTM7	-0.335936	0.14031	-2.394245	0.01665461	0.170530006
C1orf56	-0.286223	0.119573	-2.393716	0.01667865	0.170622778
HIST1H3E	1.206618	0.504391	2.392225	0.01674658	0.171163876
SLC35G5	1.205195	0.503866	2.391895	0.01676163	0.171164081
SRSF3	-0.138268	0.057822	-2.391279	0.0167898	0.171298085
TMEM156	-0.315642	0.132025	-2.390768	0.01681316	0.171382872
ETFRF1	-0.365199	0.152882	-2.38876	0.01690534	0.172017957
IGLC3	-0.448147	0.187664	-2.388026	0.01693916	0.172017957
SEZ6	0.609776	0.255366	2.387851	0.01694721	0.172017957
MRTO4	-0.369884	0.154921	-2.387572	0.01696008	0.172017957
LSM7	0.24735	0.103626	2.386949	0.01698883	0.172017957
MAST4	0.912281	0.382216	2.386819	0.01699488	0.172017957
ARID1A	0.267873	0.112238	2.386656	0.01700238	0.172017957
NR5A2	-1.400758	0.587064	-2.386037	0.01703102	0.172017957
KRT10	-0.211233	0.08853	-2.385997	0.01703288	0.172017957
CLCN7	-0.339923	0.142476	-2.385834	0.01704045	0.172017957
LIMK1	0.451287	0.189174	2.38557	0.01705266	0.172017957
PXMP4	-0.609007	0.255297	-2.385482	0.01705676	0.172017957
IDS	0.201988	0.084686	2.38515	0.01707218	0.172021105
GRAP2	0.989957	0.415313	2.383639	0.0171424	0.172575937
ARID3B	0.512164	0.214932	2.382914	0.01717621	0.172763585
PHETA2	1.821283	0.764359	2.382757	0.01718353	NA
KLK1	1.287474	0.540597	2.381579	0.01723859	0.173237922
STRAP	-0.262982	0.110464	-2.380693	0.01728012	0.173502117
APP	0.556629	0.233884	2.379936	0.01731567	0.173622242
TLR7	0.577798	0.242794	2.379788	0.01732258	0.173622242
POLR2I	-0.250013	0.105087	-2.3791	0.01735497	0.17377433
GPR65	0.390253	0.164053	2.378817	0.01736828	0.17377433
NINJ2	-0.437966	0.184208	-2.377559	0.01742766	0.174069734
SLC31A2	1.300688	0.54709	2.377466	0.01743206	0.174069734
ATRX	0.158209	0.066563	2.376819	0.01746264	0.174069734
RHNO1	-0.525028	0.220904	-2.376729	0.01746693	0.174069734
ENSG00000263394	0.970841	0.408504	2.376574	0.01747424	0.174069734
SNRPD3	-0.197425	0.08311	-2.375469	0.01752668	0.174439466
ENSG00000260641	-2.019112	0.850769	-2.373278	0.01763097	NA
TMEM101	0.31414	0.132517	2.37057	0.01776069	0.176614192
ELOA	-0.297595	0.125581	-2.369737	0.01780074	0.176665439
FOLR2	-0.718149	0.303054	-2.369705	0.01780226	0.176665439
FGFR1OP2	0.249787	0.105418	2.369495	0.0178124	0.176665439
HIST1H2AG	1.691772	0.714206	2.368746	0.01784852	0.176869644
TENT4B	0.550037	0.232257	2.368221	0.01787388	0.176966951
CIB1	0.317358	0.134025	2.367898	0.01788946	0.176967328

TTC7A	-0.437313	0.184773	-2.366755	0.01794482	0.177360864
GSDMD	-0.268794	0.113598	-2.366195	0.01797199	0.177475366
SLC25A42	-0.398135	0.168295	-2.36569	0.01799653	0.177563638
MCM6	0.922178	0.390156	2.363611	0.01809781	0.178408354
KLK4	1.799422	0.761511	2.362963	0.0181295	0.178480235
GADD45A	-0.548106	0.232031	-2.362213	0.01816619	0.178480235
ADAM28	0.215418	0.091197	2.362123	0.0181706	0.178480235
SFXN4	-0.61273	0.259414	-2.361976	0.01817782	0.178480235
CBLN3	-0.652289	0.276201	-2.36165	0.01819379	0.178480235
UBE2D3	-0.126196	0.053453	-2.360883	0.01823147	0.178480235
BYSL	0.933433	0.395375	2.360882	0.01823152	0.178480235
NKILA	-1.680653	0.711923	-2.360721	0.01823942	NA
MRPL2	-0.262369	0.11114	-2.360719	0.01823954	0.178480235
PIM1	-0.409211	0.173352	-2.360584	0.01824618	0.178480235
ZFP69B	1.946399	0.824684	2.360174	0.01826638	NA
QRSL1	-0.337304	0.142934	-2.359848	0.01828245	0.178681476
AZIN1.AS1	-1.298493	0.5504	-2.359178	0.01831548	0.178848006
HDX	1.483382	0.628854	2.358865	0.0183309	0.178848006
UQCRH	-0.163911	0.069497	-2.358523	0.01834783	0.17885989
SERPINB1	-0.250558	0.10627	-2.35774	0.01838658	0.17896099
C1orf122	-0.305764	0.129698	-2.357505	0.01839821	0.17896099
GTF3A	-0.167083	0.070886	-2.357047	0.01842092	0.17896099
ATP10D	0.547785	0.232415	2.35693	0.01842673	0.17896099
BEND4	0.903296	0.383302	2.356616	0.01844229	0.17896099
MXI1	0.596754	0.25327	2.356198	0.01846308	0.17896099
ENSG00000224610	-2.132759	0.905228	-2.356045	0.01847067	NA
FNIP1	-0.337602	0.143303	-2.355865	0.01847962	0.17896099
KCTD6	0.518775	0.220271	2.35517	0.01851425	0.17896099
BAX	-0.37787	0.16047	-2.354775	0.01853392	0.17896099
PIK3R1	0.389995	0.165644	2.354409	0.01855217	0.17896099
PPIE	-0.294909	0.125258	-2.354406	0.01855231	0.17896099
FAM217B	0.382042	0.162278	2.354249	0.01856016	0.17896099
C6orf203	0.44492	0.18899	2.354202	0.01856253	0.17896099
MRPL41	-0.178551	0.075888	-2.352827	0.01863129	0.179471924
DCXR	-0.267061	0.113549	-2.351947	0.01867545	0.179669744
POGZ	0.307733	0.13085	2.351789	0.01868338	0.179669744
TCL1A	-0.627904	0.267167	-2.350229	0.01876189	0.180176691
AGRN	-0.964904	0.410578	-2.350112	0.01876775	0.180176691
LAPTM5	-0.203404	0.086585	-2.349184	0.01881462	0.180430728
PAFAH1B1	0.225548	0.09602	2.348961	0.0188259	0.180430728
CCDC138	-0.721319	0.307141	-2.348494	0.01884952	0.180505117
KDM6B	0.419681	0.178778	2.347503	0.01889972	0.180737596
SEC61G	-0.184324	0.078523	-2.347387	0.0189056	0.180737596
JAK3	-0.461986	0.196848	-2.346913	0.01892969	0.180737596
AUTS2	1.201411	0.511944	2.346763	0.01893729	0.180737596
ENSG00000245904	0.351593	0.149876	2.345901	0.01898113	0.181004312
BRK1	-0.136614	0.058246	-2.345445	0.01900437	0.181074226

GLRX3	-0.240696	0.10266	-2.344601	0.01904747	0.181333217
LRRCC1	0.95291	0.406526	2.344032	0.01907655	0.18145833
NFKBIB	-0.354354	0.151222	-2.343266	0.01911575	0.181679355
CELF1	0.209135	0.089275	2.342592	0.01915032	0.181762878
ZMYND8	0.360727	0.154003	2.342336	0.01916346	0.181762878
CNOT3	0.388151	0.165723	2.342161	0.01917242	0.181762878
NDUFAF6	-0.329319	0.1407	-2.340577	0.01925397	0.182384067
TMEM243	0.163285	0.069783	2.33988	0.01928993	0.182421174
ABCB1	1.01428	0.433475	2.33988	0.01928991	0.182421174
ELK1	-0.413381	0.17682	-2.337856	0.01939474	0.183139447
DNA2	-1.172159	0.501396	-2.337792	0.01939805	0.183139447
NUDT13	1.357627	0.580864	2.337254	0.01942597	0.183206221
SNHG32	0.178381	0.076328	2.337036	0.0194373	0.183206221
CASP1	0.505716	0.216421	2.336725	0.01945347	0.183206963
PSMD6	-0.266797	0.11424	-2.335403	0.01952236	0.183703753
ENG	0.79487	0.340436	2.334858	0.01955082	0.183819652
OAS3	0.960993	0.411754	2.333898	0.01960105	0.184139921
SEC61A2	-0.674673	0.289139	-2.333388	0.01962778	0.184181145
TARBP2	-0.404685	0.173447	-2.333197	0.01963779	0.184181145
CARD8	-0.262014	0.112357	-2.331978	0.01970186	0.184629915
MRPL34	-0.203932	0.08747	-2.331451	0.0197296	0.184737837
EPRS	-0.305223	0.131011	-2.329742	0.01981979	0.185429883
YWHAG	0.331089	0.142141	2.329295	0.01984344	0.185498725
LTBP3	0.278726	0.119695	2.32863	0.01987869	0.18556955
SYNPO	-0.82446	0.354068	-2.328537	0.01988362	0.18556955
ZNF267	-0.338326	0.145332	-2.327959	0.0199143	0.185591152
ENSG00000280433	-1.028761	0.441931	-2.327879	0.01991853	0.185591152
SIGIRR	-0.264875	0.113842	-2.326686	0.01998199	0.185920243
PRDX3	0.295373	0.126955	2.326601	0.01998651	0.185920243
C16orf74	-0.39101	0.168128	-2.325669	0.02003621	0.186084331
CENPC	-0.289117	0.124316	-2.325657	0.02003683	0.186084331
INPP5F	0.753375	0.324125	2.324334	0.0201076	0.186589355
PHTF2	-0.356323	0.153349	-2.323615	0.02014614	0.186703791
RBX1	-0.131757	0.056707	-2.323492	0.02015273	0.186703791
ITGAM	-0.590877	0.25439	-2.322725	0.02019392	0.18693334
BAG6	-0.24655	0.106174	-2.322143	0.02022524	0.187071177
S100A10	-0.41006	0.176691	-2.320779	0.0202988	0.187599132
KDELR2	-0.282172	0.121619	-2.320134	0.0203336	0.187768371
PIP4P2	0.533153	0.229922	2.318847	0.02040335	0.188259732
SBDS	-0.248096	0.107046	-2.317665	0.02046753	0.188698987
USF1	-0.729659	0.314891	-2.317178	0.02049406	0.188790741
FANCL	0.735084	0.317353	2.316297	0.02054204	0.189079773
CBWD2	-0.463302	0.200069	-2.315717	0.02057373	0.189218466
ENSG00000007237	1.078939	0.466266	2.314002	0.02066762	0.189928621
PVT1	-0.545624	0.235979	-2.312175	0.02076806	0.190405223
FDPS	-0.183729	0.079467	-2.31201	0.02077713	0.190405223
ROMO1	-0.160351	0.069366	-2.311649	0.02079706	0.190405223

SRP72	-0.220015	0.095187	-2.311404	0.02081055	0.190405223
DCTD	-0.277439	0.120039	-2.311232	0.02082002	0.190405223
CCNJ	-0.872721	0.377614	-2.311144	0.02082491	0.190405223
ABHD15	0.420509	0.181969	2.310881	0.02083942	0.190405223
YWHAE	0.183046	0.07922	2.31059	0.02085554	0.190405223
DCUN1D3	-1.330407	0.575852	-2.310328	0.02086999	0.190405223
ARL6IP6	0.315613	0.136683	2.309091	0.02093855	0.190797073
TAX1BP1	-0.216731	0.093865	-2.308948	0.02094646	0.190797073
DYNLL1	0.207553	0.089918	2.308251	0.02098518	0.190935036
GPR137B	0.883456	0.382797	2.307898	0.02100482	0.190935036
PIM3	-0.79338	0.343786	-2.30777	0.02101191	0.190935036
PSMD9	-0.232882	0.100975	-2.306336	0.02109185	0.191315359
KLF10	-0.580772	0.25182	-2.306299	0.02109391	0.191315359
ENSG00000275964	-0.666843	0.289163	-2.306116	0.02110417	0.191315359
PPA2	-0.296557	0.128786	-2.302716	0.02129481	0.192796347
POLR2L	-0.15613	0.067806	-2.302599	0.02130141	0.192796347
PSMB6	-0.153238	0.066591	-2.301195	0.02138063	0.193359704
SPPL2B	0.22714	0.098722	2.300799	0.02140302	0.19337465
PSMB4	-0.205259	0.089235	-2.300207	0.02143648	0.19337465
PHF3	0.24518	0.1066	2.300005	0.02144793	0.19337465
MYO7B	-1.359557	0.591121	-2.299965	0.02145022	0.19337465
ENSG00000224521	-1.706002	0.742631	-2.297242	0.02160499	NA
PSMC3	-0.216174	0.094126	-2.296656	0.0216384	0.194912544
POLA2	-0.704975	0.306996	-2.296365	0.02165505	0.194912544
NUDT5	-0.253367	0.110409	-2.294809	0.02174405	0.195497115
TTYH3	0.726925	0.316794	2.29463	0.02175433	0.195497115
RER1	-0.229109	0.099918	-2.292966	0.02184998	0.1962018
CRNDE	-1.601689	0.699228	-2.290653	0.02198349	NA
KMT2A	0.213925	0.093393	2.290586	0.02198738	0.19718489
PTTG1IP	0.529582	0.231216	2.290427	0.02199661	0.19718489
LGMN	0.663175	0.2896	2.289965	0.02202332	0.19718489
AIDA	0.213548	0.093272	2.289532	0.02204846	0.19718489
BMI1	0.31702	0.138468	2.289486	0.0220511	0.19718489
WDR13	-0.315277	0.137719	-2.289275	0.02206337	0.19718489
IFIT3	0.695749	0.304213	2.287046	0.02219316	0.198189272
GCHFR	-0.239236	0.104652	-2.28601	0.0222537	0.198574135
STX11	-0.447319	0.195744	-2.285221	0.0222999	0.198830562
ENSG00000272277	-0.964178	0.421991	-2.284834	0.02232256	0.198876851
EAPP	-0.199872	0.087489	-2.284535	0.0223401	0.198877531
A2M.AS1	-2.405551	1.054999	-2.280145	0.02259909	NA
ENSG00000267121	0.32664	0.143282	2.279695	0.02262581	0.201263629
IGHV1.2	2.071262	0.908779	2.279171	0.02265688	NA
MARK2	0.393841	0.172852	2.278484	0.02269776	0.201746028
UBR1	0.450211	0.197669	2.277598	0.02275056	0.202057628
POLR2J3	0.30915	0.135755	2.277267	0.02277029	0.202075233
HFE	-0.875356	0.384518	-2.276501	0.02281607	0.202323795
ACSF2	-0.413586	0.181724	-2.275905	0.02285172	0.20248221

SMYD5	-0.636399	0.279751	-2.274879	0.0229132	0.202766037
MSH2	-0.4843	0.2129	-2.274776	0.02291937	0.202766037
ATP5F1B	-0.223676	0.098376	-2.273681	0.02298517	0.203055458
CKLF	0.350155	0.154025	2.273363	0.02300431	0.203055458
RAB33B	0.543319	0.239025	2.273069	0.02302204	0.203055458
ZNF74	1.189331	0.523232	2.273046	0.02302341	0.203055458
ENSG00000259943	-0.582878	0.256588	-2.271649	0.02310775	0.203435893
SPTY2D1	0.497001	0.218788	2.27161	0.02311008	0.203435893
ENSG00000276216	-1.361777	0.599521	-2.271443	0.02312015	0.203435893
RGMB	0.917524	0.404016	2.27101	0.02314638	0.203494225
CD74	-0.243202	0.107111	-2.27056	0.0231736	0.203494225
PDE7B	1.0918	0.480917	2.270247	0.02319257	0.203494225
VBP1	-0.216407	0.095327	-2.270154	0.02319827	0.203494225
PODXL2	-0.914576	0.403001	-2.269415	0.02324308	0.203711648
SMARCA4	-0.330171	0.145504	-2.269156	0.02325884	0.203711648
PGD	-0.281383	0.124047	-2.26835	0.02330785	0.203984035
GARS	-0.365817	0.161326	-2.267565	0.02335572	0.204245904
TLCD1	-1.652176	0.728694	-2.267312	0.02337116	NA
GPRIN3	1.21321	0.53527	2.26654	0.02341836	0.204636542
ANAPC15	-0.295997	0.130735	-2.26411	0.02356736	0.205780636
HSH2D	-0.246179	0.10882	-2.262256	0.02368162	0.206619843
RPA3	0.187696	0.082992	2.261598	0.02372224	0.206815761
PSMC5	-0.230315	0.101877	-2.260729	0.02377607	0.20690735
ZNF70	1.02847	0.45493	2.26072	0.02377659	0.20690735
PSMB2	-0.244604	0.108205	-2.260548	0.02378726	0.20690735
TM2D3	0.303671	0.134371	2.259955	0.02382406	0.20706923
GNG11	1.161354	0.51413	2.258874	0.02389123	0.207391852
FADS1	-0.659297	0.291883	-2.258771	0.0238976	0.207391852
APBB1	0.861901	0.381663	2.258276	0.02392844	0.207501291
DENND6B	-0.427136	0.189216	-2.257397	0.02398325	0.207818363
SPATA20	0.875647	0.387999	2.25683	0.0240187	0.207967244
SLC25A5	-0.165817	0.0735	-2.256008	0.0240701	0.208207282
PTPN2	0.247248	0.109605	2.255803	0.024083	0.208207282
TYW3	0.349464	0.154982	2.254865	0.02414179	0.208451352
FAM234A	-0.421327	0.18686	-2.254769	0.02414784	0.208451352
POLE2	1.198928	0.532018	2.253549	0.02422456	0.208955203
PHKG2	-0.332109	0.147401	-2.253106	0.02425247	0.209037584
SOD1	-0.137932	0.061231	-2.252628	0.02428263	0.209139214
BANP	0.336349	0.14935	2.252088	0.02431671	0.209274443
COPS2	-0.304603	0.135304	-2.251251	0.02436962	0.209571382
FCGBP	-1.362771	0.60572	-2.249838	0.02445922	0.209653344
SP110	0.176791	0.078589	2.249552	0.02447742	0.209653344
LACTB2.AS1	-0.83193	0.369832	-2.249479	0.02448204	0.209653344
ITPR2	0.377368	0.16777	2.249308	0.02449288	0.209653344
NAGK	0.276758	0.123076	2.248666	0.02453377	0.209653344
STK38L	-0.483356	0.214958	-2.248602	0.02453782	0.209653344
TNIP2	-0.317087	0.141019	-2.248546	0.02454137	0.209653344

ZFP36L2	0.385855	0.171604	2.248514	0.02454341	0.209653344
NSUN5	-0.219434	0.097591	-2.248491	0.02454487	0.209653344
ENSG00000279278	0.430342	0.19144	2.247927	0.02458087	0.209654966
CYTH2	0.331784	0.14761	2.247702	0.0245952	0.209654966
TMEM170A	0.271363	0.120745	2.247417	0.0246134	0.209654966
GCFC2	-0.474439	0.211112	-2.247334	0.02461872	0.209654966
CYB5R4	-0.223178	0.099366	-2.246032	0.02470198	0.210163531
WNT16	1.250037	0.556605	2.245823	0.02471535	0.210163531
PWWP2A	0.422079	0.188041	2.244611	0.02479314	0.210667694
ENSG00000272758	0.780125	0.347687	2.243758	0.02484795	0.210975974
MGLL	-1.073254	0.478434	-2.243264	0.02487977	0.211088737
ZC3H12A	-0.362815	0.161863	-2.241492	0.0249942	0.211730606
EMP3	-0.174021	0.077643	-2.241303	0.02500643	0.211730606
TRAF2	-0.370818	0.165453	-2.24123	0.02501121	0.211730606
ZNF528	0.64461	0.287665	2.240839	0.02503651	0.211787294
PARP15	0.403233	0.179997	2.240216	0.02507693	0.211964379
TRAF3	-0.334428	0.149302	-2.239942	0.02509468	0.211964379
MFGE8	-0.752129	0.335896	-2.239172	0.02514471	0.212229523
NASP	0.237224	0.105957	2.238861	0.02516493	0.212242914
USP10	0.302694	0.135221	2.23851	0.02518782	0.21227868
OLA1	-0.217316	0.097095	-2.238179	0.02520938	0.212303224
ENTPD1.AS1	0.376512	0.168252	2.237783	0.02523524	0.21236393
ENSG00000255856	-1.807333	0.80839	-2.23572	0.02537009	NA
TOP2A	-1.299239	0.58125	-2.235251	0.02540086	0.213465257
ZNF677	0.586399	0.262346	2.235209	0.0254036	0.213465257
RMI1	0.76999	0.344595	2.23448	0.02545152	0.21355142
NPC2	0.29162	0.130519	2.23432	0.02546201	0.21355142
POU2F1	0.378774	0.169535	2.234197	0.02547012	0.21355142
DAGLB	0.666695	0.298449	2.233868	0.02549176	0.213575551
HEXB	0.28376	0.12705	2.233455	0.02551899	0.213646442
SUGP2	0.285073	0.127751	2.231483	0.02564916	0.214421653
ENSG00000268439	1.462558	0.655429	2.231453	0.02565114	0.214421653
RFX1	0.349659	0.156714	2.231197	0.02566808	0.214421653
MIR762HG	-0.621393	0.278625	-2.230211	0.02573345	0.214810141
ADGRB2	-1.867646	0.837677	-2.229553	0.02577712	NA
LSP1	-0.139924	0.062766	-2.2293	0.02579394	0.215017994
SLC35A3	0.475366	0.213253	2.229121	0.02580589	0.215017994
BCCIP	-0.304763	0.136727	-2.228984	0.025815	0.215017994
ZBTB44	0.234083	0.105051	2.228282	0.0258617	0.215103594
ARL6IP4	-0.152667	0.068514	-2.228262	0.02586306	0.215103594
S100A4	-0.296967	0.133322	-2.227436	0.02591815	0.215374119
SUPV3L1	-0.43627	0.195882	-2.227207	0.02593342	0.215374119
SNX18	0.614176	0.275846	2.226516	0.02597965	0.215600786
CRLF3	0.244343	0.109795	2.225459	0.02605044	0.21603081
MIS18A	0.45893	0.206449	2.222974	0.02621752	0.21725817
SHROOM3	-2.338417	1.052335	-2.222121	0.0262751	NA
AEBP2	0.382027	0.17194	2.221867	0.02629229	0.21763259

MICU2	-0.34956	0.157336	-2.221739	0.02630094	0.21763259
CD2BP2	-0.205576	0.092563	-2.220931	0.02635564	0.217926877
OAS2	0.517154	0.232892	2.220571	0.02638	0.217970004
DHRS4.AS1	0.456606	0.205667	2.220119	0.02641068	0.21806529
PRRG4	2.478839	1.116834	2.219524	0.02645112	NA
DNAAF4	-0.837609	0.377467	-2.219023	0.02648516	0.218521738
INSIG2	0.36663	0.165243	2.218734	0.02650485	0.218525848
MINDY1	1.326998	0.598437	2.217439	0.0265931	0.218906033
C19orf48	0.409683	0.184762	2.217357	0.02659872	0.218906033
TSC22D2	0.422742	0.190664	2.217212	0.02660864	0.218906033
LINC01857	0.811925	0.366487	2.215426	0.02673084	0.219752576
SCAMP2	-0.217361	0.098153	-2.214521	0.02679298	0.220104556
TLK2	0.471465	0.212928	2.214201	0.02681494	0.220126076
ICA1	-1.305978	0.589942	-2.213738	0.02684677	NA
PPCDC	-0.439927	0.198815	-2.212745	0.02691526	0.220790424
SFR1	-0.345329	0.156142	-2.211635	0.02699192	0.221259882
ENSG00000272669	1.634385	0.739218	2.210967	0.02703815	NA
RN7SL832P	1.648246	0.74566	2.210453	0.02707372	NA
SLC25A19	0.383003	0.173271	2.210429	0.02707542	0.221784689
BCL2	-0.305653	0.138325	-2.20968	0.0271274	0.222050765
CYHR1	0.350716	0.15877	2.208949	0.02717819	0.222306668
PTGES2	-0.3482	0.157663	-2.208509	0.02720881	0.222397322
HPRT1	-0.294771	0.133504	-2.207948	0.02724789	0.222557033
RAB1A	0.259573	0.117698	2.20542	0.0274246	0.223783587
MAP3K14	0.416541	0.188887	2.205238	0.02743737	0.223783587
ZNF284	0.816042	0.370137	2.2047	0.02747519	0.223790224
TMUB1	-0.230523	0.104562	-2.204667	0.02747749	0.223790224
TNFSF12	0.32817	0.148951	2.203215	0.02757959	0.224184409
HMOX1	0.412472	0.187222	2.203122	0.02758613	0.224184409
SHPRH	0.348331	0.158126	2.202875	0.02760353	0.224184409
ING2	0.459429	0.20856	2.20286	0.02760465	0.224184409
CPM	1.007934	0.457682	2.202257	0.02764717	0.224369696
UBXN11	-0.501471	0.227772	-2.201636	0.02769102	0.224565479
PDIA3	-0.215986	0.098118	-2.201284	0.02771594	0.224607645
EBLN3P	0.24689	0.112185	2.200726	0.0277554	0.224767417
TNFRSF13C	-0.165249	0.07512	-2.199809	0.02782048	0.225041641
CDK14	-0.480868	0.218607	-2.199691	0.02782881	0.225041641
RERE	0.376818	0.171335	2.199306	0.02785618	0.225041641
N4BP2L2	0.130936	0.05954	2.199135	0.02786832	0.225041641
ZFAND5	0.303727	0.138172	2.198177	0.02793647	0.225104968
PELP1	0.469655	0.213696	2.197771	0.02796546	0.225104968
SETBP1	0.356051	0.162026	2.197491	0.02798537	0.225104968
RRBP1	0.569926	0.259367	2.197375	0.02799369	0.225104968
PPP1CA	-0.157882	0.07185	-2.197371	0.02799396	0.225104968
NR4A2	0.784038	0.356809	2.197359	0.02799479	0.225104968
STK38	-0.300485	0.136799	-2.196548	0.02805277	0.22525649
TRMT6	-0.415359	0.189115	-2.196328	0.02806849	0.22525649

TXNIP	-0.283634	0.129144	-2.196256	0.02807364	0.22525649
ZNF439	0.255371	0.11629	2.195989	0.02809276	0.22525649
LINC00638	1.186105	0.540373	2.194975	0.02816537	0.225348133
THEM4	-0.368639	0.167951	-2.194923	0.02816915	0.225348133
HNRNPA1L2	-0.37756	0.172022	-2.19484	0.02817505	0.225348133
ENSG00000230709	0.551812	0.251427	2.194725	0.02818336	0.225348133
CCL5	-0.683932	0.311943	-2.192488	0.02834427	0.226475658
CLIP1	0.293829	0.13405	2.191935	0.0283842	0.226635716
CYSLTR1	-0.389707	0.177893	-2.190678	0.02847509	0.227202074
LENG8	-0.232388	0.106124	-2.189781	0.02854013	0.227561535
ENSG00000277369	-1.376315	0.628656	-2.189296	0.02857536	NA
LIX1L	0.350239	0.159987	2.18917	0.02858451	0.227755943
MALAT1	0.143718	0.0657	2.187496	0.02870637	0.228566943
ENSG00000263470	-2.087438	0.954293	-2.187419	0.02871197	NA
SPIN2B	0.514065	0.2351	2.186579	0.02877327	0.228910328
APOL3	-0.367487	0.168082	-2.186354	0.02878971	0.228910328
RANBP9	-0.446245	0.20416	-2.185761	0.02883309	0.229040496
UBAP2L	0.328844	0.150472	2.185419	0.02885815	0.229040496
CRIP2	-1.052476	0.481615	-2.185306	0.02886643	0.229040496
CAST	-0.215801	0.098773	-2.184814	0.0289025	0.229098041
PARP11	0.492646	0.225503	2.184658	0.02891392	0.229098041
XPO4	-0.393963	0.18043	-2.183464	0.02900164	0.229633249
HELZ2	0.523949	0.240016	2.182974	0.02903774	0.229759355
CHRNA6	-1.558914	0.714684	-2.181263	0.02916397	NA
IL2RA	-0.601376	0.275787	-2.180581	0.02921444	0.23099693
NUDT12	1.398805	0.641585	2.180235	0.02924004	0.231038906
ADGRE2	1.505991	0.691151	2.17896	0.02933466	0.231494384
UFC1	-0.15603	0.071609	-2.17891	0.02933835	0.231494384
ENSG00000273253	0.617049	0.283295	2.178119	0.02939719	0.231798088
APBB1IP	0.235163	0.107987	2.177692	0.02942897	0.23187823
UBE2T	0.535619	0.245986	2.177436	0.02944809	0.23187823
KCNA3	-0.859755	0.394916	-2.177055	0.02947643	0.231941031
IFIT1	1.804023	0.828771	2.176744	0.02949968	0.231963626
CALHM6	0.404419	0.185874	2.175768	0.02957264	0.23237684
NIFK.AS1	0.425726	0.195797	2.174328	0.02968048	0.233063371
NCBP2AS2	-0.267557	0.123116	-2.173203	0.02976508	0.233566604
GXYLT1	0.454379	0.209126	2.172751	0.02979907	0.233672322
TMEM189	0.44266	0.203904	2.170921	0.02993718	0.234403832
TEX264	-0.254463	0.117247	-2.170316	0.02998293	0.234403832
ZNF780A	0.403257	0.185806	2.170312	0.02998323	0.234403832
TBCD	0.64583	0.29759	2.170202	0.02999156	0.234403832
ENSG00000273117	-1.182932	0.545092	-2.170153	0.02999529	0.234403832
UBE2V2	-0.19763	0.0911	-2.16938	0.03005383	0.234700187
CLDND2	0.712202	0.32871	2.166658	0.03026096	0.236155783
CARS	-0.33331	0.153921	-2.16546	0.03035246	0.236707571
MICB	0.522348	0.241264	2.165048	0.03038403	0.236736849
PSMD4	-0.178976	0.082673	-2.164868	0.03039779	0.236736849

ENSG00000267939	-1.822026	0.842196	-2.163423	0.03050865	NA
ANKRD10	-0.326094	0.150799	-2.162435	0.03058465	0.237984591
ENOX2	-0.420838	0.194631	-2.162238	0.03059981	0.237984591
SNX29	-0.275376	0.127421	-2.161149	0.03068386	0.238475362
SNU13	-0.130817	0.06055	-2.16046	0.03073712	0.23868825
USP34	0.188619	0.087313	2.160252	0.03075318	0.23868825
CCDC144A	0.924674	0.428391	2.158481	0.03089047	0.239590559
CD59	-0.310102	0.143704	-2.157922	0.03093388	0.239763906
TRERF1	-0.885593	0.410495	-2.157381	0.03097597	0.239926797
ACAT2	-0.266527	0.123628	-2.155879	0.03109309	0.240670198
COPS7B	-0.258643	0.120009	-2.155186	0.03114725	0.240925694
PSMA3	-0.201136	0.093343	-2.154797	0.03117769	0.240997397
COX4I1	-0.116744	0.054206	-2.153699	0.03126381	0.241244245
CDC37L1	0.386088	0.179271	2.153655	0.03126723	0.241244245
NAGPA.AS1	1.785399	0.829207	2.153139	0.03130776	NA
EDEM1	-0.274988	0.127718	-2.153093	0.03131141	0.241244245
KLHL26	-0.860925	0.399955	-2.152556	0.03135359	0.241244245
DOCK7	1.09096	0.506852	2.152421	0.03136424	0.241244245
BCL6	0.538152	0.250089	2.151843	0.03140973	0.241244245
ZNF518A	0.257354	0.119599	2.151819	0.0314116	0.241244245
HTRA2	-0.295581	0.137363	-2.151818	0.03141166	0.241244245
ING3	-0.308914	0.143566	-2.151714	0.03141987	0.241244245
ZNF296	-0.316787	0.147261	-2.1512	0.03146039	0.241244245
TRAPPC8	0.396644	0.18439	2.151118	0.03146686	0.241244245
NUAK2	0.275202	0.127947	2.150907	0.03148354	0.241244245
PAQR7	-0.693332	0.322348	-2.150878	0.0314858	0.241244245
ELP6	-0.295612	0.137454	-2.150619	0.03150625	0.241244245
ZNF516	0.991761	0.461264	2.150094	0.03154776	0.241399715
IARS	-0.261243	0.121539	-2.149456	0.03159824	0.241623625
PDLIM1	-0.138065	0.06425	-2.148855	0.03164593	0.241812066
PIP4K2A	-0.231342	0.107671	-2.14861	0.03166536	0.241812066
NFATC2	0.397581	0.185152	2.147321	0.03176773	0.242431202
PM20D2	0.519012	0.241842	2.146079	0.0318667	0.242981161
SIDT1.AS1	-0.567314	0.264374	-2.145881	0.03188247	0.242981161
COPZ1	-0.21088	0.09833	-2.144613	0.03198379	0.243590233
SNHG30	-0.292229	0.13632	-2.143696	0.03205722	0.243986266
FHOD1	0.499112	0.232931	2.142742	0.03213378	0.244405637
CCDC112	0.377288	0.1761	2.142458	0.03215668	0.244416527
HEBP2	-0.501527	0.234167	-2.141751	0.03221354	0.244685376
C12orf42	0.573585	0.267867	2.141308	0.03224919	0.244792851
RSU1	-0.263633	0.12316	-2.140579	0.03230797	0.244913898
GFPT1	0.524898	0.245214	2.140577	0.03230816	0.244913898
MRPL45	0.26962	0.125989	2.140024	0.03235283	0.245089375
GYPC	0.291776	0.136408	2.138995	0.03243609	0.245556749
RETREG2	0.247172	0.115573	2.138666	0.03246273	0.245595096
RNF146	0.323124	0.151168	2.137521	0.03255564	0.246134452
IL6ST	0.879379	0.411517	2.136918	0.0326047	0.246182787

ENSG00000276728	0.542341	0.253828	2.136648	0.03262665	0.246182787
NHSL2	-0.329325	0.154146	-2.136445	0.03264314	0.246182787
NDUFV2	0.16864	0.078937	2.136379	0.03264852	0.246182787
ZFYVE16	0.405641	0.190138	2.133406	0.0328914	0.247850113
TERF1	0.248214	0.116419	2.13208	0.03300025	0.248368679
MIR29B2CHG	0.375195	0.17598	2.132036	0.03300385	0.248368679
UTP14C	0.634967	0.297945	2.131153	0.03307654	0.248751315
ARL5A	0.186054	0.087358	2.129777	0.03319003	0.249440055
ANKRD40	-0.335545	0.157653	-2.128376	0.03330595	0.250146164
ITGA6.AS1	-0.645551	0.303626	-2.126141	0.03349155	0.251171464
ZNF232	0.643168	0.302523	2.12601	0.03350242	0.251171464
DEGS2	1.476816	0.694666	2.125935	0.03350865	0.251171464
NDUFAB1	-0.201612	0.094886	-2.124777	0.03360527	0.2514239
CCT7	-0.186237	0.087651	-2.12475	0.03360752	0.2514239
OSBPL9	-0.283322	0.133345	-2.124737	0.03360857	0.2514239
ALOX5	-0.276892	0.130368	-2.123937	0.03367539	0.251758325
FTH1	0.247894	0.116764	2.12303	0.03375135	0.251996807
PLEKHA1	0.402688	0.189696	2.122803	0.03377034	0.251996807
FMO5	0.830732	0.391344	2.122763	0.03377368	0.251996807
PBXIP1	-0.258336	0.121717	-2.122435	0.03380119	0.25203689
MTHFD2	-0.413098	0.194688	-2.121844	0.03385081	0.252241704
IGF1R	1.420201	0.669974	2.119786	0.03402413	0.253027663
MEF2A	0.339319	0.160118	2.119182	0.03407508	0.253027663
FAM98C	0.313264	0.147842	2.118915	0.03409762	0.253027663
TRIM35	0.839711	0.396298	2.118888	0.03409996	0.253027663
ENSG00000270562	1.15662	0.545944	2.118567	0.03412707	0.253027663
OTUB1	-0.195456	0.092261	-2.118509	0.03413195	0.253027663
TMEM237	1.674042	0.790212	2.118473	0.03413506	NA
ARHGAP5	0.568218	0.268238	2.118336	0.03414663	0.253027663
CD200	0.770205	0.36359	2.118331	0.03414708	0.253027663
POLR3D	0.421808	0.199139	2.118161	0.03416146	0.253027663
SCARB1	-1.060393	0.500694	-2.117845	0.03418823	0.253027663
MRPL3	-0.27013	0.127558	-2.117697	0.03420074	0.253027663
TAMM41	0.588141	0.277873	2.11658	0.03429554	0.253203433
PIP5K1B	0.610286	0.288338	2.116563	0.03429697	0.253203433
PPP6C	-0.219526	0.103723	-2.116474	0.03430452	0.253203433
GTF2H5	-0.24674	0.116596	-2.116203	0.03432758	0.253203433
BLVRA	-0.432603	0.204433	-2.116107	0.03433568	0.253203433
SET	-0.152402	0.072036	-2.115627	0.03437653	0.25321578
SDHB	-0.185193	0.087538	-2.115565	0.03438184	0.25321578
FAM216A	0.44976	0.212664	2.114883	0.03443991	0.25331665
NAGS	1.237353	0.585111	2.114733	0.03445273	0.25331665
TMEM106A	0.335589	0.158699	2.114621	0.03446228	0.25331665
MANBA	0.337927	0.159831	2.11428	0.03449137	0.253366902
LARP7	-0.200502	0.094889	-2.113023	0.03459884	0.253992488
KCNN1	1.698386	0.803881	2.112732	0.03462368	NA
VAPA	-0.154568	0.073187	-2.111961	0.03468976	0.254495905

AHI1	0.351184	0.166322	2.111464	0.03473248	0.254645212
ENSG00000272871	0.937503	0.444131	2.110871	0.03478337	0.254854241
LINC01250	-3.131219	1.483587	-2.110574	0.03480898	NA
MDS2	-1.182526	0.560301	-2.110519	0.03481372	0.254912512
CEP170	0.312034	0.147869	2.110207	0.03484052	0.254944843
SH3RF1	-0.83762	0.397012	-2.109808	0.03487489	0.255032449
MED28	0.191446	0.09076	2.109364	0.0349132	0.255148708
RNASEH2C	-0.138185	0.065548	-2.108163	0.03501689	0.255742373
FAM133B	0.149338	0.070861	2.107468	0.035077	0.256017148
ENSG00000261770	1.289838	0.612316	2.10649	0.03516179	0.256271086
LRRC7	-0.763264	0.362352	-2.106413	0.03516848	0.256271086
UTP4	-0.411346	0.195294	-2.106288	0.03517932	0.256271086
RSL1D1	-0.155223	0.073713	-2.105788	0.0352228	0.256423759
KLHL34	1.657041	0.787324	2.104649	0.03532184	NA
ASAH1	0.355673	0.169035	2.104142	0.03536607	0.257302284
ENSG00000271746	-1.427301	0.678623	-2.103231	0.03544557	NA
MED8	-0.269606	0.128214	-2.102778	0.03548518	0.257879742
DPH5	0.28164	0.133941	2.102714	0.03549074	0.257879742
ZNF485	0.970984	0.46192	2.102063	0.03554778	0.258129471
LPAR2	-0.589229	0.2804	-2.101387	0.03560699	0.258394656
B3GALNT2	0.515933	0.245696	2.09988	0.0357394	0.259100316
PEX16	-0.225549	0.107438	-2.099346	0.03578638	0.259100316
TACO1	-0.353594	0.168433	-2.099309	0.03578966	0.259100316
DMAP1	-0.287324	0.13687	-2.099246	0.03579526	0.259100316
BOLA1	0.349672	0.166595	2.098941	0.03582211	0.259129967
INO80B	0.417098	0.198746	2.098649	0.03584786	0.25915155
FOXP4	0.663615	0.316394	2.097435	0.03595505	0.259761555
AHR	-0.574787	0.27413	-2.096767	0.03601419	0.259955273
NDUFV3	0.264824	0.12631	2.096617	0.03602753	0.259955273
DUT	-0.146186	0.069759	-2.095586	0.03611892	0.260374241
MRPS35	-0.274286	0.130896	-2.095446	0.03613133	0.260374241
DNAJC3.DT	0.956413	0.456522	2.094997	0.03617128	0.260497295
ZNF385A	0.940442	0.44904	2.094341	0.03622965	0.260752724
KDM2B	-0.247263	0.118128	-2.093167	0.03633428	0.261246345
PFDN2	0.166002	0.079311	2.093056	0.03634413	0.261246345
MTAP	0.478886	0.228846	2.092618	0.03638329	0.261362863
VAMP3	-0.292446	0.139824	-2.091526	0.03648097	0.261826983
AZIN1	-0.243781	0.116565	-2.091381	0.03649389	0.261826983
CSDE1	-0.142548	0.068195	-2.090312	0.03658975	0.262331591
NAV2	-1.135472	0.543266	-2.090083	0.0366103	0.262331591
MAVS	0.307204	0.147092	2.08851	0.03675189	0.263180481
PDPR	-0.616332	0.295193	-2.087897	0.03680713	0.263407653
TAF9	-0.199268	0.095451	-2.087645	0.03682988	0.263407653
RARA.AS1	0.703981	0.337316	2.087005	0.03688764	0.263511664
ENSG00000254531	0.485694	0.232727	2.086972	0.03689071	0.263511664
SNX10	-0.296366	0.142033	-2.086604	0.03692391	0.26351546
RACGAP1	-0.745042	0.357122	-2.086241	0.03695682	0.26351546

BLOC1S3	0.414578	0.198724	2.086198	0.03696067	0.26351546
RARA	-0.273034	0.130903	-2.085774	0.0369991	0.263624378
METTL23	-0.167994	0.080571	-2.085052	0.03706461	0.263925998
FCER2	-0.763355	0.366157	-2.084776	0.03708967	0.263939382
SNHG25	-0.33541	0.160937	-2.084104	0.03715074	0.264208849
WWP1	0.511222	0.245403	2.083197	0.03723331	0.264630725
POR	-0.388654	0.186719	-2.081487	0.03738936	0.265540092
CLNS1A	0.283795	0.136366	2.081118	0.0374231	0.265540092
ALDH6A1	0.502928	0.241698	2.080813	0.03745101	0.265540092
LZTFL1	0.651398	0.313069	2.080686	0.03746261	0.265540092
ENSG00000272189	1.001026	0.481145	2.080507	0.03747905	0.265540092
FBXL12	-0.370068	0.177895	-2.080266	0.03750118	0.265540092
FAM177A1	-0.212141	0.102012	-2.079564	0.03756556	0.265718383
SCAMP3	-0.285409	0.13725	-2.079482	0.03757304	0.265718383
SRA1	-0.242225	0.116516	-2.078898	0.03762673	0.265932917
SLTM	0.158493	0.076262	2.078265	0.03768497	0.266179286
DERL1	-0.246153	0.118486	-2.077483	0.03775705	0.266459882
DCK	-0.144862	0.069746	-2.077001	0.03780146	0.266459882
ARF4	-0.298756	0.143849	-2.076869	0.03781364	0.266459882
PCK2	-0.424394	0.204348	-2.076819	0.0378183	0.266459882
ENSG00000228434	0.382708	0.184312	2.076411	0.03785593	0.266560063
PATL2	0.364449	0.175623	2.075173	0.03797048	0.267201432
CNKSR3	-1.158748	0.558542	-2.074594	0.03802418	0.267414005
ZNF506	0.325285	0.156819	2.07427	0.03805425	0.267460285
FAM41C	-0.538853	0.25986	-2.073625	0.03811419	0.267646681
ARL11	0.467167	0.225321	2.073342	0.03814044	0.267646681
SKA2	-0.272679	0.131524	-2.073226	0.03815129	0.267646681
ZNF430	0.215546	0.104003	2.072499	0.03821895	0.267956234
GATAD2B	0.308534	0.148992	2.070816	0.03837601	0.268712649
BCL2A1	-0.381107	0.184051	-2.070656	0.038391	0.268712649
NEB	1.193679	0.576494	2.070585	0.03839764	0.268712649
DCTN3	-0.211833	0.102357	-2.069556	0.03849398	0.269221417
TLE4	-0.389209	0.188115	-2.068996	0.03854649	0.269423193
LYN	0.237825	0.115035	2.067416	0.03869496	0.270189617
H2AFY	-0.198238	0.095891	-2.067324	0.03870361	0.270189617
SCCPDH	0.369098	0.178686	2.065621	0.0388643	0.271145164
CEP126	0.62897	0.304563	2.065154	0.03890843	0.271286855
ATF3	-0.79091	0.383123	-2.064375	0.0389822	0.271634859
GLRX2	0.333318	0.161684	2.061534	0.0392521	0.273348285
TWNK	-0.676239	0.328126	-2.060913	0.03931137	0.273416929
JTB	-0.103045	0.050001	-2.060865	0.03931593	0.273416929
CD2	-2.306236	1.119233	-2.060551	0.03934591	NA
ENSG00000229255	-1.387686	0.673481	-2.060468	0.03935381	NA
RARS	0.292035	0.141732	2.060466	0.03935397	0.273416929
TFRC	-0.530245	0.257347	-2.060424	0.03935801	0.273416929
ABHD4	0.600663	0.291609	2.059822	0.03941556	0.273649706
CSTF3	0.366746	0.178264	2.057318	0.03965562	0.27514864

MED23	-0.421329	0.2049	-2.056269	0.03975661	0.275670492
LAMC1	1.117449	0.543529	2.055916	0.03979066	0.275670492
PLCB2	0.557548	0.27121	2.055783	0.03980347	0.275670492
PPP3CA	0.183586	0.08933	2.055152	0.03986433	0.275697549
CD1D	0.484891	0.23595	2.055062	0.03987304	0.275697549
GUCD1	-0.228831	0.111354	-2.05499	0.03988002	0.275697549
RAB11FIP3	-1.045402	0.509018	-2.053762	0.03999876	0.276350662
HCFC1	0.356511	0.173692	2.052542	0.04011703	0.276999721
STK26	0.375227	0.182873	2.051843	0.04018496	0.277300566
MRPS24	0.814401	0.396982	2.051481	0.04022009	0.277374885
ABHD14A	0.316385	0.154321	2.050179	0.04034699	0.278081635
SLC5A6	-0.450174	0.219668	-2.04934	0.04042889	0.278429381
C21orf62.AS1	0.844339	0.412079	2.048973	0.04046472	0.278429381
PCCB	-0.578038	0.28212	-2.048911	0.04047081	0.278429381
RNPS1	-0.124504	0.060787	-2.048202	0.04054019	0.278524931
LINC02202	-1.180736	0.576504	-2.048097	0.0405505	0.278524931
EIF4H	-0.161303	0.07876	-2.04802	0.04055808	0.278524931
CASP7	0.398385	0.194695	2.0462	0.04073668	0.279582762
SPATA2L	0.473648	0.231548	2.04557	0.04079873	0.279685886
SEC13	-0.293678	0.143569	-2.045548	0.04080083	0.279685886
DCTPP1	-0.206192	0.100904	-2.043444	0.04100854	0.280888401
PDCL3	-0.270704	0.132485	-2.043271	0.04102559	0.280888401
GNA11	1.395906	0.683466	2.042394	0.04111245	0.281313892
WFS1	-1.40508	0.688079	-2.042035	0.0411481	NA
ATP5F1C	-0.193839	0.094989	-2.040648	0.04128579	0.282330341
PTP4A2	-0.172707	0.084653	-2.040168	0.04133355	0.282382106
YDJC	-0.239327	0.117313	-2.040074	0.04134296	0.282382106
MBOAT2	-1.688592	0.827902	-2.039603	0.0413899	NA
NPHP3	0.381052	0.186855	2.039293	0.04142075	0.282743791
PTPN20	-1.34917	0.66206	-2.037837	0.04156629	0.283567246
GLI4	-0.600314	0.294661	-2.037303	0.04161972	0.283677815
ALDOA	-0.12106	0.059425	-2.037177	0.04163232	0.283677815
COX8A	-0.108718	0.053379	-2.036702	0.04167988	0.283831987
APBB2	-1.215692	0.597039	-2.036202	0.04173005	0.284003808
TTC28	0.62354	0.306372	2.035236	0.04182714	0.284494486
CHP1	-0.232745	0.114385	-2.034745	0.04187655	0.284518238
RPRD2	0.298063	0.14649	2.034705	0.04188061	0.284518238
CEP85L	0.43149	0.212154	2.033851	0.04196659	0.284750739
ENSG00000237491	-0.58088	0.285658	-2.033484	0.04200369	0.284750739
MTX3	-0.539546	0.265338	-2.033426	0.04200955	0.284750739
CYFIP1	-0.953944	0.469144	-2.033373	0.04201486	0.284750739
ZMYM6	0.447637	0.220211	2.032764	0.04207635	0.284874621
IL18BP	-0.747213	0.367597	-2.032697	0.04208318	0.284874621
POLR1B	-0.565726	0.278373	-2.032259	0.04212746	0.285004897
EHD3	-0.521672	0.256756	-2.031784	0.04217552	0.285031944
LY96	0.282265	0.138943	2.031514	0.04220288	0.285031944
GOPC	0.345331	0.169999	2.031366	0.04221792	0.285031944

DPYD	0.74109	0.364848	2.031231	0.04223159	0.285031944
OAZ2	0.18981	0.09347	2.030703	0.04228517	0.285224467
PSMD8	-0.152346	0.075053	-2.029846	0.04237221	0.285507644
ZNF626	0.341341	0.168165	2.029796	0.0423773	0.285507644
TPP1	0.272744	0.134395	2.029419	0.04241567	0.285594958
GDPD1	1.18851	0.585711	2.029175	0.04244042	0.285594958
DYRK1B	0.467209	0.230286	2.02882	0.04247666	0.285669983
ENSG00000258377	-1.338801	0.660165	-2.02798	0.04256226	NA
LINC02413	-0.721535	0.355917	-2.027259	0.0426359	0.286571648
MMAB	-0.259223	0.12792	-2.026452	0.04271845	0.286901033
MTHFD2L	-0.380884	0.187986	-2.026136	0.04275084	0.286901033
ENSG00000260979	-0.728284	0.359461	-2.026042	0.0427605	0.286901033
ENSG00000269983	1.325527	0.654377	2.025634	0.04280228	0.287012226
TOB1	0.354859	0.175231	2.02509	0.0428581	0.287217366
WDR76	-0.410969	0.203046	-2.024022	0.04296793	0.287784031
AP5S1	-0.49991	0.247037	-2.023623	0.04300893	0.287889312
SLFN13	-0.788378	0.389718	-2.022948	0.04307852	0.288185702
IGHG3	0.498481	0.246529	2.022	0.04317631	0.28860915
FBXL17	0.513996	0.254221	2.021844	0.04319252	0.28860915
CRIPT	0.237104	0.117327	2.020878	0.0432924	0.289099281
ZNF420	0.436299	0.215945	2.02042	0.04333987	0.289099281
ZNF257	0.673246	0.333224	2.020399	0.04334204	0.289099281
FAM43A	0.621517	0.307691	2.019941	0.04338954	0.289190044
ZC3H14	0.334401	0.165565	2.019756	0.04340872	0.289190044
PPP3R1	0.416049	0.206014	2.019516	0.0434336	0.289190044
KMT2E	0.105626	0.05231	2.019245	0.04346177	0.289190044
BRD4	0.192904	0.095542	2.019044	0.04348264	0.289190044
SH2B2	-1.159086	0.574213	-2.018564	0.04353251	0.289299552
CBR4	0.274482	0.13599	2.018397	0.04354992	0.289299552
LYZ	1.38644	0.68697	2.018195	0.04357094	NA
ZNF33A	0.214248	0.10622	2.017027	0.04369273	0.289916275
CTNNBL1	-0.228751	0.11342	-2.016855	0.0437107	0.289916275
DMD	0.478684	0.237352	2.016774	0.04371915	0.289916275
KCNG1	1.420877	0.704649	2.016433	0.04375475	0.289983489
ENSG00000224934	-1.971117	0.977909	-2.015644	0.04383725	NA
R3HDM4	-0.180717	0.089657	-2.015636	0.04383809	0.290140342
TAF1A	0.53062	0.263272	2.015482	0.04385412	0.290140342
AMIGO2	0.847007	0.420254	2.015463	0.04385615	0.290140342
CDK5RAP1	-0.302392	0.150067	-2.01505	0.04389946	0.290140342
NPAT	-0.29448	0.146145	-2.014989	0.04390583	0.290140342
GPRASP1	1.541166	0.765042	2.014487	0.04395847	NA
CAPN10.DT	0.425877	0.211414	2.014419	0.04396558	0.290366629
EFNA3	0.849456	0.421762	2.014063	0.04400297	0.290445097
GATD1	0.368069	0.182858	2.012871	0.04412822	0.291103063
TRIR	0.104451	0.051913	2.012026	0.04421725	0.291521457
RFESD	-1.104588	0.549298	-2.01091	0.044335	0.292022499
NMNAT1	0.486839	0.24211	2.010819	0.04434454	0.292022499

SGPP1	0.444953	0.221374	2.00996	0.04443543	0.292451916
PRRG2	-0.652407	0.324763	-2.008868	0.0445511	0.293043769
MAN1A1	0.537891	0.267817	2.008426	0.04459803	0.293183146
ZNF500	0.432052	0.215234	2.007361	0.04471123	0.29375765
ZNF441	-0.363664	0.181244	-2.006492	0.04480383	0.293972422
TRMT112	0.137446	0.068505	2.006375	0.04481625	0.293972422
SLC37A2	0.804213	0.400838	2.006327	0.04482137	0.293972422
SPAG1	0.518556	0.258512	2.005929	0.04486385	0.294046562
ENSG00000255284	0.477979	0.238346	2.005397	0.04492067	0.294046562
POLR2J	-0.172268	0.085938	-2.004559	0.04501025	0.294046562
ENO1.AS1	-1.292785	0.644934	-2.004523	0.04501404	NA
GAB1	1.167674	0.582523	2.004512	0.04501521	0.294046562
GALK1	0.329624	0.164466	2.004205	0.04504806	0.294046562
ABTB1	-0.228451	0.114016	-2.003676	0.04510475	0.294046562
TSN	-0.211148	0.105385	-2.003591	0.04511386	0.294046562
CNN2	-0.286875	0.143182	-2.003563	0.04511687	0.294046562
SMCO4	0.551804	0.275414	2.003542	0.04511911	0.294046562
ZNF589	-0.53517	0.267115	-2.003523	0.04512117	0.294046562
NDUFV2.AS1	-0.438963	0.219109	-2.003403	0.04513405	0.294046562
SEC11C	-0.218902	0.109279	-2.00314	0.04516227	0.294046562
STOML1	-0.875596	0.437215	-2.002667	0.04521304	0.294046562
ETV3	0.658882	0.329005	2.002652	0.04521467	0.294046562
HMGN2	0.113676	0.056764	2.002602	0.04522005	0.294046562
STAM2	0.589683	0.294526	2.002145	0.04526911	0.29419755
LINC00944	-0.697183	0.348391	-2.001152	0.04537605	0.294724289
EPN2	0.672325	0.336121	2.000248	0.04547351	0.295188909
TMEM208	-0.212369	0.1062	-1.99971	0.04553155	0.295310525
ELF2	0.194883	0.097461	1.999594	0.04554411	0.295310525
XPC	-0.254643	0.127366	-1.999302	0.04557574	0.295319034
IQGAP1	0.171581	0.085829	1.999102	0.0455973	0.295319034
PDS5A	0.270788	0.135492	1.998549	0.04565722	0.295423861
PIGH	-0.32172	0.160983	-1.998473	0.04566538	0.295423861
SMPD2	-0.335815	0.16806	-1.99818	0.04569714	0.295461435
KRAS	0.205385	0.102814	1.997633	0.04575644	0.295557365
SCP2	-0.166532	0.083368	-1.997565	0.04576389	0.295557365
GIMAP7	-2.669102	1.33678	-1.996666	0.04586152	NA
SREBF2.AS1	-0.615028	0.308068	-1.996403	0.04589008	0.296204332
MFSD2B	-0.751783	0.376733	-1.995535	0.04598459	0.296554147
ZBTB32	-0.472615	0.236849	-1.995427	0.04599637	0.296554147
SLC25A25	0.515894	0.258589	1.995034	0.04603917	0.296662122
PPP1R37	0.412794	0.206947	1.994683	0.04607743	0.296709433
THAP3	0.33196	0.166459	1.994242	0.04612564	0.296709433
SYBU	-1.012427	0.507695	-1.994165	0.04613402	0.296709433
BAD	-0.209413	0.105022	-1.993989	0.0461533	0.296709433
PLCL2	-0.413922	0.207607	-1.993774	0.04617681	0.296709433
ZSWIM6	0.606091	0.30407	1.993266	0.04623236	0.29689882
RPL7L1	0.198218	0.09953	1.991533	0.04642231	0.29795063

AFTPH	0.20394	0.102418	1.991259	0.04645247	0.29797623
DHRS11	-0.837461	0.420728	-1.990506	0.0465352	0.298226612
DTX3L	0.347655	0.174676	1.990279	0.04656024	0.298226612
ETV6	-0.280018	0.140699	-1.990189	0.04657008	0.298226612
RBM5	0.225611	0.113407	1.989393	0.04665785	0.298620731
ATG4C	0.306685	0.154275	1.987908	0.04682187	0.299502162
ENSG00000268352	1.589442	0.799589	1.987825	0.04683099	NA
TMEM230	-0.174561	0.08785	-1.98703	0.0469191	0.299955573
RPP25L	-0.258737	0.130249	-1.986485	0.04697954	0.300173397
ZNF395	0.293244	0.147661	1.985934	0.04704071	0.30039567
DTD2	-0.5111	0.257428	-1.985413	0.04709855	0.30059649
TRIM38	0.167652	0.084487	1.984363	0.04721544	0.301005022
SAE1	-0.281942	0.142082	-1.984363	0.04721542	0.301005022
LYRM1	-0.251057	0.126573	-1.983485	0.04731324	0.30134174
COX6A1	-0.11696	0.058969	-1.983414	0.04732119	0.30134174
OTUD1	0.617889	0.311605	1.982925	0.04737586	0.301370606
CD47	0.158388	0.079877	1.982899	0.04737866	0.301370606
RTRAF	-0.119793	0.06044	-1.982021	0.04747685	0.301647974
ZNF292	0.194634	0.098204	1.981928	0.04748731	0.301647974
CRACR2B	-0.624437	0.31513	-1.98152	0.04753303	0.301647974
CDKN2A	-0.774931	0.391133	-1.981248	0.04756342	0.301647974
RBM42	-0.236386	0.119318	-1.981143	0.04757526	0.301647974
PRMT6	0.527092	0.266072	1.981017	0.04758932	0.301647974
PDCD7	0.391464	0.197624	1.980853	0.04760771	0.301647974
HSPA5	-0.319645	0.161442	-1.979934	0.04771091	0.302133699
MST1	1.330508	0.672121	1.979567	0.04775218	0.302187597
AHRR	-1.310865	0.662275	-1.979337	0.04777811	0.302187597
TARBP1	-0.444572	0.224628	-1.979151	0.04779904	0.302187597
MRRF	0.384764	0.194463	1.978592	0.04786191	0.302417132
ENSG00000224220	-0.67378	0.340635	-1.978013	0.0479272	0.302578412
TXLNB	-0.725263	0.366705	-1.977785	0.047953	0.302578412
VPS35	0.199472	0.100884	1.97724	0.04801452	0.302578412
CXorf65	-0.838996	0.424338	-1.977189	0.04802031	0.302578412
LRCH4	-0.172122	0.087057	-1.97711	0.04802924	0.302578412
GRAPL	0.881017	0.445646	1.976943	0.04804806	0.302578412
PWWP2B	0.562526	0.284592	1.976603	0.04808653	0.302578412
SNX21	1.264245	0.639675	1.976387	0.04811093	0.302578412
TXNDC15	0.294988	0.149288	1.975962	0.04815911	0.302578412
CHRNE	1.489995	0.754122	1.975802	0.04817724	0.302578412
DHRS1	-0.289412	0.146491	-1.975636	0.04819604	0.302578412
DONSON	0.937354	0.474549	1.975252	0.04823951	0.302578412
LZTS2	0.532498	0.269636	1.974875	0.04828235	0.302578412
ENSG00000262049	-0.394189	0.199606	-1.974832	0.04828723	0.302578412
LTO1	-0.392347	0.198681	-1.974756	0.0482959	0.302578412
KARS	-0.196168	0.099345	-1.974608	0.04831263	0.302578412
ENSG00000272053	-0.648341	0.328429	-1.974069	0.04837388	0.302795499
PXK	0.29097	0.147449	1.973358	0.04845475	0.302973321

LINC00672	0.650568	0.329691	1.973264	0.04846553	0.302973321
PTCD1	-0.514881	0.260965	-1.972989	0.04849684	0.302973321
KLHL5	-0.325922	0.165201	-1.972884	0.04850873	0.302973321
ERF	0.507834	0.257565	1.971674	0.04864689	0.303669656
EIF3E	0.168396	0.085429	1.971173	0.04870407	0.303859993
HMGCS1	-0.308212	0.15653	-1.969021	0.04895068	0.305015115
ADAM15	0.765381	0.388722	1.968965	0.04895706	0.305015115
TMEM59	-0.117538	0.059699	-1.968856	0.04896959	0.305015115
WDR11	-0.239539	0.1217	-1.968274	0.04903658	0.305015115
PARP12	-0.441403	0.224299	-1.967926	0.04907658	0.305015115
SOGA1	0.52001	0.26427	1.967722	0.04910006	0.305015115
MAD2L1	-0.652494	0.331633	-1.967518	0.04912353	0.305015115
H3F3B	-0.111063	0.056455	-1.967268	0.04915229	0.305015115
PDPK1	0.307101	0.156106	1.967259	0.04915333	0.305015115
APC	0.348693	0.177263	1.9671	0.04917164	0.305015115
LMCD1	-1.181356	0.600589	-1.966994	0.04918389	0.305015115
C4orf36	0.713331	0.362742	1.966496	0.04924136	0.3052053
NCK1	0.225183	0.114566	1.965529	0.04935308	0.30556225
CYB5R1	-0.336082	0.170999	-1.965405	0.04936737	0.30556225
PRR7	-0.585461	0.29792	-1.965161	0.04939565	0.30556225
SLC25A17	0.45408	0.231076	1.965069	0.0494063	0.30556225
CD226	-0.982828	0.50031	-1.964437	0.04947942	0.30575812
DNMT3A	0.466579	0.237545	1.964172	0.04951015	0.30575812
BRD3OS	0.385572	0.196328	1.963922	0.04953915	0.30575812
LRPAP1	-0.188928	0.096211	-1.963678	0.04956749	0.30575812
ENPP4	-0.639681	0.325792	-1.963465	0.04959221	0.30575812
ERLIN1	0.619619	0.31561	1.963243	0.049618	0.30575812
NCKAP5L	0.759262	0.386797	1.962945	0.04965257	0.30575812
IFNAR1	-0.186854	0.095191	-1.962943	0.0496528	0.30575812
FBXL5	-0.26722	0.136189	-1.962126	0.04974776	0.306177302
AP1S3	-0.30821	0.157184	-1.960826	0.04989929	0.3066074
DGAT2	0.67582	0.34467	1.960773	0.04990548	0.3066074
INTS2	0.764816	0.390065	1.960742	0.04990913	0.3066074
CEP19	0.464908	0.237153	1.960373	0.04995219	0.3066074
HUWE1	0.295783	0.150881	1.960372	0.04995229	0.3066074

Cluster 6	log2FC	lfcSE	stat	pvalue	padj
RPL8	0.291116	0.078616	3.702999	0.000213066	0.999975
RPS5	0.217438	0.064063	3.394151	0.000688418	0.999975
ENSG00000272211	-1.924375	0.586996	-3.278343	0.001044186	0.999975
RPS9	0.324713	0.111146	2.921486	0.003483661	0.999975
GATD3	3.793876	1.342504	2.825971	0.004713757	0.999975
ZBED2	2.666112	0.957528	2.784371	0.005363168	0.999975
IGHA1	-1.029965	0.372817	-2.762654	0.005733349	0.999975
ARRDC3	-1.260022	0.483171	-2.607816	0.009112182	0.999975
MT.ND2	-0.306245	0.124422	-2.461346	0.013841672	0.999975
MT.ND3	-0.342426	0.140316	-2.440388	0.014671479	0.999975
CKS2	1.249205	0.527946	2.366163	0.017973543	0.999975
H3F3B	-0.451573	0.194619	-2.320289	0.02032526	0.999975
CCDC191	-1.459879	0.642009	-2.273921	0.022970712	0.999975
FKBP5	1.523412	0.684697	2.224943	0.026085098	0.999975
ENSG00000279483	-0.50764	0.23418	-2.167736	0.030178767	0.999975
RGCC	2.272594	1.080785	2.102726	0.035489745	0.999975
USP53	1.261884	0.605639	2.08356	0.037200171	0.999975
ZNF107	1.405976	0.680553	2.06593	0.038835044	0.999975
HLA.C	-0.577522	0.282867	-2.041673	0.041183964	0.999975
FCGR2B	1.173359	0.577103	2.033187	0.042033681	0.999975
HLA.A	-0.478721	0.237665	-2.014272	0.043980934	0.999975
MT.CO1	0.170556	0.084934	2.00809	0.04463373	0.999975
RPS6KA2	1.484232	0.739227	2.007816	0.044662896	0.999975
RPS8	0.139498	0.070415	1.981086	0.047581593	0.999975
TCL1B	-1.198105	0.609515	-1.965671	0.04933664	0.999975

Cluster 7	log2FC	lfcSE	stat	pvalue	padj
NFKBIA	-0.589599	0.108085	-5.454954	4.90E-08	0.000153717
CD83	-0.596054	0.114193	-5.219688	1.79E-07	0.000219207
ENSG00000272211	-1.304309	0.251281	-5.190645	2.10E-07	0.000219207
RSRP1	0.510122	0.111103	4.591446	4.40E-06	0.003453258
RPL8	0.392014	0.088595	4.424784	9.65E-06	0.006058769
IL4R	-0.603296	0.147597	-4.087463	4.36E-05	0.022808867
CKS2	1.314245	0.332853	3.948426	7.87E-05	NA
DHTKD1	-0.720035	0.183639	-3.920932	8.82E-05	NA
ARRDC3	-0.922866	0.238396	-3.871148	0.000108324	0.048560153
HHEX	0.438362	0.115716	3.788267	0.000151702	0.059505099
TNFAIP8L2	1.098924	0.291262	3.772975	0.000161313	NA
LMO4	0.839182	0.228088	3.679203	0.000233964	NA
UCP2	-0.468388	0.128721	-3.638776	0.000273937	0.095512832
CYB5A	-0.644184	0.181004	-3.558953	0.000372336	0.099575711
PFKFB3	-0.663773	0.18778	-3.534837	0.000408027	0.099575711
SPINT2	-0.474838	0.135254	-3.510707	0.000446917	0.099575711
SYNGR2	-0.742773	0.211756	-3.507677	0.000452037	0.099575711
HLA.A	-0.527609	0.150451	-3.506836	0.000453468	0.099575711
VSIR	-1.593327	0.455605	-3.497167	0.000470227	NA
GATD3	2.725647	0.783069	3.480721	0.000500065	NA
YARS	-0.822365	0.236412	-3.478522	0.000504188	0.099575711
ENSG00000273319	0.68188	0.196131	3.476652	0.000507716	0.099575711
THAP5	0.568686	0.166482	3.415895	0.000635727	0.112460356
SLC25A26	-0.578526	0.170352	-3.396068	0.000683615	0.112460356
SRSF5	-0.338015	0.099678	-3.391053	0.000696246	0.112460356
FTL	0.437397	0.129289	3.383085	0.000716765	0.112460356
ALDH2	-1.031022	0.30603	-3.369021	0.000754357	NA
S1PR4	0.631195	0.191337	3.298861	0.000970778	0.145061986
SGK1	-1.427222	0.436534	-3.269442	0.001077599	NA
RPS5	0.171608	0.053141	3.229312	0.001240885	0.176995282
TMEM170A	0.7315	0.228467	3.201776	0.00136583	NA
ATP6V1C2	-2.601158	0.819725	-3.173208	0.001507645	NA
CCDC191	-1.198102	0.378156	-3.168275	0.001533463	NA
EGLN2	0.428155	0.136386	3.139285	0.001693604	0.223077602
UBE2J1	0.533853	0.170173	3.137124	0.001706138	0.223077602
SMIM10	-3.105246	0.992002	-3.130281	0.00174639	NA
SPATA13	1.458187	0.470059	3.102137	0.001921288	NA
LRIF1	0.669042	0.215724	3.101373	0.001926257	NA
POLM	-0.485731	0.157549	-3.083037	0.002048995	0.241153095
ERGIC2	0.545944	0.177197	3.081	0.002063065	0.241153095
PTPN18	0.525551	0.170673	3.079292	0.002074931	0.241153095
SOX4	-1.160561	0.38091	-3.046815	0.002312798	NA
GALNT2	-0.902337	0.29659	-3.042372	0.002347219	0.258279367
IL21R	-0.991515	0.326304	-3.038622	0.002376632	NA
CLEC2B	0.454505	0.14964	3.037322	0.002386903	0.258279367
LTBP3	0.618855	0.205083	3.01758	0.002548016	0.266522484

ZNF384	-0.90725	0.302328	-3.000878	0.002692023	NA
SLC38A2	0.493172	0.164858	2.991489	0.002776203	0.27727229
ZMAT2	-0.392499	0.131451	-2.985894	0.002827506	0.27727229
BLZF1	0.962376	0.322434	2.984717	0.002838405	NA
TTC32	-0.900678	0.301862	-2.983738	0.002847509	NA
TNFRSF13B	1.188875	0.400008	2.972126	0.002957449	0.281226501
ATP5F1EP2	-0.611148	0.207654	-2.943114	0.003249289	0.288292273
TIMM10	-0.545754	0.185467	-2.942594	0.003254751	0.288292273
PNPLA8	0.539085	0.183511	2.937625	0.003307368	0.288292273
NUFIP2	0.618614	0.211229	2.928645	0.003404427	NA
RPS9	0.268888	0.09216	2.91762	0.003527141	0.291458824
HLA.C	-0.569329	0.195148	-2.917415	0.003529457	0.291458824
PSMB10	-0.376336	0.12979	-2.899568	0.003736774	0.300666567
FNBP1	-0.437726	0.1515	-2.88928	0.003861248	0.302914882
ENSG00000166927	-1.011916	0.351132	-2.881865	0.003953289	NA
ZNF213.AS1	-1.064327	0.369396	-2.881259	0.003960901	NA
UNC119	-0.521173	0.181803	-2.866691	0.004147879	0.317464465
ZBED2	1.360155	0.476039	2.857236	0.004273474	NA
TSC22D1	-1.089259	0.381678	-2.853868	0.004319047	NA
RPS15A	0.21629	0.076095	2.842354	0.004478168	0.329987664
ZFP36L2	0.633505	0.223871	2.829776	0.004658059	0.329987664
ENSG00000257275	-0.774008	0.275222	-2.812309	0.004918727	0.329987664
CIRBP	-0.279231	0.099329	-2.811177	0.004936062	0.329987664
LINC00662	0.539275	0.19185	2.810915	0.00494009	0.329987664
COL19A1	-0.704555	0.250692	-2.810444	0.004947324	0.329987664
TPP1	0.476629	0.169983	2.803979	0.005047612	0.329987664
HSH2D	-0.353241	0.126405	-2.794515	0.00519776	0.332868817
NSMAF	0.864556	0.310467	2.784693	0.00535784	NA
RNF130	0.729023	0.262284	2.779523	0.00544388	0.34165788
SLC7A7	-1.472032	0.532204	-2.765916	0.005676308	NA
DDX6	-0.409017	0.148651	-2.751526	0.005931836	0.364982375
ZNF267	-0.471753	0.171855	-2.745064	0.006049904	0.365088445
XRRA1	-0.847882	0.30968	-2.737927	0.006182773	0.36606681
UNC50	0.556374	0.203385	2.735569	0.006227258	NA
CHP1	-0.465002	0.170259	-2.731141	0.006311541	0.366770658
RPLP2	0.206919	0.076733	2.696614	0.007004847	0.399658381
SLC50A1	-0.606358	0.22551	-2.688826	0.00717037	0.401796824
IGHG1	1.300883	0.490485	2.652238	0.007996008	0.42838818
ATP5MGL	-0.977288	0.368573	-2.651545	0.008012438	NA
NOP10	0.387292	0.146104	2.650796	0.008030222	0.42838818
NDUFA7	-0.527851	0.199574	-2.644893	0.008171678	0.42838818
RBM3	-0.299262	0.11335	-2.640162	0.008286646	0.42838818
CLEC2D	-0.298164	0.113005	-2.638495	0.008327495	0.42838818
CLK1	0.488751	0.186085	2.626495	0.008626926	0.436633762
SFXN1	0.602102	0.229929	2.618643	0.008828026	NA
RUBCNL	-0.313043	0.119763	-2.61386	0.008952568	0.445923143
ITGB3BP	-0.518411	0.199336	-2.600686	0.009303757	0.455216371

CRIPT	0.526297	0.202727	2.596085	0.009429275	0.455216371
NAB2	1.108011	0.428529	2.585614	0.009720574	NA
RASGRP1	-1.129173	0.436989	-2.583982	0.0097667	NA
DMAC2L	-0.552195	0.214137	-2.5787	0.009917275	NA
ASAH1	0.465621	0.180623	2.577862	0.009941357	0.467786817
NUP88	-0.406019	0.158046	-2.568996	0.010199376	0.467786817
SYNJ2BP	0.452199	0.176202	2.566362	0.010277149	0.467786817
SKAP2	0.379343	0.14783	2.566065	0.010285943	0.467786817
MIR155HG	0.73704	0.287723	2.561631	0.010418206	NA
PPM1A	0.757275	0.295797	2.560116	0.010463711	NA
MRPL54	-0.31938	0.12502	-2.554639	0.010629787	0.469893581
ENSG00000245904	0.295859	0.115815	2.554575	0.010631754	0.469893581
GNPDA1	1.057631	0.415654	2.544498	0.010943481	NA
RPL38	0.121179	0.047641	2.543613	0.010971248	0.478163552
CHRAC1	-0.492513	0.194327	-2.534456	0.011262199	0.484120268
ENSG00000226571	1.431657	0.564893	2.534386	0.011264451	NA
ZCCHC10	0.368837	0.146648	2.51512	0.011899177	0.497621402
TOMM20	0.255903	0.101759	2.514795	0.011910159	0.497621402
IZUMO4	-0.630715	0.25137	-2.509109	0.012103602	0.497621402
MIF	-0.369093	0.147284	-2.505999	0.012210595	0.497621402
NRP2	-1.104423	0.440829	-2.50533	0.012233704	NA
TMEM99	0.804338	0.322419	2.494698	0.012606431	NA
GPM6B	0.589582	0.236571	2.492202	0.012695368	NA
EIF5	0.302731	0.121666	2.488223	0.012838317	0.516495366
CD5	-1.680969	0.676053	-2.486446	0.012902616	NA
ZNF138	0.605276	0.243672	2.483984	0.01299217	NA
FKBP5	0.816051	0.32929	2.478216	0.013204103	NA
FAM41C	-1.068826	0.431298	-2.478161	0.01320617	NA
RNF149	0.47673	0.19248	2.476778	0.013257446	NA
LY96	0.523387	0.211341	2.476509	0.013267445	0.526695745
UBE2J2	-0.35492	0.143563	-2.472224	0.013427552	0.526695745
CAPZB	-0.290764	0.118008	-2.463938	0.013741998	0.532375187
RIN3	0.62335	0.253603	2.457973	0.013972368	0.534698661
RND1	-1.217249	0.495818	-2.455032	0.014087212	NA
RPS28	0.112823	0.046269	2.438443	0.014750667	0.554477763
MGAT1	0.649552	0.266753	2.435035	0.014890355	0.554477763
DBI	-0.275258	0.113186	-2.431913	0.015019315	0.554477763
GLIDR	-1.07206	0.440917	-2.431432	0.015039286	NA
TELO2	-0.780202	0.321514	-2.42665	0.015238967	NA
ZBTB1	0.636054	0.263347	2.415275	0.015723333	0.573633941
TRIM14	1.04446	0.432959	2.412375	0.015848967	NA
GYPC	0.244222	0.101309	2.410669	0.015923291	0.573633941
MCRS1	-0.582877	0.242152	-2.40707	0.016081067	NA
BOD1	0.622027	0.25843	2.406945	0.016086611	0.573633941
KCTD18	-0.687714	0.286056	-2.404122	0.016211382	NA
FNIP1	-0.488311	0.203415	-2.400561	0.01636996	0.577179056
ENSG00000277654	-0.639811	0.266727	-2.398746	0.016451311	NA

PDE4D	-1.437387	0.59925	-2.398642	0.016455992	NA
IGHM	-0.462739	0.193859	-2.386984	0.016987208	0.589008319
MAPKAPK2	-0.486684	0.204057	-2.385034	0.017077551	NA
SESN3	0.367948	0.154278	2.384962	0.017080866	0.589008319
NFKBIB	-0.601624	0.252923	-2.378687	0.01737443	NA
UBB	-0.259926	0.109355	-2.376897	0.017458972	0.595391791
ENSG00000237491	-1.049311	0.441935	-2.374353	0.017579734	NA
DNAJA1	0.35214	0.148396	2.372975	0.017645455	0.595391791
APH1A	-0.276539	0.117055	-2.362475	0.018153369	0.606013541
TRMT61B	-0.668557	0.283415	-2.358937	0.018327358	NA
KIAA0040	0.556219	0.236294	2.353922	0.018576502	0.610470541
SLC35A4	-0.488628	0.207633	-2.353325	0.018606364	NA
FXYD5	0.316651	0.134634	2.351936	0.018675963	0.610470541
PLTP	-1.870831	0.797371	-2.34625	0.018963379	NA
SCAND1	-0.330193	0.141114	-2.339908	0.019288476	0.623992129
IL13RA1	-0.996821	0.426562	-2.33687	0.019445944	NA
CERS4	-0.413139	0.176959	-2.334653	0.019561564	0.624242488
SLC17A5	0.907611	0.388898	2.333798	0.01960628	NA
HCK	-1.128729	0.483904	-2.332549	0.019671836	NA
DUSP14	1.041223	0.446394	2.332522	0.019673258	NA
IGHD	-0.4356	0.186792	-2.332008	0.019700296	0.624242488
CTDSP2	0.382699	0.164364	2.32836	0.019893005	0.624242488
FXYD7	1.007824	0.433147	2.326747	0.019978715	NA
ENSG00000258377	-2.091176	0.899501	-2.324817	0.02008176	NA
ARL11	0.654361	0.282232	2.31852	0.020421094	0.633328028
RHBDD2	-0.599664	0.258932	-2.315911	0.020563122	NA
IQSEC1	-0.425062	0.183573	-2.315489	0.020586188	0.633328028
HTR3A	-1.22267	0.528102	-2.315214	0.020601196	NA
PIM1	-0.355945	0.154162	-2.308907	0.020948719	0.638224085
PDPK1	0.587186	0.254604	2.306273	0.021095373	NA
FBXL3	0.727554	0.316108	2.301599	0.021357807	0.644430758
RPS14	0.090191	0.039419	2.28801	0.022136922	0.657517493
HIST1H2BH	1.754652	0.767736	2.285489	0.022284151	NA
INKA1	0.636941	0.279259	2.280824	0.022558884	NA
RPL23A	0.152884	0.067062	2.279758	0.022622039	0.657517493
PTGIR	-1.256031	0.551964	-2.275567	0.022871959	NA
PRDM8	-0.685827	0.302545	-2.26686	0.023398781	NA
H3F3B	-0.197658	0.087278	-2.264697	0.023531298	0.657517493
PSMC3	-0.343157	0.151526	-2.264676	0.023532587	0.657517493
CCNG2	0.595164	0.262929	2.263592	0.023599221	NA
SLA	0.390024	0.172726	2.258043	0.023942953	0.657517493
MRPL38	-0.881614	0.390504	-2.257632	0.023968581	NA
YWHAE	0.34078	0.151022	2.25649	0.024039941	0.657517493
MYLIP	-0.703816	0.312333	-2.25342	0.024232695	NA
C7orf50	-0.502197	0.223136	-2.250633	0.024408789	0.657517493
TRMT112	0.257489	0.11442	2.250383	0.024424661	0.657517493
A4GALT	1.054016	0.468546	2.249547	0.024477693	NA

FCRL3	-1.056591	0.469706	-2.249472	0.024482472	0.657517493
ATP1B3	-0.495195	0.220176	-2.249091	0.024506676	0.657517493
AKIRIN2	0.374651	0.166624	2.248477	0.02454582	0.657517493
H2AFZ	0.325059	0.14465	2.24721	0.024626626	0.657517493
RPS3	0.140592	0.062602	2.245831	0.024714856	0.657517493
GSTO1	0.306363	0.136424	2.245672	0.024725004	0.657517493
GBGT1	0.866527	0.386301	2.243137	0.024888003	NA
HYI	-0.982748	0.438532	-2.240997	0.025026246	NA
UBE2L3	-0.331356	0.148028	-2.238479	0.025189867	0.662078259
ZNF224	0.366937	0.164067	2.236509	0.02531848	0.662078259
IRF5	0.494119	0.221047	2.235353	0.025394205	NA
PRICKLE1	0.797476	0.357057	2.233467	0.025518145	NA
IGLC7	-3.737968	1.67412	-2.232796	NA	NA
SRP72	-0.305074	0.136804	-2.23	0.02574744	0.666809765
RBM23	-0.244954	0.110088	-2.22507	0.026076534	0.666809765
CD1D	0.353763	0.159074	2.22389	0.026155875	0.666809765
U2AF1	-0.505831	0.227694	-2.221541	0.026314327	NA
IDE	-0.785005	0.353973	-2.2177	0.026575263	NA
LBH	-0.33423	0.150746	-2.217169	0.026611528	0.666809765
SCRN1	1.008914	0.455228	2.216281	0.026672285	NA
ENSG00000250850	0.908368	0.41014	2.214778	0.02677529	NA
HIST1H2AG	1.388216	0.628361	2.209264	0.027156295	NA
HLX	1.491108	0.675678	2.206832	0.027325771	NA
CYB561A3	-0.306831	0.139175	-2.204637	0.027479596	0.666809765
SVBP	-0.281622	0.127882	-2.202207	0.027650676	0.666809765
AKAP13	0.268659	0.122082	2.200655	0.02776045	0.666809765
FAM91A1	0.575982	0.262191	2.196804	0.028034418	NA
FLCN	0.512732	0.233412	2.196679	0.028043402	0.666809765
PFDN5	0.134653	0.061299	2.196647	0.028045695	0.666809765
SLC15A2	1.208311	0.550179	2.196216	0.028076506	NA
CD79B	-0.239211	0.109007	-2.194452	0.028202959	0.666809765
GSTP1	-0.282609	0.128905	-2.192378	0.028352192	0.666809765
CYTH1	-0.237575	0.108412	-2.191402	0.028422746	0.666809765
THG1L	0.485503	0.221637	2.190537	0.028485289	NA
RASGRP3	-0.517549	0.23646	-2.188734	0.028616163	0.666809765
SPN	-1.00512	0.459407	-2.187865	0.028679413	NA
RBM39	0.181451	0.082971	2.18693	0.02874766	0.666809765
ATG4B	-0.40115	0.183538	-2.185654	0.028840911	0.666809765
SRRT	-0.415245	0.190056	-2.184857	0.02889934	0.666809765
GOLIM4	-0.800833	0.367021	-2.181978	0.029111137	NA
NBPF3	-1.83386	0.841469	-2.179356	0.029305234	NA
CAPN12	0.561631	0.258242	2.174824	0.029643341	NA
GRHPR	-0.531943	0.245067	-2.170604	0.029961139	0.681518633
VPS26A	0.483213	0.222631	2.170471	0.029971183	0.681518633
BBX	0.317405	0.146727	2.163235	0.030523081	0.68496208
CD74	-0.261628	0.120969	-2.162766	0.030559175	0.68496208
CRK	0.603806	0.280116	2.155553	0.031118548	NA

IFI16	0.269488	0.125049	2.155061	0.031157092	0.692431746
RPS8	0.129299	0.060061	2.152808	0.03133375	0.692431746
SLC16A1.AS1	0.470025	0.218349	2.15263	0.031347749	NA
PSMD9	-0.35387	0.164857	-2.146519	0.031831558	0.698513479
SEL1L3	0.489558	0.228431	2.143134	0.032102302	0.698983806
RNF43	-0.894971	0.418165	-2.140232	0.032336057	NA
ZMYND8	0.464683	0.217397	2.137488	0.032558311	NA
LYST	-0.560405	0.262617	-2.133927	0.032848776	0.698983806
RPL30	0.091023	0.042655	2.133915	0.032849702	0.698983806
ZDHHC4	0.37938	0.177907	2.132463	0.032968793	0.698983806
PLGRKT	-0.575713	0.270106	-2.131431	0.033053622	0.698983806
KDM6B	0.501702	0.235467	2.130664	0.033116805	NA
DNAH1	-1.07023	0.502309	-2.130623	0.033120196	NA
CMTM7	-0.355945	0.167258	-2.128114	0.033327602	0.698983806
LINC00926	-0.360153	0.169317	-2.127095	0.033412228	0.698983806
LY9	0.823538	0.38766	2.124383	0.033638117	0.699049087
COPS2	0.508754	0.239593	2.123406	0.033719822	NA
ATP6V1C1	0.538654	0.253728	2.122956	0.033757554	NA
USP31	-1.315533	0.619971	-2.121926	0.033843959	NA
ENSG00000233184	0.724912	0.341725	2.121329	0.033894142	NA
ATP6V0E2	-0.727399	0.343179	-2.119589	0.03404073	NA
ZBTB20	0.421772	0.199106	2.11833	0.034147139	0.704958699
WDR76	-0.702221	0.331709	-2.116977	0.034261838	NA
HSBP1	0.348161	0.16466	2.114427	0.034478825	0.705977608
DGLUCY	0.459021	0.217131	2.114024	0.034513219	NA
CCT8	0.50686	0.239937	2.112467	0.034646447	0.705977608
TM7SF2	-0.718787	0.34039	-2.111659	0.034715689	NA
SYPL1	0.307146	0.145706	2.107992	0.035031668	0.70853716
A1BG.AS1	-0.779226	0.369975	-2.10616	0.035190421	NA
CD82	-0.281067	0.13348	-2.105691	0.03523122	0.70853716
RCHY1	0.532909	0.253371	2.103271	0.035442104	NA
GPR137	-0.394081	0.187373	-2.103187	0.035449437	0.70853716
ZBED4	0.964253	0.459219	2.099769	0.035749138	NA
KLHL24	0.401475	0.19137	2.0979	0.035914002	0.713279364
KCNK6	0.647335	0.309418	2.092103	0.03642926	NA
SCAI	0.433206	0.207132	2.091451	0.036487654	NA
AKAP17A	0.417894	0.199831	2.091239	0.036506633	0.714258789
BHLHE40	-0.340186	0.162738	-2.090391	0.036582658	0.714258789
JADE1	0.429627	0.205594	2.089684	0.036646165	0.714258789
EXOC3.AS1	-0.733095	0.351173	-2.087561	0.036837486	NA
GKAP1	0.542437	0.259904	2.087067	0.036882099	NA
STK17A	0.271012	0.130178	2.081853	0.037355925	0.723598096
ENSG00000279483	-0.321927	0.154885	-2.078482	0.037664943	0.724331578
NIFK	0.398885	0.191947	2.078097	0.037700446	NA
PTPMT1	-0.39142	0.188356	-2.078081	0.037701893	NA
TMED10	-0.347678	0.167445	-2.076369	0.037859879	0.724331578
ODF3B	-0.593033	0.285878	-2.074425	0.038039827	NA

XBP1	0.612489	0.295352	2.073762	0.038101391	NA
ATAD2B	-0.718112	0.346591	-2.071932	0.03827175	NA
C9orf40	1.266525	0.611473	2.071269	0.038333655	NA
KRCC1	0.374971	0.181345	2.067725	0.038665915	0.724331578
TMEM70	0.380905	0.184318	2.066565	0.038775143	0.724331578
RPL32	0.104419	0.050537	2.066198	0.038809787	0.724331578
SNAPC4	-0.950698	0.461235	-2.061203	0.039283663	NA
UBE2N	0.248752	0.120712	2.060714	0.039330347	0.724331578
TMEM179B	-0.263375	0.127834	-2.060298	0.039370057	0.724331578
TRAF1	-0.812658	0.394496	-2.059991	0.039399369	NA
EIF4H	-0.237889	0.115493	-2.059769	0.03942061	0.724331578
ARIH2	0.391899	0.190457	2.057677	0.03962116	NA
MSANTD2	0.994508	0.483476	2.056997	0.039686557	NA
RPS16	0.122027	0.059374	2.055239	0.039855947	0.724331578
HINT2	-0.290594	0.141521	-2.053359	0.040037786	0.724331578
PKM	-0.28655	0.139625	-2.052281	0.040142399	0.724331578
ATG16L2	-0.428955	0.20903	-2.052124	0.040157659	NA
LDHA	-0.383699	0.186982	-2.052061	0.040163701	0.724331578
C2orf69	0.504298	0.245864	2.051128	0.040254441	NA
FOXO3	0.563727	0.274849	2.051043	0.040262726	NA
NDUFAF7	-0.569285	0.277944	-2.0482	0.040540368	NA
MSL2	0.529244	0.258426	2.047953	0.040564635	NA
MESD	0.358592	0.175158	2.047248	0.040633708	NA
GM2A	0.596525	0.291818	2.044165	0.040937212	0.734062701
TSPYL1	-0.319823	0.156779	-2.039957	0.04135462	0.734741573
CLEC4A	-1.280321	0.628309	-2.037725	0.04157748	NA
RPL11	0.096874	0.047564	2.036726	0.041677501	0.734741573
LSR	0.851386	0.418164	2.036008	0.041749599	NA
ENSG00000241490	0.735201	0.361432	2.034132	0.04193833	NA
NUP210	0.371336	0.18256	2.034047	0.04194682	NA
MARCHF5	0.53725	0.264358	2.032279	0.042125404	NA
C17orf49	-0.493849	0.243047	-2.031912	0.042162551	NA
LYRM7	0.327975	0.161412	2.031911	0.04216266	0.734741573
HMGB2	-0.287986	0.141806	-2.030843	0.042270934	0.734741573
EIF3J.DT	0.322452	0.158914	2.029091	0.042449009	0.734741573
RGS10	0.525015	0.258774	2.028854	0.042473145	0.734741573
CBWD3	-0.532876	0.262812	-2.02759	0.042602075	NA
SAP18	-0.179306	0.088483	-2.02644	0.042719666	0.734741573
CLMN	0.77198	0.381218	2.025034	0.042863893	NA
SEC13	-0.425312	0.210056	-2.024758	0.042892263	NA
AMD1	0.357958	0.176883	2.023691	0.043001953	0.734741573
LIMD2	-0.20878	0.103208	-2.022911	0.043082361	0.734741573
DYNLT1	0.361132	0.178802	2.019724	0.043412011	NA
RPL24	0.098783	0.048944	2.018303	0.043559756	0.73766782
PCM1	0.367383	0.182168	2.016726	0.043724096	0.73766782
ENSG00000170161	-0.582861	0.289239	-2.015152	0.043888704	NA
ZNF12	0.67141	0.333183	2.015136	0.043890379	NA

DYNLL1	0.267539	0.133164	2.009091	0.044527454	0.747204011
MGME1	0.425643	0.212231	2.005569	0.044902285	NA
FLAD1	-0.579028	0.288858	-2.00454	0.045012237	NA
NR6A1	-1.515757	0.756448	-2.003783	0.045093298	NA
PPFIBP1	-0.908897	0.453633	-2.003595	0.045113512	NA
NELFE	-0.266902	0.133322	-2.001933	0.045291981	0.748357928
KCTD5	0.573497	0.286638	2.00077	0.045417199	NA
IQCH.AS1	0.823908	0.411829	2.000605	0.045434984	NA
PRAG1	1.086219	0.543807	1.997435	0.045777983	NA
ZFPL1	-0.477726	0.239302	-1.99633	0.045898048	NA
PCNX4	0.355185	0.178018	1.995216	0.046019357	0.748357928
LNPEP	0.307306	0.154099	1.994214	0.046128687	0.748357928
TMPO.AS1	-1.348536	0.676641	-1.992984	0.046263146	NA
UBE2A	-0.28647	0.143789	-1.992292	0.046339029	0.748357928
RGS19	0.349943	0.175747	1.991177	0.046461394	0.748357928
RPS18	0.17632	0.088567	1.990814	0.046501301	0.748357928
JAK3	-0.48513	0.24383	-1.989624	0.046632361	0.748357928
GNB5	0.287794	0.144663	1.989404	0.046656613	0.748357928
THEM4	-0.761094	0.382941	-1.987497	0.04686735	NA
SAMD10	-1.004783	0.506011	-1.985693	0.047067422	NA
MYO1G	0.340083	0.171322	1.985048	0.047139186	0.748357928
GPS2	-0.396858	0.199946	-1.98483	0.047163381	0.748357928
STK16	-0.331904	0.16756	-1.980812	0.047612306	0.748357928
PRCP	-0.425816	0.215063	-1.979956	0.047708528	NA
ZNF775	-1.231133	0.62186	-1.97976	0.047730509	NA
CD48	0.248769	0.125692	1.979192	0.047794424	0.748357928
PTPN2	0.286286	0.144693	1.97858	0.047863262	0.748357928
H1FX	-0.870562	0.440459	-1.976489	0.048099418	NA
RPS27A	0.104657	0.052961	1.976121	0.048141022	0.748357928
TMEM156	0.462051	0.233992	1.974643	0.048308711	0.748357928
TMEM251	0.507835	0.257214	1.974366	0.048340107	NA
EXOSC8	0.329322	0.166852	1.973735	0.048411937	0.748357928
MOV10	0.834246	0.422751	1.973376	0.048452762	NA
ERAP1	0.523951	0.265538	1.973163	0.048476979	NA
ENSG00000280007	-1.04824	0.531819	-1.971048	0.048718379	NA
PTP4A3	1.212435	0.615729	1.969106	0.048940952	NA
AK6	0.325493	0.165349	1.968523	0.049007916	0.753857068
S100A13	-0.887426	0.451977	-1.96343	0.049596211	NA
FCGR2B	0.735124	0.374712	1.961836	0.049781586	NA
NTHL1	-0.505914	0.257899	-1.961676	0.049800174	NA

Cluster 8	log2FC	lfcSE	stat	pvalue	padj
ARRDC3	-1.217494	0.178462	-6.822133	8.97E-12	3.94E-08
GPR183	0.795786	0.127664	6.233426	4.56E-10	1.00E-06
CKS2	1.291314	0.212905	6.0652	1.32E-09	1.93E-06
RPL8	0.404548	0.068476	5.907912	3.46E-09	3.80E-06
RPS5	0.359067	0.064754	5.54507	2.94E-08	2.16E-05
RPL19	0.184563	0.033289	5.544213	2.95E-08	2.16E-05
NPM1	0.262409	0.048778	5.379614	7.46E-08	4.68E-05
IL4R	-0.645521	0.12056	-5.354341	8.59E-08	4.71E-05
RENBP	1.070374	0.206719	5.17792	2.24E-07	0.000109444
NOP10	0.39864	0.077639	5.134518	2.83E-07	0.000124179
ENSG00000271204	0.588149	0.116237	5.059895	4.19E-07	0.000167413
ENSG00000272211	-1.425394	0.283833	-5.021947	5.12E-07	0.000187125
RPS9	0.393657	0.08104	4.85753	1.19E-06	0.000401378
ZNF107	1.251603	0.259418	4.824653	1.40E-06	0.000439775
UQCRH	0.274445	0.057058	4.809932	1.51E-06	0.000441872
CD48	0.417354	0.089388	4.669027	3.03E-06	0.00083034
PHF21A	-0.634812	0.138881	-4.570911	4.86E-06	0.001254015
GATM	2.07213	0.455871	4.545434	5.48E-06	NA
CD79B	-0.384521	0.085029	-4.52221	6.12E-06	0.001492535
RPS23	0.212636	0.047223	4.502789	6.71E-06	0.001528853
RPL24	0.258941	0.05761	4.49475	6.97E-06	0.001528853
RPL9	0.238295	0.053381	4.464038	8.04E-06	0.001681356
BLVRB	0.986088	0.221522	4.451422	8.53E-06	0.00170219
RPL30	0.200286	0.045132	4.43775	9.09E-06	0.001735081
NFKBIA	-0.460984	0.105277	-4.378765	1.19E-05	0.002183183
ENSG00000259888	2.25305	0.526238	4.281426	1.86E-05	NA
HNRNPDL	-0.305029	0.071915	-4.241538	2.22E-05	0.003898211
CIRBP	-0.322846	0.076738	-4.207118	2.59E-05	0.004143345
RPS15	0.154384	0.036701	4.20652	2.59E-05	0.004143345
RPL34	0.185431	0.044196	4.195705	2.72E-05	0.004143345
TIMM10	-0.623214	0.148586	-4.194308	2.74E-05	0.004143345
ZBED2	2.386924	0.573599	4.161311	3.16E-05	NA
HRK	-1.99613	0.48311	-4.131832	3.60E-05	0.005266291
FAM102A	0.995495	0.244181	4.076869	4.56E-05	0.006464078
C16orf74	-0.706452	0.174051	-4.058885	4.93E-05	0.006764375
DEF8	-0.710954	0.175525	-4.050451	5.11E-05	0.006800381
RPL38	0.232586	0.057815	4.022916	5.75E-05	0.007421941
BHLHE41	2.403203	0.599118	4.011237	6.04E-05	NA
GLUL	-0.932084	0.233066	-3.999229	6.35E-05	0.007775757
RAB9A	0.840757	0.210272	3.998427	6.38E-05	0.007775757
COX17	0.386403	0.097603	3.958947	7.53E-05	0.008750432
RNF130	0.771157	0.194861	3.957482	7.57E-05	0.008750432
RPL36	0.161432	0.040922	3.944843	7.99E-05	0.00897768
EEF1B2	0.350161	0.088968	3.935797	8.29E-05	0.00897768
DYNLL1	0.585873	0.148958	3.933132	8.38E-05	0.00897768
RPS18	0.232238	0.05933	3.91433	9.07E-05	0.009223867

TMEM204	-2.749617	0.702557	-3.913731	9.09E-05	NA
RPL32	0.16719	0.042732	3.912527	9.13E-05	0.009223867
RPL26	0.195267	0.049946	3.909601	9.24E-05	0.009223867
LPIN1	0.766975	0.199106	3.8521	0.00011711	0.011424634
MIF	-0.398733	0.103734	-3.843795	0.00012115	0.011561584
CYTIP	0.246049	0.064642	3.806321	0.00014105	0.013174581
RACK1	0.199933	0.052678	3.795376	0.00014742	0.013482777
RPS16	0.261779	0.069298	3.77758	0.00015836	0.014187756
ZNF385A	-1.756664	0.46519	-3.77623	0.00015922	NA
RPL37	0.158753	0.04252	3.733619	0.00018875	0.01657204
CDC42	-0.299827	0.081159	-3.694338	0.00022046	0.018630438
GSTO1	0.524447	0.141969	3.694086	0.00022068	0.018630438
MHENCR	-0.445944	0.121253	-3.677786	0.00023527	0.01948719
HSPE1	0.327608	0.089297	3.668766	0.00024372	0.019813871
ARL17A	-0.827737	0.226323	-3.65733	0.00025486	NA
COMMD6	0.192184	0.052588	3.654518	0.00025767	0.0205664
RPSA	0.32729	0.089872	3.641736	0.00027081	0.02122921
RHBDD2	-0.76753	0.212399	-3.613624	0.00030195	NA
RPL13	0.212496	0.058924	3.606294	0.0003106	0.023921725
ATP6V1C1	0.914417	0.253945	3.600845	0.00031719	NA
KDM4B	0.887889	0.24667	3.599501	0.00031883	0.024131978
SLC50A1	-0.391264	0.109143	-3.584883	0.00033723	0.025092155
GATD3	4.009907	1.119135	3.583042	0.00033962	NA
DDX6	-0.447103	0.125117	-3.573476	0.00035227	0.025716228
IGHD	-0.482851	0.135262	-3.569742	0.00035733	0.025716228
H3F3B	-0.326028	0.091991	-3.544112	0.00039394	0.02768146
RPL23A	0.202605	0.057202	3.541903	0.00039725	0.02768146
NSMAF	1.222799	0.346017	3.533932	0.00040943	0.027990041
RPS12	0.249997	0.070807	3.530669	0.00041451	0.027990041
RPS3	0.223737	0.063441	3.526679	0.00042081	0.027990041
CLEC2B	0.391386	0.111302	3.516414	0.00043742	0.028660695
HVCN1	-0.303504	0.086506	-3.508481	0.00045067	0.029094991
IGHG1	1.38147	0.396309	3.485838	0.0004906	NA
ENSG00000273319	0.609357	0.175188	3.478297	0.00050461	0.031370701
RPS15A	0.210148	0.060423	3.477931	0.0005053	0.031370701
CHORDC1	0.731069	0.210268	3.476839	0.00050736	0.031370701
DDX58	-0.817431	0.23527	-3.474433	0.00051193	NA
RPS13	0.166302	0.048221	3.44873	0.00056323	0.034341307
TNFRSF13B	1.086378	0.31639	3.433667	0.00059548	0.035810116
KANK2	-2.061716	0.60185	-3.425633	0.00061337	NA
RPL13A	0.186535	0.054478	3.424043	0.00061697	0.03660121
ANP32B	0.255031	0.074645	3.416593	0.0006341	0.037115961
SRSF6	-0.427	0.125787	-3.394631	0.00068721	0.03969545
STX7	0.271701	0.080448	3.377325	0.00073195	0.041584408
SLC5A3	-0.654081	0.19396	-3.372255	0.00074556	0.041584408
MT.CO1	0.246372	0.073142	3.368392	0.00075608	0.041584408
RPL7A	0.24526	0.072826	3.367765	0.0007578	0.041584408

EYA3	1.064287	0.316477	3.362923	0.00077122	NA
FAM177B	0.864073	0.257921	3.350138	0.00080771	0.043409957
ZNF580	-0.448836	0.134052	-3.348216	0.00081334	0.043409957
ENSG00000282988	1.03827	0.310122	3.347944	0.00081413	NA
ERP29	0.209545	0.062631	3.345706	0.00082074	0.043409957
TULP4	-0.478669	0.144348	-3.316087	0.00091287	0.047708478
BCL7A	-0.758484	0.228985	-3.312381	0.00092506	0.047776398
ADPRH	1.80564	0.545422	3.31054	0.00093116	NA
IGHG3	1.795687	0.544381	3.298585	0.00097174	NA
RPS6	0.213225	0.064681	3.296589	0.00097867	0.049254035
ATP5IF1	-0.250812	0.076091	-3.296203	0.00098001	0.049254035
RPL35	0.166079	0.050504	3.288404	0.00100757	0.049254035
MGAT1	0.516778	0.157222	3.286935	0.00101284	0.049254035
RPL3	0.1851	0.056326	3.286259	0.00101528	0.049254035
RPS8	0.266669	0.081186	3.28468	0.00102098	0.049254035
LNPEP	0.483267	0.147617	3.273787	0.00106117	0.050636029
RPL11	0.148564	0.045437	3.269665	0.00107675	0.050827271
IFI44L	1.927422	0.590733	3.262762	0.00110332	NA
MINDY2	0.618274	0.1897	3.259221	0.00111719	0.052174963
ATP6V0E2	-1.165972	0.358835	-3.249325	0.00115679	0.053071878
TOMM7	0.28808	0.088684	3.248398	0.00116057	0.053071878
NT5DC4	2.493167	0.769642	3.239385	0.00119788	NA
RPL10	0.216421	0.066888	3.23558	0.00121396	0.054819577
HEXA	0.481334	0.148869	3.233283	0.00122376	0.054819577
RPS3A	0.15488	0.047989	3.227412	0.00124915	0.054866855
HHEX	0.366127	0.113448	3.227261	0.00124981	0.054866855
CREBL2	0.857545	0.266345	3.219676	0.00128336	NA
RPL37A	0.19417	0.060577	3.205318	0.00134913	0.058597288
NCF1	-0.271932	0.084937	-3.201585	0.00136674	0.058597288
UCP2	-0.472651	0.147709	-3.199883	0.00137483	0.058597288
NDUFB11	0.305684	0.095813	3.190407	0.00142072	0.059592037
RPL7	0.19423	0.060912	3.188716	0.00142906	0.059592037
TMEM140	0.645271	0.202487	3.186733	0.0014389	0.059592037
HSD17B4	0.708932	0.222796	3.18198	0.00146272	0.060012565
H2AFZ	0.230241	0.072522	3.174781	0.0014995	0.060951735
KRTCAP2	-0.291761	0.092105	-3.16771	0.00153645	0.061880803
ENSG00000225885	-1.094481	0.34566	-3.166354	0.00154363	NA
TAGAP	-0.589948	0.187219	-3.151111	0.00162651	0.064912434
CLK1	0.458962	0.1458	3.147896	0.0016445	0.065039323
NAP1L1	0.232863	0.074402	3.129787	0.00174933	0.068567591
THRAP3	-0.322731	0.103254	-3.125599	0.00177443	0.068935845
PDE4DIP	-0.780491	0.250517	-3.115519	0.00183622	NA
MAML3	-1.554957	0.501649	-3.099689	0.00193724	NA
CERS4	-0.461364	0.148962	-3.097186	0.00195367	0.074956823
SNHG30	-0.518163	0.167382	-3.095689	0.00196356	0.074956823
SLC2A1	-0.557297	0.180877	-3.081085	0.00206248	0.078054236
TCL1B	-1.154096	0.374997	-3.077618	0.00208662	NA

FAM117B	-0.771052	0.250642	-3.076303	0.00209585	0.078113118
FAU	0.108294	0.035209	3.075766	0.00209962	0.078113118
DDX5	-0.37573	0.122465	-3.068061	0.00215452	0.079046774
RPL21	0.170037	0.055437	3.067201	0.00216073	0.079046774
RPL22	0.151871	0.049592	3.06238	0.00219585	0.079667471
LINC00847	0.644298	0.210917	3.054747	0.00225251	0.081053349
LAMC1	1.415897	0.463668	3.053688	0.00226047	NA
DNAJA1	0.450464	0.148032	3.043022	0.00234215	0.083593939
EIF3K	0.255737	0.08477	3.016825	0.00255437	0.08838612
HMGB2	-0.428466	0.14208	-3.015662	0.00256419	0.08838612
CFLAR	0.390029	0.129349	3.015325	0.00256704	0.08838612
ITM2C	0.52249	0.173295	3.015026	0.00256957	0.08838612
SLC16A7	0.588206	0.195149	3.01414	0.00257709	0.08838612
C15orf61	0.6099	0.202569	3.010828	0.00260537	0.088448968
THUMPD3.AS1	-0.285959	0.095028	-3.009217	0.00261922	0.088448968
ZNF318	0.5283	0.176139	2.999342	0.00270564	0.090669754
USF1	-1.014657	0.338345	-2.998886	0.00270969	NA
ENSG00000273448	-0.798308	0.266559	-2.994866	0.00274566	NA
SLC38A2	0.409797	0.13726	2.985558	0.00283061	0.092949339
MRPS36	0.450947	0.151134	2.983754	0.00284736	0.092949339
ST13	0.194851	0.065321	2.982995	0.00285443	0.092949339
RPL18	0.16029	0.053777	2.980645	0.00287642	0.092949339
RNF146	0.595573	0.199835	2.980315	0.00287952	0.092949339
PCNX4	0.488015	0.164161	2.972785	0.00295111	0.093243854
THOC3	-0.61551	0.207049	-2.972776	0.0029512	0.093243854
CRK	0.619961	0.208555	2.972654	0.00295237	0.093243854
APBB2	-0.927301	0.312349	-2.968801	0.00298965	NA
ENSG00000254397	1.284079	0.433249	2.963836	0.0030383	NA
RPS28	0.220297	0.074359	2.962602	0.00305051	0.095655295
L3HYPDH	-0.8042	0.271691	-2.959984	0.00307655	NA
PET100	-0.292324	0.099029	-2.951893	0.00315832	0.095989574
HSH2D	-0.460622	0.156103	-2.950767	0.00316986	0.095989574
TRA2B	-0.272252	0.092323	-2.948894	0.00318914	0.095989574
TTC32	-0.974175	0.330421	-2.948281	0.00319546	0.095989574
UBE2J2	-0.47339	0.160605	-2.947536	0.00320317	0.095989574
UBA52	0.113328	0.038449	2.947507	0.00320348	0.095989574
TCTN1	-0.799977	0.271503	-2.946471	0.00321423	0.095989574
CREBRF	0.363545	0.123567	2.942083	0.00326012	0.096702219
RPLP2	0.21293	0.072486	2.937526	0.00330843	0.097476517
NAB2	1.188394	0.405107	2.933532	0.00335129	NA
SNHG9	-0.402351	0.13729	-2.930659	0.00338244	0.097726963
RPL31	0.170144	0.058059	2.930531	0.00338383	0.097726963
IL2RG	-0.473417	0.16172	-2.927376	0.00341835	0.097726963
TBCA	0.261583	0.08937	2.926983	0.00342268	0.097726963
TMED2	-0.349307	0.119361	-2.926478	0.00342824	0.097726963
FBXO6	-1.11263	0.380435	-2.924628	0.00344868	NA
RPL5	0.213283	0.072967	2.922999	0.00346678	0.098188071

HLA.A	-0.448438	0.15394	-2.913073	0.00357891	0.099666718
ZCCHC7	-0.399721	0.137289	-2.911522	0.00359673	0.099666718
RPL10A	0.303686	0.104379	2.909454	0.00362061	0.099666718
FLCN	0.534245	0.183707	2.908137	0.00363589	0.099666718
ERGIC2	0.427973	0.147209	2.907241	0.00364632	0.099666718
NCBP2AS2	-0.528589	0.181977	-2.904704	0.00367601	0.099666718
ZFAS1	0.235	0.080908	2.904542	0.00367791	0.099666718
RPL29	0.195076	0.067213	2.902366	0.00370356	0.099746089
RPL28	0.200354	0.069233	2.893898	0.00380492	0.101851258
CPM	-0.98715	0.342269	-2.884133	0.00392493	NA
FXYD7	0.788078	0.273311	2.883453	0.00393341	0.104652547
CLECL1	1.122528	0.389918	2.878883	0.00399087	NA
MX1	0.940284	0.327446	2.871569	0.0040844	NA
LRWD1	0.670971	0.233741	2.870575	0.00409726	NA
CYB5A	-0.548135	0.190966	-2.870327	0.00410048	0.108374098
ENSG00000007237	-1.526777	0.532067	-2.869519	0.00411096	NA
NAA10	-0.479825	0.167267	-2.868621	0.00412266	0.108374098
CCDC25	0.449436	0.156808	2.866161	0.00415482	0.10856951
MIR29B2CHG	0.623473	0.21779	2.862731	0.00420007	0.109102435
SLA	0.635674	0.222539	2.856462	0.00428392	0.110625846
SNAP23	-0.328977	0.115454	-2.849428	0.00437979	0.112440123
FKBP5	1.043427	0.366638	2.845933	0.00442816	NA
CCNG2	0.638149	0.224242	2.845804	0.00442995	0.113066714
C17orf58	1.951667	0.686577	2.842604	0.00447467	NA
LINC00926	-0.351679	0.123933	-2.837659	0.00454457	0.115321805
C7orf50	-0.412248	0.14543	-2.834681	0.00458715	0.115733162
NUDT14	0.578658	0.204755	2.826096	0.00471191	0.118201596
CCDC144A	2.017015	0.715657	2.818412	0.00482618	NA
SMIM20	0.434261	0.154236	2.815557	0.00486927	0.119148619
DENND2D	0.560654	0.199188	2.814693	0.00488238	0.119148619
SKAP2	0.40548	0.144069	2.814479	0.00488564	0.119148619
CAPG	0.376022	0.133609	2.814347	0.00488765	0.119148619
IGHA1	-0.440108	0.156493	-2.812312	0.00491868	0.119148619
PDE7B	-0.888183	0.315847	-2.812071	0.00492237	NA
YWHAE	0.378263	0.134556	2.811185	0.00493594	0.119148619
RPL35A	0.182555	0.064944	2.810943	0.00493965	0.119148619
RPS27A	0.117945	0.04205	2.804847	0.00503405	0.120762097
NOTCH2	0.48643	0.17366	2.801055	0.00509358	0.121526177
CD22	0.565926	0.202896	2.789244	0.00528312	0.125217652
RPS20	0.294541	0.10565	2.787884	0.00530535	0.125217652
ZNF606	-0.631708	0.227066	-2.782042	0.00540181	0.126812611
SAR1A	-0.414871	0.149576	-2.773643	0.00554324	0.129440628
ZBTB16	1.896645	0.684417	2.771183	0.0055853	NA
KLK4	2.097178	0.757052	2.770191	0.00560234	NA
MCL1	-0.387814	0.140408	-2.762051	0.00574396	0.133417822
CTNNAL1	-1.309362	0.47423	-2.761026	0.005762	NA
RPL15	0.125514	0.045484	2.759512	0.00578877	0.133751077

EIF3E	0.205428	0.074616	2.753137	0.00590272	0.135669821
AK6	0.488757	0.177665	2.751009	0.0059412	0.135776419
ST3GAL5	0.542655	0.197367	2.749468	0.00596921	0.135776419
RBM26.AS1	-1.062394	0.386637	-2.747784	0.00599995	NA
HLA.C	-0.489031	0.178099	-2.74584	0.00603561	0.136264219
CDKN1A	-0.625944	0.228038	-2.744911	0.00605274	0.136264219
ARIH2OS	-0.997916	0.36407	-2.740998	0.00612529	NA
CBWD2	-0.850094	0.311903	-2.725508	0.00642027	NA
CD83	-0.293883	0.107879	-2.724194	0.00644586	0.144374
BATF	1.361252	0.49975	2.723867	0.00645226	NA
EPB42	-0.825033	0.303266	-2.720496	0.00651841	NA
RSRP1	0.34076	0.125352	2.718437	0.00655911	0.145453486
LBH	-0.344096	0.126581	-2.718376	0.00656032	0.145453486
ENSG00000167414	-1.570447	0.577844	-2.717773	0.0065723	NA
KMT2A	0.411723	0.151575	2.716302	0.00660157	0.145632644
SENP2	-0.677381	0.249493	-2.715025	0.00662708	NA
RPL27A	0.143485	0.052942	2.710238	0.0067235	0.147580761
FAM32A	-0.331268	0.122434	-2.70568	0.00681647	0.148877059
IFI27L2	0.328382	0.121505	2.702621	0.00687951	0.149510138
CDC42SE1	-0.384156	0.142248	-2.700616	0.00692112	0.149673539
CLEC2D	-0.323012	0.119843	-2.695293	0.00703267	0.150842777
FAM184B	-1.705132	0.632758	-2.694761	0.00704391	0.150842777
SDSL	1.619495	0.601376	2.692983	0.00708159	NA
NDUFA13	-0.266138	0.099167	-2.683734	0.0072805	0.154762438
SCAND1	-0.32201	0.120021	-2.682956	0.00729745	0.154762438
WDR11	-0.653822	0.244423	-2.674959	0.00747383	0.157740984
DOK3	0.966309	0.361262	2.674814	0.00747707	NA
LGALS1	1.509988	0.564889	2.67307	0.00751605	0.157873067
NPHP3	0.580063	0.217557	2.666262	0.00767	NA
RPS14	0.220589	0.082841	2.6628	0.00774935	0.1612456
NMT2	-0.403234	0.151461	-2.662289	0.00776112	0.1612456
PRKCB	-0.51205	0.192438	-2.660862	0.00779409	0.1612456
RPS27	0.131641	0.049503	2.659272	0.00783098	0.1612456
TAGLN2	-0.291718	0.10975	-2.658014	0.00786026	0.1612456
LARP1	0.450068	0.169612	2.653514	0.00796585	0.161568993
SRSF5	-0.280454	0.105692	-2.653513	0.00796588	0.161568993
CNN3	-0.764477	0.288195	-2.652643	0.00798644	0.161568993
TTC37	0.519351	0.196378	2.644648	0.00817759	0.164058004
LYRM7	0.377395	0.142716	2.644374	0.00818422	0.164058004
GCSH	-0.592392	0.224302	-2.641051	0.00826493	NA
KDSR	0.567778	0.21532	2.636903	0.00836667	0.166953072
APLP2	-0.486868	0.184777	-2.634892	0.00841642	0.167185854
ADAMTS6	0.909157	0.345764	2.629415	0.0085532	NA
RDH5	0.489907	0.18637	2.628682	0.00857165	0.169502502
IZUMO4	-0.665777	0.253458	-2.626775	0.00861983	0.169690741
UGT8	1.185548	0.451563	2.625431	0.00865394	NA
LMO4	0.535233	0.204148	2.621786	0.00874704	NA

MME	-2.927514	1.117051	-2.620753	0.00877359	NA
CBLN3	-0.895808	0.341863	-2.620375	0.00878332	NA
NEIL1	-0.652103	0.249121	-2.617619	0.00885457	0.173533753
RHOF	0.377704	0.144594	2.61217	0.00899696	0.175540709
NREP	-0.776085	0.297286	-2.610569	0.00903916	NA
LSM2	0.265121	0.102139	2.59569	0.00944012	0.183372272
WASF1	-1.154465	0.444884	-2.594981	0.0094596	NA
HIP1R	-0.493956	0.190455	-2.593557	0.00949888	0.183700872
TMEM191B	-0.762152	0.29419	-2.590675	0.0095788	NA
RPL23	0.13102	0.050612	2.588713	0.00963353	0.18548766
ENSG00000251364	1.27111	0.491199	2.587768	0.00966001	NA
TMEM205	-0.529083	0.204727	-2.584343	0.00975648	0.187034608
ENSG00000187186	1.408872	0.545715	2.581701	0.00983147	NA
SWAP70	0.367357	0.14237	2.5803	0.00987146	0.187705356
VDAC2	0.247381	0.095934	2.578656	0.00991854	0.187705356
SESN3	0.380322	0.147491	2.578615	0.00991974	0.187705356
HCST	1.012116	0.392653	2.577632	0.009948	NA
CCT3	0.276578	0.107401	2.575198	0.01001828	0.188350167
ENSG00000279483	-0.256777	0.09974	-2.574462	0.01003962	0.188350167
IL23A	-0.92928	0.361176	-2.572927	0.01008425	NA
ATP9B	-0.684426	0.26608	-2.572255	0.01010386	NA
PSMA4	-0.255906	0.099537	-2.570952	0.01014195	0.189460247
TMEM14A	0.611083	0.238183	2.565604	0.01029962	0.191064685
CXCR4	0.144937	0.056504	2.565091	0.01031488	0.191064685
RGS10	0.907023	0.354312	2.559953	0.01046863	0.192311524
RPLP1	0.219036	0.085565	2.559885	0.01047068	0.192311524
RNASEK	-0.554511	0.216678	-2.559146	0.01049298	NA
LIMD1	-0.917506	0.35885	-2.556798	0.01056405	0.192311524
EIF4B	0.192	0.07513	2.555563	0.01060161	0.192311524
C9orf16	-0.276647	0.108311	-2.554188	0.01064358	0.192311524
RPL18A	0.163712	0.064156	2.551768	0.01071779	0.192311524
EIF5	0.225671	0.088464	2.551006	0.01074125	0.192311524
ASF1A	0.555118	0.217666	2.550313	0.01076262	0.192311524
ACYP2	-0.421799	0.16542	-2.549865	0.01077645	0.192311524
HCK	-1.01233	0.397034	-2.549729	0.01078066	NA
ENSG00000268400	-2.224688	0.873754	-2.546125	0.01089262	NA
MRPL28	-0.337067	0.132445	-2.544967	0.01092882	0.193208439
C19orf48	0.650353	0.255635	2.544073	0.01095681	0.193208439
TCL1A	-0.492815	0.193716	-2.544012	0.01095875	0.193208439
PPIL3	0.542645	0.213793	2.538181	0.01114303	0.195289652
ENSG00000224505	-0.485315	0.19126	-2.537468	0.01116576	0.195289652
ENSG00000262049	-0.672482	0.265604	-2.531893	0.01134487	NA
IFIT5	0.791867	0.312843	2.531196	0.01136742	NA
DUSP10	1.016273	0.401519	2.531073	0.01137141	0.198097192
FAAP100	-0.807209	0.319194	-2.528901	0.01144203	NA
RPLP0	0.296406	0.117433	2.524049	0.01160116	0.201300801
ADA	-0.487317	0.193329	-2.520663	0.0117134	0.20244807

NACA	0.145762	0.058015	2.512468	0.01198901	0.205265105
LSM6	0.372828	0.148429	2.511824	0.01201089	0.205265105
RPS21	0.13942	0.055509	2.511655	0.01201666	0.205265105
MDFIC	0.972304	0.387211	2.511043	0.01203751	NA
PIGF	0.6144	0.24475	2.510311	0.0120625	NA
ACP1	-0.23363	0.093083	-2.509917	0.01207597	0.205384579
BIN2	-0.614188	0.244822	-2.508712	0.01211722	0.205384579
NSMCE4A	-0.626845	0.250077	-2.506611	0.01218947	0.205814493
SLC26A6	-1.129513	0.450804	-2.50555	0.01222612	NA
HIPK2	1.015212	0.405386	2.504311	0.01226902	NA
LINC01006	0.928601	0.371288	2.501025	0.01238344	NA
XKR8	0.750656	0.300255	2.500065	0.01241706	NA
LIMD2	-0.220668	0.088332	-2.49816	0.01248398	0.209979628
EEF1A1	0.221435	0.08871	2.496179	0.01255393	0.210087649
PSMC4	-0.450975	0.180732	-2.49527	0.01258612	0.210087649
SEM1	0.23522	0.094339	2.49335	0.01265441	0.210427541
VARS	-1.116425	0.448749	-2.487863	0.01285131	NA
TARBP1	-0.930259	0.373959	-2.487594	0.01286103	NA
TLE2	-1.396397	0.561393	-2.487379	0.01286884	NA
ACCS	1.217358	0.489421	2.487343	0.01287014	NA
TCEAL4	0.396263	0.159435	2.485414	0.01294007	0.214365705
THAP8	1.194755	0.481127	2.48324	0.01301935	NA
WARS	1.276757	0.514197	2.483011	0.01302771	NA
MT1F	1.36643	0.551174	2.479127	0.01317043	NA
LGALS3BP	-1.302909	0.525588	-2.478956	0.01317674	NA
ENSG00000225938	-0.448517	0.181176	-2.475584	0.01330186	NA
GLS	0.517679	0.209926	2.466002	0.01366305	NA
PRDX3	0.390982	0.158584	2.465457	0.01368386	0.225752816
JUND	-0.300382	0.121899	-2.464197	0.01373208	0.225752816
HINT1	0.18137	0.073641	2.462903	0.01378172	0.225752816
RPL27	0.132237	0.053758	2.459856	0.01389928	0.22683203
CCT4	0.284231	0.115718	2.456246	0.01403971	0.228275355
PPP1R14A	-0.769508	0.313512	-2.454476	0.01410899	0.228555211
LILRA4	1.713189	0.698207	2.453697	0.0141396	NA
NEK6	1.664151	0.678484	2.452749	0.01417691	NA
TCF7	-0.565165	0.230878	-2.447898	0.01436925	0.231915407
AIP	0.29976	0.122742	2.442192	0.01459837	0.234750381
ENSG00000270659	-1.266716	0.519042	-2.44049	0.01466734	NA
SMARCE1	-0.383929	0.157416	-2.438944	0.01473027	0.23600682
WDR46	-0.407025	0.167102	-2.435786	0.01485948	0.237211398
ADRB2	1.207859	0.496598	2.432267	0.01500464	NA
MTX2	0.762875	0.313726	2.431659	0.01502985	NA
ARF5	-0.345778	0.142363	-2.428854	0.01514664	0.240686674
SELENOF	-0.21497	0.088542	-2.427893	0.01518684	0.240686674
RAB33A	1.876969	0.773413	2.426866	0.01522986	NA
PDPK1	0.526584	0.216995	2.426716	0.01523616	NA
HOXB2	1.224795	0.50473	2.426635	0.01523959	NA

RPS4X	0.228274	0.094194	2.423436	0.01537447	0.24278385
IFNG.AS1	-1.729449	0.713862	-2.422664	0.01540716	NA
C11orf1	0.732996	0.302584	2.422457	0.01541597	NA
ZFPL1	-0.505832	0.209051	-2.419655	0.01553522	0.244443083
DNAJB1	0.551825	0.228294	2.417164	0.01564195	NA
MFAP1	0.572747	0.237069	2.415952	0.01569412	NA
YBX3	0.510069	0.211191	2.4152	0.01572657	0.246570082
SMIM10	-2.96597	1.228452	-2.414396	0.01576131	NA
TCEAL8	0.340345	0.141182	2.410679	0.01592284	0.248758909
TMEM71	-0.69673	0.289153	-2.409555	0.01597197	NA
PTPN7	-0.613514	0.254705	-2.408722	0.01600851	0.249210453
NSUN7	-1.10649	0.459596	-2.407526	0.01606102	NA
LGALS3	1.462824	0.607674	2.407252	0.0160731	NA
BLNK	0.228004	0.094741	2.406597	0.01610195	0.249779287
EXOG	-0.42336	0.176019	-2.40519	0.01616408	0.249860263
DOCK9	-1.226726	0.510929	-2.40097	0.01635168	NA
ENSG00000275457	-1.153587	0.480496	-2.400824	0.01635818	NA
VSIR	-0.617321	0.257164	-2.400498	0.01637277	NA
CETN3	0.555939	0.231612	2.4003	0.01638165	NA
RPL39	0.181223	0.075579	2.397784	0.01649458	0.254074409
RGS16	-1.075882	0.448982	-2.396271	0.01656285	NA
SYNGR2	-0.424975	0.177423	-2.395268	0.0166082	0.254930082
ACTG1	0.444112	0.185528	2.393781	0.01667572	0.255074606
PSMB3	-0.337922	0.141268	-2.392052	0.01675445	0.255389041
PRAG1	1.214425	0.507775	2.391661	0.01677234	NA
CHMP6	-0.68359	0.285939	-2.390684	0.01681702	NA
POLE3	-0.349142	0.14609	-2.38992	0.01685207	0.25585425
MRPL32	0.346513	0.145055	2.388843	0.01690153	0.25585425
PTPRS	1.786998	0.749132	2.385425	0.01705939	NA
ENSG00000125726	-1.460738	0.613357	-2.381544	0.01724023	NA
LRIF1	0.413023	0.173658	2.378368	0.01738946	0.262315137
ZNF680	0.443364	0.186513	2.377116	0.01744858	0.262315137
ZNF844	-0.708838	0.298272	-2.376482	0.01747861	NA
LILRB1	0.537768	0.226345	2.375871	0.01750759	0.262315137
SREBF2.AS1	-1.032379	0.435088	-2.372806	0.01765354	NA
RPS19	0.177518	0.075032	2.36591	0.01798581	0.268137769
MTMR10	-1.242421	0.525384	-2.364786	0.01804051	NA
PDCD4	0.464537	0.196464	2.364486	0.01805509	0.268137769
GUCD1	-0.38043	0.160927	-2.363987	0.01807945	0.268137769
HELZ2	0.672957	0.284812	2.362814	0.01813678	NA
C21orf62	-0.994212	0.420839	-2.362451	0.01815455	NA
PIGM	0.749959	0.31751	2.362002	0.01817655	NA
CD37	-0.151398	0.064111	-2.361489	0.01820173	0.26825635
ARPC3	-0.197925	0.083819	-2.361327	0.01820966	0.26825635
ATP6AP2	-0.387576	0.164378	-2.357829	0.01838218	0.269892209
ZNF217	0.6834	0.290003	2.35653	0.01844659	NA
PGBD4	-1.133658	0.481218	-2.355812	0.01848228	NA

ERI3	-0.517547	0.219967	-2.35284	0.01863066	NA
DNAJC15	0.458541	0.194977	2.351765	0.0186846	0.272790138
CCT8	0.343638	0.146143	2.351382	0.01870383	0.272790138
KLK2	1.646026	0.701556	2.346251	0.01896332	NA
KCTD10	-0.83192	0.3547	-2.345417	0.01900582	NA
CLTA	0.223657	0.095409	2.344198	0.01906803	0.276869121
WDFY1	0.756786	0.322946	2.343385	0.01910965	0.276869121
ENSG00000275799	0.639039	0.27277	2.342775	0.01914093	NA
RCSD1	-0.388272	0.166159	-2.33675	0.01945217	0.279847398
ZNF638	-0.387972	0.16606	-2.33634	0.01947352	0.279847398
TCIRG1	-0.492072	0.210733	-2.335047	0.01954096	0.279847398
HNRNPA0	-0.174913	0.074954	-2.333587	0.01961738	0.279847398
ENSA	-0.320771	0.137477	-2.333271	0.01963394	0.279847398
MAP3K1	0.407206	0.174618	2.331979	0.01970179	0.279905691
RBM18	0.521686	0.223741	2.331651	0.01971903	NA
THEMIS2	0.644877	0.276819	2.329596	0.01982754	NA
RNF34	0.438395	0.188264	2.328619	0.01987928	0.281516201
PSMB10	-0.280321	0.120491	-2.3265	0.01999192	0.282201017
PTPRE	-0.712445	0.306401	-2.325205	0.02006101	NA
ISG20	-0.311248	0.133961	-2.323415	0.02015684	0.283617115
CLEC4A	-1.327424	0.571381	-2.323184	0.02016927	NA
DIS3L	0.69288	0.298886	2.318211	0.02043784	NA
FAM162A	0.366733	0.158208	2.318051	0.02044654	0.28437404
GPSM3	-0.162552	0.070155	-2.31705	0.02050099	0.28437404
NPM3	0.511422	0.220835	2.315859	0.02056596	NA
TNFAIP8	0.226098	0.097631	2.315841	0.02056697	0.28437404
PDE3B	-0.595308	0.257153	-2.314993	0.02061334	0.28437404
DPP9	-0.577639	0.249615	-2.314124	0.02066092	NA
FUT11	0.566357	0.244795	2.313602	0.02068955	NA
PPCS	-0.259587	0.112288	-2.311791	0.02078919	0.28437404
YWHAG	0.49031	0.212174	2.310883	0.02083934	NA
CYB561A3	-0.407153	0.176298	-2.309459	0.02091811	0.28437404
HMOX1	0.922811	0.399632	2.309153	0.02093511	NA
USP34	0.290042	0.12561	2.309068	0.0209398	0.28437404
CHPT1	0.342234	0.148251	2.308478	0.02097259	0.28437404
TAOK3	0.383847	0.166289	2.308314	0.0209817	0.28437404
TOP2A	-1.798637	0.779319	-2.307959	0.02100144	NA
ITGA5	-1.202907	0.521337	-2.30735	0.02103532	NA
MCM7	-0.42612	0.184703	-2.307057	0.02105165	0.28437404
ENSG00000089009	0.15901	0.068923	2.307055	0.02105175	0.28437404
RAD9A	-0.306003	0.132715	-2.305711	0.02112676	0.28437404
ZKSCAN1	-0.295495	0.128166	-2.305564	0.02113498	0.28437404
BMS1	-0.473172	0.205259	-2.305243	0.02115294	0.28437404
LINC.PINT	0.282542	0.122593	2.304719	0.0211823	0.28437404
RBM4	-0.355773	0.154513	-2.302538	0.02130484	0.285147097
PTCD2	1.022943	0.444712	2.300235	0.0214349	NA
UNC119	-0.400876	0.174291	-2.300043	0.02144576	0.285202512

PTPRC	0.240985	0.10478	2.299905	0.02145361	0.285202512
AFF3	0.277665	0.120775	2.299019	0.02150388	0.285202512
S1PR2	-0.817213	0.355848	-2.296521	0.02164611	NA
ALDH9A1	0.48389	0.211014	2.293162	0.02183871	0.286101378
MT.CO3	0.216848	0.094571	2.292972	0.02184963	0.286101378
CAMTA1	-0.369718	0.161255	-2.292747	0.02186257	0.286101378
DDT	0.283884	0.12383	2.292529	0.02187511	0.286101378
SEPTIN6	0.233544	0.101952	2.290729	0.02197908	0.286101378
CD1D	1.039622	0.45402	2.289815	0.02203206	NA
ZNF197	-1.07089	0.468226	-2.287124	0.02218857	NA
ENSG00000223881	-1.639721	0.716968	-2.287022	0.02219455	NA
TOMM5	-0.342731	0.149893	-2.286502	0.02222493	0.286101378
AKAP9	0.195426	0.085493	2.28587	0.02226185	0.286101378
NUAK2	-0.338909	0.148276	-2.28566	0.02227415	0.286101378
CDC25B	-0.532614	0.233048	-2.285421	0.02228818	0.286101378
IGKC	-0.38846	0.169988	-2.285226	0.02229959	0.286101378
PDCD4.AS1	0.655938	0.287063	2.284994	0.0223132	NA
AKIRIN2	0.324191	0.141889	2.284828	0.02232292	0.286101378
GLRX2	0.584377	0.25597	2.282993	0.02243081	NA
ARHGDIB	-0.210431	0.09218	-2.282836	0.02244003	0.286101378
EVI2A	0.72716	0.318658	2.281944	0.02249262	0.286101378
RBIS	0.223301	0.097879	2.281403	0.02252461	0.286101378
CSKMT	-0.3101	0.13595	-2.280987	0.02254922	0.286101378
LDB1	0.981602	0.430629	2.279462	0.02263965	NA
STK4	0.23334	0.102505	2.276378	0.02282339	0.288745517
SOAT1	0.715767	0.314662	2.274722	0.02292263	NA
MRPL42	0.393101	0.172951	2.272902	0.02303209	0.290548499
ITGA6.AS1	-0.77291	0.341209	-2.265208	0.02349991	NA
A4GALT	1.479946	0.653592	2.264328	0.02355396	NA
ZC3H12A	-0.593941	0.262434	-2.263201	0.02362332	NA
GYPC	0.276182	0.122065	2.262587	0.02366116	0.297179097
ENSG00000257275	-0.562004	0.248447	-2.26207	0.02369309	0.297179097
GNLY	-2.494631	1.102895	-2.261893	0.02370401	NA
TMUB1	-0.281321	0.124585	-2.258063	0.02394171	0.298927988
PARP14	0.293207	0.129908	2.257031	0.02400611	0.298927988
VOPP1	-0.34828	0.154342	-2.25654	0.02403681	0.298927988
RB1	-0.322782	0.143313	-2.25228	0.02430461	0.299993012
NR6A1	-1.504241	0.667894	-2.252215	0.0243087	NA
CNIH4	0.457404	0.203154	2.251514	0.02435301	0.299993012
ZDHHC14	-0.817393	0.363104	-2.251125	0.02437762	0.299993012
ENSG00000267364	1.006415	0.447094	2.251016	0.02438452	NA
GTF2F2	0.407047	0.180843	2.250838	0.02439579	0.299993012
SNHG10	-0.495528	0.220175	-2.250606	0.02441048	NA
WASHC2C	0.604669	0.26875	2.249934	0.02445312	NA
PIM1	-0.344468	0.153187	-2.248682	0.02453271	0.300806621
ZNF33A	0.369942	0.164591	2.247643	0.02459899	0.300806621
ENSG00000254614	-0.801353	0.356579	-2.247336	0.02461853	NA

ENSG00000273253	0.717714	0.319435	2.246825	0.02465121	NA
UXT	0.208344	0.092753	2.246224	0.02468966	0.301076732
IL21R	-0.836003	0.372442	-2.244654	0.02479038	NA
FCGR2B	0.556411	0.24807	2.24296	0.02489937	0.302792871
LACTB2	0.503431	0.224637	2.241086	0.02502048	NA
ZNF266	-0.747711	0.333757	-2.240283	0.02507253	NA
ENSG00000263394	0.986304	0.440487	2.239121	0.02514805	NA
RNF185	0.609168	0.272114	2.23865	0.02517867	NA
RBM19	-0.5211	0.232941	-2.237041	0.02528365	NA
RSBN1L	0.360431	0.161303	2.234493	0.02545065	0.308641797
CCDC90B	0.322839	0.144662	2.231673	0.02563657	0.309217094
AGPAT2	-0.340226	0.152456	-2.231637	0.02563896	0.309217094
GNPDA1	0.984259	0.441507	2.229317	0.02579283	NA
ENSG00000258056	1.010512	0.453299	2.229238	0.02579806	NA
LAPTM5	-0.179307	0.080496	-2.227521	0.0259125	0.311659978
IFI30	0.469818	0.211162	2.224915	0.02608695	0.31278158
HDX	1.714021	0.770907	2.223384	0.02618992	NA
ENSG00000278834	-1.36409	0.61385	-2.222189	0.02627053	NA
ZBTB25	0.462361	0.208073	2.222113	0.02627567	0.31278158
RPS29	0.128578	0.057867	2.221964	0.02628574	0.31278158
ACAT1	-0.533146	0.239975	-2.22167	0.02630561	0.31278158
KIAA0040	0.338496	0.152418	2.220837	0.026362	0.31278158
MRPL41	-0.368541	0.16604	-2.219593	0.02644638	0.312937001
EPS8L2	0.813793	0.366738	2.219007	0.02648624	NA
C1QBP	0.346505	0.156242	2.217745	0.0265722	0.313576828
NFE2L2	0.307081	0.138586	2.215821	0.02670378	0.313576828
CRIPT	0.383884	0.173303	2.215107	0.02675274	0.313576828
NBPF15	0.920714	0.415662	2.215054	0.02675635	NA
EIF4A2	0.158604	0.071619	2.214559	0.02679035	0.313576828
ENSG00000273748	-0.855945	0.386679	-2.213581	0.02685761	0.313576828
ZNHIT3	0.268025	0.121394	2.207885	0.02725226	0.317340681
KLHL6	0.655891	0.297544	2.204354	0.02749946	0.318784124
SPPL2A	-0.365946	0.166035	-2.20403	0.02752226	0.318784124
TOE1	-0.536737	0.243534	-2.203954	0.02752759	NA
ENSG00000180448	-0.286637	0.13019	-2.201682	0.02768781	0.318784124
PHF3	0.273361	0.124208	2.200838	0.02774747	0.318784124
LY6E	0.352685	0.16026	2.200712	0.02775644	0.318784124
DLGAP1.AS1	-0.345767	0.157172	-2.199929	0.02781192	0.318784124
ZNF441	-0.479249	0.217866	-2.199749	0.02782473	NA
SETBP1	0.57817	0.262964	2.198662	0.02790197	NA
APBB1	0.783933	0.356676	2.197889	0.02795703	NA
DGCR6	-1.642796	0.747816	-2.196791	0.0280354	NA
ATF7IP2	-0.495903	0.225758	-2.196615	0.02804793	0.320652083
JARID2	-0.329517	0.15038	-2.191226	0.02843543	0.324237753
THG1L	0.513257	0.234379	2.18986	0.02853438	NA
P4HA1	-0.563782	0.257526	-2.189221	0.02858075	NA
TXN	0.36722	0.167969	2.18624	0.02879804	0.327521726

FAM78A	0.565669	0.259389	2.180772	0.02920032	NA
ATP2B1.AS1	-0.354655	0.16286	-2.177665	0.029431	0.33385547
ENSG00000272182	-1.415776	0.650777	-2.175518	0.02959134	NA
KDELR2	-0.325908	0.149855	-2.174828	0.02964298	0.335393453
PRICKLE1	0.63481	0.29195	2.174381	0.0296765	NA
SPON1	-0.582867	0.268228	-2.173031	0.02977797	NA
IRS2	0.441792	0.203327	2.172811	0.02979452	0.336241457
GLMP	0.727388	0.334801	2.172598	0.02981059	NA
PILRB	0.581353	0.267728	2.171435	0.02989828	0.336547255
MBNL2	-0.579997	0.267245	-2.170285	0.0299853	NA
GPRIN3	1.270375	0.585449	2.169917	0.03001312	NA
USP53	0.908881	0.418899	2.169688	0.03003047	NA
RP9	0.511163	0.235692	2.168775	0.03009981	NA
TMEM243	0.248947	0.114863	2.167344	0.03020866	0.339021816
STAM2	0.770944	0.355713	2.167322	0.03021028	NA
MAVS	0.391034	0.180491	2.166506	0.03027256	0.339021816
BAG2	0.666968	0.307911	2.166104	0.03030325	NA
FAM160B1	-0.841211	0.38839	-2.165893	0.03031938	NA
TLE3	-0.345045	0.159495	-2.163364	0.03051318	0.340403987
COL9A3	-0.738666	0.341521	-2.162872	0.03055106	0.340403987
FCRL4	1.767295	0.817246	2.162502	0.03057951	NA
PSMC3	-0.319856	0.148083	-2.159982	0.03077409	0.34202085
IL3RA	-2.152479	0.997293	-2.158321	0.03090285	NA
IMPDH2	0.254463	0.117992	2.156604	0.03103652	0.344066507
OPTN	-0.649321	0.301236	-2.155524	0.03112087	0.344132549
NAIP	0.841034	0.390213	2.15532	0.0311368	NA
ENSG00000259943	-0.75118	0.34864	-2.154601	0.0311931	NA
ENSG00000232698	-1.97676	0.917809	-2.15378	0.03125742	NA
GBGT1	0.823322	0.38292	2.150116	0.03154604	NA
ARPC1B	-0.169744	0.079043	-2.147507	0.03175295	0.349596089
S100A4	0.557542	0.259655	2.147239	0.03177422	0.349596089
CD40	-0.296761	0.13832	-2.145465	0.03191565	0.3502743
C6orf120	0.788246	0.367557	2.144554	0.03198852	NA
PEG10	1.186232	0.553142	2.144533	0.03199017	NA
MGAT4A	-0.652293	0.304207	-2.144238	0.0320138	NA
MGST2	-1.55322	0.724552	-2.143699	0.03205703	NA
C9orf72	-0.648897	0.30283	-2.142774	0.03213121	NA
CASZ1	-1.197984	0.559388	-2.141597	0.03222592	NA
IL10RB	0.357049	0.166726	2.141528	0.03223151	0.352858627
POLR1D	0.322755	0.150836	2.139773	0.03237311	0.353371728
CD55	-0.308352	0.14416	-2.138954	0.03243936	0.353371728
WASH9P	-0.500355	0.234059	-2.137732	0.03253848	0.353574085
TRIP10	0.689658	0.323003	2.135144	0.03274923	NA
HLA.DMA	-0.253601	0.11882	-2.134329	0.03281587	0.354384105
TLK1	0.284064	0.133108	2.13409	0.03283538	0.354384105
KRCC1	0.286483	0.134257	2.133848	0.0328552	0.354384105
SNHG32	0.208281	0.097662	2.132662	0.03295242	0.354561603

RBM11	-1.772798	0.831416	-2.132263	0.03298526	NA
MED6	-0.31004	0.145548	-2.130157	0.03315865	0.355908244
FBXO44	-0.432052	0.202856	-2.129849	0.03318412	NA
SAG	-2.092472	0.983331	-2.127943	0.03334184	NA
LINC01215	-0.39059	0.183588	-2.127536	0.03337557	0.356425413
NOC3L	0.409471	0.192475	2.127399	0.03338696	0.356425413
QARS	0.279366	0.131365	2.126635	0.0334504	0.356425413
GPR160	0.812755	0.382669	2.123913	0.03367746	NA
RPS11	0.225168	0.106081	2.122613	0.03378628	0.358705594
ZFAND2A	-0.457092	0.215432	-2.121745	0.03385914	0.358705594
MBTPS1	-0.460218	0.217045	-2.12038	0.03397397	0.358705594
HSP90AB1	0.227383	0.107253	2.120055	0.03400137	0.358705594
SMIM10L1	-0.31695	0.149577	-2.118976	0.03409252	0.358705594
SDR39U1	-0.351727	0.166116	-2.117363	0.03422905	0.358705594
TMEM102	0.723851	0.341877	2.117285	0.03423567	NA
TSN	-0.326394	0.154157	-2.117277	0.03423637	0.358705594
MYL9	1.289513	0.609178	2.116809	0.03427606	NA
ENSG00000228106	-0.618851	0.292382	-2.116583	0.03429529	NA
MRPL45	0.401627	0.189942	2.114473	0.03447487	0.360344457
TPP1	0.368899	0.174591	2.112933	0.03460652	0.360662686
JMY	0.765115	0.362226	2.112259	0.03466425	NA
RGS2	0.528935	0.250419	2.112196	0.03466963	0.360662686
ENSG00000258017	-1.531335	0.725274	-2.111387	0.03473907	NA
KLF2	-0.587745	0.278651	-2.109251	0.03492295	0.36243913
AMZ2	-0.333879	0.15844	-2.107294	0.03509214	0.36333609
CENPB	-0.663766	0.314991	-2.107252	0.03509576	NA
PYCR3	-0.59008	0.280041	-2.107118	0.03510732	NA
ENSG00000275964	-0.784723	0.372477	-2.10677	0.03513756	NA
LAS1L	-0.574022	0.272503	-2.106475	0.03516311	NA
SLC16A3	0.578274	0.274603	2.105853	0.03521714	NA
KLHL5	-0.514584	0.244527	-2.104405	0.03534317	0.364326957
TMEM179B	-0.318569	0.151391	-2.104282	0.03535382	0.364326957
SMARCC1	0.377925	0.17972	2.102852	0.03547869	0.364332788
FAM210B	0.916268	0.435771	2.102637	0.03549751	NA
PPP1R16B	0.475143	0.226003	2.102376	0.03552037	0.364332788
CYBB	-0.494413	0.23537	-2.100579	0.03567795	0.365096032
KLHL21	0.828682	0.394538	2.100384	0.03569506	NA
PARP15	0.705882	0.336386	2.09843	0.03586721	0.366179214
ATP5MC2	0.159432	0.076017	2.097321	0.0359652	0.366327624
PLEK	-0.655757	0.312826	-2.096235	0.03606131	NA
BIRC3	-0.389781	0.18598	-2.095822	0.03609797	0.366828923
C17orf49	-0.63357	0.302733	-2.092832	0.03636413	NA
PTTG1IP	0.478212	0.228641	2.091539	0.03647976	NA
CD180	0.430647	0.20604	2.090111	0.0366078	0.368871265
RAB11B	-0.305894	0.146353	-2.090107	0.03660815	0.368871265
GUK1	-0.218209	0.104404	-2.090045	0.03661378	0.368871265
NSMCE2	0.414374	0.198283	2.089808	0.03663505	0.368871265

RBBP8	-0.686223	0.328688	-2.087764	0.03681914	NA
KRR1	0.392701	0.188288	2.085639	0.03701136	0.370563313
NBPF12	-0.633476	0.303744	-2.085558	0.03701865	NA
ABI1	0.317746	0.152402	2.084921	0.03707643	0.370563313
ENSG00000281706	1.320804	0.633579	2.084671	0.03709916	NA
MRPL44	0.602743	0.289194	2.084218	0.03714032	0.370563313
SCAI	0.356894	0.171261	2.083918	0.03716764	0.370563313
LINC01003	-1.289205	0.618905	-2.083042	0.03724739	NA
WDSUB1	0.558743	0.268267	2.082784	0.03727088	0.370563313
TMBIM4	-0.187969	0.090267	-2.08236	0.03730956	0.370563313
SMOX	-1.60638	0.77227	-2.080077	0.0375185	NA
ANXA6	-0.302652	0.145537	-2.079547	0.03756711	0.372279016
INTS6L	0.749556	0.360514	2.079134	0.037605	NA
RAB3A	1.1057	0.532193	2.077628	0.03774367	NA
NOL12	-0.45346	0.218446	-2.075843	0.0379085	0.373265392
ENSG00000272583	-1.039383	0.500889	-2.075078	0.03797927	NA
HNRNPH1	-0.193177	0.093106	-2.0748	0.03800512	0.373265392
PFDN5	0.104459	0.050347	2.074785	0.03800649	0.373265392
SCRN2	0.449362	0.216583	2.074782	0.03800675	0.373265392
ARFIP1	-0.638555	0.3078	-2.074575	0.0380259	NA
CYBC1	-0.227367	0.109653	-2.073516	0.03812432	0.373584304
HIST3H2A	1.32284	0.638403	2.072109	0.03825528	NA
ATP5PB	0.254334	0.122955	2.068505	0.03859255	0.377330316
NHLRC2	0.609431	0.294637	2.06841	0.03860147	NA
YTHDC2	0.481696	0.233067	2.066769	0.03875594	NA
VAT1	0.517763	0.250687	2.06538	0.03888708	0.379365102
NAA40	-0.708357	0.343184	-2.064074	0.03901071	NA
ETNK1	0.412258	0.199752	2.063848	0.03903214	0.37993593
GPBP1L1	-0.285501	0.138401	-2.062856	0.03912628	0.380009644
COCH	-0.941859	0.456965	-2.061117	0.03929188	NA
C2CD3	1.030966	0.500687	2.059102	0.03948444	NA
EAF2	-0.487194	0.236648	-2.058728	0.03952034	0.381719331
NCBP2	-0.278977	0.135567	-2.057854	0.0396042	0.381719331
SOD2	-0.390598	0.189865	-2.057244	0.03966275	0.381719331
HSD17B11	0.175638	0.08539	2.0569	0.03969588	0.381719331
SH3GLB1	0.262898	0.127839	2.056472	0.03973707	0.381719331
ENSG00000260261	-0.79762	0.388498	-2.053088	0.04006409	NA
ATP13A3	0.695154	0.338696	2.052446	0.04012637	NA
C1orf56	-0.378251	0.184359	-2.051713	0.04019752	0.384365647
ANKRD17	0.437805	0.213416	2.051417	0.04022635	0.384365647
TRMT13	0.338925	0.165305	2.050298	0.04033535	0.384365647
LINC01857	0.506938	0.247285	2.050017	0.04036277	0.384365647
POLE2	1.281485	0.625184	2.049772	0.04038666	NA
HSBP1	0.332448	0.162267	2.048771	0.04048451	0.384638321
SPCS2	-0.194972	0.095204	-2.047932	0.04056664	0.384638321
IRF1	-0.233434	0.114061	-2.04657	0.04070031	0.3847556
SRSF2	-0.194361	0.094995	-2.046021	0.0407543	0.3847556

LY96	0.451443	0.220901	2.043648	0.04098833	0.385395946
NDUFS5	0.187255	0.091632	2.043553	0.0409977	0.385395946
EXD3	-0.549349	0.268977	-2.042367	0.04111509	NA
E2F4	-0.414634	0.203065	-2.04188	0.04116346	0.386100663
ZNF263	-0.777316	0.380907	-2.040699	0.04128074	0.386100663
KCNMB3	-0.751939	0.368482	-2.040642	0.04128646	NA
ANK1	-1.354002	0.663748	-2.039933	0.04135705	NA
IKBIP	0.406458	0.199285	2.039579	0.04139227	0.386100663
ZFYVE21	0.589182	0.288913	2.039305	0.04141963	NA
PRR4	0.388028	0.190279	2.039256	0.04142447	0.386100663
ZNF827	0.918001	0.450179	2.03919	0.04143105	NA
RPS6KB2.AS1	-1.156091	0.567064	-2.038732	0.04147678	NA
RASSF5	-0.277228	0.136155	-2.03612	0.04173835	0.387840009
LINC00667	-0.395366	0.194223	-2.035628	0.04178777	0.387840009
MYC	0.342591	0.168486	2.03335	0.04201718	0.388641884
EIF3L	0.188132	0.09254	2.032975	0.04205508	0.388641884
CD47	0.179202	0.088184	2.032137	0.04213976	0.388641884
OPA3	0.660306	0.324967	2.031919	0.0421619	NA
CD151	-0.601159	0.295995	-2.030973	0.04225776	NA
TTN.AS1	0.801579	0.394692	2.030897	0.04226543	NA
ALG11	-1.023927	0.504594	-2.02921	0.04243695	NA
CERS2	0.46276	0.228349	2.026549	0.04270854	0.392673635
KCTD5	0.456834	0.225437	2.026436	0.04272008	NA
ZNF706	0.19809	0.09777	2.026088	0.04275581	0.392673635
DHX57	-0.537466	0.265514	-2.024251	0.04294434	0.393213066
THOC2	0.31543	0.155862	2.023771	0.04299368	0.393213066
VASP	-0.269599	0.133289	-2.022658	0.04310842	0.393350934
RPRML	-2.029939	1.003659	-2.022538	0.0431208	NA
CHTF18	-0.738699	0.365331	-2.022	0.04317631	NA
BNIP2	-0.271892	0.134474	-2.021888	0.04318796	0.393350934
CDK11A	-0.385768	0.191042	-2.019285	0.04345764	0.394284009
SNAPC2	0.429861	0.212891	2.019165	0.04347004	0.394284009
TOB1	0.829636	0.411005	2.018558	0.04353322	NA
EPC1	-0.207882	0.103024	-2.017797	0.04361242	0.394759882
DIPK1B	-1.177899	0.584412	-2.015529	0.04384927	NA
CHD7	0.477435	0.237062	2.013964	0.04401331	0.397568778
ENSG00000268584	-0.884426	0.439285	-2.01333	0.04407994	NA
IK	-0.236393	0.117473	-2.012312	0.04418705	0.397995581
FAM122A	0.435463	0.216455	2.011792	0.04424188	0.397995581
ENSG00000258308	-1.873531	0.93178	-2.010701	0.04435702	NA
ARL3	0.451558	0.224701	2.009593	0.04447427	NA
SIMC1	-0.44894	0.223401	-2.009574	0.04447624	NA
RIN3	0.533268	0.265394	2.009342	0.04450083	0.399506463
FOXO3	0.668193	0.332546	2.009326	0.04450262	NA
CTBP1.AS	-1.410965	0.702423	-2.008712	0.04456771	NA
PAPOLG	0.706824	0.351918	2.008489	0.04459134	NA
GNA12	0.522306	0.260161	2.007627	0.04468296	NA

RASSF1.AS1	-1.074224	0.535339	-2.006624	0.04478976	NA
ARMC1	0.425427	0.212137	2.005435	0.04491661	0.40241615
ZNF117	-1.05132	0.524478	-2.004508	0.04501564	NA
ATP2A3	-0.31653	0.157919	-2.00438	0.04502933	0.402604392
SGPP1	0.841284	0.420054	2.002799	0.04519886	NA
GTF2F1	0.253829	0.126752	2.002561	0.04522443	0.403029689
GPATCH4	0.456565	0.228029	2.002225	0.04526051	0.403029689
GAB1	0.93316	0.466128	2.001938	0.04529144	NA
EXOSC5	0.486519	0.243065	2.001596	0.0453282	NA
ZCCHC9	0.394145	0.197119	1.999534	0.04555064	0.404492737
AZIN1	-0.367393	0.183789	-1.998993	0.04560909	0.404492737
TSC22D1	-0.722011	0.361538	-1.997051	0.04581959	NA
LAMB1	-0.892231	0.447048	-1.995828	0.04595266	NA
DARS	0.262559	0.131632	1.994651	0.04608091	0.407853204
NFATC2	0.58603	0.293811	1.99458	0.04608874	NA
PCLAF	-1.416759	0.71072	-1.993413	0.04621628	NA
SHKBP1	-0.285051	0.143043	-1.992756	0.04628821	0.408863667
ENSG00000271737	-0.925611	0.465032	-1.990424	0.04654429	NA
KDM5B	0.627232	0.315302	1.98931	0.04666705	NA
PTGES2	-0.39006	0.196131	-1.988773	0.04672631	0.411904589
FOXRED1	-0.66109	0.332668	-1.987238	0.046896	NA
CCDC66	0.34872	0.175483	1.987206	0.0468996	0.412603652
LINC02422	0.50562	0.254467	1.986979	0.04692477	NA
PSMC1	-0.280645	0.141309	-1.986035	0.04702949	0.412918902
SIGLEC14	0.727423	0.36633	1.985704	0.04706618	NA
IFITM3	-1.254177	0.631868	-1.984873	0.04715866	NA
SENP5	-0.384632	0.193935	-1.983307	0.04733319	0.414569198
SMPD2	-0.44758	0.225747	-1.98266	0.04740539	NA
NRBF2	0.343806	0.173407	1.982652	0.04740632	0.414569198
ENSG00000266088	-2.201717	1.110794	-1.982111	0.04746684	NA
HOOK1	1.051888	0.531104	1.98057	0.04763951	NA
RPRD1A	-0.37395	0.18882	-1.980459	0.04765203	0.41588948
POU2F2	0.22034	0.111348	1.978832	0.04783493	0.416657411
SEPSECS.AS1	0.763464	0.386022	1.977775	0.04795415	NA
IDH2	-0.303586	0.15351	-1.977621	0.04797152	0.41678685
WRNIP1	0.700629	0.354386	1.977023	0.04803899	NA
KAT2A	-0.380379	0.1924	-1.977017	0.04803967	0.41678685
FAAP24	-0.830852	0.420424	-1.976222	0.04812962	NA
KHDC4	-0.255416	0.129316	-1.975129	0.04825348	0.417816127
POU2AF1	-0.201423	0.102065	-1.973481	0.0484408	0.418447872
ATP6V1A	0.442154	0.224124	1.972811	0.04851708	0.418447872
ARMH1	-0.874963	0.444209	-1.969713	0.04887131	NA
LHFPL4	0.937625	0.476065	1.96953	0.04889231	NA
TRIM4	0.448559	0.22776	1.969434	0.04890325	0.419928263
HLA.F	-0.213038	0.108186	-1.969189	0.04893139	0.419928263
MICOS10	0.200228	0.101701	1.968803	0.04897569	0.419928263
GCNT2	0.631227	0.320629	1.968716	0.04898575	NA

MYH3	-1.247331	0.63374	-1.968205	0.04904451	NA
ENSG00000225342	-0.676192	0.344048	-1.965399	0.04936807	NA
MT.ND6	0.420339	0.214127	1.96304	0.04964157	0.424807975
ENSG00000280011	-1.106075	0.563746	-1.962009	0.04976142	NA
LTB	-0.342365	0.174502	-1.961961	0.049767	0.424920632
DDX11	-0.859189	0.438054	-1.961378	0.04983494	NA
RND1	-1.145074	0.583838	-1.961286	0.04984561	NA
LYST	-0.389073	0.198393	-1.961121	0.04986487	0.424920632
SEPTIN7	0.195252	0.099596	1.960434	0.04994511	0.424920632

Cluster 9	log2FC	lfcSE	stat	pvalue	padj
DHTKD1	-1.036891	0.13692	-7.572973	3.65E-14	3.61E-10
ARL17A	-1.124521	0.17256	-6.516703	7.19E-11	3.55E-07
CKS2	0.734163	0.117336	6.25691	3.93E-10	1.29E-06
TMEM70	0.594117	0.09883	6.011529	1.84E-09	3.70E-06
CHORDC1	0.699586	0.116432	6.00852	1.87E-09	3.70E-06
SRSF7	0.386678	0.065027	5.946443	2.74E-09	4.52E-06
TNFRSF12A	1.411121	0.239721	5.886518	3.94E-09	5.57E-06
SDCBP	0.640324	0.109344	5.856048	4.74E-09	5.86E-06
ARRDC3	-0.602893	0.105291	-5.72598	1.03E-08	1.13E-05
KDM6B	0.611342	0.112803	5.419565	5.97E-08	5.66E-05
ENSG00000273748	-1.210698	0.223786	-5.410064	6.30E-08	5.66E-05
RHOF	-0.500456	0.092829	-5.391149	7.00E-08	5.77E-05
ENSG00000130313	-0.417909	0.079602	-5.249987	1.52E-07	0.000115674
DUSP14	0.72054	0.140978	5.111018	3.20E-07	0.000226268
RIN3	1.240795	0.245838	5.04721	4.48E-07	0.000281731
HNRNPH3	0.398148	0.078935	5.043971	4.56E-07	0.000281731
ALOX5	-0.588493	0.117962	-4.988837	6.07E-07	0.000353243
FLYWCH2	-0.55958	0.112548	-4.971946	6.63E-07	0.000364047
IZUMO4	-0.729663	0.147306	-4.95338	7.29E-07	0.000379493
PIGX	-0.830526	0.168058	-4.941902	7.74E-07	0.000382411
ENSG00000254614	-1.593047	0.323019	-4.931746	8.15E-07	0.000383661
PSME1	-0.249706	0.051184	-4.878609	1.07E-06	0.000480084
MACROD2	-1.300687	0.268543	-4.843488	1.28E-06	0.000536692
MRPL39	0.486389	0.100541	4.837707	1.31E-06	0.000536692
GATD3	5.27088	1.091011	4.831189	1.36E-06	0.000536692
RDX	0.529223	0.109924	4.814448	1.48E-06	0.000561249
TTC32	-0.768015	0.161168	-4.765318	1.89E-06	0.000690393
ENSG00000272211	-1.296262	0.273841	-4.733635	2.21E-06	0.000778641
PFKL	-0.458855	0.097263	-4.71765	2.39E-06	0.000789912
HMOX1	1.756464	0.372393	4.716695	2.40E-06	0.000789912
ARL4C	-1.24735	0.266228	-4.685268	2.80E-06	0.000867381
CD48	0.506911	0.108373	4.677459	2.90E-06	0.000867381
HSPH1	0.782082	0.167249	4.676141	2.92E-06	0.000867381
TRADD	-0.488355	0.104528	-4.67198	2.98E-06	0.000867381
GALNT2	-0.762319	0.164446	-4.635677	3.56E-06	0.0010049
LMO4	0.813322	0.179415	4.533195	5.81E-06	0.001559153
C17orf49	-0.730199	0.161111	-4.532268	5.84E-06	0.001559153
POU2F2	0.402547	0.089293	4.508146	6.54E-06	0.001668709
RFTN1	-0.541688	0.120294	-4.503046	6.70E-06	0.001668709
FCRL5	1.556078	0.34569	4.501366	6.75E-06	0.001668709
MIR181A1HG	-1.029829	0.229113	-4.494856	6.96E-06	0.001678617
SEC14L1	-0.455058	0.101435	-4.486218	7.25E-06	0.001706479
BHLHE41	1.486264	0.332854	4.465207	8.00E-06	0.001839059
BRIX1	0.487664	0.109992	4.43364	9.27E-06	0.002081801
UPP1	0.624087	0.140979	4.426807	9.56E-06	0.002101065
SLCO4A1	1.048956	0.238372	4.400497	1.08E-05	0.002321131

SIPA1L3	-0.66239	0.150891	-4.389863	1.13E-05	0.002364021
GNPDA1	1.305779	0.297718	4.385957	1.15E-05	0.002364021
UTP4	0.643434	0.146809	4.382783	1.17E-05	0.002364021
DIRAS1	-1.094427	0.250166	-4.374807	1.22E-05	0.002403084
GTF2IRD2	-0.611267	0.1401	-4.363086	1.28E-05	0.002485857
PPIL4	0.498588	0.114766	4.344399	1.40E-05	0.002604967
FADS1	-0.986989	0.227201	-4.344112	1.40E-05	0.002604967
SOCS3	1.304265	0.300501	4.340297	1.42E-05	0.002604967
NR4A3	1.020945	0.235658	4.332322	1.48E-05	0.002652048
POLR2J	-0.283007	0.06558	-4.31543	1.59E-05	0.002731389
ENSG00000272831	-0.681885	0.158173	-4.311003	1.63E-05	0.002731389
GTF2B	0.564457	0.130951	4.310448	1.63E-05	0.002731389
KDM4B	0.557955	0.12946	4.309872	1.63E-05	0.002731389
GNG11	1.953506	0.453606	4.306613	1.66E-05	0.002731389
NR4A1	1.072268	0.249681	4.294555	1.75E-05	0.00283686
TAF1A	0.742445	0.173161	4.287593	1.81E-05	0.002843904
RHEB	0.33249	0.077621	4.283495	1.84E-05	0.002843904
TCL1B	-2.212826	0.516612	-4.28334	1.84E-05	0.002843904
CMTM7	-0.767365	0.179637	-4.271764	1.94E-05	0.002905385
C2orf68	-0.423452	0.099129	-4.271725	1.94E-05	0.002905385
MED21	0.435666	0.102441	4.25283	2.11E-05	0.003114621
ASF1A	0.564089	0.134735	4.186671	2.83E-05	0.004106238
SRSF11	0.27241	0.06511	4.183862	2.87E-05	0.004106238
DNTTIP2	0.368259	0.088709	4.151335	3.31E-05	0.004668201
PHKG1	-0.779234	0.188066	-4.143414	3.42E-05	0.004684519
HSPD1	0.668359	0.161329	4.142825	3.43E-05	0.004684519
AK6	0.42589	0.102852	4.140802	3.46E-05	0.004684519
KBTBD8	1.100314	0.265918	4.1378	3.51E-05	0.004684519
DNAJB9	0.442506	0.108046	4.095545	4.21E-05	0.005487885
ATF3	0.696111	0.169984	4.095153	4.22E-05	0.005487885
CALM3	-0.413642	0.101244	-4.085609	4.40E-05	0.005644192
TNFRSF1B	0.916502	0.224657	4.079558	4.51E-05	0.00571886
PPIF	0.671223	0.164852	4.071674	4.67E-05	0.005782479
KLF10	0.723687	0.177762	4.071092	4.68E-05	0.005782479
TSPAN33	-0.518677	0.127767	-4.059535	4.92E-05	0.00600124
UCP2	-0.416064	0.102741	-4.04965	5.13E-05	0.006067688
ZNRD1ASP	-0.764972	0.188936	-4.048843	5.15E-05	0.006067688
DUSP22	-0.478055	0.118083	-4.048457	5.16E-05	0.006067688
SPAG9	0.520383	0.12888	4.037717	5.40E-05	0.006264011
HDAC2	0.420517	0.104205	4.035476	5.45E-05	0.006264011
NAF1	0.696883	0.173411	4.018678	5.85E-05	0.006626298
RAB8B	0.426532	0.106186	4.01684	5.90E-05	0.006626298
PTGIR	-1.621316	0.403962	-4.013538	5.98E-05	0.006644205
C22orf39	-0.368901	0.092102	-4.005364	6.19E-05	0.006801786
MFSD10	-0.315581	0.078939	-3.997784	6.39E-05	0.006875458
HSPE1	0.434901	0.10879	3.997616	6.40E-05	0.006875458
LINC02256	-0.737371	0.184864	-3.988716	6.64E-05	0.00695771

ADD3	0.66307	0.166336	3.986319	6.71E-05	0.00695771
SLC9A7	-0.599321	0.15052	-3.981684	6.84E-05	0.00695771
FKBP5	0.842042	0.211505	3.98119	6.86E-05	0.00695771
WBP1L	-0.632431	0.158859	-3.981076	6.86E-05	0.00695771
CYB5A	-0.72591	0.182398	-3.979805	6.90E-05	0.00695771
ZBTB10	0.507817	0.127805	3.973363	7.09E-05	0.007076457
FKBP4	0.467207	0.118047	3.957796	7.56E-05	0.007477743
IKZF2	-1.174491	0.296937	-3.955349	7.64E-05	0.007477743
MREG	-0.827408	0.209307	-3.953077	7.72E-05	0.007477743
HHIP.AS1	-3.01619	0.764353	-3.946071	7.94E-05	NA
SPHK1	0.976011	0.247709	3.940149	8.14E-05	0.007815796
NCBP3	0.973206	0.247359	3.934385	8.34E-05	0.007906437
LDLRAD4	0.825748	0.210023	3.931704	8.43E-05	0.007906437
DDX21	0.563809	0.143445	3.930485	8.48E-05	0.007906437
PNPLA6	-0.671466	0.171039	-3.925796	8.64E-05	0.007948085
BRK1	-0.247383	0.063032	-3.924724	8.68E-05	0.007948085
IGFBP4	-0.870549	0.222294	-3.916213	9.00E-05	0.008158297
SLC8B1	-0.974201	0.249295	-3.907826	9.31E-05	0.00836988
MPHOSPH10	0.408308	0.10459	3.903899	9.47E-05	0.008430274
SRSF5	-0.339977	0.08737	-3.891237	9.97E-05	0.008652686
ENSG00000237181	0.853165	0.219318	3.890077	0.000100213	0.008652686
ARNTL2	-1.118711	0.287736	-3.887982	0.000101081	0.008652686
BUD23	0.380294	0.097901	3.884484	0.000102547	0.008652686
MRPL44	0.418568	0.107788	3.883244	0.000103072	0.008652686
EIF5	0.335682	0.086451	3.882894	0.00010322	0.008652686
HOMER2	-1.03943	0.267744	-3.882177	0.000103526	0.008652686
HIST1H4C	0.480682	0.123904	3.879464	0.000104687	0.008652686
SGPL1	-0.473697	0.122129	-3.878669	0.00010503	0.008652686
NFE2L2	0.320994	0.082971	3.868761	0.00010939	0.008937418
NDUFA7	-0.441474	0.114345	-3.860877	0.000112981	0.009155148
SMIM10	-3.542224	0.918152	-3.857994	0.000114321	NA
COMMD2	-0.34333	0.089115	-3.85266	0.000116842	0.009391039
C16orf74	-0.742968	0.193033	-3.84892	0.00011864	0.009458667
PPM1F	-0.801184	0.208463	-3.843288	0.000121397	0.009601027
ENSG00000267002	-0.595631	0.155071	-3.841026	0.000122521	0.009613034
MAP2K3	0.282386	0.07356	3.838858	0.000123608	0.009621937
FNBP1	-0.300945	0.078446	-3.836358	0.000124872	0.009644448
CCDC115	-0.388487	0.101412	-3.830769	0.000127743	0.009789707
IL32	-4.463641	1.166944	-3.82507	0.000130735	NA
PDE6D	-0.554787	0.145111	-3.823187	0.000131738	0.01001816
SLC7A6	0.77807	0.203619	3.821197	0.000132805	0.010022242
SNHG30	-0.510018	0.13367	-3.815507	0.000135903	0.010178345
NFKBIE	0.48957	0.12866	3.805153	0.000141717	0.010474609
DMAC2L	-0.473061	0.124336	-3.804696	0.000141978	0.010474609
GLS	0.441147	0.116473	3.787558	0.000152135	0.011140783
LRRC7	-1.34822	0.356164	-3.785394	0.000153465	0.011155571
CLECL1	0.921328	0.24429	3.771454	0.000162299	0.011711587

SEPTIN9	-0.324157	0.086078	-3.765849	0.000165984	0.011890706
CDC42	-0.255972	0.068113	-3.758019	0.000171264	0.012116529
SUPV3L1	0.488269	0.129952	3.757314	0.000171747	0.012116529
BLVRB	0.903571	0.240582	3.755765	0.000172813	0.012116529
NPW	2.367068	0.630913	3.751811	0.000175562	NA
NUAK2	-0.519425	0.138455	-3.751564	0.000175735	0.012234593
DDX24	0.250263	0.06697	3.73693	0.00018628	0.012878102
CCT8	0.383085	0.102607	3.733512	0.000188828	0.012963556
PER2	0.72825	0.195617	3.722845	0.00019699	0.013430658
SERTAD3	0.495462	0.13321	3.719407	0.000199691	0.013521553
PPM1D	0.587289	0.15818	3.712796	0.000204982	0.013785371
CDC42EP3	0.752321	0.203027	3.705518	0.00021096	0.014091527
KLHL2	-0.693865	0.187502	-3.700566	0.000215119	0.014156218
ENSG00000055483	0.733255	0.198198	3.699606	0.000215935	0.014156218
BTLA	-0.694274	0.187679	-3.699266	0.000216224	0.014156218
MYO1C	-0.489504	0.132524	-3.693698	0.000221016	0.014374796
COMMD3	-0.373967	0.101508	-3.6841	0.000229512	0.014829801
B3GNT7	0.684245	0.186015	3.678441	0.000234664	0.015064221
C12orf29	0.461219	0.125471	3.675915	0.000236999	0.015115938
VPS28	-0.281936	0.076754	-3.67322	0.000239513	0.01517836
EGR3	1.586717	0.432478	3.668899	0.000243597	0.01526504
GADD45A	0.893827	0.243649	3.668509	0.000243969	0.01526504
COQ10B	0.315722	0.08615	3.664784	0.000247548	0.015391546
CLEC2D	-0.451514	0.123362	-3.660069	0.000252147	0.015579539
CD5	-2.278785	0.622824	-3.658793	0.000253406	NA
XRRA1	-1.08298	0.296433	-3.653374	0.000258816	0.015892287
GLRX2	0.456387	0.125056	3.649451	0.000262801	0.016037353
PRAG1	1.710855	0.46922	3.64617	0.000266178	0.016134923
PUS7	0.75419	0.206926	3.644738	0.000267664	0.016134923
ENSG00000283013	-0.676218	0.185723	-3.640999	0.000271582	0.016201082
ENSG00000224505	-0.789046	0.216875	-3.63826	0.000274486	0.016201082
ESF1	0.320033	0.088	3.636722	0.00027613	0.016201082
SORL1	-0.542158	0.149135	-3.635358	0.000277595	0.016201082
COMMD8	-0.350362	0.096387	-3.634967	0.000278016	0.016201082
DHX57	-0.867011	0.238555	-3.634431	0.000278594	0.016201082
ENSG00000277654	-0.770691	0.212343	-3.629471	0.000284002	0.016337385
C1GALT1	0.48618	0.134027	3.627473	0.000286209	0.016337385
CDC37L1	0.449611	0.123975	3.626622	0.000287153	0.016337385
AKIRIN1	0.291757	0.080457	3.626267	0.000287549	0.016337385
NDUFB10	-0.282556	0.078016	-3.621782	0.00029258	0.016475267
ENSG00000257275	-0.982007	0.27131	-3.619498	0.000295175	0.016475267
SNX29	-0.66782	0.184532	-3.618991	0.000295754	0.016475267
TERF2IP	0.339884	0.093937	3.618215	0.000296641	0.016475267
PEX6	1.354965	0.374752	3.615626	0.000299623	0.016547873
CYREN	-0.382263	0.105887	-3.610115	0.000306061	0.016803429
TYW3	0.552146	0.153001	3.608772	0.000307649	0.016803429
LCN8	3.870969	1.072845	3.608136	0.000308405	NA

HMGXB4	-0.350484	0.097223	-3.604971	0.000312188	0.016888766
GNL3	0.485154	0.134593	3.604605	0.000312628	0.016888766
BLOC1S1	-0.319289	0.088778	-3.596473	0.000322561	0.017298732
ABTB1	-0.46612	0.129732	-3.592957	0.000326947	0.017298732
RRN3	0.450646	0.125425	3.592953	0.000326952	0.017298732
FCRL4	1.866408	0.519494	3.592742	0.000327217	0.017298732
ADGRE5	0.50164	0.139887	3.586035	0.000335744	0.017655159
MXD1	0.724635	0.202191	3.583917	0.000338479	0.017704798
RCL1	0.571303	0.160129	3.567772	0.00036003	0.018662088
TTPAL	-0.437013	0.122502	-3.567389	0.000360556	0.018662088
JMJD6	0.38856	0.109021	3.564072	0.000365145	0.018801188
YWHAE	0.346754	0.097386	3.560629	0.000369967	0.018950754
PILRB	0.694769	0.195277	3.55787	0.000373874	0.019052178
EIF3A	0.293979	0.082811	3.549988	0.000385248	0.019531104
CCDC28B	-0.587041	0.165665	-3.54355	0.000394779	0.019837359
C15orf65	1.904351	0.537795	3.541035	0.00039856	0.019837359
NRDE2	-0.460799	0.130138	-3.540849	0.000398841	0.019837359
NOP56	0.439343	0.124089	3.540536	0.000399316	0.019837359
NDUFAF4	0.55782	0.157828	3.534363	0.00040876	0.020191321
FOSL1	1.748144	0.494773	3.533223	0.000410526	0.020191321
PRMT5	0.480171	0.136187	3.525831	0.000422157	0.020660591
ENSG00000273015	-0.444634	0.126354	-3.518963	0.000433237	0.021069704
CEP95	0.455904	0.129591	3.518021	0.000434778	0.021069704
RFK	0.412542	0.117535	3.509963	0.000448168	0.021612647
EIPR1	-0.461538	0.131549	-3.508486	0.000450665	0.021627543
PRMT3	0.87902	0.250795	3.504938	0.000456714	0.021645685
CHRNB1	-0.591314	0.168716	-3.504786	0.000456974	0.021645685
TSTD1	-0.486489	0.138822	-3.504415	0.000457612	0.021645685
HSP90AA1	0.326743	0.093291	3.502401	0.000461085	0.021706105
NFRKB	-0.615612	0.175864	-3.500502	0.000464383	0.021757763
H3F3B	0.21481	0.061444	3.496023	0.000472249	0.021944933
NUCB2	-0.920566	0.263342	-3.495702	0.000472817	0.021944933
BYSL	0.874043	0.250316	3.491762	0.000479846	0.022167088
IGHD	-0.66644	0.191637	-3.477607	0.000505912	0.023262524
SLC1A5	0.674009	0.194041	3.473538	0.000513644	0.023451031
BAG2	0.564351	0.162537	3.472132	0.000516341	0.023451031
COMMD10	0.722497	0.208109	3.471724	0.000517128	0.023451031
DNAJB4	0.618887	0.178428	3.468547	0.00052328	0.023621688
LRRC61	-1.038709	0.299999	-3.462374	0.000535432	0.024060374
FAM91A1	0.491471	0.142085	3.458994	0.000542196	0.024184367
HLX	1.115258	0.322523	3.457915	0.000544372	0.024184367
GYG1	0.583577	0.168816	3.456885	0.000546458	0.024184367
CXCR3	2.251813	0.651649	3.455562	0.000549146	0.024184367
SLAMF7	1.083111	0.313497	3.454936	0.000550423	0.024184367
ENSG00000229043	-1.680558	0.48645	-3.45474	0.000550824	NA
TMEM273	1.475328	0.427213	3.45338	0.000553608	0.024216682
HSPA8	0.485957	0.140771	3.452108	0.000556225	0.024223974

USE1	-0.296884	0.086133	-3.446806	0.000567255	0.024588172
ATF6B	-0.310929	0.090236	-3.44571	0.000569562	0.024588172
COX4I1	-0.181102	0.052584	-3.444063	0.000573043	0.024630875
KRT10	-0.212414	0.061922	-3.43037	0.000602759	0.025691031
TTC19	-0.366355	0.106811	-3.429917	0.000603767	0.025691031
RB1	-0.361462	0.105409	-3.429137	0.000605504	0.025691031
PDPR	-0.924242	0.269646	-3.427609	0.000608921	0.025725614
MRPL28	-0.279958	0.081816	-3.421811	0.000622055	0.026126205
MIDN	0.440615	0.128845	3.419717	0.000626863	0.026126205
ZBED1	-0.428249	0.125244	-3.419327	0.000627762	0.026126205
ERGIC1	-0.609334	0.17823	-3.418802	0.000628974	0.026126205
RNGTT	-0.512928	0.150255	-3.413709	0.00064085	0.026481618
UROD	-0.398973	0.116903	-3.412844	0.000642888	0.026481618
CENPH	-0.644743	0.189429	-3.403608	0.00066502	0.027173437
SMIM27	-0.403514	0.118558	-3.40351	0.000665261	0.027173437
MPP6	0.647434	0.190286	3.402416	0.000667929	0.027173437
ZNF627	-0.706108	0.207965	-3.395323	0.000685478	0.027773088
WDR13	-0.342481	0.100976	-3.391721	0.00069455	0.02801477
NIFK	0.555046	0.163696	3.390713	0.00069711	0.02801477
KLF6	0.310981	0.091747	3.389538	0.000700104	0.028021176
TFB2M	0.601725	0.177911	3.38217	0.000719155	0.028667625
EPS15	-0.439301	0.130001	-3.379222	0.000726913	0.028756964
RAB10	0.306805	0.090795	3.379089	0.000727264	0.028756964
ORMDL3	-0.534946	0.158361	-3.37801	0.000730123	0.028756964
ALOX5AP	-0.724149	0.214465	-3.376535	0.000734051	0.028796951
TDG	0.343715	0.101867	3.374146	0.00074045	0.028933162
GNL2	0.56509	0.167681	3.370042	0.000751568	0.029104937
USF1	-1.035801	0.307448	-3.369023	0.000754352	0.029104937
ENSG00000275964	-1.026401	0.304739	-3.368129	0.000756803	0.029104937
RPF2	0.493952	0.146664	3.367907	0.000757412	0.029104937
LAMTOR4	-0.288598	0.085711	-3.367124	0.000759566	0.029104937
TEX30	0.416212	0.123669	3.365527	0.000763976	0.029160887
NMT2	-0.729419	0.217101	-3.359818	0.000779939	0.02961263
TAGLN2	-0.307227	0.091459	-3.359158	0.000781802	0.02961263
HDDC2	-0.401636	0.119721	-3.354776	0.000794292	0.029970877
PRAF2	-0.505899	0.151111	-3.347872	0.000814346	0.030610731
EIF4A3	0.439458	0.131369	3.345205	0.000822217	0.030789527
ARL8B	0.413481	0.123685	3.34302	0.000828719	0.030857504
OXLD1	-0.402846	0.120524	-3.342468	0.000830368	0.030857504
STIP1	0.410878	0.122985	3.340872	0.000835158	0.030857504
METTL26	-0.358694	0.10738	-3.34042	0.000836517	0.030857504
NABP1	0.413595	0.123987	3.335801	0.000850541	0.031193507
UGT8	0.926004	0.27772	3.334306	0.000855125	0.031193507
ENSG00000259321	-0.796999	0.239153	-3.332593	0.000860407	0.031193507
NEDD4L	1.750494	0.525278	3.332509	0.000860667	0.031193507
TXN	0.378739	0.11368	3.33164	0.000863358	0.031193507
NR1D2	0.651331	0.195552	3.33073	0.000866187	0.031193507

PFKFB3	-0.461751	0.138654	-3.330239	0.000867713	0.031193507
EEA1	0.406694	0.122288	3.325715	0.00088192	0.031544912
TXLNG	0.494112	0.148601	3.325099	0.00088387	0.031544912
DBI	-0.41222	0.124034	-3.323449	0.000889118	0.031618051
SLC50A1	-0.374451	0.112718	-3.322023	0.000893672	0.031666114
MED23	-0.618517	0.18628	-3.32036	0.000899016	0.031741683
NDE1	-0.408583	0.123263	-3.314718	0.000917355	0.032273933
RBM25	0.250439	0.075616	3.311994	0.000926334	0.032474255
SLC35F6	-0.540641	0.163615	-3.304347	0.000951978	0.033150549
IGKC	-0.437217	0.132324	-3.304148	0.000952656	0.033150549
ELOVL5	0.311669	0.094352	3.303258	0.000955685	0.033150549
TBC1D2B	-0.91796	0.278332	-3.298081	0.00097348	0.033346802
IGKV3.11	-1.313108	0.398171	-3.297853	0.000974273	0.033346802
MME	-3.059808	0.927958	-3.297358	0.000975991	NA
NCBP2AS2	-0.333481	0.101158	-3.296631	0.00097852	0.033346802
AKAP1	0.735645	0.223187	3.296092	0.000980398	0.033346802
DESI1	0.457862	0.138922	3.295827	0.000981323	0.033346802
NHLRC3	-0.542112	0.164488	-3.295753	0.000981582	0.033346802
HDAC1	-0.34021	0.103291	-3.293696	0.000988794	0.033392489
SMARCE1	-0.257639	0.078228	-3.293443	0.000989682	0.033392489
IL13RA1	-0.516074	0.157287	-3.281107	0.001034005	0.034762575
RAB1A	0.342263	0.104342	3.280204	0.001037321	0.034762575
LSM3	-0.179841	0.054954	-3.27258	0.001065708	0.03544463
ENSG00000273272	2.400283	0.733847	3.270821	0.001072357	0.03544463
PIKFYVE	0.489691	0.149718	3.270757	0.0010726	0.03544463
ZPR1	0.351946	0.107604	3.270739	0.001072669	0.03544463
ALG12	-0.602471	0.184244	-3.269967	0.001075601	0.03544463
CNOT6L	-0.572825	0.175465	-3.264611	0.001096146	0.03600166
PEX13	0.572294	0.175387	3.263044	0.001102224	0.036081409
LPIN1	0.61914	0.189905	3.260269	0.001113067	0.036316113
SYNGR2	-0.309925	0.095151	-3.257209	0.001125135	0.036589108
MTMR4	-0.596503	0.183267	-3.254826	0.001134617	0.036776474
ERV3.1	-0.373353	0.114788	-3.252549	0.001143747	0.036928072
ENSG00000279483	-0.393798	0.121102	-3.2518	0.001146765	0.036928072
HNRNPA2B1	0.233781	0.071936	3.249843	0.001154687	0.037009148
IER5	0.348513	0.107257	3.249331	0.00115677	0.037009148
COA7	0.775145	0.238669	3.247788	0.00116306	0.037090372
COPS3	0.395851	0.122002	3.244631	0.001176029	0.037339413
NOP16	0.476449	0.146869	3.244052	0.001178424	0.037339413
STRBP	-0.322054	0.099338	-3.241989	0.001186985	0.037441768
FLCN	0.605237	0.186718	3.241451	0.001189229	0.037441768
EIF2AK4	-0.541861	0.167304	-3.23878	0.001200422	0.037674185
FAM30A	-0.381707	0.117979	-3.235385	0.001214787	0.038004374
CDK5R1	2.59867	0.803798	3.232987	0.001225031	NA
PTPN22	0.88927	0.27521	3.231242	0.001232534	0.038332738
VAMP8	-0.226835	0.070203	-3.231126	0.001233038	0.038332738
SVBP	-0.326746	0.101228	-3.227821	0.001247371	0.038564219

POLR3D	0.534481	0.165601	3.227527	0.001248654	0.038564219
VDAC1	0.424042	0.13142	3.226624	0.001252599	0.038564219
ZDHHC12	-0.382687	0.118664	-3.224958	0.00125991	0.038564219
NDUFB7	-0.348012	0.107942	-3.224067	0.001263838	0.038564219
ERF	0.613262	0.190241	3.223611	0.001265853	0.038564219
TNFRSF10B	0.392048	0.121634	3.223173	0.00126779	0.038564219
ARL6IP1	0.255948	0.079448	3.221585	0.001274838	0.038659645
DYNLL1	0.341547	0.106118	3.218568	0.001288326	0.038865253
CTNND1	-0.666803	0.20719	-3.218311	0.00128948	0.038865253
ENSG00000224610	-2.788744	0.867077	-3.21626	0.001298731	NA
ARCN1	0.45143	0.14067	3.209137	0.00133134	0.039669698
H1FX	-0.852359	0.265655	-3.208521	0.001334196	0.039669698
P2RY8	-0.499522	0.155718	-3.207862	0.001337259	0.039669698
AKIRIN2	0.271007	0.084503	3.207057	0.001341006	0.039669698
SNX17	-0.237403	0.07403	-3.206855	0.001341948	0.039669698
KRAS	0.263672	0.082225	3.206702	0.001342662	0.039669698
SLC35B2	0.753584	0.235028	3.206359	0.001344259	0.039669698
TMEM219	-0.334469	0.104475	-3.201436	0.001367447	0.040132837
GLA	0.715302	0.223441	3.201304	0.001368073	0.040132837
SCRN1	0.995163	0.311043	3.199437	0.001376963	0.040274119
CCDC71	-0.477753	0.14962	-3.193109	0.0014075	0.040925833
SLC16A3	0.641545	0.200916	3.193104	0.001407524	0.040925833
C15orf62	-0.863952	0.27078	-3.190607	0.001419743	0.041160047
RCC1	0.379239	0.119014	3.186516	0.001439974	0.041624513
ENSG00000225205	-1.118996	0.351378	-3.184589	0.001449595	0.041780451
NOP58	0.36613	0.115005	3.183586	0.001454631	0.041803714
ARF5	-0.414133	0.130193	-3.180906	0.001468155	0.042070086
SNHG25	-0.660432	0.207843	-3.177552	0.001485239	0.042350325
PYGO2	-0.421619	0.132709	-3.177008	0.001488028	0.042350325
ZNF605	-0.891716	0.280743	-3.176269	0.001491823	0.042350325
SLC6A6	-0.375541	0.118257	-3.175639	0.00149507	0.042350325
NAMPT	0.645435	0.203405	3.173159	0.001507897	0.04252758
ATP5IF1	-0.394101	0.124214	-3.172768	0.001509931	0.04252758
BDP1	0.227355	0.071715	3.170282	0.001522909	0.042771256
DDAH2	0.441207	0.139439	3.164161	0.001555307	0.04349661
ODF3B	-0.582021	0.183968	-3.163711	0.001557713	0.04349661
PPP2R2A	0.337608	0.106754	3.162474	0.001564349	0.04349661
ATP1A1	0.470394	0.14876	3.162104	0.001566336	0.04349661
CYBC1	-0.287712	0.091133	-3.157074	0.001593607	0.044075452
MOB4	0.276373	0.087553	3.156619	0.001596097	0.044075452
ITGAX	2.297958	0.728047	3.156333	0.001597663	NA
MDK	-1.083175	0.343258	-3.155574	0.001601828	0.044110516
RPP25	0.83756	0.265612	3.153324	0.001614224	0.044328386
LARP4	0.466593	0.148064	3.151288	0.001625521	0.044514965
MIS12	0.38037	0.120778	3.149327	0.001636469	0.044690965
DHX15	0.348862	0.110845	3.147309	0.001647806	0.04475536
SYNC	-0.484734	0.154016	-3.147296	0.001647881	0.04475536

WDR33	0.340708	0.108331	3.145068	0.00166048	0.044870024
COL9A3	-1.767209	0.561921	-3.144945	0.00166118	0.044870024
ZMAT2	-0.230714	0.073398	-3.14334	0.001670319	0.044993946
CHD1	0.296982	0.094715	3.13555	0.001715323	0.046080659
ENSG00000278834	-1.455694	0.465069	-3.130061	0.001747699	NA
TLN1	-0.316443	0.101143	-3.12867	0.001755994	0.047045418
RNF168	-0.434804	0.139113	-3.125541	0.001774781	0.047350316
RSL1D1	0.335578	0.107379	3.125181	0.001776954	0.047350316
TEX10	0.460703	0.147707	3.119027	0.001814495	0.048220683
ADAMTS6	1.72907	0.554428	3.118653	0.001816796	NA
C1orf122	-0.390939	0.125582	-3.113011	0.001851893	0.048985376
HLA.G	-1.671651	0.537039	-3.112717	0.001853736	0.048985376
ACTR1B	-0.460377	0.147946	-3.111796	0.001859528	0.048985376
PPCS	-0.269473	0.086632	-3.110565	0.001867298	0.048985376
ZMAT3	-0.494285	0.158911	-3.110447	0.001868044	0.048985376
NCL	0.339941	0.109485	3.104898	0.001903447	0.049781686
ZNF428	-0.311832	0.100523	-3.102102	0.00192152	0.050121767
FANCB	-0.869468	0.280523	-3.099458	0.001938753	0.050359414
ADSS	0.378201	0.122034	3.099142	0.001940819	0.050359414
UQCR10	-0.175256	0.056623	-3.095171	0.001966994	0.050784457
TAF6	-0.407319	0.131601	-3.095099	0.001967474	0.050784457
GEMIN4	0.644821	0.208756	3.088874	0.002009164	0.051501488
STAT3	0.329695	0.106785	3.087473	0.00201866	0.051501488
PRPF38B	0.203355	0.065865	3.087459	0.002018757	0.051501488
RNF26	-0.441348	0.142955	-3.087332	0.002019622	0.051501488
AGTRAP	-0.728439	0.235963	-3.087085	0.002021301	0.051501488
RFFL	-0.348922	0.113141	-3.08396	0.00204265	0.051792051
PUS1	0.475695	0.15428	3.083323	0.002047027	0.051792051
FIBP	-0.28184	0.091414	-3.083121	0.002048421	0.051792051
DCTN1	-0.457377	0.148417	-3.081711	0.002058147	0.051905201
TMEM123	-0.403772	0.13107	-3.080589	0.002065913	0.051968484
RCHY1	0.325138	0.105604	3.078835	0.002078121	0.05198793
UTP6	0.368739	0.119774	3.078619	0.002079621	0.05198793
DNAJA2	0.28549	0.092749	3.078087	0.002083339	0.05198793
APBB2	-1.510385	0.490789	-3.077461	0.002087721	0.05198793
MOB3A	-0.293442	0.095383	-3.07647	0.00209467	0.05202993
DDX6	-0.259188	0.084273	-3.075585	0.002100902	0.052053921
IQSEC1	-0.378106	0.123	-3.074046	0.002111769	0.052192372
EIF4E	0.37061	0.120631	3.072254	0.002124487	0.052310772
AP2M1	-0.23329	0.075944	-3.071881	0.002127142	0.052310772
NFAT5	0.424549	0.138336	3.068965	0.002148019	0.052685745
HSP90AB1	0.448568	0.14623	3.067559	0.002158146	0.052685745
CHCHD5	-0.463726	0.151193	-3.067106	0.002161422	0.052685745
IVNS1ABP	0.508949	0.165955	3.06679	0.002163708	0.052685745
TFEC	2.378492	0.775583	3.066714	NA	NA
GCSAM	-0.918239	0.299668	-3.064192	0.002182591	0.053014973
JTB	-0.193223	0.063076	-3.063353	0.00218872	0.053033552

ENSG00000273156	-1.148535	0.376102	-3.053786	0.00225973	0.05462027
HCG18	0.388026	0.12713	3.052201	0.002271702	0.05477573
RAB11B	-0.265055	0.086866	-3.051322	0.002278362	0.054802646
ALKBH4	-0.470894	0.154419	-3.049449	0.002292617	0.055011684
QPCT	0.794921	0.260762	3.048448	0.002300265	0.055061556
UPF2	-0.221209	0.072634	-3.045551	0.002322542	0.055432884
NECAP2	-0.231867	0.076147	-3.044976	0.002326992	0.055432884
HNRNPU	0.279532	0.091981	3.039006	0.002373602	0.056291085
RNF19A	0.517102	0.170161	3.038904	0.002374406	0.056291085
TXNL1	0.283732	0.093392	3.038081	0.002380899	0.056309969
FAM210A	0.335889	0.110598	3.037011	0.002389367	0.056375375
LINC01116	-0.589463	0.194328	-3.033336	0.002418663	0.05693071
NDUFB8	-0.293736	0.096931	-3.030356	0.002442658	0.057358947
APOBEC3D	-0.554345	0.183017	-3.028931	0.002454204	0.057493519
ENSG00000277969	0.599455	0.197976	3.027925	0.002462394	0.05754901
FLOT2	-0.409079	0.135313	-3.023214	0.002501055	0.058016699
ENSG00000230709	1.208414	0.399717	3.023171	0.002501405	0.058016699
KRTCAP2	-0.231463	0.076568	-3.022971	0.002503065	0.058016699
KCTD6	0.661969	0.219011	3.022542	0.00250661	0.058016699
BAG4	0.497818	0.164825	3.020277	0.002525436	0.058016699
PDE4D	-0.828446	0.274351	-3.019653	0.002530644	0.058016699
CDK4	0.353245	0.116994	3.019344	0.00253323	0.058016699
MYCBP2	0.227035	0.07521	3.018695	0.002538663	0.058016699
C19orf25	-0.227423	0.075344	-3.018444	0.002540765	0.058016699
MPPE1	-0.482281	0.15978	-3.018405	0.002541092	0.058016699
SNRPB2	0.273956	0.090822	3.016411	0.002557864	0.058265072
RGCC	2.043628	0.678184	3.013382	0.002583539	0.058714634
ICOSLG	-0.581677	0.193105	-3.012236	0.002593312	0.058801566
UQCR11	-0.187073	0.062126	-3.011206	0.002602121	0.05886629
RRP15	0.339734	0.112874	3.009859	0.002613688	0.058992965
NEDD8	-0.169892	0.056467	-3.008665	0.00262398	0.059090356
CLK1	0.396913	0.132029	3.006243	0.002644977	0.059251537
LSP1	-0.407743	0.135634	-3.006194	0.002645402	0.059251537
ATF7IP2	-0.475064	0.158051	-3.005767	0.002649118	0.059251537
TANK	0.272297	0.090712	3.001785	0.002684016	0.059804658
PCMTD2	-0.653287	0.217701	-3.000852	0.002692253	0.059804658
LINC00339	1.024025	0.341256	3.000756	0.002693102	0.059804658
BTN2A2	-0.46377	0.15458	-3.000198	0.002698045	0.059804658
TEAD2	-2.70088	0.900407	-2.999622	0.002703151	NA
ESRRA	0.47024	0.156811	2.99877	0.002710723	0.059951246
LYAR	0.520003	0.173564	2.996025	0.002735237	0.060358377
PTPRS	2.217846	0.74034	2.995713	0.002738042	NA
IRF2	-0.253658	0.084772	-2.992251	0.002769284	0.060967185
BIN1	0.651319	0.217722	2.991524	0.002775889	0.060967185
SNAP23	-0.369511	0.123552	-2.990719	0.002783219	0.060967185
RGS10	0.606567	0.202919	2.989202	0.002797068	0.060967185
DNAJC25	0.619816	0.207391	2.988636	0.002802258	0.060967185

NAA15	0.321976	0.107749	2.9882	0.002806263	0.060967185
WDR70	-0.341121	0.11418	-2.987575	0.002812002	0.060967185
CREBRF	0.334403	0.111943	2.987246	0.002815028	0.060967185
RRP9	0.552456	0.184961	2.986888	0.002818329	0.060967185
MIIP	-0.488848	0.163931	-2.982036	0.002863381	0.06180651
ZNF558	-0.898206	0.301422	-2.979891	0.002883512	0.061951821
FADS3	-0.485954	0.163102	-2.979453	0.002887632	0.061951821
MRPS30	0.309159	0.103783	2.978908	0.002892772	0.061951821
SRPRB	0.417839	0.140278	2.978654	0.002895179	0.061951821
CYTH1	-0.221695	0.074478	-2.976638	0.002914282	0.062124684
GID8	-0.285094	0.095795	-2.976095	0.002919443	0.062124684
CEP162	-0.505404	0.169837	-2.975815	0.00292211	0.062124684
DNAJB1	0.76453	0.257071	2.974001	0.002939442	0.062359071
NEU1	0.33784	0.113624	2.97331	0.002946063	0.062365692
HLA.DQB2	-3.149047	1.060136	-2.970419	0.002973937	NA
SNRNP48	0.412657	0.139004	2.968676	0.002990855	0.063165199
MAD2L2	-0.42312	0.142556	-2.968086	0.002996609	0.063165199
IDH2	-0.450589	0.15188	-2.966744	0.003009714	0.063240703
PSMB8.AS1	-0.379979	0.128107	-2.966096	0.003016061	0.063240703
LMBRD1	-0.274783	0.092652	-2.965758	0.003019382	0.063240703
NAPB	-0.554397	0.187555	-2.955917	0.003117411	0.065155859
ZNF79	0.7582	0.256755	2.953007	0.003146944	0.065458301
NTMT1	0.308503	0.104544	2.950925	0.003168235	0.065458301
EIF4A1	0.311876	0.105689	2.950879	0.003168707	0.065458301
SAR1B	0.312182	0.1058	2.95067	0.003170858	0.065458301
SERTAD1	0.393964	0.133572	2.949451	0.003183388	0.065458301
GEM	1.733493	0.58794	2.948419	0.003194033	0.065458301
GPR137	-0.293155	0.09943	-2.94836	0.003194653	0.065458301
PMAIP1	0.348358	0.118158	2.948241	0.003195876	0.065458301
GCH1	0.536203	0.181897	2.947835	0.003200078	0.065458301
EXOG	-0.464753	0.157668	-2.947665	0.003201837	0.065458301
CHTOP	0.192429	0.065296	2.947007	0.003208656	0.065458301
WASF1	-0.631796	0.214418	-2.946564	0.003213255	0.065458301
FAM53B	-0.697857	0.236874	-2.946112	0.003217958	0.065458301
NFATC2	0.650542	0.220897	2.945006	0.003229482	0.065557831
VAMP5	-0.716502	0.243403	-2.943692	0.003243222	0.065684398
RBM39	0.15217	0.051703	2.943141	0.003249006	0.065684398
ATXN1	0.988741	0.336073	2.942047	0.003260502	0.06578229
MIF	-0.353688	0.120276	-2.940641	0.003275342	0.065860191
PHLDA2	1.203851	0.409415	2.940419	0.003277687	0.065860191
VKORC1	-0.260467	0.088653	-2.938048	0.003302864	0.066231465
APH1A	-0.219315	0.074714	-2.935377	0.003331425	0.066627876
TXNDC11	0.417379	0.142211	2.934941	0.003336112	0.066627876
ATP5ME	-0.212985	0.072662	-2.931182	0.003376754	0.067303609
ARL5B	0.657384	0.224524	2.927903	0.003412563	0.067880488
ARID5A	-0.365629	0.125004	-2.92493	0.00344534	0.068394839
ANAPC15	-0.4625	0.158221	-2.92312	0.003465432	0.068655836

ADAMTS7	-0.555737	0.190273	-2.920741	0.003492	0.069043818
NHSL2	-0.457172	0.156591	-2.919529	0.003505603	0.069174434
ELL2	0.529804	0.181681	2.91612	0.003544145	0.069719585
MED4	-0.203253	0.069707	-2.915839	0.003547335	0.069719585
RAN	0.369069	0.126616	2.914877	0.003558282	0.06975689
TMOD2	-0.520623	0.178636	-2.914434	0.003563345	0.06975689
BCAR3	0.682126	0.234188	2.912725	0.003582894	0.069910761
CCM2	-0.260263	0.08936	-2.912512	0.003585349	0.069910761
GM2A	0.77757	0.267041	2.911799	0.003593538	0.069932517
ARID1A	0.343499	0.118072	2.909243	0.00362305	0.070368307
PHKB	-0.313624	0.107905	-2.906487	0.003655117	0.07079506
TMX2	0.311017	0.107021	2.906126	0.003659344	0.07079506
HEXIM1	0.436515	0.150286	2.904563	0.003677656	0.070804082
NPHP3	0.635524	0.218832	2.90417	0.003682279	0.070804082
GPN2	0.331638	0.114235	2.903121	0.003694641	0.070804082
PCIF1	-0.390841	0.13465	-2.902656	0.003700132	0.070804082
NAB2	0.724602	0.249691	2.901993	0.003707974	0.070804082
NRAS	-0.290094	0.099968	-2.901864	0.003709501	0.070804082
DUSP4	1.24312	0.428434	2.90154	0.003713329	0.070804082
MTRR	0.410652	0.141545	2.901222	0.003717107	0.070804082
CNIH1	0.269906	0.093092	2.89934	0.003739496	0.071093572
FADS2	-1.683043	0.581389	-2.894865	0.003793222	NA
FAM117B	-0.651686	0.225291	-2.892642	0.003820162	0.07248775
TELO2	-0.482583	0.167038	-2.889057	0.003863985	0.073178847
ACSL5	0.639744	0.221925	2.882697	0.003942866	0.074529964
CCDC86	0.507131	0.175963	2.882029	0.003951228	0.074545499
DEDD2	0.434284	0.150724	2.881327	0.003960045	0.074569528
SCOC	-0.25528	0.088627	-2.880395	0.003971775	0.074590121
FOSB	0.864587	0.300199	2.880041	0.003976228	0.074590121
VPS37A	0.416985	0.144905	2.87765	0.004006492	0.0750155
WDR3	0.654003	0.227746	2.871631	0.004083596	0.076314611
ICA1L	-0.594894	0.207212	-2.870942	0.004092503	0.076324809
FOXO3	0.530844	0.184938	2.870396	0.004099583	0.076324809
PHF21A	-0.437675	0.152686	-2.866509	0.004150258	0.07708713
THEMIS2	1.219728	0.425576	2.866062	0.004156124	0.07708713
PPP1CA	-0.184288	0.064321	-2.865133	0.004168337	0.077168871
RGMB	0.934051	0.326172	2.863678	0.004187531	0.077379309
ALKBH1	0.473921	0.165528	2.863087	0.004195359	0.077379324
DUSP10	0.650248	0.227225	2.861692	0.004213857	0.077575767
ZNF239	1.555979	0.543988	2.860317	0.004232173	NA
PRPF18	0.316758	0.110845	2.857661	0.004267758	0.07842203
NDUFB2	-0.206868	0.072429	-2.856152	0.004288103	0.0786497
ENSG00000159882	-0.451199	0.158026	-2.855221	0.004300692	0.078734512
LRP10	-0.271785	0.095232	-2.85393	0.004318198	0.078908881
ING1	0.437437	0.153312	2.853253	0.004327422	0.078931541
WARS2	0.562595	0.197383	2.850268	0.004368245	0.079529418
ELOC	0.264366	0.092818	2.848208	0.004396623	0.079898917

PVT1	0.65944	0.231803	2.844831	0.004443499	0.080602631
SMAP2	-0.272068	0.095665	-2.843956	0.004455713	0.080676158
ENOPH1	0.363002	0.127729	2.841975	0.004483494	0.081030758
IPO7	0.370151	0.130315	2.840432	0.004505247	0.081275313
ZNF773	0.603116	0.212393	2.839624	0.004516677	0.081290884
TAPBP	-0.294156	0.103623	-2.838723	0.004529442	0.081290884
TFG	0.344006	0.121231	2.837616	0.004545179	0.081290884
PLIN2	1.075425	0.379044	2.8372	0.004551105	0.081290884
PDE3B	-1.045072	0.368403	-2.836766	0.004557306	0.081290884
AKAP2	-0.911994	0.321554	-2.836205	0.004565307	0.081290884
CENPC	-0.331602	0.116938	-2.835707	0.004572432	0.081290884
DNAJA1	0.299999	0.105802	2.835487	0.004575588	0.081290884
SLC9A9	-0.762309	0.26891	-2.834811	0.004585284	0.081290884
ANP32A	-0.211992	0.074787	-2.834598	0.004588338	0.081290884
FUZ	-0.584742	0.206467	-2.832141	0.004623749	0.081771696
RPL7L1	0.243354	0.085982	2.830294	0.004650521	0.082098298
GPR65	0.614853	0.217286	2.829695	0.004659247	0.08210573
ENSG00000254397	1.210117	0.428194	2.826096	0.004711912	0.082861453
C15orf40	-0.30973	0.109628	-2.825283	0.004723889	0.082861453
SLC1A4	-0.78387	0.277471	-2.825053	0.004727277	0.082861453
NUDT2	-0.567116	0.200831	-2.823853	0.004745014	0.082877208
TAF4B	0.606491	0.214792	2.823613	0.004748574	0.082877208
MED17	0.287497	0.101858	2.82253	0.004764639	0.082877208
FAM184B	-1.89945	0.673259	-2.821276	0.004783296	0.082877208
ABI1	0.250594	0.088827	2.821125	0.004785549	0.082877208
SNHG9	-0.297303	0.105385	-2.821109	0.004785789	0.082877208
FABP5	0.622852	0.220816	2.820682	0.004792175	0.082877208
SLC2A5	-0.872799	0.309451	-2.820476	0.004795242	0.082877208
ENSG00000256940	-0.673614	0.238961	-2.818929	0.004818411	0.083034689
MRPL11	-0.210822	0.074793	-2.818747	0.004821152	0.083034689
HSPA1B	2.218248	0.787566	2.816586	0.004853701	0.083389307
C9orf16	-0.363381	0.12903	-2.816261	0.004858612	0.083389307
FTSJ1	0.410095	0.14571	2.81447	0.004885769	0.083603917
FAM53C	0.326594	0.116047	2.814322	0.00488803	0.083603917
C1orf52	0.272247	0.096792	2.812706	0.00491266	0.083768918
WDTC1	-0.408472	0.145231	-2.812577	0.004914624	0.083768918
NAA50	0.201961	0.071855	2.810678	0.004943726	0.084030849
VDAC2	0.245703	0.087427	2.810371	0.004948438	0.084030849
GTF3C6	-0.170288	0.060603	-2.809888	0.00495588	0.084030849
TAF1D	0.210482	0.074922	2.809361	0.004963991	0.084030849
TM2D3	0.40144	0.142936	2.808535	0.004976744	0.08410272
CABLES1	-1.093528	0.389769	-2.805579	0.005022632	0.084733339
VAPA	0.166715	0.059455	2.804043	0.005046621	0.084953554
SMC6	-0.25994	0.09272	-2.803485	0.005055362	0.084953554
SRBD1	-0.568067	0.202657	-2.803096	0.005061465	0.084953554
FIP1L1	0.238076	0.084964	2.80208	0.005077433	0.084960037
SURF6	0.277328	0.098989	2.801596	0.005085051	0.084960037

BORCS6	0.332316	0.118631	2.801259	0.005090358	0.084960037
ZC2HC1A	0.92669	0.330856	2.800888	0.005096227	0.084960037
TGIF2	0.29133	0.104049	2.799916	0.005111584	0.085005136
CEBPZ	0.290611	0.103814	2.799348	0.005120588	0.085005136
ABCF2	0.369309	0.131956	2.798725	0.005130486	0.085005136
IL4R	-0.263518	0.094162	-2.798546	0.005133327	0.085005136
MAML3	-1.367324	0.488761	-2.797529	0.005149518	0.085130661
PAXIP1.AS1	-0.482965	0.172772	-2.79538	0.005183869	0.085555468
FRY.AS1	-1.807199	0.64659	-2.79497	0.00519045	NA
ZEB1.AS1	-0.84766	0.303419	-2.793696	0.005210938	0.085825223
COASY	-0.321992	0.115294	-2.792804	0.005225332	0.085825223
UNC50	0.279436	0.100058	2.792747	0.005226258	0.085825223
ZNF24	0.240567	0.086196	2.790929	0.005255705	0.086165677
IER5L	2.356979	0.845089	2.78903	0.005286622	NA
ATP13A3	0.606864	0.217658	2.788157	0.005300889	0.086711475
ZNRD2	-0.301882	0.108286	-2.787812	0.005306539	0.086711475
C12orf60	1.052553	0.377742	2.786437	0.005329104	0.0869365
TVP23A	1.230851	0.44198	2.784854	0.005355186	0.087123914
RPL22L1	0.471179	0.169205	2.78467	0.005358218	0.087123914
GATM	1.714832	0.616043	2.783622	0.005375558	NA
USP10	0.280527	0.10085	2.781619	0.005408849	0.087709605
PAGR1	-0.536689	0.192954	-2.781431	0.005411983	0.087709605
ZNF670	0.606432	0.218313	2.777814	0.005472587	0.088546639
RUBCNL	-0.442257	0.159299	-2.776265	0.005498738	0.088824389
CARD8	-0.468791	0.169009	-2.773757	0.00554131	0.08936605
ZNF654	0.517065	0.186452	2.773183	0.005551087	0.089377917
PSMA5	0.257746	0.092989	2.771792	0.005574864	0.089614811
MAPRE2	0.303878	0.109848	2.766362	0.005668562	0.090973063
LRRC8B	0.708769	0.256552	2.762676	0.005732971	0.091730066
MCRIP1	-0.283667	0.102702	-2.76204	0.005744153	0.091730066
EGR1	1.55872	0.564353	2.761961	0.005745526	0.091730066
ENSG00000233178	-0.853011	0.308904	-2.761415	0.005755145	0.091730066
ZFHX2	-0.721479	0.261309	-2.761019	0.005762125	0.091730066
HSPA9	0.455797	0.165155	2.75981	0.005783498	0.091922288
HLA.C	-0.503593	0.182514	-2.759196	0.005794377	0.091947369
CD37	-0.347918	0.126133	-2.758346	0.005809475	0.092039212
MRPL54	-0.322248	0.117015	-2.753914	0.005888726	0.09314551
PDGFA	2.062811	0.749119	2.75365	0.005893481	NA
LY9	0.847642	0.30791	2.752883	0.005907294	0.093289952
HERC1	-0.499666	0.181798	-2.748462	0.005987558	0.0944067
KANK2	-2.111799	0.768417	-2.748245	0.005991529	NA
SLC38A2	0.197532	0.072003	2.743368	0.006081255	0.09573135
RPP21	-0.44084	0.160802	-2.741519	0.006115575	0.096118562
ILF2	0.235994	0.086168	2.738787	0.006166627	0.096728316
ARL14EPL	1.328684	0.485231	2.73825	0.006176703	0.096728316
ACSL4	0.566347	0.206968	2.736399	0.006211559	0.096728316
TMEM64	0.582235	0.212775	2.736388	0.006211782	0.096728316

SQSTM1	0.320471	0.117123	2.73619	0.006215521	0.096728316
CWC22	0.249239	0.091093	2.736101	0.006217202	0.096728316
SRD5A1	-0.519806	0.190001	-2.735801	0.006222862	0.096728316
TMEM256	-0.303465	0.111172	-2.729698	0.006339243	0.098382656
TVP23B	0.464608	0.170243	2.729082	0.006351092	0.098412065
RMND5B	-0.310795	0.114002	-2.726221	0.006406412	0.099062864
GRPEL1	0.431148	0.158208	2.725205	0.006426164	0.099062864
GRHPR	-0.659806	0.242122	-2.725099	0.006428229	0.099062864
SPIN2B	0.685981	0.251751	2.724845	0.006433174	0.099062864
MID1IP1	-0.56089	0.205945	-2.723491	0.006459604	0.099315148
MRPL32	0.25943	0.095313	2.721858	0.006491602	0.099530729
TOP1	0.277788	0.102062	2.721748	0.006493761	0.099530729
NOCT	-0.982614	0.361301	-2.719654	0.006535027	NA
POMGNT1	-0.403796	0.148548	-2.718287	0.006562094	0.100422389
ANKRD46	0.540382	0.198894	2.716941	0.006588836	0.1005846
POLH	0.543295	0.199993	2.71657	0.006596217	0.1005846
ARHGAP18	0.998862	0.36774	2.716219	0.006603217	0.1005846
MBNL2	-0.633747	0.233429	-2.71495	0.006628577	0.100815561
MBTPS1	-0.38271	0.140994	-2.714365	0.006640303	0.100838761
ATG101	0.240928	0.08884	2.711943	0.006689014	0.101422691
TIMMDC1	0.291736	0.10767	2.709554	0.006737378	0.101999566
PSENEN	-0.247882	0.091626	-2.70537	0.006822837	0.103085183
VOPP1	-0.282891	0.10458	-2.705024	0.006829941	0.103085183
PSMD9	-0.247665	0.091598	-2.703823	0.006854675	0.103300789
HCG11	-0.568683	0.210428	-2.702502	0.006881986	0.103554506
AEBP2	0.383502	0.142063	2.699519	0.00694397	0.104328401
EIF5B	0.278584	0.103265	2.697764	0.006980695	0.104563683
METTL8	0.507618	0.188193	2.697324	0.006989921	0.104563683
TENT5A	0.553342	0.205178	2.69689	0.00699904	0.104563683
HIST1H2BJ	0.647225	0.240054	2.69617	0.007014177	0.104563683
ZNF441	-0.614422	0.227919	-2.695786	0.007022278	0.104563683
CYTOR	1.089933	0.404316	2.695747	0.007023092	0.104563683
ENSG00000267633	-1.785993	0.662784	-2.694683	0.007045571	NA
INSIG2	0.687706	0.255297	2.693748	0.007065351	0.105034675
YWHAG	0.317044	0.117806	2.69125	0.00711848	0.105665604
CBX6	-0.276887	0.10298	-2.688753	0.007171953	0.106299739
CERS4	-0.317425	0.11817	-2.686181	0.007227394	0.106961099
OPTN	-0.386366	0.143912	-2.684741	0.007258613	0.107262549
KCTD5	0.369916	0.137917	2.682156	0.007314942	0.107798354
CD3EAP	0.624532	0.232854	2.682076	0.00731668	0.107798354
SPAG4	0.929045	0.346698	2.679695	0.007368926	0.108277314
PPP4R2	0.277838	0.103688	2.679573	0.007371608	0.108277314
RIOK3	0.279736	0.104414	2.679099	0.007382046	0.108277314
PPA2	0.429166	0.160259	2.677944	0.007407559	0.108490567
TMEM216	0.301797	0.112741	2.676898	0.007430718	0.108534582
BLOC1S2	-0.253664	0.094763	-2.676817	0.007432522	0.108534582
CPNE3	-0.338336	0.126463	-2.675381	0.007464428	0.108839725

TMEM141	-0.310514	0.116113	-2.674244	0.007489797	0.109042941
ZBED2	1.468844	0.549534	2.672891	0.007520064	0.109042941
ENSG00000253982	-0.473082	0.176997	-2.672827	0.007521495	0.109042941
DDX31	0.568723	0.212838	2.672096	0.007537898	0.109042941
UTP18	0.31668	0.118538	2.671546	0.007550275	0.109042941
HDX	1.688083	0.631898	2.671448	0.007552487	NA
LARP1	0.332754	0.124583	2.67093	0.007564139	0.109042941
DDB2	-0.254943	0.095457	-2.670768	0.007567783	0.109042941
MED22	-0.392309	0.146905	-2.670497	0.007573894	0.109042941
NOP2	0.384951	0.144159	2.670332	0.007577635	0.109042941
DHRS7B	-0.329447	0.12351	-2.667378	0.007644557	0.109846056
FAM41C	-0.472334	0.177173	-2.665944	0.007677257	0.110155825
PEX12	1.120397	0.42057	2.663998	0.007721803	0.110634413
CCNL1	0.244577	0.091876	2.662044	0.007766772	0.111117668
CCL22	2.092216	0.786351	2.660664	0.007798671	0.111412804
PSMG3	-0.319581	0.120139	-2.660089	0.007812	0.111442188
ENSG00000260793	-0.84039	0.316158	-2.658135	0.007857443	0.11192893
TRIM27	0.24935	0.093827	2.657559	0.007870878	0.111958993
TRMT1L	0.484249	0.182619	2.651694	0.008008911	0.113758752
UCK2	0.589643	0.222824	2.64622	0.008139694	0.115450521
RHOA	0.184031	0.069574	2.645104	0.008166573	0.115647139
NBDY	-0.371228	0.14037	-2.644636	0.008177878	0.115647139
NOM1	0.335832	0.127008	2.644191	0.00818865	0.115647139
DHRSX	-0.405468	0.153548	-2.640664	0.008274383	0.116691233
PKIG	-0.537533	0.203668	-2.639269	0.008308509	0.116916338
RBM38	-0.322821	0.122326	-2.639011	0.008314835	0.116916338
PPP2R5C	-0.246451	0.093408	-2.638423	0.008329267	0.116916338
CENPM	0.616754	0.233789	2.638082	0.008337651	0.116916338
EIF4A2	0.202075	0.076672	2.635585	0.008399245	0.117613224
L3HYPDH	-0.766639	0.29099	-2.634587	0.008423979	0.11764915
RUNX3	0.249165	0.094577	2.634521	0.008425612	0.11764915
CMC2	0.252355	0.09581	2.633915	0.008440673	0.117693216
PEX10	-0.592394	0.224961	-2.633317	0.008455531	0.117734337
CPNE2	-1.888523	0.718534	-2.6283	0.008581275	NA
ZNF302	-0.263681	0.100335	-2.627999	0.008588872	0.119422772
HRK	-1.441138	0.548651	-2.626696	0.008621843	0.11971284
LPP.AS2	-1.387343	0.528364	-2.625733	0.008646272	NA
VPS25	-0.283334	0.107923	-2.625328	0.008656554	0.12002622
NPM1	0.326466	0.124439	2.623507	0.008702958	0.120500615
PSD3	1.435757	0.547332	2.623192	0.008711017	NA
ASB1	0.355522	0.135579	2.622244	0.008735282	0.120693255
DOCK10	-0.447107	0.170521	-2.62201	0.008741288	0.120693255
TGM2	2.23817	0.853756	2.621558	0.008752879	NA
HEATR1	0.418826	0.159802	2.620912	0.008769479	0.120758776
HGSNAT	-0.49343	0.188269	-2.620874	0.008770463	0.120758776
YBEY	-0.294974	0.112696	-2.617436	0.008859318	0.121671211
RRAGC	0.268095	0.102435	2.617219	0.008864936	0.121671211

FTL	0.252897	0.096641	2.616884	0.008873654	0.121671211
WDR76	-0.569838	0.217828	-2.615997	0.008896741	0.12177956
TFEB	0.350023	0.133819	2.615634	0.008906193	0.12177956
LINC02166	1.259314	0.481775	2.613905	0.008951389	NA
ZNF692	-0.4675	0.178909	-2.61306	0.008973542	0.122530994
PIGF	0.639347	0.244903	2.610619	0.009037863	0.123239053
ENSG00000224746	-1.375713	0.527094	-2.609996	0.009054321	NA
PET100	-0.234241	0.089782	-2.609011	0.009080431	0.123648959
ENSG00000237499	-0.905875	0.347275	-2.608521	0.009093451	0.123655922
MGAT5B	-1.124268	0.431137	-2.607684	0.009115698	NA
TUT4	0.256567	0.098448	2.60611	0.009157698	0.124358521
OGFRL1	-0.46376	0.178085	-2.604153	0.00921017	0.124899506
KIAA1586	-0.519872	0.199735	-2.602815	0.00924619	0.125162789
ELF2	-0.227534	0.087429	-2.602492	0.009254906	0.125162789
HLA.A	-0.452282	0.173929	-2.600387	0.009311858	0.125723231
EMC2	0.372337	0.14323	2.599576	0.009333909	0.125723231
FUOM	-0.492584	0.189488	-2.599554	0.009334498	0.125723231
RNASEH2B	0.33054	0.127262	2.597326	0.009395284	0.126369768
SELENOF	-0.13418	0.05168	-2.596386	0.009421011	0.126486822
TICAM1	0.427995	0.164884	2.595737	0.00943884	0.126486822
MRPL43	-0.191647	0.073842	-2.59537	0.009448921	0.126486822
THRAP3	-0.223144	0.085985	-2.595143	0.009455165	0.126486822
GNG3	-2.363786	0.910903	-2.594992	0.009459308	NA
CTNNBIP1	-0.89522	0.345223	-2.593164	0.00950975	0.12704512
VMP1	0.226869	0.087529	2.591934	0.009543801	0.127327961
PBX4	1.170384	0.451642	2.591395	0.009558769	0.127355788
ENSG00000279927	-1.240121	0.478555	-2.591388	0.00955896	NA
NR4A2	1.254824	0.484443	2.590241	0.009590874	0.127434772
NBPF9	0.855254	0.330233	2.589848	0.009601834	0.127434772
GPS1	-0.362204	0.139858	-2.589793	0.009603369	0.127434772
USP14	0.299088	0.11551	2.589268	0.009618018	0.127458084
TOB1	0.524218	0.202564	2.587914	0.009655898	0.127788766
TUNAR	-1.864856	0.720847	-2.587035	0.00968058	NA
CCDC159	-0.563557	0.217903	-2.586278	0.009701857	0.128109597
ZNF296	0.5271	0.203818	2.586129	0.009706058	0.128109597
ADNP2	0.736761	0.28508	2.5844	0.009754866	0.128582143
MCOLN2	1.290717	0.499698	2.582994	0.009794713	0.128935469
G2E3	0.404072	0.156533	2.581387	0.009840424	0.129364942
DEGS1	0.305943	0.118555	2.580599	0.009862897	0.129488175
NUDT16	-0.486751	0.188655	-2.580112	0.009876829	0.129499116
ENSG00000260879	-1.66763	0.646537	-2.579327	0.009899289	NA
KDM5B	0.423766	0.164391	2.577796	0.009943256	0.130197383
HLA.DMB	-0.453577	0.176052	-2.576377	0.009984178	0.130560292
TOX2	2.387925	0.926855	2.576375	0.009984226	NA
CD99	-0.239714	0.093123	-2.574152	0.010048626	0.131128541
ENSG00000272195	-1.597807	0.620729	-2.574083	0.010050623	NA
ZNF827	0.750003	0.291381	2.573961	0.010054161	0.131128541

XPO6	0.37905	0.14735	2.572448	0.0100982	0.131508112
MIR4435.2HG	1.700101	0.661031	2.571892	0.010114431	0.131508112
TCTN3	0.356638	0.138684	2.571593	0.010123171	0.131508112
FBXO25	-0.283036	0.110155	-2.569437	0.010186387	0.13215567
MIR155HG	0.543112	0.21153	2.567543	0.010242216	0.132622582
GPX1	-0.465096	0.181171	-2.567164	0.010253419	0.132622582
CHRNE	1.572247	0.612545	2.566747	0.010265743	NA
TFDP2	-0.50127	0.195311	-2.566525	0.010272319	0.132622582
ZBTB20	0.469308	0.182893	2.566031	0.010286977	0.132622582
LACTB2	0.421564	0.164297	2.565858	0.010292091	0.132622582
KLK1	1.702505	0.663617	2.565495	0.010302867	0.132622582
ENSG00000272155	1.311911	0.511385	2.565407	0.01030547	NA
MICOS13	-0.180787	0.070482	-2.564991	0.010317855	0.132642804
TSEN15	-0.462504	0.180418	-2.563519	0.010361712	0.133033613
TMEM220.AS1	-1.51187	0.590302	-2.56118	0.010431737	NA
TAGAP	-0.548219	0.214084	-2.560764	0.010444238	0.133919246
DLAT	-0.409439	0.160151	-2.556585	0.010570539	0.135363149
MCRS1	-0.259547	0.101666	-2.552933	0.010682014	0.136613702
PPBP	3.467389	1.358769	2.551862	0.010714909	NA
MGME1	0.394043	0.154432	2.55157	0.01072387	0.136971806
FAM228B	-0.471274	0.184807	-2.550089	0.010769531	0.137377526
HM13	0.281178	0.110295	2.549323	0.010793232	0.13744886
JAM3	0.737115	0.289177	2.54901	0.01080293	0.13744886
IGHM	-0.401936	0.157735	-2.548176	0.010828786	0.137451266
RBMS1	-0.442909	0.173821	-2.548072	0.010832014	0.137451266
BRD2	0.157584	0.061854	2.547659	0.01084483	0.137451266
HHEX	0.359748	0.141257	2.54676	0.010872818	0.137629551
ENSG00000261335	-2.046899	0.804467	-2.544415	0.010946084	NA
MIR22HG	0.526243	0.206864	2.543908	0.010961993	0.1385809
HIST1H2BH	1.582929	0.622708	2.542007	0.01102179	NA
LINC01857	0.499478	0.196504	2.541822	0.011027621	0.139232524
RCSD1	-0.527754	0.207812	-2.539573	0.01109879	0.139389701
ARHGAP1	-0.458251	0.180445	-2.539566	0.011099006	0.139389701
STAM2	0.624845	0.246066	2.539341	0.011106159	0.139389701
PDCD5	0.290694	0.114477	2.539318	0.011106875	0.139389701
SGF29	0.386486	0.152238	2.538698	0.011126581	0.139389701
BIN3	-0.377949	0.148876	-2.53869	0.011126823	0.139389701
XIAP	0.311603	0.122771	2.53809	0.011145938	0.139389701
ZNF74	1.548173	0.610006	2.537963	0.011149971	NA
ATP6V0E2	-1.026929	0.404642	-2.537872	0.011152868	0.139389701
LIMS1	0.362431	0.142978	2.534872	0.011248827	0.140342673
ABHD17B	0.372709	0.147048	2.534602	0.01125751	0.140342673
TOP2A	-1.11912	0.441611	-2.534174	0.011271281	NA
DIPK1B	-1.949302	0.769575	-2.53296	0.011310372	NA
GSDMD	-0.460588	0.181851	-2.532782	0.011316133	0.140895833
TMEM205	-0.260204	0.102811	-2.530901	0.01137701	0.141475622
PYHIN1	1.142996	0.452152	2.5279	0.011474689	NA

CD68	0.809709	0.320581	2.525751	0.011545141	0.143231586
PSMD6	0.26107	0.103366	2.525688	0.011547195	0.143231586
RNF114	0.226575	0.08973	2.525069	0.011567555	0.143304323
HVCN1	-0.513658	0.203563	-2.523336	0.011624733	0.143749607
ITGB3BP	-0.610933	0.242147	-2.522983	0.011636393	0.143749607
PITPNB	0.259126	0.102719	2.522659	0.011647121	0.143749607
CTSC	0.710801	0.28204	2.520216	0.011728298	0.14455658
UTP14A	0.377726	0.149902	2.519812	0.011741749	0.14455658
KCNRG	1.415555	0.561848	2.519463	0.011753409	NA
ATP6V0D1	0.26645	0.105766	2.519248	0.011760563	0.144608114
NUDT1	-0.354408	0.140708	-2.518746	0.011777343	0.144634548
RAD51AP1	-1.210683	0.481144	-2.516261	0.011860719	NA
TMC8	-0.412218	0.163857	-2.51572	0.011878944	0.145491569
METAP2	0.192302	0.076444	2.515579	0.011883706	0.145491569
JADE1	0.397977	0.158231	2.51517	0.011897512	0.145491569
PRDM8	-0.515733	0.205069	-2.514918	0.011905996	0.145491569
SQOR	-0.394754	0.157008	-2.514221	0.011929582	0.145599806
SLC35D1	0.745652	0.296982	2.510761	0.012047122	0.146640929
ZNF207	0.189697	0.075561	2.510511	0.012055664	0.146640929
ECH1	-0.230842	0.091954	-2.510402	0.012059385	0.146640929
TPCN1	-0.50691	0.202001	-2.509442	0.012092201	0.146859334
ADA	-0.9045	0.360721	-2.50748	0.012159561	0.147360194
NETO2	1.013812	0.40435	2.50726	0.012167131	0.147360194
CD79B	-0.383823	0.153112	-2.506814	0.012182491	0.147360194
UBA2	0.189006	0.075406	2.506507	0.012193065	0.147360194
RGS2	0.868266	0.34653	2.505604	0.012224257	0.147404178
IFI30	0.441351	0.17615	2.505538	0.012226525	0.147404178
TERF1	0.228977	0.091483	2.502938	0.012316717	0.148019196
TCEAL3	-0.415313	0.165936	-2.502855	0.012319616	0.148019196
C7orf50	-0.397556	0.158846	-2.502773	0.012322456	0.148019196
MFSD6	0.82442	0.32969	2.500595	0.01239849	0.148751789
CNKSR3	-1.554084	0.621947	-2.498741	0.012463547	NA
MAD2L1BP	0.303341	0.12145	2.497655	0.012501774	0.149809136
DGAT2	0.562929	0.225589	2.495373	0.01258248	0.150567651
HEXIM2	0.529806	0.212347	2.495005	0.012595534	0.150567651
ORAI3	-0.339965	0.136314	-2.493984	0.012631842	0.150819306
PEG10	1.401822	0.562252	2.493225	0.012658871	NA
ASCL2	1.242845	0.498822	2.491559	0.012718386	NA
ENSG00000279529	-0.571196	0.229375	-2.49023	0.012766046	0.15215436
EVI2B	-0.235565	0.094604	-2.489996	0.01277444	0.15215436
JRK	-0.609964	0.245135	-2.488272	0.012836534	0.152709962
EMC10	-0.235438	0.094666	-2.487039	0.012881146	0.153056498
NDUFA1	-0.157091	0.063192	-2.485919	0.012921738	0.153354509
CIRBP	-0.176718	0.071106	-2.485286	0.012944738	0.153443267
NT5C2	-0.485568	0.195413	-2.484832	0.012961245	0.153454934
OAZ2	-0.221656	0.089229	-2.484128	0.012986906	0.153574825
NDUFS8	-0.25388	0.102308	-2.481521	0.013082293	0.154211537

ANAPC16	-0.149422	0.060216	-2.481444	0.013085129	0.154211537
PELI1	0.365525	0.147307	2.481378	0.013087546	0.154211537
TTC39B	-1.653687	0.667369	-2.477921	0.013215024	NA
ENSG00000237950	-1.626822	0.656752	-2.477071	0.013246551	NA
SERPINB9	0.359955	0.14537	2.476133	0.013281406	0.156186709
CDCA7L	-0.42427	0.171354	-2.475989	0.013286771	0.156186709
ALKBH6	-0.646057	0.261082	-2.474538	0.013340863	0.156636308
CHP1	-0.298569	0.12071	-2.473433	0.013382178	0.156731376
ZC3H12D	-0.481398	0.194653	-2.473114	0.013394137	0.156731376
TOMM5	-0.235099	0.095064	-2.47305	0.013396522	0.156731376
TNFRSF13B	0.992687	0.40152	2.472325	0.013423757	0.15686437
GPSM3	-0.221702	0.089724	-2.470941	0.013475817	0.157203795
C1orf162	-0.410279	0.166057	-2.470707	0.013484606	0.157203795
WARS	0.740969	0.300256	2.467786	0.013595145	0.15830578
DHRS3	2.089275	0.846891	2.466995	0.013625234	0.158469482
SH3BGRL	-0.336224	0.136313	-2.466565	0.013641603	0.15847343
RSL24D1	0.191742	0.077756	2.465954	0.013664875	0.158485212
ACAT1	-0.540452	0.219188	-2.465697	0.01367468	0.158485212
SELENOK	0.208901	0.084828	2.462635	0.013792014	0.159657904
DDIT4	0.440672	0.178993	2.461949	0.013818419	0.159663473
AFDN	0.879367	0.357246	2.461519	0.013835024	0.159663473
ACP2	-0.714483	0.290342	-2.460832	0.013861513	0.159663473
SLBP	0.438191	0.178072	2.460754	0.013864541	0.159663473
L1CAM	-0.772048	0.313773	-2.460529	0.013873247	0.159663473
TUBB4B	0.397162	0.161487	2.459406	0.013916696	0.159977278
SSH2	-0.292756	0.119089	-2.458285	0.013960251	0.16016657
NFKBID	0.252663	0.102786	2.458148	0.013965566	0.16016657
ENSG00000265218	-1.178396	0.479506	-2.45752	0.01399001	NA
HK2	0.8673	0.353068	2.456467	0.014031067	0.160576103
RABGGTB	0.233927	0.095232	2.456398	0.01403376	0.160576103
IQCG	-0.630361	0.256685	-2.455775	0.0140581	0.160668646
KIF21A	1.301007	0.529847	2.455438	0.014071319	NA
SPINT2	-0.308985	0.12596	-2.453036	0.01416562	0.161310725
POLR2B	-0.26232	0.10694	-2.452977	0.014167945	0.161310725
TP53TG1	-0.393333	0.160372	-2.452632	0.014181526	0.161310725
SUB1	0.2079	0.084774	2.452415	0.014190105	0.161310725
CCDC191	-0.669602	0.273065	-2.452174	0.014199602	0.161310725
RGL1	-1.377406	0.561763	-2.451934	0.014209077	NA
DCAF11	-0.266933	0.10887	-2.451855	0.014212183	0.161310725
MKRN2	0.383121	0.156334	2.450665	0.014259254	0.161659388
GPR146	-0.764396	0.312029	-2.449756	0.014295294	0.161882328
CIITA	0.674999	0.275612	2.449087	0.014321869	0.161913742
BABAM1	-0.257303	0.105071	-2.448862	0.014330824	0.161913742
NOL6	0.696388	0.284472	2.448005	0.014364983	0.162114404
LMF2	-0.301854	0.123413	-2.445889	0.014449564	0.162882995
SLC16A1.AS1	0.346484	0.141841	2.442768	0.014575117	0.164111171
PTPN12	0.519693	0.212793	2.442248	0.014596106	0.164160527

XRN2	0.248282	0.101749	2.440141	0.014681519	0.164933523
B9D2	-0.343694	0.140895	-2.439366	0.014713055	0.165100181
C6orf226	-0.582658	0.238931	-2.438606	0.01474405	0.16526041
HDAC6	-0.362506	0.148699	-2.43785	0.014774895	0.165418583
ST13	0.143587	0.058912	2.437321	0.014796543	0.165473553
UBAC1	-0.506203	0.207825	-2.435716	0.014862344	0.166021623
ADD1	-0.224485	0.092211	-2.43446	0.01491404	0.166411058
CASP10	0.592484	0.243694	2.43126	0.015046397	0.167698621
KLHDC10	-0.547345	0.225214	-2.430334	0.015084906	0.167938486
TIAL1	0.202117	0.083202	2.429248	0.015130195	0.168253213
RHOBTB2	1.519136	0.62557	2.428404	0.015165452	NA
PRPF19	0.285078	0.117394	2.42838	0.015166453	0.168466911
ENSG00000278158	-0.376959	0.155295	-2.427367	0.015208843	0.168562717
EXOSC3	0.224857	0.092634	2.427359	0.015209179	0.168562717
METRNL	1.006883	0.414913	2.426733	0.015235484	0.16866517
ALDH4A1	-1.296345	0.53459	-2.424935	0.015311149	NA
FCRL2	0.521189	0.214958	2.424605	0.015325061	0.169467059
PTPN20	-1.406811	0.580374	-2.423974	0.015351686	NA
PLEKHO2	0.511239	0.210922	2.423829	0.015357841	0.16963979
ZNF165	0.497921	0.205484	2.423163	0.015386024	0.169639955
ZNF213.AS1	-0.616592	0.254473	-2.423018	0.015392175	0.169639955
SLC25A36	0.360501	0.148862	2.421703	0.015447988	0.170065488
IDI1	0.332979	0.137595	2.41999	0.015520928	0.17067841
ZKSCAN7	-1.727758	0.714859	-2.416922	0.015652355	NA
TRMT2B	-0.431399	0.178497	-2.41684	0.015655908	0.171751961
PRKAR1B	0.714768	0.295782	2.41654	0.015668804	0.171751961
ZNF28	-0.471522	0.195181	-2.415817	0.01569995	0.171751961
WDR47	0.65126	0.269587	2.415769	0.015702001	0.171751961
DLGAP1.AS1	-0.410454	0.169912	-2.41569	0.015705419	0.171751961
SRGAP2	0.653255	0.27065	2.413654	0.015793439	0.172523691
MMUT	0.577441	0.239399	2.412044	0.01586335	0.173096107
ACSL1	0.504578	0.209256	2.411297	0.015895912	0.173260186
MKKS	-0.287554	0.119296	-2.410423	0.015934039	0.173484485
ADAM15	0.724463	0.300737	2.40896	0.015998057	0.173989868
RSRC2	0.35086	0.14571	2.407931	0.016043194	0.174145601
UQCRQ	-0.179256	0.074447	-2.407831	0.016047607	0.174145601
SLC39A13	-0.514898	0.213923	-2.406931	0.016087189	0.17429812
PSMD14	0.336576	0.139849	2.406711	0.016096923	0.17429812
ENSG00000259529	-0.472326	0.196298	-2.406168	0.016120867	0.174366398
CYP2R1	-0.451009	0.187481	-2.405624	0.016144872	0.174378088
ENSA	-0.128826	0.053558	-2.405345	0.016157225	0.174378088
ATP2B1	-0.255261	0.106156	-2.404592	0.016190549	0.174547183
CEP164	-0.393797	0.16382	-2.403835	0.016224115	0.174718519
PAK1IP1	0.446433	0.185755	2.403338	0.016246177	0.174765726
SETBP1	0.919371	0.382878	2.401208	0.016341023	0.17559495
THG1L	0.518422	0.215951	2.400642	0.016366329	0.175675927
NDUFA4	-0.142319	0.059299	-2.400003	0.016394921	0.17572204

VTI1A	-0.311034	0.129612	-2.399743	0.016406589	0.17572204
CNOT9	-0.242906	0.101238	-2.399356	0.01642395	0.17572204
COMMD9	-0.212928	0.088807	-2.397639	0.016501137	0.175810936
RAD17	-0.335083	0.13976	-2.397569	0.016504254	0.175810936
LRWD1	0.408834	0.170541	2.397285	0.016517069	0.175810936
DDX20	0.335802	0.140103	2.396826	0.01653776	0.175810936
LMNA	0.500817	0.208951	2.396814	0.016538305	0.175810936
GABPB1	-0.232146	0.096857	-2.3968	0.016538961	0.175810936
TMEM11	0.207678	0.086685	2.395778	0.016585144	0.176083082
ZNF506	0.368328	0.153778	2.39519	0.016611771	0.176083082
ENSG00000236255	0.988413	0.412682	2.395096	0.016616014	NA
TRIP11	0.231037	0.096464	2.395052	0.016617997	0.176083082
TMCO3	-0.350324	0.146321	-2.394226	0.016655504	0.176291554
ZNF330	0.315122	0.131711	2.39252	0.016733102	0.176603013
SDAD1	0.235691	0.098518	2.392373	0.016739823	0.176603013
MCL1	0.232196	0.097067	2.392108	0.016751899	0.176603013
ENSG00000237188	-0.786321	0.328728	-2.39201	0.016756385	0.176603013
NOLC1	0.340005	0.142168	2.391565	0.016776732	0.176629153
DENND5A	0.423112	0.177027	2.390105	0.016843552	0.177131192
UPF3A	-0.299758	0.125435	-2.389741	0.016860252	0.177131192
FBXO11	0.298268	0.124853	2.388955	0.016896372	0.177322223
SERPINB6	0.465288	0.194859	2.387817	0.016948761	0.177683401
EYA3	0.519395	0.217608	2.386836	0.016994061	0.177888909
ANKAR	-0.533763	0.223649	-2.386614	0.017004352	0.177888909
YY1	0.151154	0.063353	2.38592	0.017036439	0.178036191
ASPH	1.41203	0.592035	2.385043	0.017077106	NA
ZNF254	-0.446518	0.187216	-2.385043	0.017077146	0.178105438
PRCP	-0.363937	0.152594	-2.385001	0.017079097	0.178105438
ATP5PD	-0.201685	0.084615	-2.383559	0.017146149	0.178509367
RRP1B	0.382715	0.160576	2.383391	0.017153945	0.178509367
RRM2B	-0.308042	0.129293	-2.38251	0.017195047	0.178748935
LRP5L	-0.796074	0.334191	-2.382096	0.017214411	0.178762252
MS4A1	-0.295127	0.123916	-2.381681	0.017233805	0.178775858
SPRY1	-0.76141	0.319774	-2.381086	0.017261699	0.178802457
PLEKHG1	-0.247168	0.103823	-2.380675	0.017280967	0.178802457
CDCA4	0.33896	0.142399	2.380354	0.017296021	0.178802457
MBOAT2	-1.438501	0.604468	-2.379781	0.017322927	NA
SESTD1	-0.424307	0.17835	-2.379072	0.017356303	0.178802457
UQCRHL	-0.543099	0.228298	-2.378899	0.017364444	0.178802457
WDR4	0.437226	0.183806	2.378743	0.017371766	0.178802457
ANKRD36C	-0.499521	0.209998	-2.378696	0.017373984	0.178802457
DIMT1	0.2895	0.121729	2.378228	0.017396062	0.178802457
MLYCD	-0.692493	0.291188	-2.378163	0.017399147	0.178802457
FARSA	0.237011	0.09969	2.377472	0.01743176	0.178951584
CARD11	0.478485	0.201302	2.376947	0.017456574	0.179020428
PWWP2B	0.63189	0.265884	2.37656	0.017474924	0.179022898
MYL12B	0.208715	0.087838	2.376138	0.017494939	0.17904241

BCL2A1	0.447838	0.188545	2.37523	0.017538033	0.179297821
SDC3	1.888946	0.79551	2.374508	0.017572365	NA
CYCS	0.196277	0.082691	2.373618	0.017614761	0.179896207
ZNF530	-0.499648	0.210556	-2.372989	0.017644768	0.180016698
SRP9	-0.190323	0.080251	-2.371614	0.017710582	0.18047462
POLG2	0.421913	0.177927	2.371271	0.017727022	0.18047462
RNF13	-0.275173	0.116064	-2.370872	0.017746169	0.18047462
MRPL13	0.193986	0.081845	2.370163	0.017780252	0.18047462
PUM3	0.380084	0.160364	2.37013	0.017781844	0.18047462
COPS2	0.290993	0.122794	2.369769	0.017799186	0.18047462
TMA16	0.349813	0.14775	2.367603	0.017903759	0.181158
EIF5A2	0.443864	0.187545	2.366711	0.017946947	0.181158
EDF1	-0.130519	0.055151	-2.366586	0.017952998	0.181158
TENT4B	0.541709	0.228906	2.366512	0.017956588	0.181158
PURA	0.389557	0.164615	2.366479	0.017958208	0.181158
IDH3A	0.277269	0.117191	2.365966	0.017983087	0.181161947
WAC	0.171262	0.072393	2.365716	0.017995249	0.181161947
TMEM170A	0.269041	0.113829	2.363557	0.018100452	0.182035675
PI4KB	-0.296391	0.125435	-2.362905	0.018132338	0.182112542
PPFIBP1	-1.025557	0.434071	-2.362647	0.018144938	0.182112542
MDN1	0.47586	0.201515	2.36141	0.018205614	0.182536203
WDR6	-0.269062	0.113973	-2.36074	0.01823851	0.182680756
STARD10	-0.337411	0.143005	-2.359433	0.018302869	0.183139842
PYM1	-0.263031	0.111529	-2.358405	0.018353638	0.1833362
ZNF148	-0.228828	0.097031	-2.358285	0.018359583	0.1833362
COX8A	-0.156719	0.06648	-2.357391	0.018403864	0.183592936
RIOK1	0.367892	0.156179	2.355585	0.018493574	0.184035056
ETNK1	0.251465	0.106758	2.355473	0.018499161	0.184035056
RUFY2	-0.412754	0.175239	-2.355375	0.018504031	0.184035056
DTX3	1.806038	0.766959	2.354803	0.018532504	NA
EXOSC5	0.386856	0.164434	2.352651	0.018640098	0.185202014
TNS3	0.704811	0.299747	2.351351	0.018705371	0.185663949
ACSM3	-0.671889	0.285846	-2.350528	0.018746781	0.185758891
PTRHD1	-0.317259	0.13498	-2.350414	0.018752516	0.185758891
CLCF1	0.521319	0.221949	2.348828	0.018832618	0.186365625
ITFG1	-0.323176	0.137634	-2.348083	0.018870327	0.186552052
SERF2	-0.161412	0.068755	-2.34765	0.018892257	0.186582266
SYNE2	-0.546962	0.23311	-2.34637	0.018957255	0.186871929
WRAP53	-0.491841	0.209649	-2.346026	0.018974773	0.186871929
SDF2L1	-0.26074	0.111145	-2.345957	0.018978294	0.186871929
PLTP	-1.358493	0.579182	-2.34554	0.01899956	NA
NDUFA13	-0.186108	0.079352	-2.345358	0.019008817	0.18698623
TAF5	0.550428	0.234893	2.343319	0.019113046	0.187824628
HACD4	-1.026091	0.438092	-2.342183	0.019171301	0.188210016
FXYD2	-0.922957	0.394394	-2.340194	0.019273702	0.18869438
SRSF10	0.176496	0.075422	2.340127	0.019277164	0.18869438
BRMS1L	0.476087	0.203466	2.339879	0.019289989	0.18869438

CCND2	0.542867	0.23202	2.339744	0.019296988	0.18869438
FCHSD2	-0.368947	0.157753	-2.338767	0.019347497	0.18891499
SPATC1L	-0.588951	0.25188	-2.338218	0.019375941	0.18891499
LARP4B	0.432595	0.185012	2.3382	0.019376876	0.18891499
IFITM3	-1.801063	0.770318	-2.338077	0.019383243	NA
SREK1	0.179513	0.076811	2.337073	0.019435402	0.189298901
NEURL1	1.718641	0.735687	2.336105	0.019485779	NA
ESS2	-0.412108	0.176434	-2.335757	0.019503905	0.189779136
ERGIC2	0.262424	0.112406	2.334604	0.019564137	0.19017803
ENSG00000233903	1.018254	0.436287	2.333911	0.019600362	NA
PRDX1	0.26727	0.114529	2.333654	0.019613857	0.190298871
ID3	0.26893	0.115268	2.333081	0.019643898	0.190298871
YPEL1	-0.589462	0.252672	-2.332918	0.019652455	0.190298871
TSC22D2	0.346273	0.14843	2.332897	0.019653565	0.190298871
SIK3	0.488601	0.209501	2.332208	0.01968974	0.190462591
OTULINL	-1.165627	0.499897	-2.331732	0.019714772	NA
EML4	0.297757	0.127703	2.331644	0.019719438	0.190563409
ANKRD36	-0.5209	0.223465	-2.331009	0.019752877	0.190700142
PLCB2	1.227244	0.526724	2.329959	0.01980831	NA
C18orf25	0.36881	0.158295	2.329893	0.019811827	0.190882327
CDKN1B	0.314229	0.13487	2.329856	0.019813764	0.190882327
SRD5A3	0.779584	0.334649	2.329555	0.019829673	0.190882327
ZCCHC7	-0.349529	0.150081	-2.328945	0.019861971	0.191007239
NMD3	0.283293	0.121677	2.328241	0.01989931	0.191180353
SERP2	-1.894184	0.813741	-2.327748	0.019925499	NA
PTGER4	0.57722	0.248128	2.326302	0.020002449	0.191689817
NAP1L1	0.26669	0.114654	2.326039	0.020016467	0.191689817
ARPP19	0.153126	0.065832	2.326008	0.020018143	0.191689817
IFI16	0.229701	0.098763	2.325787	0.020029899	0.191689817
MIAT	-0.994781	0.427791	-2.325388	0.020051201	0.191693069
SMIM14	-0.499389	0.214786	-2.325055	0.020069019	0.191693069
ITGB1BP1	-0.39392	0.169497	-2.32405	0.020122798	0.192021218
EPB41L2	-0.387114	0.166626	-2.323248	0.020165851	0.192114794
ATP5F1E	-0.128896	0.055486	-2.323036	0.020177221	0.192114794
APOBEC3C	-0.34238	0.147401	-2.322781	0.020190903	0.192114794
CRYZ	0.742194	0.319793	2.320857	0.020294546	0.192915269
MBOAT7	0.643383	0.277272	2.3204	0.020319224	0.192964309
POLE3	-0.159428	0.068745	-2.319108	0.020389204	0.19344306
ARPC5	-0.203922	0.087981	-2.3178	0.020460196	0.19393049
PSMB10	-0.255317	0.110176	-2.317359	0.020484178	0.193971821
ADGRB2	-1.280403	0.552588	-2.317101	0.020498208	NA
ECE2	0.542106	0.23402	2.316497	0.020531133	0.194230414
IMPACT	-0.517473	0.223521	-2.315102	0.020607328	0.194764857
ALKBH7	-0.240569	0.103954	-2.314188	0.02065741	0.194991887
PIK3AP1	-0.291207	0.125849	-2.313944	0.020670797	0.194991887
ADAP1	1.568436	0.678165	2.312765	0.020735557	NA
NCF1	-0.333274	0.144107	-2.312688	0.020739801	0.195456317

TIMELESS	-0.582487	0.251942	-2.31199	0.020778222	0.195631908
PNKD	-0.280993	0.121589	-2.311005	0.020832586	0.195957135
PRKAG1	-0.254027	0.109987	-2.309602	0.020910192	0.196352006
ETFDH	-0.383697	0.166162	-2.309171	0.020934111	0.196352006
MFSD1	0.46342	0.200687	2.30917	0.020934151	0.196352006
KIAA1324L	1.321439	0.57239	2.308636	0.02096381	NA
HUS1	0.3462	0.150001	2.30798	0.021000249	0.196785267
AP3B1	-0.48765	0.211714	-2.303345	0.02125942	0.199025217
MMADHC	0.190724	0.082874	2.30138	0.021370149	0.199872557
TMEM230	-0.156168	0.067883	-2.300545	0.021417365	0.19992941
USP24	-0.330338	0.143606	-2.300305	0.021430958	0.19992941
ZNF322	0.395146	0.171855	2.299301	0.021487825	0.19992941
NUP210	0.359576	0.156391	2.29921	0.021493039	0.19992941
GTF2H1	0.230625	0.100325	2.298776	0.021517641	0.19992941
C12orf43	-0.294141	0.127993	-2.298105	0.021555819	0.19992941
SSNA1	-0.214349	0.093325	-2.2968	0.0216302	0.19992941
DNAJC16	0.557873	0.242952	2.296229	0.02166278	0.19992941
WDR34	-0.423157	0.184288	-2.296168	0.021666252	0.19992941
EZH2	0.368938	0.160676	2.296165	0.021666446	0.19992941
KLHDC2	-0.266019	0.115865	-2.295934	0.021679663	0.19992941
GTF2H5	-0.277771	0.120993	-2.295766	0.021689265	0.19992941
DNAL1	-0.628855	0.273921	-2.295758	0.021689748	0.19992941
BORCS7	-0.22212	0.096762	-2.295517	0.021703514	0.19992941
HECA	0.326496	0.142239	2.295406	0.021709871	0.19992941
FKBPL	0.515661	0.224699	2.294901	0.021738795	0.19992941
MYO1E	0.5553	0.241997	2.294653	0.021752991	0.19992941
IFRD2	0.254329	0.110859	2.294164	0.021781107	0.19992941
TADA2A	0.463716	0.202171	2.293677	0.021809038	0.19992941
ACTL6A	0.365343	0.159286	2.29362	0.021812334	0.19992941
GAR1	0.385429	0.168046	2.293589	0.021814121	0.19992941
JAM2	-0.766354	0.33416	-2.293377	0.021826337	0.19992941
MRM1	0.510331	0.222564	2.292963	0.021850113	0.19992941
ZNF33B	-0.371611	0.162085	-2.292689	0.021865893	0.19992941
RAB1B	-0.261098	0.113897	-2.292413	0.021881815	0.19992941
MAGEH1	-0.458892	0.200217	-2.291968	0.021907475	0.199979041
C8orf82	-0.947703	0.413559	-2.291577	0.021930072	NA
MSMP	-0.847985	0.370129	-2.291054	0.021960275	0.200276089
BBS12	-0.861729	0.376216	-2.290515	0.021991454	0.200375591
ENSG00000235501	-1.880291	0.82133	-2.289326	0.022060446	NA
APOO	0.613507	0.267996	2.289237	0.022065568	0.200620931
COLGALT1	-0.458769	0.200415	-2.289091	0.022074055	0.200620931
SRPK1	-0.205804	0.089954	-2.287863	0.022145486	0.200620931
BMI1	0.28568	0.124888	2.287495	0.022166946	0.200620931
MRPL57	-0.159107	0.06956	-2.287345	0.022175683	0.200620931
TFAP4	0.634835	0.277549	2.287288	0.02217903	0.200620931
WDR90	0.973195	0.425538	2.286976	0.022197214	0.200620931
SUPT16H	-0.245186	0.107225	-2.286644	0.022216587	0.200620931

TMEM192	0.353201	0.154481	2.286377	0.02223224	0.200620931
ELP6	-0.362182	0.158428	-2.286104	0.022248175	0.200620931
ANAPC7	-0.328574	0.143742	-2.285863	0.022262281	0.200620931
ENSG00000224086	-0.511738	0.223875	-2.285821	0.022264728	0.200620931
RAB14	0.172591	0.075515	2.285523	0.022282195	0.200620931
CCDC69	-0.359428	0.15739	-2.283671	0.022390846	0.200985042
SEMA7A	0.265266	0.116163	2.283557	0.022397574	0.200985042
HES1	0.787996	0.345076	2.283546	0.022398242	0.200985042
RPS6KB2.AS1	-1.036145	0.453761	-2.28346	0.022403259	NA
DNAJA3	0.276691	0.121172	2.283449	0.022403957	0.200985042
ROR1	-1.508746	0.660998	-2.282529	0.022458148	0.201288528
TRMT11	0.363256	0.159262	2.280863	0.022556533	0.201811084
ENSG00000267458	-0.698436	0.306217	-2.280851	0.022557278	0.201811084
NXPE3	-0.535066	0.234747	-2.279328	0.022647561	0.202435617
TSN	-0.187849	0.082435	-2.278763	0.022681187	0.202507585
CRYZL1	-0.263477	0.115636	-2.278504	0.022696581	0.202507585
KLK4	2.292028	1.006234	2.277828	0.022736841	NA
IGHE	-1.034939	0.454354	-2.277823	0.022737099	0.202686171
MBNL1	-0.233556	0.102575	-2.276929	0.022790483	0.202713135
MAST3	0.577398	0.253603	2.276783	0.022799164	0.202713135
BCL7A	-0.569198	0.250005	-2.276742	0.022801639	0.202713135
TTC14	-0.238261	0.104725	-2.275113	0.022899129	0.202900531
RP9	0.399979	0.175812	2.275042	0.022903423	0.202900531
HIP1R	-0.37381	0.164326	-2.274806	0.022917549	0.202900531
SLC9B2	0.570332	0.250783	2.274205	0.022953665	0.202900531
EHD4	-0.292361	0.128562	-2.274081	0.022961088	0.202900531
MYD88	-0.337537	0.148433	-2.274007	0.022965583	0.202900531
GSTO1	0.228665	0.100557	2.273993	0.022966386	0.202900531
FOSL2	0.788304	0.346949	2.272107	0.023080064	0.203722783
EBNA1BP2	0.483369	0.212957	2.269802	0.023219575	0.20477138
AKAP17A	0.242813	0.106993	2.269438	0.023241718	0.204783977
FXYD7	0.828837	0.365568	2.267258	0.023374457	0.205770153
NOC3L	0.205926	0.090876	2.266021	0.023450121	0.205949378
MDM4	-0.193829	0.085539	-2.265983	0.023452452	0.205949378
HIF1A	0.21887	0.096593	2.265903	0.023457313	0.205949378
XRCC1	-0.375908	0.16596	-2.265057	0.023509181	0.206221618
LINC00623	-0.394895	0.174396	-2.264362	0.023551862	0.206412864
ENSG00000268858	-0.861421	0.380443	-2.264257	0.023558338	NA
BACE2	1.561015	0.689556	2.263798	0.023586535	NA
CCNH	0.265573	0.117324	2.263591	0.023599295	0.206645373
ZMAT5	-0.301637	0.133286	-2.263079	0.023630828	0.206738381
AKAP12	-2.061008	0.910778	-2.26291	0.023641258	NA
CHIC2	0.305521	0.135043	2.262391	0.023673283	0.206926677
WDFY1	0.34956	0.154546	2.261844	0.023707039	0.206965742
CDK6	-0.549082	0.242792	-2.261535	0.023726145	0.206965742
KIAA0040	0.352709	0.155976	2.261302	0.023740557	0.206965742
MCM6	0.836724	0.370267	2.259783	0.023834718	0.207577592

APBB1IP	0.436508	0.193189	2.259493	0.023852736	0.207577592
ITPKB	-0.322861	0.142916	-2.259104	0.023876947	0.207605537
OVCH1.AS1	-1.513783	0.670118	-2.258979	0.02388469	NA
ADAM10	-0.2896	0.128216	-2.258681	0.023903226	0.207651397
ENSG00000272335	1.339095	0.592957	2.258334	0.023924814	NA
BBS9	-0.72371	0.320681	-2.256791	0.024021134	0.20849248
LIPT2	-0.877108	0.388686	-2.2566	0.024033107	NA
PPRC1	0.471031	0.2088	2.255894	0.024077289	0.208598628
CEP68	-0.527971	0.234057	-2.255741	0.02408688	0.208598628
CCDC130	0.268828	0.119223	2.254833	0.024143795	0.208598628
PNPT1	0.385747	0.171133	2.254077	0.024191342	0.208598628
SKAP2	0.303963	0.134853	2.254034	0.024194049	0.208598628
S100A11	0.271057	0.12026	2.253923	0.024200989	0.208598628
PHF6	0.464225	0.205976	2.253786	0.024209619	0.208598628
APBB1	0.60904	0.270279	2.253376	0.024235447	0.208598628
MRPS2	0.340367	0.151048	2.253371	0.02423576	0.208598628
BAZ2B	-0.463529	0.20574	-2.252985	0.02426008	0.208598628
FXYD1	0.857365	0.380561	2.2529	0.024265469	0.208598628
MT.ATP8	-0.497061	0.22072	-2.252002	0.024322159	0.208904311
RANBP2	0.334196	0.148445	2.251307	0.024366076	0.209085104
TMC6	-0.318735	0.141615	-2.250721	0.024403189	0.209085104
PITPNA.AS1	-0.404405	0.179692	-2.250544	0.024414409	0.209085104
RBBP8	0.37643	0.16729	2.250164	0.024438549	0.209085104
MANF	0.226393	0.100619	2.25	0.024448956	0.209085104
MAD1L1	0.403781	0.179498	2.249509	0.024480155	0.209170972
UBE2S	0.317481	0.141264	2.24742	0.024613181	0.210125998
GNG7	-0.318255	0.141697	-2.246034	0.024701807	0.210422703
SNAPC1	0.302371	0.134629	2.245954	0.024706936	0.210422703
CDKN2AIP	0.230542	0.102691	2.245014	0.024767225	0.210422703
AHSA1	0.272119	0.121212	2.244979	0.024769494	0.210422703
ENSG00000274422	0.42953	0.191346	2.244784	0.024782025	0.210422703
DOP1B	-0.47116	0.209903	-2.244661	0.024789924	0.210422703
NAT10	0.474598	0.211444	2.244552	0.02479693	0.210422703
HTR3A	-0.949032	0.422982	-2.243671	0.024853576	NA
SLC25A38	0.201028	0.08964	2.242605	0.024922286	0.211305077
R3HDM2	-0.368116	0.164185	-2.242074	0.024956583	0.211393241
PIGQ	-0.455492	0.203183	-2.241782	0.024975451	0.211393241
NET1	1.625391	0.725063	2.241723	0.024979257	NA
SRP68	0.265977	0.118694	2.240871	0.025034441	0.211711277
PSMA6	-0.269392	0.120295	-2.239429	0.025128033	0.21227619
ENSG00000256092	-0.428999	0.191595	-2.239092	0.025149944	0.21227619
ABL1	-0.626343	0.279761	-2.23885	0.025165658	0.21227619
NRP2	-0.454591	0.203085	-2.238422	0.025193522	0.212330062
UAP1L1	0.978226	0.43708	2.238094	0.02521492	NA
MPV17L2	0.385681	0.172384	2.237339	0.025264166	0.212561378
C11orf68	-0.396517	0.177277	-2.236713	0.025305134	0.212561378
SNU13	0.182631	0.081663	2.236393	0.025326019	0.212561378

TRIM4	0.308705	0.138047	2.236229	0.025336792	0.212561378
HILPDA	-0.405234	0.181228	-2.236042	0.025349048	0.212561378
ATP6V1C2	-1.144509	0.511882	-2.235886	0.025359253	NA
ECHS1	-0.194186	0.086852	-2.235827	0.025363132	0.212561378
ARL6IP6	0.205287	0.091832	2.235461	0.025387094	0.212561378
LUCAT1	0.691547	0.309393	2.235171	0.025406112	0.212561378
RNF7	-0.146008	0.065327	-2.235038	0.025414841	0.212561378
GRWD1	0.256998	0.115002	2.234716	0.025435981	0.212561378
GPBP1L1	-0.201619	0.090244	-2.234148	0.025473325	0.21269366
AFF4	0.250708	0.112301	2.232454	0.025584972	0.213445599
KATNAL1	-0.551881	0.247305	-2.231583	0.025642535	0.213678967
TMEM242	-0.313826	0.140661	-2.231079	0.025675908	0.213678967
GXYLT1	0.414175	0.185662	2.230805	0.025694051	0.213678967
NASP	0.204543	0.091693	2.230724	0.025699402	0.213678967
RIOX2	0.402433	0.180468	2.229943	0.025751217	0.213869357
RRP1	0.273363	0.122599	2.229727	0.025765568	0.213869357
DNAJC2	0.250912	0.112617	2.228006	0.025880127	0.214542254
ZNF516	0.809183	0.363211	2.227857	0.025890037	0.214542254
ARHGAP19	-0.993988	0.446428	-2.226539	0.025978081	NA
GLRX3	0.230897	0.103734	2.225865	0.026023212	0.215465224
RHBDD2	-0.235033	0.105644	-2.224769	0.026096732	0.215893135
PTTG1	0.418449	0.188142	2.224108	0.026141149	0.216037931
DCAF16	-0.329282	0.148083	-2.223639	0.026172752	0.216037931
SUZ12	0.239167	0.107562	2.223534	0.026179794	0.216037931
JAML	-1.518141	0.682818	-2.223346	0.026192462	NA
UBR5.AS1	-0.41512	0.186735	-2.223044	0.026212826	0.216130105
MARCHF3	-0.449193	0.202127	-2.222329	0.026261104	0.216347727
DHX30	-0.247802	0.11158	-2.220845	0.026361486	0.216993884
ARHGEF26	-0.50225	0.226209	-2.220288	0.026399236	0.217065202
KYNU	0.677413	0.305177	2.219736	0.026436701	0.217065202
RND1	-0.587313	0.264605	-2.219581	0.026447194	0.217065202
NUDT8	-0.736347	0.331774	-2.219423	0.026457978	0.217065202
ITGA6.AS1	-1.076428	0.485052	-2.219204	0.026472861	NA
SPATA33	-0.369488	0.166504	-2.219096	0.026480224	0.217067578
MARCKSL1	-0.334529	0.150783	-2.218617	0.026512789	0.217074311
RBBP9	-0.454814	0.205043	-2.218141	0.026545213	0.217074311
TMA7	-0.15179	0.068432	-2.218116	0.026546919	0.217074311
MFSD13A	-0.666137	0.300374	-2.217694	0.026575728	0.217130288
GPR157	0.805301	0.363225	2.217084	0.026617344	0.217290723
PFDN2	0.220127	0.099305	2.216669	0.026645689	0.21734264
ZKSCAN1	-0.255832	0.115495	-2.215085	0.026754262	0.218048337
CD84	0.595609	0.26896	2.214485	0.026795417	0.218203866
PLAAT3	-0.879036	0.397118	-2.21354	0.026860431	0.21855327
RPRD1B	0.346731	0.156706	2.212623	0.026923635	0.218887379
ENSG00000236514	1.355805	0.61307	2.2115	0.027001198	NA
SLC35E4	0.67018	0.303122	2.210922	0.027041222	0.219662715
ICAM1	0.371017	0.167948	2.209113	0.027166765	0.220329225

SRI	0.214177	0.096952	2.209098	0.027167846	0.220329225
RIC3	0.590801	0.267573	2.207996	0.027244521	0.220769947
CCDC144A	1.621383	0.734555	2.207301	0.027293031	NA
FKBP8	-0.186224	0.084369	-2.207255	0.027296264	0.220827863
LINC01678	-0.486828	0.220558	-2.207254	0.027296343	0.220827863
RUNX1	-0.296405	0.134362	-2.206017	0.027382787	0.221346058
UXS1	-0.303255	0.137508	-2.205367	0.02742836	0.221533306
STX10	-0.370611	0.168115	-2.204507	0.027488714	0.221641433
DOCK11	-0.318264	0.144394	-2.204129	0.027515279	0.221641433
DKC1	0.3126	0.141829	2.204059	0.027520169	0.221641433
HLA.DQA1	0.492753	0.223583	2.203899	0.027531426	0.221641433
RSU1	-0.321072	0.145725	-2.203273	0.027575493	0.221759209
NT5E	0.877692	0.398404	2.203019	0.027593375	0.221759209
ATP5F1B	0.220339	0.10003	2.202736	0.027613351	0.221759209
ENSG00000256116	-1.296161	0.589735	-2.19787	0.027958378	NA
NAA38	-0.256133	0.116577	-2.19711	0.028012606	0.224680679
PNO1	0.490547	0.223284	2.19697	0.028022585	0.224680679
TMEM50B	-0.395893	0.180253	-2.196322	0.028068938	0.224869955
DUSP16	1.301333	0.592599	2.195976	0.02809366	NA
ENSG00000259038	-0.570205	0.25973	-2.195376	0.028136649	0.22492712
LYST	-0.332156	0.151303	-2.195302	0.028141973	0.22492712
MTG1	-0.373842	0.170294	-2.195269	0.02814433	0.22492712
SNHG15	0.319504	0.145568	2.194875	0.028172575	0.224970987
AMD1	0.261053	0.118956	2.194539	0.028196689	0.224981816
SNRNP40	0.194141	0.088504	2.193576	0.028265908	0.22535223
LYRM2	-0.202502	0.092332	-2.193193	0.028293474	0.225390241
CMTM8	-1.159208	0.528627	-2.192867	0.028316997	NA
ITGAE	-0.255645	0.116617	-2.192184	0.028366234	0.225787911
GBF1	0.460848	0.210321	2.191165	0.028439821	0.226030992
DNPH1	-0.353121	0.161171	-2.190968	0.028454134	0.226030992
TMEM209	-0.433734	0.197978	-2.190813	0.028465364	0.226030992
LY86	-0.376052	0.171708	-2.190067	0.02851941	0.2262784
ATIC	0.423083	0.193233	2.189501	0.028560447	0.226422278
PLCL2	-0.465945	0.213077	-2.186745	0.028761108	0.227794693
TET2	0.325822	0.149016	2.186492	0.028779645	0.227794693
STK33	-1.098065	0.502463	-2.185363	0.028862234	NA
KMO	0.380217	0.174026	2.184821	0.028901999	0.22845423
LPAR5	0.416692	0.19073	2.184723	0.028909189	0.22845423
C17orf99	-0.925237	0.423708	-2.183667	0.028986714	NA
P2RY11	0.668255	0.306187	2.182508	0.02907204	0.229557655
AFTPH	0.192576	0.088249	2.182182	0.029096103	0.229564302
BZW2	0.391993	0.179716	2.181181	0.029170014	0.22996392
LGALS3	0.567566	0.260382	2.17974	0.029276714	0.230289344
IAH1	-0.151116	0.069328	-2.17973	0.029277466	0.230289344
TGS1	0.337502	0.15484	2.17968	0.029281176	0.230289344
HAUS4	-1.024393	0.47029	-2.178217	0.029389878	0.23096052
RUNDC3B	1.907377	0.876085	2.177159	0.029468706	NA

C17orf75	0.770171	0.353845	2.176579	0.029511968	0.231735757
SLC16A1	0.415385	0.190974	2.175093	0.029623182	0.232424422
FAM129C	-0.462288	0.212656	-2.17388	0.029714115	0.232869887
ENSG00000170161	-0.445115	0.204772	-2.173708	0.029727068	0.232869887
PLLP	-1.29517	0.595971	-2.173209	0.029764554	NA
MNAT1	0.431393	0.19851	2.173159	0.029768373	0.233008816
INTS13	0.395343	0.182048	2.171646	0.0298824	0.233600481
ENSG00000258056	0.644702	0.296889	2.171529	0.029891221	0.233600481
BTG3	0.236603	0.108994	2.170785	0.029947433	0.233854913
ZMYM5	0.288818	0.133075	2.170341	0.029981024	0.233932442
UBTD2	0.699621	0.322442	2.169761	0.030024936	0.23409031
AFF3	-0.377304	0.173961	-2.168897	0.030090536	0.234416898
ENSG00000238009	-1.430163	0.659431	-2.168784	0.030099092	NA
NEK6	0.957212	0.441477	2.168204	0.030143143	0.234641817
EZH1	-0.245726	0.113437	-2.166183	0.030297176	0.235655294
UBXN2A	-0.268215	0.123842	-2.165781	0.030327944	0.235709162
CA2	0.394123	0.182023	2.165245	0.030368965	0.235842565
ASB2	0.857734	0.396742	2.161942	0.030622617	0.237505856
ENSG00000263089	0.564757	0.26124	2.161831	0.030631192	0.237505856
NUDT9	0.307236	0.14215	2.161346	0.030668604	0.237507533
METTL2A	0.299058	0.138375	2.161206	0.030679458	0.237507533
ENSG00000265100	-1.213359	0.561497	-2.160934	0.030700454	NA
RPS6KB1	0.281966	0.130501	2.160649	0.030722438	0.237654165
WDR45	-0.190061	0.087995	-2.159906	0.030779948	0.237912876
GLI4	-0.864597	0.400445	-2.159092	0.030843041	0.238075055
CXorf56	-0.529915	0.245443	-2.159014	0.030849094	0.238075055
H3F3A	-0.167041	0.077444	-2.156932	0.03101095	0.239059979
TRIM3	-0.659902	0.305971	-2.156751	0.031025081	0.239059979
RPL39L	0.760094	0.352516	2.156193	0.031068575	0.239208668
MARS2	0.735022	0.341149	2.154551	0.031196973	0.239378298
ADH5	-0.288371	0.133865	-2.15419	0.031225311	0.239378298
DCAF7	0.191894	0.089092	2.153877	0.031249819	0.239378298
ZNF587B	0.582211	0.270366	2.153415	0.031286116	0.239378298
KPNB1	0.160381	0.074479	2.153383	0.031288616	0.239378298
ZBTB21	0.336598	0.156314	2.153338	0.031292158	0.239378298
PUM1	0.339384	0.15761	2.153316	0.031293873	0.239378298
MRPL55	-0.272642	0.126622	-2.153193	0.031303487	0.239378298
MYL6	-0.155426	0.072193	-2.152913	0.031325495	0.239378298
ARHGAP5	0.355533	0.165165	2.152597	0.031350392	0.239378298
BLZF1	0.347341	0.161376	2.152366	0.031368566	0.239378298
RHOT2	-0.223972	0.104066	-2.152205	0.031381173	0.239378298
CD2	-3.331441	1.548248	-2.151748	0.03141719	NA
RPL17	-0.242804	0.112853	-2.151502	0.031436575	0.239613703
ASB8	-0.251178	0.116762	-2.151199	0.031460508	0.239613703
RNF146	0.255696	0.119035	2.148067	0.031708408	0.241150196
RFXANK	-0.265693	0.123691	-2.148034	0.031711031	0.241150196
AEBP1	-0.51387	0.239297	-2.147412	0.031760513	0.241340841

ENSG00000273221	0.457166	0.213137	2.144938	0.031957803	0.242485049
NIPSNAP2	-0.207438	0.096735	-2.144401	0.032000805	0.242485049
SIDT1.AS1	-0.723752	0.33751	-2.144384	0.032002132	0.242485049
FDPS	-0.200057	0.093297	-2.144296	0.032009204	0.242485049
HYLS1	-0.33524	0.156504	-2.142061	0.03218855	0.24365697
POLA2	-0.551953	0.257736	-2.14154	0.032230481	0.243787707
GDI2	0.169113	0.079	2.140656	0.032301816	0.244140484
ENSG00000255135	-0.665127	0.310785	-2.14015	0.032342637	0.244262271
DCAF6	-0.270711	0.126527	-2.13955	0.03239116	0.24433274
PRMT2	-0.216212	0.101061	-2.139423	0.032401398	0.24433274
PM20D2	0.561045	0.262296	2.138977	0.032437491	0.244418472
ENSG00000250616	-1.079588	0.504817	-2.138572	0.032470373	NA
ENSG00000262202	0.669232	0.312977	2.138276	0.032494344	0.244660382
PDCL3	-0.285507	0.13356	-2.137667	0.032543758	0.244845963
DPY19L4	1.212873	0.567893	2.135741	0.032700527	NA
CDKN2D	0.369095	0.172828	2.135623	0.032710185	0.245910942
ZDHHC3	-0.290585	0.136173	-2.133945	0.032847323	0.246616993
INKA1	0.571947	0.268034	2.133863	0.032853993	0.246616993
ENSG00000271895	-1.357098	0.636147	-2.133308	0.032899446	NA
TUBA1C	0.347349	0.162936	2.131817	0.033021933	0.247554483
ZNF17	0.39326	0.184479	2.131731	0.033028967	0.247554483
CCDC138	-0.63308	0.297339	-2.129151	0.033241802	0.248820643
PTP4A1	0.376844	0.177043	2.128548	0.03329171	0.248820643
IWS1	0.243433	0.11438	2.128291	0.033312966	0.248820643
NT5DC3	0.720833	0.338736	2.128008	0.033336447	0.248820643
C19orf48	0.434159	0.204028	2.127938	0.033342201	0.248820643
FUNDC1	-0.444212	0.20876	-2.127857	0.033348913	0.248820643
THRB	1.461888	0.687361	2.126811	0.033435753	NA
TMSB10	-0.221581	0.104212	-2.126255	0.033482018	0.249415253
PHTF2	-0.383778	0.180497	-2.126228	0.033484257	0.249415253
WDR43	0.377304	0.177511	2.125522	0.033543096	0.249415253
CTR9	0.303726	0.142901	2.12543	0.03355073	0.249415253
CCDC82	0.229567	0.10802	2.125218	0.033568464	0.249415253
SYNGR3	1.036381	0.48769	2.125079	0.033579982	0.249415253
LRRC8C	0.567693	0.267221	2.124433	0.033633938	0.249481875
H2AFZ	0.203285	0.095692	2.124368	0.033639423	0.249481875
RAB31	0.854649	0.402483	2.123443	0.033716724	0.249684979
MMD	-0.623475	0.293616	-2.123436	0.033717322	0.249684979
TTC28	-0.757998	0.357288	-2.121531	0.033877167	0.250544936
NAA35	0.364412	0.171775	2.121448	0.033884137	0.250544936
XPNPEP3	-0.381739	0.180005	-2.12071	0.033946204	0.250816271
PCYOX1L	-1.618157	0.763252	-2.120083	0.03399902	NA
ADORA2A.AS1	0.815942	0.384994	2.119364	0.034059696	0.251466879
MT1X	-0.691752	0.326596	-2.118067	0.034169398	0.252088558
ELL3	-1.261002	0.595606	-2.117175	0.034244992	NA
GCLC	0.502537	0.237364	2.117155	0.034246711	0.252211434
KLF13	-0.255664	0.120764	-2.117046	0.034255969	0.252211434

THAP9.AS1	-0.302344	0.14282	-2.116968	0.034262589	0.252211434
ATP5MF	-0.130522	0.061664	-2.116662	0.03428857	0.252214887
EIF2S1	0.272091	0.128593	2.115905	0.034352916	0.252447918
LSM8	-0.132575	0.062663	-2.115688	0.034371323	0.252447918
SGSM3	-0.283764	0.134188	-2.114667	0.034458361	0.252802127
UPF1	0.367735	0.173909	2.114522	0.034470693	0.252802127
PA2G4	0.212031	0.100298	2.113999	0.034515377	0.252942194
RAB40C	-0.529741	0.250747	-2.112653	0.034630453	0.25332361
TMSB4Y	-1.737448	0.822506	-2.112383	0.034653609	NA
POU2AF1	-0.251377	0.119005	-2.112315	0.03465947	0.25332361
PLEKHF1	0.58284	0.275945	2.112158	0.034672934	0.25332361
ENSG00000070423	0.214965	0.101787	2.111909	0.03469425	0.25332361
CCDC112	-0.41366	0.195872	-2.111891	0.034695797	0.25332361
SDHAF1	-0.25617	0.121338	-2.111217	0.034753669	0.25332361
HSF2	0.458244	0.217081	2.11094	0.034777428	0.25332361
STMP1	-0.248632	0.117793	-2.110761	0.034792849	0.25332361
MTMR12	-0.428076	0.202812	-2.110701	0.034798044	0.25332361
ZBTB26	-0.617633	0.29278	-2.109546	0.034897454	0.253545672
CELF1	0.216121	0.102453	2.109468	0.03490418	0.253545672
TRBC2	-0.19133	0.090701	-2.109453	0.034905488	0.253545672
VKORC1L1	0.482584	0.22889	2.108367	0.034999266	0.253854013
TYK2	-0.268946	0.127564	-2.108317	0.03500359	0.253854013
PPP1R10	0.267538	0.126927	2.107805	0.035047829	0.253854013
MRPS26	0.229357	0.108854	2.107021	0.035115799	0.253854013
SDSL	2.136029	1.013772	2.107012	0.035116572	NA
CBLL1	0.263162	0.124925	2.106557	0.035155955	0.253854013
ZNHIT3	0.191115	0.090727	2.106491	0.035161757	0.253854013
MRTFA	-0.386516	0.183498	-2.106383	0.035171075	0.253854013
TACC1	0.274098	0.130147	2.106061	0.035199093	0.253854013
JMY	0.569959	0.270658	2.10583	0.03521915	0.253854013
MRPL47	0.184077	0.087418	2.105713	0.035229284	0.253854013
RPRD1A	-0.2463	0.116968	-2.1057	0.035230397	0.253854013
ZNF704	-1.313527	0.623846	-2.105531	0.035245071	NA
IGLL5	-0.718624	0.341364	-2.105157	0.035277636	0.254009256
GPATCH4	0.296096	0.140714	2.104236	0.035357841	0.254274577
ENSG00000266962	-0.316508	0.150421	-2.104144	0.035365926	0.254274577
CNFN	-0.498149	0.236909	-2.102705	0.035491583	0.254951352
ANKRD37	0.334599	0.15915	2.102408	0.035517584	0.254951352
ZBTB22	-0.569427	0.270875	-2.102181	0.035537423	0.254951352
EIF3J	0.224287	0.106719	2.101659	0.035583176	0.255026177
PPP3CA	-0.235126	0.111899	-2.101238	0.035620111	0.255026177
DDX39A	0.244645	0.116458	2.100715	0.03566595	0.255026177
USP39	0.260361	0.123954	2.100457	0.035688702	0.255026177
ELF4	0.421215	0.200545	2.100354	0.035697732	0.255026177
ABI3	0.605785	0.288428	2.100298	0.035702633	0.255026177
PTPMT1	-0.353258	0.168264	-2.099433	0.035778723	0.255385168
RRP8	0.284861	0.135792	2.097773	0.035925176	0.255900562

ENSG00000259436	-0.426695	0.20341	-2.097716	0.035930268	0.255900562
PTPN9	-0.499903	0.23831	-2.097704	0.03593126	0.255900562
RBM41	0.362569	0.172862	2.097442	0.035954469	0.255900562
ENSG00000227070	-0.963493	0.459513	-2.096769	0.03601402	0.256067104
ILF3	0.174508	0.083246	2.096285	0.036056958	0.256067104
ZNF394	0.231915	0.110655	2.095841	0.036096336	0.256067104
DYNC1LI2	0.318083	0.151772	2.095799	0.036100069	0.256067104
LAMTOR2	-0.262631	0.125349	-2.095197	0.036153511	0.256067104
NFKB1	0.258468	0.123377	2.094939	0.03617644	0.256067104
ZBTB8OS	-0.156862	0.074877	-2.094936	0.03617671	0.256067104
ZNF585A	0.536705	0.256204	2.094835	0.036185657	0.256067104
CCT2	0.315041	0.15041	2.09455	0.036210986	0.256067104
DYNLT1	0.264086	0.126123	2.093875	0.036271105	0.256308895
LRIF1	0.286921	0.137152	2.091991	0.036439339	0.257313791
DENND4A	-0.290781	0.139041	-2.091341	0.036497513	0.257382914
AASDHPPT	0.278913	0.133368	2.0913	0.036501198	0.257382914
ENSG00000273271	-1.222557	0.584681	-2.090982	0.036529688	NA
GADD45B	0.278201	0.133069	2.090649	0.036559534	0.257387685
HDAC8	-0.439944	0.21046	-2.090394	0.03658242	0.257387685
TNFAIP8L2	0.522456	0.249937	2.090348	0.036586527	0.257387685
POLB	0.544117	0.260358	2.089875	0.036628995	0.257387685
AKAP8L	0.273338	0.130807	2.089624	0.036651573	0.257387685
SLC25A53	-0.326731	0.156364	-2.089552	0.036658088	0.257387685
PITPNM2	-1.006653	0.481774	-2.089469	0.036665525	NA
PF4	3.99046	1.911254	2.087876	0.036809037	NA
SRSF3	0.140006	0.06706	2.087794	0.036816443	0.258316078
HAUS1	-0.262684	0.125907	-2.086339	0.036947942	0.259054862
RNF149	0.216502	0.103789	2.085987	0.036979794	0.25909443
ARFGAP3	0.176499	0.084628	2.085597	0.037015166	0.259158588
CLNS1A	0.35781	0.171656	2.084465	0.037117857	0.259693653
SYNGR1	1.032011	0.495351	2.083392	0.037215534	0.260192905
EFNA4	0.423789	0.203529	2.082209	0.037323415	0.260762741
NXT1	0.176074	0.084593	2.081423	0.037395228	0.260778355
CIB1	0.166744	0.080122	2.081127	0.037422268	0.260778355
RARA.AS1	-0.529721	0.254542	-2.081074	0.037427169	0.260778355
MAFK	0.478782	0.23007	2.08103	0.037431164	0.260778355
LRMP	0.298334	0.143423	2.080101	0.037516227	0.261186913
ELOB	-0.170842	0.082165	-2.079259	0.037593529	0.261367276
TP53RK	0.28936	0.139187	2.078926	0.037624179	0.261367276
FCGR2B	0.600635	0.288947	2.078701	0.037644805	0.261367276
RNASEK	-0.307572	0.147966	-2.078668	0.037647886	0.261367276
SUSD3	0.733858	0.353137	2.07811	0.037699267	0.26145725
TRAPPC1	-0.14131	0.068004	-2.077952	0.03771374	0.26145725
ENSG00000166927	-0.659751	0.317697	-2.07667	0.037831996	0.262093282
LITAF	0.273098	0.131535	2.076239	0.037871862	0.262185736
NDEL1	0.294826	0.142057	2.0754	0.037949509	0.262361642
RBM7	0.210594	0.101478	2.075264	0.03796208	0.262361642

MICOS10	0.126242	0.060844	2.074845	0.038000936	0.262361642
AGPAT2	-0.26798	0.129158	-2.074818	0.038003426	0.262361642
YTHDF1	0.395035	0.190511	2.073557	0.038120484	0.26292579
TPT1.AS1	-0.28914	0.139459	-2.073299	0.038144435	0.26292579
CD274	1.258626	0.607133	2.073065	0.038166203	NA
ERCC1	0.456646	0.220325	2.072599	0.038209606	0.26292579
SF3A3	0.217368	0.104878	2.072577	0.038211699	0.26292579
ADIPOR2	0.291118	0.140466	2.072508	0.038218123	0.26292579
FAM216A	0.483647	0.233434	2.071876	0.038277045	0.26314803
ENSG00000263531	-0.993429	0.479552	-2.071577	0.038304934	NA
POLR2G	-0.193344	0.093339	-2.071408	0.038320683	0.263264955
PLD6	1.448186	0.699232	2.07111	0.038348538	NA
CWC25	0.19946	0.096327	2.070668	0.038389839	0.26329839
RAB11A	-0.149824	0.072367	-2.070345	0.038420088	0.26329839
LGALS3BP	-2.088372	1.008837	-2.070079	0.038444972	NA
MIR29B2CHG	0.399485	0.193006	2.069805	0.038470589	0.26329839
IKBKB	-0.278738	0.134677	-2.06968	0.038482293	0.26329839
HGS	-0.243697	0.117748	-2.069649	0.038485198	0.26329839
MRTFB	-0.589349	0.284758	-2.069648	0.03848535	0.26329839
CTPS1	0.413225	0.199689	2.069339	0.038514271	0.263314028
QDPR	0.746213	0.360702	2.068778	0.03856696	0.263362109
TRNT1	0.235321	0.113753	2.068696	0.038574584	0.263362109
C11orf98	-0.508485	0.245852	-2.068257	0.038615835	0.263461797
ENSG00000260274	-1.125561	0.54438	-2.067601	0.038677531	NA
IL21R	-0.333394	0.161267	-2.067335	0.038702618	0.263741433
ECE1	-0.316057	0.152887	-2.067255	0.038710178	0.263741433
NOB1	0.269662	0.130493	2.066491	0.038782131	0.263938602
CAPZB	-0.133255	0.06449	-2.066268	0.038803137	0.263938602
G3BP1	0.212739	0.102967	2.066098	0.038819212	0.263938602
SNX2	-0.205286	0.099412	-2.065001	0.03892287	0.26446151
NOL8	0.271769	0.131628	2.064668	0.0389544	0.264493955
SRP54	0.218617	0.105905	2.064281	0.038991087	0.264561352
PALLD	-1.497115	0.725548	-2.063427	0.039072095	NA
KIF5B	0.193042	0.093587	2.062715	0.039139757	0.265387955
PIK3CB	-0.613522	0.29748	-2.062397	0.039169959	0.265396516
RANGRF	-0.256484	0.124378	-2.062137	0.039194711	0.265396516
ZNF43	-0.286593	0.139016	-2.061588	0.039246976	0.265412336
RBM4	-0.226407	0.109824	-2.061548	0.039250742	0.265412336
LINC01535	-2.126877	1.032261	-2.060406	0.039359763	NA
GABARAP	-0.290407	0.140956	-2.060275	0.03937222	0.265951563
PCTP	-0.523009	0.25387	-2.060149	0.03938429	0.265951563
CNKSR1	1.376373	0.668249	2.059671	0.039430027	NA
SH2D2A	1.18506	0.575609	2.058792	0.039514186	0.26664658
PXMP2	-0.429745	0.208913	-2.057054	0.039681072	0.267590093
NDUFA3	-0.307345	0.149485	-2.056027	0.039779959	0.268074077
FUS	0.15948	0.077591	2.055403	0.039840121	0.268286744
MPC2	-0.212119	0.103214	-2.055137	0.039865793	0.268286744

FAHD2B	-1.119184	0.5451	-2.053171	0.040055974	NA
CFL1	-0.117452	0.057207	-2.053087	0.040064139	0.269438148
DHX33	0.439714	0.21428	2.052056	0.040164201	0.269927457
LAMTOR1	-0.165291	0.080578	-2.051335	0.040234327	0.270215051
ENSG00000077463	-0.219468	0.107028	-2.050554	0.04031039	0.270475321
ENSG00000272221	0.812066	0.396173	2.049776	0.040386285	0.270475321
SLC41A3	-0.356236	0.1738	-2.049695	0.040394245	0.270475321
LGMN	0.383443	0.187093	2.049483	0.040414874	0.270475321
B3GNT2	0.382399	0.1866	2.049298	0.040432956	0.270475321
PREX1	0.497337	0.242692	2.049254	0.040437237	0.270475321
HESX1	-2.001203	0.976604	-2.049145	0.040447957	NA
SACS	0.411861	0.201031	2.04875	0.040486581	0.270524681
ENSG00000130749	-0.277767	0.135587	-2.048619	0.040499345	0.270524681
LSM10	-0.202204	0.098725	-2.048164	0.040543972	0.270639907
NR6A1	-0.486724	0.237718	-2.047483	0.040610696	0.270878667
FEM1C	0.460038	0.224711	2.04724	0.04063454	0.270878667
NSMAF	0.507071	0.24775	2.046706	0.040686949	0.270881224
IFNAR1	-0.166318	0.081263	-2.046656	0.040691914	0.270881224
FCGRT	-0.401164	0.196065	-2.046081	0.040748437	0.270881224
GART	0.301032	0.147129	2.046036	0.040752796	0.270881224
MRPL2	-0.192711	0.094207	-2.045613	0.040794509	0.270881224
SOCS4	0.378165	0.184871	2.045564	0.040799327	0.270881224
LINC02158	-0.964832	0.471747	-2.045231	0.040832072	NA
HIC1	0.756974	0.370353	2.043924	0.040961067	0.271768741
ENSG00000230138	-0.677373	0.331452	-2.043652	0.040987982	0.271768741
MOSPD3	-0.365622	0.17902	-2.042353	0.041116529	0.272438341
TUFT1	0.847785	0.415197	2.041886	0.041162871	0.272562724
SUPT7L	-0.309734	0.151715	-2.041558	0.041195384	0.272595426
ATF4	0.232126	0.113803	2.039715	0.04137875	0.273356143
TCL1A	-0.688422	0.337529	-2.039595	0.041390661	0.273356143
SPAG7	-0.152642	0.074841	-2.039569	0.041393298	0.273356143
ARPC3	-0.120761	0.059232	-2.038801	0.041469917	0.273679304
CCS	-0.253197	0.124246	-2.037869	0.041563058	0.273785188
EXOC2	-0.337752	0.165739	-2.03785	0.041564965	0.273785188
IKZF1	-0.35819	0.175772	-2.037809	0.041569044	0.273785188
MFSD2A	0.658919	0.323526	2.036682	0.041681923	0.274335444
PSMA4	-0.128825	0.06326	-2.036421	0.041708089	0.274335444
ZNF564	-0.534714	0.26265	-2.035844	0.041766007	0.274533737
TFAM	0.244306	0.12003	2.035382	0.041812491	0.274656666
GTF2IRD2B	-0.571457	0.280834	-2.03486	0.041864916	0.274818433
SFI1	-0.303165	0.149019	-2.0344	0.041911312	0.274940431
TBRG4	0.258954	0.127317	2.033928	0.041958901	0.275070087
SPN	-1.161106	0.570936	-2.033688	0.041983087	NA
ENSG00000262580	1.571579	0.772968	2.033175	0.042034799	NA
AHI1	-0.309027	0.152054	-2.032358	0.042117437	0.275926433
SYNGAP1.AS1	1.074637	0.528952	2.031635	0.042190598	NA
PADI4	-1.42105	0.700473	-2.028702	0.042488628	NA

PAQR4	-1.847922	0.911077	-2.028283	0.04253139	NA
PLEC	-0.435024	0.214483	-2.028248	0.042534916	0.278476939
ARFRP1	-0.216381	0.106716	-2.027636	0.04259745	0.278542235
NMB	0.825288	0.407027	2.027599	0.04260124	0.278542235
NAA20	0.149495	0.073745	2.027189	0.042643074	0.27863148
FKBP3	-0.166794	0.082301	-2.02664	0.042699263	0.278811339
KLHDC3	-0.234971	0.11596	-2.026316	0.042732402	0.278811339
PIK3CG	-0.647883	0.31977	-2.026093	0.042755208	0.278811339
NKRF	0.432512	0.213617	2.024711	0.04289709	0.279552166
PFKP	-0.453646	0.224135	-2.023987	0.042971479	0.27972708
C4orf46	-0.457214	0.225936	-2.023645	0.043006648	0.27972708
ZBTB1	0.401665	0.198488	2.023624	0.043008817	0.27972708
CCT6A	0.229614	0.113492	2.023175	0.043055069	0.279843796
MKNK2	-0.33837	0.167289	-2.022672	0.043107018	0.279997357
MAPKAP1	-0.239579	0.118473	-2.022215	0.043154132	0.280016468
SMAD4	0.329805	0.163101	2.022094	0.043166609	0.280016468
TSPYL1	-0.237818	0.117656	-2.021298	0.043248916	0.280366417
EWSR1	0.251324	0.124484	2.018921	0.043495467	0.281779939
NUP35	0.324255	0.160636	2.018571	0.043531781	0.281830507
SLC11A1	2.009457	0.995867	2.017796	0.043612555	NA
NOP14	0.376801	0.186751	2.017668	0.043625835	0.282254585
COL19A1	-0.429471	0.21295	-2.016769	0.043719615	0.282676334
DOCK7	-0.646593	0.320662	-2.016431	0.043754964	0.282718437
TAF2	0.311128	0.154317	2.016159	0.043783323	0.282718437
ENSG00000130811	0.119271	0.059196	2.014841	0.043921363	0.283312042
VAPB	0.260044	0.129088	2.014468	0.043960378	0.283312042
DDX60L	-0.454591	0.225674	-2.014371	0.043970578	0.283312042
MCTS1	0.333683	0.165666	2.014187	0.043989883	0.283312042
CEP89	-0.303305	0.150612	-2.013819	0.044028551	0.283352037
S100PBP	-0.34294	0.170353	-2.013112	0.044102825	0.283352037
STK17A	0.256427	0.127405	2.012696	0.044146598	0.283352037
ENSG00000268713	0.661715	0.328803	2.0125	0.044167214	0.283352037
UBE4B	0.263975	0.131181	2.012292	0.04418912	0.283352037
HSPA1A	1.405214	0.698318	2.012282	0.04419023	0.283352037
CBLN3	-0.778164	0.386719	-2.01222	0.044196727	0.283352037
PRSS21	1.392564	0.692175	2.011868	0.044233819	NA
ZBTB11	0.308675	0.153476	2.011228	0.044301421	0.283644296
ABLIM1	-0.263731	0.131145	-2.010989	0.044326648	0.283644296
DOLPP1	0.728258	0.36217	2.010816	0.044344875	0.283644296
MAP3K12	-0.663104	0.329788	-2.010701	0.044357079	0.283644296
ABCB1	0.468912	0.233448	2.008634	0.044576	0.284859947
KLHL5	-0.396431	0.197425	-2.008006	0.044642661	0.284975708
ZNF493	-0.301566	0.150188	-2.00792	0.044651767	0.284975708
MAPKAPK5	0.233694	0.11645	2.00682	0.04476887	0.285538744
C16orf86	-0.916028	0.456608	-2.00616	0.044839153	NA
TMEM183A	-0.172913	0.086203	-2.005892	0.044867799	0.285985207
ZBTB24	-0.251594	0.125473	-2.005164	0.044945563	0.286038783

DAZAP1	-0.319052	0.159116	-2.005149	0.044947123	0.286038783
ACADVL	-0.229658	0.114545	-2.004959	0.044967438	0.286038783
PELI3	-0.879604	0.438785	-2.004635	0.0450021	NA
EBPL	-0.684647	0.341546	-2.004551	0.045011095	0.286038783
TMEM67	-0.500505	0.249702	-2.004412	0.045025988	0.286038783
SECISBP2	0.245747	0.122617	2.004189	0.045049806	0.286038783
CARMIL1	-1.354252	0.675874	-2.003705	0.045101699	0.286083149
SOX5	1.532821	0.765016	2.003645	0.045108089	NA
AGO1	-0.279586	0.139545	-2.003558	0.045117401	0.286083149
GCHFR	-0.329644	0.164558	-2.003205	0.045155295	0.286083149
POMC	-0.497997	0.248642	-2.002864	0.04519191	0.286083149
VARS	0.466805	0.233102	2.002578	0.045222643	0.286083149
FUBP1	0.267605	0.133635	2.002505	0.045230423	0.286083149
COLEC12	-0.852368	0.425932	-2.001182	0.045372782	NA
LY6E	0.375423	0.187622	2.000959	0.045396802	0.286883279
DTWD1	0.231429	0.115669	2.000791	0.045414964	0.286883279
ABCD1	-0.915219	0.457446	-2.000715	0.045423151	NA
TMEM115	0.210536	0.105251	2.000329	0.045464798	0.287014684
TXNL4B	0.289599	0.144804	1.999941	0.045506679	0.287095743
RBM43	-0.739303	0.369841	-1.998976	0.045610902	0.287569757
ENSG00000274265	-0.359474	0.179885	-1.998355	0.045678141	0.287810136
CD7	-3.958443	1.981178	-1.998024	NA	NA
ATP5F1EP2	-0.328432	0.164478	-1.996812	0.045845617	0.288557208
KPTN	-0.600094	0.300539	-1.996725	0.045855085	0.288557208
GLO1	0.243746	0.122112	1.996091	0.045924025	0.288710651
ZNF226	-0.262834	0.131696	-1.995765	0.045959551	0.288710651
SGO2	-0.4494	0.225185	-1.995695	0.045967081	0.288710651
ENSG00000277825	-1.240192	0.621797	-1.994528	0.046094394	NA
SELENOI	0.521369	0.261445	1.994181	0.046132327	0.289181026
ASH1L.AS1	-0.556324	0.279018	-1.993865	0.046166801	0.289181026
ENSG00000277511	-0.583822	0.292814	-1.993837	0.046169909	0.289181026
ALKBH2	0.446685	0.224037	1.993796	0.046174322	0.289181026
BNIP2	-0.226939	0.11383	-1.993669	0.04618823	0.289181026
PCNX4	0.280813	0.140877	1.993313	0.046227198	0.289241825
GTPBP4	0.367056	0.184218	1.992511	0.046315017	0.289608005
KLHL8	0.411593	0.206643	1.991811	0.046391845	0.289905047
HNRNPD	0.203728	0.102314	1.991198	0.046459145	0.289972299
NUP155	-0.374686	0.188173	-1.991178	0.046461271	0.289972299
TLR1	-0.338097	0.169891	-1.990077	0.046582443	0.290545131
ENSG00000273759	-1.214043	0.610187	-1.989625	0.04663227	NA
IFI35	-0.31581	0.158747	-1.989388	0.046658414	0.290638546
NOL10	0.235213	0.118238	1.989313	0.046666708	0.290638546
CEP128	-0.784995	0.39464	-1.989141	0.046685617	0.290638546
PRPF40B	-0.687368	0.345712	-1.988268	0.046782051	0.290874955
ENSG00000273619	-1.212648	0.609902	-1.988267	0.046782132	NA
SLC17A9	0.668022	0.335982	1.988265	0.046782438	0.290874955
SCML1	0.549742	0.276603	1.987476	0.046869668	0.291061619

MSANTD4	0.334078	0.168093	1.987461	0.046871343	0.291061619
C2orf76	-0.302783	0.152368	-1.987184	0.046902025	0.291069318
ZNF132	1.219827	0.613987	1.986731	0.046952224	NA
DYNLT3	-0.201425	0.101387	-1.986694	0.046956279	0.291223195
IKZF3	0.16951	0.085344	1.9862	0.047011089	0.29138033
TPM1	0.580774	0.292498	1.985565	0.047081643	0.291634787
GGCX	-0.352396	0.17756	-1.98466	0.047182268	0.291959526
WEE1	0.49893	0.251415	1.98449	0.04720121	0.291959526
SNHG10	-0.441687	0.222602	-1.984202	0.047233376	0.291959526
SAMD9	-0.260885	0.131492	-1.984033	0.047252199	0.291959526
CCDC28A	-0.287178	0.144773	-1.983646	0.047295278	0.292043175
SHROOM3	-2.279327	1.149289	-1.98325	0.04733948	NA
CRLF3	0.234174	0.11812	1.982505	0.047422752	0.292504102
MGST2	-1.204259	0.6075	-1.982317	0.047443739	NA
ZNF502	-0.603565	0.304552	-1.981814	0.047500102	0.292504102
GRN	-0.310413	0.156636	-1.981749	0.047507326	0.292504102
DELE1	-0.321433	0.162209	-1.981593	0.047524849	0.292504102
PLEKHB2	0.213172	0.107584	1.981454	0.047540377	0.292504102
FAM200B	-0.328176	0.165646	-1.981193	0.047569689	0.292504102
C11orf71	-0.446279	0.225265	-1.981127	0.047577038	0.292504102
MAP4K5	0.323114	0.163183	1.980076	0.047694996	0.293040121
RPA2	0.146453	0.073973	1.979822	0.047723507	0.293040121
BORCS5	0.40652	0.205405	1.979115	0.047803008	0.293346084
C1orf109	0.302175	0.152732	1.978467	0.047876029	0.293611923
POLR3F	0.368517	0.18631	1.977971	0.047931993	0.293772895
PLEKHG2	0.872735	0.441437	1.977031	0.048038087	0.294240725
CCT4	0.20185	0.102123	1.976546	0.04809298	0.294394553
AP1S1	-0.289188	0.146365	-1.975797	0.048177785	0.294628952
UBE2T	0.563556	0.285246	1.975681	0.048190877	0.294628952
LINC01597	-1.192254	0.603574	-1.975323	0.048231529	NA
UQCRB	-0.120694	0.061112	-1.974965	0.048272094	0.294836402
HLA.F	-0.250229	0.126707	-1.974856	0.048284456	0.294836402
LCMT1.AS1	1.481025	0.750222	1.974116	0.048368547	NA
C1orf112	-0.763788	0.386903	-1.97411	0.048369258	0.295162932
U2AF1L4	-0.263336	0.133412	-1.97386	0.048397644	0.295162932
KCMF1	0.315641	0.159992	1.972859	0.048511609	0.295675564
ENSG00000236935	0.899521	0.456305	1.971317	0.048687621	0.296461654
HNRNPF	0.228374	0.115869	1.970965	0.048727833	0.296461654
MGAT1	0.19165	0.097238	1.970942	0.048730547	0.296461654
IL2RA	-0.456854	0.231984	-1.969332	0.048914985	0.297312802
METTL15	-0.288796	0.146689	-1.968761	0.048980516	0.297312802
MRPL34	-0.22649	0.115047	-1.968683	0.048989562	0.297312802
RSRP1	0.264484	0.134347	1.968672	0.04899075	0.297312802
PLCE1	-0.282948	0.143832	-1.967205	0.049159569	0.297988604
RPP38	0.202456	0.102917	1.967181	0.049162393	0.297988604
ENSG00000231609	-1.015367	0.516528	-1.965753	0.049327114	NA
CD247	-2.32587	1.183413	-1.965392	0.049368863	NA

HTATIP2	0.224313	0.114136	1.96532	0.049377247	0.299107515
RASSF5	-0.195172	0.099325	-1.964979	0.049416665	0.299121516
KIF16B	-0.553866	0.281908	-1.964705	0.049448375	0.299121516
RSBN1	0.22981	0.116982	1.964491	0.049473175	0.299121516
CFAP54	1.699606	0.86528	1.964226	0.04950389	NA
YPEL3	-0.250508	0.127541	-1.964144	0.049513389	0.299121516
KATNA1	0.269825	0.137386	1.963994	0.049530844	0.299121516
POLR1C	0.331428	0.168829	1.963093	0.049635381	0.299569828
ENSG00000268400	-0.967054	0.492741	-1.962602	0.049692476	0.299731432
MNDA	1.736916	0.885673	1.961127	0.049864212	NA
ENSG00000230555	-1.026782	0.523691	-1.960663	0.04991832	NA

Cluster 10	log2FC	lfcSE	stat	pvalue	padj
LEF1	-3.277376	0.470469	-6.966191	3.26E-12	3.54E-08
SMIM10	-2.838875	0.413627	-6.863377	6.73E-12	3.66E-08
POU2F2	0.4511	0.071072	6.347059	2.19E-10	6.77E-07
SESN3	0.564108	0.089628	6.293883	3.10E-10	6.77E-07
TMEM123	-0.644281	0.102377	-6.293245	3.11E-10	6.77E-07
CST7	-4.156998	0.664327	-6.257454	3.91E-10	7.10E-07
ACAP1	0.563093	0.091739	6.138009	8.36E-10	1.06E-06
MIF	-0.612702	0.099896	-6.133411	8.60E-10	1.06E-06
ARL4C	-1.322753	0.215755	-6.130801	8.74E-10	1.06E-06
DNAJB1	0.8305	0.139241	5.964473	2.45E-09	2.67E-06
BIN1	0.599304	0.105109	5.701719	1.19E-08	1.17E-05
KLK4	2.735244	0.481551	5.680067	1.35E-08	1.22E-05
RAB31	1.79837	0.322444	5.577306	2.44E-08	2.05E-05
FAM102A	1.04648	0.190936	5.480778	4.23E-08	3.08E-05
MACROD2	-0.983326	0.179427	-5.480355	4.24E-08	3.08E-05
KRCC1	0.473217	0.08688	5.446771	5.13E-08	3.20E-05
ATP6V1C2	-1.827083	0.33565	-5.443419	5.23E-08	3.20E-05
CDC42EP3	1.038097	0.19078	5.441325	5.29E-08	3.20E-05
FCRL4	1.588379	0.293878	5.404894	6.48E-08	3.71E-05
APBB2	-1.928416	0.363193	-5.30962	1.10E-07	5.70E-05
GATD3	4.160742	0.783649	5.309447	1.10E-07	5.70E-05
SLC50A1	-0.565696	0.110325	-5.127539	2.94E-07	0.000143736
ZNF322	0.846105	0.165219	5.12111	3.04E-07	0.000143736
HHIP.AS1	-3.065624	0.600415	-5.105843	3.29E-07	0.000149348
PCIF1	-0.608118	0.119519	-5.088036	3.62E-07	0.000157509
HOMER2	-1.276196	0.252408	-5.056087	4.28E-07	0.000177221
CIRBP	-0.344716	0.068325	-5.045268	4.53E-07	0.000177221
SRGN	-0.574961	0.114095	-5.039311	4.67E-07	0.000177221
ENSG00000272211	-1.017528	0.202	-5.037278	4.72E-07	0.000177221
RSRP1	0.501517	0.099776	5.026419	5.00E-07	0.000181299
FTL	0.359395	0.072357	4.966944	6.80E-07	0.000238803
SCRN1	1.270594	0.25674	4.94895	7.46E-07	0.000253784
MRPL54	-0.427416	0.087079	-4.908344	9.18E-07	0.000302933
SWAP70	0.299062	0.061665	4.849815	1.24E-06	0.000395591
ADA	-0.64187	0.134141	-4.785045	1.71E-06	0.000531602
TTC32	-0.83229	0.177982	-4.676253	2.92E-06	0.00086433
PHF23	0.502801	0.107549	4.675088	2.94E-06	0.00086433
RUBCNL	-0.513987	0.110385	-4.656326	3.22E-06	0.000921994
GART	-0.489767	0.105712	-4.633011	3.60E-06	0.001005753
LPIN1	0.611573	0.132172	4.627105	3.71E-06	0.00100898
CBFA2T3	1.217315	0.264884	4.595652	4.31E-06	0.001145202
LDHA	-0.410841	0.089721	-4.579085	4.67E-06	0.001185692
TMEM170A	0.454331	0.099288	4.575866	4.74E-06	0.001185692
CCDC191	-1.220477	0.267116	-4.569091	4.90E-06	0.001185692
TNFRSF13B	1.421134	0.311057	4.56873	4.91E-06	0.001185692
HMOX1	1.113125	0.244073	4.560632	5.10E-06	0.001185692

NCBP3	0.81316	0.178464	4.556426	5.20E-06	0.001185692
BHLHE41	1.787357	0.392362	4.555381	5.23E-06	0.001185692
SYNGR2	-0.401018	0.088213	-4.546019	5.47E-06	0.001214343
CRK	0.552476	0.121901	4.532191	5.84E-06	0.001270709
SERTAD3	0.604573	0.134151	4.506662	6.59E-06	0.00140543
RCC1	0.441141	0.097979	4.502409	6.72E-06	0.001406286
CAV1	-0.758524	0.168976	-4.488943	7.16E-06	0.001469902
KIAA0040	0.633006	0.143127	4.422684	9.75E-06	0.001916346
APAF1	1.126218	0.254767	4.420581	9.84E-06	0.001916346
DEDD2	0.468402	0.105992	4.419223	9.91E-06	0.001916346
DBI	-0.360214	0.081629	-4.412792	1.02E-05	0.001916346
ID3	0.396926	0.089952	4.412634	1.02E-05	0.001916346
ABRACL	-0.497587	0.113155	-4.397384	1.10E-05	0.002013896
NAB2	0.627813	0.142863	4.394516	1.11E-05	0.002013896
PLGRKT	-0.586997	0.133919	-4.383226	1.17E-05	0.002070129
JMJD6	0.589244	0.134488	4.381392	1.18E-05	0.002070129
PARK7	-0.25979	0.059791	-4.344949	1.39E-05	0.002398771
GID8	-0.354723	0.081692	-4.342217	1.41E-05	0.002398771
IL27RA	0.592806	0.136702	4.336498	1.45E-05	0.002424136
CHCHD5	-0.406058	0.093832	-4.327499	1.51E-05	0.00245418
KCTD7	0.959004	0.221627	4.327115	1.51E-05	0.00245418
ENSG00000253701	1.078406	0.249945	4.314579	1.60E-05	0.002559455
MAML3	-1.585732	0.370282	-4.282496	1.85E-05	0.002915146
MIR29B2CHG	0.656564	0.153586	4.274897	1.91E-05	0.002973288
RNF43	-1.124725	0.264223	-4.256729	2.07E-05	0.003179959
HIP1R	-0.451171	0.106575	-4.23337	2.30E-05	0.003480084
MT1X	-0.733507	0.173469	-4.228454	2.35E-05	0.003508269
MAP3K8	-0.574588	0.136166	-4.21976	2.45E-05	0.003539832
HPRT1	-0.363316	0.086108	-4.2193	2.45E-05	0.003539832
H1FX	-1.31288	0.311304	-4.217362	2.47E-05	0.003539832
KRTCAP2	-0.301749	0.071689	-4.209137	2.56E-05	0.003579605
LMBRD1	-0.392894	0.093347	-4.208975	2.57E-05	0.003579605
ADI1	-0.357616	0.085524	-4.181494	2.90E-05	0.003989874
GLUL	-0.596497	0.143014	-4.170889	3.03E-05	0.004127944
IL21R	-0.751451	0.180553	-4.161935	3.16E-05	0.004212585
SUSD3	0.881508	0.211869	4.160626	3.17E-05	0.004212585
SNHG9	-0.419864	0.101178	-4.149768	3.33E-05	0.004277747
SIAH2	-0.571151	0.137719	-4.14723	3.37E-05	0.004277747
CFAP73	-1.173217	0.283068	-4.144647	3.40E-05	0.004277747
TMEM120A	-0.642795	0.155276	-4.139697	3.48E-05	0.004277747
CHMP6	-0.48437	0.117006	-4.139686	3.48E-05	0.004277747
ASPSCR1	-0.457915	0.110651	-4.13838	3.50E-05	0.004277747
ENSG00000226571	1.310699	0.31672	4.13836	3.50E-05	0.004277747
FIS1	-0.321783	0.078016	-4.124578	3.71E-05	0.004440432
ASF1A	0.528484	0.128161	4.123595	3.73E-05	0.004440432
ARID5A	-0.533026	0.129347	-4.120906	3.77E-05	0.004440432
WASHC2C	0.603819	0.14657	4.119668	3.79E-05	0.004440432

FCGR2B	1.048985	0.25579	4.100957	4.11E-05	0.00476401
STX7	0.340396	0.083491	4.077064	4.56E-05	0.005225232
LACC1	-1.428536	0.350698	-4.073403	4.63E-05	0.005252788
CD5	-2.145451	0.528118	-4.062445	4.86E-05	0.005410952
RNF34	0.513099	0.126327	4.061682	4.87E-05	0.005410952
DUS4L	-0.699471	0.173469	-4.032244	5.52E-05	0.006073805
GALNT2	-0.727716	0.180724	-4.02668	5.66E-05	0.006157082
SP110	0.41821	0.104009	4.020908	5.80E-05	0.006229706
TOB2	0.401639	0.099929	4.019257	5.84E-05	0.006229706
LCN8	4.124227	1.027611	4.013411	5.98E-05	0.006324082
NINJ1	-0.642947	0.160316	-4.010508	6.06E-05	0.006340799
GBGT1	0.844982	0.212169	3.982598	6.82E-05	0.007065908
ENSG00000283013	-0.686143	0.173259	-3.960222	7.49E-05	0.00766786
EIF2AK2	0.398931	0.100775	3.958626	7.54E-05	0.00766786
DDAH2	0.563955	0.143222	3.937636	8.23E-05	0.008292817
GRHPR	-0.517961	0.131772	-3.930745	8.47E-05	0.008455868
TIMM10	-0.521002	0.132812	-3.922841	8.75E-05	0.00861942
TPP1	0.373897	0.095339	3.921759	8.79E-05	0.00861942
ATF2	0.597069	0.152735	3.909192	9.26E-05	0.008999243
MED21	0.507331	0.130924	3.874996	0.00010663	0.010270095
ENSG00000273319	0.723996	0.187195	3.867594	0.00010991	0.010493919
LINC00638	2.615354	0.677791	3.858646	0.00011402	NA
EIF4A2	0.172071	0.044775	3.843006	0.00012154	0.011410479
PPP1R18	0.352145	0.091636	3.842855	0.00012161	0.011410479
ATP1B3	-0.362639	0.094586	-3.833941	0.00012611	0.011731121
WDFY1	0.571664	0.149572	3.822012	0.00013237	0.01220922
LRIF1	0.55601	0.145769	3.814312	0.00013656	0.012389798
MX2	1.128416	0.295843	3.814241	0.0001366	0.012389798
CYB5R3	0.376574	0.098913	3.807101	0.00014061	0.01264753
ARL17A	-0.507676	0.13344	-3.804526	0.00014208	0.012675022
CDCA4	0.473937	0.124731	3.799662	0.00014489	0.012821316
RPL8	0.222603	0.05868	3.793478	0.00014855	0.013039018
CYB5A	-0.441237	0.116508	-3.787192	0.00015236	0.013266257
MRPL23	-0.310776	0.082139	-3.783528	0.00015462	0.013356279
NME1	-0.297206	0.078669	-3.777931	0.00015814	0.013552427
SRP14.AS1	0.64525	0.171287	3.767078	0.00016517	0.013969781
ZNRD2	-0.339556	0.090152	-3.766468	0.00016557	0.013969781
GCSH	-0.455329	0.121068	-3.760919	0.00016929	0.014173523
STRAP	-0.276573	0.073596	-3.757989	0.00017128	0.014202314
TGOLN2	0.307672	0.081905	3.756453	0.00017234	0.014202314
CPM	-1.184342	0.315429	-3.7547	0.00017355	0.014202314
SERP2	-1.646412	0.439616	-3.745113	0.00018031	0.0146457
DOCK8	0.335985	0.08981	3.741086	0.00018323	0.014772158
ORAI2	0.28864	0.077262	3.735874	0.00018706	0.014880448
HIST1H4C	0.278735	0.074617	3.735551	0.0001873	0.014880448
PIP5K1B	-0.735708	0.19707	-3.733231	0.00018904	0.014909403
CHORDC1	0.414949	0.111266	3.729355	0.00019197	0.014950666

SGPP1	1.006149	0.269824	3.728911	0.00019231	0.014950666
ZNF217	0.538212	0.144879	3.714909	0.00020328	0.015691236
IMPACT	-0.596928	0.161575	-3.694435	0.00022038	0.016723228
HMCES	-0.895007	0.242267	-3.694293	0.0002205	0.016723228
BCAT1	-0.751113	0.203365	-3.693423	0.00022126	0.016723228
ZBTB20	0.45887	0.124583	3.683253	0.00023028	0.017285
ELOB	-0.171579	0.04661	-3.681157	0.00023218	0.01730842
OSBPL8	0.366561	0.099728	3.675599	0.00023729	0.017569304
SPDL1	0.719033	0.196592	3.657483	0.0002547	0.018731067
NAGK	0.41231	0.11284	3.653938	0.00025825	0.018864304
PIGQ	-0.574912	0.158039	-3.637793	0.00027498	0.01995284
HLA.A	-0.487569	0.13451	-3.624786	0.0002892	0.020845414
SRPK1	-0.328468	0.09082	-3.616686	0.0002984	0.021366967
SNX8	-0.446884	0.123711	-3.612317	0.00030347	0.021588277
TCF4	0.539482	0.149453	3.609699	0.00030655	0.021665686
PDE7B	-1.258658	0.349355	-3.602808	0.0003148	0.021875174
SERPINB6	0.691573	0.191977	3.602371	0.00031533	0.021875174
ARF5	-0.376263	0.104454	-3.602191	0.00031555	0.021875174
NFKBIA	-0.399197	0.111018	-3.595777	0.00032343	0.022279456
LILRB1	0.569778	0.158618	3.592144	0.00032797	0.022445818
MLST8	-0.405681	0.112985	-3.590563	0.00032996	0.022445818
NTHL1	-0.46736	0.13037	-3.584868	0.00033725	0.022798878
XXYLT1.AS2	1.671067	0.466732	3.580359	0.00034312	0.023052748
RDX	0.507969	0.142349	3.568466	0.00035908	0.023976644
PRCP	-0.475806	0.133711	-3.558462	0.00037303	0.024756644
PHGDH	-1.138611	0.320125	-3.556773	0.00037544	0.024765287
RUVBL2	-0.357687	0.101009	-3.541153	0.00039838	0.026120408
ENSG00000279483	-0.37212	0.105171	-3.538237	0.00040281	0.026252489
PMS2	-0.496263	0.140516	-3.531733	0.00041285	0.026596437
NDUFB10	-0.276813	0.078381	-3.531652	0.00041297	0.026596437
CLCF1	0.552388	0.156611	3.527139	0.00042008	0.026894775
RGS19	0.367913	0.104401	3.52403	0.00042504	0.027053211
SOX4	-0.844678	0.240823	-3.507457	0.00045241	0.028465332
DAZAP1	-0.393787	0.112277	-3.507279	0.00045272	0.028465332
SKAP2	0.337699	0.096338	3.505351	0.00045601	0.028465332
POLR2I	-0.282294	0.080555	-3.504373	0.00045768	0.028465332
WDR11	-0.366921	0.104836	-3.499941	0.00046536	0.028778341
SSPN	2.104394	0.60192	3.496138	0.00047205	0.029026752
MRPL44	0.406948	0.116504	3.492992	0.00047764	0.029205869
LINC02245	-0.648651	0.186277	-3.482182	0.00049735	0.030082548
LRWD1	0.636316	0.182739	3.482095	0.00049751	0.030082548
CD53	0.267677	0.076965	3.477892	0.00050537	0.030389463
CARMIL1	-1.613174	0.464413	-3.473574	0.00051358	0.030712923
PAM	-1.173035	0.33791	-3.471438	0.00051768	0.030789153
ENSG00000263394	1.324308	0.382038	3.466428	0.00052742	0.031198237
ADAMTS6	1.001567	0.28911	3.464315	0.00053158	0.031274391
ZC3H6	0.695359	0.200941	3.460506	0.00053916	0.031431822

SNAPC1	0.542533	0.156798	3.46007	0.00054004	0.031431822
IKZF3	0.283912	0.082131	3.456808	0.00054662	0.031645515
HDDC2	-0.27251	0.078931	-3.452498	0.00055542	0.031985264
TUNAR	-2.844977	0.824074	-3.452331	0.00055577	NA
KLHL6	0.59585	0.172759	3.449024	0.00056262	0.032158821
LGALS1	1.081508	0.313724	3.447325	0.00056617	0.032158821
NRP2	-0.882205	0.25595	-3.446785	0.0005673	0.032158821
TNFRSF12A	1.229474	0.357137	3.442579	0.0005762	0.032422967
ARRDC3	-0.398424	0.115761	-3.441772	0.00057792	0.032422967
PALLD	-2.006014	0.582944	-3.441179	0.00057919	NA
PDGFA	1.164959	0.340099	3.425353	0.000614	0.034270724
UCP2	-0.380483	0.111185	-3.422052	0.0006215	0.034512471
METRNL	1.146097	0.33545	3.416594	0.0006341	0.03503308
ACADVL	-0.260936	0.076431	-3.413995	0.00064018	0.035190361
MIR22HG	0.82831	0.242999	3.408693	0.00065275	0.035701158
ZBTB16	2.984238	0.876424	3.405018	0.0006616	NA
UBE2E3	-0.29012	0.085287	-3.401702	0.00066968	0.036189787
BOLA2B	-0.550829	0.161954	-3.401143	0.00067105	0.036189787
CKS2	0.384342	0.113012	3.400894	0.00067166	0.036189787
MYO1F	0.721188	0.212621	3.391896	0.00069411	0.037215075
PNPLA8	0.41256	0.121725	3.389274	0.00070078	0.037388715
RBM18	0.426689	0.125995	3.386546	0.00070779	0.0375782
TCL1B	-1.454291	0.430222	-3.380326	0.000724	0.038252431
CLEC2D	-0.367385	0.108847	-3.375242	0.00073751	0.038449568
MME	-2.427719	0.719397	-3.37466	0.00073907	0.038449568
ENSG00000273748	-0.970034	0.287806	-3.370443	0.00075047	0.038449568
ENSG00000271133	-1.172809	0.347985	-3.370291	0.00075089	0.038449568
RASSF1	0.433225	0.128557	3.369913	0.00075192	0.038449568
HIKESHI	-0.300314	0.089118	-3.369828	0.00075215	0.038449568
GIT2	0.292208	0.086716	3.369715	0.00075246	0.038449568
POLE3	-0.325387	0.096655	-3.366495	0.0007613	0.038719584
MED31	0.485503	0.144505	3.359761	0.0007801	0.039340533
DYNLT1	0.366542	0.109127	3.358856	0.00078266	0.039340533
RANBP1	-0.384017	0.11435	-3.358258	0.00078435	0.039340533
BLVRB	0.601073	0.179239	3.353475	0.00079804	0.039843198
B3GNT2	0.636379	0.18996	3.350069	0.00080791	0.040152238
PHKG1	-0.510924	0.152745	-3.344948	0.00082298	0.04071511
ABI1	0.281673	0.084272	3.342406	0.00083056	0.040903934
CD180	0.688933	0.206233	3.340556	0.00083611	0.040991837
TTYH3	-0.762129	0.228448	-3.33612	0.00084957	0.041408734
MAN1B1	0.496102	0.148754	3.335053	0.00085283	0.041408734
TSPAN18	1.658886	0.497605	3.333738	0.00085687	0.041408734
VANGL2	-2.408766	0.72262	-3.333377	0.00085799	NA
CLDN15	0.603652	0.181126	3.33278	0.00085983	0.041408734
AEBP1	-0.773865	0.232792	-3.324276	0.00088648	0.04242464
TSEN15	-0.393745	0.11847	-3.323574	0.00088872	0.04242464
PFKL	-0.329829	0.099313	-3.321126	0.00089655	0.042552718

LMO4	0.628586	0.18935	3.319706	0.00090112	0.042552718
GINM1	0.382032	0.115102	3.319084	0.00090313	0.042552718
ENSG00000170161	-0.590855	0.178117	-3.317223	0.00090917	0.042652669
IFI30	0.489469	0.147711	3.313682	0.00092076	0.043010955
TSN	-0.314937	0.095091	-3.311965	0.00092643	0.04309094
ADRB2	1.444305	0.436454	3.30918	0.0009357	0.043336653
CREBRF	0.428199	0.129588	3.304317	0.00095208	0.043908671
PAQR8	0.965309	0.292722	3.297704	0.00097479	0.044766182
FLCN	0.515815	0.156663	3.292513	0.00099296	0.045409284
UBE2J2	-0.279344	0.084905	-3.29007	0.00100162	0.045613699
ENSG00000228463	0.588979	0.179232	3.286123	0.00101577	0.046064998
MRPL40	-0.286567	0.087246	-3.284598	0.00102128	0.046122867
ENSG00000244459	0.916276	0.279618	3.276889	0.00104958	0.047204926
PPM1K	0.36092	0.110531	3.265319	0.00109341	0.048605485
IDH1	0.371797	0.113886	3.264642	0.00109603	0.048605485
TMEM273	1.328272	0.406976	3.263762	0.00109944	0.048605485
AIM2	1.829987	0.560822	3.263044	0.00110223	0.048605485
ATM	0.265042	0.081242	3.262391	0.00110477	0.048605485
PTPMT1	-0.332222	0.101856	-3.261687	0.00110751	0.048605485
GCFC2	-0.419894	0.128798	-3.260106	0.0011137	0.048680951
ZNF580	-0.329866	0.101218	-3.258964	0.0011182	0.048681818
ANAPC15	-0.32864	0.100949	-3.255513	0.00113188	0.049001128
ARL4D	1.222175	0.375494	3.254847	0.00113454	0.049001128
SPINT2	-0.333962	0.102747	-3.250333	0.0011527	0.049588817
RALGPS2	0.340866	0.104958	3.247634	0.00116369	0.0498645
IGHG3	1.294635	0.399006	3.244648	0.00117596	0.050192756
ZNF506	0.531242	0.163899	3.241284	0.00118993	0.05059045
DDB2	-0.357473	0.110355	-3.239301	0.00119823	0.050620779
CXCR5	0.303144	0.093595	3.238894	0.00119994	0.050620779
LRRC61	-0.823771	0.254506	-3.236751	0.00120899	0.050770718
HCST	0.647922	0.20029	3.234917	0.00121678	0.050770718
NHP2	-0.219483	0.067852	-3.234751	0.00121749	0.050770718
TERF2IP	0.305621	0.094535	3.232888	0.00122546	0.050889493
GAPT	0.353617	0.109415	3.231903	0.00122969	0.050889493
FKBP11	-0.345394	0.107002	-3.22792	0.00124694	0.051407894
TBXAS1	1.475507	0.458056	3.221238	0.00127638	0.052423073
PEMT	-0.558684	0.173535	-3.219437	0.00128443	0.052555225
TSEN2	-0.54239	0.168845	-3.212354	0.00131652	0.053666745
TSC22D4	0.288565	0.089883	3.210458	0.00132523	0.053820342
TOP2B	-0.326787	0.102057	-3.202018	0.00136468	0.055216454
GLS	0.352735	0.11037	3.195945	0.00139373	0.056182983
CAPG	0.629508	0.197314	3.190387	0.00142082	0.0569359
NAALADL1	0.538607	0.168844	3.18997	0.00142287	0.0569359
IKZF2	-1.147974	0.359997	-3.188845	0.00142842	0.056948606
MLX	0.317424	0.099643	3.185616	0.00144446	0.057377906
CENPS.CORT	-1.597934	0.501643	-3.185398	0.00144555	NA
GAMT	-0.430922	0.135383	-3.182982	0.00145767	0.057474981

MAN1A1	-0.498369	0.156612	-3.182181	0.0014617	0.057474981
GPR18	0.69022	0.216916	3.181974	0.00146275	0.057474981
LYST	-0.680412	0.21396	-3.180095	0.00147227	0.057640917
EIF2AK4	-0.569315	0.179108	-3.178606	0.00147985	0.0577301
APOL6	0.928926	0.292481	3.17602	0.00149311	0.05803926
INKA1	0.816122	0.257101	3.174321	0.00150188	0.058172297
KLK1	1.738649	0.548122	3.172013	0.00151386	0.058428696
TMED8	0.383947	0.121319	3.164769	0.00155206	0.059691241
LINC01869	1.102404	0.348789	3.16066	0.00157412	0.060326547
POLD2	-0.321993	0.102009	-3.156502	0.00159674	0.060978649
SRSF5	-0.316044	0.100177	-3.154871	0.00160569	0.061105999
ENSG00000173821	0.327994	0.104018	3.153242	0.00161468	0.061233921
FKBP1A	-0.242199	0.07684	-3.151983	0.00162166	0.061285205
DAPL1	-0.646932	0.205442	-3.148979	0.00163842	0.061338813
SEPTIN7	0.215355	0.06839	3.148917	0.00163877	0.061338813
G2E3	0.478877	0.152087	3.1487	0.00163999	0.061338813
CHD1	0.280883	0.08933	3.144343	0.0016646	0.062046336
FBXO10	0.766051	0.243824	3.141814	0.00167905	0.062371085
CD200R1	2.968525	0.945343	3.140155	0.00168858	NA
USF1	-0.737922	0.23521	-3.137286	0.0017052	0.063127147
CCND3	0.255384	0.081476	3.134457	0.00172173	0.063303322
PDCD4	0.504245	0.160875	3.134391	0.00172211	0.063303322
TRIM4	0.397666	0.126908	3.13349	0.00172741	0.063303322
PLEKHF2	0.349142	0.111505	3.131173	0.00174109	0.063590793
XIAP	0.388681	0.124293	3.127137	0.00176518	0.064089999
IRF5	0.536665	0.171665	3.126241	0.00177057	0.064089999
SLC39A8	-0.622535	0.199152	-3.125932	0.00177243	0.064089999
MED4	-0.243699	0.078083	-3.121022	0.00180224	0.064952412
GPR146	-1.109994	0.356254	-3.115739	0.00183485	0.065590426
PRDX2	-0.325956	0.104645	-3.114887	0.00184015	0.065590426
NAE1	-0.332721	0.106863	-3.113526	0.00184867	0.065590426
MRPS36	0.1952	0.062709	3.112763	0.00185345	0.065590426
ZNF391	1.813362	0.5827	3.111999	0.00185825	0.065590426
CDKN1B	0.344224	0.110633	3.111396	0.00186205	0.065590426
NDUFB5	0.214915	0.069074	3.111383	0.00186213	0.065590426
SIGLEC10	0.907879	0.292722	3.101501	0.00192542	0.067601032
ENSG00000261448	1.015254	0.328428	3.091257	0.00199311	0.069729316
ENSG00000282988	1.043358	0.337654	3.090021	0.00200143	0.069729316
BAZ2B	-0.539688	0.174724	-3.088807	0.00200962	0.069729316
CGAS	0.434233	0.140596	3.088504	0.00201167	0.069729316
ING5	-0.392356	0.127091	-3.087215	0.00202041	0.069810085
ATP6V0E2	-0.758153	0.245928	-3.082822	0.00205048	0.070447527
CPNE2	-1.007628	0.326873	-3.082629	0.00205181	0.070447527
MXD1	0.923766	0.299921	3.080027	0.00206982	0.070842454
LINC01588	0.753389	0.244699	3.078839	0.00207809	0.070902684
GEMIN8	-0.508848	0.16535	-3.077389	0.00208823	0.070920494
SOCS3	0.841574	0.273513	3.076901	0.00209165	0.070920494

MORC3	0.416089	0.135307	3.075137	0.00210406	0.071085225
ITM2A	1.106756	0.359996	3.074357	0.00210957	0.071085225
YWHAG	0.339718	0.110673	3.069563	0.00214372	0.071449468
GTF2B	0.414608	0.135113	3.068604	0.00215062	0.071449468
NRAS	-0.384654	0.125391	-3.067645	0.00215752	0.071449468
GFOD2	0.49015	0.159807	3.067131	0.00216124	0.071449468
TMEM208	-0.229162	0.074717	-3.067081	0.0021616	0.071449468
FCRL2	0.558254	0.182021	3.066979	0.00216234	0.071449468
DHX57	-0.509999	0.166353	-3.065771	0.00217109	0.071449468
PTPRC	0.259718	0.084722	3.065523	0.00217289	0.071449468
SEL1L3	0.678428	0.221527	3.062509	0.0021949	0.071955562
NUFIP2	0.459699	0.150241	3.059751	0.00221521	0.072046605
SPN	-0.760901	0.248706	-3.05944	0.00221751	0.072046605
CAPN12	0.545266	0.178224	3.059437	0.00221753	0.072046605
CDK2AP1	-1.018562	0.33343	-3.054798	0.00225212	0.072872947
AHDC1	0.605646	0.198297	3.054234	0.00225636	0.072872947
CASP3	-0.328064	0.10762	-3.048363	0.00230092	0.073939094
TPI1	-0.227463	0.074625	-3.048097	0.00230295	0.073939094
IFI27L2	0.384482	0.12635	3.042984	0.00234245	0.074817482
TXNDC17	-0.309225	0.10165	-3.042046	0.00234976	0.074817482
IL2RA	-0.610469	0.200687	-3.041895	0.00235094	0.074817482
CD109	-1.099535	0.361595	-3.040794	0.00235955	0.074872838
LINC02422	0.876445	0.288589	3.037003	0.00238943	0.075600573
ACP5	0.346548	0.114318	3.031449	0.00243383	0.076485254
DRAM1	-0.682549	0.225172	-3.031234	0.00243556	0.076485254
IGHG2	0.827481	0.273017	3.030873	0.00243848	0.076485254
POLR2B	-0.247149	0.081612	-3.028343	0.00245899	0.076906989
SNAI3	0.82452	0.272359	3.027323	0.0024673	0.076945782
RNF146	0.421161	0.139355	3.022214	0.00250933	0.078033114
BAZ1A	0.220574	0.073044	3.019731	0.00252999	0.078451317
PHLPP1	-0.722619	0.23943	-3.018078	0.00254383	0.078656382
SLAMF7	0.56021	0.185883	3.013772	0.00258022	0.07955546
CPSF6	-0.285945	0.095043	-3.008598	0.00262456	0.080694196
MRNIP	0.435426	0.144773	3.00764	0.00263285	0.080721022
POLR2J	-0.190177	0.063279	-3.005357	0.0026527	0.081100924
AKAP12	-1.417331	0.471873	-3.003626	0.00266784	0.081335332
ENSG00000237499	-0.541161	0.180339	-3.000796	0.00269275	0.081865695
HNRNPDL	-0.162754	0.054351	-2.994498	0.00274897	0.083341944
YIPF5	0.347507	0.116211	2.990312	0.00278693	0.084258044
CTSC	0.547221	0.183199	2.98703	0.00281702	0.084403678
KMT2C	0.288281	0.096535	2.986296	0.00282379	0.084403678
MPRIP.AS1	0.922887	0.309181	2.98494	0.00283634	0.084403678
NRROS	-0.434109	0.145441	-2.984786	0.00283777	0.084403678
MNDA	2.512035	0.842142	2.98291	0.00285522	0.084403678
ENSG00000277654	-0.70333	0.235832	-2.982339	0.00286055	0.084403678
TGM2	1.665306	0.55849	2.981802	0.00286557	0.084403678
SLFN11	0.921867	0.309177	2.981683	0.00286669	0.084403678

CTSA	0.264505	0.088714	2.981538	0.00286805	0.084403678
GPR157	0.984421	0.330187	2.981405	0.00286929	0.084403678
ENSG00000231856	-0.941988	0.316086	-2.98016	0.00288098	0.084519132
TM6SF1	-1.325592	0.44526	-2.97712	0.0029097	NA
KCNK6	0.444685	0.149402	2.976434	0.00291622	0.085322816
MTSS1	-0.483069	0.162377	-2.974985	0.00293003	0.085467544
HMGXB4	-0.324587	0.109132	-2.974265	0.00293691	0.085467544
NT5DC2	-0.875488	0.294435	-2.97345	0.00294472	0.085467544
ALOX5AP	-0.844113	0.284061	-2.971588	0.00296264	0.085758838
MAGOH	-0.259345	0.087347	-2.969119	0.00298654	0.086111307
CD68	0.98782	0.332745	2.968699	0.00299064	0.086111307
NOTCH1	0.714165	0.240775	2.966104	0.00301599	0.086329364
FLOT1	0.441415	0.14882	2.966088	0.00301614	0.086329364
GNG11	0.861992	0.290687	2.965361	0.00302328	0.086329364
PRKAG1	-0.271966	0.091735	-2.964684	0.00302994	0.086329364
PAPOLA	-0.185725	0.062674	-2.963358	0.00304302	0.086475895
FANCE	1.313977	0.443847	2.960431	0.00307209	NA
PILRB	0.651346	0.22003	2.960257	0.00307383	0.087123716
CABLES1	-1.028597	0.347762	-2.957759	0.00309884	0.087511366
ASAH1	0.29492	0.099727	2.957288	0.00310358	0.087511366
NDUFB8	-0.211612	0.071594	-2.955725	0.00311935	0.087517515
STX12	0.344468	0.116545	2.955672	0.00311988	0.087517515
ENSG00000256092	-0.486923	0.164898	-2.952872	0.00314832	0.088074005
SNRPD3	-0.212404	0.07195	-2.95213	0.0031559	0.088074005
GATM	1.053087	0.356923	2.950465	0.00317296	0.088323643
ENSG00000237596	-1.533408	0.520178	-2.94785	0.00319993	NA
PSMA4	-0.236314	0.080196	-2.946683	0.00321202	0.089182798
ADAM28	0.391702	0.13306	2.943787	0.00324223	0.089354836
PPP2R5B	0.628186	0.213431	2.943277	0.00324757	0.089354836
ENSG00000007237	-0.720346	0.244755	-2.943132	0.00324909	0.089354836
CLPX	-0.360856	0.122617	-2.942945	0.00325106	0.089354836
AIP	0.333967	0.113669	2.938064	0.00330268	0.090545117
SPTSSA	-0.28894	0.098393	-2.936594	0.00331838	0.090717158
ACADM	0.327859	0.111672	2.935917	0.00332563	0.090717158
SNHG25	-0.494429	0.168579	-2.932914	0.00335797	0.091370258
ZNHIT3	0.322919	0.110232	2.92944	0.00339573	0.092164034
TNKS1BP1	-1.460891	0.498878	-2.928351	0.00340765	NA
TICAM1	0.49031	0.167458	2.927961	0.00341193	0.092164034
GPX1	-0.241373	0.082439	-2.927905	0.00341254	0.092164034
HNRNPA1L2	-0.400936	0.136994	-2.926662	0.00342621	0.092304005
ABI3	0.493622	0.1688	2.924302	0.0034523	0.092558662
ATP6V1A	0.359967	0.123096	2.924269	0.00345267	0.092558662
CD55	-0.40689	0.13918	-2.923477	0.00346146	0.092566287
SLC16A1.AS1	0.345749	0.118336	2.921749	0.00348072	0.092752793
ENSG00000130520	-0.152287	0.052129	-2.921324	0.00348547	0.092752793
ARHGAP44	1.281321	0.438929	2.919197	0.00350935	0.093160285
NCBP2AS2	-0.266981	0.091513	-2.917403	0.0035296	0.093469883

MRPL12	-0.310519	0.106477	-2.916303	0.00354207	0.09357248
CRTC3	0.332833	0.114193	2.914648	0.0035609	0.09384225
TYROBP	1.34313	0.462303	2.905306	0.00366895	0.096456106
LINC01857	0.598692	0.20627	2.902462	0.00370242	0.097101491
TOE1	-0.450609	0.155332	-2.900938	0.00372047	0.097340479
MARCHF1	0.59172	0.204314	2.89613	0.00377796	0.098607422
OSTM1	0.476005	0.164472	2.894148	0.00380189	0.098890163
THAP3	0.414824	0.143419	2.892389	0.00382325	0.098890163
RPRD1A	-0.299454	0.103564	-2.891481	0.0038343	0.098890163
SH3BP1	0.473008	0.163592	2.891385	0.00383549	0.098890163
ENSG00000173727	0.389867	0.134855	2.891019	0.00383995	0.098890163
ENSG00000268205	0.416456	0.144095	2.890156	0.00385051	0.098890163
CYB561D2	-0.356395	0.12338	-2.888593	0.0038697	0.098890163
HSPA1B	1.028903	0.356252	2.888128	0.00387542	0.098890163
ITSN2	0.236258	0.081814	2.887759	0.00387997	0.098890163
EBNA1BP2	-0.300412	0.104056	-2.887034	0.00388893	0.098890163
CD52	0.337835	0.117029	2.886757	0.00389235	0.098890163
FKBP3	-0.248586	0.086126	-2.886315	0.00389782	0.098890163
BCL7A	-0.603221	0.209155	-2.88409	0.00392546	0.099359863
NSUN7	-1.205148	0.418289	-2.881135	0.00396246	0.100063667
SLC39A4	-0.304596	0.105835	-2.878039	0.00400156	0.100730092
HHEX	0.294764	0.102434	2.877582	0.00400736	0.100730092
TFDP2	-0.431927	0.150176	-2.876133	0.0040258	0.100907058
BCAS4	0.460764	0.160274	2.874849	0.00404221	0.100907058
PTGIR	-1.166665	0.405818	-2.874848	0.00404222	0.100907058
CALHM2	1.194381	0.415861	2.872068	0.00407796	0.101566263
LPAR5	0.527067	0.183561	2.871343	0.00408732	0.101567053
GPR171	-2.560546	0.891961	-2.870692	0.00409575	NA
ENSG00000274422	0.538295	0.187595	2.869454	0.00411181	0.101943001
LY9	0.335801	0.117149	2.866442	0.00415114	0.102424692
FIBP	-0.215919	0.075328	-2.866385	0.00415189	0.102424692
IL13RA1	-0.552111	0.192655	-2.865807	0.00415947	0.102424692
ENSG00000277007	1.396793	0.487463	2.865433	0.0041644	NA
ZNF827	0.650009	0.227053	2.862811	0.00419901	0.103164869
SIGLEC5	1.531081	0.535134	2.861117	0.00422151	0.103484064
CREM	-0.333704	0.1168	-2.857058	0.00427588	0.104230081
CNR1	1.055477	0.369436	2.856995	0.00427672	0.104230081
YARS	-0.300193	0.105098	-2.856311	0.00428595	0.104230081
BCL10	0.295582	0.103495	2.855993	0.00429025	0.104230081
C12orf65	0.275893	0.096637	2.854946	0.00430442	0.104341431
TRIR	0.144698	0.050707	2.853594	0.00432278	0.104553613
PARPBP	-0.775915	0.271983	-2.852806	0.00433351	0.104580738
ADSL	-0.234819	0.082366	-2.850938	0.00435905	0.104964272
SCRN2	-0.385334	0.135368	-2.846559	0.00441946	0.105963639
IFI16	0.24285	0.085315	2.846518	0.00442002	0.105963639
ITGB7	0.625871	0.21995	2.845511	0.00443403	0.105965257
FABP5	-0.345447	0.121443	-2.844523	0.0044478	0.105965257

CHRAC1	-0.249343	0.087665	-2.844269	0.00445134	0.105965257
CALM3	-0.345769	0.12164	-2.842564	0.00447523	0.105965257
ENSG00000197302	0.346292	0.121843	2.842129	0.00448133	0.105965257
AKAP9	0.27198	0.095697	2.842102	0.00448172	0.105965257
LMCD1	2.059185	0.724589	2.841868	NA	NA
LGALS9	-0.437763	0.154053	-2.841638	0.00448824	0.105965257
CLK1	0.384679	0.135437	2.840288	0.00450728	0.106184573
DNAJC5B	1.817025	0.640212	2.838162	0.00453741	0.106663402
GTF3C6	-0.250734	0.088416	-2.83584	0.00457053	0.107210509
HAUS3	0.522862	0.184663	2.831444	0.00463383	0.108461621
HIST3H2A	1.037825	0.366772	2.829618	0.00466036	0.108706066
GALE	0.587611	0.207684	2.829351	0.00466425	0.108706066
CYTOR	0.802714	0.283994	2.82652	0.00470568	0.109437233
NDUFV3	0.306736	0.108627	2.82374	0.00474669	0.109914953
SENP2	-0.376443	0.133344	-2.823094	0.00475627	0.109914953
IGHG4	1.675629	0.593547	2.823077	0.00475652	0.109914953
SRFBP1	0.299359	0.106068	2.822328	0.00476763	0.109938416
TOMM5	-0.290584	0.103002	-2.821154	0.00478513	0.110108495
HOXB7	3.212599	1.139324	2.819741	0.00480625	NA
TMEM60	0.353257	0.125283	2.819662	0.00480743	0.11038824
SNAP23	-0.263468	0.093478	-2.81851	0.00482472	0.110551988
RGMB	0.969383	0.344159	2.816673	0.0048524	0.110754271
SERINC1	0.428549	0.152173	2.816196	0.0048596	0.110754271
SMIM4	-0.36521	0.129696	-2.815901	0.00486407	0.110754271
ADCK2	0.528263	0.187681	2.814689	0.00488245	0.110940692
CNIH4	0.284952	0.101408	2.809958	0.0049548	0.112350111
PEX10	-0.452887	0.161222	-2.809094	0.00496811	0.112417631
C1GALT1	0.429176	0.15283	2.808189	0.0049821	0.112500458
COX7A2L	0.171392	0.061073	2.806357	0.00501052	0.112712825
SKIL	0.383409	0.136627	2.806247	0.00501222	0.112712825
SYBU	2.823791	1.006601	2.805274	0.00502738	NA
ZNF79	1.20111	0.428472	2.80324	0.0050592	0.113300316
ZC3H12D	-0.519966	0.185492	-2.803168	0.00506033	0.113300316
MRPL1	-0.232849	0.083122	-2.80128	0.00509004	0.113300316
NFATC2	0.493118	0.176033	2.801277	0.00509008	0.113300316
PODXL2	-0.804734	0.287276	-2.801257	0.0050904	0.113300316
IVD	0.516398	0.184417	2.80017	0.00510756	0.11345047
IGHA1	0.532557	0.190668	2.793107	0.00522045	0.115721699
SNHG30	-0.373464	0.133752	-2.792212	0.00523491	0.115806451
NSMCE4A	-0.267356	0.095814	-2.790357	0.00526499	0.116107018
ARL6IP6	0.392486	0.140673	2.79006	0.00526983	0.116107018
HERC4	0.318632	0.11434	2.786698	0.0053248	0.117081122
MTHFR	0.442247	0.158862	2.783839	0.00537197	0.117880079
TMEM147	-0.190314	0.068412	-2.781882	0.00540447	0.118296406
MIDN	0.392968	0.141285	2.781389	0.00541268	0.118296406
FGFBP2	-2.639702	0.949386	-2.780432	0.00542867	NA
TYW3	0.360485	0.129706	2.779253	0.0054484	0.118838489

VPREB1	-2.015525	0.725765	-2.777103	0.00548457	0.119388158
HNRNPA0	-0.179594	0.064749	-2.773687	0.0055425	0.120310316
RIMKLB	-0.595519	0.214733	-2.773303	0.00554904	0.120310316
RENBP	0.489896	0.176702	2.772445	0.00556368	0.120387954
ZNF189	0.73835	0.266486	2.770685	0.00559385	0.120781503
AKAP13	0.266755	0.096298	2.770091	0.00560407	0.120781503
SLC2A14	-3.578463	1.293368	-2.766779	0.00566131	NA
MDH2	-0.205997	0.074465	-2.766375	0.00566833	0.121925109
SSR4	-0.127114	0.045965	-2.765474	0.00568401	0.122021135
SLC9B2	-0.356629	0.129055	-2.763381	0.00572059	0.122564767
DTD2	-0.421648	0.1527	-2.761293	0.00575729	0.123108772
ENSG00000228106	-0.292012	0.105807	-2.759866	0.00578252	0.123185072
ZMAT2	-0.205612	0.074502	-2.75981	0.0057835	0.123185072
UBXN6	-0.245202	0.088879	-2.758836	0.00580077	0.12322371
MICAL1	-0.356528	0.12925	-2.758431	0.00580795	0.12322371
ARHGAP25	0.342627	0.124274	2.757022	0.00583304	0.123289566
RNASEH2C	-0.175438	0.063634	-2.756984	0.00583371	0.123289566
CTBP1	0.259199	0.094044	2.756161	0.00584843	0.12336102
SMIM29	0.254558	0.092399	2.754995	0.00586931	0.12348711
TRIM52	0.424453	0.154123	2.753993	0.00588731	0.12348711
CLK3	0.320354	0.116364	2.753031	0.00590462	0.12348711
IL7	0.539462	0.195966	2.752843	0.00590802	0.12348711
ALDH16A1	-0.400819	0.145634	-2.752229	0.00591911	0.12348711
RASAL1	-0.752294	0.273363	-2.751999	0.00592327	0.12348711
FAM91A1	0.399644	0.145319	2.750121	0.00595733	0.12348711
RBM8A	-0.148003	0.053822	-2.749868	0.00596193	0.12348711
MTHFD2L	-0.398312	0.144864	-2.749562	0.0059675	0.12348711
IGHD	-0.561346	0.20416	-2.749542	0.00596786	0.12348711
PF4	3.819515	1.389779	2.74829	0.0059907	0.123660509
NDUFB7	-0.177497	0.064595	-2.747838	0.00599897	0.123660509
PPIL4	0.380577	0.138782	2.742272	0.00610158	0.125538036
ENTPD1	0.611725	0.22344	2.737757	0.00618598	0.127034284
GLIPR2	0.517707	0.189151	2.737012	0.00620001	0.127082645
WWOX	-0.59747	0.218417	-2.735459	0.00622934	0.127443865
MRPS34	-0.215028	0.078736	-2.730988	0.00631448	0.128779821
ITGB3BP	-0.353334	0.129389	-2.730788	0.0063183	0.128779821
A4GALT	1.04391	0.382719	2.727614	0.00637943	0.12978254
FXR1	0.206351	0.075721	2.72516	0.00642703	0.130507141
ACTN1	1.784665	0.655084	2.724329	0.00644322	NA
EIF3B	-0.273384	0.100349	-2.724325	0.00644331	0.130593963
EVI2A	0.392817	0.144243	2.723298	0.00646337	0.130757083
LY96	0.395527	0.145303	2.722085	0.00648714	0.130783268
LINC01781	2.015826	0.740566	2.722006	0.0064887	0.130783268
ZBTB32	1.428501	0.52543	2.718725	0.00655341	0.131843415
ZNF704	-1.065388	0.392214	-2.716347	0.00660067	0.132386591
GRN	0.249027	0.091711	2.715338	0.00662082	0.132386591
DMAC2L	-0.244325	0.090001	-2.714707	0.00663345	0.132386591

PIGF	0.440787	0.162438	2.713579	0.00665607	0.132386591
NHSL2	-0.441899	0.162883	-2.712982	0.00666808	0.132386591
ENSG00000268403	-0.548348	0.202149	-2.712598	0.0066758	0.132386591
ARID1A	0.252002	0.092903	2.712516	0.00667746	0.132386591
HLA.C	-0.426295	0.157159	-2.712503	0.00667771	0.132386591
CRIP2	2.000822	0.738468	2.709422	0.00674005	NA
SAMD4A	0.930207	0.343438	2.708512	0.00675857	0.133482607
ZC3H7B	-0.404322	0.149327	-2.707633	0.00677649	0.133482607
GTF3C2	-0.322704	0.119189	-2.707501	0.00677919	0.133482607
GIHCG	-0.377141	0.139302	-2.707361	0.00678206	0.133482607
ENSG00000273141	-2.346237	0.866645	-2.707263	0.00678404	NA
ZNF557	0.388199	0.143459	2.705994	0.00681004	0.133791411
PDCD7	0.411573	0.152174	2.704626	0.00683813	0.134064575
TNRC6C	0.57742	0.213571	2.703637	0.00685852	0.134064575
CREB3L2	-0.461694	0.170775	-2.703522	0.00686089	0.134064575
IFIT2	0.855645	0.316613	2.702492	0.00688218	0.13423958
ENSG00000272758	0.798884	0.2959	2.699842	0.00693725	0.135071558
PTGS1	0.726321	0.26913	2.698768	0.00695967	0.135266156
MRPS26	-0.194726	0.072191	-2.697386	0.00698862	0.135586747
UPP1	0.777786	0.288414	2.696768	0.00700161	0.135597054
OGFRL1	-0.472763	0.175403	-2.695294	0.00703266	0.135956428
COL9A3	-0.963205	0.357762	-2.692309	0.00709591	0.136935923
TIMM8B	-0.218384	0.08115	-2.691104	0.00712159	0.137188282
HDAC2	0.235091	0.08744	2.688594	0.00717536	0.137979878
GLIDR	-0.609097	0.226713	-2.686639	0.0072175	0.138545345
ENSG00000277476	1.682267	0.626571	2.68488	0.00725559	NA
ENSG00000275964	-0.691639	0.25795	-2.681288	0.00733393	0.140532604
PSMB3	-0.191027	0.071303	-2.679103	0.00738197	0.14108733
CORO1A	0.2819	0.105234	2.678793	0.00738881	0.14108733
MYBBP1A	-0.416186	0.155507	-2.676323	0.00744349	0.141882498
LETM1	-0.23693	0.088604	-2.674048	0.00749417	0.142598765
HMGB3	-0.691481	0.258729	-2.672606	0.00752647	0.142804944
TLR7	0.830027	0.310597	2.672362	0.00753194	0.142804944
CBWD2	-0.469113	0.17559	-2.671638	0.0075482	0.142804944
BEND4	0.62042	0.23226	2.671226	0.00755748	0.142804944
STMP1	-0.208163	0.07795	-2.670482	0.00757425	0.142847251
VPS13C	0.272	0.101891	2.669512	0.00759616	0.142847251
ZNF567	0.390269	0.146278	2.667988	0.0076307	0.142847251
TMEM177	-0.55307	0.207393	-2.666767	0.00765846	0.142847251
SRSF11	0.2335	0.087566	2.666546	0.0076635	0.142847251
IGHG1	1.230969	0.461675	2.666308	0.00766894	0.142847251
RAB3GAP2	0.41596	0.15601	2.666248	0.0076703	0.142847251
NUDCD3	-0.308357	0.115656	-2.666154	0.00767246	0.142847251
APRT	-0.145978	0.054757	-2.665918	0.00767784	0.142847251
ARMT1	0.507397	0.190419	2.664644	0.007707	0.143144955
PSMB1	-0.211928	0.079578	-2.663142	0.00774147	0.143540311
FOXP4	0.579676	0.217949	2.659691	0.00782125	0.144703821

ENSG00000258056	0.74439	0.280005	2.658487	0.00784924	0.144703821
MSI2	-0.470468	0.17697	-2.658464	0.00784978	0.144703821
KLF6	0.330515	0.124363	2.657661	0.0078685	0.144703821
TMEM179B	-0.235551	0.088634	-2.657567	0.0078707	0.144703821
SLC9A3R1	0.39983	0.150523	2.656282	0.00790075	0.145011483
WDR70	-0.315216	0.118757	-2.654295	0.00794744	0.145143499
STXBP4	-0.751793	0.283259	-2.654085	0.00795237	0.145143499
ZBTB25	0.357084	0.13456	2.653721	0.00796097	0.145143499
KIF5B	0.247898	0.093416	2.653707	0.00796129	0.145143499
PARP15	0.395097	0.148937	2.652777	0.00798325	0.145300567
VSIG10L	1.944709	0.733584	2.650969	0.00802613	NA
ODF3B	-0.479896	0.181115	-2.649676	0.0080569	0.146381567
AKIRIN2	0.241015	0.090988	2.648868	0.00807618	0.146381567
ATP5MC3	-0.26071	0.098434	-2.648583	0.008083	0.146381567
CDCA7L	-0.408438	0.154433	-2.644754	0.00817504	0.147581605
ZNF350	-0.340718	0.128831	-2.644698	0.00817638	0.147581605
TCTN1	-0.879453	0.332747	-2.643011	0.00821725	0.147971301
NDUFAF8	-0.200264	0.075785	-2.64254	0.00822867	0.147971301
BCL2L13	-0.326805	0.12369	-2.642125	0.00823876	0.147971301
RASSF3	0.305845	0.115905	2.638753	0.00832116	0.149205093
CD1D	0.632491	0.239821	2.637347	0.00835574	0.149292306
TUT7	0.344687	0.130709	2.637053	0.00836298	0.149292306
HES6	-0.728287	0.276192	-2.636883	0.00836717	0.149292306
UFD1	-0.262211	0.099492	-2.635488	0.00840163	0.1496618
ARL11	0.586558	0.223055	2.629651	0.00854725	0.151913356
PHF19	-0.57885	0.220153	-2.629306	0.00855594	0.151913356
RCBTB1	-0.678867	0.258331	-2.627893	0.00859156	0.152297272
ACP1	-0.157244	0.059856	-2.627037	0.0086132	0.152432582
ZNHIT2	-0.363008	0.138295	-2.624886	0.00866781	0.152705037
ENSG00000268400	-1.205398	0.459248	-2.624723	0.00867195	0.152705037
COX17	0.327034	0.124616	2.624339	0.00868172	0.152705037
PRICKLE1	0.771523	0.294001	2.624222	0.00868471	0.152705037
EPS15	-0.312013	0.118935	-2.623399	0.00870572	0.152827566
IKZF1	-0.251169	0.09588	-2.619609	0.00880307	0.154287582
ENSG00000274536	-0.607384	0.232023	-2.617773	0.00885056	0.154870589
IL4R	-0.418254	0.159937	-2.615115	0.00891974	0.155664241
ZNF747	-0.422674	0.161659	-2.614598	0.00893324	0.155664241
TPM1	0.601327	0.230007	2.614385	0.00893882	0.155664241
PTPRS	1.299226	0.497218	2.612991	0.00897537	0.155773287
SUMO1	-0.150843	0.057742	-2.612363	0.00899187	0.155773287
RPLP2	0.134009	0.051339	2.610298	0.00904633	0.155773287
PYURF	-0.186765	0.071565	-2.609727	0.00906145	0.155773287
XRRA1	-0.716451	0.274586	-2.60921	0.00907515	0.155773287
RASSF2	0.338692	0.129834	2.608644	0.00909018	0.155773287
INTS6	-0.237649	0.091101	-2.608639	0.0090903	0.155773287
FAAP24	-0.654178	0.250775	-2.608629	0.00909057	0.155773287
ZNF749	-1.343054	0.514938	-2.608189	0.00910228	0.155773287

NDUFC2	-0.260392	0.099838	-2.608148	0.00910337	0.155773287
CNP	0.323403	0.124009	2.607904	0.00910986	0.155773287
HIVEP3	-0.54982	0.21085	-2.607642	0.00911683	0.155773287
C16orf74	-0.489808	0.187907	-2.606654	0.00914317	0.155978496
PM20D2	0.505336	0.193991	2.604944	0.00918893	0.156480562
ATXN2	0.35045	0.13461	2.603453	0.00922898	0.156480562
H3F3B	-0.137925	0.052987	-2.602994	0.00924135	0.156480562
FRG1	-0.183836	0.070633	-2.602677	0.00924992	0.156480562
ENSG00000264443	0.699829	0.268965	2.601931	0.00927004	0.156480562
SAMD9L	0.427152	0.164178	2.601751	0.00927491	0.156480562
U2SURP	-0.171928	0.066109	-2.600676	0.00930403	0.156480562
MAP3K1	0.332755	0.127992	2.599817	0.00932734	0.156480562
LACTB2	0.380433	0.146333	2.599769	0.00932864	0.156480562
ENSG00000254397	1.062596	0.408762	2.599547	0.00933468	0.156480562
ITFG1	-0.317718	0.122239	-2.599156	0.00934532	0.156480562
NIPAL2	0.731787	0.281583	2.598827	0.00935428	0.156480562
CHTOP	0.167985	0.064655	2.598182	0.00937188	0.156480562
MVB12A	-0.29026	0.11172	-2.598109	0.00937388	0.156480562
SDSL	1.217314	0.469015	2.595468	0.00944623	0.157446755
ENSG00000070423	-0.261132	0.100637	-2.594802	0.00946454	0.157510803
PWWP2B	0.951105	0.366785	2.593085	0.00951193	0.158027513
ADPRH	0.794367	0.306395	2.592626	0.00952463	0.158027513
C17orf49	-0.368065	0.142015	-2.591734	0.00954936	0.158196654
NEMP1	0.605112	0.233603	2.590346	0.00958796	0.158594786
METTL1	-0.390984	0.151053	-2.588384	0.00964275	0.159259076
CD3D	-2.441079	0.94353	-2.587178	0.00967657	NA
DNAJB4	0.564918	0.218426	2.586308	0.00970102	0.159835816
SMKR1	-0.500141	0.193396	-2.586094	0.00970705	0.159835816
RFX1	0.373275	0.14447	2.583754	0.00977315	0.160461946
CEP104	0.414367	0.160377	2.583704	0.00977456	0.160461946
ARID3B	0.582522	0.225513	2.583091	0.00979195	0.160505469
HLA.DQA1	0.395143	0.153045	2.581874	0.00982653	0.160830038
ELP5	-0.38953	0.15094	-2.580698	0.00986007	0.161136564
PCNX4	0.342688	0.132864	2.579246	0.00990164	0.161573302
ARMCX1	-1.459737	0.566256	-2.577877	0.00994094	NA
RUNX2	1.307329	0.507237	2.577354	0.00995599	0.162205817
OXSM	0.548403	0.212818	2.576861	0.0099702	0.162205817
SPOP	0.284479	0.110593	2.572306	0.01010237	0.164083645
UAP1L1	0.887678	0.345152	2.571846	0.01011578	0.164083645
IER3IP1	-0.199029	0.077492	-2.568384	0.01021738	0.165322787
ERLIN2	-0.513128	0.1998	-2.568209	0.01022255	0.165322787
PLCB2	0.70612	0.275093	2.566844	0.01026288	0.165728691
MIR181A1HG	-0.666649	0.25985	-2.565515	0.01030228	0.166107227
RPA3	-0.194609	0.075884	-2.564558	0.01033074	0.166107227
PSMA6	-0.267148	0.104171	-2.564512	0.0103321	0.166107227
HPS3	0.42328	0.165101	2.56376	0.01035452	0.166222042
DNAJC30	-0.27515	0.10735	-2.563113	0.01037384	0.166287043

ENSG00000272277	-1.170768	0.456902	-2.562407	0.01039495	0.166380292
CCDC121	2.502368	0.977453	2.56009	0.01046452	NA
FOLR2	1.769788	0.691378	2.5598	0.01047325	NA
ARMCX3	0.27105	0.106045	2.555993	0.01058852	0.169025683
MTRF1	0.486485	0.190368	2.5555	0.01060354	0.169025683
ENSG00000275464	-1.29125	0.505304	-2.555392	0.01060681	0.169025683
ENSG00000229728	-1.544646	0.604631	-2.554691	0.01062822	NA
C8orf82	-0.920965	0.360542	-2.554395	0.01063725	0.169262943
STYX	0.412077	0.161397	2.553187	0.01067422	0.169603239
DHTKD1	-0.368052	0.144309	-2.550447	0.01075848	0.170692914
CFD	1.735498	0.680604	2.549938	0.01077419	NA
PBX4	1.088538	0.427084	2.548767	0.01081046	0.17102892
ICMT	-0.577014	0.226391	-2.548746	0.01081109	0.17102892
CENPU	-0.658069	0.258281	-2.547884	0.01083786	0.171203508
RPS28	0.083048	0.032607	2.54693	0.01086754	0.171423561
PPP2R5A	0.397861	0.156309	2.545344	0.01091701	0.171954697
GCSAM	-0.650631	0.256039	-2.541137	0.01104926	0.173786324
BCAP31	-0.222655	0.0877	-2.538834	0.01112226	0.17468213
RPGRIP1L	-0.638574	0.251738	-2.536665	0.0111914	0.175514688
TOB1	0.497788	0.196313	2.53568	0.01122291	0.175613187
JMJD4	-0.3381	0.133359	-2.535255	0.01123657	0.175613187
NCOA3	0.280505	0.110655	2.534958	0.01124609	0.175613187
MIR155HG	0.403296	0.159224	2.532895	0.0113125	0.176397195
FAM184B	-1.730781	0.683497	-2.532244	0.01133351	0.176471962
KLK2	2.049721	0.810396	2.529281	0.01142964	NA
PSMB10	-0.228253	0.090244	-2.529274	0.01142986	0.177703874
ZNF668	0.509937	0.201652	2.528801	0.01144528	0.177703874
PLEKHO2	0.818926	0.323978	2.527719	0.01148062	0.177982281
FLI1	0.339322	0.134278	2.527015	0.01150366	0.177982281
CMSS1	-0.235849	0.093341	-2.526752	0.01151227	0.177982281
RFTN1	-0.272714	0.107958	-2.526106	0.01153346	0.178056921
LY86.AS1	2.248861	0.890618	2.525056	0.011568	NA
PDE4B	0.274566	0.108765	2.524391	0.01158989	0.178674657
TAF1A	0.519586	0.206023	2.52198	0.01166965	0.179649878
PPBP	2.500724	0.991709	2.521632	0.01168117	NA
MSMP	-0.933922	0.370388	-2.521472	0.0116865	0.179655202
BTG2	0.23137	0.09181	2.520084	0.0117327	0.179800265
INTS10	-0.242339	0.096183	-2.519552	0.01175045	0.179800265
NAB1	0.535384	0.212496	2.519504	0.01175204	0.179800265
BAG1	-0.205895	0.08173	-2.519205	0.01176202	0.179800265
SPIB	0.176736	0.070174	2.518546	0.01178405	0.179884464
PIAS2	-0.296739	0.11793	-2.516223	0.01186202	0.180591
UST	0.887116	0.352594	2.515968	0.01187061	0.180591
BBX	0.210842	0.083811	2.515686	0.01188011	0.180591
NADSYN1	-0.287444	0.114305	-2.514717	0.01191279	0.180835125
NUBP2	-0.189601	0.075469	-2.512309	0.01199441	0.181218555
MIF.AS1	-0.461719	0.183801	-2.512052	0.01200315	0.181218555

ASPH	0.740717	0.294891	2.511835	0.01201053	0.181218555
HMGB2	-0.253368	0.100877	-2.511651	0.01201678	0.181218555
PDE4DIP	-0.387581	0.154333	-2.51133	0.01202773	0.181218555
MYL6B	-0.276933	0.110287	-2.51103	0.01203795	0.181218555
NUDT8	-0.608676	0.242535	-2.509639	0.01208546	0.181682561
TMEM154	0.465664	0.185643	2.508379	0.01212867	0.181920139
GADD45G	-0.842329	0.335933	-2.507428	0.01216134	0.181920139
TMEM187	0.771817	0.307899	2.506724	0.01218557	0.181920139
RHOBTB2	1.076577	0.429561	2.506224	0.01220283	0.181920139
ZBTB7B	0.442231	0.176455	2.506192	0.01220395	0.181920139
ZSCAN22	0.938886	0.374656	2.505994	0.01221076	0.181920139
WDR89	0.385545	0.153862	2.505777	0.01221827	0.181920139
FAM76B	0.338578	0.135173	2.504785	0.0122526	0.182182021
ENSG00000249684	2.060176	0.82273	2.504074	0.01227724	NA
HMMR	1.087422	0.434282	2.503953	0.01228143	NA
MGME1	0.337666	0.135005	2.501137	0.01237952	0.183678529
ENSG00000167414	-0.969877	0.387808	-2.500923	0.01238699	0.183678529
RHOBTB1	-2.221979	0.888472	-2.5009	0.01238781	NA
SMARCE1	-0.184485	0.073818	-2.499192	0.01244768	0.184327259
ASB2	1.436665	0.575031	2.498411	0.01247514	0.184482953
CHMP5	0.23546	0.094318	2.496456	0.01254414	0.185251532
MBLAC1	-1.224827	0.491214	-2.493469	0.01265016	0.186564115
RSBN1	0.276603	0.110993	2.492079	0.01269979	0.187042609
ZNF420	0.432136	0.17362	2.488975	0.01281118	0.188428222
TTI2	0.495703	0.199415	2.485793	0.01292632	0.189865162
IRF2BPL	0.399072	0.160607	2.484783	0.01296305	0.190148089
EXOG	-0.315479	0.127108	-2.481965	0.01306602	0.191400422
SLC9A9	-0.611432	0.246452	-2.480935	0.01310382	0.191586162
CENPB	-0.445141	0.179445	-2.480661	0.0131139	0.191586162
DGCR6	-1.505	0.607114	-2.478941	0.01317731	NA
GCNT2	0.55493	0.223922	2.478222	0.01320389	0.192642204
AUP1	-0.146328	0.059137	-2.474378	0.01334683	0.194368899
REEP4	0.555059	0.224355	2.474026	0.01335998	0.194368899
ZNF616	0.868461	0.351113	2.473454	0.01338141	0.194368899
PPP1R16B	0.403422	0.163124	2.473107	0.0133944	0.194368899
TAF13	0.459115	0.185686	2.472529	0.01341606	0.194368899
AKAP17A	0.248973	0.100716	2.472023	0.0134351	0.194368899
ARL4A	-0.383464	0.155158	-2.471447	0.01345677	0.194368899
BLM	0.525023	0.212454	2.471225	0.0134651	0.194368899
C9orf16	-0.228832	0.092637	-2.470196	0.01350391	0.194670874
CFAP298	-0.268028	0.108554	-2.469067	0.0135466	0.195028009
RACGAP1	0.765263	0.31025	2.466606	0.01364003	0.196113778
ENSG00000278932	1.923096	0.780236	2.464763	0.0137104	NA
RIN3	0.555621	0.225458	2.464407	0.01372403	0.196920389
KCNN4	0.731511	0.296857	2.46419	0.01373232	0.196920389
C6orf226	-0.433474	0.175951	-2.463605	0.01375475	0.196982562
AHI1	-0.374095	0.15192	-2.462442	0.01379943	0.197362715

TRMT6	-0.396373	0.161065	-2.460946	0.01385714	0.197927974
ATP5ME	-0.179212	0.072858	-2.459743	0.01390364	0.198294587
FBXL17	0.507753	0.206459	2.459341	0.01391925	0.198294587
PPCS	-0.191214	0.077786	-2.458195	0.01396372	0.198418986
TTC9C	0.285951	0.116327	2.458177	0.01396444	0.198418986
BACE2	1.621129	0.659657	2.457533	0.01398949	NA
ENSG00000273338	-0.441985	0.179969	-2.455889	0.01405365	0.199426185
BAG4	0.406111	0.165453	2.454541	0.01410647	0.19991506
ENSG00000176320	2.192084	0.893158	2.454308	0.01411561	NA
ZNF284	0.723975	0.295122	2.453138	0.01416159	0.200280014
ENSG00000188242	-0.693042	0.282534	-2.45295	0.01416902	0.200280014
HDX	1.097874	0.447907	2.451121	0.01424122	0.200951434
CERS4	-0.273239	0.111489	-2.450812	0.01425345	0.200951434
METAP2	-0.160181	0.065394	-2.44948	0.01430628	0.201435394
NDFIP2	-0.816799	0.333983	-2.44563	0.01445994	0.203335925
SDHAF3	-0.223449	0.091416	-2.444324	0.01451239	0.203738151
SLC25A5	-0.169442	0.069341	-2.443594	0.0145418	0.203738151
CD27	1.249254	0.511252	2.443522	0.0145447	0.203738151
CRLF3	0.244995	0.100307	2.442458	0.01458762	0.204076722
MPP1	-0.647211	0.2651	-2.441387	0.01463099	0.20440473
TMEM156	0.373798	0.153136	2.440951	0.01464863	0.20440473
CNBP	-0.198843	0.081497	-2.439873	0.01469244	0.204492364
MTCH2	-0.250632	0.102724	-2.439872	0.01469249	0.204492364
C7orf26	-0.409975	0.168176	-2.437774	0.014778	0.205419798
COL4A4	1.989942	0.816578	2.436928	0.01481265	NA
ENSG00000262089	0.873271	0.358428	2.436391	0.01483465	0.205944267
CHTF18	-0.549726	0.225677	-2.435895	0.01485501	0.20596423
FSD1	-1.19947	0.492519	-2.435376	0.01487633	NA
NFE2L1	0.3092	0.126982	2.434997	0.01489191	0.2061739
GCC2	0.246813	0.101401	2.434033	0.01493164	0.2061739
SLF2	0.310426	0.127551	2.433747	0.01494343	0.2061739
FCGR2A	2.511286	1.031876	2.43371	0.01494498	NA
SMC5	-0.242846	0.099814	-2.43298	0.01497512	0.2061739
GNPDA1	0.340383	0.139906	2.432944	0.01497663	0.2061739
ENSG00000265778	-0.999918	0.41102	-2.432771	0.01498379	0.2061739
CD48	0.224889	0.09249	2.431493	0.01503674	0.206641206
YWHAQ	-0.191062	0.078668	-2.428704	0.01515289	0.207061635
ARHGAP9	-0.247544	0.101942	-2.428273	0.01517091	0.207061635
TOMM7	0.166739	0.068679	2.427821	0.01518985	0.207061635
PIGT	-0.262534	0.108156	-2.42737	0.01520875	0.207061635
POLD4	0.23882	0.098389	2.4273	0.01521167	0.207061635
RPS9	0.154049	0.063466	2.427277	0.01521263	0.207061635
RASGEF1B	-0.493497	0.20337	-2.426593	0.01524134	0.207061635
PSMC3IP	0.571455	0.235524	2.42631	0.01525323	0.207061635
LGALS3	0.782886	0.322672	2.42626	0.01525534	0.207061635
APBB1	0.828546	0.341499	2.426207	0.01525757	0.207061635
ERP29	0.145845	0.060132	2.425402	0.01529145	0.207201175

TMEM134	0.165877	0.068401	2.425058	0.01530593	0.207201175
ITGA10	0.868764	0.358464	2.423574	0.0153686	0.207674025
TAGAP	-0.514927	0.212488	-2.423328	0.01537902	0.207674025
SLCO3A1	-1.545305	0.63786	-2.42264	0.01540818	NA
MCM7	-0.309483	0.127855	-2.420569	0.01549625	0.208997812
CTSB	0.26225	0.108458	2.417979	0.01560698	0.210026276
GLI4	-0.953225	0.39426	-2.417754	0.01561662	0.210026276
VAT1	0.387218	0.160192	2.417215	0.01563976	0.210026276
TRAM1	-0.168327	0.069643	-2.416984	0.0156497	0.210026276
RGL1	-1.234665	0.511177	-2.415339	0.01572056	NA
PCBP4	0.606615	0.251647	2.410576	0.01592735	0.213489314
RFC4	0.343542	0.142549	2.40999	0.01595298	0.213568428
SNAP29	0.286317	0.118826	2.409543	0.0159725	0.213568428
ZNF667.AS1	0.324697	0.134822	2.408337	0.0160254	0.214004281
SRD5A3	0.479742	0.199237	2.407903	0.01604442	0.214004281
ENSG00000237491	-0.583535	0.2424	-2.407324	0.0160699	0.214081746
LINC01250	-2.766859	1.149611	-2.406779	0.01609389	NA
CHCHD3	-0.265076	0.110215	-2.405078	0.01616903	0.215139084
ENSG00000276449	1.048697	0.436256	2.403858	0.01622307	0.215530326
SLC29A2	-0.588059	0.244687	-2.403315	0.0162472	0.215530326
ENSG00000089127	0.66073	0.275035	2.402348	0.01629021	0.215530326
GLRX	0.385678	0.160614	2.401277	0.01633798	0.215530326
ING3	-0.326934	0.136152	-2.401252	0.0163391	0.215530326
SEPTIN6	0.152384	0.06346	2.401244	0.01633946	0.215530326
LY6E	0.252344	0.105137	2.400151	0.01638829	0.215530326
PCGF5	-0.251931	0.104969	-2.400061	0.01639232	0.215530326
ITM2C	-0.369723	0.154048	-2.400046	0.01639302	0.215530326
STT3B	-0.338552	0.141092	-2.399514	0.01641684	0.215530326
ZFAND6	0.351161	0.14639	2.398807	0.01644859	0.215530326
HPSE	-2.055749	0.857031	-2.398686	0.016454	NA
ETFRF1	0.313782	0.130823	2.39853	0.01646101	0.215530326
SAR1B	0.265649	0.110757	2.398498	0.01646248	0.215530326
RBM38	-0.315403	0.131516	-2.398204	0.01647567	0.215530326
ENSG00000262429	-1.087831	0.453698	-2.397698	0.01649848	NA
MAGEH1	-0.407146	0.169937	-2.395863	0.01658129	0.216465864
PTTG1IP	0.398566	0.1664	2.39522	0.01661041	0.216465864
NDUFA7	-0.279841	0.116859	-2.394699	0.01663401	0.216465864
EML4	0.219004	0.091458	2.394578	0.01663951	0.216465864
RANGRF	-0.231037	0.09649	-2.394421	0.01664663	0.216465864
ANGPTL6	1.474415	0.616004	2.393518	0.01668768	NA
ZNF43	-0.308936	0.129092	-2.393143	0.01670471	0.216722989
LENG1	0.271828	0.113593	2.392994	0.0167115	0.216722989
FUT7	1.705627	0.712839	2.392726	0.01672374	NA
BLOC1S2	-0.177242	0.074077	-2.392673	0.01672614	0.216722989
LINC02193	-0.732803	0.306491	-2.390942	0.01680519	0.217488288
B4GALT1.AS1	-0.981338	0.411189	-2.386585	0.01700567	0.219759516
TMEM161A	-0.386871	0.162125	-2.386252	0.01702107	0.219759516

DUSP12	-0.250611	0.105057	-2.385484	0.01705665	0.219910123
TESMIN	-2.337267	0.979865	-2.385295	0.01706545	NA
TXLNB	1.014102	0.425213	2.384928	0.01708247	0.219910123
ZNF879	-0.529627	0.222094	-2.384694	0.01709335	0.219910123
GNL3L	-0.293324	0.123197	-2.380931	0.01726894	0.221671608
GANC	0.446424	0.187503	2.380887	0.017271	0.221671608
ACSL3	-0.310835	0.130619	-2.379715	0.01732602	0.222115854
MMAB	-0.15846	0.066643	-2.377734	0.0174194	0.222683932
SAR1A	-0.245148	0.103107	-2.377613	0.0174251	0.222683932
DUSP14	0.446502	0.187805	2.377473	0.01743171	0.222683932
STAG3	0.458965	0.193149	2.376225	0.0174908	0.223176886
OTUD6B.AS1	0.246687	0.103836	2.375733	0.01751415	0.223213163
HLA.G	-1.345857	0.56667	-2.375027	0.01754766	0.223378643
AHCYL1	0.253389	0.106737	2.37396	0.01759847	0.223763675
PPP1R16A	-0.411149	0.173227	-2.373465	0.01762207	0.223802374
PELI3	-1.04999	0.44261	-2.372269	0.01767921	0.223912874
COX11	-0.212351	0.089519	-2.372139	0.01768546	0.223912874
HINT2	-0.210999	0.088954	-2.371992	0.01769249	0.223912874
FBXL3	0.307428	0.129645	2.371311	0.0177251	0.224065082
ZNF532	0.60182	0.253838	2.370876	0.01774596	0.224068503
RNF19A	0.36518	0.154109	2.369622	0.0178063	0.224433154
LPCAT2	-1.852675	0.782028	-2.369064	0.01783317	NA
HEXA	0.266488	0.112488	2.369039	0.01783437	0.224433154
NUP210	0.259321	0.109465	2.368991	0.0178367	0.224433154
RASA4	0.443264	0.18722	2.367609	0.01790343	0.225012602
C19orf48	0.33371	0.141009	2.366579	0.01795335	0.225379803
SAP25	0.70138	0.296496	2.365562	0.01800272	0.225531429
RING1	-0.251801	0.106448	-2.365477	0.01800687	0.225531429
SULT1A3	0.598288	0.253082	2.364014	0.01807815	0.225648098
FAM129C	-0.387281	0.163835	-2.363844	0.01808645	0.225648098
PACSIN2	0.311162	0.13164	2.363734	0.01809177	0.225648098
SEMA4A	-1.066171	0.451314	-2.36237	0.0181585	0.225648098
IL7R	-1.205665	0.510428	-2.362068	0.01817332	0.225648098
ENSG00000271646	-0.375553	0.158995	-2.362042	0.01817459	0.225648098
ADGRE2	1.090221	0.461563	2.36202	0.01817566	0.225648098
HAUS4	0.507005	0.214661	2.36189	0.01818205	0.225648098
STMN1	-0.457091	0.193624	-2.360717	0.01823962	0.226104824
FUS	0.111086	0.047089	2.359061	0.01832125	0.226858294
RPS15A	0.128049	0.054299	2.358214	0.01836309	0.22688864
DAXX	0.234096	0.09927	2.358168	0.01836539	0.22688864
SKP2	-0.426967	0.181123	-2.357337	0.01840653	0.227139097
ZNF574	0.494825	0.210048	2.355766	0.01848454	0.227791444
CHD2	0.236758	0.10053	2.355095	0.01851796	0.227791444
MAVS	0.233871	0.099308	2.355011	0.01852218	0.227791444
CTSS	0.180807	0.076865	2.352282	0.01865861	0.229149592
NUAK2	-0.341641	0.145258	-2.351961	0.01867472	0.229149592
PSME2	-0.163232	0.069432	-2.350954	0.01872537	0.22928596

TUBB4A	1.734952	0.738156	2.350387	0.0187539	NA
HACD2	-0.316085	0.134486	-2.350319	0.01875734	0.22928596
GRK2	0.253267	0.107773	2.349996	0.01877359	0.22928596
TMEM87B	0.504156	0.214545	2.349882	0.01877936	0.22928596
BLOC1S1	-0.124437	0.05296	-2.349648	0.01879117	0.22928596
SNRPD1	-0.170706	0.072668	-2.349113	0.01881821	0.229315436
TRIM5	0.590703	0.251495	2.348766	0.01883572	0.229315436
USP40	0.623864	0.265705	2.347957	0.01887671	0.229557675
DNPEP	-0.186351	0.079404	-2.346873	0.01893173	0.229872145
ATP6V0E1	0.177714	0.075732	2.346615	0.01894481	0.229872145
HAUS6	0.369096	0.157326	2.346066	0.01897272	0.229943531
C10orf143	1.018612	0.434252	2.345669	0.01899295	0.229943531
ZNF785	0.393869	0.16802	2.344182	0.01906885	0.230575085
ENSG00000272086	-0.839239	0.358145	-2.343295	0.01911428	0.230575085
CCT8	0.244129	0.104187	2.343184	0.01911993	0.230575085
SUDS3	0.225493	0.096242	2.342991	0.01912985	0.230575085
TBX21	1.511667	0.645304	2.342566	0.01915165	NA
SAT2	0.270239	0.115361	2.342554	0.01915229	0.230590125
ENSG00000185527	-0.919756	0.392797	-2.341557	0.0192035	0.230951257
ZNF540	0.909221	0.388522	2.340205	0.01927315	0.231293146
RPS27A	0.109318	0.046713	2.34018	0.01927443	0.231293146
DEF8	-0.347402	0.148515	-2.339168	0.01932672	0.231665245
PRDM1	0.886535	0.379193	2.337952	0.01938976	0.232113258
TNS3	0.539321	0.230713	2.337624	0.01940675	0.232113258
GATD3B	-1.148076	0.49139	-2.336385	0.0194712	0.232628421
MRPL38	-0.482878	0.206767	-2.335379	0.01952366	0.23299941
EIF4B	0.148084	0.063448	2.333919	0.01959996	0.233653846
PGBD2	0.366129	0.156938	2.332946	0.019651	0.233792846
WDR55	-0.265462	0.113792	-2.332877	0.01965458	0.233792846
DDX17	0.155255	0.066579	2.331889	0.01970654	0.234154988
HIST1H3E	0.866868	0.371949	2.330608	0.01977404	0.234185309
GGCT	-0.199812	0.085737	-2.330527	0.01977831	0.234185309
STAT3	0.263556	0.113122	2.329827	0.01981531	0.234185309
PTPDC1	0.737376	0.316498	2.329797	0.0198169	0.234185309
TNFAIP3	-0.360999	0.154988	-2.329206	0.01984817	0.234185309
ENSG00000271204	0.491627	0.211072	2.329194	0.01984878	0.234185309
SLC25A43	0.417865	0.179419	2.328988	0.01985971	0.234185309
ZNF341.AS1	-1.726044	0.741849	-2.326679	0.01998237	NA
SRSF6	-0.284116	0.122174	-2.325496	0.02004545	0.236062291
ZNF81	-0.662846	0.285073	-2.325181	0.02006226	0.236062291
ACTR3B	-0.524815	0.225776	-2.324487	0.0200994	0.236243958
RAB6A	0.227252	0.097794	2.323788	0.02013688	0.236397749
CDC42	-0.154902	0.06668	-2.323083	0.02017467	0.236397749
RPP21	-0.28946	0.124616	-2.32282	0.02018882	0.236397749
SYTL1	0.278021	0.119718	2.322291	0.02021728	0.236397749
CYP4V2	0.744595	0.320717	2.32166	0.02025126	0.236397749
MRPL57	-0.154827	0.066702	-2.321167	0.02027784	0.236397749

TBC1D10C	0.216359	0.093217	2.32102	0.02028578	0.236397749
PDE6B	-0.429582	0.185108	-2.320702	0.02030295	0.236397749
NCK2	0.367291	0.158301	2.320214	0.02032932	0.236397749
FAM13A	-0.824861	0.355512	-2.320207	0.02032969	0.236397749
TAF12	0.193221	0.083303	2.319503	0.0203678	0.236565601
ENSG00000228506	-1.41462	0.609927	-2.319328	0.02037724	NA
UXT	0.094432	0.040719	2.319137	0.02038759	0.236565601
CCDC126	0.497156	0.214489	2.317862	0.02045681	0.23679632
NOL9	-0.345235	0.14895	-2.317789	0.02046077	0.23679632
NIPAL3	0.33358	0.143935	2.317569	0.02047274	0.23679632
ENSG00000259976	0.653086	0.282147	2.3147	0.02062935	0.238107566
DSTN	-0.165202	0.071373	-2.314634	0.02063299	0.238107566
RESF1	0.214164	0.092567	2.313614	0.0206889	0.238107566
SRP9	-0.165677	0.071611	-2.313557	0.02069205	0.238107566
TUBGCP5	-0.592635	0.256165	-2.313494	0.02069549	0.238107566
C3orf14	1.505504	0.651199	2.311894	0.02078355	0.238592996
FAR1	-0.394609	0.170705	-2.311645	0.02079724	0.238592996
ZNF397	0.267439	0.115697	2.311533	0.02080345	0.238592996
C20orf27	-0.225854	0.097747	-2.310611	0.02085438	0.238925292
DCLRE1C	0.205137	0.088806	2.309928	0.02089215	0.239068425
ROCK1	0.159337	0.068997	2.30932	0.02092583	0.239068425
ELMO1	0.293779	0.127221	2.309195	0.02093276	0.239068425
TFEC	0.87407	0.378623	2.308549	0.02096862	0.239083173
MGAT5B	-1.185703	0.513652	-2.30838	0.02097799	0.239083173
C2orf42	0.478185	0.207202	2.307817	0.02100931	0.239189625
SUZ12	0.227188	0.098552	2.305257	0.0211522	0.240564835
CPNE3	-0.227969	0.098938	-2.30416	0.02121366	0.240912648
PRPSAP2	0.297002	0.128911	2.303921	0.02122705	0.240912648
TOLLIP.AS1	-1.484193	0.644298	-2.30358	0.02124623	NA
ENSG00000262580	1.215657	0.527778	2.303349	0.02125921	NA
ENSG00000238142	0.453114	0.196777	2.302674	0.02129722	0.24145718
MAP2K3	-0.226434	0.098369	-2.301876	0.02134215	0.241714861
TLE6	-2.663642	1.1576	-2.301004	0.02139139	NA
NDRG3	0.375065	0.163132	2.299156	0.02149609	0.24320526
PIEZO1	0.445128	0.193654	2.298578	0.02152892	0.243323712
KLHDC3	-0.208757	0.090845	-2.297949	0.02156467	0.243414673
NUCB2	-0.64808	0.282062	-2.29765	0.02158169	0.243414673
SIDT1.AS1	-0.719195	0.313252	-2.295898	0.02168173	0.24417482
BLNK	0.255481	0.111288	2.295684	0.02169396	0.24417482
ADARB1	-0.438637	0.191115	-2.295149	0.0217246	0.24426708
RAF1	0.247854	0.108048	2.293912	0.02179553	0.244811702
GOLPH3L	0.457726	0.199667	2.29244	0.02188029	0.245510366
ERGIC1	-0.223546	0.097536	-2.291938	0.02190924	0.245535904
LPCAT1	0.419561	0.183085	2.291618	0.02192768	0.245535904
GABPB1	-0.231923	0.101246	-2.290696	0.02198102	0.245864101
PTPRN2	0.448723	0.195921	2.29033	0.02200217	0.245864101
PSME4	-0.277673	0.121331	-2.288556	0.02210518	0.246761829

SENP1	-0.469863	0.205346	-2.288151	0.02212871	0.246771378
TRA2B	-0.182702	0.079879	-2.28725	0.02218126	0.246847181
SGCE	0.893512	0.390653	2.287226	0.02218264	0.246847181
IL9R	1.197403	0.523653	2.286633	0.02221728	0.246847181
ARHGAP18	0.519619	0.227257	2.286479	0.02222623	0.246847181
THOC3	-0.416684	0.182376	-2.284752	0.02232737	0.247717688
PIM1	-0.502911	0.220187	-2.284022	0.02237027	0.247913827
ABHD11	-0.39269	0.172002	-2.283057	0.02242702	0.247913827
SDAD1	-0.203163	0.089004	-2.282626	0.02245243	0.247913827
C2orf76	-0.339066	0.148558	-2.28238	0.02246692	0.247913827
MSMO1	0.224658	0.09844	2.282183	0.02247854	0.247913827
DTX3L	0.27911	0.122302	2.282129	0.02248171	0.247913827
ZSWIM7	-0.312498	0.136973	-2.281467	0.02252083	0.248093836
BANK1	0.382661	0.167783	2.280686	0.02256706	0.24835178
RASA3	0.634116	0.278242	2.279013	0.02266631	0.24896572
TTC17	-0.249566	0.109525	-2.278632	0.02268896	0.24896572
LEPROTL1	-0.329022	0.144404	-2.278489	0.02269745	0.24896572
DUS3L	-0.392644	0.172373	-2.277882	0.02273362	0.24896572
RPP40	-0.449814	0.197498	-2.277558	0.02275295	0.24896572
MAN2B1	-0.286357	0.125736	-2.277438	0.0227601	0.24896572
ERICH1	0.170391	0.074871	2.275784	0.02285893	0.249538639
PSMG4	-0.239379	0.105198	-2.275514	0.02287514	0.249538639
LINC01684	-0.695435	0.30563	-2.275412	0.02288125	0.249538639
LTBP4	0.468703	0.20606	2.274596	0.0229302	0.249643967
ALG5	-0.224343	0.098635	-2.274486	0.02293679	0.249643967
USP54	-0.92714	0.407818	-2.273415	0.02300116	0.25009448
PPFIBP2	0.366772	0.161406	2.272361	0.02306472	0.250535358
MYO9B	0.277595	0.122301	2.269764	0.0232219	0.251922842
FAM241A	-0.482224	0.212486	-2.269437	0.02324178	0.251922842
LINC01480	-0.512778	0.225982	-2.269106	0.02326189	0.251922842
CD83	-0.349592	0.154093	-2.268712	0.02328586	0.251931654
RNF167	0.178144	0.0786	2.266455	0.02342356	0.253169815
FASTKD2	-0.342749	0.151279	-2.265681	0.02347094	0.253181499
OSBPL1A	-1.090993	0.481533	-2.265666	0.02347183	0.253181499
DUSP18	0.430548	0.190063	2.265298	0.02349442	0.253181499
HNRNPK	-0.120148	0.053052	-2.264729	0.0235293	0.253214203
ERI1	0.436387	0.192709	2.264484	0.02354435	0.253214203
TEX30	0.29607	0.130784	2.263801	0.02358633	0.253214203
C1orf216	0.36741	0.162335	2.263292	0.02361769	0.253214203
SMAD2	0.271318	0.11989	2.263051	0.02363255	0.253214203
AKT3	0.468315	0.206946	2.262978	0.02363705	0.253214203
SPRYD4	-0.378497	0.16746	-2.260225	0.02380728	0.254541243
PXDC1	-0.664004	0.293823	-2.259882	0.02382859	0.254541243
CNKSR2	0.849597	0.376065	2.259173	0.02387261	0.254541243
ZNF318	0.352542	0.156049	2.259169	0.02387289	0.254541243
IER2	-0.153674	0.068032	-2.258839	0.02389342	0.254541243
MAP2K1	0.270838	0.119908	2.258713	0.02390125	0.254541243

MZB1	-0.401087	0.177841	-2.255317	0.02411344	0.256550013
ELP6	-0.304793	0.135172	-2.254852	0.02414265	0.256609963
SLC35D2	1.273117	0.56478	2.254184	0.02418461	NA
ICAM3	0.170265	0.075605	2.252022	0.02432091	0.25820965
ZNF860	0.636377	0.282684	2.251197	0.02437307	0.25820965
RNF123	-0.498106	0.22128	-2.251018	0.02438443	0.25820965
ZFC3H1	0.288466	0.128153	2.25096	0.02438805	0.25820965
MRFAP1L1	0.273865	0.121715	2.25006	0.02444511	0.258382079
ZBTB4	0.414285	0.18413	2.249955	0.02445181	0.258382079
SRSF2	-0.173019	0.076941	-2.248727	0.02452984	0.258730444
RBM26.AS1	-0.642821	0.285891	-2.248482	0.02454546	0.258730444
MAPK9	0.450942	0.200569	2.248315	0.0245561	0.258730444
EYA3	0.575063	0.255901	2.247212	0.02462649	0.259037028
LPP	0.334735	0.148975	2.24692	0.02464515	0.259037028
ZNF16	-0.365768	0.162799	-2.246741	0.02465659	0.259037028
TMEM101	0.27397	0.121989	2.245849	0.0247137	0.259386623
DGKG	1.814856	0.808119	2.245776	0.02471833	NA
EID1	-0.149974	0.066795	-2.245297	0.02474908	0.259507737
TENT4B	0.420864	0.187516	2.244424	0.02480515	0.259726917
SIGMAR1	-0.314892	0.140312	-2.244228	0.02481771	0.259726917
ENSG00000267364	1.207889	0.538403	2.243467	0.02486672	0.25998981
PPP1R13L	0.762148	0.339908	2.242218	0.02494729	0.260505171
POMGNT1	-0.293187	0.130773	-2.241961	0.02496388	0.260505171
KLHDC7B	-0.644262	0.287528	-2.240692	0.02504601	0.261111858
SEPHS1	-0.407634	0.181971	-2.240107	0.02508397	0.261257359
ENSG00000267939	-1.613054	0.720179	-2.239796	0.02510418	NA
ARL5A	0.177759	0.079373	2.239555	0.02511982	0.261380659
SH2D2A	-0.625106	0.2794	-2.237313	0.02526589	0.262649451
ZNF362	-0.536652	0.23993	-2.236707	0.02530551	0.262775796
CMTM8	-1.163331	0.520183	-2.236389	0.02532633	0.262775796
PHOSPHO2	0.520158	0.232737	2.234962	0.02541985	0.263494875
PRR4	0.426181	0.190768	2.234025	0.02548144	0.263882029
ENSG00000268568	-0.818675	0.366762	-2.232171	0.02560367	0.264895723
FAM160B2	-0.476232	0.213483	-2.230774	0.02569611	0.265599676
TCF3	0.217504	0.097572	2.229157	0.02580349	0.266456565
ZCCHC10	0.212387	0.09531	2.228393	0.02585431	0.266567859
PARS2	0.7457	0.334667	2.228187	0.02586807	0.266567859
NOTCH2	0.277182	0.124415	2.227892	0.02588775	0.266567859
NME2	-0.327136	0.146889	-2.227101	0.02594055	0.266625977
NT5DC4	0.780724	0.350561	2.227073	0.02594238	0.266625977
ENSG00000262165	-1.705363	0.765746	-2.227061	0.02594321	NA
AIMP2	-0.319103	0.143414	-2.225045	0.02607824	0.267640928
CBX3	-0.145456	0.065377	-2.224865	0.02609032	0.267640928
BCL2L11	-0.338002	0.15199	-2.223839	0.02615927	0.268095575
SIGLEC14	0.835957	0.376022	2.223159	0.02620507	0.268312297
TGFBR2	0.198349	0.089262	2.222101	0.02627646	0.268554337
PPIL2	-0.338824	0.152481	-2.222078	0.02627806	0.268554337

P4HB	-0.153552	0.069139	-2.220914	0.02635681	0.269071758
KDM1B	0.724752	0.326377	2.220599	0.02637813	0.269071758
IGFBP4	-0.841472	0.379211	-2.219009	0.0264861	0.269920176
FBXO8	0.427447	0.192764	2.217468	0.02659111	0.2705651
CYP3A5	0.536829	0.242107	2.217319	0.02660133	0.2705651
NFE2L2	0.176376	0.079557	2.216987	0.02662396	0.2705651
SH2D3C	0.416807	0.188051	2.216454	0.02666043	0.270682906
ZNF285	0.802175	0.362041	2.215701	0.02671196	0.270953345
AGFG2	0.79636	0.359832	2.213142	0.02688786	0.272301643
C6orf203	0.331907	0.149978	2.21304	0.02689492	0.272301643
TMSB4Y	-1.220146	0.551932	-2.210679	0.02705807	0.273698957
FAM214B	0.728775	0.330134	2.207509	0.02727849	0.274984419
HAUS1	-0.205091	0.09291	-2.207416	0.02728504	0.274984419
UCK2	-0.373644	0.169293	-2.207083	0.02730827	0.274984419
TRMT1L	0.390759	0.177052	2.207029	0.02731205	0.274984419
ENSG00000245869	0.501748	0.227355	2.206896	0.0273213	0.274984419
MRPS15	-0.180286	0.081706	-2.206529	0.027347	0.274984419
H2AFY	-0.171287	0.077664	-2.205495	0.02741939	0.274984419
IGKV1.12	-0.721682	0.327235	-2.205395	0.02742641	0.274984419
CHID1	-0.268407	0.121706	-2.205367	0.02742832	0.274984419
NRBF2	0.239368	0.108545	2.205232	0.02743781	0.274984419
ARMC7	0.408997	0.185543	2.204323	0.02750167	0.275370865
SLC27A3	0.820377	0.372311	2.203474	0.02756133	0.275714579
HIPK1	0.37359	0.169694	2.201555	0.02769677	0.276815106
P4HA1	-0.36953	0.167943	-2.200333	0.02778331	0.27716654
ARID3A	0.452174	0.205535	2.199987	0.02780782	0.27716654
ZNF430	0.232573	0.105748	2.199304	0.02785628	0.27716654
ENSG00000251364	1.016704	0.46229	2.199279	0.02785809	0.27716654
RABEPK	-0.273592	0.124402	-2.199263	0.02785926	0.27716654
PITPNM2	-0.788615	0.358693	-2.19858	0.0279078	0.277242904
HMG20B	-0.161939	0.073672	-2.198118	0.02794067	0.277242904
QTRT1	-0.20914	0.095147	-2.198081	0.02794335	0.277242904
NCAPH2	-0.321046	0.146161	-2.196528	0.02805417	0.277756874
FHOD1	0.383185	0.174473	2.196245	0.02807443	0.277756874
PRKACA	0.391523	0.178286	2.196039	0.02808916	0.277756874
DIS3L	0.449165	0.204544	2.195926	0.02809724	0.277756874
SSU72	-0.1819	0.082874	-2.1949	0.02817077	0.278116949
ZNF417	0.687691	0.313375	2.194467	0.02820184	0.278116949
AHCTF1	0.359915	0.164019	2.194349	0.02821032	0.278116949
POR	-0.309238	0.140959	-2.193812	0.02824893	0.278245531
MRFAP1	0.171336	0.078135	2.192831	0.02831954	0.278688866
THRB	1.643647	0.749602	2.192694	0.02832947	NA
ZNF225	0.649641	0.296801	2.188813	0.02861041	0.281296881
SNRPA1	-0.213985	0.097785	-2.188325	0.02864593	0.281391976
C15orf62	-0.580335	0.265295	-2.187509	0.02870537	0.281721573
RMND5B	-0.291806	0.133426	-2.187021	0.028741	0.281817119
SIAE	1.404104	0.642149	2.18657	0.02877394	NA

DCBLD2	-0.969631	0.443716	-2.185251	0.02887045	0.282540603
JRKL	0.526143	0.240823	2.184769	0.02890579	0.282540603
ENSG00000253140	1.373869	0.628843	2.184759	0.02890654	0.282540603
USP44	0.942871	0.431686	2.184161	0.02895039	0.282540603
UROS	-0.293434	0.134354	-2.184037	0.02895953	0.282540603
URB1.AS1	-0.576235	0.263857	-2.183887	0.02897054	0.282540603
DUSP2	-0.454766	0.208342	-2.182785	0.02905165	0.28291859
GABARAPL2	0.156619	0.071756	2.182654	0.02906128	0.28291859
HIST2H2BF	1.443723	0.661525	2.182418	0.02907873	NA
SERAC1	-0.680878	0.312133	-2.181369	0.02915611	0.283588122
SIRT7	0.221833	0.101769	2.179781	0.02927367	0.284307116
ELMSAN1	0.30635	0.140549	2.179665	0.02928228	0.284307116
LINC01678	-0.398703	0.182998	-2.178723	0.02935228	0.28458321
FAM120A	0.208974	0.095922	2.178578	0.02936301	0.28458321
AFF2	-1.539875	0.707048	-2.177894	0.02941396	NA
PDP2	0.664169	0.305141	2.176599	0.0295105	0.285754654
NADK2	-0.48566	0.223163	-2.176253	0.02953638	0.285754654
MRPL19	-0.202806	0.093218	-2.175601	0.02958508	0.2859716
TMF1	0.235138	0.108106	2.175074	0.02962458	0.285983262
ENSG00000259153	0.825537	0.379682	2.174286	0.02968365	0.285983262
ECHDC2	0.678637	0.31214	2.174146	0.02969416	0.285983262
SGPL1	-0.286972	0.132004	-2.173968	0.02970754	0.285983262
MCM8	0.555549	0.255562	2.173833	0.02971767	0.285983262
BRD3OS	0.320661	0.147662	2.171586	0.02988688	0.287319492
SUN1	-0.224307	0.103306	-2.171289	0.02990932	0.287319492
CMTM3	-0.788146	0.363091	-2.170656	0.02995717	0.287525452
ZNF107	0.415916	0.191796	2.168538	0.03011781	0.288812563
FANCL	0.535299	0.246892	2.168154	0.03014697	0.288837727
CYREN	-0.219751	0.101437	-2.166382	0.030282	0.289312401
SEC24D	0.513469	0.237064	2.165949	0.03031507	0.289312401
USP30	-0.751546	0.347002	-2.165825	0.03032456	0.289312401
PTMA	-0.132137	0.061029	-2.165153	0.03037594	0.289312401
CALR	-0.176128	0.081347	-2.165149	0.03037625	0.289312401
RBM19	-0.260749	0.120438	-2.165006	0.03038723	0.289312401
COG3	-0.374917	0.173192	-2.164745	0.03040722	0.289312401
HADHA	0.166765	0.077049	2.164393	0.03043417	0.289312401
KCP	1.043722	0.482228	2.164373	0.03043575	0.289312401
RNF32	1.082795	0.500489	2.163476	0.03050456	NA
NAP1L1	0.158312	0.073175	2.163461	0.03050575	0.289724757
TRIM32	0.780535	0.361279	2.160478	0.0307357	0.291362447
ZBTB37	0.439344	0.2034	2.160003	0.03077244	0.291362447
SLC4A7	0.382898	0.177278	2.159872	0.03078256	0.291362447
ARFGEF2	0.334734	0.154981	2.159837	0.03078526	0.291362447
ASAP1	0.43818	0.202954	2.159015	0.030849	0.291711961
ENSG00000260274	-0.727068	0.336951	-2.157784	0.03094466	0.292166368
SPATC1L	-0.403092	0.186815	-2.157705	0.03095074	0.292166368
PMF1	-0.203886	0.094539	-2.15663	0.0310345	0.292368533

MEST	-0.404967	0.187784	-2.156562	0.03103981	0.292368533
OSBPL3	0.616572	0.285927	2.156396	0.03105274	0.292368533
NUDT16L1	-0.231286	0.107278	-2.155941	0.03108825	0.292449903
SNHG19	-0.272469	0.126435	-2.155007	0.03116131	0.292884061
NAA16	0.438021	0.203326	2.154279	0.03121831	0.293166614
TMEM242	-0.249928	0.116037	-2.153866	0.0312507	0.293217789
KIAA1958	-0.691241	0.321252	-2.151712	0.03142003	0.294552583
MSANTD4	0.398821	0.185384	2.151321	0.03145089	0.294588209
NDUFS6	-0.158064	0.073503	-2.150437	0.03152063	0.294598454
S100A11	0.375498	0.174638	2.150153	0.03154308	0.294598454
RIPOR2	0.191997	0.089298	2.150076	0.03154919	0.294598454
BAZ2A	0.220993	0.102791	2.149922	0.03156139	0.294598454
NDUFAF4	0.228758	0.106419	2.149594	0.03158732	0.294598454
KLF13	0.25062	0.116648	2.148521	0.03167242	0.29499381
SLC38A2	0.20449	0.095191	2.148214	0.03169676	0.29499381
IFRD2	-0.19669	0.091567	-2.148034	0.03171102	0.29499381
LINC00339	0.788101	0.367112	2.146759	0.03181245	0.295396108
TMEM121	-0.817756	0.380979	-2.14646	0.03183629	0.295396108
KDM4D	-1.509386	0.703333	-2.146047	0.03186925	NA
ZNF384	-0.362672	0.169017	-2.145773	0.03189108	0.295396108
DPM1	0.21498	0.100192	2.145675	0.0318989	0.295396108
EIF2AK3	-0.43099	0.200875	-2.145563	0.03190784	0.295396108
LRRC56	0.791657	0.368994	2.145447	0.03191711	0.295396108
FAM43A	0.693924	0.323515	2.144952	0.03195665	0.295510799
SBNO1	0.177258	0.082665	2.144299	0.03200893	0.295742908
MOSPD3	-0.265407	0.123805	-2.143751	0.03205287	0.295897696
ARCN1	0.287918	0.134379	2.142576	0.03214714	0.296259619
DAGLB	0.515282	0.240555	2.142055	0.03218906	0.296259619
DNTTIP1	0.267349	0.12481	2.142045	0.03218989	0.296259619
UBR5	0.268147	0.125191	2.141907	0.03220095	0.296259619
MTRNR2L12	-0.300746	0.140517	-2.140281	0.03233209	0.296992567
FLYWCH1	0.416308	0.194514	2.140242	0.03233519	0.296992567
CD320	-0.228405	0.106774	-2.139158	0.03242285	0.29754662
TMSB10	-0.125138	0.058527	-2.138135	0.03250581	0.297880265
BAG6	-0.211753	0.099041	-2.138035	0.03251394	0.297880265
ATRAID	-0.160855	0.075349	-2.134811	0.03277646	0.299763754
CYTIP	0.249963	0.117091	2.134778	0.03277912	0.299763754
ENSG00000265206	0.25093	0.117559	2.134497	0.03280215	0.299763754
NAA10	-0.171707	0.080469	-2.133814	0.03285802	0.300022379
CHKB	-0.355581	0.166745	-2.132485	0.03296702	0.300574865
SERTAD1	0.372615	0.174739	2.132403	0.03297376	0.300574865
MYLK.AS1	-0.604498	0.283568	-2.131759	0.03302665	0.300794734
TRIM16	-0.43312	0.203246	-2.131018	0.0330877	0.300794734
TM9SF4	-0.355591	0.166871	-2.130937	0.03309433	0.300794734
TMX2	0.200479	0.094088	2.130766	0.03310842	0.300794734
TENT5A	0.442287	0.207883	2.127574	0.03337239	0.302768837
PDPR	-0.542707	0.255096	-2.127466	0.03338135	0.302768837

ISCA1	-0.189378	0.08904	-2.126881	0.03342994	0.302957071
HIST1H1B	1.429306	0.672256	2.126133	0.03349217	NA
VWCE	-0.669377	0.314964	-2.125253	0.0335655	0.303871832
NR6A1	-0.514664	0.242195	-2.124999	0.03358672	0.303871832
PDIA3	-0.169025	0.079557	-2.124585	0.03362126	0.303884195
RPS5	0.127103	0.059833	2.124296	0.03364538	0.303884195
MT.CO1	0.142723	0.067196	2.12398	0.03367184	0.303884195
ARF4.AS1	-0.684189	0.322293	-2.12288	0.03376388	0.304197039
ZNF468	-0.413884	0.194967	-2.122839	0.03376731	0.304197039
MDFIC	0.426475	0.200924	2.122565	0.03379036	0.304197039
JMJD8	-0.311155	0.146645	-2.121827	0.03385227	0.304502535
PHF13	0.680196	0.320723	2.120822	0.03393679	0.304881309
FAM168B	0.291286	0.137378	2.120327	0.03397851	0.304881309
GPSM3	0.147442	0.069541	2.120209	0.03398843	0.304881309
RNF26	-0.241934	0.11412	-2.119996	0.03400642	0.304881309
ZNF2	0.71944	0.339653	2.11816	0.03416156	0.30602009
SAMD4B	0.24813	0.117216	2.116864	0.03427137	0.306751314
FGFR1OP2	0.221093	0.104481	2.116119	0.03433466	0.306981429
SPRYD7	0.28116	0.13288	2.115898	0.03435349	0.306981429
POMP	-0.152288	0.071998	-2.115153	0.03441688	0.307040192
FAF2	-0.281803	0.133269	-2.114544	0.03446885	0.307040192
HDAC8	-0.304031	0.143783	-2.114508	0.03447188	0.307040192
ENSG00000260806	-0.715066	0.338173	-2.114496	0.03447291	0.307040192
ENSG00000188206	0.375135	0.177441	2.114137	0.03450358	0.307062087
SHLD1	-0.284198	0.134498	-2.113017	0.03459927	0.307662162
LYRM4	-0.142288	0.067352	-2.112603	0.03463475	0.307726226
SDF2L1	-0.259178	0.122764	-2.111185	0.03475641	0.308550389
EEF1E1	-0.196266	0.092983	-2.110768	0.03479227	0.308550389
ABHD3	0.326659	0.154776	2.110532	0.03481256	0.308550389
STK10	0.263097	0.12483	2.107649	0.03506139	0.310503005
WDR45	-0.231757	0.11003	-2.106298	0.03517845	0.311286387
LINC00937	-1.35238	0.642501	-2.104868	0.03530281	NA
ZNF276	0.315368	0.149838	2.104726	0.03531516	0.311929232
VPS37A	0.289076	0.137349	2.104683	0.03531888	0.311929232
ZFAND4	0.827942	0.39342	2.104474	0.03533708	0.311929232
HLA.DPA1	0.212132	0.100833	2.103791	0.03539671	0.312202409
MYO1D	0.928425	0.4414	2.103363	0.03543406	NA
ENSG00000006025	0.552209	0.262562	2.103155	0.03545226	0.312439155
FBXO25	-0.296229	0.140906	-2.102309	0.0355262	0.312837546
SQSTM1	0.191245	0.090991	2.101786	0.03557206	0.312988153
KPNB1	0.142089	0.067619	2.10131	0.03561377	0.313101994
TCOF1	-0.199932	0.095193	-2.100282	0.03570402	0.313642101
FERMT3	0.241829	0.115163	2.099882	0.03573924	0.313698273
POLR2E	-0.16088	0.076626	-2.09954	0.03576932	0.313709326
CXCR3	1.530395	0.72909	2.099049	0.03581259	0.313835969
SGPP2	1.202238	0.572893	2.098539	0.03585752	0.313970976
MAPKAPK2	-0.265552	0.126561	-2.09822	0.03588569	0.313970976

NAP1L6	-1.315932	0.627204	-2.098093	0.03589697	NA
ARHGEF1	0.222297	0.105968	2.09777	0.03592547	0.314066487
BLOC1S3	0.397707	0.189758	2.095857	0.03609484	0.315104456
MOB4	0.21293	0.101605	2.09566	0.03611233	0.315104456
WASH9P	-0.320799	0.153093	-2.09545	0.03613105	0.315104456
ZNF654	0.448546	0.214106	2.094967	0.03617391	0.315225683
RNF6	0.222172	0.106086	2.094258	0.03623697	0.315284403
UBXN8	-0.401322	0.191631	-2.09424	0.03623859	0.315284403
CEP295	0.383743	0.183275	2.093814	0.03627652	0.315362305
TWISTNB	-0.259132	0.123801	-2.093131	0.0363375	0.315597774
CLTC	0.218343	0.104327	2.092861	0.0363616	0.315597774
ZFYVE21	0.325943	0.155809	2.091933	0.0364445	0.316065303
ENSG00000255224	-1.034855	0.495075	-2.090299	0.03659099	NA
ZBTB21	0.4563	0.218372	2.089552	0.03665809	0.317664488
COL4A3	2.086439	0.998766	2.089017	0.03670622	NA
NDUFV2	-0.128396	0.061467	-2.088874	0.03671908	0.317737306
RAB33A	1.081183	0.517607	2.08881	0.03672487	0.317737306
SLC43A3	-0.268852	0.128744	-2.088272	0.03677333	0.317903839
MUS81	0.258835	0.123979	2.087728	0.03682239	0.318075328
PITPNM1	0.393947	0.18876	2.087024	0.03688595	0.318371695
SLC11A2	0.513263	0.246138	2.085268	0.03704498	0.319490928
CBWD6	-0.729618	0.350072	-2.084198	0.0371422	0.319840705
OFD1	0.260841	0.125153	2.084174	0.03714431	0.319840705
UGT8	0.526486	0.252764	2.082917	0.03725877	0.320572708
TOMM20	0.128609	0.061754	2.082591	0.03728855	0.32057546
TMCC1.AS1	1.091859	0.524354	2.082294	0.0373156	NA
ZNF354B	0.477306	0.229269	2.081864	0.03735491	0.32089251
TIMM50	-0.232472	0.111697	-2.081279	0.03740834	0.321098087
NAGPA	0.299106	0.143789	2.080175	0.0375095	0.321528531
SFT2D1	-0.231577	0.11133	-2.080087	0.03751757	0.321528531
HSD17B11	0.236607	0.113805	2.079062	0.03761163	0.321660172
WAC	0.201373	0.096861	2.078979	0.03761926	0.321660172
ZNF234	0.375102	0.180428	2.078954	0.03762159	0.321660172
DEF6	0.291164	0.140102	2.078234	0.03768778	0.321879798
STK17A	0.279152	0.134335	2.078032	0.03770643	0.321879798
MBOAT2	-1.328035	0.639194	-2.077672	0.03773961	NA
CCL4	-0.836865	0.402818	-2.077525	0.0377531	0.322025646
KPNA2	-0.2304	0.110918	-2.0772	0.03778307	0.32202892
SMIM12	-0.150522	0.072544	-2.074899	0.0379959	0.323588278
MRPL53	-0.210799	0.10161	-2.07458	0.03802549	0.323588278
BLCAP	-0.258237	0.124579	-2.072879	0.03818357	0.324337964
CHD7	0.240452	0.116004	2.072792	0.03819165	0.324337964
EP300	0.23007	0.11102	2.072325	0.03823518	0.324337964
USP53	0.574689	0.277324	2.072264	0.03824081	0.324337964
RAB7A	-0.150477	0.072623	-2.072031	0.03826258	0.324337964
DSCAML1	-1.466393	0.707846	-2.071627	0.03830028	NA
CNOT4	0.213743	0.10323	2.070543	0.03840157	0.324607027

CCDC106	-0.306168	0.147877	-2.070421	0.03841298	0.324607027
ZNF593	-0.220241	0.106392	-2.070087	0.03844422	0.324607027
MTF1	0.342291	0.165367	2.069891	0.03846256	0.324607027
CNN2	0.213138	0.10299	2.069497	0.03849952	0.324607027
TRMT112	0.225275	0.108856	2.069477	0.03850133	0.324607027
RBM17	-0.153119	0.07399	-2.069458	0.03850309	0.324607027
DDX18	-0.117823	0.056948	-2.068968	0.03854908	0.324743169
IGHV3.23	-0.816429	0.394682	-2.068572	0.0385863	0.324805331
ENSG00000272030	-1.06707	0.51585	-2.068566	0.03858688	NA
NAV2.AS3	-1.574985	0.761396	-2.068549	0.03858842	NA
SEC23A	0.265365	0.128333	2.067786	0.03866019	0.325175842
SMYD5	-0.389446	0.188394	-2.067194	0.03871586	0.325392584
COPB1	0.210727	0.10199	2.06615	0.03881428	0.325968032
MAD2L1BP	0.328359	0.15904	2.064629	0.0389581	0.326923608
RAPGEF5	-1.458862	0.706863	-2.063854	0.03903157	NA
OSBP	0.386787	0.187426	2.063681	0.03904798	0.32742539
CD79B	-0.274297	0.133002	-2.062356	0.03917386	0.327520276
MANBA	-0.256172	0.124232	-2.062046	0.03920334	0.327520276
RPN1	-0.222503	0.107919	-2.061766	0.03923004	0.327520276
MEA1	-0.165622	0.080337	-2.061591	0.03924665	0.327520276
TMEM183A	-0.153282	0.074356	-2.061467	0.0392585	0.327520276
GNA13	0.276344	0.134056	2.061414	0.03926353	0.327520276
SREBF1	-0.441295	0.214081	-2.061347	0.03926993	0.327520276
WBP1L	-0.316554	0.153597	-2.060938	0.03930895	0.327594654
MPHOSPH8	0.198649	0.096483	2.0589	0.0395038	0.328711855
DHX30	-0.201792	0.098013	-2.058821	0.03951138	0.328711855
ZNF354A	-0.281657	0.136821	-2.058581	0.0395344	0.328711855
BET1	0.239065	0.116148	2.058274	0.03956381	0.328711855
HLA.DQB2	-1.655289	0.804389	-2.057822	0.03960723	NA
SLC15A2	0.867654	0.4217	2.057513	0.03963689	0.329067789
ENSG00000183308	-1.475592	0.71734	-2.057034	0.03968296	NA
AP2S1	-0.20105	0.097746	-2.056861	0.03969956	0.329222621
CD40	-0.142075	0.069095	-2.056229	0.03976045	0.329222621
SNAPC4	-0.340751	0.165727	-2.056098	0.03977307	0.329222621
IAH1	0.132634	0.064523	2.055613	0.03981985	0.329222621
SYNC	-0.331044	0.161063	-2.055368	0.03984351	0.329222621
TP53INP1	0.496324	0.241478	2.055365	0.03984377	0.329222621
RANBP2	0.226541	0.110232	2.055121	0.03986727	0.329222621
GM2A	0.346106	0.168564	2.053262	0.0400472	0.330244592
REL	-0.238591	0.116203	-2.053215	0.04005172	0.330244592
ATP6V1G1	0.164226	0.080041	2.051764	0.04019258	0.33051498
ENSG00000254281	-1.124641	0.548217	-2.051453	0.04022287	NA
PEX6	0.696786	0.339672	2.051348	0.0402331	0.33051498
EEA1	0.283789	0.138378	2.050834	0.04028311	0.33051498
ENSG00000231609	-0.751287	0.366347	-2.05075	0.04029129	0.33051498
WDR18	-0.224129	0.109296	-2.050667	0.04029943	0.33051498
ZNF451	0.27914	0.136159	2.050098	0.0403549	0.33051498

LINC01006	0.45486	0.221875	2.05007	0.04035756	0.33051498
FKBP5	0.446788	0.217964	2.049821	0.04038194	0.33051498
PCMTD2	-0.401156	0.195737	-2.049469	0.0404163	0.33051498
DCAF8	-0.223977	0.109287	-2.049446	0.04041849	0.33051498
MAST3	0.395336	0.192899	2.049446	0.04041855	0.33051498
PPP4C	-0.106648	0.052057	-2.048698	0.04049167	0.330864334
RAB23	1.149433	0.561107	2.04851	0.04051007	NA
CLIP1	0.423952	0.207032	2.04776	0.04058348	0.331365788
LINC00158	0.621479	0.303674	2.046535	0.04070376	0.33154185
NSMF	-0.467396	0.228425	-2.046165	0.04074011	0.33154185
ZNF714	0.452005	0.220945	2.045785	0.04077751	0.33154185
CEP78	-0.301331	0.147332	-2.045249	0.04083036	0.33154185
RALB	0.261558	0.127913	2.044802	0.04087437	0.33154185
DOK1	0.342455	0.167496	2.044561	0.04089815	0.33154185
TAPBP	-0.168321	0.082328	-2.04453	0.04090121	0.33154185
EGR2	-0.485173	0.237361	-2.044027	0.04095086	0.33154185
TNFSF12	0.406039	0.198662	2.043871	0.04096628	0.33154185
ATP5PF	-0.145813	0.071358	-2.043407	0.04101215	0.33154185
FAM49B	0.136804	0.06695	2.043388	0.04101403	0.33154185
ENSG00000266962	0.32115	0.157174	2.043277	0.04102499	0.33154185
PDE7A	0.230438	0.112785	2.043168	0.04103584	0.33154185
SCAND1	-0.168606	0.082574	-2.041861	0.04116529	0.33154185
AP2M1	-0.150334	0.073636	-2.041588	0.04119242	0.33154185
ENSG00000262049	-0.375738	0.184081	-2.041155	0.04123545	0.33154185
TCIRG1	0.337821	0.165528	2.040868	0.04126396	0.33154185
SLC2A4RG	0.889086	0.43573	2.040451	0.04130547	0.33154185
TBC1D14	-0.296489	0.145318	-2.04028	0.04132245	0.33154185
DEGS1	0.287689	0.141008	2.040229	0.04132753	0.33154185
IL2RB	1.05746	0.518333	2.040117	0.04133869	0.33154185
GTF2IRD2	-0.382595	0.18758	-2.039639	0.04138629	0.33154185
IL12RB1	0.427373	0.209548	2.039498	0.04140033	0.33154185
SHF	-0.519376	0.254671	-2.039399	0.04141018	0.33154185
SP140	0.270877	0.132826	2.039341	0.041416	0.33154185
CIAO1	-0.204919	0.100486	-2.039271	0.04142296	0.33154185
ENSG00000179094	-0.506302	0.248281	-2.039226	0.0414275	0.33154185
GPR65	0.222614	0.109208	2.038432	0.04150678	0.331932217
ENSG00000260018	-0.771665	0.378845	-2.036891	0.04166096	0.332865555
LNPEP	0.248281	0.121906	2.036655	0.04168465	0.332865555
EAPP	-0.214149	0.105196	-2.035721	0.04177837	0.333296344
IGHV3.15	-1.256005	0.616991	-2.035695	0.041781	NA
CLEC11A	-0.67367	0.330976	-2.035405	0.04181018	0.333296344
ATOX1	-0.263233	0.129354	-2.034982	0.04185268	0.333296344
PPP2R5C	-0.165233	0.0812	-2.034898	0.04186109	0.333296344
JOSD1	-0.301589	0.148274	-2.033991	0.04195254	0.33378032
TBX6	1.081285	0.53179	2.033295	0.04202276	NA
NMT2	-0.46911	0.230782	-2.032696	0.04208322	0.334412741
CELF1	0.153868	0.0757	2.032595	0.04209348	0.334412741

CAB39	0.312177	0.153662	2.03158	0.0421962	0.334984305
CD58	-0.306539	0.150948	-2.030764	0.04227894	0.335157169
CASZ1	-0.572284	0.28184	-2.030532	0.04230253	0.335157169
CWC15	-0.13922	0.068575	-2.030171	0.04233921	0.335157169
FNIP1	-0.26044	0.128286	-2.030151	0.04234115	0.335157169
CHMP2B	0.227296	0.112022	2.029025	0.04245575	0.335820008
UQCC2	-0.21831	0.107717	-2.026704	0.04269266	0.337304564
DCXR	-0.262029	0.129296	-2.02658	0.04270541	0.337304564
ZC3H11A	0.242236	0.119583	2.025682	0.04279738	0.337728274
TMEM14B	-0.21476	0.106036	-2.02536	0.04283036	0.337728274
NINJ2	-0.492289	0.243088	-2.025148	0.04285215	0.337728274
IKBKG	0.256981	0.12692	2.024749	0.04289317	0.337807005
PKNOX1	-0.301903	0.149142	-2.024263	0.0429431	0.337955678
LYPLAL1	0.297315	0.14694	2.023373	0.04303473	0.338432048
GSEC	-1.47115	0.727157	-2.023154	0.04305724	NA
DCAF15	0.386229	0.190913	2.023064	0.04306659	0.338438077
RNF4	0.22615	0.111806	2.022699	0.04310415	0.338488876
GIN1	0.463803	0.229363	2.022138	0.04316208	0.338699397
ENSG00000279576	-0.490346	0.242573	-2.021437	0.0432345	0.339023273
IRF4	-0.221806	0.10978	-2.020463	0.04333542	0.339406472
HRK	-0.761769	0.377144	-2.019835	0.04340048	0.339406472
NUTF2	-0.137446	0.068049	-2.019794	0.04340472	0.339406472
PPP1R12A	0.207362	0.102667	2.019756	0.04340872	0.339406472
RTL8A	-0.301348	0.149249	-2.019089	0.04347801	0.339406472
ZNF823	0.834231	0.413205	2.018927	0.04349477	0.339406472
PLEKHG2	0.72793	0.360565	2.018861	0.04350166	0.339406472
G6PC3	-0.403898	0.20011	-2.018375	0.04355227	0.339557918
CA11	-0.279549	0.138555	-2.017607	0.04363218	0.339804013
OSTC	-0.144861	0.071803	-2.017472	0.04364627	0.339804013
JAML	-1.519208	0.753529	-2.016125	0.04378688	0.340621284
RBM15B	-0.314018	0.155795	-2.015588	0.0438431	0.340621284
HPS1	0.207494	0.102946	2.015568	0.04384513	0.340621284
ALG1L2	1.465484	0.728132	2.012663	0.0441501	0.342745813
UBE2W	0.226182	0.112407	2.012172	0.04420185	0.342903014
KCTD18	-0.308384	0.153367	-2.010758	0.04435107	0.343558126
XPO4	-0.297325	0.147868	-2.010748	0.04435211	0.343558126
ZBTB1	0.326096	0.162221	2.010199	0.04441017	0.343558126
CENPX	0.15826	0.07873	2.010176	0.04441256	0.343558126
GTF2H2C	-0.336497	0.167498	-2.008963	0.0445411	0.34430777
RBM4	-0.218645	0.108861	-2.008474	0.044593	0.344464293
VRK2	0.323793	0.161381	2.006393	0.04481434	0.345650708
MRPS7	-0.150362	0.07495	-2.006158	0.04483936	0.345650708
RPL30	0.07545	0.03761	2.006135	0.04484186	0.345650708
MEGF6	-0.991337	0.494381	-2.005208	0.04494085	NA
MRPL34	-0.12671	0.063226	-2.004073	0.04506224	0.347103609
ENSG00000272008	1.082952	0.540495	2.003629	0.04510983	NA
ATF7IP2	-0.259694	0.12965	-2.003038	0.04517317	0.347445105

UBA6.AS1	-0.466892	0.233097	-2.002997	0.04517766	0.347445105
ZNF587	0.326344	0.162947	2.002767	0.04520234	0.347445105
TFEB	0.347029	0.173337	2.002048	0.04527953	0.347557653
CSRNP2	0.59878	0.299085	2.002036	0.04528085	0.347557653
ENSG00000224086	-0.406208	0.202967	-2.001349	0.04535481	0.347880011
TLR10	0.229825	0.114862	2.000882	0.04540509	0.348020389
TRAF5	0.245004	0.122484	2.000285	0.04546953	0.348269074
TATDN2	0.424612	0.212471	1.998445	0.04566841	0.348906735
ZFYVE1	0.535515	0.267968	1.998428	0.04567031	0.348906735
LARP4B	0.282928	0.141593	1.998184	0.04569675	0.348906735
CAMK2D	-0.318462	0.159383	-1.99809	0.04570688	0.348906735
KCTD3	-0.964063	0.482493	-1.998085	0.0457074	NA
LRRC14	-0.375261	0.187815	-1.998033	0.04571307	0.348906735
NOP58	-0.134273	0.067218	-1.99757	0.04576334	0.348939233
RASSF7	0.258881	0.129609	1.997403	0.04578144	0.348939233
PURA	0.297059	0.14877	1.996762	0.04585103	0.349148619
MAMLD1	-1.386903	0.694614	-1.996652	0.04586305	NA
RTCA	0.28336	0.141924	1.996559	0.04587307	0.349148619
ENSG00000203546	-0.68703	0.344287	-1.995514	0.04598681	0.349705126
ENSG00000225302	1.479244	0.741306	1.995458	0.04599295	NA
RPL39L	0.456604	0.228899	1.994782	0.04606664	0.349705126
GRAP	0.371712	0.186349	1.994703	0.0460753	0.349705126
GFM2	0.432767	0.216996	1.994358	0.04611289	0.349705126
CFLAR	0.260042	0.130405	1.994111	0.04613993	0.349705126
POLR2H	-0.185951	0.093253	-1.994056	0.04614594	0.349705126
ZC3H8	-0.244595	0.122676	-1.993826	0.0461711	0.349705126
OTUD1	0.54634	0.27423	1.99227	0.04634143	0.350330631
MRPL28	-0.156484	0.078555	-1.992037	0.04636699	0.350330631
SH3YL1	-0.295353	0.148274	-1.991938	0.04637789	0.350330631
FANCM	-0.522481	0.262303	-1.991896	0.04638244	0.350330631
PMF1.BGLAP	-1.711529	0.859358	-1.991636	0.046411	NA
RTN4	0.153864	0.07729	1.99074	0.04650944	0.350885153
CBX7	0.295546	0.148469	1.990627	0.04652186	0.350885153
POLR3A	-0.346302	0.174015	-1.99007	0.04658327	0.350885153
FAM49A	0.375152	0.188558	1.989586	0.04663659	0.350885153
DTWD1	0.228358	0.11478	1.989527	0.04664303	0.350885153
ENSG00000263884	-1.56198	0.785146	-1.989414	0.04665558	NA
GSTO2	1.769103	0.889352	1.989204	0.04667864	NA
CCDC66	0.314885	0.158297	1.989202	0.04667893	0.350885153
NECTIN1	-0.864675	0.434689	-1.989178	0.04668152	0.350885153
METTL26	-0.196644	0.098877	-1.988779	0.04672555	0.350973673
ACSL5	0.342347	0.172173	1.988389	0.04676863	0.35105155
MFSD13A	-0.491484	0.247215	-1.988085	0.0468023	0.35105155
ATP6V0A2	-0.287689	0.144727	-1.98781	0.04683268	0.35105155
IL6ST	-0.443596	0.223218	-1.987275	0.04689193	0.35108654
RYBP	0.383279	0.192875	1.987185	0.04690186	0.35108654
C9orf72	-0.333596	0.167948	-1.986308	0.0469991	0.351572622

ZNF439	0.272964	0.137529	1.984776	0.04716936	0.35245554
PRR3	-0.254217	0.128106	-1.984427	0.04720821	0.35245554
NBPF9	0.59719	0.300973	1.984199	0.0472337	0.35245554
CETP	-0.618396	0.311698	-1.983955	0.04726088	0.35245554
ENSG00000272221	0.843618	0.425255	1.983792	0.04727904	0.35245554
IMP3	-0.162517	0.081941	-1.983352	0.04732808	0.352579629
IFT22	-0.276538	0.139454	-1.983009	0.04736646	0.352624155
ENSG00000262292	0.81881	0.41303	1.982445	0.04742946	0.352838191
RBAK.RBAKDN	0.268423	0.13545	1.981716	0.04751108	0.352838191
SERTAD2	0.289154	0.145914	1.981674	0.04751578	0.352838191
ENSG00000273329	-0.441899	0.223002	-1.981592	0.04752488	0.352838191
DDX5	-0.191589	0.096754	-1.980172	0.04768424	0.353779996
TACC1	0.27281	0.137824	1.979407	0.04777017	0.354066456
CYP1B1	1.335432	0.674768	1.979099	0.04780484	NA
ENSG00000261386	0.530764	0.268235	1.978729	0.04784649	0.354066456
MTFP1	-0.215783	0.109053	-1.978692	0.04785074	0.354066456
HVCN1	-0.280421	0.141734	-1.978507	0.04787155	0.354066456
MED11	0.220799	0.111625	1.97805	0.04792311	0.354066456
PCTP	-0.383395	0.193829	-1.978009	0.04792775	0.354066456
SNRNP200	-0.175482	0.088726	-1.977806	0.04795057	0.354066456
CDC5L	0.188314	0.095288	1.976263	0.04812496	0.355113225
MYL12A	0.153001	0.077435	1.975859	0.04817075	0.355188817
QPRT	-0.740432	0.374789	-1.975597	0.04820047	0.355188817
KIF1C	0.56593	0.286601	1.974626	0.04831061	0.355759582
GLRX2	0.223504	0.113245	1.973633	0.0484235	0.35603483
PET100	-0.136227	0.069025	-1.973592	0.04842812	0.35603483
FAM98B	-0.334184	0.169361	-1.973207	0.048472	0.35603483
NUP50.DT	-0.400603	0.203028	-1.973147	0.04847883	0.35603483
CYTH3	-0.64716	0.328071	-1.972624	0.04853843	0.356223109
CAPN2	0.833107	0.422394	1.972348	0.04856993	0.356223109
INPP5D	0.20252	0.102716	1.971649	0.04864968	0.356567785
VAMP5	-0.35476	0.180058	-1.97025	0.04880973	0.357344465
STAT5A	-0.258068	0.130989	-1.970149	0.04882132	0.357344465
LBX2.AS1	1.453918	0.738623	1.968417	0.04902011	NA
MFSD4B	0.443491	0.225421	1.967391	0.04913816	0.359416393
ZNF91	0.229166	0.116499	1.967111	0.04917044	0.359416393
FAN1	0.580656	0.295352	1.965979	0.04930101	0.360128977
FUNDC2	-0.190464	0.0969	-1.965582	0.04934692	0.360222556
CPPED1	0.64959	0.330544	1.965217	0.04938917	0.360289362
FAM136A	-0.164219	0.083589	-1.964588	0.04946198	0.360578842
ENSG00000240859	-0.989368	0.503729	-1.964089	0.04951974	NA
CNST	0.339213	0.172708	1.964081	0.04952067	0.360765041
SPIN3	1.842443	0.938139	1.963933	0.04953783	NA
RSBN1L	0.178039	0.090686	1.963261	0.04961589	0.361136521
BTF3L4	-0.184792	0.094146	-1.962821	0.04966698	0.361136521
CREBBP	0.194204	0.098943	1.962785	0.0496712	0.361136521
ATAT1	0.325775	0.166026	1.962198	0.04973949	0.361391583

PRR7	-0.411719	0.209936	-1.961162	0.04986009	0.36184936
QPCTL	-0.804294	0.410127	-1.961086	0.04986899	0.36184936
EAF1	-0.311075	0.15866	-1.960644	0.04992056	0.361982234
