# Supplementary material for: Single-cell landscape analysis unravels molecular programming of the human B cell compartment in chronic GVHD
Source: JCI Insight. 2023 Jun 8;8(11):e169732. doi: 10.1172/jci.insight.169732 (PMC10393230; doi:10.1172/jci.insight.169732)
Supplement: Supplemental table 7 [file jciinsight-8-169732-s114.pdf]

Supplemental Table 7. All DEGs by cluster in the blood B cell scRNA-Seq dataset for ATRA-treated B cells. Log2FC values represent the change in expression for the gene of interest in ATRA-treated B cells when compared to untreated B cells from all 8 allo-HCT patients combined, along with associated statistical data.

Cluster 1	log2FC	lfcSE	stat	pvalue	padj
VPREB3	3.239544	0.091617	35.359826	< 2.22e-16	< 2.22e-16
AHI1	1.715068	0.138638	12.370799	< 2.22e-16	< 2.22e-16
PLAAT4	2.070436	0.176793	11.711066	< 2.22e-16	< 2.22e-16
RRBP1	1.952289	0.168521	11.584832	< 2.22e-16	< 2.22e-16
DUS2	1.59709	0.151949	10.510684	< 2.22e-16	< 2.22e-16
CD38	3.185154	0.336321	9.470584	< 2.22e-16	< 2.22e-16
DENND6B	2.261815	0.245054	9.22987	< 2.22e-16	< 2.22e-16
CD1D	3.322237	0.371023	8.954262	< 2.22e-16	5.40E-16
SLC12A4	2.522336	0.289223	8.721086	< 2.22e-16	3.87E-15
DPEP2	1.010012	0.121526	8.311097	< 2.22e-16	1.20E-13
RARA.AS1	2.080188	0.261336	7.95983	1.72E-15	1.98E-12
LCAT	2.443899	0.311124	7.855068	4.00E-15	4.21E-12
AGO1	1.045363	0.13458	7.767574	8.00E-15	7.78E-12
RAB37	1.342816	0.176705	7.599198	2.98E-14	2.69E-11
VPREB1	4.622933	0.622501	7.426388	1.12E-13	9.40E-11
MRPS6	0.957351	0.131557	7.277101	3.41E-13	2.69E-10
C16orf54	1.165205	0.162778	7.158264	8.17E-13	6.07E-10
SLC5A3	1.030773	0.148455	6.943318	3.83E-12	2.69E-09
P2RX5	1.038367	0.15263	6.803172	1.02E-11	6.81E-09
SIGIRR	0.78388	0.117274	6.684187	2.32E-11	1.47E-08
RMDN2	1.974119	0.296347	6.661511	2.71E-11	1.63E-08
PNOC	1.665266	0.250408	6.65021	2.93E-11	1.68E-08
AGPAT5	0.888394	0.135542	6.55438	5.59E-11	3.07E-08
FAM3C	0.801948	0.123515	6.492692	8.43E-11	4.44E-08
MPEG1	1.614321	0.25443	6.344853	2.23E-10	1.13E-07
SEPTIN9	0.568186	0.090558	6.274284	3.51E-10	1.71E-07
FCRL1	1.113825	0.182636	6.098604	1.07E-09	5.01E-07
GPX1	0.568352	0.093355	6.088055	1.14E-09	5.16E-07
GRN	0.862574	0.142076	6.071216	1.27E-09	5.53E-07
MS4A1	-0.60819	0.100407	-6.05726	1.38E-09	5.83E-07
MAPKAPK2	1.028439	0.178178	5.771964	7.84E-09	3.19E-06
P2RX5.TAX1BP3	1.623417	0.288252	5.631932	1.78E-08	7.04E-06
GBP4	0.775912	0.142806	5.433327	5.53E-08	2.12E-05
SEL1L3	0.986761	0.183249	5.384804	7.25E-08	2.70E-05
XYLT1	1.234467	0.233752	5.281092	1.28E-07	4.64E-05
APOL3	0.821357	0.157845	5.203578	1.95E-07	6.86E-05
MYL2	4.501103	0.881083	5.108602	3.25E-07	0.00011083
FAM117A	0.697754	0.137884	5.060428	4.18E-07	0.00013909
IRAK2	1.101905	0.218317	5.047276	4.48E-07	0.00014519
ASB2	4.8577	0.97452	4.984712	6.21E-07	0.000196014
ENSG00000214797	2.590847	0.521524	4.967835	6.77E-07	0.000208646

CD72	0.972445	0.197063	4.934683	8.03E-07	0.000241512
RABGAP1L	0.787875	0.160196	4.918192	8.73E-07	0.000256659
DBNL	0.583976	0.120595	4.84246	1.28E-06	0.000368258
PLAAT3	3.245782	0.68321	4.750783	2.03E-06	0.000568941
PPP1R18	0.534823	0.115085	4.647187	3.36E-06	0.000924256
ICAM2	0.810193	0.175718	4.610751	4.01E-06	0.001078589
CHST15	0.876363	0.190651	4.596685	4.29E-06	0.001129952
TRABD	0.437682	0.095562	4.580065	4.65E-06	0.001198602
SPRY1	0.889388	0.195263	4.55483	5.24E-06	0.001324857
CD79B	0.281374	0.062569	4.497007	6.89E-06	0.00170738
MCL1	0.502184	0.113125	4.439203	9.03E-06	0.002193938
ATXN10	0.57549	0.130103	4.423342	9.72E-06	0.002316874
ETFB	0.547662	0.124261	4.407367	1.05E-05	0.002448264
SMC6	-0.575894	0.131498	-4.379475	1.19E-05	0.002732963
TNFRSF13B	-1.642074	0.380392	-4.316788	1.58E-05	0.003572002
ST3GAL1	0.879332	0.204035	4.309722	1.63E-05	0.003623368
LHFPL2	1.250313	0.291873	4.283752	1.84E-05	0.003947746
GPR183	-0.615168	0.143628	-4.283057	1.84E-05	0.003947746
SREBF1	1.408247	0.329841	4.269477	1.96E-05	0.004125996
ZNF563	1.007111	0.236517	4.258092	2.06E-05	0.00427062
PPP2R5C	0.432457	0.101785	4.248723	2.15E-05	0.00438135
PXK	0.504086	0.119392	4.222119	2.42E-05	0.004853766
SNX18	0.965772	0.230012	4.198787	2.68E-05	0.005297792
SLC2A5	1.180447	0.281795	4.189021	2.80E-05	0.005445882
GDPGP1	1.365762	0.329251	4.14809	3.35E-05	0.006418204
GLRX	-0.497023	0.119937	-4.144032	3.41E-05	0.006435413
SPOCK2	0.588104	0.143118	4.109215	3.97E-05	0.007376723
CABLES1	-1.920071	0.468108	-4.101773	4.10E-05	0.007507689
PUS1	0.899317	0.220029	4.087256	4.37E-05	0.00787892
NRM	0.645795	0.16107	4.009416	6.09E-05	0.010832141
PLEKHA2	0.38904	0.097543	3.9884	6.65E-05	0.011673421
CCL2	4.592547	1.154323	3.978563	6.93E-05	0.0120003
ENSG00000273319	0.69243	0.175228	3.951598	7.76E-05	0.013254993
SLC5A5	3.627945	0.920823	3.939892	8.15E-05	0.0137331
BTBD7	0.510624	0.12976	3.935144	8.31E-05	0.013823163
IRF4	0.900479	0.231307	3.893004	9.90E-05	0.01624674
NEURL2	1.992856	0.515316	3.867248	0.00011007	0.017462707
NCF1	0.279332	0.072236	3.866939	0.00011021	0.017462707
ENSG00000275799	0.608916	0.157499	3.866149	0.000110567	0.017462707
C12orf65	0.551271	0.142983	3.85551	0.000115489	0.018014788
CD82	-0.558455	0.145518	-3.837713	0.000124185	0.019124953
AP3B1	0.549058	0.143175	3.834866	0.000125633	0.019124953
NMT2	-0.492979	0.128716	-3.829968	0.00012816	0.019257731
HCK	-0.970205	0.253495	-3.827306	0.000129553	0.019257731
CD79A	0.424419	0.11279	3.762925	0.000167938	0.024673174
TAF4B	0.957342	0.255662	3.74456	0.00018071	0.026244523
ADAM19	0.675578	0.181298	3.726341	0.00019428	0.027690576

PWP1	0.393781	0.105703	3.725342	0.00019505	0.027690576
SMAD3	0.69559	0.188467	3.690778	0.000223569	0.031386638
ANAPC16	0.28018	0.076185	3.677639	0.000235403	0.032684785
FCER2	1.329128	0.36175	3.674161	0.000238633	0.03277307
IRF2BPL	1.159442	0.317642	3.650152	0.000262085	0.035460423
ENSG00000226380	0.809307	0.221821	3.648464	0.000263813	0.035460423
PSD3	-1.23821	0.340215	-3.639488	0.000273181	0.036333093
CLEC17A	0.804011	0.221566	3.628759	0.000284787	0.037482161
SLC25A5	-0.261237	0.072351	-3.610706	0.000305364	0.039776046
SIDT1	-0.694796	0.192758	-3.604495	0.000312761	0.040323775
H3F3A	0.311838	0.086677	3.597704	0.000321039	0.040972978
CALHM6	0.467378	0.130364	3.585167	0.000336862	0.042562567
PAOX	0.793202	0.221514	3.580819	0.000342519	0.042848803
CD180	0.729607	0.204661	3.564947	0.000363929	0.045080856
IFI30	0.62223	0.178034	3.495007	0.00047405	0.058056203
ENSG00000089127	0.683138	0.195581	3.492866	0.000477867	0.058056203
ARHGEF1	0.344339	0.099399	3.464226	0.000531759	0.063988347
PLPP3	3.212139	0.930052	3.453719	0.000552912	0.065906108
RASSF3	0.650355	0.188736	3.445853	0.000569261	0.067220664
CTNNB1	0.479561	0.13937	3.440933	0.000579712	0.067820985
IL1B	3.239503	0.942287	3.437916	0.00058621	0.067951984
CIB1	0.28996	0.08454	3.429843	0.00060393	0.069369612
ENSG00000261448	1.426513	0.416719	3.423197	0.000618892	0.070447717
CHL1	1.625927	0.475561	3.418963	0.000628603	0.070914292
LINC00513	0.539292	0.158102	3.41103	0.000647179	0.072363758
TRANK1	-0.5732	0.16894	-3.392915	0.00069153	0.076644563
PALLD	2.910568	0.858589	3.389944	0.000699069	0.07680638
MMACHC	1.436586	0.424087	3.38748	0.000705378	0.076831517
MOGS	-0.825814	0.244105	-3.383023	0.000716926	0.077421837
CPNE5	0.929721	0.275187	3.378509	0.0007288	0.078037158
ENSG00000227486	0.600087	0.177822	3.374646	0.000739107	0.078475794
MAP3K1	0.434953	0.129105	3.369001	0.000754411	0.079433194
NFATC3	0.393448	0.117273	3.354977	0.000793717	0.082881061
AKAP12	2.973588	0.891141	3.336832	0.000847393	0.087760738
FXYD5	-0.23377	0.070442	-3.318616	0.000904647	0.092928574
SMIM3	3.085858	0.930725	3.31554	0.000914661	0.093199532
ZFP36L1	-0.350535	0.105899	-3.310096	0.00093264	0.094271229
GABPB1	0.286211	0.08673	3.300024	0.000966765	0.096945016
RINL	0.468097	0.142199	3.291838	0.000995349	0.099025497
ZNF3	0.60168	0.182972	3.28837	0.001007692	0.099470182
MBD6	0.796908	0.242657	3.284087	0.001023132	0.099808849
SLC45A3	1.001573	0.305075	3.283045	0.001026921	0.099808849
CERK	0.677761	0.206673	3.27939	0.001040318	0.100339071
LMNA	-2.649884	0.810167	-3.270786	0.001072491	0.102658488
TSPAN18	3.381633	1.035753	3.264903	0.001095015	0.104026384
ACP5	0.858481	0.264079	3.250852	0.001150596	0.107761145
C9orf16	-0.265397	0.081644	-3.250657	0.001151385	0.107761145

ERAP1	0.573238	0.17693	3.239914	0.001195659	0.111081971
NUP210	0.378681	0.117395	3.225711	0.001256602	0.115891755
TNFRSF18	-1.254858	0.38961	-3.220808	0.001278297	0.117038323
PLXNB2	1.598995	0.50009	3.197417	0.001386645	0.125072093
A1BG	-0.350948	0.10983	-3.195369	0.001396521	0.125072093
CYTIP	-0.191769	0.06003	-3.194545	0.001400514	0.125072093
MEF2A	0.557722	0.174643	3.193491	0.001405638	0.125072093
DDIT4	1.094364	0.345667	3.165946	0.001545796	0.136581347
GLIPR2	0.514372	0.162644	3.162559	0.001563889	0.137220376
ENSG00000261766	0.722994	0.22896	3.157724	0.001590058	0.138554331
C8orf58	-1.504791	0.478008	-3.148042	0.001643679	0.142197975
LCK	0.68596	0.218032	3.146145	0.001654381	0.142197975
POU2AF1	0.281908	0.090007	3.132072	0.001735774	0.148185837
ADD1	0.349358	0.111919	3.121532	0.001799126	0.152563443
TIPARP	-0.811544	0.260505	-3.115278	0.001837715	0.154796898
LY86	-0.340526	0.1094	-3.112663	0.001854078	0.155140887
LINC02576	-1.2031	0.389879	-3.085827	0.002029872	0.167963121
BIN2	0.65178	0.211258	3.085237	0.002033902	0.167963121
USP8	0.274906	0.089377	3.075788	0.002099467	0.171665307
ZNF26	0.42254	0.137417	3.074875	0.002105906	0.171665307
SCIMP	1.09172	0.355341	3.072315	0.002124056	0.172034932
CD83	-0.339848	0.111081	-3.059454	0.00221741	0.177331465
DAAM1	-0.680209	0.222331	-3.059439	0.002217521	0.177331465
LRRC61	0.746927	0.244587	3.053829	0.002259406	0.178834861
CTSS	0.274396	0.089874	3.053137	0.002264628	0.178834861
EML6	-1.88302	0.617265	-3.050583	0.002283972	0.179242171
CD99	-0.536373	0.175987	-3.047802	0.002305215	0.179792531
KANSL3	0.769244	0.253388	3.035839	0.002398678	0.18593433
FCMR	0.389535	0.128777	3.024889	0.002487243	0.191623905
JDP2	1.1989	0.397545	3.015759	0.002563373	0.196292198
RNF207	2.091936	0.695054	3.009747	0.002614655	0.197836104
VPS26B	-1.765548	0.586615	-3.009724	0.00261485	0.197836104
TMEM41B	0.539439	0.179554	3.004335	0.002661625	0.200176353
ENSG00000240859	2.230564	0.743793	2.998903	0.002709534	0.202573711
CD5	-1.499014	0.502599	-2.982521	0.002858848	0.212479679
LINC02453	1.283003	0.431427	2.973858	0.002940813	0.217293411
IK	0.25904	0.087237	2.969396	0.002983859	0.218666894
AHNAK	-0.997392	0.336119	-2.967373	0.003003564	0.218666894
FOXN3	0.351252	0.118445	2.965521	0.003021704	0.218666894
PTPN6	0.514237	0.173446	2.964817	0.003028627	0.218666894
ISG20	-0.322367	0.109012	-2.957182	0.003104653	0.222882312
PLEKHO1	0.455326	0.15422	2.952438	0.003152751	0.224621733
CLCN4	1.536938	0.520767	2.951296	0.003164438	0.224621733
FCRL2	0.606028	0.206891	2.929207	0.003398279	0.239872927
VHL	0.377488	0.129243	2.920772	0.003491652	0.245094578
NDUFB9	0.261051	0.089684	2.910774	0.003605345	0.25167701
MLXIP	0.434494	0.149919	2.898197	0.003753152	0.26055539

GBP7	1.029267	0.355378	2.896258	0.003776417	0.260737883
CYBB	0.509676	0.176108	2.89412	0.003802231	0.261093386
CDC26	-0.303667	0.105364	-2.882069	0.003950727	0.269823947
SLC2A3	-0.661164	0.229751	-2.877747	0.004005262	0.271966012
WNT10A	-1.001444	0.348185	-2.876185	0.00402514	0.271966012
ENSG00000271204	0.530962	0.185095	2.868591	0.004123041	0.276036758
AK1	0.796151	0.277838	2.86552	0.00416325	0.276036758
PDCD4	0.545849	0.190519	2.865069	0.004169183	0.276036758
SLCO4A1	-1.052977	0.367557	-2.864796	0.004172776	0.276036758
HTR3A	1.228382	0.429412	2.860616	0.004228187	0.278245559
GCHFR	-0.50638	0.177908	-2.846303	0.004423003	0.289557724
SLC1A4	-1.380221	0.485606	-2.842266	0.004479412	0.291739039
ATG2A	0.611877	0.215487	2.839505	0.004518354	0.292766152
OTUD7A	1.829475	0.644723	2.837614	0.004545213	0.293003883
SLC43A2	0.295343	0.104212	2.834064	0.004596013	0.294774735
WDFY2	-0.471378	0.166765	-2.826605	0.004704428	0.29851522
CTSA	0.465178	0.1646	2.82611	0.004711712	0.29851522
ICA1	2.948529	1.045229	2.82094	0.004788319	0.29851522
TP53I11	-0.685886	0.243157	-2.820758	0.004791027	0.29851522
ENSG00000270019	0.839659	0.297783	2.819698	0.004806886	0.29851522
COA1	0.413426	0.146807	2.816121	0.004860741	0.29851522
FADS3	-0.390184	0.138588	-2.815417	0.0048714	0.29851522
ENSG00000279278	0.650736	0.231171	2.814953	0.00487844	0.29851522
SRPRB	-0.675226	0.239918	-2.814404	0.004886778	0.29851522
NANP	1.271546	0.45184	2.814153	0.004890594	0.29851522
TIMM23B	0.871122	0.309796	2.811923	0.004924637	0.299148003
CTDSP1	0.342076	0.121884	2.806563	0.005007313	0.30271482
AP3S1	0.387119	0.138436	2.796381	0.005167842	0.307947269
ENSG00000258377	1.668491	0.596931	2.795114	0.005188143	0.307947269
DNMT3A	0.900307	0.32224	2.793901	0.005207649	0.307947269
SEMA4A	1.567559	0.56124	2.793027	0.005221736	0.307947269
ZDBF2	-1.903363	0.681745	-2.7919	0.005239953	0.307947269
CD22	0.492349	0.176351	2.79187	0.005240441	0.307947269
CRX	1.549056	0.55514	2.790389	0.005264472	0.307947269
ABCB1	0.642207	0.230693	2.783819	0.005372296	0.312806264
CENPM	0.483589	0.173891	2.780996	0.005419244	0.313097887
ARHGAP4	0.387309	0.139376	2.77888	0.005454662	0.313097887
IZUMO4	-0.551379	0.198513	-2.777549	0.005477066	0.313097887
ARID3A	0.557055	0.20066	2.776115	0.005501266	0.313097887
KLF8	0.466097	0.167912	2.775839	0.005505954	0.313097887
MYADM	0.62661	0.225833	2.774658	0.005525986	0.313097887
VSIG10L	1.898372	0.68531	2.770093	0.005604028	0.316102206
MANF	0.386559	0.139775	2.765591	0.005681981	0.319074819
FAM167A	1.387309	0.502111	2.762955	0.005728074	0.319203412
LSP1	0.326205	0.11808	2.762572	0.005734798	0.319203412
ST13	-0.150864	0.054643	-2.76091	0.005764048	0.319424348
MYC	0.336197	0.121878	2.758467	0.005807322	0.320417098

GPANK1	0.313919	0.114017	2.753273	0.005900272	0.32413018
IL21R	0.663703	0.241482	2.748454	0.005987701	0.327278568
POLD4	0.263775	0.096016	2.747209	0.006010477	0.327278568
TP53	0.375057	0.13659	2.745858	0.006035291	0.327278568
ROR1	-1.690949	0.616916	-2.740971	0.006125786	0.329213979
GEN1	-0.932234	0.340134	-2.740787	0.006129214	0.329213979
TBC1D9	0.794872	0.290129	2.73972	0.006149149	0.329213979
WDR97	1.699877	0.621382	2.735638	0.00622595	0.331919296
SERPINB6	0.596341	0.218109	2.734143	0.006254279	0.332028615
RAB3IP	-0.525092	0.192537	-2.727223	0.006386984	0.336325938
SEC61B	-0.330612	0.12123	-2.727147	0.006388463	0.336325938
ENSG00000272644	1.707326	0.626865	2.723596	0.006457553	0.338230306
RNF7	-0.272583	0.100156	-2.721577	0.006497125	0.338230306
TTBK2	-0.805726	0.296268	-2.719582	0.006536458	0.338230306
PPP1CA	-0.181757	0.066842	-2.719187	0.00654426	0.338230306
CDC42SE1	0.52517	0.193186	2.718469	0.006558482	0.338230306
ENSG00000260136	-1.402884	0.51684	-2.714348	0.006640639	0.34107509
ENSG00000261884	0.639585	0.235975	2.710395	0.006720309	0.342487575
APOBEC3D	0.472068	0.17425	2.709135	0.006745881	0.342487575
CECR7	1.534627	0.5665	2.708959	0.006749458	0.342487575
SLC25A45	0.605708	0.224269	2.700814	0.006916993	0.348983406
NKILA	1.264259	0.468234	2.700059	0.006932713	0.348983406
TOP1MT	-0.603868	0.224366	-2.691434	0.007114553	0.356715793
ENSG00000130520	-0.287846	0.107007	-2.689978	0.007145663	0.356859512
TBL1X	-0.615529	0.229058	-2.687214	0.007205076	0.358409974
HNRNPK	0.192157	0.071782	2.676955	0.007429462	0.367678199
HIVEP2	0.742035	0.277287	2.676048	0.007449594	0.367678199
BRD3OS	-0.711749	0.266378	-2.671948	0.007541243	0.369681575
BHLHE40	0.672632	0.251934	2.669878	0.007587878	0.369681575
C7orf50	-0.246975	0.09254	-2.668834	0.007611517	0.369681575
ZCCHC17	0.335489	0.12579	2.66705	0.007652029	0.369681575
ENSG00000259768	1.272001	0.476976	2.6668	0.007657716	0.369681575
METTL7A	0.345447	0.129634	2.664791	0.007703613	0.369681575
TCTN3	-0.47027	0.176527	-2.664006	0.007721622	0.369681575
ENSG00000249476	-1.971213	0.740165	-2.663208	0.007739954	0.369681575
TENT5C	-0.359334	0.135079	-2.660178	0.007809932	0.369681575
LBR	0.309841	0.116477	2.660105	0.007811626	0.369681575
HINT2	0.287688	0.10815	2.660088	0.007812029	0.369681575
AGAP3	2.592503	0.975285	2.658199	0.007855942	0.37037249
CNOT7	0.208678	0.078559	2.656304	0.007900233	0.371075981
TRBC2	-0.221709	0.083612	-2.651635	0.008010312	0.372658303
P2RY8	0.351964	0.132881	2.648723	0.008079653	0.372658303
ENSG00000273445	3.330759	1.257797	2.64809	0.008094809	NA
CARD11	0.561688	0.212226	2.646645	0.008129469	0.372658303
CYBC1	-0.231295	0.087393	-2.646614	0.008130218	0.372658303
CARNMT1	0.654125	0.247161	2.64655	0.008131743	0.372658303
ARL3	-0.514534	0.194482	-2.645662	0.008153118	0.372658303

PLCXD1	0.696559	0.263318	2.645317	0.008161433	0.372658303
FUNDC1	0.522282	0.197501	2.644454	0.008182292	0.372658303
GTF2H5	0.30721	0.11622	2.643359	0.008208804	0.372658303
RBX1	-0.209763	0.07938	-2.642532	0.008228862	0.372658303
NIPAL4	1.37713	0.521728	2.639553	0.008301536	0.374606811
CCDC12	0.23257	0.088202	2.636802	0.008369173	0.37520945
DHFR2	0.681154	0.258352	2.636533	0.0083758	0.37520945
SH3KBP1	-0.275976	0.10476	-2.63435	0.00842985	0.37520945
IQSEC1	-0.371486	0.141025	-2.634196	0.008433675	0.37520945
GMEB1	0.429146	0.163149	2.630385	0.008528813	0.375931114
ENSG00000234484	1.965427	0.74722	2.630318	0.008530491	0.375931114
CTSZ	0.342007	0.130061	2.629583	0.008548978	0.375931114
JCHAIN	1.128514	0.429337	2.628501	0.008576206	0.375931114
MPV17L	1.107449	0.421568	2.62698	0.008614645	0.375931114
JADE1	0.360064	0.137092	2.626436	0.008628415	0.375931114
EMC6	-0.381964	0.145747	-2.62074	0.008773915	0.3786344
FKBP5	0.96305	0.367573	2.620027	0.008792273	0.3786344
DDX28	0.474302	0.181073	2.619391	0.008808703	0.3786344
EIF4G3	0.513396	0.196003	2.619328	0.00881033	0.3786344
KLF3	0.393256	0.150359	2.615448	0.008911061	0.380108621
HDAC9	0.538837	0.206045	2.615146	0.00891892	0.380108621
TMEM175	0.678585	0.259543	2.614536	0.008934884	0.380108621
GPR157	0.867439	0.332227	2.610981	0.009028283	0.382082665
CYLD	-0.299242	0.114632	-2.610471	0.009041766	0.382082665
ENSG00000270589	1.720093	0.659846	2.606811	0.009138976	0.384527372
RALA	-0.390998	0.150037	-2.606006	0.009160486	0.384527372
PTPN12	-0.784838	0.301782	-2.600678	0.009303975	0.38900645
SLCO5A1	1.279357	0.492105	2.599765	0.009328766	0.38900645
SELL	0.569664	0.219339	2.597178	0.009399309	0.390319449
DPEP3	1.616677	0.622673	2.596349	0.009422037	0.390319449
INVS	0.89976	0.346835	2.594205	0.009480991	0.391478158
RASA3	0.75459	0.291538	2.588305	0.009644963	0.3969515
FUCA1	-0.68137	0.263622	-2.584646	0.009747899	0.399885384
MMP7	1.879867	0.728215	2.581472	0.009837996	0.402275332
MOSPD3	-0.468293	0.182502	-2.565965	0.010288931	0.419356899
AFF3	-0.253559	0.098878	-2.564363	0.010336548	0.419943048
TNK2	0.423042	0.165314	2.559026	0.010496598	0.425078567
PCBP4	-1.001591	0.391846	-2.556084	0.010585762	0.427319807
ADCK1	1.205057	0.472055	2.552789	0.010686434	0.427679351
SH3BGRL3	-0.185185	0.072644	-2.549208	0.010796774	0.427679351
DMD	-0.611274	0.239794	-2.549161	0.010798232	0.427679351
SVBP	-0.269944	0.105896	-2.549135	0.010799062	0.427679351
MMP11	0.71074	0.278834	2.548967	0.010804249	0.427679351
KCTD10	0.624265	0.245024	2.547777	0.01084117	0.427679351
CCDC106	-0.532162	0.208965	-2.546663	0.010875854	0.427679351
ZNF337.AS1	0.934388	0.366969	2.546231	0.010889297	0.427679351
ENSG00000180448	0.288126	0.113172	2.545911	0.010899308	0.427679351

PSMB10	0.342761	0.134739	2.543881	0.01096283	0.428545269
SEC23IP	-0.564106	0.221823	-2.543042	0.01098921	0.428545269
ENSG00000228106	0.449616	0.176998	2.54024	0.011077648	0.430664882
SRGN	0.543289	0.214103	2.537515	0.011164252	0.43270038
TELO2	0.557883	0.220199	2.533547	0.011291457	0.436292224
GTF3A	0.201389	0.07956	2.531282	0.011364629	0.43662599
ENSG00000263884	1.563218	0.617594	2.531141	0.011369209	0.43662599
ENSG00000253535	0.789119	0.312334	2.526526	0.011519682	0.440414568
ENSG00000166927	-0.835111	0.330609	-2.525981	0.011537572	0.440414568
SP110	0.340164	0.134758	2.524267	0.011593983	0.440759899
CLIC3	1.503333	0.595927	2.522681	0.011646399	0.440759899
ZNF239	1.365026	0.541133	2.522534	0.011651271	0.440759899
FBP1	1.124717	0.446415	2.519445	0.011753992	0.44321587
HSD11B1L	-1.075723	0.4272	-2.518076	0.011799788	0.44321587
PLA2G7	3.754507	1.491405	2.517431	0.011821428	0.44321587
ENSG00000176320	2.680585	1.065726	2.515266	0.011894276	0.444627727
ENSG00000261786	2.637035	1.049446	2.512788	0.011978135	0.44563712
DNAJA2	0.307701	0.122504	2.511769	0.012012782	0.44563712
KDM2B	0.384072	0.152935	2.511349	0.012027088	0.44563712
METTL21A	-0.315669	0.125912	-2.50706	0.012173991	0.449761338
MATR3	0.958311	0.382632	2.504524	0.012261629	0.451152774
ENSG00000258181	0.650882	0.25998	2.503583	0.012294267	0.451152774
ZBTB25	0.366285	0.146369	2.502478	0.012332719	0.451152774
ENSG00000278002	0.519955	0.207902	2.500962	0.01238565	0.451152774
SBF2.AS1	-0.652	0.2608	-2.500001	0.012419288	0.451152774
MLH1	-0.741818	0.296862	-2.498864	0.012459225	0.451152774
ECHS1	0.332092	0.132901	2.498796	0.0124616	0.451152774
IKZF1	0.32648	0.130777	2.496465	0.012543819	0.452831876
GALK1	-0.57284	0.229713	-2.493718	0.012641284	0.455050207
GYPC	0.23282	0.093439	2.491682	0.012713988	0.455574482
SLF2	-0.391315	0.157112	-2.490667	0.012750348	0.455574482
IER3.AS1	1.417004	0.569012	2.490286	0.012764018	0.455574482
BSCL2	1.074307	0.431607	2.489084	0.012807257	0.45561258
PTPA	-0.811897	0.326292	-2.488254	0.012837204	0.45561258
ENDOD1	0.649393	0.261832	2.480192	0.013131165	0.462544168
C1orf56	0.562825	0.226929	2.480181	0.013131558	0.462544168
MOB4	0.293409	0.118315	2.479889	0.013142331	0.462544168
MAP3K2	-0.324779	0.131303	-2.473503	0.013379568	0.468920828
RNF122	1.039932	0.420579	2.472617	0.013412798	0.468920828
FBXO10	0.776173	0.313982	2.472029	0.013434851	0.468920828
SLC35E3	0.291658	0.118571	2.459767	0.013902727	0.48050934
CNST	0.484397	0.196931	2.459727	0.013904257	0.48050934
REL	-0.253587	0.103101	-2.459588	0.01390967	0.48050934
UHRF2	0.426281	0.173331	2.459346	0.01391905	0.48050934
DPPA4	-1.351698	0.549836	-2.458368	0.013957018	0.48050934
TNFSF10	0.497905	0.202852	2.45452	0.014107269	0.483508011
ENSG00000260793	0.617817	0.251741	2.454179	0.014120653	0.483508011

PI4K2A	-1.771477	0.72385	-2.447297	0.014393213	0.491508772
ISCA1	0.288784	0.118099	2.445267	0.014474489	0.492701428
MICU1	0.457542	0.187174	2.44448	0.014506128	0.492701428
ABI3	0.422575	0.173356	2.437621	0.014784274	0.500802403
SERPINB1	0.265955	0.109199	2.435505	0.014871029	0.502394269
IRF1	0.289272	0.118826	2.434422	0.014915581	0.502555643
ENSG00000283013	0.340998	0.140291	2.430648	0.015071832	0.505600014
UBE2G1	0.360361	0.148331	2.429437	0.015122281	0.505600014
GABPB2	0.516299	0.212562	2.428933	0.015143332	0.505600014
ENSG00000226571	-1.528334	0.629361	-2.428391	0.015166	0.505600014
ZFP36	0.205473	0.084776	2.423734	0.015361837	0.510240671
PLP2	-0.478333	0.197618	-2.420491	0.015499571	0.510240671
HSH2D	0.310247	0.128189	2.420235	0.015510497	0.510240671
ZNF660	-1.269792	0.524732	-2.419886	0.01552536	0.510240671
FLNA	-0.49394	0.204173	-2.419219	0.015553886	0.510240671
LTBP3	-0.42503	0.175717	-2.418837	0.015570235	0.510240671
ENSG00000273247	0.261598	0.108189	2.417977	0.015607056	0.510240671
MFSD14B	-0.878911	0.363703	-2.416563	0.015667809	0.510240671
CCDC157	0.905777	0.374939	2.415797	0.015700808	0.510240671
ENSG00000234773	1.947449	0.806431	2.414899	0.015739593	0.510240671
CCDC71L	-1.091306	0.452113	-2.413788	0.015787634	0.510240671
ZNF587	0.494212	0.20475	2.413738	0.015789798	0.510240671
MDP1	0.53899	0.223621	2.41029	0.015939842	0.513775275
CYTH2	-0.399349	0.165786	-2.408816	0.01600436	0.5145422
RPS6KA2	-1.114612	0.46337	-2.405447	0.01615271	0.515196363
RSF1	0.24001	0.099798	2.404952	0.016174586	0.515196363
PARP12	-0.869379	0.361499	-2.404927	0.0161757	0.515196363
KDM3A	0.573482	0.238488	2.404653	0.016187808	0.515196363
C15orf39	0.784129	0.326348	2.402735	0.016272963	0.515313967
RUBCN	-0.334316	0.139143	-2.402675	0.016275638	0.515313967
RHBDF2	0.274379	0.114238	2.401817	0.016313857	0.515313967
CR2	0.87206	0.363309	2.400324	0.01638056	0.516044636
PHGDH	2.173084	0.90565	2.399473	0.016418674	0.516044636
UBE2O	0.430057	0.179473	2.396225	0.016564929	0.519349575
TM2D3	0.394095	0.164571	2.39468	0.016634858	0.520251066
SESN3	-0.297235	0.124201	-2.393177	0.016703167	0.521097579
AP4M1	0.454816	0.190154	2.391833	0.016764487	0.521722384
LINC02352	0.774256	0.324087	2.389036	0.016892666	0.524419736
SERPINF2	-1.645888	0.689512	-2.387034	0.016984907	0.52472111
NCF4	-0.354058	0.148354	-2.38658	0.017005885	0.52472111
CDCA7L	0.359273	0.150585	2.38585	0.017039689	0.52472111
LINC.PINT	0.34669	0.14544	2.38373	0.017138188	0.52472111
FMN1	1.519336	0.637388	2.383691	0.017140006	0.52472111
TBC1D2B	-0.911031	0.382274	-2.383189	0.017163392	0.52472111
CHP1	-0.295747	0.124171	-2.381769	0.017229695	0.52472111
GRINA	0.340936	0.143156	2.381564	0.017239303	0.52472111
CHMP1B	0.244881	0.102879	2.38028	0.017299479	0.52472111

PARP14	-0.24377	0.102442	-2.379599	0.017331484	0.52472111
TBCD	-0.933958	0.392583	-2.37901	0.017359195	0.52472111
PHKG1	0.474791	0.199984	2.374145	0.017589651	0.530418225
PEAK3	1.446709	0.610007	2.371625	0.017710048	0.532777272
LARGE1	1.21848	0.514004	2.370567	0.017760842	0.533036196
PHLDB1	1.587751	0.670127	2.369327	0.017820482	0.533558749
PRICKLE1	0.55251	0.233377	2.367456	0.01791085	0.534330983
EPHB6	-0.724934	0.306359	-2.366292	0.017967289	0.534330983
MICALL1	-1.027208	0.434325	-2.365068	0.018026766	0.534330983
KCNN1	1.256269	0.531438	2.363906	0.018083416	0.534330983
TMC8	0.336725	0.142446	2.36388	0.018084662	0.534330983
ITGB2	0.627966	0.265686	2.363566	0.018100013	0.534330983
TGFBR2	-0.259453	0.109853	-2.361819	0.018185507	0.535603454
LIPT2	0.725518	0.307354	2.360531	0.0182488	0.536217646
DAPP1	-0.257548	0.109195	-2.358594	0.018344323	0.537773833
SNHG21	-0.515024	0.218572	-2.356313	0.018457349	0.539764755
TTC37	-0.489557	0.207835	-2.355502	0.018497676	0.539764755
IER3	1.033816	0.439935	2.349928	0.018777041	0.546654183
TRAF7	-0.58744	0.250202	-2.347864	0.018881399	0.548428674
FTH1	-0.151138	0.0644	-2.346858	0.018932456	0.548650417
MAP9	-0.502275	0.214131	-2.345638	0.018994569	0.549190805
FYN	0.589602	0.251795	2.341592	0.019201676	0.552844767
ERP27	-1.782987	0.761622	-2.341039	0.019230149	0.552844767
SHF	0.835387	0.357094	2.339402	0.019314651	0.552844767
APOBEC3C	0.195566	0.083612	2.33897	0.019337009	0.552844767
ENSG00000235609	1.424145	0.608891	2.338917	0.019339722	0.552844767
MLH3	0.436529	0.186809	2.336764	0.019451452	0.553899544
FOXO3	0.628405	0.26895	2.336517	0.019464297	0.553899544
ANKRD44	0.232766	0.099678	2.33517	0.019534522	0.554648737
RASSF1	0.28674	0.123043	2.330409	0.019784562	0.560313006
SPN	1.109225	0.476372	2.328487	0.019886244	0.560313006
ZSWIM9	0.576346	0.247532	2.328366	0.019892682	0.560313006
BICDL1	1.328377	0.570859	2.32698	0.019966323	0.560313006
ITGB2.AS1	2.162025	0.929189	2.326787	0.019976582	0.560313006
INKA1	-0.416588	0.179074	-2.326346	0.020000092	0.560313006
LNPEP	0.255904	0.110181	2.322577	0.020201881	0.56471409
AGPAT2	-0.326441	0.14082	-2.31815	0.020441189	0.570128543
LINC00894	1.831305	0.790265	2.317329	0.020485822	0.570128543
ZNF665	0.584575	0.252475	2.315376	0.02059236	0.571834
MIPEP	0.796687	0.344389	2.313338	0.020704078	0.572436104
ENSG00000228835	0.595745	0.257574	2.31291	0.02072761	0.572436104
HNRNPL	0.326013	0.140987	2.312365	0.02075756	0.572436104
RIMS2	1.343742	0.581283	2.311681	0.020795265	0.572436104
ENSG00000251364	0.887372	0.384102	2.31025	0.020874337	0.573363572
ZCCHC14	-1.997077	0.865786	-2.306665	0.021073526	0.576349343
WDR82	0.390823	0.169433	2.306651	0.02107427	0.576349343
TIMP1	-0.798901	0.346511	-2.305557	0.021135404	0.576772843

ARHGAP15	0.231316	0.100462	2.30252	0.021305887	0.578001421
MDK	0.834485	0.36256	2.301649	0.021354953	0.578001421
ABRAXAS1	-0.305005	0.132576	-2.300611	0.021413636	0.578001421
PLAA	-0.694919	0.302134	-2.300034	0.021446321	0.578001421
RHOT1	-0.483248	0.21012	-2.299865	0.02145586	0.578001421
CD1C	0.866353	0.376823	2.299098	0.021499396	0.578001421
CHAF1A	1.148004	0.499446	2.298554	0.021530297	0.578001421
TOMM70	0.445433	0.193812	2.298271	0.021546393	0.578001421
TTC7A	-0.584736	0.254707	-2.295724	0.021691675	0.579830668
MSRB1	-0.452415	0.197146	-2.29482	0.021743468	0.579830668
CCNA2	1.203582	0.524723	2.293749	0.021804893	0.579830668
OLA1	-0.256756	0.111962	-2.293254	0.021833383	0.579830668
CCT6A	-0.25735	0.112232	-2.293019	0.021846905	0.579830668
CFL1	-0.198969	0.086818	-2.291787	0.021917958	0.579830668
CCNDBP1	0.190834	0.08328	2.291477	0.021935818	0.579830668
TP53TG1	-0.35641	0.155658	-2.289699	0.022038757	0.581335477
SLC25A46	0.387271	0.16925	2.288156	0.022128447	0.582485259
FSD1L	-0.759424	0.332187	-2.286133	0.022246487	0.584374973
RHOBTB2	0.73562	0.322398	2.281713	0.022506306	0.589973394
TOGARAM1	0.35358	0.155335	2.276248	0.022831157	0.597249821
HACD1	-2.012375	0.884652	-2.274765	0.022920054	0.598336526
PLEKHF1	0.614144	0.270265	2.272374	0.023063951	0.600049424
GNAZ	-1.379817	0.607288	-2.272097	0.023080651	0.600049424
FXYD1	-0.519569	0.228852	-2.270323	0.023187988	0.6016021
SYVN1	0.352891	0.155509	2.269269	0.023251955	0.602025521
FLI1	0.308315	0.135976	2.267423	0.023364401	0.602967907
PRDX2	-0.332808	0.146799	-2.267105	0.023383797	0.602967907
FAM32A	-0.25181	0.111152	-2.265454	0.023484812	0.604339302
ACAD10	0.547299	0.242238	2.259341	0.023862158	0.612801568
NME6	0.458722	0.203331	2.256034	0.024068532	0.61684768
IGLC7	-2.028622	0.899738	-2.25468	0.024153429	0.617770394
AVP	-2.71625	1.205579	-2.253066	0.024254962	0.618513991
DNAJC7	0.196142	0.087071	2.252663	0.024280407	0.618513991
LINC01679	1.730186	0.768524	2.251309	0.024365954	0.619326689
DVL3	-0.612676	0.272292	-2.250068	0.024444623	0.619326689
UBL7.AS1	-0.441842	0.196389	-2.249836	0.02445936	0.619326689
ENSG00000261187	1.27818	0.569061	2.246121	0.024696282	0.623052696
NOC3L	0.386443	0.17212	2.245195	0.02475563	0.623052696
RASSF6	2.579071	1.150004	2.242663	0.024918567	0.623052696
CCDC127	-0.607329	0.270847	-2.242334	0.024939792	0.623052696
ACACA	0.836169	0.372915	2.24225	0.024945209	0.623052696
KLHL5	-0.45924	0.204923	-2.241037	0.025023666	0.623052696
SUFU	0.943795	0.421142	2.241036	0.02502372	0.623052696
AMFR	0.649628	0.289956	2.240437	0.025062588	0.623052696
NFE2L3	1.500876	0.669943	2.240303	0.025071236	0.623052696
ZNF132	1.418344	0.633227	2.239866	0.02509963	0.623052696
GPR160	0.742987	0.332109	2.237178	0.025274709	0.623962637

ENSG00000260228	1.45073	0.648473	2.237147	0.025276764	0.623962637
CLPB	-1.607606	0.71885	-2.236358	0.025328331	0.623962637
FAM204A	0.238035	0.106452	2.236085	0.025346216	0.623962637
SLC25A19	0.438057	0.195953	2.23552	0.025383205	0.623962637
GGA2	0.24689	0.110536	2.233578	0.025510854	0.624713394
STK17A	0.276436	0.123765	2.233551	0.025512632	0.624713394
NADSYN1	-0.378716	0.169628	-2.232624	0.025573773	0.624838294
SC5D	-0.441357	0.197743	-2.231975	0.025616639	0.624838294
PIP5K1B	0.732446	0.328736	2.22807	0.025875875	0.628737691
LINC01781	1.074229	0.482135	2.228067	0.025876027	0.628737691
ZNF254	-0.760826	0.341653	-2.226894	0.025954374	0.629430941
TECPR2	-1.399737	0.628882	-2.225755	0.026030572	0.6300695
CLCF1	-0.513196	0.230864	-2.222936	0.026220103	0.633443602
IKBKB	0.358594	0.161415	2.221562	0.02631289	0.63447206
DERL1	0.28979	0.130529	2.220124	0.02641034	0.635231347
ABCD1	1.220562	0.549898	2.219615	0.02644493	0.635231347
TRIM66	0.725483	0.327087	2.218012	0.026554002	0.636641006
TBCB	-0.235903	0.106423	-2.216653	0.026646824	0.637203402
SLC37A1	-0.550708	0.248493	-2.216192	0.026678322	0.637203402
ENSG00000233912	1.217653	0.549904	2.214302	0.026808024	0.639093168
SLC25A23	-1.462637	0.660946	-2.212945	0.026901425	0.640112068
C15orf62	-0.76582	0.346279	-2.211568	0.026996526	0.64061435
WBP2NL	0.551434	0.249385	2.211172	0.027023937	0.64061435
C5orf56	0.406402	0.184053	2.208066	0.027239645	0.643511481
ADSL	-0.377702	0.171138	-2.207005	0.027313681	0.643511481
GPR146	-0.59958	0.271692	-2.206838	0.027325386	0.643511481
LSM11	0.872381	0.395492	2.205811	0.027397236	0.643511481
PPP1R3E	0.337426	0.152975	2.20576	0.027400805	0.643511481
UXT	-0.141656	0.064256	-2.20456	0.027484957	0.643886047
HEATR6	-0.75212	0.34124	-2.204081	0.027518676	0.643886047
PLD4	-1.053973	0.478647	-2.201987	0.027666253	0.644575006
HCG11	-0.638142	0.289849	-2.201639	0.027690794	0.644575006
ARHGEF12	-1.018518	0.462825	-2.200655	0.027760475	0.644575006
LINC02256	0.415055	0.18863	2.200367	0.027780901	0.644575006
DCP1A	0.302748	0.137632	2.199692	0.027828778	0.644575006
CLEC16A	-0.689991	0.313763	-2.199084	0.027871929	0.644575006
SLX4	-1.147494	0.522145	-2.197652	0.027973913	0.644575006
CD44	-0.264719	0.120502	-2.196811	0.02803393	0.644575006
FMC1	0.261218	0.118963	2.195793	0.028106749	0.644575006
MPC2	-0.278091	0.126709	-2.19472	0.028183682	0.644575006
DHRS3	2.518512	1.14779	2.194227	0.028219068	0.644575006
BCAP31	0.233643	0.106491	2.194015	0.028234355	0.644575006
TAF12	0.261137	0.119024	2.19398	0.028236829	0.644575006
RAB20	1.155632	0.526871	2.193389	0.028279355	0.644575006
ENSG00000281325	-0.768133	0.350279	-2.192917	0.028313346	0.644575006
HLA.DOB	0.436499	0.199126	2.192073	0.028374239	0.644799471
SHISAL2A	0.313166	0.142957	2.19064	0.02847786	0.645657205

RHOBTB3	1.373052	0.62714	2.189387	0.028568691	0.645657205
CNP	0.3339	0.152525	2.189151	0.028585834	0.645657205
GPR18	0.532041	0.243082	2.188731	0.028616386	0.645657205
SEC22A	0.56845	0.25994	2.186848	0.028753653	0.647597861
TRAPPC6A	0.238975	0.109368	2.185052	0.028885012	0.649319341
COA4	0.259064	0.118642	2.183575	0.028993492	0.649319341
MTX3	-1.015116	0.465076	-2.182688	0.029058829	0.649319341
FLOT1	0.3034	0.139024	2.182365	0.029082572	0.649319341
ZBTB41	-0.828926	0.379967	-2.181574	0.029141012	0.649319341
SNAPC3	0.336785	0.154381	2.181518	0.0291451	0.649319341
ENSG00000251034	1.012695	0.46449	2.180229	0.029240466	0.649319341
ENSG00000274292	1.246521	0.571741	2.180219	0.029241211	0.649319341
UTP20	0.594243	0.272766	2.17858	0.029362895	0.650877512
LMAN1	0.387071	0.177753	2.17758	0.029437292	0.651383866
TLR6	-0.573843	0.263732	-2.175853	0.02956626	0.651542107
ZSCAN18	0.250816	0.11528	2.175702	0.029577564	0.651542107
TTC30B	1.062807	0.488592	2.175244	0.029611822	0.651542107
TCEAL9	-1.706381	0.785055	-2.173582	0.029736532	0.651542107
TSPYL2	0.421014	0.193703	2.173499	0.029742744	0.651542107
L1CAM	-1.423521	0.654989	-2.173352	0.029753842	0.651542107
LUC7L	-0.340324	0.15668	-2.172093	0.02984866	0.652487579
SNAP47	0.289037	0.133183	2.170232	0.029989299	0.653883832
HLA.DOA	0.307293	0.141618	2.169879	0.030016037	0.653883832
GPATCH2	-0.567211	0.261523	-2.168874	0.03009226	0.654416024
MTERF4	0.278899	0.128697	2.167099	0.03022732	0.655915529
MBNL3	0.590262	0.272458	2.16643	0.030278332	0.655915529
PRDM4	0.534088	0.246587	2.165925	0.03031695	0.655915529
ENSG00000268027	0.43425	0.200802	2.162577	0.030573707	0.660339815
BBS12	-1.468608	0.679452	-2.161459	0.03065989	0.661071185
QDPR	-0.987717	0.457362	-2.159595	0.030804056	0.662082344
CTNND1	-0.490595	0.227224	-2.159079	0.03084405	0.662082344
RBM28	0.372015	0.172323	2.158822	0.030863989	0.662082344
PREPL	0.368373	0.17072	2.157755	0.030946887	0.662103571
SPIN3	1.879501	0.871224	2.15731	0.030981484	0.662103571
ZNF92	0.25114	0.116442	2.156788	0.031022186	0.662103571
SUMF2	0.225372	0.104617	2.154254	0.031220227	0.665206686
CD24	-0.239238	0.111133	-2.152718	0.031340876	0.666653139
SERINC2	1.44335	0.670836	2.15157	0.031431231	0.667126857
VPS50	0.553901	0.257497	2.151094	0.031468746	0.667126857
TRIM14	0.625171	0.291253	2.14649	0.031833924	0.672821883
BTG2	-0.198453	0.09246	-2.146365	0.031843885	0.672821883
NPM3	-0.490237	0.228479	-2.145655	0.031900511	0.67289309
SH3D19	0.82711	0.386655	2.139142	0.032424137	0.682798291
ZNF699	0.509133	0.2382	2.137419	0.032563964	0.682911044
TUT1	0.489672	0.22921	2.136349	0.032650972	0.682911044
FCHO1	0.48166	0.225565	2.135351	0.032732322	0.682911044
ACTA2	1.483712	0.694979	2.134901	0.03276911	0.682911044

AIMP1	-0.254614	0.119272	-2.13474	0.032782261	0.682911044
REST	0.256922	0.12036	2.134619	0.032792122	0.682911044
CHD1	0.25635	0.120102	2.134427	0.032807836	0.682911044
LAIR1	0.508902	0.238712	2.131866	0.033017838	0.686118431
SF3A1	0.357428	0.167763	2.130554	0.03312593	0.686118431
PRKACB	0.353013	0.165731	2.130041	0.033168201	0.686118431
ABHD15	0.341429	0.160302	2.129909	0.033179134	0.686118431
RIN3	-0.40897	0.192174	-2.128123	0.033326857	0.688047117
ZFP36L2	-0.383412	0.180348	-2.125949	0.03350752	0.689671883
GSN	-0.699171	0.328888	-2.125862	0.033514724	0.689671883
KDELR1	-0.279378	0.131502	-2.124513	0.03362723	0.690861865
S100A11	-0.388031	0.183057	-2.119728	0.03402901	0.696649329
SP100	0.180055	0.084959	2.119305	0.034064734	0.696649329
TMEM256	-0.176429	0.083253	-2.119191	0.03407434	0.696649329
SH3GLB1	-0.236761	0.111856	-2.116659	0.034288828	0.698608424
ERAL1	-0.518651	0.245183	-2.11536	0.034399239	0.698608424
ZFYVE19	0.365614	0.172841	2.115325	0.034402255	0.698608424
YPEL3	0.263204	0.124456	2.114837	0.03444384	0.698608424
SLC38A1	-0.235704	0.111474	-2.114425	0.034478998	0.698608424
PLCE1	0.431497	0.204099	2.114156	0.034501912	0.698608424
SFXN1	-0.433175	0.204962	-2.113444	0.034562825	0.698722073
IFT172	0.701767	0.332651	2.109619	0.034891211	0.70423395
NUP88	0.309631	0.146876	2.108115	0.035021041	0.704438711
NME7	0.43872	0.208128	2.107931	0.035036942	0.704438711
CERS4	-0.300979	0.142809	-2.107565	0.035068615	0.704438711
ENSG00000250222	0.962686	0.457249	2.105387	0.035257666	0.707112086
KLHDC3	0.237298	0.112776	2.104166	0.035363952	0.7080658
ENSG00000282988	0.579271	0.275452	2.102988	0.035466847	0.7080658
ENSG00000124593	-1.082636	0.514827	-2.102913	0.03547334	0.7080658
CACTIN	0.674938	0.32116	2.101561	0.035591755	0.708892787
ZNF219	-1.297419	0.617478	-2.101159	0.035626982	0.708892787
ATP11A	-0.706412	0.336717	-2.097943	0.035910209	0.711931657
SH3BP1	-0.540626	0.257863	-2.096564	0.036032224	0.711931657
SOBP	1.498224	0.714731	2.096207	0.036063837	0.711931657
ENSG00000130749	0.396124	0.189012	2.095757	0.036103724	0.711931657
ZNF341	0.792604	0.378299	2.095181	0.036154891	0.711931657
CNNM3	0.753244	0.359681	2.094203	0.03624186	0.711931657
ATP6V0A1	0.43907	0.209665	2.09415	0.036246642	0.711931657
ADAM10	-0.351164	0.167703	-2.093956	0.03626391	0.711931657
TALDO1	-0.180999	0.086449	-2.093699	0.036286821	0.711931657
NEK5	-0.694951	0.332047	-2.092931	0.036355354	0.712170391
CXCR4	0.102829	0.049158	2.091806	0.03645585	0.713033542
SRSF8	0.175442	0.083906	2.090932	0.036534182	0.713461187
PACRGL	-1.322773	0.633279	-2.088769	0.036728525	0.714167985
GALT	0.285382	0.136685	2.08789	0.036807729	0.714167985
NT5E	0.546445	0.261723	2.087879	0.036808733	0.714167985
DDB2	-0.317375	0.152018	-2.087738	0.036821444	0.714167985

RAPGEF5	-1.279098	0.612774	-2.087389	0.03685299	0.714167985
CDC42SE2	0.233307	0.111837	2.086141	0.036965815	0.715257377
ENSG00000133961	0.245553	0.117744	2.085477	0.03702599	0.715326271
KDM3B	0.356287	0.17093	2.084409	0.037123	0.716105495
UQCR11	0.169444	0.081347	2.082966	0.037254287	0.717542551
WARS2	-0.932644	0.448177	-2.080971	0.037436564	0.719955842
NOC2L	0.251269	0.120836	2.079421	0.037578664	0.720615042
HACD2	-0.438897	0.211092	-2.079173	0.037601417	0.720615042
ATP2A1.AS1	-1.63471	0.786397	-2.078733	0.037641941	0.720615042
SPOP	0.243103	0.117016	2.077523	0.03775336	0.721654625
ENSG00000277825	0.992944	0.478287	2.076041	0.037890155	0.723175387
SLC12A6	0.307395	0.148144	2.074983	0.037988131	0.723951784
HIC1	2.343222	1.129301	2.074931	0.03799297	NA
EXD3	-0.564914	0.272402	-2.073821	0.038095947	0.724913081
CD37	-0.087913	0.042425	-2.072222	0.038244759	0.726650414
PCF11.AS1	0.815818	0.393884	2.071215	0.038338689	0.727341346
ENSG00000233038	3.800671	1.836036	2.070042	0.038448405	NA
ENSG00000272994	0.399917	0.193314	2.068745	0.038569983	0.728381965
SMPD1	0.478095	0.231145	2.06838	0.038604349	0.728381965
ZFYVE16	-0.433854	0.209803	-2.067908	0.038648682	0.728381965
INPP5B	0.421953	0.204068	2.067711	0.038667203	0.728381965
CDK13	0.345609	0.167176	2.067343	0.038701825	0.728381965
ICOSLG	-0.289957	0.140334	-2.066195	0.038810116	0.728381965
SYTL1	-0.296991	0.143742	-2.066139	0.038815391	0.728381965
ATP5MG	-0.139275	0.067422	-2.065722	0.038854725	0.728381965
PPP1R3F	1.352746	0.655104	2.064934	0.038929235	0.728697608
CYB561	1.160127	0.562254	2.063349	0.0390795	0.729784367
RCC1L	0.551677	0.267401	2.063103	0.039102811	0.729784367
KCNH4	1.781503	0.864952	2.059656	0.039431425	0.73420698
TLR10	-0.456937	0.221879	-2.059399	0.039455998	0.73420698
MOB1B	-1.025403	0.498178	-2.058306	0.039560727	0.734301946
LPCAT4	-0.445491	0.216504	-2.057656	0.03962316	0.734301946
ICA1L	0.356813	0.173452	2.057128	0.039673898	0.734301946
HERPUD1	-0.196352	0.095459	-2.056924	0.039693568	0.734301946
CPLANE2	1.07898	0.525378	2.053721	0.04000267	0.738938211
ARHGDIB	0.136773	0.066649	2.052142	0.040155833	0.739000925
MYO1C	-0.555726	0.270812	-2.052075	0.040162393	0.739000925
EMC7	0.255531	0.12459	2.050983	0.040268632	0.739000925
ARMC2	1.271583	0.620013	2.050898	0.04027685	0.739000925
TMEM154	0.329512	0.160685	2.050676	0.040298507	0.739000925
RASA4	0.601647	0.29358	2.049346	0.040428269	0.740306056
ENSG00000262429	0.937988	0.4586	2.04533	0.040822294	0.743590377
FCRLB	-1.487255	0.72729	-2.044927	0.040862071	0.743590377
UBLCP1	-0.428732	0.209658	-2.04491	0.040863764	0.743590377
SERPINF1	-0.839981	0.411	-2.04375	0.040978289	0.743590377
ENSG00000273188	0.831819	0.407032	2.043621	0.040991024	0.743590377
EHD1	0.290315	0.14211	2.042895	0.041062799	0.743590377

VOPP1	-0.258127	0.126357	-2.042846	0.041067733	0.743590377
FGD2	0.242216	0.118574	2.042737	0.041078439	0.743590377
DNAH1	0.99446	0.487061	2.041756	0.041175748	0.744285511
DDX24	0.195025	0.095587	2.040278	0.04132261	0.745873118
NAA30	-0.50431	0.247411	-2.038346	0.041515383	0.748283678
FAM81A	-1.704392	0.836882	-2.036599	0.041690239	0.750364906
TMEM65	0.636569	0.31275	2.035396	0.041811031	0.751468529
SUGP2	-0.332255	0.163319	-2.03439	0.041912259	0.752217892
OGFOD3	-0.529047	0.260384	-2.031797	0.042174184	0.755845121
MYBL2	-1.465411	0.721794	-2.030233	0.04233281	0.756548845
ABLIM2	-0.645798	0.318091	-2.03023	0.042333204	0.756548845
ENSG00000257275	-0.288028	0.141951	-2.029061	0.042452064	0.757005681
CD27	-1.119643	0.552008	-2.028311	0.042528478	0.757005681
RESF1	-0.243124	0.11993	-2.027218	0.042640159	0.757005681
HNRNPH2	0.267236	0.131891	2.026185	0.042745826	0.757005681
TGIF2	-0.355489	0.175477	-2.025842	0.042781016	0.757005681
ATP2B1	-0.176809	0.087277	-2.025838	0.042781353	0.757005681
SLC9A9	0.474443	0.234225	2.025588	0.042806983	0.757005681
HMGN1	-0.124636	0.06154	-2.025285	0.042838074	0.757005681
RAB4A	-0.289522	0.143073	-2.023605	0.043010769	0.758495733
PTP4A3	1.258874	0.622664	2.021755	0.043201708	0.758495733
TMEM30A	-0.38882	0.192363	-2.02128	0.043250811	0.758495733
EAF2	0.301543	0.149207	2.020969	0.043282976	0.758495733
CIZ1	0.333585	0.165114	2.02033	0.043349151	0.758495733
SYNGAP1	0.674213	0.333836	2.019591	0.043425816	0.758495733
EIF4ENIF1	0.55961	0.277104	2.019493	0.043435976	0.758495733
ISCU	-0.164427	0.081456	-2.01861	0.043527727	0.758495733
CCR7	0.205454	0.101879	2.016647	0.043732366	0.758495733
ENSG00000179743	0.378122	0.187524	2.016387	0.043759479	0.758495733
DNAL1	0.540311	0.26798	2.016231	0.043775798	0.758495733
QSOX2	-0.481164	0.238749	-2.015354	0.043867569	0.758495733
MTRR	-0.601987	0.298807	-2.014634	0.04394301	0.758495733
MAP3K7CL	0.912051	0.453039	2.013185	0.044095152	0.758495733
MR1	0.388923	0.193249	2.01255	0.044161986	0.758495733
ENSG00000273702	0.617712	0.307071	2.011627	0.04425924	0.758495733
FASTK	0.233334	0.116001	2.011473	0.044275569	0.758495733
GOLGA2	-0.430175	0.21392	-2.010911	0.044334909	0.758495733
MCM5	0.345623	0.171879	2.01085	0.044341311	0.758495733
KNL1	0.644069	0.320452	2.009877	0.04444423	0.758495733
ENSG00000276728	0.447097	0.222451	2.009866	0.044445357	0.758495733
PATJ	0.566934	0.282083	2.009815	0.044450775	0.758495733
WWP2	-0.519236	0.25836	-2.009737	0.044459074	0.758495733
TYW3	-0.382347	0.190284	-2.009345	0.044500576	0.758495733
CBY1	-0.756685	0.376628	-2.009105	0.044525994	0.758495733
CLN8	-0.348765	0.173658	-2.008347	0.044606436	0.758495733
CWC27	0.307716	0.153234	2.008153	0.044627045	0.758495733
RBIS	-0.191284	0.095255	-2.008121	0.044630416	0.758495733

FLII	-0.354527	0.176574	-2.007812	0.044663302	0.758495733
SKIL	0.265892	0.132516	2.006484	0.044804596	0.759071263
GID8	-0.248046	0.12366	-2.005877	0.044869426	0.759071263
MAGEF1	0.329518	0.164282	2.005802	0.044877423	0.759071263
SNN	0.403436	0.201253	2.00462	0.045003695	0.760189425
TXLNB	-1.284842	0.642049	-2.001158	0.045375354	0.762899353
CDKN1A	0.362049	0.180946	2.000872	0.045406215	0.762899353
LINC01800	-1.431797	0.71568	-2.000612	0.045434225	0.762899353
RAB5IF	-0.225589	0.11279	-2.000088	0.045490769	0.762899353
CPTP	-0.469557	0.23484	-1.99948	0.045556467	0.762899353
ATM	0.218922	0.109499	1.99931	0.045574812	0.762899353
MARK4	-0.843457	0.421897	-1.999199	0.045586784	0.762899353
MCM9	0.482461	0.241467	1.998042	0.045712136	0.763794243
TCTN1	-0.598982	0.299852	-1.99759	0.045761159	0.763794243
RABAC1	0.166206	0.083371	1.993556	0.046200616	0.770111849
CLIC4	-0.420929	0.211216	-1.992883	0.046274287	0.770323612
KCTD20	-0.334211	0.167882	-1.990752	0.046508139	0.773197807
HSD17B14	-1.507572	0.757755	-1.989523	0.04664348	0.774428877
MRNIP	0.283273	0.142448	1.988607	0.046744586	0.775089042
CCR9	2.068483	1.040811	1.987377	0.04688067	0.776326697
BCAS4	0.327796	0.165015	1.986457	0.04698256	0.776995616
ARHGEF9	0.420172	0.21162	1.985501	0.047088787	0.777734403
LEPROTL1	0.345382	0.174029	1.984621	0.047186667	0.778333594
NQO1	1.17675	0.593213	1.983688	0.04729064	0.779031607
SGMS1	0.463294	0.233819	1.981423	0.047543863	0.781354689
HIP1R	0.276783	0.139727	1.98088	0.047604729	0.781354689
TERF2IP	0.238609	0.120494	1.980254	0.047674958	0.781354689
S100A6	-0.444495	0.22453	-1.979671	0.04774049	0.781354689
L3MBTL1	-1.064023	0.537475	-1.979668	0.047740864	0.781354689
PACS1	0.277686	0.140382	1.978078	0.047919937	0.783200105
ENSG00000282393	0.921454	0.465953	1.977567	0.047977593	0.783200105
PLSCR1	-0.380906	0.192667	-1.977017	0.048039728	0.783202533
ARHGEF3	0.457083	0.231327	1.975916	0.048164266	0.784221006
TACC3	0.379616	0.192209	1.975023	0.048265574	0.784367271
ENTPD1	-0.395833	0.200448	-1.974742	0.048297407	0.784367271
MREG	-1.043232	0.52844	-1.974172	0.048362222	0.784411642
GRAP	0.406186	0.205906	1.972674	0.048532713	0.785201836
LINC01857	0.408394	0.207028	1.972652	0.04853523	0.785201836
NAPA	-0.375885	0.19064	-1.971704	0.048643368	0.785588294
GABARAPL1	0.614577	0.311754	1.971353	0.048683469	0.785588294
ARL16	-0.377823	0.191777	-1.970118	0.0488248	0.785909779
GARS	0.35652	0.180966	1.970092	0.048827794	0.785909779
SLC51A	1.010721	0.513342	1.968905	0.048964038	0.787100028
MAP4	-0.258308	0.131281	-1.967593	0.04911492	0.788522256
ZFAND2B	-0.259967	0.132169	-1.966935	0.049190696	0.788736604
ENSG00000265206	-0.210333	0.107078	-1.964296	0.049495804	0.791826839

Cluster 2	log2FC	lfcSE	stat	pvalue	padj
VPREB3	3.034294	0.09097	33.35473	< 2.22e-16	< 2.22e-16
CD38	3.051433	0.245412	12.433914	< 2.22e-16	< 2.22e-16
PLAAT4	2.141232	0.191363	11.189352	< 2.22e-16	< 2.22e-16
RARA.AS1	2.480806	0.3052	8.128454	4.35E-16	9.66E-13
AHI1	1.631033	0.201404	8.098314	5.57E-16	9.90E-13
DUS2	1.54804	0.1959	7.902192	2.74E-15	4.06E-12
SLC12A4	2.387136	0.30835	7.741645	9.81E-15	1.25E-11
RRBP1	1.550321	0.20328	7.626543	2.41E-14	2.68E-11
FCRL1	1.141723	0.153163	7.454285	9.04E-14	8.92E-11
P2RX5.TAX1BP3	1.854819	0.255359	7.263568	3.77E-13	3.35E-10
LCAT	2.825853	0.39723	7.113893	1.13E-12	NA
RAB37	1.279916	0.185126	6.913766	4.72E-12	3.81E-09
TRABD	0.633739	0.092243	6.870337	6.41E-12	4.74E-09
CD1D	2.367338	0.346709	6.828033	8.61E-12	5.88E-09
CD72	0.992145	0.146633	6.766193	1.32E-11	8.39E-09
P2RX5	1.032489	0.153563	6.723568	1.77E-11	1.05E-08
SPRY1	1.113203	0.170928	6.512705	7.38E-11	4.10E-08
FAM3C	0.701706	0.109645	6.399772	1.56E-10	8.13E-08
SEPTIN9	0.600936	0.094237	6.376852	1.81E-10	8.92E-08
ICAM2	0.911121	0.14622	6.231178	4.63E-10	2.17E-07
DENND6B	1.960659	0.315262	6.219136	5.00E-10	2.22E-07
AGPAT5	0.788069	0.127548	6.178608	6.47E-10	2.74E-07
MRPS6	0.867285	0.145842	5.946723	2.74E-09	1.10E-06
AGO1	1.077876	0.182502	5.906115	3.50E-09	1.35E-06
DPEP2	0.984316	0.167034	5.892897	3.79E-09	1.41E-06
PNOC	1.384339	0.242749	5.702745	1.18E-08	4.19E-06
SIGIRR	0.770474	0.137781	5.592008	2.24E-08	7.67E-06
RMDN2	1.974604	0.357137	5.528988	3.22E-08	1.06E-05
DBNL	0.547936	0.100017	5.478442	4.29E-08	1.36E-05
LINC.PINT	0.581551	0.109126	5.329166	9.87E-08	3.02E-05
MPEG1	1.15014	0.21742	5.289932	1.22E-07	3.62E-05
PLAAT3	2.976095	0.575361	5.172568	2.31E-07	NA
CD79B	0.31698	0.061772	5.131428	2.88E-07	8.24E-05
MS4A1	-0.511386	0.100792	-5.073662	3.90E-07	0.000108362
ANAPC16	0.329325	0.065126	5.056728	4.27E-07	0.000114848
GRN	0.717523	0.146776	4.888571	1.02E-06	0.000265457
ATXN10	0.636677	0.137254	4.638691	3.51E-06	0.000890181
CD180	0.806356	0.17532	4.599329	4.24E-06	0.001046215
C12orf65	0.638003	0.141541	4.507559	6.56E-06	0.001574925
H3F3A	0.377295	0.085155	4.430708	9.39E-06	0.002196346
CLEC17A	0.901524	0.203849	4.422517	9.76E-06	0.002222817
FAM117A	0.690666	0.157203	4.393468	1.12E-05	0.002478231
NCF1	0.315745	0.072588	4.349795	1.36E-05	0.002953296
SLC5A3	0.760359	0.175875	4.32329	1.54E-05	0.003252263
SMAD3	0.725938	0.168691	4.303366	1.68E-05	0.003424573
GBP4	0.648942	0.150861	4.301597	1.70E-05	0.003424573

MRPS36	0.4364	0.101629	4.294071	1.75E-05	0.003464082
CD83	-0.307799	0.073406	-4.193126	2.75E-05	0.005314908
RABGAP1L	0.662136	0.158571	4.175634	2.97E-05	0.005618159
PTK2B	0.61848	0.149258	4.143692	3.42E-05	0.00620956
PXK	0.524563	0.126607	4.143253	3.42E-05	0.00620956
JAK1	0.345714	0.083953	4.117949	3.82E-05	0.006793517
FOXO3	1.06231	0.258502	4.10949	3.97E-05	0.006909032
C16orf54	0.727527	0.177723	4.09361	4.25E-05	0.007257618
CD22	0.692782	0.170625	4.060254	4.90E-05	0.008164017
FCER2	1.426549	0.351588	4.057445	4.96E-05	0.008164017
SPOCK2	0.589008	0.145329	4.052919	5.06E-05	0.008172292
PLEKHO1	0.548176	0.135903	4.033581	5.49E-05	0.008716723
CXCR4	0.167131	0.041666	4.011164	6.04E-05	0.009419179
CD79A	0.467968	0.116992	3.999995	6.33E-05	0.009594201
RINL	0.57086	0.142763	3.99866	6.37E-05	0.009594201
ENSG00000225489	1.243173	0.313387	3.966893	7.28E-05	0.010784007
RUBCN	-0.620071	0.157412	-3.939163	8.18E-05	0.01191106
IRAK2	0.943004	0.239851	3.931624	8.44E-05	0.012092674
CHST15	0.796998	0.203098	3.924198	8.70E-05	0.012273864
ZNF595	1.278442	0.32695	3.910205	9.22E-05	0.012645263
WWP2	0.901898	0.230696	3.909471	9.25E-05	0.012645263
CPNE5	1.003339	0.256921	3.905251	9.41E-05	0.012673004
ATP6V0A1	0.824013	0.213807	3.854012	0.0001162	0.015410946
HSH2D	0.352237	0.09204	3.827006	0.00012971	0.016950204
LINC02397	0.377386	0.09976	3.782944	0.00015499	0.019831511
NUP210	0.456328	0.120691	3.780961	0.00015622	0.019831511
NAGPA.AS1	2.091888	0.554679	3.77135	0.00016237	NA
DPEP3	2.176361	0.578113	3.764595	0.00016682	NA
C8orf89	2.214185	0.58949	3.756103	0.00017258	NA
SLC25A45	0.892522	0.23864	3.740032	0.000184	0.0230281
KLF8	0.641582	0.171802	3.734421	0.00018815	0.023220539
SERPINB6	0.770903	0.206916	3.725674	0.00019479	0.023711477
NFATC3	0.526412	0.141463	3.721197	0.00019828	0.023809719
GPR183	-0.573418	0.154236	-3.717788	0.00020098	0.023811467
SLC2A5	1.11038	0.298976	3.713938	0.00020406	0.02385875
DCK	0.300162	0.08092	3.70936	0.00020778	0.023892997
VNN2	0.687887	0.185564	3.707	0.00020973	0.023892997
LINC00513	0.64305	0.173747	3.701079	0.00021469	0.024147948
TNFRSF13B	-1.719854	0.466864	-3.683844	0.00022974	0.025449635
LY86	-0.368345	0.100057	-3.681369	0.00023199	0.025449635
TNFRSF18	-2.210402	0.603909	-3.660155	0.00025206	0.027314967
TGFBR2	-0.479643	0.131921	-3.635837	0.00027708	0.029664207
DDIT4	1.044095	0.288007	3.625242	0.00028869	0.030396426
IRF4	0.830838	0.229298	3.623396	0.00029076	0.030396426
ST3GAL1	0.722354	0.199544	3.620025	0.00029457	0.030437048
MAPKAPK2	0.739362	0.20576	3.593326	0.00032648	0.033346417
ENSG00000263394	0.863715	0.24097	3.584331	0.00033794	0.034124546

ICA1	3.909597	1.103795	3.541959	0.00039717	NA
ZFP36L1	-0.331665	0.09443	-3.512286	0.00044427	0.044357121
ATP6V1E1	0.453004	0.129498	3.498151	0.0004685	0.0462562
BTBD7	0.564849	0.162038	3.485898	0.00049049	0.047895408
ZNF563	0.885071	0.255207	3.468046	0.00052426	0.050636435
POLM	0.453001	0.131241	3.451675	0.00055712	0.053231787
SLC43A2	0.455163	0.13226	3.441422	0.00057867	0.054702305
CD82	-0.492381	0.143432	-3.432846	0.00059728	0.05586772
CDKN1A	0.583798	0.170294	3.428168	0.00060767	0.056247438
NAPSA	1.538831	0.449544	3.423095	0.00061913	NA
FCMR	0.435349	0.12748	3.41505	0.0006377	0.056985812
TMEM154	0.483157	0.141506	3.414389	0.00063925	0.056985812
SNX18	0.792416	0.232295	3.411253	0.00064665	0.056985812
IRF2BPL	0.982681	0.288097	3.410937	0.0006474	0.056985812
XYLT1	0.788127	0.231068	3.410806	0.00064771	0.056985812
NMT2	-0.535934	0.157922	-3.393663	0.00068965	0.060080327
ADD1	0.492168	0.145328	3.386614	0.00070761	0.061046617
SMARCB1	0.335671	0.099216	3.383247	0.00071634	0.061205906
VPREB1	2.942504	0.873941	3.366938	0.00076008	0.064324317
LINC02132	1.439941	0.427899	3.365144	0.00076504	NA
S100A4	-0.955883	0.284539	-3.359414	0.00078108	0.065477983
CENPQ	0.829763	0.247661	3.350393	0.00080697	0.067016196
ZNF594	0.89897	0.269653	3.333805	0.00085667	0.070484568
PCBP1	-0.283969	0.085288	-3.329518	0.00086996	0.070821831
SNRPD3	0.332836	0.10003	3.327368	0.00087671	0.070821831
CSTF1	0.635894	0.192474	3.303795	0.00095386	0.076360032
CABLES1	-1.757833	0.534508	-3.288696	0.00100653	0.07985718
AKAP12	2.524871	0.770353	3.27755	0.00104712	NA
SEL1L3	0.569252	0.174335	3.265266	0.00109361	0.085998498
CIB1	0.31545	0.096709	3.261859	0.00110684	0.086275488
PIGX	-1.144981	0.353234	-3.241423	0.00118935	0.091900174
CALHM6	0.40424	0.124858	3.237601	0.00120539	0.092337292
CHP1	-0.477359	0.147973	-3.225978	0.00125543	0.095348431
P2RY8	0.493458	0.153215	3.22069	0.00127883	0.096302025
CCNG1	-0.409235	0.127266	-3.215594	0.00130175	0.096562933
GMFG	0.220015	0.068432	3.215093	0.00130402	0.096562933
PPP1R15B	0.713021	0.222123	3.21003	0.00132721	0.097467707
FCHO1	0.698151	0.217973	3.202923	0.0013604	0.098762607
NKAPL	1.728687	0.539817	3.202356	0.00136308	NA
SNAP23	0.317389	0.099137	3.201515	0.00136707	0.098762607
MTG1	0.65184	0.203817	3.198161	0.00138307	0.099112725
ETFB	0.492788	0.154321	3.193273	0.0014067	0.099999538
UROS	0.509674	0.159764	3.190176	0.00142186	0.100275034
ZNF3	0.558199	0.175475	3.181083	0.00146726	0.102661612
LINC02649	1.32698	0.417498	3.178408	0.00148086	NA
ENSG00000214797	1.716383	0.540774	3.173937	0.00150386	NA
ENSG00000231760	1.899841	0.598717	3.173187	0.00150775	NA

GPX1	0.321333	0.101854	3.154853	0.00160579	0.111010085
ENSG00000262292	0.869054	0.275557	3.153807	0.00161156	0.111010085
SCN4A	2.275882	0.723333	3.146383	0.00165303	NA
EMC6	-0.455152	0.145744	-3.122961	0.00179042	0.122381819
CD47	-0.285346	0.091687	-3.11218	0.00185711	0.125971707
PSMB10	0.362142	0.116465	3.109448	0.00187437	0.126031766
PELI1	-1.065683	0.342932	-3.107564	0.00188636	0.126031766
ENSG00000277602	1.495227	0.482609	3.098213	0.00194692	NA
DNAJC7	0.325543	0.105174	3.095292	0.00196619	0.130384826
MFSD10	-0.418778	0.135428	-3.092248	0.00198647	0.13065308
MARCKS	-0.784003	0.253699	-3.090285	0.00199964	0.13065308
ATF6	-0.74151	0.240761	-3.079863	0.00207096	0.134325088
AP3B1	0.58747	0.191602	3.066091	0.00216877	0.138756114
SIDT1	-0.697752	0.227588	-3.065852	0.0021705	0.138756114
ENSG00000271204	0.54742	0.17905	3.057353	0.00223301	0.141732498
SFXN2	1.435299	0.470387	3.051315	0.00227842	NA
SNAPC2	-0.78357	0.25796	-3.037565	0.00238498	0.150304588
GCHFR	-0.630412	0.209066	-3.015379	0.00256658	0.159947013
ENSG00000273319	0.451729	0.149852	3.014506	0.00257398	0.159947013
CENPM	0.568498	0.188962	3.008537	0.00262509	0.161990065
SPINT2	-0.308891	0.10275	-3.006234	0.00264505	0.162096034
ARHGAP15	0.305227	0.102058	2.99071	0.0027833	0.169025782
PAOX	0.671463	0.224622	2.9893	0.00279617	0.169025782
MAPK1	-0.470702	0.157934	-2.980377	0.00287893	0.172852755
FAM86C1	1.354672	0.455096	2.976671	0.00291397	NA
TFG	0.467798	0.158542	2.950626	0.00317131	0.187535564
COA1	0.400695	0.135852	2.949499	0.00318289	0.187535564
INPP5B	0.694187	0.235388	2.949121	0.0031868	0.187535564
CXCR5	0.440628	0.149564	2.94608	0.00321829	0.188143029
SH2B2	1.685222	0.572054	2.945912	0.00322004	NA
ENSG00000254777	2.094552	0.715085	2.929094	0.00339951	NA
EWSR1	0.386306	0.131894	2.928921	0.00340141	0.197548355
ZNF44	0.489026	0.167242	2.924056	0.00345502	0.199359236
MCL1	0.35031	0.120005	2.919137	0.00351002	0.200392536
RESF1	-0.385416	0.132063	-2.918426	0.00351803	0.200392536
KCNN1	1.972787	0.678895	2.905879	0.00366223	NA
GPER1	-2.128222	0.736459	-2.889802	0.00385484	NA
FDFT1	0.339286	0.117431	2.889244	0.00386169	0.21856695
ERAP1	0.620592	0.215271	2.88284	0.00394108	0.22026481
LHFPL2	1.223049	0.424254	2.882825	0.00394127	0.22026481
IFNAR2	0.43595	0.151788	2.872093	0.00407763	0.226461374
MAP3K2	-0.51615	0.179882	-2.869382	0.00411275	0.226847466
ENSG00000273783	1.875864	0.653948	2.868521	0.00412396	NA
RNPEPL1	0.498494	0.173835	2.867626	0.00413564	0.226847466
ARHGEF3	0.639045	0.223016	2.865463	0.004164	0.227001588
HIVEP3	1.353874	0.472499	2.865347	0.00416553	NA
TSPAN18	2.510452	0.87804	2.859154	0.00424772	NA

ANKRD36C	-0.718136	0.251199	-2.858828	0.00425209	0.230390513
FADS3	-0.369165	0.129286	-2.855411	0.00429811	0.230759865
NRM	0.516376	0.180901	2.854472	0.00431084	0.230759865
ENSG00000253535	0.895944	0.315156	2.84286	0.00447108	0.236567569
MYC	0.395322	0.139216	2.839628	0.00451662	0.236567569
ATP5F1B	0.290086	0.10222	2.837852	0.00454182	0.236567569
FLOT1	0.383436	0.13521	2.835859	0.00457026	0.236567569
ZNF581	-0.32674	0.115232	-2.835499	0.00457541	0.236567569
SLC44A2	0.315237	0.111185	2.835244	0.00457907	0.236567569
FGF9	2.99957	1.05902	2.832403	0.00461996	NA
MZB1	-0.559387	0.197594	-2.830985	0.00464049	0.238354846
APOL3	0.495284	0.175483	2.822402	0.00476655	0.240899862
EFCAB5	1.247835	0.442375	2.820763	0.00479096	0.240899862
SLC27A5	-0.988445	0.350448	-2.820516	0.00479465	0.240899862
HOXB3	2.793942	0.991072	2.819112	0.00481567	NA
ZNF780A	0.641343	0.227503	2.819056	0.00481651	0.240899862
SELL	0.58601	0.20799	2.817496	0.00483997	0.240899862
MYO9B	0.409979	0.145555	2.816653	0.0048527	0.240899862
EIF2AK4	-0.783045	0.278371	-2.812958	0.0049088	0.241298667
LINC02062	1.597238	0.567814	2.812957	0.00490882	NA
C7orf50	-0.320533	0.114035	-2.810838	0.00494127	0.241298667
POP7	-0.534824	0.190298	-2.810452	0.0049472	0.241298667
PSMC6	0.320808	0.114207	2.809014	0.00496935	0.241298667
STMN3	0.435974	0.155477	2.804099	0.00504574	0.243676164
TCIRG1	-0.613782	0.219326	-2.798488	0.00513425	0.246610727
DMD	-0.635901	0.227852	-2.790848	0.00525702	0.251149959
ENSG00000283013	0.401087	0.143879	2.787674	0.00530879	0.251525614
ENSG00000251364	1.165921	0.418594	2.785323	0.00534744	NA
KDM2B	0.450505	0.161748	2.78523	0.00534898	0.251525614
CAPRIN2	-0.995901	0.357572	-2.78518	0.0053498	0.251525614
USF3	-0.440345	0.158205	-2.783378	0.00537961	0.251595712
UBE2E1	0.379504	0.136444	2.781383	0.00541279	0.251822086
MCM7	0.465233	0.16746	2.77817	0.0054666	0.251874484
ENSG00000227486	0.413467	0.14884	2.777932	0.0054706	0.251874484
CCDC154	1.246366	0.449052	2.775546	0.00551092	NA
TMEM38B	0.566989	0.204503	2.772516	0.00556248	0.254784475
C1orf159	-1.512527	0.545962	-2.770386	0.00559899	NA
ENSG00000230709	-1.846118	0.66723	-2.766841	0.00566024	NA
ENSG00000259802	1.930093	0.701928	2.7497	0.00596498	NA
FCGRT	0.364299	0.132491	2.74961	0.00596663	0.271894637
TENT5C	-0.415571	0.151488	-2.743262	0.00608321	0.275792631
MAP3K1	0.423318	0.154406	2.741587	0.00611433	0.275796412
UBB	0.214956	0.078454	2.739879	0.00614619	0.275833405
GPR146	-0.804629	0.294723	-2.730122	0.00633109	0.281446068
LINC00926	0.220367	0.08073	2.7297	0.0063392	0.281446068
MOB3C	0.776791	0.284717	2.728295	0.00636627	0.281446068
HTR3A	1.272951	0.467018	2.725697	0.00641659	NA

TBC1D22B	0.830079	0.304612	2.725032	0.00642952	0.282835297
CYBA	0.230959	0.084831	2.722578	0.00647747	0.283541055
TLE3	-0.411522	0.15138	-2.718459	0.00655868	0.285688442
PAXIP1.AS2	0.676074	0.250911	2.694474	0.00704997	0.305590517
GTPBP8	-0.778239	0.289022	-2.692662	0.00708842	0.30576543
EPHB6	-0.719319	0.267679	-2.687248	0.00720435	0.309264875
CLDND2	1.011259	0.377235	2.68071	0.00734661	0.313855824
SLC38A2	-0.399758	0.150721	-2.652309	0.00799434	0.338718053
PCBP4	-1.321227	0.498239	-2.651795	0.0080065	0.338718053
HLA.DMB	0.25139	0.094872	2.649788	0.00805423	0.338718053
ENSG00000224505	0.426816	0.161235	2.647171	0.00811682	0.338718053
PLP2	-0.491261	0.18562	-2.646596	0.00813065	0.338718053
LGALS1	-0.970207	0.366836	-2.644796	0.00817402	0.338718053
ABI3	0.47154	0.178349	2.643912	0.00819541	0.338718053
MEF2A	0.453422	0.171716	2.640542	0.00827736	0.340521563
KCTD21.AS1	1.120297	0.424467	2.639303	0.00830768	NA
SPIB	0.293631	0.111291	2.638402	0.00832978	0.341098651
RUNX2	3.85235	1.462854	2.633447	0.0084523	NA
ZNF419	0.614211	0.233534	2.630072	0.00853669	0.34612604
KEAP1	0.975044	0.370889	2.628941	0.00856513	0.34612604
FTH1	-0.129098	0.049147	-2.626785	0.00861958	0.34612604
FCRL2	0.52605	0.200312	2.626155	0.00863556	0.34612604
DNMT3A	0.854599	0.325598	2.624706	0.00867238	0.34612604
GYPC	0.251402	0.095844	2.623036	0.00871501	0.34612604
PGK1	0.326832	0.12462	2.622637	0.00872521	0.34612604
PLXNB2	1.530281	0.584206	2.619419	0.00880796	NA
NIPAL4	1.428364	0.545399	2.618933	0.00882052	NA
VHL	0.311016	0.118799	2.618009	0.00884444	0.346257513
IKZF1	0.372212	0.142221	2.617147	0.00886681	0.346257513
SNX2	0.22646	0.086557	2.616324	0.00888822	0.346257513
GABPB1	0.250804	0.095882	2.615766	0.00890275	0.346257513
PHF6	-0.606867	0.232193	-2.613631	0.00895858	0.346257513
GIT2	0.287888	0.110155	2.613488	0.00896233	0.346257513
HLA.DMA	0.248027	0.094997	2.61089	0.00903068	0.347388027
SHISAL2A	0.302659	0.11619	2.604852	0.00919141	0.350651463
MIEF2	0.738812	0.28375	2.603739	0.00922131	0.350651463
PRDM4	0.684546	0.262956	2.603271	0.0092339	0.350651463
LRPAP1	0.317244	0.121959	2.601228	0.00928906	0.351245008
ARID4B	0.257501	0.099055	2.599575	0.00933394	0.351446627
PUS1	0.649057	0.250024	2.595983	0.00943207	0.352762648
LSP1	0.248013	0.095611	2.593992	0.00948688	0.352762648
PPP1R18	0.343389	0.132447	2.592651	0.00952395	0.352762648
ENSG00000089127	0.493801	0.190487	2.592306	0.0095335	0.352762648
OS9	0.311454	0.120202	2.591085	0.00956739	0.352762648
CARS2	0.61878	0.239069	2.588287	0.00964544	0.352948343
YARS2	-0.650445	0.251384	-2.587455	0.00966878	0.352948343
NIFK	0.348849	0.134885	2.586274	0.00970198	0.352948343

GALK1	-0.762558	0.295166	-2.583489	0.00978067	0.352948343
WDR11	0.44031	0.170446	2.583281	0.00978657	0.352948343
SLC25A5	-0.199945	0.077425	-2.582429	0.00981074	0.352948343
KRAS	-0.402493	0.156521	-2.571499	0.01012593	0.361266309
AARS	0.586217	0.228026	2.57083	0.01014551	0.361266309
ZKSCAN4	1.012114	0.393788	2.570202	0.01016392	0.361266309
HSPA4L	1.33966	0.521281	2.569939	0.01017163	NA
ENSG00000271937	1.651796	0.642791	2.569724	0.01017796	NA
SMC6	-0.337741	0.131612	-2.566195	0.01028209	0.363816256
POU2AF1	0.279311	0.108928	2.564179	0.01034203	0.363816256
STAU2	0.486674	0.189838	2.563627	0.01035849	0.363816256
GPT2	0.843466	0.329474	2.560034	0.01046619	0.365202267
ZNF555	0.943131	0.368472	2.559571	0.01048015	0.365202267
ALKBH2	0.560683	0.219481	2.554581	0.01063158	0.369032017
C22orf15	1.738637	0.682307	2.548174	0.01082884	NA
CTNNA1	-0.855451	0.336559	-2.541753	0.01102981	0.379982132
DAPK3	0.539287	0.212179	2.541666	0.01103257	0.379982132
ILVBL	0.635741	0.250395	2.538947	0.01111868	0.3813939
SC5D	-0.483658	0.190592	-2.537667	0.0111594	0.3813939
CHL1	1.69234	0.667188	2.536526	0.01119583	NA
TRMT10C	0.366775	0.144801	2.53296	0.01131038	0.383660249
CENPS	-0.995926	0.393195	-2.532908	0.01131206	0.383660249
ZNF827	0.8948	0.353365	2.532224	0.01133414	NA
GHRL	0.621355	0.24549	2.531083	0.0113711	0.383967059
MMP11	0.734328	0.290305	2.529505	0.01142235	0.383967059
SHISA5	0.296411	0.117222	2.528634	0.01145074	0.383967059
ETV6	-0.682143	0.270087	-2.52564	0.01154879	0.385787423
WDR5B	0.679952	0.269359	2.524331	0.01159186	0.385787423
TBC1D9	0.596108	0.236281	2.522884	0.01163969	0.385933936
SH3KBP1	-0.299272	0.118762	-2.519926	0.01173796	0.386591191
ADAMTS7	-1.210683	0.48107	-2.516649	0.01184769	0.386591191
TECR	0.312332	0.124113	2.516511	0.01185232	0.386591191
CCDC22	0.551429	0.219126	2.51649	0.01185303	0.386591191
FXYD5	-0.225537	0.089649	-2.515777	0.01187704	0.386591191
ARL14EPL	3.348189	1.331915	2.513816	0.01194328	NA
SEMA4A	1.685778	0.671139	2.511815	0.01201122	NA
ISG20	-0.276371	0.110052	-2.511268	0.01202984	0.390135508
PTP4A3	1.526123	0.607924	2.510384	0.01205999	NA
ZNF202	0.874463	0.348662	2.508058	0.01213968	0.392266158
U2SURP	0.25854	0.103205	2.505101	0.01224165	0.394127967
TMEM263	0.490411	0.195942	2.502839	0.01232016	0.39522346
XPO4	0.501553	0.200749	2.49841	0.01247518	0.397390593
GRK5	-0.541747	0.216888	-2.497816	0.01249609	0.397390593
PDIA3	0.36076	0.144472	2.497086	0.01252187	0.397390593
PARP14	-0.312246	0.125252	-2.49293	0.01266939	0.400641302
PWP1	0.306952	0.123206	2.491378	0.01272488	0.40096913
EPN1	0.387759	0.155724	2.490033	0.01277314	0.401067418

CCDC57	-0.43046	0.173047	-2.487538	0.01286308	0.402469378
LINC00996	2.294011	0.923728	2.483427	0.01301251	NA
ENDOD1	0.874248	0.352338	2.48128	0.01309113	0.405752013
BANK1	-0.259879	0.104744	-2.481093	0.01309802	0.405752013
SLC12A2	-0.729747	0.294146	-2.480904	0.01310498	0.405752013
ANKAR	0.499334	0.201877	2.473453	0.01338144	0.411480079
ENSG00000236514	0.845073	0.341661	2.473422	0.0133826	0.411480079
C15orf62	-1.088993	0.441134	-2.468622	0.01356343	0.415519324
RASGEF1B	-0.466615	0.189107	-2.467461	0.01360749	0.415519324
ENSG00000282393	1.231029	0.499614	2.463958	0.01374121	NA
FUT2	1.801679	0.731345	2.463517	0.01375815	NA
PNMA1	-1.047758	0.425494	-2.462448	0.01379923	0.419808282
RBM7	0.339964	0.138122	2.461327	0.01384243	0.419808282
ZNF791	0.333792	0.135957	2.45513	0.01408336	0.423486902
C16orf74	0.372945	0.151927	2.45476	0.01409787	0.423486902
KLHDC7B	0.828158	0.337399	2.454535	0.0141067	0.423486902
INSIG1	0.316616	0.129323	2.44826	0.0143548	0.428431493
ENO1	0.261875	0.106978	2.447933	0.01436784	0.428431493
RNF34	-0.489195	0.200076	-2.445041	0.01448357	0.430438013
SLC16A1	1.073688	0.439156	2.444893	0.01448953	NA
ENSG00000281571	0.821669	0.33659	2.441156	0.01464031	0.433645975
MKNK2	-0.360033	0.147647	-2.438472	0.01474948	0.435428201
PIM2	-0.27375	0.112431	-2.434834	0.01489862	0.436711981
ABCC4	2.534362	1.040917	2.43474	0.01490248	NA
ENSG00000166927	-0.889464	0.365376	-2.434378	0.01491741	0.436711981
ANKZF1	-0.350784	0.144129	-2.43382	0.01494041	0.436711981
ENSG00000223881	1.150294	0.473363	2.430045	0.01509694	NA
PBRM1	-0.474302	0.195232	-2.429428	0.01512266	0.440572674
LZTR1	-1.237756	0.509728	-2.428266	0.01517121	NA
SNRNP35	0.391987	0.161428	2.428256	0.01517165	0.440572674
RFC3	0.797418	0.328658	2.426288	0.01525417	0.441526321
CALCOCO1	0.326282	0.134665	2.422918	0.01539638	0.44300078
CR2	0.810639	0.334599	2.422719	0.01540482	0.44300078
SF3A3	0.387419	0.16003	2.420917	0.01548141	0.443767048
FYTTD1	-0.316992	0.131049	-2.418886	0.01556814	0.444818261
IRF2BP2	-0.560265	0.232293	-2.411892	0.01586999	0.451989593
TACC1	-0.506115	0.210328	-2.406312	0.01611449	0.457045692
CDK13	0.420209	0.174687	2.405499	0.01615039	0.457045692
VTI1A	-0.575207	0.239603	-2.400669	0.01636514	0.459352878
SDHAF3	-0.475127	0.197945	-2.4003	0.01638165	0.459352878
LAMTOR5	-0.178771	0.074485	-2.400096	0.01639078	0.459352878
SLC45A3	0.840447	0.350328	2.399027	0.01643869	0.459352878
UTP23	0.413543	0.172722	2.39427	0.0166535	0.463896532
ABTB2	3.064802	1.282388	2.389918	0.01685212	NA
KANK1	1.314955	0.551143	2.38587	0.01703878	NA
MAMSTR	1.091971	0.457817	2.385171	0.01707119	NA
DNAJC17	-0.645973	0.271057	-2.383164	0.01716456	0.474861359

TRUB1	0.76824	0.322417	2.382757	0.01718353	0.474861359
VPS13A	-0.595074	0.249984	-2.380448	0.01729159	0.474861359
COMMD4	-0.492049	0.206717	-2.380299	0.01729861	0.474861359
TM7SF3	0.475231	0.199803	2.378495	0.01738347	0.474861359
MFNG	0.384512	0.161708	2.377807	0.01741594	0.474861359
SLC12A6	0.363736	0.152978	2.377696	0.0174212	0.474861359
PDE7A	0.260916	0.109863	2.374924	0.01755254	0.476978292
COPS7A	-0.627235	0.264474	-2.371633	0.01770969	0.479781396
DAP3	-0.309069	0.130431	-2.369595	0.0178076	0.480500582
SNX9	0.356089	0.150359	2.368261	0.0178719	0.480500582
ENSG00000255031	-1.318319	0.557231	-2.365837	0.01798936	NA
GLIPR2	0.450073	0.190281	2.365309	0.01801501	0.480500582
VIM	-0.5173	0.218704	-2.365292	0.01801585	0.480500582
CTSS	0.225162	0.095228	2.36444	0.01805735	0.480500582
LBR	0.319211	0.135009	2.364372	0.01806068	0.480500582
RNF207	1.732224	0.733224	2.362475	0.01815338	NA
B3GNTL1	1.125878	0.476798	2.361332	0.01820941	NA
AGBL5	-1.236885	0.523809	-2.361328	0.01820964	NA
HSD17B1	1.094302	0.464091	2.357948	0.01837628	NA
UBE2J2	0.284817	0.120793	2.357889	0.01837918	0.484061438
SESN3	-0.330321	0.140097	-2.357808	0.0183832	0.484061438
UBE2Z	-0.52662	0.223446	-2.35681	0.01843268	0.484061438
RAD50	-0.349128	0.148164	-2.356362	0.0184549	0.484061438
BTG2	-0.245463	0.104181	-2.356121	0.0184669	0.484061438
SATB1	0.389716	0.165505	2.354701	0.01853761	0.484485824
ENSG00000226571	-1.905699	0.80968	-2.353644	0.01859042	NA
SNAI3.AS1	-1.507628	0.640973	-2.352091	0.01866822	NA
ACTN1	2.129071	0.906536	2.34858	0.01884515	NA
SFT2D3	1.190923	0.507385	2.34718	0.01891614	NA
WASF2	0.217863	0.092941	2.344103	0.01907291	0.497014354
YIPF4	0.218657	0.093378	2.341631	0.01919971	0.49885565
CARD8.AS1	0.401919	0.172155	2.334637	0.0195624	0.506797218
C1orf50	0.469816	0.201335	2.333502	0.0196218	0.506858345
ENSG00000253106	1.769714	0.758832	2.332155	0.01969256	NA
COMMD6	-0.154394	0.066234	-2.331024	0.0197521	0.508745396
CD99	-0.458725	0.196888	-2.329877	0.01981263	0.508776722
CYTH4	-0.396739	0.17036	-2.328835	0.01986783	0.508776722
R3HDM1	-0.557268	0.239509	-2.326707	0.01998087	0.510201118
ARL2BP	-0.328278	0.141163	-2.325523	0.02004401	0.510346966
MORC2	-1.425361	0.613413	-2.323655	0.02014398	NA
RNF215	1.241696	0.534851	2.321574	0.02025589	NA
PFDN6	0.275056	0.11848	2.321535	0.02025799	0.514321462
ENSG00000272871	-1.733007	0.74684	-2.320452	0.02031644	NA
ENSG00000238198	1.713099	0.738266	2.320437	0.02031724	NA
ISYNA1	0.707331	0.305038	2.31883	0.02040425	0.515457894
GDPGP1	0.967447	0.417261	2.318563	0.02041877	0.515457894
CKLF	-0.466515	0.201355	-2.316871	0.02051075	0.516313061

HNRNPK	0.172969	0.074706	2.315321	0.02059535	0.516978257
NUMBL	-1.128584	0.487522	-2.314942	0.02061612	NA
CD63	-0.41276	0.178563	-2.311572	0.02080125	0.520675889
HSD3B7	2.214864	0.959043	2.309452	0.0209185	NA
CLIC1	-0.212251	0.091974	-2.307718	0.0210148	0.52154266
TOX2	1.860684	0.806374	2.307472	0.02102854	NA
STK17B	-0.275227	0.119295	-2.307121	0.02104807	0.52154266
MRNIP	0.31072	0.134709	2.306602	0.02107701	0.52154266
CD58	-0.719132	0.311901	-2.30564	0.02113074	0.52154266
UNC79	1.805713	0.783373	2.305048	0.02116389	NA
ORMDL2	-0.36344	0.157685	-2.304841	0.0211755	0.52154266
WDPCP	0.725749	0.314996	2.30399	0.02122318	0.52154266
HNRNPH2	0.300727	0.130548	2.303571	0.02124673	0.52154266
EBNA1BP2	-0.493921	0.214585	-2.301748	0.02134941	0.522619356
C12orf42	-0.46036	0.200278	-2.29861	0.02152708	0.525520868
ENSG00000276476	1.625532	0.707445	2.297749	0.02157609	NA
ENSG00000248559	0.795527	0.34655	2.295563	0.0217009	0.528312905
TNNI2	1.007175	0.439022	2.294135	0.02178275	NA
ENSG00000279278	0.610151	0.265987	2.293914	0.02179541	0.529164062
CD3D	2.458991	1.072589	2.292577	0.02187239	NA
RAB5IF	-0.286447	0.125073	-2.290244	0.0220072	0.532323324
EIF2S3	-0.238107	0.104018	-2.289094	0.02207386	0.532323324
RASA3	0.65652	0.286871	2.288554	0.02210526	0.532323324
DLGAP1.AS1	-0.319341	0.139622	-2.287178	0.02218541	0.532809697
PAG1	-1.016597	0.44471	-2.28598	0.02225542	0.533050306
SLC26A2	-0.813646	0.356499	-2.282322	0.02247035	0.534298316
HIP1	-0.998881	0.437696	-2.282133	0.02248147	0.534298316
SLCO3A1	3.093415	1.356913	2.279745	0.02262284	NA
ZNF821	0.404687	0.177604	2.278596	0.02269107	0.534298316
CD200R1	3.360459	1.475364	2.277715	0.02274357	NA
AP1G2	-0.291607	0.12806	-2.277105	0.02277997	0.534298316
ULK1	0.77476	0.340262	2.276954	0.02278895	0.534298316
KIF2A	0.321561	0.141232	2.276829	0.02279647	0.534298316
PIK3IP1	0.28821	0.126607	2.276405	0.02282177	0.534298316
ECHS1	0.320503	0.140809	2.276159	0.02283652	0.534298316
KATNB1	0.7838	0.344445	2.275544	0.02287331	0.534298316
GBP7	0.897925	0.394701	2.274952	0.02290881	0.534298316
ENSG00000251136	0.670324	0.294802	2.273814	0.02297718	0.5344901
AP3S1	0.316169	0.13926	2.270346	0.02318663	0.534870118
LMCD1	2.773108	1.221566	2.270126	0.02319997	NA
RRM2B	-0.478278	0.210752	-2.269392	0.02324449	0.534870118
RPA3	0.199451	0.087898	2.269132	0.02326032	0.534870118
TMUB2	0.314655	0.138734	2.268041	0.02332673	0.534870118
NOTCH1	0.780018	0.34404	2.267233	0.02337598	0.534870118
UBE2V1	0.574914	0.253757	2.265613	0.0234751	0.534870118
DTX3L	0.470538	0.207704	2.265426	0.02348658	0.534870118
S1PR4	0.285693	0.126111	2.265404	0.02348789	0.534870118

KHNYN	-0.554106	0.244678	-2.264632	0.02353525	0.534870118
ENSG00000272583	-1.173289	0.519029	-2.260545	0.02378744	NA
OTUD7B	2.654478	1.175232	2.258683	0.02390309	NA
CISH	1.833614	0.812138	2.257763	0.02396047	NA
RAP2C.AS1	-1.824414	0.808291	-2.257126	0.02400022	NA
TMCO4	0.708119	0.313902	2.255861	0.02407935	0.545839615
SLC9A6	-1.524543	0.676251	-2.254405	0.02417068	NA
MKLN1	-0.48689	0.215977	-2.254365	0.02417324	0.546573618
DNASE2	0.333829	0.148255	2.251725	0.02433965	0.548939469
ABTB1	0.252579	0.112379	2.247574	0.02460336	0.553482167
RORA	1.586803	0.707419	2.243089	0.0248911	NA
UTP4	0.572378	0.255341	2.241619	0.02498599	0.560125051
SETBP1	0.587245	0.262043	2.241021	0.02502472	0.560125051
SLCO5A1	1.513022	0.675188	2.240891	0.02503316	NA
FAM135A	-0.689457	0.307928	-2.239021	0.02515457	0.561616927
UEVLD	-1.240096	0.554651	-2.235813	0.02536403	NA
MCCC1	0.48929	0.219065	2.233534	0.02551372	0.565745108
LPCAT1	-0.795328	0.356116	-2.233338	0.02552666	0.565745108
CTBP2	2.965935	1.328123	2.233178	0.02553722	NA
ARIH2	0.312235	0.139853	2.232593	0.02557578	0.565745108
ENSG00000268027	0.405466	0.181635	2.232315	0.02559414	0.565745108
TAF4B	0.724828	0.325841	2.224486	0.02611579	0.575843346
CIC	0.508508	0.228983	2.220722	0.02636982	0.579738079
CCL4	1.239672	0.558426	2.219939	0.0264229	0.579738079
ENSG00000267152	0.955316	0.430573	2.218708	0.02650658	NA
WDR27	-1.180727	0.532244	-2.218394	0.02652798	NA
ZNF710	-1.567938	0.707382	-2.216536	0.0266548	NA
SMIM20	0.399323	0.180218	2.21577	0.02670726	0.584533698
ENSG00000268798	0.963653	0.435278	2.213879	0.02683712	NA
DPY19L1	-1.667804	0.753592	-2.213139	0.02688804	NA
ENSG00000259001	1.053146	0.476207	2.21153	0.02699914	NA
CENPT	-0.403481	0.182463	-2.211302	0.02701497	0.589815673
POLB	-0.834733	0.377787	-2.209532	0.02713768	0.590227874
CTNNBL1	0.319472	0.144615	2.209114	0.02716669	0.590227874
ALKBH1	0.729601	0.330544	2.207272	0.02729507	0.591570737
REC8	0.592677	0.269173	2.201846	0.02767619	0.598371272
ZNF525	-0.965896	0.438899	-2.200723	0.02775563	0.598632301
HMGB3	-1.679684	0.76378	-2.199173	0.02786561	NA
TPRN	-1.564819	0.711589	-2.19905	0.02787436	NA
ARHGAP4	0.296491	0.134832	2.198962	0.0278806	0.598962369
C1orf122	-0.437903	0.199182	-2.1985	0.0279135	0.598962369
JUN	0.237378	0.108014	2.197663	0.02797315	0.598962369
ENSG00000272909	1.602725	0.72986	2.195934	0.02809668	NA
DMAC1	0.252192	0.114888	2.195105	0.0281561	0.60143044
RUBCNL	0.205292	0.093682	2.19138	0.02842431	0.603162241
PDE4DIP	-0.558289	0.254824	-2.190883	0.02846023	0.603162241
SERHL2	-1.37776	0.628906	-2.190725	0.0284717	NA

TMEM184C	-0.85459	0.390106	-2.190661	0.02847632	0.603162241
AATF	0.316719	0.144746	2.188097	0.02866253	0.603162241
CYB5R4	0.28147	0.128692	2.187152	0.02873142	0.603162241
KATNBL1	-0.326255	0.149196	-2.186757	0.02876029	0.603162241
SLC25A32	0.459155	0.210017	2.186279	0.02879518	0.603162241
TBL1X	-0.589699	0.269739	-2.186182	0.0288023	0.603162241
LINC02576	-0.95578	0.437575	-2.184265	0.02894281	0.603162241
ZNF699	0.560028	0.256438	2.183869	0.02897188	0.603162241
CDC42BPB	-1.004036	0.459815	-2.183564	0.02899427	NA
TET3	0.787919	0.360953	2.182887	0.02904412	0.603162241
HMGB2	0.214939	0.09847	2.182784	0.0290517	0.603162241
ENSG00000250541	1.753766	0.803642	2.182272	0.02908943	NA
HARBI1	0.930962	0.426692	2.181812	0.02912339	NA
NFXL1	-1.030394	0.472962	-2.178598	0.02936152	NA
SNAPC3	0.34851	0.159996	2.178245	0.02938777	0.608717311
COMMD7	-0.463307	0.212828	-2.176909	0.02948735	0.609359443
SLC41A1	0.660721	0.303913	2.174048	0.02970151	0.612361032
IL4R	0.384996	0.177303	2.171401	0.02990091	0.614752003
PPP2R5C	0.230384	0.106162	2.170116	0.02999806	0.614752003
PLCL2	-0.606107	0.279343	-2.16976	0.03002503	0.614752003
ACVR2A	-1.664037	0.767425	-2.168339	0.03013287	NA
SPN	1.081333	0.499704	2.163947	0.03046839	NA
KLF2	0.422931	0.19572	2.160895	0.03070348	0.62668642
ITGB2	0.751286	0.347768	2.160306	0.03074896	0.62668642
CENPJ	-0.800443	0.370744	-2.15902	0.03084865	0.627279318
PTP4A2	-0.296579	0.137505	-2.156863	0.0310163	0.629248561
NRDE2	0.352676	0.163947	2.151161	0.03146351	0.635433559
LYPLAL1	0.333166	0.154878	2.151152	0.03146419	0.635433559
ZFP36L2	-0.444329	0.206719	-2.149433	0.0316001	0.636731185
CTNND1	-0.491109	0.228671	-2.147669	0.03174006	0.63692922
ENSG00000255046	0.730054	0.340087	2.146666	0.03181991	0.63692922
PDLIM2	-0.492249	0.229499	-2.144885	0.03196205	0.63692922
PTPA	-0.740542	0.345329	-2.144456	0.03199634	0.63692922
ATG13	-0.586446	0.273488	-2.14432	0.03200724	0.63692922
DHTKD1	-0.398354	0.185856	-2.143344	0.03208549	0.63692922
ZNF844	0.543806	0.253757	2.143018	0.03211167	0.63692922
UBL4A	-0.423356	0.197802	-2.140305	0.03233012	0.639330234
AMPD3	-0.598599	0.279755	-2.13973	0.03237662	0.639330234
SH2D4A	1.637538	0.765504	2.139164	0.03242239	NA
ENSG00000274605	0.880701	0.411777	2.138782	0.03245335	NA
ENSG00000268093	1.472849	0.690128	2.134167	0.03282908	NA
TMEM222	-0.413552	0.19393	-2.132475	0.03296785	0.648235682
DDX20	0.466635	0.218892	2.131806	0.03302278	0.648235682
DIS3L	0.491174	0.230563	2.130325	0.03314479	0.648235682
ARRDC2	-0.314701	0.147746	-2.13001	0.03317078	0.648235682
ENSG00000272009	0.367123	0.172379	2.129749	0.03319235	0.648235682
ENSG00000278831	1.498052	0.703585	2.129169	0.03324025	NA

SGK1	-1.235664	0.580868	-2.127272	0.03339747	0.650178266
SH3BGRL3	-0.165652	0.077889	-2.126783	0.03343816	0.650178266
POMP	0.164002	0.077152	2.1257	0.03352823	0.65050618
COX16	0.433455	0.204145	2.123272	0.03373107	0.651622013
USP12	-0.807625	0.380424	-2.12296	0.03375721	0.651622013
GPSM1	1.321748	0.622876	2.122007	0.03383715	NA
RNF149	-0.412711	0.194511	-2.121789	0.03385545	0.651622013
BCAS4	0.319505	0.150603	2.121508	0.03387907	0.651622013
LINC00894	1.614172	0.76122	2.120508	0.03396322	NA
TRANK1	-0.386252	0.18226	-2.119237	0.03407047	0.653004745
PEAK1	0.604203	0.285148	2.118912	0.03409793	0.653004745
TBC1D19	-1.108959	0.523369	-2.118888	0.03409993	NA
TMEM159	-0.523949	0.247604	-2.11608	0.03433799	0.656187838
CDK12	-0.346851	0.16412	-2.113395	0.03456694	0.658744737
TFB2M	0.596375	0.282363	2.112083	0.03467933	0.658744737
KIAA1586	0.56627	0.268132	2.11191	0.03469419	0.658744737
SUMO4	0.965728	0.457332	2.111655	0.03471608	NA
LILRA2	1.687359	0.800287	2.108441	0.03499288	NA
HCK	-0.586759	0.278342	-2.108049	0.03502675	0.663533123
LRRC42	-1.015187	0.481605	-2.107924	0.03503753	NA
PATL2	0.401757	0.190654	2.107252	0.03509572	0.663533123
BTBD3	-1.427998	0.678323	-2.105188	0.03527496	NA
TRADD	-0.334564	0.158982	-2.104421	0.03534174	0.666765883
STK38	0.448444	0.213441	2.101026	0.03563868	0.668143221
EVL	0.24623	0.11728	2.099503	0.0357726	0.668143221
WDFY2	-0.407922	0.194384	-2.098535	0.03585792	0.668143221
TMC8	0.318986	0.152016	2.098365	0.03587291	0.668143221
ANKH	-0.795206	0.378967	-2.098351	0.03587411	0.668143221
NSMCE1	0.251369	0.119824	2.097814	0.03592155	0.668143221
SCPEP1	0.448259	0.213702	2.097593	0.03594108	0.668143221
SREBF1	0.859335	0.41019	2.09497	0.03617365	NA
SEC61B	-0.26419	0.12612	-2.094751	0.03619312	0.670889638
AP2S1	-0.25512	0.121823	-2.09419	0.03624309	0.670889638
SELENOW	-0.29627	0.141527	-2.093379	0.03631532	0.670889638
COQ8A	0.428399	0.204806	2.091724	0.03646323	0.6722246
ABRAXAS2	0.460359	0.220228	2.090372	0.03658443	0.673027589
CD81	0.359844	0.172211	2.08955	0.03665827	0.673027589
MPP7	-1.445318	0.691909	-2.088885	0.03671807	NA
PRDM2	-0.231193	0.110774	-2.087069	0.0368819	0.675737256
ECE2	-1.427793	0.684793	-2.085	0.03706934	NA
AUH	0.545157	0.261486	2.084841	0.03708373	0.676106133
DYNC1I2	-0.233778	0.112173	-2.084076	0.03715328	0.676106133
RHOG	-0.279282	0.134011	-2.08402	0.03715836	0.676106133
TSC22D1	-0.719538	0.345392	-2.08325	0.03722841	0.676106133
TMEM156	0.329436	0.15818	2.082657	0.03728247	0.676106133
CCR7	0.177221	0.085137	2.081595	0.03737947	0.676484632
TTC3	-0.188894	0.090834	-2.079562	0.03756569	0.677724336

WDR81	-0.718024	0.34534	-2.079183	0.03760051	0.677724336
ENSG00000249141	1.563952	0.752657	2.077909	0.03771772	NA
SCML4	0.963599	0.464079	2.076369	0.03785985	NA
ZNF211	-0.609282	0.293464	-2.076169	0.0378783	0.680096389
EIF4B	-0.147729	0.071157	-2.076095	0.03788518	0.680096389
CCNDBP1	0.170529	0.082196	2.074655	0.03801849	0.681113496
ZMAT2	0.201125	0.097027	2.072878	0.03818366	0.68269622
ENSG00000260261	-1.152807	0.556179	-2.072728	0.0381976	NA
CD68	-1.144494	0.552323	-2.072145	0.03825194	NA
ZNF682	0.676604	0.326737	2.070794	0.03837808	0.684497804
LINC01991	-1.698018	0.820375	-2.069807	0.03847046	NA
ENSG00000251661	-1.580089	0.763529	-2.069456	0.03850332	NA
RHEX	-0.77452	0.374299	-2.069255	0.03852214	0.684497804
UBE2G1	0.347468	0.167977	2.068539	0.03858938	0.684497804
UBTF	0.310753	0.150257	2.068142	0.03862671	0.684497804
LUC7L	-0.399969	0.193504	-2.06698	0.03873603	0.684497804
ZFYVE27	0.346623	0.16772	2.06668	0.03876435	0.684497804
GUCD1	0.281474	0.136279	2.065428	0.03888256	0.684497804
CYTIP	-0.151744	0.073475	-2.065236	0.03890067	0.684497804
TAX1BP3	-0.425046	0.205982	-2.063513	0.03906393	0.686012029
NEB	1.265365	0.61321	2.063511	0.03906412	NA
ENSG00000257497	1.088646	0.528107	2.061413	0.03926368	NA
GABARAPL2	0.175382	0.085088	2.061185	0.03928537	0.687500692
ENSG00000272112	-1.577666	0.765724	-2.06036	0.03936414	NA
COPA	-0.350062	0.169914	-2.060232	0.03937633	0.687500692
PTPRE	-0.643914	0.312552	-2.060186	0.03938081	0.687500692
LINC00158	2.985874	1.450108	2.05907	0.0394875	NA
SOCS7	-1.294861	0.629251	-2.057781	0.03961118	NA
ASCC1	0.511005	0.248339	2.057695	0.0396194	0.689415317
NCBP2	-0.253837	0.123376	-2.057422	0.03964565	0.689415317
LMNA	-1.7363	0.843935	-2.057385	0.03964919	NA
ENSG00000278730	-1.003273	0.488139	-2.0553	0.03985007	NA
SLK	-0.657208	0.319929	-2.054231	0.03995332	0.692434773
TFB1M	0.437085	0.212797	2.054006	0.03997513	0.692434773
GLT8D1	0.445273	0.217038	2.051596	0.04020899	0.693979782
FUOM	0.541198	0.263809	2.051477	0.04022053	0.693979782
PALLD	2.711564	1.322488	2.05035	0.04033025	NA
DNAJC11	0.616504	0.300774	2.049721	0.04039163	0.694060698
PLEKHB1	0.877162	0.427969	2.04959	0.04040441	0.694060698
FAM89A	1.223155	0.596973	2.04893	0.04046895	NA
BRK1	0.16727	0.081645	2.04875	0.04048653	0.694060698
ENAH	1.864233	0.91014	2.048293	0.04053129	NA
IRF8	0.261305	0.127593	2.047953	0.04056461	0.694060698
TBKBP1	1.764625	0.861863	2.047454	0.04061352	NA
BIN2	0.511444	0.24998	2.045937	0.04076256	0.694060698
FALEC	0.523721	0.256008	2.045721	0.04078388	0.694060698
MYL2	2.370648	1.159078	2.045288	0.0408265	NA

ZNF430	0.254532	0.124458	2.045125	0.0408425	0.694060698
H2AFY	0.298912	0.146164	2.045049	0.04085007	0.694060698
AGAP3	1.787074	0.873999	2.04471	0.04088345	NA
QPRT	2.141693	1.047514	2.044549	0.04089939	NA
MAN2A2	-0.93186	0.456006	-2.043523	0.04100067	0.695029737
PLEKHB2	0.326656	0.159899	2.042888	0.04106354	0.695029737
CPEB4	0.505881	0.247961	2.04016	0.04133438	0.698187465
RAB13	-0.871541	0.427346	-2.039429	0.04140725	0.698187465
SLC5A5	2.477055	1.215873	2.037265	0.04162351	NA
ATPSCKMT	0.875822	0.430469	2.034576	0.04189359	NA
NIN	-0.345018	0.169578	-2.034567	0.0418945	0.70399374
MOB1B	-1.225211	0.602497	-2.033555	0.04199648	NA
TRBC2	-0.148396	0.07303	-2.031988	0.04215485	0.70399374
PLOD1	-1.537878	0.756964	-2.03164	0.04219009	NA
PORCN	0.697619	0.343496	2.030936	0.04226151	0.70399374
ICA1L	0.376887	0.185596	2.03068	0.04228746	0.70399374
GTPBP10	0.465368	0.229186	2.030523	0.04230341	0.70399374
MDM4	-0.155543	0.076607	-2.030413	0.04231459	0.70399374
COL9A2	-1.549929	0.763537	-2.029935	0.04236318	NA
CLCN7	0.419922	0.207016	2.028451	0.04251426	0.70399374
ENSG00000269983	1.136111	0.560102	2.0284	0.04251943	NA
GEN1	-0.854771	0.421467	-2.028084	0.04255168	0.70399374
RFX1	-0.447451	0.220745	-2.027003	0.04266207	0.70399374
CHMP2A	-0.252295	0.124506	-2.026366	0.0427273	0.70399374
ENSG00000268746	1.786184	0.881563	2.026155	0.04274889	NA
JTB	-0.137319	0.06783	-2.024456	0.0429233	0.70399374
SRPRA	0.386249	0.190823	2.024114	0.04295839	0.70399374
RFFL	0.501083	0.247623	2.023574	0.04301405	0.70399374
SP4	-0.648675	0.320776	-2.022204	0.04315525	0.70399374
UAP1	-0.522621	0.258469	-2.021989	0.04317752	0.70399374
ZNF793.AS1	-1.573025	0.778043	-2.021771	0.04320003	NA
RBM14	0.365542	0.180937	2.020269	0.04335553	0.70399374
EMC2	0.462688	0.2291	2.019589	0.04342602	0.70399374
FAM174B	1.367019	0.677194	2.018651	0.04352348	NA
CDC42SE1	0.279777	0.138606	2.018514	0.04353778	0.70399374
COBLL1	-0.43267	0.214379	-2.018244	0.04356584	0.70399374
RIC1	-0.755823	0.374738	-2.016936	0.04370224	0.70399374
BARD1	0.414328	0.205468	2.016512	0.04374649	0.70399374
PSMB3	0.209246	0.103808	2.015695	0.04383183	0.70399374
HADHA	-0.23802	0.11812	-2.015074	0.04389686	0.70399374
UBA2	0.248664	0.123408	2.01498	0.04390678	0.70399374
ADAM10	-0.361002	0.179161	-2.014953	0.04390953	0.70399374
COPS9	-0.242521	0.120377	-2.014675	0.04393875	0.70399374
TM9SF2	0.286711	0.142354	2.01407	0.04400221	0.70399374
PYGM	0.975405	0.484308	2.014017	0.04400773	NA
PDCD2L	0.686914	0.34111	2.013762	0.04403449	0.70399374
RCC1	0.398581	0.197942	2.013623	0.04404912	0.70399374

ENSG00000231212	1.492263	0.741091	2.013604	0.04405114	NA
FAM129A	2.030821	1.009271	2.012166	0.04420244	NA
TBC1D8	1.094406	0.543993	2.011802	0.04424077	NA
C8orf88	-0.857199	0.426312	-2.010732	0.04435378	0.706649081
KLHDC10	-0.593551	0.29522	-2.010538	0.04437432	0.706649081
ITM2B	0.230838	0.114893	2.009163	0.0445198	0.707423971
ENSG00000255856	1.597027	0.794913	2.00906	0.04453076	NA
MYO15B	-0.764663	0.380699	-2.008575	0.0445822	0.707423971
GCSH	-0.49434	0.246272	-2.007295	0.04471828	0.708117478
STYXL1	0.509049	0.253679	2.006666	0.04478528	0.708117478
ENSG00000273329	0.647006	0.322631	2.005408	0.04491949	0.708977872
GOSR2	0.39963	0.199457	2.003591	0.04511387	0.709337577
ENSG00000231856	-1.007585	0.502987	-2.003205	0.04515534	NA
ANKRD44	0.19768	0.098714	2.002558	0.04522473	0.709337577
TAGAP	0.24493	0.12233	2.002206	0.04526258	0.709337577
ENSG00000272758	0.628261	0.313857	2.001741	0.04531265	0.709337577
ZNF658	1.106835	0.552984	2.001569	0.04533112	NA
RELA	0.313001	0.156385	2.001473	0.04534141	0.709337577
FAM50A	-0.462897	0.231522	-1.999367	0.04556862	0.711639235
ADAM19	0.363067	0.181821	1.996836	0.04584305	0.714668942
IGLV2.14	-1.070371	0.53695	-1.993426	0.04621483	NA
ENSG00000277959	-1.486169	0.745839	-1.992615	0.04630362	NA
STK3	-0.707039	0.354831	-1.992608	0.04630438	0.718949978
S100A11	-0.427628	0.214677	-1.991957	0.04637582	0.718949978
ANKRD36	-0.58821	0.295387	-1.991318	0.04644593	0.718949978
PAXBP1	-0.784999	0.394297	-1.990881	0.04649397	0.718949978
MPPE1	0.330866	0.166212	1.990624	0.0465222	0.718949978
TPCN2	0.75472	0.379572	1.988344	0.04677369	NA
LAPTM5	-0.16618	0.08358	-1.988267	0.04678214	0.720460663
ZBTB8OS	0.240033	0.120791	1.987169	0.04690368	0.720460663
UTP14A	-0.537342	0.270586	-1.985843	0.04705071	0.720460663
SUCLG2	0.330778	0.166682	1.984488	0.04720148	0.720460663
RAB7A	0.204762	0.103182	1.984472	0.04720323	0.720460663
SYVN1	0.322361	0.162443	1.984458	0.04720482	0.720460663
APH1A	-0.201953	0.101769	-1.984417	0.04720942	0.720460663
TARDBP	-0.265617	0.133887	-1.983886	0.04726858	0.720460663
IL10RB.DT	-1.408163	0.709867	-1.983702	0.04728912	NA
ZMIZ1	-1.641262	0.82745	-1.983519	0.04730944	NA
ANKRD34A	1.402839	0.708359	1.980407	0.04765776	NA
ABCB4	0.296939	0.149959	1.980137	0.0476881	0.724010686
CYREN	-0.416447	0.210339	-1.979882	0.04771676	0.724010686
BTBD19	3.874803	1.957147	1.979822	0.04772348	NA
DNAAF4	-1.347148	0.680462	-1.979754	0.04773114	NA
CIR1	0.219098	0.110677	1.979623	0.04774592	0.724010686
LITAF	0.188483	0.09528	1.978202	0.04790594	0.725199614
MAPK14	-0.549451	0.277899	-1.977163	0.04802325	0.725739175
YPEL2	-0.283695	0.143546	-1.976339	0.04811641	0.72579273

ITPR1	0.378465	0.191561	1.975688	0.04819015	0.72579273
SDR42E1	-1.513508	0.766472	-1.974643	0.04830869	NA
SLC9A3R1	0.393943	0.199542	1.974238	0.04835463	0.727037679
KDM4D	1.687461	0.855017	1.973599	0.04842741	NA
ENSG00000260572	1.570798	0.795933	1.973531	0.04843514	NA
HIP1R	0.274686	0.139304	1.971835	0.04862842	0.727704943
PPP2R5B	-0.531331	0.269482	-1.971674	0.04864687	0.727704943
PAGR1	0.454342	0.230465	1.97141	0.04867695	0.727704943
CYBC1	-0.182722	0.092706	-1.970976	0.04872659	0.727704943
ENSG00000232010	1.116885	0.566882	1.970224	0.04881272	NA
DMXL1	-0.393243	0.199607	-1.970086	0.0488285	0.72800338
ZNF616	-1.084889	0.550723	-1.969937	0.04884563	NA
ZBTB32	2.39419	1.216582	1.967965	0.04907208	NA
MAF1	0.196153	0.09976	1.966258	0.04926882	0.731476049
IER3	1.209917	0.615375	1.966147	0.04928164	NA
SP100	0.164572	0.083708	1.966031	0.04929499	0.731476049
MCM3	0.390211	0.198488	1.965916	0.04930837	0.731476049

Cluster 3	log2FC	lfcSE	stat	pvalue	padj
VPREB3	3.887109	0.113796	34.15865	< 2.22e-16	< 2.22e-16
DENND6B	3.462668	0.157059	22.046985	< 2.22e-16	< 2.22e-16
GRN	2.005959	0.102087	19.649487	< 2.22e-16	< 2.22e-16
CD1D	5.570262	0.292138	19.067205	< 2.22e-16	< 2.22e-16
CD38	4.41865	0.241855	18.269795	< 2.22e-16	< 2.22e-16
PLAAT4	2.769813	0.163928	16.896484	< 2.22e-16	< 2.22e-16
NRIP1	2.270827	0.14061	16.1498	< 2.22e-16	< 2.22e-16
RABGAP1L	1.649413	0.117939	13.985332	< 2.22e-16	< 2.22e-16
P2RX5	1.592606	0.119737	13.300881	< 2.22e-16	< 2.22e-16
RNF207	3.360265	0.253871	13.236125	< 2.22e-16	< 2.22e-16
AHI1	1.619582	0.124304	13.029232	< 2.22e-16	< 2.22e-16
SLC2A5	3.260712	0.250436	13.020164	< 2.22e-16	< 2.22e-16
RARA.AS1	2.30394	0.184471	12.489433	< 2.22e-16	< 2.22e-16
IRAK2	1.543204	0.126304	12.218144	< 2.22e-16	< 2.22e-16
ASB2	8.698217	0.743805	11.694221	< 2.22e-16	< 2.22e-16
ENDOD1	1.92285	0.166119	11.575132	< 2.22e-16	< 2.22e-16
APOL3	1.200093	0.104814	11.449705	< 2.22e-16	< 2.22e-16
SLC12A4	2.492814	0.219336	11.365275	< 2.22e-16	< 2.22e-16
LCAT	2.663433	0.235088	11.329504	< 2.22e-16	< 2.22e-16
AGO1	1.175843	0.104094	11.296007	< 2.22e-16	< 2.22e-16
C12orf65	1.139716	0.101061	11.277446	< 2.22e-16	< 2.22e-16
DUS2	1.622667	0.147321	11.014527	< 2.22e-16	< 2.22e-16
P2RX5.TAX1BP3	1.95049	0.179825	10.846608	< 2.22e-16	< 2.22e-16
SLC5A3	1.500122	0.144479	10.382947	< 2.22e-16	< 2.22e-16
RMDN2	2.307724	0.227744	10.13297	< 2.22e-16	< 2.22e-16
SEPTIN9	0.708708	0.072207	9.814982	< 2.22e-16	< 2.22e-16
ZNF563	1.534761	0.158476	9.684476	< 2.22e-16	< 2.22e-16
PLAAT3	3.583579	0.376796	9.510663	< 2.22e-16	< 2.22e-16
MS4A1	-0.808967	0.086907	-9.30845	< 2.22e-16	< 2.22e-16
GBP4	1.026648	0.110676	9.276144	< 2.22e-16	< 2.22e-16
SMAD3	1.037043	0.112778	9.195401	< 2.22e-16	< 2.22e-16
FAM117A	0.993384	0.111553	8.905053	< 2.22e-16	< 2.22e-16
CD72	1.337239	0.150967	8.857796	< 2.22e-16	2.94E-16
MRPS6	1.35474	0.153951	8.799831	< 2.22e-16	4.79E-16
RRBP1	1.693871	0.192593	8.795065	< 2.22e-16	4.86E-16
ABCB1	1.708441	0.199898	8.546577	< 2.22e-16	4.19E-15
PNOC	1.961278	0.231614	8.467879	< 2.22e-16	8.03E-15
GPX1	0.891985	0.106989	8.33715	< 2.22e-16	2.38E-14
DPEP2	1.082573	0.131339	8.242603	< 2.22e-16	5.14E-14
RARA	0.885835	0.107989	8.203043	2.34E-16	6.97E-14
LGMN	1.648739	0.204954	8.044434	8.66E-16	2.51E-13
CD24	-0.863758	0.109604	-7.880722	3.25E-15	9.21E-13
DDX24	0.49968	0.063984	7.809511	5.74E-15	1.59E-12
FXYD5	-0.579303	0.074307	-7.796118	6.38E-15	1.72E-12
TENT5C	-0.812347	0.104363	-7.783891	7.03E-15	1.86E-12
CNP	0.857932	0.110331	7.775982	7.49E-15	1.93E-12

PAOX	0.994067	0.128896	7.712134	1.24E-14	3.13E-12
IFI30	1.195293	0.161045	7.422108	1.15E-13	2.85E-11
CDKN1A	0.82084	0.113982	7.201474	5.96E-13	1.45E-10
KCNN1	2.347863	0.328489	7.147466	8.84E-13	2.10E-10
VPREB1	4.315472	0.606483	7.115575	1.11E-12	2.60E-10
TSPAN18	5.109549	0.719588	7.100655	1.24E-12	2.84E-10
CD1C	1.96068	0.277103	7.075638	1.49E-12	3.34E-10
FCRL2	1.156541	0.164273	7.040356	1.92E-12	4.22E-10
TRABD	0.585181	0.083228	7.031081	2.05E-12	4.43E-10
RASGEF1B	-0.819782	0.11725	-6.991718	2.72E-12	5.76E-10
SIGIRR	0.866777	0.125167	6.924974	4.36E-12	9.09E-10
HIPK2	1.217363	0.17947	6.783093	1.18E-11	2.41E-09
IRF2BPL	0.998802	0.148002	6.74859	1.49E-11	3.01E-09
CACYBP	0.494565	0.073622	6.717666	1.85E-11	3.66E-09
CTSZ	0.650209	0.097794	6.648787	2.96E-11	5.76E-09
SLC16A3	0.972008	0.146426	6.638216	3.18E-11	6.09E-09
RAB20	2.148246	0.324046	6.629455	3.37E-11	6.36E-09
PLXNB2	1.744811	0.26476	6.59015	4.39E-11	8.16E-09
AP3B1	0.926273	0.140724	6.582198	4.64E-11	8.48E-09
SPRY1	1.236919	0.188046	6.577747	4.78E-11	8.60E-09
ARID5B	-0.623276	0.095484	-6.527532	6.69E-11	1.19E-08
FAM3C	0.7931	0.122298	6.485003	8.87E-11	1.55E-08
FADS3	-0.614174	0.095564	-6.426854	1.30E-10	2.24E-08
DBNL	0.575332	0.089881	6.401055	1.54E-10	2.62E-08
XYLT1	0.917143	0.143874	6.374613	1.83E-10	3.07E-08
MCL1	0.499427	0.078583	6.355433	2.08E-10	3.43E-08
CABLES1	-1.585296	0.249685	-6.349177	2.16E-10	3.52E-08
BHLHE40	0.906383	0.144304	6.281049	3.36E-10	5.40E-08
SLC43A2	0.555293	0.088521	6.273019	3.54E-10	5.61E-08
LY9	-0.823716	0.132355	-6.223547	4.86E-10	7.60E-08
BTBD7	0.640597	0.103166	6.209358	5.32E-10	8.21E-08
IER3	1.555287	0.250562	6.207199	5.39E-10	8.22E-08
SLC45A3	1.174046	0.190212	6.172315	6.73E-10	1.01E-07
SLC5A5	4.358378	0.710893	6.130854	8.74E-10	1.30E-07
NMT2	-0.793189	0.130464	-6.079739	1.20E-09	1.77E-07
SPOCK2	0.696679	0.114845	6.066238	1.31E-09	1.90E-07
GPR183	-0.989255	0.163507	-6.050223	1.45E-09	2.07E-07
ZNF3	0.693639	0.114886	6.037652	1.56E-09	2.21E-07
AGPAT5	0.857699	0.143752	5.966536	2.42E-09	3.39E-07
SIGLEC10	1.270609	0.213582	5.949059	2.70E-09	3.73E-07
VHL	0.563169	0.09558	5.892136	3.81E-09	5.21E-07
HIC1	3.729863	0.635065	5.873197	4.27E-09	5.77E-07
LHFPL2	1.271461	0.216828	5.863923	4.52E-09	6.04E-07
FOXO3	0.998689	0.170848	5.845497	5.05E-09	6.67E-07
NUP210	0.520615	0.089294	5.830369	5.53E-09	7.22E-07
CLEC17A	0.92956	0.160111	5.805734	6.41E-09	8.28E-07
RHOBTB3	2.743626	0.475744	5.767027	8.07E-09	1.03E-06

SREBF1	1.180648	0.206016	5.73085	9.99E-09	1.26E-06
HDAC9	0.690904	0.121547	5.684244	1.31E-08	1.64E-06
ENSG00000166927	-1.402074	0.247638	-5.661797	1.50E-08	1.85E-06
CHST15	0.921738	0.163098	5.65142	1.59E-08	1.95E-06
FXYD7	-1.496551	0.266737	-5.610589	2.02E-08	2.45E-06
LBR	0.449293	0.081131	5.537885	3.06E-08	3.68E-06
ATXN10	0.633054	0.114949	5.507249	3.64E-08	4.33E-06
FXYD1	-1.319706	0.24028	-5.49237	3.97E-08	4.67E-06
KANSL3	0.990913	0.180556	5.488131	4.06E-08	4.73E-06
GDPGP1	1.312121	0.240043	5.466191	4.60E-08	5.31E-06
PLEKHO1	0.515916	0.094459	5.461812	4.71E-08	5.39E-06
HCK	-1.30449	0.239268	-5.451998	4.98E-08	5.64E-06
SMC6	-0.449224	0.082717	-5.430869	5.61E-08	6.29E-06
ARL14EPL	4.386995	0.813118	5.395276	6.84E-08	7.60E-06
RESF1	-0.40539	0.075352	-5.379974	7.45E-08	8.20E-06
MDM4	-0.379953	0.070893	-5.359512	8.34E-08	9.10E-06
CASP7	0.70752	0.132085	5.356556	8.48E-08	9.17E-06
ENSG00000214797	2.313623	0.432797	5.345741	9.00E-08	9.64E-06
GBP7	1.449089	0.271768	5.332079	9.71E-08	1.03E-05
PUS1	0.843946	0.158514	5.324117	1.01E-07	1.07E-05
SEL1L3	0.694927	0.130842	5.311199	1.09E-07	1.14E-05
ARHGEF3	0.611048	0.115355	5.297122	1.18E-07	1.21E-05
PPCDC	0.996469	0.188125	5.296859	1.18E-07	1.21E-05
SLCO5A1	1.79338	0.342955	5.229194	1.70E-07	1.73E-05
FCRL1	0.98629	0.190106	5.188096	2.12E-07	2.14E-05
JUNB	0.43347	0.083864	5.168719	2.36E-07	2.35E-05
MAPKAPK2	0.764263	0.147987	5.164405	2.41E-07	2.39E-05
NDUFB9	0.362113	0.070294	5.151397	2.59E-07	2.54E-05
ENSG00000261448	1.760381	0.343043	5.131662	2.87E-07	2.78E-05
MZB1	-1.040178	0.202719	-5.131127	2.88E-07	2.78E-05
JCHAIN	2.189688	0.427454	5.12263	3.01E-07	2.89E-05
ZFP36L1	-0.592864	0.116713	-5.079669	3.78E-07	3.60E-05
SH3KBP1	-0.431457	0.085466	-5.04828	4.46E-07	4.21E-05
C15orf39	0.933854	0.185365	5.037927	4.71E-07	4.40E-05
GGA2	0.528325	0.105271	5.01871	5.20E-07	4.83E-05
CPEB4	0.916013	0.184396	4.967634	6.78E-07	6.25E-05
BRK1	0.270058	0.05446	4.958795	7.09E-07	6.49E-05
CIB1	0.340434	0.068888	4.941834	7.74E-07	7.02E-05
LBH	-0.456258	0.092623	-4.925966	8.39E-07	7.56E-05
REC8	0.773535	0.15715	4.922261	8.56E-07	7.65E-05
GPR146	-1.34108	0.272539	-4.920685	8.62E-07	7.65E-05
ICAM1	1.242197	0.252793	4.913899	8.93E-07	7.86E-05
PALLD	2.421305	0.497314	4.86876	1.12E-06	9.82E-05
HTR3A	1.748883	0.359481	4.865027	1.14E-06	9.93E-05
PIP5K1B	1.084064	0.223207	4.85677	1.19E-06	0.000102776
CD180	0.859127	0.176978	4.854435	1.21E-06	0.000103246
CHST7	2.046558	0.421798	4.851984	1.22E-06	0.000103784

SERPINF1	-1.742716	0.361004	-4.827419	1.38E-06	0.000116605
FOXP1	-0.208179	0.043243	-4.814116	1.48E-06	0.00012377
CYP2R1	-0.669923	0.139855	-4.790121	1.67E-06	0.000138555
ENSG00000198106	-1.016351	0.213417	-4.762288	1.91E-06	0.000158006
APOBEC3H	1.639532	0.344562	4.758307	1.95E-06	0.000160043
ISCA1	0.382806	0.080669	4.745377	2.08E-06	0.000169445
MPEG1	1.521988	0.321243	4.73781	2.16E-06	0.000174699
KLF13	0.397485	0.084114	4.725568	2.29E-06	0.000184307
NFAT5	0.643041	0.136231	4.720228	2.36E-06	0.000187943
C16orf54	0.625699	0.13261	4.718336	2.38E-06	0.000188434
FYTTD1	-0.408813	0.086794	-4.710147	2.48E-06	0.000194866
SYTL1	-0.612436	0.1303	-4.700194	2.60E-06	0.000203263
GLIPR2	0.636961	0.136359	4.671204	2.99E-06	0.000232643
DDX28	0.621814	0.133314	4.664294	3.10E-06	0.000239036
STK17B	-0.489648	0.105323	-4.649039	3.33E-06	0.000255751
PSMB10	0.418783	0.090864	4.608915	4.05E-06	0.000308434
KDM2B	0.4484	0.097353	4.605927	4.11E-06	0.000310903
SPN	1.556909	0.339755	4.582447	4.60E-06	0.000345752
ANAPC16	0.369124	0.080692	4.574482	4.77E-06	0.000355927
NANP	1.05869	0.231471	4.573746	4.79E-06	0.000355927
H3F3A	0.385573	0.085612	4.503712	6.68E-06	0.000493027
BCL11A	-0.469864	0.104836	-4.481899	7.40E-06	0.000542852
ENSG00000274536	-1.142875	0.255221	-4.477977	7.54E-06	0.000549527
CD79B	0.369764	0.082616	4.475711	7.62E-06	0.000550738
ICAM2	0.671641	0.150091	4.474902	7.64E-06	0.000550738
LINC02576	-1.993854	0.447288	-4.457654	8.29E-06	0.000593358
EHD1	0.499822	0.112779	4.431882	9.34E-06	0.000664919
NXT1	0.394871	0.089313	4.42121	9.81E-06	0.000694467
MIR181A2HG	1.507331	0.3412	4.41773	9.97E-06	0.000701565
RUNX2	3.096334	0.701543	4.413604	1.02E-05	0.000710868
CERK	0.838742	0.190491	4.40304	1.07E-05	0.000741664
ITGB7	0.52488	0.11924	4.401883	1.07E-05	0.000741664
PCBP2	-0.202665	0.046247	-4.38228	1.17E-05	0.000803604
TGFBR2	-0.400988	0.091509	-4.381934	1.18E-05	0.000803604
BCAR3	1.770814	0.406444	4.356843	1.32E-05	0.000896294
ST3GAL1	0.670778	0.154233	4.349124	1.37E-05	0.000923152
CLIC3	2.296545	0.528866	4.342393	1.41E-05	0.000946523
CHRAC1	-0.406758	0.093805	-4.336227	1.45E-05	0.000966781
CTNND1	-0.575463	0.13274	-4.335268	1.46E-05	0.000966781
SMIM3	1.888098	0.435993	4.330571	1.49E-05	0.000982153
SELENOF	0.216076	0.050068	4.315686	1.59E-05	0.001044924
PPFIBP2	0.770316	0.178979	4.303951	1.68E-05	0.001090747
SLC2A3	-0.647438	0.150435	-4.303765	1.68E-05	0.001090747
CPNE5	1.279915	0.298616	4.286152	1.82E-05	0.001174452
GAK	0.457713	0.107467	4.259102	2.05E-05	0.001318813
RINL	0.446452	0.10489	4.256377	2.08E-05	0.001327807
MDM2	0.496295	0.117238	4.233224	2.30E-05	0.001464354

KMT2E	-0.233903	0.055368	-4.224508	2.39E-05	0.001514099
C9orf16	-0.326691	0.07739	-4.221366	2.43E-05	0.001524221
CHP1	-0.375552	0.08898	-4.220622	2.44E-05	0.001524221
PSME2	0.338298	0.080194	4.218486	2.46E-05	0.001530672
PPP1R18	0.458979	0.109026	4.20983	2.56E-05	0.001576997
RPS4X	-0.393031	0.09337	-4.209404	2.56E-05	0.001576997
NRM	0.58498	0.139311	4.199101	2.68E-05	0.001641976
UBTF	0.448848	0.107412	4.178747	2.93E-05	0.001783428
MCM5	0.531499	0.127213	4.178017	2.94E-05	0.001783428
SNAP23	0.279611	0.067384	4.149509	3.33E-05	0.002010467
CTSB	0.475908	0.115365	4.125243	3.70E-05	0.002218475
NIN	-0.441364	0.107008	-4.124591	3.71E-05	0.002218475
DMD	-0.764948	0.185744	-4.118288	3.82E-05	0.002268617
TELO2	0.67223	0.163607	4.108815	3.98E-05	0.00235194
POU2AF1	0.356068	0.086794	4.102451	4.09E-05	0.002405621
TFAM	0.423631	0.103424	4.096071	4.20E-05	0.002460679
MAST4	-1.418296	0.346938	-4.088038	4.35E-05	0.002534941
SIGLEC14	1.092079	0.267623	4.080658	4.49E-05	0.002597933
TOE1	0.592041	0.145106	4.080072	4.50E-05	0.002597933
NPM1	-0.294616	0.072288	-4.075587	4.59E-05	0.002635728
EBF1	-0.63628	0.156918	-4.054863	5.02E-05	0.002866807
ERAP1	0.525501	0.129917	4.044899	5.23E-05	0.002977191
LSP1	0.421493	0.104366	4.038617	5.38E-05	0.00304224
MYC	0.531569	0.131655	4.037598	5.40E-05	0.00304224
SMARCB1	0.349729	0.086748	4.031542	5.54E-05	0.003106996
PTPRE	-0.96363	0.239299	-4.026878	5.65E-05	0.003154366
FAM167A	1.447748	0.360402	4.017034	5.89E-05	0.003264198
SESN3	-0.357581	0.089025	-4.016617	5.90E-05	0.003264198
PAG1	-1.064004	0.265862	-4.002095	6.28E-05	0.003455163
ZNF44	0.458085	0.114726	3.992874	6.53E-05	0.003575811
C7orf50	-0.70333	0.176779	-3.978584	6.93E-05	0.003771726
ENSG00000261766	0.760216	0.191104	3.978031	6.95E-05	0.003771726
NCF1	0.390785	0.098364	3.972861	7.10E-05	0.003837039
ATP6V1E1	0.423805	0.107201	3.953361	7.71E-05	0.004144908
A4GALT	1.504609	0.3813	3.946001	7.95E-05	0.004255088
TTC3	-0.336015	0.085185	-3.944507	8.00E-05	0.004262493
GTF2E2	0.429522	0.108978	3.941351	8.10E-05	0.004299692
ABI3	0.577494	0.146729	3.935791	8.29E-05	0.004380921
RUBCN	-0.60453	0.153684	-3.933582	8.37E-05	0.004401828
PHF21A	-0.454951	0.11595	-3.923682	8.72E-05	0.004566595
TOP1	0.335956	0.085666	3.921709	8.79E-05	0.004583955
CD48	-0.427216	0.109237	-3.910896	9.20E-05	0.00477319
DDIT4	1.288359	0.329721	3.907419	9.33E-05	0.004821324
CCNDBP1	0.281604	0.072423	3.888339	0.000100933	0.005193876
TNFSF4	0.742124	0.190963	3.886221	0.000101817	0.005216793
DPEP3	1.860634	0.478944	3.88487	0.000102385	0.005223378
RGS3	0.84847	0.219478	3.865852	0.000110702	0.005623579

NEURL2	1.574146	0.407316	3.864683	0.000111234	0.005626527
TP53I11	-0.705275	0.182836	-3.857421	0.00011459	0.005771739
ATP13A2	0.709937	0.184337	3.851304	0.00011749	0.00589286
ENSG00000233038	4.054184	1.053725	3.847478	0.00011934	NA
WDR45	0.296611	0.077112	3.846503	0.000119816	0.005984249
ATP11A	-1.066278	0.278027	-3.835157	0.000125484	0.006241133
ZFP36L2	-0.6403	0.16724	-3.828631	0.000128858	0.00638222
ULK1	0.699476	0.183189	3.818336	0.000134355	0.006626868
ZCCHC7	0.362161	0.094948	3.814312	0.000136563	0.006707955
MACROD2	-0.873483	0.229806	-3.800954	0.00014414	0.007050996
ATF7IP2	0.510117	0.134535	3.791718	0.000149609	0.007288512
PWP1	0.298863	0.078848	3.790366	0.000150425	0.007298392
CR2	1.175512	0.310321	3.788057	0.00015183	0.007336612
P2RY8	0.42613	0.112742	3.779678	0.000157032	0.007557223
CCR7	0.414306	0.109779	3.774016	0.000160641	0.007699739
ZBED2	-1.220586	0.323577	-3.772169	0.000161835	0.007725824
SSR2	-0.294638	0.078214	-3.767091	0.000165161	0.00785307
LMAN1	0.359306	0.095471	3.763519	0.000167539	0.007934415
ENSG00000278158	-0.417806	0.111101	-3.760587	0.000169515	0.007973008
ARHGEF1	0.420163	0.111736	3.760321	0.000169696	0.007973008
RHOA	0.250468	0.066837	3.747457	0.000178636	0.008360038
RPL12	-0.591133	0.157959	-3.742307	0.000182338	0.008499827
TMEM65	0.663069	0.177567	3.73418	0.000188327	0.008711839
SNX18	0.785195	0.210274	3.734147	0.000188352	0.008711839
ATP2B1	-0.324333	0.086918	-3.731488	0.000190352	0.008770229
PSAP	0.249245	0.066958	3.722401	0.000197337	0.009052631
EIF3E	-0.308708	0.082951	-3.721548	0.000198005	0.009052631
PFDN5	-0.22885	0.061639	-3.712768	0.000205005	0.009336757
FTH1	-0.250061	0.06744	-3.707917	0.000208971	0.009481078
SIDT1	-0.537254	0.145023	-3.704609	0.000211717	0.009569117
ST13	-0.24678	0.066734	-3.697962	0.000217337	0.00978595
ZNF830	0.339915	0.091946	3.696909	0.000218241	0.009789542
ETFRF1	0.445182	0.120517	3.693922	0.000220821	0.009868054
SNRPB2	0.259412	0.070352	3.687325	0.000226623	0.010089411
SLC25A5	-0.303777	0.08243	-3.685273	0.000228458	0.010133128
PDCD4	0.704872	0.191356	3.683553	0.000230005	0.010163833
REL	-0.442769	0.120399	-3.677517	0.000235515	0.010368765
RGS1	-1.571377	0.42802	-3.671268	0.00024135	0.010586459
PXK	0.474109	0.129178	3.67019	0.00024237	0.010592124
MAP3K1	0.392712	0.107101	3.666732	0.00024567	0.010697011
GPER1	-2.612246	0.713151	-3.66296	0.000249317	0.010774617
HLA.DPB1	-0.51473	0.140525	-3.662913	0.000249363	0.010774617
CLCN7	0.497676	0.1359	3.662084	0.000250172	0.010774617
LYRM9	-0.617622	0.168779	-3.659348	0.000252858	0.010850989
TAGLN2	0.255376	0.069996	3.648445	0.000263832	0.011281207
AHRR	-2.236515	0.613565	-3.645113	0.000267274	0.011364525
CD79A	0.474686	0.13024	3.64471	0.000267693	0.011364525

SLA	0.468441	0.129454	3.618602	0.000296198	0.012529928
ENSG00000237513	-0.641704	0.178684	-3.591286	0.00032905	0.013870266
MAN2A2	-0.696302	0.194421	-3.581414	0.00034174	0.014354298
RPL10	-0.456714	0.12773	-3.575612	0.000349409	0.014624733
IER2	0.239844	0.067111	3.573859	0.000351759	0.014671424
NFKBIA	0.283904	0.079462	3.572837	0.000353135	0.014677328
ENSG00000251364	1.376933	0.385592	3.570954	0.000355684	0.01469197
SDE2	0.667279	0.186917	3.569922	0.000357088	0.01469197
TBC1D9	0.603621	0.169123	3.569133	0.000358164	0.01469197
PISD	0.529688	0.148416	3.568938	0.000358431	0.01469197
SRP14	0.13772	0.038664	3.561922	0.000368149	0.015038451
VSIG10L	2.0132	0.566504	3.553725	0.000379816	0.015461874
MDK	0.8345	0.234977	3.551414	0.000383168	0.015545101
SUSD3	-0.728447	0.205927	-3.537413	0.000404067	0.016337235
ENSG00000131408	0.245936	0.069705	3.528225	0.000418356	0.016857627
BATF2	1.774538	0.503394	3.52515	0.000423243	0.016996908
WDR11	0.353432	0.100286	3.524254	0.000424677	0.016997098
LPP	-0.51529	0.146475	-3.517929	0.000434928	0.017348966
UXT	-0.205126	0.058496	-3.506633	0.000453815	0.018041787
TSG101	0.382985	0.109899	3.484884	0.000492351	0.019508582
MYADM	0.517799	0.148705	3.482052	0.000497586	0.019643464
CD44	-0.329777	0.094742	-3.480803	0.000499912	0.019643464
ECHS1	0.381139	0.109511	3.480375	0.000500713	0.019643464
TOP1MT	-0.638538	0.183694	-3.476103	0.000508757	0.019870504
OTUD7A	1.758607	0.505997	3.475531	0.000509843	0.019870504
PXDC1	1.747081	0.503367	3.470791	0.000518928	0.020158468
AFF3	-0.348175	0.100367	-3.469029	0.000522342	0.020225028
EML2	0.532355	0.153551	3.466961	0.000526378	0.020282028
GTF2B	0.457466	0.131989	3.465927	0.000528407	0.020282028
ST6GAL1	-0.390586	0.112735	-3.464629	0.000530964	0.020282028
MTSS1	-0.418186	0.120703	-3.464577	0.000531066	0.020282028
TAF12	0.29561	0.08534	3.46393	0.000532346	0.020282028
DNAJC7	0.267624	0.077343	3.46023	0.000539715	0.020497091
ACP5	1.041597	0.301122	3.459054	0.000542076	0.020521193
CHCHD10	1.556237	0.450444	3.454896	0.000550505	0.020774134
FCGRT	-0.462273	0.13388	-3.452877	0.000554642	0.020864005
DPH5	-0.409872	0.1188	-3.450115	0.000560349	0.021012186
FCER2	1.437275	0.41746	3.442901	0.00057551	0.021512848
C1orf162	-0.270686	0.078776	-3.436123	0.000590102	0.021989174
SIGLEC5	1.368259	0.399086	3.428478	0.000606975	0.022547223
OLA1	-0.352954	0.10325	-3.41846	0.000629765	0.023320937
TMEM134	-0.288398	0.0844	-3.417047	0.000633044	0.023369531
DTX4	0.640149	0.187533	3.413535	0.00064126	0.023599568
ENTPD1	-0.724426	0.212318	-3.411982	0.000644923	0.023610967
ENSG00000089127	0.555286	0.162758	3.411721	0.000645543	0.023610967
TMEM59	0.206948	0.060705	3.409096	0.000651785	0.023766176
FAU	-0.251635	0.073846	-3.407573	0.000655434	0.023826113

C8orf58	-0.819553	0.240834	-3.402979	0.000666554	0.02415648
STK17A	0.362104	0.106459	3.401344	0.000670554	0.024227586
COA1	0.415377	0.122158	3.400325	0.000673057	0.024244336
SLC25A19	0.422716	0.124392	3.398246	0.000678194	0.024355554
CXXC5	-0.374829	0.110388	-3.395559	0.000684885	0.024521783
FAM129C	-0.36877	0.108753	-3.390899	0.000696636	0.02486762
NAP1L1	-0.357927	0.105596	-3.389575	0.000700011	0.024913274
LARGE1	1.432508	0.424098	3.37778	0.000730735	0.025845512
RAB7A	0.267524	0.079218	3.37707	0.000732624	0.025845512
ZNF581	-0.404375	0.119765	-3.376417	0.000734365	0.025845512
CARHSP1	0.251882	0.074605	3.376216	0.000734902	0.025845512
NUBP1	0.343553	0.101803	3.374681	0.000739012	0.025913379
EMC6	-0.312578	0.092802	-3.36822	0.000756552	0.026450407
RAB37	0.699093	0.207711	3.365706	0.00076348	0.026603435
CYBA	0.28184	0.083756	3.365011	0.000765405	0.026603435
MRPL39	0.407429	0.121244	3.360393	0.000778316	0.02697329
ZNF337.AS1	0.908178	0.270832	3.353289	0.000798574	0.027594895
NDUFA2	0.249105	0.07439	3.348648	0.000812069	0.027979891
TMEM159	-0.527198	0.157798	-3.340962	0.000834888	0.028682987
CCNI	-0.21217	0.063589	-3.336587	0.000848139	0.029054244
ZNF280B	-1.187887	0.357429	-3.323418	0.000889217	0.030373904
POLM	0.364002	0.109771	3.316008	0.000913132	0.031101436
NCOA4	0.338916	0.102242	3.314826	0.000917001	0.031143967
ENSG00000225489	1.196544	0.361518	3.309775	0.000933709	0.031621069
GFOD1	-0.749875	0.226689	-3.307943	0.00093984	0.031738287
AP3S1	0.502581	0.152018	3.306065	0.000946161	0.031861238
ALOX5	0.323351	0.09785	3.30457	0.000951221	0.03194115
TBL1X	-0.531599	0.161086	-3.300086	0.000966552	0.032364511
ETFB	0.392031	0.118828	3.299151	0.000969779	0.032381355
SNU13	0.160653	0.048714	3.297906	0.000974089	0.032434163
PPP3CA	-0.250518	0.076008	-3.29592	0.000980999	0.032573013
LARGE2	-0.453981	0.137818	-3.294052	0.000987543	0.032644875
KEAP1	0.539933	0.163927	3.293735	0.000988656	0.032644875
NEIL1	-0.868306	0.263767	-3.29194	0.000994989	0.032762975
HERC2	-0.449128	0.136529	-3.289622	0.001003219	0.032942715
CD22	0.524729	0.159597	3.287841	0.001009588	0.032969805
PCNA	0.46281	0.140764	3.28784	0.001009591	0.032969805
CD47	-0.272887	0.083387	-3.272539	0.001065863	0.034712088
MRPS22	0.349795	0.107271	3.260857	0.001110761	0.036075442
HERPUD1	-0.300123	0.092135	-3.257419	0.001124304	0.036415821
UBE2D1	0.303751	0.093443	3.250669	0.001151338	0.037115388
NPIPB11	-1.576177	0.484907	-3.250469	0.001152148	0.037115388
MMP7	1.794069	0.552361	3.248	0.001162192	0.037337763
RPL3	-0.379696	0.11703	-3.244423	0.001176887	0.037632703
CD37	-0.261224	0.08052	-3.244226	0.001177704	0.037632703
RACK1	-0.392914	0.121211	-3.241571	0.001188728	0.037883128
LMNA	-1.581539	0.488632	-3.236667	0.001209346	0.038390519

EXOSC5	0.471437	0.145703	3.235604	0.001213855	0.038390519
EHD4	-0.396358	0.122503	-3.235491	0.001214338	0.038390519
CYTH1	0.205042	0.063414	3.233366	0.001223409	0.038574695
HIGD1A	0.386785	0.1197	3.231302	0.001232275	0.038751454
PIM1	-0.355713	0.11012	-3.230214	0.001236977	0.038796686
LINC02202	-2.359982	0.73082	-3.229223	0.00124127	NA
GPR155	-0.766562	0.237664	-3.225395	0.001257991	0.039351949
WDR92	-0.604733	0.187594	-3.223631	0.001265766	0.03949122
ERP29	-0.263559	0.081811	-3.221568	0.001274912	0.039672443
PSD3	-0.838979	0.260795	-3.217013	0.001295329	0.040202557
DMXL1	-0.373384	0.116384	-3.208202	0.001335677	0.041346851
POLR2G	0.337568	0.105353	3.204154	0.001354598	0.041823647
ENSG00000188206	-0.420189	0.131245	-3.201568	0.001366816	0.042091561
PTP4A3	1.569688	0.490933	3.197357	0.001386933	0.042600708
BCL2L11	-0.701156	0.219591	-3.193014	0.00140796	0.043135111
RPL11	-0.280579	0.087966	-3.189617	0.001424615	0.043533152
GABARAPL2	0.179016	0.056207	3.184928	0.001447901	0.044114224
SNN	0.587842	0.184607	3.184299	0.001451053	0.044114224
CAMK2D	-0.328817	0.103534	-3.17592	0.001493622	0.045155277
COQ8A	0.4684	0.147504	3.17551	0.001495734	0.045155277
PTK2B	0.424935	0.133824	3.175324	0.001496692	0.045155277
TCF4	-0.249942	0.078851	-3.1698	0.001525439	0.045906053
TERF2IP	0.265009	0.083764	3.163776	0.001557368	0.046725143
C1orf131	-0.3944	0.124685	-3.163187	0.001560518	0.046725143
PCBP1	-0.208612	0.066016	-3.160031	0.001577521	0.046975543
DALRD3	-0.318211	0.10071	-3.159681	0.001579421	0.046975543
RAPGEF5	-1.731895	0.548166	-3.159438	0.001580737	0.046975543
CRYL1	-0.954199	0.302314	-3.156318	0.001597744	0.047362542
NUP88	0.305764	0.097027	3.151327	0.001625305	0.048059705
BPTF	-0.25094	0.079757	-3.146319	0.001653395	0.048768996
RPS9	-0.562421	0.178848	-3.144686	0.001662654	0.048920706
IMPA1	0.352955	0.112379	3.140751	0.001685151	0.049460221
MIDN	0.395264	0.125895	3.13962	0.00169167	0.04952925
MYBL2	-1.827875	0.582548	-3.137723	0.001702658	0.049728497
TXLNG	0.488621	0.155891	3.134369	0.001722239	0.050152605
ENSG00000270022	1.343041	0.428567	3.133794	0.001725618	0.050152605
SLC25A6	-0.366016	0.116939	-3.129967	0.001748257	0.050686651
SPTAN1	-0.328742	0.105085	-3.128343	0.001757949	0.050843649
TNFRSF18	-0.909015	0.290709	-3.126896	0.001766622	0.050892908
CTSA	0.44194	0.141347	3.126631	0.001768215	0.050892908
PRDM2	-0.313101	0.100261	-3.12285	0.00179109	0.05142677
OXA1L	-0.256881	0.082285	-3.121834	0.001797281	0.051480184
EFCAB5	0.606405	0.195011	3.109591	0.001873468	0.053533444
QPRT	1.607041	0.516981	3.108508	0.001880344	0.053601091
NEDD9	0.47518	0.152925	3.107272	0.001888228	0.053697062
CHPT1	-0.457603	0.147309	-3.106414	0.001893711	0.053724446
P4HTM	-0.466559	0.150431	-3.101475	0.001925589	0.054498757

SRD5A1	-0.495908	0.16021	-3.095362	0.001965726	0.055502567
RAP2B	-0.490272	0.158447	-3.09423	0.001973247	0.055582909
RPL29	-0.286758	0.092809	-3.089751	0.002003247	0.056294554
RASA2	-0.401102	0.129947	-3.086656	0.002024218	0.056617672
ELOA	0.3405	0.110314	3.086648	0.002024271	0.056617672
WARS	1.084846	0.351558	3.085829	0.002029856	0.05664061
AGO2	-0.329806	0.106914	-3.08479	0.002036959	0.056705684
MEF2A	0.416359	0.135034	3.083354	0.002046815	0.056787286
FKBP2	0.262469	0.085135	3.082972	0.002049444	0.056787286
ENSG00000272800	-3.001255	0.975002	-3.078204	0.002082526	NA
BTG2	-0.310344	0.100823	-3.078109	0.002083188	0.057588048
DHRS3	3.509207	1.140687	3.076397	0.002095189	0.057707179
CYBC1	-0.26465	0.086047	-3.075636	0.002100539	0.057707179
IL1B	2.475638	0.804975	3.07542	0.002102062	0.057707179
METTL7A	0.320272	0.104199	3.073662	0.00211449	0.057894003
LIPA	0.34807	0.113283	3.072563	0.002122293	0.057894003
SPATA2	0.605993	0.197281	3.071729	0.002128229	0.057894003
RPL22L1	-0.337668	0.109928	-3.071712	0.002128349	0.057894003
LYN	-0.292462	0.09527	-3.069826	0.002141835	0.058127825
NSL1	0.273961	0.089267	3.069	0.002147766	0.058156016
NIPAL4	0.903536	0.29455	3.067514	0.002158475	0.058313152
ING1	0.398925	0.13022	3.063478	0.002187801	0.058971419
RPS23	-0.27427	0.089564	-3.062289	0.00219651	0.059053826
VPS51	-0.316043	0.103224	-3.061706	0.002200795	0.059053826
UBA52	-0.19416	0.063454	-3.059841	0.002214544	0.059288941
NSUN2	0.427026	0.139759	3.055451	0.002247221	0.060028577
TOM1	0.420389	0.137767	3.051459	0.002277318	0.060696138
ASCC1	0.509801	0.167112	3.050664	0.002283357	0.060720952
EIF3F	-0.321336	0.105387	-3.049115	0.002295165	0.060898726
EMG1	0.263463	0.086429	3.048307	0.002301345	0.060926686
IQCD	0.711534	0.233547	3.046646	0.0023141	0.061128225
LINC01588	-0.364961	0.119836	-3.04551	0.002322863	0.061223674
KCNC3	0.706594	0.232312	3.041571	0.002353468	0.061893084
EVI2B	0.200371	0.065923	3.039487	0.002369816	0.062185431
FEM1A	0.349582	0.115058	3.038314	0.002379062	0.062290552
WDR12	0.515445	0.16991	3.03363	0.002416304	0.063126606
DPM2	-0.257888	0.08515	-3.028648	0.002456509	0.064036227
RASAL1	-2.291322	0.756794	-3.027669	0.002464477	NA
SLC1A4	-0.89242	0.295217	-3.022929	0.002503411	0.065031676
MRPS14	0.24243	0.080204	3.02266	0.002505637	0.065031676
GEN1	-0.718159	0.237654	-3.021869	0.002512196	0.065059855
SHISAL2A	0.346368	0.114695	3.019903	0.002528557	0.065341207
TARS2	0.496246	0.164396	3.018596	0.002539486	0.065481286
IQGAP1	-0.271574	0.089992	-3.017768	0.002546434	0.065518324
SC5D	-0.4982	0.165192	-3.015878	0.002562367	0.065540282
FKBP5	0.730085	0.242113	3.015472	0.002565795	0.065540282
TIMP1	-0.521446	0.172938	-3.015211	0.002568001	0.065540282

MICU2	0.31925	0.105885	3.015053	0.002569342	0.065540282
GNG2	0.383926	0.127466	3.011996	0.002595361	0.066062221
TSTD1	-0.325043	0.108011	-3.00935	0.00261807	0.066497865
CCDC50	-0.329307	0.109481	-3.007908	0.002630525	0.066671743
ECH1	0.265036	0.088229	3.003935	0.002665124	0.067302502
LPIN1	-0.650132	0.216461	-3.003463	0.002669257	0.067302502
RPL34	-0.332353	0.11067	-3.003106	0.002672397	0.067302502
AKR1B1	0.243817	0.081282	2.999627	0.002703104	0.067871892
ENSG00000166928	-1.946723	0.649069	-2.999253	0.002706425	0.067871892
BUD31	0.206981	0.069028	2.998509	0.002713037	0.067894473
WDFY4	-0.373188	0.124496	-2.997597	0.002721173	0.067955013
CCDC86	0.615744	0.205573	2.995266	0.002742058	0.068333001
SLAMF7	-1.020792	0.341101	-2.992637	0.002765783	0.068670447
VBP1	0.239724	0.080122	2.99199	0.00277165	0.068670447
TNFRSF13B	-1.091315	0.364763	-2.991849	0.00277293	0.068670447
CMTM7	0.425682	0.142442	2.988468	0.002803802	0.069290631
SERPINE2	0.871463	0.291881	2.985679	0.002829498	0.069780593
GSTK1	-0.24018	0.080572	-2.980949	0.002873569	0.070720726
ENSG00000271857	3.285174	1.104337	2.974794	0.002931858	NA
HSD17B11	-0.233257	0.07843	-2.974065	0.00293883	0.072177419
TNFRSF13C	-0.293122	0.098598	-2.972904	0.002949971	0.072301662
IL2RA	-0.987686	0.33249	-2.970573	0.002972449	0.072702671
GSTP1	0.255974	0.086271	2.967088	0.003006345	0.073380753
LSM2	-0.24628	0.08306	-2.965066	0.003026179	0.073686507
SNW1	0.286778	0.096744	2.964313	0.003033595	0.073686507
NME4	-0.574872	0.193981	-2.963555	0.003041076	0.073686507
BCCIP	0.32558	0.109871	2.963293	0.003043667	0.073686507
HOXB3	1.886742	0.637197	2.961001	0.003066407	0.074086142
EIF6	0.209246	0.070696	2.959812	0.003078267	0.074221825
LAIR1	0.695603	0.235313	2.956078	0.003115782	0.074974296
EIF4B	-0.246113	0.083297	-2.954647	0.003130265	0.075170622
SYNE3	-0.47506	0.160836	-2.953689	0.00314	0.075252387
LDHB	-0.247669	0.083883	-2.952531	0.003151806	0.075383343
TRIM25	0.518148	0.175691	2.949207	0.003185901	0.076045802
CHCHD6	-0.701889	0.238191	-2.946745	0.003211383	0.076500424
HIVEP2	0.519871	0.176513	2.945223	0.003227221	0.076723944
PPIF	-0.547311	0.185878	-2.944471	0.003235075	0.076757158
RPS18	-0.30767	0.104552	-2.942743	0.003253179	0.07703294
SPA17	1.700093	0.578064	2.941009	0.003271445	0.07716779
DNAJA2	0.263263	0.089528	2.940584	0.003275946	0.07716779
RIMKLB	-0.458217	0.155838	-2.940341	0.003278516	0.07716779
NEK3	-1.524734	0.518662	-2.939743	0.003284841	0.07716779
ARL4C	-0.68056	0.23167	-2.937627	0.003307344	0.077543188
IGBP1	-0.355638	0.121191	-2.934522	0.003340614	0.078169062
ADD1	0.290829	0.099134	2.933687	0.003349613	0.078225644
STAT4	-0.718723	0.245243	-2.930654	0.003382498	0.07879336
RPS28	-0.271987	0.092821	-2.930224	0.00338718	0.07879336

HGSNAT	-0.593403	0.202716	-2.927259	0.003419639	0.079364194
EPHB6	-0.504089	0.172234	-2.926765	0.003425072	0.079364194
NDUFB3	0.228263	0.078024	2.925537	0.003438628	0.079464836
DUSP4	-1.714408	0.58609	-2.925161	0.003442785	0.079464836
RPL19	-0.219826	0.075184	-2.923819	0.003457654	0.079653365
PLD4	-0.896546	0.306795	-2.922293	0.003474641	0.079889868
ZDHHC21	-0.508914	0.174349	-2.918943	0.003512207	0.080487988
UTP3	0.30794	0.105503	2.918766	0.003514198	0.080487988
ATF4	0.294738	0.101014	2.917809	0.003524997	0.080580086
PDE3B	-0.76197	0.261281	-2.916283	0.003542295	0.08082008
TOB1	-0.435213	0.149383	-2.913411	0.00357504	0.08136333
CPEB2	-1.273	0.437074	-2.912549	0.003584923	0.08136333
WBP2	0.330261	0.113398	2.912399	0.00358664	0.08136333
GNAZ	-0.894725	0.307281	-2.911746	0.003594143	0.081378247
ENSG00000274213	0.999664	0.343473	2.910457	0.003609006	0.08155943
CAMK2G	-0.409461	0.140948	-2.905045	0.003672001	0.082825562
PSMB4	0.282266	0.09722	2.90338	0.00369158	0.083109491
TMEM140	0.538252	0.185453	2.902365	0.003703571	0.083221821
SLC35B2	-0.499909	0.172302	-2.901348	0.003715613	0.083257706
TMEM175	0.615363	0.212118	2.901047	0.003719176	0.083257706
UBE2T	0.507186	0.174946	2.899107	0.003742271	0.083559092
TAF7	0.177683	0.061297	2.898736	0.003746698	0.083559092
SSBP1	0.184829	0.063903	2.892352	0.003823692	0.085116527
C14orf28	-0.444455	0.153756	-2.890645	0.003844526	0.085420342
ABHD14B	-0.249603	0.086421	-2.88823	0.003874164	0.085755467
ZDHHC23	-0.67241	0.232829	-2.887999	0.003877012	0.085755467
ENSG00000259436	-0.460383	0.159431	-2.887655	0.003881252	0.085755467
ENSA	0.184622	0.063972	2.885982	0.003901945	0.085940265
FMNL3	0.395544	0.137065	2.88581	0.003904075	0.085940265
RDH10	2.048292	0.710157	2.884282	0.003923072	NA
COX6A1	0.208533	0.072358	2.881954	0.003952172	0.086838214
COMMD6	-0.161615	0.056107	-2.880502	0.003970423	0.086972109
GADD45GIP1	0.201776	0.070054	2.880306	0.003972899	0.086972109
SNRPC	0.224752	0.078232	2.872876	0.004067537	0.088880159
DDX21	0.228829	0.079729	2.87007	0.00410381	0.089360257
IRF1	0.329123	0.114687	2.869756	0.004107887	0.089360257
FLOT1	0.27693	0.09652	2.86914	0.004115892	0.089360257
SPCS2	0.188049	0.065548	2.868857	0.004119578	0.089360257
CMC2	0.301254	0.105059	2.867484	0.004137492	0.089585377
RABAC1	0.220316	0.076996	2.861404	0.004217691	0.090999671
TMEM135	0.944355	0.330036	2.861372	0.004218122	0.090999671
TACO1	0.392032	0.137146	2.858499	0.004256511	0.091513746
POP4	0.288604	0.100966	2.858436	0.004257348	0.091513746
CD5	-1.085725	0.380142	-2.8561	0.004288804	0.091922953
CHEK1	-1.29734	0.454271	-2.855874	0.004291852	0.091922953
HIVEP3	0.774619	0.27137	2.854472	0.004310843	0.092163645
GPANK1	0.248032	0.087011	2.850594	0.004363772	0.093008659

SMS	0.269483	0.094547	2.850244	0.004368567	0.093008659
HSPA8	-0.368022	0.129137	-2.849861	0.00437384	0.093008659
EEF1E1	0.274095	0.096366	2.844306	0.004450828	0.094476782
FBXO15	-0.897055	0.315792	-2.840652	0.004502137	0.095395546
CLYBL	-0.751729	0.264708	-2.839837	0.004513665	0.095469641
CEP170	0.339907	0.119745	2.838601	0.004531176	0.095669775
KLHL14	-0.525911	0.185549	-2.834354	0.00459184	0.096778719
SDHD	0.246952	0.087148	2.833714	0.004601049	0.096801185
PABPN1	-0.222454	0.078551	-2.831963	0.004626324	0.097160976
CEP68	-0.372755	0.131661	-2.831177	0.004637704	0.097228203
GTDC1	-0.648396	0.229187	-2.829118	0.004667656	0.097683841
RPL32	-0.265698	0.093953	-2.827988	0.004684152	0.0978568
IKZF2	-0.706443	0.24988	-2.827125	0.004696802	0.097948928
SMIM20	0.341121	0.120711	2.825941	0.004714193	0.098139435
RHOBTB2	-1.200366	0.425214	-2.82297	0.004758098	0.098880272
C12orf42	-0.483092	0.171175	-2.822211	0.004769375	0.098937553
RPL30	-0.237906	0.084325	-2.821294	0.004783041	0.098937553
ENSG00000230709	-0.943923	0.334613	-2.820938	0.004788344	0.098937553
MADCAM1	-1.121647	0.39767	-2.82055	0.004794147	0.098937553
HSBP1	0.246655	0.087535	2.817777	0.004835736	0.099622867
ENSG00000277511	-0.544062	0.193253	-2.815283	0.004873432	0.100225764
FDFT1	0.305941	0.108705	2.814413	0.004886638	0.100323771
PRDX6	0.209221	0.074399	2.812146	0.004921217	0.100730989
ENO1	0.309995	0.110299	2.810501	0.004946441	0.100730989
REM2	0.870621	0.309819	2.810095	0.004952686	0.100730989
ENSG00000253535	0.722683	0.257188	2.809943	0.004955034	0.100730989
TECPR1	0.567744	0.202058	2.809803	0.004957181	0.100730989
RPL41	-0.246876	0.087872	-2.809493	0.004961962	0.100730989
ITGAE	0.277516	0.098787	2.809245	0.004965791	0.100730989
ZNF785	0.400138	0.142801	2.80207	0.005077584	0.10282324
NFATC3	0.326743	0.116637	2.801377	0.005088502	0.102841123
RPS6	-0.296592	0.105898	-2.800721	0.005098855	0.102841123
ACP1	0.173264	0.061872	2.800369	0.005104422	0.102841123
HHIP.AS1	-2.005427	0.716714	-2.798084	0.005140679	NA
PADI4	-1.219189	0.436038	-2.796062	0.005172944	0.104045314
SCAF4	-0.283876	0.101572	-2.794816	0.005192926	0.104084557
SHQ1	0.599835	0.21463	2.794738	0.005194185	0.104084557
GPR18	0.588343	0.210551	2.794304	0.005201163	0.104084557
PDIA3	0.309279	0.110732	2.793039	0.005221546	0.104316828
MOSPD1	0.465103	0.166656	2.790799	0.005257807	0.104760154
UBB	0.261478	0.0937	2.79058	0.005261362	0.104760154
POU2F2	-0.20246	0.072612	-2.788236	0.005299593	0.105238241
ARHGAP27	0.362506	0.130023	2.788023	0.00530308	0.105238241
ATF3	0.723982	0.25978	2.7869	0.005321497	0.105427719
GPR137	0.227145	0.081523	2.786248	0.005332198	0.105463957
ENSG00000276136	-0.752841	0.270413	-2.784046	0.005368547	0.106006506
EPB41L4A.AS1	-0.226878	0.081511	-2.783416	0.005378987	0.10603651

ZMYM3	-0.526635	0.189312	-2.781834	0.005405273	0.106378279
TNIP1	0.225298	0.081109	2.777741	0.005473831	0.107549474
TXNDC12	0.254275	0.091776	2.770611	0.005595115	0.109751037
ANXA7	0.294487	0.106367	2.768596	0.005629842	0.110250303
HNRNPC	0.217481	0.078618	2.766286	0.005669883	0.110851798
DAD1	0.227143	0.082151	2.76496	0.005692974	0.111012271
SLFN11	-0.716388	0.259116	-2.764743	0.005696768	0.111012271
TTC39C	-0.557497	0.201724	-2.763657	0.005715766	0.111200181
ZW10	0.640998	0.232029	2.762584	0.005734586	0.111308981
PLEK	1.002769	0.363024	2.762271	0.005740086	0.111308981
ANKRD37	0.476764	0.172778	2.759402	0.005790721	0.112107979
ZNF652	-0.381399	0.138285	-2.758072	0.005814337	0.112284752
TSPAN14	-0.349589	0.126763	-2.757824	0.005818744	0.112284752
ITGB2	0.642127	0.233004	2.75587	0.005853624	0.11277476
RSRC1	-0.260374	0.094573	-2.753162	0.005902264	0.113173044
KATNBL1	-0.26527	0.096354	-2.753092	0.005903529	0.113173044
ENSG00000274184	-0.680999	0.247441	-2.752166	0.005920246	0.113173044
KBTBD6	-0.400964	0.14572	-2.751606	0.005930376	0.113173044
ENSG00000235609	1.456059	0.529191	2.751482	0.005932631	0.113173044
RPL38	-0.233734	0.084959	-2.751125	0.005939103	0.113173044
EBLN2	0.43511	0.158186	2.750627	0.00594814	0.113173044
FBXO11	-0.358919	0.130492	-2.750499	0.005950463	0.113173044
TEX264	0.252923	0.092012	2.748808	0.005981247	0.113479027
CWC15	0.212266	0.077228	2.748567	0.005985644	0.113479027
LGALS3	-0.64217	0.233757	-2.747168	0.006011236	0.113571799
NECAP2	0.202153	0.073589	2.74705	0.006013393	0.113571799
HNRNPH3	0.212001	0.077183	2.746734	0.0060192	0.113571799
HMGN1	-0.191737	0.069866	-2.744342	0.006063239	0.114221439
CD83	-0.200914	0.073228	-2.743665	0.006075754	0.114276095
SPIDR	-0.438614	0.159942	-2.742336	0.006100395	0.114558288
FANCG	-0.667994	0.24385	-2.739369	0.006155723	0.115414954
CAMKMT	-0.504749	0.184324	-2.738384	0.006174187	0.11551968
MAGOH	0.211515	0.07726	2.737703	0.006186996	0.11551968
MDH1	0.315649	0.115305	2.737519	0.006190463	0.11551968
ARHGEF40	-1.997523	0.729908	-2.736679	0.00620628	NA
IL6	-1.471908	0.538368	-2.734018	0.006256656	0.116376659
SERPINB1	0.316514	0.115782	2.733708	0.006262564	0.116376659
PIM2	-0.208933	0.076437	-2.733393	0.006268547	0.116376659
ZNF277	-0.331538	0.121337	-2.732384	0.006287781	0.116376659
LRPAP1	0.246572	0.090253	2.732	0.006295108	0.116376659
ZMIZ1	-0.976177	0.357401	-2.731318	0.006308154	0.116376659
RNF41	-0.223343	0.081773	-2.731242	0.006309612	0.116376659
NFKBIB	0.331435	0.121361	2.730976	0.006314709	0.116376659
RNF167	0.222799	0.081708	2.726775	0.006395664	0.117686157
LTA4H	-0.303548	0.11138	-2.725333	0.006423666	0.118018736
CKLF	-0.471538	0.173067	-2.724589	0.00643816	0.118102486
MRM2	0.262039	0.096216	2.723449	0.006460416	0.118328148

MAN1B1	-0.391724	0.143944	-2.72137	0.006501193	0.1187172
EEF1D	-0.266816	0.098046	-2.721348	0.006501632	0.1187172
IRF4	0.425386	0.156661	2.715336	0.006620859	0.120397354
PDIA6	0.253323	0.093295	2.715297	0.006621632	0.120397354
SEC61B	-0.272077	0.100206	-2.715177	0.006624032	0.120397354
SNRPB	0.211932	0.07811	2.713262	0.00666245	0.120910748
TRIM33	-0.302707	0.111688	-2.710296	0.006722325	0.121811402
BANK1	-0.337276	0.124513	-2.708763	0.006753456	0.122189241
PSME1	0.152106	0.056247	2.704242	0.006846037	0.123676051
SAP18	0.167265	0.061867	2.703597	0.006859352	0.123728559
ZKSCAN1	-0.253914	0.093974	-2.70195	0.006893404	0.124154389
PRPF19	0.38301	0.141788	2.701287	0.006907177	0.124214232
SERPINB6	0.450539	0.166836	2.700484	0.006923872	0.124326384
TANK	0.177057	0.065583	2.699751	0.006939129	0.124412404
ENSG00000176320	2.490283	0.922711	2.698877	0.00695739	NA
CCDC28A	0.242878	0.090062	2.696794	0.007001047	0.125333494
IK	0.205563	0.076313	2.69368	0.007066793	0.126320259
PTBP1	0.231591	0.08604	2.691678	0.007109357	0.126890273
TSC22D1	-0.524518	0.194923	-2.690901	0.007125928	0.126995355
MSI2	-0.335271	0.124621	-2.690319	0.007138383	0.127026875
TTC21A	-0.384677	0.143181	-2.686648	0.007217291	0.128239077
FCHSD2	-0.373494	0.139045	-2.686131	0.007228474	0.128246068
RASGRF1	-1.522952	0.567407	-2.684055	0.007273514	0.128736724
GRAP	0.457636	0.170514	2.683859	0.007277789	0.128736724
CENPT	-0.321645	0.119946	-2.681587	0.00732739	0.129361403
EIF3L	-0.347213	0.129497	-2.681245	0.007334869	0.129361403
EIF3H	-0.225828	0.08428	-2.679484	0.007373566	0.129851236
FBXO28	0.410914	0.153526	2.676521	0.007439087	0.130694141
ESR2	-0.792838	0.296241	-2.676326	0.00744342	0.130694141
HIST1H4H	0.597245	0.22326	2.675113	0.00747041	0.130940296
CGAS	0.335266	0.125347	2.674706	0.00747947	0.130940296
DHRS1	-0.304448	0.113904	-2.672848	0.007521038	0.131421773
MYL2	2.924506	1.094339	2.672395	0.007531189	NA
CNIH4	0.259232	0.097029	2.67169	0.007547029	0.131421773
ZNF506	0.361326	0.135249	2.671557	0.007550024	0.131421773
GPR132	0.362305	0.135618	2.671505	0.007551196	0.131421773
UBXN1	-0.249598	0.093475	-2.67022	0.007580159	0.131732965
CRIPT	0.281271	0.105371	2.669327	0.007600331	0.131796502
NUP62	0.288274	0.108005	2.669078	0.00760599	0.131796502
IGHA1	-0.458329	0.171769	-2.668289	0.007623865	0.131913957
JAML	-2.462621	0.923713	-2.666002	0.007675912	0.132621468
RNF181	0.221979	0.083296	2.664957	0.007699824	0.132841514
INKA1	-0.387966	0.14566	-2.663499	0.007733256	0.13312008
ANAPC10	0.377426	0.141715	2.663277	0.007738368	0.13312008
CCNG1	-0.219009	0.082354	-2.659357	0.007828985	0.134484312
CXCR5	0.265282	0.099821	2.657567	0.007870699	0.134792798
FLNA	-0.315869	0.118858	-2.657522	0.007871735	0.134792798

PURA	-0.360907	0.135828	-2.657091	0.007881814	0.134792798
LY86	-0.452301	0.170277	-2.656266	0.007901136	0.134792798
MANF	0.301765	0.11361	2.656159	0.007903641	0.134792798
LINC00342	-0.789288	0.297328	-2.654602	0.007940197	0.13522224
RPL36	-0.152903	0.057624	-2.653466	0.007966989	0.1354844
VIM	-0.426281	0.160767	-2.651549	0.008012351	0.13606116
SRSF8	0.245487	0.092634	2.650081	0.008047255	0.136274599
AARS	0.379159	0.143076	2.650056	0.008047848	0.136274599
PABPC1	-0.248246	0.093765	-2.647532	0.008108178	0.137100874
CUL1	0.355378	0.134271	2.646729	0.008127453	0.137231583
RPL13A	-0.253197	0.095718	-2.645243	0.008163226	0.137474913
SF3B6	0.184311	0.069678	2.64517	0.008164994	0.137474913
DAPK2	0.715183	0.270663	2.642338	0.008233585	0.138241999
DNAJA1	0.263185	0.099603	2.642329	0.008233813	0.138241999
ECSIT	0.239639	0.090778	2.63984	0.008294516	0.139064758
DAAM1	-0.511532	0.193904	-2.638072	0.008337892	0.139595103
POLR2C	0.233272	0.088463	2.636946	0.008365617	0.139862287
RPS14	-0.28326	0.107487	-2.635309	0.008406064	0.14030083
PRCD	-0.818879	0.310789	-2.634836	0.008417794	0.14030083
EAPP	0.266378	0.101121	2.634239	0.008432621	0.14030083
ZBTB17	0.425062	0.161376	2.633979	0.008439059	0.14030083
DPP8	0.271766	0.103198	2.633448	0.008452289	0.140324527
LGALS1	-0.71027	0.269871	-2.631892	0.008491097	0.140772196
RIMS3	-1.462898	0.55613	-2.630498	0.008525991	0.141019768
RPL39	-0.240046	0.09126	-2.630348	0.008529756	0.141019768
MKNK2	-0.41495	0.157898	-2.627968	0.008589656	0.14174608
RASGRP1	-0.670919	0.25533	-2.627656	0.008597537	0.14174608
YTHDF2	0.284465	0.108326	2.626019	0.008638997	0.142232345
SLC39A7	0.368201	0.140303	2.624326	0.008682065	0.142681685
DNAJC4	-0.300978	0.114702	-2.624003	0.008690295	0.142681685
DNAAF4	-0.686537	0.261762	-2.622755	0.008722187	0.143007768
MORF4L1	0.14004	0.053429	2.621041	0.008766174	0.143530999
MPHOSPH9	0.388393	0.148304	2.618895	0.008821519	0.144110574
RCBTB1	-0.548472	0.209442	-2.618728	0.008825818	0.144110574
ASB16.AS1	-0.454821	0.17385	-2.616167	0.00889231	0.1449971
LRWD1	0.443358	0.169525	2.615296	0.008915004	0.145168023
PCBP4	-0.576958	0.220665	-2.614635	0.008932281	0.145250369
MED11	0.200789	0.076832	2.613363	0.008965614	0.145593249
PDLIM2	-0.457294	0.17508	-2.611912	0.009003741	0.146012926
MAT2B	0.240793	0.092229	2.610811	0.009032781	0.146284288
XBP1	0.388073	0.148695	2.609862	0.009057877	0.146318104
GOLGB1	-0.226719	0.086872	-2.609801	0.009059487	0.146318104
B4GALT1	-0.464908	0.178261	-2.608023	0.00910669	0.146717936
PSMD4	0.234063	0.089769	2.607379	0.009123818	0.146717936
NDUFAB1	0.179358	0.068794	2.607176	0.00912925	0.146717936
PCDH9	-0.681491	0.261407	-2.607012	0.009133614	0.146717936
MCM3	0.341512	0.131101	2.604956	0.009188595	0.147401931

SLC25A13	-0.481118	0.184776	-2.603782	0.009220128	0.147708443
TOR3A	0.234973	0.090272	2.602954	0.009242444	0.147866671
PSMB3	0.207689	0.079818	2.602027	0.009267455	0.148067528
RDH5	-0.421758	0.162189	-2.600407	0.009311316	0.148568616
FAM133B	0.149455	0.057605	2.594498	0.00947293	0.150944663
CAPRIN2	-0.491516	0.189582	-2.592633	0.009524433	0.151504696
SQSTM1	0.312109	0.120415	2.591946	0.009543462	0.151504696
ABCG1	0.747906	0.288562	2.591844	0.009546313	0.151504696
RPL35	-0.236621	0.091347	-2.59036	0.009587552	0.15195631
AHSA2P	-0.3981	0.153882	-2.587052	0.009680098	0.153218811
NDUFB4	0.148902	0.057589	2.585571	0.009721783	0.153559482
MIR497HG	1.214799	0.469874	2.58537	0.009727458	0.153559482
SLC25A25.AS1	0.602762	0.233248	2.584208	0.009760303	0.153873638
FCHO1	0.531592	0.205781	2.58329	0.009786315	0.15404053
HAX1	0.200348	0.077567	2.58292	0.009796807	0.15404053
GPKOW	0.449726	0.174307	2.580083	0.009877658	0.155106626
TMX1	0.25956	0.100675	2.578188	0.009932004	0.15575426
TAF4B	0.62023	0.240854	2.575129	0.010020266	0.156931366
PPP1R11	0.211273	0.082115	2.572882	0.010085562	0.157495146
PSMC6	0.266102	0.103454	2.572179	0.010106077	0.157495146
CHMP2B	0.232795	0.090517	2.571843	0.010115888	0.157495146
PAXX	0.463056	0.180051	2.571797	0.010117219	0.157495146
SIPA1L1	-0.308187	0.119842	-2.571608	0.010122734	0.157495146
CEP19	0.443037	0.172319	2.571032	0.010139582	0.157495146
SLC39A1	0.226381	0.088062	2.570711	0.01014901	0.157495146
NDUFA4	0.133212	0.051968	2.563367	0.010366242	0.160656479
CHMP1B	0.210397	0.082133	2.561671	0.010417009	0.161233047
RPS21	-0.226139	0.088297	-2.561101	0.010434102	0.161287614
NINJ1	-0.568232	0.222065	-2.558851	0.010501876	0.162124419
TPRA1	0.421008	0.164571	2.558218	0.010521004	0.162209051
CASZ1	-0.638242	0.249593	-2.557131	0.010553957	0.162218692
IKZF3	-0.247823	0.096924	-2.55689	0.010561272	0.162218692
RAB4B	-0.262665	0.10273	-2.556847	0.01056257	0.162218692
ENSG00000245869	0.592735	0.231961	2.555328	0.010608776	0.162718094
UVSSA	-0.981497	0.384245	-2.554355	0.010638459	0.162779798
ARID1B	-0.274844	0.1076	-2.554299	0.010640187	0.162779798
GPT2	0.759581	0.297652	2.551905	0.010713568	0.163691756
TMEM41B	0.336225	0.13187	2.549664	0.010782686	0.164536311
ZNF250	-0.519641	0.20391	-2.548382	0.010822394	0.164780363
GAB2	-0.630348	0.247378	-2.548122	0.010830469	0.164780363
ENSG00000257275	-0.462642	0.181584	-2.547806	0.010840266	0.164780363
ENSG00000238045	0.589099	0.231283	2.547093	0.010862452	0.164906726
FHIT	-0.64913	0.255063	-2.544982	0.010928344	0.165695445
APEX1	-0.244665	0.09616	-2.544343	0.01094834	0.165787162
SORBS3	-0.76852	0.302125	-2.543719	0.010967915	0.165872269
PRDX5	0.214936	0.084521	2.542981	0.010991112	0.166011884
KBTBD7	-0.360935	0.141969	-2.54235	0.011010973	0.166100807

DPY30	0.193387	0.076099	2.541245	0.011045849	0.166415718
EIF2S3	-0.214612	0.084492	-2.540028	0.011084354	0.166580232
ZNF780A	0.36155	0.142355	2.539781	0.011092178	0.166580232
JUND	-0.295547	0.116376	-2.539572	0.011098809	0.166580232
SLCO4A1	-0.581439	0.22904	-2.538591	0.01112998	0.166703408
BRF2	0.504368	0.198693	2.538431	0.011135064	0.166703408
RPS7	-0.260886	0.102815	-2.537427	0.011167053	0.166972025
CARS2	0.370402	0.146045	2.536214	0.011205826	0.167341267
DNASE2	0.272774	0.107712	2.532434	0.011327368	0.168851097
MRM3	0.396128	0.156437	2.532187	0.011335339	0.168851097
SIDT2	-0.438522	0.173218	-2.53162	0.01135371	0.168913086
HMGXB4	0.244336	0.096547	2.530745	0.011382072	0.169123369
TMX3	-0.402711	0.159191	-2.529743	0.011414603	0.169394986
MMD	-0.509315	0.201407	-2.528785	0.011445814	0.169646366
LIN7B	-0.568516	0.224927	-2.527558	0.01148588	0.17002821
IGKC	0.34177	0.135382	2.524486	0.011586769	0.171308366
LY6E	-0.297529	0.118027	-2.520866	0.011706649	0.172865759
MT.ATP8	-0.515926	0.204851	-2.518542	0.011784167	0.17362088
THEMIS2	-0.791189	0.314188	-2.518204	0.011795493	0.17362088
CXCR4	0.20855	0.082823	2.518022	0.011801604	0.17362088
TRA2A	-0.210958	0.083804	-2.517283	0.01182638	0.17365076
CLCN3	0.361581	0.14365	2.51709	0.011832852	0.17365076
SERPINB9	-0.266946	0.106116	-2.515603	0.011882908	0.174170325
APIP	0.261792	0.10409	2.51505	0.011901554	0.174228791
PHF12	-0.352557	0.140243	-2.513891	0.011940739	0.174587415
C15orf65	-0.965435	0.384562	-2.510482	0.01205663	0.176065312
GNG11	-1.031079	0.410898	-2.509332	0.012095985	0.176423282
MTMR10	0.600918	0.239596	2.508045	0.01214012	0.176668457
MTMR4	-0.323624	0.129038	-2.507975	0.01214252	0.176668457
ACTR10	0.320043	0.127648	2.507238	0.012167887	0.176680966
MAP3K2	-0.261201	0.104185	-2.507086	0.012173106	0.176680966
ENSG00000257194	-1.931388	0.770451	-2.506829	0.01218195	NA
MCTP2	-0.525529	0.209667	-2.506487	0.012193738	0.176764591
ALDH1B1	-1.017225	0.40593	-2.505915	0.012213503	0.176835458
PSMB1	0.159037	0.063511	2.504093	0.012276565	0.177334245
RPS5	-0.272657	0.108886	-2.504058	0.012277789	0.177334245
SAFB	0.248087	0.099104	2.503303	0.012304013	0.177497338
ENSG00000247134	-1.075747	0.429988	-2.501806	0.012356145	0.178033329
SEC23B	0.454513	0.181756	2.500679	0.012395552	0.178384901
RPS27L	0.318426	0.127382	2.499776	0.012427177	0.17862376
ZCCHC2	-0.548618	0.219881	-2.49507	0.012593217	0.180791753
RFX3	-0.539938	0.216515	-2.493768	0.012639502	0.181161201
PPHLN1	0.176395	0.070742	2.49349	0.012649432	0.181161201
SVBP	-0.186794	0.074957	-2.491999	0.01270264	0.181615279
LINC02422	-0.508918	0.204242	-2.491746	0.012711694	0.181615279
MRNIP	0.2567	0.103079	2.490319	0.012762864	0.182127453
LENG8	-0.270098	0.108521	-2.488906	0.012813693	0.182633536

CCT7	0.256036	0.103001	2.485769	0.0129272	0.184030688
TPT1	-0.19304	0.077715	-2.483958	0.012993098	0.184747556
PAFAH1B3	-0.379415	0.152786	-2.483315	0.013016589	0.184860442
ENO2	-0.481993	0.194197	-2.481974	0.013065673	0.1853361
OGFR	0.224823	0.090643	2.480327	0.013126207	0.185972851
LACTB2.AS1	0.769314	0.310306	2.479209	0.013167416	0.186334613
CNR1	0.766783	0.309557	2.477036	0.013247871	0.186763507
MIB2	-0.294535	0.118915	-2.476845	0.013254947	0.186763507
ATP5MF	0.190488	0.076917	2.476531	0.013266615	0.186763507
QARS	-0.368184	0.148682	-2.476326	0.013274213	0.186763507
SEPTIN1	-0.18124	0.073201	-2.475934	0.013288832	0.186763507
TENT4A	-0.697663	0.281832	-2.475454	0.013306697	0.186763507
FKBP1B	-0.770049	0.311204	-2.474417	0.013345394	0.186763507
PTRHD1	-0.231526	0.093574	-2.474254	0.013351474	0.186763507
TMEM230	0.218436	0.088284	2.47424	0.013351999	0.186763507
NBEAL1	-0.442563	0.178874	-2.474164	0.01335484	0.186763507
POLR3D	0.367579	0.148679	2.472305	0.013424478	0.187516771
TUT1	0.40791	0.165048	2.471461	0.013456212	0.187739426
GPR160	0.558237	0.226023	2.46982	0.013518111	0.188381931
SPINK2	-1.932957	0.782649	-2.469763	0.013520274	NA
C16orf72	0.288018	0.116686	2.468328	0.013574584	0.188947406
RPL27A	-0.213881	0.086706	-2.466729	0.013635354	0.189571283
ENSG00000273188	0.790962	0.320877	2.465002	0.013701265	0.190265112
WWC3	-0.271033	0.110022	-2.463437	0.013761211	0.190874586
COMMD7	-0.33689	0.136796	-2.462713	0.013789012	0.19103728
PRDX3	0.224744	0.091319	2.461094	0.013851421	0.191678513
LSR	-0.706768	0.287365	-2.459483	0.013913745	0.192197244
IL21R	0.628993	0.255762	2.459289	0.013921244	0.192197244
ZNF524	-0.251126	0.102163	-2.458081	0.013968185	0.192537965
MRPL13	0.249373	0.101474	2.457501	0.013990749	0.192537965
NUDT1	0.213084	0.086711	2.457404	0.013994515	0.192537965
TNFRSF14	-0.179769	0.073176	-2.456681	0.014022698	0.19258561
RNF122	0.811948	0.330532	2.456485	0.014030381	0.19258561
TGS1	0.283984	0.115633	2.455896	0.014053389	0.192678933
ICA1	2.4801	1.009863	2.455878	0.014054067	NA
GNG7	-0.236735	0.096422	-2.455202	0.014080549	0.192828901
SPATS2L	-1.867155	0.760513	-2.455126	0.014083527	NA
WDFY2	-0.288072	0.117401	-2.453755	0.014137307	0.193383399
FNBP1	0.245859	0.100218	2.453232	0.014157911	0.193442633
SERPINF2	-1.311971	0.534937	-2.452572	0.014183903	0.19355012
LINC00996	1.403227	0.57223	2.452206	0.014198343	0.19355012
PLIN2	0.565355	0.230718	2.450412	0.014269285	0.194294378
PKNOX1	0.350039	0.143019	2.447496	0.014385274	0.195649602
MIIP	-0.237377	0.097079	-2.445194	0.014477406	0.196550619
TARS	0.281691	0.11521	2.445015	0.014484592	0.196550619
MARCKS	-0.565564	0.231631	-2.441661	0.014619886	0.198160308
KLB	-1.789308	0.732838	-2.441615	0.014621747	NA

RPL26	-0.252971	0.10369	-2.439687	0.014699985	0.199019049
ECE1	0.402117	0.164878	2.438881	0.014732828	0.199236774
RTN3	0.282992	0.116088	2.43774	0.014779419	0.19963972
PPP6R1	-0.297433	0.122092	-2.436144	0.014844764	0.200294784
UBE2L3	0.153553	0.063043	2.43567	0.014864232	0.200330071
CHMP5	0.202224	0.083074	2.434252	0.014922601	0.200888969
GALK1	-0.33158	0.136436	-2.430308	0.015086016	0.202679855
FCMR	0.325837	0.134077	2.430218	0.015089734	0.202679855
ENSG00000255328	-1.555154	0.640056	-2.429717	0.015110599	0.202725616
IL7R	-0.936904	0.385674	-2.429266	0.015129433	0.202725616
MYO1E	-0.632013	0.260204	-2.42891	0.015144304	0.202725616
CCDC154	0.817866	0.336951	2.427255	0.015213558	0.203423582
HIST1H2BD	0.49501	0.204209	2.424037	0.015349032	0.204994931
OTUD1	-0.572286	0.236126	-2.423646	0.015365566	0.204994931
ENSG00000234915	-1.871117	0.772083	-2.423465	0.015373225	NA
ABHD15	0.346534	0.143087	2.421845	0.015441936	0.205750019
VNN2	0.372078	0.153656	2.421496	0.015456782	0.205750019
KMT2D	-0.376037	0.155467	-2.418762	0.015573429	0.206959764
LINC02397	0.245584	0.101542	2.41855	0.015582484	0.206959764
NRBP2	-1.247203	0.515943	-2.417327	0.015634951	0.207424848
PSMC5	0.187526	0.077606	2.41639	0.015675263	0.207727816
TTC21B	-0.517184	0.214177	-2.414754	0.015745843	0.208430781
RPS15A	-0.243503	0.100935	-2.412474	0.015844678	0.209274333
EEF2	-0.333373	0.138195	-2.412342	0.015850427	0.209274333
CACTIN	-0.594788	0.246588	-2.412067	0.015862385	0.209274333
DEGS2	1.091716	0.453123	2.409315	0.015982517	0.21062547
FAM219B	-0.252067	0.10484	-2.404305	0.016203231	0.213297679
HACL1	0.32781	0.136409	2.403132	0.016255319	0.213746649
ATF5	0.578022	0.240689	2.401531	0.016326605	0.214446805
SNHG32	-0.209919	0.087432	-2.400924	0.016353727	0.214565951
SBDS	0.220084	0.091781	2.397932	0.016487914	0.216088014
SELENON	-1.081719	0.451363	-2.39656	0.016549801	0.21666023
MARCHF1	-0.431321	0.180051	-2.395544	0.016595716	0.216857518
PSMB6	0.168884	0.070503	2.39542	0.016601358	0.216857518
GFI1	1.734235	0.724818	2.392649	0.016727217	NA
AVPI1	-0.977045	0.408382	-2.392476	0.016735126	0.218152014
PLP2	-0.326303	0.136402	-2.392218	0.016746909	0.218152014
METTL9	-0.174028	0.072758	-2.391874	0.01676258	0.218152014
AIF1	-2.185122	0.91358	-2.391824	0.016764884	NA
RBM22	0.255631	0.10689	2.391527	0.016778445	0.218152014
NIP7	0.267919	0.112043	2.391226	0.016792218	0.218152014
PDHA1	0.296607	0.124209	2.387969	0.016941753	0.21985439
GRAPL	0.835323	0.349976	2.386803	0.016995586	0.220312464
SLC2A4RG	-0.947281	0.397034	-2.385892	0.017037763	0.220448138
MAGOHB	0.269031	0.112765	2.385776	0.017043143	0.220448138
TGFBR1	-0.448955	0.188412	-2.382832	0.017180032	0.221475901
CWF19L2	0.206137	0.086512	2.38275	0.017183848	0.221475901

PPDPF	-0.227682	0.095569	-2.382397	0.017200326	0.221475901
WNT10A	-0.632878	0.265662	-2.382265	0.017206505	0.221475901
AEN	0.335975	0.141043	2.382067	0.017215759	0.221475901
CYTIP	-0.178062	0.074781	-2.3811	0.017261021	0.221818121
FGF9	1.252731	0.526208	2.380678	0.017280816	0.221832675
PPT1	0.244372	0.102737	2.378623	0.017377436	0.222773188
CCDC88A	-0.514317	0.216278	-2.378041	0.017404898	0.222773188
IL17RA	-0.410946	0.172817	-2.377926	0.017410305	0.222773188
AP1S2	0.207884	0.087491	2.376063	0.017498481	0.223474027
ENSG00000277283	-0.7807	0.328611	-2.375758	0.017512954	0.223474027
RPA2	0.206293	0.086839	2.375578	0.017521477	0.223474027
RAP2C.AS1	-1.023337	0.431233	-2.373049	0.017641921	0.224769043
MID1IP1	-0.218677	0.092221	-2.371221	0.017729418	0.225397929
TTC7A	-0.333471	0.140637	-2.371153	0.017732696	0.225397929
RPL4	-0.221468	0.093413	-2.370831	0.017748167	0.225397929
ENSG00000274712	-0.652868	0.275657	-2.368406	0.017864903	0.226420207
SMAP2	-0.318827	0.134619	-2.368368	0.017866758	0.226420207
TOX4	0.211169	0.08923	2.36656	0.017954285	0.227062298
PRRC2B	-0.253429	0.107089	-2.366532	0.017955629	0.227062298
ENSG00000259274	-1.272175	0.53772	-2.365869	0.0179878	0.227227399
TOMM20	-0.1915	0.080965	-2.365228	0.018018972	0.227379532
DNAH1	0.915823	0.387316	2.364537	0.018052612	0.227525172
LRRC14	-0.547581	0.231613	-2.364205	0.018068795	0.227525172
ELOF1	0.2498	0.105693	2.363452	0.018105571	0.227747002
FIP1L1	0.256143	0.10848	2.361192	0.018216313	0.228662012
ENSG00000258572	-1.006725	0.426365	-2.361182	0.018216785	0.228662012
KCNC4	-0.605366	0.256601	-2.359169	0.018315888	0.22966346
CLDN12	-0.724859	0.307451	-2.357638	0.018391606	0.23036988
SCAMP2	0.221941	0.094181	2.356537	0.018446229	0.230699106
CEP70	0.904942	0.384048	2.356326	0.018456705	0.230699106
LINC01089	-0.259801	0.110285	-2.355711	0.018487288	0.230838646
WDR74	0.251037	0.106633	2.354214	0.018561915	0.231259629
ENSG00000261884	0.346708	0.147279	2.354088	0.018568214	0.231259629
CEP78	-0.367962	0.156365	-2.353226	0.018611329	0.231259629
EIF5B	0.153016	0.065024	2.35322	0.018611639	0.231259629
PFKL	-0.23344	0.099206	-2.353087	0.018618278	0.231259629
BICD1	-0.340544	0.144779	-2.352157	0.018664889	0.231535901
LAMTOR5	-0.153106	0.0651	-2.351867	0.018679476	0.231535901
HHEX	-0.200039	0.08508	-2.351185	0.018713704	0.231718536
ARHGAP15	0.190404	0.081066	2.348756	0.018836244	0.232993162
RNPS1	0.146399	0.062387	2.346624	0.018944368	0.234087005
SINHCAF	0.191663	0.081736	2.344916	0.019031375	0.234917923
HACD4	-0.540741	0.230698	-2.343934	0.01908155	0.235202961
KIFC2	-0.723404	0.308683	-2.343516	0.01910296	0.235202961
RPL35A	-0.247177	0.105482	-2.343304	0.019113827	0.235202961
ABTB1	0.190569	0.081358	2.342357	0.019162389	0.235335762
DNASE1L3	-1.145063	0.488858	-2.342321	0.019164214	0.235335762

TFEB	0.473086	0.202024	2.341735	0.019194343	0.235454541
PPP2R5C	0.173217	0.073981	2.341362	0.019213503	0.235454541
RGS2	-0.88454	0.378022	-2.339918	0.019287955	0.236123503
LINC00672	-0.677297	0.289554	-2.339101	0.019330195	0.236397148
EIF2AK3	-0.373163	0.159615	-2.337892	0.019392826	0.236784405
METTL21A	-0.237608	0.101648	-2.337566	0.01940979	0.236784405
CYTOR	-0.841631	0.360123	-2.337069	0.019435611	0.236784405
SLC23A2	0.390221	0.166989	2.336803	0.019449444	0.236784405
PFDN2	0.162779	0.069677	2.336199	0.019480872	0.236784405
RPS6KA1	0.279148	0.119489	2.336189	0.019481379	0.236784405
SLC6A16	-0.472243	0.202214	-2.335358	0.019524752	0.237069186
C1orf43	0.174801	0.074866	2.334856	0.019550934	0.237109968
SMOX	1.054663	0.451767	2.33453	0.019568005	0.237109968
LCMT1	-0.259724	0.111316	-2.333204	0.019637467	0.237535214
FAM177B	-0.517393	0.22178	-2.33291	0.019652895	0.237535214
TMEM154	0.311973	0.133738	2.332716	0.019663048	0.237535214
ATP23	-0.456181	0.195685	-2.331199	0.019742891	0.238257608
SESTD1	-0.313004	0.134322	-2.330251	0.01979288	0.238618625
FAM111A	-0.291789	0.125291	-2.328899	0.019864427	0.239173531
EIF3K	-0.229759	0.098667	-2.328621	0.019879149	0.239173531
ENSG00000233461	0.493031	0.211926	2.326434	0.019995435	0.240237034
EEF1B2	-0.335836	0.144383	-2.326008	0.020018134	0.240237034
PSMA5	0.173857	0.074753	2.325748	0.020031999	0.240237034
HSPA5	0.324701	0.13963	2.325441	0.020048384	0.240237034
RPL37	-0.183246	0.078826	-2.324688	0.02008864	0.240477007
IFNAR1	0.213178	0.091736	2.323813	0.02013553	0.240795825
GFM1	0.330769	0.142389	2.323004	0.020178926	0.241072252
ZNF169	-0.36109	0.155537	-2.321571	0.020256062	0.241467499
EPM2AIP1	-0.17611	0.075858	-2.321562	0.020256547	0.241467499
DDAH2	-0.277642	0.119608	-2.321257	0.020272951	0.241467499
IARS	0.270708	0.116658	2.320526	0.020312444	0.241695722
HSD17B8	-0.627331	0.270445	-2.319624	0.020361203	0.242033624
CDV3	-0.287191	0.123832	-2.319194	0.020384506	0.242068555
KHDRBS2	-0.639885	0.275996	-2.31846	0.020424329	0.242299402
ENSG00000225889	-0.798578	0.344679	-2.316873	0.020510624	0.243080551
LIMS2	-1.466188	0.632887	-2.316667	0.020521879	NA
HIST1H2BC	0.59423	0.256567	2.316084	0.020553687	0.243326062
LINC02035	-0.998813	0.431314	-2.315743	0.02057228	0.243326062
ERGIC1	-0.242214	0.104731	-2.312733	0.020737349	0.245034663
FSIP2	0.671352	0.29038	2.311981	0.020778721	0.245279694
SYK	-0.223658	0.096757	-2.311532	0.020803463	0.245328136
PAFAH1B2	-0.171707	0.074351	-2.309403	0.020921249	0.246472627
LCK	0.507652	0.219922	2.308329	0.020980855	0.246798296
BCL9	-1.212189	0.525176	-2.308157	0.020990416	0.246798296
MOB4	0.229295	0.09939	2.307022	0.021053583	0.247296384
RTKN	-0.777793	0.337265	-2.30618	0.021100558	0.247410377
SLC9A3R1	0.338124	0.146621	2.306102	0.021104915	0.247410377

KARS	0.202393	0.087782	2.305625	0.021131568	0.247478768
EEF1A1	-0.331651	0.143943	-2.304043	0.021220211	0.247501918
PPP1R8	0.268472	0.116534	2.30381	0.021233296	0.247501918
HIST1H1D	-0.354896	0.154055	-2.303692	0.021239948	0.247501918
ATP1B3	0.288205	0.125116	2.303502	0.021250607	0.247501918
ETFA	0.195814	0.085009	2.303447	0.02125368	0.247501918
ZEB1	-0.317379	0.137809	-2.303029	0.021277212	0.247501918
PLEKHJ1	-0.213917	0.092898	-2.302699	0.021295764	0.247501918
NACA	-0.1797	0.078045	-2.302536	0.021304946	0.247501918
TIMMDC1	0.262431	0.114006	2.301901	0.021340771	0.247501918
ENSG00000273002	0.638803	0.277513	2.301883	0.021341757	0.247501918
UEVLD	-0.537364	0.233538	-2.300969	0.021393391	0.247858903
DHRS12	-0.3379	0.146966	-2.299177	0.021494879	0.248792238
SRP72	0.200252	0.08717	2.297265	0.021603676	0.249222744
SHMT2	-0.208475	0.090754	-2.29715	0.021610209	0.249222744
NDUFAF4	0.251626	0.109556	2.296775	0.021631632	0.249222744
HMOX1	0.797512	0.34727	2.296521	0.021646117	0.249222744
C11orf80	-0.378831	0.164985	-2.296148	0.021667418	0.249222744
ATP5MG	-0.132663	0.057778	-2.296091	0.02167068	0.249222744
ATP5PF	0.139408	0.060727	2.295665	0.021695039	0.249222744
SH3BP5	-0.342827	0.149342	-2.295582	0.021699801	0.249222744
ATP6AP2	0.265611	0.115764	2.294412	0.021766857	0.249751568
LY75	-0.470279	0.205022	-2.293799	0.021802022	0.249913824
RNPC3	-0.337367	0.147116	-2.293214	0.021835714	0.250058893
GPR65	-0.261475	0.114082	-2.291991	0.021906166	0.250624245
C22orf15	1.348436	0.588399	2.291704	0.021922704	NA
PITPNM1	-0.455087	0.19874	-2.289858	0.022029526	0.251793251
KANK2	-1.189264	0.51959	-2.288852	0.022087959	0.252042725
ENSG00000251136	0.456376	0.199436	2.288334	0.022118106	0.252042725
ATXN1L	0.317574	0.138779	2.288334	0.022118074	0.252042725
RPL8	-0.250023	0.109287	-2.287758	0.022151637	0.252042725
MYL5	-0.459129	0.200706	-2.287576	0.02216222	0.252042725
NOP10	0.15878	0.069418	2.287296	0.022178572	0.252042725
HIST1H4C	-0.297505	0.130098	-2.286774	0.022209046	0.252147971
CINP	0.258065	0.112973	2.284312	0.022353225	0.253492713
SLC4A1AP	0.315968	0.138368	2.28353	0.022399143	0.253492713
RPS15	-0.182294	0.079838	-2.283307	0.022412309	0.253492713
DDX18	0.151706	0.066443	2.283259	0.022415094	0.253492713
GADD45A	0.460148	0.20156	2.282936	0.022434116	0.253492713
SMYD4	-0.319452	0.140027	-2.28135	0.02252774	0.254308872
S100A4	-0.488687	0.214316	-2.280216	0.02259491	0.254825134
LINC02362	-1.581621	0.693868	-2.279426	0.022641739	NA
PTPMT1	-0.222825	0.097829	-2.277693	0.022744852	0.256104495
RPL7A	-0.250804	0.110119	-2.277583	0.022751438	0.256104495
TINCR	1.274262	0.559699	2.276693	0.022804547	NA
EIF1	0.14463	0.06353	2.276569	0.022811973	0.256542969
COX7B	0.149991	0.065904	2.275887	0.022852802	0.256759221

C4orf3	0.178263	0.078348	2.275272	0.022889614	0.256929975
PCSK7	-0.222168	0.097661	-2.27488	0.02291312	0.25695119
ARL6IP4	-0.152881	0.067271	-2.272621	0.023049032	0.258231707
DIS3L2	-0.31694	0.13954	-2.27132	0.023127624	0.258647294
MCPH1	0.252793	0.1113	2.271286	0.023129644	0.258647294
COA4	0.207274	0.091285	2.270619	0.02317007	0.258855842
LPP.AS2	-0.852202	0.375675	-2.268452	0.023301661	0.259905069
RPS27	-0.184624	0.081391	-2.268353	0.023307715	0.259905069
TLR7	-0.599306	0.264246	-2.26799	0.023329795	0.25990747
ICAM3	-0.242982	0.107173	-2.2672	0.023377996	0.260200599
ENSG00000260257	-0.404185	0.17838	-2.26587	0.023459332	0.260861629
KAT6A	0.206541	0.091168	2.265488	0.02348275	0.260877987
PSMC3	0.200548	0.088552	2.264738	0.02352875	0.261144955
GHITM	0.198451	0.087643	2.264318	0.023554552	0.261187461
NT5C2	0.300373	0.132709	2.263394	0.023611436	0.261574223
LZTS3	-1.161672	0.513433	-2.26256	0.023662848	0.261814993
MLXIP	0.282571	0.124903	2.262327	0.02367722	0.261814993
TPI1	0.133827	0.059168	2.261805	0.023709475	0.261928003
SPPL2B	-0.250488	0.110809	-2.260537	0.023787966	0.262551114
TRIOBP	-0.361662	0.160112	-2.25881	0.023895226	0.263490303
UBTD2	-0.690358	0.305768	-2.257787	0.02395894	0.263948026
MBD6	0.407128	0.180429	2.256452	0.02404232	0.264621349
HNRNPF	0.228983	0.101505	2.255877	0.024078362	0.264772882
TCF3	-0.321965	0.142839	-2.254048	0.02419316	0.265789365
JUN	0.277184	0.12304	2.252788	0.024272531	0.26641512
BOLA2.SMG1P6	0.424942	0.1887	2.251943	0.024325885	0.26675442
SLC25A3	-0.220642	0.098034	-2.250664	0.024406822	0.267395294
LINC01003	-0.722452	0.321431	-2.247607	0.02460127	0.269108307
VANGL2	-1.413905	0.629125	-2.247416	0.024613471	0.269108307
ENSG00000265206	-0.214659	0.095534	-2.246925	0.024644816	0.269108307
PSMA3	0.20632	0.091829	2.246786	0.024653735	0.269108307
ARHGAP5	-0.253563	0.112894	-2.246032	0.024701938	0.269387096
LINC01184	-0.360545	0.160581	-2.24525	0.024752059	0.269686271
TKT	-0.304539	0.13569	-2.244371	0.024808569	0.270054447
NPM3	-0.412401	0.183857	-2.243051	0.024893556	0.270460488
RPF1	0.223169	0.099515	2.242562	0.024925099	0.270460488
ZCCHC17	0.204127	0.091028	2.242479	0.024930436	0.270460488
STARD9	-0.481844	0.2149	-2.242171	0.024950352	0.270460488
TPD52L2	0.231255	0.103145	2.242027	0.024959633	0.270460488
SLX4	-0.747464	0.334027	-2.237734	0.025238435	0.273232497
LRP10	0.184993	0.082684	2.237344	0.025263873	0.273259013
ENSG00000254281	-1.178622	0.526894	-2.236924	0.025291307	0.273307062
DDX17	-0.161857	0.07242	-2.234963	0.025419759	0.274348948
HS2ST1	-0.481022	0.215253	-2.234679	0.025438454	0.274348948
TMED9	0.197633	0.088462	2.23411	0.025475805	0.274348948
CNTRL	-0.169519	0.075906	-2.233266	0.025531426	0.274348948
TSPYL2	0.373364	0.16719	2.233172	0.025537578	0.274348948

PSMA6	0.291871	0.130705	2.233044	0.025546043	0.274348948
TMEM219	-0.171505	0.076817	-2.232633	0.025573162	0.274348948
LPXN	0.374483	0.167778	2.232006	0.025614572	0.274348948
RPL15	-0.184318	0.082587	-2.231816	0.025627113	0.274348948
SLC38A1	-0.199369	0.089334	-2.231728	0.02563296	0.274348948
AHCY	-0.363336	0.162814	-2.231597	0.025641598	0.274348948
UCK2	0.583459	0.26152	2.231028	0.025679295	0.274505193
LINC00924	-1.846145	0.827579	-2.230779	0.025695789	NA
FAM76A	-0.238027	0.10681	-2.228514	0.025846274	0.276041924
PLEKHB2	0.257552	0.115634	2.227302	0.0259271	0.276585262
STRADB	-0.358282	0.160877	-2.227054	0.025943684	0.276585262
TTC33	0.363744	0.163369	2.226517	0.02597958	0.276719776
CDC123	0.220312	0.098988	2.225635	0.02603861	0.277040716
AGBL5	-0.590292	0.265255	-2.225371	0.026056324	0.277040716
PRPSAP2	0.296274	0.133159	2.224953	0.026084406	0.277065055
ENSG00000227373	1.361434	0.611979	2.224643	0.026105229	0.277065055
RBM7	0.235222	0.105774	2.223825	0.026160217	0.277400977
TOMM5	0.189793	0.085381	2.222879	0.026223972	0.277486127
UBE2O	0.315573	0.141976	2.222719	0.026234761	0.277486127
MAP4K4	-0.235724	0.106077	-2.222198	0.026269924	0.277486127
KIAA1109	-0.303818	0.136773	-2.221337	0.026328164	0.277486127
PIP4K2A	-0.401734	0.180855	-2.221306	0.026330229	0.277486127
CLIC4	-0.254941	0.114784	-2.221047	0.026347783	0.277486127
MRPS18A	0.214946	0.096787	2.220805	0.026364146	0.277486127
BOD1L1	0.180001	0.081053	2.22077	0.026366576	0.277486127
CALM3	-0.220639	0.09936	-2.220596	0.02637834	0.277486127
TIGIT	-1.919363	0.864645	-2.219828	0.026430422	NA
ZFP14	-0.225698	0.101742	-2.21834	0.026531691	0.278852531
MGMT	-0.270996	0.122281	-2.216167	0.02668004	0.27987236
KANSL1L	-0.552306	0.249219	-2.216146	0.026681522	0.27987236
FAM30A	-0.202499	0.091395	-2.215657	0.026714986	0.27987236
FAM209B	-1.754859	0.792243	-2.215051	0.026756543	NA
SGO1	0.333485	0.150571	2.214804	0.026773551	0.27987236
DTX2	0.392021	0.17701	2.214683	0.026781808	0.27987236
RPL18	-0.198471	0.089625	-2.214465	0.026796775	0.27987236
RPL5	-0.24085	0.108772	-2.214267	0.0268104	0.27987236
FAR1	-0.410079	0.185207	-2.21417	0.026817079	0.27987236
LENG1	0.242459	0.109617	2.211863	0.02697615	0.281107896
TOR1AIP1	0.284498	0.128656	2.211302	0.027014929	0.281107896
PRMT1	0.187039	0.084589	2.211163	0.027024532	0.281107896
METTL1	0.653393	0.295508	2.211083	0.02703006	0.281107896
SERP1	-0.146606	0.066338	-2.20997	0.02710723	0.281239742
ZC3H14	-0.263676	0.119315	-2.20991	0.027111426	0.281239742
TFIP11	0.331356	0.149943	2.209877	0.027113716	0.281239742
AK2	0.222251	0.100681	2.207481	0.027280439	0.282722384
HOMER2	-0.691802	0.313522	-2.206549	0.027345599	0.282906524
RPS8	-0.28199	0.127797	-2.206546	0.027345806	0.282906524

ACYP1	0.229107	0.10387	2.205715	0.027403963	0.283261659
IL6ST	-0.449563	0.203862	-2.205232	0.027437805	0.283365063
TMSB4Y	-1.622997	0.736086	-2.204903	0.027460915	NA
CORO2B	-1.653715	0.750177	-2.204434	0.027493863	0.283681546
ZBTB18	-0.312808	0.14192	-2.204116	0.027516179	0.283681546
IFI27L1	0.403311	0.183052	2.203256	0.027576733	0.283943174
RBM42	0.300124	0.136232	2.203036	0.02759223	0.283943174
CCR9	1.846828	0.838381	2.20285	0.027605316	NA
SPIB	0.160954	0.07307	2.202738	0.027613217	0.283943174
INPP5B	0.321446	0.146032	2.201195	0.027722254	0.284818009
MPZL3	-1.052623	0.478642	-2.199184	0.027864808	0.286035386
LINC00630	-1.70836	0.777645	-2.196839	0.02803193	NA
AIMP1	0.164146	0.074754	2.195815	0.028105171	0.288247034
ENSG00000260030	2.714015	1.236601	2.194739	0.02818234	NA
NUCKS1	-0.18145	0.082695	-2.194214	0.028220054	0.288247034
PTPN18	-0.380619	0.173466	-2.194197	0.028221257	0.288247034
OPA3	0.290335	0.132345	2.193768	0.028252087	0.288247034
BCAS2	0.231604	0.10558	2.19364	0.028261268	0.288247034
RUNX3	0.212531	0.096887	2.193592	0.028264743	0.288247034
ENSG00000231856	-0.65629	0.299196	-2.193514	0.028270379	0.288247034
INPP5E	-0.562215	0.256314	-2.19346	0.028274253	0.288247034
CASP4	-0.150796	0.068763	-2.192976	0.028309104	0.288355025
RUNX1	0.291417	0.132979	2.191447	0.028419468	0.289231347
SEC61A2	-0.440864	0.201209	-2.191079	0.028446043	0.289254159
HDAC1	0.21931	0.100123	2.1904	0.028495244	0.289506806
SLC7A5	-1.187776	0.542477	-2.189542	0.028557498	0.289891523
RAB11A	0.155457	0.071043	2.188217	0.028653834	0.290621265
TPRKB	0.153221	0.07004	2.187627	0.028696794	0.290691999
FAM49B	0.170715	0.078043	2.18745	0.028709717	0.290691999
RPL7	-0.280586	0.128322	-2.186578	0.028773352	0.29108837
UBE2G1	0.240739	0.110144	2.185682	0.028838865	0.291318186
ENSG00000133961	0.211768	0.096893	2.185597	0.028845083	0.291318186
GTPBP1	0.27914	0.127824	2.183785	0.02897805	0.292412633
KPNA2	-0.325365	0.149074	-2.182574	0.029067232	0.29306377
MPP1	-0.601845	0.275854	-2.181754	0.029127658	0.293186466
ABLIM2	-0.496415	0.227532	-2.18174	0.02912873	0.293186466
ANKLE1	0.609294	0.27936	2.181036	0.029180756	0.29328622
SLC2A1	0.258138	0.118361	2.180938	0.029187987	0.29328622
AFF2	-1.551188	0.711357	-2.180604	0.02921269	NA
CLASP1	-0.42242	0.193737	-2.180376	0.02922962	0.293456493
GRK3	0.256981	0.11788	2.180033	0.029255032	0.293463772
TSPAN33	0.294479	0.135123	2.179341	0.02930632	0.293730378
TMEM8A	-0.458771	0.210563	-2.17878	0.029347984	0.293900158
QSOX2	-0.478023	0.219931	-2.173513	0.029741758	0.297592826
ENSG00000261732	0.374614	0.172392	2.173043	0.029777078	0.297695646
SELENOT	0.16132	0.074327	2.17042	0.029975019	0.299422729
ENSG00000267416	-1.689397	0.778397	-2.170355	0.029979991	NA

ATP5F1B	0.198203	0.091382	2.168944	0.03008695	0.300288476
FIBP	-0.204635	0.094364	-2.168579	0.030114672	0.300313004
NUDT14	-0.455513	0.210135	-2.167714	0.030180429	0.300512029
ZNF93	-0.415104	0.191499	-2.167652	0.030185191	0.300512029
SEPTIN6	-0.160795	0.074191	-2.167315	0.030210866	0.300515958
ENSG00000230155	0.400134	0.184752	2.165788	0.030327379	0.301422708
ENSG00000261684	-1.670038	0.771214	-2.165467	0.030351928	NA
TWF2	0.189627	0.08759	2.16495	0.030391529	0.301807939
AGAP4	-0.627402	0.289865	-2.164466	0.030428591	0.301923761
ALG2	-0.345791	0.159798	-2.163929	0.030469797	0.302080467
RPL7L1	0.159567	0.073757	2.163408	0.030509793	0.302224925
SEC22C	-0.23394	0.108194	-2.162221	0.030601139	0.302672153
ZFC3H1	-0.245091	0.113355	-2.16216	0.030605866	0.302672153
HLCS	-0.883918	0.409011	-2.161112	0.030686657	0.303058577
HLA.DPA1	-0.292825	0.135505	-2.160992	0.030695931	0.303058577
DOP1B	-0.380207	0.176099	-2.159055	0.030845884	0.304063381
CD59	0.252338	0.116876	2.159017	0.030848863	0.304063381
CYLD	-0.207409	0.096094	-2.158398	0.030896917	0.304284713
MRPS28	0.260188	0.120614	2.157191	0.030990787	0.304943418
C5orf56	0.343315	0.159195	2.156568	0.031039353	0.304943418
ARMC7	0.426967	0.197986	2.15655	0.031040762	0.304943418
FAM13A	0.576891	0.267607	2.155742	0.031103844	0.305003406
C3orf33	-0.948159	0.439833	-2.155725	0.031105125	0.305003406
PSD4	-0.357542	0.165876	-2.155486	0.031123844	0.305003406
MAN2A1	-0.338244	0.157036	-2.153933	0.031245422	0.305397625
ENSG00000229728	1.089926	0.506147	2.153381	0.031288782	0.305397625
BIRC6	-0.189383	0.08795	-2.153312	0.031294171	0.305397625
ZNRD2	0.219641	0.102013	2.15307	0.031313154	0.305397625
SCOC.AS1	0.502352	0.233321	2.153055	0.031314317	0.305397625
TBL1XR1	-0.22139	0.102828	-2.153006	0.031318222	0.305397625
MTG1	0.291312	0.135394	2.151592	0.031429491	0.306231445
FAM172A	-0.274565	0.127679	-2.150435	0.031520802	0.306869597
RPL28	-0.263577	0.122608	-2.149748	0.031575181	0.30701592
SEC62	-0.15366	0.071483	-2.149592	0.031587488	0.30701592
DNAJC10	-0.195491	0.090985	-2.148603	0.03166587	0.307526307
NFYC	0.251775	0.117218	2.147923	0.031719909	0.307799638
NDUFS4	-0.311012	0.14483	-2.147425	0.031759478	0.307932234
ENSG00000253891	0.902322	0.420332	2.146692	0.031817797	0.308246257
ARID4B	0.164176	0.076495	2.146231	0.031854543	0.308350943
INSL3	1.015962	0.473508	2.145608	0.031904292	0.308581223
SLC27A5	-0.468355	0.218338	-2.145088	0.03194581	0.308731577
CDK7	0.370387	0.172697	2.144722	0.031975073	0.308763356
POLR3GL	-0.16475	0.076856	-2.143619	0.032063423	0.309261925
DIMT1	0.294396	0.137361	2.14322	0.032095422	0.309261925
R3HCC1L	0.404618	0.1888	2.143104	0.032104754	0.309261925
PIKFYVE	0.282316	0.131813	2.141794	0.032210074	0.309826581
SLC26A1	1.239409	0.578777	2.141428	0.0322395	0.309826581

GNPDA2	0.269302	0.12576	2.141403	0.032241565	0.309826581
RXRB	0.264809	0.1238	2.139011	0.032434811	0.311311519
SUCLG1	0.235742	0.11022	2.138842	0.032448471	0.311311519
PEG10	-1.067023	0.49906	-2.138066	0.032511424	0.311663951
NOP53	-0.193527	0.090594	-2.136209	0.032662376	0.312757336
CNEP1R1	-0.37629	0.176172	-2.13592	0.032685943	0.312757336
TMEM50A	0.185324	0.086775	2.135679	0.032705564	0.312757336
ENSG00000280206	0.46866	0.219484	2.135281	0.032738098	0.312757336
CSK	0.205589	0.096292	2.135049	0.032757036	0.312757336
TCTA	0.329409	0.1544	2.133487	0.032884825	0.313725452
U2AF1L4	-0.223919	0.104978	-2.133016	0.032923414	0.313841716
PQLC3	-0.298234	0.139963	-2.130796	0.033105957	0.315288519
SCAF11	-0.121643	0.057096	-2.130526	0.033128238	0.315288519
ZNF432	-0.566098	0.265787	-2.129892	0.033180528	0.315533545
LMO2	-1.841243	0.864648	-2.12947	0.033215399	NA
H6PD	-0.393771	0.184967	-2.128875	0.033264582	0.316064021
ANK1	-0.919506	0.431982	-2.128574	0.033289489	0.316064021
SH2B3	0.421989	0.198303	2.127994	0.033337562	0.316267835
SDR42E1	-0.955591	0.449375	-2.126487	0.033462759	0.317173015
UBE2V2	0.15699	0.073836	2.126203	0.033486341	0.317173015
USP4	0.251674	0.118451	2.124706	0.033611135	0.318011383
WDR81	-0.423523	0.199352	-2.1245	0.033628359	0.318011383
NT5DC1	-0.284363	0.133948	-2.122946	0.033758366	0.318888407
ITPA	0.238906	0.112575	2.122188	0.03382197	0.318888407
UBL7.AS1	-0.347004	0.163516	-2.122137	0.033826264	0.318888407
NOTCH2	0.263917	0.124369	2.122057	0.033832927	0.318888407
PPARA	-0.345469	0.162819	-2.121792	0.033855234	0.318888407
LAMA5	-0.767504	0.361894	-2.120799	0.033938712	0.319421587
DCAF5	-0.279734	0.131978	-2.119541	0.03404475	0.32016609
STK4	-0.141427	0.066783	-2.117697	0.034200716	0.321260197
BHLHE40.AS1	1.117988	0.527968	2.117527	0.034215143	0.321260197
SAR1B	0.235107	0.111054	2.11705	0.034255585	0.321386065
CCDC59	0.15085	0.071274	2.116482	0.034303831	0.32158489
CCDC141	-0.733807	0.346769	-2.116124	0.034334261	0.321616517
KLC2	-0.415114	0.196292	-2.114779	0.034448802	0.322369133
PHB2	-0.219721	0.103909	-2.114544	0.034468846	0.322369133
JARID2	-0.260249	0.123166	-2.112992	0.034601419	0.323354617
WDR54	-0.219666	0.104018	-2.111805	0.034703193	0.324050948
ZNF616	0.465721	0.220643	2.110743	0.034794416	0.324647744
TLE1	0.644997	0.305782	2.109338	0.034915387	0.325297269
ENSG00000180448	0.256264	0.121501	2.109152	0.034931503	0.325297269
MICAL3	-0.222643	0.105573	-2.108895	0.034953687	0.325297269
LTV1	0.279383	0.132507	2.108437	0.034993234	0.325297269
PDHB	0.187156	0.088773	2.10826	0.035008481	0.325297269
ERCC5	0.309739	0.146933	2.108032	0.035028225	0.325297269
SHISA5	0.201499	0.095674	2.106097	0.035195913	0.326570234
PSENEN	0.196835	0.093472	2.105817	0.035220244	0.326570234

CDKN2D	0.242431	0.115188	2.10465	0.035321774	0.327256373
HIST1H4E	0.630377	0.299649	2.103716	0.035403251	0.327755799
AP1S3	-0.470892	0.223874	-2.10338	0.03543259	0.327772135
PRKRA.AS1	-0.466277	0.221763	-2.102593	0.03550138	0.32815311
MAGED1	-0.590218	0.280874	-2.101366	0.035608872	0.328709533
ZMYND11	-0.460651	0.21928	-2.100743	0.035663544	0.328709533
HSP90B1	0.194311	0.092499	2.100689	0.035668236	0.328709533
PARP10	0.281275	0.133899	2.100644	0.035672188	0.328709533
ZNF736	-0.409225	0.194866	-2.100038	0.035725466	0.328945478
EIF4EBP3	0.610618	0.290842	2.099482	0.035774412	0.329044957
UTRN	-0.328975	0.156737	-2.098902	0.03582551	0.329044957
MYO5A	-0.376894	0.179586	-2.098685	0.035844691	0.329044957
LINC00426	-0.591129	0.28167	-2.098659	0.035846994	0.329044957
ENSG00000272211	-0.608261	0.289981	-2.097592	0.035941213	0.329628698
SLC9A7	0.272433	0.129896	2.097311	0.035966049	0.329628698
RIPK2	0.258272	0.123187	2.096575	0.036031181	0.329971228
SF3B5	0.160961	0.076817	2.09539	0.036136316	0.33047394
DARS	0.156958	0.074908	2.09533	0.036141678	0.33047394
ERGIC3	-0.201142	0.096061	-2.093885	0.036270234	0.331274425
CCNT2	-0.253883	0.121259	-2.09372	0.036284958	0.331274425
NSMCE2	0.241816	0.115645	2.091014	0.036526809	0.333085423
ZNF689	0.330075	0.157865	2.090874	0.036539362	0.333085423
LINC02245	-0.688339	0.329309	-2.090253	0.036595056	0.333337495
UPP1	0.502321	0.240426	2.089291	0.03668158	0.333665521
ENSG00000266378	-1.305069	0.624713	-2.089068	0.036701562	0.333665521
CCNJ	-0.718751	0.344079	-2.088916	0.036715277	0.333665521
IFI35	0.263997	0.126423	2.088198	0.036779961	0.333998008
LARP7	0.146255	0.070086	2.086797	0.036906533	0.334891574
PIK3IP1	0.276289	0.132428	2.08633	0.036948748	0.335008068
CLK4	-0.242209	0.116125	-2.085758	0.037000529	0.335008068
EXOSC3	0.210881	0.101121	2.085439	0.037029511	0.335008068
COX7A2L	-0.121577	0.058299	-2.08541	0.037032102	0.335008068
RTL6	-0.68457	0.328426	-2.084395	0.037124224	0.335586049
STX12	0.192456	0.092362	2.083718	0.037185784	0.335887097
APOBEC3B	1.099998	0.528045	2.083152	0.037237344	0.335927213
KCTD20	-0.245413	0.117859	-2.082255	0.037319222	0.335927213
SLC25A11	0.20035	0.096226	2.082071	0.037336028	0.335927213
PPARD	-0.398691	0.191513	-2.081791	0.037361537	0.335927213
ENSG00000228172	1.188341	0.570851	2.081701	0.037369804	0.335927213
RAE1	0.300161	0.144202	2.081534	0.03738503	0.335927213
GDE1	-0.270803	0.1301	-2.081501	0.037388046	0.335927213
AKIRIN1	-0.171988	0.082649	-2.080944	0.037438987	0.336130842
CHRNA6	-1.263293	0.607253	-2.080342	0.037494182	0.336340198
ENSG00000269549	0.893758	0.429731	2.079806	0.037543318	0.336340198
PSMB2	0.163	0.078374	2.079764	0.03754719	0.336340198
PEX1	-0.38731	0.186317	-2.078765	0.037638912	0.336907943
PRCC	0.203321	0.097832	2.078257	0.037685652	0.337072495

PARP15	-0.276276	0.13301	-2.077107	0.037791688	0.337700274
ZBTB8OS	0.196256	0.094495	2.07688	0.037812658	0.337700274
GOLGA8B	-0.286326	0.1379	-2.076324	0.037864015	0.337905066
AHNAK	-0.468356	0.22568	-2.075312	0.037957619	0.338267044
QSOX1	0.534408	0.257512	2.07527	0.03796149	0.338267044
TXLNA	-0.308238	0.148568	-2.07472	0.03801248	0.338467681
ENSG00000233184	-0.436776	0.210573	-2.074228	0.038058133	0.338552517
IMPA2	-0.598862	0.288747	-2.074004	0.03807897	0.338552517
ENSG00000267136	1.215255	0.586191	2.073137	0.038159498	NA
HES6	-0.515911	0.248873	-2.072989	0.038173286	0.339137406
SDHB	0.229595	0.110814	2.071886	0.038276082	0.339796708
CETP	-0.876725	0.423253	-2.0714	0.03832146	0.33980827
RPL14	-0.186989	0.090292	-2.070936	0.038364823	0.33980827
C12orf10	-0.240165	0.115987	-2.070616	0.038394727	0.33980827
SORD	-0.446479	0.215664	-2.070248	0.038429114	0.33980827
ATP2C1	-0.32316	0.156112	-2.070051	0.038447529	0.33980827
GTF2F1	-0.185731	0.089724	-2.070037	0.038448904	0.33980827
ANP32B	-0.133121	0.064328	-2.069391	0.03850938	0.339962398
ZEB1.AS1	-0.470035	0.227173	-2.069064	0.038540113	0.339962398
BRD3	-0.216064	0.104433	-2.068935	0.038552142	0.339962398
TK2	-1.306241	0.631555	-2.068293	0.038612437	0.340241688
TRIAP1	0.233044	0.112715	2.067549	0.038682452	0.340606156
SLIRP	0.138357	0.066944	2.066767	0.038756083	0.341001894
KCNH4	1.383732	0.669898	2.065586	0.03886763	NA
SSR3	-0.170902	0.082743	-2.065448	0.038880663	0.341681158
DNAJC28	-0.815473	0.394837	-2.065341	0.038890772	0.341681158
GNAI2	-0.186525	0.09033	-2.064938	0.038928849	0.341763094
PCTP	0.350667	0.169884	2.064157	0.039002808	0.342159691
SLC37A1	-0.332956	0.161394	-2.062993	0.039113251	0.342875525
PKN1	0.238791	0.115781	2.062442	0.039165706	0.343082353
ZNF675	0.306525	0.148651	2.062053	0.039202689	0.343153438
FAM111B	-0.325856	0.158076	-2.061389	0.039265903	0.343453854
MRPS10	0.167472	0.081255	2.061059	0.039297437	0.343476934
TGOLN2	-0.160973	0.07815	-2.059796	0.039418015	0.344277693
OSTM1	0.258631	0.125604	2.059109	0.039483834	0.344599368
CENPJ	-0.376578	0.182955	-2.058314	0.039560033	0.344686459
HNRNPR	0.181934	0.0884	2.058079	0.03958251	0.344686459
MEF2D	-0.318054	0.154553	-2.057892	0.039600508	0.344686459
FUOM	0.400455	0.194606	2.057774	0.03961179	0.344686459
LIG1	-0.347329	0.168812	-2.057493	0.039638798	0.344686459
SLC35F5	-0.465916	0.226575	-2.056341	0.039749648	0.345275544
RYK	-0.359689	0.174944	-2.056024	0.03978017	0.345275544
C15orf62	-0.458929	0.223262	-2.055559	0.039825015	0.345275544
TRAPPC4	0.217752	0.105937	2.055487	0.039831951	0.345275544
ENSG00000268713	-0.666326	0.324202	-2.055282	0.039851775	0.345275544
CYB5R4	0.212227	0.103297	2.05453	0.039924467	0.345453989
TMCO1	0.170405	0.08295	2.0543	0.039946673	0.345453989

ENSG00000272871	-1.041617	0.507075	-2.054167	0.039959555	0.345453989
ARF4	0.242423	0.118109	2.052537	0.040117526	0.346253356
KLHDC1	-0.693823	0.338073	-2.052288	0.04014172	0.346253356
EAF2	0.291498	0.142051	2.052065	0.04016335	0.346253356
CCNG2	-0.340182	0.16578	-2.052012	0.040168535	0.346253356
TCEAL8	-0.224727	0.109539	-2.051564	0.040212072	0.346377461
BRD3OS	-0.404626	0.197309	-2.050728	0.040293471	0.346827292
QDPR	-0.573674	0.279976	-2.049009	0.040461231	0.347956971
NKX6.3	-1.90625	0.930609	-2.048389	0.040521889	NA
OCIAD1	0.159066	0.077663	2.048155	0.040544815	0.347956971
CELF2	-0.224732	0.109726	-2.048125	0.040547756	0.347956971
DIPK1A	0.380963	0.186009	2.048094	0.040550827	0.347956971
AKR7A2	-0.208159	0.101646	-2.047887	0.040571074	0.347956971
ATP2B1.AS1	-0.28007	0.136793	-2.047403	0.040618514	0.348112673
ZC3H15	0.122926	0.0601	2.045369	0.040818481	0.349574413
RIOK1	0.286957	0.140317	2.045067	0.040848279	0.34957775
NDUFS7	0.180358	0.088236	2.044043	0.040949279	0.350009595
RBM8A	0.121729	0.059563	2.043705	0.040982701	0.350009595
NTPCR	0.222213	0.108764	2.043072	0.041045282	0.350009595
HIVEP1	-0.381666	0.186817	-2.042992	0.041053253	0.350009595
TEX2	-0.906359	0.44376	-2.042453	0.041106597	0.350009595
IFNAR2	0.209682	0.102665	2.042395	0.041112413	0.350009595
EP400P1	-0.386763	0.189385	-2.042208	0.041130875	0.350009595
ERN1	-0.918301	0.449668	-2.042174	0.041134298	0.350009595
WDR60	-0.42057	0.206002	-2.041585	0.041192687	0.350255699
ESYT1	-0.256525	0.125725	-2.040368	0.041313707	0.351033622
DIP2C	-0.977516	0.479331	-2.039334	0.041416749	0.351140548
DHRS4.AS1	-0.361526	0.177304	-2.039016	0.041448436	0.351140548
MAMSTR	0.648895	0.318305	2.038595	0.041490481	0.351140548
TCTN1	-0.618864	0.303608	-2.038365	0.041513425	0.351140548
TRIB1	0.608794	0.298687	2.038234	0.04152654	0.351140548
TASP1	0.351618	0.172535	2.037951	0.041554823	0.351140548
EHMT1	-0.286459	0.140594	-2.037493	0.041600671	0.351140548
SLC12A6	0.214551	0.105311	2.037315	0.041618464	0.351140548
SOGA1	-0.455843	0.223796	-2.036868	0.041663303	0.351140548
USF3	-0.269251	0.132194	-2.03679	0.041671106	0.351140548
RPS2	-0.368212	0.180789	-2.036692	0.041680904	0.351140548
ZC3H6	-0.428493	0.210391	-2.03665	0.041685079	0.351140548
AMT	0.798641	0.392203	2.036296	0.041720655	0.351140548
COX16	0.25764	0.126536	2.036105	0.04173985	0.351140548
TRIB2	-0.733113	0.36014	-2.035632	0.041787351	0.35129154
ENSG00000272004	1.124426	0.552502	2.035152	0.041835627	NA
CAPZB	0.146661	0.072067	2.035057	0.041845131	0.351528671
OAT	0.405567	0.199347	2.034478	0.041903447	0.351769969
AZIN1.AS1	-1.095852	0.53899	-2.033157	0.042036646	0.352503521
SUCLG2	-0.253852	0.124864	-2.033024	0.042050138	0.352503521
NAPB	0.376976	0.185483	2.032404	0.042112819	0.352780182

RNASE6	-0.989079	0.4868	-2.031797	0.042174255	0.352978843
LINC01869	-1.154229	0.568143	-2.031583	0.042195923	0.352978843
SLC25A32	0.285056	0.140366	2.030798	0.042275524	0.353396031
ZNF79	-0.807044	0.397462	-2.030494	0.042306362	0.353405291
BAG6	0.211423	0.104176	2.029485	0.042408957	0.353850809
ENSG00000246528	0.895395	0.441268	2.02914	0.042444037	0.353850809
RBM41	-0.245168	0.120865	-2.028445	0.042514891	0.353850809
IKZF4	-1.263898	0.623105	-2.028386	0.042520891	0.353850809
ENSG00000228106	0.248537	0.122537	2.028262	0.042533562	0.353850809
TP53INP1	-0.328271	0.161852	-2.028215	0.042538303	0.353850809
TBCK	-0.375747	0.185389	-2.026807	0.042682143	0.354214083
TAP1	0.212508	0.104853	2.02672	0.042691048	0.354214083
ENSG00000238260	1.101881	0.543707	2.026608	0.042702556	0.354214083
CFAP298	0.223407	0.110237	2.026604	0.042702905	0.354214083
CREM	-0.463104	0.228573	-2.026068	0.042757862	0.354214083
ERO1A	-0.326673	0.161239	-2.026024	0.042762295	0.354214083
PRX	1.190932	0.587836	2.025958	0.042769101	NA
HLA.DRA	-0.306756	0.151438	-2.025622	0.042803556	0.354214083
TRPS1	-0.388151	0.191636	-2.025458	0.042820361	0.354214083
ABRAXAS2	0.260172	0.128517	2.024407	0.042928262	0.354266099
EIF2S1	0.247317	0.122179	2.024228	0.042946735	0.354266099
NDUFS1	0.310983	0.153634	2.02418	0.042951618	0.354266099
ARMH1	-0.716618	0.354113	-2.023697	0.043001363	0.354266099
TAPT1	-0.451551	0.223151	-2.023522	0.04301932	0.354266099
NSMF	-0.594375	0.293812	-2.022975	0.043075715	0.354266099
GOLGA6L9	-0.936386	0.462911	-2.022823	0.043091418	0.354266099
ZDHHC13	-0.331561	0.163963	-2.022174	0.043158403	0.354266099
ODF2L	-0.193263	0.095581	-2.021982	0.043178205	0.354266099
RPL21	-0.188583	0.093268	-2.021953	0.043181162	0.354266099
POGK	-0.230507	0.114003	-2.021929	0.043183638	0.354266099
SND1	-0.373334	0.184643	-2.021923	0.043184283	0.354266099
CA13	-0.589883	0.291959	-2.020433	0.043338545	0.354878137
YJEFN3	0.533388	0.264002	2.020393	0.043342615	0.354878137
EGLN2	-0.173496	0.085881	-2.020191	0.043363564	0.354878137
ERCC4	-0.354318	0.175432	-2.019684	0.043416143	0.354878137
NBN	0.260381	0.128943	2.019349	0.043450907	0.354878137
CAMSAP1	-0.452306	0.224041	-2.018853	0.043502492	0.354878137
MRPL33	0.159692	0.079103	2.018782	0.043509868	0.354878137
ATF7IP	-0.292289	0.144787	-2.018752	0.04351299	0.354878137
KCTD17	-0.346442	0.171624	-2.018612	0.043527578	0.354878137
DHX15	0.251035	0.124488	2.01654	0.043743541	0.35639443
SLC33A1	-0.341214	0.169432	-2.013871	0.044023097	0.358426409
MRPL24	0.279542	0.138851	2.013255	0.044087862	0.358569801
CCDC92	-0.360833	0.17924	-2.013129	0.044101039	0.358569801
ENSG00000226266	-0.95921	0.476573	-2.012725	0.044143543	0.358670055
MICAL1	-0.294233	0.146217	-2.012304	0.044187904	0.358785253
CRNKL1	0.27215	0.135358	2.010587	0.044369135	0.360010861

ENSG00000254802	0.62573	0.311311	2.009984	0.044432885	0.360026991
MIR222HG	0.940383	0.467911	2.009748	0.044457844	0.360026991
NUFIP2	-0.247482	0.12316	-2.009441	0.044490353	0.360026991
FOS	-0.865222	0.430582	-2.009423	0.044492273	0.360026991
SLC25A1	-0.314987	0.156807	-2.008754	0.044563273	0.360356208
USP15	-0.159488	0.079456	-2.007239	0.044724259	0.361192081
C1GALT1C1	0.375574	0.187113	2.007209	0.044727412	0.361192081
GUCY2C	-0.441528	0.220146	-2.005609	0.044898007	0.362323567
IDS	-0.157463	0.078576	-2.003956	0.045074788	0.363426646
OAS3	0.557196	0.278075	2.003759	0.045095844	0.363426646
SMARCD1	-0.30328	0.151394	-2.003254	0.04515005	0.363616969
WDR33	0.184703	0.092262	2.00195	0.045290078	0.364497733
RPS3A	-0.168451	0.084225	-2.000004	0.045499791	0.36539207
KLHL28	-0.264663	0.132353	-1.999678	0.045535083	0.36539207
ENSG00000279278	0.447282	0.223683	1.999627	0.045540598	0.36539207
GBA2	-0.302281	0.151188	-1.999371	0.045568247	0.36539207
ZFYVE16	-0.26293	0.131514	-1.999256	0.045580677	0.36539207
USF2	-0.206555	0.10333	-1.99898	0.045610511	0.36539207
ANKZF1	-0.184065	0.092082	-1.998926	0.045616373	0.36539207
SLC9A9	0.456931	0.228668	1.998224	0.045692379	0.365716514
TMEM183A	-0.158244	0.07921	-1.997765	0.045742155	0.365716514
BTG3	0.214225	0.107236	1.9977	0.045749176	0.365716514
CD82	-0.193488	0.096885	-1.99709	0.045815358	0.365999436
LY86.AS1	-1.557779	0.780033	-1.997067	0.04581787	NA
SMAD2	-0.206986	0.103672	-1.996539	0.045875324	0.366232352
UBLCP1	-0.280951	0.140754	-1.99604	0.045929519	0.366418923
ENTPD7	-2.008404	1.006294	-1.995841	0.045951189	NA
ENSG00000272918	-0.478537	0.239788	-1.995668	0.045970114	0.366496812
SLC48A1	-0.320201	0.160499	-1.995033	0.046039276	0.366802195
XYLT2	-0.47538	0.238345	-1.994508	0.046096618	0.367013056
ENSG00000224905	-1.401524	0.704032	-1.990712	0.046512606	NA
FAM107B	0.19089	0.095903	1.990437	0.04654277	0.370317208
ENSG00000273156	-0.601679	0.302398	-1.989693	0.046624796	0.370634809
MRTO4	0.228596	0.114901	1.989509	0.046645047	0.370634809
TLK1	-0.172144	0.08661	-1.987575	0.046858764	0.372084253
GLYCTK	0.4753	0.239288	1.98631	0.046998933	0.37294814
TANC2	-0.779969	0.392751	-1.98591	0.047043291	0.373051103
CHD4	-0.169751	0.08553	-1.984702	0.047177649	0.373867143
CCNL2	-0.191668	0.096589	-1.984363	0.047215433	0.373917289
TANGO2	-0.341974	0.172375	-1.983891	0.047268048	0.374084741
XPC	0.173153	0.087312	1.983152	0.047350421	0.374487326
ANKMY1	0.377909	0.19064	1.982317	0.047443733	0.374975835
HACD1	-0.976264	0.492558	-1.982027	0.047476184	0.374982987
SRP68	0.268289	0.135444	1.980811	0.047612427	0.375773158
AGRN	-0.673912	0.340262	-1.980568	0.047639788	0.375773158
ZMYM6	-0.30643	0.15474	-1.980289	0.047671063	0.375773158
ENSG00000237310	0.540441	0.272971	1.97985	0.04772034	0.375912317

AMD1	-0.24024	0.121422	-1.978546	0.047867106	0.376818733
ENSG00000245522	0.656337	0.331914	1.977431	0.047992987	0.377428826
LINC01800	-1.109225	0.560981	-1.977297	0.048008108	0.377428826
FAM160B2	-0.344348	0.174202	-1.976718	0.048073533	0.377693382
ABRAXAS1	-0.182855	0.092618	-1.974289	0.048348843	0.379312316
BCLAF3	-0.521934	0.26441	-1.973956	0.048386783	0.379312316
DIAPH1	-0.258306	0.130883	-1.973568	0.048430842	0.379312316
STAG2	0.175673	0.08902	1.973404	0.048449517	0.379312316
LFNG	0.286662	0.145267	1.973345	0.048456314	0.379312316
PABPC3	-0.708831	0.359227	-1.973209	0.048471702	0.379312316
GLIPR1	-0.223762	0.113419	-1.972882	0.048508988	0.379312316
PHF8	0.338162	0.171425	1.972655	0.048534873	0.379312316
RALGPS1	-0.411049	0.208429	-1.972131	0.048594664	0.379530075
CD68	-0.481184	0.244029	-1.971834	0.048628608	0.379545803
ZNF165	0.46312	0.234903	1.971537	0.048662538	0.379561413
NDUFA5	0.130256	0.066082	1.971131	0.048708916	0.379659431
FAM122A	0.210403	0.106775	1.970533	0.048777322	0.379659431
CPM	0.477639	0.242416	1.970329	0.048800675	0.379659431
CCDC71L	-0.451899	0.229387	-1.970033	0.048834557	0.379659431
ZNF227	0.299428	0.151992	1.970031	0.0488348	0.379659431
ZNF493	-0.200018	0.101578	-1.969112	0.048940236	0.380230446
XPO5	0.438253	0.222609	1.968712	0.04898619	0.380332696
LMF2	-0.196861	0.100019	-1.968229	0.049041704	0.380332696
RBM28	0.230145	0.116934	1.968162	0.049049384	0.380332696
MED29	0.188213	0.095649	1.967743	0.049097563	0.380458106
GSTM4	-0.501431	0.255025	-1.966203	0.04927522	0.381586016
ZBTB3	0.489601	0.24905	1.965873	0.04931333	0.381632523
LRRFIP1	-0.206278	0.104952	-1.965456	0.049361455	0.381756415

Cluster 4	log2FC	lfcSE	stat	pvalue	padj
VPREB3	2.019106	0.072711	27.769031	< 2.22e-16	< 2.22e-16
PLAAT4	1.838477	0.168514	10.909953	< 2.22e-16	< 2.22e-16
CD38	2.041542	0.242906	8.404655	< 2.22e-16	1.63E-13
AHI1	1.6348	0.213267	7.665507	1.78E-14	5.07E-11
MS4A1	-0.681183	0.102817	-6.625227	3.47E-11	7.90E-08
DENND6B	1.924781	0.295721	6.508777	7.58E-11	1.31E-07
RRBP1	1.118218	0.172054	6.499241	8.07E-11	1.31E-07
MRPS6	0.98125	0.151477	6.477872	9.30E-11	1.32E-07
GRN	1.188852	0.192162	6.18673	6.14E-10	7.77E-07
SEPTIN9	0.623549	0.106302	5.865849	4.47E-09	5.09E-06
CD1D	2.324307	0.413927	5.615264	1.96E-08	2.03E-05
DPEP2	1.100664	0.200651	5.485457	4.12E-08	3.91E-05
SIGIRR	0.996736	0.186979	5.330728	9.78E-08	8.57E-05
SLC12A4	3.593533	0.687828	5.224465	1.75E-07	0.000142061
AGO1	1.414221	0.272122	5.197005	2.03E-07	0.000153744
DUS2	1.411895	0.279878	5.044688	4.54E-07	0.000301683
DBNL	0.727911	0.144438	5.039591	4.67E-07	0.000301683
TRABD	0.647435	0.128577	5.035387	4.77E-07	0.000301683
SMAD3	1.343852	0.268596	5.003248	5.64E-07	0.000337849
ASB2	3.76393	0.755296	4.983386	6.25E-07	0.000355737
BTG2	-0.355278	0.071452	-4.972278	6.62E-07	0.000358803
RARA.AS1	3.047223	0.621406	4.903755	9.40E-07	0.000486648
DDIT4	1.723666	0.352862	4.884815	1.04E-06	0.000512543
P2RX5	0.575582	0.131412	4.379999	1.19E-05	0.005630863
FAM3C	0.754908	0.173038	4.362661	1.28E-05	0.005757808
CABLES1	-1.88776	0.433206	-4.357647	1.31E-05	0.005757808
VPREB1	3.446085	0.796029	4.329094	1.50E-05	0.006314477
IRAK2	1.78763	0.414715	4.310504	1.63E-05	0.006579526
EVI2B	0.472179	0.109701	4.304233	1.68E-05	0.006579526
MCM5	0.862363	0.203026	4.247552	2.16E-05	0.00820317
RCBTB2	-2.644794	0.628014	-4.211362	2.54E-05	0.009323965
GNG3	-2.84684	0.688089	-4.137312	3.51E-05	0.01250427
APOL3	1.053283	0.25726	4.094232	4.24E-05	0.014615757
AGPAT5	0.772669	0.189625	4.074713	4.61E-05	0.015086889
SLC5A3	0.983941	0.241909	4.067407	4.75E-05	0.015086889
NEIL1	-0.648514	0.159472	-4.066633	4.77E-05	0.015086889
CD79B	0.281948	0.069999	4.027874	5.63E-05	0.01732164
FAM117A	0.955156	0.238447	4.005729	6.18E-05	0.017991356
DGKD	-0.658789	0.164579	-4.002875	6.26E-05	0.017991356
H3F3A	0.353311	0.088463	3.993899	6.50E-05	0.017991356
TTC39C	-0.947967	0.237479	-3.991789	6.56E-05	0.017991356
CHST15	1.032448	0.258825	3.988974	6.64E-05	0.017991356
MCTP2	-0.936432	0.235139	-3.982456	6.82E-05	0.018062122
GSTP1	0.320295	0.080715	3.96823	7.24E-05	0.018739005
RAB37	1.052529	0.268594	3.918661	8.90E-05	0.022531676
PLAAT3	2.539781	0.652036	3.895155	9.81E-05	0.024232958

STK17B	-0.421192	0.108261	-3.890539	0.00010002	0.024232958
PNOC	1.045482	0.27215	3.841565	0.00012225	0.028701033
LINC01353	-1.776717	0.4628	-3.839063	0.00012351	0.028701033
RMDN2	2.010232	0.529096	3.799368	0.00014507	0.033037293
SPOCK2	0.624823	0.166368	3.755667	0.00017288	0.038467374
TMC8	0.861967	0.229756	3.751663	0.00017567	0.038467374
CD24	-0.498975	0.133813	-3.7289	0.00019232	0.041319156
ISG20	-0.41323	0.111768	-3.697224	0.00021797	0.045963454
P2RX5.TAX1BP3	2.092047	0.567045	3.689383	0.0002248	0.046541448
HHIP.AS1	-2.512868	0.685064	-3.668079	0.00024438	0.049692002
MAP3K1	0.707603	0.194228	3.643161	0.00026931	0.053800529
ZNF3	1.02196	0.281769	3.626944	0.0002868	0.056070273
XYLT1	1.251459	0.345885	3.618131	0.00029674	0.056070273
ZNF730	-2.444933	0.676102	-3.616217	0.00029894	0.056070273
CYBC1	-0.359055	0.099324	-3.614983	0.00030037	0.056070273
PAOX	1.258947	0.34892	3.60812	0.00030842	0.056645434
ZNF684	-1.603205	0.448503	-3.574569	0.00035081	0.063406663
ETFB	0.647252	0.181402	3.568045	0.00035966	0.063990415
C7orf50	-0.343987	0.096691	-3.557585	0.00037428	0.06556805
GBP4	0.716485	0.202228	3.542955	0.00039567	0.066412
RIMS3	-2.958722	0.835496	-3.541276	0.0003982	0.066412
TRBC2	-0.295395	0.083458	-3.539423	0.000401	0.066412
ENSG00000260778	-1.781111	0.503354	-3.538488	0.00040243	0.066412
RAPGEF5	-2.043015	0.582975	-3.504463	0.00045753	0.074426844
IL2RA	-1.418319	0.407653	-3.479235	0.00050285	0.080646883
CD3G	-4.41776	1.276568	-3.460653	0.00053887	NA
KMT2E	-0.281799	0.081484	-3.458323	0.00054355	0.085963929
ACP5	0.785353	0.22794	3.445445	0.00057012	0.088931041
MIR3142HG	-3.230923	0.940713	-3.434548	0.00059354	NA
ATP6V0A1	1.119726	0.327725	3.416666	0.00063393	0.097547961
AP3S1	0.540275	0.158578	3.406998	0.00065682	0.09878158
PPP1R18	0.430009	0.126364	3.402938	0.00066665	0.09878158
DEF8	-0.797763	0.234471	-3.402399	0.00066797	0.09878158
RUBCN	-0.492578	0.145013	-3.396788	0.00068182	0.099536627
FOXO3	1.425621	0.420656	3.389044	0.00070137	0.101094586
PFKL	-0.453355	0.133915	-3.385407	0.00071073	0.101163287
MAPKAPK2	1.000857	0.297038	3.369452	0.00075318	0.105882048
POU2AF1	0.429354	0.128636	3.337752	0.00084459	0.117284695
ZNF638	-0.43517	0.130751	-3.328229	0.000874	0.11990662
GPER1	-1.914	0.578057	-3.311094	0.00092932	0.125978078
MPEG1	0.775383	0.234481	3.306814	0.00094364	0.12641398
ENSG00000257815	-0.72811	0.220488	-3.302265	0.00095908	0.126988273
DCAF12	-2.5553	0.777234	-3.287685	0.00101015	0.132213048
PARP2	-0.977608	0.297773	-3.283065	0.00102685	0.132872257
MDH2	0.469474	0.143337	3.27531	0.00105546	0.135039774
USP46.AS1	-2.661087	0.812849	-3.273778	0.0010612	NA
CERS4	-0.54055	0.165506	-3.266036	0.00109064	0.137990711

LCAT	1.894977	0.585315	3.237531	0.00120569	0.150870253
SNAP23	0.454782	0.140879	3.228169	0.00124585	0.153235778
GALNT2	0.625639	0.194185	3.221864	0.0012736	0.153235778
MPZL1	-0.935655	0.290427	-3.221651	0.00127454	0.153235778
SPRY1	0.590263	0.183267	3.22078	0.00127842	0.153235778
RNF207	1.794268	0.56012	3.203361	0.00135833	0.160685825
LSM7	-0.394039	0.123093	-3.201151	0.0013688	0.160685825
ABI3	0.737404	0.230826	3.194638	0.00140007	0.162574673
RPS19	0.269826	0.084535	3.191891	0.00141345	0.162574673
ENSG00000183308	-2.16586	0.679947	-3.185339	0.00144585	0.164638386
NKX6.3	-1.891098	0.59524	-3.177034	0.0014879	0.167090843
SLC38A6	-1.359711	0.428213	-3.175317	0.00149673	0.167090843
TNFRSF4	-3.254171	1.026025	-3.171629	0.00151587	NA
UBAP2L	-0.628907	0.198788	-3.163703	0.00155776	0.172215367
SNX18	1.386266	0.438825	3.159039	0.0015829	0.173312614
RABGAP1L	0.495643	0.157317	3.150612	0.00162929	0.176692366
BCLAF1	-0.357172	0.113515	-3.146482	0.00165248	0.177516512
RNGTT	-0.845863	0.270113	-3.131513	0.00173908	0.183292494
GPX1	0.289211	0.092413	3.129533	0.00175084	0.183292494
RINL	0.698354	0.223206	3.12874	0.00175557	0.183292494
GPT2	1.218762	0.389851	3.12623	0.00177063	0.183292494
TOX2	-2.205178	0.705397	-3.126151	0.00177111	NA
NOB1	0.643095	0.206111	3.120133	0.0018077	0.184807357
ENSG00000275580	-2.236424	0.717147	-3.118503	0.00181772	0.184807357
MARCHF1	-0.41155	0.132125	-3.114853	0.00184036	0.185453279
TMEM88	-2.481371	0.79668	-3.114641	0.00184169	NA
SLC25A5	-0.241472	0.078265	-3.085296	0.0020335	0.203117672
ZNF436	-1.750639	0.570301	-3.069673	0.00214293	0.212187543
CD226	-2.984439	0.972486	-3.068875	0.00214866	NA
ATXN10	0.649424	0.211973	3.063707	0.00218613	0.214524251
FOXP1	-0.216662	0.070776	-3.061242	0.00220421	0.214524251
RNF130	1.255243	0.411304	3.05186	0.00227429	0.219468462
FAM167A	1.831743	0.601001	3.047822	0.00230507	0.220569726
TMEM52	-1.929527	0.634287	-3.042038	0.00234982	0.22141415
PPP3CC	-0.497331	0.163506	-3.041659	0.00235278	0.22141415
TBKBP1	-2.757435	0.912457	-3.02199	0.00251119	NA
LINC02202	-2.177106	0.720748	-3.020619	0.00252258	0.235448087
SMIM20	0.799289	0.264915	3.01716	0.00255155	0.23621573
C15orf39	1.30374	0.432834	3.012102	0.00259445	0.237085769
CD79A	0.324997	0.107931	3.011151	0.00260259	0.237085769
FAM207A	1.174581	0.391941	2.996831	0.00272802	0.246539597
CERK	1.040938	0.347995	2.991248	0.0027784	0.249115052
ZNF540	-0.993128	0.333501	-2.977883	0.00290247	0.258206527
PXK	0.639143	0.215094	2.971464	0.00296384	0.261621821
ENSG00000272368	-0.810855	0.273699	-2.962581	0.00305072	0.267081353
ATP6V0D1	-0.472731	0.159789	-2.95847	0.0030917	0.267081353
SERPINB1	0.494473	0.167163	2.958037	0.00309605	0.267081353

MYO9B	0.728603	0.247297	2.946271	0.0032163	0.275368807
EVL	0.35031	0.119585	2.929381	0.00339638	0.288616166
DYNLL2	-0.817013	0.279438	-2.923774	0.00345816	0.291689435
CCNB1	-1.35297	0.464198	-2.91464	0.003561	0.294017078
SYNGAP1	1.700978	0.583599	2.914635	0.00356104	0.294017078
TCL1A	-0.463623	0.159078	-2.914445	0.00356322	0.294017078
AGAP3	-2.844526	0.977131	-2.911099	0.0036016	NA
ST3GAL1	0.919136	0.315825	2.910269	0.00361117	0.295830491
TBRG1	-0.4538	0.156137	-2.906431	0.00365578	0.297345068
DDX17	-0.251451	0.086638	-2.90232	0.0037041	0.299138998
QSOX2	-0.728164	0.251368	-2.896805	0.00376984	0.301780632
HSH2D	0.344468	0.119022	2.894152	0.00380184	0.301780632
CYTH3	-1.071464	0.37037	-2.892958	0.00381632	0.301780632
LBH	-0.355331	0.123007	-2.88871	0.00386825	0.303777898
HCK	-0.990814	0.343814	-2.88183	0.00395374	0.305685353
CD37	-0.150251	0.052148	-2.881243	0.00396111	0.305685353
ASTE1	-0.713552	0.247736	-2.880291	0.00397308	0.305685353
C3orf52	-2.244779	0.783844	-2.863809	0.0041858	NA
SUSD6	-0.55667	0.194791	-2.857787	0.00426607	0.326025125
ENSG00000271204	0.616583	0.218022	2.828083	0.00468276	0.355483992
RPL18A	0.238805	0.084637	2.821527	0.00477956	0.360429428
CD109	-3.466531	1.22973	-2.818937	0.00481829	NA
SYNGR2	0.417417	0.148279	2.815086	0.00487642	0.365314349
AGPAT2	-0.462852	0.164783	-2.808861	0.00497172	0.370019207
FCMR	0.34628	0.123474	2.804481	0.00503976	0.372647831
PRR7	-1.174821	0.420613	-2.793115	0.00522031	0.383507423
MPG	0.455178	0.163256	2.78812	0.00530149	0.38697453
NFATC3	0.487519	0.17501	2.785663	0.00534184	0.387436308
MRNIP	0.527187	0.190122	2.772884	0.0055562	0.40043301
ENSG00000275636	-2.562055	0.926709	-2.764682	0.00569783	0.40728113
BRPF1	-0.83243	0.301249	-2.763258	0.00572275	0.40728113
ARNT	-0.71353	0.259353	-2.751193	0.00593787	0.417969599
TADA2A	-0.859186	0.312437	-2.749948	0.00596048	0.417969599
RAB11B	-0.326395	0.118745	-2.748709	0.00598306	0.417969599
ENSG00000258168	-2.974757	1.083558	-2.745359	0.00604447	NA
MSI2	-0.454049	0.165545	-2.742758	0.00609255	0.423023871
ENSG00000249790	-2.219031	0.809382	-2.741637	0.00611338	NA
STXBP5	-1.28726	0.46977	-2.740192	0.00614034	0.423757558
ALG2	-0.660249	0.24156	-2.733266	0.00627096	0.42861821
JAML	-2.666264	0.976304	-2.730976	0.0063147	0.42861821
PBX3	-0.90763	0.332403	-2.730507	0.00632369	0.42861821
GRK5	-0.647486	0.237309	-2.728448	0.00636332	0.428751874
LGALS4	-1.597368	0.585869	-2.726493	0.00640113	0.428762627
SLC39A13	-0.905211	0.332757	-2.720335	0.00652157	0.434275725
COL8A1	-2.078186	0.765496	-2.714823	0.00663112	NA
SIDT2	-0.66648	0.245501	-2.714773	0.00663213	0.439070143
EIF3K	-0.178443	0.065818	-2.711171	0.0067046	0.440545333

NPW	-2.355475	0.869486	-2.709044	0.00674774	NA
ENSG00000198106	-0.40619	0.149999	-2.707946	0.0067701	0.440545333
DTX4	1.320224	0.48754	2.707928	0.00677048	0.440545333
LINC02362	-2.428144	0.897015	-2.706915	0.00679116	NA
UMPS	1.432644	0.530164	2.702266	0.00688685	0.445571622
RUNX3	0.685018	0.253816	2.698876	0.00695741	0.446471436
HIST1H2AK	-1.880797	0.69715	-2.697836	0.00697918	0.446471436
MCL1	0.395912	0.146855	2.695932	0.00701921	0.446523503
CR2	1.259428	0.467527	2.693809	0.00706406	0.446880002
NIPSNAP1	1.005815	0.373954	2.689675	0.00715217	0.449559663
BSPRY	-2.145912	0.797949	-2.689285	0.00716053	NA
ZWINT	-1.939284	0.721425	-2.688128	0.00718537	0.449559663
TLK1	-0.368437	0.13733	-2.682863	0.0072995	0.452800386
NEK1	1.277288	0.476355	2.681378	0.00733197	0.452800386
CNTRL	-0.371317	0.138551	-2.68001	0.007362	0.452800386
ZNF440	1.96557	0.733844	2.678457	0.00739623	0.452800386
GGT1	-2.271364	0.8491	-2.675026	0.00747234	NA
BYSL	-1.140473	0.426432	-2.674454	0.0074851	0.455790532
CHST12	0.563546	0.210874	2.672432	0.00753037	0.45610811
ITGB2.AS1	-2.542449	0.951727	-2.671407	0.0075534	NA
LINC02576	-1.151109	0.431283	-2.669033	0.00760699	0.456300385
TBC1D9	0.846599	0.317228	2.668738	0.00761369	0.456300385
SLC29A1	-2.043063	0.766071	-2.666937	0.0076546	0.456350475
SQSTM1	0.355099	0.13329	2.664104	0.00771938	0.456622353
PHF20L1	-0.390421	0.146596	-2.663234	0.00773936	0.456622353
TBL1X	-0.724496	0.272609	-2.657634	0.00786913	0.457414698
SMS	0.59855	0.225335	2.656265	0.00790115	0.457414698
ENSG00000255224	-1.713427	0.645095	-2.656084	0.00790539	0.457414698
CD72	0.463777	0.17474	2.654104	0.00795193	0.457414698
PSMB10	0.405134	0.152649	2.654031	0.00795364	0.457414698
CYB5RL	-1.122885	0.424216	-2.646965	0.00812179	0.464737579
SLC2A5	0.682062	0.257909	2.644587	0.00817906	0.46567457
PIK3R5	1.707172	0.645999	2.642683	0.00822519	0.46597124
JDP2	1.799591	0.6818	2.639469	0.00830359	0.46777391
IGHV3.15	-1.526894	0.578803	-2.63802	0.00833917	0.46777391
LINC02210.CRHR1	-2.097084	0.795675	-2.635603	0.0083988	NA
PPT1	0.715946	0.272065	2.631527	0.0085002	0.472787049
CDKN3	-1.906865	0.724748	-2.631073	0.00851158	0.472787049
PMEPA1	-0.570898	0.217207	-2.628362	0.00857971	0.474257852
SLC25A53	-0.808965	0.308331	-2.623692	0.00869824	0.478487389
KNL1	1.612216	0.61499	2.621534	0.00875351	0.479212516
MZB1	-0.509555	0.194846	-2.615173	0.00891822	0.485893672
RAB20	1.7434	0.668046	2.609701	0.00906213	0.490129536
E2F5	-0.50735	0.194466	-2.60894	0.00908232	0.490129536
PON2	-1.799118	0.690023	-2.607332	0.0091251	0.490129536
MRPL27	0.566745	0.217529	2.605374	0.00917741	0.490625151
VHL	0.389554	0.150106	2.595198	0.00945364	0.499689112

GBP2	0.567616	0.218974	2.592169	0.00953729	0.499689112
RARA	0.552616	0.213415	2.589398	0.00961438	0.499689112
RAMP2.AS1	-1.744364	0.673713	-2.58918	0.00962048	0.499689112
ABHD11	-0.712902	0.275492	-2.587744	0.00966068	0.499689112
ENSG00000263470	-2.174558	0.840526	-2.587138	0.00967769	NA
TMEM134	-0.313823	0.121344	-2.586227	0.0097033	0.499689112
C2orf42	-0.782734	0.302866	-2.584427	0.0097541	0.499689112
LEPR	-1.927145	0.74612	-2.582888	0.0097977	0.499689112
SCYL2	-0.73937	0.286283	-2.582653	0.00980439	0.499689112
RFC4	0.905059	0.350529	2.581983	0.00982344	0.499689112
DNAJB9	-0.428769	0.16617	-2.580301	0.00987143	0.499689112
MYCBP2	0.292748	0.113531	2.578564	0.0099212	0.499689112
PIGN	-1.190063	0.461909	-2.576398	0.00998357	0.499689112
ENSG00000271976	-1.490505	0.579244	-2.573191	0.01007657	0.499689112
ACAP2	-0.384998	0.149626	-2.573067	0.01008018	0.499689112
TAF11	-0.470879	0.183045	-2.572479	0.01009731	0.499689112
NMT2	-0.440777	0.171398	-2.571657	0.0101213	0.499689112
NOTCH2	0.623468	0.242657	2.569336	0.01018935	0.499689112
HERC6	-0.877929	0.341813	-2.568448	0.01021549	0.499689112
ENSG00000251364	1.729416	0.673412	2.568139	0.0102246	0.499689112
ENSG00000260735	-1.350401	0.526626	-2.56425	0.01033989	0.501817629
ANXA2R	-0.555091	0.216618	-2.562535	0.0103911	0.501817629
ZNF438	-1.33029	0.519265	-2.561873	0.01041095	0.501817629
DDX42	-0.554152	0.216402	-2.560757	0.01044444	0.501817629
PATL2	0.635849	0.248476	2.559	0.01049737	0.502241679
PLEKHO1	0.395129	0.154596	2.555879	0.01059199	0.504648264
SLC35B1	-0.614564	0.240819	-2.55197	0.01071156	0.508218837
AGAP5	-1.371699	0.538335	-2.54804	0.01083302	0.511848878
LOR	-2.704562	1.062101	-2.546426	0.01088322	NA
WIPF1	-0.251667	0.098894	-2.544799	0.01093407	0.513677351
PI4K2A	-1.218819	0.479113	-2.54391	0.01096194	0.513677351
KCNJ5	-1.768801	0.696222	-2.54057	0.01106721	0.516484849
BCL2L11	1.23776	0.487804	2.537413	0.01116752	0.517533917
CARD19	-0.664605	0.262144	-2.535268	0.01123612	0.517533917
CHIT1	-1.464909	0.57783	-2.535188	0.01123869	0.517533917
SNHG10	-0.621024	0.24508	-2.533959	0.01127819	0.517533917
BPGM	-0.693117	0.273661	-2.532757	0.01131694	0.517533917
SRRT	0.689045	0.272348	2.53002	0.01140559	0.518013266
NFXL1	-0.935862	0.369961	-2.529626	0.0114184	0.518013266
ENSG00000231105	-2.215406	0.87612	-2.528658	0.01144997	NA
ST6GALNAC6	0.59584	0.236148	2.523166	0.01163036	0.525535303
SIGLEC10	1.001494	0.397248	2.521079	0.01169955	0.525845192
SLC43A2	0.349748	0.138779	2.520177	0.01172958	0.525845192
GRINA	0.572236	0.2272	2.518641	0.01178088	0.526074022
NHEJ1	-1.883261	0.748161	-2.517186	0.01182963	NA
GZMM	-3.05829	1.215316	-2.516457	0.01185412	NA
CTBP2	-2.631201	1.04616	-2.515104	0.01189972	NA

CYTIP	-0.180013	0.071598	-2.514224	0.01192945	0.530627594
LRRC3	-1.606058	0.639477	-2.51152	0.01202124	0.532629995
NEURL4	-1.278809	0.509902	-2.507953	0.01214329	0.53595203
SLC8B1	-0.685661	0.273596	-2.506112	0.0122067	0.5366708
ANAPC16	0.218059	0.08713	2.502673	0.01232593	0.537621442
MTERF3	-0.691389	0.276625	-2.499373	0.01244133	0.537621442
ENSG00000282951	-1.775605	0.710493	-2.499117	0.01245031	0.537621442
CENPE	-1.85636	0.742906	-2.498783	0.01246205	0.537621442
IER3	1.783737	0.713861	2.498717	0.0124644	0.537621442
NXPH4	-2.141789	0.857818	-2.496787	0.01253243	NA
MBD6	1.269334	0.508608	2.495703	0.01257078	0.540135859
ENSG00000262049	-0.662291	0.266119	-2.4887	0.01282112	0.540135859
USP13	-1.255952	0.504822	-2.487912	0.01284955	0.540135859
C1QBP	0.286962	0.115351	2.487731	0.01285608	0.540135859
LSP1	0.327184	0.131523	2.487652	0.01285896	0.540135859
CXXC5	-0.30879	0.124223	-2.485771	0.01292713	0.540135859
TLR1	-0.770746	0.310143	-2.485131	0.01295036	0.540135859
DDX46	-0.254497	0.102471	-2.483601	0.01300613	0.540135859
PPM1B	-0.448243	0.180498	-2.483371	0.01301455	0.540135859
HSPA1A	-1.885731	0.759922	-2.481479	0.01308384	0.540135859
ENSG00000188206	-0.502815	0.202753	-2.479935	0.01314065	0.540135859
LINC01800	-1.888924	0.761697	-2.479888	0.01314236	0.540135859
PBXIP1	-0.401042	0.161799	-2.478649	0.0131881	0.540135859
HDAC9	0.882529	0.356063	2.478574	0.01319086	0.540135859
FEZ2	-0.44044	0.17792	-2.475488	0.01330544	0.540135859
EPHX2	-1.925411	0.777863	-2.475258	0.01331399	0.540135859
ZNF780A	0.931144	0.376242	2.474854	0.01332908	0.540135859
GIMAP4	-2.639886	1.066905	-2.47434	0.01334825	NA
PRKCB	-0.285816	0.115605	-2.472346	0.01342295	0.540637122
ZNF791	0.342666	0.138619	2.471988	0.0134364	0.540637122
NT5DC4	-3.109179	1.258143	-2.471244	0.0134644	NA
ENSG00000233184	-0.655517	0.265483	-2.469152	0.01354336	0.54302211
SPRYD7	0.97612	0.395709	2.466765	0.01363397	0.544736783
SPAG7	-0.290466	0.117918	-2.463285	0.01376706	0.548131245
ENSG00000267317	-1.383111	0.562315	-2.459671	0.01390645	0.549544937
PRRT3.AS1	-1.716327	0.69817	-2.458323	0.01395875	0.549544937
ZER1	1.662651	0.676366	2.458211	0.0139631	0.549544937
CRIPT	0.537341	0.218665	2.457376	0.01399561	0.549544937
MIXL1	-1.824188	0.742341	-2.457344	0.01399685	NA
NDUFB3	0.332748	0.135837	2.449619	0.01430075	0.556958093
KCNN1	1.92106	0.784404	2.449069	0.01432258	0.556958093
SLX4IP	-0.706315	0.288455	-2.448611	0.01434084	0.556958093
HEATR6	1.503821	0.614542	2.447061	0.01440264	0.556958093
SSBP3	-0.897658	0.36693	-2.446403	0.01442897	0.556958093
MAST1	-1.96018	0.801618	-2.445278	0.01447403	NA
OAZ1	-0.147406	0.060327	-2.443454	0.01454744	0.558459605
MRPL15	0.664536	0.272237	2.441017	0.01464595	0.558459605

ENSG00000258572	-1.409511	0.577487	-2.440768	0.01465606	0.558459605
PPP1R15A	0.656213	0.268996	2.439494	0.01470786	0.558459605
GYPC	0.241361	0.098944	2.439365	0.01471308	0.558459605
PHF21A	-0.443835	0.182088	-2.437484	0.01478987	0.559509206
ENSG00000257275	-0.470473	0.193314	-2.433717	0.01494467	0.562066958
BCL11A	-0.278942	0.114629	-2.433438	0.0149562	0.562066958
YES1	-1.868039	0.769018	-2.429123	0.0151354	NA
LINC02391	-1.714081	0.705684	-2.428963	0.01514209	0.567180978
VSIG1	-2.510084	1.03408	-2.42736	0.01520915	NA
PFDN1	-0.326213	0.13441	-2.426996	0.01522442	0.567556563
D2HGDH	-0.844451	0.348034	-2.426344	0.01525181	0.567556563
ENSG00000268439	-1.227701	0.506693	-2.42297	0.01539421	0.57098962
CACNB1	-1.934391	0.799557	-2.419329	0.01554918	NA
SYK	-0.302703	0.125331	-2.415217	0.01572583	0.579599439
NANS	0.755806	0.312942	2.415164	0.01572813	0.579599439
ENSG00000259802	-1.923628	0.797477	-2.412142	0.01585909	0.582540168
SRSF7	-0.25287	0.104958	-2.409257	0.01598503	0.585278215
PKN3	-2.042917	0.848689	-2.407144	0.01607783	NA
CD180	0.595168	0.247401	2.405687	0.0161421	0.587786498
SEC23B	1.289462	0.53608	2.405355	0.01615677	0.587786498
FUZ	1.042957	0.434016	2.403037	0.01625954	0.588415441
ARAP2	-0.506704	0.210895	-2.402635	0.01627741	0.588415441
DMXL2	-2.255961	0.939213	-2.401969	0.01630711	NA
ATMIN	-0.721158	0.300369	-2.400907	0.01635448	0.58933053
ASNS	-0.769923	0.320877	-2.399435	0.0164204	0.589839396
L3MBTL3	-0.703059	0.293314	-2.396953	0.01653205	0.591861474
C21orf62	-0.880485	0.3675	-2.395877	0.01658065	0.591861474
ENSG00000226310	-1.794859	0.749512	-2.394703	0.01663385	NA
SREK1	-0.377354	0.157848	-2.390615	0.01682018	0.595539551
TAGLN2	0.222726	0.093249	2.38851	0.01691683	0.595539551
RANBP2	-0.530615	0.222172	-2.388309	0.0169261	0.595539551
SHARPIN	-0.376825	0.157861	-2.387068	0.01698333	0.595539551
ZNF165	1.449335	0.607317	2.386454	0.01701171	0.595539551
BANK1	-0.299113	0.125366	-2.385909	0.01703696	0.595539551
ARMH1	-1.015319	0.425597	-2.385632	0.01704978	0.595539551
ENSG00000260267	-1.123065	0.471287	-2.382973	0.01717346	0.597037336
PRPF38B	-0.24768	0.10396	-2.382457	0.01719753	0.597037336
GPBP1	-0.228193	0.095856	-2.38057	0.01728587	0.597117947
ENSG00000269399	1.635743	0.687374	2.379698	0.01732683	0.597117947
RAB39B	-0.778586	0.327267	-2.379053	0.01735717	0.597117947
ENSG00000267811	-1.800522	0.757536	-2.376812	0.01746298	0.598948651
TUSC2	-0.379092	0.159664	-2.374302	0.01758217	0.601225685
SLC16A11	1.171932	0.493833	2.373136	0.01763779	0.601321783
VNN2	0.705394	0.29787	2.368126	0.01787846	0.607707439
SNTB1	-2.357717	0.995921	-2.367373	0.01791488	NA
BCDIN3D	-0.603855	0.255254	-2.365705	0.01799576	0.608692929
AHSA2P	-0.566307	0.23942	-2.365323	0.01801436	0.608692929

YIPF2	1.167042	0.494045	2.362216	0.01816607	0.610045912
HES1	-1.084546	0.459317	-2.361215	0.01821518	0.610045912
ENSG00000261386	-0.545494	0.231121	-2.360205	0.01826485	0.610045912
ESCO1	-0.422674	0.17909	-2.360127	0.0182687	0.610045912
GTPBP2	-0.9543	0.404799	-2.357465	0.01840019	0.612640203
SNX9	0.60707	0.257914	2.353773	0.01858398	0.616705298
ENSG00000229729	-0.982885	0.417901	-2.351958	0.01867487	0.616705298
ZNF837	-0.79659	0.338959	-2.350107	0.01876802	0.616705298
HAVCR2	-1.904016	0.810777	-2.348383	0.01885513	NA
VASH1	-1.959421	0.834457	-2.34814	0.01886745	NA
TMEM115	0.797799	0.339791	2.347909	0.01887911	0.616705298
TYW1B	-1.141883	0.486483	-2.347221	0.01891404	0.616705298
ENSG00000273466	-1.763636	0.751815	-2.345839	0.01898429	0.616705298
PGD	-0.5973	0.254629	-2.345771	0.01898776	0.616705298
SNX19	-0.769842	0.328499	-2.343513	0.0191031	0.616705298
MRPS18C	-0.24835	0.106007	-2.342782	0.01914056	0.616705298
APLF	-1.227165	0.523858	-2.342551	0.01915243	0.616705298
NSMCE1	0.435625	0.186014	2.341898	0.01918598	0.616705298
JMJD1C.AS1	-2.180158	0.931096	-2.341496	0.01920662	NA
EMC8	-0.579988	0.247704	-2.341456	0.0192087	0.616705298
SNAI3.AS1	-1.18126	0.504738	-2.34034	0.0192662	0.616705298
TOP1MT	-0.599403	0.256187	-2.339712	0.01929864	0.616705298
CD47	-0.269569	0.115249	-2.339015	0.01933466	0.616705298
DSCR9	-1.823468	0.780233	-2.337081	0.01943496	NA
ENSG00000259943	-0.762833	0.326944	-2.333224	0.01963641	0.621176303
CHTF8	-0.368415	0.157906	-2.333125	0.01964161	0.621176303
CD5	-1.09205	0.468225	-2.332318	0.01968395	0.621176303
FNBP1	0.339	0.145425	2.331109	0.01974763	0.621176303
USF3	-0.389961	0.167305	-2.33084	0.01976182	0.621176303
WHAMM	0.90121	0.386773	2.330076	0.01980214	0.621176303
PRXL2A	0.769986	0.330648	2.32872	0.01987391	0.621714783
PQLC3	-0.569706	0.244788	-2.327342	0.01994708	0.62229416
TPCN1	0.635103	0.27331	2.323743	0.02013926	0.624928308
TSR2	0.428168	0.184261	2.323706	0.02014127	0.624928308
TLE4	-0.506999	0.218359	-2.321862	0.02024036	0.626296139
RNF157	-1.93675	0.83446	-2.320961	0.02028895	NA
DCXR.DT	-1.560416	0.672405	-2.32065	0.02030577	0.626617294
SOX5	-2.998396	1.292286	-2.320227	0.02032862	NA
ENSG00000268292	-1.684873	0.726772	-2.318298	0.02043315	NA
MTHFD2L	-0.562359	0.24271	-2.317005	0.02050347	0.631008009
NAA10	-0.279574	0.120804	-2.314279	0.02065243	0.632744357
ARSA	-0.79951	0.345519	-2.31394	0.02067102	0.632744357
LBX2.AS1	-2.475568	1.070149	-2.313292	0.02070657	NA
SNX21	-1.937744	0.83801	-2.312315	0.0207603	NA
ENSG00000244198	-1.486913	0.643206	-2.311722	0.02079299	0.63477152
GNG4	-3.401706	1.47199	-2.310957	0.02083524	NA
DUSP2	-1.827935	0.791022	-2.310851	0.02084106	NA

ENSG00000277369	-1.865815	0.808178	-2.308669	0.02096195	NA
ENSG00000237481	-1.732902	0.750652	-2.308528	0.02096981	NA
TBC1D16	-1.351839	0.585711	-2.30803	0.02099745	0.639299353
PPP1R14B	-0.670838	0.291082	-2.304639	0.02118681	0.643344403
TMEM206	-0.732753	0.318222	-2.30265	0.02129856	0.645017935
ENSG00000278668	-1.547737	0.672923	-2.300022	0.02144697	0.646541705
PRKAR2A.AS1	-1.653119	0.718785	-2.299881	0.02145499	NA
PDHA1	0.677676	0.294676	2.299731	0.02146347	0.646541705
TNIP2	0.58193	0.253151	2.298749	0.02151922	0.646541705
RAB5A	-0.44363	0.193379	-2.294099	0.02178483	0.650060218
LYG1	-1.62115	0.706721	-2.293904	0.02179601	0.650060218
PTMS	-1.651829	0.720303	-2.293241	0.02183415	0.650060218
RBM8A	-0.214662	0.093631	-2.292646	0.0218684	0.650060218
CHRAC1	-0.422853	0.184513	-2.291721	0.02192176	0.650060218
PACS1	0.468456	0.204753	2.287903	0.02214318	0.654121726
UTRN	-0.444459	0.194309	-2.287381	0.02217362	0.654121726
NAA40	1.110652	0.486254	2.2841	0.02236566	0.658082089
EXD3	-0.707254	0.309828	-2.282731	0.02244622	0.658750184
ZNF563	0.84654	0.371169	2.280743	0.02256365	0.659488106
NAPA	-0.445472	0.195421	-2.279548	0.02263453	0.659488106
ACTG1	0.427215	0.187427	2.27937	0.02264511	0.659488106
P3H4	-1.600333	0.702678	-2.277477	0.02275777	0.659806978
ENSG00000254427	-1.477257	0.648773	-2.277001	0.02278616	0.659806978
ZNF568	0.84888	0.372926	2.276269	0.02282989	0.659806978
PTCD2	-0.784207	0.344726	-2.274869	0.02291377	0.659899239
DENND1A	-0.967658	0.425717	-2.273007	0.02302574	0.659899239
HSPA1B	-2.214868	0.974792	-2.272144	0.02307781	0.659899239
ABCB1	0.695688	0.306405	2.270486	0.02317813	0.659899239
ZCCHC18	-0.555648	0.244757	-2.270205	0.02319515	0.659899239
PLEKHN1	-1.638242	0.722099	-2.268724	0.02328514	0.659899239
FLT1	-1.471556	0.650012	-2.263892	0.02358079	0.659899239
LMNB2	-1.237817	0.546824	-2.263651	0.0235956	0.659899239
MTA1	-0.68586	0.303162	-2.262353	0.02367562	0.659899239
NDUFS7	0.29793	0.131757	2.261205	0.02374659	0.659899239
ERAP1	0.630575	0.278879	2.261106	0.02375267	0.659899239
FXYD5	-0.216566	0.095783	-2.261009	0.02375873	0.659899239
IL27RA	0.565003	0.25007	2.259379	0.02385985	0.659899239
NFYC	-0.411114	0.18209	-2.257753	0.02396106	0.659899239
RBM45	-0.786269	0.34827	-2.257644	0.02396786	0.659899239
AP3B1	0.490303	0.217593	2.253298	0.02424037	0.659899239
TXLNB	-1.37567	0.610549	-2.253171	0.02424838	0.659899239
RPE	-0.690506	0.306461	-2.25316	0.02424904	0.659899239
LINC01410	-1.688032	0.749791	-2.251338	0.02436414	0.659899239
HSBP1	-0.283658	0.126013	-2.251023	0.02438405	0.659899239
MOB4	0.427148	0.189798	2.250543	0.02441449	0.659899239
IFT27	0.684514	0.30445	2.248363	0.02455307	0.659899239
MIR4453HG	-0.833863	0.370883	-2.248319	0.02455584	0.659899239

CADM4	-1.95502	0.869663	-2.248019	0.02457495	NA
RPTOR	-1.030826	0.458903	-2.246284	0.02468584	0.659899239
PHF13	-0.938354	0.417875	-2.245538	0.02473359	0.659899239
KLF12	0.957005	0.426221	2.245329	0.02474705	0.659899239
RPL37A	0.118753	0.052907	2.244569	0.0247958	0.659899239
APOLD1	0.565597	0.252124	2.243326	0.0248758	0.659899239
ST13	-0.190455	0.084903	-2.243217	0.0248828	0.659899239
THG1L	0.726327	0.323832	2.242917	0.02490217	0.659899239
C1GALT1	-0.438612	0.195568	-2.242763	0.02491213	0.659899239
SMG7	-0.549163	0.245144	-2.240168	0.02508005	0.659899239
ENSG00000255320	-2.056687	0.918171	-2.239981	0.02509213	0.659899239
LY96	0.665945	0.297349	2.239607	0.02511645	0.659899239
HIPK1	-0.719453	0.321251	-2.239533	0.02512127	0.659899239
CFLAR	0.426647	0.190517	2.23942	0.02512862	0.659899239
BOD1L1	0.35416	0.158182	2.238938	0.02515992	0.659899239
ZC3H10	0.963522	0.430448	2.238414	0.02519404	0.659899239
TIGAR	-0.801247	0.357997	-2.238137	0.0252121	0.659899239
LINC00513	0.479778	0.214408	2.237686	0.02524154	0.659899239
ENSG00000272563	-1.878373	0.839427	-2.237683	0.02524172	NA
FOSB	-1.723031	0.770217	-2.237072	0.02528161	0.659899239
PCDH9	-0.417456	0.18661	-2.237045	0.02528337	0.659899239
TACC1	-0.472141	0.211116	-2.236409	0.02532502	0.659899239
ECHS1	0.419097	0.187978	2.2295	0.02578062	0.665630252
SDHC	0.312849	0.140335	2.229303	0.02579373	0.665630252
SPATS2	-0.46722	0.209593	-2.229175	0.02580227	0.665630252
TOR4A	-0.91581	0.410866	-2.228972	0.02581574	0.665630252
MIR222HG	-1.221936	0.548488	-2.227826	0.02589215	0.665630252
DOCK6	-1.738165	0.780525	-2.226917	0.02595281	NA
DBF4	-0.525369	0.236141	-2.22481	0.02609398	0.665630252
IRF2BP2	0.6399	0.287704	2.22416	0.0261377	0.665630252
GALC	-1.036991	0.466504	-2.222897	0.02622275	0.665630252
ENSG00000267042	-1.717358	0.77282	-2.222197	0.02626998	0.665630252
KIAA0355	-0.365163	0.164341	-2.221981	0.02628461	0.665630252
PTGR2	1.472803	0.662854	2.221912	0.02628928	0.665630252
PKN1	0.537444	0.241889	2.221861	0.0262927	0.665630252
BLVRB	0.792377	0.356657	2.221681	0.02630488	0.665630252
CFL1	-0.160823	0.072468	-2.219218	0.02647192	0.66723237
DPH1	-1.565162	0.705366	-2.218936	0.02649105	NA
CENPN	-1.018731	0.459461	-2.217232	0.02660727	0.66723237
GP1BA	-1.364741	0.615647	-2.216759	0.02663954	0.66723237
ENSG00000279278	0.739701	0.333953	2.214987	0.02676094	0.66723237
MRM1	1.632328	0.737005	2.214813	0.02677291	0.66723237
E2F4	-0.372898	0.168502	-2.213013	0.02689675	0.66723237
TMEM217	-2.491155	1.125856	-2.212678	0.02691988	NA
DDA1	-0.43813	0.198019	-2.212561	0.02692794	0.66723237
COG3	-0.650971	0.294223	-2.21251	0.02693145	0.66723237
ICAM2	0.465149	0.210264	2.212219	0.02695154	0.66723237

FAM89A	-1.424404	0.644089	-2.211504	0.02700099	0.66723237
RESF1	-0.275407	0.124543	-2.211334	0.02701275	0.66723237
ENSG00000261684	-1.885435	0.853384	-2.209364	0.02714931	NA
LRP5	-1.803913	0.817058	-2.207815	0.02725719	NA
ZNF2	1.564225	0.708727	2.20709	0.02730775	0.671174561
DGUOK	-0.237465	0.107627	-2.206375	0.02735772	0.671174561
PLP2	-0.382655	0.17346	-2.206012	0.02738314	0.671174561
TAF4B	1.207808	0.548179	2.203309	0.02757297	0.671174561
PAXBP1	-0.707881	0.321359	-2.202774	0.02761069	0.671174561
NKTR	-0.301174	0.136726	-2.202763	0.02761149	0.671174561
POMT2	-1.285093	0.583491	-2.20242	0.02763566	0.671174561
PARP16	1.173203	0.532912	2.201496	0.02770092	0.671174561
CCR7	0.247423	0.11243	2.20069	0.02775798	0.671174561
ZNF721	-0.397161	0.180582	-2.199336	0.02785407	0.671174561
UBL7	0.371226	0.168794	2.199288	0.02785747	0.671174561
ENSG00000250155	-1.903804	0.865795	-2.19891	0.02788432	NA
CAMSAP2	-2.572131	1.169967	-2.198465	0.02791598	NA
CAPN7	0.77386	0.352143	2.197572	0.02797959	0.671174561
POU2F2	-0.19921	0.090718	-2.195913	0.02809814	0.671174561
DDX60L	-0.617902	0.281422	-2.195642	0.02811756	0.671174561
AHCYL2	-1.360983	0.619928	-2.19539	0.02813567	0.671174561
SMARCE1	-0.261218	0.118985	-2.195378	0.02813647	0.671174561
TFEC	-2.31054	1.052605	-2.195069	0.02815868	NA
GDPD5	-1.898826	0.865128	-2.19485	0.02817436	0.671174561
HP1BP3	-0.221554	0.101048	-2.192561	0.02833904	0.672564676
LYSMD3	-0.665121	0.303381	-2.19236	0.0283535	0.672564676
ENSG00000224934	-1.704545	0.777612	-2.192025	0.02837774	NA
TAB1	1.111974	0.507385	2.191579	0.02840991	0.672564676
TMEM251	0.73956	0.337781	2.189466	0.02856302	0.674786474
ENSG00000248734	-1.999353	0.913338	-2.189061	0.02859239	NA
TMEM79	1.054428	0.481943	2.187869	0.02867918	0.676127881
GSEC	-1.800759	0.823425	-2.186913	0.02874891	NA
CNP	0.448747	0.205247	2.186373	0.02878834	0.676292736
NDUFS5	0.230537	0.105511	2.184968	0.02889121	0.676292736
PIP4K2A	-0.382062	0.174863	-2.184922	0.02889457	0.676292736
FCRL1	0.522213	0.239051	2.184524	0.02892373	0.676292736
LARP7	-0.333103	0.152551	-2.183556	0.0289949	0.676567527
ENSG00000251136	1.198885	0.54956	2.181537	0.02914375	0.677291172
AGRN	-1.331841	0.61051	-2.181521	0.02914487	0.677291172
AMDHD1	-2.050631	0.941029	-2.179136	0.02932154	NA
PCM1	-0.282266	0.129618	-2.177678	0.02943	0.681395336
RPS4Y2	-1.367754	0.628122	-2.177528	0.02944116	0.681395336
TPP1	0.407964	0.187423	2.176705	0.02950258	0.681431691
SF3B1	-0.256732	0.117996	-2.175762	0.02957308	0.681677406
FCGR2A	-2.585582	1.188718	-2.175101	0.02962255	NA
RGS12	-1.181069	0.543229	-2.174163	0.0296929	0.683056734
GNG11	-1.078414	0.496295	-2.172929	0.02978564	0.68380865

ENSG00000269051	-1.571159	0.723785	-2.170753	0.02994984	0.684942119
EGR1	-1.739952	0.80157	-2.170681	0.02995532	0.684942119
TUBB4A	-1.962094	0.904039	-2.170364	0.02997926	NA
NEU3	-1.146811	0.528513	-2.169884	0.03001567	0.684946708
SYT1	-2.398898	1.106947	-2.16713	0.03022495	NA
MAT2A	-0.320607	0.147971	-2.166687	0.03025873	0.689112236
RRS1	0.753775	0.348235	2.164558	0.03042159	0.689304536
TNNC2	-1.364012	0.630284	-2.164124	0.03045486	0.689304536
TRIM2	-1.596485	0.737907	-2.163531	0.03050033	0.689304536
ENSG00000278238	-1.193392	0.551741	-2.162958	0.0305444	0.689304536
NUP37	0.716347	0.331413	2.161491	0.0306574	0.689304536
TAB3	1.369841	0.633961	2.160767	0.03071337	0.689304536
ENSG00000272540	-1.64009	0.759102	-2.160565	0.03072894	NA
ENSG00000272004	-1.503122	0.696204	-2.159026	0.03084816	0.689304536
CDK5R1	-2.309481	1.069795	-2.158806	0.0308652	NA
DUSP16	-2.52136	1.167974	-2.158747	0.03086978	NA
PILRA	-1.716363	0.795219	-2.158352	0.03090045	NA
SMCHD1	-0.235404	0.109114	-2.157423	0.03097268	0.689304536
YWHAB	-0.199066	0.092271	-2.157393	0.03097505	0.689304536
UBP1	-0.755285	0.350137	-2.157115	0.03099672	0.689304536
DIS3L	0.743888	0.344863	2.157056	0.03100133	0.689304536
FAM41C	-0.826608	0.383495	-2.155461	0.0311258	0.689304536
ENSG00000234290	-1.829402	0.848746	-2.155418	0.03112912	NA
SRPK1	0.604246	0.280394	2.154993	0.03116237	0.689304536
ANKDD1A	-1.54105	0.715233	-2.154614	0.03119206	0.689304536
LINC01134	-1.427557	0.662638	-2.154354	0.03121246	0.689304536
L3MBTL1	-0.99065	0.4599	-2.154057	0.03123572	0.689304536
SUSD3	-0.404371	0.187943	-2.151569	0.03143134	0.6894829
TYMP	0.503205	0.234208	2.148536	0.03167117	0.6894829
ZNF362	-0.736137	0.342639	-2.148433	0.03167936	0.6894829
RPL22	0.118238	0.055043	2.148109	0.03170508	0.6894829
FAM111A	-0.452441	0.210713	-2.14719	0.03177814	0.6894829
USP31	-1.255672	0.585036	-2.146315	0.03184783	0.6894829
RABGGTA	0.744612	0.346971	2.146038	0.03186994	0.6894829
SAR1B	-0.522322	0.24341	-2.145855	0.03188458	0.6894829
GMEB1	0.578731	0.269726	2.145624	0.031903	0.6894829
PRDM2	-0.249221	0.116186	-2.145013	0.03195184	0.6894829
ARHGAP9	0.356961	0.166514	2.143733	0.03205431	0.6894829
ELMO3	-1.116766	0.520956	-2.143686	0.03205809	0.6894829
P2RX1	0.712146	0.332281	2.143203	0.03209683	0.6894829
MAATS1	-2.023426	0.944487	-2.142355	0.03216489	NA
ARID2	-0.495741	0.231432	-2.142064	0.03218837	0.6894829
C1orf21	-2.971906	1.388085	-2.141011	0.03227314	NA
GDI2	0.26631	0.124392	2.140892	0.03228275	0.6894829
LSM12	-0.418613	0.195537	-2.140835	0.03228737	0.6894829
LSM11	-1.102105	0.514856	-2.140607	0.03230571	0.6894829
HSD3B7	-2.163566	1.01075	-2.140555	0.03230995	NA

CTSZ	0.393507	0.183859	2.140261	0.0323337	0.6894829
COL18A1	-2.465398	1.152334	-2.139483	0.03239655	NA
ENSG00000236617	-1.564454	0.73159	-2.138431	0.03248175	NA
MAPK3	0.85259	0.398937	2.137155	0.03258537	0.693550617
ENSG00000276216	-1.651082	0.772824	-2.136426	0.03264472	NA
ZNF653	-1.235282	0.578236	-2.136294	0.03265543	0.693745081
PRR14	-0.37983	0.177967	-2.134273	0.03282048	0.693803074
BMPR2	0.896086	0.419901	2.13404	0.03283953	0.693803074
PGM1	-0.713674	0.334427	-2.134022	0.03284095	0.693803074
ERAL1	-0.561169	0.263236	-2.131814	0.03302218	0.693895289
CLCN6	0.859149	0.403061	2.131561	0.03304293	0.693895289
FAM3A	-0.457097	0.21445	-2.131487	0.03304904	0.693895289
C1RL.AS1	1.557306	0.730958	2.130499	0.03313046	0.693895289
C21orf58	-1.06669	0.500777	-2.130071	0.03316574	0.693895289
IMPDH1	0.802704	0.376941	2.129524	0.03321094	0.693895289
EDN1	-2.011547	0.946179	-2.125968	0.03350589	NA
CIAO1	-0.460967	0.217123	-2.12307	0.03374796	0.7038242
THADA	-0.582443	0.274905	-2.118708	0.03411514	0.704196182
UCP3	-1.447442	0.683279	-2.118376	0.03414325	0.704196182
NT5C	-0.304044	0.143549	-2.118049	0.0341709	0.704196182
ENSG00000269910	-1.892038	0.893297	-2.118038	0.03417186	NA
TAF1A	1.114823	0.526601	2.117015	0.03425862	0.704196182
ENSG00000259888	-2.640074	1.247196	-2.116808	0.03427616	NA
LTBP4	0.935006	0.442043	2.115192	0.03441355	0.704196182
S1PR1	-0.357955	0.169261	-2.11481	0.03444612	0.704196182
FAM81A	-1.577	0.746135	-2.11356	0.03455287	NA
C17orf67	-1.006031	0.476452	-2.111503	0.03472912	0.704196182
ENSG00000247363	-1.200022	0.568358	-2.111384	0.0347393	0.704196182
ZNF606	-0.420954	0.199446	-2.110619	0.03480507	0.704196182
LINC01521	-1.055928	0.50031	-2.110549	0.0348111	0.704196182
NAT9	-0.404117	0.191512	-2.110141	0.0348462	0.704196182
GIT2	0.328878	0.155958	2.108763	0.03496504	0.704196182
DDX47	-1.235052	0.586051	-2.107413	0.03508183	0.704196182
ZNF182	-0.7312	0.347011	-2.107138	0.03510558	0.704196182
ENSG00000232533	1.427488	0.677481	2.107055	0.03511285	0.704196182
USP4	0.561046	0.266285	2.106934	0.03512333	0.704196182
DOCK5	-1.663217	0.790353	-2.104399	0.03534367	0.704196182
SLF1	0.591046	0.280942	2.103796	0.03539624	0.704196182
TMED4	-0.291818	0.138774	-2.102839	0.03547984	0.704196182
SLC30A1	-1.249182	0.594081	-2.102713	0.03549087	0.704196182
GINM1	-0.437584	0.208134	-2.102412	0.0355172	0.704196182
ZNF280B	-0.822919	0.391516	-2.101879	0.03556388	0.704196182
BAZ2A	-0.259785	0.123646	-2.101039	0.03563755	0.704196182
RPS6	0.130908	0.062341	2.099878	0.03573955	0.704196182
ARHGAP22	-1.347964	0.641994	-2.099652	0.0357595	0.704196182
MEF2A	0.43563	0.207533	2.099085	0.03580939	0.704196182
UAP1	-0.588977	0.280611	-2.098912	0.03582468	0.704196182

ENSG00000258768	-1.275899	0.60795	-2.09869	0.03584424	0.704196182
SLC17A5	1.231152	0.586819	2.098008	0.03590442	0.704196182
MAP3K11	0.568674	0.271135	2.09738	0.03595993	0.704196182
PEAK3	-1.49946	0.714971	-2.097232	0.03597301	0.704196182
SMAP1	-0.352075	0.167882	-2.097153	0.03597999	0.704196182
ZNF543	-0.80181	0.382459	-2.096458	0.0360416	0.704196182
ENSG00000273319	0.346128	0.165117	2.096257	0.03605936	0.704196182
PRELID2	-1.095932	0.52291	-2.095833	0.03609699	0.704196182
CD3EAP	1.257697	0.600225	2.095376	0.03613755	0.704196182
NGLY1	-0.262321	0.125197	-2.095258	0.03614805	0.704196182
ENSG00000223821	-1.388817	0.662876	-2.095139	0.03615861	0.704196182
CPNE5	0.614822	0.293482	2.094925	0.03617764	0.704196182
EFCAB13	-0.837672	0.400312	-2.092547	0.03638958	0.705192613
RGP1	-0.381159	0.182182	-2.092184	0.03642209	0.705192613
CLDND2	1.261046	0.603126	2.09085	0.03654148	0.705192613
PRDM4	0.858006	0.410402	2.09065	0.03655944	0.705192613
C15orf62	-0.825882	0.395072	-2.090462	0.03657635	0.705192613
AP1G2	-0.300823	0.143954	-2.08971	0.03664385	0.705192613
FMO4	-1.369492	0.655415	-2.089504	0.03666234	0.705192613
ATP6V1E1	0.433479	0.207537	2.088688	0.03673586	0.705415216
TYSND1	0.878365	0.420725	2.087741	0.03682119	0.705863384
LMO7	-0.78062	0.374096	-2.086685	0.03691661	0.706503326
ADAM9	-1.848007	0.885877	-2.086077	0.03697162	NA
FBXO5	1.000934	0.479952	2.085489	0.03702489	0.707386682
MYBL1	-1.529004	0.733891	-2.083422	0.03721276	0.709774252
AKAP17A	-0.337113	0.16196	-2.081454	0.03739239	0.709774252
OSM	-1.577968	0.758301	-2.080925	0.03744077	0.709774252
ZNF146	-0.377569	0.181446	-2.080893	0.03744372	0.709774252
FAM230J	-1.572793	0.755972	-2.08049	0.03748064	0.709774252
C8orf37	-1.042266	0.501085	-2.080018	0.03752385	0.709774252
RHOA	0.201274	0.096809	2.079086	0.03760945	0.709961027
ENSG00000251417	-1.504543	0.723844	-2.078545	0.03765916	NA
ATP2C1	0.885978	0.426275	2.078419	0.03767077	0.709961027
SCAND1	0.244952	0.118013	2.075643	0.03792701	0.709961027
SLC23A2	1.117248	0.538267	2.075637	0.03792755	0.709961027
ELMSAN1	-0.317193	0.152835	-2.075396	0.03794982	0.709961027
ANK1	-1.327126	0.639597	-2.074942	0.03799193	0.709961027
ATP6V1C2	-1.530569	0.738351	-2.072957	0.0381763	0.709961027
RABAC1	0.225555	0.108817	2.072792	0.03819161	0.709961027
AZIN1	0.55654	0.268511	2.072693	0.03820087	0.709961027
TTC30A	-1.561307	0.753291	-2.072649	0.03820499	0.709961027
YDJC	-0.323752	0.15625	-2.072016	0.03826395	0.709961027
WRNIP1	1.108753	0.535296	2.071291	0.03833164	0.709961027
ARF5	-0.287739	0.138927	-2.071156	0.03834426	0.709961027
CASD1	-0.649313	0.313677	-2.070001	0.03845227	0.710016833
CNOT11	-0.588521	0.28437	-2.069564	0.03849319	0.710016833
ZNF561	0.646407	0.312406	2.069125	0.03853433	0.710016833

ANKRD28	0.961773	0.465639	2.065493	0.03887638	0.714167523
MAP2K6	-1.365669	0.661241	-2.06531	0.03889368	0.714167523
METTL7A	0.411379	0.199254	2.064599	0.03896097	0.714167523
ENSG00000276718	-1.649323	0.798957	-2.064346	0.0389849	NA
NCF1	0.184135	0.089243	2.063304	0.03908378	0.714167523
GGA2	0.263789	0.127916	2.062215	0.03918723	0.714167523
TMBIM1	-0.419115	0.203281	-2.061754	0.03923119	0.714167523
BBS4	0.885316	0.429445	2.061538	0.03925178	0.714167523
C6orf52	-1.573911	0.763502	-2.061437	0.03926134	0.714167523
HAL	-1.588908	0.771341	-2.059931	0.03940516	0.714645898
TESPA1	0.996182	0.48362	2.059847	0.03941316	0.714645898
SCRN2	-0.466816	0.226721	-2.058984	0.03949579	0.715005713
INKA1	-0.458592	0.222916	-2.057238	0.03966338	0.716899903
ARRDC3	0.440062	0.214088	2.05552	0.03982881	0.717022016
AP1M1	-0.393735	0.19156	-2.055412	0.03983919	0.717022016
C19orf66	0.322227	0.156785	2.055207	0.03985904	0.717022016
MIER1	-0.350878	0.170825	-2.054017	0.039974	0.717955812
ENSG00000258944	-1.549733	0.754791	-2.053194	0.04005376	NA
TMCC3	-1.494838	0.728073	-2.053143	0.04005869	NA
ZNF442	-1.265285	0.617119	-2.050309	0.04033431	0.721999775
BCAT1	-2.447301	1.194789	-2.048312	0.04052944	NA
TOB1	0.834748	0.407931	2.046297	0.04072711	0.721999775
DDX59	0.755326	0.369139	2.046186	0.04073808	0.721999775
NDUFB6	0.411155	0.200999	2.045562	0.04079948	0.721999775
EIF3M	0.224352	0.109705	2.045052	0.0408497	0.721999775
COX10	-0.870564	0.425837	-2.04436	0.04091799	0.721999775
ENSG00000203546	-1.128706	0.552248	-2.043839	0.04096947	0.721999775
SYT15	-1.416541	0.693117	-2.043724	0.04098081	0.721999775
NQO1	-1.385863	0.67821	-2.043412	0.04101166	0.721999775
EMC6	-0.340082	0.166437	-2.043305	0.04102227	0.721999775
LBR	0.355334	0.173946	2.042779	0.04107428	0.721999775
ITGB1BP1	0.508464	0.249032	2.041758	0.04117555	0.721999775
ZNF253	0.708087	0.346876	2.041326	0.04121847	0.721999775
IDS	-0.222205	0.108895	-2.040538	0.04129673	0.721999775
ENSG00000179094	1.338349	0.655912	2.04044	0.04130656	0.721999775
CHL1	1.545735	0.757628	2.04023	0.04132745	0.721999775
RBM7	0.421241	0.206487	2.040034	0.04134691	0.721999775
RPL7A	0.100561	0.0493	2.039774	0.0413728	0.721999775
SIGLEC14	1.224206	0.600488	2.038685	0.04148147	0.721999775
ADAT1	-0.635607	0.31182	-2.038376	0.04151237	0.721999775
ERGIC1	-0.31111	0.15264	-2.038193	0.04153068	0.721999775
ITGAV	-1.50154	0.73697	-2.03745	0.04160495	0.722188301
SNHG21	-0.496309	0.243706	-2.036504	0.04169977	0.722732564
FCHO1	0.705826	0.347031	2.033898	0.04196191	0.726170622
ARRDC4	-1.659617	0.816625	-2.032289	0.04212441	NA
ITPR1	0.489385	0.241139	2.029476	0.0424098	0.732807855
DUBR	-1.627262	0.802303	-2.028239	0.04253587	0.733136166

GNG5	-0.187496	0.092453	-2.028026	0.04255757	0.733136166
CCNA1	-2.989636	1.474168	-2.028015	0.04255868	NA
ABHD14A	0.628427	0.310134	2.026309	0.04273317	0.734299645
C3orf38	0.551625	0.272259	2.026105	0.04275408	0.734299645
RFTN1	0.438298	0.216433	2.025095	0.04285759	0.734968889
RSPH9	-2.032226	1.004436	-2.023251	0.04304725	NA
SPPL2B	-0.27487	0.135882	-2.022854	0.04308824	0.737813196
AKIRIN1	-0.319612	0.158065	-2.022025	0.04317381	0.738168338
GLI4	-1.044792	0.517466	-2.019055	0.04348153	0.741832366
UNC119B	1.265676	0.627409	2.017306	0.04366363	0.741832366
STT3A	1.021139	0.506292	2.016899	0.04370608	0.741832366
UTP3	0.469303	0.232713	2.016662	0.04373082	0.741832366
ENSG00000273272	-1.541436	0.764442	-2.016421	0.04375601	NA
LRRCC1	1.406848	0.697715	2.016364	0.04376189	0.741832366
MAP2K4	1.037883	0.514772	2.016201	0.04377899	0.741832366
SPATS2L	-1.852171	0.918648	-2.016193	0.04377983	NA
MIA2	-0.511335	0.25374	-2.015197	0.04388399	0.742277696
VTA1	0.748546	0.371541	2.014704	0.04393564	0.742277696
MLX	-0.321617	0.159765	-2.013065	0.04410781	0.74408246
LRRC46	-1.373395	0.682847	-2.01128	0.04429594	0.746150648
KCNRG	-1.349121	0.671376	-2.009487	0.04448556	0.747585861
RMND5B	-0.390603	0.194448	-2.008774	0.04456108	0.747585861
GRK6	-0.569985	0.28377	-2.008614	0.0445781	0.747585861
TNFRSF12A	-1.733783	0.863221	-2.008503	0.04458984	NA
DTX2	-0.690866	0.344684	-2.004348	0.04503284	0.754101454
OPA3	-0.555181	0.27708	-2.003681	0.04510428	0.754188651
KDM4A.AS1	-1.170488	0.584377	-2.002966	0.04518092	0.754362298
ENSG00000185527	1.480781	0.739556	2.002256	0.04525717	0.754529201
RBBP9	-0.689774	0.344674	-2.001234	0.04536715	0.75525698
TFB2M	0.969339	0.484844	1.99928	0.04557807	0.757660509
IL4I1	-2.141782	1.072162	-1.99763	0.0457568	NA
MTRF1	-0.60353	0.30219	-1.997185	0.0458051	0.760324573
MFSD10	-0.358158	0.179513	-1.995169	0.04602443	0.761320511
CALU	-0.589675	0.295683	-1.99428	0.04612149	0.761320511
POLE3	-0.28727	0.144062	-1.994077	0.04614362	0.761320511
DENND5A	0.941901	0.472352	1.994067	0.04614472	0.761320511
GLCE	-0.991229	0.497214	-1.993567	0.04619939	0.761320511
LINC01816	-1.470278	0.737836	-1.99269	0.04629537	0.761799751
PERP	-1.797468	0.902063	-1.992619	0.04630318	NA
TLR10	0.522375	0.262473	1.990199	0.04656897	0.762238737
BROX	-0.30234	0.152057	-1.98834	0.04677414	0.762238737
GOLIM4	1.124515	0.565593	1.988206	0.04678891	0.762238737
TRPM7	0.485001	0.243989	1.987795	0.04683441	0.762238737
EIF4EBP1	0.405913	0.204235	1.987476	0.04686965	0.762238737
HIVEP2	0.886916	0.446638	1.985761	0.04705989	0.762238737
ENSG00000268713	-0.986326	0.496749	-1.985561	0.04708204	0.762238737
MAFG.DT	-1.525398	0.768356	-1.985274	0.04711402	0.762238737

DHRS3	1.946255	0.980503	1.984957	0.04714928	0.762238737
CLCN7	0.483556	0.243629	1.984804	0.04716623	0.762238737
ZNF607	-1.04922	0.528706	-1.984505	0.04719954	0.762238737
ZNF524	-0.357734	0.18033	-1.983772	0.04728125	0.762238737
EXOC1	0.599343	0.302167	1.983485	0.04731333	0.762238737
LMO2	-1.776133	0.895823	-1.982683	0.04740285	NA
IKZF1	0.36683	0.18504	1.982437	0.04743034	0.762238737
SLC5A5	1.53602	0.774984	1.982002	0.04747903	0.762238737
CIB1	0.167983	0.08478	1.981395	0.04754698	0.762238737
ACVR1B	-1.244522	0.628126	-1.981324	0.04755493	0.762238737
SREBF2.AS1	1.316854	0.664637	1.981313	0.04755619	0.762238737
TNNI2	1.395194	0.70431	1.980939	0.04759808	0.762238737
CASK	-0.869079	0.438977	-1.979785	0.04772766	0.762238737
NRBF2	-0.374689	0.189349	-1.978831	0.04783507	0.762238737
CENPS.CORT	-1.687807	0.853126	-1.978379	0.04788593	0.762238737
C19orf73	-1.069167	0.54064	-1.977596	0.04797425	0.762238737
FGR	0.80077	0.405187	1.976299	0.04812087	0.762238737
RBM18	0.692092	0.350203	1.976258	0.04812553	0.762238737
USP5	-0.563911	0.285389	-1.975941	0.04816143	0.762238737
MYOM1	-1.063744	0.538392	-1.975779	0.04817982	0.762238737
ENSG00000263335	-1.227053	0.621093	-1.975633	0.04819636	0.762238737
ZFP69B	-1.605911	0.812955	-1.9754	0.04822276	NA
HPSE	-1.665608	0.843185	-1.975376	0.04822548	NA
CCDC151	-1.565717	0.79294	-1.974573	0.04831666	NA
AGAP4	1.347108	0.682413	1.974037	0.04837754	0.76239884
EXOC4	0.529862	0.268514	1.973315	0.04845964	0.76239884
RCL1	0.882282	0.447206	1.972874	0.04850989	0.76239884
LINC02245	-1.11363	0.564486	-1.972822	0.04851583	0.76239884
PUS1	0.63322	0.321008	1.972599	0.04854125	0.76239884
KATNBL1	-0.319536	0.162165	-1.97044	0.04878796	0.764514148
KYAT3	-0.514857	0.26136	-1.969918	0.0488478	0.764514148
MPP7	1.372286	0.696779	1.969471	0.04889904	0.764514148
ADAP1	-1.519289	0.771575	-1.969075	0.04894448	0.764514148
PEX6	1.084709	0.551327	1.96745	0.04913139	0.765684782
CENPB	0.999717	0.508223	1.967086	0.04917331	0.765684782
SERGEF	-0.465176	0.23653	-1.966671	0.04922115	0.765684782
ADARB1	-1.004348	0.510855	-1.966013	0.04929708	0.765819701

Cluster 5	log2FC	lfcSE	stat	pvalue	padj
VPREB3	3.700958	0.109845	33.692593	< 2.22e-16	< 2.22e-16
PLAAT4	2.964256	0.13949	21.250729	< 2.22e-16	< 2.22e-16
DENND6B	3.435999	0.198172	17.3385	< 2.22e-16	< 2.22e-16
GRN	1.851849	0.112157	16.511196	< 2.22e-16	< 2.22e-16
CD38	5.601764	0.374535	14.956598	< 2.22e-16	< 2.22e-16
CD1D	3.892037	0.261573	14.879352	< 2.22e-16	< 2.22e-16
ASB2	4.829368	0.326659	14.784131	< 2.22e-16	< 2.22e-16
RABGAP1L	1.629215	0.121193	13.443113	< 2.22e-16	< 2.22e-16
CNP	1.783749	0.152885	11.667247	< 2.22e-16	< 2.22e-16
RNF207	3.11202	0.293551	10.601302	< 2.22e-16	< 2.22e-16
C12orf65	1.214399	0.118834	10.219329	< 2.22e-16	< 2.22e-16
SEPTIN9	0.642307	0.062991	10.1968	< 2.22e-16	< 2.22e-16
ENDOD1	1.633086	0.171849	9.503002	< 2.22e-16	< 2.22e-16
P2RX5	1.621094	0.171127	9.473039	< 2.22e-16	< 2.22e-16
PNOC	1.076261	0.114763	9.378134	< 2.22e-16	< 2.22e-16
SLC12A4	2.283579	0.247878	9.212494	< 2.22e-16	< 2.22e-16
DUS2	1.338061	0.147912	9.046335	< 2.22e-16	< 2.22e-16
NRIP1	1.94529	0.221981	8.763306	< 2.22e-16	1.36E-15
SIGIRR	0.945989	0.112723	8.392181	< 2.22e-16	3.24E-14
SLC5A3	1.217119	0.147147	8.27148	< 2.22e-16	8.52E-14
RAB20	2.567239	0.321534	7.984338	1.41E-15	8.67E-13
DDIT4	1.452503	0.186226	7.799666	6.21E-15	3.64E-12
RAB37	1.16335	0.149549	7.77904	7.31E-15	4.09E-12
MAP3K1	0.738729	0.096115	7.685909	1.52E-14	8.16E-12
MPEG1	1.513519	0.200536	7.547374	4.44E-14	2.29E-11
RARA.AS1	2.419415	0.324355	7.459164	8.71E-14	4.32E-11
DNAJC7	0.466735	0.063728	7.323818	2.41E-13	1.15E-10
AGPAT5	1.146471	0.162161	7.069943	1.55E-12	7.08E-10
CACYBP	0.562876	0.079659	7.066043	1.59E-12	7.08E-10
AHI1	1.140805	0.163239	6.988552	2.78E-12	1.19E-09
HIC1	3.093494	0.444076	6.966134	3.26E-12	1.35E-09
JCHAIN	1.6258	0.2349	6.921252	4.48E-12	1.80E-09
P2RX5.TAX1BP3	1.49181	0.22024	6.773567	1.26E-11	4.91E-09
IRAK2	1.341597	0.199227	6.734012	1.65E-11	6.26E-09
C15orf39	0.955626	0.142441	6.708905	1.96E-11	7.22E-09
SIGLEC10	0.930928	0.139691	6.664189	2.66E-11	9.53E-09
S100A4	-0.882047	0.133314	-6.616288	3.68E-11	1.26E-08
PPCDC	1.300801	0.196726	6.612239	3.79E-11	1.26E-08
CLIC3	2.072955	0.313544	6.611369	3.81E-11	1.26E-08
ANAPC16	0.299951	0.045763	6.554477	5.58E-11	1.80E-08
SLC2A5	2.173665	0.335077	6.487061	8.75E-11	2.75E-08
HTR3A	1.607236	0.250088	6.42667	1.30E-10	4.00E-08
MRPS6	1.036729	0.162541	6.378266	1.79E-10	5.37E-08
PLAAT3	2.236839	0.352403	6.34739	2.19E-10	6.41E-08
TRABD	0.501229	0.079751	6.284896	3.28E-10	9.39E-08
NUP210	0.513913	0.084782	6.061546	1.35E-09	3.78E-07

PPP1R18	0.514904	0.085277	6.038049	1.56E-09	4.28E-07
CR2	1.690965	0.280291	6.032888	1.61E-09	4.32E-07
DPEP2	1.009331	0.167751	6.016857	1.78E-09	4.68E-07
GBP4	0.917511	0.153686	5.970052	2.37E-09	6.09E-07
PSMB10	0.509484	0.085378	5.96741	2.41E-09	6.09E-07
TSPAN18	3.947041	0.669404	5.896354	3.72E-09	9.21E-07
SERPINF1	-1.472244	0.251135	-5.86237	4.56E-09	1.11E-06
ITGAL	0.691671	0.118201	5.851628	4.87E-09	1.16E-06
MS4A1	-0.726376	0.126051	-5.76256	8.28E-09	1.94E-06
VPREB1	3.752328	0.654167	5.73604	9.69E-09	2.23E-06
ZFP36L1	-0.456848	0.080141	-5.700527	1.19E-08	2.70E-06
LCAT	1.940799	0.341016	5.691229	1.26E-08	2.80E-06
GPR183	-0.501253	0.088168	-5.685182	1.31E-08	2.85E-06
DHRS3	4.017502	0.708439	5.67092	1.42E-08	3.02E-06
CD1C	1.589152	0.280287	5.669726	1.43E-08	3.02E-06
SLC16A3	0.862125	0.152764	5.643508	1.67E-08	3.46E-06
VHL	0.656437	0.116673	5.626302	1.84E-08	3.77E-06
IFI30	0.883572	0.158404	5.577961	2.43E-08	4.90E-06
ETFB	0.651573	0.116903	5.573598	2.50E-08	4.95E-06
FAM3C	0.59137	0.106188	5.569088	2.56E-08	5.00E-06
RMDN2	1.60587	0.290199	5.533687	3.14E-08	6.03E-06
GRINA	0.653072	0.122196	5.344454	9.07E-08	1.72E-05
EVI2B	0.318786	0.059889	5.322903	1.02E-07	1.91E-05
CD72	0.944799	0.178529	5.29213	1.21E-07	2.23E-05
RRBP1	1.359371	0.257466	5.279808	1.29E-07	2.35E-05
CD24	-0.821602	0.157056	-5.231266	1.68E-07	3.01E-05
PLP2	-0.428349	0.083036	-5.158613	2.49E-07	4.39E-05
ABI3	0.587428	0.114119	5.14751	2.64E-07	4.60E-05
MAPKAPK2	0.889025	0.172959	5.140096	2.75E-07	4.72E-05
FCRL2	1.008846	0.197075	5.119092	3.07E-07	5.21E-05
SEL1L3	0.768423	0.150693	5.099277	3.41E-07	5.71E-05
PIP5K1B	1.379175	0.272721	5.05709	4.26E-07	7.03E-05
CTSZ	0.586082	0.11626	5.041114	4.63E-07	7.55E-05
GLIPR2	0.850813	0.168928	5.036544	4.74E-07	7.64E-05
KCNH4	3.584812	0.713398	5.02498	5.03E-07	8.01E-05
C5orf56	0.788613	0.157873	4.995247	5.88E-07	9.23E-05
NAPSA	1.878063	0.376612	4.986726	6.14E-07	9.53E-05
CTSA	0.722062	0.145153	4.97449	6.54E-07	0.000100357
GDPGP1	1.371444	0.280366	4.891619	1.00E-06	0.000151615
FCRL1	0.715618	0.146895	4.871615	1.11E-06	0.000165855
PAXX	0.572733	0.117693	4.86632	1.14E-06	0.0001684
ENSG00000233038	4.065694	0.839693	4.841879	1.29E-06	0.000186682
VSIG10L	1.659909	0.342858	4.841387	1.29E-06	0.000186682
JUN	0.463564	0.09626	4.815748	1.47E-06	0.00020997
CARHSP1	0.290532	0.060496	4.802505	1.57E-06	0.000220952
ENSG00000260030	3.557533	0.740974	4.801159	1.58E-06	0.000220952
LSP1	0.296186	0.062438	4.743671	2.10E-06	0.00028925

ZNF3	0.669403	0.141147	4.742592	2.11E-06	0.00028925
TNFRSF13B	-0.456744	0.096504	-4.732881	2.21E-06	0.000300251
GTF2E2	0.530131	0.112373	4.71761	2.39E-06	0.000320314
C16orf54	0.619489	0.132778	4.665617	3.08E-06	0.000408756
RHOG	-0.294041	0.063137	-4.657214	3.21E-06	0.000421448
XYLT1	0.969948	0.208439	4.653393	3.27E-06	0.000424999
CD22	0.577297	0.124784	4.626377	3.72E-06	0.000475357
CPNE5	0.698905	0.151078	4.626118	3.73E-06	0.000475357
CPEB4	0.952137	0.206434	4.61231	3.98E-06	0.000503082
DERL3	0.608568	0.132198	4.603472	4.16E-06	0.000519826
RGS1	-1.23171	0.268839	-4.581596	4.61E-06	0.000571743
FCMR	0.405685	0.088989	4.55883	5.14E-06	0.000629575
SPOCK2	0.788657	0.173049	4.557408	5.18E-06	0.000629575
C16orf72	0.535427	0.117934	4.540056	5.62E-06	0.000677289
BTG2	-0.349145	0.07733	-4.515021	6.33E-06	0.000755388
GPX1	0.418863	0.092829	4.512202	6.42E-06	0.000758476
BTBD7	0.585085	0.129956	4.502166	6.73E-06	0.000787973
PHKB	0.53323	0.118617	4.495381	6.94E-06	0.000806192
BICDL1	1.344904	0.300526	4.475172	7.63E-06	0.000878432
GPR146	-1.326492	0.297355	-4.460965	8.16E-06	0.000930431
APOL3	0.732505	0.166252	4.405988	1.05E-05	0.001190292
ARL14EPL	3.188668	0.728453	4.377313	1.20E-05	0.001346325
SMAD3	0.817083	0.187022	4.368918	1.25E-05	0.001387063
MCM5	0.444662	0.101977	4.360415	1.30E-05	0.001429748
ENSG00000198106	-0.900354	0.206925	-4.351113	1.35E-05	0.001479141
PUS1	0.766459	0.176676	4.338212	1.44E-05	0.001555488
AGO1	0.706477	0.164682	4.289954	1.79E-05	0.001919048
ULK1	0.991701	0.231389	4.285868	1.82E-05	0.001938512
IRF2BPL	0.880022	0.205709	4.277984	1.89E-05	0.001991983
S100A6	-0.531998	0.124511	-4.272709	1.93E-05	0.002023129
IRF1	0.443977	0.10506	4.225953	2.38E-05	0.002472576
IL1B	3.408768	0.80965	4.210177	2.55E-05	0.002630504
LGALS3	-0.671683	0.159683	-4.206364	2.60E-05	0.002654025
RARA	0.539695	0.128388	4.20364	2.63E-05	0.002665028
ZNF70	1.588848	0.379716	4.184305	2.86E-05	0.002879617
ATXN10	0.496026	0.118729	4.1778	2.94E-05	0.002940229
PLXNB2	0.981605	0.235956	4.160124	3.18E-05	0.003152856
LHFPL2	1.138933	0.273967	4.157195	3.22E-05	0.003157567
PALLD	3.451708	0.830477	4.156294	3.23E-05	0.003157567
CHST15	1.003222	0.242978	4.128864	3.65E-05	0.003532114
AP3S1	0.554941	0.134525	4.125193	3.70E-05	0.003554596
ACSL1	0.896184	0.217311	4.12397	3.72E-05	0.003554596
TRIB1	0.855719	0.208511	4.103948	4.06E-05	0.003848358
CTSB	0.431515	0.105846	4.076824	4.57E-05	0.004294235
CD180	0.61154	0.151893	4.02614	5.67E-05	0.005294465
ENSG00000260979	1.444956	0.360575	4.007361	6.14E-05	0.005655598
FLNB	1.04812	0.26156	4.007191	6.14E-05	0.005655598

STK17B	-0.35928	0.089767	-4.002372	6.27E-05	0.005731123
KAT6A	0.515946	0.129016	3.999086	6.36E-05	0.00577035
ABCB1	1.635519	0.411374	3.975747	7.02E-05	0.006322144
HOXB3	2.026502	0.513363	3.947506	7.90E-05	0.007066667
TBL1X	-0.755506	0.191676	-3.941583	8.09E-05	0.007193553
CERK	0.73534	0.187019	3.931888	8.43E-05	0.007392072
TAF12	0.39048	0.099314	3.931759	8.43E-05	0.007392072
RINL	0.445053	0.113595	3.917904	8.93E-05	0.007777061
MIDN	0.5019	0.129679	3.870314	0.0001087	0.009400306
MCL1	0.480371	0.124311	3.86427	0.00011142	0.009571882
CR1	0.896373	0.232182	3.860647	0.00011309	0.009650598
VIM	-0.550273	0.143357	-3.83847	0.0001238	0.01049561
LMNA	-1.583459	0.414097	-3.823882	0.00013137	0.011064003
ENSG00000254802	1.091236	0.288097	3.787742	0.00015202	0.012720518
HIVEP2	0.80081	0.211763	3.781631	0.0001558	0.012936621
PLEKHO1	0.560609	0.148296	3.780342	0.00015661	0.012936621
SREBF1	1.547743	0.410277	3.772437	0.00016166	0.013268555
TNFRSF18	-1.410252	0.374864	-3.762036	0.00016854	0.013745266
FAM167A	1.264201	0.336428	3.757716	0.00017147	0.01389672
ZBTB32	-0.886804	0.236827	-3.744516	0.00018074	0.014556472
RABAC1	0.204748	0.054789	3.737041	0.0001862	0.014902838
AHNAK	-0.491273	0.131972	-3.722555	0.00019722	0.015687272
STK17A	0.446005	0.119965	3.717793	0.00020097	0.01588778
ZNF337.AS1	1.339585	0.360815	3.71266	0.00020509	0.016084353
CLDND2	1.136594	0.306228	3.7116	0.00020595	0.016084353
SPRY1	1.242648	0.335574	3.703049	0.00021302	0.016497535
CRYL1	-0.83304	0.225017	-3.702121	0.00021381	0.016497535
PDLIM2	-0.537412	0.145352	-3.697321	0.00021789	0.016712444
ABRACL	-0.442637	0.119855	-3.693095	0.00022154	0.016892195
GLYCTK	1.017642	0.276121	3.685494	0.00022826	0.017302069
SLC5A5	3.381942	0.918155	3.683409	0.00023014	0.017342258
PTK2B	0.490465	0.133722	3.667789	0.00024466	0.018329341
ERAP1	0.615803	0.168106	3.66319	0.00024909	0.018553842
TBC1D9	0.431841	0.118025	3.658907	0.00025329	0.018758279
LGALS1	-0.805962	0.2205	-3.655157	0.00025702	0.018870017
CD5	-2.155243	0.589965	-3.653173	0.00025902	0.018870017
TMEM159	-0.563205	0.154176	-3.652999	0.0002592	0.018870017
CXCR5	0.438386	0.120147	3.648736	0.00026353	0.019078063
LIMS2	-1.485598	0.409391	-3.628798	0.00028474	0.020412838
CD79A	0.422789	0.116521	3.628439	0.00028514	0.020412838
ACP5	0.681978	0.189349	3.601692	0.00031615	0.022507958
ITGAX	0.914734	0.254354	3.596311	0.00032276	0.022852275
DPEP3	2.249946	0.626805	3.589548	0.00033125	0.023325174
CCNDBP1	0.258515	0.072366	3.572307	0.00035385	0.024781032
A4GALT	1.174359	0.32963	3.562664	0.00036711	0.025570776
BHLHE40	0.506076	0.142226	3.558258	0.00037332	0.025863619
LARGE2	-0.787548	0.221906	-3.549007	0.00038669	0.026535056

CHCHD10	1.594884	0.449579	3.547505	0.0003889	0.026535056
MKNK2	-0.360593	0.101653	-3.547306	0.00038919	0.026535056
NDUFB9	0.225383	0.063648	3.541055	0.00039853	0.027028754
MMP11	1.001768	0.283135	3.53813	0.00040297	0.027186872
ZNF563	0.71897	0.203914	3.525858	0.00042211	0.028330001
FCHO1	0.480628	0.136486	3.521454	0.00042919	0.028655439
CD83	-0.470595	0.133879	-3.515065	0.00043965	0.029202452
FXYD1	-0.800011	0.228976	-3.493859	0.00047609	0.031409338
CORO1B	-0.536899	0.15371	-3.492933	0.00047775	0.031409338
DBNL	0.407287	0.116774	3.487821	0.00048697	0.031853502
ICAM1	0.964852	0.277072	3.482314	0.0004971	0.032351682
PWP1	0.343248	0.099396	3.453332	0.00055371	0.035854576
PARP15	-0.597307	0.173215	-3.448355	0.00056401	0.036339248
TMEM65	0.777975	0.226343	3.437155	0.00058786	0.037687283
TAF4B	1.100883	0.320708	3.432664	0.00059768	0.038127456
CHP1	-0.383286	0.111858	-3.426532	0.00061134	0.038806599
ARL4C	-0.950825	0.278214	-3.417606	0.00063175	0.039905227
ATG2A	0.599927	0.176231	3.404211	0.00066355	0.041710055
CASP8AP2	0.489981	0.144116	3.399917	0.00067406	0.042164964
RAB13	-0.946474	0.278855	-3.394144	0.00068844	0.042855965
NCF1	0.404405	0.119447	3.38565	0.0007101	0.043991919
SYNGAP1	0.940269	0.278562	3.375442	0.00073697	0.045438338
ITPRIPL2	0.91283	0.27087	3.369997	0.00075169	0.046125147
HSPA4	-0.396909	0.118926	-3.337452	0.0008455	0.051635762
EGR2	-1.627897	0.489528	-3.32544	0.00088279	0.053658797
PPFIBP2	0.544933	0.164033	3.322084	0.00089348	0.054053405
ZBED2	-1.151471	0.34723	-3.316163	0.00091262	0.054953626
FAM117A	0.531499	0.160568	3.310113	0.00093258	0.055894198
ENSG00000214797	2.339041	0.70808	3.303358	0.00095534	0.056993366
DTX4	0.934969	0.283601	3.29677	0.00097804	0.058078237
GALK1	-0.494748	0.150178	-3.294409	0.00098629	0.058299642
TMEM41B	0.420241	0.12787	3.286473	0.00101451	0.059693687
LAT2	-0.358	0.109306	-3.275215	0.00105582	0.061842015
SP110	0.247899	0.07581	3.270003	0.00107546	0.062707712
ST6GAL1	-0.400251	0.12303	-3.253277	0.00114082	0.065523983
NBN	0.460487	0.141615	3.251689	0.00114722	0.065523983
SEC62	-0.17989	0.055326	-3.25146	0.00114814	0.065523983
ICAM2	0.6064	0.186534	3.250878	0.00115049	0.065523983
ENSG00000203739	0.849724	0.261392	3.250771	0.00115093	0.065523983
VNN2	0.44994	0.138469	3.249389	0.00115653	0.065523983
IGFBP4	-1.306893	0.402356	-3.248105	0.00116176	0.065523983
INPP5B	0.558395	0.171949	3.24745	0.00116444	0.065523983
CYTIP	-0.235562	0.072673	-3.241405	0.00118942	0.066638488
DNAH1	1.035113	0.320013	3.234595	0.00121815	0.067952909
FKBP2	0.24485	0.075845	3.228305	0.00124526	0.069063884
FXYD2	-1.141267	0.353607	-3.227496	0.00124879	0.069063884
PGPEP1	0.700368	0.218481	3.205623	0.00134771	0.074215991

SLC9A3R1	0.462086	0.144251	3.203344	0.00135842	0.074411775
ZNF821	0.533151	0.166484	3.202414	0.00136281	0.074411775
GCHFR	-0.326468	0.102037	-3.199499	0.00137667	0.074851251
IL2RA	-0.880955	0.275918	-3.192818	0.00140892	0.075967184
ECHS1	0.298873	0.093608	3.192804	0.00140898	0.075967184
GBP7	1.239939	0.388635	3.190493	0.0014203	0.076258329
FOXO3	0.654952	0.205573	3.185989	0.0014426	0.07713412
EMC6	-0.396305	0.124546	-3.181994	0.00146265	0.077882929
NEURL2	1.477558	0.467017	3.163822	0.00155712	0.082509962
EXOSC5	0.442381	0.139868	3.162846	0.00156235	0.082509962
DTWD2	-1.190787	0.377199	-3.15692	0.00159445	0.083861578
ENSG00000266088	-2.900208	0.919269	-3.154909	0.00160548	0.084098608
ENSG00000166927	-0.878213	0.278612	-3.152101	0.001621	0.084567713
ENSG00000259436	-0.719899	0.228916	-3.144811	0.00166194	0.086353955
ENSG00000230709	-0.731747	0.232793	-3.143338	0.00167033	0.086441271
MFSD10	-0.375896	0.119803	-3.137606	0.00170334	0.087640481
ING1	0.460153	0.146688	3.136958	0.00170711	0.087640481
LSS	-0.66197	0.211256	-3.133493	0.00172739	0.088329938
ENSG00000235609	2.322698	0.741598	3.132019	0.00173609	0.088423817
PIM2	-0.289045	0.092343	-3.130107	0.00174743	0.088650924
RUNX3	0.34099	0.109205	3.122473	0.00179338	0.090625613
TGM2	0.64626	0.207289	3.117677	0.00182283	0.091522195
NFKBIA	0.204358	0.065557	3.117272	0.00182533	0.091522195
LPAR2	0.839257	0.269423	3.115016	0.00183935	0.091867679
NXT1	0.36692	0.118118	3.106382	0.00189392	0.094227955
CIB1	0.415448	0.133887	3.102982	0.00191581	0.094950511
UBTF	0.41875	0.135405	3.092582	0.00198423	0.097964916
ADCY7	-0.545548	0.176874	-3.084398	0.00203965	0.100316466
EMP3	-0.238975	0.077527	-3.082477	0.00205286	0.10058208
CD99	-0.316898	0.103608	-3.058611	0.00222366	0.108048827
MDK	1.396077	0.456521	3.058077	0.00222762	0.108048827
MYADM	0.650036	0.212589	3.057703	0.0022304	0.108048827
RHOQ	0.502833	0.164586	3.055147	0.00224951	0.108566015
PDIA3	0.293888	0.096251	3.053365	0.00226291	0.108805221
SERPINB9	-0.30481	0.099975	-3.04886	0.00229711	0.109675769
SIPA1L1	-0.318792	0.104565	-3.04874	0.00229803	0.109675769
EHD1	0.352212	0.115968	3.037153	0.00238825	0.113560594
SNX18	0.810992	0.267365	3.033281	0.00241911	0.114605128
ENSG00000089127	0.769627	0.254597	3.022918	0.0025035	0.118168778
ATP2B1	-0.195606	0.064741	-3.021376	0.00251628	0.118338784
CARNS1	-1.404742	0.466009	-3.014407	0.00257482	0.120651381
KANSL3	0.724137	0.24089	3.006089	0.00264632	0.123552336
RASGEF1B	-0.445257	0.14845	-2.999375	0.00270534	0.125801954
UROS	0.342305	0.114163	2.998398	0.00271403	0.125801954
CHRAC1	-0.338273	0.112909	-2.995979	0.00273565	0.126349857
LSM10	-0.210074	0.070332	-2.986896	0.00281826	0.12970013
IRF4	0.650003	0.217882	2.983284	0.00285173	0.130773676

PRDM4	0.511776	0.171979	2.975811	0.00292215	0.13352763
APOL1	0.631416	0.21269	2.968717	0.00299046	0.136088133
LINC00513	0.479824	0.161676	2.967809	0.0029993	0.136088133
GPER1	-1.584968	0.53507	-2.96217	0.00305479	0.138119395
FLOT1	0.387399	0.130927	2.958891	0.00308748	0.138980184
CASP7	0.521853	0.176415	2.958102	0.0030954	0.138980184
PLEKHO2	0.526477	0.178767	2.945049	0.00322903	0.144476799
SGMS1.AS1	0.705697	0.240139	2.938701	0.00329591	0.146927846
RESF1	-0.246297	0.08384	-2.937695	0.00330662	0.146927846
RAB8B	-0.383351	0.130628	-2.934672	0.00333901	0.147857241
RBX1	-0.162737	0.055483	-2.933098	0.00335598	0.1480998
DDX24	0.284961	0.09727	2.929577	0.00339424	0.149276911
RUFY3	0.545002	0.186226	2.92656	0.00342733	0.150219578
PRDX1	-0.250814	0.085869	-2.920876	0.00349049	0.152469365
MAP2K3	-0.29399	0.100727	-2.918678	0.00351519	0.153029601
MPHOSPH9	0.546785	0.187536	2.915631	0.0035497	0.154011511
DAAM1	-0.538234	0.18479	-2.912685	0.00358336	0.154950423
CDYL2	1.831296	0.629965	2.906982	0.00364934	0.15690365
SH3BGRL3	-0.239615	0.082436	-2.906678	0.00365289	0.15690365
FADS3	-0.437285	0.150561	-2.904375	0.00367987	0.157537687
TRAF3IP2	-0.718381	0.247913	-2.897714	0.00375893	0.160389322
GTF2B	0.391244	0.135395	2.889657	0.00385663	0.16401477
GPR132	0.402885	0.139729	2.883331	0.00393494	0.166794749
PAOX	0.560575	0.195057	2.873905	0.0040543	0.171291034
ENSG00000263264	0.763866	0.265953	2.872184	0.00407646	0.171664032
C7orf50	-0.429702	0.149933	-2.865963	0.00415743	0.174503728
HSH2D	0.310357	0.10838	2.863602	0.00418853	0.174705765
MIR22HG	-0.647691	0.226185	-2.86354	0.00418936	0.174705765
SMIM20	0.408352	0.142703	2.861546	0.00421581	0.175241491
GGA2	0.329312	0.115195	2.858736	0.00425333	0.176232781
AHCY	-0.32637	0.114806	-2.842796	0.00447197	0.184698132
RAB3IP	-0.524639	0.184668	-2.840983	0.00449748	0.185158073
HCK	-0.542546	0.191215	-2.837366	0.00454874	0.186672164
PTPA	-0.659631	0.233073	-2.830151	0.00465261	0.190029857
LINC02576	-1.806206	0.638426	-2.829153	0.00466714	0.190029857
SLC25A39	0.222704	0.078732	2.828628	0.0046748	0.190029857
BATF2	2.352515	0.833937	2.820976	0.00478778	0.194010444
PACS1	0.341314	0.121096	2.818546	0.00482417	0.194872197
BANK1	-0.224634	0.079954	-2.809551	0.00496107	0.199776221
ARID5B	-0.28674	0.102286	-2.803322	0.00505792	0.201949322
LARGE1	1.030888	0.36774	2.803303	0.00505821	0.201949322
RAB7A	0.209617	0.074813	2.801868	0.00508077	0.201949322
PCBP1	-0.186822	0.06668	-2.801791	0.00508197	0.201949322
MYO9B	0.303576	0.108379	2.801067	0.0050934	0.201949322
DUSP10	-0.512705	0.183164	-2.799164	0.00512351	0.202519898
ENSG00000261448	0.802082	0.286698	2.797658	0.00514747	0.202844771
ENSG00000176320	2.292757	0.821232	2.791851	0.00524074	0.205702427

STAT5A	0.417966	0.149746	2.791163	0.00525191	0.205702427
ITGB7	0.4283	0.153968	2.78175	0.00540667	0.2111222
CYBA	0.224601	0.080874	2.777185	0.00548319	0.213463477
HRK	-0.588766	0.212628	-2.768994	0.00562297	0.217800377
TMCO3	-0.408087	0.147407	-2.768439	0.00563255	0.217800377
GPR18	0.599111	0.216465	2.767702	0.0056453	0.217800377
SMYD4	-0.474857	0.171994	-2.760899	0.00576424	0.221310625
CHD7	0.313425	0.113538	2.760538	0.00577063	0.221310625
MYO7B	-1.804508	0.654451	-2.757284	0.00582837	0.222663067
RHBDF2	0.249735	0.090595	2.756607	0.00584046	0.222663067
KATNBL1	-0.298804	0.108539	-2.752957	0.00590597	0.223989853
PIK3IP1	0.33875	0.12306	2.752732	0.00591002	0.223989853
ENSG00000262292	0.765643	0.278632	2.747865	0.00599847	0.226600494
ADPGK	0.255649	0.093064	2.747013	0.00601408	0.226600494
TLR10	-0.384397	0.140419	-2.737507	0.00619069	0.231959891
CD47	-0.215769	0.078822	-2.73742	0.00619232	0.231959891
ZNF736	-0.764694	0.279766	-2.733338	0.00626959	0.233848529
CDK14	-0.59391	0.217323	-2.732843	0.00627903	0.233848529
SMC5	-0.299908	0.109901	-2.728902	0.00635457	0.235979653
ZNF318	-0.508355	0.186901	-2.719925	0.00652968	0.241785773
HDAC6	-0.458905	0.168808	-2.718493	0.006558	0.242138751
MYO1C	-0.425446	0.156626	-2.716317	0.00660127	0.242949273
GPR155	-0.609086	0.2243	-2.715496	0.00661766	0.242949273
ENSG00000261087	0.948387	0.350588	2.705133	0.0068277	0.249948215
CHIT1	-1.344916	0.497541	-2.703123	0.00686912	0.250752185
SGK1	-0.742648	0.27497	-2.700831	0.00691664	0.251773444
CD82	-0.254069	0.094245	-2.695833	0.00702129	0.254862925
PXDC1	1.092524	0.406294	2.689001	0.00716661	0.259407225
CFL1	-0.13263	0.049347	-2.687685	0.00719492	0.259702338
SLC2A3	-0.43105	0.16046	-2.686339	0.00722399	0.260023092
RUBCN	-0.414983	0.154642	-2.683515	0.00728527	0.261498562
NAB2	-0.740443	0.2767	-2.675977	0.00745117	0.266104529
PPP3CA	-0.228362	0.085349	-2.675629	0.00745891	0.266104529
SF1	0.149492	0.055899	2.674334	0.00748778	0.266104529
DUOX1	0.475809	0.177942	2.673958	0.00749619	0.266104529
SLC45A3	0.984882	0.369036	2.668797	0.00761234	0.268549295
AP1S3	-0.405669	0.152024	-2.668449	0.00762023	0.268549295
CREM	-0.537844	0.201581	-2.668125	0.00762758	0.268549295
HINT2	0.263035	0.098847	2.661041	0.00778994	0.273518037
RAP1A	-0.180722	0.068021	-2.656852	0.00788742	0.276068646
SFXN4	0.669188	0.251946	2.656083	0.00790543	0.276068646
POU2AF1	0.301961	0.113728	2.655112	0.00792821	0.276116056
SERPINB1	0.279179	0.105252	2.652489	0.00799008	0.277520629
ADGRB2	-2.420612	0.913445	-2.649981	0.00804964	0.278837856
RAB30.DT	-0.323709	0.12226	-2.647697	0.00810422	0.279915777
CLIC1	-0.177311	0.066989	-2.646864	0.00812421	0.279915777
NRM	0.445585	0.168455	2.645131	0.00816593	0.280388388

SLC38A2	-0.267944	0.101347	-2.643823	0.00819755	0.280388388
ABLIM2	-0.868425	0.328502	-2.64359	0.0082032	0.280388388
HLA.DOA	0.434147	0.164402	2.640769	0.00827182	0.281985794
FYTTD1	-0.229564	0.087014	-2.638247	0.00833358	0.282779928
ANXA2	-0.340888	0.129221	-2.638027	0.008339	0.282779928
TKT	-0.189066	0.071711	-2.636498	0.00837666	0.283311324
ENSG00000261766	0.603576	0.229409	2.631006	0.00851326	0.287177587
PSME1	0.139398	0.053025	2.628927	0.00856546	0.287836121
ENSG00000273188	0.997499	0.3795	2.628452	0.00857745	0.287836121
ITGAM	0.654662	0.249555	2.623315	0.00870787	0.291453623
MYOM1	-1.241343	0.47384	-2.61975	0.00879943	0.293755116
LGMN	0.733988	0.280316	2.618435	0.00883342	0.294114279
REL	-0.223481	0.085406	-2.616685	0.00887881	0.294114279
EHMT1	-0.213652	0.081665	-2.61621	0.00889119	0.294114279
MYC	0.398696	0.15244	2.615434	0.00891142	0.294114279
FXYD5	-0.330434	0.126364	-2.61494	0.00892431	0.294114279
XIAP	0.303122	0.116017	2.612732	0.00898218	0.295266322
ARHGEF2	0.263398	0.100912	2.610175	0.00904959	0.296725161
MYL2	1.96892	0.754915	2.608136	0.00910369	0.297741439
MLXIP	0.479515	0.183923	2.607154	0.00912982	0.297840253
BMF	-0.3873	0.148628	-2.605829	0.00916522	0.298123304
MARCKSL1	-0.339877	0.130466	-2.605099	0.00918477	0.298123304
AKR1A1	-0.219132	0.084166	-2.603556	0.00922621	0.298715868
PIN1	-0.200582	0.077191	-2.598527	0.00936247	0.302367841
BRK1	0.150187	0.057819	2.597534	0.00938958	0.302485282
SMC6	-0.293346	0.113101	-2.593654	0.0094962	0.305157131
AIM2	0.324903	0.125414	2.590645	0.00957964	0.305638878
METTL21A	-0.266265	0.10278	-2.590636	0.00957989	0.305638878
ALDH1B1	0.904353	0.349097	2.590547	0.00958235	0.305638878
FCER2	0.947455	0.365956	2.588987	0.00962588	0.306179444
ENSG00000261884	0.459909	0.177692	2.588239	0.00964682	0.306179444
COMMD7	-0.25852	0.099929	-2.58704	0.00968042	0.306491191
TUBB6	-0.687056	0.265715	-2.585686	0.00971854	0.306943855
LINC01869	-0.714285	0.276629	-2.582102	0.00982006	0.30894585
PIK3R5	0.489321	0.18953	2.581757	0.00982988	0.30894585
EHD4	-0.477297	0.185136	-2.578096	0.00993464	0.311385737
RALA	-0.396995	0.154032	-2.577359	0.00995584	0.311385737
PDCD4	0.638841	0.248044	2.575511	0.0100092	0.312296865
SYAP1	-0.410878	0.159745	-2.572081	0.01010893	0.314646676
CTSS	0.210522	0.081903	2.570393	0.01015832	0.315422065
FXYD7	-0.855358	0.333348	-2.565962	0.01028901	0.318032378
GSAP	0.352199	0.137263	2.56587	0.01029175	0.318032378
PBX4	0.936722	0.365938	2.559783	0.01047375	0.322882171
POLM	0.375847	0.147108	2.554896	0.01062194	0.326668954
KAT2A	0.331817	0.129994	2.552547	0.01069386	0.328097647
AK1	0.562186	0.221203	2.541492	0.01103806	0.337853654
TRBC2	-0.170151	0.067011	-2.539143	0.01111244	0.33904116

C19orf38	0.625296	0.246325	2.538499	0.01113293	0.33904116
NMT2	-0.472337	0.186122	-2.537781	0.01115579	0.33904116
TBC1D8	1.391661	0.548571	2.536885	0.01118436	0.339109685
TMSB10	-0.244093	0.096379	-2.532641	0.01132067	0.342437063
TMEM87B	-0.499138	0.197258	-2.530377	0.01139399	0.343847624
NBEAL2	1.021037	0.403691	2.529253	0.01143058	0.344145799
ANKRD37	-0.560568	0.221956	-2.525575	0.01155091	0.346958151
IFNAR2	0.32295	0.128113	2.520823	0.01170807	0.350860756
RBMX2	-0.2888	0.11464	-2.519181	0.0117628	0.351683205
SPN	0.979166	0.389017	2.517024	0.01183508	0.352825411
SUCLG2	0.278775	0.110783	2.516409	0.01185577	0.352825411
NTAN1	-0.274303	0.109462	-2.505916	0.01221347	0.362633163
GAK	0.272129	0.108774	2.501769	0.01235747	0.365262825
APOBEC3H	1.279193	0.511389	2.501409	0.01237001	0.365262825
ENSG00000272155	1.129277	0.451545	2.500921	0.01238708	0.365262825
CSNK2A2	-0.450696	0.180362	-2.498845	0.01245989	0.366571111
SMARCA4	0.34137	0.136717	2.496909	0.0125281	0.367108152
WASF1	-0.64925	0.260123	-2.495934	0.01256258	0.367108152
SRR	0.814002	0.326135	2.495905	0.01256361	0.367108152
CARD19	-0.386753	0.155079	-2.493902	0.01263474	0.368351166
ESYT1	-0.34753	0.139409	-2.492891	0.01267076	0.36856751
VTI1A	-0.36552	0.147019	-2.486199	0.01291158	0.374726654
IQSEC1	-0.336108	0.135367	-2.48293	0.01303067	0.37733301
SYNGR3	0.812296	0.327298	2.481821	0.01307129	0.377660581
USP7	0.317171	0.127867	2.480473	0.0131208	0.378243109
OSM	-1.713731	0.69155	-2.478102	0.01320834	0.37991667
ENSG00000262420	1.605898	0.648981	2.474492	0.01334256	0.382414876
PLGRKT	-0.432468	0.174847	-2.473408	0.01338311	0.382414876
JDP2	0.567481	0.229436	2.473379	0.01338422	0.382414876
TERF2IP	0.208095	0.084372	2.4664	0.01364787	0.38908502
ARRDC5	0.689183	0.279971	2.461621	0.01383109	0.392599222
IQGAP1	-0.198456	0.080621	-2.461595	0.01383207	0.392599222
ENSG00000249679	-1.375267	0.559305	-2.458887	0.01393686	0.394704184
A1BG	-0.264237	0.10751	-2.45779	0.01397949	0.395043082
PRRT3	0.657785	0.267888	2.455452	0.01407074	0.396751658
EIF4A3	0.367442	0.149696	2.454583	0.01410482	0.396793972
ENSG00000229127	-1.202067	0.489871	-2.453844	0.01413382	0.396793972
PISD	0.58726	0.239718	2.4498	0.01429354	0.400116785
UNKL	0.342648	0.139897	2.449278	0.01431428	0.400116785
RHOBTB3	1.450464	0.592822	2.44671	0.01441668	0.40210673
SLC25A5	-0.17857	0.073034	-2.445016	0.01448456	0.402277281
LY9	-0.465548	0.190408	-2.445	0.01448523	0.402277281
POLH	0.747853	0.30623	2.442132	0.01460081	0.404615238
FAM177A1	-0.238275	0.097618	-2.440885	0.01465133	0.405110131
PLD4	-0.569548	0.233408	-2.440141	0.01468155	0.405110131
BCL2L11	-0.637324	0.261447	-2.437682	0.01478178	0.406563864
SNRNP35	0.303067	0.124353	2.437143	0.01480381	0.406563864

WDR11	0.288873	0.118559	2.436531	0.01482889	0.406563864
RB1	-0.253183	0.104114	-2.431792	0.01502431	0.411047321
FBXO10	0.638157	0.26275	2.428762	0.01515048	0.412881907
ASCC1	0.425653	0.175264	2.428643	0.01515545	0.412881907
SSR3	-0.193731	0.079859	-2.425898	0.01527054	0.415139738
PLEC	-0.357721	0.147537	-2.424614	0.01532466	0.415306227
ATP6V0A1	0.434039	0.179043	2.424224	0.01534113	0.415306227
DNAAF4	-0.92159	0.380844	-2.419863	0.01552636	0.419439425
AFF3	-0.254875	0.10541	-2.417943	0.0156085	0.420776438
AP3B1	0.290696	0.120295	2.416527	0.01566938	0.421133702
MBD6	0.600578	0.248604	2.415796	0.01570087	0.421133702
ENSG00000133961	0.296196	0.12263	2.415357	0.0157198	0.421133702
AP1G2	-0.24231	0.100431	-2.412697	0.01583499	0.422913437
MZB1	-0.545124	0.225976	-2.412308	0.01585187	0.422913437
DMPK	-1.03311	0.428755	-2.409558	0.01597188	0.424674681
PLPP5	-0.34149	0.141739	-2.409285	0.0159838	0.424674681
GHRL	0.42316	0.175773	2.407429	0.0160653	0.425961786
UPP1	0.669754	0.278306	2.406537	0.01610459	0.426126827
SLC25A25	-0.551863	0.229457	-2.405082	0.01616882	0.42694966
TTC13	-0.747484	0.3109	-2.404261	0.0162052	0.4270353
H1FX	0.726421	0.30232	2.402822	0.0162691	0.427844225
GABARAPL1	0.955856	0.397983	2.401753	0.01631671	0.428222191
CUL2	0.442529	0.184347	2.400517	0.01637193	0.428798011
RPS6KC1	-0.551725	0.230063	-2.398151	0.01647808	0.430299079
ISG20	-0.234119	0.097641	-2.397752	0.01649602	0.430299079
LSM2	-0.169237	0.070632	-2.396042	0.01657318	0.431438302
CAMTA1	-0.247776	0.103498	-2.394006	0.01666547	0.432966118
GAPDH	-0.166369	0.069523	-2.393018	0.0167104	0.433259991
PARD6A	-0.343746	0.143743	-2.391392	0.01678461	0.43411166
ARAP1	-0.438498	0.183409	-2.390824	0.01681063	0.43411166
ATP2C1	-0.418733	0.175352	-2.387954	0.01694247	0.43664128
MRNIP	0.390127	0.164011	2.378664	0.01737549	0.446788713
TMEM175	0.487856	0.205152	2.378027	0.01740555	0.446788713
CYBB	0.249476	0.104996	2.376041	0.01749951	0.447705914
USP18	1.066879	0.44906	2.375804	0.01751077	0.447705914
C1orf162	-0.413777	0.174399	-2.372582	0.01766422	0.450228587
FLNA	-0.332372	0.140107	-2.372267	0.01767932	0.450228587
NOTCH2	0.35843	0.151242	2.36991	0.01779242	0.452215162
LINC02397	0.190483	0.080456	2.367549	0.01790635	0.45414393
ITGA3	0.94543	0.399442	2.366879	0.01793879	0.45414393
POU2F2	-0.2758	0.116704	-2.363242	0.01811585	0.457727252
SESTD1	-0.453593	0.192274	-2.359101	0.01831928	0.461961314
ACTB	-0.184134	0.078124	-2.356953	0.01842559	0.463219487
GNAS	0.166345	0.070586	2.356641	0.01844107	0.463219487
ZNF71	-0.446702	0.189619	-2.355788	0.01848346	0.463380951
SLFN5	-0.822224	0.349303	-2.353903	0.01857746	0.464833169
TNF	-0.834597	0.355	-2.350977	0.01872417	0.467596184

CHST7	0.851677	0.362421	2.349965	0.01877518	0.467963074
H3F3A	0.166006	0.07078	2.345394	0.019007	0.472666187
USP15	-0.210329	0.0897	-2.344801	0.01903723	0.472666187
ENSG00000268516	-0.383899	0.164074	-2.33979	0.0192946	0.478134948
NPEPPS	-0.258783	0.110661	-2.338515	0.01936054	0.478318615
MED18	0.562273	0.24056	2.33735	0.01942097	0.478318615
CD79B	0.246878	0.105624	2.337332	0.01942191	0.478318615
RAB4B	-0.287394	0.122993	-2.336675	0.01945609	0.478318615
UBE2D4	-0.398102	0.170474	-2.335271	0.01952925	0.478318615
BMS1	0.320974	0.137486	2.334594	0.01956462	0.478318615
CREBRF	0.289654	0.12408	2.334419	0.01957381	0.478318615
DIPK1A	0.592258	0.253759	2.333938	0.01959896	0.478318615
DMD	-0.538248	0.230887	-2.331223	0.01974162	0.480889521
HOXB4	1.003484	0.431193	2.327226	0.01995322	0.485126692
PPP2R5C	0.211944	0.09121	2.323682	0.02014254	0.48727933
TBC1D10C	-0.157738	0.067889	-2.323475	0.02015366	0.48727933
S100A11	-0.179892	0.077425	-2.323446	0.0201552	0.48727933
RBM26	0.212685	0.091589	2.322179	0.02022332	0.488010556
HIPK2	0.495951	0.213811	2.319574	0.02036395	0.488784346
ANKRD36C	-0.46125	0.198856	-2.319511	0.02036735	0.488784346
PSMB9	0.195864	0.084448	2.319347	0.02037623	0.488784346
HNRNPC	0.16281	0.070214	2.318777	0.02040711	0.488784346
EVL	0.191877	0.082844	2.316121	0.02055164	0.491332969
CD37	-0.204147	0.088187	-2.314927	0.02061692	0.491980781
RBM19	0.470923	0.203591	2.313079	0.02071832	0.492983008
SCARB1	-1.17448	0.507825	-2.312767	0.02073543	0.492983008
ABCA2	0.965678	0.417805	2.311314	0.02081552	0.493264455
FAM107B	0.24349	0.105354	2.311163	0.02082383	0.493264455
SHISAL2A	0.358296	0.155183	2.308866	0.02095103	0.495366883
IVNS1ABP	-0.367613	0.159324	-2.307325	0.0210367	0.496037741
MAP4K1	0.218734	0.094814	2.306972	0.02105639	0.496037741
SH3BP5	-0.414427	0.17973	-2.305831	0.02112005	0.496629494
HGSNAT	-0.5211	0.226223	-2.303485	0.02125158	0.49881219
CALR	0.291645	0.126878	2.298629	0.02152604	0.503978282
GTF3A	0.161865	0.070431	2.298209	0.0215499	0.503978282
SACS	-0.537661	0.234242	-2.295324	0.02171456	0.506909173
ENSG00000251364	1.142158	0.498502	2.291182	0.02195288	0.511222508
MT1X	-0.501389	0.218908	-2.290409	0.02199762	0.511222508
MFGE8	0.764362	0.33381	2.289812	0.0220322	0.511222508
RBM47	0.584623	0.255393	2.289106	0.02207317	0.511222508
SS18L2	-0.130855	0.057175	-2.288684	0.0220977	0.511222508
BCL6	-0.530113	0.231814	-2.286799	0.02220758	0.512336884
C12orf74.PLEKHG7	0.515004	0.225277	2.286092	0.0222489	0.512336884
HTT	0.339849	0.148677	2.285814	0.02226515	0.512336884
KDM4B	-0.401432	0.175678	-2.285042	0.02231039	0.512462803
HP1BP3	-0.138889	0.060821	-2.283584	0.02239597	0.513513202
LUZP1	-0.513851	0.225286	-2.280884	0.02255532	0.515346467

NAP1L6	1.50205	0.658542	2.280874	0.02255591	0.515346467
TCEAL3	-0.504185	0.221161	-2.279718	0.02262442	0.515996898
ZNF496	0.773493	0.339586	2.277754	0.02274123	0.517744712
P2RX4	0.379534	0.16673	2.276343	0.02282552	0.517864211
SIDT2	-0.320645	0.140861	-2.27632	0.02282686	0.517864211
PTTG1	-0.596723	0.26269	-2.27159	0.02311127	0.52078436
CDC25B	0.310526	0.136714	2.27135	0.02312581	0.52078436
MCPH1	0.321158	0.141415	2.271031	0.02314512	0.52078436
SVBP	-0.195813	0.086225	-2.270942	0.02315047	0.52078436
ENSG00000230155	0.541316	0.238378	2.270824	0.02315765	0.52078436
JUND	-0.394523	0.173793	-2.270072	0.02320322	0.520900051
CIC	-0.374637	0.165151	-2.268447	0.02330201	0.52220808
ENSG00000260349	0.590548	0.260495	2.267022	0.02338889	0.523245205
MAST4	-0.815612	0.360135	-2.264738	0.02352874	0.52448022
HOXB7	0.669018	0.295428	2.26457	0.02353908	0.52448022
CNR1	0.608707	0.268848	2.264129	0.0235662	0.52448022
CARS2	0.40128	0.17742	2.261752	0.02371271	0.525279656
TCEA2	0.355204	0.15706	2.261577	0.02372358	0.525279656
TSEN15	-0.37269	0.164793	-2.261563	0.02372441	0.525279656
SYBU	-1.177985	0.521159	-2.260319	0.02380147	0.525389244
JAK1	0.146553	0.064859	2.259559	0.02384865	0.525389244
DCK	0.151574	0.067083	2.25951	0.02385168	0.525389244
OAS3	0.913701	0.405415	2.253741	0.02421245	0.530689823
OGG1	0.309392	0.137289	2.253585	0.0242223	0.530689823
C9orf72	-0.4749	0.21074	-2.25349	0.02422828	0.530689823
YIF1A	-0.208551	0.092572	-2.252838	0.02426936	0.530689823
LPCAT4	-0.363824	0.161529	-2.252381	0.02429823	0.530689823
CCDC154	0.850702	0.378265	2.248961	0.024515	0.533654712
ZNF627	0.476021	0.211712	2.248434	0.02454854	0.533654712
TXNDC11	0.347263	0.154457	2.248282	0.02455822	0.533654712
TRMT1	0.244642	0.108907	2.246341	0.02468215	0.535444677
CD84	-0.451552	0.201086	-2.245565	0.02473189	0.535622107
ZNF706	0.178516	0.079595	2.24281	0.02490908	0.537776675
MIPEP	-0.812592	0.362324	-2.242721	0.02491484	0.537776675
MIIP	-0.221393	0.098784	-2.241188	0.0250139	0.538622077
CYB561A3	-0.321543	0.143493	-2.240822	0.02503761	0.538622077
SMARCB1	0.275637	0.123084	2.239426	0.02512823	0.538957086
B3GNT2	-0.41359	0.184711	-2.239126	0.0251477	0.538957086
UBB	0.163526	0.073047	2.238651	0.02517866	0.538957086
CLIC4	-0.278977	0.124742	-2.236429	0.0253237	0.540769931
DUSP2	-0.891828	0.398838	-2.236069	0.02534728	0.540769931
VAMP1	-0.419052	0.187529	-2.234599	0.0254437	0.541378963
SMIM12	-0.21221	0.094976	-2.234353	0.02545985	0.541378963
DENND6A	0.406568	0.182165	2.231861	0.02562414	0.543974786
FAM111B	-0.326995	0.146649	-2.229781	0.02576201	0.546001967
ZNF827	0.613148	0.275095	2.228862	0.02582306	0.546397383
TPM4	-0.200063	0.089815	-2.227492	0.02591444	0.547431948

MFN1	-0.340681	0.153156	-2.224408	0.02612101	0.550892529
POLR2G	0.186643	0.083958	2.223042	0.02621296	0.55117102
ABHD15	0.386817	0.174012	2.222941	0.02621976	0.55117102
FIP1L1	0.254676	0.114704	2.220292	0.02639892	0.552546992
DNPEP	0.282895	0.127432	2.21996	0.02642148	0.552546992
ENSG00000271857	1.457882	0.656796	2.219687	0.02644003	0.552546992
SLC37A1	-0.425837	0.191867	-2.219441	0.02645674	0.552546992
ENSG00000275441	-1.126339	0.508741	-2.213973	0.02683061	0.559448683
MTG1	0.371053	0.167835	2.210823	0.02704807	0.56307172
SAMD1	-0.558498	0.25277	-2.209511	0.02713913	0.56396083
CCDC12	0.145771	0.066002	2.208587	0.02720335	0.56396083
RPUSD3	0.231998	0.105057	2.208305	0.02722302	0.56396083
USF3	-0.386615	0.175179	-2.206965	0.02731651	0.56396083
ADAM28	0.199346	0.090332	2.206813	0.02732716	0.56396083
COMMD5	-0.315502	0.142991	-2.206438	0.02735337	0.56396083
HAUS4	-0.61031	0.276746	-2.205308	0.02743251	0.564689071
BACE2	-0.611189	0.277438	-2.202974	0.02759656	0.567051167
GTF3C5	0.321146	0.145815	2.202425	0.02763527	0.567051167
HACD4	-0.386985	0.175889	-2.200164	0.02779525	0.569330375
ENSG00000259772	-0.751649	0.341774	-2.199258	0.02785961	0.569330375
BIN3	-0.321989	0.146426	-2.198986	0.0278789	0.569330375
RUNX2	1.212432	0.551706	2.197604	0.02797737	0.570437354
ROMO1	-0.14705	0.066962	-2.196038	0.02808924	0.571813421
CCDC86	-0.696137	0.317095	-2.195363	0.02813759	0.571894371
NEK1	0.388509	0.177109	2.193618	0.02826288	0.573536098
PPP1CC	0.235584	0.107436	2.192792	0.02832236	0.573839535
CCDC85B	-0.172474	0.078767	-2.189666	0.02854846	0.577512465
SNX8	-0.308831	0.141124	-2.188372	0.0286425	0.578055849
KCNN1	1.538863	0.703299	2.188063	0.02866504	0.578055849
FAAP20	-0.21484	0.09833	-2.184897	0.02889638	0.581562862
FURIN	0.362469	0.16596	2.184072	0.02895696	0.581562862
PPP1R16B	0.376072	0.172207	2.183835	0.02897434	0.581562862
LINS1	-0.400295	0.183529	-2.181099	0.02917606	0.583679983
CTF1	1.789331	0.820747	2.180125	0.02924817	0.583679983
TRAF4	-0.422154	0.193638	-2.180117	0.0292488	0.583679983
VWCE	1.520267	0.697515	2.179549	0.02929092	0.583679983
C22orf39	-0.209663	0.096205	-2.179342	0.0293063	0.583679983
POLR1C	0.447676	0.205549	2.177956	0.02940934	0.584473692
ENSG00000166928	-1.403549	0.644543	-2.177586	0.02943686	0.584473692
NFAT5	0.270893	0.124513	2.175622	0.02958354	0.584897943
TFAM	0.282103	0.129667	2.1756	0.02958519	0.584897943
MPPED2	2.243387	1.031216	2.175477	0.0295944	0.584897943
TP53I11	-0.506795	0.2331	-2.174157	0.02969337	0.585602738
DDAH2	-0.180063	0.082834	-2.173789	0.02972095	0.585602738
RABEP2	-0.238793	0.109915	-2.172522	0.02981634	0.586050572
FAM114A2	0.408757	0.188173	2.172238	0.02983768	0.586050572
DAPK2	0.458197	0.210988	2.171676	0.02988012	0.586050572

WDFY2	-0.289202	0.133257	-2.170263	0.02998689	0.587250945
PMM1	-0.327234	0.15115	-2.16496	0.03039078	0.59425741
MPZL1	-0.455942	0.210662	-2.164334	0.03043873	0.59429305
SRP9	-0.129233	0.059754	-2.16274	0.03056122	0.59578188
ATAD2B	-0.393018	0.181957	-2.159949	0.03077661	0.598921171
FAM160B1	0.442619	0.204968	2.159451	0.03081521	0.598921171
ALKBH7	0.16143	0.074845	2.156844	0.03101782	0.601511468
BCL11A	-0.276142	0.128049	-2.156536	0.03104184	0.601511468
AUH	0.416266	0.193277	2.153727	0.03126157	0.604859779
TMEM256	-0.143269	0.066552	-2.152759	0.03133765	0.60527463
TMEM121	-1.124501	0.522475	-2.152259	0.03137696	0.60527463
SERINC5	-0.681691	0.317134	-2.149537	0.03159183	0.608138056
CTNND1	-0.575661	0.267851	-2.149184	0.03161978	0.608138056
SIDT1	-0.439339	0.204499	-2.148372	0.0316842	0.608468933
WDR7	0.642788	0.299321	2.147487	0.03175451	0.608911535
PROC	-1.38314	0.644393	-2.146424	0.03183919	0.609628315
PRDM8	0.691473	0.322446	2.144461	0.03199597	0.611721211
NECAP2	0.183933	0.085803	2.143655	0.03206053	0.612047462
PARP9	-0.302492	0.141154	-2.142989	0.032114	0.612161272
DALRD3	-0.244122	0.113973	-2.141926	0.03219943	0.612883063
C16orf74	0.359373	0.168007	2.139033	0.03243301	0.6164186
TRIM38	0.176786	0.082682	2.138146	0.0325049	0.616875071
ENSG00000250132	-1.439858	0.674062	-2.136089	0.03267213	0.618248412
ZFAND4	0.713954	0.334236	2.136076	0.03267322	0.618248412
UGT8	0.476019	0.222937	2.135217	0.03274332	0.618666248
ANKRD13A	0.2833	0.132744	2.134177	0.03282826	0.619362944
REC8	0.412764	0.193487	2.133298	0.03290025	0.619660038
EBF1	-0.264867	0.124187	-2.132812	0.03294018	0.619660038
PELI1	-0.356815	0.167456	-2.130803	0.0331054	0.621860296
LSM7	-0.219933	0.103463	-2.125714	0.03352712	0.625929521
CEP70	0.807045	0.379754	2.125179	0.03357164	0.625929521
SLC43A2	0.348229	0.163903	2.124597	0.03362026	0.625929521
ENSG00000273319	0.339442	0.159796	2.124217	0.03365196	0.625929521
LSR	-0.693752	0.326647	-2.123862	0.0336817	0.625929521
CSAD	0.395833	0.186443	2.123079	0.03374725	0.625929521
IMP3	0.142731	0.06723	2.123038	0.03375068	0.625929521
ANKRD39	0.344987	0.162561	2.122193	0.03382149	0.625929521
ZNF835	-0.871493	0.410786	-2.121526	0.03387755	0.625929521
SQSTM1	0.26439	0.124639	2.121247	0.03390101	0.625929521
NCF2	-0.580834	0.273828	-2.121164	0.03390799	0.625929521
SPATA2L	-0.426149	0.200915	-2.121048	0.03391778	0.625929521
C9orf16	-0.136338	0.064291	-2.120624	0.0339535	0.625929521
LETM2	-1.143164	0.539491	-2.118969	0.03409309	0.627604984
PECAM1	-0.548738	0.259068	-2.118119	0.03416503	0.628032148
TRIM26	0.397076	0.187533	2.117361	0.03422923	0.628315976
FAM129C	-0.254932	0.120508	-2.115482	0.03438891	0.630227922
ARHGEF1	0.293082	0.138608	2.114458	0.03447619	0.630227922

SLC2A13	0.495667	0.234467	2.114021	0.03451344	0.630227922
UBE2F	-0.267041	0.126333	-2.113785	0.03453365	0.630227922
ZFP36L2	-0.361548	0.171085	-2.113267	0.03457793	0.630227922
PRDM2	-0.1543	0.073063	-2.11187	0.03469764	0.631516626
CLCN3	0.348465	0.165054	2.11122	0.03475342	0.631639745
UNC119	-0.26387	0.125023	-2.110576	0.03480875	0.631754255
PIH1D1	-0.218924	0.103766	-2.109776	0.0348777	0.632115301
ADAL	-0.486977	0.230887	-2.109158	0.03493093	0.632190999
KEAP1	0.352782	0.167442	2.106889	0.03512717	0.634850989
ATF3	-0.80584	0.382584	-2.10631	0.03517743	0.634868824
TRAPPC1	-0.143876	0.068326	-2.105721	0.03522863	0.634903584
SLC2A11	0.437776	0.207973	2.104972	0.03529373	0.635111677
EID1	0.203543	0.09672	2.104455	0.03533875	0.635111677
AIFM1	0.404115	0.192231	2.102237	0.03553251	0.637704597
UXT	-0.114008	0.054287	-2.100095	0.03572049	0.640073576
CYREN	-0.27571	0.131316	-2.099602	0.03576385	0.640073576
RNF24	-0.539503	0.257196	-2.097635	0.0359374	0.642287538
LY6E	-0.278954	0.133173	-2.094668	0.0362005	0.645835775
ZNF154	-0.537774	0.256829	-2.093897	0.03626912	0.645835775
PSMA5	0.192528	0.091956	2.093705	0.03628629	0.645835775
ISOC1	-0.415295	0.198627	-2.090828	0.03654347	0.649516
MRPL24	0.308353	0.147749	2.087013	0.036887	0.654718787
C9orf139	0.422902	0.202803	2.085282	0.03704375	0.656596668
TFEB	0.234093	0.112291	2.084697	0.03709682	0.65663405
INKA2	-0.681637	0.327087	-2.083961	0.03716375	0.656916473
SNAP23	0.178698	0.085813	2.082413	0.03730473	0.658505183
RUBCNL	0.262113	0.125943	2.081204	0.03741528	0.659543107
PRX	1.426858	0.685887	2.080311	0.03749698	0.659543107
DDX28	0.395261	0.190021	2.080092	0.03751708	0.659543107
TIAM2	0.477791	0.229925	2.078024	0.03770713	0.661981098
RECQL	-0.260273	0.125418	-2.075249	0.03796344	0.664702552
NIFK	0.20252	0.097589	2.075229	0.03796532	0.664702552
RPS19	0.161766	0.078163	2.069585	0.03849121	0.670432373
KLHL5	-0.33747	0.163073	-2.06944	0.0385048	0.670432373
QSOX2	-0.386959	0.186988	-2.069432	0.03850557	0.670432373
HMGN1	-0.136599	0.066021	-2.069034	0.0385429	0.670432373
HAUS8	0.366481	0.177136	2.068929	0.03855272	0.670432373
RUNX1	-0.533248	0.257902	-2.067643	0.03867357	0.671021597
TNFRSF17	0.642299	0.31067	2.067461	0.03869075	0.671021597
RCBTB2	-0.907649	0.439344	-2.065918	0.0388362	0.671521775
ZNF133	0.795028	0.384908	2.065501	0.03887564	0.671521775
TCAF2	0.449746	0.217742	2.065498	0.03887593	0.671521775
PDIA6	0.210175	0.10188	2.062963	0.03911617	0.674641031
MSC	-0.743887	0.360674	-2.062489	0.03916122	0.674641031
TPM3	-0.132926	0.064559	-2.058985	0.03949569	0.677475415
SLAMF1	-0.766392	0.37222	-2.058978	0.03949632	0.677475415
PHB2	-0.1567	0.07611	-2.058851	0.03950851	0.677475415

GDPD5	-0.618897	0.300645	-2.058564	0.03953605	0.677475415
RFX5	0.206839	0.100516	2.057771	0.0396121	0.677877182
LINC00894	1.995285	0.970346	2.05626	0.03975744	0.679461958
LY75	-0.424165	0.206463	-2.054435	0.0399336	0.681568756
RNMT	-0.217427	0.105911	-2.052916	0.04008077	0.683072952
RASSF5	-0.247238	0.120461	-2.052431	0.04012775	0.683072952
KCNMB4	-1.832278	0.893933	-2.049681	0.04039557	0.68551004
WIPF1	-0.131474	0.064152	-2.049407	0.04042236	0.68551004
HDLBP	0.257507	0.125655	2.049323	0.04043052	0.68551004
IMPAD1	0.506815	0.247615	2.046785	0.04067919	0.68807422
ENSG00000250303	-0.553021	0.270203	-2.04669	0.04068854	0.68807422
CNTRL	-0.169076	0.082664	-2.04533	0.04082232	0.689431737
NEK7	-0.416045	0.203796	-2.04148	0.0412031	0.694951745
PSME2	0.195335	0.09574	2.040279	0.04132257	0.696055678
SIGLEC5	0.816391	0.400246	2.039723	0.04137794	0.696078442
POLRMT	0.472251	0.231744	2.037811	0.04156886	0.698378524
AGPAT2	-0.239093	0.117376	-2.036989	0.0416511	0.69884901
GADD45G	-2.001874	0.984388	-2.033624	0.04198954	0.703611436
TMEM128	0.306624	0.150836	2.032829	0.0420698	0.704040758
FAM219B	-0.216246	0.106438	-2.031654	0.04218869	0.705114647
FAM217B	0.306594	0.151044	2.029836	0.04237319	0.706420026
ENSG00000261200	1.08251	0.53338	2.029527	0.04240464	0.706420026
WAC.AS1	0.246333	0.121395	2.029184	0.04243951	0.706420026
WFS1	1.421618	0.70086	2.028392	0.04252027	0.706420026
IFIT1	-1.572046	0.775098	-2.02819	0.04254089	0.706420026
CXCL16	0.401324	0.197939	2.027521	0.0426092	0.706643691
PLIN2	0.401698	0.198431	2.024377	0.04293137	0.711071472
NAA40	-0.410233	0.202733	-2.023516	0.04301997	0.711624248
STK40	-0.320393	0.158546	-2.020822	0.04329819	0.715122244
AKT3	-0.335951	0.16628	-2.020395	0.04334242	0.715122244
ENSG00000269968	-0.653615	0.323749	-2.018895	0.04349817	0.716774265
LBX2.AS1	-1.421387	0.704578	-2.017359	0.04365803	0.718489561
DDX42	0.260185	0.129131	2.014896	0.04391559	0.721487695
SP140	0.15833	0.078613	2.014039	0.04400543	0.721487695
LIPA	0.244862	0.121585	2.013927	0.04401715	0.721487695
FAM32A	-0.177174	0.087994	-2.01348	0.04406416	0.721487695
RTP4	0.459026	0.228099	2.012402	0.0441776	0.722427079
NFIC	0.355419	0.176779	2.010524	0.04437575	0.724747667
STMN1	-0.314457	0.156455	-2.009893	0.04444254	0.724919693
HSP90AB1	-0.10182	0.050685	-2.008882	0.04454961	0.725747432
IGLC2	-0.378776	0.188616	-2.008183	0.04462389	0.726039714
ITPKB	-0.29628	0.147685	-2.006167	0.04483843	0.728610352
FNBP1	0.200518	0.100011	2.004955	0.04496781	0.729792501
PSD3	-0.751997	0.375245	-2.004018	0.04506818	0.730282464
ENSG00000223881	0.800331	0.399444	2.003615	0.04511135	0.730282464
LMAN1	0.281933	0.140763	2.00289	0.04518914	0.730623872
IDI1	-0.178935	0.089451	-2.00037	0.04546034	0.734087704

TENT5C	-0.335258	0.167729	-1.998805	0.04562943	0.735316756
LRRC58	-0.239469	0.119818	-1.99861	0.04565058	0.735316756
U2AF1L4	-0.242088	0.121376	-1.994529	0.04609431	0.741537101
ENSG00000267390	0.409055	0.205338	1.992109	0.04635911	0.744403888
UTP3	0.255969	0.128554	1.99114	0.04646548	0.744403888
TMEM242	-0.246229	0.123669	-1.991038	0.04647666	0.744403888
RGL2	-0.405487	0.203681	-1.990794	0.04650358	0.744403888
INSIG1	-0.186129	0.09363	-1.98791	0.04682159	0.747573029
CRYM.AS1	-1.126905	0.566907	-1.987811	0.04683258	0.747573029
PGD	-0.232237	0.116863	-1.987262	0.04689339	0.747573029
M6PR	0.197287	0.099298	1.98681	0.04694349	0.747573029
ENSG00000272356	0.828222	0.417036	1.985972	0.04703648	0.747573029
ABI2	-0.276494	0.139251	-1.985582	0.04707977	0.747573029
ING2	-0.38468	0.193761	-1.985331	0.04710766	0.747573029
ENSG00000267519	-0.316654	0.159746	-1.982242	0.04745217	0.752113934
BACH1.AS1	0.957411	0.483631	1.979632	0.04774493	0.755824568
ORAI2	0.167358	0.0846	1.978215	0.04790443	0.756492178
MAP3K11	-0.260701	0.131786	-1.978214	0.04790452	0.756492178
CLEC17A	0.512395	0.259348	1.975705	0.04818824	0.75980879
PLEKHJ1	-0.234145	0.118545	-1.975158	0.0482502	0.75980879
CD2AP	0.307351	0.155689	1.97413	0.04836702	0.75980879
PAN3.AS1	-1.18562	0.600594	-1.974078	0.04837287	0.75980879
COPS6	0.151822	0.076928	1.973559	0.04843192	0.75980879
MRM1	0.61121	0.30975	1.973239	0.04846832	0.75980879
FAM120A	-0.207311	0.105127	-1.972003	0.04860931	0.761093082
SYPL1	-0.175085	0.088817	-1.971311	0.04868831	0.761404858
ZMAT3	-0.280928	0.142564	-1.970537	0.04877682	0.761700739
PYCARD	-0.200132	0.101584	-1.970113	0.04882546	0.761700739
PIP4K2A	-0.208149	0.105784	-1.967681	0.04910471	0.765130884
ENSG00000275418	-1.705396	0.867139	-1.966694	0.0492185	0.765977681
ITGB1	0.376965	0.191828	1.965122	0.04940013	0.767877091

Cluster 6	log2FC	lfcSE	stat	pvalue	padj
VPREB3	2.398998	0.203538	11.786475	< 2.22e-16	< 2.22e-16
RIMS3	-5.167318	1.001637	-5.158873	2.48E-07	0.00128195
ENSG00000260526	-4.125399	0.852792	-4.837518	1.31E-06	0.00452259
EDA2R	-4.021795	0.861283	-4.669537	3.02E-06	0.00684525
NRG2	-4.327405	0.930589	-4.650177	3.32E-06	0.00684525
LCA5	-4.897325	1.069226	-4.58025	4.64E-06	0.00764376
ENSG00000269242	-4.18984	0.919395	-4.557171	5.18E-06	0.00764376
ENSG00000272455	-4.841394	1.075238	-4.502626	6.71E-06	0.00789628
ENSG00000254281	-3.84343	0.854632	-4.497173	6.89E-06	0.00789628
STEAP1B	-3.874637	0.872533	-4.440678	8.97E-06	0.00925456
ENSG00000269737	-4.208938	0.966671	-4.354054	1.34E-05	0.011862
EFNB1	-3.871302	0.890542	-4.34713	1.38E-05	0.011862
KLHL17	-3.704641	0.858975	-4.312861	1.61E-05	0.01279326
PRKCD	-3.803037	0.888424	-4.280655	1.86E-05	0.01305754
ENSG00000255882	-4.022862	0.943551	-4.263534	2.01E-05	0.01305754
LINC01550	-4.34193	1.018711	-4.26218	2.02E-05	0.01305754
DNAAF5	-3.494397	0.833306	-4.193414	2.75E-05	0.01668119
ENSG00000250222	-4.213041	1.009901	-4.171736	3.02E-05	0.01733114
MMP14	-4.860452	1.172839	-4.144175	3.41E-05	0.0185238
PEX7	-3.487203	0.84625	-4.120772	3.78E-05	0.01871218
TTLL1	-4.583909	1.11291	-4.118848	3.81E-05	0.01871218
MON1A	-3.714765	0.912946	-4.068986	4.72E-05	0.02124004
LINC02362	-4.934308	1.213878	-4.064914	4.81E-05	0.02124004
TUNAR	-3.846624	0.947802	-4.058469	4.94E-05	0.02124004
DOCK7	-3.292774	0.817924	-4.025771	5.68E-05	0.02245931
DOCK9.DT	-4.097002	1.018956	-4.020785	5.80E-05	0.02245931
TTC30B	-3.857915	0.963479	-4.004153	6.22E-05	0.02245931
LRRC7	-3.109566	0.777362	-4.000152	6.33E-05	0.02245931
RPGRIP1L	-3.439908	0.861063	-3.994956	6.47E-05	0.02245931
BORCS8.MEF2B	-5.014476	1.255869	-3.992832	6.53E-05	0.02245931
KIF9.AS1	-3.666927	0.921242	-3.980416	6.88E-05	0.02263599
ENSG00000262429	-3.567989	0.897462	-3.975644	7.02E-05	0.02263599
SCAT1	-4.683505	1.180855	-3.966199	7.30E-05	0.02264674
C11orf65	-4.689411	1.185613	-3.955262	7.65E-05	0.02264674
UBTD2	-3.459114	0.876161	-3.948033	7.88E-05	0.02264674
TFR2	-3.944148	0.999173	-3.947413	7.90E-05	0.02264674
CFAP53	-4.527794	1.150363	-3.935969	8.29E-05	0.02290411
DOK2	-3.74273	0.95193	-3.93173	8.43E-05	0.02290411
MTCL1	-3.874723	0.98824	-3.920832	8.82E-05	0.02335067
ETS2	-4.775205	1.219986	-3.914148	9.07E-05	0.02340676
ENSG00000261845	-3.845214	0.984296	-3.906561	9.36E-05	0.02356461
MYLK	-4.546023	1.16574	-3.89969	9.63E-05	0.02366621
RUSC1.AS1	-3.945453	1.016404	-3.881778	0.000103696	0.02488694
SUOX	-4.43226	1.144944	-3.871159	0.000108319	0.02540577
LINC01011	-4.442503	1.149527	-3.864636	0.000111255	0.02551449
FITM1	-4.446148	1.153677	-3.853892	0.000116255	0.02608149

NKX6.3	-3.905024	1.017993	-3.836002	0.000125054	0.02689026
LILRB3	-3.425206	0.892919	-3.835967	0.000125071	0.02689026
FAM66C	-3.500045	0.914734	-3.8263	0.000130084	0.02739727
CCNF	-2.946367	0.771533	-3.818847	0.000134077	0.02767351
ZNF778	-3.177171	0.833927	-3.809892	0.000139027	0.02813262
ENSG00000272323	-3.141245	0.82734	-3.796799	0.000146576	0.02908978
TESMIN	-3.819005	1.008768	-3.785813	0.000153207	0.02947101
FBXL18	-3.241699	0.856642	-3.784192	0.000154209	0.02947101
ENSG00000274922	-5.552477	1.47433	-3.766102	0.000165816	0.03110746
MORN4	-3.734529	0.994498	-3.755192	0.000173209	0.03110746
NLRX1	-3.212485	0.856005	-3.752882	0.000174813	0.03110746
GNMT	-4.312586	1.149147	-3.75286	0.000174829	0.03110746
MPZ	-2.957587	0.789586	-3.745746	0.000179858	0.03135851
NR2F6	-3.613754	0.96662	-3.738546	0.000185088	0.03135851
ENSG00000267731	-3.383145	0.905024	-3.738182	0.000185356	0.03135851
YY2	-3.61555	0.970452	-3.725637	0.000194823	0.03194385
DSP	-3.107111	0.834096	-3.725123	0.00019522	0.03194385
KCTD21	-4.078626	1.096127	-3.720943	0.000198481	0.03194385
FAM214B	-2.774365	0.746635	-3.715826	0.000202541	0.03194385
DNASE1L3	-3.008675	0.810167	-3.713649	0.000204292	0.03194385
STAM	-2.619392	0.708722	-3.695936	0.000219078	0.03288635
CA3.AS1	-5.642094	1.527626	-3.693373	0.000221299	0.03288635
ENSG00000272750	-2.921729	0.791337	-3.692141	0.000222374	0.03288635
PRICKLE3	-3.155034	0.85471	-3.691351	0.000223066	0.03288635
CABP4	-2.947856	0.800761	-3.681318	0.000232031	0.03306141
ENSG00000269973	-3.479697	0.945278	-3.681136	0.000232197	0.03306141
KIF18A	-3.43613	0.933906	-3.679311	0.000233865	0.03306141
TTC22	-4.808618	1.310051	-3.670558	0.000242021	0.03375218
ENSG00000271855	-3.705484	1.014062	-3.654099	0.000258086	0.03551268
FBXL19.AS1	-2.814771	0.771252	-3.649614	0.000262635	0.03566307
ZNF687.AS1	-3.562818	0.977671	-3.644191	0.000268235	0.03586075
RPP40	-3.089919	0.848526	-3.641513	0.000271041	0.03586075
TRAM2.AS1	-3.20783	0.88724	-3.615517	0.000299749	0.03905293
ADNP.AS1	-3.733385	1.033335	-3.612948	0.000302736	0.03905293
PAK1	-3.064607	0.849034	-3.609523	NA	NA
ENSG00000228242	-5.384954	1.494191	-3.603927	0.000313445	0.03993525
DCLRE1B	-3.250855	0.903101	-3.599657	0.000318637	0.04010162
EGLN3	-4.804824	1.339552	-3.586889	0.000334647	0.04129041
PRRG2	-2.822238	0.787066	-3.58577	0.000336085	0.04129041
PLAAT4	2.053964	0.574489	3.575291	0.000349839	0.04168856
PEAK3	-4.080305	1.141274	-3.575219	0.000349934	0.04168856
ENSG00000233818	-4.985398	1.394871	-3.574093	0.000351444	0.04168856
ENSG00000042317	-4.130983	1.157234	-3.569703	0.000357386	0.04191163
ENSG00000272941	-3.277561	0.919008	-3.566411	0.000361903	0.04196451
ZNF624	-3.086788	0.866695	-3.561563	0.000368654	0.04208215
ENSG00000175773	-5.239481	1.471828	-3.559845	0.000371073	0.04208215
SEC61A2	-2.63324	0.741372	-3.551845	0.00038254	0.04291102

NPEPL1	-2.729884	0.770632	-3.542398	0.000396507	0.04399949
ENSG00000272821	-4.440453	1.255205	-3.537631	0.000403733	0.04432477
ENSG00000273226	-3.41913	0.96992	-3.525167	0.000423216	0.04524716
SLC35E4	-4.653346	1.320466	-3.524019	0.000425054	0.04524716
FSTL3	-3.109234	0.882334	-3.523873	0.000425288	0.04524716
IQCK	-3.011326	0.855732	-3.519008	0.000433164	0.04561477
ENSG00000261542	-3.709394	1.061484	-3.494534	0.00047489	0.04903247
POMT2	-3.815747	1.091959	-3.494404	0.000475121	0.04903247
ENSG00000270871	-4.236244	1.214806	-3.487178	0.000488147	0.04987797
TRPV1	-3.532576	1.013879	-3.484217	0.000493579	0.04993854
CDCA5	-4.759384	1.367524	-3.480291	0.000500869	0.05018413
LINC00887	-4.785735	1.378655	-3.471307	0.000517932	0.05087775
ENSG00000251034	-3.429275	0.989654	-3.465127	0.000529981	0.05087775
CHEK2	-2.927888	0.845368	-3.463449	0.000533298	0.05087775
ZNF862	-4.033071	1.16489	-3.46219	0.000535799	0.05087775
MSANTD3	-2.819357	0.814344	-3.462119	0.00053594	0.05087775
CHKB.DT	-3.61049	1.043258	-3.460785	0.000538603	0.05087775
LRRC3	-3.881096	1.122047	-3.458942	0.000542302	0.05087775
ENSG00000260879	-3.183433	0.921722	-3.453788	0.000552772	0.05130562
ENKD1	-2.956688	0.857182	-3.449313	0.000562015	0.05130562
PPP1R3D	-3.012203	0.874426	-3.444777	0.000571531	0.05130562
NRSN2	-3.128599	0.908241	-3.444679	0.000571737	0.05130562
POLR1B	-2.83851	0.824239	-3.443795	0.000573611	0.05130562
RAB3A	-3.499463	1.016592	-3.442346	0.000576691	0.05130562
ENSG00000273576	-3.230707	0.93972	-3.437946	NA	NA
LMNTD1	-3.023067	0.880233	-3.434396	0.000593876	0.05154893
TBC1D16	-3.415565	0.994953	-3.432889	0.000597186	0.05154893
NPHP4	-3.901238	1.136677	-3.432142	0.000598833	0.05154893
HDX	-4.180261	1.218121	-3.431728	0.000599748	0.05154893
ENSG00000179979	-3.740624	1.091251	-3.427832	0.000608421	0.05154893
ACTL10	-3.232564	0.943154	-3.427398	0.000609396	0.05154893
ENSG00000226853	-5.030499	1.469038	-3.424349	0.000616275	0.05170696
ZSCAN30	-2.312337	0.676597	-3.417599	0.000631762	0.05257892
C16orf46	-4.210545	1.235031	-3.409261	0.000651391	0.05370047
LIG4	-2.60251	0.764163	-3.405701	0.000659943	0.05370047
ADPRH	-3.2079	0.942024	-3.405327	0.000660849	0.05370047
ZMYM1	-2.464825	0.725161	-3.399003	0.000676321	0.05452834
TSPAN32	-3.26166	0.960615	-3.395387	0.000685316	0.05466684
ENSG00000231105	-4.72428	1.391924	-3.394065	0.000688633	0.05466684
ARHGAP19	-2.523122	0.744025	-3.39118	0.000695924	0.05470289
TMEM164	-3.486981	1.028438	-3.390561	NA	NA
HIST3H2A	-3.856289	1.137649	-3.389701	0.000699688	0.05470289
POLE2	-3.679127	1.087092	-3.384376	0.000713403	0.05513297
ENSG00000279571	-4.99802	1.477207	-3.383426	0.000715874	0.05513297
TIGD2	-2.829888	0.837385	-3.379434	0.000726353	0.05552566
ENSG00000229729	-2.699717	0.799362	-3.377338	0.000731911	0.05553917
ZDHHC9	-3.900873	1.157008	-3.371518	0.000747551	0.05573145

SACS	-2.859752	0.848294	-3.371181	0.000748467	0.05573145
APOO	-2.552369	0.757294	-3.37038	0.000750646	0.05573145
ZNF18	-3.179919	0.94519	-3.364318	0.00076733	0.05636206
ATXN7L2	-3.149872	0.936532	-3.363337	0.000770063	0.05636206
KANK1	-3.557882	1.058576	-3.361007	NA	NA
AMY2B	-3.24104	0.965542	-3.356705	0.000788771	0.05732478
LY86.AS1	-3.562673	1.062368	-3.353521	0.000797904	0.05733628
MMAA	-2.691076	0.80264	-3.352781	0.000800041	0.05733628
ENSG00000224046	-2.852037	0.851657	-3.348808	0.000811601	0.0574775
KLHL23	-3.264355	0.974935	-3.348279	0.000813151	0.0574775
MAMLD1	-4.859202	1.455732	-3.337978	NA	NA
CUBN	-4.114567	1.235128	-3.331287	0.000864454	0.06068818
ENSG00000260081	-3.048897	0.916462	-3.326812	0.000878455	0.06125442
HLCS	-3.108206	0.935158	-3.323723	0.000888245	0.0615214
DLGAP3	-4.461326	1.343549	-3.320554	0.000898389	0.06180917
SDCBP2	-4.412018	1.330759	-3.315414	0.000915074	0.06211332
SEMA4F	-2.931694	0.884712	-3.313728	0.000920609	0.06211332
BNIPL	-3.729451	1.125481	-3.31365	0.000920866	0.06211332
ENSG00000272588	-3.286454	0.993652	-3.30745	0.000941496	0.06309249
KCNMB4	-3.909978	1.18417	-3.301873	0.000960415	0.06364056
ENSG00000249476	-2.818741	0.8538	-3.301408	0.000962009	0.06364056
PART1	-3.761656	1.14281	-3.291585	0.000996247	0.06470832
SCD	-3.315942	1.007757	-3.290418	0.001000386	0.06470832
CEP70	-3.881799	1.179858	-3.290056	0.001001676	0.06470832
LACTB2.AS1	-2.84584	0.865716	-3.287267	0.001011648	0.06470832
SEMA3D	-4.315024	1.312699	-3.287139	0.00101211	0.06470832
MIR22HG	-3.275872	0.996881	-3.286122	0.00101577	0.06470832
ENSG00000234665	-4.876589	1.48745	-3.278489	0.001043645	0.06594748
ENSG00000260219	-3.60513	1.100372	-3.276284	0.001051829	0.06594748
ZNF416	-2.468212	0.753515	-3.275596	0.001054393	0.06594748
KRBA2	-3.013205	0.92151	-3.269857	0.00107602	0.06689475
KDM1B	-3.337258	1.021162	-3.268098	0.001082729	0.06690876
HLA.DQB2	-3.755159	1.150887	-3.262838	0.001103024	0.06775718
ENSG00000278831	-3.96782	1.21784	-3.258081	0.001121684	0.06825556
LINC02604	-3.68793	1.13225	-3.25717	0.00112529	0.06825556
IGIP	-2.823666	0.867845	-3.253652	0.00113932	0.06825556
NIPAL4	-2.640692	0.811742	-3.253119	0.001141459	0.06825556
MMP25.AS1	-2.570065	0.790197	-3.252435	0.001144207	0.06825556
ENSG00000236514	-2.463099	0.758815	-3.245983	0.001170458	0.06901608
HIST1H4E	-3.023311	0.931774	-3.244685	0.001175808	0.06901608
PRKCI	-2.818437	0.869191	-3.2426	0.001184445	0.06901608
PSPH	-2.510956	0.774411	-3.242409	0.001185238	0.06901608
ZNF512B	-3.77413	1.164434	-3.241172	0.001190394	0.06901608
PTPRJ	-3.921015	1.21073	-3.238555	0.001201368	0.06926325
ENSG00000272010	-3.93619	1.216526	-3.235598	0.001213881	0.06959587
NUDT8	-2.532327	0.783881	-3.2305	0.001235741	0.06992444
IFT46	-2.566647	0.794587	-3.230164	0.001237192	0.06992444

GCNT1	-2.716194	0.841049	-3.22953	0.001239939	0.06992444
ENSG00000238260	-3.639212	1.127459	-3.227801	0.001247458	0.06996613
EXD2	-3.169497	0.983091	-3.224012	0.001264083	0.07042853
ENSG00000240219	-4.307335	1.337035	-3.221557	0.00127496	0.07042853
RHPN1	-2.758824	0.856436	-3.221284	0.001276176	0.07042853
LEKR1	-3.756345	1.167902	-3.216319	0.001298464	0.07127737
PIK3R2	-2.912027	0.90758	-3.208563	0.001333999	0.07284056
MTFR2	-3.626688	1.130934	-3.206807	0.00134217	0.072901
MAP3K10	-3.695985	1.153443	-3.204307	0.001353882	0.07315217
AGBL5	-2.7487	0.858495	-3.201765	0.001365882	0.07330167
CAB39L	-3.563841	1.113878	-3.199489	0.001376715	0.07330167
CLCN4	-3.349161	1.047017	-3.198766	0.00138017	0.07330167
PAG1	-2.485823	0.777513	-3.197146	0.001387946	0.07330167
C17orf58	-3.868296	1.210253	-3.196271	0.001392163	0.07330167
TMPRSS13	-3.630838	1.139109	-3.187436	0.001435402	0.07505791
C1orf159	-2.768466	0.868813	-3.186491	0.001440098	0.07505791
CAPN3	-3.141899	0.986455	-3.18504	0.001447338	0.07505791
ENSG00000267469	-2.924437	0.920091	-3.178421	0.001480795	0.07640905
ENSG00000247765	-3.492106	1.099867	-3.175026	0.00149823	0.07670695
EHBP1L1	-2.181921	0.687348	-3.174406	0.001501434	0.07670695
ZC4H2	-3.407431	1.075152	-3.169254	0.001528307	0.07728382
NDC1	-2.490428	0.786022	-3.168393	0.00153284	0.07728382
ALDH7A1	-4.604992	1.45362	-3.167948	0.001535192	0.07728382
MACROD2	-3.201606	1.011404	-3.165507	0.001548127	0.07755666
DHX32	-2.873851	0.909186	-3.160904	0.001572805	0.07811062
PTGS1	-3.191974	1.010022	-3.160301	0.001576063	0.07811062
CSRNP2	-2.917432	0.923692	-3.158446	0.001586128	0.07811062
TNFSF8	-4.195958	1.329243	-3.156653	0.001595909	0.07811062
ENSG00000278932	-3.826659	1.21233	-3.156449	0.001597029	0.07811062
ENSG00000247121	-2.713115	0.860915	-3.151433	0.001624717	0.07908999
LDLR	-3.453749	1.097736	-3.146247	0.0016538	0.08012779
BBC3	-2.39947	0.763015	-3.14472	0.001662457	0.08017085
SHPK	-2.367097	0.753584	-3.141118	0.00168304	0.08078592
CKAP5	-2.321548	0.74021	-3.136336	0.001710729	0.08141824
SLC35D1	-2.254322	0.718825	-3.13612	0.001711992	0.08141824
ARL6	-3.821935	1.220222	-3.132163	0.001735235	0.08214506
ZNF142	-2.715292	0.868701	-3.125694	0.001773862	0.08338511
HMGN3.AS1	-4.13163	1.322359	-3.124439	0.001781442	0.08338511
ENSG00000280011	-3.152186	1.009106	-3.123742	0.00178567	0.08338511
PARPBP	-2.485522	0.79699	-3.118636	0.0018169	0.08409163
POLE	-2.798369	0.897314	-3.118605	0.001817096	0.08409163
IRAK1BP1	-2.902662	0.931573	-3.115873	NA	NA
AHI1	2.027739	0.651876	3.110619	0.001866959	0.08601346
ENSG00000258623	-2.860982	0.920162	-3.109216	0.001875843	0.08603869
NOTCH1	-2.140962	0.689154	-3.106651	0.001892194	0.08640463
PKD2	-3.310615	1.066902	-3.103016	0.001915593	0.08691742
ENSG00000268400	-3.350807	1.080106	-3.102294	0.001920269	0.08691742

TAF1A.AS1	-3.126288	1.008336	-3.100441	0.001932325	0.08708119
LINC02453	-3.635038	1.174494	-3.094983	0.001968241	0.08781241
ENSG00000261351	-2.736258	0.884164	-3.094739	0.001969858	0.08781241
KIAA1324L	-2.800463	0.90586	-3.091496	0.001991507	0.08781241
ADNP2	-3.074121	0.994777	-3.090263	0.001999797	0.08781241
DCBLD1	-3.178094	1.028483	-3.09008	0.002001025	0.08781241
CD38	1.779398	0.576028	3.089081	0.002007765	0.08781241
GNE	-2.497922	0.808643	-3.08903	0.002008113	0.08781241
ENSG00000260806	-4.227174	1.370825	-3.083671	0.002044633	0.08870691
BRICD5	-2.546845	0.825957	-3.083507	0.00204576	0.08870691
NUSAP1	-2.569461	0.833899	-3.081262	0.002061249	0.08900454
TNFSF13B	-3.043429	0.988527	-3.078751	NA	NA
ARMC5	-2.968671	0.964679	-3.077368	0.002088372	0.08979998
ZNF554	-3.666642	1.192678	-3.074293	0.002110021	0.089814
RAB40C	-2.836495	0.923511	-3.071424	0.002130406	0.089814
HOXB2	-2.313424	0.753701	-3.069421	0.002144744	0.089814
IQGAP2	-2.24789	0.73249	-3.068834	0.002148956	0.089814
ENSG00000276509	-1.963352	0.639802	-3.068684	0.002150039	0.089814
CD1D	1.917144	0.625299	3.065963	0.002169703	0.089814
KIFC2	-2.670815	0.871154	-3.065834	0.002170638	0.089814
MPND	-2.374872	0.774754	-3.065323	0.002174347	0.089814
PIGN	-3.101769	1.012309	-3.064054	0.002183593	0.089814
PSEN2	-2.497214	0.815196	-3.06333	0.002188885	0.089814
ENSG00000242349	-2.651453	0.865688	-3.062826	0.002192574	0.089814
MALT1	-1.834874	0.599208	-3.062166	0.002197417	0.089814
ZNF346	-1.945092	0.635483	-3.060807	0.002207414	0.089814
RGS12	-2.93364	0.959114	-3.058699	0.002223002	0.089814
HLX	-3.598661	1.17662	-3.058473	0.002224682	0.089814
TVP23C	-2.42819	0.794036	-3.058034	0.002227944	0.089814
GPR180	-2.317601	0.758391	-3.055946	0.002243519	0.09008995
CCDC152	-2.445733	0.800707	-3.054467	0.00225461	0.09018438
CORO2B	-3.47848	1.139942	-3.051453	0.002277365	0.09074288
NRIP2	-2.711394	0.88984	-3.047057	0.00231094	0.0912037
PEG10	-3.179617	1.043773	-3.046273	0.002316976	0.0912037
EAF1.AS1	-3.030847	0.995279	-3.045222	0.002325083	0.0912037
ENSG00000272058	-2.359317	0.774799	-3.045068	0.002326275	0.0912037
SOX12	-2.150159	0.706317	-3.044185	0.002333118	0.0912037
ENSG00000229043	-3.114231	1.023892	-3.041562	NA	NA
IQCN	-2.459249	0.808806	-3.040591	0.002361146	0.09195104
ENSG00000258646	-3.745214	1.232595	-3.038478	0.002377766	0.09225018
FAM174A	-2.076234	0.683849	-3.036098	0.002396618	0.09240297
ACVR1B	-4.688807	1.544415	-3.035976	NA	NA
C19orf44	-3.362897	1.107783	-3.0357	0.002399776	0.09240297
BPNT1	-2.540745	0.837685	-3.033057	0.002420902	0.09240297
ENSG00000262151	-2.867587	0.945548	-3.032725	0.002423564	0.09240297
VPS13B	-1.907605	0.629082	-3.032363	0.002426473	0.09240297
ENSG00000275672	-2.71387	0.895572	-3.030321	0.002442941	0.09255643

CABLES1	-2.205369	0.727931	-3.029642	0.00244844	0.09255643
RNF25	-2.041613	0.674343	-3.027561	0.002465363	0.09285602
ENSG00000111321	-2.838534	0.938003	-3.026145	0.002476935	0.09295261
FMN1	-4.79826	1.587407	-3.022704	0.002505273	0.09367543
ZNF597	-3.829147	1.267532	-3.020947	0.002519851	0.09386733
ZSWIM4	-3.197723	1.058615	-3.020667	NA	NA
LAMC1	-2.536916	0.840067	-3.019898	0.002528597	0.09386733
FBXO31	-2.524307	0.836297	-3.018433	0.002540855	0.09398433
FSIP2	-2.34746	0.779963	-3.009707	0.002614994	0.09615034
ENSG00000272716	-2.599152	0.864127	-3.007838	0.002631134	0.09615034
UTP15	-2.987143	0.993123	-3.007829	0.002631212	0.09615034
ZNF614	-2.569989	0.854689	-3.00693	0.002639007	0.09615034
IRS1	-2.329382	0.775213	-3.004828	0.002657312	0.09615034
C2CD2L	-3.96558	1.320108	-3.003982	0.002664715	0.09615034
ANKRD27	-2.110673	0.702718	-3.003583	0.002668205	0.09615034
ZNF726	-2.51181	0.836453	-3.002929	0.002673948	0.09615034
GIMAP1	-3.102008	1.033378	-3.001815	0.002683754	0.09616785
B4GALT5	-3.015814	1.007144	-2.994423	0.002749646	0.09818804
ENSG00000257764	-2.775421	0.927253	-2.993163	0.002761019	0.09821684
PAFAH2	-2.670179	0.892934	-2.990341	0.002786657	0.09821684
PDZD8	-2.204744	0.737329	-2.990176	0.002788167	0.09821684
PYGM	-2.5112	0.839828	-2.990137	0.002788521	0.09821684
HIST1H2AK	-4.554066	1.524851	-2.986564	0.002821315	0.0990339
DIP2C	-2.687206	0.900238	-2.984994	0.002835835	0.09920616
TNRC18	-3.03931	1.018841	-2.983106	0.002853395	0.09948325
PRDM11	-3.944431	1.323463	-2.980386	0.002878855	0.09979265
LINC01481	-4.620901	1.551017	-2.979272	0.002889343	0.09979265
C4orf46	-2.555922	0.858162	-2.978366	0.002897894	0.09979265
ZNF469	-5.29595	1.778332	-2.978043	0.002900949	0.09979265
DGCR6	-4.150123	1.394952	-2.9751	0.002928927	0.10042037
KIF1B	-2.956675	0.994292	-2.973648	0.002942824	0.10056272
LINC00342	-3.511573	1.184142	-2.965499	0.003021919	0.10226809
XXYLT1.AS2	-2.748232	0.926743	-2.965473	0.003022176	0.10226809
MSH5	-3.0371	1.024352	-2.964899	0.003027827	0.10226809
ALDH2	-2.076013	0.700306	-2.964438	0.003032368	0.10226809
SLC35G5	-3.330036	1.124038	-2.962566	0.003050868	0.10255687
TNNT3	-4.390437	1.483427	-2.959657	0.003079813	0.10307844
BBS7	-2.192699	0.741026	-2.959003	0.00308636	0.10307844
PINK1	-2.459105	0.832742	-2.953021	0.003146801	0.1045856
CLDN12	-3.03685	1.028556	-2.952536	0.003151756	0.1045856
ENSG00000272182	-3.509284	1.189724	-2.949662	0.003181221	0.10510526
ENSG00000261188	-2.656217	0.90071	-2.949025	0.003187786	0.10510526
EID2	-2.924313	0.993396	-2.943755	0.003242566	0.10633117
ENSG00000227908	-5.20051	1.76766	-2.942031	0.003260672	0.10633117
LZTR1	-2.483661	0.844344	-2.941527	0.003265984	0.10633117
SPATA2	-2.326696	0.791102	-2.941084	0.003270661	0.10633117
LRRC8A	-2.86775	0.975249	-2.940533	0.003276484	0.10633117

DCUN1D4	-2.053278	0.698653	-2.938912	0.00329367	0.10649917
ZNF555	-2.788479	0.949075	-2.9381	0.0033023	0.10649917
ZKSCAN4	-2.770595	0.944352	-2.933859	0.003347765	0.1075668
TRIM35	-3.327027	1.13435	-2.932981	0.003357241	0.1075668
TTC21B	-2.088195	0.712363	-2.931364	0.003374768	0.1075668
ENSG00000274712	-2.025255	0.691139	-2.930313	0.003386209	0.1075668
EVI5	-1.655924	0.565125	-2.930193	0.00338752	0.1075668
C1orf198	-2.976125	1.016112	-2.928934	0.003401267	0.10767201
LINC01754	-4.172716	1.425937	-2.926298	0.003430219	0.1080245
PAQR3	-2.278466	0.778692	-2.926016	0.003433337	0.1080245
SEC22A	-1.944372	0.665344	-2.922354	0.00347396	0.10897043
ZNF75D	-2.691371	0.922394	-2.917811	0.003524977	0.11023564
ENSG00000261669	-2.186248	0.749858	-2.915551	0.003550613	0.1107019
RFNG	-2.383704	0.818616	-2.911869	0.003592733	0.11123969
FBRSL1	-1.99905	0.686607	-2.911491	0.003597081	0.11123969
COCH	-2.2071	0.758463	-2.909965	0.003614689	0.11123969
DDX31	-3.104492	1.0669	-2.909825	0.003616313	0.11123969
ZNF354C	-2.817994	0.968697	-2.909057	0.003625202	0.11123969
ZBTB8A	-3.365899	1.157293	-2.908426	0.003632536	0.11123969
WDR81	-1.914841	0.658612	-2.907391	0.003644573	0.11127809
POU5F2	-2.908996	1.001275	-2.905292	0.003669102	0.11147516
COL4A4	-5.206904	1.792399	-2.904991	0.003672631	0.11147516
ENSG00000276136	-2.286916	0.787886	-2.902599	0.003700804	0.11200087
TRIM25	-1.871778	0.645149	-2.901313	0.003716029	0.1121328
RHEBL1	-2.281659	0.786964	-2.899319	0.00373974	0.1125193
LINC01772	-2.409876	0.831492	-2.898256	0.003752438	0.11257315
PHLPP2	-3.210443	1.109391	-2.893878	0.003805165	0.11300656
NEURL4	-2.584395	0.893088	-2.893776	0.003806399	0.11300656
CPNE8	-2.572049	0.888917	-2.893464	0.00381018	0.11300656
FAM234B	-2.037276	0.704106	-2.893422	0.003810686	0.11300656
GTF3C1	-2.853905	0.987238	-2.890797	NA	NA
ENSG00000267563	-2.464181	0.853272	-2.88792	0.003877979	0.11467263
FAM8A1	-2.895748	1.0039	-2.8845	0.003920358	0.11559456
BRAF	-1.690877	0.587296	-2.879087	0.003988285	0.11726241
KLHL42	-2.54441	0.88443	-2.876893	0.004016123	0.11774543
ENSG00000276718	-5.100237	1.774677	-2.873896	0.004054426	0.11799076
LLGL1	-2.721592	0.947064	-2.873714	0.004056757	0.11799076
LINC00910	-2.474587	0.861155	-2.873567	NA	NA
ENSG00000268093	-4.656927	1.620944	-2.872971	0.00406631	0.11799076
EFL1	-3.00737	1.046891	-2.872667	0.004070224	0.11799076
CECR2	-2.079626	0.724659	-2.869801	0.004107304	0.11873215
C8orf37	-2.803034	0.977263	-2.868249	0.004127501	0.1189827
MAP3K9	-2.170958	0.75762	-2.865498	0.004163543	0.11968736
ENSG00000260101	-2.554617	0.89315	-2.860233	0.004233301	0.12106036
WASHC5	-2.009818	0.702703	-2.860123	0.004234766	0.12106036
ENSG00000272993	-2.300559	0.804941	-2.858048	0.004262565	0.12128417
WDR24	-2.493559	0.87255	-2.857784	0.0042661	0.12128417

ZNF660	-2.254017	0.78955	-2.854811	0.004306238	0.12193111
DENND4C	-2.140218	0.749809	-2.854351	0.004312486	0.12193111
LSG1	-1.854053	0.650005	-2.852368	0.004339488	0.12235933
EML6	-2.529797	0.887681	-2.849893	0.004373397	0.12297946
ENSG00000273329	-2.212184	0.776751	-2.847995	0.004399564	0.12337909
AGFG2	-4.940277	1.735397	-2.846771	NA	NA
SERPINE2	-2.463554	0.865532	-2.846288	0.004423215	0.12370618
ERP27	-3.668763	1.289841	-2.844352	0.004450186	0.1241241
PTP4A3	-2.185186	0.768806	-2.842313	0.004478749	0.12458408
PASK	-2.158233	0.75972	-2.840828	0.004499661	0.12482931
TWNK	-2.201728	0.77611	-2.836877	0.004555715	0.12597515
KANSL1L	-2.546907	0.898	-2.8362	0.004565379	0.12597515
PIGA	-2.402657	0.848008	-2.833294	0.004607101	0.12674087
MDFIC	-2.261188	0.798284	-2.83256	0.004617691	0.12674087
STPG1	-2.882077	1.018851	-2.828753	0.004672969	0.12779449
DONSON	-3.41808	1.208592	-2.82815	0.004681789	0.12779449
MAPK11	-2.714472	0.96007	-2.827369	0.004693228	0.12779449
ENSG00000228037	-3.512041	1.242613	-2.826335	0.004708404	0.12787034
ZFYVE1	-2.273949	0.805157	-2.824229	0.004739451	0.12803016
SDR42E1	-2.713906	0.961305	-2.823148	0.004755462	0.12803016
ZXDC	-2.224262	0.788019	-2.822598	0.004763624	0.12803016
PRMT7	-1.904927	0.674889	-2.822579	0.004763913	0.12803016
LETM2	-3.779077	1.339806	-2.820615	0.004793167	0.12815129
WDPCP	-2.001736	0.709744	-2.820363	0.004796933	0.12815129
ENSG00000280007	-2.478882	0.879105	-2.819779	0.004805673	0.12815129
ENSG00000277511	-2.388774	0.847697	-2.817957	0.004833027	0.12854855
TMCC3	-4.675816	1.661382	-2.814413	0.004886637	0.12964035
DCAKD	-2.290402	0.814194	-2.813091	0.004906782	0.12984099
ZDBF2	-2.780618	0.988902	-2.811823	0.004926153	0.12986216
CD68	-2.672293	0.950523	-2.811393	0.004932749	0.12986216
NOD1	-2.245561	0.799083	-2.810174	0.004951479	0.13002358
FAM71D	-3.006419	1.07051	-2.808399	0.004978855	0.13016394
CAPN2	-2.160449	0.769338	-2.808192	0.00498205	0.13016394
ZNF839	-2.845793	1.015604	-2.802069	0.005077607	0.13232552
MAPK13	-2.02279	0.722392	-2.800127	0.005108249	0.13278873
PPM1F	-2.047846	0.731763	-2.798511	0.005133881	0.13311972
ST3GAL6	-3.510679	1.255256	-2.796783	0.005161411	0.13349814
MMP17	-2.96331	1.060481	-2.794306	0.005201119	0.13418886
C21orf58	-2.910166	1.042326	-2.791991	0.005238476	0.13481564
TKFC	-1.993677	0.714379	-2.790783	0.005258072	0.13498334
SERPINF2	-3.484994	1.249696	-2.788674	0.005292426	0.13533341
ZSWIM3	-3.028297	1.086058	-2.788337	0.005297936	0.13533341
PSMC3IP	-2.768676	0.993697	-2.786239	0.005332356	0.13562557
FHIT	-1.983024	0.711772	-2.786038	0.005335657	0.13562557
ENSG00000270175	-4.448271	1.598613	-2.782582	0.005392827	0.13617853
TCAP	-3.739004	1.343807	-2.782396	0.005395918	0.13617853
ACTR3B	-2.207652	0.793541	-2.782026	0.00540207	0.13617853

ENSG00000260267	-2.155431	0.774906	-2.781538	0.005410193	0.13617853
GALE	-2.902035	1.043756	-2.780378	0.005429569	0.13633371
TRABD2A	-2.289072	0.823813	-2.778631	0.005458856	0.13650732
HAUS5	-2.423989	0.87266	-2.777703	0.005474469	0.13650732
TRAF3IP2.AS1	-2.012394	0.724508	-2.777602	0.005476166	0.13650732
SCRG1	-2.566507	0.924882	-2.774957	0.005520904	0.13702705
FXR2	-2.183157	0.78678	-2.7748	0.005523571	0.13702705
SGK1	-3.638223	1.312977	-2.770973	0.005588905	0.13796671
ZNF565	-2.398648	0.865693	-2.770783	0.005592166	0.13796671
ZBTB20.AS2	-3.289027	1.187528	-2.769642	0.005611795	0.13796671
ENSG00000142046	-2.273516	0.820924	-2.76946	0.005614924	0.13796671
ZDHHC23	-2.059221	0.743766	-2.768639	0.005629089	0.13798621
ZNF549	-2.140771	0.774448	-2.764254	0.005705309	0.1395232
KCNMB3	-1.790023	0.647919	-2.762724	0.005732116	0.13956936
RWDD2B	-2.219745	0.803498	-2.762603	0.005734245	0.13956936
ENSG00000274341	-2.983169	1.081193	-2.759145	0.005795283	0.14062028
UBOX5	-2.729669	0.990093	-2.756983	0.005833739	0.14062028
PREP	-2.382649	0.864317	-2.756683	0.005839086	0.14062028
AMMECR1L	-1.905445	0.69141	-2.755883	0.005853397	0.14062028
MROH8	-4.097207	1.486843	-2.755642	0.005857701	0.14062028
RNASEL	-1.949564	0.707594	-2.755202	0.005865591	0.14062028
SDHAF4	-2.180274	0.791446	-2.7548	0.005872804	0.14062028
GPSM2	-2.193564	0.796903	-2.752612	0.005912187	0.14123558
ATL1	-2.789411	1.013699	-2.751715	0.005928418	0.14129624
PHC2	-2.543746	0.924966	-2.750097	0.005957758	0.14166834
VWA8	-3.02578	1.101694	-2.746479	0.006023871	0.14263735
FAR1	-2.343563	0.853455	-2.745971	0.006033203	0.14263735
NPIPA1	-2.298791	0.837328	-2.745388	0.006043939	0.14263735
F8A1	-2.492492	0.908555	-2.74336	0.006081401	0.14263735
SRF	-1.888021	0.688231	-2.743297	0.006082563	0.14263735
SNAPC4	-2.298547	0.838196	-2.742256	0.00610188	0.14263735
ZSCAN5A	-2.280244	0.831639	-2.741867	0.006109113	0.14263735
BLM	-1.744419	0.636357	-2.74126	0.0061204	0.14263735
FSD1L	-2.247186	0.819804	-2.741126	0.006122902	0.14263735
ALYREF	-2.185845	0.797655	-2.740339	0.006137592	0.14265753
RPL39L	-2.323903	0.850595	-2.732093	0.006293344	0.14567942
PPME1	-2.188206	0.800965	-2.731962	0.006295835	0.14567942
NPIPB2	-4.028795	1.476905	-2.727864	0.006374588	0.14717169
L3MBTL1	-2.223445	0.81611	-2.724443	0.006441013	0.14837333
WASF1	-2.040136	0.749285	-2.722778	0.006473553	0.14879079
NOP14.AS1	-2.686564	0.987455	-2.720695	0.006514484	0.14939883
ENSG00000261732	-2.055747	0.756156	-2.718681	0.006554269	0.14943477
ZBTB49	-2.661284	0.978974	-2.718442	0.006559011	0.14943477
ENSG00000279467	-3.071414	1.129872	-2.718373	0.00656038	0.14943477
LRR1	-1.769122	0.650966	-2.717688	0.006573971	0.14943477
TRUB1	-2.026609	0.746045	-2.71647	0.006598224	0.14965642
MTHFD1L	-2.904172	1.069923	-2.714376	NA	NA

MTFR1	-1.863289	0.686702	-2.713385	0.00665996	0.15072541
LSM14B	-2.4738	0.911996	-2.712511	0.006677557	0.15079298
DGAT1	-2.526286	0.931934	-2.710799	0.006712123	0.15106579
ENSG00000225205	-2.397089	0.884649	-2.70965	0.006735421	0.15106579
TTLL12	-2.409049	0.889468	-2.708414	0.006760553	0.15106579
SREBF2.AS1	-2.501486	0.923816	-2.707776	0.006773569	0.15106579
COG4	-1.629328	0.601728	-2.707748	0.006774135	0.15106579
MCM2	-2.748416	1.015142	-2.707419	0.006780853	0.15106579
SHLD3	-2.035156	0.75186	-2.706831	0.006792888	0.15106579
MTURN	-2.563438	0.947262	-2.706154	0.006806744	0.15106579
CAMKK1	-2.695607	0.997329	-2.702827	0.006875258	0.15225892
HEATR5A	-2.288362	0.847488	-2.70017	0.006930397	0.15302533
CCDC138	-2.381702	0.882199	-2.699733	0.006939521	0.15302533
ENSG00000227540	-2.384569	0.883801	-2.698083	0.006974015	0.15309561
DNMBP	-2.080635	0.771302	-2.697562	0.006984919	0.15309561
SGSM2	-1.984638	0.736037	-2.696383	0.007009696	0.15309561
LINC00294	-2.494454	0.925161	-2.696238	0.007012749	0.15309561
DZANK1	-3.520174	1.305797	-2.695805	0.00702188	0.15309561
SRD5A1	-1.731641	0.642498	-2.695169	0.007035303	0.15309561
SPINT1.AS1	-3.430892	1.273278	-2.694535	0.007048687	0.15309561
CLHC1	-2.621487	0.973107	-2.693935	0.007061387	0.15309561
SCMH1	-1.694768	0.629551	-2.692026	0.00710194	0.15330575
TRAPPC9	-2.49637	0.92742	-2.691735	0.007108137	0.15330575
GAB1	-2.278267	0.846554	-2.691226	0.007118999	0.15330575
RAD52	-2.464795	0.916046	-2.690687	0.0071305	0.15330575
DOCK9	-2.744302	1.020623	-2.688849	0.007169874	0.15360348
TRAF1	-2.721323	1.012151	-2.688652	0.007174116	0.15360348
AAK1	-2.693208	1.002816	-2.685646	NA	NA
LIX1.AS1	-2.340542	0.872156	-2.683627	0.007282838	0.15560846
PPP1R13L	-2.24596	0.838338	-2.679063	0.007382851	0.15741946
FAM149B1	-2.182696	0.815005	-2.678138	0.007403263	0.15748993
ICE1	-1.937014	0.723433	-2.677532	0.007416677	0.15748993
FPGT	-2.575907	0.962336	-2.676724	0.007434575	0.15754582
ETFBKMT	-1.884376	0.704913	-2.673204	0.007513051	0.15884199
KCTD21.AS1	-2.592371	0.970225	-2.671927	0.007541715	0.15884199
ZNF337	-2.293866	0.858615	-2.671589	0.007549295	0.15884199
MTRNR2L3	-4.918176	1.841163	-2.671233	0.007557308	0.15884199
TTC31	-1.555504	0.582678	-2.669575	0.007594727	0.15910635
ENSG00000261220	-3.73044	1.398367	-2.667713	0.007636951	0.15910635
MAMSTR	-2.821283	1.057763	-2.667218	0.007648202	0.15910635
ENSG00000277972	-1.644373	0.616584	-2.666908	0.007655272	0.15910635
ZSCAN32	-1.78644	0.669916	-2.666663	0.00766084	0.15910635
ENSG00000255328	-3.553741	1.332823	-2.666327	0.007668507	0.15910635
ENSG00000278390	-2.153813	0.808067	-2.665389	0.007689933	0.15910635
MYH11	-2.046721	0.76793	-2.665245	0.007693224	0.15910635
HSPA13	-1.850309	0.694995	-2.662335	0.007760068	0.16002379
FECH	-2.132023	0.800921	-2.661965	0.007768597	0.16002379

ENSG00000253645	-2.337086	0.878481	-2.660373	0.007805413	0.16046188
MRTFA	-2.040788	0.767463	-2.659134	0.007834186	0.1607332
ZNF362	-2.343427	0.881425	-2.65868	NA	NA
POFUT1	-2.350643	0.884564	-2.657403	0.00787452	0.16124018
MBTPS2	-2.099855	0.790821	-2.655285	0.007924132	0.16166952
ZNF471	-2.704981	1.01876	-2.655171	0.00792682	0.16166952
CEP97	-1.882584	0.709709	-2.652613	0.00798713	0.16257827
AMT	-2.855463	1.077197	-2.650827	0.0080295	0.16311898
LIN37	-1.996344	0.753964	-2.647796	0.008101829	0.16426497
GTSF1	-2.256642	0.852645	-2.646637	0.008129658	0.16450602
NCDN	-3.228072	1.221229	-2.643299	0.00821025	0.1658117
ENSG00000228989	-2.570034	0.972888	-2.641654	0.008250239	0.16629388
COLGALT1	-1.933286	0.731961	-2.641242	NA	NA
PLEKHM3	-4.795651	1.816201	-2.640485	0.008278734	0.16654296
CTDP1	-3.400033	1.288487	-2.638779	0.008320524	0.16676871
C9orf72	-1.796604	0.680865	-2.638707	0.008322276	0.16676871
MOV10	-2.204912	0.836027	-2.637371	0.008355148	0.16710295
KIF21B	-2.49057	0.945289	-2.634718	0.008420714	0.16808852
ITGA5	-3.203737	1.217106	-2.632257	0.008481962	0.16876272
FAM83G	-2.237982	0.850498	-2.63138	0.008503893	0.16876272
TAF5L	-2.272214	0.863586	-2.631138	0.008509941	0.16876272
SPTY2D1	-1.715862	0.652235	-2.630741	0.008519901	0.16876272
PIAS4	-1.81109	0.688679	-2.629803	0.008543444	0.16881514
NIPA1	-2.793187	1.06252	-2.628833	0.008567844	0.16881514
SEPT7.AS1	-2.616331	0.995301	-2.628683	0.008571621	0.16881514
TADA2A	-1.881105	0.716564	-2.625172	0.008660524	0.17024116
CASP10	-2.333965	0.889561	-2.623726	0.008697364	0.1706403
SERPINB8	-2.257115	0.861008	-2.621479	0.008754918	0.17144355
QSOX2	-1.603941	0.612526	-2.618566	0.008830029	0.17209907
NINJ2	-1.870061	0.714414	-2.617616	0.008854632	0.17209907
ZNF608	-1.805078	0.689685	-2.617251	0.008864115	0.17209907
ZNF385A	-1.952736	0.746144	-2.617104	0.008867923	0.17209907
HSDL1	-1.64867	0.629995	-2.616956	0.008871774	0.17209907
SLC27A1	-4.023208	1.538169	-2.615582	0.008907557	0.17246902
FBP1	-1.894477	0.724586	-2.614563	0.00893417	0.17266037
C7orf26	-1.911894	0.732477	-2.610176	0.009049575	0.17416752
DUSP3	-1.927416	0.73852	-2.609837	0.009058536	0.17416752
ENSG00000276075	-2.917671	1.118347	-2.608914	0.009083011	0.17416752
HYI	-1.878512	0.720065	-2.608811	0.009085752	0.17416752
SMCO4	-2.615153	1.002763	-2.607946	0.009108722	0.17416752
NPIPB15	-2.451242	0.939976	-2.60777	0.009113417	0.17416752
PIGK	-1.931095	0.741434	-2.60454	0.00919977	0.17549284
TFB1M	-1.578768	0.606892	-2.6014	0.009284422	0.17678087
ENSG00000215014	-2.801157	1.076984	-2.600928	NA	NA
NOL6	-2.489558	0.957523	-2.599999	0.009322398	0.17702081
PAXBP1	-2.089184	0.803634	-2.599671	0.00933133	0.17702081
NUP50.DT	-2.517683	0.969059	-2.598069	0.009374954	0.17752207

GALC	-2.280506	0.878197	-2.596805	0.009409522	0.1778503
GINS4	-1.839745	0.709068	-2.594597	0.009470185	0.17839939
CRAMP1	-2.056051	0.792517	-2.594331	0.009477523	0.17839939
CNPY4	-2.443577	0.942061	-2.593863	0.009490433	0.17839939
IGHG2	-2.737026	1.056464	-2.590742	0.009576937	0.17969817
MAPRE3	-3.143797	1.213942	-2.589743	0.009604761	0.17973139
MATK	-4.65357	1.797142	-2.589428	0.009613539	0.17973139
ZNF496	-1.889087	0.730766	-2.585078	0.009735702	0.1814126
ENSG00000268858	-2.298437	0.889278	-2.584611	0.009748886	0.1814126
SLC20A2	-1.7796	0.688606	-2.584353	0.009756201	0.1814126
FCRLB	-2.67651	1.036297	-2.582762	0.00980128	0.18192303
IPPK	-2.300024	0.892202	-2.577918	0.009939745	0.18416189
ARHGAP31	-5.473319	2.124305	-2.576522	0.009979987	0.18457611
GINS1	-4.632642	1.799777	-2.574008	0.010052784	0.18558986
WEE1	-2.260219	0.878344	-2.573273	0.01007417	0.18565256
SLC25A23	-2.617093	1.017694	-2.571592	0.010123214	0.18590113
ENSG00000273064	-2.861206	1.112627	-2.571576	0.010123685	0.18590113
FDXACB1	-2.443004	0.950896	-2.569159	0.010194571	0.18687028
MAN1B1.DT	-2.501584	0.974378	-2.567366	0.010247445	0.18750644
TSPAN5	-2.188559	0.853481	-2.564273	0.010339215	0.18885079
KLHL9	-1.914675	0.746871	-2.563595	0.010359424	0.18888561
FURIN	-1.778086	0.694466	-2.560364	0.010456249	0.19031479
PUS10	-1.905087	0.74482	-2.55778	0.010534263	0.19119454
ACTL6A	-1.569523	0.613685	-2.557537	0.010541637	0.19119454
ZHX1	-2.380432	0.932265	-2.553385	0.010668152	0.19296921
ATAD3B	-1.876136	0.734846	-2.5531	0.010676882	0.19296921
GZF1	-2.0579	0.807057	-2.549881	0.010775977	0.19441973
ENSG00000071655	-1.698338	0.666368	-2.548647	0.010814158	0.19476808
HEATR3	-2.168558	0.851391	-2.547076	0.010862974	0.19530644
FYN	-1.962515	0.770593	-2.546758	NA	NA
CROT	-2.168574	0.852307	-2.544357	0.010947919	0.19649134
FLNB	-2.112013	0.83083	-2.542052	0.011020364	0.19744819
N6AMT1	-1.982374	0.780658	-2.539364	0.011105437	0.19840002
APEX2	-1.534189	0.604461	-2.538109	0.011145314	0.19840002
ZBED2	-2.400715	0.945896	-2.538034	0.011147717	0.19840002
TAF4	-2.005696	0.790404	-2.537558	0.011162899	0.19840002
FAM220A	-2.216501	0.873681	-2.536969	0.011181685	0.19840002
ZNF781	-1.894908	0.747143	-2.536204	0.011206152	0.19840002
FAM135A	-1.773748	0.699388	-2.536144	0.011208063	0.19840002
DOCK6	-3.812249	1.50465	-2.533645	0.011288315	0.19947844
UBE2Q2	-1.561904	0.617083	-2.531106	0.011370338	0.20058442
GALNT7	-1.857158	0.734144	-2.529692	0.011416255	0.20081972
ENSG00000232010	-3.10423	1.227212	-2.529498	0.011422595	0.20081972
GNGT2	-2.233776	0.88341	-2.528584	0.011452376	0.20086926
DMWD	-2.014541	0.796823	-2.528217	0.011464341	0.20086926
LINC00494	-1.821989	0.720945	-2.527223	0.011496844	0.20098192
SMARCD2	-1.601646	0.633904	-2.52664	0.011515943	0.20098192

GGACT	-4.514342	1.787396	-2.525654	0.011548332	0.20098192
ENSG00000238045	-2.096237	0.830126	-2.525202	0.011563181	0.20098192
TANC2	-2.841946	1.1255	-2.525051	0.011568145	0.20098192
LIN7B	-1.949334	0.772718	-2.522699	0.011645816	0.20181863
ZNF236	-1.566845	0.621181	-2.522364	0.011656913	0.20181863
TTC33	-1.627234	0.64568	-2.520185	0.01172931	0.20181863
PRR7	-2.176313	0.86357	-2.520135	0.011730997	0.20181863
ENSG00000223473	-2.254932	0.894986	-2.519518	0.011751552	0.20181863
TSBP1.AS1	-2.795586	1.109572	-2.519517	0.011751592	0.20181863
MPZL1	-1.653972	0.656477	-2.519469	0.011753198	0.20181863
MOXD1	-4.361216	1.732506	-2.517287	0.011826248	0.20251187
PCNT	-1.894491	0.752651	-2.517091	0.011832816	0.20251187
PDCL	-1.439794	0.572329	-2.515676	0.01188042	0.20276594
NAIF1	-1.941734	0.771913	-2.515483	0.011886957	0.20276594
EIF3C	-1.70349	0.677532	-2.514256	0.011928395	0.20292589
URB1.AS1	-2.449908	0.974656	-2.513613	NA	NA
ELMO2	-1.923168	0.765161	-2.513417	0.011956803	0.20292589
ENSG00000271109	-1.783643	0.709671	-2.513339	0.011959448	0.20292589
PHYH	-2.323496	0.924635	-2.512881	0.011974987	0.20292589
PSTK	-2.12655	0.84676	-2.511395	0.012025502	0.20344784
WDYHV1	-2.063101	0.822066	-2.509654	0.012084956	0.20366749
KLHDC7B	-2.237573	0.891833	-2.508959	0.012108768	0.20366749
HS3ST3B1	-2.290317	0.912981	-2.508613	0.012120605	0.20366749
ZNF280B	-2.883679	1.149621	-2.508372	0.012128875	0.20366749
TCEANC	-1.728631	0.689332	-2.50769	0.012152315	0.20366749
IFIT5	-1.539161	0.61415	-2.506166	0.012204817	0.20366749
ENSG00000268713	-2.998346	1.196655	-2.505605	0.012224201	0.20366749
RAPGEF6	-1.515322	0.60479	-2.505533	0.012226707	0.20366749
IRGQ	-2.199758	0.878111	-2.505102	0.012241602	0.20366749
ZNF354B	-1.87303	0.748365	-2.50283	0.012320487	0.20366749
RCBTB2	-4.605208	1.840134	-2.502648	0.012326815	0.20366749
ATXN7L1	-1.811107	0.723706	-2.502545	0.012330382	0.20366749
WHAMM	-1.458332	0.582757	-2.502471	0.012332967	0.20366749
TBXA2R	-2.312759	0.924348	-2.502043	0.012347881	0.20366749
JADE3	-2.17026	0.867535	-2.50164	0.012361954	0.20366749
STK11IP	-1.717696	0.686698	-2.501385	0.012370847	0.20366749
LINC02576	-2.669145	1.067337	-2.500751	0.012393014	0.20366749
PCNX3	-2.079896	0.831715	-2.500731	0.012393719	0.20366749
SAP130	-1.88591	0.754767	-2.498666	0.012466172	0.20426854
FASTKD1	-2.161588	0.865133	-2.498561	0.012469882	0.20426854
FAM241A	-2.097998	0.839739	-2.498391	NA	NA
ABHD16A	-3.409827	1.365392	-2.497325	0.012513421	0.20461067
HIVEP1	-2.219667	0.888989	-2.496844	0.012530421	0.20461067
ENSG00000278743	-1.639579	0.657055	-2.495345	0.012583454	0.20487526
ENSG00000226571	-2.435371	0.975997	-2.495264	0.012586329	0.20487526
ENSG00000258056	-2.588499	1.037666	-2.494538	0.012612111	0.20490112
ACTR8	-1.778061	0.713098	-2.493431	0.012651505	0.20490112

CHKA	-3.680544	1.476154	-2.493334	0.012654979	0.20490112
UNG	-1.82293	0.731223	-2.492987	0.012667336	0.20490112
BRPF3	-2.427101	0.974534	-2.490525	0.012755463	0.20600372
ARL10	-1.894874	0.762117	-2.486329	0.01290685	0.20812295
BCAS3	-1.895297	0.762871	-2.484427	0.012976027	0.20868363
KDM1A	-1.54447	0.62186	-2.483631	0.013005038	0.20868363
DRAM1	-2.638802	1.062911	-2.482618	0.013042075	0.20868363
ENSG00000257303	-1.928375	0.777204	-2.481171	0.013095162	0.20868363
ENSG00000272189	-2.83682	1.143493	-2.480838	0.013107398	0.20868363
GOLGA1	-2.195272	0.884902	-2.480808	0.013108485	0.20868363
CD27	-3.194143	1.287634	-2.480628	0.013115098	0.20868363
PUDP	-2.222122	0.896008	-2.480026	0.013137281	0.20868363
PTPDC1	-2.223415	0.896549	-2.479972	0.013139273	0.20868363
TMEM169	-2.674933	1.07871	-2.479752	0.013147366	0.20868363
ENSG00000237596	-2.269146	0.915482	-2.478636	0.013188582	0.20868363
ENSG00000267058	-2.09257	0.844285	-2.478513	0.01319312	0.20868363
RAPGEF2	-1.986694	0.801666	-2.478206	0.013204497	0.20868363
ZSWIM1	-1.965688	0.793461	-2.47736	0.013235846	0.20885922
TMEM8A	-1.939871	0.783548	-2.475751	0.013295641	0.20948247
ARRDC3.AS1	-2.934488	1.185911	-2.474459	0.013343802	0.20978084
ANK1	-2.104216	0.850479	-2.474153	0.013355234	0.20978084
SSX2IP	-2.449766	0.990676	-2.472822	0.01340509	0.21024396
ARNT	-1.644841	0.6663	-2.46862	0.013563525	0.21240604
CD24	-1.189637	0.482108	-2.467572	0.013603283	0.21270589
COL4A3	-5.203833	2.110128	-2.466121	0.013658503	0.21299641
RTN4IP1	-2.124972	0.861873	-2.465527	0.013681179	0.21299641
ENSG00000272918	-1.958428	0.794346	-2.465459	0.013683781	0.21299641
ATG4A	-2.297916	0.933399	-2.46188	0.013821085	0.2145592
PELI2	-2.004812	0.814501	-2.461399	0.013839652	0.2145592
RNF144B	-1.72035	0.698983	-2.46122	0.013846553	0.2145592
MTA3	-1.840617	0.748528	-2.458982	0.013933144	0.21538984
GCH1	-1.733149	0.705176	-2.457751	0.013980989	0.21538984
RPS6KA2	-1.829861	0.74453	-2.457739	0.013981486	0.21538984
S100A13	-2.052986	0.835334	-2.457683	0.013983642	0.21538984
CARMIL2	-1.681087	0.684188	-2.457053	0.014008185	0.2154463
LIX1L.AS1	-3.280673	1.335683	-2.456177	0.014042416	0.21560464
MARCHF8	-1.529544	0.62285	-2.45572	0.014060264	0.21560464
ENSG00000272927	-1.917071	0.781393	-2.453402	0.014151226	0.21667753
HIST1H3E	-1.866497	0.762011	-2.449436	0.014308004	0.21866242
CDK7	-1.787812	0.730001	-2.449053	0.014323236	0.21866242
ENSG00000279080	-1.741648	0.711592	-2.447537	0.014383619	0.21925989
PIGW	-1.885263	0.771045	-2.445076	0.014482165	0.2204365
PRKCZ	-2.697087	1.103885	-2.443269	0.014554903	0.22083485
KLK1	-2.27048	0.929429	-2.442876	0.014570732	0.22083485
ZNF446	-1.821065	0.745473	-2.442832	0.014572532	0.22083485
NBPF19	-1.713519	0.702055	-2.440719	0.014658072	0.22148442
ZNF417	-1.910365	0.782726	-2.440656	0.014660623	0.22148442

MIF.AS1	-2.51313	1.029894	-2.440184	0.014679781	0.22148442
PEX3	-2.027194	0.831965	-2.436634	0.014824685	0.22259626
ARFGAP1	-1.655582	0.679613	-2.436065	0.014848011	0.22259626
ZNF529.AS1	-2.102203	0.863134	-2.435548	0.014869239	0.22259626
CEP128	-2.002381	0.822207	-2.435372	0.014876468	0.22259626
BAP1	-1.470285	0.603756	-2.43523	0.014882316	0.22259626
PLCB3	-2.817153	1.156839	-2.435216	0.014882889	0.22259626
USP12	-1.741953	0.715648	-2.434092	0.014929218	0.22277034
NAB1	-2.276905	0.935502	-2.433886	0.014937701	0.22277034
ZNF771	-2.635878	1.08335	-2.43308	0.014970977	0.22294442
GPR107	-3.712455	1.526613	-2.431824	NA	NA
MCU	-2.469967	1.016825	-2.429098	0.015136444	0.22424033
RNF103	-1.828102	0.752684	-2.428776	0.015149869	0.22424033
HELB	-1.958322	0.806314	-2.428735	0.01515159	0.22424033
ZNF384	-1.501105	0.618114	-2.428524	0.015160421	0.22424033
SLC35B4	-1.923403	0.792054	-2.428375	0.015166643	0.22424033
TMEM273	-2.128324	0.877134	-2.426453	0.015247226	0.22451229
C19orf47	-2.419183	0.997214	-2.425942	0.015268694	0.22451229
TDRD7	-1.972215	0.81299	-2.425878	0.015271419	0.22451229
DGKQ	-1.768343	0.729157	-2.425189	0.015300402	0.22451229
CHL1	-2.650204	1.093021	-2.424659	0.015322756	0.22451229
PABPC1L	-2.140863	0.883033	-2.424443	0.015331899	0.22451229
ALMS1	-2.135024	0.880685	-2.424276	0.015338932	0.22451229
SORBS3	-2.155996	0.889556	-2.423677	0.015364245	0.22451229
CCDC51	-2.21927	0.916169	-2.422337	0.015421047	0.22451229
SELENOO	-1.711069	0.70662	-2.421482	0.01545737	0.22451229
AHCTF1	-1.54466	0.638022	-2.421015	0.015477242	0.22451229
PLPP6	-2.124081	0.87737	-2.420964	0.015479421	0.22451229
PCBP4	-3.105855	1.282923	-2.42092	0.015481264	0.22451229
FOXK2	-2.055671	0.849347	-2.420295	0.01550792	0.22451229
SLX1A	-3.185506	1.316835	-2.419062	0.015560581	0.22451229
MATR3	-2.855896	1.180748	-2.418718	0.015575319	0.22451229
MMP28	-2.909925	1.203131	-2.418626	0.015579233	0.22451229
LMAN2L	-2.013953	0.832783	-2.418341	0.01559147	0.22451229
STXBP5	-2.931519	1.212444	-2.417858	0.015612156	0.22451229
FAM185A	-2.744593	1.135221	-2.417672	0.015620138	0.22451229
SERTAD1	-1.447397	0.598911	-2.416713	0.015661352	0.22479159
ADRB2	-2.751721	1.139468	-2.414919	0.01573873	0.22519122
ZHX1.C8orf76	-3.227096	1.336349	-2.41486	0.015741241	0.22519122
CR1	-1.729495	0.716413	-2.414103	0.015773993	0.22519122
MBLAC2	-2.347232	0.972956	-2.412475	0.01584463	0.22519122
STAM2	-1.959554	0.812484	-2.411807	0.015873676	0.22519122
ENSG00000225342	-1.854898	0.769163	-2.411578	0.015883635	0.22519122
BIK	-3.006029	1.246502	-2.411572	0.015883929	0.22519122
ANKHD1	-1.432713	0.59411	-2.411529	0.015885797	0.22519122
LTBP4	-1.381068	0.572757	-2.411262	0.015897419	0.22519122
MACC1	-3.40061	1.410437	-2.411033	0.015907403	0.22519122

RTP4	-2.126502	0.882303	-2.410173	NA	NA
MSANTD2	-2.852623	1.184756	-2.407773	0.01605016	0.22673282
ANAPC1	-1.623705	0.674424	-2.407544	0.016060241	0.22673282
KIAA0232	-1.607981	0.668195	-2.406454	0.016108214	0.22709941
SERPINF1	-1.723489	0.716769	-2.404523	0.016193576	0.22799142
MOB3C	-2.628118	1.093568	-2.403251	0.01625005	0.22847482
LINC00886	-2.163194	0.900978	-2.400941	0.016352971	0.22950715
DRC3	-2.559104	1.066149	-2.400325	0.016380533	0.22950715
GSE1	-1.63285	0.680583	-2.399193	0.016431245	0.22950715
NKX3.1	-3.656202	1.523961	-2.399143	0.016433474	0.22950715
GNPNAT1	-2.245802	0.936095	-2.399117	0.016434669	0.22950715
PSMG3.AS1	-2.17769	0.90809	-2.3981	0.016480384	0.22983454
ENSG00000273002	-2.435681	1.016138	-2.396998	0.016530014	0.23021558
ZBP1	-2.218969	0.925923	-2.396495	0.01655274	0.2302214
RABGEF1	-2.000525	0.835192	-2.395288	0.016607295	0.23053622
LMF1	-1.802932	0.753027	-2.394247	0.016654549	0.23053622
WASIR2	-2.891518	1.207838	-2.393962	0.016667483	0.23053622
DTWD2	-1.918991	0.801857	-2.393184	0.016702848	0.23053622
CCDC61	-2.915189	1.218201	-2.393029	0.01670994	0.23053622
CCHCR1	-1.538993	0.643136	-2.39295	0.01671354	0.23053622
MINDY3	-1.987659	0.83077	-2.39255	0.016731747	0.23053622
FGD4	-4.884804	2.042666	-2.391386	0.016784884	0.23077756
PELP1	-1.658492	0.693585	-2.391187	0.016793987	0.23077756
EMILIN2	-3.497076	1.463615	-2.389342	0.016878593	0.23163176
PATZ1	-1.647119	0.689994	-2.387148	0.01697965	0.23222317
OGDH	-1.533903	0.642763	-2.386421	0.017013245	0.23222317
FTO	-1.990057	0.833932	-2.386355	0.017016308	0.23222317
METTL6	-1.637201	0.686146	-2.386085	0.017028833	0.23222317
SPIN2B	-1.457851	0.61101	-2.385969	0.0170342	0.23222317
PGAP3	-3.038267	1.274235	-2.384385	0.017107704	0.23230711
MYO1B	-2.911304	1.221236	-2.383899	0.017130285	0.23230711
SMOX	-2.622031	1.100072	-2.383508	0.017148525	0.23230711
ZNF212	-2.134317	0.895997	-2.382058	0.017216196	0.23230711
FAF1	-1.733296	0.727675	-2.381966	0.017220491	0.23230711
KIAA0556	-2.216512	0.930755	-2.381413	0.017246372	0.23230711
HMGCS1	-1.398991	0.587601	-2.380853	0.017272606	0.23230711
STX3	-1.660846	0.697587	-2.380843	0.017273053	0.23230711
DHRS4	-2.077705	0.872734	-2.380685	0.017280489	0.23230711
TNKS	-2.13455	0.896616	-2.380673	0.017281052	0.23230711
MCAT	-1.659715	0.697205	-2.380525	0.017287971	0.23230711
CXorf65	-1.776365	0.746579	-2.379339	0.017343727	0.23261491
BRF1	-2.693167	1.132148	-2.378811	0.017368558	0.23261491
ZNF34	-2.014913	0.8471	-2.378601	0.017378498	0.23261491
ZNF564	-1.866351	0.785267	-2.376709	0.017467885	0.23326115
ULK3	-1.468249	0.617788	-2.376622	0.017471983	0.23326115
CBFA2T3	-1.680113	0.707078	-2.376134	0.017495116	0.23326821
PTGIR	-3.712148	1.563495	-2.374262	0.017584064	0.23415166

TESK2	-1.511072	0.636742	-2.373129	0.017638083	0.23432106
C19orf73	-3.479416	1.466491	-2.372613	0.017662737	0.23432106
BOLA2.SMG1P6	-1.757713	0.740912	-2.372362	0.017674751	0.23432106
DHX34	-1.988305	0.838207	-2.372094	0.017687607	0.23432106
FKBP14	-3.356703	1.417474	-2.368088	0.017880288	0.23641031
DENND6B	1.513204	0.639059	2.367864	0.017891129	0.23641031
LCMT2	-3.051336	1.289078	-2.367068	0.017929634	0.23661615
GTPBP2	-2.084459	0.880977	-2.366077	0.017977681	0.23694722
SEMA7A	-2.154315	0.910846	-2.36518	0.018021289	0.23721901
WDR7	-2.522081	1.066609	-2.36458	0.018050525	0.23730118
ENSG00000218018	-2.224224	0.941711	-2.361898	0.018181667	0.23872112
RPS19	0.17398	0.07369	2.360974	0.018226997	0.23880129
PLAG1	-2.734874	1.158437	-2.360831	0.018234052	0.23880129
TMEM177	-2.723671	1.154131	-2.359931	0.01827832	0.2388704
F11R	-2.632141	1.115417	-2.359783	0.018285622	0.2388704
KCTD9	-1.975329	0.837472	-2.35868	0.018340058	0.23927863
NAGLU	-1.922693	0.815511	-2.357655	0.018390782	0.23963746
AGTPBP1	-2.019493	0.856897	-2.356751	0.018435583	0.23984085
PIGG	-1.491115	0.632793	-2.356403	0.018452872	0.23984085
SLFN5	-2.673421	1.134932	-2.355577	0.018493969	0.23985811
SLC37A4	-2.377225	1.009248	-2.355442	0.018500684	0.23985811
NEK3	-2.492585	1.059064	-2.353573	0.018593972	0.24030449
CARNS1	-3.4914	1.483452	-2.353564	0.018594382	0.24030449
ENSG00000236935	-1.577866	0.670476	-2.353353	0.01860497	0.24030449
PLEKHG2	-2.586105	1.099359	-2.352375	0.018653952	0.24063598
VPS8	-1.410492	0.599863	-2.351357	0.01870507	0.24099415
RIPOR3	-2.104461	0.895447	-2.350178	0.018764431	0.24138612
ENSG00000263264	-1.594208	0.678437	-2.349824	0.018782273	0.24138612
SSBP4	-1.22502	0.521427	-2.349358	0.018805801	0.2413879
FAM229A	-1.976707	0.841769	-2.348277	0.018860482	0.24178903
ZNF865	-1.871947	0.797802	-2.346379	0.0189568	0.24261771
TAS2R4	-2.340071	0.997439	-2.346078	0.018972141	0.24261771
SNX15	-3.184988	1.358031	-2.345298	0.019011867	0.24282483
MTMR1	-1.559702	0.665346	-2.344195	0.019068213	0.24312289
ENSG00000261505	-2.286493	0.9755	-2.343919	0.01908232	0.24312289
ENSG00000253948	-2.035863	0.869188	-2.342258	0.019167443	0.2434254
MX2	-1.951512	0.833233	-2.342097	0.019175753	0.2434254
DAG1	-2.787333	1.190112	-2.342076	0.019176827	0.2434254
BACH1.AS1	-2.283163	0.975386	-2.340778	0.019243598	0.24375585
RAI1	-1.992998	0.851471	-2.340652	0.019250098	0.24375585
TTC39C	-1.551198	0.66294	-2.339878	0.019290034	0.24392747
REPS1	-2.057805	0.879623	-2.339417	0.019313872	0.24392747
PMM2	-2.03289	0.869307	-2.338518	0.0193604	0.24392747
RGCC	-2.508969	1.07307	-2.338123	0.019380877	0.24392747
FAM120B	-1.7426	0.745617	-2.337125	0.019432691	0.24392747
MYEF2	-1.570133	0.671891	-2.336885	0.019445141	0.24392747
SEC16A	-1.937716	0.829269	-2.336657	0.019457041	0.24392747

ZNF436	-3.383804	1.448985	-2.335292	0.019528146	0.24392747
IGKV1.12	-1.928392	0.825831	-2.335093	0.01953857	0.24392747
RFX7	-1.683136	0.720855	-2.334915	0.019547864	0.24392747
PXN.AS1	-1.763744	0.75551	-2.334506	0.01956924	0.24392747
SERGEF	-1.422645	0.609448	-2.334319	0.019579034	0.24392747
LDB1	-1.927114	0.825562	-2.334305	0.01957974	0.24392747
OGT	-1.383662	0.59309	-2.332972	0.019649592	0.24392747
ZNF585A	-1.626782	0.697333	-2.332864	0.019655307	0.24392747
RYBP	-1.747275	0.748988	-2.332849	0.019656072	0.24392747
SNX19	-2.181562	0.935254	-2.332587	0.019669833	0.24392747
CRY2	-1.604478	0.688023	-2.332013	0.019700025	0.24392747
ZNF577	-1.727649	0.740944	-2.331688	0.0197171	0.24392747
WWP1	-1.661864	0.712885	-2.33118	0.019743858	0.24392747
SDCBP2.AS1	-2.023367	0.868072	-2.330874	0.019760016	0.24392747
CD3EAP	-1.692723	0.72662	-2.329584	0.01982813	0.24447587
ENSG00000259321	-1.599098	0.686708	-2.328645	0.019877898	0.24479703
MREG	-3.045476	1.308774	-2.326968	0.019966958	0.24540102
NFYA	-1.621571	0.696931	-2.326731	0.019979584	0.24540102
PPFIBP1	-1.797049	0.772594	-2.325993	0.020018926	0.24540102
NBPF15	-2.075173	0.892295	-2.325657	0.020036844	0.24540102
TATDN2	-1.824585	0.784883	-2.32466	0.020090168	0.24540102
LAMA5	-2.418766	1.040482	-2.324659	0.020090216	0.24540102
VPS26B	-2.794443	1.202118	-2.324599	0.020093398	0.24540102
MOSPD2	-1.820675	0.783412	-2.324033	0.020123703	0.24540464
TANGO2	-1.438519	0.619081	-2.323638	0.020144924	0.24540464
VILL	-1.554429	0.669186	-2.322865	0.020186388	0.24540464
HIST2H2BE	-1.756682	0.756271	-2.32282	0.020188812	0.24540464
TRAPPC11	-1.6031	0.690385	-2.322038	0.020230884	0.24562674
GABARAPL1	-1.844282	0.794926	-2.320069	0.02033717	0.24650471
RAP2A	-2.882886	1.242934	-2.31942	0.020372272	0.24650471
ENSG00000269514	-2.066247	0.890865	-2.319372	0.020374856	0.24650471
ITPK1	-1.682742	0.725741	-2.318653	0.020413834	0.24668709
HABP4	-2.032453	0.876765	-2.318128	0.020442349	0.24674274
ENSG00000261353	-2.935641	1.267114	-2.316793	0.020515008	0.24707046
MORC2	-2.219768	0.958222	-2.316549	0.020528294	0.24707046
ENSG00000273437	-1.752501	0.756765	-2.31578	0.020570258	0.24707046
DPYSL2	-1.793734	0.774655	-2.315525	0.020584207	0.24707046
ANKFY1	-1.507585	0.651103	-2.315434	0.020589205	0.24707046
ENSG00000242861	-1.948453	0.841704	-2.314892	0.020618863	0.24713899
ZCCHC4	-2.33713	1.009631	-2.314837	NA	NA
CCDC126	-1.691411	0.730799	-2.314468	NA	NA
GATD3	-3.076753	1.329537	-2.314154	0.020659287	0.24733624
IFT27	-1.543376	0.667581	-2.311892	0.020783619	0.24853644
TACC1	-1.353301	0.585712	-2.310522	0.020859245	0.2491521
ENSG00000273674	-2.84826	1.233096	-2.309846	NA	NA
DHCR7	-1.961739	0.849698	-2.308749	0.020957516	0.24989258
ADAM15	-3.432467	1.486907	-2.308461	0.02097349	0.24989258

USP44	-2.359461	1.022255	-2.308094	0.020993882	0.24989258
ITM2A	-1.811288	0.78492	-2.307607	0.021020981	0.24992687
ENSG00000262089	-2.445852	1.060576	-2.306154	0.021102018	0.25053696
WIPI1	-1.486204	0.644631	-2.305513	0.021137866	0.25053696
SLC41A1	-1.564846	0.678779	-2.305383	0.021145125	0.25053696
PIEZO1	-1.866395	0.81061	-2.302456	0.021309481	0.25219478
VMAC	-1.476889	0.642053	-2.300261	0.021433427	0.2533711
TAB1	-1.556018	0.67692	-2.298674	0.02152346	0.25412259
MRPS24	-2.458222	1.069595	-2.298273	0.021546247	0.25412259
COG6	-1.613498	0.702219	-2.297715	0.021578	0.25420657
TTC4	-2.716051	1.183061	-2.295783	0.021688301	0.25471042
DNAJC27.AS1	-3.823416	1.66549	-2.29567	0.021694762	0.25471042
FES	-3.226407	1.405572	-2.295441	0.021707844	0.25471042
ARF4.AS1	-3.091603	1.346964	-2.295238	0.021719494	0.25471042
ENSG00000174171	-3.045507	1.327671	-2.293871	0.02179789	0.25523056
ZNF235	-1.860442	0.811144	-2.293603	0.021813309	0.25523056
UVSSA	-1.890854	0.82485	-2.29236	0.021884885	0.25577804
ENSG00000260136	-2.419052	1.05635	-2.29001	0.022020717	0.25703644
PRKAR1B	-2.532087	1.10589	-2.289637	0.022042369	0.25703644
RHOB	-2.690393	1.1758	-2.288138	0.022129504	0.25707543
GADD45A	-1.902209	0.831439	-2.287852	0.022146155	0.25707543
BAG4	-1.398784	0.611508	-2.287434	0.02217052	0.25707543
SLFN13	-2.215068	0.968598	-2.28688	0.022202847	0.25707543
XXYLT1	-2.681348	1.17265	-2.286571	0.022220853	0.25707543
RUVBL1	-1.497562	0.655068	-2.286117	0.022247389	0.25707543
TNFRSF10D	-3.759896	1.644913	-2.285773	0.022267564	0.25707543
MYH3	-2.435476	1.065619	-2.285504	0.022283317	0.25707543
GPM6A	-3.001369	1.313402	-2.285187	0.022301869	0.25707543
PTER	-1.416179	0.619769	-2.28501	0.022312251	0.25707543
BBS10	-2.338296	1.023377	-2.284882	0.022319728	0.25707543
RNFT2	-3.044044	1.333135	-2.283372	0.02240844	0.25780948
ZNF232	-1.373398	0.602152	-2.280816	0.022559364	0.25874063
KLHL8	-1.335569	0.585684	-2.280359	0.022586425	0.25874063
PGAP2	-1.48035	0.649181	-2.280336	0.022587773	0.25874063
NAA35	-1.951339	0.855759	-2.280243	0.022593276	0.25874063
TMSB4X	-0.149662	0.065654	-2.27955	0.022634372	0.25874063
ZC2HC1A	-2.045181	0.89727	-2.279337	0.022647035	0.25874063
PITPNC1	-2.582361	1.13319	-2.278841	0.022676496	0.25874063
PLEKHF1	-1.911404	0.839023	-2.278129	0.022718879	0.25874063
SLC35B1	-1.386001	0.608483	-2.277797	0.022738678	0.25874063
FDXR	-2.359017	1.036151	-2.27671	0.022803542	0.25874063
CANT1	-1.321783	0.58075	-2.275994	0.022846397	0.25874063
MAP3K7CL	-2.423621	1.064902	-2.275911	0.022851367	0.25874063
ENSG00000264112	-2.789982	1.226132	-2.275433	0.022879944	0.25874063
ZBTB21	-2.013707	0.885133	-2.275034	0.022903879	0.25874063
CHAF1A	-1.843005	0.810351	-2.274329	0.022946196	0.25874063
SLC22A18	-3.074344	1.351902	-2.274088	0.022960688	0.25874063

CD99L2	-2.169538	0.954028	-2.274083	0.022960975	0.25874063
OTUD6B	-1.540616	0.677481	-2.274035	0.022963883	0.25874063
IKZF2	-1.960461	0.862252	-2.273651	0.022986982	0.25874063
FBXW8	-2.697832	1.186597	-2.273587	0.022990809	0.25874063
ENSG00000260793	-1.768538	0.778456	-2.271853	0.023095396	0.25952627
IMPA2	-2.467305	1.086155	-2.271596	0.023110915	0.25952627
SGCB	-1.873456	0.825046	-2.27073	0.023163337	0.25983222
CCDC7	-2.608772	1.149205	-2.270067	0.023203514	0.26000029
MINDY1	-2.437091	1.073932	-2.269317	0.023249055	0.26015206
FAM91A1	-1.268636	0.559113	-2.269014	0.023267476	0.26015206
NFYB	-1.437206	0.634066	-2.266651	0.02341157	0.2611291
ENSG00000261098	-1.580047	0.697256	-2.266093	0.023445701	0.2611291
GCLM	-1.580193	0.69737	-2.265933	0.023455477	0.2611291
C16orf70	-1.51678	0.669387	-2.265923	0.023456073	0.2611291
DPPA4	-2.194625	0.969762	-2.263055	0.023632284	0.26259904
ZNF782	-3.477663	1.536924	-2.262742	0.023651618	0.26259904
GLI4	-2.930099	1.295052	-2.262534	0.023664448	0.26259904
APOBEC3F	-1.360346	0.601614	-2.26116	0.023749348	0.26325808
LCN8	-3.552102	1.571057	-2.260964	0.023761484	NA
GIPC1	-2.626404	1.16384	-2.25667	0.02402869	0.26577449
SH3D19	-2.729542	1.209729	-2.256326	0.024050235	0.26577449
AMPD2	-1.862922	0.825681	-2.256224	0.024056626	0.26577449
CNNM3	-2.773281	1.229575	-2.255479	0.024103251	0.26577449
GLRX5	-1.907465	0.845726	-2.255418	0.024107131	0.26577449
PDIK1L	-2.119767	0.940207	-2.254576	0.024159977	0.26577449
GNAQ	-3.978749	1.764753	-2.254564	0.024160682	NA
AGPS	-1.548057	0.686687	-2.254383	0.024172074	0.26577449
TRIM3	-3.113505	1.381628	-2.253505	0.024227348	0.26577449
FAM118B	-1.845692	0.819068	-2.253404	0.024233704	0.26577449
ENSG00000261474	-2.176639	0.966071	-2.253084	0.024253863	0.26577449
TBC1D2B	-1.857222	0.824354	-2.252943	0.024262724	0.26577449
MTX3	-2.722536	1.208628	-2.252584	0.024285402	0.26577449
ENSG00000267519	-1.460825	0.64903	-2.250782	0.024399347	0.26608835
SAMD1	-1.652777	0.734351	-2.250663	0.024406917	0.26608835
TRAFD1	-1.456333	0.647271	-2.249958	0.024451633	0.26608835
DDHD2	-1.403212	0.623789	-2.249498	0.024480852	0.26608835
LRSAM1	-2.028943	0.901984	-2.249423	0.024485594	0.26608835
GLCE	-3.020752	1.342926	-2.249381	0.024488281	0.26608835
MAN2A2	-2.100137	0.933744	-2.249158	0.024502436	0.26608835
ENSG00000282393	-2.540584	1.129713	-2.248877	0.024520351	0.26608835
WNT10A	-1.452935	0.646616	-2.246984	0.024641076	0.2668302
KMT2B	-2.141679	0.953398	-2.246364	0.024680709	0.2668302
PPARGC1B	-2.90431	1.292956	-2.246256	0.024687585	0.2668302
LINC02018	-1.884832	0.839232	-2.245899	0.024710458	0.2668302
ZNF10	-3.198486	1.42422	-2.245782	0.024717991	0.2668302
ENSG00000237440	-1.790168	0.797484	-2.24477	0.024782883	0.26725115
RORA.AS1	-2.536834	1.130461	-2.24407	0.024827894	0.26744039

LMBRD2	-2.391643	1.066193	-2.243162	0.024886342	0.26744039
MLLT3	-2.236234	0.996966	-2.243041	0.024894191	0.26744039
BTBD9	-2.404937	1.072565	-2.24223	0.024946503	0.26744039
USP49	-3.168366	1.413093	-2.242149	0.024951747	0.26744039
ANKRD42	-1.407226	0.627642	-2.242084	0.02495592	0.26744039
SGCE	-2.33882	1.043377	-2.241586	0.024988147	0.26750797
RHOBTB2	-1.795825	0.801622	-2.240238	0.025075454	0.26800757
INVS	-2.185497	0.97564	-2.240064	0.025086755	0.26800757
SLC2A8	-1.578836	0.70553	-2.2378	0.025234086	0.26874212
TRIB2	-2.714738	1.213258	-2.23756	0.025249763	0.26874212
PSKH1	-1.875505	0.838319	-2.237221	0.025271928	0.26874212
UBE2M	-1.719799	0.768752	-2.237132	0.025277719	0.26874212
TCFL5	-1.814223	0.811143	-2.236626	0.025310813	0.26874212
MICA	-1.396274	0.624412	-2.236141	0.025342556	0.26874212
NUP58	-1.387815	0.620717	-2.235823	0.02536334	0.26874212
ZNF300	-3.026006	1.353521	-2.235656	0.025374345	0.26874212
METTL25	-1.800304	0.805464	-2.235115	0.025409774	0.26874212
RFX2	-1.661274	0.743292	-2.235022	0.025415922	0.26874212
ITPR3	-1.673523	0.749278	-2.233515	0.025514984	0.26878092
PRKACA	-1.647618	0.737688	-2.233489	0.025516691	0.26878092
PPP2R3A	-2.879442	1.289274	-2.233382	0.025523736	0.26878092
SLC22A5	-3.006375	1.346109	-2.233382	0.02552377	0.26878092
SFXN4	-2.165588	0.96983	-2.232956	0.025551818	0.268802
FRS3	-3.151601	1.411739	-2.232425	0.025586907	0.26889703
IMPACT	-1.705256	0.764115	-2.231673	0.025636554	0.269099
ENSG00000272871	-2.950537	1.322644	-2.230788	0.025695192	0.269099
FBXO2	-2.957966	1.326182	-2.230438	0.025718358	0.269099
CETP	-2.806043	1.258129	-2.230331	0.025725488	0.269099
GPD2	-2.692988	1.20769	-2.229868	0.025756208	0.269099
ENSG00000187951	-1.936767	0.868615	-2.229718	NA	NA
ZNF382	-1.921578	0.86182	-2.229676	0.025768989	0.269099
LIG3	-1.742918	0.781795	-2.22938	0.025788654	0.269099
VCPKMT	-1.441312	0.646703	-2.228709	0.025833242	0.26910586
FBXO6	-1.693789	0.760285	-2.227834	0.025891596	0.26910586
CEP85	-3.026524	1.358534	-2.227787	0.025894721	0.26910586
TNFSF12	-1.262097	0.566577	-2.22758	0.025908519	0.26910586
NDRG2	-3.317205	1.489264	-2.227413	0.025919692	0.26910586
ZNF792	-2.710692	1.217298	-2.22681	0.025959944	0.26925289
ENSG00000279816	-1.659854	0.745958	-2.225132	0.026072379	0.26975977
SLC38A5	-2.281343	1.025342	-2.224958	0.026084027	0.26975977
ZCCHC2	-1.559392	0.700947	-2.224694	0.026101771	0.26975977
ENSG00000197813	-3.159184	1.420215	-2.22444	0.026118824	0.26975977
TMEM254	-2.092639	0.940878	-2.224133	0.026139512	0.26975977
RPUSD2	-1.312322	0.59029	-2.223182	0.02620354	0.27015038
NCAPD2	-1.753094	0.78889	-2.222227	0.026267965	0.27054431
HCFC2	-3.142239	1.414359	-2.22167	0.026305634	0.27066216
CERNA1	-1.683341	0.758069	-2.220564	0.026380539	0.27095978

SLC39A8	-2.992493	1.348182	-2.21965	0.026442534	0.27095978
PPP2R2D	-1.772723	0.79885	-2.219095	0.02648025	0.27095978
ENSG00000230555	-1.934517	0.871783	-2.219036	0.026484304	0.27095978
SLC35A2	-1.583816	0.713856	-2.218676	0.026508779	0.27095978
NMB	-2.056348	0.927051	-2.21816	0.026543957	0.27095978
DDX51	-2.036351	0.918104	-2.217995	NA	NA
SHMT1	-1.68973	0.76188	-2.217843	0.026565522	0.27095978
HEATR5B	-1.711655	0.771849	-2.217603	0.026581882	0.27095978
ENSG00000232527	-2.614381	1.179131	-2.21721	0.02660876	0.27095978
CLECL1	-1.615784	0.728826	-2.216967	0.026625326	0.27095978
PLEKHO2	-2.231032	1.006435	-2.216767	0.026639011	0.27095978
ENTR1	-1.602126	0.722812	-2.216519	0.026655997	0.27095978
CHD1L	-1.34147	0.60536	-2.215986	0.026692477	0.27095978
UQCRHL	-1.641252	0.740689	-2.215845	0.026702141	0.27095978
ENSG00000269898	-2.468251	1.114636	-2.214401	0.026801178	0.27108887
ANO10	-2.644354	1.194175	-2.214377	0.026802848	0.27108887
CNN3	-1.734974	0.784	-2.212977	0.026899266	0.27108887
ATAD3A	-1.666803	0.753349	-2.212524	0.026930453	0.27108887
DDX60	-2.313419	1.045891	-2.211912	0.026972723	0.27108887
ABCD3	-1.744666	0.788905	-2.211504	0.027000956	0.27108887
MIR4458HG	-1.493175	0.675193	-2.211477	0.027002835	0.27108887
PTPRK	-2.083326	0.942154	-2.211238	0.027019366	0.27108887
VAV3	-1.603309	0.725133	-2.211055	0.027032009	0.27108887
FCGBP	-1.675283	0.757866	-2.210526	0.027068675	0.27108887
TSTD2	-1.461404	0.661135	-2.210449	0.02707403	0.27108887
NKIRAS1	-1.480936	0.670084	-2.210075	0.027099925	0.27108887
OTUD3	-2.153337	0.974372	-2.209974	0.027106938	0.27108887
LRIG2	-1.527846	0.69143	-2.20969	0.027126719	0.27108887
KIAA0753	-3.158811	1.429688	-2.209441	0.027143987	0.27108887
ZNF584	-2.089215	0.945605	-2.209395	0.027147171	0.27108887
GNA11	-2.696166	1.220432	-2.20919	0.027161424	0.27108887
PNPO	-2.399969	1.087179	-2.207519	0.027277824	0.27166948
COPG2	-2.787044	1.262936	-2.206798	0.027328205	0.27166948
ATP11A	-1.777783	0.805651	-2.206642	0.027339063	0.27166948
RTKN	-3.736852	1.693638	-2.206405	0.027355624	0.27166948
ENSG00000268220	-3.238035	1.467754	-2.206116	0.027375882	0.27166948
MTMR10	-2.373933	1.076081	-2.206092	0.027377545	0.27166948
ATP13A3	-1.670063	0.757615	-2.204368	0.02749848	0.27224364
C20orf96	-2.065415	0.937048	-2.204171	0.027512307	0.27224364
ICMT	-2.243743	1.01815	-2.203744	0.02754234	0.27224364
ABHD6	-2.257152	1.024273	-2.203663	0.02754806	0.27224364
ERLIN1	-2.244403	1.018614	-2.20339	0.027567307	0.27224364
LARGE2	-1.322934	0.600856	-2.201747	0.027683171	0.2731265
INPPL1	-1.534411	0.697397	-2.200198	0.027792881	0.27376647
BPGM	-1.451905	0.660038	-2.199731	0.027825989	0.27376647
THNSL1	-2.632481	1.196764	-2.199665	0.027830642	0.27376647
ZNF436.AS1	-2.73624	1.244298	-2.199024	0.027876241	0.27376647

AIM2	-3.297573	1.499642	-2.198907	0.027884559	0.27376647
CSTF2	-2.520481	1.146433	-2.198542	0.027910509	0.27376647
RTCA	-1.260262	0.573311	-2.198216	0.02793373	0.27376647
ZKSCAN5	-2.757501	1.254686	-2.197762	0.027966046	0.27382315
NOXA1	-3.609831	1.643451	-2.196495	0.028056562	NA
AIFM1	-1.625103	0.739869	-2.196473	0.028058118	0.27446425
PLEKHA8	-2.362213	1.075811	-2.195752	0.028109734	0.27470876
C8orf88	-2.047783	0.932927	-2.19501	0.028162895	0.27479716
SCLY	-1.997053	0.909868	-2.194882	0.028172035	0.27479716
C12orf60	-2.55177	1.162963	-2.194197	0.02822127	0.27501747
ENSG00000232412	-3.281052	1.496577	-2.19237	0.028352778	NA
TBC1D7	-2.315807	1.056327	-2.192319	0.028356466	0.27607427
ITGA3	-3.081626	1.406291	-2.191314	0.028429094	0.2765205
TMEM50B	-1.212514	0.553591	-2.19027	0.028504653	0.27682307
ZSCAN22	-2.789589	1.273702	-2.190143	0.028513849	0.27682307
PILRA	-3.714045	1.69614	-2.189704	0.028545728	NA
MYLK.AS1	-1.747186	0.798054	-2.189307	0.028574527	0.27696459
TTC39B	-3.16316	1.445141	-2.188824	0.0286096	0.27696459
FAM161B	-3.043502	1.390766	-2.188364	0.028643075	0.27696459
CARD6	-3.028846	1.384182	-2.188185	0.02865611	0.27696459
DUS2	1.309778	0.598596	2.188085	0.028663385	0.27696459
IMPDH1	-1.519631	0.694616	-2.187728	0.028689452	0.27696459
SCML4	-2.479729	1.134559	-2.185633	0.028842447	0.27790611
ZNF252P.AS1	-3.5555	1.627079	-2.185204	0.028873923	0.27790611
NHLRC4	-2.571025	1.17659	-2.18515	0.028877834	0.27790611
HIST4H4	-2.216553	1.014587	-2.184684	0.028912004	0.27790611
CENPP	-3.109799	1.423622	-2.184428	0.028930824	0.27790611
GYS1	-1.53434	0.702567	-2.183907	0.028969066	0.27790611
TTC34	-3.206266	1.46846	-2.18342	0.029004891	0.27790611
CLSTN3	-2.320198	1.062719	-2.183265	0.029016319	0.27790611
SESN1	-1.386268	0.635019	-2.183034	0.029033273	0.27790611
ENSG00000271254	-1.915599	0.877652	-2.182639	0.029062387	0.27790611
MORN3	-3.564706	1.633972	-2.181619	0.029137635	0.27790611
ACOX1	-1.642076	0.752781	-2.181347	0.029157772	0.27790611
POT1	-1.600753	0.73384	-2.181338	0.029158404	0.27790611
SPRYD3	-1.646556	0.754979	-2.180929	0.029188694	0.27790611
NUDCD1	-1.761963	0.807907	-2.180899	0.029190913	0.27790611
ENSG00000260495	-3.349439	1.536124	-2.180448	0.029224239	NA
INTS14	-1.530894	0.702475	-2.179286	0.029310434	0.2787868
CXCL8	-3.615112	1.659318	-2.178673	0.029355938	0.27896251
ZNF570	-1.516839	0.696609	-2.177462	0.029446111	0.27921175
SETD1A	-1.500032	0.688926	-2.177349	0.029454527	0.27921175
SAMD10	-2.897713	1.33102	-2.177062	0.029475959	0.27921175
C9orf64	-2.456256	1.128366	-2.176825	0.029493659	0.27921175
ENSG00000248559	-1.719844	0.790207	-2.176448	0.02952179	0.27921175
DDX11	-1.463981	0.672805	-2.175937	0.029559988	0.27921175
ZNF398	-2.081741	0.956781	-2.175777	0.029571941	0.27921175

BIRC5	-3.193004	1.468032	-2.175023	0.029628381	0.27921175
ENSG00000231113	-2.296933	1.056218	-2.174676	0.029654412	0.27921175
SH3BP5.AS1	-1.772796	0.81537	-2.174224	0.029688288	0.27921175
RSPRY1	-1.425553	0.655753	-2.173917	0.029711345	0.27921175
CFAP44	-2.15937	0.993327	-2.173875	0.029714499	0.27921175
RCBTB1	-1.653871	0.761259	-2.172547	0.029814426	0.27921175
UCKL1	-1.198348	0.551628	-2.172385	0.029826604	0.27921175
MEI1	-1.407657	0.648811	-2.169596	0.030037462	0.27921175
ETAA1	-1.604742	0.739708	-2.169428	0.030050234	0.27921175
MIR222HG	-3.095518	1.42689	-2.169417	0.030051074	0.27921175
DSE	-1.71272	0.789616	-2.169054	0.030078621	0.27921175
RANGAP1	-1.562574	0.720462	-2.16885	0.030094064	0.27921175
ERLIN2	-1.689753	0.779769	-2.166991	0.030235512	0.27921175
LINC01684	-2.258449	1.042251	-2.166895	0.030242859	0.27921175
B4GAT1	-3.183429	1.469132	-2.166877	0.03024421	0.27921175
FKBP5	-1.501382	0.692961	-2.166618	0.030263964	0.27921175
CSE1L	-1.402091	0.647223	-2.166318	0.030286926	0.27921175
FANCM	-1.520517	0.701939	-2.166166	0.030298482	0.27921175
CCDC71L	-1.767435	0.81629	-2.165204	0.030372034	0.27921175
PDLIM7	-1.544802	0.713484	-2.165155	0.030375852	0.27921175
CEP192	-1.603365	0.740669	-2.164753	0.030406569	0.27921175
SLC9A8	-2.017821	0.932338	-2.164259	0.030444461	0.27921175
SLX4	-2.980691	1.377554	-2.163757	0.030483033	0.27921175
MEGF8	-2.169296	1.002565	-2.163746	0.030483883	0.27921175
ZNF763	-2.983476	1.379178	-2.163228	0.030523639	0.27921175
TMEM94	-1.765097	0.816046	-2.162989	0.030542063	0.27921175
HYAL2	-2.600008	1.20216	-2.162781	0.030557995	0.27921175
LPAR2	-2.317102	1.071738	-2.162005	0.030617797	0.27921175
IGHV3.7	-4.180724	1.933865	-2.161849	0.030629832	NA
PLEKHB1	-2.325346	1.075782	-2.161541	0.030653599	0.27921175
IL9R	-2.276614	1.053355	-2.161298	0.030672352	0.27921175
GALNT3	-1.868066	0.864488	-2.160893	0.030703605	0.27921175
PLD2	-2.528779	1.170414	-2.160585	0.03072741	0.27921175
CDC42BPB	-1.942635	0.899138	-2.160552	0.030729984	0.27921175
ASXL1	-1.274605	0.59002	-2.160273	0.030751565	0.27921175
ENSG00000237181	-2.512623	1.163635	-2.159289	0.030827745	0.27921175
RELL2	-1.782038	0.825492	-2.158758	0.030868953	0.27921175
SART3	-1.460847	0.676732	-2.15868	0.030875029	0.27921175
ENSG00000228801	-1.970536	0.91288	-2.158592	0.030881834	0.27921175
L2HGDH	-2.647986	1.22677	-2.158503	0.030888739	0.27921175
AASS	-2.657255	1.232089	-2.156706	0.03102856	0.27921175
L1TD1	-2.130898	0.988085	-2.156593	0.03103741	0.27921175
ENSG00000276148	-2.670167	1.2382	-2.156491	0.031045363	0.27921175
SKP2	-1.786401	0.82839	-2.156475	0.031046622	0.27921175
STYX	-1.45886	0.676641	-2.156033	0.031081055	0.27921175
ENSG00000247134	-3.508108	1.627256	-2.155843	0.031095948	0.27921175
ACAP3	-2.238224	1.038843	-2.154535	0.031198219	0.27921175

HS6ST1	-2.727869	1.266451	-2.153947	0.031244304	0.27921175
MRPS6	1.14753	0.532899	2.153371	0.031289507	0.27921175
DFFB	-2.755885	1.279904	-2.153196	0.031303248	0.27921175
RSPH3	-1.459062	0.677646	-2.153132	0.031308287	0.27921175
PEMT	-1.990224	0.924379	-2.153039	0.031315592	0.27921175
GPR89B	-1.501704	0.69758	-2.152732	0.031339726	0.27921175
FBXO4	-1.393897	0.647597	-2.152415	0.031364706	0.27921175
GNAZ	-2.318842	1.077551	-2.151954	0.031400943	0.27921175
NEURL1	-5.549515	2.578839	-2.151944	0.031401798	0.27921175
ACACA	-2.408222	1.119119	-2.15189	0.031406009	0.27921175
ZNF197	-2.522463	1.172246	-2.151821	0.031411486	0.27921175
PIK3R4	-2.207732	1.026018	-2.151747	0.031417284	0.27921175
ANKRD52	-1.705687	0.792737	-2.151643	0.031425493	0.27921175
SUV39H1	-2.13228	0.991008	-2.151627	0.031426779	0.27921175
FASN	-3.631958	1.688033	-2.151592	0.03142953	NA
NLK	-1.69867	0.789783	-2.150805	0.031491576	0.27921175
ZNF616	-2.370116	1.101988	-2.150763	0.03149491	0.27921175
MAP11	-2.141329	0.99588	-2.150187	0.03154045	0.27921175
CASTOR3	-1.991885	0.926413	-2.150106	0.031546869	0.27921175
CARMIL1	-2.065739	0.960894	-2.14981	0.031570266	0.27921175
TCTEX1D2	-1.458871	0.678612	-2.149786	0.03157213	0.27921175
PRADC1	-1.6593	0.771977	-2.149416	0.031601441	0.27921175
TBC1D10B	-1.515375	0.705037	-2.149356	0.031606198	0.27921175
IGFBP4	-2.027947	0.943514	-2.149355	0.031606292	0.27921175
ZNF35	-2.427183	1.129425	-2.149043	0.031631021	0.27921175
ZNF519	-1.621937	0.754757	-2.148953	0.031638091	0.27921175
TFRC	-1.763132	0.820635	-2.148497	0.031674315	0.27921175
PHRF1	-1.437132	0.669091	-2.147886	0.031722805	0.27921175
MAGED1	-2.145539	0.999158	-2.147346	0.031765712	0.27921175
ALG1L2	-3.196517	1.488626	-2.147293	0.031769925	0.27921175
LINC00526	-1.817176	0.84634	-2.1471	0.031785301	0.27921175
TENT4A	-2.313154	1.077527	-2.146725	0.0318152	0.27921175
CHAC2	-3.125282	1.455941	-2.146571	0.031827449	0.27921175
CPSF1	-1.779888	0.829394	-2.14601	0.031872154	0.27921175
COLEC12	-1.808663	0.843007	-2.145488	0.031913814	0.27921175
ENSG00000229999	-1.36261	0.635264	-2.144951	0.031956733	0.27921175
AGAP6	-3.179378	1.482525	-2.144569	NA	NA
AP4B1	-2.130763	0.993607	-2.144472	0.031995089	0.27921175
SNAI3	-1.796435	0.837807	-2.144212	0.032015927	0.27921175
NBPF10	-1.458569	0.68028	-2.14407	0.032027252	0.27921175
TLR7	-1.980624	0.92381	-2.143973	0.032035027	0.27921175
DNAH14	-2.242476	1.046302	-2.143239	0.032093894	0.27921175
ENSG00000271806	-3.133165	1.462076	-2.142957	0.032116586	0.27921175
FICD	-2.244831	1.047674	-2.14268	0.03213877	0.27921175
ZNF251	-3.463982	1.616786	-2.142512	0.032152307	0.27921175
ENSG00000247363	-2.33024	1.087701	-2.142354	0.032165011	0.27921175
PPAT	-2.030711	0.948318	-2.141382	0.032243231	0.27921175

ASCC1	-1.337097	0.624423	-2.141332	0.032247298	0.27921175
CASP2	-1.2907	0.602884	-2.140877	0.032283949	0.27921175
PGBD4	-1.667926	0.779108	-2.140816	0.032288919	0.27921175
HSPA14	-1.535337	0.717187	-2.140775	0.032292157	0.27921175
KIAA0319L	-1.298759	0.606694	-2.140714	0.032297108	0.27921175
ZC3H18	-1.33132	0.622003	-2.140374	0.032324548	0.27921175
ENSG00000237188	-2.005334	0.937016	-2.140128	0.032344406	0.27921175
RBBP9	-2.026884	0.9471	-2.140095	0.032347083	0.27921175
ENSG00000266385	-3.104152	1.450498	-2.140059	0.032349982	0.27921175
ZNF473	-1.846959	0.863186	-2.139699	0.032379133	0.27921175
ZBTB40	-1.396699	0.6528	-2.13955	0.032391138	0.27921175
TIGD7	-2.217097	1.036522	-2.138977	0.032437501	0.27921175
MAP2K4	-1.48134	0.692588	-2.138848	0.032447941	0.27921175
INKA2	-2.595036	1.213644	-2.138218	0.032499016	0.27921175
ZBED6CL	-3.181218	1.488166	-2.137677	0.032542959	0.27921175
RBM45	-1.499903	0.701666	-2.137633	0.03254657	0.27921175
CTNNBIP1	-1.852292	0.866727	-2.137111	0.032588986	0.27921175
LSM11	-3.144949	1.471692	-2.136961	0.032601169	0.27921175
TRIM39	-1.386368	0.648765	-2.136934	0.032603379	0.27921175
CD5	-2.234099	1.04549	-2.136891	0.03260683	0.27921175
PARP3	-1.617862	0.75723	-2.136553	0.032634394	0.27921175
INTS7	-1.981348	0.927736	-2.135681	0.032705414	0.27921175
WDCP	-2.345792	1.098428	-2.13559	0.032712844	0.27921175
ENSG00000274605	-2.549495	1.194134	-2.135017	0.032759661	0.27921175
PAXIP1	-2.502523	1.172171	-2.134948	0.032765279	0.27921175
PSTPIP1	-1.584527	0.742283	-2.134668	0.032788144	0.27921175
RALGPS1	-1.53689	0.719977	-2.134638	0.032790629	0.27921175
LINC02352	-1.945725	0.911632	-2.134331	0.032815674	0.27921175
ZNF682	-1.886575	0.883932	-2.1343	0.032818203	0.27921175
RAB40B	-2.668205	1.250711	-2.133349	0.032896073	0.27961792
ENSG00000267169	-2.919149	1.368529	-2.133056	0.032920133	0.27961792
ENSG00000224738	-3.122231	1.464259	-2.132294	0.032982687	0.27988973
IL2RA	-1.939577	0.909789	-2.131898	0.033015209	0.27988973
PTTG1	-3.035039	1.423781	-2.131676	0.033033497	0.27988973
CDC14A	-2.161552	1.014192	-2.131305	0.033064029	0.2799186
LINC01597	-2.292319	1.075959	-2.13049	0.033131222	0.28025755
GDPD1	-3.020591	1.418211	-2.12986	0.033183133	0.28046677
TSPAN10	-2.297506	1.079047	-2.129198	0.033237857	0.28050697
PKIA	-3.508995	1.648077	-2.129145	0.03324225	0.28050697
OSBPL10	-1.300324	0.610925	-2.12845	0.033299781	0.2805482
DGCR8	-1.699695	0.798568	-2.128429	0.033301507	0.2805482
RUSC1	-1.350458	0.63472	-2.127645	0.033366487	0.28060428
IGHV1.18	-3.566494	1.676323	-2.12757	0.033372777	NA
MYD88	-1.276718	0.600146	-2.127345	0.033391451	0.28060428
AZIN1.AS1	-2.937454	1.38097	-2.127095	0.033412153	0.28060428
GOLGA5	-1.266802	0.595789	-2.12626	0.033481631	0.28060428
KCNN4	-1.599882	0.752543	-2.125968	0.033505925	0.28060428

LINC01569	-3.577341	1.682984	-2.125594	0.033537065	0.28060428
NCOR2	-1.663002	0.782416	-2.125471	0.033547306	0.28060428
ZNF852	-2.647458	1.24561	-2.125431	0.033550668	0.28060428
ZNF155	-1.748988	0.822907	-2.125378	0.033555067	0.28060428
POLA2	-2.586442	1.217381	-2.124596	0.033620355	0.28060428
PDK1	-1.3016	0.612709	-2.124337	0.033641956	0.28060428
RAD51AP1	-1.772201	0.834309	-2.124153	0.033657384	0.28060428
ABCB9	-4.168407	1.962396	-2.124142	0.033658298	NA
MAP1LC3B2	-1.910672	0.89952	-2.124102	0.033661637	0.28060428
KIF13B	-2.870324	1.352385	-2.122417	0.033802742	0.28097348
XRCC4	-1.434474	0.676059	-2.121818	0.033853041	0.28097348
SLC29A2	-2.48831	1.172746	-2.12178	0.033856195	0.28097348
DUSP14	-1.699136	0.800817	-2.121754	0.033858405	0.28097348
XG	-3.358979	1.583175	-2.121673	0.033865247	0.28097348
SIK2	-2.362804	1.113707	-2.121567	0.033874129	0.28097348
MGAT3	-2.828482	1.333554	-2.121011	0.033920877	0.28097348
TMEM64	-2.125865	1.002535	-2.120489	0.033964801	0.28097348
ZNF93	-2.507341	1.182481	-2.120407	0.033971746	0.28097348
GK5	-1.911987	0.90174	-2.12033	0.033978188	0.28097348
CLSTN1	-1.325172	0.625101	-2.119934	0.034011581	0.28102443
ENSG00000269399	-1.574415	0.742815	-2.119524	0.03404619	0.28108534
GSK3B	-1.320605	0.623189	-2.119108	0.034081301	0.2811503
KPTN	-2.634794	1.243592	-2.118696	0.034116178	0.28121322
NSMF	-3.009196	1.421821	-2.116438	0.03430756	0.28245649
ENSG00000251661	-2.45966	1.162677	-2.115514	0.034386201	0.28245649
STARD5	-2.053993	0.970951	-2.115445	0.034392062	0.28245649
MEAK7	-2.297898	1.086442	-2.115067	0.034424276	0.28245649
VSIR	-1.312943	0.62081	-2.114887	0.034439612	0.28245649
SUV39H2	-3.005332	1.421305	-2.114488	0.034473643	0.28245649
KSR1	-2.288819	1.082481	-2.11442	0.034479439	0.28245649
NAA80	-1.320795	0.624684	-2.114343	0.034485967	0.28245649
RBM25	-1.121416	0.530859	-2.112454	0.034647539	0.2835548
USP30	-3.891089	1.84223	-2.112163	0.034672513	NA
ZMYM6	-1.279166	0.605828	-2.111432	0.034735162	0.28384215
RMDN1	-1.404955	0.665423	-2.111372	0.034740311	0.28384215
ENSG00000257151	-2.345973	1.111322	-2.110976	0.034774364	0.28384215
ZNF827	-1.414065	0.669931	-2.110763	0.034792667	0.28384215
SLC9B2	-2.017344	0.955976	-2.110245	0.034837229	0.2839812
HERC5	-1.929931	0.915183	-2.108792	0.034962511	0.28416534
SLC2A13	-1.404182	0.665885	-2.108745	0.03496658	0.28416534
TRAK2	-1.784025	0.846124	-2.108467	0.034990649	0.28416534
ZNF440	-2.600759	1.233566	-2.108325	0.035002886	0.28416534
ENSG00000269886	-2.962546	1.405931	-2.107177	0.035102255	0.28416534
TIMM23B	-1.853796	0.879766	-2.107146	0.035104884	0.28416534
ARHGAP18	-3.206623	1.521844	-2.107065	0.035111988	0.28416534
KCTD2	-2.722274	1.292017	-2.106996	0.035117968	0.28416534
ARHGAP22	-3.282888	1.55836	-2.10663	0.035149624	0.28416534

GALNT6	-2.671023	1.267983	-2.106513	0.035159795	0.28416534
KIAA1841	-2.97514	1.412431	-2.106396	0.035169945	0.28416534
INPP5E	-2.252208	1.06963	-2.105596	0.035239504	0.28416534
DZIP3	-1.460429	0.693777	-2.105042	0.035287683	0.28416534
ENSG00000237773	-3.019101	1.434225	-2.10504	0.035287857	0.28416534
DBF4B	-2.033716	0.966183	-2.104897	0.035300307	0.28416534
COG3	-1.283755	0.609902	-2.104855	0.035303957	0.28416534
KBTBD8	-2.883484	1.370186	-2.104447	0.03533951	0.28416534
PECR	-1.6611	0.789446	-2.104135	0.035366703	0.28416534
CASZ1	-2.315979	1.100805	-2.103896	0.035387548	0.28416534
LRP2BP	-2.475845	1.176938	-2.103632	0.035410525	0.28416534
ZNF879	-1.565388	0.744934	-2.101378	0.035607783	0.28552628
METTL1	-2.758884	1.313104	-2.10104	0.035637437	0.28554219
CDC42EP3	-1.405964	0.669338	-2.100529	0.035682376	0.28568047
ENSG00000273080	-3.354857	1.597591	-2.099947	0.035733489	NA
GEMIN4	-2.193471	1.044655	-2.099709	0.035754421	0.28603537
CYB5RL	-3.219008	1.533693	-2.098861	0.035829152	0.28637307
LIPT1	-1.314786	0.626506	-2.0986	0.035852133	0.28637307
ENSG00000231025	-2.388297	1.138459	-2.097834	0.035919851	0.28658821
MANEA	-2.722434	1.297961	-2.09747	0.035951975	0.28658821
GSDME	-1.932204	0.921447	-2.096923	0.036000408	0.28658821
PANX1	-3.825042	1.825147	-2.095745	0.036104853	NA
ZNF749	-3.417112	1.630683	-2.09551	0.036125714	0.28658821
ARMCX2	-1.673141	0.798495	-2.095369	0.036138236	0.28658821
TAF5	-2.063949	0.985028	-2.09532	0.036142525	0.28658821
MMUT	-2.381482	1.136685	-2.095112	0.036161054	0.28658821
ENSG00000271789	-2.202368	1.051368	-2.094764	0.036192018	0.28658821
ZNF599	-2.642397	1.261463	-2.094707	0.036197016	0.28658821
LAMB1	-2.115379	1.009935	-2.094569	0.036209354	0.28658821
PLTP	-2.906522	1.388571	-2.093175	0.036333576	0.28658821
ADORA2A.AS1	-1.593533	0.761328	-2.093097	0.036340498	0.28658821
RRP1B	-1.410395	0.673869	-2.092981	0.03635086	0.28658821
CALHM2	-1.771256	0.84637	-2.092767	0.03636995	0.28658821
FAM200A	-1.644075	0.785826	-2.092162	0.036424069	0.28658821
PCNA	-1.492774	0.71353	-2.092095	0.036429984	0.28658821
YEATS2	-2.382427	1.138842	-2.091973	0.036440924	0.28658821
ENSG00000270562	-1.865959	0.892489	-2.090737	NA	NA
SYCE1L	-1.601403	0.765982	-2.090653	0.036559185	0.28658821
AP1AR	-1.480424	0.708118	-2.090647	0.036559737	0.28658821
ARMCX4	-2.530733	1.210539	-2.090584	0.0365654	0.28658821
GBF1	-2.142781	1.025191	-2.090128	0.03660627	0.28658821
GRAP2	-1.813254	0.867628	-2.089898	NA	NA
GMIP	-1.111247	0.53184	-2.08944	0.036668158	0.28658821
LINC02656	-2.82312	1.35131	-2.089173	0.036692158	0.28658821
WDR66	-2.302275	1.102075	-2.089037	0.036704437	0.28658821
APTR	-2.355959	1.127847	-2.088899	0.036716802	0.28658821
ACAD9	-1.85274	0.886951	-2.088886	0.036717994	0.28658821

AP5B1	-2.179607	1.043522	-2.088702	0.03673454	0.28658821
PACS2	-2.825343	1.35295	-2.088283	0.036772283	0.28658821
MIB1	-1.530265	0.732794	-2.08826	0.036774369	0.28658821
ACSM1	-2.961548	1.418254	-2.088164	0.036783	0.28658821
VAV3.AS1	-2.410185	1.154593	-2.087476	0.036845171	0.28658821
CCNG2	-1.297397	0.621529	-2.08743	0.036849309	0.28658821
LPCAT4	-1.401284	0.67133	-2.087325	0.036858773	0.28658821
SEC31B	-2.028511	0.971889	-2.087184	0.036871537	0.28658821
ALG9	-2.079611	0.996426	-2.087071	0.036881736	0.28658821
AUTS2	-2.258063	1.082053	-2.086833	0.036903243	0.28658821
SURF2	-1.274405	0.610711	-2.086756	0.036910224	0.28658821
ENSG00000205664	-2.025232	0.970641	-2.086489	0.036934333	0.28658821
ITGAV	-3.90844	1.873528	-2.086139	0.036966058	NA
HIST1H2BK	-2.176652	1.043516	-2.085883	0.036989234	0.28679857
ENSG00000275764	-1.887468	0.905229	-2.085072	0.037062771	0.287153
ENSG00000271380	-3.104498	1.489654	-2.08404	0.037156548	0.2876636
HECTD4	-1.432117	0.687642	-2.082648	0.037283339	0.28807502
RP2	-1.477783	0.709578	-2.082621	0.037285754	0.28807502
ENSG00000273156	-1.575845	0.756695	-2.082537	0.037293433	0.28807502
TMEM184B	-2.131184	1.023545	-2.08216	0.037327883	0.28812547
EVA1B	-2.490347	1.196827	-2.08079	0.037453097	0.2888759
NSDHL	-1.633383	0.78523	-2.080133	0.03751332	0.28891121
ANKRD36	-1.286668	0.618704	-2.079618	0.037560543	0.28891121
ENSG00000249141	-3.743599	1.800146	-2.079608	0.037561499	0.28891121
SNAPC1	-1.304518	0.627317	-2.079519	0.037569656	0.28891121
SHROOM1	-3.701237	1.780385	-2.078897	0.037626779	NA
TEDC1	-2.702136	1.300073	-2.07845	0.037667942	0.28945135
SLC25A16	-2.166005	1.042499	-2.077704	0.037736611	0.28976326
SECISBP2L	-1.097146	0.528286	-2.076803	0.037819789	0.28991802
ENSG00000273759	-3.259214	1.569743	-2.076272	0.037868758	0.28991802
ARL5B	-1.533169	0.738543	-2.075937	0.03789974	0.28991802
CHD3	-1.334779	0.643031	-2.075761	0.037916017	0.28991802
SOCS7	-2.219978	1.06954	-2.075637	0.037927557	0.28991802
ITGA6.AS1	-1.606196	0.773944	-2.075338	0.037955225	0.28991802
ZNF844	-1.35501	0.653044	-2.074913	0.037994618	0.28991802
ENSG00000262049	-1.275486	0.614762	-2.074765	0.038008361	0.28991802
ZNF717	-1.563199	0.753704	-2.074022	NA	NA
STK33	-2.013881	0.971014	-2.073997	0.038079554	0.28991802
ENSG00000272764	-2.086219	1.005945	-2.07389	0.038089551	0.28991802
NRSN2.AS1	-2.630958	1.268725	-2.073703	0.038106919	0.28991802
RPL18A	0.154175	0.074369	2.073098	0.038163134	0.28991802
STK36	-2.819617	1.360239	-2.072884	0.038183093	0.28991802
TRAPPC12.AS1	-2.039723	0.984265	-2.072332	0.038234493	0.28991802
CRAT	-1.868533	0.901669	-2.072304	0.038237071	0.28991802
MFSD3	-2.01901	0.974346	-2.072171	0.03824954	0.28991802
TBC1D9B	-1.250736	0.603816	-2.071384	0.038322927	0.28991802
FAM76A	-1.211797	0.585026	-2.071355	0.038325627	0.28991802

POGLUT3	-3.524144	1.701469	-2.071236	0.038336708	NA
LAT	-2.530352	1.221948	-2.070753	0.038381918	0.28991802
VSTM4	-2.489895	1.202676	-2.070295	0.038424719	0.28991802
ZNF787	-1.747471	0.844308	-2.069709	0.038479568	0.28991802
DIABLO	-2.490364	1.203323	-2.069573	0.038492364	0.28991802
VPS54	-2.313261	1.117842	-2.069398	0.038508747	0.28991802
CUL7	-2.975183	1.437916	-2.069093	0.038537314	0.28991802
ENSG00000266844	-3.390136	1.638581	-2.068946	0.038551153	NA
LINC00893	-1.994994	0.964426	-2.068581	0.038585416	0.28991802
IFI44	-2.979831	1.440596	-2.068471	0.038595771	0.28991802
C3orf18	-3.782926	1.828906	-2.068409	0.038601537	NA
RBL1	-1.606741	0.776916	-2.068102	0.038630443	0.28991802
HMGN4	-1.633237	0.789855	-2.067769	0.038661772	0.28991802
CPPED1	-1.841091	0.890432	-2.067638	0.038674046	0.28991802
ENSG00000167912	-6.197237	2.997612	-2.067391	NA	NA
URB1	-2.561106	1.239054	-2.066986	0.038735499	0.28991802
KEAP1	-1.947081	0.942389	-2.066113	0.038817838	0.28991802
PIP5K1C	-1.589616	0.769686	-2.06528	0.038896556	0.28991802
ARSG	-2.419456	1.171754	-2.064816	0.038940451	0.28991802
RBM27	-1.285049	0.622467	-2.064443	0.038975751	0.28991802
NEXN	-3.747824	1.8157	-2.06412	0.039006299	0.28991802
ITPKB.AS1	-1.885655	0.913568	-2.064055	0.039012536	0.28991802
WRN	-1.98827	0.963354	-2.063904	0.039026863	0.28991802
ENSG00000261187	-5.12956	2.485391	-2.063884	0.039028683	0.28991802
TSHZ1	-3.307164	1.602485	-2.063772	0.039039311	NA
SYCP3	-1.529138	0.741001	-2.06361	0.039054668	0.28991802
ENSG00000249592	-1.943846	0.941988	-2.063556	0.03905987	0.28991802
MS4A1	-0.436323	0.211475	-2.063235	0.039090312	0.28991802
ZNF485	-2.198328	1.065481	-2.063226	0.039091201	0.28991802
LDAH	-1.490725	0.722582	-2.063051	0.039107749	0.28991802
RPS6KB1	-1.194016	0.578864	-2.062687	0.03914235	0.28991802
XPO4	-1.271956	0.616756	-2.062332	0.039176146	0.28991802
ACSL4	-1.612931	0.782264	-2.061876	0.039219584	0.28991802
ZNF700	-1.806351	0.876299	-2.061342	0.039270427	0.28991802
ENSG00000275441	-1.713604	0.831477	-2.060917	0.039310946	0.28991802
SLC28A2	-3.280279	1.59197	-2.060516	0.039349255	0.28991802
MIOS	-1.208637	0.586709	-2.060028	0.039395881	0.28991802
ZNF117	-2.635685	1.279472	-2.059979	0.039400533	0.28991802
BBS4	-1.345742	0.65333	-2.05982	0.039415772	0.28991802
CASK	-2.718444	1.319814	-2.059717	0.039425558	0.28991802
PFAS	-2.450695	1.189893	-2.059593	0.039437454	0.28991802
ZNF202	-1.938747	0.9415	-2.05921	0.039474082	0.28991802
HIST1H2AE	-2.949254	1.432537	-2.058763	0.039516962	0.28991802
TMEM220.AS1	-2.704424	1.313857	-2.058384	0.039553244	0.28991802
LNX1	-2.273956	1.104734	-2.058374	0.039554259	0.28991802
CMTR1	-1.371876	0.666726	-2.057629	0.03962572	0.28991802
CLEC16A	-1.853821	0.901132	-2.057213	0.039665729	0.28991802

ENSG00000258376	-3.259684	1.584595	-2.057109	0.039675767	0.28991802
BRCA2	-1.448533	0.704238	-2.056881	0.039697683	0.28991802
PRR22	-2.878235	1.399503	-2.056612	0.039723533	0.28991802
FAM110A	-1.247702	0.606757	-2.056344	0.039749374	0.28991802
LMNB2	-3.205213	1.558718	-2.056314	0.039752266	0.28991802
CPT2	-1.340737	0.652228	-2.055625	0.039818664	0.28991802
XPNPEP1	-1.347479	0.655556	-2.055474	0.039833268	0.28991802
NBEAL2	-2.50071	1.216687	-2.055344	0.039845827	0.28991802
FBXO33	-1.230538	0.598806	-2.054987	0.039880254	0.28991802
NAGA	-2.673746	1.301456	-2.054427	0.039934418	0.28991802
ERCC4	-1.455863	0.708739	-2.054161	0.039960106	0.28991802
ZNF816	-1.42362	0.693099	-2.053992	0.039976476	0.28991802
ENSG00000259520	-5.200321	2.532083	-2.053772	NA	NA
ADHFE1	-2.621527	1.276527	-2.053639	0.040010635	0.28991802
C8orf58	-1.681655	0.81887	-2.053627	0.040011761	0.28991802
PDP1	-1.586428	0.7726	-2.053363	0.040037345	0.28991802
WDR82	-1.149837	0.55998	-2.053351	0.040038527	0.28991802
ENSG00000267787	-2.436066	1.186815	-2.052609	0.040110501	0.28991802
NEIL2	-1.716965	0.836591	-2.052336	0.040137049	0.28991802
MAU2	-1.23722	0.602935	-2.051994	0.040170289	0.28991802
ZNF609	-1.519703	0.740616	-2.051946	0.040174917	0.28991802
FLYWCH1	-1.31781	0.642256	-2.051844	0.04018486	0.28991802
ATPSCKMT	-2.753117	1.34204	-2.051441	0.040223979	0.28991802
SLC1A4	-3.106136	1.514179	-2.051367	0.040231254	0.28991802
CREG1	-2.904673	1.415981	-2.05135	0.040232851	0.28991802
IQCG	-1.554443	0.757793	-2.051276	0.040240058	0.28991802
KIAA0391	-3.794815	1.850004	-2.051247	0.040242905	NA
NPC1	-1.454611	0.709184	-2.051105	0.040256744	0.28991802
ZNF543	-1.857633	0.905692	-2.051064	0.040260742	0.28991802
WHRN	-3.125076	1.523737	-2.050929	0.040273876	0.28991802
MTOR	-2.493808	1.215977	-2.050868	0.040279799	0.28991802
BRD3OS	-1.249926	0.609657	-2.050214	0.040343593	0.28991802
KDM8	-2.864574	1.397267	-2.050127	0.040352076	0.28991802
ENSG00000124593	-2.655873	1.29547	-2.050123	0.040352405	0.28991802
ELK1	-1.172311	0.571873	-2.049949	0.040369399	0.28991802
CSTF3.DT	-3.704703	1.807501	-2.049627	0.040400813	NA
ACOX3	-1.875283	0.91534	-2.04873	0.040488539	0.29019907
RRM1	-1.380433	0.673813	-2.048688	0.040492644	0.29019907
TTL	-1.576569	0.769667	-2.048379	0.040522877	0.29019907
ZNF254	-1.229011	0.600039	-2.048218	0.040538672	0.29019907
NEMP2	-1.962051	0.958059	-2.047943	0.040565582	0.29019907
PCGF6	-1.276388	0.623373	-2.047551	0.040603974	0.29019907
NPHP3	-1.281532	0.625953	-2.047329	0.040625805	0.29019907
ZNF287	-1.633051	0.79768	-2.047251	0.040633494	0.29019907
PLBD2	-3.244654	1.584972	-2.047136	0.04064469	NA
ZYX	-1.35746	0.663637	-2.045486	0.040806962	0.29035871
EPG5	-1.297722	0.634656	-2.044766	0.040877904	0.29035871

ENSG00000277283	-1.943193	0.950378	-2.044652	0.040889149	0.29035871
GINS2	-2.362231	1.155434	-2.044453	0.04090879	0.29035871
KIAA1143	-1.088387	0.532399	-2.044305	0.040923386	0.29035871
IGHMBP2	-1.677002	0.820372	-2.044197	0.040934105	0.29035871
ENSG00000228427	-1.935039	0.946601	-2.044196	0.040934145	0.29035871
UBLCP1	-1.374661	0.672482	-2.044162	0.040937573	0.29035871
MYO9A	-1.301067	0.636605	-2.043759	0.040977331	0.29035871
B3GNT2	-1.277757	0.625203	-2.043749	0.04097833	0.29035871
MAPK14	-1.330257	0.65092	-2.043658	0.040987333	0.29035871
LRRC34	-2.899366	1.418895	-2.043397	0.04101314	0.29035871
NFYC.AS1	-2.026785	0.992062	-2.043002	0.041052255	0.29035871
ZNF718	-2.389407	1.169558	-2.042999	0.041052515	0.29035871
TONSL	-2.377811	1.164029	-2.042743	0.04107788	0.29035871
ZNF623	-1.563911	0.766038	-2.041557	0.041195466	0.29087564
KRBA1	-2.376085	1.163928	-2.041437	0.041207383	0.29087564
RPP14	-1.268741	0.621809	-2.040404	0.041310142	0.29133865
ENSG00000273456	-3.553166	1.74163	-2.040139	0.041336496	NA
RND1	-2.426378	1.189452	-2.039913	0.041359036	0.29133865
ADAM12	-3.331066	1.633396	-2.03935	0.041415122	NA
MEGF6	-1.886625	0.925221	-2.039107	0.041439386	0.29133865
IGLV6.57	-2.979745	1.461645	-2.038625	0.041487469	0.29133865
ERMARD	-2.130004	1.044825	-2.038622	0.041487735	0.29133865
WWP2	-1.258519	0.617475	-2.03817	0.041532975	0.29133865
ENSG00000273270	-2.138425	1.049332	-2.037893	0.041560678	0.29133865
RPE	-1.346356	0.660746	-2.037628	0.0415871	0.29133865
B3GNT7	-2.228386	1.093638	-2.03759	0.041590982	0.29133865
ASB7	-1.705413	0.837029	-2.037459	0.041604051	0.29133865
SLC35G2	-2.491639	1.222948	-2.037403	0.041609663	0.29133865
HMBS	-1.604177	0.787453	-2.037173	0.041632756	0.29133865
RALGDS	-1.38809	0.681405	-2.0371	0.041639972	0.29133865
NBAS	-1.809157	0.888341	-2.036556	0.041694549	0.29134263
INTS2	-2.362106	1.159948	-2.036389	0.041711315	0.29134263
ZMYND19	-3.417364	1.67825	-2.036267	0.041723601	NA
NPIPB4	-2.84102	1.395435	-2.035938	0.041756557	0.29134263
MINPP1	-1.610451	0.791107	-2.035693	0.041781212	0.29134263
FARP2	-1.239332	0.608906	-2.035342	0.041816523	0.29134263
ZFP30	-1.865538	0.916662	-2.035143	0.04183649	0.29134263
C22orf46	-1.524151	0.749103	-2.034636	0.041887483	0.29134263
MAP1LC3A	-1.71318	0.842023	-2.0346	0.041891186	0.29134263
ROR1	-1.879798	0.923931	-2.034565	0.041894619	0.29134263
KLHDC4	-1.189946	0.585081	-2.033815	0.041970228	0.29167189
PABPC3	-1.862856	0.915963	-2.033767	NA	NA
ZP3	-2.311222	1.13665	-2.033363	0.042015864	0.29179254
ACADS	-2.357753	1.159705	-2.033063	0.04204612	0.2918063
SEPSECS.AS1	-2.090767	1.028619	-2.032596	0.042093326	0.29193759
HCFC1R1	-1.055373	0.51931	-2.032262	0.042127187	0.29197621
CNNM2	-2.170912	1.06894	-2.030902	0.042264972	0.29271842

C2orf15	-2.686734	1.323223	-2.030446	0.042311185	0.29271842
IMMP2L	-1.625967	0.801078	-2.029724	0.042384621	0.29271842
ENSG00000203325	-2.662513	1.311884	-2.029535	0.04240384	0.29271842
ARNTL	-2.308282	1.137467	-2.029318	0.042425871	0.29271842
ENSG00000228835	-1.593909	0.785555	-2.029021	0.042456119	0.29271842
ARHGAP32	-2.669827	1.316669	-2.027714	0.042589426	0.29271842
FAM193B	-1.445692	0.712998	-2.027624	0.042598624	0.29271842
MKS1	-3.416607	1.68513	-2.027504	0.042610939	NA
DIPK1B	-2.282881	1.126135	-2.027183	0.042643721	0.29271842
MFN2	-1.666436	0.822053	-2.027165	0.042645591	0.29271842
PIK3CB	-2.269789	1.119711	-2.027121	0.042650016	0.29271842
CASP9	-2.033561	1.003244	-2.026985	0.04266399	0.29271842
RAB12	-2.758517	1.360955	-2.026898	0.042672801	0.29271842
TTBK2	-1.234591	0.609207	-2.026554	0.042708058	0.29271842
C20orf194	-1.928743	0.951764	-2.026493	0.042714307	0.29271842
PGM3	-1.267194	0.625332	-2.026433	0.042720461	0.29271842
CPSF4	-1.189374	0.586978	-2.026267	0.042737477	0.29271842
TMEM39A	-1.424882	0.703253	-2.026131	0.042751381	0.29271842
TEF	-2.573064	1.270073	-2.025918	0.042773195	0.29271842
TMEM38A	-1.860599	0.918888	-2.024838	0.04288405	0.29328257
ACP2	-2.412726	1.191941	-2.0242	0.042949625	0.2933847
LYSMD3	-1.620099	0.800379	-2.024164	NA	NA
C1RL	-2.287603	1.130296	-2.023898	0.042980654	0.2933847
DDIT4	1.274923	0.630091	2.023394	0.043032556	0.2933847
GPR157	-2.836623	1.402167	-2.023027	0.043070379	0.2933847
MSRB2	-2.050391	1.013777	-2.022525	0.043122102	0.2933847
NREP	-1.29653	0.641145	-2.022211	0.043154535	0.2933847
ASPH	-3.123278	1.544562	-2.022112	0.043164806	0.2933847
AKAP5	-2.346152	1.160524	-2.021632	0.04321436	0.2933847
CIP2A	-1.741551	0.861464	-2.021618	0.043215791	0.2933847
HIP1	-1.738912	0.860205	-2.021509	0.043227145	0.2933847
INPP5F	-2.284918	1.130472	-2.021207	0.043258358	0.2933847
MAP3K20	-1.51476	0.749524	-2.020963	0.043283585	0.2933847
GGT7	-2.569151	1.271702	-2.020246	0.043357853	0.2933847
SLC16A13	-1.451539	0.71852	-2.020178	0.043364915	0.2933847
PANK4	-1.829412	0.905578	-2.020159	0.043366854	0.2933847
AP3S2	-1.661583	0.822514	-2.020128	0.043370113	0.2933847
PGGHG	-1.13419	0.561485	-2.019983	0.043385143	0.2933847
GRK5	-1.241947	0.615108	-2.019071	0.043479806	0.2933847
AUH	-1.255072	0.621655	-2.018921	0.043495395	0.2933847
MTM1	-1.629766	0.807278	-2.018841	0.043503756	0.2933847
USP40	-2.372032	1.175364	-2.018125	0.04357824	0.2933847
VTA1	-1.25579	0.62231	-2.017948	0.043596678	0.2933847
SLC38A6	-2.680845	1.328598	-2.0178	0.043612089	0.2933847
SPINT1	-2.588621	1.283107	-2.017463	0.043647177	0.2933847
ZSCAN9	-1.460657	0.724013	-2.017446	0.043648947	0.2933847
ZFYVE21	-1.186397	0.588077	-2.017417	0.043652058	0.2933847

BCOR	-1.219746	0.60465	-2.017278	0.04366656	0.2933847
PON2	-3.35896	1.665331	-2.016992	0.0436963	0.2933935
KBTBD4	-1.203012	0.596623	-2.016368	0.043761461	0.29360618
RANBP10	-1.480274	0.73421	-2.016144	0.043784875	0.29360618
NINJ1	-1.979283	0.982303	-2.014942	0.043910726	0.29425889
GDPD3	-3.239654	1.608328	-2.0143	0.04397805	NA
ENSG00000258572	-3.380427	1.679289	-2.013011	0.044113503	NA
AGER	-1.656427	0.822881	-2.01296	0.044118885	0.29485671
ENSG00000277959	-2.631599	1.307454	-2.012766	0.044139232	0.29485671
DBR1	-1.732625	0.861096	-2.012115	0.044207861	0.29485671
MED26	-1.512151	0.751561	-2.012014	0.044218472	0.29485671
THAP7.AS1	-1.637574	0.813977	-2.011819	0.044239066	0.29485671
IFT74	-1.390212	0.691027	-2.011806	0.044240361	0.29485671
SLC1A5	-2.582367	1.283611	-2.011799	0.044241174	0.29485671
UBXN2B	-1.5019	0.746549	-2.011791	0.044241971	0.29485671
EEF1AKNMT	-1.269679	0.631239	-2.011409	0.044282297	0.29485671
SLC15A2	-2.119299	1.053656	-2.011377	0.044285649	0.29485671
BCO2	-2.482749	1.234735	-2.010754	0.044351448	0.29493494
LYSMD1	-3.219276	1.601052	-2.010725	0.044354558	0.29493494
FBXL22	-2.167295	1.078205	-2.010096	0.044420978	0.29501811
CASP1	-1.209252	0.601612	-2.01002	0.044429069	0.29501811
OR2A1.AS1	-2.223365	1.106306	-2.00972	0.044460807	0.29501811
WNT2B	-2.253869	1.121735	-2.009271	0.044508384	0.29501811
IFT81	-2.518368	1.253384	-2.009256	0.044509999	0.29501811
TPCN2	-2.389267	1.189861	-2.008022	0.044640933	0.29534326
FMO5	-2.439071	1.214921	-2.007596	0.044686261	0.29534326
SETD1B	-1.37084	0.68285	-2.007527	0.044693569	0.29534326
VPS39	-2.629211	1.309796	-2.007343	0.044713131	0.29534326
IGF1R	-2.483717	1.237447	-2.00713	0.044735797	0.29534326
ENSG00000167807	-2.971341	1.480519	-2.00696	0.044753955	0.29534326
ENSG00000256448	-3.035725	1.512637	-2.006909	0.044759385	0.29534326
GPAM	-2.586904	1.289338	-2.006382	0.044815535	0.29539158
MIR762HG	-1.960423	0.977132	-2.006303	0.044823954	0.29539158
CCDC102B	-2.458514	1.225954	-2.005387	0.04492165	0.29553707
VTI1A	-1.129692	0.563493	-2.0048	0.044984405	0.29553707
MX1	-1.404616	0.700685	-2.004634	0.045002187	0.29553707
SNX25	-1.344006	0.670475	-2.004559	0.045010167	0.29553707
BEND7	-1.566922	0.781808	-2.004228	0.045045692	0.29553707
TP53BP2	-1.688315	0.84254	-2.00384	0.04508721	0.29553707
ROM1	-3.088515	1.541617	-2.003425	0.045131712	0.29553707
CCDC157	-2.562511	1.279336	-2.003001	0.045177225	0.29553707
ENSG00000273723	-1.589411	0.793682	-2.002578	0.045222589	0.29553707
DPH6	-2.745269	1.370991	-2.002398	0.045241953	0.29553707
SENP3	-1.385301	0.691926	-2.002094	0.045274607	0.29553707
ALG10B	-2.494353	1.245925	-2.002009	0.04528374	0.29553707
NHLRC2	-1.246961	0.622893	-2.001885	0.04529713	0.29553707
DNASE1L1	-1.642964	0.820718	-2.001862	0.045299618	0.29553707

AXIN1	-1.574093	0.786329	-2.001826	0.045303472	0.29553707
KLK4	-3.269269	1.633471	-2.001425	0.045346606	0.29553707
B3GLCT	-1.99768	0.998143	-2.001397	0.045349674	0.29553707
ERI1	-1.376624	0.687897	-2.001207	0.045370094	0.29553707
TTN.AS1	-1.616487	0.807923	-2.000793	0.045414689	0.29553707
JAM3	-1.945976	0.97286	-2.000263	0.045471875	0.29553707
ENSG00000278238	-2.579802	1.289825	-2.000118	0.04548755	0.29553707
CRYBG1	-1.263326	0.631642	-2.000066	0.045493095	0.29553707
C5orf34	-2.45206	1.226897	-1.998587	0.045653059	0.29553707
PSD3	-1.612943	0.807062	-1.998536	0.045658594	0.29553707
FLYWCH2	-1.119727	0.560323	-1.998361	0.045677532	0.29553707
RXRA	-2.836599	1.419499	-1.998311	0.045682995	0.29553707
SIGMAR1	-1.46666	0.734072	-1.99798	0.045718829	0.29553707
ANKRD37	-2.122283	1.062505	-1.997434	0.04577806	0.29553707
ENSG00000280433	-3.013307	1.508632	-1.997377	0.045784273	0.29553707
DCAF4	-1.663835	0.833065	-1.997244	NA	NA
ZNF8	-2.509938	1.256751	-1.997164	0.045807334	0.29553707
CYP2U1	-1.743108	0.872924	-1.996863	0.045840115	0.29553707
ZNF837	-2.283674	1.143785	-1.996593	0.045869448	0.29553707
ENSG00000256591	-3.191188	1.59844	-1.996439	0.045886189	NA
KLHL21	-2.099898	1.051868	-1.996351	0.0458957	0.29553707
FANCD2	-1.369955	0.686276	-1.996216	0.0459104	0.29553707
TMEM87B	-1.635191	0.819151	-1.996202	0.045911895	0.29553707
SRR	-3.497003	1.751929	-1.996087	0.045924406	NA
ENSG00000266340	-2.604756	1.305085	-1.995852	0.045950049	0.29553707
PMS2	-1.207678	0.605101	-1.99583	0.045952471	0.29553707
RANBP9	-1.321454	0.662146	-1.995716	0.045964893	0.29553707
TESK1	-1.867006	0.935689	-1.995328	0.04600715	0.29553707
UBR7	-1.329842	0.666511	-1.99523	0.04601777	0.29553707
CHUK	-2.103473	1.054344	-1.995054	0.046037044	0.29553707
DISP1	-2.023605	1.014366	-1.994946	0.0460488	0.29553707
ENSG00000272990	-2.567262	1.288008	-1.993204	0.046239143	0.29602119
ZNF219	-3.752319	1.883127	-1.9926	0.04630523	NA
EDRF1	-1.194914	0.599743	-1.992376	0.046329866	0.29602119
NCEH1	-2.966327	1.488849	-1.992363	0.046331253	0.29602119
PSMG1	-1.360691	0.682958	-1.992349	0.046332797	0.29602119
TBX6	-2.251112	1.129986	-1.99216	0.046353518	0.29602119
AGAP5	-3.285068	1.649034	-1.992117	0.046358244	NA
ACSF2	-1.449976	0.727875	-1.992067	0.046363652	0.29602119
ECI1	-1.377265	0.691469	-1.991795	0.04639354	0.29602119
C2CD3	-1.65586	0.831496	-1.991423	0.046434364	0.29602119
LMBR1	-1.918143	0.963296	-1.991229	0.046455689	0.29602119
POLI	-2.463948	1.237403	-1.991224	0.046456215	0.29602119
LINC00562	-2.329664	1.170088	-1.991015	0.046479237	0.29602119
CCDC88A	-2.193289	1.10166	-1.990895	0.046492445	0.29602119
ENSG00000242282	-2.712111	1.362286	-1.990852	0.046497127	0.29602119
ENSG00000253891	-3.255295	1.635636	-1.990232	0.046565405	NA

CAMK2G	-1.099166	0.552297	-1.990173	0.046571926	0.2963146
TMTC3	-2.188072	1.099838	-1.989449	0.046651686	0.29663919
PPIP5K1	-3.261628	1.639794	-1.989048	0.04669594	NA
TIGD6	-3.773193	1.897014	-1.989017	0.0466993	NA
KLHDC1	-2.622193	1.318473	-1.988811	0.046722082	0.29690387
SPIN4	-3.402602	1.711075	-1.988576	0.046748024	NA
PSPN	-2.763661	1.390079	-1.988132	0.046797043	0.2970904
SLCO4A1	-1.577432	0.793602	-1.987686	0.046846415	0.2970904
ESRRA	-1.535545	0.772569	-1.987582	0.046857955	0.2970904
ENSG00000250073	-3.023829	1.521421	-1.987504	0.046866586	0.2970904
HEATR6	-1.55464	0.782324	-1.987207	0.046899482	0.29711642
ENSG00000237310	-2.044942	1.029211	-1.986902	0.046933249	0.29714793
TBC1D19	-2.342134	1.179285	-1.986064	0.047026267	0.29714982
ARHGEF19	-1.582342	0.796778	-1.985926	0.047041513	0.29714982
RABL3	-1.80312	0.907981	-1.985856	0.047049282	0.29714982
FHL3	-2.64703	1.332969	-1.985815	0.047053837	0.29714982
DTX3	-2.463229	1.240682	-1.985383	0.047101848	0.29714982
CATSPER2	-1.447292	0.729073	-1.985114	0.047131784	0.29714982
PPP2CB	-2.2307	1.123731	-1.985084	0.047135102	0.29714982
ZNF850	-2.951134	1.4869	-1.984757	0.047171568	0.29719816
DNAJC11	-1.726214	0.870115	-1.983891	NA	NA
MGST2	-2.509689	1.265293	-1.983484	0.047313432	0.29727795
LMNTD2	-3.208562	1.618013	-1.983026	0.047364518	0.29727795
CCDC122	-1.544881	0.779248	-1.982528	0.047420202	0.29727795
ZNF184	-1.74587	0.880656	-1.982464	0.047427319	0.29727795
ENSG00000258472	-2.630156	1.326759	-1.982391	0.047435496	0.29727795
NUBPL	-2.082468	1.050611	-1.982149	0.047462592	0.29727795
FBXO30	-1.694318	0.854966	-1.981739	0.047508491	0.29727795
ENSG00000277496	-1.988014	1.003198	-1.981677	0.047515425	0.29727795
SEPSECS	-2.157356	1.088692	-1.981603	0.047523696	0.29727795
UHRF1BP1L	-1.676913	0.846299	-1.981466	0.047539064	0.29727795
THEMIS2	-1.476122	0.744972	-1.981447	0.047541144	0.29727795
ARMC7	-1.431147	0.722442	-1.980984	0.047593076	0.29727795
TBC1D31	-2.549962	1.28725	-1.980938	0.047598264	0.29727795
SLC31A2	-3.567965	1.801395	-1.980668	0.047628522	NA
PLCB2	-1.892406	0.955559	-1.980418	0.047656623	0.29727795
SLC39A13	-1.594724	0.805275	-1.980346	0.047664645	0.29727795
URGCP	-2.266372	1.144438	-1.980336	0.047665765	0.29727795
ENSG00000227388	-1.646278	0.831401	-1.980125	0.047689484	0.29727795
DPY19L4	-2.448194	1.236457	-1.980007	0.04770274	0.29727795
GPHN	-2.843423	1.436364	-1.979598	0.047748723	0.29737797
ENSG00000244567	-3.80769	1.923579	-1.979481	0.047761842	NA
ENSG00000269246	-3.074838	1.553457	-1.979352	0.047776422	0.29737797
BAHD1	-1.786658	0.902853	-1.978903	0.047826949	0.29741172
LRRC56	-2.376893	1.201184	-1.978792	0.047839483	0.29741172
VARS	-3.51418	1.776336	-1.978331	0.047891351	0.29755493
SEC24B.AS1	-3.452465	1.745881	-1.977492	0.047986096	NA

CRY1	-2.289243	1.15799	-1.976912	0.048051609	0.29807267
ATP1B3	-1.039214	0.525831	-1.976328	0.048117686	0.29807267
NAMPT	-1.329224	0.672593	-1.976268	0.048124393	0.29807267
MAD2L1	-3.539237	1.791192	-1.975912	0.04816478	0.29807267
ZNF823	-2.829263	1.432098	-1.975607	0.048199306	0.29807267
CA5B	-1.554503	0.786983	-1.975269	0.048237607	0.29807267
ENSG00000260572	-4.122201	2.087097	-1.975088	0.04825814	NA
CDC37L1.DT	-2.084225	1.055258	-1.975086	0.048258349	0.29807267
ENSG00000262165	-4.150487	2.101451	-1.975058	0.048261539	NA
MORN1	-3.06201	1.55045	-1.974917	0.048277521	0.29807267
ENPP4	-2.771715	1.403526	-1.974823	0.048288232	0.29807267
ELL2	-3.35586	1.699326	-1.974819	0.048288737	0.29807267
KLF16	-2.146418	1.086912	-1.974786	0.048292394	0.29807267
ENSG00000271553	-3.169659	1.605362	-1.97442	0.048333987	NA
ENSG00000259976	-1.387978	0.703041	-1.974251	0.048353264	0.29826998
GATM	-2.245584	1.137979	-1.97331	0.048460284	0.29848057
DHX58	-1.892204	0.95894	-1.973224	0.048470008	0.29848057
MCCC1	-1.273784	0.645546	-1.973188	0.04847417	0.29848057
CORO2A	-2.993728	1.517625	-1.97264	0.048536558	0.29851309
TRMT44	-2.196102	1.113284	-1.972634	0.048537303	0.29851309
ABCB10	-2.371427	1.202399	-1.972246	0.048581519	0.29860707
MRRF	-1.215018	0.61615	-1.97195	0.048615346	0.29863713
ENSG00000240401	-3.470054	1.760733	-1.970801	0.048746659	NA
NCAPD3	-2.26721	1.150701	-1.970287	0.048805471	0.29915374
FAM120C	-2.28554	1.160331	-1.969732	0.048869151	0.29915374
TMEM234	-1.459528	0.741017	-1.969628	0.048881029	0.29915374
PRPF40B	-2.471568	1.255094	-1.969228	0.048926869	0.29915374
CTSC	-1.192008	0.605374	-1.969044	0.048948036	0.29915374
TEX2	-2.615999	1.328932	-1.968497	0.049010933	0.29915374
CDC37L1	-1.253404	0.636735	-1.968485	0.049012244	0.29915374
ZNF620	-2.464361	1.252067	-1.968234	0.049041167	0.29915374
ZNF284	-2.656641	1.349767	-1.968222	0.049042495	0.29915374
MATN1.AS1	-2.675799	1.359921	-1.967614	0.049112494	0.29915374
SLC37A2	-1.785035	0.907257	-1.967508	0.049124697	0.29915374
FCHSD1	-1.732031	0.880424	-1.96727	0.049152048	0.29915374
TAGLN	-1.937697	0.985093	-1.96702	0.049180901	0.29915374
SAC3D1	-2.949279	1.499468	-1.966884	0.049196563	0.29915374
CMTM3	-1.50295	0.764346	-1.966322	0.049261378	0.29915374
IGHE	-2.136293	1.086851	-1.96558	0.049347096	0.29915374
NUP214	-1.160458	0.590429	-1.965447	0.049362503	0.29915374
SEC61A1	-1.165111	0.592845	-1.965288	0.049380933	0.29915374
SRD5A3	-1.646539	0.837817	-1.965273	0.049382704	0.29915374
TMEM181	-2.828247	1.439393	-1.964888	0.049427178	0.29915374
CHKB	-1.255349	0.6389	-1.96486	0.049430411	0.29915374
TAF13	-2.501176	1.272969	-1.964836	0.049433191	0.29915374

Cluster 7	log2FC	lfcSE	stat	pvalue	padj
VPREB3	2.854232	0.24388	11.703412	< 2.22e-16	< 2.22e-16
GRN	1.384602	0.231108	5.991136	2.08E-09	1.34E-05
LPCAT2	-5.543954	0.964884	-5.745721	9.15E-09	3.93E-05
CIB2	-4.434359	0.792031	-5.598717	2.16E-08	5.92E-05
LINC01800	-4.802941	0.859533	-5.587848	2.30E-08	5.92E-05
TNFSF14	-5.086043	0.95761	-5.311186	1.09E-07	0.000202244
ZNF318	-2.077798	0.391348	-5.309334	1.10E-07	0.000202244
SLC9B1	-4.473759	0.847329	-5.279838	1.29E-07	0.00020796
JCHAIN	2.42661	0.463884	5.23107	1.69E-07	0.000240944
LINC00618	-4.765824	0.916332	-5.200979	1.98E-07	0.000242661
TUFT1	-4.97192	0.959824	-5.180031	2.22E-07	0.000242661
CRYM	-4.577346	0.885031	-5.171961	2.32E-07	0.000242661
MS4A1	-0.749186	0.145259	-5.157569	2.50E-07	0.000242661
KLK2	-4.17595	0.813331	-5.134377	2.83E-07	0.000242661
IGLV1.51	-4.208837	0.821642	-5.122471	3.02E-07	0.000242661
NPHP1	-3.86081	0.75517	-5.112507	3.18E-07	0.000242661
LINC01991	-3.349752	0.655412	-5.110913	3.21E-07	0.000242661
MIR210HG	-4.301304	0.848352	-5.070191	3.97E-07	0.000267909
ZNF233	-4.180884	0.825341	-5.065646	4.07E-07	0.000267909
GPR161	-4.172761	0.824446	-5.06129	4.16E-07	0.000267909
ENSG00000260331	-4.527788	0.899803	-5.031979	4.85E-07	0.000297437
HIST1H2AH	-4.482653	0.895069	-5.008166	5.50E-07	0.000321389
ENSG00000274015	-4.906912	0.986615	-4.973481	6.58E-07	0.000354517
NECAB3	-4.088924	0.822322	-4.97241	6.61E-07	0.000354517
ENSG00000273165	-4.977281	1.003239	-4.961213	7.01E-07	0.000360555
EPB41L4A	-5.219148	1.064983	-4.900686	9.55E-07	0.000472628
MTRNR2L3	-4.692298	0.961392	-4.880732	1.06E-06	0.000503686
ZNF221	-4.116133	0.848031	-4.853751	1.21E-06	0.000556717
CEP72	-4.040483	0.83654	-4.829995	1.37E-06	0.000598109
P3H4	-4.935412	1.022717	-4.825787	1.39E-06	0.000598109
TEF	-4.067284	0.855083	-4.756594	1.97E-06	0.000743237
TTC30A	-4.306932	0.906205	-4.752711	2.01E-06	0.000743237
UACA	-3.767404	0.792837	-4.751802	2.02E-06	0.000743237
ENSG00000272854	-4.764412	1.002702	-4.751574	2.02E-06	0.000743237
ADAM12	-4.339169	0.913464	-4.750236	2.03E-06	0.000743237
ENSG00000224934	-4.990534	1.052318	-4.742421	2.11E-06	0.000743237
LMO2	-4.09339	0.863585	-4.739995	2.14E-06	0.000743237
ENSG00000265100	-4.784995	1.013791	-4.719902	2.36E-06	0.000785934
ENSG00000266378	-3.936342	0.834331	-4.717963	2.38E-06	0.000785934
LINC02099	-4.588187	0.979534	-4.684053	2.81E-06	0.000904734
PRR5	-4.883462	1.048718	-4.656603	3.21E-06	0.000983078
C5orf17	-4.640488	0.996903	-4.654903	3.24E-06	0.000983078
ENSG00000230747	-4.351161	0.935709	-4.65012	3.32E-06	0.000983078
ENSG00000267633	-4.652077	1.001992	-4.64283	3.44E-06	0.000983078
ARHGAP5.AS1	-5.287809	1.138941	-4.642743	3.44E-06	0.000983078
ENSG00000280011	-3.694563	0.798081	-4.629306	3.67E-06	0.001026264

REPS2	-3.591461	0.77812	-4.615562	3.92E-06	0.001042159
KIF18A	-3.422656	0.741562	-4.615466	3.92E-06	0.001042159
H1FX.AS1	-4.881934	1.059098	-4.609522	4.04E-06	0.001042159
FRMPD1	-4.215548	0.914671	-4.608813	4.05E-06	0.001042159
BCL9	-4.043987	0.878794	-4.601745	4.19E-06	0.001057024
ENSG00000225335	-4.210724	0.91691	-4.592296	4.38E-06	0.001084779
ENSG00000274292	-4.220849	0.924366	-4.56621	4.97E-06	0.001181146
GSTM2	-4.329527	0.948581	-4.564212	5.01E-06	0.001181146
ENSG00000272579	-3.920037	0.859401	-4.56136	5.08E-06	0.001181146
ENSG00000186019	-4.814581	1.056069	-4.558966	5.14E-06	0.001181146
TTYH2	-4.346782	0.959662	-4.529494	5.91E-06	0.001334671
KCNQ1	-4.448622	0.983255	-4.524385	6.06E-06	0.001338089
ENSG00000261079	-3.979876	0.88018	-4.521661	6.14E-06	0.001338089
ENSG00000272800	-4.733309	1.048059	-4.516264	6.29E-06	0.001349751
ATP6V0E2.AS1	-4.354996	0.967784	-4.499966	6.80E-06	0.001433599
RGS9	-3.994482	0.889548	-4.490465	7.11E-06	0.001474888
DMXL2	-4.315991	0.962479	-4.484246	7.32E-06	0.001494457
SIRPA	-4.239828	0.946592	-4.479045	7.50E-06	0.001507405
ADM2	-3.530647	0.789514	-4.471922	7.75E-06	0.001534531
ARHGAP21	-4.252485	0.952805	-4.463122	8.08E-06	0.001574728
HAVCR2	-3.926323	0.88089	-4.457221	8.30E-06	0.001594528
PPP1R26	-4.227115	0.949149	-4.453582	8.44E-06	0.001597958
ADCY4	-4.353299	0.980581	-4.43951	9.02E-06	0.001670629
SPAG5	-4.711914	1.06177	-4.437791	9.09E-06	0.001670629
ENSG00000272004	-4.723485	1.066328	-4.429674	9.44E-06	0.001673969
BCAT1	-4.389299	0.991422	-4.427274	9.54E-06	0.001673969
ENSG00000234915	-4.65916	1.052498	-4.426765	9.57E-06	0.001673969
KRTCAP3	-3.760739	0.850035	-4.424218	9.68E-06	0.001673969
VASH2	-4.671327	1.056268	-4.422482	9.76E-06	0.001673969
ENSG00000255495	-3.807211	0.86172	-4.418156	9.95E-06	0.001685353
ENSG00000271993	-3.716114	0.844066	-4.402633	1.07E-05	0.001762981
KIF15	-4.668126	1.060914	-4.400098	1.08E-05	0.001762981
LINC00937	-3.684647	0.837416	-4.400017	1.08E-05	0.001762981
APPL2	-2.757797	0.627219	-4.396864	1.10E-05	0.001766416
ENSG00000228242	-3.975467	0.905726	-4.389261	1.14E-05	0.001806725
SPA17	-4.166914	0.951043	-4.381417	1.18E-05	0.00184898
LINC00894	-4.143467	0.946231	-4.378917	1.19E-05	0.00184898
ENSG00000279714	-4.221214	0.965577	-4.371702	1.23E-05	0.001888409
IL6	-4.076217	0.932966	-4.369093	1.25E-05	0.001888624
TGFB1I1	-4.065201	0.931531	-4.364001	1.28E-05	0.001910676
ENSG00000267282	-4.127756	0.946686	-4.360217	1.30E-05	0.001921668
LINC02076	-3.671787	0.845613	-4.342158	1.41E-05	0.002047396
NR5A2	-3.79566	0.874306	-4.341339	1.42E-05	0.002047396
IFNG.AS1	-4.316183	0.995578	-4.335353	1.46E-05	0.002054564
BEX3	-4.044332	0.9329	-4.335227	1.46E-05	0.002054564
NMNAT3	-4.192455	0.967501	-4.333282	1.47E-05	0.002054564
ZNF135	-2.822113	0.651795	-4.329756	1.49E-05	0.00206529

NBPF3	-3.939929	0.91272	-4.31669	1.58E-05	0.002168038
SLC2A4RG	-3.085661	0.715548	-4.312307	1.62E-05	0.002188205
ENSG00000231294	-4.53909	1.05368	-4.307844	1.65E-05	0.002209552
CRYGS	-3.709445	0.861707	-4.304765	1.67E-05	0.002216095
ENSG00000266998	-3.841419	0.892808	-4.302625	1.69E-05	0.002216095
FAM230J	-3.886918	0.904192	-4.298775	1.72E-05	0.002232161
PIK3R4	-2.842747	0.661827	-4.2953	1.74E-05	0.002244742
NAGPA.AS1	-3.959636	0.92269	-4.291403	1.78E-05	0.002261889
CAPS	-2.571078	0.599615	-4.287885	1.80E-05	0.002275476
ADNP.AS1	-3.488229	0.815597	-4.276901	1.90E-05	0.00234093
ENSG00000280433	-2.983525	0.69798	-4.274512	1.92E-05	0.00234093
MMP23B	-4.435831	1.038069	-4.273157	1.93E-05	0.00234093
PDE1B	-4.270393	0.999387	-4.273013	1.93E-05	0.00234093
IGLV4.69	-3.828319	0.896487	-4.270355	1.95E-05	0.002346873
GP1BA	-3.7887	0.889549	-4.259127	2.05E-05	0.002445052
ENSG00000258940	-3.497781	0.821732	-4.256597	2.08E-05	0.002450182
CCRL2	-4.271755	1.005623	-4.247867	2.16E-05	0.00252445
ENSG00000271797	-3.768724	0.888475	-4.241791	2.22E-05	0.002570424
C7orf61	-3.806533	0.899596	-4.231379	2.32E-05	0.002668323
MOK	-3.567918	0.843664	-4.229077	2.35E-05	0.002671917
ENSG00000265778	-3.484069	0.825297	-4.221595	2.43E-05	0.002737959
ENSG00000260911	-4.49304	1.0653	-4.217628	2.47E-05	0.002752812
PCOLCE	-2.663865	0.631779	-4.216453	2.48E-05	0.002752812
LBH	-1.190953	0.282945	-4.209126	2.56E-05	0.002819302
PLK4	-4.670154	1.110366	-4.20596	2.60E-05	0.002834846
SNX21	-4.153112	0.988158	-4.202883	2.64E-05	0.002849524
ENSG00000223881	-3.633799	0.866504	-4.193634	2.75E-05	0.002943555
ENSG00000258944	-3.89742	0.930255	-4.189627	2.79E-05	0.002971244
GNRH1	-3.226316	0.771254	-4.183208	2.87E-05	0.003021525
FBLN2	-5.529076	1.322084	-4.182093	2.89E-05	0.003021525
TMEM225B	-3.712975	0.889524	-4.174116	2.99E-05	0.003104115
BIK	-4.110607	0.985511	-4.171043	3.03E-05	0.003117799
ENSG00000273314	-3.648801	0.875314	-4.168563	3.07E-05	0.003117799
ARHGAP22	-3.708922	0.889928	-4.167667	3.08E-05	0.003117799
IGKV3.20	-3.30255	0.793177	-4.163701	3.13E-05	0.003147687
PLCB3	-3.157559	0.75893	-4.160541	3.17E-05	0.003166828
SLC24A1	-4.426526	1.066337	-4.151151	3.31E-05	0.003272657
DPH1	-3.824819	0.922074	-4.148061	3.35E-05	0.003272657
ZNF286B	-3.5125	0.846841	-4.147766	3.36E-05	0.003272657
LINC02202	-3.559638	0.859417	-4.141921	3.44E-05	0.003331958
UNC79	-4.221184	1.020988	-4.134409	3.56E-05	0.003417142
CHPF	-4.26766	1.033798	-4.128136	3.66E-05	0.003485675
LMTK2	-3.638692	0.88237	-4.123769	3.73E-05	0.003526335
ENSG00000232811	-4.929516	1.197754	-4.115635	3.86E-05	0.003602824
ENSG00000261071	-3.42605	0.832483	-4.115461	3.86E-05	0.003602824
ENSG00000251330	-4.309099	1.048366	-4.1103	3.95E-05	0.0036578
ENSG00000260329	-4.352736	1.059567	-4.108035	3.99E-05	0.003667464

LIMA1	-3.791788	0.92448	-4.101535	4.10E-05	0.003726259
BMERB1	-3.7337	0.911313	-4.097056	4.18E-05	0.003726259
GPER1	-3.81926	0.932216	-4.096968	4.19E-05	0.003726259
RAB3B	-4.551274	1.110914	-4.096873	4.19E-05	0.003726259
ENSG00000258377	-3.904975	0.953308	-4.096239	4.20E-05	0.003726259
RGPD5	-3.322301	0.811579	-4.093626	4.25E-05	0.003742704
C17orf51	-3.077283	0.752561	-4.08908	4.33E-05	0.003780867
ENSG00000228037	-3.797794	0.928983	-4.088118	4.35E-05	0.003780867
ADD2	-3.358689	0.821968	-4.086153	4.39E-05	0.003787422
TRIM46	-3.957146	0.970562	-4.07717	4.56E-05	0.003910474
DLG2	-3.364345	0.825783	-4.074129	4.62E-05	0.003910849
PROB1	-3.372621	0.828057	-4.072933	4.64E-05	0.003910849
ACVR1B	-3.710472	0.91138	-4.071268	4.68E-05	0.003910849
SLC36A1	-3.77719	0.927824	-4.071022	4.68E-05	0.003910849
ENSG00000278817	-3.561807	0.876071	-4.065662	4.79E-05	0.003976019
LINC00987	-3.761046	0.927353	-4.055679	5.00E-05	0.004123124
ZNF391	-3.232661	0.797773	-4.052104	5.08E-05	0.004159979
ENSG00000255537	-3.416272	0.843921	-4.048096	5.16E-05	0.00420506
SPATS2L	-3.788828	0.937488	-4.041467	5.31E-05	0.004298544
MDS2	-3.437447	0.851917	-4.034956	5.46E-05	0.00439187
HIST1H1B	-3.911168	0.970237	-4.031147	5.55E-05	0.004435941
SERPINH1	-3.786815	0.940696	-4.025545	5.68E-05	0.004505825
CISH	-5.334528	1.325491	-4.024568	5.71E-05	0.004505825
GRM3	-4.390616	1.091434	-4.022797	5.75E-05	0.004512164
ENSG00000269814	-4.01477	0.999378	-4.017268	5.89E-05	0.004564297
ATXN7L2	-3.433164	0.854609	-4.017235	5.89E-05	0.004564297
LINC02021	-3.328407	0.829698	-4.011591	6.03E-05	0.00464684
CENPO	-3.30168	0.823781	-4.007959	6.12E-05	0.004690765
ENSG00000260317	-3.555302	0.888885	-3.999733	6.34E-05	0.004828088
LINC00562	-2.752813	0.689172	-3.994378	6.49E-05	0.004856361
U2AF1L5	-4.629025	1.158939	-3.994191	6.49E-05	0.004856361
IGLV2.11	-4.955237	1.240613	-3.994184	6.49E-05	0.004856361
LMNTD1	-2.962577	0.745943	-3.971587	7.14E-05	0.005310077
PTP4A3	-2.93351	0.739155	-3.968733	7.23E-05	0.005343201
IL18BP	-3.231153	0.815351	-3.962898	7.40E-05	0.005444249
CDKN2A	-3.234637	0.817527	-3.956611	7.60E-05	0.005557709
MESP1	-3.737419	0.944952	-3.955143	7.65E-05	0.005560352
ENSG00000244567	-3.319767	0.839924	-3.952463	7.74E-05	0.005582298
PBX4	-3.420559	0.865633	-3.951514	7.77E-05	0.005582298
ENSG00000262429	-3.048579	0.772337	-3.947211	7.91E-05	0.005651968
CHL1	-3.188933	0.809218	-3.940758	8.12E-05	0.005753669
ZSCAN22	-2.800066	0.710625	-3.940288	8.14E-05	0.005753669
CCDC150	-3.608686	0.916472	-3.937585	8.23E-05	0.005760706
FAM89A	-3.672598	0.932754	-3.937372	8.24E-05	0.005760706
SLC27A1	-3.33452	0.847778	-3.933246	8.38E-05	0.005804756
PLCG1	-3.217266	0.81803	-3.932946	8.39E-05	0.005804756
CCDC151	-4.718677	1.201714	-3.926622	8.61E-05	0.005927584

MIR34AHG	-3.612462	0.920331	-3.925175	8.67E-05	0.005931605
H3F3A	0.616242	0.157145	3.921491	8.80E-05	0.005962539
TIMP2	-3.563246	0.908673	-3.921374	8.80E-05	0.005962539
ENSG00000179428	-4.54731	1.160547	-3.918248	8.92E-05	0.006008755
ENSG00000273064	-3.352114	0.856827	-3.912242	9.14E-05	0.006128119
ITPRIPL1	-3.411754	0.872785	-3.909045	9.27E-05	0.006177609
CPLANE2	-3.460296	0.885995	-3.905549	9.40E-05	0.006235291
NR2F6	-3.124952	0.803868	-3.887394	0.000101326	0.006685974
LEMD2	-2.238784	0.577321	-3.877884	0.000105369	0.006882733
ZNF460.AS1	-2.969149	0.765781	-3.877284	0.000105629	0.006882733
DGAT1	-2.931119	0.75624	-3.875911	0.000106227	0.006882733
ENSG00000254162	-3.555778	0.917524	-3.875404	0.000106448	0.006882733
ABHD16A	-3.061883	0.791019	-3.870809	0.000108475	0.006923436
ENSG00000228989	-2.886272	0.745693	-3.870591	0.000108572	0.006923436
ENSG00000228436	-2.895411	0.74816	-3.870044	0.000108816	0.006923436
UCP3	-3.758426	0.971391	-3.869118	0.00010923	0.006923436
ENSG00000232874	-3.562511	0.923107	-3.859259	0.000113731	0.007173428
B4GALT1.AS1	-3.422182	0.887437	-3.856253	0.000115138	0.007177898
ANGPTL6	-3.753424	0.973389	-3.856037	0.00011524	0.007177898
PLBD2	-2.82075	0.73161	-3.855538	0.000115476	0.007177898
ENSG00000268573	-4.08696	1.062423	-3.846831	0.000119656	0.007401961
GPC2	-3.92958	1.0227	-3.842359	0.000121858	0.007497217
ENSG00000272455	-4.284779	1.115519	-3.841064	0.000122502	0.007497217
ZFP37	-3.443268	0.896642	-3.840181	0.000122943	0.007497217
LINC01001	-3.159199	0.824447	-3.8319	0.000127157	0.007717613
ASMTL.AS1	-3.345049	0.875387	-3.821225	0.00013279	0.008021659
CLIP4	-4.212752	1.103885	-3.816295	0.000135471	0.008145333
SUOX	-3.206146	0.841204	-3.811379	0.000138193	0.008270395
ZNF23	-4.004328	1.052664	-3.803994	0.000142382	0.008481593
CBX8	-3.087937	0.812124	-3.802296	0.000143361	0.008500588
HYAL3	-2.93912	0.77325	-3.800995	0.000144116	0.008506172
RAP2C.AS1	-3.027803	0.796846	-3.799732	0.000144853	0.008510581
COX16	-1.708394	0.450324	-3.793696	0.000148421	0.008680614
LINC00205	-4.212801	1.112228	-3.787712	0.000152041	0.008852075
ENSG00000250073	-3.427297	0.905929	-3.783186	0.000154834	0.008961866
SEMA3D	-4.356997	1.151911	-3.782406	0.00015532	0.008961866
ENSG00000225032	-3.474579	0.919103	-3.780401	0.000156576	0.008994044
NPIPA5	-3.916495	1.038647	-3.770765	0.000162748	0.009307017
ENSG00000240401	-2.627216	0.69781	-3.764946	0.000166585	0.009484287
ATL1	-3.284274	0.872737	-3.763189	0.00016776	0.009509126
DOK2	-3.267438	0.869054	-3.759764	0.000170074	0.009597969
IL1RAP	-2.958248	0.787643	-3.755825	0.000172771	0.009668624
LINC01816	-4.073885	1.08477	-3.755529	0.000172975	0.009668624
CCDC144A	-3.340462	0.889798	-3.754179	0.00017391	0.009668624
TRPV1	-2.953452	0.786837	-3.753574	0.000174331	0.009668624
EVI5L	-3.297243	0.879326	-3.749737	0.00017702	0.009734677
CAMKMT	-2.209662	0.589463	-3.748599	0.000177825	0.009734677

ENSG00000267421	-5.173216	1.380277	-3.747956	0.000178282	0.009734677
LINC00504	-3.519666	0.939196	-3.747529	0.000178585	0.009734677
ENSG00000262319	-3.628189	0.968416	-3.746519	0.000179305	0.009734677
ENSG00000261220	-2.853401	0.76434	-3.733156	0.000189095	0.010161507
PARD6B	-2.639803	0.707209	-3.732705	0.000189434	0.010161507
DEPDC4	-4.682953	1.254619	-3.73257	0.000189536	0.010161507
ELMO3	-3.062703	0.820774	-3.731483	0.000190356	0.010163101
ZNF607	-2.945722	0.789841	-3.729511	0.000191852	0.010200655
FGFRL1	-4.041242	1.084162	-3.727524	0.00019337	0.010239061
MPV17L	-3.046469	0.81809	-3.723878	0.000196186	0.010345585
ENSG00000281706	-3.357669	0.902279	-3.721322	0.000198183	0.010372764
LRP8	-3.166717	0.851004	-3.721155	0.000198314	0.010372764
ZNF133	-2.839953	0.763964	-3.717389	0.000201293	0.010485968
INSL3	-3.850403	1.03711	-3.712627	0.000205119	0.010642188
IGHV4.34	-3.348007	0.902209	-3.7109	0.000206524	0.010672062
TBC1D16	-2.743451	0.741977	-3.697489	0.000217743	0.011200504
ENSG00000278869	-3.119107	0.843909	-3.696024	0.000219002	0.011200504
ENSG00000264235	-3.319218	0.898215	-3.695349	0.000219585	0.011200504
FUT8.AS1	-4.215064	1.140871	-3.694601	0.000220232	0.011200504
ENSG00000248367	-3.04004	0.82424	-3.688296	0.000225761	0.011436501
PRELID3A	-3.961834	1.074816	-3.686056	0.000227756	0.011492296
TMEM255B	-3.176722	0.863687	-3.678095	0.000234983	0.01181064
RDH10	-3.434936	0.934936	-3.673978	0.000238803	0.011866401
AREL1	-2.467368	0.671821	-3.672658	0.000240041	0.011866401
SRRM2.AS1	-3.395936	0.924758	-3.672242	0.000240432	0.011866401
CTIF	-3.27902	0.892958	-3.672088	0.000240577	0.011866401
ENSG00000273272	-4.170949	1.136189	-3.671	0.000241603	0.011866401
TTC8	-3.568724	0.972146	-3.670976	0.000241626	0.011866401
LIX1.AS1	-3.206987	0.874435	-3.667497	0.000244936	0.011983248
ZNF713	-2.845459	0.776285	-3.665482	0.000246873	0.012007949
FAH	-3.037122	0.828861	-3.664213	0.000248101	0.012007949
CMC4	-3.116199	0.850813	-3.662614	0.000249655	0.012007949
AMIGO2	-3.241087	0.884941	-3.66249	0.000249775	0.012007949
YY2	-3.182461	0.86919	-3.661409	0.000250832	0.012007949
PHLDA3	-4.01495	1.096623	-3.661196	0.000251041	0.012007949
ENSG00000277763	-3.739141	1.022285	-3.657631	0.000254557	0.012087938
FAM161B	-3.036298	0.830135	-3.657596	0.000254592	0.012087938
PALM2	-3.234207	0.884756	-3.655477	0.000256704	0.012105386
FIRRE	-3.802494	1.040257	-3.655341	0.000256841	0.012105386
PLD6	-2.972285	0.813744	-3.652603	0.000259595	0.01219054
ASCL2	-3.669775	1.005466	-3.649824	0.000262421	0.012243123
GPX1	0.8225	0.225378	3.649421	0.000262832	0.012243123
RGL1	-3.757966	1.029946	-3.648701	0.000263569	0.012243123
SOX5	-5.291275	1.45093	-3.646817	0.000265509	0.012247397
ENSG00000233903	-2.971941	0.814953	-3.646763	0.000265565	0.012247397
ZNF707	-2.327706	0.638945	-3.643045	0.000269431	0.012339935
ABCD3	-2.287726	0.62798	-3.64299	0.00026949	0.012339935

ENSG00000237753	-2.968918	0.815692	-3.639753	0.0002729	0.012431532
NT5DC3	-2.863669	0.786882	-3.63926	0.000273422	0.012431532
ERI2	-3.327989	0.915533	-3.635027	0.000277951	0.012592953
DOCK7	-2.84053	0.78197	-3.632532	0.000280654	0.01264999
ENSG00000260778	-3.181529	0.875959	-3.632052	0.000281176	0.01264999
PKN3	-3.980279	1.097693	-3.62604	0.0002878	0.012902877
FAM83G	-2.479763	0.684895	-3.620648	0.000293866	0.013129063
CCSAP	-2.678273	0.740438	-3.617149	0.000297866	0.013261717
ENSG00000272973	-3.169224	0.876514	-3.615715	0.00029952	0.013289371
ENSG00000261879	-2.899097	0.802642	-3.611941	0.000303913	0.01343799
CCDC62	-3.359547	0.932006	-3.60464	0.000312586	0.013774129
ZNF554	-2.932097	0.813782	-3.60305	0.000314506	0.01381141
VPS33B.DT	-3.435176	0.954901	-3.597418	0.000321392	0.014065835
PAM	-2.739065	0.761764	-3.595688	0.000323535	0.014111605
WDR66	-2.954481	0.822104	-3.593803	0.000325886	0.014166147
ENSG00000266385	-3.324618	0.926765	-3.587337	0.000334072	0.014473083
FAM85B	-3.218599	0.899198	-3.57941	0.000344371	0.014869201
WNT10A	-2.59469	0.725227	-3.57776	0.000346551	0.014913285
ENSG00000272264	-3.206755	0.896744	-3.575997	0.000348895	0.014964111
ENSG00000272933	-2.878142	0.80563	-3.572537	0.000353539	0.015024343
PLEKHD1	-4.167094	1.166549	-3.572155	0.000354056	0.015024343
ANK1	-3.000097	0.839933	-3.571828	0.000354499	0.015024343
ENSG00000261420	-3.235404	0.905962	-3.571237	0.000355299	0.015024343
KDM4A.AS1	-2.988097	0.836936	-3.570283	0.000356596	0.015024343
STAT5A	-1.663659	0.466042	-3.569762	0.000357305	0.015024343
ENSG00000270127	-3.233125	0.907463	-3.562819	0.000366893	0.015377246
TSPAN4	-3.053424	0.858204	-3.557922	0.0003738	0.015615855
OPRL1	-3.037623	0.854492	-3.554887	0.000378141	0.015696767
UBAC2.AS1	-3.06082	0.861023	-3.554863	0.000378177	0.015696767
ENSG00000256950	-3.447868	0.971322	-3.549667	0.000385719	0.01595836
ENSG00000276259	-2.720152	0.766871	-3.547077	0.000389531	0.016064389
ADRB2	-2.664915	0.751902	-3.544231	0.000393759	0.016186911
CCDC146	-3.53153	0.996893	-3.542536	0.000396299	0.016239407
BOLA3.AS1	-2.650047	0.74828	-3.541516	0.000397834	0.016250583
ITGA1	-2.938675	0.830118	-3.540069	0.000400022	0.016262903
ALYREF	-2.575437	0.727712	-3.539088	0.000401512	0.016262903
ENSG00000265218	-3.714154	1.049548	-3.538815	0.000401928	0.016262903
PTPRCAP	-3.216475	0.909469	-3.53665	0.000405236	0.016345361
ENSG00000271976	-3.5023	0.991105	-3.533734	0.000409733	0.016475102
THNSL1	-2.906977	0.823119	-3.53166	0.00041296	0.016553135
HARBI1	-2.734103	0.77439	-3.530656	0.000414531	0.016564504
GTSF1	-2.483003	0.704116	-3.52641	0.000421234	0.016775329
ENSG00000228835	-2.699685	0.765723	-3.525669	0.000422414	0.016775329
ARHGEF10	-2.812681	0.797978	-3.524761	0.000423865	0.016779696
ABCB9	-3.482918	0.988351	-3.52397	0.000425133	0.016779696
CHRNE	-4.083154	1.159268	-3.522182	0.00042801	0.016790455
ENSG00000264548	-4.289746	1.218168	-3.521473	0.000429157	0.016790455

SFT2D3	-2.999537	0.851908	-3.520963	0.000429982	0.016790455
PUSL1	-2.167713	0.615868	-3.519766	0.000431928	0.016790455
KLHL17	-2.990535	0.849781	-3.519183	0.000432878	0.016790455
C19orf73	-2.886415	0.820386	-3.518362	0.00043422	0.016790455
ITGAV	-3.085513	0.877023	-3.518166	0.00043454	0.016790455
ASB2	2.581441	0.734278	3.51562	0.000438729	0.016880511
CCDC183	-2.947207	0.838428	-3.515157	0.000439494	0.016880511
RNF31	-2.87003	0.816664	-3.514334	0.000440858	0.016882491
C16orf95	-3.952635	1.125101	-3.513139	0.000442846	0.016903966
LTB4R	-2.963365	0.843787	-3.511983	0.000444777	0.016903966
ENSG00000231595	-4.005126	1.14053	-3.511635	0.00044536	0.016903966
LSM11	-2.976659	0.847877	-3.510723	0.000446891	0.016912181
ENSG00000263531	-2.702147	0.770475	-3.507118	0.000452988	0.01709264
SMIM13	-2.89729	0.826436	-3.505765	0.000455297	0.017129553
DENND6B	2.036788	0.581272	3.504017	0.000458296	0.017192122
MYH7B	-3.205589	0.91524	-3.502456	0.000460989	0.017242875
FRAT2	-3.220271	0.919829	-3.500944	0.000463613	0.017290732
STAT4	-2.467322	0.705721	-3.496171	0.000471985	0.017515081
C3orf33	-2.889488	0.826614	-3.49557	0.00047305	0.017515081
PRRT2	-2.845434	0.814098	-3.495197	0.000473712	0.017515081
ENSG00000182376	-3.885772	1.112584	-3.492564	0.000478407	0.017638006
ACRBP	-3.123201	0.894504	-3.491545	0.000480235	0.017654803
LINC00996	-3.188512	0.913475	-3.490531	0.000482062	0.017671487
TTLL1	-2.811544	0.806753	-3.485011	0.000492118	0.01798886
RASGEF1A	-2.868924	0.823931	-3.481995	0.000497693	0.018141136
MBOAT2	-3.697023	1.062146	-3.480712	0.000500083	0.01816471
NBPF26	-2.680334	0.770181	-3.480134	0.000501164	0.01816471
ENSG00000237357	-2.803743	0.806513	-3.476377	0.000508238	0.018369376
FRY	-3.157204	0.909196	-3.472522	0.000515593	0.018540801
SMC6	-0.983165	0.283138	-3.472381	0.000515863	0.018540801
RGMB	-2.83056	0.815586	-3.470587	0.000519323	0.018552754
NXPH4	-4.029833	1.161422	-3.469741	0.000520961	0.018552754
TCEAL9	-3.057189	0.881289	-3.468999	0.000522401	0.018552754
MEGF8	-2.68823	0.775037	-3.468518	0.000523337	0.018552754
DDIAS	-2.639673	0.761045	-3.468483	0.000523405	0.018552754
EIF2AK3.DT	-3.23366	0.93385	-3.462718	0.000534748	0.01882932
ENSG00000272374	-3.689577	1.065564	-3.462557	0.000535069	0.01882932
MAPK8IP1	-3.23902	0.935572	-3.462075	0.000536027	0.01882932
LIPE	-1.955731	0.565088	-3.460932	0.000538309	0.01882932
LRCH1	-1.855196	0.536056	-3.460824	0.000538524	0.01882932
S100A2	-2.705099	0.781916	-3.459579	0.00054102	0.018832878
NRF1	-2.164591	0.625728	-3.459314	0.000541553	0.018832878
SEPHS2	-2.943237	0.851072	-3.458271	0.000543654	0.018854971
ENSG00000277602	-3.641887	1.053702	-3.456279	0.000547689	0.018941908
SDSL	-2.867187	0.829726	-3.455583	0.000549105	0.018941908
ENSG00000240710	-2.802249	0.811625	-3.452641	0.000555127	0.019098434
GPD1L	-3.476405	1.007332	-3.4511	0.000558306	0.019156592

RAB23	-2.865518	0.831176	-3.447547	0.000565701	0.019293129
PDE6A	-2.7494	0.79754	-3.447352	0.000566111	0.019293129
CHKA	-2.624369	0.761342	-3.447031	0.000566783	0.019293129
JUND	-1.016003	0.294961	-3.444531	0.000572051	0.019408884
HIST1H2BF	-3.01909	0.876626	-3.443988	0.000573201	0.019408884
PRR22	-4.013721	1.165682	-3.443238	0.000574793	0.019411694
OSGEPL1.AS1	-3.342809	0.971409	-3.441198	0.000579145	0.01949918
ZNF646	-2.524337	0.733706	-3.440527	0.000580582	0.01949918
ENSG00000249604	-4.12344	1.198828	-3.43956	0.000582661	0.01949918
ERMAP	-2.955464	0.859347	-3.439196	0.000583445	0.01949918
ZNF675	-1.889528	0.549688	-3.437455	0.000587208	0.019543475
JAM2	-2.981331	0.867386	-3.437144	0.000587884	0.019543475
BTBD6	-2.625583	0.764033	-3.43648	0.000589327	0.019543475
ENSG00000269982	-3.708108	1.079723	-3.434314	0.000594056	0.019649669
ENSG00000263826	-4.066479	1.18471	-3.432467	0.000598117	0.019733261
PLAAT4	1.665887	0.485757	3.429463	0.000604778	0.01980767
CCDC163	-3.063407	0.893343	-3.429151	0.000605473	0.01980767
FAXDC2	-2.864108	0.835357	-3.428603	0.000606697	0.01980767
CD1D	2.384216	0.695671	3.427216	0.000609803	0.01980767
NHEJ1	-3.880063	1.132225	-3.426936	0.000610432	0.01980767
VASH1	-3.969134	1.158366	-3.426493	0.000611429	0.01980767
PGAP3	-2.847163	0.831003	-3.426175	0.000612145	0.01980767
MYH10	-3.959201	1.155933	-3.425112	0.000614545	0.01980767
ENSG00000183889	-3.683788	1.075645	-3.424724	0.000615423	0.01980767
CMBL	-4.016329	1.172797	-3.424573	0.000615767	0.01980767
TNNC2	-2.946065	0.860653	-3.423058	0.000619208	0.019860743
PIWIL1	-3.20268	0.935775	-3.42249	0.000620504	0.019860743
NPC2	-0.663923	0.194041	-3.421562	0.000622626	0.019879219
ZNF730	-3.708216	1.084073	-3.420634	0.000624753	0.019897757
MRVI1.AS1	-2.995157	0.876215	-3.41829	0.00063016	0.020020407
ENSG00000270681	-3.792978	1.110082	-3.416844	0.000633516	0.02007745
FAM153B	-3.995798	1.169724	-3.416016	0.000635445	0.020089111
FMNL2	-3.491837	1.023469	-3.411764	0.000645439	0.020350935
PSD3	-2.218121	0.650256	-3.411152	0.00064689	0.020350935
ENSG00000282393	-2.821237	0.827508	-3.409316	0.000651259	0.02039317
SLC15A2	-2.446851	0.717708	-3.409256	0.000651402	0.02039317
TMEM217	-3.673709	1.077866	-3.408317	0.000653648	0.02041381
FES	-2.537902	0.745283	-3.405285	0.000660949	0.020574895
ENSG00000260100	-3.824481	1.123344	-3.404551	0.00066273	0.020574895
ENSG00000264112	-2.881966	0.846593	-3.404191	0.000663603	0.020574895
MALINC1	-3.839129	1.12991	-3.397731	0.000679472	0.021016252
ENSG00000274400	-3.820724	1.124962	-3.396313	0.000683003	0.021074805
SLC35D2	-3.25529	0.958945	-3.394657	0.000687146	0.021151942
SETMAR	-2.859525	0.843026	-3.391977	0.000693904	0.021308967
ZNF20	-2.930228	0.864266	-3.390424	0.000697847	0.021365531
ENSG00000267127	-4.091735	1.207021	-3.389945	0.000699066	0.021365531
C19orf18	-3.769226	1.112979	-3.386611	0.000707617	0.021457456

SYNJ1	-3.828505	1.130762	-3.385773	0.00070978	0.021457456
PRDM11	-2.839465	0.838705	-3.385533	0.000710403	0.021457456
TK1	-2.53955	0.750137	-3.385449	0.000710619	0.021457456
ZNF746	-2.501064	0.738787	-3.385366	0.000710835	0.021457456
ENSG00000259363	-2.961551	0.874934	-3.384885	0.00071208	0.021457456
KCTD12	-3.776352	1.11616	-3.383343	0.000716091	0.021527909
ENSG00000159239	-3.086558	0.912691	-3.381822	0.000720068	0.021549655
WDR7	-2.583635	0.764071	-3.38141	0.000721149	0.021549655
ACTA2	-2.842125	0.840603	-3.381056	0.000722078	0.021549655
ENSG00000249476	-2.700277	0.798778	-3.38051	0.000723514	0.021549655
PAN3.AS1	-3.636753	1.076217	-3.379199	0.000726973	0.021568667
IGKV3.15	-3.551195	1.050961	-3.378998	0.000727505	0.021568667
ENSG00000271806	-3.071796	0.909759	-3.376496	0.000734156	0.021715818
EAF1.AS1	-2.729577	0.808865	-3.374575	0.000739297	0.021817748
LIMS2	-3.526118	1.046932	-3.368048	0.000757025	0.022289276
PTTG1	-2.288439	0.679582	-3.367424	0.00075874	0.022289276
ENSG00000272931	-3.456688	1.027024	-3.365731	0.00076341	0.022304903
SNTA1	-2.819256	0.837734	-3.365337	0.000764503	0.022304903
ENSG00000214265	-2.670897	0.793793	-3.364726	0.000766197	0.022304903
DOLPP1	-2.768039	0.822665	-3.364723	0.000766206	0.022304903
ENSG00000236255	-3.550315	1.055869	-3.362458	0.00077252	0.022415999
ATP2B4	-3.06963	0.913008	-3.362105	0.000773506	0.022415999
LINC01353	-2.637751	0.785238	-3.359175	0.000781755	0.022604126
CHAF1B	-3.847435	1.146343	-3.356269	0.000790016	0.022790989
COL8A1	-3.644123	1.085963	-3.35566	0.00079176	0.022790989
TIGD5	-2.364102	0.705125	-3.352741	0.000800157	0.022899112
ENSG00000271746	-3.33608	0.99508	-3.352576	0.000800633	0.022899112
PITPNM2	-2.415833	0.721296	-3.349295	0.000810176	0.022899112
LINC00924	-3.311481	0.988785	-3.349038	0.000810926	0.022899112
FAM13A.AS1	-2.953625	0.882033	-3.348658	0.00081204	0.022899112
TRAF6	-2.15379	0.643217	-3.348465	0.000812606	0.022899112
TP53I11	-1.71587	0.512513	-3.347953	0.000814107	0.022899112
ZNF775	-2.72437	0.813805	-3.347694	0.000814871	0.022899112
ENSG00000273759	-2.750845	0.821719	-3.347671	0.000814938	0.022899112
CROT	-2.369992	0.707953	-3.347668	0.000814944	0.022899112
ZNF433	-2.673976	0.79877	-3.347618	0.000815092	0.022899112
ENSG00000274561	-2.709768	0.80995	-3.3456	0.000821046	0.023016123
C1QTNF6	-2.877584	0.861073	-3.34186	0.000832191	0.023277832
SMIM2.AS1	-3.733199	1.119089	-3.335926	0.000850158	0.023728809
ZBTB20.AS2	-2.659404	0.797666	-3.333983	0.00085612	0.023843499
DOK7	-3.276738	0.98313	-3.332964	0.000859262	0.023876867
ZNF471	-2.85143	0.855671	-3.332392	0.000861029	0.023876867
ZNF57	-2.590116	0.777846	-3.329858	0.000868903	0.0240434
NACC1	-2.002299	0.601525	-3.328707	0.0008725	0.024091108
ATP6V1C2	-3.005478	0.903347	-3.327048	0.000877712	0.024106047
ENSG00000271789	-3.33043	1.001095	-3.326788	0.000878532	0.024106047
HACD1	-3.692228	1.109861	-3.326747	0.000878661	0.024106047

LILRA2	-3.737501	1.123749	-3.325922	0.000881267	0.024126089
BEX5	-3.131982	0.942567	-3.322821	0.000891122	0.024300639
ENSG00000259327	-3.714848	1.118012	-3.322727	0.00089142	0.024300639
AHI1	2.219974	0.668848	3.319099	0.000903085	0.024510479
LY6G5C	-3.360661	1.012545	-3.319024	0.000903327	0.024510479
ENSG00000236559	-3.424284	1.031985	-3.318154	0.000906144	0.024510479
ST3GAL4	-3.457367	1.042012	-3.317972	0.000906737	0.024510479
ABL1	-2.574678	0.77657	-3.315451	0.000914955	0.024680779
ENSG00000259038	-2.722749	0.82168	-3.313638	0.000920906	0.024703184
MYLK3	-3.605337	1.088054	-3.313566	0.000921145	0.024703184
SPON2	-2.768333	0.835485	-3.313444	0.000921546	0.024703184
ZNF610	-2.879164	0.869843	-3.309982	0.000933019	0.024888387
TRAPPC10	-2.220136	0.670791	-3.309727	0.00093387	0.024888387
SELENOI	-2.558563	0.773071	-3.309611	0.000934257	0.024888387
LGALS4	-3.399472	1.028797	-3.304319	0.000952075	0.025256869
ENSG00000272195	-3.517757	1.064603	-3.30429	0.000952173	0.025256869
FRY.AS1	-2.532258	0.766478	-3.303759	0.000953978	0.025256869
PIGZ	-3.400181	1.029519	-3.302688	0.000957629	0.025262942
RNFT2	-2.692514	0.815377	-3.302171	0.000959394	0.025262942
ACSS2	-2.530886	0.766478	-3.301966	0.000960098	0.025262942
FBXL14	-3.613859	1.094872	-3.300715	0.000964387	0.025324024
STX18.AS1	-4.258193	1.290357	-3.300011	0.000966812	0.025331129
ENSG00000266990	-3.549915	1.075979	-3.299242	0.000969463	0.025331129
SLC12A9.AS1	-3.164448	0.959236	-3.298923	0.000970564	0.025331129
UBAP1L	-2.66989	0.810249	-3.295148	0.000983699	0.025621982
IGHV1.24	-3.220389	0.978022	-3.292759	0.000992095	0.025669699
ARHGEF5	-3.584559	1.08868	-3.292574	0.000992749	0.025669699
CDKL5	-2.783422	0.845575	-3.291749	0.000995664	0.025669699
NR4A3	-3.535366	1.074069	-3.291562	0.000996328	0.025669699
CLEC4A	-2.722081	0.827088	-3.291163	0.000997742	0.025669699
C22orf15	-3.366077	1.022903	-3.290709	0.000999352	0.025669699
SMIM11A	-3.736608	1.135731	-3.290046	0.001001712	0.025669699
ENSG00000272990	-2.86761	0.87161	-3.290014	0.001001822	0.025669699
ENSG00000272086	-2.711337	0.824329	-3.289143	0.001004929	0.025669699
RAB33A	-2.565657	0.78022	-3.288376	0.001007673	0.025669699
IFIT1	-3.433522	1.044264	-3.287983	0.00100908	0.025669699
CCDC13	-3.164749	0.962552	-3.287874	0.001009471	0.025669699
ARHGEF40	-3.092842	0.941074	-3.286502	0.001014402	0.025744192
USP31	-2.702913	0.823181	-3.283497	0.001025276	0.025968954
SLC39A11	-2.678219	0.815911	-3.282487	0.001028958	0.025997893
ENSG00000272871	-3.165814	0.964638	-3.281868	0.001031218	0.025997893
MCU	-2.393401	0.729519	-3.280791	0.001035164	0.025997893
MEIG1	-3.465356	1.056339	-3.280535	0.001036105	0.025997893
NR6A1	-2.923138	0.891086	-3.280421	0.001036521	0.025997893
P2RX5	1.089591	0.332212	3.279807	0.00103878	0.026003849
GTF2IRD1	-2.681898	0.818116	-3.278141	0.001044931	0.026102123
DCUN1D3	-3.286731	1.002893	-3.277251	0.00104823	0.026102123

XXYLT1	-2.878503	0.878383	-3.277046	0.001048994	0.026102123
EID3	-2.422359	0.7394	-3.276114	0.001052459	0.026102123
CDKL1	-2.850522	0.870183	-3.275774	0.001053727	0.026102123
PSMA8	-4.391236	1.340645	-3.275466	0.001054877	0.026102123
BORCS8.MEF2B	-2.859752	0.873441	-3.27412	0.001059916	0.026176478
TXK	-3.680572	1.124453	-3.273211	0.001063331	0.026210501
ENSG00000261542	-3.380166	1.033256	-3.271374	0.001070263	0.026330917
SOCS6	-3.267891	0.999312	-3.270142	0.001074935	0.026395394
ZBTB42	-3.242272	0.991802	-3.269073	0.001079005	0.026419264
FAM72A	-3.295033	1.008023	-3.268809	0.001080013	0.026419264
ZNF710.AS1	-3.222984	0.986362	-3.267549	0.001084832	0.026438442
ENSG00000259146	-4.189593	1.28219	-3.267529	0.001084907	0.026438442
TMEM44.AS1	-2.928627	0.896745	-3.265842	0.00109139	0.026546157
ZNF41	-2.731861	0.836789	-3.264695	0.001095821	0.026603648
MVB12B	-4.582504	1.404302	-3.26319	0.001101658	0.026694979
MARVELD3	-3.289954	1.008383	-3.262604	0.001103936	0.026699885
CD38	2.363815	0.72464	3.262053	0.001106086	0.026701713
TSSK3	-3.5626	1.092368	-3.261353	0.001108817	0.026717518
PCAT1	-3.286347	1.007908	-3.260561	0.001111919	0.026742172
LINC00449	-3.406443	1.045119	-3.259382	0.001116553	0.026803516
C10orf143	-2.803141	0.86017	-3.258821	0.001118762	0.026806526
ENSG00000245522	-2.661117	0.816765	-3.258118	0.001121538	0.026823107
BACE2	-3.014706	0.925471	-3.257482	0.001124055	0.026833426
ENSG00000226853	-3.773671	1.159872	-3.253523	0.001139833	0.027058412
MORF4L2.AS1	-2.591477	0.796558	-3.253343	0.001140557	0.027058412
CNN3	-1.489094	0.457722	-3.253272	0.001140844	0.027058412
MYO1B	-2.758694	0.848056	-3.252964	0.001142081	0.027058412
SLC29A1	-3.017917	0.92788	-3.252488	0.001143994	0.027058412
ENSG00000268746	-3.745637	1.152828	-3.249086	0.001157763	0.027297985
FGD4	-4.428305	1.363001	-3.248938	0.001158366	0.027297985
KLHL25	-4.326892	1.332429	-3.247372	0.001164759	0.027398451
ENSG00000272750	-2.599531	0.800938	-3.245606	0.001172008	0.027442551
ENSG00000273058	-4.317765	1.330368	-3.245541	0.001172277	0.027442551
PART1	-3.420974	1.054113	-3.245358	0.001173032	0.027442551
KIF5C	-2.820185	0.869194	-3.244596	0.001176173	0.027466087
HIST1H2AE	-2.692967	0.830205	-3.243736	0.001179729	0.027484571
TNK1	-3.287398	1.013574	-3.243373	0.001181236	0.027484571
ENSG00000275672	-2.369997	0.730939	-3.242401	0.001185272	0.027528688
HNRNPA1	0.567499	0.175058	3.24177	0.001187899	0.027539982
C3orf67	-3.541704	1.092821	-3.240882	0.001191605	0.02757618
COL6A3	-3.39679	1.048272	-3.24037	0.001193746	0.02757618
ENSG00000269482	-3.891527	1.201297	-3.239439	0.00119765	0.027616771
CTF1	-3.607046	1.114355	-3.236892	0.001208392	0.027755861
PLSCR3	-3.6174	1.117743	-3.236344	0.001210715	0.027755861
STS	-3.334793	1.030563	-3.235895	0.001212618	0.027755861
ENSG00000177699	-4.555651	1.407939	-3.235688	0.001213498	0.027755861
DLGAP3	-3.306057	1.02187	-3.2353	0.00121515	0.027755861

CDK18	-2.725222	0.84243	-3.234954	0.001216624	0.027755861
METTL6	-1.881308	0.581672	-3.23431	0.00121937	0.027769269
ZNF850	-2.825405	0.874339	-3.231475	0.00123153	0.027996646
CYP27A1	-4.813277	1.490325	-3.229682	0.001239278	0.028077135
CENPE	-3.723183	1.152814	-3.229646	0.001239435	0.028077135
ENSG00000240291	-2.687229	0.832199	-3.229073	0.001241924	0.028084065
FRAT1	-3.69321	1.144013	-3.228294	0.001245309	0.028086187
C2CD2L	-2.978174	0.922593	-3.228047	0.001246383	0.028086187
ENSG00000272941	-2.531636	0.784407	-3.227454	0.001248971	0.0280953
ENSG00000273271	-3.192374	0.989627	-3.225837	0.001256048	0.02818437
PLCH2	-3.593441	1.114055	-3.225549	0.001257312	0.02818437
ENSG00000255455	-3.411417	1.057918	-3.224653	0.001261254	0.028195179
ENSG00000239407	-3.713598	1.151719	-3.224395	0.001262391	0.028195179
CRX	-3.204929	0.994154	-3.223775	0.001265129	0.028195179
SMIM10	-3.299909	1.023723	-3.223439	0.001266612	0.028195179
LINC02361	-2.756593	0.8553	-3.222956	0.00126875	0.028195179
SLC49A4	-2.663981	0.827114	-3.220813	0.001278276	0.028322455
SLC9A6	-2.65395	0.824035	-3.220677	0.00127888	0.028322455
ENSG00000260625	-3.462847	1.076133	-3.217861	0.001291504	0.02850521
LIX1L.AS1	-2.668424	0.829257	-3.217848	0.001291563	0.02850521
IPO13	-2.46656	0.76674	-3.216943	0.001295642	0.028510616
ALG9	-2.496164	0.775975	-3.216811	0.001296239	0.028510616
FCSK	-2.655909	0.825894	-3.215798	0.001300825	0.028562661
DNAH14	-3.137733	0.975979	-3.214959	0.00130463	0.028597394
BMPR1A	-2.926079	0.910516	-3.213649	0.001310596	0.028649867
KANK2	-3.56335	1.108884	-3.213456	0.001311477	0.028649867
ASRGL1	-3.943349	1.227324	-3.212964	0.001313729	0.028650424
ZNF577	-2.467969	0.768442	-3.211651	0.001319748	0.028732992
SLC16A13	-2.106497	0.656204	-3.210124	0.001326778	0.028837257
PLCD1	-2.840358	0.884958	-3.209596	0.001329217	0.028841553
ENSG00000273289	-3.330307	1.038058	-3.20821	0.00133564	0.028932111
ENSG00000242282	-2.601608	0.811319	-3.206641	0.001342945	0.028984954
ZBTB8A	-2.561067	0.798731	-3.206422	0.001343967	0.028984954
MBLAC2	-2.581746	0.805338	-3.205791	0.001346917	0.028984954
AMACR	-2.772297	0.864788	-3.205754	0.00134709	0.028984954
ENSG00000271971	-2.8512	0.889725	-3.204584	0.001352577	0.029017126
ARHGEF39	-3.218791	1.004636	-3.203937	0.001355624	0.029017126
SULT1A1	-3.536972	1.103973	-3.203857	0.001355999	0.029017126
ENSG00000184441	-3.5364	1.104054	-3.203104	0.001359549	0.029017126
ENSG00000261353	-3.449319	1.07689	-3.203038	0.001359861	0.029017126
MAP4K3	-2.833783	0.884855	-3.202538	0.001362222	0.029019397
TRAM2.AS1	-2.463629	0.769666	-3.200906	0.001369961	0.029091546
RBFADN	-3.455083	1.079491	-3.20066	0.00137113	0.029091546
OSBPL1A	-2.623251	0.81975	-3.200064	0.001373971	0.029091546
ENSG00000256633	-4.158213	1.299713	-3.199331	0.001377467	0.029091546
ENSG00000228395	-3.324904	1.03948	-3.198623	0.001380858	0.029091546
ENSG00000262222	-2.550457	0.797443	-3.198295	0.001382429	0.029091546

CCDC24	-2.830599	0.885056	-3.198216	0.001382805	0.029091546
BORCS8	-1.598538	0.499851	-3.19803	0.001383697	0.029091546
ENSG00000267698	-3.548699	1.110317	-3.196114	0.001392921	0.029186276
ENSG00000279081	-2.67103	0.835816	-3.195716	0.001394844	0.029186276
PDLIM2	-1.717535	0.537455	-3.195682	0.001395007	0.029186276
SGCB	-2.19169	0.686496	-3.192576	0.001410098	0.029454107
UPB1	-3.188169	0.998839	-3.191876	0.00141352	0.029477742
FNTB	-2.595851	0.813536	-3.190824	0.001418677	0.029526241
AMPD2	-2.146233	0.672746	-3.19026	0.001421448	0.029526241
ENSG00000235978	-3.473085	1.088741	-3.19	0.00142273	0.029526241
SAXO2	-4.182969	1.312479	-3.187076	0.001437192	0.029778347
ENSG00000259959	-3.629273	1.138971	-3.186448	0.001440312	0.029795008
CA3.AS1	-3.729472	1.170773	-3.185477	0.001445154	0.029847179
TBC1D8B	-2.579711	0.810084	-3.184497	0.001450057	0.029849431
TP53INP2	-3.238391	1.016944	-3.184435	0.00145037	0.029849431
PLP2	-0.862914	0.27101	-3.184065	0.001452222	0.029849431
PRR11	-3.241987	1.018503	-3.183089	0.001457127	0.029902472
ZNF438	-2.485999	0.781195	-3.182302	0.001461092	0.029936094
PHKB	2.170914	0.682285	3.18183	0.001463476	0.029937273
HLX	-2.494917	0.784313	-3.181023	0.00146756	0.029937805
CD5	-2.690642	0.845975	-3.180522	0.0014701	0.029937805
NRL	-2.856248	0.898143	-3.180171	0.001471879	0.029937805
ENSG00000261140	-3.534467	1.111472	-3.179989	0.001472809	0.029937805
ACVR2B	-3.402902	1.070314	-3.179349	0.001476061	0.029956586
ENSG00000227896	-3.630343	1.142303	-3.178092	0.001482478	0.029976508
INTS8	-1.576284	0.496052	-3.17766	0.00148469	0.029976508
ENSG00000278740	-3.363382	1.05856	-3.177317	0.001486443	0.029976508
HEATR4	-3.406248	1.072195	-3.176892	0.001488623	0.029976508
XXYLT1.AS2	-2.690836	0.847006	-3.176879	0.001488691	0.029976508
ENSG00000260572	-3.372103	1.061797	-3.175844	0.001494014	0.03003669
TMPRSS13	-3.241814	1.020995	-3.175151	0.001497585	0.030061514
WNT7B	-3.870993	1.219642	-3.173875	0.001504183	0.030070781
FOXP4	-2.109524	0.664685	-3.173719	0.001504992	0.030070781
LINC00526	-2.152924	0.678363	-3.173706	0.001505058	0.030070781
PIP5KL1	-3.75477	1.183354	-3.17299	0.001508775	0.0300983
ABHD1	-3.190382	1.005634	-3.172507	0.001511291	0.030101823
TTLL11	-3.329322	1.050177	-3.170247	0.001523093	0.030290003
ENSG00000254777	-4.287254	1.352559	-3.169735	0.001525783	0.030296677
MTHFD1L	-2.49467	0.78741	-3.168196	0.001533879	0.030335505
SGSM1	-3.643091	1.15007	-3.167714	0.001536428	0.030335505
PSAT1	-3.667438	1.157793	-3.167612	0.001536965	0.030335505
ENSG00000226822	-4.358374	1.376036	-3.167341	0.001538398	0.030335505
ENSG00000175773	-3.843078	1.213427	-3.167128	0.001539526	0.030335505
ENSG00000244733	-4.190713	1.323423	-3.166572	0.001542473	0.030344363
ENSG00000267213	-4.227128	1.335099	-3.166154	0.001544692	0.030344363
HAP1	-4.041583	1.27703	-3.16483	0.001551737	0.030436288
ITPRIPL2	-3.367078	1.064071	-3.164334	0.001554381	0.030441738

CAPS2	-3.395141	1.073109	-3.163836	0.001557045	0.030447568
NRAV	-2.629968	0.831394	-3.163325	0.001559782	0.030454805
ENSG00000250222	-2.641577	0.83535	-3.162239	0.001565609	0.030522259
GGT1	-3.670505	1.16133	-3.160605	0.001574417	0.030551463
C9orf40	-2.408985	0.762364	-3.159888	0.001578297	0.030551463
ENSG00000223969	-3.399637	1.076025	-3.15944	0.001580725	0.030551463
ITIH4	-4.04646	1.280796	-3.159332	0.001581312	0.030551463
ENSG00000273456	-2.643123	0.836609	-3.15933	0.001581321	0.030551463
KNTC1	-2.323535	0.735453	-3.159324	0.001581353	0.030551463
ZMIZ1	-2.254543	0.713894	-3.158091	0.001588062	0.030563108
MAFF	-2.756849	0.873029	-3.157797	0.001589661	0.030563108
SLC5A10	-3.335722	1.056414	-3.157591	0.001590788	0.030563108
UBXN6	-1.140686	0.361266	-3.157468	0.001591457	0.030563108
SYNPO	-3.125293	0.989969	-3.15696	0.001594231	0.030570742
HMMR	-4.092758	1.297084	-3.155354	0.001603036	0.030693844
ENSG00000181097	-3.177751	1.007524	-3.154022	0.001610371	0.030720727
ENSG00000272817	-2.923508	0.926944	-3.15392	0.001610934	0.030720727
FBXL16	-3.857846	1.223238	-3.153799	0.001611603	0.030720727
POLE2	-2.776395	0.880717	-3.152425	0.001619201	0.030819918
MMACHC	-3.264064	1.035651	-3.151702	0.001623217	0.030839888
ENSG00000241764	-2.518323	0.799221	-3.150972	0.001627279	0.030839888
TPH1	-3.935287	1.248923	-3.150943	0.001627441	0.030839888
LMLN	-3.157279	1.002423	-3.149649	0.00163467	0.030931318
ENSG00000248559	-2.144734	0.681303	-3.147989	0.001643977	0.03102509
PROCR	-3.936329	1.25047	-3.147879	0.001644597	0.03102509
USP27X.AS1	-3.765597	1.196386	-3.147477	0.001646859	0.03102509
CAMK4	-4.019583	1.277559	-3.146301	0.001653499	0.031104637
ENSG00000273117	-3.295257	1.047709	-3.145202	0.001659723	0.031165225
ENSG00000256116	-4.110927	1.307182	-3.144877	0.001661564	0.031165225
NKAPL	-2.953062	0.939362	-3.14369	0.00166832	0.031207698
EDN1	-4.042754	1.286016	-3.143627	0.001668679	0.031207698
ENSG00000254452	-3.856862	1.22713	-3.142994	0.001672295	0.031229921
TCAP	-3.288011	1.046289	-3.142546	0.001674853	0.031232358
ENSG00000174171	-3.716082	1.182719	-3.141982	0.001678081	0.031233876
ZNF726	-2.472035	0.786976	-3.141182	0.001682672	0.031233876
LINC01550	-3.387971	1.078623	-3.141014	0.001683638	0.031233876
POGLUT3	-2.880948	0.917254	-3.140839	0.001684644	0.031233876
BAIAP2.DT	-3.796033	1.208905	-3.140058	0.001689142	0.031253258
KIAA0391	-3.810944	1.213782	-3.139728	0.001691046	0.031253258
GSTM3	-3.70896	1.181567	-3.139017	0.001695154	0.031253258
NRSN2	-2.410987	0.768301	-3.138077	0.001700599	0.031253258
LINC01963	-3.854619	1.22839	-3.137945	0.00170137	0.031253258
WNT2B	-2.346928	0.747919	-3.137944	0.001701371	0.031253258
KIF21B	-2.625472	0.836746	-3.137717	0.001702692	0.031253258
ENSG00000229178	-2.979573	0.949854	-3.136874	0.001707593	0.031298565
MAS1	-4.024431	1.283417	-3.135716	0.001714352	0.031366383
SPDYE3	-3.679669	1.174046	-3.134177	0.00172337	0.031366383

STKLD1	-3.581791	1.142841	-3.134111	0.001723756	0.031366383
SLC25A10	-3.116064	0.994386	-3.133656	0.00172643	0.031366383
BHLHE40.AS1	-3.817399	1.218252	-3.133506	0.001727312	0.031366383
USP51	-2.70076	0.86217	-3.132517	0.001733147	0.031366383
SLC22A18	-2.613754	0.834412	-3.132452	0.001733527	0.031366383
ACVR1	-2.57669	0.822606	-3.13235	0.001734132	0.031366383
ENSG00000232347	-3.062643	0.977759	-3.132307	0.001734384	0.031366383
RAB11FIP5	-2.877695	0.918778	-3.132089	0.00173567	0.031366383
NECTIN1	-3.315639	1.059253	-3.130169	0.001747058	0.03144716
FBXO36	-3.697562	1.181515	-3.129509	0.001750986	0.03144716
ENSG00000215022	-3.59168	1.147969	-3.128725	0.001755667	0.03144716
AMBRA1	-2.499262	0.798921	-3.128297	0.001758225	0.03144716
LY9	-1.434643	0.458694	-3.12767	0.001761978	0.03144716
TLE2	-3.853073	1.23213	-3.127165	0.001765007	0.03144716
ANKRD50	-3.848943	1.230835	-3.127099	0.001765407	0.03144716
ENSG00000273199	-2.446917	0.782594	-3.126673	0.001767962	0.03144716
OSM	-3.291731	1.052908	-3.126325	0.001770058	0.03144716
POLN	-3.398434	1.087088	-3.126181	0.001770927	0.03144716
ENSG00000259407	-4.041831	1.292935	-3.12609	0.001771473	0.03144716
C11orf65	-3.886708	1.243541	-3.125516	0.001774933	0.03144716
CRYL1	-2.166704	0.693242	-3.125465	0.001775244	0.03144716
C2orf15	-2.650817	0.848202	-3.125218	0.001776734	0.03144716
ZNF492	-3.117531	0.997604	-3.125018	0.00177794	0.03144716
LINC01806	-4.177491	1.336982	-3.124568	0.001780664	0.03144716
MTF1	-1.636604	0.523834	-3.124278	0.001782419	0.03144716
CORO2A	-2.516519	0.805545	-3.123995	0.001784132	0.03144716
LOR	-3.569483	1.142966	-3.122999	0.001790181	0.031458384
POLR3G	-3.812949	1.221059	-3.122657	0.001792263	0.031458384
ENSG00000250155	-3.375344	1.081448	-3.121133	0.001801567	0.031458384
ZNF300	-2.830044	0.906779	-3.120987	0.001802463	0.031458384
KREMEN1	-3.185174	1.02064	-3.120762	0.001803836	0.031458384
ZGRF1	-2.53478	0.812265	-3.120631	0.001804641	0.031458384
NEURL1	-3.873088	1.241478	-3.119739	0.001810115	0.031458384
SOBP	-4.099371	1.314163	-3.119378	0.001812333	0.031458384
KCNH4	-3.435688	1.101489	-3.11913	0.001813858	0.031458384
KIF9.AS1	-3.558305	1.140872	-3.118934	0.001815063	0.031458384
LINC01521	-3.047839	0.977237	-3.118834	0.001815681	0.031458384
ENSG00000276131	-3.7968	1.217445	-3.118662	0.00181674	0.031458384
C14orf132	-3.863501	1.238913	-3.118459	0.001817994	0.031458384
CNKSR3	-3.474014	1.114074	-3.118296	0.001818997	0.031458384
CACNA1C.AS2	-4.152247	1.332223	-3.11678	0.001828378	0.031553075
TUBB4A	-3.748202	1.20265	-3.116619	0.001829377	0.031553075
ENSG00000275454	-2.594935	0.833471	-3.113407	0.001849405	0.031855823
CHRNA10	-3.528965	1.134522	-3.110531	0.001867515	0.032082555
RBPMS	-3.234352	1.040267	-3.109157	0.001876218	0.032082555
NAP1L5	-2.996347	0.96373	-3.109114	0.001876495	0.032082555
FASTKD5	-1.962821	0.631447	-3.108451	0.001880707	0.032082555

ADAMDEC1	-4.010105	1.290275	-3.107946	0.001883925	0.032082555
WARS2.AS1	-2.817413	0.906541	-3.107871	0.001884404	0.032082555
ENSG00000267309	-3.936455	1.266633	-3.10781	0.001884794	0.032082555
ENSG00000277283	-2.31087	0.743595	-3.107701	0.001885484	0.032082555
RAP1GAP2	-4.194106	1.349956	-3.106847	0.00189094	0.032082555
FAM174B	-2.931478	0.94365	-3.106532	0.001892959	0.032082555
KLHL11	-3.831711	1.233568	-3.106203	0.001895067	0.032082555
MCM4	-2.429717	0.782215	-3.1062	0.001895082	0.032082555
LRRC29	-2.998494	0.965536	-3.105522	0.001899432	0.032082555
DPY19L4	-2.362026	0.760652	-3.105263	0.001901098	0.032082555
ENSG00000260276	-3.507886	1.129703	-3.105139	0.001901896	0.032082555
PYGM	-2.809763	0.905146	-3.104212	0.001907868	0.032082555
ZNF674	-2.933958	0.94518	-3.104125	0.001908428	0.032082555
DRAM1	-1.935519	0.623643	-3.10357	0.00191201	0.032082555
GLS2	-3.189181	1.027678	-3.103289	0.001913826	0.032082555
ENSG00000274315	-3.142751	1.012844	-3.102896	0.001916367	0.032082555
ENSG00000269929	-3.593978	1.158311	-3.102773	0.001917166	0.032082555
PRELID2	-2.527116	0.814481	-3.102733	0.001917423	0.032082555
DISP2	-3.139046	1.011842	-3.102309	0.001920176	0.032086881
VANGL2	-3.354456	1.082078	-3.100014	0.001935116	0.032107808
RPS10P7	-2.548739	0.822216	-3.099841	0.001936242	0.032107808
ENSG00000267226	-3.668338	1.1836	-3.099304	0.001939758	0.032107808
DNM3	-2.846744	0.918532	-3.099232	0.00194023	0.032107808
ANKRD23	-3.359365	1.083993	-3.099064	0.001941328	0.032107808
ZNF440	-2.227863	0.718888	-3.09904	0.001941491	0.032107808
ENSG00000269226	-3.34363	1.079087	-3.098572	0.001944556	0.032107808
ENSG00000276216	-3.326284	1.073567	-3.098347	0.001946036	0.032107808
ENSG00000239791	-3.577288	1.154598	-3.098298	0.001946359	0.032107808
ENSG00000254837	-2.609706	0.842334	-3.098183	0.001947111	0.032107808
OR2A1.AS1	-2.525554	0.81538	-3.097395	0.001952296	0.032107808
ENSG00000268836	-3.784681	1.222023	-3.097063	0.001954485	0.032107808
ERBB2	-2.845371	0.918836	-3.096713	0.001956791	0.032107808
EFCAB7	-2.173704	0.70196	-3.09662	0.001957406	0.032107808
RMC1	-1.646809	0.531846	-3.0964	0.001958858	0.032107808
CISD1	-1.279855	0.413394	-3.095964	0.00196174	0.032114136
NT5DC4	-3.238852	1.046359	-3.095353	0.001965785	0.032139465
PAM16	-3.206018	1.035878	-3.094975	0.001968292	0.032139608
TMEM254.AS1	-3.5232	1.1391	-3.092967	0.00198166	0.032201624
ORC6	-2.310332	0.746965	-3.092959	0.001981713	0.032201624
ENSG00000276791	-3.676214	1.188575	-3.092958	0.001981722	0.032201624
CCDC136	-2.984403	0.96492	-3.092901	0.0019821	0.032201624
ZNF630	-2.856833	0.923869	-3.092248	0.001986466	0.03223185
MAP2K6	-2.905586	0.93999	-3.091083	0.00199428	0.032317886
ITPR1.DT	-3.255172	1.053221	-3.090683	0.001996967	0.032320717
GNA11	-2.844184	0.920514	-3.089779	0.002003056	0.032369376
ZSCAN20	-3.426709	1.109263	-3.089177	0.00200712	0.032369376
DOCK6	-3.105296	1.005237	-3.089118	0.00200752	0.032369376

RIMS3	-3.763639	1.218564	-3.088586	0.002011116	0.032382558
TGM2	-3.343834	1.082795	-3.088152	0.002014054	0.032382558
EEF1AKMT3	-4.048325	1.311036	-3.087882	0.002015888	0.032382558
FSD2	-3.8455	1.245609	-3.087246	0.002020206	0.03241146
ENSG00000237976	-2.236498	0.724784	-3.085746	0.002030425	0.032503669
PCOTH	-3.990069	1.293101	-3.085661	0.002031006	0.032503669
RPA4	-2.852244	0.925416	-3.082119	0.002055324	0.03274388
ENSG00000232698	-3.36851	1.092936	-3.082076	0.002055623	0.03274388
STK4	-0.653587	0.212077	-3.081834	0.002057294	0.03274388
KDM8	-2.48971	0.807878	-3.081791	0.002057595	0.03274388
NPIPB13	-3.212262	1.042513	-3.081268	0.002061208	0.03274388
NPR2	-3.768583	1.223067	-3.081257	0.002061284	0.03274388
SNX15	-2.514764	0.81634	-3.080533	0.002066301	0.032747506
ENSG00000272913	-3.001542	0.974477	-3.080157	0.002068913	0.032747506
MROH8	-3.073152	0.997736	-3.080124	0.002069148	0.032747506
ACSM1	-2.673816	0.868197	-3.079734	0.002071859	0.032750133
ENSG00000260285	-3.83416	1.245254	-3.079018	0.002076838	0.032788557
MIR4453HG	-1.998438	0.649344	-3.077628	0.002086555	0.032801894
TMEM104	-3.029592	0.984552	-3.077126	0.002090068	0.032801894
IGFBP4	-2.658555	0.864004	-3.077018	0.002090825	0.032801894
AP4B1.AS1	-3.010317	0.978352	-3.076925	0.002091476	0.032801894
ENSG00000259071	-3.530101	1.147316	-3.076833	0.002092126	0.032801894
ENSG00000267731	-4.091151	1.329753	-3.076624	0.002093589	0.032801894
TRIP13	-3.031726	0.985495	-3.076348	0.002095528	0.032801894
FSTL3	-2.639375	0.858126	-3.075742	0.002099794	0.032828738
ZNF572	-2.841979	0.924276	-3.074816	0.002106326	0.032890895
CHCHD4	-1.826911	0.594375	-3.073668	0.002114447	0.032977683
ENSG00000236266	-4.84097	1.575424	-3.072805	0.00212057	0.03298794
SCARB1	-3.632663	1.182257	-3.07265	0.002121674	0.03298794
LINC02132	-3.02804	0.985618	-3.072227	0.002124684	0.03298794
KHDRBS3	-3.798935	1.236648	-3.071961	0.002126573	0.03298794
FANCC	-2.918899	0.950233	-3.071772	0.002127923	0.03298794
BAG3	-3.99269	1.300483	-3.07016	0.002139444	0.033126619
ENSG00000254615	-2.43037	0.792029	-3.068534	0.002151115	0.033267307
EIF2S3B	-3.139777	1.02356	-3.067506	0.00215853	0.0333419
ENSG00000234694	-2.853229	0.930286	-3.067047	0.00216185	0.033353149
ENSG00000248544	-2.90612	0.947798	-3.066181	0.002168122	0.033371621
CLBA1	-3.093126	1.008802	-3.066138	0.002168432	0.033371621
ENSG00000270589	-3.778253	1.232485	-3.065557	0.002172649	0.033371621
DYNC2H1	-3.014958	0.98381	-3.064572	0.002179819	0.033371621
LRRC46	-3.828617	1.249503	-3.064111	0.002183177	0.033371621
C3orf14	-2.884141	0.941285	-3.064045	0.002183662	0.033371621
PPM1J	-3.896755	1.271845	-3.063861	0.002185003	0.033371621
CFAP45	-4.123587	1.346085	-3.063392	0.002188429	0.033371621
ENSG00000272323	-2.936333	0.9586	-3.063147	0.002190224	0.033371621
DACT1	-3.872347	1.264179	-3.063131	0.00219034	0.033371621
ENSG00000270091	-3.124602	1.020228	-3.062651	0.002193855	0.033371621

PPIAL4G	-2.412978	0.787936	-3.062402	0.002195687	0.033371621
RNLS	-3.000897	0.980012	-3.062103	0.00219788	0.033371621
ENSG00000253704	-3.942453	1.287583	-3.061902	0.002199358	0.033371621
LINC00525	-4.041583	1.320233	-3.061265	0.002204038	0.033403254
ENSG00000275418	-3.99256	1.304918	-3.059624	0.002216148	0.033459292
ASB16	-3.98089	1.301131	-3.059561	0.002216615	0.033459292
ENSG00000267281	-3.805913	1.244013	-3.059384	0.002217926	0.033459292
ENSG00000232748	-3.44328	1.125492	-3.059356	0.002218138	0.033459292
ANKS6	-3.316957	1.084596	-3.058243	0.002226388	0.033538695
SLC38A11	-3.392451	1.109443	-3.057796	0.002229717	0.033538695
ENSG00000250541	-3.977212	1.300794	-3.057526	0.002231723	0.033538695
LINC00863	-2.994742	0.97956	-3.057232	0.002233909	0.033538695
NSMCE1.DT	-3.0141	0.986017	-3.056842	0.002236822	0.033538695
ENSG00000237596	-3.100497	1.014514	-3.056139	0.002242071	0.033538695
LAT	-2.535317	0.829618	-3.056006	0.002243064	0.033538695
LRRC37A3	-3.11424	1.019109	-3.055847	0.002244254	0.033538695
TMEM9B.AS1	-2.958333	0.968531	-3.054454	0.002254705	0.033655784
FUT4	-3.818363	1.25058	-3.053275	0.002263586	0.033684844
PIF1	-4.067583	1.332237	-3.053197	0.002264174	0.033684844
USP18	-3.092165	1.012778	-3.053153	0.002264505	0.033684844
FOXRED2	-2.500383	0.819112	-3.052554	0.002269029	0.033713153
LINC01569	-2.944353	0.964902	-3.051452	0.002277373	0.033792458
ZNF490	-3.158572	1.035242	-3.051047	0.002280449	0.033792458
PET117	-1.552173	0.508858	-3.050306	0.002286084	0.033792458
ENSG00000236723	-3.539753	1.160491	-3.050219	0.002286746	0.033792458
LSINCT5	-3.162202	1.036747	-3.05012	0.002287498	0.033792458
ENSG00000269514	-2.805407	0.920125	-3.048941	0.002296492	0.033826937
ENSG00000259536	-3.711993	1.217513	-3.048832	0.002297333	0.033826937
ENSG00000278831	-4.898547	1.606956	-3.048338	0.002301107	0.033826937
TMEM237	-2.83857	0.931261	-3.048093	0.002302983	0.033826937
TMEM170B	-3.107723	1.019637	-3.047872	0.002304682	0.033826937
PEX12	-2.92952	0.961328	-3.047366	0.002308564	0.033826937
ENSG00000274828	-2.982916	0.97891	-3.04718	0.002309993	0.033826937
RABL2A	-1.717889	0.563785	-3.047067	0.002310863	0.033826937
FAM229A	-2.250589	0.738792	-3.046311	0.002316682	0.03387357
POLR2D	-1.38385	0.454533	-3.044555	0.002330249	0.034002458
TPM2	-3.96528	1.30265	-3.04401	0.002334473	0.034002458
DUSP2	-2.86773	0.942118	-3.043918	0.002335189	0.034002458
TBX6	-2.518735	0.827559	-3.043572	0.002337875	0.034002458
KIAA1324	-3.243305	1.065662	-3.043465	0.002338709	0.034002458
PANX1	-2.698995	0.887107	-3.042468	0.002346469	0.034018674
TSSK6	-4.492856	1.47679	-3.042313	0.002347676	0.034018674
HSD17B1	-2.421345	0.796033	-3.041765	0.002351953	0.034018674
ENSG00000273576	-2.647559	0.870442	-3.041627	0.002353029	0.034018674
ENSG00000275464	-2.38512	0.784268	-3.041207	0.002356322	0.034018674
RCAN1	-4.080121	1.341622	-3.041185	0.002356491	0.034018674
ENSG00000272909	-3.95136	1.299384	-3.04095	0.002358332	0.034018674

HESX1	-3.345385	1.100552	-3.039734	0.002367869	0.034106513
ENSG00000273619	-3.397631	1.117826	-3.039499	0.002369723	0.034106513
ENSG00000272444	-4.357968	1.434115	-3.038787	0.002375331	0.034127594
ENSG00000268129	-3.087355	1.016156	-3.038269	0.002379411	0.034127594
IGLV6.57	-2.977575	0.980156	-3.037857	0.002382671	0.034127594
TPST1	-2.338146	0.769695	-3.037757	0.002383461	0.034127594
ENSG00000267834	-3.828061	1.260235	-3.037577	0.002384881	0.034127594
WIPI1	-2.089354	0.687933	-3.037148	0.002388285	0.034127594
WFS1	-3.05988	1.007546	-3.036962	0.002389754	0.034127594
ENSG00000250132	-2.944129	0.96955	-3.036592	0.002392691	0.034131662
ENSG00000235119	-2.488936	0.820046	-3.035119	0.002404412	0.034137536
ENSG00000212939	-2.135478	0.703629	-3.03495	0.002405755	0.034137536
ENSG00000090932	-3.295496	1.085884	-3.034851	0.002406542	0.034137536
FYCO1	-2.421996	0.798073	-3.034806	0.002406907	0.034137536
DOCK9.DT	-3.223791	1.062288	-3.034763	0.002407247	0.034137536
ZHX1.C8orf76	-2.285563	0.753332	-3.033938	0.002413837	0.034137536
ATP6V1F	-0.690871	0.227716	-3.033912	0.002414048	0.034137536
KCNMB1	-3.220598	1.061742	-3.033315	0.002418827	0.034137536
ENSG00000275807	-3.920791	1.292615	-3.033224	0.002419559	0.034137536
ZNF575	-2.870155	0.946242	-3.033215	0.002419634	0.034137536
ZNF624	-2.646696	0.872799	-3.032423	0.002425993	0.034146257
CDCA8	-3.032904	1.000303	-3.031986	0.002429502	0.034146257
POPDC2	-3.119738	1.028969	-3.031907	0.002430142	0.034146257
TRIP6	-1.847748	0.609452	-3.031817	0.002430867	0.034146257
ZWINT	-3.105726	1.024519	-3.031399	0.00243423	0.034156208
FAM81A	-3.161264	1.043332	-3.029969	0.002445785	0.034213544
P2RX7	-2.601473	0.858648	-3.029732	0.002447708	0.034213544
VN1R1	-2.55753	0.844215	-3.029477	0.002449777	0.034213544
TMEM181	-2.208907	0.729244	-3.029039	0.002453333	0.034213544
ENSG00000223653	-2.783425	0.919012	-3.028714	0.002455973	0.034213544
ENSG00000272367	-2.998019	0.989999	-3.028304	0.002459306	0.034213544
SSX2IP	-2.296127	0.758253	-3.028183	0.002460293	0.034213544
ENSG00000256325	-2.913154	0.962272	-3.027369	0.002466925	0.034213544
CEACAM21	-1.966064	0.649463	-3.027214	0.002468189	0.034213544
ENSG00000229127	-3.023658	0.998928	-3.026902	0.002470738	0.034213544
ENSG00000257120	-4.03744	1.333872	-3.026857	0.002471111	0.034213544
SLC29A3	-2.576617	0.851284	-3.026741	0.002472059	0.034213544
PTOV1.AS1	-4.126736	1.363494	-3.026589	0.002473302	0.034213544
CHRNA6	-4.060021	1.341573	-3.026315	0.002475543	0.034213544
C3orf52	-2.878639	0.951541	-3.025238	0.002484373	0.034298736
ITPK1.AS1	-3.323471	1.098832	-3.024549	0.002490042	0.034321507
C15orf48	-4.601991	1.521626	-3.024389	0.002491357	0.034321507
MEIOC	-3.823414	1.264516	-3.023619	0.002497709	0.034372211
KIAA0825	-3.638561	1.203607	-3.023048	0.002502425	0.034400325
SSPN	-3.179614	1.052162	-3.021982	0.002511258	0.034482592
HEXA.AS1	-3.823935	1.2655	-3.021679	0.002513769	0.034482592
GAS8	-2.976352	0.985468	-3.020242	0.002525732	0.034609789

ENSG00000269910	-4.028183	1.334207	-3.019159	0.002534775	0.034685768
ENSG00000183308	-3.872785	1.282832	-3.018933	0.002536668	0.034685768
NDST1	-4.07053	1.348577	-3.01839	0.002541215	0.034686125
SLFN12L	-3.800147	1.259041	-3.018286	0.002542086	0.034686125
MYO15A	-2.980818	0.987828	-3.017548	0.002548284	0.034689643
LINC01483	-4.678217	1.550416	-3.017396	0.002549567	0.034689643
ENSG00000226644	-3.037312	1.006826	-3.016719	0.002555267	0.034689643
INTS2	-2.10077	0.696446	-3.016414	0.002557836	0.034689643
FANCE	-2.925198	0.969888	-3.016015	0.002561202	0.034689643
PFKFB3	-1.188392	0.394039	-3.015923	0.002561986	0.034689643
THRB	-3.344608	1.10906	-3.015715	0.002563737	0.034689643
PTMS	-2.483856	0.823741	-3.015337	0.002566941	0.034689643
CTRL	-3.632205	1.204793	-3.014797	0.002571514	0.034689643
IRAK3	-2.179738	0.723041	-3.014681	0.002572498	0.034689643
HSF5	-3.318075	1.100664	-3.014612	0.002573085	0.034689643
ENSG00000272991	-3.287434	1.090569	-3.014422	0.002574696	0.034689643
ENSG00000272892	-3.312795	1.099272	-3.013626	0.002581459	0.03472139
POC1A	-3.013142	0.999878	-3.01351	0.002582449	0.03472139
ENSG00000260806	-2.843631	0.943972	-3.01241	0.002591822	0.034721656
ENSG00000272156	-2.830806	0.93978	-3.0122	0.002593619	0.034721656
ENSG00000185065	-3.49036	1.158848	-3.011922	0.002595991	0.034721656
RASA4B	-4.310749	1.43125	-3.011876	0.002596383	0.034721656
C5orf22	-2.196173	0.729172	-3.011874	0.002596401	0.034721656
C1orf54	-3.374439	1.120476	-3.01161	0.00259866	0.034721656
AIF1	-3.522893	1.169926	-3.011209	0.002602093	0.03473147
FAN1	-2.719036	0.903328	-3.010021	0.002612296	0.03483152
FBXL6	-2.945123	0.978654	-3.009361	0.002617977	0.034871132
ENSG00000228192	-4.342067	1.443429	-3.008162	0.00262833	0.034919084
LINC01993	-4.342067	1.443429	-3.008162	0.00262833	0.034919084
RAPGEF5	-2.847743	0.946819	-3.007697	0.002632357	0.034919084
RNF227	-2.525975	0.839881	-3.007539	0.002633727	0.034919084
HOXB4	-2.981695	0.991507	-3.007235	0.002636356	0.034919084
TMEM177	-2.201386	0.732072	-3.007062	0.00263786	0.034919084
ENSG00000271427	-4.237872	1.409577	-3.006486	0.002642865	0.034919201
MAP3K21	-3.289112	1.094027	-3.006427	0.002643379	0.034919201
ENSG00000273080	-2.755538	0.916641	-3.006124	0.002646011	0.034919201
KCNMB4	-3.166886	1.054032	-3.004545	0.002659787	0.034974707
SIRT1	-1.85747	0.618241	-3.004443	0.002660677	0.034974707
ENSG00000267152	-2.347241	0.781334	-3.004144	0.002663293	0.034974707
IGLV1.40	-3.283977	1.093174	-3.004074	0.002663906	0.034974707
ENSG00000269051	-3.036338	1.010768	-3.003993	0.002664616	0.034974707
RAMP2.AS1	-3.668311	1.221234	-3.003775	0.002666526	0.034974707
ENSG00000253200	-3.356187	1.117599	-3.003032	0.00267304	0.034986772
IGLV3.1	-2.885423	0.960853	-3.00298	0.002673497	0.034986772
ENSG00000255337	-3.050844	1.016234	-3.002107	0.00268118	0.034986772
ENSG00000257410	-3.975245	1.324179	-3.002046	0.002681717	0.034986772
ENSG00000261335	-3.909571	1.302626	-3.001301	0.002688288	0.034986772

ENSG00000267751	-3.04263	1.013795	-3.001227	0.00268894	0.034986772
KCNJ14	-3.605471	1.201354	-3.001172	0.002689428	0.034986772
ATAD3C	-3.800685	1.266579	-3.000747	0.002693182	0.034986772
LINC01358	-2.4793	0.826295	-3.000503	0.00269534	0.034986772
C1QTNF12	-3.938972	1.312919	-3.000163	0.002698354	0.034986772
AURKA	-2.850153	0.950185	-2.999578	0.002703536	0.034986772
ZNF385D	-3.483503	1.161359	-2.999504	0.002704194	0.034986772
PPARGC1B	-3.659979	1.220214	-2.999456	0.002704624	0.034986772
NAV2.AS3	-3.091292	1.030652	-2.999356	0.002705513	0.034986772
ENSG00000250790	-3.173941	1.058404	-2.998799	0.002710457	0.034987603
RACGAP1	-2.262577	0.75451	-2.998737	0.002711016	0.034987603
ENSG00000272654	-3.072691	1.024812	-2.998298	0.002714924	0.035002938
CRB2	-2.404916	0.802281	-2.997598	0.002721161	0.03504292
ENSG00000267563	-2.300195	0.767412	-2.997339	0.002723472	0.03504292
ZNF449	-2.68489	0.895982	-2.996591	0.00273017	0.035094
DENND1A	-2.300623	0.768028	-2.995493	0.002740017	0.035153878
TMEM8B	-3.15676	1.053847	-2.995462	0.002740292	0.035153878
CD3D	-4.955616	1.654626	-2.995007	0.002744383	0.035171295
ENSG00000267787	-2.646053	0.88382	-2.993883	0.002754519	0.035222066
C4orf46	-1.997228	0.667241	-2.993263	0.002760121	0.035222066
PROC	-3.036805	1.014743	-2.992683	0.002765367	0.035222066
ENSG00000266677	-3.322771	1.110348	-2.99255	0.002766578	0.035222066
ENSG00000266844	-3.987123	1.332356	-2.992537	0.002766694	0.035222066
ENSG00000277368	-3.128045	1.045361	-2.99231	0.002768747	0.035222066
SIRT4	-3.506181	1.171734	-2.992301	0.002768828	0.035222066
SDC3	-3.144964	1.051073	-2.992145	0.002770244	0.035222066
CAHM	-3.054714	1.021038	-2.991772	0.002773629	0.035230294
ARMCX4	-2.478977	0.828753	-2.991212	0.002778728	0.035231687
FILIP1	-2.911379	0.973418	-2.990882	0.002781726	0.035231687
ENSG00000261845	-2.964102	0.991054	-2.990857	0.002781953	0.035231687
RHOBTB1	-3.218499	1.076391	-2.990083	0.002789012	0.035286355
DLG3	-3.31639	1.109345	-2.989503	0.002794317	0.035318735
LY6G5B	-3.801077	1.271935	-2.988421	0.002804226	0.035409202
ZNF229	-2.695862	0.902282	-2.987825	0.002809706	0.035443618
DNHD1	-2.202949	0.737651	-2.986436	0.002822502	0.035570165
WDR11.AS1	-3.279239	1.098311	-2.98571	0.002829207	0.035619768
ENSG00000232063	-3.259212	1.092182	-2.984129	0.002843866	0.035769329
TLCD2	-2.803588	0.939887	-2.982898	0.002855331	0.035834737
PLEKHG2	-2.514286	0.843159	-2.981984	0.002863871	0.035834737
CTBP1.AS	-2.77808	0.931697	-2.981741	0.002866143	0.035834737
CRYM.AS1	-3.786915	1.270296	-2.981129	0.00287188	0.035834737
LCTL	-3.071212	1.030227	-2.981102	0.002872128	0.035834737
TNRC18	-2.222693	0.745611	-2.981036	0.00287275	0.035834737
ENSG00000253891	-2.792056	0.936665	-2.980849	0.002874502	0.035834737
GK	-2.084975	0.699463	-2.980823	0.002874752	0.035834737
ENSG00000272853	-2.799372	0.939249	-2.980436	0.002878384	0.035834737
ARHGAP11A	-2.663911	0.893834	-2.980318	0.002879489	0.035834737

ENSG00000253106	-3.334555	1.118948	-2.98008	0.002881728	0.035834737
IGHV4.39	-3.484764	1.169497	-2.979712	0.002885194	0.035834737
SRC	-2.964304	0.994832	-2.979704	0.002885271	0.035834737
RILPL1	-3.669064	1.231522	-2.979293	0.002889143	0.035848223
HIC2	-2.763153	0.927701	-2.978494	0.002896683	0.035858308
EIF4E3	-2.591234	0.869984	-2.978483	0.002896788	0.035858308
MCOLN2	-3.219587	1.081105	-2.978051	0.002900875	0.035858308
C11orf21	-2.931049	0.984242	-2.977975	0.002901594	0.035858308
MTFR2	-3.197322	1.073744	-2.977733	0.00290389	0.035858308
EGLN3	-3.892164	1.307252	-2.977362	0.002907402	0.03586725
PRICKLE3	-2.461964	0.827107	-2.976597	0.002914673	0.03592251
C8orf89	-3.840097	1.290374	-2.975957	0.002920755	0.035949917
DPCD	-2.202294	0.740074	-2.975776	0.002922485	0.035949917
GTF2H4	-3.452042	1.160189	-2.975413	0.002925941	0.035958049
BCL2L2	-2.312492	0.777281	-2.975103	0.002928901	0.035960087
ENSG00000260526	-3.077799	1.034838	-2.974185	0.002937677	0.036021409
POLR3B	-2.94225	0.989326	-2.973995	0.002939495	0.036021409
MAMLD1	-5.352021	1.800298	-2.972853	0.002950457	0.036097567
ENSG00000271122	-2.412908	0.811672	-2.972763	0.00295132	0.036097567
ZNF75D	-2.195433	0.738765	-2.971759	0.002960992	0.036174168
EPHB6	-1.555778	0.523561	-2.971529	0.002963206	0.036174168
CPS1	-4.485172	1.510104	-2.970107	0.002976956	0.036254856
SLC26A1	-3.508082	1.181156	-2.970042	0.002977591	0.036254856
ZNF484	-2.386594	0.803574	-2.969972	0.002978269	0.036254856
LEKR1	-2.782457	0.937049	-2.969381	0.002984002	0.03629032
MYH3	-2.339352	0.788042	-2.968561	0.002991974	0.036299993
RLN2	-2.682312	0.903648	-2.968316	0.00299436	0.036299993
SPAG8	-2.841574	0.957411	-2.967976	0.002997681	0.036299993
LETM2	-3.737732	1.25939	-2.967892	0.002998498	0.036299993
MPPED2	-3.139562	1.057857	-2.96785	0.002998904	0.036299993
ENSG00000272588	-3.05771	1.030503	-2.9672	0.003005249	0.036342615
SIAE	-2.68965	0.906655	-2.966564	0.003011477	0.036352275
TRIM72	-3.838548	1.293947	-2.966541	0.003011699	0.036352275
DCBLD1	-2.759904	0.930996	-2.964465	0.003032096	0.036564177
GLCE	-2.339167	0.789344	-2.963433	0.003042286	0.036652714
TK2	-2.649941	0.894365	-2.96293	0.003047263	0.036670758
CSTF3.DT	-3.585728	1.210288	-2.962705	0.003049484	0.036670758
TIGD2	-2.35873	0.796326	-2.962018	0.003056303	0.036718447
ACVR2A	-3.237249	1.093378	-2.960777	0.003068643	0.036832309
MTCL1	-3.714461	1.2551	-2.959495	0.003081439	0.036951417
MYBL1	-2.999162	1.013578	-2.958984	0.003086546	0.036978206
FSD1L	-1.925351	0.650817	-2.958358	0.003092822	0.036997835
ENSG00000105650	-3.279828	1.10879	-2.958026	0.003096164	0.036997835
YPEL4	-3.508084	1.18598	-2.957961	0.003096811	0.036997835
ENSG00000269604	-3.476796	1.175556	-2.957576	0.00310068	0.037009699
ENSG00000225302	-3.915325	1.324789	-2.955432	0.003122319	0.03714677
LINC01176	-3.123082	1.056837	-2.955122	0.003125459	0.03714677

TTLL4	-2.211565	0.748384	-2.95512	0.003125471	0.03714677
PGAP1	-2.972258	1.005817	-2.955068	0.003125996	0.03714677
PLAUR	-3.001448	1.015715	-2.955009	0.003126599	0.03714677
ENSG00000282034	-3.908283	1.323032	-2.954034	0.003136494	0.037211659
TESMIN	-2.707492	0.916582	-2.953901	0.003137845	0.037211659
IFT140	-3.467099	1.17394	-2.953388	0.003143067	0.03723927
ARG2	-3.764207	1.274737	-2.952929	0.003147743	0.037260362
LINC01725	-4.61642	1.564084	-2.951516	0.003162181	0.037324174
FRG1.DT	-4.633825	1.570056	-2.951375	0.003163628	0.037324174
ZNF182	-2.125676	0.720287	-2.951152	0.003165911	0.037324174
CACNB3	-2.752722	0.932763	-2.951149	0.003165943	0.037324174
FCMR	1.142231	0.387081	2.950887	0.003168628	0.037324174
ENSG00000267416	-2.92336	0.990734	-2.950701	0.003170539	0.037324174
CDKN3	-3.580399	1.213684	-2.950025	0.003177487	0.037360445
ENSG00000273674	-2.995359	1.015433	-2.949836	0.003179427	0.037360445
ENSG00000251867	-3.303905	1.120432	-2.948776	0.003190348	0.037454569
DMPK	-3.2253	1.094028	-2.948096	0.003197374	0.037472806
L1CAM	-2.343288	0.794857	-2.948062	0.003197726	0.037472806
CYP4V2	-2.205987	0.748529	-2.947095	0.003207747	0.037536789
LRRC37A	-3.688137	1.251634	-2.946656	0.003212299	0.037536789
ENSG00000245213	-3.734406	1.267525	-2.946218	0.003216858	0.037536789
ENSG00000276564	-3.338727	1.133229	-2.946206	0.003216982	0.037536789
TBXAS1	-2.681434	0.910155	-2.94613	0.003217772	0.037536789
ZNF132	-3.560202	1.208597	-2.945732	0.003221914	0.037551053
AVEN	-3.126669	1.061637	-2.94514	0.003228083	0.037588907
PTPN20	-2.946891	1.000716	-2.944782	0.00323182	0.037598398
CAB39L	-2.32987	0.791275	-2.944449	0.003235303	0.037604923
DNTTIP1	-1.405781	0.477512	-2.943969	0.00324033	0.037629353
C1orf74	-2.479207	0.842222	-2.94365	0.003243662	0.037634079
AFF2	-3.344044	1.13641	-2.942638	0.003254287	0.037704614
ENSG00000277022	-2.691259	0.914628	-2.942464	0.003256121	0.037704614
CHIC1	-2.191394	0.744889	-2.941907	0.003261974	0.037704614
PRDM15	-2.394582	0.814007	-2.941723	0.003263917	0.037704614
TRABD	1.073856	0.365049	2.941678	0.003264393	0.037704614
ENSG00000273002	-2.45326	0.834393	-2.940172	0.003280299	0.037809039
ECT2	-3.773141	1.283485	-2.939762	0.003284646	0.037809039
ENSG00000224738	-3.638941	1.237841	-2.939748	0.003284789	0.037809039
RNF212	-2.388044	0.81234	-2.939711	0.003285187	0.037809039
RUSC1.AS1	-3.457847	1.176474	-2.939161	0.003291019	0.037842306
CLEC18A	-3.714838	1.264163	-2.938576	0.003297242	0.037876742
TNFRSF21	-4.451572	1.515138	-2.938064	0.003302686	0.037876742
MAPRE3	-2.310943	0.786557	-2.938049	0.003302845	0.037876742
EPHA4	-4.864721	1.656583	-2.936599	0.003318329	0.037984286
CDC7	-2.740319	0.933174	-2.936557	0.00331878	0.037984286
BNIPL	-3.525742	1.200726	-2.936342	0.003321079	0.037984286
GALC	-2.366264	0.806064	-2.93558	0.003329247	0.038043894
STXBP5.AS1	-2.799702	0.953871	-2.935095	0.003334459	0.038069635

RNF130	-1.347366	0.459116	-2.934693	0.003338774	0.038085109
CLCN4	-2.931707	0.999336	-2.933656	0.003349951	0.038178761
GSDMB	-2.300381	0.784286	-2.933091	0.003356058	0.038214512
SLC27A4	-2.31103	0.788044	-2.932617	0.003361187	0.038236497
SCN4A	-3.729693	1.271912	-2.932351	0.003364059	0.038236497
RBMS2	-2.961725	1.010107	-2.932089	0.003366904	0.038236497
ELF1	-0.864411	0.295015	-2.930064	0.003388921	0.038449683
CCDC171	-2.750175	0.938686	-2.929814	0.003391652	0.038449683
CSRNP2	-2.350635	0.802622	-2.928693	0.003403901	0.038494192
ENSG00000268093	-3.616131	1.234794	-2.928529	0.003405705	0.038494192
ALDH7A1	-3.12629	1.067584	-2.928379	0.00340734	0.038494192
RAD54B	-4.089859	1.396638	-2.928361	0.003407545	0.038494192
ENSG00000205682	-2.777672	0.948709	-2.927845	0.003413207	0.038524328
PIK3CD.AS2	-2.418133	0.826379	-2.926178	0.00343154	0.038612467
IGKV4.1	-2.434902	0.832122	-2.926136	0.003432009	0.038612467
SLC7A5	-2.698473	0.92226	-2.925936	0.003434213	0.038612467
PACRGL	-2.419573	0.82697	-2.925829	0.003435397	0.038612467
NETO2	-2.696745	0.921834	-2.925413	0.003439989	0.038612467
ADAM15	-2.267629	0.775182	-2.925286	0.0034414	0.038612467
PRKCI	-2.096032	0.716578	-2.925058	0.00344392	0.038612467
ENSG00000251143	-3.053383	1.043907	-2.924958	0.003445023	0.038612467
LINC02447	-3.206431	1.096409	-2.924485	0.003450269	0.038637603
NEXN	-2.261227	0.773504	-2.923353	0.003462833	0.038743691
CRACR2A	-2.398496	0.820557	-2.923012	0.003466637	0.038743691
SENP8	-3.409159	1.166394	-2.92282	0.003468775	0.038743691
TMEM182	-3.111455	1.064826	-2.922032	0.003477555	0.03878997
LINC01134	-3.126584	1.070051	-2.921901	0.003479023	0.03878997
HASPIN	-3.438048	1.176915	-2.921238	0.003486435	0.03878997
RGS17	-3.5555	1.21714	-2.921192	0.003486951	0.03878997
GRTP1	-3.776923	1.29298	-2.921099	0.003487992	0.03878997
ZRANB3	-1.937428	0.663446	-2.920248	0.003497529	0.038842482
ENSG00000270696	-3.636587	1.245412	-2.919988	0.003500452	0.038842482
POT1.AS1	-3.056766	1.046889	-2.919856	0.003501933	0.038842482
ASF1B	-3.707177	1.269766	-2.919576	0.003505083	0.038842482
ENSG00000273702	-2.092369	0.716728	-2.919333	0.003507808	0.038842482
ENSG00000234773	-3.811772	1.305836	-2.919028	0.003511244	0.038845766
ENSG00000272821	-3.078969	1.054933	-2.91864	0.003515623	0.038845766
ZNF443	-2.650195	0.908118	-2.918337	0.00351904	0.038845766
ENSG00000224220	-2.922921	1.001606	-2.918236	0.003520181	0.038845766
ENSG00000231105	-3.718442	1.274735	-2.917031	0.003533803	0.038933021
ENSG00000264207	-2.956751	1.013645	-2.916949	0.003534736	0.038933021
ENSG00000262621	-2.642677	0.906108	-2.916514	0.003539665	0.038933021
LSM14B	-2.261436	0.775546	-2.915928	0.003546324	0.038933021
CECR7	-3.675091	1.260439	-2.915724	0.003548642	0.038933021
ENSG00000250685	-3.716372	1.274633	-2.91564	0.003549603	0.038933021
ENSG00000231760	-3.617099	1.240627	-2.915542	0.003550715	0.038933021
LINC00106	-3.443407	1.181108	-2.915403	0.003552294	0.038933021

SAG	-4.721453	1.619804	-2.91483	0.003558822	0.038971369
TRIM61	-2.269379	0.778722	-2.914237	0.003565593	0.038997506
TAT.AS1	-2.89737	0.994262	-2.91409	0.00356727	0.038997506
DNAJC9.AS1	-4.507503	1.546981	-2.913741	0.003571258	0.039007959
PINLYP	-3.089244	1.06059	-2.91276	0.003582496	0.039097522
ARHGAP33	-3.618574	1.242922	-2.911343	0.003598785	0.039213622
ALDH1B1	-2.486865	0.854287	-2.911041	0.003602264	0.039213622
ST6GALNAC3	-2.561265	0.879845	-2.91104	0.003602277	0.039213622
SUV39H2	-2.945155	1.01196	-2.910346	0.003610286	0.039267582
PRKCA	-3.595608	1.236048	-2.908955	0.003626395	0.039409478
ENSG00000262580	-2.437776	0.838259	-2.908142	0.003635834	0.039468603
GNG3	-3.599	1.237764	-2.907662	0.003641422	0.039468603
LENG9	-4.153177	1.428685	-2.906992	0.003649227	0.039468603
BANK1	-0.806032	0.277281	-2.90691	0.003650181	0.039468603
ENSG00000235381	-3.288509	1.131354	-2.906703	0.003652598	0.039468603
SH2D4A	-2.732318	0.940007	-2.906699	0.003652649	0.039468603
LINC01504	-3.639828	1.252245	-2.906642	0.003653307	0.039468603
TNFRSF25	-4.651552	1.600639	-2.90606	0.00366011	0.03950892
ENSG00000272172	-2.52508	0.869032	-2.905623	0.003665227	0.039530995
ENSG00000166928	-4.281521	1.474154	-2.904392	0.003679673	0.039613671
MATN1.AS1	-2.338528	0.805187	-2.904327	0.003680432	0.039613671
FMO4	-2.9984	1.032526	-2.903945	0.003684925	0.039613671
ARHGAP10	-4.137533	1.424809	-2.903921	0.003685207	0.039613671
XKR6	-2.37175	0.816992	-2.903028	0.003695734	0.039627561
ENSG00000232098	-3.773252	1.299933	-2.902652	0.003700173	0.039627561
CRY2	-1.915512	0.659924	-2.902625	0.00370049	0.039627561
PRC1	-3.417957	1.177627	-2.90241	0.003703032	0.039627561
CENPS.CORT	-3.884914	1.338519	-2.902397	0.00370319	0.039627561
EXT1	-4.560734	1.571546	-2.902068	0.003707076	0.039627561
ENSG00000235501	-3.223729	1.11087	-2.901985	0.003708058	0.039627561
PANX2	-2.700354	0.93096	-2.900612	0.003724351	0.039768653
ENSG00000268744	-2.855842	0.984877	-2.899695	0.003735265	0.039821029
ENSG00000233757	-3.761668	1.297589	-2.898968	0.003743929	0.039821029
HSD17B14	-2.644594	0.912264	-2.898934	0.00374434	0.039821029
ACY1	-3.28984	1.135033	-2.898453	0.003750081	0.039821029
ENSG00000254428	-3.260545	1.124933	-2.898434	0.003750317	0.039821029
CDK5R1	-4.721173	1.628913	-2.898357	0.003751228	0.039821029
ARF5	-0.832199	0.287143	-2.8982	0.003753112	0.039821029
ENSG00000165521	-4.284618	1.478446	-2.898054	0.003754859	0.039821029
CDK10	-1.505317	0.519529	-2.897465	0.00376192	0.039821029
ENSG00000214797	-2.91663	1.006629	-2.897423	0.003762421	0.039821029
FKBP7	-2.840227	0.980292	-2.897328	0.003763559	0.039821029
ATPSCKMT	-2.157937	0.744926	-2.896849	0.003769306	0.039821029
SGCA	-3.494988	1.206537	-2.896709	0.003770988	0.039821029
PRDX1	-0.754443	0.26046	-2.896577	0.003772584	0.039821029
CCDC180	-2.315914	0.799672	-2.896081	0.003778548	0.039838858
RPL34.AS1	-2.941744	1.015823	-2.895922	0.003780465	0.039838858

ADGRE2	-2.665403	0.92056	-2.895413	0.003786594	0.039870789
HECTD4	-1.608863	0.555733	-2.895031	0.003791207	0.039886725
ZNF396	-3.518565	1.215623	-2.894454	0.00379819	0.039900767
ENSG00000204758	-2.797126	0.96639	-2.894408	0.003798744	0.039900767
CCBE1	-3.508491	1.212422	-2.893787	0.003806266	0.039928999
DNA2	-3.06016	1.057534	-2.893674	0.003807638	0.039928999
UBE2D3.AS1	-2.802716	0.968893	-2.8927	0.003819456	0.040020308
ENSG00000259408	-3.161634	1.093172	-2.892165	0.003825975	0.04002923
LINC02019	-3.534152	1.221994	-2.892119	0.003826529	0.04002923
ENSG00000262777	-4.476827	1.548254	-2.891533	0.003833673	0.040071377
JAKMIP2	-2.62689	0.908663	-2.89094	0.003840909	0.040083304
PPIL6	-2.761713	0.955303	-2.890929	0.003841044	0.040083304
TBCEL	-2.347843	0.812265	-2.890491	0.003846406	0.040106734
USP6	-3.155952	1.092193	-2.889556	0.003857867	0.040193666
ENSG00000258768	-3.30385	1.143669	-2.888817	0.003866943	0.040255626
PRSS27	-2.580054	0.893297	-2.888238	0.00387407	0.040297221
DSP	-2.816755	0.97549	-2.88753	0.003882802	0.040347315
ENSG00000276718	-3.674272	1.272549	-2.887332	0.003885239	0.040347315
LYG1	-2.864917	0.99236	-2.886975	0.003889657	0.040347315
DUSP7	-2.920973	1.011845	-2.88678	0.003892068	0.040347315
CTBP2	-4.541451	1.573372	-2.886444	0.003896216	0.040347315
MAGEE1	-2.823726	0.978312	-2.886325	0.003897701	0.040347315
JMY	-2.204084	0.763863	-2.885444	0.003908624	0.040414964
TDRKH.AS1	-2.298328	0.796567	-2.885291	0.003910518	0.040414964
CORO2B	-3.486454	1.208811	-2.884202	0.003924075	0.040470086
ARL4D	-2.546189	0.882836	-2.884103	0.003925305	0.040470086
LINC01133	-4.407111	1.528254	-2.883755	0.003929648	0.040470086
ENSG00000269886	-4.650243	1.612613	-2.883669	0.003930713	0.040470086
HIST2H4A	-3.221615	1.117307	-2.883373	0.003934407	0.040470086
ENSG00000275202	-4.350045	1.508678	-2.883348	0.003934723	0.040470086
CLDN7	-3.154621	1.094633	-2.881899	0.003952862	0.040592754
ENSG00000272927	-2.066245	0.716995	-2.881814	0.003953933	0.040592754
GOLGA6L4	-2.657689	0.922283	-2.88164	0.003956114	0.040592754
PYHIN1	-3.666607	1.272805	-2.88073	0.003967552	0.040677686
GSTO2	-3.708607	1.28782	-2.879754	0.00397985	0.040735497
ENSG00000233427	-4.35326	1.511735	-2.879645	0.003981234	0.040735497
ADAT3	-2.696771	0.93658	-2.879381	0.003984566	0.040735497
ENSG00000273270	-1.894851	0.658099	-2.879279	0.003985855	0.040735497
ENSG00000111321	-2.447842	0.850344	-2.878651	0.003993801	0.04078431
EPN2	-2.107221	0.732118	-2.878253	0.003998844	0.040803433
PON2	-2.659489	0.924291	-2.877327	0.004010592	0.04089088
AP5S1	-1.814807	0.630825	-2.876881	0.004016276	0.040916411
CSNK2A3	-3.111957	1.08186	-2.876488	0.004021269	0.040934865
KCNRG	-3.612369	1.256274	-2.875463	0.004034358	0.040997929
NFS1	-1.980766	0.688884	-2.875326	0.0040361	0.040997929
ENSG00000269194	-2.640903	0.918548	-2.875083	0.004039214	0.040997929
ZNF34	-1.90917	0.664058	-2.875005	0.004040209	0.040997929

ACACA	-2.276897	0.792066	-2.874631	0.004044994	0.041014139
ENSG00000274425	-2.480902	0.86328	-2.87381	0.004055528	0.041071962
ZNF354B	-1.777216	0.618605	-2.872943	0.004066676	0.041071962
ATP6V0C	-4.122843	1.435072	-2.872916	0.004067019	0.041071962
RPRML	-4.953394	1.724202	-2.872862	0.004067714	0.041071962
MADCAM1	-2.364242	0.823098	-2.87237	0.004074052	0.041071962
HSPA1L	-4.72169	1.643877	-2.872289	0.004075095	0.041071962
KCNA7	-4.72169	1.643877	-2.872289	0.004075095	0.041071962
ZFPM1	-3.235423	1.126461	-2.872201	0.004076233	0.041071962
GEM	-2.697681	0.939511	-2.871366	0.004087016	0.041140708
ENSG00000234141	-3.723098	1.296715	-2.871178	0.004089451	0.041140708
ENSG00000247121	-2.261277	0.78769	-2.870771	0.004094721	0.041152864
LYSMD4	-2.286928	0.796677	-2.870583	0.004097154	0.041152864
FGL2	-3.076765	1.072084	-2.869893	0.004106108	0.041152864
LINC00174	-4.405093	1.534944	-2.869873	0.004106367	0.041152864
TSBP1.AS1	-3.887483	1.354659	-2.869713	0.004108446	0.041152864
HYAL2	-2.26032	0.787729	-2.869413	0.004112349	0.041152864
EFNA3	-2.752118	0.959178	-2.869248	0.004114491	0.041152864
ENSG00000257605	-4.461823	1.555123	-2.869113	0.004116246	0.041152864
XRCC2	-3.258981	1.136132	-2.868488	0.004124385	0.041173865
OCRL	-3.802276	1.325546	-2.868461	0.004124746	0.041173865
SPIN2A	-3.005655	1.048053	-2.867845	0.004132774	0.041202803
ENSG00000272663	-2.702418	0.942349	-2.867748	0.00413405	0.041202803
ABCA1	-4.613766	1.609347	-2.866856	0.004145713	0.041287063
CNNM4	-2.986292	1.042046	-2.865796	0.00415962	0.041330373
DNAJC17	-1.661677	0.579841	-2.865745	0.00416029	0.041330373
TBC1D23	-1.662882	0.580295	-2.865581	0.004162447	0.041330373
CCDC134	-2.714871	0.947418	-2.865546	0.00416291	0.041330373
LAPTM5	-0.509865	0.177947	-2.865272	0.004166512	0.041334242
SHF	-2.30998	0.806284	-2.86497	0.004170484	0.041341773
C18orf54	-2.428509	0.847928	-2.864053	0.004182578	0.041429741
HCG27	-2.576921	0.900043	-2.863108	0.004195071	0.041501129
ENSG00000263080	-3.573321	1.248106	-2.862995	0.004196568	0.041501129
ARHGEF12	-2.023397	0.706848	-2.862564	0.004202287	0.041501129
FAM168A	-2.176306	0.760273	-2.862534	0.004202687	0.041501129
ENSG00000228265	-2.651148	0.926441	-2.861648	0.004214446	0.041557109
ZNF578	-2.6187	0.915167	-2.861444	0.004217161	0.041557109
LRFN4	-3.097792	1.082623	-2.861377	0.004218045	0.041557109
ZGLP1	-3.295516	1.152586	-2.859236	0.004246623	0.041806659
AGAP9	-2.535993	0.887052	-2.8589	0.004251133	0.041819057
ENSG00000273391	-3.833492	1.341076	-2.858519	0.004256234	0.041837252
ENSG00000224746	-2.709077	0.947983	-2.857728	0.004266858	0.041909662
TAS2R14	-3.666741	1.283432	-2.856981	0.004276918	0.041957883
RFC2	-1.391225	0.486974	-2.856879	0.004278289	0.041957883
LINC00630	-4.032122	1.411684	-2.85625	0.00428678	0.041997032
ENSG00000255468	-3.454713	1.209612	-2.856051	0.004289461	0.041997032
ENSG00000259715	-2.800324	0.980619	-2.855671	0.004294606	0.041997032

ENSG00000179979	-3.146776	1.10196	-2.855616	0.004295336	0.041997032
RCBTB2	-4.393728	1.538819	-2.855259	0.004300169	0.042004359
PCDH11Y	-4.407703	1.543895	-2.854925	0.004304706	0.042004359
KLHL42	-2.086481	0.73096	-2.85444	0.004311279	0.042004359
TNFSF13B	-2.185985	0.765919	-2.854069	0.004316311	0.042004359
ZNF282	-2.571898	0.901196	-2.853873	0.004318974	0.042004359
TMEM220.AS1	-2.386784	0.83643	-2.853536	0.004323556	0.042004359
ENSG00000258634	-2.643618	0.926484	-2.853389	0.004325565	0.042004359
NPHP4	-3.443738	1.207297	-2.852436	0.004338561	0.042004359
PPP2R5D	-1.875303	0.657462	-2.852338	0.004339888	0.042004359
LINC02453	-2.918314	1.023137	-2.852319	0.004340147	0.042004359
KPTN	-2.164551	0.758881	-2.852292	0.004340519	0.042004359
EGR2	-3.506104	1.229327	-2.852053	0.004343791	0.042004359
ENSG00000260360	-3.822418	1.340268	-2.85198	0.00434479	0.042004359
NCKIPSD	-2.296953	0.805429	-2.851837	0.004346737	0.042004359
KDM4D	-3.577377	1.254413	-2.851833	0.004346793	0.042004359
ENSG00000233654	-3.086191	1.08222	-2.851722	0.004348318	0.042004359
PCYOX1L	-3.088096	1.083159	-2.851008	0.004358091	0.042037683
ENSG00000225075	-3.150046	1.104895	-2.850992	0.004358302	0.042037683
ZDHHC23	-2.06946	0.725938	-2.850741	0.004361755	0.042039473
ENSG00000229196	-3.678035	1.290615	-2.849832	0.004374232	0.04206885
ENSG00000272092	-3.629298	1.273667	-2.849487	0.004378973	0.04206885
ENSG00000272841	-3.352598	1.176804	-2.848901	0.004387051	0.04206885
ENSG00000267198	-3.227716	1.133072	-2.848642	0.004390629	0.04206885
TRIM16L	-2.354353	0.826513	-2.848536	0.004392094	0.04206885
ENSG00000277117	-2.341819	0.822158	-2.84838	0.004394241	0.04206885
CFD	-2.621879	0.920691	-2.84773	0.004403231	0.04206885
ENSG00000258017	-2.736207	0.960883	-2.847597	0.004405061	0.04206885
ENSG00000255320	-3.452409	1.212424	-2.847526	0.004406043	0.04206885
ZNF763	-2.60041	0.913237	-2.847465	0.004406888	0.04206885
BIVM	-2.722385	0.956084	-2.847434	0.004407324	0.04206885
ENSG00000259820	-3.027334	1.063288	-2.847143	0.004411355	0.04206885
ENSG00000227374	-4.479607	1.573432	-2.847028	0.004412946	0.04206885
ENSG00000267042	-4.589874	1.61224	-2.846893	0.004414819	0.04206885
LINC02158	-3.334326	1.171267	-2.846769	0.004416536	0.04206885
FAM218A	-2.582358	0.907253	-2.846348	0.00442238	0.04206885
IL3RA	-3.76203	1.321724	-2.846304	0.004422994	0.04206885
VWCE	-4.321357	1.51826	-2.846257	0.004423654	0.04206885
B3GALT6	-2.224017	0.781704	-2.84509	0.004439888	0.042149895
USP20	-2.100795	0.738421	-2.844982	0.004441389	0.042149895
PPA1	1.466558	0.515497	2.844938	0.004442003	0.042149895
ENSG00000229180	-1.849835	0.65033	-2.844454	0.004448757	0.042152462
ENSG00000272030	-3.779548	1.328745	-2.844449	0.004448826	0.042152462
ENSG00000213121	-4.355578	1.531622	-2.843768	0.004458351	0.042203125
NET1	-3.175216	1.116722	-2.843335	0.004464407	0.042203125
ENSG00000279765	-2.25811	0.794204	-2.843237	0.004465778	0.042203125
DECR2	-2.288943	0.80512	-2.842983	0.004469343	0.042203125

SLC14A2	-2.669229	0.938912	-2.842895	0.004470573	0.042203125
CALD1	-4.566	1.606597	-2.842032	0.004482696	0.04228655
ARMCX1	-3.129057	1.101093	-2.841773	0.004486342	0.042289939
STON1	-3.812719	1.342015	-2.841041	0.004496657	0.042333328
MPZL3	-2.778402	0.978279	-2.840092	0.004510057	0.042333328
SLC25A15	-2.840849	1.000276	-2.840065	0.004510431	0.042333328
RIMS2	-2.971005	1.046186	-2.839844	0.004513556	0.042333328
HMGN3.AS1	-3.014399	1.061471	-2.839832	0.004513731	0.042333328
RNF215	-3.02563	1.065428	-2.839825	0.004513834	0.042333328
ZDHHC9	-2.319935	0.816932	-2.839815	0.004513976	0.042333328
TREML2	-2.860669	1.007864	-2.838349	0.004534757	0.042477125
BGLAP	-3.365793	1.186227	-2.837393	0.004548364	0.042477125
TMEM201	-3.094424	1.090638	-2.837261	0.004550237	0.042477125
ENSG00000268439	-2.673507	0.942333	-2.837116	0.004552313	0.042477125
ENSG00000239415	-2.575766	0.907943	-2.836925	0.004555036	0.042477125
SPINT1.AS1	-2.135042	0.752616	-2.836829	0.004556408	0.042477125
PYGB	-2.099374	0.740044	-2.836823	0.00455649	0.042477125
BIRC5	-2.696927	0.950719	-2.836725	0.004557891	0.042477125
ENSG00000239941	-4.632587	1.633292	-2.83635	0.00456324	0.042477125
TTC26	-2.261784	0.797457	-2.836245	0.004564748	0.042477125
PHLDB1	-2.903475	1.023727	-2.836182	0.004565637	0.042477125
TIGD3	-2.904335	1.024113	-2.835953	0.004568924	0.042477125
NRG4	-3.698141	1.304241	-2.835473	0.004575786	0.042510213
NPL	-3.649688	1.28754	-2.834621	0.004588008	0.042572138
ZNF711	-2.90855	1.026107	-2.834547	0.004589069	0.042572138
CA13	-2.234107	0.788238	-2.834306	0.004592533	0.042573578
IGHV1.2	-3.419923	1.206718	-2.834069	0.004595938	0.042574466
NUF2	-3.656958	1.290665	-2.83339	0.00460571	0.042634294
GNG4	-4.396946	1.552366	-2.832416	0.004619777	0.042705179
RIPOR3	-2.227645	0.786487	-2.8324	0.004620005	0.042705179
ADCY9	-2.898782	1.023724	-2.831605	0.004631507	0.042771863
PLPP3	-2.583243	0.912342	-2.831442	0.004633868	0.042771863
SNX24	-2.955737	1.044072	-2.83097	0.004640713	0.04280434
LINC00271	-2.938595	1.038331	-2.830115	0.004653129	0.042888119
LINC02413	-3.525277	1.245735	-2.829876	0.004656606	0.042889438
OXT	-2.011744	0.711019	-2.829381	0.004663818	0.042925141
STK36	-2.814934	0.995115	-2.828752	0.004672993	0.042978483
ENSG00000263004	-2.606398	0.921628	-2.828036	0.004683453	0.042978483
SLC16A1	-2.750088	0.972564	-2.827666	0.004688869	0.042978483
THSD1	-2.77834	0.982706	-2.827234	0.004695195	0.042978483
FNDC3B	-3.055716	1.080837	-2.827177	0.00469604	0.042978483
ENSG00000260030	-3.078255	1.088971	-2.826756	0.004702214	0.042978483
MAST2	-2.30402	0.815245	-2.82617	0.004710832	0.042978483
CFAP53	-3.072916	1.087353	-2.826054	0.004712538	0.042978483
ZSWIM3	-2.676553	0.947115	-2.826008	0.004713212	0.042978483
SLC26A11	-2.335874	0.826595	-2.8259	0.004714794	0.042978483
FAM238A	-4.380313	1.550119	-2.825792	0.004716392	0.042978483

KLHL23	-2.333408	0.825845	-2.82548	0.004720987	0.042978483
ENSG00000232934	-3.450289	1.221154	-2.825433	0.004721682	0.042978483
ZNF710	-2.273814	0.804786	-2.825365	0.004722677	0.042978483
CCNT2.AS1	-2.72715	0.965256	-2.825312	0.004723457	0.042978483
KLHL15	-2.791693	0.988112	-2.82528	0.004723934	0.042978483
ACAD11	-3.218928	1.139398	-2.825113	0.004726397	0.042978483
ENSG00000228606	-2.77324	0.981772	-2.824729	0.004732056	0.042999554
ENSG00000273055	-3.640148	1.290357	-2.821039	0.004786838	0.04345158
MST1	-2.848161	1.009724	-2.820732	0.004791417	0.04345158
RNASEH2B.AS1	-4.064638	1.441042	-2.820623	0.004793046	0.04345158
TTC24	-2.768554	0.981623	-2.820384	0.004796619	0.04345158
MAD2L1	-2.221079	0.787548	-2.820246	0.004798686	0.04345158
SGTB	-1.773326	0.628904	-2.819708	0.004806743	0.043479759
PDGFRB	-3.11063	1.103222	-2.819587	0.004808557	0.043479759
RHOBTB2	-2.218537	0.786981	-2.819047	0.004816642	0.043520908
PLPP6	-2.053469	0.728482	-2.818832	0.004819872	0.043520908
ENSG00000261242	-4.501706	1.597756	-2.817518	0.004839638	0.043648354
TMC3.AS1	-2.497441	0.886421	-2.817443	0.004840771	0.043648354
FXYD2	-1.471203	0.522288	-2.816841	0.004849852	0.043699615
NR4A2	-2.232731	0.792703	-2.816604	0.004853436	0.043701301
MIR3142HG	-3.992278	1.417539	-2.816344	0.004857367	0.043706117
PFN2	-3.215618	1.141921	-2.815973	0.004862978	0.043718386
HLCS	-2.327809	0.826713	-2.815741	0.004866487	0.043718386
ZNF410	-3.177942	1.128865	-2.815165	0.004875216	0.043718386
STK39	-2.716101	0.964834	-2.815097	0.004876255	0.043718386
ENSG00000272155	-2.213414	0.786269	-2.815084	0.004876452	0.043718386
CSGALNACT1	-1.733002	0.615651	-2.814908	0.004879117	0.043718386
ENSG00000235522	-4.795325	1.704312	-2.813642	0.00489838	0.043860441
ZNF597	-2.936863	1.044093	-2.812836	0.004910669	0.043926121
ENSG00000277767	-3.029363	1.077025	-2.812713	0.004912543	0.043926121
TLR2	-4.290986	1.525732	-2.812411	0.004917156	0.04393684
IGLV1.44	-2.859327	1.016904	-2.811797	0.004926551	0.043990236
CST3	-2.499917	0.889245	-2.81128	0.004934488	0.044018396
ZFP36L1	-0.44311	0.157627	-2.811127	0.004936836	0.044018396
RAB11FIP1	-1.274679	0.453499	-2.810763	0.004942422	0.044018396
DCLRE1A	-3.342456	1.18919	-2.8107	0.004943389	0.044018396
SLC25A25.AS1	-2.237811	0.796651	-2.809022	0.004969231	0.04417005
MYO18A	-3.252321	1.157834	-2.80897	0.004970033	0.04417005
ENSG00000116957	-1.909536	0.67981	-2.808925	0.004970718	0.04417005
HSPB1	-1.047125	0.372899	-2.808063	0.004984041	0.044257873
DCAF15	-1.589084	0.565987	-2.807636	0.004990654	0.044286032
CHEK1	-2.695902	0.960336	-2.807249	0.004996664	0.044287396
SORBS3	-2.218958	0.790457	-2.807183	0.004997692	0.044287396
HIPK1.AS1	-2.237713	0.797236	-2.806839	0.005003024	0.044304137
CDCA5	-4.184926	1.491093	-2.806617	0.005006471	0.044304166
ENSG00000228172	-2.691437	0.959128	-2.806129	0.005014059	0.044340821
RADX	-2.499148	0.890742	-2.805693	0.005020845	0.044352256

SSTR2	-2.54866	0.90847	-2.805442	0.005024759	0.044352256
ENSG00000263069	-3.51794	1.254069	-2.805221	0.005028214	0.044352256
ZNF547	-2.505761	0.893268	-2.805161	0.00502914	0.044352256
ENSG00000259804	-3.551167	1.266149	-2.8047	0.005036341	0.044382756
KHDC1	-2.624925	0.936034	-2.804307	0.005042488	0.044382756
IGLV3.19	-3.351776	1.195237	-2.804278	0.005042946	0.044382756
ENSG00000279571	-4.455073	1.588847	-2.803965	0.005047831	0.044387488
HSPA4L	-2.455169	0.875713	-2.803621	0.005053225	0.044387488
ZSCAN31	-3.997185	1.425742	-2.803582	0.005053833	0.044387488
ENSG00000251259	-2.612974	0.932316	-2.802669	0.005068173	0.04446552
DNAJC27.AS1	-2.870704	1.024309	-2.802576	0.005069629	0.04446552
IGSF6	-4.445298	1.586451	-2.802039	0.005078073	0.044509238
MYOM2	-2.556652	0.912656	-2.80133	0.005089237	0.044576727
INAFM2	-4.417994	1.577471	-2.800681	0.005099489	0.044608366
ANKRD34A	-4.524763	1.615604	-2.800662	0.005099783	0.044608366
ENSG00000272669	-2.951017	1.053855	-2.800213	0.005106892	0.044640207
MSS51	-3.676186	1.313137	-2.799546	0.005117458	0.044702197
ZNF426.DT	-3.490956	1.247104	-2.799249	0.005122161	0.044712918
TIGIT	-3.222451	1.151394	-2.798738	0.005130276	0.044730811
THAP9	-2.614144	0.934062	-2.798682	0.005131163	0.044730811
ZBTB33	-1.736005	0.620382	-2.798284	0.00513749	0.044755639
ENSG00000266918	-2.412721	0.862311	-2.797971	0.005142475	0.044757162
PPP1R32	-2.892652	1.033889	-2.797836	0.005144621	0.044757162
PRPF40B	-2.176801	0.77814	-2.797442	0.005150896	0.044781476
ENSG00000203279	-2.77232	0.991323	-2.796585	0.005164577	0.044801815
RNF157	-3.675046	1.314234	-2.796341	0.005168478	0.044801815
LRRC25	-4.255215	1.521727	-2.796307	0.005169032	0.044801815
TEC	-2.176	0.7782	-2.796198	0.005170774	0.044801815
ZNF66	-2.74131	0.980441	-2.795996	0.005173997	0.044801815
IL18	-3.135429	1.121403	-2.795988	0.005174127	0.044801815
ENSG00000270964	-4.219314	1.509425	-2.795312	0.005184967	0.044811117
CTSK	-3.29173	1.177626	-2.795224	0.005186373	0.044811117
CDKN2C	-3.195585	1.14331	-2.795028	0.005189516	0.044811117
NRSN2.AS1	-2.238074	0.800753	-2.794962	0.005190582	0.044811117
HPSE	-3.53813	1.265953	-2.794835	0.005192615	0.044811117
UBTD1	-3.403823	1.218248	-2.79403	0.005205567	0.044892783
ENSG00000230177	-2.127162	0.761634	-2.792894	0.005223883	0.044993694
CCNA1	-3.519475	1.260164	-2.79287	0.005224262	0.044993694
LINC00896	-4.029944	1.443067	-2.792625	0.005228221	0.044995111
ENSG00000150076	-3.599545	1.289038	-2.792427	0.00523142	0.044995111
ENSG00000203325	-2.286497	0.818926	-2.792068	0.005237232	0.04501501
ZNF236.DT	-2.884048	1.033162	-2.791476	0.005246824	0.045067344
ENSG00000262692	-4.344501	1.556538	-2.791131	0.005252419	0.045085307
TSPYL5	-2.494422	0.893857	-2.790628	0.005260587	0.045125312
MYL9	-2.197767	0.787923	-2.789315	0.005281961	0.045142747
NDST2	-2.46223	0.882778	-2.789185	0.005284087	0.045142747
AHCYL2	-2.971302	1.065295	-2.789181	0.005284145	0.045142747

ENSG00000254427	-3.013906	1.08061	-2.789077	0.005285845	0.045142747
ZBTB8B	-4.315151	1.547241	-2.788934	0.00528819	0.045142747
ENSG00000266086	-4.315151	1.547241	-2.788934	0.00528819	0.045142747
MIR222HG	-2.68224	0.961831	-2.78868	0.005292336	0.045142747
PTPRF	-3.729919	1.337701	-2.788305	0.00529846	0.045142747
C5	-3.252064	1.166422	-2.788069	0.005302328	0.045142747
NKX3.1	-3.078872	1.104317	-2.788033	0.005302922	0.045142747
TUBGCP5	-2.065681	0.74093	-2.787956	0.00530417	0.045142747
AFAP1L2	-2.9749	1.067067	-2.787923	0.00530472	0.045142747
ENSG00000231212	-2.923473	1.048792	-2.787468	0.005312176	0.045174297
MIXL1	-4.374933	1.569691	-2.78713	0.00531772	0.045174297
GDPD1	-2.259145	0.810631	-2.786895	0.005321569	0.045174297
SPESP1	-3.453238	1.239334	-2.786366	0.005330269	0.045174297
DCUN1D2	-2.177143	0.781358	-2.786356	0.005330424	0.045174297
KCP	-3.266138	1.172231	-2.786258	0.005332049	0.045174297
ENSG00000270024	-4.423028	1.587524	-2.786116	0.00533437	0.045174297
COMMD7	-1.094367	0.392854	-2.785679	0.00534157	0.045174297
LINC02656	-2.552368	0.91626	-2.785637	0.005342267	0.045174297
ENSG00000249141	-3.067112	1.101129	-2.785424	0.005345772	0.045174297
ENSG00000245498	-3.593018	1.289971	-2.785347	0.005347047	0.045174297
SLC27A3	-2.418552	0.868864	-2.78358	0.005376256	0.045383928
ENSG00000262420	-3.275267	1.176743	-2.783332	0.005380378	0.045383928
IZUMO4	-1.101219	0.395702	-2.782953	0.005386661	0.045383928
ENSG00000269176	-2.875067	1.03312	-2.782898	0.005387574	0.045383928
YRDC	-2.84329	1.021814	-2.782591	0.005392682	0.045383928
LARGE1	-2.369424	0.851524	-2.78257	0.005393023	0.045383928
PLK2	-4.018963	1.444548	-2.782159	0.00539986	0.045411763
ENSG00000270022	-2.845802	1.023316	-2.78096	0.005419836	0.045549984
TTYH1	-4.090595	1.471742	-2.779423	0.005445552	0.045736235
ENSG00000259520	-4.134816	1.488531	-2.777784	0.005473107	0.04592137
ING2	-1.47853	0.532288	-2.777687	0.005474732	0.04592137
EPHA1.AS1	-2.193778	0.789894	-2.777306	0.005481147	0.045941257
ENSG00000261613	-3.605582	1.298316	-2.777123	0.005484244	0.045941257
ENSG00000259153	-2.464129	0.887534	-2.776378	0.005496825	0.046016687
LY6E.DT	-2.614447	0.941844	-2.775881	0.005505238	0.046042955
GGT7	-2.111566	0.760767	-2.775574	0.005510439	0.046042955
MAPKBP1	-2.145577	0.773067	-2.775408	0.005513242	0.046042955
COQ3	-2.759263	0.994204	-2.775347	0.005514276	0.046042955
CAPN14	-4.461281	1.607813	-2.774752	0.005524387	0.046075089
LRRC27	-1.918331	0.691377	-2.774652	0.00552608	0.046075089
AHRR	-3.418659	1.23228	-2.774256	0.005532804	0.046075089
HAL	-3.545346	1.278113	-2.773891	0.005539025	0.046075089
RPL35	-0.148872	0.053672	-2.77375	0.00554143	0.046075089
CTU1	-4.357563	1.571023	-2.77371	0.0055421	0.046075089
DDX43	-2.637649	0.95097	-2.773642	0.005543272	0.046075089
ENSG00000261684	-4.004894	1.444164	-2.773158	0.005551523	0.046075089
ENSG00000255328	-3.175841	1.145221	-2.773124	0.005552098	0.046075089

MAST1	-4.466241	1.61078	-2.77272	0.005558994	0.046075089
ENSG00000261732	-1.811899	0.653575	-2.772289	0.005566364	0.046075089
RBKS	-2.708021	0.976825	-2.77227	0.005566691	0.046075089
DPH5	-1.107225	0.399404	-2.772196	0.005567947	0.046075089
ENSG00000280987	-2.478818	0.89419	-2.772138	0.005568949	0.046075089
DSCR9	-3.8278	1.381078	-2.771603	0.0055781	0.046075089
ENSG00000184271	-2.45735	0.88662	-2.771595	0.005578243	0.046075089
ENSG00000269737	-3.00634	1.084714	-2.771551	0.005579	0.046075089
YBEY	-1.079212	0.389438	-2.771201	0.005584996	0.046095024
VRK1	-1.476334	0.532845	-2.770663	0.005594223	0.046141579
FZD6	-3.504192	1.26492	-2.770288	0.00560068	0.046144081
ENSG00000270175	-2.472971	0.892747	-2.770068	0.005604465	0.046144081
ARL14EPL	-2.548671	0.920091	-2.77002	0.005605285	0.046144081
NEK11	-3.737486	1.349386	-2.769768	0.00560963	0.046150323
B3GNTL1	-2.252584	0.81336	-2.769479	0.005614604	0.046161734
SPATA5	-1.950695	0.704458	-2.769073	0.005621601	0.046189745
IL17RC	-4.45461	1.609172	-2.768262	0.005635618	0.046275363
GPAT2	-4.323565	1.56244	-2.767188	0.005654207	0.046333227
FITM1	-3.221279	1.164163	-2.767034	0.005656887	0.046333227
ENSG00000250075	-2.629137	0.950189	-2.766963	0.005658121	0.046333227
ITPKC	-2.747738	0.993145	-2.766704	0.00566262	0.046333227
KIAA1324L	-2.470835	0.893071	-2.766674	0.00566313	0.046333227
ENSG00000234290	-4.347293	1.571435	-2.766448	0.005667064	0.046333227
ST6GALNAC2	-3.602314	1.302166	-2.766401	0.005667871	0.046333227
ENSG00000227908	-4.204376	1.520023	-2.765995	0.00567494	0.046361555
ENSG00000258738	-2.66284	0.962924	-2.76537	0.005685819	0.046420961
SPTY2D1OS	-3.432225	1.241538	-2.764494	0.005701118	0.046479903
PIK3IP1.AS1	-2.836221	1.026091	-2.764102	0.005707969	0.046479903
RASAL2	-2.934472	1.061652	-2.764062	0.005708661	0.046479903
NINL	-2.979253	1.077934	-2.763856	0.005712277	0.046479903
ENSG00000274922	-4.497049	1.627097	-2.763848	0.005712417	0.046479903
HIST1H3H	-3.135087	1.134373	-2.763717	0.005714713	0.046479903
NEU3	-2.561439	0.927064	-2.762958	0.005728016	0.04655055
ENSG00000275180	-2.563291	0.927843	-2.762635	0.005733692	0.04655055
TTC12	-2.499415	0.904732	-2.762603	0.005734252	0.04655055
ENSG00000267011	-3.225702	1.168112	-2.761466	0.005754244	0.046661746
TIGD7	-2.03989	0.738713	-2.761412	0.005755203	0.046661746
DSCAML1	-3.087603	1.118428	-2.760664	0.0057684	0.046739298
ENSG00000259888	-3.291486	1.192412	-2.760358	0.005773798	0.046753592
ENSG00000261360	-2.985062	1.081541	-2.760009	0.005779974	0.046765401
CDYL2	-3.756899	1.361262	-2.759865	0.005782525	0.046765401
PANK1	-2.966543	1.075325	-2.75874	0.005802464	0.046896653
LINC01250	-4.321618	1.566633	-2.758539	0.005806044	0.046896653
IQCC	-2.5641	0.929653	-2.758128	0.005813347	0.046926182
C19orf33	-2.895117	1.049977	-2.757313	0.005827848	0.04696095
QPCTL	-2.511724	0.910941	-2.757284	0.005828376	0.04696095
LINC02210.CRHR1	-3.339639	1.211304	-2.757062	0.005832335	0.04696095

ENSG00000261441	-3.605937	1.307949	-2.75694	0.005834498	0.04696095
C16orf46	-3.381739	1.226687	-2.756807	0.005836875	0.04696095
ENSG00000272434	-4.47823	1.624515	-2.756657	0.005839552	0.04696095
PGP	-2.027114	0.73543	-2.756367	0.005844734	0.046973261
ENSG00000260060	-3.15884	1.14616	-2.75602	0.005850942	0.046993801
SLC39A14	-3.962116	1.437908	-2.755473	0.005860742	0.047020022
LINC01285	-4.011848	1.456048	-2.755299	0.005863858	0.047020022
HFE	-2.428032	0.881246	-2.755226	0.005865169	0.047020022
UTP15	-2.044844	0.742342	-2.754585	0.005876661	0.047082813
ENSG00000224905	-3.619316	1.31416	-2.754091	0.005885545	0.047115174
MSANTD3	-2.106678	0.765062	-2.753605	0.005894294	0.047115174
FEM1C	-2.08519	0.75726	-2.753597	0.005894436	0.047115174
ENSG00000257261	-2.754088	1.000255	-2.753386	0.005898223	0.047115174
LNX2	-2.020268	0.733751	-2.753343	0.005899009	0.047115174
EXOC6B	-2.463626	0.894885	-2.753008	0.005905049	0.04713416
KIZ.AS1	-2.436049	0.885045	-2.752458	0.005914974	0.047184108
PDSS1	-2.468975	0.897314	-2.751516	0.005932005	0.04729065
IER3.AS1	-2.572568	0.935073	-2.751196	0.005937804	0.047299008
SYNGAP1.AS1	-2.227591	0.809723	-2.751053	0.005940405	0.047299008
ITCH.AS1	-3.428862	1.246959	-2.74978	0.005963528	0.047440347
PHLPP1	-2.084751	0.758197	-2.749615	0.005966532	0.047440347
CDC42BPA	-4.376074	1.591711	-2.749288	0.005972483	0.047440347
SRCAP	-2.630904	0.957178	-2.748606	0.005984933	0.047440347
ENSG00000276449	-2.270366	0.826118	-2.748233	0.005991745	0.047440347
ENSG00000250616	-2.828964	1.029427	-2.748096	0.005994249	0.047440347
NUDT17	-3.361029	1.223063	-2.748043	0.005995213	0.047440347
ENSG00000260219	-2.787931	1.014517	-2.748039	0.005995284	0.047440347
SCML4	-2.610444	0.949935	-2.748023	0.005995581	0.047440347
DNAJA4	-2.894283	1.05325	-2.747956	0.005996804	0.047440347
LINC02035	-2.771458	1.008591	-2.747852	0.005998713	0.047440347
ENSG00000245975	-4.031448	1.467295	-2.747538	0.006004455	0.047456587
PPP2R3A	-2.370426	0.862919	-2.746985	0.006014589	0.047507501
ENSG00000226266	-2.666397	0.971014	-2.745992	0.006032827	0.047611697
SLC35G1	-3.411937	1.242584	-2.74584	0.006035626	0.047611697
GCNA	-2.54785	0.927996	-2.745539	0.006041159	0.047611697
TMEM121	-3.821664	1.391993	-2.745462	0.006042582	0.047611697
HIST1H2BK	-2.158094	0.786122	-2.74524	0.006046662	0.047614684
KLHL34	-4.100939	1.494083	-2.744786	0.006055029	0.04765141
WDR97	-4.41398	1.608337	-2.744438	0.006061468	0.047663704
SARM1	-2.103814	0.766612	-2.744301	0.006064	0.047663704
NATD1	-2.497055	0.91001	-2.743987	0.006069791	0.047680102
LIPT2	-2.177118	0.793665	-2.743121	0.006085822	0.047776856
ZDHHC18	-1.945881	0.709549	-2.742419	0.006098851	0.047849945
UNC5CL	-3.248639	1.18478	-2.741977	0.006107062	0.047885169
LRRC8C	-2.141388	0.78125	-2.740976	0.006125697	0.047940112
ENSG00000282386	-2.574743	0.939363	-2.740946	0.006126255	0.047940112
MAGIX	-3.125011	1.140227	-2.740691	0.006131009	0.047940112

ENSG00000279467	-2.692841	0.982562	-2.740633	0.006132103	0.047940112
MED27	-2.529994	0.92327	-2.740255	0.006139155	0.047940112
RFNG	-2.057375	0.750804	-2.740229	0.006139637	0.047940112
ENSG00000275413	-3.20454	1.169502	-2.740088	0.006142277	0.047940112
RIC8B	-2.682593	0.979048	-2.740002	0.006143875	0.047940112
TEX2	-2.516946	0.91877	-2.739473	0.006153786	0.047988338
UHRF1BP1	-2.189775	0.799507	-2.738906	0.006164403	0.048042016
PRSS21	-4.292263	1.56729	-2.738652	0.006169154	0.048049945
ENSG00000273373	-2.181969	0.796883	-2.738129	0.006178988	0.048085387
POMT2	-2.536118	0.926263	-2.738012	0.006181179	0.048085387
HAND2.AS1	-3.954951	1.444608	-2.737732	0.006186445	0.048097276
LLGL1	-2.186543	0.798979	-2.736672	0.00620642	0.048223436
FUT11	-1.549017	0.566232	-2.735658	0.006225568	0.048337248
KLHDC9	-3.197609	1.168977	-2.735391	0.006230631	0.048337248
ABCD1	-2.560589	0.936218	-2.735034	0.006237385	0.048337248
ZNF513	-2.187223	0.799756	-2.734862	0.006240649	0.048337248
ENSG00000245466	-2.796497	1.022599	-2.734695	0.006243806	0.048337248
CBLL1	-1.433595	0.52425	-2.734564	0.00624629	0.048337248
TTC22	-3.268891	1.19548	-2.734375	0.006249877	0.048337248
KLB	-3.431404	1.255164	-2.733828	0.006260267	0.048337248
ENSG00000255224	-3.970289	1.452314	-2.733768	0.006261409	0.048337248
ENSG00000260495	-2.370255	0.867087	-2.733584	0.00626491	0.048337248
ENTR1	-1.789442	0.654618	-2.733568	0.006265226	0.048337248
ARF4.AS1	-2.447484	0.89536	-2.733519	0.006266148	0.048337248
SYT5	-4.19074	1.533384	-2.733001	0.006276012	0.048384329
PLPPR2	-4.334269	1.586493	-2.731982	0.006295465	0.048503969
DLG4	-4.250484	1.555932	-2.731793	0.00629907	0.048503969
PIGB	-1.793852	0.656813	-2.731146	0.006311446	0.048570199
STIMATE	-3.175454	1.162872	-2.7307	0.006319997	0.048606932
DGCR6	-2.765962	1.013042	-2.730353	0.006326653	0.048623523
ENSG00000266340	-2.488098	0.911327	-2.730194	0.006329712	0.048623523
FBXO45	-2.318563	0.849398	-2.729654	0.006340087	0.048650764
NEDD4L	-2.596456	0.951275	-2.729448	0.006344039	0.048650764
ZC2HC1C	-2.204809	0.807853	-2.729221	0.006348406	0.048650764
LRATD2	-3.186803	1.167716	-2.729092	0.006350905	0.048650764
ENSG00000228005	-3.240938	1.18758	-2.729026	0.006352163	0.048650764
GSTA4	-3.059298	1.121239	-2.728498	0.00636235	0.048678369
SCRG1	-2.67262	0.979539	-2.728447	0.006363334	0.048678369
LINC01481	-4.200504	1.539695	-2.728139	0.006369272	0.048694842
DNAJB5	-3.890297	1.426679	-2.726821	0.006394766	0.048860718
ENSG00000230709	-2.2449	0.823405	-2.726363	0.006403659	0.048899631
HIST2H2BF	-2.508832	0.920367	-2.725905	0.006412549	0.048938478
ENSG00000132481	-4.154198	1.524658	-2.724676	0.006436461	0.049091847
NBEAL2	-2.224102	0.81696	-2.722412	0.006480731	0.049398934
ENSG00000276075	-3.471097	1.275182	-2.72204	0.006488028	0.049398934
LINC01004	-3.47283	1.275824	-2.722029	0.006488241	0.049398934
LRRFIP1	-0.673056	0.247384	-2.720693	0.00651452	0.049569677

ZNF485	-2.044715	0.751631	-2.720371	0.006520862	0.049588611
MYBPC2	-3.140532	1.154951	-2.719192	0.006544168	0.049698704
DNAJC5B	-2.298146	0.845185	-2.719106	0.00654587	0.049698704
ENSG00000229728	-3.880276	1.427069	-2.719052	0.006546926	0.049698704
ANKS1A	-2.356635	0.867194	-2.717542	0.006576884	0.049877582
TBC1D2	-3.857391	1.419479	-2.71747	0.006578308	0.049877582
NPIPB6	-3.884665	1.429616	-2.717278	0.00658212	0.049877582
SMYD3	-1.785778	0.657288	-2.716887	0.006589908	0.049892952
ENSG00000269318	-4.107146	1.511844	-2.716647	0.006594685	0.049892952
MORN4	-4.219507	1.553235	-2.716592	0.006595781	0.049892952
TTC30B	-2.78022	1.023716	-2.715812	0.006611341	0.04998127
TIGD6	-2.38776	0.879294	-2.715542	0.006616736	0.049992684
ZNF571	-1.948713	0.718066	-2.713834	0.006650953	0.05022172
ZNF831	-4.225178	1.55715	-2.713404	0.006659584	0.0502286
ENSG00000280316	-2.409851	0.888193	-2.713207	0.006663538	0.0502286
MZF1.AS1	-2.889304	1.064969	-2.713041	0.006666885	0.0502286
LINC02362	-2.43306	0.896812	-2.713012	0.006667479	0.0502286
UST	-2.412378	0.889503	-2.712051	0.006686837	0.050326495
GPRC5D.AS1	-3.522637	1.298918	-2.711978	0.006688296	0.050326495
TFR2	-2.353497	0.868276	-2.710541	0.00671736	0.050457977
PAQR4	-4.095563	1.510977	-2.71054	0.006717366	0.050457977
ENSG00000272037	-3.237383	1.19439	-2.710491	0.006718377	0.050457977
FBXL13	-4.181355	1.542743	-2.710339	0.006721456	0.050457977
ZNF74	-2.211579	0.816073	-2.710026	0.006727802	0.05047617
IL24	-3.559364	1.313588	-2.709651	0.0067354	0.050484504
HIST1H4J	-2.172019	0.801606	-2.709584	0.00673676	0.050484504
RCOR1	-1.96949	0.726957	-2.709225	0.006744065	0.050509831
ZNF613	-2.121375	0.78319	-2.708636	0.006756047	0.050557471
ENSG00000266993	-3.323522	1.22706	-2.708526	0.006758285	0.050557471
ZNF404	-2.326762	0.85912	-2.708308	0.006762716	0.050561223
TRIM2	-4.31042	1.592278	-2.707077	0.006787841	0.050693036
ENSG00000257221	-3.16416	1.168966	-2.706802	0.006793482	0.050693036
LINC00663	-2.056705	0.759833	-2.706788	0.006793772	0.050693036
ZEB2.AS1	-2.364687	0.873651	-2.706674	0.006796105	0.050693036
PRKAR2A.AS1	-4.049832	1.496572	-2.706072	0.006808423	0.050737887
ENSG00000234484	-4.159488	1.537154	-2.705966	0.006810592	0.050737887
ACCS	-1.831461	0.676864	-2.705803	0.006813948	0.050737887
ENSG00000271895	-2.833421	1.047318	-2.705406	0.006822095	0.050769167
ZNF436	-3.429625	1.268135	-2.704463	0.006841482	0.050881552
DUSP11	1.91346	0.707576	2.704244	0.006845994	0.050881552
ENSG00000259915	-3.869735	1.431064	-2.704096	0.00684906	0.050881552
TOLLIP.AS1	-2.858894	1.057404	-2.70369	0.006857429	0.050894781
FGF14.AS2	-3.245785	1.200531	-2.703626	0.006858751	0.050894781
ELOVL5	-0.907018	0.335544	-2.703125	0.006869089	0.050931
SMOX	-3.484356	1.289067	-2.703006	0.006871549	0.050931
SMURF1	-2.140038	0.791874	-2.702498	0.006882064	0.050955648
WDR25	-2.304931	0.852959	-2.702277	0.006886637	0.050955648

GNMT	-4.049505	1.498661	-2.702082	0.006890674	0.050955648
ENSG00000282246	-2.334154	0.863836	-2.70208	0.006890715	0.050955648
ENSG00000272716	-2.508432	0.928468	-2.701689	0.006898819	0.050957865
ENSG00000263809	-2.691184	0.996161	-2.701554	0.006901618	0.050957865
TEAD2	-3.996894	1.479753	-2.701054	0.006912009	0.050957865
BDH1	-1.805174	0.668346	-2.700956	0.006914055	0.050957865
ENO1.AS1	-2.748807	1.017769	-2.700816	0.006916963	0.050957865
PRDM1	-3.150423	1.166542	-2.700651	0.006920388	0.050957865
MAPK11	-2.5291	0.936572	-2.700381	0.006926004	0.050957865
TMEM52	-3.321424	1.230023	-2.700294	0.006927821	0.050957865
DIPK1B	-2.505207	0.927801	-2.700156	0.006930704	0.050957865
C6orf132	-4.132684	1.530701	-2.699864	0.006936781	0.050957865
SLC26A6	-1.967012	0.728567	-2.699837	0.00693735	0.050957865
DFFB	-2.061024	0.763404	-2.69978	0.006938539	0.050957865
ENSG00000227741	-4.116178	1.524934	-2.69925	0.006949597	0.05100996
AMY2B	-2.612278	0.967854	-2.699041	0.006953964	0.051012917
CLDN12	-2.291188	0.849526	-2.697018	0.006996358	0.051284803
CCDC102A	-2.324917	0.862148	-2.696656	0.007003966	0.051284803
ZBED2	-1.786821	0.662636	-2.696534	0.007006529	0.051284803
HCG25	-2.446146	0.907161	-2.696484	0.007007579	0.051284803
PPL	-2.596894	0.963124	-2.696323	0.007010956	0.051284803
ZFP41	-2.374694	0.880792	-2.696088	0.007015915	0.051291917
GOLGA6L10	-2.897638	1.074892	-2.695748	0.007023075	0.051301898
ENSG00000228506	-4.237163	1.571855	-2.695645	0.007025254	0.051301898
SLC16A5	-2.747713	1.019431	-2.695339	0.007031711	0.051312838
ENSG00000259939	-4.110396	1.525082	-2.695196	0.007034728	0.051312838
ARSA	-1.742578	0.646753	-2.694348	0.007052652	0.051414434
ABHD2	-1.775244	0.659016	-2.693782	0.007064632	0.051462993
YTHDC2	-1.507478	0.55964	-2.693656	0.007067312	0.051462993
ANKRD6	-2.500483	0.928532	-2.692942	0.007082457	0.051544106
PUS7	-2.690395	0.999211	-2.692518	0.007091466	0.051580492
FBXO41	-2.567383	0.953727	-2.691949	0.007103586	0.05163946
ENSG00000249209	-3.908829	1.452203	-2.691654	0.007109875	0.051655989
KCNA3	-2.896977	1.076729	-2.690536	0.007133743	0.051800154
ENSG00000244198	-4.152675	1.54365	-2.690166	0.007141657	0.051809117
PI4K2A	-2.291575	0.851854	-2.690101	0.007143031	0.051809117
ZNF436.AS1	-2.326595	0.865179	-2.689149	0.007163437	0.05191955
KLRD1	-3.022934	1.124212	-2.688937	0.007168	0.05191955
YES1	-3.346473	1.244692	-2.688596	0.007175322	0.05191955
MMAA	-2.541706	0.9456	-2.687928	0.007189681	0.05191955
C15orf65	-2.323236	0.864499	-2.687379	0.007201528	0.05191955
JMJD1C.AS1	-2.968914	1.104778	-2.687341	0.007202348	0.05191955
RWDD2A	-2.101565	0.782027	-2.687331	0.007202544	0.05191955
GINS3	-2.848612	1.060042	-2.687263	0.007204012	0.05191955
ENSG00000272696	-3.42289	1.273798	-2.687153	0.007206399	0.05191955
CCDC121	-3.89415	1.44925	-2.68701	0.00720948	0.05191955
CLHC1	-2.180559	0.811635	-2.686627	0.007217757	0.05191955

PTCH2	-2.920315	1.087012	-2.686553	0.007219341	0.05191955
LY86.AS1	-4.350818	1.619495	-2.686528	0.007219891	0.05191955
ARMC2	-2.376931	0.884794	-2.686423	0.007222164	0.05191955
FAM230H	-4.283478	1.594602	-2.686237	0.007226181	0.05191955
GDPD3	-2.659691	0.990167	-2.686103	0.007229087	0.05191955
PRRG4	-2.539675	0.945523	-2.686	0.007231301	0.05191955
LUC7L3	-0.598569	0.22286	-2.685849	0.007234569	0.05191955
DACT3	-2.644594	0.984702	-2.68568	0.007238243	0.05191955
BORA	-2.062971	0.768147	-2.685647	0.007238958	0.05191955
ENSG00000228302	-2.979523	1.109519	-2.685418	0.007243916	0.051926165
L2HGDH	-2.160384	0.804546	-2.685221	0.007248197	0.051927921
ENC1	-2.525034	0.940648	-2.684355	0.007266987	0.052033569
PER3	-3.112322	1.159774	-2.683558	0.007284325	0.052128705
ENSG00000273367	-4.182381	1.55885	-2.682991	0.00729669	0.052169611
SFMBT2	-3.319919	1.237426	-2.682924	0.00729815	0.052169611
PDIA3	-0.860728	0.320863	-2.682538	0.007306579	0.052176127
MIAT	-3.090878	1.152233	-2.682511	0.007307172	0.052176127
STX1A	-4.264129	1.589831	-2.682128	0.007315553	0.052180183
HS3ST3B1	-2.42552	0.904453	-2.681752	0.007323767	0.052180183
IL12A	-2.397993	0.894202	-2.681714	0.007324594	0.052180183
CRYBG3	-2.140778	0.798297	-2.681681	0.007325336	0.052180183
TRPV3	-3.034866	1.131829	-2.681381	0.007331893	0.052180183
CLDN11	-3.436798	1.281731	-2.681373	0.007332072	0.052180183
PSMD5	-1.575579	0.587693	-2.680953	0.007341272	0.052216112
ENSG00000255121	-2.658721	0.991865	-2.680527	0.007350631	0.052216112
ENSG00000272644	-3.871655	1.444395	-2.680469	0.007351911	0.052216112
PERP	-2.524494	0.941834	-2.680403	0.007353353	0.052216112
ENSG00000262413	-3.369403	1.257299	-2.679875	0.007364974	0.052269787
GADD45G	-3.554122	1.326403	-2.679519	0.007372793	0.052296434
LNX1	-2.183629	0.814999	-2.679303	0.007377567	0.052301462
KLHL26	-2.127421	0.794625	-2.677263	0.007422636	0.052591989
AGFG2	-2.525441	0.943385	-2.677	0.007428455	0.052604257
ENSG00000251034	-4.232461	1.581172	-2.676787	0.007433194	0.052608859
ENSG00000272379	-2.651422	0.990676	-2.676375	0.007442324	0.052644519
POMGNT2	-2.382008	0.890235	-2.675707	0.007457185	0.052685955
ENSG00000267317	-2.367901	0.885006	-2.675576	0.007460106	0.052685955
MAPK12	-4.070053	1.521197	-2.675559	0.007460466	0.052685955
KCNAB3	-3.237431	1.210116	-2.675306	0.00746611	0.052696894
OXNAD1	-2.039791	0.762513	-2.675088	0.007470966	0.05270226
GSEC	-3.79852	1.420184	-2.674667	0.007480343	0.052739491
SLC43A1	-2.817811	1.053619	-2.674412	0.007486049	0.05274077
ZNF805	-3.790265	1.417465	-2.673974	0.007495833	0.05274077
ENSG00000258623	-2.59361	0.969955	-2.67395	0.007496367	0.05274077
CDCA3	-4.064456	1.520034	-2.673925	0.00749692	0.05274077
LINC01209	-4.056297	1.517171	-2.673592	0.007504371	0.052756407
HAAO	-2.482477	0.928564	-2.673459	0.007507343	0.052756407
LATS2.AS1	-2.59097	0.969365	-2.672852	0.007520942	0.052789293

ENSG00000232412	-3.095001	1.158032	-2.672638	0.007525737	0.052789293
BCORL1	-2.302915	0.861713	-2.672485	0.007529166	0.052789293
H2BFS	-2.493688	0.933242	-2.672071	0.007538465	0.052789293
ZNF219	-2.306529	0.86321	-2.672036	0.007539248	0.052789293
C20orf144	-3.045872	1.139908	-2.672032	0.007539346	0.052789293
PWP2	-3.303416	1.236322	-2.67197	0.007540742	0.052789293
ENSG00000260641	-3.696671	1.3837	-2.671583	0.007549433	0.052818632
CCDC85C	-2.53457	0.948773	-2.671418	0.007553142	0.052818632
EPHX2	-3.431721	1.284759	-2.671101	0.007560286	0.052839869
ROM1	-2.559892	0.958518	-2.670677	0.007569855	0.052878026
FRS3	-2.308871	0.864621	-2.670385	0.007576437	0.052895291
ATG9B	-3.381813	1.266928	-2.669302	0.007600903	0.05303732
ENSG00000228863	-3.672922	1.376408	-2.668484	0.007619444	0.053084988
MYOM1	-2.677632	1.003446	-2.668437	0.007620499	0.053084988
CNNM2	-1.999635	0.749383	-2.668375	0.007621918	0.053084988
CYB561	-2.455254	0.920228	-2.668092	0.007628337	0.053084988
RAD51	-3.787149	1.419423	-2.668091	0.007628363	0.053084988
ENSG00000238287	-2.379847	0.892081	-2.667748	0.007636155	0.053091087
TSPAN32	-2.916409	1.093234	-2.667689	0.007637491	0.053091087
ZNF79	-2.312296	0.867023	-2.666937	0.007654603	0.053181306
ACACB	-2.1892	0.821183	-2.665911	0.007678003	0.053315092
FBXO9	-1.145931	0.4299	-2.665575	0.007685672	0.053316585
STEAP4	-2.915739	1.093865	-2.665539	0.007686505	0.053316585
DARS2	-2.089831	0.78418	-2.66499	0.007699068	0.053365068
DGKH	-3.164691	1.187559	-2.664871	0.00770179	0.053365068
RFESD	-2.644511	0.992545	-2.664375	0.007713162	0.053415099
ENSG00000274104	-2.361649	0.886521	-2.66395	0.007722897	0.053436731
ZNF77	-2.086476	0.783299	-2.663703	0.007728581	0.053436731
LINC01023	-3.253782	1.221529	-2.663696	0.007728745	0.053436731
ADCK1	-2.515952	0.944751	-2.663084	0.007742813	0.053505249
ASAH2B	-2.344101	0.880501	-2.662236	0.007762336	0.053585173
DDX31	-2.130156	0.800143	-2.66222	0.007762708	0.053585173
TSNARE1	-3.454332	1.297724	-2.661839	0.007771501	0.053610664
MATR3	-2.175025	0.817166	-2.661669	0.007775435	0.053610664
ZNF512B	-2.831081	1.063709	-2.661519	0.0077789	0.053610664
TNRC6B	-0.568096	0.213514	-2.660696	0.007797941	0.053702197
ZFYVE28	-2.723595	1.023683	-2.660584	0.007800529	0.053702197
CPNE2	-3.075126	1.155998	-2.660149	0.007810604	0.053725097
PODXL2	-2.459151	0.924563	-2.659797	0.007818786	0.053725097
FAM227A	-4.07145	1.530779	-2.659725	0.007820458	0.053725097
CA2	-3.062051	1.151268	-2.65972	0.007820557	0.053725097
NEB	-2.962883	1.114131	-2.659367	0.007828762	0.053752764
SH2B1	-1.297963	0.488181	-2.658774	0.007842548	0.0538187
PSMD6.AS1	-3.339999	1.25661	-2.657945	0.007861877	0.053895822
C1orf198	-2.448144	0.921071	-2.657933	0.007862163	0.053895822
KIF11	-3.827322	1.440126	-2.65763	0.007869217	0.053915448
ANXA1	-3.673819	1.382717	-2.656957	0.007884945	0.053994456

PPP1R3F	-3.745095	1.40987	-2.656341	0.007899379	0.054064529
ALPK1	-1.793884	0.675401	-2.656028	0.00790671	0.054072884
MBTPS2	-2.056169	0.774218	-2.655802	0.007911997	0.054072884
ENSG00000258572	-2.425017	0.913153	-2.655652	0.00791553	0.054072884
CLDN23	-4.044959	1.523197	-2.655572	0.00791741	0.054072884
GHRLOS	-3.903784	1.47024	-2.655203	0.007926081	0.054103387
ENSG00000234665	-3.28367	1.236966	-2.654617	0.007939855	0.054143853
CLUH	-2.427324	0.9144	-2.654552	0.007941371	0.054143853
CCDC51	-1.730673	0.651998	-2.654414	0.007944633	0.054143853
TSC22D1.AS1	-4.158289	1.566941	-2.653762	0.007960003	0.054219881
IGKV1.5	-2.789788	1.051457	-2.653258	0.007971888	0.054272109
PABPN1	-0.779402	0.293774	-2.65307	0.007976328	0.054273619
TMEM44	-4.12375	1.554597	-2.652618	0.007987026	0.054317685
DARS.AS1	-2.096765	0.790783	-2.651505	0.008013392	0.054448745
TNFRSF4	-3.812563	1.437953	-2.651381	0.008016327	0.054448745
EXOC8	-2.016082	0.760479	-2.651069	0.008023753	0.054448745
CTPS2	-2.346152	0.885026	-2.65094	0.008026812	0.054448745
MORN3	-2.407462	0.908163	-2.650913	0.008027455	0.054448745
ENSG00000255237	-3.423996	1.291789	-2.650585	0.008035252	0.054472911
SEPTIN8	-4.181623	1.578195	-2.649624	0.008058137	0.054598207
YIPF5	-1.076953	0.406518	-2.649213	0.008067958	0.054598207
ENSG00000262151	-3.975427	1.50062	-2.649189	0.008068509	0.054598207
KLHDC8B	-3.344692	1.262578	-2.649097	0.008070707	0.054598207
MAP3K7CL	-2.079194	0.784935	-2.648874	0.00807604	0.054605572
TANC2	-2.804465	1.059075	-2.648034	0.008096149	0.05470089
LINC02604	-2.973483	1.122946	-2.64793	0.00809864	0.05470089
VPS9D1	-2.509257	0.947887	-2.647211	0.008115865	0.054788474
C9orf139	-3.998113	1.510519	-2.646847	0.00812462	0.054801777
EGR1	-2.756945	1.041677	-2.646641	0.008129562	0.054801777
FOCAD	-3.21855	1.216229	-2.646336	0.008136889	0.054801777
HIST1H2BH	-2.271726	0.858456	-2.646291	0.008137968	0.054801777
MSH5	-2.388134	0.902462	-2.646243	0.008139131	0.054801777
SOCS7	-2.047875	0.773945	-2.646021	0.008144481	0.054809119
C17orf67	-1.978035	0.747602	-2.645838	0.008148885	0.054810093
ZNF709	-3.784006	1.430556	-2.64513	0.00816596	0.054896242
ENSG00000269246	-2.590177	0.97931	-2.6449	0.00817151	0.054904867
MORC4	-3.636321	1.375159	-2.64429	0.008186242	0.054971132
IRAK1BP1	-2.091168	0.790869	-2.644138	0.008189917	0.054971132
PRMT6	-1.734249	0.655963	-2.643823	0.008197559	0.054993738
NRG2	-4.03364	1.526078	-2.643142	0.008214065	0.055075755
PSMD6.AS2	-2.855858	1.080653	-2.642716	0.0082244	0.055091737
ZNF658	-2.385491	0.902675	-2.642691	0.008225012	0.055091737
SEC22C	-1.285853	0.486637	-2.642322	0.008233979	0.055094441
ENSG00000272693	-4.084819	1.54592	-2.642322	0.008233964	0.055094441
ENSG00000255062	-2.538534	0.960971	-2.641636	0.008250668	0.055177412
AGPAT4	-2.218324	0.839812	-2.641453	0.008255127	0.055178555
CCDC153	-3.118634	1.180785	-2.641154	0.008262409	0.055198556

CAMK1	-3.523402	1.334561	-2.640121	0.008287637	0.055338363
BAIAP2	-2.837961	1.075069	-2.639794	0.008295641	0.055363079
ENSG00000238260	-2.946988	1.116504	-2.639479	0.008303361	0.055385871
KCNJ5	-4.375737	1.658634	-2.638156	0.008335812	0.055555709
GALNT14	-2.498015	0.946903	-2.638089	0.008337458	0.055555709
SELPLG	-2.466298	0.934997	-2.637761	0.008345549	0.055580836
CFAP54	-3.20165	1.213929	-2.637426	0.008353773	0.055606826
FMN1	-4.330042	1.641909	-2.6372	0.008359353	0.0556152
TCFL5	-2.358655	0.894628	-2.636463	0.008377532	0.055707339
ENSG00000262312	-2.636291	1.000307	-2.635482	0.008401777	0.055839701
ADNP	-1.113101	0.422392	-2.635235	0.008407902	0.055850597
SEC24B.AS1	-2.221291	0.842974	-2.635066	0.008412097	0.055850597
RIOX2	-1.660203	0.630115	-2.634762	0.008419623	0.055853534
TMSB15B	-2.196211	0.833602	-2.634605	0.008423514	0.055853534
C3orf18	-3.234477	1.227774	-2.634424	0.008428005	0.055853534
HOOK1	-2.682645	1.018334	-2.634348	0.008429903	0.055853534
SUFU	-2.139797	0.812351	-2.634079	0.008436582	0.055858924
ENSG00000275709	-4.032011	1.530812	-2.633903	0.008440957	0.055858924
DCBLD2	-2.462275	0.934884	-2.633776	0.008444119	0.055858924
ATP8B1	-2.256048	0.856635	-2.633617	0.008448082	0.055858924
AGBL3	-2.429923	0.922718	-2.63344	0.008452487	0.055859349
ENSG00000215068	-2.878819	1.093252	-2.633262	0.008456905	0.055859853
ENSG00000248787	-4.148543	1.576077	-2.632195	0.00848351	0.056006833
HS6ST1	-2.545478	0.967206	-2.631785	0.008493755	0.056045713
CCR7	0.4769	0.181225	2.631533	0.008500054	0.056058532
TEDC2	-3.261565	1.23989	-2.630527	0.008525263	0.056195984
CRACR2B	-4.124536	1.568195	-2.630117	0.008535547	0.056234963
GCK	-3.677685	1.398532	-2.629676	0.008546636	0.056279205
SH3BGR	-3.786443	1.440042	-2.629398	0.008553613	0.056288646
STOML1	-2.276719	0.865913	-2.629271	0.008556819	0.056288646
OXER1	-3.547869	1.349676	-2.628682	0.008571659	0.0563305
PAQR7	-2.281453	0.867912	-2.62867	0.008571937	0.0563305
ENSG00000260805	-2.185041	0.831527	-2.627744	0.008595307	0.056430825
MLXIPL	-4.046651	1.539987	-2.627718	0.008595976	0.056430825
TRIM24	-1.99767	0.760493	-2.62681	0.008618935	0.056510223
B4GAT1	-1.999177	0.76109	-2.62673	0.008620971	0.056510223
PEAK3	-3.973157	1.512615	-2.626682	0.008622193	0.056510223
ENSG00000261177	-4.471762	1.70261	-2.626415	0.008628945	0.056510223
DHRS11	-2.205324	0.839684	-2.626373	0.008630029	0.056510223
COL4A3	-4.041357	1.539151	-2.625705	0.00864697	0.056592355
ENSG00000260879	-2.462569	0.938326	-2.624427	0.008679485	0.056776272
DHX35	-2.357416	0.898373	-2.624095	0.008687951	0.056802774
ECHDC3	-2.090524	0.796845	-2.623501	0.008703125	0.056873089
IL11RA	-2.111923	0.805122	-2.623109	0.008713145	0.056909664
ZNF81	-2.406731	0.917664	-2.622671	0.008724349	0.056929748
PRNCR1	-2.247285	0.856878	-2.622643	0.008725069	0.056929748
ENSG00000270012	-3.608365	1.376223	-2.621934	0.008743236	0.056961958

ENSG00000261889	-2.49537	0.951749	-2.621877	0.008744697	0.056961958
ENSG00000257831	-2.541027	0.96919	-2.621804	0.008746577	0.056961958
AGAP5	-2.326562	0.887405	-2.621759	0.008747732	0.056961958
ENSG00000272186	-2.989987	1.140526	-2.621587	0.00875214	0.056961958
FKBP8	-0.617441	0.235576	-2.62098	0.008767741	0.057034643
ENSG00000177406	-1.945167	0.742238	-2.620677	0.008775534	0.057056493
PHF2	-2.081342	0.794302	-2.620342	0.008784174	0.05708382
NDUFB2.AS1	-2.479621	0.94652	-2.619723	0.008800113	0.057131443
NIPSNAP3B	-1.928395	0.736109	-2.619713	0.008800382	0.057131443
FAS	-1.945689	0.743184	-2.618045	0.008843513	0.057368136
SHLD3	-1.999199	0.763658	-2.617926	0.00884659	0.057368136
ZNF778	-2.038969	0.77889	-2.617786	0.008850218	0.057368136
TLCD1	-2.825362	1.079457	-2.617391	0.008860477	0.057378224
TUBG2	-2.320254	0.886479	-2.617383	0.008860693	0.057378224
ZNF618	-3.06364	1.170838	-2.616622	0.008880455	0.057477271
UGDH.AS1	-2.941209	1.124449	-2.61569	0.008904731	0.057605414
VLDLR	-3.445856	1.317869	-2.614719	0.008930095	0.057725358
VASH1.AS1	-2.451082	0.937494	-2.614505	0.008935698	0.057725358
FBF1	-2.08723	0.798339	-2.614465	0.008936731	0.057725358
TTC34	-2.273214	0.869541	-2.614269	0.008941856	0.057729483
EOGT	-3.158406	1.208758	-2.612934	0.008976872	0.057926486
ENSG00000214770	-2.036152	0.779417	-2.612403	0.008990828	0.057987463
IER5L	-3.227445	1.235544	-2.612166	0.008997046	0.057998494
LINC01535	-3.19293	1.222741	-2.611289	0.009020168	0.058082381
SLC48A1	-1.526799	0.584706	-2.611224	0.009021866	0.058082381
FKBP1B	-1.999374	0.765704	-2.611159	0.009023601	0.058082381
ENSG00000271741	-2.790994	1.068952	-2.610962	0.009028797	0.058086763
NOL6	-2.518088	0.964913	-2.609654	0.009063383	0.058280135
ZNF558	-1.91703	0.734853	-2.608727	0.009087975	0.058388519
HTRA4	-5.029205	1.927876	-2.608676	0.00908932	0.058388519
DAGLB	-1.727897	0.662485	-2.608205	0.009101843	0.058388519
RTKN	-1.919493	0.735975	-2.608097	0.009104719	0.058388519
ENSG00000272335	-2.377759	0.911684	-2.608096	0.00910475	0.058388519
SMG8	-3.507973	1.345085	-2.607994	0.009107465	0.058388519
ITGA11	-4.737151	1.816594	-2.607711	0.009114999	0.058407718
SRR	-2.166708	0.830954	-2.607495	0.009120729	0.058415339
PUS10	-2.035986	0.780873	-2.60732	0.009125391	0.058416125
ZMYND10	-3.24438	1.244418	-2.607147	0.009130017	0.058416673
LINC02062	-3.569915	1.369505	-2.60672	0.009141415	0.058438542
ENSG00000277476	-2.673821	1.025758	-2.606678	0.009142518	0.058438542
FAM71F2	-3.308587	1.269478	-2.606257	0.009153766	0.058481381
MEX3A	-3.899752	1.497026	-2.604999	0.009187459	0.058667512
ENSG00000260267	-2.213884	0.850168	-2.604055	0.009212805	0.058768349
MRPS6	0.734447	0.282046	2.604	0.009214271	0.058768349
ENSG00000274184	-1.998089	0.767362	-2.603841	0.009218548	0.058768349
ENSG00000282951	-3.41716	1.31246	-2.603629	0.009224263	0.058768349
TUNAR	-2.932626	1.126408	-2.60352	0.009227193	0.058768349

ENSG00000246528	-2.372495	0.91131	-2.603391	0.009230655	0.058768349
MCPH1.AS1	-3.818388	1.467124	-2.602635	0.00925104	0.058840575
ERMN	-2.98046	1.145172	-2.602631	0.009251145	0.058840575
ENSG00000272008	-4.015116	1.543115	-2.601956	0.009269379	0.058927421
BRPF3	-2.067033	0.794508	-2.601653	0.009277576	0.058950408
LRFN1	-2.673798	1.027824	-2.601415	0.009283994	0.058962069
HOXC4	-3.577424	1.375422	-2.600965	0.009296187	0.059010377
TWSG1	-2.285875	0.879361	-2.599473	0.009336712	0.059231298
ENSG00000224610	-3.730368	1.435119	-2.599345	0.009340196	0.059231298
CYB5D1	-3.065592	1.179526	-2.599004	0.009349457	0.059260818
KCNQ5	-2.689711	1.035042	-2.59865	0.009359121	0.059292864
NEK10	-4.01952	1.546887	-2.598458	0.009364358	0.059296845
LINC02649	-2.611137	1.005025	-2.598082	0.009374609	0.059332559
WDR48	-1.283437	0.494065	-2.597709	0.009384792	0.059367807
TMEM231	-3.33865	1.285631	-2.596896	0.009407051	0.059453463
MEGF11	-2.379374	0.916244	-2.596877	0.009407574	0.059453463
TMCC1.AS1	-2.969956	1.144182	-2.595702	0.009439778	0.059549034
LRTOMT	-1.907526	0.734918	-2.595562	0.009443644	0.059549034
ZW10	-1.939447	0.747239	-2.595483	0.009445814	0.059549034
CBWD6	-2.33762	0.900661	-2.595448	0.009446771	0.059549034
EPHX1	-2.681255	1.033123	-2.595292	0.009451055	0.059549034
RERE.AS1	-4.026487	1.551523	-2.595184	0.009454034	0.059549034
TRMT5	-2.019623	0.778231	-2.595145	0.009455093	0.059549034
GGA2	1.215692	0.468571	2.594469	0.009473724	0.05957058
LINC02576	-2.747224	1.0589	-2.594413	0.00947526	0.05957058
THRA	-1.868744	0.720303	-2.594385	0.009476034	0.05957058
MACC1	-2.534604	0.976971	-2.594349	0.009477033	0.05957058
ENSG00000226571	-1.899516	0.732434	-2.59343	0.009502384	0.05970077
ENSG00000267136	-3.985565	1.537007	-2.593069	0.009512377	0.059734383
ENSG00000233038	-2.514266	0.969868	-2.592381	0.009531425	0.059773242
THNSL2	-2.855029	1.101329	-2.592348	0.009532329	0.059773242
CENPF	-2.454517	0.946834	-2.592342	0.009532501	0.059773242
ZMYND19	-2.228459	0.859865	-2.591638	0.009552018	0.059866449
RECK	-1.993482	0.769429	-2.590859	0.00957366	0.059972873
HMGB3	-2.25299	0.86971	-2.590506	0.009583488	0.060005226
ANKEF1	-3.489029	1.347152	-2.589929	0.009599576	0.060056012
ENSG00000251417	-2.635333	1.017558	-2.58986	0.009601492	0.060056012
GPM6A	-2.301059	0.888538	-2.589713	0.009605601	0.060056012
DDN.AS1	-2.25899	0.872453	-2.589239	0.009618835	0.060081494
SNED1	-3.517556	1.358733	-2.588851	0.00962968	0.060081494
P2RY11	-2.285571	0.882934	-2.588609	0.009636435	0.060081494
SUV39H1	-2.084353	0.805223	-2.588542	0.009638325	0.060081494
LINC01869	-1.819084	0.702777	-2.588422	0.00964168	0.060081494
PCF11.AS1	-2.102905	0.812444	-2.58837	0.009643146	0.060081494
PLEKHM3	-3.903367	1.508131	-2.588215	0.009647483	0.060081494
HECTD2	-2.264234	0.87486	-2.58811	0.009650425	0.060081494
ITIH5	-3.859126	1.491209	-2.587917	0.009655837	0.060081494

ITGA6.AS1	-2.040847	0.788612	-2.587897	0.009656371	0.060081494
ARHGAP44	-2.587348	0.999893	-2.587625	0.009664015	0.060099992
TM6SF1	-3.146084	1.216129	-2.586966	0.009682519	0.060185975
SYP	-2.017419	0.780137	-2.585981	0.009710226	0.060329059
FAM114A1	-2.310389	0.893584	-2.585531	0.009722909	0.060378701
KIAA0513	-2.941513	1.137823	-2.585211	0.009731945	0.060405661
PRRT3.AS1	-4.51287	1.745849	-2.584915	0.009740311	0.060428437
MBLAC1	-2.821731	1.092103	-2.583759	0.009773018	0.06049933
ENSG00000273384	-2.654585	1.027422	-2.583733	0.009773729	0.06049933
IQCN	-2.046889	0.792325	-2.583396	0.009783291	0.06049933
ENSG00000278107	-4.008627	1.551696	-2.583385	0.009783604	0.06049933
SCFD2	-3.056396	1.183131	-2.583311	0.00978571	0.06049933
BBS12	-2.413687	0.93437	-2.583225	0.009788157	0.06049933
ZNF8	-2.056518	0.796105	-2.583225	0.00978816	0.06049933
RNF32	-2.583285	1.00004	-2.583182	0.009789353	0.06049933
ENSG00000270871	-4.137997	1.602018	-2.58299	0.009794823	0.060504077
LARS2	-2.938557	1.1378	-2.582666	0.00980402	0.060531827
ENSG00000256591	-2.357036	0.912732	-2.582396	0.009811706	0.060550227
REM2	-2.180508	0.844686	-2.581443	0.009838826	0.060686885
ENSG00000254893	-3.819535	1.479702	-2.581287	0.009843284	0.060686885
PYROXD2	-2.620448	1.01525	-2.581087	0.009848974	0.060692885
ENSG00000253140	-4.1138	1.594139	-2.580577	0.009863534	0.060745347
BSCL2	-2.778597	1.076784	-2.580458	0.009866929	0.060745347
BICDL1	-2.3431	0.908123	-2.580157	0.009875542	0.060763352
ATP11A	-1.917119	0.743062	-2.580026	0.009879298	0.060763352
TVP23A	-4.606143	1.785558	-2.579666	0.009889585	0.060769829
ENSG00000257839	-2.276017	0.882294	-2.579659	0.009889797	0.060769829
ZNF596	-2.280586	0.884182	-2.579318	0.009899547	0.060800702
MUC20	-2.563788	0.994127	-2.578935	0.009910547	0.060839222
L3MBTL1	-2.032656	0.788264	-2.578649	0.009918749	0.060860536
PXDC1	-2.247855	0.871858	-2.578237	0.009930591	0.060895997
EXTL2	-1.977308	0.766958	-2.578118	0.009933994	0.060895997
CCDC32	1.695457	0.657721	2.577773	0.00994392	0.060903764
LINC01238	-3.200801	1.241838	-2.577471	0.009952633	0.060903764
GPSM1	-3.927082	1.523642	-2.577431	0.009953786	0.060903764
TNNT3	-3.607384	1.399621	-2.5774	0.009954681	0.060903764
SETBP1	-1.577627	0.61217	-2.577105	0.009963161	0.060903764
ENSG00000259065	-3.922038	1.521887	-2.577088	0.009963661	0.060903764
ENSG00000266490	-2.60987	1.012959	-2.576481	0.009981169	0.060981815
ENSG00000262873	-4.279857	1.661259	-2.576273	0.009987185	0.06098961
CATSPERG	-2.44744	0.950165	-2.575806	0.010000676	0.061017012
PPM1L	-2.879284	1.117826	-2.575789	0.010001156	0.061017012
FGF22	-2.510528	0.974887	-2.575199	0.010018257	0.061092377
GLI1	-2.294114	0.890957	-2.574888	0.01002726	0.061118313
ENSG00000230454	-3.831677	1.488213	-2.574683	0.010033192	0.061119068
ENSG00000277369	-3.932848	1.527704	-2.574353	0.010042783	0.061119068
FSIP2	-2.115373	0.821866	-2.573866	0.010056933	0.061119068

ZNF853	-3.832563	1.48904	-2.573849	0.01005741	0.061119068
PRMT2	-0.737032	0.286376	-2.573646	0.010063324	0.061119068
CDC14B	-2.401956	0.933384	-2.573385	0.010070914	0.061119068
PRH1	-2.960371	1.150388	-2.573368	0.010071401	0.061119068
ZNF839	-2.364108	0.918727	-2.573243	0.010075035	0.061119068
GPD2	-2.084962	0.810251	-2.573232	0.010075378	0.061119068
FAM66C	-2.584995	1.004598	-2.573163	0.010077383	0.061119068
ITGAX	-2.899602	1.126926	-2.57302	0.010081528	0.061119068
SPEF2	-2.461024	0.956509	-2.572922	0.010084385	0.061119068
VPS37C	-1.861059	0.723437	-2.572523	0.010096027	0.061160817
CDC37L1.DT	-2.24952	0.87461	-2.572027	0.010110492	0.061219623
VRK3	1.819368	0.707634	2.571057	0.010138871	0.061362586
FAM122C	-1.95658	0.761337	-2.569926	0.010172033	0.061512264
DISC1	-3.888432	1.513075	-2.569887	0.010173164	0.061512264
ENSG00000267811	-4.071313	1.584354	-2.5697	0.010178668	0.061516639
ENSG00000278238	-2.365382	0.920594	-2.56941	0.010187191	0.061539241
NQO1	-2.734116	1.064756	-2.567833	0.010233637	0.061790805
LYSMD1	-2.199689	0.856713	-2.567591	0.010240791	0.061805
ZKSCAN3	-2.19686	0.8558	-2.567025	0.010257515	0.061876907
ENSG00000237773	-1.902298	0.741212	-2.566469	0.010273972	0.06193538
CERS6	-2.025826	0.7894	-2.566285	0.010279423	0.06193538
PLXNA1	-2.223723	0.866594	-2.566047	0.010286474	0.06193538
MFAP3	-2.417183	0.94205	-2.565875	0.010291586	0.06193538
LETM1	-1.286872	0.501543	-2.565827	0.010293013	0.06193538
SVOP	-3.623308	1.412222	-2.565679	0.010297421	0.06193538
FIZ1	-2.365406	0.921984	-2.565561	0.010300902	0.06193538
C9orf163	-3.883125	1.513676	-2.565362	0.010306829	0.061942067
RAB35	-1.42387	0.555149	-2.564843	0.010322256	0.0619919
TNF	-2.266509	0.883733	-2.5647	0.010326512	0.0619919
ENSG00000277007	-2.323186	0.905878	-2.564567	0.010330453	0.0619919
THUMPD1	-1.10305	0.430174	-2.564198	0.010341468	0.0619919
TRAIP	-2.517436	0.981768	-2.564187	0.010341783	0.0619919
ENSG00000275457	-2.028625	0.791283	-2.563715	0.010355839	0.0619919
SPINDOC	-2.168688	0.845938	-2.563649	0.01035782	0.0619919
NAV1	-2.479754	0.967348	-2.563456	0.010363572	0.0619919
ALG10	-2.246756	0.876473	-2.563407	0.010365051	0.0619919
C6orf52	-3.990712	1.556835	-2.563348	0.010366798	0.0619919
ENSG00000267939	-3.756653	1.465551	-2.563304	0.010368118	0.0619919
B3GNT7	-1.679794	0.655506	-2.56259	0.010389452	0.062090611
PPP2CB	-1.795735	0.7008	-2.56241	0.010394867	0.062094126
C8orf58	-1.827991	0.713495	-2.562022	0.01040646	0.062134533
SLC25A12	-1.973351	0.770412	-2.561423	0.010424424	0.062212926
ENSG00000229729	-2.035301	0.794695	-2.561111	0.010433815	0.062240102
JUNB	0.872324	0.340712	2.560293	0.01045838	0.062357726
IPO11	-1.990996	0.777803	-2.55977	0.010474144	0.062404134
ENSG00000225205	-2.255066	0.880984	-2.559713	0.010475863	0.062404134
PPP1R9A	-4.070383	1.590308	-2.559495	0.010482451	0.062414485

SERP2	-2.635966	1.029999	-2.559193	0.010491534	0.06243967
ENSG00000268352	-2.771673	1.083237	-2.558696	0.010506565	0.06250022
PIGV	-1.764412	0.689849	-2.557679	0.010537325	0.062636907
DSEL	-3.541186	1.384566	-2.557615	0.010539279	0.062636907
B4GALT2	-4.025378	1.574159	-2.557161	0.010553045	0.062689766
ARMCX2	-1.678805	0.656659	-2.556584	0.010570549	0.062722303
ENSG00000273156	-2.451317	0.958831	-2.556568	0.010571055	0.062722303
KANK1	-2.715596	1.062232	-2.556499	0.010573146	0.062722303
CPEB2	-2.267609	0.887122	-2.556141	0.010584024	0.062757899
ENSG00000215014	-2.000258	0.782969	-2.55471	0.010627631	0.062987437
C10orf25	-2.639064	1.033099	-2.554512	0.010633678	0.062994261
ENSG00000187186	-2.385993	0.934196	-2.554059	0.010647508	0.063024642
MMP17	-2.164478	0.847503	-2.553948	0.010650931	0.063024642
IFITM3	-2.327317	0.911293	-2.553864	0.010653501	0.063024642
NR1I3	-3.349636	1.311785	-2.553494	0.010664801	0.063062498
BACH1.AS1	-2.644511	1.035921	-2.552811	0.010685744	0.06315731
AREG	-3.806045	1.491217	-2.552308	0.01070118	0.063207231
EMILIN2	-2.171181	0.850704	-2.552216	0.010704015	0.063207231
ENSG00000267260	-3.567137	1.398003	-2.551594	0.010723137	0.063274636
SMAD2	-1.095929	0.429519	-2.551525	0.010725265	0.063274636
ASPHD2	-2.2569	0.884611	-2.551292	0.010732449	0.063288001
TLR4	-5.086459	1.993928	-2.550974	0.010742243	0.063291857
OTUD7B	-3.364107	1.318911	-2.55067	0.010751601	0.063291857
ATP13A4	-3.721872	1.459183	-2.550655	0.010752084	0.063291857
ENSG00000275055	-2.476494	0.970934	-2.550632	0.010752778	0.063291857
CABP4	-3.590612	1.407866	-2.550392	0.010760173	0.063306424
ENSG00000273010	-3.637135	1.426394	-2.54988	0.010775988	0.063370492
ENSG00000258376	-1.832873	0.718924	-2.549466	0.010788793	0.063416812
ENSG00000273149	-3.175403	1.245789	-2.548908	0.010806073	0.063481032
FUT2	-2.977597	1.168294	-2.548671	0.010813434	0.063481032
ENSG00000272572	-2.580404	1.012465	-2.548636	0.01081452	0.063481032
RPS6KB2.AS1	-2.077861	0.815386	-2.548317	0.010824413	0.063482652
ENSG00000272356	-2.504851	0.98296	-2.548272	0.010825793	0.063482652
TTLL7	-2.454219	0.963138	-2.54815	0.010829597	0.063482652
NDRG2	-2.765398	1.085575	-2.547404	0.010852756	0.063589443
SLFN12	-2.187563	0.858926	-2.546859	0.010869723	0.063641631
ENSG00000226578	-3.637253	1.428166	-2.546801	0.010871555	0.063641631
CCDC154	-1.969336	0.773372	-2.546426	0.010883216	0.063651946
PDIA5	-2.224388	0.873617	-2.546181	0.010890863	0.063651946
IGHMBP2	-1.914241	0.751841	-2.546071	0.010894304	0.063651946
TXLNB	-2.61538	1.027246	-2.546011	0.010896188	0.063651946
ZNF324B	-2.34962	0.922885	-2.545951	0.010898052	0.063651946
ENSG00000259802	-3.890316	1.528641	-2.544951	0.010929305	0.063805522
MRPS24	-1.936353	0.761	-2.544485	0.010943906	0.063861789
URB2	-2.276436	0.894768	-2.544163	0.010953996	0.06389169
CDAN1	-2.035048	0.800074	-2.543574	0.010972493	0.063970578
LINC01431	-2.174928	0.855259	-2.543006	0.01099033	0.064015016

FAM86C1	-2.447718	0.962565	-2.542912	0.010993306	0.064015016
ZNF425	-3.839724	1.510004	-2.542856	0.010995041	0.064015016
TNFRSF9	-4.090476	1.608866	-2.542459	0.011007537	0.064048669
CPT1B	-3.433845	1.350654	-2.542357	0.011010776	0.064048669
LSR	-1.618965	0.636839	-2.54219	0.011016036	0.06405031
CHEK2	-1.95266	0.768309	-2.541504	0.011037667	0.064147089
ENSG00000269983	-2.313012	0.910308	-2.54091	0.01105644	0.064222995
ZNF517	-3.082186	1.213089	-2.540775	0.01106071	0.064222995
FBXO4	-1.470595	0.578888	-2.54038	0.011073219	0.064266628
AQP11	-4.003223	1.576245	-2.539722	0.011094074	0.064358634
HCN3	-2.546221	1.002793	-2.539129	0.011112881	0.064438682
ENSG00000229255	-5.056356	1.991667	-2.538756	0.011124739	0.064458728
CTH	-2.869587	1.130388	-2.538586	0.011130159	0.064458728
CCDC61	-3.399895	1.339307	-2.538548	0.011131367	0.064458728
AGBL2	-2.964594	1.167976	-2.538232	0.011141397	0.064487788
SYCP3	-2.033726	0.801786	-2.536493	0.011196896	0.064779886
AFDN	-2.275893	0.897339	-2.53627	0.011204033	0.064783502
ADHFE1	-3.120857	1.230545	-2.536159	0.011207591	0.064783502
ENSG00000257497	-2.16292	0.852888	-2.535995	0.011212847	0.064784777
DEGS2	-2.880123	1.135845	-2.535666	0.011223358	0.064816404
SUSD1	-2.273611	0.896829	-2.535165	0.011239425	0.064847611
FBXL19	-2.244959	0.885617	-2.534909	0.01124764	0.064847611
ENSG00000267383	-2.277509	0.898463	-2.534896	0.011248077	0.064847611
ENSG00000273486	-2.516727	0.992843	-2.53487	0.011248921	0.064847611
KCTD3	-2.71072	1.069476	-2.534625	0.011256776	0.06486383
TMEM273	-2.012485	0.794098	-2.534304	0.011267106	0.064894293
RELCH	-1.749155	0.690479	-2.533249	0.011301053	0.065060691
ENSG00000251602	-3.479311	1.373697	-2.532808	0.011315292	0.065113536
SEPTIN1	-0.785617	0.310202	-2.532599	0.011322028	0.065123169
CAMSAP1	-1.876602	0.741535	-2.530699	0.011383536	0.065356246
MYZAP	-3.071657	1.213763	-2.53069	0.011383841	0.065356246
ENSG00000260507	-2.275933	0.899436	-2.530399	0.011393285	0.065356246
LINC02550	-5.100469	2.015768	-2.530286	0.011396971	0.065356246
SLX1A	-2.285564	0.903305	-2.530225	0.011398946	0.065356246
ZFHX2	-2.262924	0.894402	-2.530099	0.011403039	0.065356246
FKBP14	-2.14011	0.845895	-2.529996	0.011406393	0.065356246
PPP1R13B	-2.329315	0.920693	-2.529959	0.011407583	0.065356246
FKBPL	-1.726598	0.682467	-2.529938	0.011408264	0.065356246
ZNF653	-2.65873	1.051214	-2.529198	0.011432338	0.065465017
ENSG00000266498	-3.834237	1.516378	-2.528549	0.011453511	0.065557085
ZNF174	-1.855997	0.734308	-2.527545	0.011486306	0.065687631
PUDP	-1.794642	0.710036	-2.527538	0.011486529	0.065687631
ENSG00000259274	-2.653833	1.050843	-2.525433	0.011555576	0.066053129
IGLV1.47	-3.328799	1.318218	-2.525227	0.011562358	0.066053168
CD68	-1.560586	0.618024	-2.525121	0.01156585	0.066053168
PDK4	-2.393249	0.947872	-2.524865	0.011574291	0.066072048
ENSG00000272256	-3.422419	1.355919	-2.524057	0.011600899	0.066154553

SAMD9L	1.77257	0.702332	2.523835	0.011608239	0.066154553
CARD16	-0.995712	0.394526	-2.523821	0.011608691	0.066154553
SMTN	-3.291107	1.304027	-2.523803	0.011609309	0.066154553
TOMM40L	-2.194476	0.869588	-2.523583	0.011616561	0.06616511
ENSG00000242539	-2.563487	1.015876	-2.523425	0.011621769	0.06616511
TENT4A	-2.025925	0.802893	-2.52328	0.011626589	0.06616511
ZNF460	-2.289792	0.907533	-2.523096	0.011632671	0.066170461
TMCC3	-2.446538	0.969894	-2.522481	0.011653034	0.066256999
PEX11A	-2.29943	0.911735	-2.522039	0.011667691	0.066281254
C15orf62	-1.566225	0.62106	-2.521856	0.011673741	0.066281254
SLC35E4	-2.505281	0.993482	-2.521716	0.011678396	0.066281254
ENSG00000265008	-3.319298	1.316291	-2.521704	0.011678778	0.066281254
NKILA	-2.505533	0.993638	-2.521576	0.011683056	0.066281254
DGKQ	-1.999992	0.793247	-2.521272	0.011693146	0.066309258
USP27X	-2.404139	0.953601	-2.521116	0.011698329	0.066309427
ENSG00000197813	-3.182981	1.262733	-2.520708	0.011711887	0.066352472
YTHDF3.AS1	-3.858829	1.530938	-2.520565	0.011716652	0.066352472
ENSG00000276651	-2.26313	0.897917	-2.520423	0.011721393	0.066352472
CACNA2D4	-3.682236	1.461095	-2.520188	0.011729201	0.066354878
RBM41	-1.262502	0.500973	-2.520101	0.011732132	0.066354878
NADK2	-1.859695	0.738069	-2.519677	0.01174626	0.06640559
RORA	-2.274577	0.902815	-2.519427	0.011754617	0.066423652
IGHV3.15	-2.368712	0.940347	-2.518976	0.01176966	0.066469859
RBM15.AS1	-3.766077	1.495144	-2.518873	0.011773126	0.066469859
SLC26A4	-3.783976	1.502667	-2.518174	0.01179652	0.066558543
MEGF9	-1.810831	0.719128	-2.518094	0.011799179	0.066558543
ENSG00000269148	-3.047794	1.210626	-2.517535	0.011817923	0.066635064
LINC01943	-3.934978	1.563142	-2.517352	0.011824068	0.066640512
RND2	-3.502695	1.391603	-2.517021	0.011835175	0.066673905
BTBD3	-2.256055	0.896653	-2.516085	0.011866667	0.066822059
SLC22A5	-1.997706	0.794112	-2.515647	0.011881422	0.06687588
SLC11A1	-3.139352	1.248124	-2.515256	0.011894602	0.066920789
ZNF816.ZNF321P	-2.287529	0.909626	-2.514803	0.011909889	0.066963896
DDX47	-2.616202	1.040355	-2.514721	0.011912673	0.066963896
GRASP	-2.266231	0.901849	-2.512872	0.011975278	0.067277052
FITM2	-2.633176	1.047919	-2.512767	0.011978839	0.067277052
IL4I1	-4.969003	1.97782	-2.512363	0.011992563	0.067310449
CLTCL1	-3.747475	1.491661	-2.512284	0.011995248	0.067310449
CRIM1	-1.943236	0.773683	-2.511671	0.01201611	0.067378845
ENSG00000279927	-3.007928	1.197653	-2.511519	0.012021285	0.067378845
ENSG00000280240	-3.33784	1.329041	-2.511464	0.012023147	0.067378845
APOBR	-3.069865	1.222551	-2.511033	0.012037859	0.067431924
NFE2L3	-3.744309	1.491645	-2.510188	0.012066694	0.067564033
OIP5	-2.342853	0.933438	-2.509919	0.012075886	0.067586091
ZNF579	-2.366698	0.943062	-2.50959	0.012087139	0.067607321
SMCO4	-1.898444	0.75655	-2.509344	0.012095571	0.067607321
MYH11	-1.887968	0.752388	-2.509301	0.012097028	0.067607321

ENSG00000245970	-2.328405	0.927949	-2.509194	0.012100696	0.067607321
TNKS2.AS1	-2.733107	1.089374	-2.508879	0.012111511	0.067634482
ENSG00000273145	-3.45875	1.378677	-2.508746	0.012116071	0.067634482
AIFM2	-1.837595	0.732694	-2.507998	0.012141741	0.067748389
ENSG00000269696	-2.246151	0.895672	-2.507783	0.012149132	0.067752352
HSPA1A	-2.621526	1.045403	-2.507671	0.012152983	0.067752352
PAX8	-3.02902	1.208056	-2.507351	0.012163978	0.06778428
LINGO3	-2.033368	0.81112	-2.506865	0.012180725	0.067848222
CARD6	-2.167536	0.864832	-2.506308	0.012199933	0.067925808
USP54	-2.351434	0.938374	-2.50586	0.012215391	0.067936267
ENSG00000272112	-2.412649	0.962808	-2.505847	0.012215836	0.067936267
ENSG00000224790	-2.922289	1.166252	-2.505711	0.012220563	0.067936267
SLC31A2	-2.235121	0.892035	-2.505642	0.012222931	0.067936267
GNLY	-3.741856	1.493711	-2.505074	0.012242583	0.068016113
ZCCHC14	-2.527232	1.009249	-2.504072	0.012277299	0.068151033
ENSG00000272822	-4.778621	1.908344	-2.504067	0.012277461	0.068151033
UNC13D	-1.305464	0.521482	-2.503375	0.012301517	0.068247826
MFSD14A	-1.210028	0.483381	-2.50326	0.012305507	0.068247826
C1orf112	-2.127021	0.850339	-2.501378	0.012371096	0.068582031
TMTC2	-3.895758	1.557643	-2.50106	0.012382228	0.068614181
IKZF4	-2.283521	0.913096	-2.500857	0.012389335	0.068624012
ENSG00000261248	-2.785008	1.113814	-2.500426	0.01240442	0.068678002
ZC3H15	1.329052	0.531597	2.500109	0.012415506	0.068709812
TMEM88	-5.124481	2.050885	-2.498668	0.012466106	0.068960183
ZNF696	-2.180011	0.872601	-2.498292	0.012479338	0.069003716
WDR11	1.74664	0.699426	2.497249	0.012516111	0.069177322
RUNDC3B	-3.158855	1.265072	-2.496976	0.012525739	0.069200807
FBP1	-2.011771	0.805761	-2.496735	0.01253425	0.069218107
WDHD1	-2.146152	0.859722	-2.496333	0.012548466	0.069266884
ENSG00000080031	-2.534389	1.015404	-2.495941	0.012562333	0.069301214
ATP8B2	-2.232713	0.894595	-2.495782	0.012567988	0.069301214
MYT1L	-3.474084	1.392031	-2.495694	0.012571085	0.069301214
DUSP5	-2.425862	0.972075	-2.495549	0.012576229	0.069301214
KRT8	-2.695693	1.080346	-2.495213	0.012588164	0.069304671
RABGAP1L	1.076536	0.431446	2.495181	0.012589297	0.069304671
TSHZ2	-4.808351	1.927223	-2.494964	0.012596993	0.069304671
C19orf66	-0.741544	0.297221	-2.494924	0.012598401	0.069304671
ENSG00000263731	-2.608118	1.045579	-2.494425	0.012616137	0.06937258
PDIK1L	-2.0186	0.809621	-2.493266	0.012657389	0.06956968
ANKRD46	-1.433156	0.574921	-2.49279	0.012674373	0.069607272
CASK	-2.206338	0.885148	-2.49262	0.012680463	0.069607272
ZNF69	-2.263852	0.908254	-2.492531	0.012683627	0.069607272
ALG11	-1.81563	0.728446	-2.492468	0.012685867	0.069607272
CXCR3	-3.879484	1.556715	-2.492096	0.012699165	0.069630496
INCENP	-3.242538	1.301154	-2.492047	0.012700923	0.069630496
ENSG00000269549	-2.935113	1.178168	-2.491251	0.012729402	0.069724801
SLC45A4	-3.286167	1.319141	-2.491141	0.012733359	0.069724801

FYN	-1.858213	0.745937	-2.491112	0.012734381	0.069724801
TUSC1	-2.892223	1.161313	-2.490476	0.012757208	0.069820076
ENSG00000256448	-3.109076	1.248658	-2.489935	0.012776654	0.069896771
ARSB	-2.158408	0.867058	-2.489347	0.0127978	0.06995866
ENSG00000228857	-2.3411	0.940458	-2.489318	0.012798841	0.06995866
ENSG00000273729	-2.780754	1.11747	-2.488437	0.012830587	0.070015682
KCTD21	-2.409759	0.968389	-2.48842	0.012831205	0.070015682
DCXR.DT	-3.008566	1.209089	-2.488291	0.012835854	0.070015682
ZNF48	-1.464042	0.588411	-2.488127	0.012841777	0.070015682
FKBP1C	-4.952173	1.99041	-2.488017	0.012845763	0.070015682
SETDB2	-1.028244	0.41328	-2.488007	0.01284611	0.070015682
SLC25A23	-2.042603	0.820998	-2.487953	0.01284808	0.070015682
ENSG00000250397	-3.811635	1.532117	-2.487822	0.012852805	0.070015682
LINC02285	-2.479783	0.996833	-2.487662	0.012858585	0.070017523
LEPR	-3.53839	1.422835	-2.486859	0.012887636	0.070146027
ENSG00000055483	-1.626138	0.653965	-2.486582	0.012897694	0.070171088
ENSG00000272010	-3.62507	1.45852	-2.485444	0.012938981	0.070349488
DRAXIN	-4.723058	1.900405	-2.48529	0.012944582	0.070349488
ITFG2.AS1	-1.696706	0.682717	-2.485227	0.012946886	0.070349488
SLC9A1	-2.264698	0.911387	-2.484891	0.012959126	0.070370518
HCST	-1.628698	0.655459	-2.48482	0.012961695	0.070370518
ENSG00000274987	-3.831961	1.542284	-2.484602	0.012969624	0.070383871
PACSIN3	-3.190613	1.284457	-2.484016	0.012991007	0.070470186
CYFIP1	-3.803128	1.53158	-2.48314	0.013022985	0.070613886
NRBP2	-2.29492	0.924267	-2.482962	0.013029484	0.070619365
BCKDHA	-2.11926	0.853589	-2.482763	0.013036785	0.070629182
MARS2	-2.047123	0.82461	-2.482535	0.013045118	0.070634197
ENSG00000255882	-3.4097	1.373529	-2.482438	0.013048689	0.070634197
SYAP1	1.772749	0.714169	2.482255	0.01305538	0.070640694
PARS2	-1.916273	0.772071	-2.481989	0.01306514	0.070663789
VWA8	-1.953241	0.787076	-2.481644	0.013077803	0.070701818
GAB2	-2.021215	0.814514	-2.481498	0.013083161	0.070701818
ENSG00000247363	-2.227033	0.897598	-2.481104	0.013097609	0.070727786
DENND2C	-3.327856	1.341327	-2.481018	0.01310078	0.070727786
ENSG00000231609	-2.274825	0.916939	-2.480889	0.013105519	0.070727786
TTLL12	-2.10648	0.849124	-2.480768	0.013109953	0.070727786
C1RL	-1.957019	0.789074	-2.480147	0.013132829	0.070821505
CACYBP	0.97769	0.394245	2.479902	0.01314186	0.07082248
COLCA1	-2.247348	0.906258	-2.479811	0.013145218	0.07082248
EARS2	-2.594431	1.046289	-2.479649	0.013151169	0.07082248
MOXD1	-2.364053	0.953422	-2.479545	0.013155027	0.07082248
ATXN7L1	-1.771803	0.71486	-2.47853	0.013192508	0.070983173
CHTF18	-1.774288	0.71589	-2.478438	0.013195908	0.070983173
ENSG00000277825	-2.663435	1.074806	-2.478061	0.01320984	0.07102842
MXD3	-2.110688	0.851877	-2.477691	0.013223553	0.071068697
HHAT	-2.01108	0.811718	-2.477561	0.013228377	0.071068697
ARHGAP20	-5.10185	2.059503	-2.477223	0.013240905	0.071076647

ZBTB32	-5.10185	2.059503	-2.477223	0.013240905	0.071076647
GTF3A	0.665503	0.268692	2.476821	0.013255829	0.071097515
ZNF792	-2.629657	1.061707	-2.476821	0.013255844	0.071097515
PHETA2	-3.163399	1.277347	-2.476538	0.013266353	0.071124236
ENSG00000235652	-3.313868	1.338474	-2.475855	0.013291743	0.071202618
ENSG00000254501	-3.064306	1.23768	-2.475847	0.013292041	0.071202618
GLIPR1	-0.727409	0.293913	-2.474909	0.013326999	0.071307958
LINC02018	-2.003581	0.80959	-2.474811	0.013330686	0.071307958
CKAP4	-3.185736	1.287265	-2.474809	0.013330743	0.071307958
SLC1A5	-2.061966	0.83321	-2.474725	0.013333873	0.071307958
DUSP6	-2.602407	1.051709	-2.474456	0.01334391	0.071308777
SCYL3	-1.55934	0.630202	-2.474347	0.013347982	0.071308777
INKA2	-1.904898	0.769881	-2.474276	0.013350652	0.071308777
HIST1H2AK	-3.720245	1.503764	-2.473956	0.013362601	0.071342981
BHLHE41	-1.832426	0.740857	-2.473386	0.013383965	0.071427405
NR4A1	-2.248856	0.909328	-2.473097	0.013394791	0.071455545
ENSG00000256940	-2.373025	0.959657	-2.472784	0.013406519	0.071488469
NAIF1	-1.753104	0.709144	-2.472143	0.013430588	0.071587148
ENSG00000260735	-3.201026	1.294989	-2.471856	0.01344135	0.071614847
MFSD4A	-2.166245	0.876691	-2.470934	0.01347608	0.071747005
ZBTB37	-1.435201	0.580841	-2.470901	0.013477307	0.071747005
NOCT	-2.137561	0.865188	-2.470633	0.013487433	0.07175958
DTX3	-2.253901	0.91231	-2.470543	0.013490823	0.07175958
ENSG00000242622	-3.423054	1.385641	-2.470376	0.013497094	0.071763268
PBLD	-3.025616	1.225193	-2.469502	0.013530141	0.071891299
IGHEP1	-2.897897	1.173502	-2.469443	0.013532348	0.071891299
RHOB	-2.116531	0.857168	-2.469214	0.013541017	0.071907663
LRRC8C.DT	-2.49999	1.012785	-2.468431	0.013570693	0.072035525
CDYL	-1.70719	0.691653	-2.468276	0.013576563	0.072036964
GIMAP1	-3.143488	1.273728	-2.467943	0.013589199	0.072074286
TMEM191C	-1.63102	0.660964	-2.467637	0.013600804	0.072106116
SNTB1	-4.910057	1.990605	-2.466616	0.013639665	0.072242913
ZNF718	-2.387126	0.967787	-2.46658	0.013641007	0.072242913
ERP27	-2.641059	1.070765	-2.466516	0.013643451	0.072242913
SAMD10	-1.937079	0.785473	-2.466129	0.013658212	0.072264111
ENSG00000272906	-2.074565	0.841227	-2.466117	0.013658686	0.072264111
ENSG00000253636	-3.599656	1.460283	-2.46504	0.013699781	0.072451742
TIMELESS	-1.547423	0.627849	-2.464642	0.013715031	0.072502589
C19orf54	-2.326882	0.944182	-2.464442	0.013722663	0.072513145
OTUD3	-1.932548	0.78424	-2.46423	0.013730791	0.072526307
ENSG00000227373	-5.092837	2.068641	-2.461924	0.013819411	0.072964448
GINS1	-3.626419	1.473326	-2.461383	0.013840257	0.072981394
ENSG00000246982	-4.853658	1.971948	-2.461353	0.01384143	0.072981394
ENSG00000272787	-4.086476	1.660274	-2.461326	0.013842439	0.072981394
CDKL3	-2.083091	0.846354	-2.461252	0.013845308	0.072981394
ENSG00000248932	-3.822336	1.553286	-2.460806	0.013862519	0.073031631
AGBL5	-1.762934	0.716435	-2.460705	0.013866449	0.073031631

VPS33B	-1.740412	0.707322	-2.460564	0.013871867	0.073031631
GRAP2	-2.494747	1.014144	-2.459953	0.013895509	0.073123346
ESAM	-2.214069	0.900094	-2.45982	0.013900653	0.073123346
TBC1D10B	-1.746466	0.710149	-2.459295	0.013921	0.073166998
EXTL3	-2.041973	0.830328	-2.459236	0.013923287	0.073166998
NUDT6	-2.695246	1.096	-2.459166	0.013926011	0.073166998
TVP23C	-2.282099	0.928496	-2.457846	0.013977313	0.073377461
IVNS1ABP	-1.444187	0.587583	-2.457842	0.013977474	0.073377461
ECE2	-2.07266	0.843445	-2.457373	0.013995722	0.073424837
COQ10A	-1.48031	0.60241	-2.457312	0.013998105	0.073424837
CTPS1	-2.454854	0.999112	-2.457037	0.014008843	0.073424837
ENSG00000262728	-2.958721	1.204189	-2.457024	0.014009324	0.073424837
PRR34	-2.971312	1.20951	-2.456624	0.014024925	0.073464112
GNGT2	-1.747481	0.711363	-2.456526	0.014028771	0.073464112
ENSG00000179094	-2.107995	0.85825	-2.456156	0.01404321	0.073464112
BTBD19	-2.879211	1.172266	-2.456107	0.014045138	0.073464112
TTC4	-2.100202	0.855096	-2.456101	0.014045365	0.073464112
ENSG00000247796	-2.682085	1.09254	-2.454907	0.014092101	0.073678611
DDR1	-2.267503	0.92396	-2.454113	0.014123263	0.073811546
ZNF283	-1.81706	0.74055	-2.453663	0.014140928	0.073873862
CUBN	-2.125092	0.866146	-2.453503	0.01414723	0.073876788
CABYR	-2.56698	1.04658	-2.452731	0.014177624	0.074005469
TNIK	-3.436221	1.401421	-2.451955	0.014208255	0.074135286
RSPH9	-3.753701	1.531728	-2.450631	0.014260616	0.07435381
ENSG00000263220	-2.096774	0.855615	-2.450604	0.014261693	0.07435381
GH1	-3.550637	1.449182	-2.450098	0.014281736	0.074378107
ENSG00000268400	-3.077938	1.256261	-2.450079	0.014282468	0.074378107
NAP1L6	-4.49742	1.835645	-2.450049	0.014283695	0.074378107
TTC13	-1.65413	0.675474	-2.448842	0.014331624	0.074587387
BACH1.IT2	-3.500036	1.429318	-2.448745	0.014335479	0.074587387
CA11	-1.301119	0.531424	-2.448364	0.014350642	0.074615491
ENSG00000273669	-2.923223	1.193972	-2.448318	0.014352479	0.074615491
ZBED4	-1.722709	0.703753	-2.447889	0.014369596	0.074651357
SVIL.AS1	-2.492383	1.018191	-2.447854	0.014370981	0.074651357
GGACT	-2.390504	0.977067	-2.446613	0.014420569	0.074873872
TRMT13	-0.976683	0.399218	-2.446491	0.014425455	0.074873872
GIHCG	-1.13635	0.464638	-2.445669	0.014458365	0.074985235
CFAP70	-2.885025	1.179687	-2.445586	0.014461703	0.074985235
IMPDH1	-1.299031	0.531188	-2.445519	0.014464394	0.074985235
GDI2	0.675992	0.276443	2.445319	0.014472414	0.074996597
SLC41A1	-1.774865	0.725997	-2.444728	0.01449613	0.075021886
PCLAF	-3.044107	1.245172	-2.444728	0.014496134	0.075021886
LINC01011	-2.106746	0.861806	-2.44457	0.014502474	0.075021886
LINC01551	-2.163102	0.884885	-2.444501	0.014505272	0.075021886
ERMP1	-3.806769	1.557297	-2.444472	0.014506447	0.075021886
IPPK	-1.952846	0.79904	-2.443991	0.014525789	0.075086826
GZMB	-5.085357	2.080898	-2.443828	0.014532346	0.075086826

ENSG00000244036	-2.067543	0.846132	-2.443524	0.014544593	0.075086826
ENSG00000261786	-3.908967	1.599773	-2.44345	0.014547567	0.075086826
CCR9	-2.289461	0.936985	-2.443435	0.014548182	0.075086826
PLGLB1	-3.049665	1.248291	-2.443071	0.014562872	0.075132507
EEF1AKMT1	-1.72132	0.70469	-2.442663	0.014579348	0.075187362
SPECC1	-2.022294	0.827977	-2.442453	0.014587826	0.075200942
TMEM140	-1.447052	0.592579	-2.441955	0.014607982	0.075231574
FRMD6.AS1	-2.620896	1.073299	-2.441906	0.014609968	0.075231574
GCM1	-4.910006	2.010754	-2.441872	0.014611308	0.075231574
SGSH	-1.950378	0.798879	-2.441395	0.014630653	0.075291765
FNBP1	0.705009	0.288785	2.441295	0.014634702	0.075291765
AGO2	-1.009924	0.413733	-2.441004	0.014646482	0.075322258
TMEM86B	-3.672815	1.504941	-2.440503	0.014666815	0.075396688
IGLC5	-3.266568	1.338694	-2.440115	0.014682596	0.075399214
GATD3B	-2.211938	0.906532	-2.44	0.014687275	0.075399214
ADAP1	-2.925382	1.198949	-2.439955	0.014689074	0.075399214
ZNF398	-1.808464	0.7412	-2.439914	0.014690746	0.075399214
ITSN1	-3.076966	1.261208	-2.439697	0.01469957	0.07541442
ZNF594	-1.687939	0.692014	-2.439171	0.014721008	0.075494306
RELL2	-1.728305	0.708668	-2.438809	0.014735755	0.075539827
USP46.AS1	-4.909056	2.013213	-2.438418	0.014751705	0.075561382
MMP14	-4.909056	2.013213	-2.438418	0.014751705	0.075561382
CHIT1	-2.663528	1.092738	-2.437481	0.014789978	0.075653345
TACC1	-1.204657	0.494226	-2.437462	0.014790787	0.075653345
ACOT7	-3.555824	1.458853	-2.437412	0.014792827	0.075653345
GALNT6	-3.527535	1.44726	-2.437389	0.014793753	0.075653345
ENSG00000227540	-1.974752	0.810235	-2.437259	0.014799057	0.075653345
BCYRN1	-3.436839	1.410312	-2.436935	0.014812352	0.075691237
STXBP4	-2.121141	0.870659	-2.436247	0.014840538	0.075805162
RASGRP1	-1.8269	0.749937	-2.436072	0.014847729	0.075811799
SLC4A8	-3.109197	1.276616	-2.435499	0.014871278	0.075901915
HIST1H2AG	-1.906062	0.782729	-2.435151	0.01488559	0.075944841
MOCS2	-1.110313	0.456011	-2.434836	0.014898522	0.075963459
ZNF442	-2.171451	0.891864	-2.434733	0.014902787	0.075963459
NBPF14	-1.010559	0.415077	-2.434632	0.014906951	0.075963459
LINC01754	-2.225908	0.914381	-2.434333	0.014919243	0.075986282
ZKSCAN8	-1.597205	0.656142	-2.434237	0.014923241	0.075986282
PRDM10	-3.477991	1.429009	-2.433848	0.014939278	0.076009191
ENSG00000260588	-2.582411	1.061044	-2.433841	0.014939554	0.076009191
C11orf98	-1.842322	0.757098	-2.433402	0.014957703	0.076049893
HGH1	-2.147314	0.882448	-2.433361	0.014959375	0.076049893
SIDT2	-1.340416	0.550886	-2.433199	0.014966085	0.076053955
MAP1LC3A	-1.888425	0.776391	-2.432312	0.015002771	0.076210286
NECTIN3	-3.853994	1.585033	-2.431491	0.015036812	0.076311443
DONSON	-2.459017	1.011346	-2.431428	0.015039418	0.076311443
ENSG00000270659	-2.049729	0.843023	-2.431403	0.015040477	0.076311443
ENSG00000271870	-1.914466	0.787749	-2.430299	0.015086386	0.076514201

PIGN	-1.906803	0.784653	-2.430122	0.01509376	0.076521438
HLA.DMA	0.636911	0.262147	2.429595	0.015115718	0.076602576
STARD5	-1.722835	0.70926	-2.429061	0.015138003	0.076685309
MON1A	-2.460064	1.012891	-2.428754	0.015150795	0.076719903
GFOD1	-1.689761	0.696056	-2.427622	0.015198168	0.076929516
CD302	-4.174133	1.71989	-2.426977	0.015225227	0.077036174
IFFO2	-2.139847	0.881769	-2.426767	0.01523403	0.077050416
METTL15	-1.308599	0.539456	-2.425774	0.015275793	0.07723129
SLC19A1	-2.211076	0.911741	-2.425114	0.015303569	0.077341329
SYNPO2L	-4.573706	1.886301	-2.424695	0.015321236	0.077400214
ENSG00000246731	-3.569313	1.472255	-2.424386	0.015334317	0.077435891
RTTN	-2.208726	0.911161	-2.42408	0.015347223	0.077443942
ZFP57	-4.643641	1.915644	-2.424063	0.015347948	0.077443942
PEMT	-1.870461	0.771685	-2.423866	0.015356283	0.077455623
AMER1	-2.742278	1.131502	-2.423574	0.015368616	0.077487456
ENSG00000272540	-3.597675	1.484687	-2.423188	0.015384981	0.077525607
ENSG00000249790	-3.518808	1.452233	-2.423033	0.01539151	0.077525607
BLZF1	-1.446487	0.59699	-2.422969	0.015394259	0.077525607
IGHV3.30	-3.584698	1.479946	-2.422182	0.01542763	0.077663271
ENSG00000142046	-2.077908	0.858057	-2.421644	0.015450468	0.077747821
PPIP5K1	-2.053999	0.848332	-2.421221	0.01546849	0.077795442
LMNTD2	-2.380655	0.983307	-2.42107	0.015474901	0.077795442
ARSD	-1.844266	0.761811	-2.420897	0.015482247	0.077795442
FBXL22	-2.590863	1.070227	-2.420853	0.015484116	0.077795442
LINC00886	-1.875989	0.77504	-2.420505	0.015498969	0.077839668
ENSG00000279345	-4.768615	1.970547	-2.419945	0.015522856	0.07792922
GIPC1	-1.723491	0.712352	-2.419439	0.015544486	0.077987916
SLC41A2	-2.216096	0.915981	-2.41937	0.015547432	0.077987916
ACSL6	-3.700651	1.529671	-2.419246	0.015552731	0.077987916
LGALS3BP	-2.149327	0.888533	-2.41896	0.015564937	0.078018718
TMEM169	-2.048965	0.847232	-2.418423	0.015587938	0.078103582
SDR42E1	-2.816105	1.164556	-2.418179	0.015598406	0.078117487
IGLV2.8	-4.825999	1.995802	-2.418075	0.015602855	0.078117487
BTG2	-0.470719	0.194733	-2.417257	0.015637977	0.078262873
UCK2	-2.04206	0.844904	-2.416912	0.015652791	0.07830026
RETREG1	-1.738785	0.719458	-2.4168	0.015657618	0.07830026
SH3BP5.AS1	-1.877136	0.77675	-2.416655	0.015663847	0.078300977
YEATS2	-2.017459	0.83499	-2.416148	0.015685673	0.078367102
AMDHD1	-3.514232	1.454603	-2.415938	0.015694715	0.078367102
POP1	-1.842201	0.762547	-2.415852	0.01569844	0.078367102
TNKS1BP1	-4.984258	2.063206	-2.415783	0.015701437	0.078367102
LAPTM4B	-2.213567	0.916471	-2.415316	0.015721578	0.078437201
CENPP	-2.032386	0.841615	-2.414866	0.015740987	0.078503597
ALS2	-1.932109	0.80031	-2.414202	0.015769707	0.078616357
PPP1R16A	-1.636218	0.677873	-2.413753	0.015789174	0.078682921
WNT16	-2.997546	1.241949	-2.413582	0.015796573	0.078689317
LINC00921	-1.797654	0.745025	-2.412878	0.015827115	0.07881095

SNAI3.AS1	-1.869111	0.774743	-2.412556	0.015841107	0.078850106
ARRDC4	-5.155572	2.13742	-2.412054	0.015862941	0.07888344
CST7	-5.155572	2.13742	-2.412054	0.015862941	0.07888344
IGKV3.11	-2.175812	0.902086	-2.411979	0.015866196	0.07888344
MPRIP.AS1	-1.920726	0.796422	-2.411694	0.015878612	0.078914678
SUMO4	-2.339367	0.970219	-2.411174	0.015901248	0.078996664
ETV2	-2.358531	0.978616	-2.410067	0.015949576	0.079206173
GPLD1	-1.862867	0.773012	-2.409882	0.015957681	0.079211566
SRFBP1	-1.252194	0.51964	-2.409736	0.015964061	0.079211566
SMKR1	-3.285143	1.363345	-2.40962	0.01596913	0.079211566
KIFC2	-2.003677	0.831626	-2.409348	0.015981045	0.079237024
XG	-3.602242	1.49533	-2.408995	0.015996532	0.079237024
MYLK	-4.788504	1.987804	-2.408942	0.015998852	0.079237024
TIRAP	-1.723799	0.715584	-2.408941	0.015998895	0.079237024
ENSG00000272936	-4.859347	2.017809	-2.408229	0.016030119	0.079353732
ENSG00000261669	-1.978548	0.821614	-2.408123	0.016034795	0.079353732
IPMK	-1.92382	0.799046	-2.407644	0.016055818	0.079422076
ENSG00000232995	-2.24666	0.933182	-2.407528	0.01606095	0.079422076
ENSG00000260018	-2.015124	0.837365	-2.406507	0.016105899	0.079613752
ENSG00000276957	-4.811839	1.999632	-2.406362	0.016112273	0.079614679
TBL1X	-1.522197	0.632828	-2.405389	0.016155268	0.079796483
CHKB.DT	-4.142755	1.722479	-2.405113	0.016167465	0.079826084
TEX9	-3.817988	1.587965	-2.404328	0.016202226	0.079931179
MKS1	-2.228416	0.926845	-2.404303	0.016203329	0.079931179
HOXB7	-4.967767	2.066316	-2.404166	0.016209411	0.079931179
MCM6	-1.854258	0.771299	-2.404072	0.016213599	0.079931179
LUCAT1	-2.409902	1.002639	-2.40356	0.016236283	0.080012355
MROH1	-1.572104	0.654159	-2.403245	0.016250317	0.080037381
METRN	-1.960451	0.815779	-2.403166	0.016253803	0.080037381
CARNS1	-2.948709	1.227352	-2.402497	0.016283551	0.080153195
LBHD1	-3.635764	1.513585	-2.402087	0.016301829	0.080206846
SH3RF1	-5.146069	2.142434	-2.401973	0.016306918	0.080206846
ENSG00000276148	-2.239222	0.932428	-2.401495	0.016328215	0.080280912
ANKRD16	-2.169132	0.903296	-2.401352	0.016334626	0.080281754
ENSG00000236140	-2.789022	1.161651	-2.400912	0.016354271	0.080318591
TBX19	-2.079655	0.866197	-2.400904	0.016354605	0.080318591
ZNF25	-2.236468	0.93159	-2.400699	0.016363803	0.0803331
ENSG00000273188	-1.966878	0.819383	-2.400438	0.016375477	0.080359749
CRNDE	-3.537567	1.474038	-2.399916	0.016398822	0.080443629
GLCCI1	-1.397233	0.582329	-2.399388	0.016422494	0.080529052
MBNL1.AS1	-2.025414	0.844311	-2.398894	0.016444658	0.080607016
ZNF487	-1.807744	0.753622	-2.398743	0.016451477	0.080609732
PARPBP	-2.13909	0.892017	-2.398038	0.016483178	0.080734315
RAB11FIP3	-3.555315	1.482807	-2.397693	0.016498703	0.080754744
ZFP69B	-3.536159	1.474834	-2.397666	0.016499901	0.080754744
LMNB2	-1.974046	0.823511	-2.39711	0.016524963	0.080805667
ENSG00000272764	-1.894155	0.790205	-2.397041	0.016528075	0.080805667

PLEKHA4	-1.868272	0.779415	-2.397017	0.016529146	0.080805667
ENSG00000279217	-3.589768	1.497746	-2.39678	0.016539861	0.080827344
RPH3AL	-2.124486	0.886486	-2.396524	0.016551402	0.080853032
SYCP2	-3.255942	1.358849	-2.396102	0.016570458	0.0809154
PRKRA.AS1	-1.730929	0.722606	-2.3954	0.016602258	0.081039927
EDRF1.DT	-1.996874	0.833768	-2.395	0.016620375	0.081088948
HIST3H2A	-2.034367	0.849458	-2.3949	0.016624904	0.081088948
MAP3K10	-2.109239	0.880849	-2.394552	0.016640666	0.081125709
ENSG00000271918	-1.984588	0.828828	-2.394451	0.016645246	0.081125709
ENSG00000268713	-2.023687	0.845204	-2.394317	0.016651356	0.081125709
ITGA5	-2.118074	0.88473	-2.394035	0.016664164	0.081157378
ZNF239	-2.071953	0.865713	-2.393349	0.01669533	0.081258177
EID2B	-1.46479	0.612037	-2.393302	0.016697491	0.081258177
CAPN3	-2.142655	0.895655	-2.392278	0.016744158	0.081454473
CPEB3	-2.091542	0.874386	-2.392012	0.016756312	0.081482792
ENSG00000274341	-2.793524	1.168118	-2.391474	0.016780854	0.081571309
TMSB4Y	-2.753981	1.151865	-2.39089	0.016807609	0.08166949
NAGS	-2.080267	0.87013	-2.390755	0.016813747	0.08166949
ENSG00000272870	-2.451461	1.025721	-2.389987	0.016848972	0.081772361
ENSG00000237181	-2.197053	0.919334	-2.389831	0.016856122	0.081772361
FAM185A	-1.749297	0.731989	-2.389787	0.016858125	0.081772361
ENSG00000262823	-3.43677	1.438136	-2.389739	0.016860346	0.081772361
ZNF835	-1.998833	0.836521	-2.38946	0.01687318	0.081803773
L1TD1	-2.37722	0.995139	-2.388832	0.016902008	0.08191267
FAM53B	-1.135386	0.475403	-2.388259	0.016928422	0.081979299
PARG	-1.543896	0.646482	-2.388149	0.01693346	0.081979299
LINC02166	-3.742107	1.566969	-2.388119	0.01693487	0.081979299
MFSD2A	-4.88031	2.043839	-2.387815	0.01694886	0.082016164
DPP8	-1.138443	0.476854	-2.387406	0.016967742	0.082076667
MEF2C	0.701973	0.294085	2.386974	0.016987709	0.082133075
CCND1	-4.566755	1.913276	-2.386877	0.01699217	0.082133075
ENSG00000266538	-1.865624	0.781838	-2.386203	0.017023334	0.082197078
TESC	-2.508519	1.051266	-2.386189	0.017024001	0.082197078
CASTOR3	-2.058325	0.862604	-2.386177	0.017024575	0.082197078
HDLBP	1.64107	0.687819	2.385904	0.017037215	0.082227247
LINC01410	-3.71712	1.55807	-2.38572	0.017045715	0.082237427
ENSG00000249249	-2.100291	0.880468	-2.385425	0.01705939	0.082258746
ENSG00000258302	-3.755731	1.5745	-2.385349	0.01706292	0.082258746
IGHG2	-1.913457	0.802365	-2.38477	0.017089799	0.082329193
MYO7B	-3.607622	1.512819	-2.384703	0.017092936	0.082329193
ARL15	-1.842253	0.772595	-2.384502	0.017102271	0.082329193
DAPL1	-1.871901	0.785074	-2.384363	0.017108695	0.082329193
PPARD	-1.594674	0.668833	-2.384264	0.0171133	0.082329193
H3F3C	-2.412424	1.011874	-2.384115	0.017120251	0.082329193
ENSG00000272948	-3.146123	1.319668	-2.384026	0.017124373	0.082329193
ZNF565	-2.216228	0.929652	-2.383933	0.017128721	0.082329193
ZC4H2	-2.071913	0.86922	-2.383647	0.01714203	0.082362397

AGPS	-1.564523	0.656462	-2.383265	0.01715985	0.082383136
GPR35	-4.775052	2.003706	-2.383111	0.017167029	0.082383136
NDC80	-4.775052	2.003706	-2.383111	0.017167029	0.082383136
ENSG00000242474	-2.067436	0.867575	-2.383005	0.017171957	0.082383136
TRG.AS1	-2.933624	1.231195	-2.382745	0.017184077	0.082410553
IGIP	-2.244911	0.94246	-2.381969	0.017220355	0.082527587
PKD2	-2.070641	0.869337	-2.381863	0.017225292	0.082527587
CNPY4	-1.673796	0.702741	-2.381811	0.017227722	0.082527587
HACE1	-2.163205	0.908461	-2.381177	0.017257431	0.082639139
ANKDD1A	-2.62075	1.100762	-2.380852	0.017272669	0.082681337
ENSG00000260274	-2.205584	0.926573	-2.380368	0.017295352	0.082759127
ZNF284	-1.694116	0.711893	-2.379735	0.017325099	0.082851964
ARHGEF19	-1.779164	0.747659	-2.379645	0.017329316	0.082851964
FBLIM1	-1.992558	0.83737	-2.379544	0.017334071	0.082851964
ENSG00000256705	-4.849072	2.038072	-2.379245	0.017348124	0.082888343
DCAF12	-4.328142	1.819543	-2.378698	0.017373904	0.082979193
ZNF212	-1.652597	0.694787	-2.378568	0.017380036	0.082979193
ZFYVE9	-3.544798	1.490433	-2.378367	0.017389494	0.082993551
RAB12	-2.174024	0.914163	-2.378158	0.017399373	0.083009914
ENSG00000272525	-4.825765	2.029683	-2.377596	0.017425928	0.083074997
C6orf47.AS1	-4.825765	2.029683	-2.377596	0.017425928	0.083074997
GRK6	-1.559143	0.656113	-2.376334	0.01748565	0.083315487
MDGA1	-3.161911	1.330627	-2.376256	0.017489324	0.083315487
ATF3	-2.04876	0.862467	-2.375465	0.01752684	0.08345452
CADM4	-4.611853	1.941619	-2.375262	0.017536499	0.08345452
ENSG00000267469	-3.332167	1.402929	-2.37515	0.017541829	0.08345452
RAB9A	-1.076593	0.453284	-2.375095	0.017544453	0.08345452
NBEA	-2.330394	0.981278	-2.374856	0.0175558	0.083477636
NKG7	-3.422568	1.441386	-2.374498	0.017572815	0.083524778
WDCP	-1.896153	0.798591	-2.374375	0.017578697	0.083524778
APLF	-1.855426	0.781541	-2.374061	0.017593656	0.083564995
COG6	-1.787025	0.752817	-2.373783	0.017606895	0.083569476
ATP1A3	-4.623871	1.947903	-2.373768	0.017607589	0.083569476
ALCAM	-1.813648	0.7641	-2.373574	0.017616876	0.083582725
ENSG00000272829	-4.720632	1.989063	-2.373295	0.017630196	0.083615087
FAM76A	-1.124133	0.473912	-2.37203	0.017690663	0.083870951
TNFSF8	-4.698074	1.980846	-2.371752	0.017703976	0.083903153
ENSG00000279210	-3.531851	1.490194	-2.370062	0.017785101	0.084230783
MPND	-2.002378	0.844934	-2.369862	0.017794724	0.084230783
NUDC	-0.729984	0.308062	-2.3696	0.017807335	0.084230783
PGAM5	-2.200093	0.928497	-2.369521	0.017811151	0.084230783
ENSG00000263264	-2.004246	0.845847	-2.369514	0.017811483	0.084230783
ENSG00000237188	-1.715184	0.723876	-2.369444	0.017814832	0.084230783
ASMTL	-1.309801	0.552808	-2.369359	0.017818932	0.084230783
NOL4L	-3.556833	1.501276	-2.369207	0.017826261	0.084234485
APOC1	-3.169729	1.338319	-2.368441	0.017863221	0.084378142
ENSG00000231113	-1.826234	0.771256	-2.36787	0.017890816	0.084477477

SLC25A30	-2.091535	0.883402	-2.367591	0.017904315	0.084510206
NUDT12	-2.20003	0.929452	-2.367018	0.017932068	0.084610165
TMEM135	-2.110366	0.891667	-2.366765	0.017944321	0.084636942
ENSG00000042317	-2.04859	0.865838	-2.36602	0.017980451	0.08477628
IL7R	-2.03549	0.860476	-2.365539	0.018003839	0.084855459
KNSTRN	-1.700028	0.718897	-2.364772	0.018041164	0.084897286
MFSD9	-1.9717	0.833849	-2.364577	0.018050651	0.084897286
ENSG00000229043	-2.39947	1.014768	-2.364549	0.018052043	0.084897286
ENSG00000268472	-4.586009	1.939486	-2.364549	0.018052026	0.084897286
TMEM229B	-4.586009	1.939486	-2.364549	0.018052026	0.084897286
SERAC1	-1.816139	0.768092	-2.36448	0.018055405	0.084897286
ALDH4A1	-2.764343	1.169148	-2.364408	0.0180589	0.084897286
WDR90	-2.139448	0.904988	-2.364063	0.018075736	0.084945398
SPNS3	-2.214256	0.937027	-2.363066	0.018124439	0.085143175
CKLF	-0.967505	0.409463	-2.362864	0.018134301	0.085158414
ZNF280B	-1.963808	0.831225	-2.362548	0.018149773	0.085173917
ENSG00000225187	-3.618323	1.531548	-2.362527	0.018150842	0.085173917
VSTM4	-2.232448	0.945172	-2.361949	0.01817913	0.085253086
AXIN2	-4.509143	1.909154	-2.361854	0.018183819	0.085253086
FILIP1L	-4.733083	2.004035	-2.361777	0.01818759	0.085253086
ITGA3	-2.070146	0.8766	-2.361562	0.018198126	0.08527141
SYT1	-3.070282	1.300206	-2.361382	0.018206961	0.085281752
MICOS13	0.930814	0.394288	2.360743	0.018238346	0.085397669
TPRN	-2.164643	0.917182	-2.360103	0.018269866	0.08551414
CELF4	-2.934942	1.243851	-2.359561	0.018296552	0.085579547
NUP210	1.530311	0.648561	2.359549	0.018297143	0.085579547
ENSG00000240859	-4.517542	1.914799	-2.359277	0.018310568	0.085611221
DZANK1	-1.956918	0.829528	-2.359074	0.018320593	0.085618695
SEPTIN9	0.577963	0.24502	2.358845	0.018331913	0.085618695
PDZD11	-1.328445	0.563194	-2.358771	0.018335586	0.085618695
ENSG00000237094	-4.699596	1.992447	-2.358706	0.018338783	0.085618695
ENSG00000264577	-1.943637	0.824129	-2.358413	0.01835325	0.085655158
DOLK	-1.981541	0.840429	-2.357774	0.0183849	0.085760121
HEATR5A	-1.87027	0.793264	-2.357689	0.018389071	0.085760121
ATG2B	-1.680602	0.712908	-2.357389	0.018403951	0.085795558
CDK2AP1	-2.790968	1.184027	-2.357183	0.018414179	0.085795558
DPF3	-5.030491	2.134157	-2.357133	0.018416673	0.085795558
LRRCC1	-1.767832	0.750062	-2.356914	0.018427512	0.085810234
SCAP	-1.465964	0.622014	-2.3568	0.018433161	0.085810234
ALG1L2	-2.279931	0.967624	-2.356215	0.018462224	0.085865073
KIF9	-2.030877	0.861936	-2.356181	0.01846393	0.085865073
CLSPN	-2.448801	1.039319	-2.35616	0.018464961	0.085865073
SLC31A1	-1.870419	0.794015	-2.355646	0.018490554	0.08594579
ENSG00000261416	-2.308428	0.979998	-2.355543	0.018495678	0.08594579
MED14OS	-1.83898	0.780959	-2.354772	0.018534061	0.086026974
LINC00638	-2.09752	0.890777	-2.354708	0.018537241	0.086026974
C5orf34	-2.137983	0.907978	-2.354665	0.018539412	0.086026974

STAC3	-2.125801	0.902849	-2.354547	0.01854529	0.086026974
HDAC4	-2.078128	0.882639	-2.354448	0.018550244	0.086026974
ENSG00000254281	-2.625892	1.11534	-2.354342	0.01855555	0.086026974
B3GLCT	-2.027812	0.86134	-2.354253	0.01855995	0.086026974
PHF13	-1.885778	0.801064	-2.354092	0.018568015	0.086033361
SYTL3	-4.653954	1.97726	-2.353739	0.01858566	0.086084122
ENSG00000268220	-2.068004	0.87868	-2.353533	0.018595939	0.086095551
PSPN	-2.080602	0.884075	-2.353422	0.01860151	0.086095551
PPP1R3D	-2.254036	0.957873	-2.353167	0.018614264	0.08609668
UCN	-2.202144	0.935828	-2.35315	0.018615137	0.08609668
OVCH1.AS1	-3.317228	1.409947	-2.352732	0.018636045	0.086162413
ZBTB16	-2.478103	1.053499	-2.352259	0.018659784	0.08624118
ZDHHC19	-3.433445	1.460345	-2.351118	0.018717091	0.086474977
PHOSPHO2	-1.702735	0.724303	-2.35086	0.018730092	0.086503981
CENPW	-1.816215	0.772642	-2.350656	0.018740339	0.086520253
REEP6	-4.467298	1.901232	-2.349685	0.018789295	0.086698365
JAZF1.AS1	-4.645243	1.977016	-2.349624	0.018792395	0.086698365
ZNF687.AS1	-2.187983	0.931267	-2.349468	0.018800276	0.086703639
IGLL1	-2.748361	1.169872	-2.349284	0.01880956	0.086715376
BRICD5	-2.16333	0.920939	-2.349048	0.018821474	0.086733683
CAPRIN2	-1.614792	0.687456	-2.348939	0.018827013	0.086733683
KPNA4	-1.227898	0.522783	-2.348772	0.018835419	0.086741351
ZNF571.AS1	-2.505309	1.067112	-2.347747	0.018887348	0.086949375
SLC28A2	-2.06124	0.878122	-2.347328	0.018908612	0.086978622
TMEM81	-4.586782	1.954153	-2.347197	0.018915231	0.086978622
PCNX3	-1.801855	0.767677	-2.347152	0.018917518	0.086978622
CNTLN	-3.027293	1.28985	-2.347011	0.018924683	0.086978622
ZNF469	-4.564568	1.944889	-2.346956	0.0189275	0.086978622
GDAP1	-1.98845	0.847322	-2.346746	0.018938144	0.086996463
LZTR1	-1.602778	0.683143	-2.346183	0.018966815	0.087097076
CUZD1	-4.49517	1.916078	-2.346026	0.018974787	0.087102598
OLMALINC	-2.790734	1.189661	-2.345824	0.018985073	0.087118734
ADAM9	-4.638025	1.97775	-2.345102	0.019021865	0.08723766
TSIX	-3.920391	1.671773	-2.34505	0.019024549	0.08723766
ENSG00000272181	-4.928768	2.102026	-2.344771	0.019038786	0.08727184
BBOF1	-2.250914	0.960096	-2.344468	0.019054231	0.087311534
NAB1	-1.801628	0.768649	-2.343888	0.019083891	0.087398481
TXNDC9	-1.032301	0.440435	-2.343821	0.019087345	0.087398481
SLC18B1	-1.552922	0.662631	-2.343571	0.019100132	0.087398481
AFG1L	-1.99736	0.852274	-2.343566	0.019100375	0.087398481
ENSG00000270019	-1.583898	0.676014	-2.342997	0.019129547	0.087483192
FN1	-3.394454	1.448802	-2.342939	0.019132486	0.087483192
ENSG00000255389	-2.360484	1.007681	-2.34249	0.019155558	0.08755757
ENSG00000259001	-2.012624	0.859242	-2.342324	0.019164058	0.087558506
ZXDA	-2.220522	0.948041	-2.342221	0.019169372	0.087558506
POMT1	-1.680275	0.717651	-2.341355	0.019213904	0.087700416
ENSG00000203546	-2.225072	0.950336	-2.341351	0.019214073	0.087700416

KCNK6	-1.410775	0.602627	-2.341043	0.019229949	0.087741754
PTK6	-3.203917	1.368764	-2.340737	0.019245727	0.087771413
ENSG00000257194	-5.049559	2.15733	-2.340652	0.019250092	0.087771413
TP53I3	-2.216853	0.947165	-2.340515	0.019257163	0.087772555
SLC41A3	-1.226149	0.523947	-2.340214	0.019272696	0.087785118
HDAC11	-2.270499	0.970217	-2.340197	0.019273565	0.087785118
PPRC1	-1.848383	0.789957	-2.339854	0.019291257	0.087804341
KIAA1328	-1.440641	0.615698	-2.339851	0.019291433	0.087804341
LRP2BP	-2.066339	0.883306	-2.339324	0.019318648	0.087872611
PIK3R2	-3.29744	1.409586	-2.339297	0.019320091	0.087872611
TST	-2.451507	1.048169	-2.338848	0.019343315	0.087947151
BOLA2.SMG1P6	-1.551765	0.663572	-2.338503	0.019361155	0.087952312
RND1	-1.837404	0.785719	-2.3385	0.019361343	0.087952312
TEX10	-1.548782	0.662319	-2.338424	0.019365266	0.087952312
DUSP16	-4.783478	2.045709	-2.338298	0.019371792	0.087952312
TEPP	-2.141516	0.915897	-2.338164	0.01937874	0.087952821
PRKAB1	-1.520907	0.65089	-2.336656	0.019457057	0.088221431
DGKZ	-1.432457	0.613055	-2.336587	0.019460679	0.088221431
YOD1	-1.840072	0.787518	-2.336547	0.019462752	0.088221431
CLCN5	-3.345656	1.431911	-2.336497	0.019465349	0.088221431
MYBL2	-3.338895	1.429155	-2.336272	0.019477066	0.088238842
SLC25A53	-1.442218	0.617352	-2.336136	0.019484152	0.088238842
TRAF3IP1	-2.613432	1.11875	-2.336028	0.019489764	0.088238842
CCNJ	-2.136189	0.914534	-2.335823	0.019500452	0.088256177
ENSG00000247373	-2.043672	0.874991	-2.335648	0.01950957	0.088266397
DLK2	-5.134031	2.198795	-2.334929	0.019547118	0.088405192
CA5A	-4.788898	2.05148	-2.334363	0.019576732	0.088508013
TRAPPC2L	1.472614	0.630893	2.334176	0.019586531	0.088521212
KTN1.AS1	-3.434683	1.471691	-2.333834	0.019604421	0.088546495
ABCB10	-1.778484	0.762053	-2.333806	0.019605888	0.088546495
SREK1	-0.825971	0.354065	-2.332821	0.019657543	0.088711802
ENSG00000272426	-2.312614	0.991403	-2.332669	0.019665538	0.088711802
SLC7A11	-4.414023	1.892294	-2.332631	0.01966753	0.088711802
LY6E	-0.642266	0.275351	-2.332537	0.019672439	0.088711802
TMPO.AS1	-2.004804	0.859527	-2.332451	0.019676963	0.088711802
GLB1L	-1.812507	0.77719	-2.33213	0.019693836	0.088756772
SNRPF	-0.553277	0.237284	-2.331707	0.01971612	0.088805168
ENSG00000245025	-2.123725	0.91082	-2.331664	0.019718378	0.088805168
C1QTNF3	-2.415922	1.036274	-2.331355	0.019734638	0.088818078
UBOX5	-1.820126	0.780737	-2.331293	0.019737911	0.088818078
LINC00887	-5.029431	2.157428	-2.331216	0.019741952	0.088818078
KIF13A	-2.344575	1.005913	-2.330793	0.019764267	0.088887388
DCLRE1B	-2.244572	0.963184	-2.330367	0.019786758	0.088957446
YPEL5	1.30863	0.561638	2.330022	0.01980498	0.089008269
ENSG00000227070	-3.82031	1.639755	-2.329806	0.019816429	0.089028628
ENSG00000275580	-4.934653	2.118332	-2.329499	0.019832635	0.089070336
GNE	-1.738511	0.746467	-2.328988	0.019859721	0.089093529

IGHV1.18	-4.641533	1.992987	-2.328934	0.019862582	0.089093529
AGER	-2.106117	0.904373	-2.328816	0.019868817	0.089093529
RNF208	-4.705033	2.020381	-2.328785	0.019870459	0.089093529
WDR62	-5.095148	2.187935	-2.328748	0.01987242	0.089093529
PLD2	-1.739519	0.747224	-2.327975	0.019913433	0.089246304
SEMA4F	-1.907093	0.819671	-2.326658	0.01998349	0.089529096
ENSG00000271855	-2.672275	1.148635	-2.326479	0.019993021	0.089540619
RASGRF1	-2.224771	0.956434	-2.326111	0.020012652	0.089597353
CLPB	-1.938209	0.833323	-2.325881	0.020024926	0.08962112
TRAM2	-1.971808	0.847865	-2.325616	0.02003903	0.089624042
ABCC2	-4.845596	2.083583	-2.325607	0.020039509	0.089624042
ELL3	-2.167319	0.932076	-2.32526	0.020058057	0.089675822
RPL38	-0.152096	0.065441	-2.324177	0.020116005	0.089903658
MTCH1	1.37291	0.590875	2.323522	0.020151126	0.090011668
ENSG00000263884	-4.712601	2.028264	-2.323465	0.020154163	0.090011668
PEX5	-1.785935	0.768967	-2.322512	0.020205357	0.090203835
MYO1D	-4.456124	1.918755	-2.322404	0.020211211	0.090203835
ADAM20	-4.667862	2.010098	-2.322206	0.020221856	0.090220048
A2M.AS1	-5.213216	2.245583	-2.321542	0.02025759	0.090348151
SH3D21	-3.293063	1.418693	-2.321196	0.020276286	0.090400198
SDCBP2	-3.408742	1.469176	-2.320172	0.020331551	0.090615194
VWA5A	-2.5522	1.1003	-2.319549	0.020365306	0.09073421
LINC01063	-3.407132	1.469466	-2.318619	0.020415712	0.090917669
ENSG00000263766	-4.583179	1.976762	-2.318529	0.020420616	0.090917669
NAA50	-0.751787	0.32431	-2.31811	0.020443364	0.090987467
IGF2BP3	-4.734752	2.042763	-2.317817	0.020459264	0.091008577
PYCR1	-3.344672	1.443061	-2.317762	0.020462253	0.091008577
CACNA1F	-4.8672	2.100209	-2.317484	0.020477375	0.091038421
WDR31	-5.246101	2.26389	-2.317295	0.020487654	0.091038421
PHGDH	-3.856429	1.664228	-2.317249	0.020490189	0.091038421
POLR1A	-1.798026	0.775978	-2.317111	0.020497704	0.091040371
THBS3	-2.118925	0.914551	-2.316902	0.020509087	0.091059495
PAQR3	-1.968515	0.849685	-2.316757	0.020516946	0.091062967
ENSG00000261584	-2.574373	1.111363	-2.31641	0.020535908	0.091115698
PLCD3	-3.133724	1.352997	-2.316136	0.02055086	0.091120182
TECPR2	-2.103932	0.908382	-2.316132	0.020551082	0.091120182
ENSG00000260228	-4.231663	1.827532	-2.315506	0.020585247	0.091240226
ENSG00000272630	-1.989909	0.859513	-2.315157	0.02060432	0.091275019
LINC00852	-2.171719	0.938066	-2.315103	0.020607285	0.091275019
ZNF510	-1.750436	0.756455	-2.314001	0.020667687	0.091511058
RAB30.DT	-0.958114	0.414115	-2.313643	0.020687336	0.091566546
DLGAP4.AS1	-2.151914	0.9304	-2.31289	0.020728703	0.091718095
PTPRJ	-2.725799	1.17866	-2.312626	0.020743223	0.091750791
PIDD1	-1.614915	0.698485	-2.312026	0.020776246	0.091865276
SPIN3	-4.80609	2.079957	-2.310668	0.020851209	0.092165064
RPS6KC1	-1.606956	0.695601	-2.310169	0.020878789	0.092255282
ENSG00000237950	-4.580951	1.983532	-2.309492	0.020916312	0.092389353

AZIN1.AS1	-1.96482	0.85096	-2.308945	0.020946615	0.092444325
TEPSIN	-1.347769	0.583727	-2.308903	0.020948955	0.092444325
SCN3A	-2.60491	1.128314	-2.308674	0.020961682	0.092444325
HSBP1L1	-2.259785	0.97892	-2.308447	0.020974266	0.092444325
FOSL2	-4.561182	1.97588	-2.30843	0.020975236	0.092444325
GPR171	-3.818017	1.653963	-2.308406	0.020976591	0.092444325
LRRIQ3	-2.383589	1.032589	-2.308361	0.020979049	0.092444325
C19orf81	-4.283968	1.856009	-2.308162	0.020990146	0.092450705
SLC25A35	-2.121088	0.918985	-2.308077	0.020994868	0.092450705
CUL7	-2.022525	0.876451	-2.307632	0.021019619	0.092511364
ENSG00000257354	-1.727076	0.748439	-2.307571	0.021023022	0.092511364
CSF2RB	-2.042439	0.885219	-2.30727	0.021039773	0.092548181
ENSG00000261200	-2.93056	1.270201	-2.307162	0.021045774	0.092548181
SYNGR1	-1.70087	0.737272	-2.306977	0.021056081	0.092561871
NEK3	-2.030388	0.880575	-2.305753	0.021124423	0.092830584
RFT1	-1.653864	0.71766	-2.304524	0.021193268	0.093014209
ZNF142	-1.817874	0.788837	-2.304498	0.021194708	0.093014209
IL6R	-4.603879	1.997854	-2.304412	0.021199511	0.093014209
ENSG00000260271	-2.582812	1.120844	-2.304347	0.021203187	0.093014209
PTAFR	-2.125072	0.922204	-2.30434	0.02120355	0.093014209
ENSG00000267169	-1.830753	0.794517	-2.304233	0.021209582	0.093014209
ARL5B	-1.66545	0.722863	-2.303965	0.021224606	0.093048385
TMOD1	-2.257316	0.980276	-2.302736	0.021293702	0.093319504
FBXW4	-1.324107	0.575053	-2.302583	0.021302328	0.09332552
GSTCD	-1.823788	0.792308	-2.301869	0.021342578	0.093417355
ENSG00000257433	-1.936048	0.841102	-2.301798	0.021346546	0.093417355
ATRN	-1.893559	0.822648	-2.301784	0.021347335	0.093417355
TOMM7	-0.303749	0.131967	-2.301696	0.021352331	0.093417355
TTTY14	-2.827152	1.228619	-2.30108	0.02138711	0.093537711
TMEM267	-1.354309	0.588727	-2.300402	0.021425438	0.093673498
CASZ1	-1.916041	0.833216	-2.299573	0.021472418	0.093847011
SH3D19	-2.060476	0.896116	-2.299342	0.021485528	0.093858743
C21orf58	-2.099807	0.91325	-2.299269	0.021489691	0.093858743
SMARCD3	-4.526562	1.969713	-2.298082	0.021557145	0.094117184
ANG	-4.376693	1.904591	-2.29797	0.021563492	0.094117184
JSRP1	-3.645391	1.58665	-2.297539	0.02158805	0.094165685
BATF2	-2.68102	1.16692	-2.297518	0.021589241	0.094165685
ENSG00000272606	-1.611504	0.701689	-2.296608	0.021641139	0.094360062
CD58	1.641577	0.714879	2.296299	0.021658793	0.094405043
CHSY1	-1.887926	0.822228	-2.296109	0.021669626	0.094420277
ZNF584	-1.73953	0.757832	-2.295402	0.021710111	0.094561454
CAMKK2	-1.81913	0.79255	-2.295286	0.021716725	0.094561454
RESF1	-0.755257	0.3291	-2.294915	0.021737978	0.094621977
ENSG00000204802	-2.020317	0.880701	-2.293988	0.021791209	0.094807787
ERCC2	-1.605931	0.700083	-2.293915	0.021795402	0.094807787
FBXW9	-2.008471	0.875744	-2.293446	0.02182233	0.094892844
RCBTB1	-1.667349	0.727201	-2.29283	0.021857785	0.095014907

EPOP	-4.783894	2.086781	-2.292475	0.021878224	0.095071633
NGRN	-1.699068	0.741326	-2.291931	0.021909643	0.095176023
TPRG1L	-1.444942	0.630562	-2.291516	0.021933609	0.095247975
ENSG00000277496	-1.943738	0.848576	-2.290588	0.021987244	0.095448673
TMEM38A	-1.842634	0.804502	-2.290404	0.021997929	0.095462849
PALLD	-2.03677	0.889529	-2.289719	0.022037619	0.095601702
GOLGA6L9	-2.981743	1.302301	-2.289595	0.022044785	0.095601702
TBKBP1	-4.653318	2.032887	-2.289019	0.022078256	0.095692188
ENSG00000259488	-2.291949	1.001297	-2.28898	0.022080524	0.095692188
ZNF354C	-2.016393	0.880974	-2.288822	0.022089724	0.095699823
CERCAM	-1.89542	0.828178	-2.288661	0.022099033	0.09570793
METRNL	-2.691144	1.176095	-2.288203	0.022125716	0.095791245
MYO5C	-2.175477	0.950893	-2.287826	0.022147656	0.095853984
KRT18	-1.604252	0.701293	-2.287564	0.022162927	0.095887822
RPS4Y2	-3.523896	1.540752	-2.287127	0.022188421	0.095965852
CENPU	-2.168529	0.948202	-2.28699	0.022196412	0.095968158
SGPP2	-4.543672	1.987053	-2.286638	0.022216955	0.09602471
ENSG00000225793	-4.549416	1.989785	-2.286386	0.022231663	0.096027358
SLC39A8	-1.760512	0.770002	-2.286372	0.022232494	0.096027358
RXRA	-2.213357	0.96818	-2.2861	0.022248422	0.096063908
KMO	-1.414918	0.618984	-2.285871	0.022261814	0.096089487
ENSG00000187951	-1.657864	0.725342	-2.285632	0.022275768	0.096097233
ZNF736	-1.472298	0.644181	-2.285534	0.02228154	0.096097233
TRAFD1	-1.495594	0.654445	-2.285286	0.022296039	0.096097233
HHIP.AS1	-4.132914	1.808491	-2.285284	0.022296203	0.096097233
ENSG00000268292	-4.358353	1.907346	-2.285035	0.022310751	0.096097233
GUCY2C	-1.567313	0.685941	-2.28491	0.022318127	0.096097233
THAP4	-1.368658	0.599003	-2.284893	0.022319079	0.096097233
GPAM	-2.022175	0.885048	-2.28482	0.022323357	0.096097233
EME2	-1.937537	0.848221	-2.284237	0.022357606	0.096212482
TONSL	-1.774065	0.776852	-2.283658	0.022391641	0.096326729
ERRFI1	-4.611442	2.019724	-2.283204	0.022418357	0.096409424
INPP5E	-1.684924	0.738174	-2.282556	0.022456533	0.096489023
AMT	-1.958075	0.857855	-2.282525	0.022458353	0.096489023
ANO10	-1.96159	0.859401	-2.282508	0.022459363	0.096489023
PTBP1	-0.852974	0.373837	-2.281672	0.022508693	0.096627242
TWNK	-1.833454	0.803598	-2.281556	0.022515585	0.096627242
SIK1	-4.771191	2.091213	-2.281542	0.022516395	0.096627242
SLC38A1	-0.677387	0.29691	-2.281454	0.022521574	0.096627242
EP300	-1.010344	0.442914	-2.281131	0.022540704	0.096677081
ENSG00000267397	-4.324094	1.895751	-2.28094	0.022552023	0.096693394
ENSG00000261135	-2.462457	1.080323	-2.279371	0.022645048	0.097059906
LINC00158	-4.661272	2.045555	-2.278732	0.022682993	0.09713566
ENSG00000182109	-2.204454	0.967435	-2.278658	0.022687384	0.09713566
LCA5	-3.579109	1.570731	-2.278626	0.022689302	0.09713566
ENSG00000237851	-4.547206	1.995644	-2.278565	0.022692919	0.09713566
ENSG00000231856	-1.869723	0.820693	-2.278225	0.022713196	0.097190121

DDTL	-4.45289	1.954727	-2.278011	0.022725894	0.097212129
SRRT	-1.068268	0.469157	-2.276994	0.022786571	0.097439283
HSPA1B	-3.203295	1.407256	-2.27627	0.022829858	0.097560062
KLRG1	-1.970773	0.865874	-2.276051	0.022842939	0.097560062
TLR7	-1.776825	0.780664	-2.276044	0.022843362	0.097560062
ITPKB.AS1	-1.818257	0.79892	-2.275893	0.022852409	0.097560062
RPL30	-0.124959	0.054906	-2.275888	0.022852726	0.097560062
SBNO2	-1.947054	0.855654	-2.275516	0.022874985	0.097576503
SAP30	-4.709601	2.069699	-2.2755	0.022875943	0.097576503
LZTS3	-4.500383	1.977805	-2.275444	0.022879328	0.097576503
ENSG00000232807	-4.485704	1.972245	-2.274415	0.02294103	0.097807236
ENSG00000254614	-1.546634	0.680075	-2.274211	0.022953306	0.097827156
CABLES2	-3.288621	1.446313	-2.273796	0.022978276	0.097871415
RAB11B.AS1	-1.412899	0.621386	-2.273785	0.022978903	0.097871415
ENSG00000269898	-1.99541	0.877792	-2.273216	0.023013176	0.097984956
TRGV5	-2.226743	0.979667	-2.272958	0.023028682	0.098018542
CHAF1A	-1.901191	0.836573	-2.272593	0.023050698	0.098079806
PLXND1	-2.174964	0.957386	-2.271774	0.023100152	0.098203413
CCDC120	-1.998141	0.87956	-2.27175	0.023101586	0.098203413
IGLV2.14	-2.527753	1.112698	-2.271733	0.023102645	0.098203413
LIN52	-1.55626	0.68519	-2.271282	0.023129914	0.098286855
ENSG00000268568	-2.156409	0.949558	-2.270962	0.023149284	0.098336692
ENSG00000130520	-0.573624	0.252622	-2.270684	0.02316614	0.098348355
ENSG00000250938	-4.584401	2.018969	-2.270664	0.023167317	0.098348355
ENSG00000260563	-3.237993	1.426162	-2.270424	0.02318184	0.098373728
MCF2L	-4.437094	1.954397	-2.270313	0.023188584	0.098373728
CYHR1	-0.981107	0.432223	-2.269908	0.023213153	0.098445499
RRP9	-1.474512	0.649846	-2.269019	0.023267192	0.09864216
GALK2	-1.390858	0.613153	-2.268368	0.023306775	0.098777427
PTCD2	-1.541908	0.67989	-2.267878	0.02333665	0.098821038
TRIM44	1.339828	0.590791	2.267856	0.023337987	0.098821038
EMC1.AS1	-3.052233	1.345888	-2.267821	0.023340105	0.098821038
SNX13	-1.302517	0.57441	-2.267574	0.023355206	0.098852448
ZNF287	-1.834293	0.808973	-2.267434	0.023363755	0.098856114
GZF1	-1.653062	0.729247	-2.266805	0.023402139	0.098985972
CCNA2	-4.037019	1.781324	-2.266302	0.023432883	0.09908344
CDCA7	-4.570022	2.017929	-2.264709	0.023530526	0.099463626
TRUB1	-1.626977	0.718523	-2.264333	0.023553624	0.099528566
ENSG00000229852	-1.407436	0.621612	-2.26417	0.023563682	0.099537806
ENSG00000261158	-4.389455	1.938766	-2.264046	0.023571283	0.099537806
ENSG00000238198	-4.463469	1.971902	-2.263535	0.023602757	0.09960523
MPP7	-1.786303	0.789167	-2.263529	0.023603112	0.09960523
SRPK1	1.633121	0.721532	2.263409	0.023610473	0.09960523
MGST2	-2.091934	0.924492	-2.262793	0.023648466	0.099732814
ZKSCAN2	-1.960809	0.866877	-2.261923	0.023702165	0.099926526
PRADC1	-1.777825	0.786083	-2.261626	0.023720507	0.099971098
LINC02242	-3.327721	1.471615	-2.261272	0.023742443	0.100030784

GSK3A	-1.501026	0.663933	-2.26081	0.023771055	0.10011855
TM4SF19.AS1	-4.139753	1.831397	-2.260435	0.023794267	0.100183518
CCT3	1.009731	0.446769	2.260071	0.023816826	0.100245697
ENSG00000259943	-1.621058	0.717682	-2.258741	0.023899496	0.100560762
MAFG.DT	-4.319371	1.913262	-2.257595	0.023970919	0.100803486
ERN1	-3.19937	1.417178	-2.257564	0.02397285	0.100803486
ZRANB1	-1.458474	0.646093	-2.257374	0.023984686	0.100820305
ENSG00000263335	-2.199253	0.974404	-2.257024	0.024006566	0.100879321
PLLP	-4.303137	1.906868	-2.256651	0.024029866	0.100944267
ENSG00000236833	-4.491528	1.991469	-2.255384	0.024109246	0.101244669
AARS2	-1.944047	0.862084	-2.255055	0.024129847	0.101271993
MSANTD2	-1.645132	0.729539	-2.255029	0.024131494	0.101271993
ACOT2	-2.377951	1.054636	-2.254759	0.024148449	0.101310104
SLC14A1	-4.278184	1.898601	-2.253335	0.024238058	0.101652899
C2orf92	-4.244822	1.884224	-2.252822	0.024270351	0.101702257
ENSG00000227518	-4.244822	1.884224	-2.252822	0.024270351	0.101702257
FSD1	-2.506883	1.112813	-2.252745	0.024275255	0.101702257
PHLDB3	-1.401893	0.622393	-2.252423	0.024295568	0.101702257
TFB1M	-1.219823	0.541563	-2.252411	0.024296323	0.101702257
TOR4A	-1.698177	0.753943	-2.252396	0.024297252	0.101702257
BAHD1	-1.752336	0.77814	-2.251956	0.02432506	0.101785543
MIR497HG	-2.087689	0.927178	-2.251659	0.024343821	0.101830932
RNPEPL1	-1.110211	0.493109	-2.251452	0.024356899	0.101852525
SLC35G5	-2.015929	0.895487	-2.25121	0.024372271	0.101883695
ACTR3B	-1.802683	0.800846	-2.250973	0.024387277	0.101913314
SLC23A3	-2.031415	0.902804	-2.250116	0.024441557	0.102106984
SPPL3	-1.300436	0.578156	-2.24928	0.024494683	0.102295712
HDX	-2.097761	0.932982	-2.248448	0.024547645	0.102445804
ENDOG	-2.129959	0.947352	-2.248328	0.024555263	0.102445804
LANCL1	-1.553614	0.691079	-2.2481	0.024569845	0.102445804
PJA1	-1.304015	0.580076	-2.248007	0.024575748	0.102445804
PRR5L	-4.33688	1.929229	-2.247987	0.024577037	0.102445804
MXRA7	-2.23293	0.993311	-2.247965	0.024578394	0.102445804
FAM111B	-0.963139	0.428571	-2.247327	0.024619112	0.102555747
PTPRK	-1.817558	0.808789	-2.24726	0.024623442	0.102555747
MFN2	-1.567291	0.697466	-2.247123	0.024632136	0.102555747
NEK6	-1.706815	0.759579	-2.247053	0.024636653	0.102555747
ENSG00000270189	-1.541621	0.686127	-2.246845	0.024649921	0.102577793
LINC01252	-2.289869	1.019243	-2.246637	0.024663217	0.10259994
NEURL4	-1.966747	0.875878	-2.245458	0.024738717	0.102880762
SLFN13	-1.898954	0.845758	-2.245269	0.02475088	0.102898086
DHCR24	-1.924371	0.857295	-2.244701	0.02478735	0.103016418
LIMD1.AS1	-4.404831	1.96277	-2.244191	0.024820134	0.103119364
ZNF599	-2.178174	0.970989	-2.243254	0.024880421	0.103336467
ENSG00000234936	-1.879076	0.837862	-2.242704	0.024915922	0.103443231
PANK3	-1.314216	0.586023	-2.242601	0.024922545	0.103443231
ADGRG5	-2.132255	0.950846	-2.242482	0.024930245	0.103443231

LILRB3	-1.989644	0.887379	-2.242158	0.024951151	0.1034966
IGSF22	-2.192062	0.977869	-2.241672	0.024982556	0.103593474
ATL2	-1.560649	0.696334	-2.241237	0.025010732	0.103676897
MANEA	-1.732958	0.773308	-2.240969	0.025028074	0.103715373
ENSG00000267838	-2.350645	1.049111	-2.240607	0.025051572	0.103779323
LRRC8B	-1.706918	0.762081	-2.239811	0.025103186	0.103913403
ERLIN1	-1.716288	0.766276	-2.239777	0.025105377	0.103913403
JHY	-2.066328	0.922593	-2.239696	0.025110641	0.103913403
BRIX1	1.624117	0.725178	2.23961	0.025116242	0.103913403
BRF1	-1.76823	0.78959	-2.239427	0.025128142	0.103929222
PREP	-1.793708	0.801211	-2.238748	0.025172349	0.104064215
ENSG00000140743	-2.191011	0.978708	-2.238677	0.025176957	0.104064215
SYT15	-2.923976	1.306286	-2.238388	0.02519575	0.104108452
ENSG00000178386	-2.166562	0.968084	-2.23799	0.025221678	0.104182129
ENSG00000272049	-4.212955	1.882699	-2.237721	0.025239261	0.104193097
CPLANE1	-1.407949	0.6292	-2.237681	0.025241855	0.104193097
DDX21	1.467384	0.655821	2.237477	0.025255174	0.104193097
SLC51A	-2.546847	1.13828	-2.237453	0.025256724	0.104193097
ZDHHC13	-1.474584	0.65937	-2.236353	0.025328634	0.104434316
CLNK	-4.390754	1.963392	-2.236311	0.025331429	0.104434316
COLCA2	-1.890803	0.845607	-2.236031	0.025349755	0.104476393
WDR91	-1.933382	0.864863	-2.235478	0.025385963	0.104544492
UEVLD	-1.667394	0.745879	-2.235476	0.025386088	0.104544492
CCDC15	-2.549774	1.140671	-2.235329	0.025395758	0.104544492
ZNF468	-1.588566	0.710695	-2.235227	0.025402445	0.104544492
ZNF782	-2.158069	0.96551	-2.235159	0.025406903	0.104544492
CCDC189	-1.569822	0.702396	-2.234952	0.025420515	0.104559704
HNRNPU	0.944997	0.422845	2.234855	0.025426853	0.104559704
UBTD2	-1.888784	0.845255	-2.234573	0.025445367	0.104602406
ENSG00000257151	-1.863529	0.834016	-2.234405	0.025456455	0.104614568
ZNF793.AS1	-1.811657	0.810906	-2.234115	0.025475524	0.104659505
ZNF486	-1.824342	0.816675	-2.233865	0.025491944	0.104693533
KLHDC1	-2.109348	0.944353	-2.233644	0.025506527	0.104720002
GLMP	-1.638665	0.733808	-2.233098	0.025542487	0.10483419
SLC19A2	-4.290613	1.921982	-2.23239	0.025589226	0.104992529
ZNF446	-1.725333	0.77291	-2.232257	0.025597996	0.10499503
ENSG00000261187	-4.481142	2.007789	-2.231879	0.025622992	0.105064065
PATZ1	-1.819612	0.815367	-2.231648	0.025638234	0.105093073
TRIM68	-2.369077	1.062254	-2.230236	0.025731787	0.105442963
MRM1	-1.669778	0.748826	-2.22986	0.025756766	0.105493635
SEPSECS	-1.923033	0.862423	-2.229803	0.02576055	0.105493635
TDRKH	-1.971307	0.884408	-2.228957	0.025816754	0.105690158
SLAIN1	-1.947122	0.873663	-2.228687	0.025834744	0.105730168
FANCB	-1.864437	0.836706	-2.228305	0.02586017	0.105800577
IVD	-1.294609	0.581135	-2.227724	0.025898936	0.105925495
RPGRIP1L	-2.286741	1.026729	-2.22721	0.025933212	0.10603198
ENSG00000271553	-3.13323	1.407581	-2.225967	0.026016363	0.106338165

TNFRSF12A	-1.755952	0.789047	-2.225408	0.026053878	0.106408709
ENSG00000261474	-1.709574	0.768226	-2.225354	0.026057501	0.106408709
ZNF556	-3.53674	1.589304	-2.22534	0.026058432	0.106408709
MROH6	-2.442546	1.097859	-2.224828	0.026092804	0.106515265
PELI3	-1.834054	0.824526	-2.224374	0.026123321	0.106606018
TAPT1.AS1	-4.453344	2.002529	-2.22386	0.026157891	0.106670375
CCDC34	-1.333811	0.599789	-2.2238	0.026161921	0.106670375
EXD2	-2.115701	0.95145	-2.22366	0.026171352	0.106670375
ENSG00000235475	-4.248709	1.910695	-2.223646	0.026172253	0.106670375
FAM3C	1.069481	0.48107	2.22313	0.026207041	0.106778338
SNX19	-1.550756	0.697729	-2.222576	0.026244381	0.106889579
VPS13B	-1.512761	0.680674	-2.222446	0.026253162	0.106889579
CEBPZOS	-0.793911	0.357238	-2.222356	0.026259265	0.106889579
RAB3A	-2.129114	0.958383	-2.221569	0.026312443	0.107072169
PINK1.AS	-3.968137	1.786526	-2.221147	0.026340986	0.107154433
ACP1	1.02923	0.463617	2.22	0.026418738	0.107436757
SEPT7.AS1	-2.431608	1.095438	-2.219758	0.026435198	0.107469731
SLC17A9	-1.516842	0.683438	-2.219429	0.026457542	0.107526593
HOXB3	-2.112492	0.95198	-2.219051	0.026483229	0.10758296
TMEM154	1.488494	0.670828	2.218891	0.026494168	0.10758296
USP49	-2.012143	0.906838	-2.218856	0.026496495	0.10758296
ENSG00000278932	-3.211245	1.44761	-2.218308	0.026533824	0.107693653
ENSG00000131845	-2.03381	0.91687	-2.21821	0.026540497	0.107693653
ANGEL1	-2.092717	0.94349	-2.218059	0.026550808	0.107701526
RGS3	-1.656687	0.747108	-2.217466	0.026591276	0.107831688
COLEC12	-2.135529	0.963204	-2.21711	0.026615602	0.107896332
LINC00926	0.536461	0.24204	2.216413	0.026663206	0.108055265
C5orf30	-1.680607	0.758328	-2.216201	0.026677715	0.108080026
ZAP70	-3.456979	1.560325	-2.215551	0.026722269	0.108226453
ENSG00000261770	-2.227093	1.005314	-2.215321	0.02673801	0.108256127
AK2	-0.906774	0.409362	-2.215093	0.026753698	0.108285572
KLF11	-2.35143	1.061716	-2.214744	0.026777614	0.108316092
RANGRF	1.540976	0.695783	2.214738	0.026778075	0.108316092
RASAL1	-1.948043	0.879641	-2.214589	0.026788279	0.108323313
IGLV2.23	-4.182205	1.888593	-2.214455	0.026797486	0.108326503
LYZ	-3.513876	1.587902	-2.212905	0.026904222	0.108696598
FGF7	-2.199497	0.993983	-2.212812	0.026910636	0.108696598
ENSG00000258056	-1.828556	0.82637	-2.212757	0.026914383	0.108696598
ENSG00000227782	-2.859634	1.292427	-2.212608	0.026924693	0.10870412
NRIP2	-2.12046	0.958427	-2.212437	0.026936468	0.108709169
PAFAH1B2	-0.698263	0.315621	-2.212345	0.026942841	0.108709169
SERTAD3	-1.066792	0.482249	-2.212119	0.026958473	0.108727228
ENSG00000204814	-4.348872	1.966005	-2.212035	0.026964217	0.108727228
ZCCHC7	1.067854	0.482897	2.211352	0.027011447	0.108778905
NAGPA	-1.030511	0.466032	-2.211243	0.027018984	0.108778905
ENSG00000272058	-1.765055	0.798225	-2.211226	0.027020178	0.108778905
ARMC9	-1.739286	0.786572	-2.211225	0.027020282	0.108778905

TPK1	-1.541782	0.697282	-2.21113	0.027026808	0.108778905
LINC00339	-1.620374	0.732831	-2.211117	0.027027758	0.108778905
PINK1	-1.681167	0.760493	-2.210629	0.027061511	0.108880694
ENSG00000176320	-2.01653	0.912298	-2.210384	0.027078512	0.108915039
AK8	-4.550481	2.058853	-2.210202	0.02709117	0.108915537
ZBTB34	-2.550508	1.154004	-2.210138	0.027095565	0.108915537
ENSG00000277959	-2.334425	1.056345	-2.209908	0.027111541	0.10892568
CNNM3	-1.82805	0.827242	-2.209813	0.027118173	0.10892568
GFI1	-1.900146	0.859897	-2.209736	0.027123485	0.10892568
MFSD1	-1.320571	0.59768	-2.209495	0.027140202	0.108958809
TRMO	-1.559534	0.705988	-2.209011	0.027173888	0.109060019
TIPARP.AS1	-2.444347	1.106996	-2.20809	0.027238029	0.109283353
ENSG00000240219	-4.305805	1.950241	-2.207832	0.027255979	0.109321285
ITGA10	-2.241092	1.015187	-2.207565	0.02727462	0.1093386
ENSG00000255139	-4.258523	1.929093	-2.207527	0.027277291	0.1093386
IQCG	-1.44394	0.654426	-2.206421	0.027354509	0.109613974
ENAH	-4.296185	1.947461	-2.206044	0.027380919	0.109685643
CCND2	-1.897677	0.860309	-2.20581	0.027397332	0.10968853
C9orf66	-2.368924	1.073974	-2.205755	0.027401164	0.10968853
ENSG00000278882	-2.437434	1.105161	-2.205502	0.027418899	0.10968853
ABCA2	-1.840508	0.834515	-2.205483	0.027420187	0.10968853
PARK7	0.60248	0.273181	2.205425	0.027424264	0.10968853
VPS54	-1.632184	0.740285	-2.204805	0.027467813	0.109795328
ENSG00000268218	-1.643119	0.745246	-2.204801	0.027468031	0.109795328
NOMO2	-4.454402	2.0206	-2.204495	0.027489544	0.109847195
ENSG00000273151	-4.316768	1.95828	-2.204367	0.027498528	0.109848979
F8	-4.155911	1.885714	-2.203893	0.027531887	0.109948105
INAVA	-4.271941	1.939571	-2.202518	0.027628707	0.110293249
SHPK	-1.800416	0.817471	-2.202423	0.027635458	0.110293249
ZMYM1	-1.905913	0.865551	-2.201966	0.027667745	0.11038787
HIVEP3	-1.826717	0.829768	-2.201479	0.027702121	0.110490761
ENSG00000233912	-4.199874	1.908067	-2.201114	0.027727941	0.110559471
ARHGAP18	-1.646741	0.748216	-2.200891	0.027743739	0.110588194
NOD2	-2.758498	1.253468	-2.200694	0.027757722	0.110609666
ENSG00000261644	-3.128077	1.421564	-2.200447	0.027775217	0.110634391
ETFBKMT	-1.697711	0.771633	-2.200152	0.02779611	0.110634391
F2R	-2.107566	0.957927	-2.200131	0.027797573	0.110634391
ECHS1	1.319472	0.599792	2.199883	0.027815223	0.110634391
TSGA13	-4.026031	1.830231	-2.199739	0.027825385	0.110634391
ZNF208	-3.627573	1.649119	-2.199704	0.0278279	0.110634391
KHDC4	-0.7975	0.362549	-2.199702	0.027828061	0.110634391
MED14	-1.367966	0.621905	-2.199636	0.027832713	0.110634391
NIPAL4	-2.057019	0.935398	-2.199085	0.027871897	0.110733394
NR1D1	-1.872346	0.851436	-2.199043	0.027874832	0.110733394
BBS10	-2.054235	0.934467	-2.198295	0.027928096	0.110877236
APOBEC3B	-2.637592	1.199858	-2.198254	0.027931038	0.110877236
SELENON	-1.814173	0.82531	-2.198171	0.027936893	0.110877236

DNAJC5	-1.685698	0.766941	-2.19795	0.027952644	0.110905543
NCKAP5L	-1.788559	0.8143	-2.196439	0.028060528	0.111281242
SIN3B	-1.449934	0.660177	-2.196281	0.028071846	0.111281242
ENSG00000263089	-1.651616	0.752042	-2.196174	0.028079485	0.111281242
ZNF623	-1.526328	0.695005	-2.19614	0.02808193	0.111281242
POLA1	-1.893721	0.862541	-2.195513	0.028126827	0.111424839
ZDBF2	-1.94023	0.883818	-2.195283	0.028143315	0.111455845
THEM6	-1.640658	0.747453	-2.194999	0.028163659	0.111479846
AKAP12	-4.188918	1.908428	-2.194957	0.028166704	0.111479846
MLEC	1.586208	0.722791	2.19456	0.028195158	0.111558149
ENSG00000228439	-1.858938	0.847262	-2.194052	0.028231651	0.111648794
NT5DC2	-2.428167	1.106731	-2.194	0.028235422	0.111648794
IKZF1	1.380166	0.629179	2.193601	0.028264142	0.111704042
P4HA3	-3.696845	1.685314	-2.193564	0.028266757	0.111704042
C12orf60	-1.881445	0.85814	-2.19247	0.028345613	0.11195866
ZFP30	-1.60855	0.733684	-2.192428	0.028348591	0.11195866
RFLNB	-4.13859	1.888119	-2.191912	0.028385857	0.11207144
KCTD2	-1.829295	0.835095	-2.190523	0.028486333	0.112433634
C9orf64	-1.669963	0.76249	-2.190144	0.028513778	0.112474931
ENSG00000272563	-4.332924	1.97838	-2.190137	0.028514279	0.112474931
TRAF3IP2.AS1	-1.715951	0.783618	-2.18978	0.028540209	0.112515385
TMEM128	-1.202205	0.549014	-2.189755	0.028542023	0.112515385
ENSG00000258646	-3.263665	1.490957	-2.188972	0.028598842	0.112685447
FUT10	-1.909285	0.87225	-2.18892	0.028602679	0.112685447
ARHGEF37	-2.215587	1.012276	-2.188719	0.02861727	0.112708423
PIGO	-1.739797	0.795203	-2.187865	0.028679467	0.112918818
C2orf76	-1.464405	0.669494	-2.187333	0.028718243	0.113036902
SPIN4	-2.298168	1.050738	-2.187193	0.02872845	0.113042497
RHPN1	-1.920992	0.878594	-2.18644	0.028783462	0.113224338
ZNF543	-1.574771	0.72034	-2.186149	0.028804718	0.113241087
CCDC7	-1.931846	0.883679	-2.186141	0.028805322	0.113241087
ENSG00000275636	-4.232316	1.93678	-2.185233	0.028871739	0.113371526
SLC44A5	-3.810596	1.743813	-2.185209	0.028873538	0.113371526
KLHL21	-1.533088	0.70161	-2.185098	0.028881632	0.113371526
PDE8A	-1.516713	0.694119	-2.185091	0.028882167	0.113371526
IDUA	-4.276794	1.957501	-2.184823	0.028901829	0.113371526
PAIP2B	-1.496315	0.684886	-2.184765	0.028906093	0.113371526
CPEB4	-1.218918	0.557919	-2.18476	0.028906414	0.113371526
IGHG4	-4.428227	2.026903	-2.184725	0.02890899	0.113371526
PPP1R12A	-0.749545	0.343259	-2.183612	0.028990761	0.113621533
ENSG00000242349	-2.188449	1.002243	-2.18355	0.028995308	0.113621533
CELSR1	-2.038413	0.933554	-2.183497	0.028999232	0.113621533
MYLPF	-1.700966	0.779469	-2.182213	0.029093848	0.113928832
DUSP4	-2.46669	1.130373	-2.182192	0.029095371	0.113928832
RARA	-0.787294	0.360867	-2.181672	0.029133773	0.114010185
SEMA6A.AS1	-3.104276	1.422889	-2.18167	0.029133869	0.114010185
MINPP1	-1.526343	0.699693	-2.181447	0.029150402	0.114040201

LRRC56	-1.981991	0.909018	-2.180364	0.02923048	0.11431872
ZNF518B	-1.553766	0.712948	-2.179353	0.029305475	0.114571769
AP3S2	-1.652546	0.758309	-2.179251	0.029312992	0.114571769
NUP107	-0.910857	0.418003	-2.179069	0.029326566	0.114590015
UCKL1.AS1	-1.845618	0.847109	-2.178725	0.029352073	0.114654865
MRFAP1L1	-1.064344	0.488783	-2.177541	0.029440248	0.114935099
NOTCH1	-1.52755	0.701509	-2.177522	0.029441679	0.114935099
LBX2.AS1	-3.935016	1.807247	-2.177354	0.02945418	0.114949025
CCDC168	-4.39124	2.017035	-2.177077	0.02947482	0.11497986
PLTP	-2.025183	0.93026	-2.177008	0.029479953	0.11497986
NSMF	-2.002232	0.919817	-2.176772	0.029497601	0.115013827
TAF1A.AS1	-2.222845	1.021332	-2.176418	0.029524008	0.115081917
MAP3K9	-1.859707	0.854757	-2.175714	0.029576616	0.115252067
MTERF2	-1.829185	0.84083	-2.175451	0.029596357	0.115261319
LINC02397	0.764358	0.351373	2.175349	0.029604007	0.115261319
MMP25.AS1	-1.659697	0.762966	-2.175324	0.029605865	0.115261319
ZNF319	-2.455281	1.128969	-2.174799	0.029645217	0.115379613
LINC02384	-3.436916	1.580487	-2.174592	0.02966067	0.115404849
WIZ	-1.58032	0.726799	-2.174355	0.029678465	0.115439182
ENSG00000277324	-1.868685	0.859585	-2.173939	0.02970968	0.115525673
ENSG00000223473	-1.82022	0.837438	-2.173557	0.029738426	0.115602516
SNHG31	-4.341713	1.998009	-2.173019	0.029778858	0.115706913
EIF4G2	0.796586	0.366605	2.172869	0.029790154	0.115706913
ZBED6CL	-2.067621	0.951575	-2.172841	0.029792259	0.115706913
EPM2A	-2.27618	1.048039	-2.171846	0.029867278	0.115963268
KATNAL2	-4.024778	1.853308	-2.171672	0.029880425	0.115979315
FAM157C	-1.977263	0.911087	-2.170223	0.029989952	0.116369333
ENSG00000233184	-1.246637	0.574498	-2.16996	0.03000987	0.116411517
SNURF	-2.208329	1.017862	-2.169576	0.030038975	0.116454591
TNFAIP1	-1.475241	0.679968	-2.169575	0.030039076	0.116454591
NDOR1	-1.933535	0.891334	-2.16926	0.030062951	0.116484182
C11orf95	-1.878609	0.866069	-2.169122	0.030073436	0.116484182
FKTN	-1.886569	0.869741	-2.169116	0.030073867	0.116484182
ZNF845	-1.945695	0.897288	-2.168417	0.030126981	0.116654789
POLQ	-4.143826	1.91185	-2.167442	0.030201146	0.11689188
ENSG00000248734	-4.102174	1.892694	-2.167374	0.030206381	0.11689188
MATK	-2.422888	1.118112	-2.166946	0.030238998	0.116923833
IDE	-1.517785	0.700473	-2.1668	0.030250139	0.116923833
DLGAP1.AS2	-1.98556	0.916387	-2.166727	0.030255705	0.116923833
CYP46A1	-4.385872	2.024217	-2.166701	0.030257663	0.116923833
ENSG00000227495	-3.164644	1.460603	-2.166669	0.030260073	0.116923833
ENSG00000275441	-2.028395	0.936251	-2.166508	0.030272397	0.116926778
ENSG00000235609	-1.875473	0.865718	-2.166379	0.030282239	0.116926778
KNL1	-1.775507	0.819602	-2.166302	0.030288098	0.116926778
ADGRB2	-1.979739	0.914015	-2.165981	0.03031264	0.116986423
HDAC7	1.477728	0.682335	2.165694	0.030334547	0.117035866
SWT1	-1.681859	0.776766	-2.165206	0.030371886	0.117144802

ENSG00000272148	-4.406848	2.03545	-2.165048	0.030383998	0.117156398
CLEC11A	-1.715439	0.792462	-2.164696	0.030410993	0.11722536
ZNF708	-1.24732	0.576304	-2.164344	0.030437987	0.117294274
FBXL18	-1.855897	0.857704	-2.163797	0.03047992	0.117394592
HIST1H2BC	-1.737686	0.803084	-2.163767	0.030482267	0.117394592
TOP2A	-3.284977	1.518288	-2.163606	0.030494581	0.117406875
ENSG00000253645	-1.755676	0.811689	-2.162993	0.030541739	0.117544707
VSIG10L	-2.100382	0.9711	-2.16289	0.030549675	0.117544707
PLPP1	-1.880709	0.869578	-2.162784	0.030557787	0.117544707
QRICH2	-4.421802	2.044837	-2.162422	0.030585645	0.117616704
ENO3	-1.940007	0.897423	-2.161753	0.030637191	0.117779723
RWDD3	-1.931937	0.894063	-2.160852	0.030706803	0.118012077
DXO	-1.258828	0.582687	-2.160385	0.030742879	0.118115445
SLC9A3R2	-3.740323	1.73189	-2.159678	0.030797627	0.118290467
DDX11	-1.633081	0.756256	-2.159429	0.030816871	0.118329059
KBTBD2	-1.47427	0.682793	-2.159174	0.030836633	0.118369619
STK17B	-0.591631	0.274049	-2.158849	0.030861896	0.118431262
ENSG00000246790	-1.637034	0.758346	-2.158689	0.030874266	0.118443407
SH2B2	-3.977486	1.84351	-2.157562	0.030961939	0.118744344
MORN1	-1.841174	0.853409	-2.157434	0.030971864	0.118747011
ZNF557	-1.24194	0.575934	-2.156393	0.031052956	0.119022457
IRS1	-1.694484	0.785892	-2.156128	0.031073654	0.11906632
ABCC4	-4.231682	1.962894	-2.155837	0.031096357	0.119117839
LACTB2.AS1	-2.128899	0.987573	-2.155688	0.031108029	0.119127087
NCEH1	-3.035638	1.408622	-2.155041	0.031158634	0.119283698
PDCD6	-0.575435	0.267032	-2.154928	0.031167467	0.119283698
FBXO30	-1.931521	0.896689	-2.15406	0.031235464	0.11950839
FCHO2	-1.80091	0.836129	-2.153866	0.031250709	0.119520392
SPIB	0.84196	0.390922	2.153783	0.031257179	0.119520392
IP6K1	-1.1304	0.525035	-2.153	0.031318642	0.119685315
NLRX1	-1.789172	0.831014	-2.152997	0.031318913	0.119685315
HCK	-1.653967	0.768316	-2.152717	0.031340964	0.119734022
ENSG00000272462	-2.966315	1.378681	-2.15156	0.031431995	0.120046152
NELFCD	-0.779058	0.362112	-2.15143	0.031442309	0.120049909
USP30	-1.889252	0.878388	-2.150818	0.031490566	0.120180072
ENSG00000261136	-1.644078	0.764441	-2.150693	0.031500439	0.120180072
IL6ST	-1.660734	0.772204	-2.150643	0.031504421	0.120180072
UCHL5	-1.159812	0.539375	-2.15029	0.03153227	0.120247349
ENSG00000249684	-3.00391	1.397071	-2.150148	0.031543482	0.120247349
ENSG00000258727	-2.299992	1.069732	-2.150065	0.031550093	0.120247349
MAMSTR	-1.802607	0.838711	-2.149259	0.031613893	0.120454831
CLIC3	1.727645	0.804084	2.148587	0.031667128	0.12060536
A4GALT	-1.342852	0.625012	-2.148524	0.031672147	0.12060536
KLRF1	-4.148453	1.931036	-2.148304	0.031689628	0.120636226
ENSG00000273240	-4.234514	1.971338	-2.148041	0.031710534	0.120680108
STRADA	-2.238594	1.042347	-2.147649	0.031741669	0.120762877
RINT1	-1.52724	0.711538	-2.146393	0.031841672	0.121080239

FASN	-1.93666	0.902298	-2.146365	0.031843905	0.121080239
DET1	-1.78642	0.832351	-2.146233	0.031854389	0.121080461
ZNF461	-1.766818	0.823268	-2.146103	0.031864727	0.121080461
MGAT5B	-1.786111	0.832324	-2.145932	0.031878399	0.121080461
NUSAP1	-1.793404	0.835738	-2.145892	0.031881604	0.121080461
OPN3	-1.153191	0.537467	-2.145605	0.031904537	0.121116824
SERPINF2	-2.45958	1.146371	-2.145536	0.031910005	0.121116824
ENSG00000273008	-2.478225	1.155428	-2.144854	0.031964485	0.121238853
LCMT2	-1.849751	0.862441	-2.144786	0.031969966	0.121238853
APH1B	-1.445737	0.674072	-2.14478	0.031970423	0.121238853
LAMC1	-1.642184	0.765807	-2.144385	0.032002029	0.121322956
CHCHD6	-1.566935	0.730779	-2.144199	0.032016967	0.121343835
DIP2C	-2.769497	1.292036	-2.143513	0.0320719	0.121516237
ENSG00000273723	-1.663805	0.776282	-2.143298	0.032089148	0.121545795
ENSG00000246889	-1.894564	0.88414	-2.142832	0.032126615	0.121561584
ENSG00000254741	-3.924393	1.831428	-2.142805	0.03212874	0.121561584
LINC01534	-1.558881	0.727496	-2.142803	0.032128894	0.121561584
PLD3	-1.118366	0.521924	-2.142776	0.032131106	0.121561584
KIAA1958	-1.806633	0.843449	-2.141959	0.032196797	0.121766436
MTMR3	-1.500427	0.700554	-2.141771	0.032211946	0.121766436
URGCP	-1.656475	0.773421	-2.14175	0.032213643	0.121766436
ENSG00000247765	-3.248253	1.516827	-2.141479	0.032235466	0.121813138
SYT11	-1.723058	0.804888	-2.140744	0.032294706	0.122001168
ZNF280C	-2.911761	1.360783	-2.139769	0.032373404	0.122262575
NOXA1	-2.209803	1.032823	-2.139577	0.032389004	0.122285598
NDFIP2	-4.331754	2.024862	-2.139283	0.032412729	0.122330609
EPOR	-1.621052	0.757837	-2.139051	0.03243153	0.122330609
NUDT13	-2.602743	1.216823	-2.138966	0.032438439	0.122330609
IBA57	-1.663985	0.777942	-2.138958	0.03243904	0.122330609
TMEM117	-1.770305	0.827693	-2.138842	0.032448463	0.122330609
DNAJC13	-1.390334	0.650205	-2.138301	0.032492355	0.122371969
ENSG00000273783	-3.957124	1.850645	-2.138241	0.032497176	0.122371969
ZCCHC4	-1.70999	0.799745	-2.138168	0.032503135	0.122371969
CCL4L2	-2.578931	1.206147	-2.138156	0.032504095	0.122371969
CENPV	-1.760142	0.823219	-2.13812	0.032506986	0.122371969
IQCK	-1.852257	0.866632	-2.137305	0.03257318	0.12258529
ENSG00000262165	-3.154432	1.475988	-2.137166	0.032584471	0.122591928
ZNF688	1.528191	0.715125	2.136957	0.032601488	0.122620096
ZNF682	-1.553915	0.727252	-2.136695	0.032622831	0.122664514
BLNK	-0.497953	0.233138	-2.135877	0.032689433	0.122861221
HEATR6	-1.362913	0.638154	-2.135711	0.032703015	0.122861221
AMN	-2.292307	1.073355	-2.135646	0.03270825	0.122861221
EDEM3	-1.471139	0.688885	-2.135536	0.032717244	0.122861221
BSPRY	-4.14359	1.940367	-2.135467	0.032722888	0.122861221
ZKSCAN7	-2.531938	1.186148	-2.134588	0.032794717	0.123072658
USP42	-1.485096	0.695744	-2.134543	0.032798332	0.123072658
IQCD	-1.769518	0.82926	-2.133853	0.03285483	0.123238727

SOX12	-1.553708	0.728152	-2.133768	0.032861745	0.123238727
BRCA1	-1.680713	0.787744	-2.133577	0.032877384	0.123261452
PRR12	-1.441304	0.675784	-2.132787	0.032942238	0.123436773
TSG101	1.452527	0.681051	2.132773	0.032943334	0.123436773
PNPLA7	-1.766546	0.828364	-2.132573	0.032959763	0.123462378
ZNF213	-1.774035	0.832059	-2.132102	0.032998471	0.123557859
GNAZ	-2.121422	0.995046	-2.131985	0.033008109	0.123557859
ENSG00000267058	-1.504011	0.705475	-2.131912	0.033014061	0.123557859
IRGQ	-1.568875	0.736122	-2.131269	0.033066952	0.123719823
FLT1	-4.154092	1.949236	-2.131139	0.033077714	0.123724111
ZFAND2B	-0.949678	0.445659	-2.130952	0.033093083	0.123739726
CCNF	-1.850787	0.868566	-2.130855	0.033101122	0.123739726
SLC38A6	-2.202477	1.033805	-2.130457	0.033133874	0.123826185
ZFAND4	-1.865388	0.875973	-2.129503	0.033212686	0.12405484
RBM11	-3.96476	1.861849	-2.129475	0.033215012	0.12405484
PRX	-4.080301	1.916204	-2.129366	0.033223982	0.12405484
ZNF555	-1.747409	0.820751	-2.129037	0.033251205	0.124120468
SSBP3	-1.694289	0.795854	-2.128895	0.033262915	0.124128169
NPIPA1	-1.697616	0.797508	-2.128651	0.033283185	0.124167798
PTPRS	-2.210845	1.038989	-2.127881	0.033346941	0.124369593
CFAP44	-1.667648	0.783898	-2.127378	0.033388703	0.124489263
IGHV3.7	-3.43029	1.612553	-2.127242	0.033400021	0.124495384
ZNF236	-1.404357	0.660288	-2.126885	0.033429635	0.124569682
SLC37A2	-2.043116	0.960799	-2.126477	0.033463548	0.124592372
TAF6L	-1.552948	0.730316	-2.126405	0.033469534	0.124592372
PPP1R12A.AS1	-4.059071	1.908894	-2.1264	0.033469986	0.124592372
SMIM11B	-4.224969	1.986962	-2.126346	0.033474456	0.124592372
VSIG10	-1.621077	0.76245	-2.126142	0.033491421	0.124619467
KDM7A.DT	-1.752625	0.82441	-2.125915	0.033510374	0.124653942
EGR3	-4.016108	1.889732	-2.125227	0.033567704	0.124831113
CD53	-0.549657	0.258685	-2.124811	0.033602389	0.124923994
TTC39B	-1.811479	0.85304	-2.123559	0.033707068	0.125276963
NHLRC4	-2.354158	1.10882	-2.123121	0.033743714	0.125349785
BCL7C	1.42615	0.671732	2.123092	0.033746145	0.125349785
DOP1A	-1.669052	0.786253	-2.122793	0.033771164	0.125400724
NPIPB2	-3.177372	1.496857	-2.122696	0.033779351	0.125400724
ZNF526	-1.233591	0.58118	-2.122563	0.033790464	0.125405798
MTMR10	-1.713066	0.807177	-2.122293	0.033813108	0.125453652
PIK3CD.AS1	-1.807503	0.851897	-2.121739	0.033859686	0.125590249
PCBP2	0.430627	0.203032	2.120984	0.03392314	0.125780228
ERCC6L2	-1.387489	0.65422	-2.12083	0.033936109	0.125780228
ENSG00000276476	-2.109643	0.994774	-2.120727	0.033944798	0.125780228
LINC01137	-2.219362	1.046541	-2.120665	0.033950006	0.125780228
ENSG00000270761	-3.030925	1.42952	-2.12024	0.033985839	0.12587674
NIP7	-1.09694	0.517438	-2.119946	0.034010591	0.125931331
PPM1F	-1.440905	0.679726	-2.119833	0.034020153	0.125931331
RIOX1	-1.750814	0.826291	-2.118883	0.034100352	0.126159481

FAM102A	-1.228629	0.579863	-2.118825	0.034105236	0.126159481
ENSG00000276900	-1.235647	0.583209	-2.118702	0.034115686	0.126159481
ENSG00000214248	-3.01086	1.421129	-2.118639	0.034121007	0.126159481
ZNF415	-2.660785	1.256706	-2.117269	0.03423701	0.126552027
ZNF749	-2.626811	1.240878	-2.116897	0.034268585	0.126632362
PSMG3.AS1	-1.651505	0.780316	-2.116457	0.034305956	0.126715918
ZNF85	-1.33064	0.628728	-2.116399	0.034310893	0.126715918
AVPI1	-1.988171	0.93953	-2.116134	0.034333439	0.126737987
HOMEZ	-1.483553	0.701108	-2.116011	0.034343895	0.126737987
PRCC	-0.990821	0.468302	-2.115776	0.034363864	0.126737987
FAM129A	-2.312949	1.093197	-2.115766	0.034364756	0.126737987
GNAQ	-2.435795	1.151268	-2.11575	0.034366118	0.126737987
MOV10	-1.440506	0.681012	-2.115242	0.034409343	0.126828591
MAATS1	-4.148103	1.961065	-2.115229	0.0344104	0.126828591
MAP11	-1.514982	0.716478	-2.114484	0.034473991	0.127026588
SFXN2	-1.748227	0.827016	-2.113898	0.034523993	0.127174412
ENSG00000224046	-2.788877	1.319951	-2.112864	0.034612369	0.127463468
ARRDC3.AS1	-2.008572	0.950856	-2.112382	0.034653663	0.127579023
FAM149B1	-1.723781	0.816255	-2.111817	0.034702114	0.127707198
LRRC3	-4.561981	2.16033	-2.111706	0.034711689	0.127707198
RPL12	0.29119	0.137898	2.111629	0.034718254	0.127707198
PLXNC1	-1.974225	0.935026	-2.111413	0.034736838	0.127739038
NMRAL1	-1.234541	0.584815	-2.110993	0.034772954	0.127827042
ENSG00000232611	-1.567348	0.742501	-2.110903	0.034780638	0.127827042
YJEFN3	-2.040854	0.966988	-2.110527	0.034813016	0.127909502
GAB1	-1.743685	0.826248	-2.110367	0.034826782	0.127923554
LINC00342	-1.597708	0.757229	-2.10994	0.034863485	0.128021821
ENSG00000231551	-1.607372	0.762053	-2.109265	0.034921735	0.128164622
PDE9A	-2.528926	1.198997	-2.109202	0.034927129	0.128164622
ABITRAM	-1.89392	0.897957	-2.109143	0.034932255	0.128164622
PIEZO1	-1.626236	0.771153	-2.108837	0.034958613	0.128224763
AOPEP	-2.021011	0.958432	-2.108664	0.034973617	0.128225374
ARNTL2	-2.13391	1.012001	-2.108605	0.03497871	0.128225374
ENSG00000262202	-1.902697	0.902443	-2.108385	0.034997673	0.128248899
ENSG00000260517	-1.724742	0.818072	-2.1083	0.035005062	0.128248899
DBR1	-1.629402	0.773022	-2.107834	0.035045311	0.128359813
PTCD1	-1.543501	0.732337	-2.107638	0.03506235	0.128385674
ENSG00000251615	-3.132645	1.486467	-2.107443	0.035079161	0.128405088
ZBP1	-1.73934	0.82537	-2.107346	0.035087611	0.128405088
MTBP	-1.899246	0.901562	-2.106618	0.035150689	0.128599351
KDM1B	-1.725491	0.819134	-2.106483	0.035162392	0.128605599
SMARCC2	-0.867227	0.411897	-2.105449	0.035252281	0.128897728
ATP6V1E1	1.397321	0.66378	2.105096	0.035282978	0.128973317
ATG5	-0.967715	0.459738	-2.104927	0.035297644	0.128990282
SLC16A7	-1.12092	0.532608	-2.104588	0.035327212	0.129061682
NDUFB3	0.930301	0.442171	2.103937	0.035383935	0.129232213
APOM	-1.74843	0.83118	-2.103553	0.035417498	0.129318089

VPS36	1.428197	0.679032	2.103284	0.03544093	0.129366936
ZNF117	-1.679653	0.79871	-2.102956	0.035469581	0.129434799
ENSG00000261351	-1.675508	0.796967	-2.102356	0.035522121	0.12955407
ZNF37A	-0.934602	0.444551	-2.102353	0.035522403	0.12955407
RNF43	-1.443493	0.686666	-2.102175	0.035537972	0.129574123
LINC01772	-1.733743	0.824887	-2.101795	0.035571247	0.129613999
PRKRA	-0.804887	0.382959	-2.101756	0.035574696	0.129613999
ENSG00000259826	-3.282296	1.56173	-2.101705	0.035579128	0.129613999
SEC24D	-1.737454	0.826831	-2.10134	0.035611113	0.129693799
EIF3J	1.114943	0.530664	2.101036	0.035637842	0.129754417
ENSG00000238009	-4.038527	1.922485	-2.100681	0.035669004	0.129831137
PGBD1	-1.931264	0.919453	-2.100449	0.035689377	0.129868556
KRBA2	-1.653731	0.787461	-2.10008	0.035721778	0.129949708
ERCC8	-1.882361	0.896533	-2.0996	0.035764025	0.130066622
ENSG00000271737	-1.72356	0.821032	-2.099261	0.035793921	0.130138564
ENSG00000259834	-1.609501	0.766805	-2.098969	0.035819603	0.130195151
AMZ1	-1.808557	0.861982	-2.098137	0.035893065	0.130425322
ARHGEF35	-4.323654	2.06083	-2.098016	0.035903705	0.130427152
LMBR1	-1.49542	0.713016	-2.097316	0.035965654	0.130615316
C7orf50	-0.594603	0.283589	-2.096708	0.036019475	0.130773866
CEP85	-1.82041	0.868422	-2.096228	0.036061937	0.1308911
DLAT	-1.218277	0.581252	-2.095954	0.036086302	0.130942597
EXOSC2	-1.288456	0.614864	-2.095514	0.036125296	0.131047133
PDE4A	-4.07356	1.94491	-2.094472	0.036217948	0.131338163
LHFPL4	-1.77046	0.845338	-2.094382	0.036225938	0.131338163
ENSG00000232010	-3.985134	1.9029	-2.094242	0.03623841	0.131346371
CYB5R4	1.384491	0.661333	2.093487	0.036305729	0.131549585
ENSG00000260257	-1.491151	0.712317	-2.09338	0.036315219	0.131549585
MASTL	-1.789921	0.855084	-2.093269	0.036325148	0.131549585
SLC30A1	-1.904101	0.909736	-2.093025	0.036346906	0.131591345
LRRC69	-1.72665	0.825064	-2.092748	0.036371612	0.131643752
C17orf49	1.521224	0.72702	2.092409	0.036401919	0.131716392
CNIH3	-3.92268	1.87489	-2.092219	0.036418907	0.131740816
ENSG00000271380	-3.117346	1.49077	-2.091098	0.036519284	0.132066786
MMP28	-4.243475	2.029787	-2.090601	0.036563844	0.132190777
ALKBH8	-1.753385	0.838986	-2.089887	0.036627974	0.132385434
ZNF529.AS1	-1.498154	0.717168	-2.088988	0.036708834	0.132640427
ENSG00000273183	-3.244703	1.553682	-2.088396	0.036762144	0.132788854
GPN3	-1.043919	0.499901	-2.088253	0.036775063	0.132788854
URB1	-1.828515	0.875647	-2.088188	0.036780872	0.132788854
STK17A	0.57731	0.276524	2.087738	0.036821512	0.132898289
KIAA0753	-1.62358	0.777737	-2.087569	0.036836723	0.132915905
ENSG00000259772	-1.735253	0.831284	-2.087437	0.036848684	0.132921788
MAGED1	-1.503754	0.720684	-2.086566	0.036927384	0.133168345
TXLNG	-1.333144	0.638976	-2.086376	0.03694462	0.133193172
EVA1B	-1.592504	0.763539	-2.085688	0.037006881	0.133380263
ZNF772	-1.779394	0.853413	-2.085032	0.037066419	0.133553837

DDX60	-1.748971	0.838864	-2.084928	0.037075799	0.133553837
SERHL2	-1.752476	0.840638	-2.084698	0.037096726	0.133578789
IQCE	-1.560255	0.748497	-2.084518	0.037113067	0.133578789
HEXA	-0.678032	0.325272	-2.084509	0.03711387	0.133578789
CCDC71L	-1.520367	0.729428	-2.084329	0.03713026	0.133600408
RANBP10	-1.480733	0.710509	-2.084045	0.037156092	0.133616505
ST3GAL3	-1.991661	0.955682	-2.08402	0.037158369	0.133616505
RBM26	1.321821	0.63429	2.083937	0.037165887	0.133616505
CMTM8	-2.90068	1.392366	-2.083274	0.037226225	0.133793043
HMGA1P4	-1.597353	0.76679	-2.083169	0.037235788	0.133793043
NDUFA2	0.647855	0.311107	2.08242	0.037304107	0.1340011
LINC01684	-1.753843	0.842357	-2.082067	0.037336349	0.134079487
MMP7	-2.05915	0.989088	-2.081868	0.037354545	0.134107404
SLC25A29	-2.241887	1.077064	-2.08148	0.037389988	0.134197204
FADS2	-1.631382	0.783853	-2.081234	0.037412493	0.134240533
EVI5	-1.275888	0.61312	-2.080974	0.037436305	0.134288524
BRMS1L	-1.71733	0.825478	-2.080406	0.037488265	0.134437433
LRP5L	-1.578845	0.7592	-2.079617	0.037560688	0.134659618
SOGA1	-1.500125	0.721855	-2.078152	0.037695393	0.135068595
ENSG00000273226	-2.027268	0.975517	-2.078148	0.037695759	0.135068595
HAPLN3	-1.812229	0.872194	-2.077783	0.037729338	0.135127906
ENSG00000269973	-2.378773	1.144885	-2.07774	0.037733315	0.135127906
ZNF252P.AS1	-4.180838	2.012658	-2.077272	0.037776492	0.135206596
XRCC3	-1.669662	0.80378	-2.077263	0.03777731	0.135206596
RITA1	-0.997974	0.480483	-2.077024	0.037799365	0.135206596
ENSG00000237481	-4.206453	2.025235	-2.077019	0.037799809	0.135206596
ENSG00000245317	-1.987047	0.956722	-2.076932	0.037807829	0.135206596
ZNF10	-1.734969	0.835546	-2.076451	0.037852288	0.135294965
TIAM1	-3.683681	1.774039	-2.076437	0.037853569	0.135294965
ZNF432	-1.610691	0.775749	-2.076304	0.037865885	0.1353014
CREB3	-0.977688	0.471143	-2.07514	0.037973589	0.135648576
IDNK	-1.555503	0.749816	-2.074513	0.038031698	0.135818445
PER2	-1.726784	0.833133	-2.072639	0.038205905	0.136402714
NFYC.AS1	-1.846884	0.891223	-2.072304	0.038237134	0.136476338
VAV3.AS1	-1.712542	0.826515	-2.072005	0.038264969	0.136536681
ZNF417	-1.590828	0.767852	-2.071791	0.03828493	0.136536681
NMRK1	-1.622709	0.783243	-2.071781	0.038285874	0.136536681
ADCY3	-1.889211	0.911993	-2.071518	0.038310395	0.136549783
NT5E	-1.491174	0.719847	-2.071514	0.038310773	0.136549783
DNAJC28	-2.366276	1.142433	-2.07126	0.038334466	0.136596394
ENSG00000272040	-3.321862	1.60416	-2.07078	0.038379318	0.136718351
THAP8	-1.622955	0.783831	-2.070544	0.038401473	0.136759413
ZNF470	-1.681506	0.812331	-2.069977	0.038454502	0.136863872
ST3GAL6	-3.192942	1.542563	-2.069894	0.038462225	0.136863872
CYTIP	-0.387679	0.187295	-2.069889	0.038462716	0.136863872
ENOSF1	-1.879917	0.908412	-2.069454	0.038503496	0.136971105
ZNF781	-1.496925	0.723601	-2.068715	0.038572826	0.137164049

SAP30L	-1.462536	0.707001	-2.068649	0.038579054	0.137164049
CLUAP1	-1.175827	0.56862	-2.067863	0.038652907	0.137388662
PSMD9	-0.855696	0.413844	-2.067678	0.038670339	0.137412663
GALNS	-1.423138	0.68833	-2.067524	0.038684812	0.13742614
SLC37A4	-1.657122	0.801832	-2.066671	0.038765212	0.137673747
TCP11L1	-1.578923	0.764048	-2.066524	0.03877905	0.137684887
HLA.G	-2.116385	1.024363	-2.06605	0.038823755	0.137805588
IGHV3.23	-1.760601	0.852278	-2.065758	0.038851358	0.137865535
TYW1B	-1.711717	0.82871	-2.06552	0.038873817	0.137907195
ZNF625	-1.751556	0.848089	-2.065296	0.038894971	0.137926488
ENSG00000124593	-1.883593	0.912047	-2.065236	0.038900694	0.137926488
MPEG1	1.439556	0.697209	2.064742	0.038947392	0.138054019
NAPSA	-1.6706	0.809311	-2.064226	0.038996312	0.138189354
F8A1	-1.713755	0.830309	-2.063998	0.039017901	0.138227788
QSOX1	-1.703532	0.825561	-2.063484	0.039066676	0.138362488
NAGLU	-1.777651	0.861546	-2.063326	0.03908162	0.138377328
PPP1R13L	-1.542528	0.747929	-2.062399	0.0391698	0.138651392
ESD	0.841589	0.408099	2.062219	0.039186932	0.138673887
DNAJC18	-1.443627	0.700105	-2.062016	0.039206222	0.138681523
DPH6	-1.521615	0.737943	-2.061969	0.039210646	0.138681523
CERNA1	-1.789546	0.868808	-2.059771	0.039420445	0.139385235
COX10	-1.573194	0.76405	-2.05902	0.039492314	0.139597059
ENSG00000260278	-2.042167	0.991864	-2.058918	0.039502051	0.139597059
KDM4A	-1.501492	0.729675	-2.057755	0.039613692	0.139938211
USP37	-1.455218	0.707211	-2.057685	0.039620339	0.139938211
LAMA5	-1.836913	0.89308	-2.056829	0.039702721	0.140172338
NIFK	1.383522	0.672698	2.056678	0.039717228	0.140172338
HERC3	-1.552021	0.754633	-2.056656	0.039719309	0.140172338
LMCD1	-6.280579	3.054614	-2.056096	0.039773266	0.140243916
CD109	-6.280579	3.054614	-2.056096	0.039773266	0.140243916
TRDC	-6.280579	3.054614	-2.056096	0.039773266	0.140243916
GPSM2	-1.475885	0.717845	-2.055993	0.039783189	0.140243916
ASH1L	0.984831	0.47909	2.055629	0.039818296	0.140329227
HLA.DQB2	-3.91484	1.905017	-2.055016	0.039877451	0.140499224
ENSG00000272894	-1.71603	0.835126	-2.054817	0.039896676	0.140528479
WSB1	-0.607154	0.295518	-2.054541	0.039923348	0.14053825
TMEM143	-1.476292	0.718569	-2.054491	0.039928234	0.14053825
SPACA9	-1.533115	0.746241	-2.054449	0.039932217	0.14053825
ZNF570	-1.608754	0.783135	-2.054247	0.03995175	0.140568546
CKB	-1.816148	0.884255	-2.053874	0.039987887	0.140633226
ENSG00000259793	-3.82862	1.864135	-2.053832	0.039991993	0.140633226
PHLDA1	-1.965404	0.957448	-2.052752	0.040096609	0.140946952
CNKSR2	-1.742231	0.848757	-2.052685	0.040103116	0.140946952
LIG4	-1.794949	0.874516	-2.052505	0.040120608	0.140969925
RAD51B	-1.725047	0.840677	-2.051974	0.040172142	0.141091341
USP10	-0.927307	0.45193	-2.051883	0.040181063	0.141091341
ENSG00000227468	-2.58772	1.261188	-2.051811	0.04018806	0.141091341

ENSG00000255031	-1.709613	0.833319	-2.051569	0.040211534	0.141116881
PFKL	-0.738751	0.360101	-2.05151	0.040217269	0.141116881
TMEM134	-0.614751	0.299721	-2.05108	0.040259191	0.141203419
EFNB1	-2.830491	1.380033	-2.051031	0.04026388	0.141203419
GRAMD4	-1.905147	0.929032	-2.05068	0.040298093	0.141284894
ZNF684	-2.019168	0.984902	-2.050122	0.040352572	0.141437358
PHLDB2	-2.363567	1.153326	-2.049348	0.040428105	0.141663252
ZKSCAN5	-1.723302	0.840949	-2.049236	0.04043904	0.141663252
ENSG00000241490	-1.3572	0.662381	-2.048972	0.040464849	0.141715083
ENSG00000251364	-1.551604	0.757339	-2.048756	0.040486	0.141750576
ENSG00000233483	-2.894944	1.413393	-2.048223	0.040538134	0.141894498
SAMHD1	-1.672363	0.817357	-2.046061	0.040750344	0.142598498
ERF	-1.418898	0.693563	-2.04581	0.040775068	0.14264622
DPY19L1	-1.90152	0.929663	-2.045386	0.040816855	0.142682797
PQLC2	-1.424026	0.696236	-2.045322	0.040823139	0.142682797
CEP70	-1.879829	0.919129	-2.045228	0.040832339	0.142682797
N6AMT1	-1.490647	0.728862	-2.045169	0.040838154	0.142682797
ENSG00000271133	-2.192493	1.07205	-2.045141	0.040840968	0.142682797
MYLK.AS1	-1.578509	0.771989	-2.04473	0.040881505	0.142785648
ZNF883	-2.601767	1.272534	-2.044557	0.040898598	0.142806585
KCTD21.AS1	-2.194261	1.073459	-2.044103	0.040943409	0.142924266
LILRA4	-2.255513	1.103784	-2.043438	0.041009144	0.143114906
KDM6B	-1.020661	0.499684	-2.042612	0.041090838	0.143361119
GORAB	-1.531171	0.750245	-2.040896	0.041261197	0.14391646
TAFA2	-2.479523	1.215482	-2.03995	0.04135526	0.144205456
TBXA2R	-1.970026	0.966008	-2.039348	0.041415275	0.1443756
INPP5A	-1.545377	0.757855	-2.039146	0.041435494	0.144406962
CDC25B	-1.108935	0.543855	-2.039026	0.041447405	0.144409359
IL10RB.DT	-1.710027	0.83872	-2.038853	0.041464667	0.144425212
ENSG00000261505	-1.708787	0.838152	-2.038756	0.041474404	0.144425212
ZNF862	-3.649967	1.790518	-2.038498	0.041500155	0.144475783
COL19A1	-0.899673	0.441572	-2.037434	0.041606549	0.144806997
EIF5A2	-1.484365	0.728638	-2.037178	0.041632195	0.144850958
ENSG00000102921	-1.257665	0.617385	-2.037083	0.041641695	0.144850958
RTN4IP1	-2.065556	1.014176	-2.036685	0.041681617	0.144950638
NBDY	-0.593545	0.291457	-2.036476	0.041702579	0.144956595
IL15RA	-1.714406	0.841889	-2.03638	0.041712185	0.144956595
ENSG00000261188	-1.825501	0.896518	-2.036212	0.041729054	0.144956595
TNFRSF14.AS1	-1.391604	0.683462	-2.036109	0.041739408	0.144956595
SDR42E2	-1.889491	0.928001	-2.036087	0.041741631	0.144956595
TP53BP2	-1.472436	0.723203	-2.035994	0.041750924	0.144956595
ETS2	-3.714451	1.824735	-2.035611	0.041789408	0.145051071
CHST10	-2.786278	1.368882	-2.03544	0.041806614	0.145071655
CLPTM1L	-1.399292	0.687689	-2.034773	0.041873706	0.145265293
CD79B	0.285921	0.140541	2.03443	0.041908229	0.145345871
BBS1	-1.898713	0.934011	-2.03286	0.042066659	0.145856023
MLLT3	-1.726754	0.84954	-2.032574	0.042095535	0.145916824

MNAT1	-1.307078	0.643183	-2.032201	0.042133297	0.146008386
ENSG00000251661	-2.197128	1.081688	-2.031203	0.042234404	0.146319352
SNTB2	-1.561506	0.769454	-2.02937	0.042420612	0.146924903
KRBA1	-1.666186	0.82109	-2.029237	0.042434135	0.146932188
ARL6	-1.837231	0.905504	-2.028959	0.042462486	0.1469908
ENSG00000259945	-3.85622	1.900798	-2.028738	0.042484997	0.147029172
FANCI	-2.200113	1.084618	-2.028468	0.042512493	0.147084765
NBPF15	-1.474821	0.727109	-2.028335	0.042526094	0.14709227
ENSG00000254469	-1.564822	0.77164	-2.027917	0.042568754	0.147200258
LINC02328	-1.654117	0.815876	-2.027411	0.042620373	0.147339157
ZSCAN12	-1.732521	0.854919	-2.026533	0.042710207	0.147610055
RNF114	-0.744444	0.367382	-2.026345	0.042729407	0.147636757
FGFBP3	-2.07959	1.026454	-2.025993	0.042765458	0.147718747
KIF3C	-1.852878	0.9146	-2.02589	0.042776098	0.147718747
ZNF341.AS1	-3.68685	1.820013	-2.025728	0.04279268	0.147736359
ENSG00000230555	-1.856392	0.916512	-2.025498	0.042816268	0.147778144
LLPH	-0.749023	0.369912	-2.024866	0.042881084	0.147962164
ENSG00000273329	-1.554382	0.767735	-2.024633	0.042905094	0.148005321
IGLV3.21	-4.102846	2.026783	-2.024314	0.04293786	0.148078651
PTPN14	-1.699533	0.839716	-2.023937	0.042976632	0.148159051
MPI	-1.300564	0.642614	-2.023863	0.042984203	0.148159051
JAM3	-1.511435	0.747092	-2.02309	0.043063828	0.148343017
BAIAP2L1	-5.366324	2.65254	-2.023089	0.043063984	0.148343017
ZNF582	-1.66835	0.824687	-2.02301	0.043072162	0.148343017
ZNF841	-1.751382	0.865807	-2.022831	0.043090538	0.14836659
PAXIP1	-1.884532	0.931758	-2.022556	0.043118975	0.148390841
FGF9	-3.817908	1.88768	-2.02254	0.043120646	0.148390841
CAMTA1	1.222003	0.604242	2.022374	0.043137766	0.148410062
ENSG00000271200	-4.005152	1.981495	-2.021278	0.043251018	0.148756916
UFSP1	-3.816779	1.888514	-2.021048	0.043274755	0.148756916
PWWP2B	-1.42638	0.705789	-2.020973	0.043282571	0.148756916
WBP1	-2.057035	1.017891	-2.02088	0.043292166	0.148756916
UBP1	-1.362908	0.674427	-2.020839	0.04329639	0.148756916
SLC29A2	-1.467626	0.726329	-2.020609	0.043320271	0.148799234
ZNF609	-1.375346	0.680707	-2.020466	0.043335043	0.148810247
PDK3	-1.350589	0.668548	-2.020182	0.043364546	0.14886678
CPNE5	1.389557	0.687871	2.020084	0.043374645	0.14886678
PHF21A	-0.832018	0.412062	-2.019156	0.043470991	0.149125893
LPP.AS2	-1.598463	0.791658	-2.019134	0.043473321	0.149125893
FAM111A.DT	-1.104128	0.546901	-2.018881	0.043499595	0.149173772
ARFGAP3	-0.870018	0.430963	-2.018776	0.043510466	0.149173772
GAB3	-1.441134	0.714017	-2.018346	0.043555279	0.149287632
PLEKHB1	-1.937543	0.960549	-2.01712	0.043683005	0.149685547
ETFB	1.201674	0.596287	2.015261	0.043877358	0.15029443
UVSSA	-1.613316	0.800575	-2.015197	0.043884058	0.15029443
PORCN	-1.540481	0.764494	-2.015033	0.043901163	0.150313004
ZNF519	-1.454662	0.721968	-2.014858	0.043919532	0.150335893

ZFAT	-1.584065	0.786306	-2.014566	0.043950173	0.150360693
TNFRSF10D	-1.809024	0.898062	-2.014365	0.043971183	0.150360693
C8orf82	-1.761417	0.874491	-2.01422	0.043986401	0.150360693
TRIM35	-1.724951	0.85642	-2.01414	0.043994799	0.150360693
ETV7	-6.153896	3.055731	-2.013887	0.044021425	0.150360693
BCL11B	-6.153896	3.055731	-2.013887	0.044021425	0.150360693
COL18A1	-6.153896	3.055731	-2.013887	0.044021425	0.150360693
NCK1.DT	-1.443993	0.717054	-2.013787	0.044031949	0.150360693
GNG10	-1.897531	0.94227	-2.013787	0.04403187	0.150360693
ENSG00000260077	-1.643325	0.816089	-2.013658	0.04404549	0.150367025
CDK5RAP3	1.190642	0.591365	2.01338	0.044074669	0.15038836
CACNB1	-3.854067	1.914319	-2.013283	0.044084842	0.15038836
NPAS1	-3.723344	1.849406	-2.013265	0.044086803	0.15038836
RPS28	-0.109436	0.054369	-2.012845	0.044130955	0.150499073
MFGE8	-2.168808	1.077614	-2.012601	0.044156596	0.150546614
ENSG00000272369	-1.607662	0.798881	-2.012391	0.044178727	0.150582166
FAM200A	-1.419766	0.705648	-2.012002	0.044219677	0.150649451
PAFAH2	-1.771072	0.880262	-2.011982	0.044221884	0.150649451
PTRHD1	-0.651776	0.323989	-2.011721	0.044249376	0.150703207
OTUD4	-1.289203	0.6409	-2.01155	0.044267348	0.150724522
LDB1	-1.552406	0.77221	-2.010343	0.044394947	0.151118992
LIMK2	-1.560663	0.776636	-2.009516	0.044482408	0.15137666
CRADD	-1.310224	0.65212	-2.009177	0.044518315	0.151458794
IGF1R	-1.551731	0.772569	-2.008535	0.044586517	0.151650732
ENSG00000218018	-1.532475	0.763052	-2.00835	0.04460612	0.151677312
NAB2	-1.354669	0.674655	-2.007942	0.044649412	0.151784408
HCRT	-1.776586	0.884864	-2.007749	0.044669938	0.151814075
C17orf99	-1.958514	0.975547	-2.007606	0.044685163	0.151825717
SENP3	-1.513493	0.753973	-2.007357	0.044711691	0.151855279
TADA2B	-1.52872	0.761579	-2.007302	0.044717467	0.151855279
ASCC3	-1.122244	0.559509	-2.005767	0.044881152	0.152370917
UBE2Q2	-1.174291	0.585514	-2.005571	0.044902017	0.152401544
C19orf44	-1.871942	0.933568	-2.005148	0.044947229	0.152458304
ENSG00000170846	-1.608018	0.801987	-2.005043	0.044958433	0.152458304
ENSG00000263470	-5.59627	2.791305	-2.004894	0.044974388	0.152458304
ZNF341	-1.615002	0.805589	-2.004746	0.044990258	0.152458304
ENTPD7	-3.794963	1.893475	-2.004231	0.045045287	0.152458304
AGAP4	-1.539236	0.768044	-2.0041	0.045059395	0.152458304
CCR1	-6.122044	3.055329	-2.003727	0.04509936	0.152458304
GIMAP7	-6.122044	3.055329	-2.003727	0.04509936	0.152458304
ANKRD20A4	-6.122044	3.055329	-2.003727	0.045099363	0.152458304
TAL2	-6.122044	3.055329	-2.003727	0.04509936	0.152458304
TNFRSF1A	-6.122044	3.055329	-2.003727	0.04509936	0.152458304
ENSG00000266088	-6.122044	3.055329	-2.003727	0.04509936	0.152458304
RASSF6	-6.11608	3.052772	-2.003451	0.045128902	0.152458304
TMX1	-0.816628	0.407644	-2.003284	0.045146778	0.152458304
EIF4ENIF1	-1.43388	0.715783	-2.003234	0.045152136	0.152458304

LCP2	-6.115013	3.052761	-2.003109	0.045165609	0.152458304
MSC	-6.115013	3.052761	-2.003109	0.045165609	0.152458304
KLRB1	-6.115013	3.052761	-2.003109	0.045165609	0.152458304
MGST1	-6.115013	3.052761	-2.003109	0.045165609	0.152458304
PTGER2	-6.115013	3.052761	-2.003109	0.045165609	0.152458304
IFT43	-1.179451	0.588815	-2.003091	0.045167565	0.152458304
CEP295	-1.287061	0.642695	-2.0026	0.045220235	0.152596056
BCL2	-0.656227	0.327723	-2.002383	0.045243577	0.152603382
ENSG00000261087	-1.618697	0.808395	-2.002359	0.045246126	0.152603382
TXNL4B	-1.369054	0.683926	-2.001757	0.045310831	0.152781567
MOB3B	-1.43722	0.718109	-2.001396	0.045349773	0.152872813
ENSG00000236935	-1.60066	0.800578	-1.99938	0.045567279	0.153514561
IFT46	-1.508714	0.7546	-1.999355	0.04556992	0.153514561
ZEB1.AS1	-1.479001	0.739808	-1.999169	0.04559008	0.153514561
ZNF629	-1.686283	0.843527	-1.999086	0.045599077	0.153514561
C15orf41	-2.000199	1.00056	-1.999079	0.045599802	0.153514561
ZNF628	-1.737818	0.869511	-1.998615	0.045650016	0.153643409
C19orf47	-1.871283	0.936803	-1.997519	0.045768821	0.154002986
FAM227B	-1.74243	0.872917	-1.996101	0.045922878	0.154480961
ENSG00000272760	-1.7349	0.869197	-1.99598	0.045936151	0.154485221
MIR22HG	-1.85745	0.93079	-1.995564	0.045981437	0.154597114
PLA2G15	-1.481749	0.742663	-1.995183	0.046022941	0.154696233
SPAG4	-4.765562	2.389386	-1.994472	0.046100529	0.154916559
KCNC4	-1.540428	0.772399	-1.994342	0.046114735	0.154923837
EIF4EBP3	-1.625742	0.815838	-1.992725	0.046291548	0.155477252
RC3H1	-1.046534	0.525338	-1.992114	0.046358542	0.15566163
FAM71D	-1.633819	0.820475	-1.991309	0.04644698	0.155917895
INPP4A	-1.487993	0.747286	-1.991197	0.046459284	0.155918519
STX3	-1.182409	0.59408	-1.99032	0.04655574	0.1561507
ZNF704	-1.611516	0.809687	-1.990296	0.046558381	0.1561507
ZNF852	-1.602658	0.80526	-1.990237	0.046564874	0.1561507
ZNF865	-1.492263	0.750071	-1.989495	0.046646629	0.156384099
RSRC1	-0.801325	0.402801	-1.989381	0.046659149	0.156385327
FAHD2B	-1.577756	0.793519	-1.988303	0.046778219	0.15674358
SNX30	-1.373285	0.691124	-1.987033	0.046918778	0.157173632
LINC01597	-1.701842	0.856756	-1.986378	0.046991356	0.157375788
COPE	0.762116	0.383719	1.986131	0.0470188	0.157411769
GPRC5C	-1.482936	0.746672	-1.986061	0.047026567	0.157411769
EIF3C	-1.457287	0.733816	-1.985901	0.047044311	0.157430209
KANSL1L	-1.976312	0.995272	-1.985701	0.047066485	0.15746346
ENSG00000262050	-5.007887	2.5223	-1.985444	0.047095046	0.157518053
KCTD19	-2.271087	1.144215	-1.984844	0.047161882	0.157700606
ENSG00000271109	-1.51982	0.765989	-1.984127	0.047241723	0.15792654
ZFP69	-1.79245	0.903549	-1.983788	0.047279426	0.158011525
CD9	-1.3706	0.691023	-1.983435	0.047318879	0.158102316
ZNF549	-1.510405	0.76162	-1.983149	0.047350797	0.158128956
APOBEC3H	-1.659149	0.836632	-1.983131	0.047352853	0.158128956

ELAC1	-1.427691	0.719953	-1.983033	0.047363721	0.158128956
PFAS	-1.622361	0.818177	-1.982898	0.047378786	0.158138222
VHL	1.022232	0.515669	1.982343	0.047440858	0.158284164
POU5F2	-1.478229	0.745719	-1.982287	0.047447114	0.158284164
ENSG00000272189	-1.76989	0.892999	-1.981962	0.047483545	0.158352507
TMCO4	-1.434328	0.723719	-1.981884	0.047492214	0.158352507
DTNB	-1.36044	0.686561	-1.981528	0.047532105	0.158444453
DGKG	-5.462763	2.758099	-1.980626	0.047633231	0.158740425
CD6	-1.537189	0.776159	-1.980508	0.047646498	0.158743525
LANCL2	-2.160305	1.090934	-1.980234	0.047677259	0.158794167
RRAGD	-2.153531	1.087558	-1.980153	0.047686381	0.158794167
ENSG00000271857	-2.181351	1.101744	-1.979907	0.047713931	0.158844798
CCDC74A	-1.860838	0.939978	-1.979662	0.047741548	0.158863888
ICA1	-3.717185	1.8779	-1.979437	0.047766801	0.158863888
STXBP5	-1.613619	0.815198	-1.97942	0.047768752	0.158863888
ERICH6.AS1	-1.497576	0.756597	-1.979357	0.047775794	0.158863888
PRR7	-1.561626	0.788976	-1.979307	0.047781398	0.158863888
AGAP2	-1.505482	0.761017	-1.978249	0.047900567	0.159208013
CHST12	1.347497	0.681184	1.978169	0.047909647	0.159208013
ANKLE1	-1.614922	0.816587	-1.977648	0.04796848	0.159362364
GATAD1	-0.972865	0.492068	-1.977096	0.048030822	0.159515346
TMEM185B	-1.752975	0.886675	-1.97702	0.048039323	0.159515346
ENSG00000223821	-2.168519	1.097018	-1.97674	0.048071015	0.159544813
ARMC5	-1.616575	0.817824	-1.97668	0.048077855	0.159544813
FXR2	-1.376611	0.696449	-1.976613	0.048085396	0.159544813
ENSG00000273389	-3.034104	1.53533	-1.976191	0.048133161	0.159642623
GPRASP1	-4.340134	2.196349	-1.976067	0.048147142	0.159642623
BICD2	-1.187198	0.600802	-1.976024	0.048152096	0.159642623
RNF145	1.326587	0.671429	1.975767	0.048181188	0.159697925
MAP3K6	-2.157217	1.092156	-1.975192	0.048246359	0.159872755
VPS37B	1.308666	0.662731	1.974656	0.048307245	0.160002194
RN7SL832P	-1.915128	0.969913	-1.974536	0.048320821	0.160002194
ZNF823	-1.59917	0.809904	-1.974519	0.048322727	0.160002194
ZFYVE26	-1.484189	0.75174	-1.974338	0.048343288	0.160029092
FAM53C	-0.973102	0.492923	-1.974145	0.048365222	0.160060523
WDR47	-1.615143	0.818566	-1.973138	0.048479874	0.16038976
TAF5L	-1.562669	0.792006	-1.973052	0.048489637	0.16038976
ENSG00000271576	-1.401514	0.710406	-1.972836	0.048514255	0.160412463
ENSG00000269038	-1.81352	0.919327	-1.972659	0.048534407	0.160412463
SLC2A5	1.474595	0.747533	1.972616	0.048539305	0.160412463
CXCL8	-3.793505	1.923143	-1.972554	0.048546369	0.160412463
ENSG00000228434	1.401521	0.710933	1.971382	0.048680226	0.16081347
NIPA1	-1.579033	0.801107	-1.971064	0.048716606	0.160892344
ENSG00000237513	-1.367427	0.693923	-1.970575	0.048772565	0.161035822
HOXB.AS1	-1.895265	0.961881	-1.970374	0.048795524	0.161070293
ABHD4	-1.451709	0.736991	-1.969777	0.048863911	0.161254665
FAM169A	-1.825096	0.926883	-1.96907	0.048945107	0.161481203

PIGBOS1	-0.884351	0.449247	-1.96852	0.04900823	0.161648011
ENSG00000281741	-5.370472	2.728388	-1.968368	0.049025723	0.16166427
RAD51C	-1.040201	0.52852	-1.968141	0.049051775	0.161708733
ENSG00000236514	-1.602306	0.81449	-1.96725	0.049154443	0.162005691
ENSG00000270006	-3.636883	1.849348	-1.966576	0.04923212	0.162184238
RASSF1.AS1	-1.765483	0.897751	-1.966561	0.049233826	0.162184238
ACTR3C	-1.918804	0.975959	-1.966071	0.049290413	0.162289437
OTUD1	-1.498939	0.762405	-1.966066	0.049290986	0.162289437
C7orf31	-1.290706	0.656533	-1.965943	0.049305218	0.162294767
ANO6	-1.517889	0.772305	-1.9654	0.049367964	0.162454679
ENSG00000272807	-1.614009	0.821286	-1.965223	0.0493884	0.162454679
SLX4	-1.562698	0.795187	-1.965195	0.049391677	0.162454679
MAP3K2.DT	-2.27313	1.156892	-1.964859	0.049430558	0.162541014

Cluster 8	log2FC	lfcSE	stat	pvalue	padj
VPREB3	3.10935	0.122778	25.324971	< 2.22e-16	< 2.22e-16
PLAAT4	1.960884	0.210039	9.3358	< 2.22e-16	< 2.22e-16
TRABD	0.640191	0.086704	7.383653	1.54E-13	5.58E-10
DUS2	1.582962	0.226163	6.999212	2.57E-12	7.00E-09
RARA.AS1	2.696763	0.39113	6.894805	5.39E-12	1.17E-08
PNOC	1.732011	0.256972	6.740081	1.58E-11	2.87E-08
MRPS6	0.760093	0.117674	6.459333	1.05E-10	1.63E-07
CD1D	2.700403	0.4383	6.161078	7.23E-10	9.83E-07
SLC12A4	2.625044	0.429696	6.109078	1.00E-09	1.21E-06
AGO1	1.263621	0.220207	5.738339	9.56E-09	1.04E-05
MS4A1	-0.629759	0.111336	-5.656375	1.55E-08	1.53E-05
DPEP2	0.827363	0.152243	5.434481	5.50E-08	4.98E-05
DENND6B	2.202848	0.407365	5.407549	6.39E-08	5.35E-05
CD38	2.706547	0.502084	5.390624	7.02E-08	5.46E-05
AHI1	1.355534	0.253283	5.351851	8.71E-08	6.03E-05
RMDN2	2.072746	0.387529	5.348621	8.86E-08	6.03E-05
SLC5A3	1.048462	0.206849	5.068733	4.00E-07	0.000256279
HOXB3	3.759693	0.747926	5.026824	4.99E-07	0.000301391
SPRY1	1.05233	0.21004	5.010133	5.44E-07	0.000311439
GBP4	0.778645	0.156072	4.989007	6.07E-07	0.000330126
SEPTIN9	0.46807	0.095327	4.910132	9.10E-07	0.000471501
H3F3A	0.411428	0.085606	4.806085	1.54E-06	0.000761109
ICAM2	0.879563	0.191799	4.585852	4.52E-06	0.002138617
P2RX5	0.903187	0.200318	4.50877	6.52E-06	0.002955674
GRN	1.048161	0.233912	4.480996	7.43E-06	0.003233049
RRBP1	1.404271	0.316165	4.44158	8.93E-06	0.003736555
CD72	0.791993	0.183131	4.324735	1.53E-05	0.006050121
TGFBR2	-0.693224	0.160452	-4.320443	1.56E-05	0.006050121
DTX4	1.335757	0.314991	4.240617	2.23E-05	0.008362059
GPR183	-0.599259	0.141671	-4.229923	2.34E-05	0.008477309
SIGIRR	0.698336	0.166491	4.194441	2.74E-05	0.009599677
CHST15	0.861948	0.2061	4.182187	2.89E-05	0.009815529
TNFRSF13B	-1.427673	0.343628	-4.154704	3.26E-05	0.010737547
FCRL1	1.088117	0.264866	4.108172	3.99E-05	0.012760532
ENSG00000271204	0.499282	0.122284	4.082963	4.45E-05	0.013821053
RABGAP1L	0.680632	0.168579	4.03747	5.40E-05	0.01632777
MAP3K1	0.726835	0.181541	4.003695	6.24E-05	0.018335742
DBNL	0.605379	0.151703	3.990543	6.59E-05	0.018872795
CD83	-0.463881	0.116666	-3.976155	7.00E-05	0.019537152
GBP7	1.592185	0.406454	3.917256	8.96E-05	0.024358845
PLAAT3	2.993368	0.767086	3.902259	9.53E-05	0.02528678
CIB1	0.421383	0.108339	3.889487	0.00010046	0.026020629
GDPGP1	1.710475	0.444553	3.847626	0.00011927	0.030174838
CD22	0.77424	0.205237	3.772418	0.00016167	0.039973674
S100A4	-0.994181	0.264569	-3.757736	0.00017146	0.041450824
QPRT	3.380332	0.901268	3.75064	0.00017638	0.041714691

LINC.PINT	0.492244	0.131834	3.733805	0.00018861	0.043656929
MPEG1	0.976972	0.262485	3.722011	0.00019764	0.043976031
LCAT	2.008887	0.539811	3.721463	0.00019807	0.043976031
ZNF563	1.12665	0.307355	3.665627	0.00024673	0.05368417
RAB37	1.220522	0.337376	3.617688	0.00029725	0.063406763
MAP2K6	2.431676	0.675407	3.600314	0.00031783	0.066494345
NCF1	0.319515	0.090509	3.530219	0.00041522	0.085228775
ZFP36L1	-0.418767	0.119695	-3.498614	0.00046768	0.094220657
FTH1	-0.159992	0.045805	-3.492877	0.00047785	0.094517925
CD79A	0.418568	0.120266	3.480346	0.00050077	0.095991472
SMAD3	0.783538	0.225207	3.479184	0.00050294	0.095991472
IGLC2	-1.1799	0.341078	-3.459327	0.00054153	0.099765738
FAM3C	0.625017	0.180692	3.459009	0.00054217	0.099765738
NUP210	0.642716	0.186023	3.455031	0.00055023	0.099765738
LBR	0.490443	0.142812	3.43418	0.00059435	0.105165554
FCER2	1.134201	0.330487	3.431911	0.00059934	0.105165554
DPEP3	2.093396	0.61505	3.403619	0.000665	0.114833074
GPX1	0.403301	0.119057	3.387456	0.00070544	0.119913892
NDUFB9	0.518556	0.15415	3.363963	0.00076832	0.128593079
SERPINB6	0.777936	0.234246	3.321025	0.00089688	0.147835052
CD82	-0.527695	0.16056	-3.286587	0.00101409	0.164661646
DDIT4	1.010938	0.309254	3.26896	0.00107943	0.172693583
CD180	0.692766	0.217279	3.18837	0.00143078	0.22558555
NMT2	-0.571509	0.179883	-3.177122	0.00148745	0.231170454
ENSG00000278107	2.800645	0.889177	3.149704	0.00163436	0.247778386
ANAPC16	0.319636	0.101513	3.148722	0.00163986	0.247778386
CYTIP	-0.226722	0.072412	-3.131012	0.00174205	0.25961361
SESN3	-0.506037	0.162255	-3.118768	0.00181609	0.266990049
CCM2	-0.49246	0.158843	-3.100293	0.00193329	0.280430411
PUS1	0.89744	0.29066	3.087594	0.00201784	0.288842855
KCNN1	2.582871	0.844058	3.060065	0.00221289	NA
FAM117A	0.579541	0.189736	3.054455	0.0022547	0.318556972
ENSG00000233038	4.005717	1.312386	3.052241	0.0022714	NA
AGPAT5	0.736702	0.243219	3.028968	0.00245391	0.342257175
USF3	-0.513871	0.170689	-3.010564	0.00260763	0.359094172
SERPINF1	-1.352308	0.453921	-2.979174	0.00289027	0.393040413
ENSG00000278831	2.483788	0.838391	2.962567	0.00305086	NA
CPNE5	0.809985	0.274727	2.948333	0.00319493	0.429106562
PLP2	-0.531445	0.180584	-2.942924	0.00325128	0.431349972
ENSG00000233912	2.280094	0.778046	2.930539	0.00338375	0.432290104
ESYT1	-0.724439	0.247604	-2.925794	0.00343579	0.432290104
ENSG00000261448	1.725677	0.589825	2.925746	0.00343631	0.432290104
RESF1	-0.437648	0.149757	-2.922388	0.00347359	0.432290104
P2RX5.TAX1BP3	1.067236	0.365727	2.918119	0.0035215	0.432290104
CLIC3	1.001622	0.343256	2.918006	0.00352278	0.432290104
CPSF4	-0.817719	0.280349	-2.916791	0.00353652	0.432290104
CGGBP1	-0.451469	0.15537	-2.905766	0.00366356	0.439786861

IFI30	0.630148	0.217205	2.901171	0.00371771	0.439786861
USP30.AS1	0.809216	0.278939	2.901051	0.00371913	0.439786861
FBXO36	2.773995	0.957396	2.897438	0.00376225	0.440101788
IRF4	0.714302	0.250773	2.848407	0.00439387	0.508520526
BTBD7	0.614928	0.216149	2.844923	0.00444222	0.508704433
PAXX	0.500771	0.176652	2.834789	0.0045856	0.515158921
GSTP1	0.255967	0.090312	2.834253	0.00459329	0.515158921
NAA80	-1.237574	0.437388	-2.829467	0.00466256	0.517591685
RAB20	1.33003	0.474126	2.805225	0.00502815	0.552537214
ADA	-0.598268	0.214155	-2.793623	0.00521212	0.567026895
UBE2E2	-0.715778	0.256598	-2.789493	0.00527906	0.568622844
TUBD1	0.815115	0.29355	2.776747	0.00549059	0.585608564
SLC43A2	0.334982	0.121054	2.76722	0.00565366	0.597147458
RNF207	2.338817	0.845431	2.766422	0.00566752	NA
IRAK2	0.897562	0.325104	2.760846	0.00576518	0.600758575
FCMR	0.398427	0.144712	2.753246	0.00590075	0.600758575
SLC45A3	1.25908	0.457539	2.751851	0.00592595	0.600758575
LY86	-0.399226	0.145182	-2.749829	0.00596264	0.600758575
IDS	-0.322417	0.117253	-2.749756	0.00596396	0.600758575
EFHD2	-1.16934	0.427635	-2.734436	0.00624873	0.623669428
PARP14	-0.390875	0.143254	-2.728535	0.00636164	0.626643802
CLIC1	-0.225944	0.082858	-2.726874	0.00639374	0.626643802
AGPAT2	-0.5096	0.187333	-2.720291	0.00652246	0.629562954
ENSG00000089127	0.750298	0.275902	2.71944	0.00653926	0.629562954
ZC3H12D	-0.985839	0.363633	-2.711079	0.00670647	0.639997014
GABPB1	0.328487	0.121359	2.706729	0.00679496	0.642803565
RHOG	-0.346188	0.128302	-2.698234	0.00697083	0.653755989
TRMT1	0.501241	0.187081	2.679276	0.00737815	0.684338709
PSAT1	2.688362	1.004097	2.677393	0.00741976	NA
AP3B1	0.487233	0.18199	2.677258	0.00742274	0.684338709
TRANK1	-0.558344	0.208927	-2.672435	0.00753029	0.684772925
PTP4A3	1.927055	0.722055	2.668849	0.00761116	0.684772925
ZSWIM6	1.052991	0.395088	2.665203	0.00769419	0.684772925
LLGL2	-1.682758	0.63179	-2.663475	0.00773382	0.684772925
TBC1D9	0.737224	0.276828	2.663112	0.00774217	0.684772925
PTMA	0.120179	0.045207	2.658429	0.00785059	0.688762361
ENSG00000245904	0.267071	0.101055	2.642841	0.00822135	0.715520638
SYNGR3	1.515633	0.576124	2.63074	0.00851992	0.73297557
OAS3	1.317763	0.501188	2.629277	0.00855666	0.73297557
ENDOD1	0.953719	0.364818	2.614235	0.00894275	0.760064182
GPR160	1.010026	0.387964	2.603403	0.00923033	0.778424071
ARHGAP22	1.65275	0.636945	2.594807	0.00946439	0.792024029
ZNF674	2.009019	0.776988	2.585649	0.00971958	0.807170646
CD24	-0.437137	0.169258	-2.58267	0.00980391	0.808005309
ZNF486	1.203446	0.46674	2.578409	0.00992565	0.811888422
KCNC3	1.139557	0.442493	2.575312	0.01001497	0.813081212
MYCBP	-1.134055	0.442479	-2.562959	0.01037842	0.829405859

MID1IP1	-0.39561	0.154373	-2.562683	0.01038669	0.829405859
SLC2A5	1.004176	0.392275	2.559879	0.01047087	0.829405859
GUK1	-0.297871	0.116437	-2.558218	0.01052101	0.829405859
POLN	1.931495	0.756563	2.552986	0.01068037	0.831057765
CTSH	-0.356644	0.139723	-2.552518	0.01069474	0.831057765
TBK1	-0.748704	0.294073	-2.545981	0.01089713	0.836684838
ENSG00000279278	0.795319	0.312585	2.544328	0.01094881	0.836684838
RAB5IF	-0.388409	0.152834	-2.541371	0.01104185	0.836684838
RRS1	0.850014	0.334869	2.538348	0.01113772	0.836684838
FAM167A	1.287398	0.507267	2.537909	0.0111517	0.836684838
SCN4A	2.31149	0.912567	2.532953	0.01131062	0.841003302
WDR33	0.483167	0.191007	2.529577	0.01142001	0.841003302
WNT10A	-1.220609	0.482685	-2.528792	0.01144557	0.841003302
NUBP2	-0.449575	0.177939	-2.526563	0.01151848	0.841003302
BIN2	0.653349	0.259616	2.516594	0.01184953	0.859406838
SSNA1	-0.414867	0.165024	-2.513976	0.01193786	0.860079589
DIAPH1	-0.68873	0.274506	-2.508982	0.01210798	0.866596497
INTS4	-0.761124	0.305559	-2.490919	0.0127413	0.901025223
LAPTM5	-0.205583	0.082545	-2.490547	0.01275465	0.901025223
MLLT6	-0.711983	0.287205	-2.479007	0.01317486	0.919472824
SNRK	0.657204	0.265137	2.478738	0.01318483	0.919472824
MLXIP	0.520524	0.21039	2.474092	0.01335752	0.925582228
HS6ST1	1.244885	0.503678	2.471591	0.01345133	0.926183774
FSD1L	-1.124781	0.457285	-2.459695	0.01390553	0.94927298
OGFRL1	-0.796514	0.324294	-2.456147	0.01404356	0.94927298
ETFB	0.403065	0.164113	2.456022	0.01404844	0.94927298
PKN3	2.746113	1.11939	2.453224	0.01415823	0.950786535
PPP1R18	0.390186	0.159198	2.450946	0.01424812	0.950952733
GABARAPL2	0.296021	0.121019	2.446082	0.01444182	0.958003176
ENSG00000261335	1.879734	0.769632	2.442381	0.01459076	0.962017216
PARD6A	-0.676668	0.278192	-2.43238	0.01499995	0.983038575
DENND6A	0.884936	0.364209	2.429746	0.01510942	0.984283834
ENSG00000231856	-1.657265	0.683915	-2.423204	0.01538427	0.996223211
IKZF2	-1.539224	0.636319	-2.418948	0.01556545	0.999772489
CD79B	0.208247	0.08636	2.411371	0.01589268	0.999772489
P2RY8	0.45907	0.190718	2.407069	0.01608115	0.999772489
DMD	-0.522894	0.217267	-2.406686	0.01609802	0.999772489
ELMSAN1	-0.445787	0.185243	-2.406497	0.01610634	0.999772489
C2orf92	2.245006	0.934052	2.403513	0.01623837	0.999772489
MRNIP	0.479002	0.199378	2.402485	0.0162841	0.999772489
ENSG00000272049	2.118701	0.882078	2.401942	0.0163083	NA
FOXK1	-0.592735	0.246972	-2.400011	0.01639458	0.999772489
SLC50A1	-0.316645	0.132054	-2.397845	0.01649185	0.999772489
DIABLO	-1.213042	0.505963	-2.397493	0.0165077	0.999772489
CD27	-1.699363	0.709067	-2.396618	0.01654715	0.999772489
FOXO3	0.826877	0.345216	2.395249	0.01660906	0.999772489
GGA2	0.294302	0.123066	2.391426	0.01678308	0.999772489

RC3H1	-0.680542	0.284782	-2.389696	0.01686233	0.999772489
RUBCN	-0.500626	0.209733	-2.386973	0.01698775	0.999772489
EP400	0.567695	0.237968	2.385592	0.01705168	0.999772489
REC8	0.831981	0.349318	2.381731	0.01723148	0.999772489
SVOP	2.166892	0.910081	2.380988	0.01726627	0.999772489
JDP2	1.089947	0.457837	2.380644	0.01728241	0.999772489
ENSG00000230555	0.993364	0.417708	2.378128	0.0174008	0.999772489
TNFRSF18	-1.374085	0.580252	-2.368085	0.01788041	0.999772489
NOD2	3.335023	1.410173	2.364974	0.01803136	NA
AFTPH	-0.493734	0.209094	-2.361305	0.01821075	0.999772489
TRAF4	-0.682176	0.289095	-2.359691	0.01829014	0.999772489
SAXO2	1.838486	0.780008	2.357009	0.01842281	NA
ZNF3	0.659187	0.279883	2.355223	0.01851159	0.999772489
STK19	-0.950465	0.403595	-2.354999	0.01852277	0.999772489
NFE2L2	-0.379269	0.161066	-2.354743	0.01853551	0.999772489
TUSC1	2.005848	0.852638	2.352521	0.01864663	0.999772489
PCBP1	-0.321614	0.136755	-2.351755	0.01868509	0.999772489
PLXNB2	1.779051	0.757811	2.347619	0.01889384	0.999772489
NEURL1	2.397878	1.022081	2.346074	0.01897233	0.999772489
TRPV3	1.736198	0.740128	2.345807	0.01898593	0.999772489
EPC1	0.269933	0.115214	2.342892	0.01913492	0.999772489
PAOX	0.69918	0.298628	2.341308	0.01921629	0.999772489
TTLL7	1.840647	0.786695	2.339721	0.01929816	0.999772489
RASGEF1B	-0.501229	0.214232	-2.339656	0.01930153	0.999772489
PDCD4.AS1	0.710941	0.304179	2.337245	0.01942646	0.999772489
MMP11	0.961662	0.411654	2.336091	0.01948651	0.999772489
RERE	-0.414363	0.177569	-2.333535	0.01962006	0.999772489
DNM2	-0.494441	0.211899	-2.333378	0.01962831	0.999772489
CD47	-0.229297	0.098269	-2.333373	0.01962859	0.999772489
PKIG	-0.424758	0.182161	-2.331769	0.01971284	0.999772489
PHF20L1	-0.391715	0.168115	-2.330047	0.01980366	0.999772489
MEF2A	0.401038	0.172263	2.328052	0.01990934	0.999772489
SLC7A11	2.120331	0.911629	2.32587	0.02002551	NA
FZD3	1.398688	0.60208	2.323093	0.02017414	0.999772489
HIRA	-0.912064	0.392633	-2.322944	0.02018219	0.999772489
ZNF233	1.914508	0.827006	2.314986	0.0206137	NA
ZNF726	1.183755	0.512399	2.310223	0.0208758	0.999772489
ABCC10	-1.218913	0.528401	-2.306797	0.02106615	0.999772489
TIGAR	-1.238275	0.536891	-2.30638	0.0210894	0.999772489
SNX18	1.05339	0.456922	2.305403	0.02114401	0.999772489
ANKRD44	0.352077	0.152957	2.301809	0.02134592	0.999772489
MAST4	-1.066939	0.463633	-2.301257	0.0213771	0.999772489
MIB2	-0.581068	0.252531	-2.300975	0.02139302	0.999772489
CDK16	-0.92236	0.401422	-2.297728	0.02157726	0.999772489
BEST1	-0.811995	0.353443	-2.297388	0.02159667	0.999772489
CBWD2	-0.953512	0.415359	-2.295633	0.02169689	0.999772489
LSR	-1.078956	0.470269	-2.294336	0.02177121	0.999772489

ECHS1	0.443461	0.193354	2.293515	0.02181835	0.999772489
MDM4	-0.267327	0.116655	-2.291598	0.02192885	0.999772489
VPS36	-0.39205	0.171256	-2.289261	0.02206418	0.999772489
KCNH4	2.741203	1.197569	2.288973	0.02208092	NA
CKLF	-0.476098	0.208067	-2.288191	0.02212638	0.999772489
MED14	-1.017595	0.444728	-2.28813	0.02212996	0.999772489
RPS6KA4	-0.916506	0.400636	-2.287631	0.022159	0.999772489
PSD3	-0.932144	0.408017	-2.284569	0.02233811	0.999772489
MGAT2	-0.746387	0.32718	-2.281271	0.0225324	0.999772489
CYLD	-0.366653	0.160726	-2.281236	0.02253451	0.999772489
FAM76A	-0.517618	0.226905	-2.281213	0.02253585	0.999772489
PCBP4	-1.31952	0.578978	-2.279051	0.02266405	0.999772489
LINC01679	2.89468	1.271439	2.276696	0.02280441	NA
ENTPD3.AS1	0.612566	0.269889	2.269697	0.02322597	0.999772489
SLC25A35	1.14633	0.505288	2.268665	0.02328868	0.999772489
TSPYL2	0.540492	0.238311	2.268008	0.02332869	0.999772489
ENSG00000258539	1.717914	0.758193	2.265801	0.02346356	0.999772489
TKT	-0.323238	0.142727	-2.264734	0.023529	0.999772489
CYBA	0.223631	0.098769	2.264179	0.02356309	0.999772489
SNX6	-0.363868	0.16078	-2.263139	0.02362711	0.999772489
COQ2	-1.170788	0.517384	-2.262898	0.02364196	0.999772489
KDM6B	-0.540082	0.238695	-2.262645	0.02365758	0.999772489
PRDM1	2.268816	1.003283	2.261391	0.02373505	NA
POU2AF1	0.251709	0.111331	2.260906	0.02376509	0.999772489
GTF2A2	-0.325222	0.144082	-2.257203	0.02399539	0.999772489
NAMPT	0.56024	0.24834	2.255941	0.0240743	0.999772489
STAG2	0.342411	0.151792	2.255799	0.02408319	0.999772489
XYLT1	0.692998	0.307268	2.255353	0.02411117	0.999772489
CBX8	1.255189	0.55657	2.25522	0.02411954	0.999772489
TRIM38	0.29782	0.132153	2.253605	0.02422105	0.999772489
ZNF318	-0.473819	0.210439	-2.251579	0.02434888	0.999772489
TOMM40L	1.763705	0.783367	2.251442	0.02435758	0.999772489
PHC2	-1.70886	0.759256	-2.250703	0.02440436	0.999772489
LIG3	1.067195	0.474448	2.249339	0.02449091	0.999772489
ABR	0.693403	0.308377	2.248555	0.02454083	0.999772489
HSH2D	0.365652	0.162756	2.246632	0.02466354	0.999772489
WASF2	0.301539	0.134519	2.241618	0.02498607	0.999772489
DENND5A	0.798262	0.356221	2.240916	0.0250315	0.999772489
EHD4	-0.649059	0.290012	-2.238042	0.02521835	0.999772489
PATJ	-0.979504	0.437719	-2.237745	0.02523766	0.999772489
ZDHHC7	-1.065171	0.476318	-2.23626	0.02533477	0.999772489
ENSG00000231769	1.842524	0.824591	2.23447	0.02545216	NA
BCCIP	0.502405	0.224975	2.233158	0.02553855	0.999772489
FUT2	2.182664	0.977646	2.232572	0.02557722	NA
ENSG00000226380	0.678649	0.304294	2.230241	0.02573148	0.999772489
METAP1D	0.901626	0.404742	2.22766	0.0259032	0.999772489
ATP6V1A	-0.583422	0.262843	-2.21966	0.02644185	0.999772489

CD44	-0.34962	0.157572	-2.218793	0.0265008	0.999772489
NUDT8	-1.359355	0.612924	-2.217818	0.02656724	0.999772489
CETP	-1.554217	0.701749	-2.214776	0.02677542	0.999772489
TMCC1.AS1	1.873398	0.84677	2.212405	0.02693871	NA
ABCA2	1.797396	0.815231	2.204769	0.02747029	0.999772489
TOP1MT	-0.67898	0.307997	-2.204503	0.02748898	0.999772489
PXDC1	2.397443	1.087534	2.204476	0.0274909	0.999772489
LINC00896	2.041739	0.927007	2.202507	0.02762951	0.999772489
ENSG00000228201	2.093368	0.950971	2.201296	0.02771509	NA
NFATC3	0.445012	0.202266	2.20013	0.0277977	0.999772489
ENSG00000254859	2.831145	1.288596	2.197077	0.02801495	NA
SYTL1	-0.362192	0.165045	-2.194502	0.02819936	0.999772489
TAPT1.AS1	1.584252	0.722407	2.19302	0.02830594	NA
POU2F2	-0.259536	0.118402	-2.191985	0.02838062	0.999772489
ATP11C	0.664804	0.30334	2.191617	0.02840716	0.999772489
C1orf131	-0.439392	0.200648	-2.18986	0.02853443	0.999772489
ARHGAP15	0.290181	0.132558	2.189093	0.02859009	0.999772489
SYNE3	-1.027197	0.469329	-2.188649	0.02862237	0.999772489
TXLNG	0.607086	0.27767	2.186355	0.02878967	0.999772489
MRPS36	0.348482	0.159414	2.186018	0.02881431	0.999772489
NCF4	-0.414935	0.189915	-2.184841	0.02890052	0.999772489
CTSA	0.558363	0.255655	2.184045	0.02895891	0.999772489
PLEKHO1	0.399209	0.182808	2.183762	0.02897971	0.999772489
VPS18	-0.961088	0.440283	-2.182886	0.0290442	0.999772489
SELENOM	-0.815375	0.373937	-2.180516	0.02921922	0.999772489
TMEM242	-0.59041	0.270819	-2.180089	0.02925085	0.999772489
SIDT1	-0.487257	0.223582	-2.179323	0.02930769	0.999772489
TRIP10	-0.84873	0.389471	-2.179191	0.02931752	0.999772489
LINC002481	2.479693	1.138112	2.178777	0.02934822	0.999772489
FUOM	0.613845	0.281863	2.177812	0.02942007	0.999772489
MZB1	-0.604746	0.277925	-2.175929	0.02956057	0.999772489
ENSG00000271870	-1.492259	0.686243	-2.174535	0.029665	0.999772489
CPT2	0.674598	0.310325	2.17384	0.02971716	0.999772489
LRRFIP1	-0.309855	0.142601	-2.172877	0.02978956	0.999772489
UGCG	0.643709	0.296269	2.172714	0.02980185	0.999772489
CXCR4	0.127097	0.058624	2.167994	0.03015914	0.999772489
RINL	0.465686	0.214812	2.167876	0.03016811	0.999772489
PSMA3	0.300135	0.138453	2.167779	0.0301755	0.999772489
ARPC1B	-0.192366	0.088795	-2.166394	0.03028106	0.999772489
SUSD1	1.83975	0.849495	2.165697	0.03033438	NA
NICN1	0.801354	0.371225	2.158673	0.03087556	0.999772489
XBP1	0.533119	0.246994	2.158426	0.03089471	0.999772489
LARGE1	0.973519	0.452254	2.152593	0.03135064	0.999772489
PRR5	1.900181	0.882932	2.152124	0.03138755	0.999772489
VIM	-0.577563	0.268378	-2.152053	0.03139317	0.999772489
ENSG00000273384	1.459387	0.678447	2.151071	0.03147059	0.999772489
IRF1	0.257688	0.12	2.147398	0.03176161	0.999772489

AFG3L2	-0.716799	0.33423	-2.144629	0.03198253	0.999772489
UBE2G1	0.352671	0.1646	2.142594	0.03214569	0.999772489
HTR3A	2.143782	1.001448	2.140682	0.03229969	0.999772489
SLC45A4	3.213488	1.501714	2.139879	0.03236452	NA
ERGIC3	-0.29267	0.136854	-2.138555	0.03247176	0.999772489
HMOX1	0.872271	0.408037	2.137725	0.0325391	0.999772489
PRPF4B	-0.393567	0.184165	-2.13704	0.03259474	0.999772489
STX10	-0.319896	0.149704	-2.136862	0.0326092	0.999772489
NMNAT3	1.468994	0.687794	2.135806	0.03269524	0.999772489
CNBP	0.184998	0.086665	2.134643	0.03279022	0.999772489
UFSP1	1.805155	0.845871	2.134077	0.03283644	NA
PALLD	2.829896	1.326777	2.13291	0.03293209	NA
TMEM38B	0.603697	0.283714	2.127834	0.03335082	0.999772489
WDR19	-0.943778	0.443732	-2.126911	0.03342745	0.999772489
LINC01136	1.866732	0.879079	2.123508	0.03371128	0.999772489
GPT2	0.84609	0.398902	2.12105	0.03391759	0.999772489
LDHA	-0.424298	0.200129	-2.12012	0.03399596	0.999772489
TLR10	-0.534576	0.252659	-2.115799	0.03436194	0.999772489
TAF4B	0.966329	0.457065	2.114204	0.03449784	0.999772489
PDCD4	0.42361	0.200367	2.114166	0.03450112	0.999772489
JAK1	0.241805	0.114471	2.112374	0.03465441	0.999772489
ARMC2	1.617436	0.766058	2.111374	0.03474018	NA
FADS3	-0.340359	0.161353	-2.10941	0.03490924	0.999772489
TMEM120B	-0.874515	0.414874	-2.107906	0.03503913	0.999772489
RBMS1	-0.64534	0.306229	-2.10738	0.03508463	0.999772489
ENTPD5	1.154427	0.547988	2.106665	0.0351466	0.999772489
ENSG00000259793	1.894036	0.899835	2.104871	0.0353025	NA
KANSL2	-0.650399	0.309408	-2.102078	0.03554642	0.999772489
FYTTD1	-0.400895	0.190716	-2.102056	0.03554834	0.999772489
SEMA7A	-1.025062	0.48765	-2.102047	0.03554915	0.999772489
LINC02158	1.835459	0.873922	2.100254	0.0357065	NA
BMF	-0.746332	0.355377	-2.100115	0.0357187	0.999772489
ENSG00000253535	0.959535	0.457138	2.099003	0.03581667	0.999772489
NBN	-0.506698	0.241478	-2.098322	0.03587668	0.999772489
SLC41A2	1.306207	0.622604	2.097973	0.03590749	0.999772489
SLC33A1	-0.742952	0.354308	-2.096908	0.03600168	0.999772489
IQCC	1.701558	0.812661	2.09381	0.03627688	0.999772489
ZNF706	0.220724	0.105423	2.093697	0.03628697	0.999772489
TRADD	-0.547967	0.261828	-2.09285	0.03636255	0.999772489
BRMS1	-0.387444	0.185216	-2.091846	0.03645227	0.999772489
ENSG00000272817	2.03992	0.975483	2.09119	0.03651102	0.999772489
ENSG00000236723	1.775336	0.849459	2.089962	0.03662123	NA
IRAK1BP1	1.2773	0.61128	2.08955	0.03665821	0.999772489
CCDC144A	1.454103	0.696395	2.088045	0.03679375	0.999772489
MAPKAPK5	-0.539244	0.258465	-2.086333	0.0369485	0.999772489
CLCN5	1.711274	0.820875	2.084694	0.03709709	NA
CD99	-0.447503	0.214857	-2.082794	0.03727002	0.999772489

ZNF800	-0.354683	0.170428	-2.081125	0.03742242	0.999772489
SLC25A45	0.763219	0.366791	2.080802	0.03745199	0.999772489
MED28	-0.25752	0.123774	-2.080569	0.03747341	0.999772489
CIRBP	0.168797	0.081195	2.078898	0.03762673	0.999772489
QSOX2	-0.690383	0.332313	-2.077506	0.03775491	0.999772489
CDCA4	-0.692	0.333315	-2.076116	0.03788325	0.999772489
SEL1L3	0.685004	0.329967	2.075979	0.0378959	0.999772489
C8orf58	-1.084731	0.522906	-2.074429	0.03803946	0.999772489
GIGYF1	-0.414052	0.19962	-2.074199	0.03806084	0.999772489
ENSG00000268439	1.134628	0.54839	2.069018	0.03854444	0.999772489
SH3BGRL3	-0.161745	0.078274	-2.06639	0.03879167	0.999772489
ENSG00000263272	-1.049571	0.508066	-2.065816	0.03884587	0.999772489
KBTBD6	-0.633443	0.306689	-2.065424	0.03888294	0.999772489
TRIO	-0.713391	0.345863	-2.06264	0.03914681	0.999772489
GLIPR2	0.503158	0.244337	2.059284	0.03946705	0.999772489
PLEKHG1	-0.394806	0.191976	-2.05654	0.03973051	0.999772489
OSTM1	0.477863	0.23252	2.055148	0.03986475	0.999772489
ODR4	0.687911	0.334726	2.055146	0.03986495	0.999772489
APMAP	0.589389	0.286803	2.05503	0.03987611	0.999772489
FAM227A	1.624411	0.790491	2.054939	0.03988493	NA
ECH1	0.260191	0.126667	2.054133	0.03996281	0.999772489
FAM177A1	-0.397143	0.193356	-2.053949	0.03998063	0.999772489
SMAGP	0.44269	0.215564	2.053634	0.04001111	0.999772489
DAD1	0.231633	0.112797	2.053529	0.04002127	0.999772489
SH3BP1	-0.827014	0.403509	-2.049556	0.04040781	0.999772489
LINC01521	1.437427	0.70136	2.049485	0.04041467	0.999772489
ENSG00000272379	1.513524	0.738728	2.048823	0.04047941	0.999772489
ASNSD1	0.389146	0.190072	2.047357	0.04062307	0.999772489
RBBP7	-0.413458	0.202048	-2.046331	0.04072384	0.999772489
TMEM97	-1.057121	0.516689	-2.045954	0.04076091	0.999772489
S100A6	-0.412724	0.202053	-2.042655	0.04108662	0.999772489
DDX49	0.526087	0.257896	2.039914	0.04135886	0.999772489
CHP1	-0.386487	0.189622	-2.038197	0.04153022	0.999772489
CHD1	0.306114	0.150191	2.038174	0.04153256	0.999772489
MAN2C1	1.0556	0.518221	2.036969	0.0416531	0.999772489
SNRPC	0.268387	0.131824	2.035954	0.04175496	0.999772489
ADAMDEC1	1.949154	0.957938	2.03474	0.04187702	NA
DNAJC14	0.6962	0.342795	2.030954	0.0422597	0.999772489
GNAI2	-0.334521	0.164735	-2.030655	0.04228998	0.999772489
C15orf39	0.684695	0.337259	2.030177	0.04233855	0.999772489
UBR1	-0.643617	0.317052	-2.030004	0.04235614	0.999772489
USP33	-0.459072	0.226178	-2.029697	0.04238731	0.999772489
CD6	-1.417473	0.69872	-2.028671	0.04249183	0.999772489
DOCK10	0.775624	0.382434	2.028123	0.04254768	0.999772489
SNAPC3	0.484227	0.239147	2.024813	0.04288654	0.999772489
GYPC	0.257542	0.12723	2.024222	0.0429473	0.999772489
FNDC3B	1.691811	0.835812	2.024153	0.04295438	0.999772489

CLEC17A	0.642398	0.317391	2.023999	0.04297029	0.999772489
CARF	0.427179	0.211128	2.023318	0.04304042	0.999772489
FUS	0.164701	0.081489	2.021156	0.04326359	0.999772489
TMEM191C	-0.877435	0.434286	-2.020408	0.04334112	0.999772489
PPP4R3B	-0.423074	0.209504	-2.019411	0.04344449	0.999772489
ENSG00000259888	1.020969	0.505797	2.018535	0.04353561	0.999772489
LRP10	0.262341	0.129973	2.018421	0.04354748	0.999772489
BAG4	-0.727055	0.360299	-2.017921	0.04359947	0.999772489
PPIL2	0.433998	0.215113	2.017532	0.04364	0.999772489
DPH3	-0.608129	0.301628	-2.016158	0.04378342	0.999772489
AUH	0.798718	0.396273	2.015574	0.04384454	0.999772489
ENSG00000277654	-0.563442	0.279661	-2.014733	0.04393267	0.999772489
ENSG00000272426	1.032424	0.512627	2.013988	0.04401079	0.999772489
ASAH1	0.616475	0.306112	2.013884	0.0440217	0.999772489
FSTL3	1.675215	0.831862	2.013814	0.04402908	NA
DDHD2	-0.719259	0.35726	-2.013264	0.04408691	0.999772489
TMEM159	-0.658787	0.327586	-2.011037	0.04432155	0.999772489
NAV1	1.719273	0.855077	2.010664	0.044361	NA
TMEM183A	-0.342784	0.170806	-2.006857	0.04476493	0.999772489
CRX	1.490059	0.74305	2.005327	0.04492806	0.999772489
ATP1A1	-0.491997	0.24552	-2.003895	0.04508128	0.999772489
RNF168	-0.505578	0.252546	-2.00192	0.04529329	0.999772489
CNIH3	1.994585	0.996507	2.001577	0.04533027	NA
ST3GAL1	0.636345	0.317936	2.001488	0.04533984	0.999772489
MTPN	0.369384	0.184562	2.001402	0.04534906	0.999772489
EXPH5	1.591366	0.795237	2.001122	0.04537926	NA
HHEX	-0.250948	0.125405	-2.001104	0.04538122	0.999772489
MAMSTR	1.195692	0.597543	2.001014	0.04539093	0.999772489
NT5DC4	1.488717	0.744033	2.000877	0.04540567	0.999772489
ELAC1	0.958154	0.47894	2.000573	0.04543838	0.999772489
GPR18	0.908561	0.454397	1.999486	0.04555579	0.999772489
FUT7	1.691499	0.847363	1.996191	0.04591311	0.999772489
IQSEC1	-0.366413	0.183572	-1.996017	0.04593206	0.999772489
CTSB	0.426884	0.214029	1.994515	0.04609578	0.999772489
NKG7	2.138781	1.072642	1.993936	0.04615901	NA
RGS1	-1.130538	0.567219	-1.993123	0.04624799	0.999772489
IL10RA	-0.286933	0.144042	-1.992002	0.0463708	0.999772489
ZNF625	1.307075	0.656551	1.990822	0.04650051	0.999772489
ICA1	2.719174	1.365999	1.990612	0.04652353	NA
BBX	0.223993	0.112526	1.990589	0.0465261	0.999772489
RNPC3	-0.532703	0.267807	-1.989134	0.04668647	0.999772489
DICER1.AS1	-1.024646	0.515221	-1.988751	0.04672875	0.999772489
CCDC154	1.108468	0.558124	1.986061	0.04702652	0.999772489
FXYD5	-0.18918	0.095317	-1.984747	0.04717259	0.999772489
LPCAT4	-0.585074	0.295365	-1.980852	0.04760791	0.999772489
ENSG00000276529	1.870662	0.944649	1.980272	0.04767294	NA
TUT1	0.597483	0.301859	1.979342	0.04777749	0.999772489

PDS5B	-0.55419	0.279998	-1.979259	0.04778681	0.999772489
APOL3	0.535771	0.270764	1.978739	0.04784544	0.999772489
PRKCA	1.888652	0.955559	1.976489	0.04809944	0.999772489
C5orf17	1.922096	0.972634	1.976177	0.04813473	NA
TBCK	0.695505	0.352178	1.974867	0.0482833	0.999772489
CFAP410	-0.451586	0.228701	-1.974571	0.0483169	0.999772489
HOXB4	2.225393	1.127955	1.972945	0.04850184	NA
ENSG00000268746	1.563986	0.792997	1.972247	0.04858143	NA
ARL13B	-0.981203	0.497527	-1.972161	0.04859128	0.999772489
WDFY4	-0.416112	0.211009	-1.972014	0.04860804	0.999772489
DERL3	0.341003	0.173086	1.970138	0.04882253	0.999772489
TFE3	-0.921791	0.467906	-1.970037	0.04883415	0.999772489
ZNF239	1.265711	0.642647	1.969528	0.04889253	0.999772489
WDR82	-0.51432	0.261256	-1.96864	0.04899445	0.999772489
ENSG00000226853	1.913425	0.972052	1.968439	0.04901755	0.999772489
GFI1	2.728829	1.386615	1.967979	0.04907045	NA
PACS1	0.315455	0.160352	1.967269	0.04915226	0.999772489
SGMS1	0.599913	0.305164	1.965873	0.04931326	0.999772489
IRF2BPL	0.76387	0.388639	1.965499	0.04935651	0.999772489

Cluster 9	log2FC	lfcSE	stat	pvalue	padj
VPREB3	4.20113	0.146171	28.741187	< 2.22e-16	< 2.22e-16
DENND6B	3.54218	0.202611	17.48262	< 2.22e-16	< 2.22e-16
P2RX5	2.231763	0.156485	14.261856	< 2.22e-16	< 2.22e-16
PLAAT4	2.687207	0.212616	12.638769	< 2.22e-16	< 2.22e-16
NRIP1	2.476226	0.205137	12.071079	< 2.22e-16	< 2.22e-16
SMAD3	1.179846	0.105888	11.142385	< 2.22e-16	< 2.22e-16
GRN	1.738068	0.156749	11.088247	< 2.22e-16	< 2.22e-16
SEPTIN9	0.950286	0.086125	11.033804	< 2.22e-16	< 2.22e-16
CD38	5.158908	0.489618	10.536598	< 2.22e-16	< 2.22e-16
ASB2	5.324524	0.533794	9.974866	< 2.22e-16	< 2.22e-16
CD1D	5.652924	0.580968	9.73018	< 2.22e-16	< 2.22e-16
SLC5A3	1.169983	0.127025	9.210632	< 2.22e-16	< 2.22e-16
DUS2	1.759822	0.197357	8.916956	< 2.22e-16	4.47E-16
RABGAP1L	1.626291	0.18776	8.661538	< 2.22e-16	4.03E-15
SLC2A5	3.241203	0.383694	8.44736	< 2.22e-16	2.41E-14
C12orf65	0.630741	0.075564	8.34708	< 2.22e-16	5.31E-14
RNF207	2.286963	0.308639	7.409839	1.26E-13	9.02E-11
DPEP2	1.587585	0.214795	7.39115	1.46E-13	9.81E-11
SLC12A4	2.092936	0.284384	7.359548	1.85E-13	1.18E-10
RARA.AS1	1.879972	0.263497	7.134706	9.70E-13	5.88E-10
FCRL2	1.518003	0.215055	7.058685	1.68E-12	9.71E-10
AHI1	1.056846	0.152232	6.942351	3.86E-12	2.13E-09
TRABD	0.663898	0.096121	6.906897	4.95E-12	2.61E-09
CD72	1.181273	0.171517	6.887213	5.69E-12	2.88E-09
HIC1	2.759468	0.401388	6.874821	6.21E-12	3.01E-09
MRPS6	0.801899	0.117638	6.816692	9.32E-12	4.35E-09
IRF2BPL	0.8984	0.138047	6.507929	7.62E-11	3.42E-08
ENDOD1	1.658461	0.259628	6.387848	1.68E-10	7.29E-08
LHFPL2	1.976063	0.310017	6.374044	1.84E-10	7.70E-08
CHST15	1.227661	0.193796	6.334821	2.38E-10	9.61E-08
RHOBTB3	2.453235	0.389877	6.292327	3.13E-10	1.22E-07
CACYBP	0.665342	0.107267	6.202664	5.55E-10	2.10E-07
H3F3A	0.462554	0.07744	5.973068	2.33E-09	8.56E-07
CD1C	2.263507	0.380394	5.95043	2.67E-09	9.54E-07
VPREB1	5.438502	0.926062	5.87272	4.29E-09	1.49E-06
LGMN	1.080394	0.185232	5.832655	5.46E-09	1.84E-06
MPEG1	1.740797	0.29928	5.816618	6.01E-09	1.97E-06
CMTM7	1.034133	0.179747	5.753288	8.75E-09	2.79E-06
RARA	0.539822	0.094112	5.735985	9.69E-09	3.02E-06
GGA2	0.801396	0.141481	5.664343	1.48E-08	4.48E-06
ABCB1	1.309048	0.232136	5.639153	1.71E-08	5.06E-06
LCAT	2.29004	0.412196	5.555705	2.76E-08	7.99E-06
ARL14EPL	2.720228	0.498375	5.458194	4.81E-08	1.36E-05
CXCR5	0.824101	0.151141	5.452514	4.97E-08	1.37E-05
IRAK2	0.78859	0.145333	5.426107	5.76E-08	1.55E-05
UBTF	0.721285	0.13304	5.421544	5.91E-08	1.55E-05

PISD	0.904754	0.166969	5.418697	6.00E-08	1.55E-05
PNOC	1.380602	0.256369	5.385214	7.24E-08	1.83E-05
BTBD7	0.58572	0.108967	5.375218	7.65E-08	1.89E-05
MS4A1	-0.654199	0.123925	-5.279002	1.30E-07	3.15E-05
FAM3C	0.486109	0.093176	5.217091	1.82E-07	4.32E-05
P2RX5.TAX1BP3	1.831872	0.357196	5.128484	2.92E-07	6.81E-05
XYLT1	1.205116	0.236275	5.100479	3.39E-07	7.76E-05
ZNF563	1.067412	0.214961	4.965617	6.85E-07	0.000153859
OTUD7A	2.807564	0.567778	4.944823	7.62E-07	0.000168112
AGPAT5	0.506045	0.102484	4.937787	7.90E-07	0.000171178
PTK2B	0.591349	0.12018	4.920522	8.63E-07	0.000183712
JCHAIN	2.119812	0.431713	4.910235	9.10E-07	0.000190278
SIGIRR	0.914882	0.186715	4.899873	9.59E-07	0.000197194
ZNF3	0.648306	0.132424	4.895699	9.80E-07	0.000198069
ACP5	0.917231	0.189588	4.838025	1.31E-06	0.000260809
CLCN3	0.603025	0.125188	4.816959	1.46E-06	0.000285224
AGO1	0.67097	0.139394	4.813474	1.48E-06	0.000285639
APOL3	0.904677	0.188653	4.795468	1.62E-06	0.000307651
KANSL3	0.895359	0.187264	4.781266	1.74E-06	0.000325128
MAP3K1	0.716176	0.152102	4.708522	2.50E-06	0.000458663
ZFP36L1	-0.408682	0.086978	-4.698681	2.62E-06	0.000474139
RRBP1	0.819423	0.175051	4.681064	2.85E-06	0.00050917
IFI30	0.811189	0.174764	4.64164	3.46E-06	0.000607752
CLIC3	2.510137	0.543473	4.618701	3.86E-06	0.000669254
PLAAT3	1.862902	0.404427	4.606277	4.10E-06	0.00070048
TELO2	0.75471	0.167351	4.509729	6.49E-06	0.001093741
RESF1	-0.28691	0.064008	-4.48238	7.38E-06	0.001226749
RMDN2	1.68112	0.375544	4.476498	7.59E-06	0.001243981
TSPAN18	3.069158	0.690164	4.446999	8.71E-06	0.001408575
C15orf39	0.651496	0.146794	4.438164	9.07E-06	0.001448331
NFAT5	0.606071	0.137091	4.420952	9.83E-06	0.001548283
CD22	0.782324	0.178897	4.37303	1.23E-05	0.001905876
SLC43A2	0.482786	0.111061	4.347033	1.38E-05	0.002119132
NCF1	0.624801	0.144051	4.337367	1.44E-05	0.002186785
CTSZ	0.512155	0.119335	4.291723	1.77E-05	0.002655435
CR2	1.792558	0.41954	4.272675	1.93E-05	0.002848992
FAM117A	0.763883	0.178869	4.270642	1.95E-05	0.002848992
SDE2	0.694657	0.165502	4.19726	2.70E-05	0.003901929
SPTBN1	0.648233	0.156394	4.144865	3.40E-05	0.004853003
ENSG00000235609	3.237231	0.785426	4.121623	3.76E-05	0.005307231
GBP4	0.750928	0.183967	4.081865	4.47E-05	0.006229945
CHCHD10	1.906597	0.468943	4.065737	4.79E-05	0.006601037
SRSF8	0.456606	0.112753	4.049621	5.13E-05	0.006993017
SEC61B	-0.244952	0.06066	-4.038096	5.39E-05	0.007263929
SMIM3	1.555549	0.385684	4.033223	5.50E-05	0.007334809
RAB37	1.577632	0.395995	3.983971	6.78E-05	0.008937229
DBNL	0.414232	0.104354	3.969495	7.20E-05	0.009372147

SLA	0.902515	0.227474	3.967546	7.26E-05	0.009372147
NDC1	0.912454	0.230741	3.954456	7.67E-05	0.009796153
CHST7	1.085951	0.277174	3.917936	8.93E-05	0.011286613
RDH10	1.568531	0.40321	3.890111	0.000100198	0.012532028
CNP	0.583369	0.150433	3.877946	0.000105342	0.013040904
IL1B	3.698407	0.95705	3.864384	0.00011137	0.013647899
CD99	-0.355767	0.093117	-3.820654	0.000133098	0.016147464
SLC25A39	0.359223	0.094684	3.793935	0.000148279	0.017811047
FCRL1	0.942339	0.248796	3.787591	0.000152115	0.018092729
ST3GAL1	0.65772	0.176646	3.723386	0.000196569	0.023153113
MFSD10	-0.293517	0.078918	-3.719283	0.000199789	0.023306166
JUNB	0.381981	0.103423	3.693401	0.000221275	0.025566738
ABI3	1.041609	0.283328	3.676339	0.000236605	0.027080127
CLCN7	0.525416	0.143422	3.663426	0.000248864	0.028216953
VHL	0.342618	0.094186	3.637666	0.00027512	0.030893198
NUBP1	0.422287	0.11616	3.63539	0.00027756	0.030893198
GPR146	-1.158499	0.319722	-3.62346	0.000290688	0.032060235
SH3BGRL3	-0.21544	0.059899	-3.59671	0.000322268	0.035223012
PALLD	2.841929	0.794161	3.578527	0.000345536	0.037428924
SREBF1	0.72351	0.20602	3.511844	0.00044501	0.047777512
RBM47	1.401788	0.400152	3.503136	0.000459815	0.048933977
CASP7	0.669455	0.192368	3.480069	0.000501284	0.052883264
PPCDC	0.629864	0.1825	3.451314	0.000557864	0.058344885
PXK	0.505045	0.146608	3.444869	0.000571337	0.059243246
ETFB	0.455694	0.13255	3.437914	0.000586214	0.059891008
RAB20	1.469759	0.427621	3.43706	0.000588064	0.059891008
HIPK2	0.624422	0.181778	3.435073	0.000592394	0.059891008
PUS1	0.516217	0.151158	3.415078	0.000637637	0.063932303
MAPKAPK2	0.536626	0.157936	3.397747	0.000679431	0.067564419
SPIB	0.469067	0.138279	3.39217	0.000693414	0.068394325
TFEB	0.447861	0.132327	3.384505	0.000713067	0.069765554
MCM5	0.386992	0.114863	3.369148	0.000754008	0.073180995
AP3S1	0.335713	0.099834	3.362702	0.000771836	0.074316801
FOXO3	0.608202	0.181538	3.350276	0.000807311	0.077120418
DENND6A	0.934039	0.279098	3.346629	0.000818005	0.07753151
PPFIBP2	0.658906	0.197748	3.332051	0.000862085	0.081076056
SLC5A5	2.896068	0.872124	3.320705	0.000897903	0.083795042
PHF21A	-0.505735	0.153249	-3.300084	0.00096656	0.089196777
DTX4	1.216386	0.36872	3.298945	0.000970489	0.089196777
CD180	0.976973	0.296647	3.293385	0.000989889	0.090295739
PPDPF	-0.288643	0.087948	-3.281964	0.001030866	0.093331856
DDX28	0.534401	0.163812	3.262271	0.001105236	0.098669751
TMEM41B	0.439924	0.134861	3.262052	0.00110609	0.098669751
PHKB	0.349303	0.107546	3.247935	0.001162459	0.102507983
CIB1	0.259249	0.079841	3.247065	0.001166016	0.102507983
SEPTIN2	0.248797	0.076949	3.233253	0.001223889	0.106821769
SLC45A3	0.77827	0.241284	3.225533	0.001257383	0.108961206

CTSA	0.434722	0.134921	3.222041	0.00127281	0.109515826
ENSG00000259436	-0.661649	0.205597	-3.218177	0.001290081	0.110220121
KCNH4	2.237763	0.698274	3.204708	0.001351996	0.114702223
SHQ1	0.611736	0.191329	3.197293	0.001387238	0.116874759
ST8SIA4	-0.35755	0.112244	-3.185475	0.001445164	0.120915335
SPOCK2	0.612804	0.192587	3.181953	0.001462857	0.121557365
PLXNB2	0.95683	0.301615	3.172355	0.001512081	0.124792955
GPR68	-1.412313	0.44553	-3.169966	0.001524568	0.124973346
ITGAE	0.367258	0.116549	3.151109	0.00162652	0.132435821
PPP1R18	0.427393	0.135836	3.14639	0.001652997	0.133694374
LAGE3	-0.252754	0.080733	-3.130744	0.00174364	0.140091654
MED18	0.673176	0.215723	3.120558	0.001805085	0.144074279
DTX1	1.332103	0.42734	3.117201	0.001825769	0.144772729
LZTFL1	0.543395	0.174798	3.108704	0.001879102	0.148034169
CLEC17A	0.627269	0.20225	3.101457	0.001925706	0.150726892
VRK2	-0.460374	0.150921	-3.050424	0.002285189	0.177717369
POU2AF1	0.362082	0.118944	3.044138	0.002333481	0.180317178
KLF3	0.521425	0.172625	3.020572	0.002522979	0.193726496
AKAP1	0.641226	0.214616	2.987789	0.002810038	0.214411197
GDPGP1	1.076511	0.360671	2.984744	0.002838155	0.21513566
PSAP	0.349393	0.117131	2.982934	0.002854998	0.21513566
FAM219B	-0.483377	0.16243	-2.975912	0.002921183	0.218764118
NFATC3	0.379651	0.127826	2.97006	0.002977414	0.221362851
MMP11	1.713663	0.577278	2.96852	0.002992376	0.221362851
BCL3	0.454619	0.154393	2.944546	0.003234286	0.2378082
EIF2AK4	-0.495479	0.168495	-2.940616	0.003275598	0.239394918
RASGEF1B	-0.467752	0.15919	-2.938332	0.003299832	0.23972194
AP1G2	-0.313637	0.106867	-2.934843	0.00333716	0.240990652
ZNF823	1.779579	0.608221	2.925875	0.00343489	0.246580419
CNR2	0.472462	0.161687	2.922079	0.003477032	0.248137356
ERG28	-0.264472	0.09072	-2.915248	0.003554061	0.252151266
HOXB3	2.533283	0.872236	2.904353	0.003680125	0.259577214
STK17A	0.368193	0.127312	2.892053	0.003827339	0.26840044
KCNN1	1.211575	0.420505	2.88124	0.003961136	0.276186785
ENSG00000268798	0.801229	0.278517	2.876772	0.004017664	0.278249627
EHD4	-0.369835	0.128625	-2.875288	0.004036592	0.278249627
BCAR3	0.667107	0.232202	2.872958	0.004066478	0.278726023
COBLL1	-0.383112	0.134109	-2.856713	0.004280528	0.291749256
LCK	0.90728	0.318297	2.850415	0.004366218	0.295927159
FSD1L	-0.524079	0.184191	-2.845302	0.004436937	0.299049575
BCDIN3D	0.401	0.142092	2.822112	0.004770844	0.319778346
FAM107B	0.245826	0.087164	2.820273	0.004798277	0.319849952
SLC38A1	-0.254028	0.090267	-2.814173	0.004890289	0.322752797
ZNF674.AS1	0.86935	0.308953	2.813862	0.004895031	0.322752797
TNK2	0.587259	0.209388	2.804646	0.005037182	0.330330252
GPX1	0.504874	0.18117	2.786748	0.005323984	0.347261137
KAT6A	0.295383	0.106191	2.781617	0.005408891	0.350912636

C16orf54	0.518489	0.187337	2.767687	0.005645569	0.364319365
PAOX	0.584958	0.211558	2.765005	0.005692192	0.36538452
DNAJC7	0.197254	0.071472	2.759873	0.005782386	0.369220573
CERK	0.787041	0.285649	2.755276	0.005864259	0.371318574
ABCD3	-0.68901	0.250131	-2.754596	0.005876456	0.371318574
DDX24	0.183126	0.066525	2.75273	0.005910069	0.37150754
RINL	0.386195	0.140442	2.749859	0.005962097	0.372846177
AHNAK	-0.781529	0.284793	-2.744198	0.006065889	0.377391619
ENSG00000254802	1.288778	0.470477	2.739301	0.006156997	0.381105565
RREB1	-0.418461	0.153038	-2.734363	0.006250105	0.384904972
CBX1	-0.237011	0.087292	-2.715164	0.006624305	0.405889222
DNAH1	1.223255	0.452037	2.706092	0.006808018	0.415049643
SIPA1L1	-0.302267	0.111842	-2.702621	0.006879523	0.417311839
ENSG00000261766	0.488254	0.18101	2.69738	0.006988752	0.421828559
TNIP1	0.261858	0.097422	2.687863	0.007191096	0.430358372
GRK3	0.289173	0.107603	2.687402	0.007201018	0.430358372
NUP210	0.413636	0.1544	2.678997	0.007384314	0.438943565
EAF2	0.441092	0.1648	2.676528	0.007438942	0.438943565
ZCCHC7	0.401552	0.150063	2.675885	0.007453213	0.438943565
LY6E	-0.499076	0.187412	-2.662992	0.007744936	0.45174774
VKORC1	-0.235663	0.08853	-2.661956	0.007768807	0.45174774
STMP1	-0.313196	0.117757	-2.659673	0.00782166	0.45174774
PXDC1	1.05569	0.397052	2.65882	0.007841496	0.45174774
CPSF1	-0.54655	0.205635	-2.657863	0.007863778	0.45174774
ZNF318	-0.56865	0.214054	-2.656568	0.007894042	0.45174774
RAB8B	-0.276925	0.104484	-2.650417	0.008039257	0.455456513
BMP2K	-0.413065	0.15585	-2.650394	0.008039806	0.455456513
TSPO	-0.339414	0.128194	-2.647664	0.008105014	0.455456513
IDS	-0.199415	0.075322	-2.647497	0.008109018	0.455456513
MDM4	-0.226061	0.085462	-2.645177	0.008164828	0.456477874
TMEM65	0.89225	0.338281	2.637602	0.008349449	0.464658324
SLC25A11	0.301582	0.114456	2.63492	0.008415705	0.465761572
ENSG00000225489	1.171301	0.444736	2.633698	0.008446056	0.465761572
STK24	0.335925	0.127945	2.62554	0.008651154	0.474811446
GEN1	-0.458882	0.174874	-2.624076	0.008688439	0.474811446
C7orf50	-0.416154	0.158894	-2.61906	0.008817248	0.479639548
SEL1L3	0.618972	0.236468	2.617569	0.008855857	0.479639548
ALOX5	0.308239	0.11789	2.614646	0.008931994	0.481613105
HMG20B	-0.290299	0.111253	-2.609352	0.009071375	0.486107425
FCHO1	0.63029	0.241634	2.608444	0.009095482	0.486107425
TEX264	0.303553	0.116581	2.603797	0.009219744	0.490587441
S100A6	-0.436979	0.168476	-2.59372	0.009494376	0.502994623
RHOF	-0.24054	0.09284	-2.590906	0.009572372	0.504921827
SPRY1	0.827068	0.319888	2.585494	0.009723968	0.510697761
GAK	0.337317	0.130728	2.580288	0.009871794	0.51622677
SLCO5A1	0.76666	0.297918	2.573393	0.010070681	0.524366971
MACROD2	-0.689297	0.269408	-2.558563	0.010510583	0.542978537

GABARAPL1	0.569782	0.22275	2.557943	0.010529323	0.542978537
SIGLEC5	1.173808	0.459083	2.556853	0.010562392	0.542978537
FTL	-0.246915	0.09664	-2.554998	0.010618849	0.543577528
NLRC3	0.822796	0.322748	2.549343	0.010792594	0.550150235
SACS	-0.506842	0.199004	-2.546895	0.010868622	0.551707635
TBC1D8	1.258568	0.494474	2.545269	0.010919366	0.551973944
A4GALT	0.997873	0.392906	2.539724	0.011094007	0.558437975
PTOV1	-0.547501	0.215696	-2.538298	0.0111393	0.558437975
OXSR1	-0.298102	0.11763	-2.534239	0.011269197	0.560376246
MRPL41	-0.208683	0.082389	-2.532913	0.011311893	0.560376246
ZBED2	-1.377891	0.544025	-2.53277	0.011316533	0.560376246
KLF6	-0.231376	0.091607	-2.525746	0.011545289	0.568264425
C16orf74	0.487421	0.193037	2.52501	0.011569511	0.568264425
ATP13A2	0.789933	0.31386	2.516836	0.011841398	0.577792002
ZNF595	1.052558	0.418293	2.51632	0.011858738	0.577792002
NXT1	0.211656	0.084194	2.513915	0.011939925	0.579420687
MTSS1	-0.376352	0.149906	-2.510598	0.012052693	0.582562844
LMNA	-0.523587	0.208868	-2.506783	0.012183532	0.583058892
ENSG00000245869	0.806325	0.321674	2.506654	0.012188006	0.583058892
PDCD4	0.45043	0.179733	2.506099	0.012207135	0.583058892
VPS13A	-0.330554	0.132243	-2.499598	0.012433427	0.58789538
SLAIN2	-0.271698	0.108701	-2.499511	0.012436481	0.58789538
TBC1D9	0.639017	0.255707	2.499019	0.012453768	0.58789538
ENSG00000229539	0.507374	0.203242	2.496407	0.012545855	0.589946969
CDC42	-0.169791	0.068101	-2.493223	0.012658926	0.592965605
TRIB1	1.036318	0.416037	2.490927	0.012741023	0.594515713
ATF7IP2	0.393143	0.157922	2.489483	0.012792887	0.594648696
TRNAU1AP	-0.244554	0.098321	-2.487307	0.01287143	0.595395116
FDFT1	0.261521	0.105195	2.486063	0.012916517	0.595395116
R3HDM2	0.406995	0.163783	2.484972	0.012956175	0.595395116
ZNF816.ZNF321P	-1.242906	0.50045	-2.483577	0.01300703	0.595476549
ING2	-0.499854	0.201497	-2.480696	0.01311262	0.598053764
BMPR1A	-1.823765	0.736099	-2.477609	0.013226613	0.600993536
MIR34AHG	-1.18223	0.477687	-2.474906	0.013327139	0.60330169
HOMER2	-0.667714	0.270034	-2.472705	0.013409476	0.604772359
BMF	-0.293442	0.119355	-2.458569	0.013949186	0.626783411
FAM234B	-1.070308	0.436502	-2.452011	0.014206044	0.635675796
TBL1X	-0.447283	0.182501	-2.450851	0.014251881	0.635675796
SGSM3	-0.327558	0.134169	-2.441385	0.01463104	0.650196988
SMARCB1	0.251275	0.103166	2.435626	0.014866048	0.654607402
ACO2	-0.256851	0.105468	-2.435338	0.014877888	0.654607402
ISG15	-0.463727	0.190443	-2.434991	0.014892157	0.654607402
INKA2	-0.569365	0.234563	-2.427342	0.015209891	0.663117811
NEB	-1.096033	0.451543	-2.427303	0.015211544	0.663117811
PLEKHO2	0.501758	0.206805	2.426231	0.01525654	0.663117811
CASZ1	-0.550307	0.227039	-2.423841	0.015357338	0.663117811
C16orf72	0.329925	0.136119	2.4238	0.015359059	0.663117811

CEP76	0.711725	0.293961	2.421159	0.015471097	0.665586365
CHP1	-0.291961	0.120659	-2.419721	0.015532423	0.665863444
VTI1B	-0.219314	0.091011	-2.409751	0.015963401	0.677562301
GTF2E2	0.330975	0.137352	2.409694	0.015965903	0.677562301
CEP350	-0.251231	0.104265	-2.409535	0.015972867	0.677562301
IGLV1.51	-1.526973	0.634305	-2.407317	0.016070205	0.679316113
GPBP1	-0.198265	0.082474	-2.403973	0.016217979	0.683182376
DDAH2	-0.333348	0.138877	-2.400314	0.016381004	0.685537486
EVI2B	0.226859	0.094559	2.39911	0.016434967	0.685537486
PLP2	-0.265446	0.110652	-2.398922	0.016443407	0.685537486
SERPINF1	-1.085649	0.453415	-2.39438	0.016648498	0.68963614
DNAJB11	0.226518	0.09461	2.394228	0.016655406	0.68963614
NRM	0.58774	0.246029	2.38891	0.016898444	0.695571284
PDP1	-0.566674	0.237243	-2.388585	0.016913413	0.695571284
UBXN2B	-0.37886	0.15872	-2.386977	0.016987552	0.696260056
DPM3	-0.289425	0.12167	-2.378776	0.017370227	0.70954744
MNT	0.447382	0.18847	2.373761	0.017607937	0.716843943
LMF2	-0.292591	0.123357	-2.371912	0.017696287	0.718031275
APOL1	0.648272	0.274187	2.364342	0.018062147	0.728893056
LSP1	0.320595	0.135622	2.363891	0.018084142	0.728893056
STK4	-0.137928	0.058528	-2.356628	0.018441733	0.740403269
CD63	-0.261132	0.110909	-2.354469	0.018549206	0.740403269
SLC9A7	0.354285	0.150478	2.354397	0.018552802	0.740403269
TMED2	-0.150194	0.063851	-2.352259	0.018659779	0.742230951
TMSB10	-0.244753	0.104212	-2.348616	0.018843349	0.744793898
POU2F2	-0.209001	0.088992	-2.348544	0.018846994	0.744793898
RUNX2	1.708259	0.730051	2.339919	0.01928794	0.75974443
MCL1	0.226048	0.096677	2.338177	0.019378065	0.760824214
JUN	0.333653	0.142909	2.33472	0.019558042	0.765309716
SNX11	-0.22347	0.095877	-2.330789	0.01976449	0.765309716
CD79B	0.356772	0.15311	2.330166	0.019797383	0.765309716
SEC22C	-0.309347	0.132816	-2.329143	0.019851511	0.765309716
MCM2	0.825798	0.354558	2.329093	0.019854125	0.765309716
GPD1L	-1.64327	0.705636	-2.328779	0.019870801	0.765309716
RGS9	-0.826129	0.355601	-2.323189	0.020169014	0.770383624
ENSG00000273188	1.066013	0.458901	2.322971	0.02018072	0.770383624
CHTF18	-0.854574	0.367916	-2.322742	0.020193043	0.770383624
SMARCA4	0.369394	0.159288	2.319038	0.020392995	0.772851463
ENSG00000272369	-1.099261	0.474096	-2.318648	0.020414119	0.772851463
FAM161A	-0.618216	0.266701	-2.318009	0.020448839	0.772851463
MIR181A2HG	1.400356	0.60478	2.31548	0.020586657	0.775643844
TRAPPC1	-0.157026	0.067879	-2.313308	0.020705702	0.776578264
KLF13	0.278118	0.120257	2.312694	0.020739479	0.776578264
ZFYVE21	-0.422378	0.18273	-2.311491	0.020805747	0.776662537
MREG	-0.484657	0.210222	-2.305456	0.021141065	0.786758888
SNX18	0.778032	0.338597	2.297809	0.021572661	0.798171103
ZNF785	0.511166	0.222475	2.297628	0.021582994	0.798171103

BTG2	-0.341531	0.148718	-2.296503	0.021647164	0.798171103
SYNPO	-1.816192	0.791235	-2.295388	0.021710886	0.798171103
VMP1	-0.198915	0.086737	-2.293306	0.021830405	0.800140385
DNAJB12	0.227664	0.099478	2.288588	0.022103298	0.807702445
UAP1L1	-0.956752	0.418809	-2.284458	0.022344604	0.812757999
ENSG00000253535	0.793871	0.34759	2.28393	0.022375632	0.812757999
MPHOSPH9	0.386918	0.169777	2.278981	0.022668173	0.82092621
AP1S1	-0.331907	0.146376	-2.267495	0.023360025	0.843463768
HDLBP	0.276333	0.121956	2.265849	0.02346064	0.844583041
KMT2E	-0.192905	0.085197	-2.264219	0.023560671	0.845674743
PAXX	0.357643	0.158229	2.260289	0.023803337	0.851864566
PKD1	0.495828	0.21971	2.256742	0.024024221	0.852764869
EXOC8	0.59023	0.261589	2.256326	0.024050243	0.852764869
PIGH	0.305861	0.135577	2.255992	0.024071162	0.852764869
CNR1	1.018237	0.45169	2.254282	0.024178429	0.852764869
MON2	-0.419408	0.186052	-2.254258	0.024179947	0.852764869
TGFB1	0.227257	0.100997	2.250134	0.024440461	0.859032694
TMEM14A	-0.760144	0.338017	-2.248832	0.024523178	0.859032694
FCMR	0.514988	0.229101	2.24787	0.024584511	0.859032694
CABLES1	-0.886468	0.394514	-2.246986	0.024640898	0.859032694
TRIM72	1.958977	0.873209	2.243423	0.024869576	0.860755606
PRDX4	-0.279723	0.124746	-2.242335	0.024939706	0.860755606
FXYD7	-0.802554	0.358515	-2.238548	0.025185317	0.860755606
RPA2	0.163575	0.073073	2.238525	0.025186816	0.860755606
RAB30	0.288258	0.128777	2.238432	0.025192908	0.860755606
PTGER4	-0.553118	0.247163	-2.237864	0.025229928	0.860755606
CAND1	-0.217975	0.097404	-2.237857	0.025230381	0.860755606
PTMS	-0.720704	0.32215	-2.237172	0.025275132	0.860755606
TMOD2	-0.401922	0.179722	-2.23635	0.025328862	0.860755606
ABCB4	0.513057	0.229639	2.234191	0.02547051	0.863151483
LILRA4	2.526109	1.131762	2.232015	0.025613999	0.865596217
ENSG00000260278	-0.866375	0.388819	-2.228224	0.025865579	0.869392884
DDIT4	0.398482	0.178952	2.226756	0.025963588	0.869392884
SLC12A6	0.279361	0.125473	2.226462	0.025983227	0.869392884
CBX4	0.801718	0.360158	2.226018	0.026012992	0.869392884
ENSG00000283013	0.413042	0.185729	2.223902	0.026155018	0.871379851
HIST1H2BD	0.361774	0.162742	2.222996	0.026216093	0.871379851
ENSG00000263394	0.881291	0.396764	2.221197	0.02633763	0.872813537
NDUFB6	0.197964	0.089164	2.220231	0.026403113	0.872813537
COPS6	0.167202	0.075369	2.218444	0.026524545	0.874445048
SUMO1	-0.121373	0.054798	-2.214927	0.026765089	0.879983899
PARP14	-0.235849	0.106612	-2.21221	0.026952177	0.882041009
PRKAG2	-0.355225	0.160685	-2.210696	0.027056907	0.882041009
TIPARP	-0.275902	0.124804	-2.210676	0.027058294	0.882041009
BRAT1	0.481258	0.217846	2.209162	0.0271634	0.882041009
EXOSC5	0.361271	0.163563	2.208762	0.027191175	0.882041009
CCDC154	0.957213	0.43368	2.207186	0.02730108	0.883244528

TRIM25	0.524036	0.238207	2.199919	0.027812645	0.89740163
CFP	-1.937156	0.881814	-2.196785	0.028035808	NA
ETFRF1	0.352526	0.160722	2.193395	0.028278934	0.910026586
ATXN10	0.291349	0.133053	2.189724	0.028544232	0.915294521
PSMD4	0.170411	0.077847	2.189046	0.028593523	0.915294521
CCAR1	0.201262	0.092084	2.185635	0.028842314	0.920828838
CCDC88B	0.257207	0.117841	2.182658	0.029060989	0.925375129
TLR10	-0.417036	0.191459	-2.178197	0.029391405	0.927971908
C16orf70	-0.560186	0.257268	-2.177441	0.029447706	0.927971908
ASH1L	-0.195403	0.089805	-2.175868	0.029565131	0.927971908
EWSR1	0.269711	0.123963	2.175731	0.029575349	0.927971908
IQGAP1	-0.183179	0.0842	-2.175526	0.029590722	0.927971908
PTCD3	0.234193	0.107656	2.175382	0.029601478	0.927971908
FLNA	-0.274315	0.12631	-2.171762	0.029873654	0.934090648
ADAM28	0.369721	0.170393	2.169809	0.030021348	0.9362956
DPEP3	2.101063	0.970733	2.164408	0.030433041	NA
EML2	0.321961	0.148845	2.163057	0.030536827	0.947957041
STIM2	-0.406045	0.187735	-2.162865	0.030551533	0.947957041
ENSG00000273472	0.631832	0.292379	2.161003	0.030695111	0.949011588
MYH3	0.757245	0.350727	2.15907	0.030844756	0.949011588
RRP36	0.355662	0.164778	2.158424	0.030894863	0.949011588
ENSG00000267169	-0.841932	0.390078	-2.158367	0.030899326	0.949011588
OTUD4	0.330881	0.153372	2.157373	0.030976639	0.949011588
PPP1R3D	-1.286487	0.596742	-2.155851	0.031095307	0.950247526
ENSG00000278376	0.604691	0.280961	2.152224	0.031379751	0.95646903
FRY.AS1	-1.43633	0.667944	-2.150375	0.031525547	0.95646903
MEI1	-0.577974	0.269053	-2.148179	0.031699504	0.95646903
CR1	1.584478	0.737789	2.147602	0.03174536	0.95646903
LINC00158	-0.383807	0.178848	-2.14599	0.031873783	0.95646903
PRKCE	0.356817	0.166317	2.145396	0.031921167	0.95646903
CFL1	-0.122644	0.057192	-2.144431	0.031998356	0.95646903
ARHGEF1	0.306842	0.143118	2.14398	0.032034498	0.95646903
ZNF33B	-0.347691	0.162175	-2.143927	0.032038771	0.95646903
C1orf50	-0.25756	0.120187	-2.142998	0.032113282	0.95646903
NSMAF	-0.52765	0.24636	-2.141781	0.032211075	0.95646903
PRMT2	-0.21615	0.100941	-2.141361	0.032244958	0.95646903
ATAD2	0.425762	0.198925	2.140316	0.032329277	0.956631184
ENSG00000231609	1.125003	0.526527	2.136648	0.032626599	0.962364926
ARL5A	-0.176546	0.082654	-2.135972	0.032681697	0.962364926
ENSG00000269482	1.917492	0.897982	2.135336	0.032733576	NA
UTP14C	0.513652	0.240882	2.132377	0.032975876	0.967049198
DDX10	0.345972	0.162336	2.131205	0.033072273	0.967049198
ASCC1	0.26807	0.125789	2.131112	0.033079906	0.967049198
PTTG1	-0.395231	0.185947	-2.125502	0.033544774	0.976213555
ATP8A1	0.417149	0.19627	2.125387	0.033554323	0.976213555
ZDHHC14	-0.480173	0.226034	-2.124338	0.033641896	0.976419821
ARID5B	-0.3847	0.181216	-2.122881	0.033763802	0.976997922

TPM3	-0.198951	0.093749	-2.122177	0.033822876	0.976997922
ENSG00000272843	-1.237232	0.583329	-2.120987	0.033922862	0.977025966
WDR82	0.225246	0.106235	2.120251	0.033984912	0.977025966
CPNE5	0.547758	0.258611	2.118079	0.034168393	0.977909547
TSC22D1	-0.82205	0.388129	-2.117979	0.034176859	0.977909547
GTF3A	0.163562	0.077269	2.116776	0.034278833	0.978519533
SMAP2	-0.202093	0.095564	-2.114727	0.034453263	0.981165578
KDM4B	0.269013	0.127266	2.113789	0.034533276	0.981165578
LAMTOR2	-0.26465	0.125283	-2.112421	0.034650341	0.982191437
TMEM219	-0.220309	0.10439	-2.11044	0.034820509	0.984714265
SH2B3	0.322157	0.152824	2.108022	0.035029128	0.988310195
POLR2M	-0.224511	0.106788	-2.102387	0.035519384	0.996406127
SLC17A9	0.689978	0.328274	2.101836	0.035567658	0.996406127
UNC79	1.692767	0.805582	2.101298	0.035614772	0.996406127
MSL3	-0.194983	0.092807	-2.100959	0.035644598	0.996406127
SUSD6	-0.27581	0.131461	-2.098041	0.035901521	0.999017259
ADO	-0.458017	0.218346	-2.097665	0.035934727	0.999017259
TANGO6	0.60547	0.288718	2.097096	0.035985043	0.999017259
ENSG00000259820	1.379818	0.658365	2.095825	0.036097742	0.999195731
ENSG00000281325	-0.426657	0.203689	-2.094648	0.036202284	0.999195731
AHCYL1	0.248652	0.118731	2.094241	0.036238553	0.999195731
DMD	-0.547915	0.26177	-2.093115	0.036338869	0.999689715
RHOC	-0.371945	0.177825	-2.091627	0.03647187	0.99991192
DSEL	-1.068615	0.5117	-2.088362	0.036765209	0.99991192
ZBTB20	0.374929	0.179592	2.087674	0.03682729	0.99991192
ZNF506	0.316728	0.151849	2.085802	0.036996537	0.99991192
SAMD1	-0.486099	0.233231	-2.084197	0.037142207	0.99991192
MEF2A	0.267289	0.128406	2.081598	0.037379201	0.99991192
ENSG00000272716	0.784418	0.377061	2.080349	0.037493533	0.99991192
SYVN1	0.313478	0.15076	2.079312	0.037588724	0.99991192
ENSG00000258623	-0.762831	0.367216	-2.077337	0.037770423	0.99991192
WDR74	0.396508	0.191552	2.069978	0.038454428	0.99991192
PIK3CD	0.273955	0.132577	2.066395	0.038791186	0.99991192
TBL1XR1	-0.217817	0.10552	-2.064221	0.038996749	0.99991192
MAD2L2	-0.294441	0.142657	-2.063974	0.039020134	0.99991192
ENSG00000089127	0.644411	0.312434	2.062552	0.039155186	0.99991192
DIAPH1	-0.287393	0.139466	-2.060669	0.03933459	0.99991192
MAP1S	0.362201	0.175798	2.060329	0.039367058	0.99991192
STX2	-0.443047	0.215134	-2.059401	0.039455836	0.99991192
TMEM191C	0.763947	0.371555	2.056079	0.03977492	0.99991192
COX11	-0.280081	0.136224	-2.056023	0.039780333	0.99991192
KCP	1.458795	0.710298	2.053779	0.039997092	0.99991192
RASGRP1	-0.421474	0.205242	-2.053549	0.040019393	0.99991192
SMC6	-0.189885	0.092557	-2.051546	0.040213851	0.99991192
SAMD4A	-0.843929	0.411679	-2.049967	0.040367658	0.99991192
SNURF	0.841268	0.410567	2.049041	0.040458143	0.99991192
TAF1B	-0.469439	0.22925	-2.047715	0.040587921	0.99991192

RBM38	0.249444	0.121892	2.046432	0.040713935	0.99991192
UTP3	0.235624	0.11517	2.045891	0.040767126	0.99991192
ZBTB39	0.703835	0.344307	2.044207	0.040933055	0.99991192
ATP2B1	-0.216847	0.106117	-2.043479	0.04100504	0.99991192
MIR181A1HG	0.468269	0.229174	2.043289	0.041023823	0.99991192
ELP4	0.439182	0.214984	2.04286	0.041066348	0.99991192
NEDD8	-0.115112	0.056349	-2.042825	0.041069732	0.99991192
AP3B1	0.432062	0.211645	2.041447	0.041206451	0.99991192
IFIT5	-0.458412	0.224626	-2.040782	0.04127253	0.99991192
TNFRSF13B	-0.818944	0.401396	-2.04024	0.041326425	0.99991192
BHLHE40	0.315968	0.154937	2.039329	0.04141718	0.99991192
FADS3	-0.332959	0.163289	-2.039078	0.041442291	0.99991192
MICOS10	0.123313	0.060509	2.037929	0.041557017	0.99991192
CD79A	0.319856	0.157203	2.034663	0.041884778	0.99991192
MAP3K2	-0.276981	0.136294	-2.032231	0.042130298	0.99991192
CTSB	0.273868	0.134807	2.031565	0.042197735	0.99991192
SAT2	-0.211369	0.104099	-2.030471	0.042308732	0.99991192
VIRMA	0.279086	0.137517	2.029463	0.04241118	0.99991192
CPEB4	0.490584	0.241843	2.02852	0.04250716	0.99991192
UBE2D4	-0.331477	0.163522	-2.027109	0.042651299	0.99991192
BNIP3	-0.254064	0.125379	-2.026368	0.042727134	0.99991192
NOP14.AS1	-0.692084	0.341706	-2.025375	0.042828859	0.99991192
TOE1	0.235814	0.116432	2.025337	0.042832799	0.99991192
CELF4	-1.827377	0.902975	-2.02373	0.042997961	NA
STRN	0.39695	0.196428	2.02084	0.043296363	0.99991192
TRBC2	-0.183245	0.090692	-2.020529	0.04332859	0.99991192
TGFBR2	-0.227654	0.112739	-2.019297	0.043456389	0.99991192
WARS	0.604007	0.299503	2.016697	0.043727171	0.99991192
CCL2	2.498478	1.239449	2.015797	NA	NA
RAB13	-0.436097	0.216417	-2.01508	0.043896276	0.99991192
IGFLR1	-0.374876	0.18644	-2.010701	0.044357056	0.99991192
FADS2	1.173248	0.583803	2.009666	0.044466499	0.99991192
IDE	-0.573144	0.285269	-2.009133	0.04452308	0.99991192
ADRB2	-0.827001	0.412324	-2.005708	0.044887439	0.99991192
MYADM	0.363771	0.181446	2.004837	0.044980466	0.99991192
CCT8	0.204432	0.102013	2.003982	0.045071955	0.99991192
LGALS1	-0.456311	0.22777	-2.003384	0.045136115	0.99991192
ATP6V1E1	0.242241	0.121023	2.001615	0.045326185	0.99991192
NMT2	-0.43517	0.217417	-2.001542	0.045334022	0.99991192
RPS6KB2	-0.170357	0.085328	-1.996498	0.045879786	0.99991192
SIK3	0.40949	0.205405	1.993577	0.046198304	0.99991192
RPP25L	0.255634	0.128242	1.993364	0.04622162	0.99991192
TAF9B	-0.359349	0.180381	-1.992172	0.046352191	0.99991192
EGLN2	-0.175615	0.088173	-1.991719	0.046401945	0.99991192
UTP23	0.254434	0.127748	1.991687	0.046405394	0.99991192
CTNND1	-0.41456	0.208315	-1.990065	0.046583819	0.99991192
SOGA1	-0.649921	0.326914	-1.988047	0.046806438	0.99991192

TP53I3	-0.908808	0.457373	-1.987017	0.046920519	0.99991192
GPR180	-0.693306	0.349462	-1.983924	0.047264299	0.99991192
SHISAL2A	0.356614	0.179791	1.983496	0.047312002	0.99991192
ENSG00000226571	-0.640096	0.323103	-1.981089	0.04758124	0.99991192
GPS2	-0.284471	0.143633	-1.980546	0.047642239	0.99991192
L1CAM	-0.628572	0.317603	-1.979112	0.047803394	0.99991192
ATG13	0.236143	0.119585	1.974683	0.048304112	0.99991192
CARHSP1	0.201049	0.101907	1.972861	0.048511379	0.99991192
SNAPIN	-0.22874	0.115969	-1.972426	0.048560953	0.99991192
CCNT2	-0.261792	0.132774	-1.971713	0.048642336	0.99991192
NRP2	-0.400248	0.203137	-1.970332	0.048800303	0.99991192
SPACA9	-0.768868	0.390329	-1.969797	0.048861677	0.99991192
EPHB6	-0.396249	0.201247	-1.96897	0.0489565	0.99991192
PFN1	-0.158729	0.080667	-1.967709	0.04910158	0.99991192
MEN1	-0.335494	0.17058	-1.96678	0.049208552	0.99991192
VPS37C	-0.476902	0.24257	-1.966036	0.049294426	0.99991192
TPRA1	0.375977	0.191241	1.965983	0.049300616	0.99991192
TP53I11	-0.342961	0.174488	-1.965525	0.049353518	0.99991192

Cluster 10	log2FC	lfcSE	stat	pvalue	padj
VPREB3	3.983177	0.144048	27.651685	< 2.22e-16	< 2.22e-16
GRN	1.648943	0.091766	17.969007	< 2.22e-16	< 2.22e-16
P2RX5	1.833474	0.107016	17.132704	< 2.22e-16	< 2.22e-16
CD1D	5.842222	0.369987	15.790325	< 2.22e-16	< 2.22e-16
DENND6B	2.997422	0.198313	15.114605	< 2.22e-16	< 2.22e-16
NRIP1	2.153634	0.153913	13.992519	< 2.22e-16	< 2.22e-16
CD38	3.832	0.302384	12.672646	< 2.22e-16	< 2.22e-16
RABGAP1L	1.670832	0.135364	12.343296	< 2.22e-16	< 2.22e-16
PLAAT4	2.455264	0.216315	11.350397	< 2.22e-16	< 2.22e-16
HIC1	4.409995	0.399595	11.036159	< 2.22e-16	< 2.22e-16
MRPS6	0.95425	0.08714	10.950712	< 2.22e-16	< 2.22e-16
SLC5A3	1.409247	0.137919	10.217929	< 2.22e-16	< 2.22e-16
FCRL2	1.845225	0.182441	10.11411	< 2.22e-16	< 2.22e-16
C12orf65	0.947412	0.096472	9.820589	< 2.22e-16	< 2.22e-16
ASB2	5.768485	0.59305	9.726808	< 2.22e-16	< 2.22e-16
RNF207	3.276308	0.348802	9.39302	< 2.22e-16	< 2.22e-16
SLC2A5	3.370011	0.365318	9.224866	< 2.22e-16	< 2.22e-16
MS4A1	-0.778299	0.085783	-9.072913	< 2.22e-16	< 2.22e-16
CXCR5	0.814065	0.093325	8.72287	< 2.22e-16	1.66E-15
CACYBP	0.908726	0.106092	8.565456	< 2.22e-16	6.26E-15
SMAD3	1.092572	0.128741	8.486565	< 2.22e-16	1.18E-14
SLC12A4	2.064839	0.246099	8.390282	< 2.22e-16	2.56E-14
CR2	2.339262	0.284215	8.230598	< 2.22e-16	9.42E-14
SEPTIN9	0.49165	0.060709	8.098435	5.57E-16	2.70E-13
CTSA	0.704792	0.08825	7.986324	1.39E-15	6.47E-13
SIGIRR	0.959555	0.120484	7.964148	1.66E-15	7.44E-13
P2RX5.TAX1BP3	1.823501	0.235258	7.751083	9.11E-15	3.93E-12
MAPKAPK2	0.969027	0.126805	7.641892	2.14E-14	8.89E-12
AHI1	1.15612	0.151991	7.606503	2.82E-14	1.13E-11
CMTM7	1.28791	0.169881	7.581229	3.42E-14	1.33E-11
IL1B	3.0507	0.408709	7.464232	8.38E-14	3.14E-11
ZNF563	1.297376	0.174453	7.436836	1.03E-13	3.75E-11
IRF2BPL	1.187376	0.160456	7.399994	1.36E-13	4.80E-11
XYLT1	1.111666	0.150364	7.393153	1.43E-13	4.91E-11
PISD	1.065856	0.144894	7.356117	1.89E-13	6.29E-11
RAB20	1.818446	0.248177	7.327207	2.35E-13	7.59E-11
CHST15	1.133829	0.156917	7.225672	4.99E-13	1.57E-10
RMDN2	1.68537	0.238714	7.060218	1.66E-12	5.09E-10
PNOC	1.005383	0.142652	7.047781	1.82E-12	5.42E-10
ST3GAL1	0.956853	0.135921	7.039798	1.93E-12	5.60E-10
ENDOD1	1.58254	0.225575	7.01559	2.29E-12	6.50E-10
GPX1	0.572024	0.082439	6.938788	3.95E-12	1.10E-09
LHFPL2	1.53976	0.228931	6.725862	1.75E-11	4.72E-09
PHF21A	-0.880336	0.131331	-6.703163	2.04E-11	5.39E-09
SLC5A5	4.873284	0.745746	6.534779	6.37E-11	1.65E-08
C1orf162	-0.859663	0.133309	-6.448634	1.13E-10	2.85E-08

PLAAT3	2.193328	0.345227	6.353284	2.11E-10	5.22E-08
RARA.AS1	2.683833	0.42503	6.314458	2.71E-10	6.46E-08
RHOBTB3	1.732839	0.274453	6.313793	2.72E-10	6.46E-08
AK2	0.492716	0.078203	6.300479	2.97E-10	6.90E-08
EXOSC5	0.628155	0.100586	6.244926	4.24E-10	9.67E-08
BTBD7	0.668557	0.107197	6.236723	4.47E-10	1.00E-07
ZNF3	0.931616	0.149856	6.216738	5.08E-10	1.11E-07
CHST7	1.90862	0.310068	6.155486	7.48E-10	1.61E-07
ACP5	0.696123	0.114313	6.089615	1.13E-09	2.38E-07
POU2F2	-0.433043	0.071133	-6.087835	1.14E-09	2.38E-07
GCHFR	-0.572332	0.09471	-6.042981	1.51E-09	3.09E-07
TMEM159	-1.167094	0.194658	-5.995626	2.03E-09	4.07E-07
MANF	0.647309	0.108233	5.980676	2.22E-09	4.38E-07
ZFP36L1	-0.441244	0.074788	-5.899905	3.64E-09	7.05E-07
SMC6	-0.484552	0.082362	-5.883189	4.02E-09	7.68E-07
SLC25A39	0.493878	0.084679	5.832327	5.47E-09	1.02E-06
CIB1	0.363813	0.06239	5.831268	5.50E-09	1.02E-06
CD1C	2.129751	0.366379	5.812975	6.14E-09	1.11E-06
MPEG1	0.992793	0.170853	5.810807	6.22E-09	1.11E-06
HSPA5	0.605095	0.104359	5.798228	6.70E-09	1.18E-06
ICAM3	-0.441281	0.07625	-5.787322	7.15E-09	1.24E-06
SRGN	0.65999	0.114093	5.784642	7.27E-09	1.24E-06
NME1	0.450916	0.078669	5.731817	9.94E-09	1.68E-06
IFI30	0.842136	0.147566	5.706849	1.15E-08	1.91E-06
LZTFL1	1.250148	0.219144	5.704698	1.17E-08	1.91E-06
VPREB1	4.898095	0.859959	5.695733	1.23E-08	1.98E-06
CD82	-0.53315	0.093652	-5.692909	1.25E-08	1.99E-06
JCHAIN	1.960433	0.344503	5.690614	1.27E-08	1.99E-06
ENSG00000254802	1.281403	0.226066	5.668268	1.44E-08	2.24E-06
NFAT5	0.657633	0.116603	5.639952	1.70E-08	2.60E-06
ACAP1	-0.518332	0.091941	-5.637669	1.72E-08	2.60E-06
ENSG00000166927	-1.155884	0.205709	-5.619012	1.92E-08	2.86E-06
DDX24	0.437937	0.078265	5.595578	2.20E-08	3.24E-06
TSPAN18	2.833456	0.509047	5.566202	2.60E-08	3.79E-06
AGPAT5	0.879902	0.158974	5.534896	3.11E-08	4.47E-06
CD72	1.180732	0.214156	5.513428	3.52E-08	4.97E-06
H3F3A	0.471046	0.085454	5.51226	3.54E-08	4.97E-06
WARS	1.094703	0.198862	5.504846	3.69E-08	5.09E-06
TSPO	-0.528454	0.096017	-5.503762	3.72E-08	5.09E-06
CR1	2.833472	0.517453	5.475801	4.36E-08	5.89E-06
DDX21	0.456191	0.083407	5.469469	4.51E-08	6.04E-06
GEN1	-0.907255	0.165989	-5.465758	4.61E-08	6.09E-06
FADS3	-0.960922	0.175937	-5.461741	4.71E-08	6.16E-06
LBH	-0.645551	0.119398	-5.406702	6.42E-08	8.30E-06
TMSB10	-0.314885	0.058527	-5.38013	7.44E-08	9.52E-06
BIN1	-0.568549	0.105831	-5.372223	7.78E-08	9.83E-06
TRABD	0.46774	0.087834	5.325268	1.01E-07	1.25E-05

RAB37	1.303589	0.244799	5.325148	1.01E-07	1.25E-05
ATP1B3	0.502045	0.094583	5.307971	1.11E-07	1.35E-05
VIM	-1.297857	0.244554	-5.307029	1.11E-07	1.35E-05
PUS1	0.637097	0.120085	5.305371	1.12E-07	1.35E-05
DUS2	0.806039	0.152685	5.279098	1.30E-07	1.54E-05
RINL	0.556501	0.106219	5.239205	1.61E-07	1.90E-05
PDLIM2	-0.841925	0.161218	-5.222278	1.77E-07	2.06E-05
GPSM3	-0.363153	0.069651	-5.213924	1.85E-07	2.13E-05
FXYD2	-1.691484	0.326885	-5.174553	2.28E-07	2.61E-05
SERPINF1	-1.894627	0.366286	-5.172533	2.31E-07	2.61E-05
NDUFA2	0.323369	0.062554	5.169432	2.35E-07	2.63E-05
LAT2	-0.678266	0.131493	-5.158177	2.49E-07	2.76E-05
LMNA	-1.712033	0.334704	-5.115066	3.14E-07	3.44E-05
MACROD2	-0.916383	0.179682	-5.100025	3.40E-07	3.69E-05
SREBF1	1.086003	0.2151	5.048818	4.45E-07	4.79E-05
ENSG00000237499	-0.958226	0.190603	-5.02733	4.97E-07	5.31E-05
IRF4	0.549635	0.10978	5.006697	5.54E-07	5.86E-05
LCAT	1.632951	0.326282	5.004722	5.59E-07	5.86E-05
OTUD7A	3.227232	0.648974	4.972821	6.60E-07	6.85E-05
NANP	1.233791	0.249027	4.95444	7.25E-07	7.47E-05
TLR10	-0.571623	0.115486	-4.949732	7.43E-07	7.55E-05
IQSEC1	-0.607643	0.122786	-4.948821	7.47E-07	7.55E-05
MAP3K1	0.629558	0.127734	4.928673	8.28E-07	8.30E-05
UBE2G1	0.64005	0.130027	4.922447	8.55E-07	8.50E-05
AP3S1	0.543852	0.110718	4.91205	9.01E-07	8.89E-05
NOLC1	0.534795	0.109214	4.896768	9.74E-07	9.52E-05
DNAJB11	0.497216	0.102186	4.86578	1.14E-06	0.000110529
DKC1	0.366208	0.075341	4.860702	1.17E-06	0.000112466
HNRNPLL	1.506956	0.310694	4.850298	1.23E-06	0.000117557
CD37	-0.63608	0.131208	-4.847864	1.25E-06	0.000118041
MRPL4	0.362829	0.075142	4.8286	1.37E-06	0.000128047
HIPK2	0.921838	0.190917	4.828481	1.38E-06	0.000128047
CD22	0.536267	0.111725	4.799876	1.59E-06	0.000146592
NEURL2	1.840873	0.385208	4.778903	1.76E-06	0.00016146
BLOC1S1	-0.253917	0.053179	-4.774727	1.80E-06	0.000163559
TP53I11	-0.759227	0.159118	-4.771485	1.83E-06	0.000164925
DDAH2	-0.683977	0.143716	-4.759229	1.94E-06	0.000173914
PLP2	-0.729683	0.153397	-4.756831	1.97E-06	0.000174648
ARL14EPL	4.160246	0.877011	4.743664	2.10E-06	NA
HCK	-1.354058	0.288816	-4.688305	2.75E-06	0.000242796
NDUFB9	0.252709	0.05415	4.666869	3.06E-06	0.000267515
TRBC2	-0.348811	0.074884	-4.658003	3.19E-06	0.000277212
TMEM191B	1.083338	0.23364	4.636774	3.54E-06	0.000303909
FKBP2	0.326492	0.070426	4.635971	3.55E-06	0.000303909
MYC	0.702075	0.15197	4.619821	3.84E-06	0.00032571
MARCHF9	-0.675111	0.146172	-4.618593	3.86E-06	0.00032571
RAB13	-0.99161	0.214804	-4.616344	3.91E-06	0.00032689

ENSG00000198106	-0.919613	0.199361	-4.612804	3.97E-06	0.000330134
GYPC	0.535855	0.116394	4.603816	4.15E-06	0.000342269
DPEP2	0.812522	0.176873	4.593819	4.35E-06	0.00035463
LAPTM5	-0.420402	0.091521	-4.593489	4.36E-06	0.00035463
XBP1	0.503329	0.10964	4.59074	4.42E-06	0.000356838
C15orf62	-1.284964	0.280006	-4.589065	4.45E-06	0.000357233
NOP16	0.350365	0.076395	4.586206	4.51E-06	0.000359676
PXK	0.693585	0.151298	4.584233	4.56E-06	0.000360619
QPRT	1.723283	0.376557	4.576423	4.73E-06	0.000371811
RRP9	0.590493	0.129313	4.566399	4.96E-06	0.000387416
RESF1	-0.423549	0.093051	-4.551793	5.32E-06	0.000412546
HNRNPC	0.287932	0.063367	4.54384	5.52E-06	0.000425593
GPATCH4	0.374344	0.08243	4.541354	5.59E-06	0.000427811
SESN3	-0.407624	0.089869	-4.53575	5.74E-06	0.000436459
RRBP1	0.832714	0.184269	4.519019	6.21E-06	0.00046934
TNK2	0.570775	0.126639	4.507083	6.57E-06	0.00049332
SDHB	0.343294	0.076259	4.501677	6.74E-06	0.000502794
CASP7	0.771851	0.171753	4.493965	6.99E-06	0.000518039
CTTNBP2NL	-1.173194	0.261438	-4.487471	7.21E-06	0.0005307
NUDC	0.300606	0.067227	4.471489	7.77E-06	0.000566496
PDIA6	0.489923	0.109582	4.470851	7.79E-06	0.000566496
PPP3CA	-0.34058	0.076286	-4.464524	8.02E-06	0.00057987
CNIH1	0.284805	0.063866	4.459432	8.22E-06	0.000590155
CD3EAP	0.666194	0.149499	4.456174	8.34E-06	0.000595513
RHOF	-0.442465	0.099649	-4.440229	8.99E-06	0.000637479
RRP15	0.362041	0.081669	4.433008	9.29E-06	0.000655222
ATP6V1E1	0.477884	0.107972	4.426	9.60E-06	0.000672786
ECE2	0.695423	0.157247	4.422485	9.76E-06	0.000679733
ISG20	-0.582019	0.131707	-4.419034	9.91E-06	0.000686564
CMSS1	0.410368	0.093348	4.396128	1.10E-05	0.000758614
ABCB1	1.131539	0.258384	4.379292	1.19E-05	0.000814829
PARP14	-0.405808	0.092717	-4.376848	1.20E-05	0.00081569
STK17B	-0.440568	0.100666	-4.376512	1.21E-05	0.00081569
GTPBP4	0.419059	0.095848	4.372133	1.23E-05	0.000827417
MRPS23	0.320303	0.073297	4.369925	1.24E-05	0.000831025
BCL2A1	0.651997	0.149543	4.359925	1.30E-05	0.000863889
GADD45GIP1	0.239598	0.054967	4.358947	1.31E-05	0.000863889
GBP4	0.711645	0.163866	4.342835	1.41E-05	0.000924512
DBNL	0.289311	0.066726	4.335784	1.45E-05	0.000949292
RBM47	1.336572	0.308361	4.334443	1.46E-05	0.000949488
C1orf216	0.699424	0.161424	4.33283	1.47E-05	0.000949488
PCSK7	-0.300184	0.069294	-4.332061	1.48E-05	0.000949488
GTF2E2	0.71023	0.164594	4.315035	1.60E-05	0.001020067
SRA1	0.399225	0.092965	4.294369	1.75E-05	0.001113755
SH3BGRL	-0.283871	0.066439	-4.272645	1.93E-05	0.001221364
PLXNA3	-0.699769	0.164314	-4.258735	2.06E-05	0.001292864
S100A4	-0.880345	0.207318	-4.246351	2.17E-05	0.001359051

LINC02201	-1.407308	0.332385	-4.23397	2.30E-05	0.001426605
MRTO4	0.445647	0.105278	4.23306	2.31E-05	0.001426605
KEAP1	0.491637	0.116595	4.216607	2.48E-05	0.001526611
NDUFAF3	0.291133	0.069148	4.210298	2.55E-05	0.001561617
GPR18	0.894958	0.212652	4.20855	2.57E-05	0.001565501
MYDGF	0.339681	0.080835	4.202173	2.64E-05	0.00159701
BHLHE40	0.535997	0.127567	4.201687	2.65E-05	0.00159701
NCL	0.279604	0.06685	4.182567	2.88E-05	0.001727754
SNRPD1	0.303879	0.072672	4.1815	2.90E-05	0.001727754
PDIA3	0.332568	0.079575	4.179301	2.92E-05	0.001735639
HP1BP3	-0.29031	0.069524	-4.175695	2.97E-05	0.001754415
C16orf54	0.549073	0.131577	4.173026	3.01E-05	0.001766142
APOL3	0.712473	0.171088	4.164378	3.12E-05	0.001825204
BMF	-0.59357	0.142906	-4.153569	3.27E-05	0.001904074
EBNA1BP2	0.431533	0.104057	4.147081	3.37E-05	0.00194908
MRPL3	0.383901	0.092758	4.138754	3.49E-05	0.00201117
HSPA9	0.341835	0.082633	4.136782	3.52E-05	0.002016798
SNRPD3	0.29769	0.071978	4.135851	3.54E-05	0.002016798
ATP13A2	0.918689	0.22377	4.105498	4.03E-05	0.002289595
SH3BGRL3	-0.371061	0.09063	-4.094221	4.24E-05	0.002392259
DCAF13	0.410603	0.100324	4.092778	4.26E-05	0.002395572
SYPL1	-0.39545	0.096772	-4.086396	4.38E-05	0.002450555
C15orf39	0.761643	0.186653	4.08053	4.49E-05	0.002501211
ADAMTS7	-1.048672	0.257116	-4.078597	4.53E-05	0.002510081
GNG11	-1.197513	0.293817	-4.075705	4.59E-05	0.002529436
ODF2L	-0.492994	0.121078	-4.071714	4.67E-05	0.002561041
ENO1	0.404024	0.099534	4.059154	4.93E-05	0.002690071
ACAP2	-0.436523	0.107658	-4.054713	5.02E-05	0.002728878
MCL1	0.350411	0.08653	4.049583	5.13E-05	0.002776408
SPOCK2	0.685469	0.169785	4.037271	5.41E-05	0.00291263
NDC1	0.681892	0.169431	4.024602	5.71E-05	0.003059789
CNP	0.495727	0.123621	4.010039	6.07E-05	0.003239841
FCRL1	0.878203	0.219201	4.006385	6.17E-05	0.003275317
WNT10A	-1.177939	0.294361	-4.001685	6.29E-05	0.003325893
EPHB6	-0.803806	0.200991	-3.999219	6.36E-05	0.003333582
GPR146	-1.471711	0.36802	-3.998997	6.36E-05	0.003333582
KDM2B	0.384589	0.096245	3.995947	6.44E-05	0.003361664
WDFY4	-0.489932	0.122664	-3.994084	6.49E-05	0.003373071
NHP2	0.269661	0.067855	3.974084	7.07E-05	0.003643345
SYNE3	-0.996242	0.250711	-3.973665	7.08E-05	0.003643345
FAM111B	-0.87226	0.219604	-3.971971	7.13E-05	0.003653195
TRAF3IP3	-0.313002	0.078953	-3.964429	7.36E-05	0.003750764
HOXB3	2.612695	0.659173	3.963597	7.38E-05	0.003750764
SH2D4A	1.931003	0.488103	3.956139	7.62E-05	0.003852913
CYTIP	-0.462805	0.117142	-3.950788	7.79E-05	0.003923035
DHRS1	-0.547629	0.138747	-3.946963	7.91E-05	0.003969046
ZNF506	0.643259	0.163414	3.936383	8.27E-05	0.004113909

MDM4	-0.334546	0.08499	-3.936307	8.27E-05	0.004113909
HPS3	-0.653865	0.166681	-3.922858	8.75E-05	0.004332043
NOC2L	0.347449	0.088666	3.918646	8.90E-05	0.004389755
ACSL1	0.635529	0.162426	3.912728	9.13E-05	0.004479786
SYTL1	-0.470599	0.120309	-3.911575	9.17E-05	0.004482331
CYB561A3	-0.486816	0.12453	-3.909231	9.26E-05	0.004507092
ENSG00000176320	3.56342	0.91365	3.900201	9.61E-05	NA
IZUMO4	-0.559301	0.143534	-3.896632	9.75E-05	0.004728225
SLC45A3	1.164326	0.299015	3.893872	9.87E-05	0.004762546
GPER1	-2.971763	0.76434	-3.888011	0.00010107	NA
PA2G4	0.250129	0.064365	3.886131	0.00010186	0.004896602
ARL4C	-0.840583	0.216585	-3.88108	0.00010399	0.004974851
CHST2	0.938527	0.241871	3.880277	0.00010434	0.004974851
CCDC124	0.371412	0.095763	3.878457	0.00010512	0.004991757
UBTF	0.449254	0.1161	3.869527	0.00010905	0.005157113
AIMP2	0.554656	0.143411	3.867604	0.00010991	0.005165184
PRDM2	-0.404278	0.104541	-3.867171	0.00011011	0.005165184
EIF6	0.262746	0.06802	3.862787	0.0001121	0.005237665
DENND3	-0.684539	0.177407	-3.85859	0.00011404	0.005307112
PEMT	0.666695	0.173369	3.845514	0.0001203	0.005575981
KLHL14	-0.881591	0.229314	-3.844465	0.00012082	0.005577657
TFAM	0.406504	0.105879	3.839341	0.00012337	0.005665483
FKBP4	0.372836	0.097133	3.838419	0.00012383	0.005665483
LY86	-0.574794	0.149775	-3.837726	0.00012418	0.005665483
CMIP	-0.530022	0.138238	-3.834132	0.00012601	0.00567019
COMMD6	-0.214734	0.056014	-3.833608	0.00012628	0.00567019
LPIN1	-0.507127	0.132298	-3.833219	0.00012648	0.00567019
RAD23A	0.186958	0.048782	3.832502	0.00012685	0.00567019
SRPRB	0.441734	0.115271	3.832144	0.00012703	0.00567019
MICOS10	0.275166	0.071812	3.831756	0.00012723	0.00567019
BRIX1	0.346183	0.090367	3.830865	0.00012769	0.00567019
TNRC6B	-0.276066	0.072136	-3.82704	0.00012969	0.005737074
RPN1	0.412912	0.107962	3.824606	0.00013098	0.005772104
LONP1	0.536267	0.140284	3.822738	0.00013198	0.005794079
GZMB	3.109875	0.815892	3.811626	0.00013806	0.006038118
ARID5B	-0.390977	0.102707	-3.806724	0.00014082	0.006125575
SIGLEC10	1.109806	0.291577	3.806217	0.00014111	0.006125575
KMO	-0.686663	0.181087	-3.791906	0.0001495	0.006441846
TMEM65	1.069985	0.282177	3.791896	0.0001495	0.006441846
NUBP1	0.396187	0.104682	3.784681	0.00015391	0.006607152
FBP1	1.835716	0.485759	3.779065	0.00015742	0.006733118
CLCF1	-0.599467	0.15872	-3.776872	0.00015881	0.006744684
BOP1	0.506693	0.134159	3.776812	0.00015885	0.006744684
SLCO5A1	1.080737	0.286263	3.775322	0.0001598	0.006760445
GPR160	0.781225	0.207596	3.7632	0.00016775	0.00707115
GRWD1	0.431138	0.114678	3.759549	0.00017022	0.007127817
CTSZ	0.694976	0.184864	3.759398	0.00017032	0.007127817

ZNF318	-0.587752	0.156776	-3.749003	0.00017754	0.007403194
RUNX2	1.892945	0.505429	3.745221	0.00018024	0.00748877
DMD	-0.885594	0.236829	-3.739386	0.00018447	0.007637456
CD6	-1.357675	0.363418	-3.735851	0.00018708	0.007718098
PKIG	-0.503393	0.135018	-3.728331	0.00019275	0.007923935
PRMT1	0.325425	0.087629	3.713692	0.00020426	0.008367357
DPEP3	3.160486	0.851123	3.713313	0.00020456	NA
IRAK2	1.055162	0.284227	3.712387	0.00020531	0.008381131
SLA	0.759604	0.204725	3.710357	0.00020697	0.008419062
ANKRD12	-0.273394	0.073735	-3.707793	0.00020907	0.008475129
PSAT1	0.834133	0.225622	3.697036	0.00021813	0.008811596
RAN	0.299508	0.081123	3.692033	0.00022247	0.008955696
HIVEP3	0.776831	0.210819	3.684828	0.00022886	0.009181106
CCM2	-0.272539	0.074009	-3.682529	0.00023093	0.009232517
SDF2L1	0.451472	0.122765	3.677534	0.0002355	0.009382888
DGAT2	0.660434	0.179712	3.674964	0.00023788	0.009445479
CCNH	0.484658	0.132164	3.667096	0.00024532	0.009707692
PSMB10	0.33045	0.090277	3.6604	0.00025182	0.009931169
TOP1	0.258836	0.070789	3.65643	0.00025575	0.010052089
ADRB2	-1.673351	0.4579	-3.654402	0.00025778	0.010097778
CCT5	0.282183	0.077355	3.647889	0.0002644	0.010322406
NXT1	0.396302	0.109009	3.63549	0.00027745	0.010795621
KIAA1109	-0.550207	0.151642	-3.628326	0.00028527	0.011062593
ACADVL	-0.277586	0.07661	-3.623369	0.00029079	0.011194402
NOP10	0.212292	0.058592	3.623197	0.00029099	0.011194402
TIMM17A	0.414431	0.114398	3.622694	0.00029155	0.011194402
DNAJC4	-0.289542	0.080055	-3.616788	0.00029828	0.011380195
HSBP1	0.255613	0.070675	3.616732	0.00029835	0.011380195
ENSG00000235609	1.963795	0.54366	3.612171	0.00030364	0.011544442
ZNF302	-0.328726	0.091151	-3.606404	0.00031047	0.011765492
PTPRE	-0.686644	0.190452	-3.605335	0.00031175	0.011775687
ENSG00000271857	2.024885	0.56234	3.600817	0.00031722	0.011943453
TOP2B	0.367658	0.10215	3.599181	0.00031922	0.011980065
SAMD9L	-0.5931	0.165097	-3.592437	0.0003276	0.012254967
DNAJB2	-0.46749	0.130379	-3.585611	0.00033629	0.012531642
EIF4A1	0.263482	0.073497	3.584944	0.00033715	0.012531642
COA7	0.50387	0.140599	3.583731	0.00033872	0.012549955
NAPSA	2.028539	0.567991	3.571429	0.00035504	0.013112772
LTA4H	-0.396388	0.111046	-3.569595	0.00035753	0.01311367
QARS	-0.248389	0.069589	-3.569382	0.00035782	0.01311367
PLK3	0.714842	0.200296	3.568928	0.00035845	0.01311367
TIMP1	-0.885954	0.248719	-3.562072	0.00036794	0.013418831
TCF3	-0.350119	0.098523	-3.553686	0.00037987	0.013810738
LDLRAD4	0.485942	0.136954	3.548215	0.00038785	0.014051622
LZIC	0.497091	0.140125	3.547495	0.00038891	0.014051622
RPF1	0.360472	0.101652	3.546119	0.00039095	0.014081456
DAP	0.551976	0.155792	3.543017	0.00039558	0.01419901

SNX18	0.760686	0.214743	3.5423	0.00039665	0.01419901
BCAR3	0.832837	0.235428	3.537548	0.00040386	0.014412605
MIF	0.353164	0.099895	3.535342	0.00040725	0.014489037
RPL10	-0.199873	0.056597	-3.531501	0.00041321	0.014656294
PPCDC	0.82614	0.234017	3.530254	0.00041516	0.014657224
MTSS1	-0.574013	0.162616	-3.529876	0.00041575	0.014657224
PRPF19	0.471438	0.133666	3.52698	0.00042033	0.01477372
EXOSC4	0.303182	0.086005	3.525155	0.00042324	0.014807633
ZFP36	-0.267399	0.075865	-3.524665	0.00042402	0.014807633
SMARCC1	0.317889	0.090222	3.523403	0.00042604	0.014807633
DIMT1	0.377055	0.107021	3.523191	0.00042638	0.014807633
SFXN4	0.48824	0.138773	3.518268	0.00043437	0.014990722
ZC3H15	0.218697	0.062163	3.518115	0.00043462	0.014990722
CKS2	0.397415	0.11298	3.517567	0.00043552	0.014990722
SNU13	0.216339	0.061545	3.515161	0.00043949	0.015082587
IFRD2	0.321526	0.091583	3.510751	0.00044684	0.015289879
APRT	0.192041	0.054763	3.506732	0.00045365	0.015444995
OAS3	1.130069	0.322342	3.505808	0.00045522	0.015444995
TENT5C	-0.710566	0.202687	-3.505729	0.00045536	0.015444995
EEF1E1	0.325317	0.093002	3.497956	0.00046884	0.015855982
HADHA	-0.269795	0.077276	-3.491333	0.00048062	0.016207236
GLRX3	0.251373	0.072042	3.489271	0.00048434	0.016228586
FCHO1	0.662066	0.189789	3.488437	0.00048585	0.016228586
CHCHD10	1.630956	0.467577	3.488103	0.00048646	0.016228586
EEF1A1	-0.252822	0.072486	-3.487853	0.00048692	0.016228586
CARD11	0.534835	0.153391	3.486742	0.00048894	0.016228586
GLYCTK	0.719994	0.206522	3.486282	0.00048978	0.016228586
ENSG00000259436	-0.958862	0.275092	-3.485611	0.00049101	0.016228586
EIF3J	0.248973	0.071498	3.482255	0.00049721	0.016386812
JUNB	0.398788	0.114692	3.477021	0.00050702	0.016662852
NIFK	0.25086	0.072195	3.474742	0.00051134	0.016725596
FXYD5	-0.46507	0.133865	-3.474171	0.00051244	0.016725596
IMP4	0.251901	0.072533	3.472933	0.0005148	0.016725596
NDUFS6	0.255198	0.073509	3.47164	0.00051729	0.016725596
TWISTNB	0.429879	0.12383	3.471518	0.00051752	0.016725596
FAM207A	0.367084	0.105742	3.471503	0.00051755	0.016725596
SH3TC1	-0.620585	0.178892	-3.469055	0.00052229	0.016799905
CKB	-0.923647	0.266271	-3.468825	0.00052274	0.016799905
EEF2KMT	0.410885	0.118772	3.459426	0.00054133	0.017339623
PWWP3A	-0.392919	0.113599	-3.458836	0.00054252	0.017339623
DAPP1	-0.414815	0.12024	-3.449908	0.00056078	0.017874193
NDUFAF4	0.366572	0.106307	3.448244	0.00056425	0.017935581
HSPD1	0.322189	0.09358	3.442921	0.00057547	0.018211773
MORC3	-0.468611	0.136135	-3.442249	0.0005769	0.018211773
POLR3GL	-0.266113	0.077315	-3.441907	0.00057763	0.018211773
TIAM1	1.826434	0.530902	3.440248	0.00058118	0.018236769
SNRPE	0.217091	0.063107	3.440073	0.00058156	0.018236769

C16orf72	0.389305	0.113237	3.437953	0.00058613	0.018330702
MFSD10	-0.311295	0.090588	-3.436379	0.00058955	0.018388105
CPNE3	-0.342092	0.099688	-3.431631	0.00059996	0.018654173
TGM2	1.890574	0.551021	3.431035	0.00060128	0.018654173
SLC38A5	0.572272	0.166931	3.428196	0.00060761	0.018800287
PAICS	0.329905	0.096454	3.420324	0.00062547	0.01930154
TMEM147	0.233822	0.068429	3.416991	0.00063317	0.019487661
TOMM5	0.35156	0.103006	3.412991	0.00064254	0.019723821
HSP90B1	0.327215	0.095912	3.411628	0.00064576	0.019753599
CALR	0.277494	0.081349	3.411145	0.00064691	0.019753599
PARK7	0.203757	0.059797	3.407487	0.00065564	0.019922647
SC5D	-0.418216	0.12274	-3.407323	0.00065604	0.019922647
C8orf58	-0.795232	0.233463	-3.406237	0.00065865	0.019922647
LARGE2	-0.652787	0.191688	-3.405467	0.00066051	0.019922647
PDHA1	0.374057	0.109847	3.405262	0.00066101	0.019922647
ATXN10	0.34376	0.101083	3.400781	0.00067194	0.020197238
CD99	-0.520428	0.153072	-3.399898	0.00067411	0.020197238
GDPGP1	0.886009	0.260637	3.399406	0.00067533	0.020197238
EIF4ENIF1	0.683243	0.201096	3.397589	0.00067983	0.020279719
MYO1C	-0.423635	0.124715	-3.396823	0.00068173	0.020284543
PTPN7	0.666959	0.196769	3.389561	0.00070005	0.02075045
MRPS12	0.261362	0.077132	3.388516	0.00070272	0.02075045
VAMP2	-0.301367	0.08896	-3.387687	0.00070485	0.02075045
KMT2E	-0.265297	0.078315	-3.38754	0.00070523	0.02075045
COA4	0.254222	0.075056	3.387119	0.00070631	0.02075045
TBC1D8	1.225927	0.362352	3.38325	0.00071633	0.020992029
NFKBIZ	0.434827	0.128638	3.380235	0.00072424	0.021170322
COX5A	0.225737	0.066811	3.37873	0.00072821	0.021233177
RSRP1	-0.336694	0.099826	-3.372793	0.0007441	0.021642088
FCER2	1.405165	0.416727	3.371907	0.0007465	0.021657745
FAM129C	-0.552377	0.164054	-3.367039	0.0007598	0.021945937
PFDN6	0.297386	0.088327	3.366892	0.0007602	0.021945937
PSMD8	0.239311	0.071122	3.364783	0.00076604	0.022041867
CCNG1	-0.334817	0.09952	-3.364323	0.00076732	0.022041867
ZNF44	0.460303	0.136943	3.361273	0.00077584	0.022231879
GLRX2	0.379486	0.112936	3.360176	0.00077893	0.022265463
SMIM20	0.463118	0.137901	3.358342	0.00078412	0.022358814
DHRS3	1.360827	0.405302	3.357567	0.00078632	0.022366745
SPCS2	0.206078	0.061452	3.353456	0.00079809	0.022619594
HIVEP2	0.785279	0.234194	3.353109	0.00079909	0.022619594
TIMMDC1	0.369367	0.110271	3.349626	0.00080921	0.02285025
RPL13A	-0.176335	0.052673	-3.347726	0.00081478	0.022951843
TRANK1	-0.557649	0.166703	-3.34517	0.00082232	0.023108401
LMF2	-0.392408	0.117516	-3.339183	0.00084025	0.023555377
ULK1	0.812984	0.243637	3.336865	0.00084729	0.023695626
IQGAP1	-0.330345	0.099026	-3.33593	0.00085015	0.023718487
EIF3H	-0.201209	0.060332	-3.335056	0.00085282	0.02373623

CERS4	-0.373841	0.112242	-3.330686	0.00086632	0.024038068
SERBP1	0.243561	0.073137	3.330212	0.0008678	0.024038068
CD320	0.355459	0.106796	3.3284	0.00087346	0.024137459
FAM30A	-0.355616	0.106866	-3.32769	0.00087569	0.024141697
ZNRD2	0.299934	0.090192	3.325508	0.00088258	0.024265751
BTG2	-0.305311	0.091824	-3.324944	0.00088436	0.024265751
TESK1	0.528849	0.159154	3.322876	0.00089095	0.024388827
SPHK1	0.825919	0.248616	3.322073	0.00089351	0.024401709
PRMT2	-0.414689	0.12493	-3.319365	0.00090222	0.024581883
ENSG00000259865	-0.489144	0.147457	-3.317196	0.00090926	0.024715697
SQSTM1	0.3016	0.090989	3.31469	0.00091745	0.024857175
CAPG	-0.654324	0.197435	-3.314121	0.00091932	0.024857175
KCNN1	1.59488	0.481379	3.313146	0.00092253	0.024857175
TBL3	0.390184	0.117782	3.312756	0.00092382	0.024857175
FKBP11	0.354556	0.107041	3.312353	0.00092515	0.024857175
EHD1	0.274836	0.083015	3.310678	0.0009307	0.024948785
UGP2	-0.30751	0.093178	-3.300232	0.00096605	0.025836817
MAP4K2	-0.423762	0.128634	-3.294333	0.00098655	0.026324695
ABCF2	0.33976	0.10333	3.288112	0.00100862	0.026851839
TBC1D10C	-0.307324	0.093605	-3.28319	0.0010264	0.027180749
IDS	-0.274142	0.083505	-3.282923	0.00102737	0.027180749
SLIRP	0.302241	0.092069	3.282755	0.00102798	0.027180749
GRPEL1	0.329147	0.100404	3.278214	0.00104466	0.027559141
DCTPP1	0.323785	0.098905	3.273683	0.00106156	0.027832687
NTHL1	0.426747	0.130366	3.273445	0.00106245	0.027832687
CCDC90B	-0.254322	0.077696	-3.273309	0.00106296	0.027832687
RPS27	-0.171496	0.052399	-3.272874	0.0010646	0.027832687
EIF3B	0.327927	0.100428	3.2653	0.00109348	0.028444197
TARS	0.308748	0.094566	3.26489	0.00109507	0.028444197
SLCO4A1	0.531094	0.162672	3.264823	0.00109532	0.028444197
TRIM25	0.74044	0.227036	3.261327	0.00110892	0.028718964
ZNF581	-0.314374	0.096409	-3.260836	0.00111084	0.028718964
BCL6	-0.775876	0.238215	-3.257044	0.00112579	0.029040882
FAM3C	0.345163	0.106013	3.25585	0.00113054	0.029098793
SRSF8	0.259103	0.079617	3.254374	0.00113643	0.029183217
RRS1	0.434724	0.133606	3.253773	0.00113883	0.029183217
CNPY2	0.206808	0.06361	3.251202	0.00114918	0.029383677
EEF1D	-0.238507	0.073649	-3.238447	0.00120182	0.030662308
POLR2H	0.301919	0.0933	3.235985	0.00121224	0.030860379
MRPL13	0.384859	0.119152	3.229988	0.00123796	0.031446224
ZNF524	-0.353467	0.109646	-3.223698	0.00126547	0.032075105
MMP11	1.022476	0.3173	3.222429	0.00127109	0.03214739
HDLBP	0.299192	0.092983	3.21771	0.00129219	0.03261018
SVBP	-0.250583	0.077951	-3.214617	0.00130619	0.032892163
PRR34.AS1	-0.362624	0.112833	-3.213805	0.00130989	0.032914106
PSMD11	0.268683	0.083747	3.208263	0.00133539	0.033482661
BTG3	0.445246	0.138893	3.205674	0.00134747	0.033712728

DPH2	0.346664	0.108204	3.2038	0.00135627	0.033860115
FBXL17	-0.676075	0.211525	-3.196186	0.00139258	0.034674634
LINC00996	1.35579	0.424252	3.195714	0.00139485	0.034674634
RFK	0.407971	0.127835	3.191399	0.00141585	0.035121637
GPR68	-2.694012	0.846761	-3.181551	0.00146489	NA
TUBB2A	0.898078	0.282362	3.180592	0.00146975	0.036377624
GAK	0.37848	0.119019	3.180002	0.00147274	0.036377624
SPINT2	-0.327543	0.10305	-3.178476	0.00148051	0.036492143
NDUFAF8	0.240859	0.075795	3.177779	0.00148408	0.036502694
CISD1	0.309074	0.097322	3.175795	0.00149427	0.036675702
RECQL	-0.369277	0.116305	-3.175085	0.00149793	0.036688148
CCDC86	0.415738	0.131166	3.169563	0.00152669	0.037313978
ETFB	0.333363	0.105227	3.168032	0.00153475	0.037432388
RHOG	-0.252224	0.079661	-3.166208	0.0015444	0.037560464
HDAC9	0.284674	0.089921	3.165822	0.00154646	0.037560464
ALG3	0.333038	0.10523	3.164866	0.00155154	0.03760555
AP1G2	-0.305062	0.096533	-3.160177	0.00157673	0.038084655
EEF2	-0.216315	0.068455	-3.159969	0.00157786	0.038084655
CCR7	0.310339	0.098243	3.158878	0.00158378	0.038148414
FER	-0.697253	0.220926	-3.156053	0.0015992	0.038440289
CCNT2	-0.436783	0.138506	-3.153521	0.00161314	0.038695305
WASF2	0.226783	0.07197	3.151052	0.00162684	0.038927928
NOP14	0.352428	0.111862	3.150569	0.00162953	0.038927928
MFNG	0.330556	0.104992	3.148391	0.00164172	0.039017206
DUSP18	-0.602719	0.191468	-3.14789	0.00164454	0.039017206
PFDN2	0.259425	0.082416	3.147737	0.0016454	0.039017206
VIM.AS1	-0.630162	0.200237	-3.147086	0.00164906	0.039017206
NTAN1	-0.299439	0.095153	-3.146915	0.00165003	0.039017206
TMED9	0.245654	0.078083	3.146049	0.00165492	0.039053435
H2AFY	0.244047	0.077714	3.140333	0.00168756	0.03974301
ZFP90	-0.586822	0.186989	-3.138269	0.00169949	0.039943165
RSL24D1	0.215323	0.068675	3.135374	0.00171635	0.040258138
ITGB7	0.688342	0.219643	3.13392	0.00172488	0.040376729
DDIT4	0.699295	0.223387	3.130418	0.00174558	0.040779267
DCTN2	-0.283052	0.090451	-3.12933	0.00175205	0.040848459
ANKRD36B	-0.644075	0.205914	-3.12788	0.00176072	0.040968499
FXYD1	-0.894094	0.286114	-3.124959	0.0017783	0.041292708
EIF3F	-0.193173	0.061827	-3.124388	0.00178176	0.041292708
CLIP4	1.232283	0.394826	3.121075	0.00180192	0.041676984
ENSG00000275418	-2.655102	0.851238	-3.119106	0.001814	NA
RUBCN	-0.449059	0.143986	-3.118762	0.00181613	0.041912786
EIF4A3	0.523364	0.167839	3.118244	0.00181932	0.041912786
RPS28	-0.101494	0.032608	-3.11259	0.00185454	0.042639679
MARCKSL1	-0.441606	0.141989	-3.110133	0.00187003	0.042911098
GALM	0.761698	0.245106	3.107634	0.00188591	0.043104877
ZNF638	-0.321608	0.1035	-3.107324	0.0018879	0.043104877
ENSG00000111540	-0.315168	0.101436	-3.107059	0.00188959	0.043104877

CARD8	-0.348885	0.112383	-3.104423	0.00190651	0.04340567
TMEM134	-0.213555	0.068819	-3.103136	0.00191482	0.043509746
ACACA	0.56745	0.183303	3.0957	0.00196349	0.044457775
POMP	0.22286	0.072003	3.095163	0.00196705	0.044457775
USF3	-0.494874	0.159894	-3.095019	0.001968	0.044457775
TIMM23	0.373916	0.120858	3.093844	0.00197582	0.044547761
FCMR	0.468744	0.151604	3.091889	0.00198887	0.044755406
SLC39A14	0.635805	0.205915	3.087702	0.00201711	0.045228313
EEF1AKNMT	0.502654	0.162797	3.08762	0.00201766	0.045228313
PSMD7	0.261507	0.084775	3.084701	0.00203757	0.045501181
STIP1	0.401724	0.130239	3.08451	0.00203887	0.045501181
ENSG00000089127	0.847796	0.274902	3.083999	0.00204239	0.045501181
PBXIP1	-0.588174	0.190746	-3.083548	0.00204548	0.045501181
TUFM	0.207071	0.067243	3.079464	0.00207374	0.046041709
CTSB	0.333205	0.10828	3.077254	0.00208917	0.046296091
RUVBL1	0.375638	0.122111	3.076196	0.0020966	0.04637234
ZNF652	-0.445459	0.145166	-3.068616	0.00215053	0.047408132
PINX1	0.397489	0.12954	3.06847	0.00215158	0.047408132
BICDL1	0.896475	0.29224	3.067605	0.00215782	0.047455721
NCKAP1L	-0.269179	0.087826	-3.064894	0.00217747	0.04767713
RPL41	-0.156314	0.051002	-3.064847	0.00217782	0.04767713
SEPTIN6	-0.19496	0.063618	-3.064522	0.00218018	0.04767713
RNPS1	0.18821	0.061459	3.062351	0.00219606	0.047934156
ARL17B	-0.631081	0.206172	-3.06095	0.00220636	0.048068903
CYBC1	-0.250991	0.082038	-3.059446	0.00221746	0.04818389
SESTD1	-0.700181	0.228883	-3.059115	0.00221992	0.04818389
SFXN3	-0.838324	0.274707	-3.051707	0.00227544	0.049297017
NNT	-0.46397	0.152304	-3.046339	0.00231647	0.050058195
ADSL	0.251158	0.082455	3.045987	0.00231918	0.050058195
FOXO3	0.491482	0.161425	3.044648	0.00232953	0.05018845
TTC3	-0.227358	0.074713	-3.043087	0.00234165	0.05035625
DNAH1	1.165388	0.383159	3.041525	0.00235383	0.05052478
CRTC3	-0.349659	0.115102	-3.037806	0.00238308	0.051058393
AHRR	-1.175932	0.387386	-3.035556	0.00240093	0.051287797
MYADM	0.640079	0.210895	3.035065	0.00240484	0.051287797
RPS27A	-0.141766	0.046713	-3.034793	0.00240701	0.051287797
SERPINE2	0.886072	0.292109	3.033363	0.00241844	0.051437245
TEX264	0.332193	0.109576	3.03162	0.00243246	0.051618633
TSFM	0.417804	0.137835	3.031199	0.00243585	0.051618633
HERC4	-0.34896	0.115212	-3.028843	0.00245492	0.051928246
SLC25A6	-0.19448	0.06432	-3.023627	0.00249764	0.052736015
RPL27A	-0.130517	0.043184	-3.022333	0.00250834	0.052830796
PPIL1	0.371309	0.122869	3.021987	0.00251121	0.052830796
SERPINB9P1	-0.886554	0.293587	-3.019733	0.00252998	0.05312948
CLIC3	1.82448	0.604696	3.017186	0.00255133	0.053481445
ENSG00000237188	-0.936266	0.310565	-3.014721	0.00257216	0.053821071
TIMM13	0.265785	0.088237	3.012158	0.00259397	0.054180046

TMBIM1	-0.382994	0.127208	-3.010773	0.00260583	0.054330242
SDE2	0.550964	0.183044	3.010008	0.00261241	0.05433937
TBC1D9	0.776526	0.258023	3.009525	0.00261656	0.05433937
CYCS	0.320198	0.10641	3.009094	0.00262028	0.05433937
CD47	-0.30747	0.102208	-3.008285	0.00262726	0.054387179
SINHCAF	0.241392	0.080318	3.005462	0.00265178	0.054797114
RABEPK	0.373907	0.12449	3.003517	0.00266878	0.055050751
PIKFYVE	0.425275	0.141703	3.00117	0.00268945	0.055294812
LBR	0.284303	0.094733	3.001093	0.00269012	0.055294812
CASP4	-0.249975	0.083356	-2.998897	0.00270959	0.055596812
SSBP2	-0.675545	0.22559	-2.99457	0.00274832	0.056276126
AP2B1	-0.240485	0.080319	-2.99412	0.00275237	0.056276126
BCCIP	0.286517	0.095747	2.992442	0.00276756	0.056452752
EIF5B	0.18811	0.062869	2.992093	0.00277072	0.056452752
EBI3	0.571534	0.191062	2.991356	0.00277742	0.056470812
RPS6KA2	-0.888935	0.297216	-2.990873	0.00278181	0.056470812
PIP5K1B	0.587975	0.196653	2.989914	0.00279056	0.056470812
LYRM9	-0.63879	0.213652	-2.989864	0.00279102	0.056470812
PNO1	0.397041	0.132888	2.987776	0.00281015	0.056759189
MICAL1	-0.391573	0.131167	-2.985315	0.00283287	0.057118881
LGMN	0.780053	0.261415	2.98396	0.00284544	0.057273057
PQLC3	-0.529184	0.177409	-2.982854	0.00285574	0.057381153
ANKRD36C	-0.454421	0.152731	-2.975295	0.00292707	0.058682841
DDX49	0.301944	0.101507	2.97462	0.00293351	0.058682841
ANAPC16	0.188552	0.063392	2.974396	0.00293566	0.058682841
HSP90AB1	0.154059	0.051813	2.973351	0.00294567	0.058724003
GPR155	-0.987262	0.332062	-2.973129	0.00294781	0.058724003
MRPS36	0.186292	0.062681	2.972051	0.00295817	0.058829693
APOBEC3C	-0.328183	0.110525	-2.969302	0.00298477	0.059257388
PAOX	0.581625	0.196169	2.964915	0.00302766	0.060006491
SEL1L3	0.655895	0.221374	2.962837	0.00304818	0.060254075
RACK1	-0.150947	0.050951	-2.962602	0.00305051	0.060254075
ZNF461	0.796171	0.268972	2.960054	0.00307585	0.060651552
OXT	-0.903701	0.3057	-2.956168	0.00311488	0.061317208
NOP58	0.199023	0.067348	2.955132	0.00312535	0.061419423
MPP6	0.495412	0.167692	2.954291	0.00313389	0.061472595
ENSG00000277511	-0.875625	0.296438	-2.953825	0.00313862	0.061472595
HEXD	-0.570147	0.193383	-2.948277	0.00319551	0.062481538
PSMA5	0.282289	0.095815	2.946181	0.00321724	0.062593568
ESYT1	-0.35008	0.118837	-2.945892	0.00322025	0.062593568
C11orf80	-0.670199	0.227518	-2.945703	0.00322222	0.062593568
SNRNP40	0.326758	0.110929	2.945651	0.00322276	0.062593568
RHOA	0.199938	0.067984	2.940967	0.00327189	0.063442032
METAP2	0.192238	0.065438	2.937686	0.00330672	0.064010561
ITGA4	0.449624	0.153141	2.936002	0.00332472	0.064231376
FAM216A	0.397064	0.135282	2.935089	0.00333453	0.064231376
PSMD4	0.227422	0.077484	2.935073	0.00333469	0.064231376

EMC4	0.265016	0.090341	2.933489	0.00335175	0.064347416
RPL7L1	0.229554	0.078253	2.933487	0.00335177	0.064347416
SLC44A1	-0.596076	0.203388	-2.930738	0.00338158	0.06481274
PPP1CC	0.210057	0.071735	2.928236	0.00340891	0.065229103
C7orf50	-0.43475	0.148728	-2.92312	0.00346543	0.066201662
CTPS1	0.466837	0.159803	2.921335	0.00348535	0.066385012
SNRPB	0.285087	0.097591	2.921237	0.00348644	0.066385012
EXOC3	-0.463387	0.159072	-2.913072	0.00357892	0.068034489
TPI1	0.217127	0.074627	2.909499	0.00362009	0.06870487
POLR3D	0.435743	0.150065	2.903688	0.00368796	0.069879023
TTC7A	-0.529696	0.182456	-2.903148	0.00369432	0.069885659
PLD2	-0.766767	0.264234	-2.901843	0.00370975	0.070063657
MTFP1	0.316626	0.109139	2.901132	0.00371818	0.070079357
DCUN1D5	0.333033	0.114809	2.900757	0.00372263	0.070079357
ATAD3B	0.393147	0.135651	2.898216	0.00375292	0.070436211
HINT2	0.258016	0.089031	2.89803	0.00375515	0.070436211
PHB	0.26725	0.092239	2.897362	0.00376315	0.070436211
PLEC	-0.772642	0.266707	-2.89697	0.00376786	0.070436211
CD24	-0.261193	0.090171	-2.896637	0.00377186	0.070436211
TSPAN17	-0.360556	0.124519	-2.895596	0.00378439	0.0705171
HTR3A	1.361698	0.470318	2.895271	0.00378831	0.0705171
PLAC8	-0.240701	0.083196	-2.893189	0.00381352	0.070872991
GNL3	0.318284	0.110234	2.887344	0.00388509	0.072001508
SEPTIN1	-0.234725	0.081298	-2.88722	0.00388662	0.072001508
ACP1	0.172766	0.059903	2.884078	0.00392562	0.072332654
NDUFAF2	0.326543	0.113224	2.884042	0.00392606	0.072332654
CCDC28B	-0.515868	0.17887	-2.884031	0.0039262	0.072332654
GAR1	0.317531	0.110109	2.883777	0.00392937	0.072332654
EIF4B	-0.182995	0.063476	-2.882883	0.00394054	0.072342104
FARSA	0.320236	0.111087	2.882741	0.00394232	0.072342104
OGG1	-0.36531	0.126818	-2.880585	0.00396938	0.072723963
LYAR	0.375672	0.130513	2.878421	0.00399671	0.072987351
OFD1	-0.360621	0.125291	-2.878271	0.00399862	0.072987351
TTC24	-1.767619	0.61416	-2.878106	0.00400071	NA
HSPE1	0.29273	0.101716	2.877922	0.00400304	0.072987351
LMAN1	0.318291	0.110615	2.877465	0.00400885	0.072987351
FAM111A	-0.540288	0.188076	-2.872709	0.00406969	0.073979257
L1CAM	-1.619025	0.563781	-2.871726	0.00408237	0.074026521
MDH2	0.213921	0.074497	2.871521	0.00408501	0.074026521
RASSF7	-0.375728	0.130873	-2.870924	0.00409273	0.07405111
RPS6	-0.137166	0.04782	-2.868362	0.00412603	0.074496691
SLC23A2	0.585569	0.20417	2.868045	0.00413017	0.074496691
S100A6	-0.720512	0.251323	-2.866873	0.00414549	0.074657304
ETS1	-0.331502	0.115662	-2.86612	0.00415537	0.074719593
RPF2	0.319184	0.111498	2.86269	0.00420061	0.075416535
GPT2	0.623589	0.218099	2.859203	0.00424707	0.076067797
PAXX	0.30484	0.106625	2.858987	0.00424996	0.076067797

LINC02328	-1.143507	0.400542	-2.8549	0.00430504	0.076935293
SSR2	-0.161808	0.056729	-2.852311	0.00434026	0.077445633
SMARCB1	0.221536	0.077702	2.851121	0.00435654	0.077585614
MCTS1	0.257828	0.090442	2.850763	0.00436144	0.077585614
WIPF1	-0.26879	0.094378	-2.848007	0.0043994	0.078141437
IARS	0.288721	0.101406	2.847173	0.00441094	0.078145465
GPATCH2L	-0.298139	0.10474	-2.846473	0.00442065	0.078145465
CCNI	-0.175976	0.061826	-2.846297	0.00442309	0.078145465
SMIM29	-0.263765	0.092681	-2.845954	0.00442785	0.078145465
DMXL1	-0.425936	0.149684	-2.845569	0.00443321	0.078145465
PRMT5	0.35825	0.126046	2.842219	0.00448008	0.078852044
MTHFD2	0.381391	0.134275	2.840379	0.004506	0.079188439
PSME2	0.197033	0.069448	2.837143	0.00455192	0.079874934
EPM2AIP1	-0.259684	0.091573	-2.83583	0.00457067	0.079929667
NOP56	0.219584	0.077446	2.835332	0.0045778	0.079929667
RPL13	-0.137972	0.048667	-2.834992	0.00458269	0.079929667
OXR1	-0.473867	0.167163	-2.834753	0.00458611	0.079929667
DISP1	-0.978753	0.345297	-2.834524	0.0045894	0.079929667
TSR1	0.288598	0.101848	2.833618	0.00460243	0.08003686
ZPR1	0.259339	0.09156	2.832456	0.0046192	0.08020858
ALYREF	0.50732	0.179171	2.831493	0.00463313	0.080330575
TMEM191C	0.686698	0.242644	2.830065	0.00465386	0.080569929
ELOF1	0.283015	0.100053	2.828649	0.00467449	0.080705418
BCL2L11	-0.430633	0.152267	-2.828136	0.004682	0.080705418
RAB7A	0.205531	0.072675	2.828101	0.0046825	0.080705418
B4GALT1	-0.414271	0.146531	-2.827186	0.0046959	0.080816706
SSBP1	0.273582	0.096879	2.823956	0.00474349	0.081411517
SRPK1	0.256901	0.090974	2.823891	0.00474446	0.081411517
CAPRIN2	-0.720208	0.255117	-2.823054	0.00475685	0.081504029
PFKFB3	-0.635359	0.225195	-2.821369	0.00478192	0.081812981
NARS	0.240383	0.085262	2.819333	0.00481236	0.082212838
DNPEP	0.223718	0.079513	2.813587	0.00489921	0.083453078
PGAM1	0.252408	0.08971	2.813581	0.0048993	0.083453078
IL7	-0.55387	0.196968	-2.811975	0.00492383	0.083748376
PYCR1	0.416762	0.148347	2.809364	0.00496395	0.084265896
FARSB	0.353422	0.125818	2.808987	0.00496976	0.084265896
GLOD4	0.300706	0.107067	2.80858	0.00497605	0.084265896
AKAP1	0.50145	0.178572	2.808116	0.00498323	0.084265896
YIF1A	0.22598	0.080616	2.803167	0.00506035	0.085228275
FXYD7	-0.928686	0.331359	-2.802654	0.0050684	0.085228275
SPIB	0.196651	0.07017	2.802488	0.00507101	0.085228275
SPTBN1	0.301197	0.107481	2.802323	0.0050736	0.085228275
ILF2	0.233204	0.083224	2.802122	0.00507678	0.085228275
SNRPC	0.2329	0.083348	2.794301	0.00520121	0.08719147
C1D	0.232217	0.083118	2.793835	0.00520871	0.087191562
ENSG00000244459	-0.78642	0.281715	-2.791549	0.00524564	0.087622118
ENSG00000259038	-1.40396	0.502986	-2.791249	0.00525051	0.087622118

RPL30	-0.104964	0.03761	-2.790847	0.00525703	0.087622118
EBF1	-0.462589	0.165822	-2.789665	0.00527625	0.087816791
GM2A	0.469938	0.168519	2.788639	0.00529301	0.087936495
U2AF1L4	-0.348781	0.125087	-2.788299	0.00529856	0.087936495
DDX17	-0.185795	0.066678	-2.786448	0.00532892	0.088189692
YTHDF2	0.270251	0.096988	2.786444	0.00532898	0.088189692
SHQ1	0.526961	0.189188	2.785379	0.00534652	0.088354241
SLC37A1	-0.476667	0.171224	-2.783886	0.00537119	0.088584972
EIF4E	0.22913	0.082314	2.783613	0.00537571	0.088584972
SSRP1	0.215526	0.077447	2.782889	0.00538772	0.08865737
DNAJC7	0.207126	0.074452	2.782002	0.00540247	0.088677861
CDK4	0.295512	0.106238	2.781606	0.00540907	0.088677861
TNFRSF13B	-0.865163	0.311049	-2.78144	0.00541183	0.088677861
NAA20	0.221484	0.079722	2.778214	0.00546586	0.089437147
LRRC59	0.268424	0.096678	2.776463	0.0054954	0.089779248
GNB5	-0.312674	0.112632	-2.77606	0.0055022	0.089779248
TMEM201	0.826319	0.297814	2.774617	0.00552668	0.090052392
NDUFB6	0.319485	0.115186	2.773651	0.00554311	0.090193777
CARNS1	-1.258642	0.453898	-2.772964	0.00555482	0.090258084
TPT1	-0.164635	0.059426	-2.770448	0.00559793	0.09083171
BUD23	0.249997	0.090493	2.762625	0.00573387	0.092907783
ECHS1	0.260218	0.094246	2.761038	0.0057618	0.093230609
PSMD13	0.226465	0.082059	2.759796	0.00578375	0.093455678
CEP135	-0.32215	0.116821	-2.757642	0.005822	0.093893201
LPP	-0.413934	0.150119	-2.757363	0.00582696	0.093893201
SPRYD4	0.461092	0.167471	2.753268	0.00590036	0.094944345
NICN1	0.670679	0.243749	2.751516	0.00593201	0.095321821
TMEM219	-0.22795	0.082918	-2.749087	0.00597616	0.095794519
AARS	0.352858	0.128359	2.748991	0.00597789	0.095794519
FLNA	-0.492881	0.179365	-2.747919	0.00599748	0.095976209
PFDN5	-0.142789	0.05199	-2.746485	0.00602377	0.096133547
CD5	-1.474209	0.536763	-2.746481	0.00602384	0.096133547
SLC16A3	0.743118	0.270655	2.745625	0.00603957	0.096252575
ATP6V0B	0.215607	0.078674	2.74053	0.00613401	0.097623961
PCBD2	-0.413962	0.151079	-2.740042	0.00614314	0.09763563
MYEF2	-0.519116	0.189747	-2.73583	0.00622231	0.098692352
GKAP1	0.535103	0.195607	2.735604	0.00622659	0.098692352
EIF2S2	0.224341	0.082064	2.733729	0.00626216	0.09912099
FAM210A	0.304515	0.111453	2.732229	0.00629073	0.099339905
MPHOSPH9	0.44896	0.164327	2.732107	0.00629306	0.099339905
PPFIBP2	0.435861	0.159923	2.725444	0.00642152	0.101230243
ZNF593	0.289416	0.106402	2.720027	0.00652765	0.10276418
DOP1A	-0.887759	0.326786	-2.716638	0.00659488	0.1036302
HNRNPK	0.144197	0.053085	2.716357	0.00660048	0.1036302
PABPC1	-0.157667	0.058055	-2.715832	0.00661095	0.103654634
PRPF31	0.224379	0.082643	2.715036	0.00662685	0.103764216
KCNH4	1.530315	0.564006	2.713296	0.00666175	0.104170429

GPR183	-0.401809	0.148226	-2.710782	0.00671247	0.104715208
PDCD2L	0.406473	0.149953	2.710677	0.00671459	0.104715208
ST6GAL1	-0.256325	0.0946	-2.709569	0.00673708	0.104840669
BANK1	-0.454694	0.167832	-2.709215	0.00674426	0.104840669
TNIP1	0.222058	0.081973	2.708928	0.0067501	0.104840669
PRPS1	0.325589	0.120219	2.708307	0.00676274	0.104840669
UBALD1	0.296529	0.109499	2.708064	0.00676769	0.104840669
ASF1A	0.343471	0.12689	2.706831	0.00679289	0.105091008
LRPAP1	0.261672	0.096744	2.704803	0.00683449	0.10550244
SIGLEC5	1.420472	0.525196	2.704651	0.00683762	0.10550244
RPP25L	0.286646	0.106052	2.702886	0.00687404	0.105893038
DDB2	-0.298952	0.110644	-2.701922	0.006894	0.105893038
BCL11A	-0.261965	0.096971	-2.70148	0.00690316	0.105893038
MRPL12	0.287653	0.10648	2.701465	0.00690348	0.105893038
PIK3CA	-0.349272	0.129301	-2.701226	0.00690844	0.105893038
PPIB	0.183681	0.068057	2.698939	0.0069561	0.106483168
MNT	0.56974	0.211184	2.697832	0.00697926	0.106697339
ISCA1	0.240342	0.089132	2.696466	0.00700795	0.10699546
JDP2	1.010952	0.375001	2.695863	0.00702066	0.106997223
CCT4	0.163616	0.060702	2.695382	0.0070308	0.106997223
C1QBP	0.297962	0.110555	2.695152	0.00703566	0.106997223
MX2	-0.794506	0.294841	-2.69469	0.00704541	0.107005675
KCTD3	-1.427453	0.529995	-2.693335	0.00707412	0.107301503
OGFRL1	-0.481368	0.179189	-2.686377	0.00722315	0.109419383
MRPS35	0.243689	0.0908	2.683788	0.00727933	0.110127039
NAALADL1	-0.456754	0.170299	-2.68208	0.0073166	0.110467778
C8orf33	0.224481	0.083703	2.681886	0.00732084	0.110467778
CAMK2G	-0.427249	0.159349	-2.681221	0.00733541	0.110544295
MAT2A	0.211173	0.078987	2.673524	0.00750588	0.11296689
PDE7A	0.301165	0.112698	2.672322	0.00753283	0.113226052
FKBP5	0.58127	0.217617	2.671073	0.00756092	0.113379864
ABRAXAS1	-0.365325	0.136775	-2.671	0.00756256	0.113379864
KLHL5	-0.41492	0.155406	-2.669917	0.00758701	0.113600058
SGSM3	-0.276001	0.103408	-2.669053	0.00760655	0.113746269
PDCD11	0.300547	0.112709	2.666572	0.00766292	0.114442183
NMT2	-0.616984	0.231466	-2.665551	0.00768623	0.114643135
PDCD5	0.2188	0.082135	2.66389	0.00772428	0.115063062
PDIA4	0.321549	0.120761	2.662698	0.0077517	0.115323874
RPL26L1	0.224332	0.084266	2.662185	0.00776351	0.115352065
UPP1	0.766389	0.287929	2.661727	0.00777409	0.115361924
SNRPG	0.171422	0.064465	2.659168	0.00783339	0.116093769
MYO7B	-2.174092	0.817589	-2.65915	0.00783381	NA
EIF3A	0.181735	0.068387	2.657462	0.00787315	0.116471721
SLC3A2	0.286677	0.107886	2.657215	0.00787891	0.116471721
IFNAR2	0.293397	0.110445	2.656492	0.00789584	0.116573887
CAST	-0.296435	0.11162	-2.655757	0.00791306	0.116679967
ZNF165	0.536538	0.202257	2.652751	0.00798388	0.117575311

HIGD2A	-0.207271	0.078149	-2.65226	0.0079955	0.117597483
PTGS1	-0.720528	0.27196	-2.649392	0.00806367	0.118450465
TRUB2	0.346772	0.130918	2.648775	0.00807841	0.118517367
SH2B3	0.544104	0.205544	2.647142	0.00811753	0.118941223
ETFA	0.272692	0.103137	2.643989	0.00819354	0.119903889
RPS23	-0.127011	0.048088	-2.641189	0.00826156	0.12074744
NPC2	-0.329709	0.124864	-2.640538	0.00827745	0.12075434
DNAJB1	0.363232	0.137588	2.640003	0.00829052	0.12075434
SLC38A1	-0.243315	0.092168	-2.639895	0.00829317	0.12075434
SH2D3C	-0.50342	0.190825	-2.638118	0.00833675	0.120962829
TXNRD1	0.294261	0.111548	2.637977	0.00834023	0.120962829
GPR137	-0.264167	0.10014	-2.637965	0.00834053	0.120962829
SRM	0.447198	0.169546	2.637617	0.00834908	0.120962829
GADD45A	0.57579	0.218452	2.635777	0.00839449	0.121469585
CHORDC1	0.292606	0.111113	2.633418	0.00845304	0.122164757
MATK	1.110841	0.421951	2.632631	0.00847264	0.122296201
GFI1	1.115809	0.423946	2.631962	0.00848934	0.122385278
HPRT1	0.226979	0.086256	2.631442	0.00850234	0.122421007
PPP2CA	0.237295	0.090199	2.630797	0.00851848	0.122501884
RPIA	0.223775	0.085137	2.628413	0.00857844	0.123211758
SAP18	0.143985	0.054821	2.626477	0.00862739	0.123762064
CEP126	-0.842042	0.320694	-2.625689	0.00864738	0.12382111
CCDC141	-0.828149	0.315428	-2.625476	0.00865279	0.12382111
ATG101	0.344421	0.131212	2.624912	0.00866713	0.123874002
CCR9	2.108226	0.80326	2.624587	0.00867542	NA
ZBTB18	-0.516713	0.196929	-2.623859	0.00869398	0.124105247
CCDC71L	-0.609571	0.232386	-2.623092	0.00871357	0.124232467
TGFBR2	-0.235813	0.089924	-2.622367	0.00873214	0.124344789
ZBTB8OS	0.221741	0.084608	2.620807	0.00877219	0.124762406
NASP	0.208072	0.07941	2.620235	0.00878692	0.124819299
WDR97	1.7268	0.65938	2.618822	0.0088234	NA
DALRD3	-0.254569	0.097362	-2.614676	0.0089312	0.126714147
MAN1B1	-0.391738	0.149867	-2.613914	0.00895117	0.126740458
TLE1	0.633288	0.242289	2.613773	0.00895484	0.126740458
TMEM230	0.283307	0.10844	2.612576	0.00898626	0.127030601
PPAN	0.255402	0.097818	2.610976	0.00902841	0.127471508
PDE4B	-0.284442	0.108987	-2.609861	0.0090579	0.127732862
RASSF2	-0.340081	0.130393	-2.608114	0.00910426	0.128099281
SPPL2B	-0.402083	0.15417	-2.608052	0.00910591	0.128099281
IKBKB	-0.356026	0.13656	-2.607106	0.00913111	0.128181831
MALAT1	-0.176095	0.067548	-2.606976	0.00913458	0.128181831
FAM41C	0.461558	0.177092	2.606319	0.00915211	0.128181831
CHCHD2	0.183599	0.070447	2.60618	0.00915585	0.128181831
LGALS1	-0.817439	0.313725	-2.605589	0.00917163	0.128191786
KANSL3	0.727623	0.279283	2.60533	0.00917859	0.128191786
PPM1K	-0.287916	0.110641	-2.602244	0.00926159	0.128962085
ALKBH2	0.298003	0.114519	2.60221	0.0092625	0.128962085

NFKBIB	0.262415	0.100859	2.601796	0.0092737	0.128962085
YPEL5	-0.345547	0.13284	-2.601225	0.00928914	0.128962085
MRPL47	0.207079	0.079608	2.601224	0.00928917	0.128962085
TCTN1	-0.865581	0.332876	-2.600313	0.00931388	0.129150925
GABARAPL1	0.728463	0.280226	2.599556	0.00933444	0.129213944
PSD3	-1.038237	0.399462	-2.599092	0.00934707	0.129213944
TFEB	0.450367	0.17329	2.598921	0.00935174	0.129213944
HM13	0.219106	0.084331	2.598149	0.00937278	0.12926196
ENSG00000276136	-0.826384	0.318104	-2.59784	0.00938121	0.12926196
GZF1	0.568627	0.218907	2.597572	0.00938855	0.12926196
CLN6	0.290213	0.111939	2.592599	0.00952538	0.130990847
RPL3	-0.143583	0.0554	-2.591779	0.00954811	0.131148465
SH2D2A	0.721915	0.278941	2.588057	0.00965189	0.132417579
MRPS7	0.193945	0.074993	2.586175	0.00970476	0.132986054
ICA1	1.945017	0.752661	2.584187	0.00976088	0.133597792
ENSG00000204758	-1.386389	0.537126	-2.581124	0.00984792	0.134622297
BARD1	-0.482156	0.186829	-2.58074	0.00985888	0.134622297
NUDT5	0.233279	0.090439	2.579418	0.0098967	0.134980354
ZNF277	-0.285118	0.110557	-2.578933	0.00991061	0.135003487
XRCC5	0.156307	0.060618	2.57855	0.00992161	0.135003487
PRELID3B	0.223588	0.086775	2.576655	0.00997615	0.135586992
KBTBD3	-0.411199	0.159671	-2.575281	0.01001588	0.135968223
IL10RA	-0.394788	0.153368	-2.57413	0.01004924	0.136262122
RAP2C	-0.308446	0.119884	-2.572863	0.01008611	0.136602769
NOP2	0.335851	0.130566	2.572265	0.01010354	0.136611048
CEBPB	0.756845	0.294267	2.571967	0.01011224	0.136611048
PKN1	0.308527	0.119973	2.571635	0.01012195	0.136611048
CEMIP2	-0.670151	0.260639	-2.571179	0.0101353	0.136632742
EML2	0.392483	0.152762	2.569246	0.01019201	0.137188239
DYNLT3	-0.293847	0.114383	-2.568971	0.01020009	0.137188239
PVT1	0.516265	0.201133	2.566792	0.01026442	0.137894063
PROC	-1.908175	0.743563	-2.56626	0.01028018	NA
CCDC59	0.176158	0.068704	2.564001	0.01034732	0.13884748
MARCHF1	-0.523948	0.204438	-2.56287	0.0103811	0.13900933
AK6	0.233272	0.091034	2.562479	0.0103928	0.13900933
PSMA6	0.267393	0.104369	2.562009	0.01040687	0.13900933
TOMM22	0.185726	0.072493	2.561998	0.01040718	0.13900933
EIF2S1	0.223707	0.087343	2.561253	0.01042954	0.139017502
PBX4	1.086479	0.424223	2.561102	0.01043406	0.139017502
MAFG.DT	0.864207	0.337505	2.560579	0.01044978	0.139017502
SF3B5	0.138803	0.054212	2.560386	0.01045559	0.139017502
CLIC1	-0.277843	0.108536	-2.559915	0.01046978	0.139047251
RCSD1	-0.315054	0.123163	-2.558019	0.01052703	0.139514222
UQCR11	0.177011	0.0692	2.557953	0.01052904	0.139514222
IER5L	-1.392427	0.544435	-2.557561	0.01054092	0.139514222
CHRNB1	-0.422094	0.16517	-2.55551	0.01060324	0.140179705
NDUFAB1	0.24681	0.096617	2.55451	0.01063375	0.140423444

CRYM	-1.389567	0.544107	-2.553847	0.01065401	0.140531476
SNX20	-0.420019	0.16459	-2.551913	0.01071333	0.14115384
ZNF33B	-0.433343	0.169879	-2.550893	0.01074474	0.141407542
M6PR	0.169228	0.066353	2.550429	0.01075905	0.141435952
MRPL22	0.189382	0.074276	2.549724	0.01078083	0.141552937
CEBPG	0.242785	0.095234	2.549353	0.01079229	0.141552937
USP6NL	-0.392052	0.153836	-2.548507	0.01081852	0.141737196
TMEM256	-0.203012	0.079699	-2.547222	0.01085843	0.141859106
TRMT1	0.216658	0.08506	2.547137	0.01086108	0.141859106
SMIM7	-0.164317	0.064513	-2.54703	0.0108644	0.141859106
FOXO4	-1.055129	0.414426	-2.546004	0.01089639	0.141936624
SLC25A15	-1.018343	0.400009	-2.545798	0.01090282	0.141936624
POLR1E	0.313677	0.12322	2.545666	0.01090694	0.141936624
PSMA7	0.193071	0.075869	2.544803	0.01093393	0.142128852
FUBP1	0.202377	0.079542	2.544263	0.01095085	0.142130414
NAP1L1	-0.186165	0.073178	-2.54402	0.01095848	0.142130414
LINC00847	0.394266	0.155018	2.543351	0.0109795	0.142244408
MCRIP2	0.328887	0.129337	2.542879	0.01099434	0.142278279
HDGF	0.246145	0.096845	2.541629	0.01103374	0.142629425
HGSNAT	-0.48864	0.192396	-2.539764	0.01109272	0.14301256
SHISAL2A	0.367588	0.144757	2.539342	0.01110611	0.14301256
ATP2A2	0.354702	0.139686	2.539276	0.01110823	0.14301256
LPAR5	-0.468398	0.184471	-2.53914	0.01111255	0.14301256
RIC8B	-0.99791	0.393152	-2.538228	0.01114155	0.143227384
PSMB3	0.180877	0.071333	2.535663	0.01122347	0.144067364
TWNK	0.441664	0.174198	2.535408	0.01123166	0.144067364
PSMD14	0.198312	0.078284	2.533225	0.01130185	0.144762899
CPEB4	0.512464	0.202335	2.532756	0.01131695	0.144762899
DRAM1	0.56941	0.224835	2.532563	0.01132321	0.144762899
MDM2	0.309435	0.122223	2.531725	0.01135029	0.144928518
NCBP2AS2	0.231936	0.091624	2.531392	0.01136108	0.144928518
LSM1	0.159296	0.063051	2.526462	0.01152177	0.146817384
MKNK2	-0.378767	0.149945	-2.526032	0.0115359	0.146836595
S1PR1	-0.5021	0.198854	-2.524967	0.01157091	0.147121294
NT5DC3	0.619346	0.245389	2.523942	0.01160472	0.147390076
LGALS3BP	-1.949695	0.773139	-2.521792	0.01167588	NA
PYCR2	0.250127	0.099241	2.520409	0.01172184	0.148606224
EIF3D	-0.17809	0.070663	-2.520267	0.01172658	0.148606224
CEP112	-1.033338	0.410074	-2.519882	0.01173941	0.148606224
WDR18	0.275584	0.109403	2.518975	0.0117697	0.148606224
MTDH	0.243801	0.096794	2.518764	0.01177674	0.148606224
WDFY2	-0.442546	0.17573	-2.518325	0.01179143	0.148606224
EHD4	-0.305749	0.121424	-2.518033	0.01180124	0.148606224
PPDPF	-0.285914	0.113549	-2.51799	0.01180266	0.148606224
CRIP3	-0.702645	0.279112	-2.517426	0.01182157	0.148683445
CCDC50	-0.217938	0.08665	-2.515167	0.0118976	0.149202546
COL19A1	-0.566319	0.225165	-2.515134	0.01189873	0.149202546

ST8SIA4	-0.399716	0.158929	-2.515057	0.01190132	0.149202546
MRPS17	0.288832	0.114864	2.514548	0.01191849	0.149257008
ADA	-0.337338	0.13433	-2.511254	0.01203031	0.150433099
NUDT14	-0.569782	0.226913	-2.511021	0.01203827	0.150433099
ANXA5	-0.385043	0.15351	-2.508262	0.01213267	0.151450144
WDR36	0.310033	0.123625	2.507861	0.01214644	0.151459014
SRSF3	0.174033	0.069405	2.507484	0.01215942	0.151459014
SORL1	-0.436197	0.173995	-2.506953	0.0121777	0.15152441
CD180	0.515492	0.205691	2.506149	0.01220541	0.151707006
SYVN1	0.330577	0.131958	2.505175	0.01223908	0.151903633
WDR43	0.248934	0.099388	2.504667	0.01225667	0.151903633
PIP4P2	-0.618877	0.2471	-2.504559	0.0122604	0.151903633
RPL34	-0.123316	0.049258	-2.503481	0.01229784	0.152205344
NUCB1	-0.251519	0.100622	-2.499636	0.01243209	0.153470659
GABBR1	-0.53894	0.215616	-2.499538	0.01243554	0.153470659
MTHFD1L	0.526206	0.210531	2.499421	0.01243965	0.153470659
AATF	0.248893	0.099621	2.498405	0.01247537	0.153655902
IFI44	-0.937714	0.375349	-2.498243	0.01248108	0.153655902
ILK	-0.290677	0.116405	-2.497107	0.01252113	0.153986017
BZW2	0.268247	0.107544	2.494306	0.01262038	0.154981963
FBXL20	-0.430621	0.172658	-2.49407	0.01262875	0.154981963
MBD2	0.29924	0.120048	2.492672	0.0126786	0.155429707
CHD7	0.288417	0.115823	2.490158	0.01276864	0.156368765
TMEM41B	0.399499	0.160522	2.488755	0.01281914	0.156791095
TBC1D2B	-0.869015	0.349267	-2.488113	0.01284229	0.156791095
ENSG00000272211	-0.502741	0.20206	-2.488078	0.01284356	0.156791095
ENSG00000225938	-0.926219	0.372489	-2.486568	0.0128982	0.157138761
DDX1	0.228677	0.091966	2.486544	0.01289905	0.157138761
LRRC1	-0.84349	0.339525	-2.484325	0.01297974	0.157940986
RAB11B	-0.216475	0.087159	-2.48368	0.01300327	0.157940986
PCNA	0.335682	0.135159	2.483615	0.01300563	0.157940986
FLOT2	-0.271795	0.109489	-2.482398	0.01305014	0.158316326
EIF3L	-0.198101	0.079821	-2.481808	0.01307178	0.158354777
ENSG00000260349	0.624229	0.251546	2.481569	0.01308054	0.158354777
MRPL40	0.216385	0.087326	2.477908	0.01321551	0.159735016
FXN	0.327326	0.132107	2.477733	0.01322201	0.159735016
LRRC25	1.679857	0.678879	2.474459	0.01334383	NA
NAT14	0.65775	0.265956	2.473155	0.01339259	0.161610067
ENSG00000267136	2.373139	0.959628	2.472977	0.01339928	NA
RAB6A	-0.242997	0.098276	-2.472601	0.01341339	0.161610067
PDCD4	0.397677	0.160857	2.472232	0.01342722	0.161610067
UTP11	0.258687	0.104643	2.472084	0.01343278	0.161610067
ATP2B1	-0.176275	0.071321	-2.471562	0.01345244	0.161679439
CCSER1	-0.658528	0.266812	-2.468134	0.01358195	0.163067545
CABLES1	-0.863119	0.349824	-2.467296	0.01361378	0.163259405
HSD17B11	-0.281073	0.113961	-2.4664	0.01364789	0.163259405
HNRNPA2B1	0.176156	0.071427	2.46624	0.01365397	0.163259405

RBM8A	0.13283	0.053867	2.465884	0.01366756	0.163259405
CDK19	-0.40743	0.165227	-2.46587	0.0136681	0.163259405
RPL12	-0.147538	0.059855	-2.464904	0.01370501	0.163532442
FAM167A	0.984911	0.399812	2.463438	0.01376117	0.164002412
SLC9B2	0.318432	0.129279	2.46314	0.01377259	0.164002412
RASGRP2	-0.417422	0.169544	-2.462032	0.01381523	0.164341954
HDDC2	0.194358	0.078988	2.460602	0.0138704	0.164712643
LIMD2	-0.253589	0.103071	-2.460332	0.01388085	0.164712643
CPNE5	0.619799	0.25194	2.460102	0.01388977	0.164712643
SLC29A1	0.605157	0.246023	2.459759	0.01390303	0.164712643
RCN1	0.334281	0.136107	2.456022	0.01404844	0.166133166
ITPA	0.29196	0.118879	2.455944	0.01405149	0.166133166
PLEKHJ1	-0.241962	0.098622	-2.453441	0.01414969	0.167124334
PRKDC	0.239646	0.097722	2.45234	0.01419304	0.167282076
FAM126A	-0.380904	0.155331	-2.45221	0.01419816	0.167282076
GDF11	-0.424775	0.173236	-2.452007	0.01420618	0.167282076
CHD2	-0.247263	0.100904	-2.450475	0.01426679	0.167825927
CAMLG	-0.222047	0.090631	-2.45	0.01428561	0.167877548
TRIAP1	0.240388	0.098141	2.449416	0.01430881	0.167980458
BTD	-0.714298	0.291765	-2.448195	0.01435738	0.168188325
CAMK2D	-0.391557	0.159944	-2.448087	0.0143617	0.168188325
CYTH4	-0.468115	0.191233	-2.447882	0.01436988	0.168188325
SNX14	-0.333855	0.136407	-2.447492	0.01438541	0.168200913
FAM214B	-0.819856	0.335105	-2.446568	0.01442234	0.168322134
TRMT10B	-0.344883	0.14097	-2.446509	0.01442472	0.168322134
GPR65	-0.269694	0.11029	-2.445312	0.01447269	0.168712657
FAM53B	-0.362471	0.148323	-2.443797	0.01453361	0.169253283
SNRNP25	-0.202422	0.082904	-2.441628	0.01462122	0.170103248
DRAP1	-0.179718	0.073624	-2.441044	0.01464488	0.170136918
SORBS3	-0.782348	0.320581	-2.440404	0.01467083	0.170136918
SLC25A19	0.28208	0.115589	2.440372	0.01467213	0.170136918
OTUD4	0.349242	0.143125	2.440114	0.01468261	0.170136918
SRSF1	0.182403	0.074773	2.439431	0.01471043	0.170289707
SETX	-0.322884	0.132407	-2.438571	0.01474547	0.170525639
LRRFIP1	-0.224625	0.09215	-2.437605	0.01478494	0.17057206
GRINA	0.280311	0.115004	2.437396	0.01479347	0.17057206
CCDC112	-0.3996	0.163953	-2.437285	0.01479803	0.17057206
FDFT1	0.209271	0.08588	2.43678	0.01481868	0.17057206
APOBEC3D	-0.398101	0.163378	-2.43668	0.01482279	0.17057206
RRP1	0.238943	0.098081	2.436175	0.01484349	0.170641448
ENSG00000225335	-2.163301	0.888446	-2.434926	0.01489484	NA
ZNF224	-0.326087	0.133921	-2.434917	0.01489521	0.17082895
MMP7	1.298591	0.533381	2.434641	0.01490658	0.17082895
PEA15	-0.271805	0.111644	-2.43456	0.01490989	0.17082895
EIF3E	-0.155126	0.063724	-2.434351	0.01491853	0.17082895
CEBPZ	0.239788	0.098548	2.433215	0.01496541	0.171197255
PEX2	0.234016	0.096261	2.431062	0.01505464	0.172003132

GLIPR1	-0.364921	0.150124	-2.430802	0.01506543	0.172003132
MRPL50	0.260567	0.107238	2.429808	0.01510684	0.172306817
SLC25A17	0.353095	0.145392	2.428574	0.01515832	0.172552534
PGM2L1	-0.481016	0.198073	-2.428482	0.01516217	0.172552534
MRPL18	0.21872	0.090074	2.428226	0.01517288	0.172552534
EHBP1	0.466588	0.192226	2.427284	0.01521235	0.172644842
PIK3CD	0.355476	0.146461	2.427106	0.01521982	0.172644842
SSB	0.181049	0.074606	2.426733	0.01523547	0.172644842
NAGK	-0.274589	0.113157	-2.426617	0.01524035	0.172644842
APMAP	0.410378	0.169149	2.426138	0.01526046	0.172704414
KLF6	-0.301776	0.124414	-2.425573	0.01528424	0.172805509
ENSG00000273247	-0.277732	0.114605	-2.423373	0.01537715	0.173687111
WDR74	0.26191	0.108109	2.422645	0.01540799	0.173866681
UBE2T	0.51974	0.214578	2.422149	0.01542901	0.173935134
FAM111A.DT	-0.550418	0.227285	-2.42171	0.01544769	0.173977144
NR6A1	0.585931	0.242005	2.42115	0.0154715	0.173977491
RPL11	-0.111328	0.045984	-2.421006	0.01547763	0.173977491
ZNF782	-0.867858	0.3587	-2.419453	0.01554388	0.174501155
VCP	0.220626	0.091198	2.419211	0.01555421	0.174501155
ZNF239	0.842101	0.348147	2.418805	0.01557158	0.174519814
MRPL32	0.234406	0.096923	2.418471	0.01558588	0.174519814
CARD19	-0.38935	0.16106	-2.417422	0.0156309	0.174855652
MECR	0.431486	0.178599	2.415951	0.01569418	0.175394911
ENSG00000274605	0.619183	0.256416	2.414755	0.01574581	0.175448185
FAM219B	-0.277731	0.115021	-2.414608	0.01575216	0.175448185
VAMP1	-0.450289	0.18649	-2.414553	0.01575451	0.175448185
USP15	-0.212484	0.088005	-2.414443	0.01575927	0.175448185
ENSG00000274265	-0.404671	0.167717	-2.412813	0.01582995	0.175820233
TBL2	0.418827	0.173587	2.412779	0.01583141	0.175820233
KCNK6	-0.362884	0.15041	-2.412627	0.01583803	0.175820233
PLPP3	1.081252	0.44835	2.411625	0.01588159	0.175996084
WIZ	0.42598	0.17664	2.411567	0.01588412	0.175996084
PLEKHB2	0.250861	0.104062	2.410678	0.01592292	0.176258098
PAFAH1B2	-0.198739	0.082465	-2.409978	0.01595349	0.176428655
IKZF3	-0.198214	0.082262	-2.409554	0.01597205	0.176466123
TMED10	0.200866	0.083492	2.405817	0.01613634	0.178100318
MCM3AP.AS1	-0.53901	0.224074	-2.405495	0.01615058	0.178100318
ILVBL	0.419047	0.17433	2.403755	0.01622767	0.178780926
UXT	-0.09809	0.040833	-2.402254	0.01629437	0.179346007
ABCA2	1.364388	0.56808	2.401756	0.01631659	0.17942085
RPL28	-0.164291	0.068513	-2.397943	0.01648745	0.181128358
AFF3	-0.373938	0.155989	-2.397203	0.01652076	0.181286957
LINC01800	-1.382686	0.576857	-2.396931	0.01653305	0.181286957
LINC00926	-0.387773	0.161811	-2.396454	0.01655457	0.181352019
CASD1	0.565885	0.23637	2.394066	0.01666277	0.182240042
IRAK3	-1.16196	0.485369	-2.393973	0.01666696	0.182240042
IGHV4.34	-1.795103	0.750611	-2.391522	0.0167787	NA

RAE1	0.348222	0.145623	2.391262	0.01679056	0.183303471
CD19	-0.186597	0.078037	-2.391149	0.01679573	0.183303471
RAB30	0.272946	0.11424	2.389234	0.01688356	0.183611251
C12orf76	-0.350074	0.146538	-2.38897	0.01689566	0.183611251
HSDL1	-0.648605	0.271506	-2.388914	0.01689824	0.183611251
PSMB7	0.164706	0.068954	2.388632	0.01691125	0.183611251
ATP6V0A1	0.470972	0.197205	2.388228	0.01692983	0.183611251
DEF6	-0.336372	0.140852	-2.388117	0.01693495	0.183611251
CD48	-0.220906	0.092522	-2.387589	0.0169593	0.183611251
S100A11	-0.416946	0.174646	-2.387374	0.01696923	0.183611251
RIPOR1	-0.387459	0.162313	-2.387109	0.01698147	0.183611251
MAGOHB	0.222477	0.093199	2.387103	0.01698175	0.183611251
MBP	-0.317382	0.132978	-2.386729	0.01699901	0.18362719
ZFP14	-0.353497	0.148163	-2.385864	0.01703907	0.183889195
ANAPC11	0.158545	0.06649	2.384504	0.01710215	0.184264055
BCLAF3	-0.698897	0.293154	-2.384063	0.01712267	0.184264055
SF3B6	0.149106	0.062546	2.383937	0.01712853	0.184264055
FKBP1B	-1.030945	0.432488	-2.383752	0.01713716	0.184264055
STOML2	0.2003	0.084062	2.382768	0.017183	0.184354872
ABLIM2	-1.013241	0.425289	-2.382475	0.01719671	0.184354872
GTF3A	0.210459	0.08834	2.38238	0.01720114	0.184354872
MAU2	-0.400264	0.168022	-2.382212	0.01720899	0.184354872
NEK11	-1.727183	0.725323	-2.381262	0.01725346	NA
TXNIP	-0.298774	0.125521	-2.380277	0.01729962	0.185155297
TMEM185A	-0.421876	0.177357	-2.378688	0.01737438	0.185635406
WNT16	1.038403	0.436552	2.378645	0.01737639	0.185635406
UBE2S	0.394132	0.165733	2.378115	0.0174014	0.185642642
NPHP3	-0.460487	0.193649	-2.377954	0.01740898	0.185642642
PSMD1	0.218072	0.091742	2.377001	0.01745404	0.185925784
UBA52	-0.116282	0.048928	-2.376587	0.01747362	0.185925784
SNRPF	0.201558	0.084817	2.376379	0.01748348	0.185925784
ST13	-0.089024	0.037485	-2.374934	0.01755207	0.186324982
SEC61B	-0.231021	0.097288	-2.374597	0.01756813	0.186324982
GSTM4	-0.762896	0.321277	-2.374577	0.01756907	0.186324982
MPG	-0.249991	0.105354	-2.372854	0.01765123	0.187025885
CCT2	0.172318	0.072631	2.372515	0.01766747	0.187027569
DUSP14	0.442962	0.186814	2.371144	0.01773313	0.18755198
C9orf72	-0.407789	0.17205	-2.370181	0.01777939	0.187870537
HDHD5	0.299938	0.126575	2.369646	0.01780511	0.187971513
DNAJA1	0.176513	0.074546	2.367828	0.01789285	0.188527372
PLEKHO1	0.304713	0.128703	2.367564	0.01790561	0.188527372
TCIRG1	-0.393047	0.166014	-2.367548	0.01790637	0.188527372
ZFAND2B	-0.229896	0.097128	-2.366941	0.01793578	0.188666242
HDAC6	-0.397038	0.167782	-2.366385	0.01796278	0.188779525
CPLANE1	-0.544371	0.230112	-2.365684	0.01799678	0.188901052
RPL32	-0.09443	0.03992	-2.365478	0.01800681	0.188901052
APOBR	-1.474011	0.623613	-2.363663	0.01809525	NA

ZEB1	-0.334132	0.141575	-2.360106	0.01826971	0.191367545
SYK	-0.278706	0.118096	-2.360002	0.01827483	0.191367545
UBAP2	0.300966	0.127601	2.358648	0.01834165	0.191894561
HHIP.AS1	-1.354319	0.574416	-2.35773	0.01838706	0.192093207
RIOK1	0.290508	0.123222	2.357597	0.01839366	0.192093207
TAF2	0.382798	0.162426	2.356757	0.01843531	0.192254499
GNAI3	-0.21341	0.090561	-2.356525	0.0184468	0.192254499
ATF4	0.22436	0.095217	2.356286	0.01845868	0.192254499
WNT2B	-0.98257	0.417079	-2.355837	0.01848101	0.192314952
KMT2A	-0.232917	0.098929	-2.354392	0.01855302	0.192891698
ZC3H18	0.260627	0.110736	2.353589	0.01859314	0.193017818
ENSG00000237773	-0.67056	0.284922	-2.353486	0.01859833	0.193017818
BAZ2A	-0.242819	0.103217	-2.352516	0.01864687	0.193349104
SERPINF2	-1.40667	0.598216	-2.35144	0.0187009	0.193736661
SNRPB2	0.153631	0.065352	2.350812	0.0187325	0.193891416
MCAT	0.342835	0.145925	2.349395	0.01880396	0.194457983
CIAPIN1	0.314601	0.133943	2.348773	0.01883537	0.19449766
HAX1	0.166378	0.070839	2.348657	0.01884123	0.19449766
UBR2	-0.296935	0.126477	-2.347744	0.0188875	0.194619775
U2AF2	0.248228	0.105744	2.347443	0.01890275	0.194619775
SP3	-0.249459	0.106269	-2.347434	0.01890324	0.194619775
ZCRB1	0.183282	0.078159	2.345005	0.01902685	0.195709301
ANKRD49	-0.263596	0.112422	-2.344694	0.01904271	0.195709301
VKORC1L1	0.407556	0.173932	2.343188	0.01911973	0.196327379
GBP7	0.808616	0.345465	2.340661	0.01924963	0.197486907
RPLP2	-0.120131	0.051339	-2.339973	0.01928512	0.197592415
ARAP2	-0.438983	0.187615	-2.339804	0.01929388	0.197592415
ABT1	0.219052	0.093663	2.338722	0.01934983	0.197802177
ZNF800	-0.321501	0.13747	-2.3387	0.01935099	0.197802177
SUV39H2	0.509764	0.217995	2.338422	0.01936537	0.197802177
CNBP	0.190534	0.081502	2.337788	0.01939827	0.197964448
ZNF436.AS1	-1.05272	0.450537	-2.336591	0.01946048	0.198175082
KAT2A	0.291251	0.124651	2.336536	0.0194633	0.198175082
PRKCI	0.661797	0.283254	2.336408	0.01947001	0.198175082
SLC2A1	-0.401529	0.171961	-2.335005	0.01954318	0.198745964
ZC3H7A	-0.398127	0.170557	-2.334271	0.01958153	0.198821436
PRR5	0.641803	0.275047	2.333433	0.01962544	0.198821436
SMURF2	-0.480489	0.205916	-2.333421	0.01962607	0.198821436
TLE3	-0.293194	0.125652	-2.333376	0.01962842	0.198821436
A1BG	-0.300113	0.128625	-2.33323	0.01963605	0.198821436
CENPP	0.591651	0.25373	2.33181	0.0197107	0.1994037
TAF4B	0.455733	0.19553	2.330757	0.01976615	0.199790991
FTSJ1	0.37668	0.161712	2.329325	0.01984185	0.200381973
TEC	-0.861996	0.370779	-2.324823	0.02008141	0.20262544
MFN1	-0.339536	0.14608	-2.324307	0.02010905	0.202728534
LINC02482	-0.741507	0.319279	-2.32244	0.02020924	0.20338878
STX7	-0.194077	0.083566	-2.322435	0.02020951	0.20338878

ATIC	0.208453	0.089776	2.321915	0.02023749	0.20349435
MRPS26	0.167751	0.072275	2.321025	0.02028547	0.203800677
PAN3	-0.249988	0.107733	-2.320443	0.0203169	0.203926389
TSTD1	-0.236449	0.101911	-2.320145	0.02033304	0.203926389
SEPTIN2	0.245264	0.105807	2.318031	0.02044762	0.204898891
SHPRH	-0.279449	0.120658	-2.316045	0.0205558	0.205805683
XRN1	-0.344454	0.148752	-2.31563	0.02057846	0.205855421
ENSG00000246528	0.906306	0.391519	2.314845	0.02062142	0.206051357
PWP1	0.164165	0.070925	2.314625	0.02063347	0.206051357
CCT7	0.202133	0.087376	2.31337	0.02070233	0.206544816
LARP4	0.312383	0.135051	2.313077	0.02071839	0.206544816
ENSG00000245869	0.518105	0.224154	2.311382	0.02081179	0.207062728
IFI27L2	0.291864	0.126301	2.310864	0.02084035	0.207062728
C19orf66	-0.220862	0.095585	-2.310635	0.02085305	0.207062728
RBM5	-0.218364	0.094508	-2.310536	0.02085848	0.207062728
HNRNPA3	0.143052	0.061913	2.310521	0.02085934	0.207062728
RPL39	-0.097779	0.042327	-2.310097	0.02088276	0.207118559
PUF60	0.221689	0.096046	2.308159	0.02099028	0.208007552
AMZ1	-1.098949	0.47655	-2.306053	0.02110769	0.20899311
EXOSC2	0.279629	0.121292	2.305427	0.02114265	0.209161224
PAK1IP1	0.25827	0.11208	2.304339	0.02120361	0.209586017
NEMP1	-0.543779	0.236082	-2.303344	0.02125947	0.209959837
HNRNPR	0.167438	0.072732	2.302117	0.0213286	0.210463861
DHTKD1	-0.335771	0.14593	-2.30091	0.02139673	0.210835445
ANKRD37	0.418019	0.181684	2.300808	0.0214025	0.210835445
COA1	0.267213	0.116354	2.296549	0.02164451	0.21303913
BATF2	2.117648	0.922151	2.296423	0.02165168	NA
ATPSCKMT	0.721317	0.314469	2.293765	0.02180398	0.214427297
INTS14	0.317167	0.138325	2.292912	0.02185305	0.214692925
UQCRFS1	0.130364	0.056862	2.292655	0.0218679	0.214692925
KCNC3	0.519308	0.226638	2.29135	0.02194319	0.215016362
RPN2	0.24857	0.108511	2.290742	0.02197837	0.215016362
AGO1	0.284126	0.124044	2.290527	0.02199076	0.215016362
S100A2	-0.903487	0.394447	-2.290513	0.02199158	0.215016362
GARS	0.312102	0.13626	2.290484	0.02199325	0.215016362
METTL1	0.345933	0.151096	2.289486	0.02205112	0.215401145
ENSG00000261766	0.409234	0.178774	2.289107	0.02207315	0.215435442
ATF6B	-0.188572	0.082392	-2.288728	0.02209518	0.215438242
HSPA14	0.281405	0.122967	2.288465	0.02211048	0.215438242
WDR83OS	0.146016	0.063846	2.286992	0.02219627	0.216093184
ADGRB2	-1.747053	0.764221	-2.286056	0.02225096	NA
RPLP1	-0.117225	0.051284	-2.285802	0.02226587	0.216528142
SYNGR3	0.567706	0.248385	2.285592	0.02227817	0.216528142
LINC01560	-0.514719	0.225259	-2.285005	0.02231254	0.216681212
MRPL20	0.201032	0.088007	2.284257	0.02235642	0.216926259
COX16	0.236234	0.103453	2.283492	0.02240139	0.21718151
RANBP1	0.260753	0.114351	2.280292	0.02259035	0.218831087

TFAP4	0.305929	0.134244	2.278904	0.02267276	0.219446641
NHLRC3	-0.242003	0.106228	-2.278152	0.02271751	0.219697015
HGH1	0.677401	0.297627	2.276008	0.02284553	0.220751577
ZNF569	-0.50554	0.222221	-2.274946	0.02290917	0.221088952
LMO2	-1.037236	0.45597	-2.274791	0.02291845	0.221088952
UCK2	0.38455	0.16936	2.270607	0.02317079	0.223337995
VPS16	-0.397952	0.175363	-2.269303	0.0232499	0.223755087
ARF1	0.184938	0.081497	2.26926	0.02325253	0.223755087
P4HB	0.156988	0.069191	2.268915	0.02327349	0.223771722
MSL3	-0.267493	0.117913	-2.268564	0.02329484	0.223782361
FCRL5	0.541639	0.238798	2.268192	0.02331753	0.223782361
GPN1	0.257013	0.113325	2.26792	0.02333411	0.223782361
SPAG1	-0.643006	0.283558	-2.267634	0.02335154	0.223782361
ATP5PF	0.161732	0.071364	2.266286	0.02343388	0.224386631
SETD4	-0.451138	0.199197	-2.264782	0.02352607	0.225084097
SMIM1	-0.533648	0.235879	-2.262382	0.02367378	0.22631124
ERICH6.AS1	-0.525076	0.23224	-2.260921	0.02376412	0.22697951
MAEA	0.27134	0.120029	2.260621	0.02378271	0.22697951
VPS51	-0.212468	0.094011	-2.260017	0.02382019	0.227025388
RBMS1	-0.378669	0.167559	-2.259915	0.02382654	0.227025388
UBXN11	-0.363969	0.161094	-2.259362	0.02386088	0.227166466
ARFGAP3	-0.274623	0.121597	-2.258471	0.02391632	0.227508171
TBL1X	-0.355594	0.157524	-2.257405	0.02398279	0.227954092
SETD5	-0.190115	0.084248	-2.256623	0.02403164	0.228231896
LATS1	-0.423573	0.187836	-2.255014	0.02413247	0.22856092
SPRY1	0.833803	0.3698	2.254738	0.02414981	0.22856092
TRNAU1AP	-0.213868	0.094854	-2.254699	0.02415225	0.22856092
METTL2B	0.293668	0.13025	2.254649	0.02415536	0.22856092
CHCHD1	0.159051	0.070548	2.254503	0.02416451	0.22856092
B3GNTL1	0.629137	0.279584	2.250265	0.02443214	0.230681377
ZBTB37	-0.463274	0.205877	-2.250245	0.02443341	0.230681377
HACD3	0.351435	0.156207	2.249796	0.02446188	0.230681377
PPIF	-0.242552	0.107815	-2.2497	0.02446801	0.230681377
CMC2	0.247278	0.109933	2.249349	0.02449029	0.230704522
TMX2	0.211206	0.093973	2.24751	0.02460746	0.231478926
ZC3H12D	-0.418567	0.186242	-2.247434	0.02461229	0.231478926
ANKH	-0.506139	0.225449	-2.245027	0.02476641	0.232740205
BMP2K	-0.306161	0.136446	-2.243822	0.02484387	0.233279751
DIPK1A	0.535025	0.238524	2.243064	0.0248927	0.233549768
TUBG1	0.321228	0.143236	2.242648	0.02491952	0.233612958
CCDC12	0.154813	0.06909	2.24076	0.02504165	0.234020448
KRCC1	-0.194777	0.086937	-2.240422	0.02506353	0.234020448
REXO2	0.22851	0.101995	2.240404	0.0250647	0.234020448
ALG1	0.383865	0.171338	2.240395	0.02506526	0.234020448
RFX1	-0.325976	0.14552	-2.240068	0.02508652	0.234020448
IL27RA	-0.30629	0.136746	-2.239841	0.02510128	0.234020448
CHRNA6	-1.897809	0.84731	-2.239805	0.0251036	NA

MBIP	0.323236	0.144314	2.239802	0.02510379	0.234020448
ENSG00000272817	1.199593	0.536354	2.236567	0.02531466	0.235797247
NUTM2B.AS1	-0.304908	0.136492	-2.23389	0.02549029	0.237243209
PDS5B	-0.285149	0.127673	-2.233421	0.02552123	0.237341269
UBB	0.232854	0.104363	2.231197	0.0256681	0.238516463
HOXB4	1.341172	0.601997	2.22787	0.02588917	NA
PHTF2	-0.361024	0.162056	-2.227773	0.02589566	0.240439032
ANKLE1	0.514927	0.231275	2.226469	0.02598276	0.240961145
NANS	0.287671	0.129214	2.226312	0.02599332	0.240961145
DDX10	0.326491	0.146755	2.224734	0.0260991	0.241537348
PSMB4	0.160017	0.07193	2.224607	0.02610762	0.241537348
GBP1	-0.524909	0.235972	-2.224456	0.02611776	0.241537348
NELFA	0.271729	0.122249	2.222743	0.02623315	0.242087287
BUD31	0.138244	0.062196	2.222727	0.02623421	0.242087287
MVP	-0.389928	0.175434	-2.222646	0.02623965	0.242087287
ZBED2	-0.887632	0.399521	-2.221742	0.02630072	0.242458426
PSAP	0.204462	0.092043	2.221364	0.02632631	0.242502224
RPL5	-0.127448	0.057385	-2.220926	0.02635598	0.242583428
TSC22D3	-0.359062	0.161697	-2.220586	0.02637902	0.242603523
AFTPH	-0.195719	0.088156	-2.220157	0.02640814	0.242679525
LINC00342	-0.699684	0.315271	-2.21931	0.02646567	0.242921159
TMEM106A	-0.348656	0.157112	-2.219155	0.02647619	0.242921159
TSEN2	0.374558	0.16882	2.218691	0.02650772	0.243018786
ZFP36L2	-0.431897	0.194718	-2.218057	0.02655091	0.24322308
SECISBP2L	-0.274209	0.123732	-2.216147	0.02668141	0.244226183
CNTRL	-0.20794	0.093853	-2.215597	0.02671915	0.244379346
SPCS1	0.160844	0.072639	2.214286	0.02680914	0.24500988
KANSL1	-0.269785	0.121856	-2.21396	0.0268315	0.245021741
CNR2	-0.290068	0.131045	-2.2135	0.02686317	0.245104953
C1orf43	0.159648	0.072134	2.213216	0.02688275	0.245104953
ENSG00000224046	-1.137362	0.513974	-2.212879	0.02690601	0.245124899
IKZF2	-0.797241	0.360466	-2.211693	0.02698787	0.245569216
MAPKBP1	-0.689233	0.311673	-2.211399	0.02700819	0.245569216
EMB	-0.238708	0.107963	-2.211013	0.02703494	0.245569216
SLC27A1	-1.063551	0.481038	-2.210951	0.02703921	0.245569216
CDC42	-0.147431	0.066706	-2.210161	0.02709401	0.245874944
LINC00869	-0.273548	0.123802	-2.209554	0.02713613	0.245938035
AUH	0.397357	0.179851	2.209364	0.02714937	0.245938035
VBP1	0.169854	0.076897	2.208854	0.02718478	0.245938035
PIK3CB	-0.571714	0.258832	-2.208828	0.02718664	0.245938035
PPP1R12A	-0.227428	0.102976	-2.20854	0.02720666	0.245938035
PTMA	0.134754	0.061029	2.208033	0.02724199	0.246013974
IMP3	0.180984	0.081974	2.207812	0.02725735	0.246013974
PCID2	0.25363	0.114934	2.206742	0.02733209	0.246497307
ITGB2	0.528122	0.239454	2.205524	0.02741733	0.247074565
GART	0.233395	0.105925	2.203394	0.02756699	0.248230956
LINC02576	-1.205051	0.547029	-2.202902	0.02760166	0.248350861

EVI5	-0.35157	0.159631	-2.202391	0.02763767	0.248482742
TCP11L2	-0.488931	0.222034	-2.202057	0.02766129	0.248503028
MYBBP1A	0.342622	0.155643	2.201335	0.02771234	0.248717335
ZNF608	-0.532241	0.241805	-2.201115	0.0277279	0.248717335
WDR46	0.309205	0.140532	2.200241	0.0277898	0.249080572
NUDCD2	0.161948	0.073661	2.19856	0.02790924	0.249958489
POC5	-0.493838	0.224774	-2.19704	0.02801756	0.25073557
HNRNPF	0.25299	0.115173	2.196608	0.02804845	0.250819127
CSDE1	-0.107733	0.049059	-2.19598	0.02809341	0.250877601
HDAC2	0.191717	0.087308	2.195862	0.02810186	0.250877601
WDTC1	0.299072	0.136213	2.195613	0.02811968	0.250877601
ZC3H8	0.269344	0.122697	2.195189	0.02815003	0.250955933
BSDC1	-0.260045	0.118549	-2.193566	0.02826665	0.251802604
ANP32A	0.135105	0.061643	2.191735	0.02839868	0.252765344
ZNF117	0.587676	0.268166	2.191465	0.02841818	0.252765344
ATP5MF	0.157284	0.071809	2.190319	0.02850112	0.253309432
CAMKK2	0.412991	0.188648	2.189214	0.02858126	0.253827757
MINDY1	-0.807206	0.368896	-2.188165	0.0286576	0.254139259
C20orf27	0.213952	0.097782	2.188039	0.02866679	0.254139259
SLC12A2	-0.299952	0.1371	-2.187832	0.02868187	0.254139259
IFT22	-0.308099	0.140844	-2.187514	0.02870504	0.254151024
CD2AP	0.290992	0.133077	2.186636	0.02876909	0.254333799
MAPK7	-0.574061	0.262565	-2.186356	0.02878956	0.254333799
RPS18	-0.121288	0.055478	-2.18622	0.02879954	0.254333799
TRIB2	-0.745784	0.341158	-2.186034	0.02881313	0.254333799
CMC1	0.247242	0.113125	2.185561	0.02884777	0.25442738
RABGGTA	-0.288538	0.132055	-2.184992	0.02888942	0.25442738
LSR	-0.524627	0.240133	-2.184732	0.0289085	0.25442738
ABI1	-0.184905	0.084637	-2.184694	0.02891131	0.25442738
MARS	0.24425	0.111816	2.184397	0.02893308	0.25442738
ENSG00000230709	-0.761461	0.348838	-2.182846	0.02904719	0.255237907
ANAPC4	-0.393495	0.180298	-2.182469	0.02907496	0.255289147
USP7	0.265038	0.121507	2.181252	0.02916476	0.255884438
CDC16	-0.278111	0.127524	-2.180856	0.02919408	0.255948695
HIPK3	-0.336827	0.154507	-2.180014	0.0292564	0.256226575
ATF7IP2	0.28269	0.129684	2.179833	0.02926982	0.256226575
NAXD	0.380284	0.174547	2.178694	0.02935438	0.256773537
MED27	0.759661	0.348851	2.177612	0.02943494	0.257071444
SUN1	-0.227757	0.104598	-2.177453	0.02944676	0.257071444
HLA.DMB	0.254266	0.116784	2.177231	0.02946336	0.257071444
B3GAT3	0.210121	0.096526	2.176843	0.02949231	0.257071444
ATP2B1.AS1	-0.365211	0.167778	-2.176754	0.02949892	0.257071444
ENSG00000226571	-0.675431	0.310425	-2.175828	0.02956812	0.257317208
TXN	0.249086	0.114481	2.175785	0.02957135	0.257317208
PSMB2	0.23429	0.107716	2.175065	0.02962526	0.257593642
AKNA	-0.260447	0.119768	-2.174595	0.02966049	0.257703328
FILIP1L	-0.903152	0.415375	-2.174306	0.02968218	0.257703328

LINC01869	-0.758293	0.348804	-2.173979	0.02970674	0.257724284
PRXL2C	-0.395063	0.181785	-2.17324	0.02976229	0.25801374
RPS15A	-0.117971	0.054299	-2.172609	0.02980978	0.25809232
CYB5R3	-0.215388	0.099142	-2.17253	0.02981572	0.25809232
FBXL6	0.688825	0.317171	2.171779	0.02987237	0.258246164
FAM107B	0.199096	0.09168	2.171639	0.02988293	0.258246164
ECE1	0.347453	0.160013	2.171411	0.02990008	0.258246164
BCYRN1	1.161531	0.535286	2.169926	0.03001244	NA
SLC25A43	-0.39161	0.180492	-2.169688	0.03003046	0.259179831
CXXC5	-0.253755	0.117019	-2.168484	0.0301219	0.259593086
DERL2	0.198881	0.091715	2.16847	0.03012297	0.259593086
TMCO1	0.155883	0.071941	2.16683	0.03024783	0.260476086
FYN	-0.42773	0.197497	-2.16575	0.03033029	0.260925989
ENSG00000233184	-0.524109	0.24202	-2.165559	0.03034493	0.260925989
ENSG00000258572	-0.876493	0.404909	-2.164669	0.03041305	0.261043504
TNFAIP8L2	-0.411845	0.190261	-2.164633	0.0304158	0.261043504
MPI	0.35351	0.163322	2.164501	0.03042591	0.261043504
MRPL54	0.188382	0.087099	2.162833	0.03055399	0.261949272
EIF2S3	-0.214293	0.099096	-2.162484	0.03058091	0.261986939
C12orf75	-0.533373	0.246824	-2.160948	0.03069937	0.262808321
NARF	-0.199436	0.09234	-2.159791	0.03078887	0.263380683
SGCB	-0.675739	0.313248	-2.157206	0.03098962	0.264582373
ATP11A	-0.526429	0.244047	-2.157085	0.03099903	0.264582373
EMP3	-0.240663	0.111572	-2.157026	0.03100364	0.264582373
MRPL36	0.19653	0.091133	2.156521	0.03104301	0.264582373
PHGDH	0.685868	0.318044	2.15652	0.03104306	0.264582373
POR	0.304364	0.141186	2.155769	0.03110169	0.264725603
FBXO10	0.513092	0.238014	2.155722	0.03110537	0.264725603
KIFC2	-0.790816	0.366964	-2.15502	0.03116024	0.264961808
CAT	-0.354499	0.164535	-2.154554	0.03119676	0.264961808
TRNT1	0.251344	0.11666	2.154494	0.03120145	0.264961808
IGBP1	-0.168505	0.078235	-2.153837	0.03125295	0.265061664
FCRL4	0.630775	0.292884	2.15367	0.03126604	0.265061664
TRADD	-0.267085	0.124037	-2.153265	0.03129782	0.265061664
CERS6	0.447534	0.207911	2.152526	0.03135595	0.265061664
TCEAL8	-0.244487	0.113582	-2.152518	0.03135657	0.265061664
PUM3	0.207416	0.096361	2.152501	0.03135794	0.265061664
GNAI2	-0.228215	0.106032	-2.152313	0.03137269	0.265061664
DAPL1	0.441408	0.20516	2.151535	0.03143403	0.26538714
PPA1	0.240366	0.11181	2.149764	0.0315739	0.266374711
NUSAP1	-0.657608	0.306015	-2.148945	0.03163879	0.266728728
RB1CC1	-0.233639	0.108777	-2.147871	0.03172402	0.267110883
AHSA1	0.229545	0.106875	2.147795	0.03173004	0.267110883
DESI1	0.359227	0.167411	2.145778	0.03189072	0.268269395
ERV3.1	-0.176297	0.082201	-2.144697	0.03197709	0.268717744
EML6	-0.746405	0.348071	-2.144402	0.03200072	0.268717744
SLC6A16	-0.612644	0.285715	-2.144244	0.03201331	0.268717744

CENPM	0.356158	0.166217	2.142734	0.0321345	0.269467595
PEX16	-0.198417	0.092608	-2.142554	0.03214896	0.269467595
TRIM23	-0.528603	0.246788	-2.141933	0.0321989	0.269691824
ISOC2	0.265437	0.12405	2.139762	0.03237404	0.270963698
U2SURP	0.141715	0.066269	2.138485	0.03247737	0.271473099
CDKL5	-1.284388	0.600621	-2.138434	0.03248157	0.271473099
ZNF490	-1.590476	0.743841	-2.138192	0.03250113	NA
RPL15	-0.113445	0.053086	-2.136998	0.03259818	0.272108322
MT.ND4L	-0.401476	0.187894	-2.136719	0.03262084	0.272108322
SMCHD1	-0.184773	0.086485	-2.136481	0.03264026	0.272108322
GPM6B	-0.340289	0.159315	-2.135952	0.03268333	0.272108322
TMEM107	-0.355444	0.166416	-2.135872	0.03268983	0.272108322
ATP5MD	0.163894	0.076738	2.135753	0.03269954	0.272108322
RELCH	-0.558069	0.261331	-2.135486	0.0327213	0.272108322
RPS25	-0.104715	0.049045	-2.135071	0.03275525	0.272196141
KARS	0.132082	0.061894	2.133998	0.03284292	0.272729846
PTPRC	-0.181024	0.084854	-2.133369	0.03289448	0.272963179
ITSN2	-0.174813	0.081954	-2.133055	0.03292022	0.272982079
ELOC	0.198345	0.093004	2.132659	0.03295272	0.273000383
ENSG00000225342	-0.654405	0.306878	-2.132456	0.03296936	0.273000383
MRPS15	0.174267	0.081734	2.132136	0.03299565	0.273023708
POLD2	0.21736	0.102024	2.130487	0.03313145	0.273862516
RAB27A	0.317947	0.149248	2.130333	0.0331441	0.273862516
ENSG00000245466	-1.78772	0.839437	-2.129665	0.03319925	NA
MGAT1	0.238846	0.112164	2.129445	0.0332175	0.274274232
NUS1	0.203884	0.095815	2.127883	0.03334681	0.275146687
MAP2K1	-0.257083	0.120877	-2.126806	0.03343617	0.275688416
ZNF596	0.841674	0.3959	2.125979	0.03350497	0.275922778
UBA7	-0.358376	0.168577	-2.125895	0.03351202	0.275922778
MRPS33	0.247464	0.116497	2.12421	0.03365262	0.276884383
ELMOD2	-0.47408	0.223247	-2.12357	0.03370613	0.277128689
MRPL37	0.175167	0.082508	2.123043	0.03375027	0.277135412
SEC61G	0.186906	0.088039	2.122991	0.03375459	0.277135412
DCLRE1C	0.18828	0.088713	2.122355	0.03380791	0.277278187
RGS1	-0.514376	0.242377	-2.122216	0.03381965	0.277278187
RHOC	-0.46452	0.21897	-2.121388	0.03388916	0.277652468
PSTPIP1	-0.548097	0.258456	-2.120662	0.03395024	0.277942994
RNASEK	-0.345377	0.162904	-2.120125	0.0339955	0.277942994
SNRNP48	-0.322679	0.152199	-2.120116	0.03399629	0.277942994
CD164	0.143144	0.067578	2.118192	0.03415877	0.279075264
SLC20A1	0.282293	0.133296	2.117794	0.03419255	0.279155173
RETREG1	-0.831608	0.39278	-2.117235	0.03423988	0.279164484
DLGAP1.AS1	-0.270584	0.127802	-2.117214	0.03424168	0.279164484
ENSG00000227486	-0.355602	0.168024	-2.116374	0.034313	0.279550015
ZNF677	-0.290054	0.137087	-2.115843	0.03435818	0.279722266
TEPP	-1.300434	0.614754	-2.115373	0.03439821	0.279852282
ENSG00000265206	-0.248588	0.117568	-2.114428	0.03447872	0.280311246

EXOSC7	0.258212	0.122159	2.113738	0.03453761	0.280593993
SIGLEC14	0.790259	0.373947	2.113295	0.03457553	0.280706001
GPATCH1	-0.409704	0.193944	-2.112489	0.0346445	0.280986371
ZNF547	-1.015811	0.480937	-2.112149	0.0346737	0.280986371
SLC35A4	0.219282	0.103824	2.112046	0.03468252	0.280986371
KLRD1	1.427827	0.676049	2.112018	0.03468487	NA
CEP70	0.709156	0.335854	2.111501	0.03472927	0.2811693
FEM1A	0.248211	0.117612	2.110427	0.03482157	0.281720577
SNX12	0.229204	0.108635	2.109859	0.03487049	0.281920242
TNPO3	0.266106	0.126156	2.109352	0.03491421	0.282077726
ENSG00000187186	-0.831156	0.394153	-2.108711	0.03496952	0.282328524
SLC2A3	-0.449679	0.213413	-2.107085	0.03511024	0.283268054
CYSLTR1	-0.477315	0.226735	-2.10517	0.0352765	0.284287142
DLGAP4	-0.239325	0.11369	-2.105068	0.03528543	0.284287142
MAATS1	-1.78493	0.848006	-2.104857	0.03530378	NA
FAM227A	1.636851	0.77766	2.104841	0.03530512	NA
FLII	-0.252362	0.119951	-2.103883	0.03538867	0.284747111
HEATR6	-0.444721	0.211387	-2.103817	0.03539443	0.284747111
MRPS34	0.165696	0.078769	2.10357	0.03541594	0.284747111
PIK3CG	0.433686	0.206287	2.102347	0.0355229	0.28540985
ZNF736	-0.459192	0.218452	-2.102025	0.03555104	0.285438816
EIF1	0.158352	0.07535	2.101542	0.03559345	0.285582208
CYTH1	0.186672	0.088911	2.099549	0.03576855	0.286789327
EIF3J.DT	-0.245593	0.117009	-2.098925	0.03582349	0.287032012
CYC1	0.199775	0.095194	2.098616	0.03585074	0.287052637
LIMS2	-1.034836	0.493178	-2.098303	0.0358784	0.287076517
ENSG00000253636	1.762343	0.840008	2.098008	0.03590444	NA
ENSG00000235501	-1.536639	0.732476	-2.09787	0.03591661	NA
ESR2	-0.484351	0.230907	-2.097602	0.03594028	0.287372596
OAS2	0.346788	0.165348	2.097325	0.0359648	0.287372596
ADRM1	0.20277	0.096776	2.095261	0.03614779	0.288391581
CCS	-0.242608	0.115797	-2.095116	0.03616071	0.288391581
NETO2	0.641932	0.306407	2.095029	0.03616845	0.288391581
MRPS10	0.184697	0.088188	2.094344	0.03622931	0.288391581
MTAP	0.298639	0.142605	2.094168	0.03624504	0.288391581
TTYH3	0.477712	0.228118	2.094146	0.03624698	0.288391581
ENSG00000267519	-0.329438	0.15733	-2.093934	0.03626585	0.288391581
CCDC146	-1.327642	0.634125	-2.093658	0.03629047	NA
TIMM21	0.302323	0.144406	2.093565	0.03629871	0.288455745
FCHSD2	-0.315206	0.150582	-2.093253	0.03632662	0.288480468
PSMB6	0.164245	0.078537	2.09132	0.03649939	0.289654812
CD52	-0.244582	0.11703	-2.089911	0.03662577	0.290073152
IGLC2	-0.300523	0.143816	-2.08964	0.03665019	0.290073152
TPRG1L	0.376761	0.180299	2.089639	0.03665023	0.290073152
MAP3K20	-0.436602	0.208938	-2.089621	0.03665184	0.290073152
SDCBP	0.304631	0.145831	2.088934	0.03671364	0.290313244
C8orf37	-0.833882	0.399229	-2.088729	0.03673209	0.290313244

SMYD2	-0.311964	0.149446	-2.087468	0.03684586	0.29101475
ABCF1	0.177861	0.085232	2.086775	0.03690849	0.291311619
CPEB3	0.783095	0.375423	2.0859	0.03698769	0.29156306
POLR1C	0.230076	0.110302	2.085869	0.03699047	0.29156306
ENSG00000257275	-0.769386	0.369032	-2.084877	0.03708049	0.291989052
VPS9D1.AS1	0.51586	0.247453	2.084678	0.03709856	0.291989052
NDUFC2	0.208123	0.099846	2.084444	0.03711981	0.291989052
RPS4X	-0.217917	0.104567	-2.084	0.03716019	0.291996676
ZCCHC18	-0.655902	0.314782	-2.083669	0.03719027	0.291996676
RRP8	0.244277	0.117237	2.083605	0.03719607	0.291996676
ADD3	-0.233034	0.111863	-2.083203	0.0372327	0.292033373
RREB1	-0.299261	0.143668	-2.083003	0.03725095	0.292033373
ITFG1	0.254654	0.122308	2.082069	0.03733613	0.292488623
FAM136A	0.174119	0.083642	2.081722	0.03736788	0.292488623
KLF13	0.242432	0.116468	2.081541	0.03738444	0.292488623
DRAM2	-0.254997	0.122585	-2.080163	0.03751055	0.29327803
XPNPEP1	-0.334403	0.160822	-2.079338	0.0375863	0.293672906
RHBDD3	0.397229	0.191095	2.078703	0.03764464	0.293739889
BTBD19	0.728173	0.350303	2.078695	0.03764537	0.293739889
STAG3	-0.402985	0.193912	-2.078181	0.03769272	0.293912256
FOXO1	-0.317185	0.152656	-2.077771	0.03773042	0.294009193
PSEN1	-0.324425	0.156365	-2.074796	0.0380054	0.295953724
PRICKLE1	0.597339	0.288048	2.073746	0.03810287	0.296514257
CNFN	-0.518492	0.250135	-2.072845	0.03818672	0.296968079
PSMC2	0.231299	0.111604	2.072499	0.03821892	0.297019982
AMN1	-0.378217	0.182644	-2.070792	0.03837824	0.297911624
ST6GALNAC4	-0.216503	0.104555	-2.070721	0.03838487	0.297911624
RILPL2	0.324118	0.156586	2.069906	0.03846113	0.298155314
CDK5RAP3	-0.225902	0.109143	-2.069776	0.03847337	0.298155314
MFSD2A	0.739553	0.357347	2.069564	0.03849315	0.298155314
AAK1	0.574539	0.277759	2.068478	0.0385951	0.298746118
ATN1	-0.347129	0.167858	-2.067992	0.03864081	0.298901033
PLEKHF2	-0.230776	0.11161	-2.067701	0.03866818	0.298913993
NPLOC4	0.286458	0.138567	2.067282	0.03870756	0.299019776
ENSG00000255240	-0.805549	0.389852	-2.066293	0.03880078	0.299540963
AP2A1	-0.259881	0.125809	-2.065675	0.03885922	0.299793175
HDAC10	-0.235521	0.114147	-2.06331	0.03908318	0.301321203
SERTAD2	-0.302841	0.146853	-2.06221	0.03918772	0.301890097
HCCS	0.234386	0.11367	2.061988	0.03920887	0.301890097
STKLD1	-2.523992	1.224232	-2.061695	0.0392368	NA
C14orf28	-0.458231	0.222379	-2.060592	0.03934202	0.302714975
CDCA8	-0.997516	0.484189	-2.060177	0.0393816	0.302760037
CRIPT	0.15995	0.077646	2.059986	0.03939992	0.302760037
AKT1S1	0.291547	0.141553	2.059634	0.03943359	0.302818724
KHDC4	-0.187486	0.091105	-2.057925	0.03959732	0.303603185
LPP.AS2	-0.7828	0.380398	-2.057846	0.03960493	0.303603185
ENSG00000228463	0.367942	0.178831	2.05748	0.03964005	0.303603185

NCBP2	0.148302	0.07208	2.05745	0.03964296	0.303603185
FAM120AOS	-0.234529	0.114004	-2.057208	0.03966622	0.303603185
TINCR	-1.549777	0.753878	-2.055741	0.03980746	NA
DUS1L	0.26395	0.128404	2.055619	0.03981925	0.304574029
QTRT1	0.195839	0.095287	2.055257	0.03985416	0.304640802
ANAPC5	0.137019	0.066703	2.054181	0.0399582	0.305235525
DDX18	0.117058	0.057028	2.052644	0.04010712	0.306172042
CXorf21	-0.302714	0.147497	-2.052333	0.04013736	0.30620197
GVQW3	-0.735838	0.358634	-2.051779	0.04019115	0.306213996
RPUSD2	0.381338	0.185858	2.051775	0.04019157	0.306213996
ASAH1	0.204231	0.09965	2.049498	0.04041346	0.307702995
CTSC	0.374477	0.182798	2.048584	0.04050282	0.308132033
HIST3H2A	0.739747	0.361137	2.04838	0.04052278	0.308132033
RABEP2	-0.243528	0.118944	-2.047421	0.04061679	0.308645157
ZNF688	-0.305086	0.149051	-2.04686	0.0406718	0.308798946
PRDM8	-0.654865	0.320012	-2.046375	0.04071947	0.308798946
NBPF19	-0.48839	0.23868	-2.046209	0.04073579	0.308798946
STX17	-0.282446	0.138069	-2.045684	0.04078751	0.308798946
ICAM2	0.407455	0.199196	2.045501	0.04080546	0.308798946
EXD3	-0.524482	0.256408	-2.0455	0.04080562	0.308798946
RFX5	0.217665	0.106421	2.045325	0.04082283	0.308798946
RGL1	1.035414	0.506389	2.044702	0.04088425	0.309062635
DEGS2	-1.25745	0.6152	-2.043969	0.04095663	0.309408698
ATP6V1D	0.233916	0.114462	2.043612	0.04099194	0.30947426
SIGMAR1	0.286911	0.140428	2.043123	0.04104028	0.30947426
EARS2	0.442844	0.216754	2.043074	0.04104511	0.30947426
STRAP	0.150408	0.073636	2.042581	0.04109397	0.30964202
CSNK1E	-0.307375	0.15054	-2.041811	0.04117031	0.309936876
RPS12	-0.122589	0.060044	-2.041649	0.04118639	0.309936876
MLXIPL	1.741798	0.853216	2.041451	0.04120599	NA
SMG9	-0.31441	0.154054	-2.04091	0.0412598	0.310042726
DOK1	-0.343103	0.168117	-2.040861	0.04126467	0.310042726
ENSG00000272182	0.803545	0.393759	2.040703	0.0412804	0.310042726
FGL2	-1.384502	0.678645	-2.040099	0.04134042	NA
TMF1	-0.221687	0.108688	-2.039655	0.04138475	0.310625914
SCAI	-0.31883	0.156345	-2.03928	0.04142209	0.310705727
NT5C3A	-0.222859	0.109381	-2.037468	0.04160321	0.31186327
DAAM1	-0.402914	0.197834	-2.036621	0.04168807	0.312282963
MMACHC	0.689199	0.338444	2.036373	0.04171289	0.312282963
HMGN2	-0.113368	0.055683	-2.03595	0.04175538	0.312335172
KIF5C	-1.13762	0.558816	-2.035769	0.04177355	0.312335172
PSMB1	0.161986	0.07959	2.035272	0.04182351	0.312507843
CDK2AP1	0.670507	0.329497	2.034941	0.04185686	0.312556281
TMED3	0.208739	0.10261	2.034297	0.04192163	0.312747634
MANBA	-0.255948	0.125825	-2.034152	0.04193625	0.312747634
TIMM44	0.187279	0.092111	2.03318	0.04203436	0.313278499
DHX33	0.28938	0.142366	2.032651	0.04208784	0.313296177

ZNF263	0.27875	0.137138	2.032623	0.04209059	0.313296177
KCNQ5	0.543538	0.267586	2.031265	0.04222811	0.313972279
JAK2	-0.561369	0.276374	-2.031193	0.0422354	0.313972279
EPS8L2	-0.519076	0.25564	-2.030494	0.04230633	0.314298713
FAM135A	-0.424096	0.208963	-2.029521	0.04240525	0.314571959
MYO1G	-0.326637	0.16097	-2.02918	0.04243992	0.314571959
RTCB	0.233379	0.115016	2.029098	0.04244833	0.314571959
ATM	-0.165363	0.081507	-2.028826	0.04247601	0.314571959
ENSG00000273669	-1.154871	0.56932	-2.028512	0.04250806	0.314571959
MRFAP1L1	0.246039	0.121303	2.028311	0.04252856	0.314571959
HMBS	0.234123	0.11543	2.028273	0.04253238	0.314571959
HK2	0.597318	0.294558	2.027843	0.0425763	0.314610495
PPM1L	0.90217	0.444925	2.027692	0.04259167	0.314610495
OPTN	-0.437817	0.216001	-2.026921	0.04267054	0.314993066
ACOT7	0.489751	0.241721	2.0261	0.04275454	0.315136813
ANXA2R	-0.367086	0.181189	-2.025983	0.04276654	0.315136813
COQ7	0.303943	0.150026	2.025937	0.04277128	0.315136813
QPCT	0.763099	0.376757	2.02544	0.04282219	0.315312282
DDT	0.147782	0.07302	2.02386	0.04298459	0.316307877
LINC01138	-0.43373	0.214387	-2.023119	0.0430609	0.316669092
TCF4	-0.302304	0.149463	-2.022596	0.04311486	0.316766403
KIF2A	-0.230317	0.113879	-2.022463	0.04312859	0.316766403
MRPS28	0.274733	0.135883	2.021841	0.04319283	0.316860542
PIAS2	-0.241256	0.119327	-2.021811	0.04319588	0.316860542
RPP25	0.419647	0.207758	2.019878	0.04339601	0.318128015
C4orf36	-0.695668	0.344948	-2.016737	0.043723	0.32032329
IRAK1	0.200749	0.099612	2.015319	0.04387128	0.321207378
AP4B1	-0.440645	0.218711	-2.014743	0.04393157	0.321446447
NUP210	0.220127	0.109305	2.013884	0.04402168	0.321746574
CCDC144A	0.844036	0.419121	2.013825	0.0440279	0.321746574
ANKRD54	0.267023	0.132711	2.012063	0.04421329	0.32223855
ZER1	0.5831	0.289821	2.011931	0.04422717	0.32223855
N4BP3	-0.330362	0.164218	-2.011727	0.04424877	0.32223855
PSMC5	0.143784	0.07148	2.011521	0.04427044	0.32223855
MDK	0.822298	0.408822	2.011386	0.04428468	0.32223855
CCT6A	0.15886	0.078986	2.011252	0.04429882	0.32223855
KIAA1841	-0.742391	0.369137	-2.011155	0.04430904	0.32223855
RPSA	-0.135636	0.067449	-2.010951	0.0443306	0.32223855
ENSG00000197180	-0.428138	0.212917	-2.01082	0.0443445	0.32223855
UQCRQ	0.182743	0.090918	2.009966	0.04443481	0.322693207
LTB	-0.337357	0.167879	-2.00952	0.04448199	0.322834366
CEPT1	-0.20778	0.103416	-2.009168	0.04451936	0.322904136
RPL7A	-0.125297	0.062395	-2.008126	0.04462995	0.323504559
AK1	0.496099	0.247087	2.007788	0.04466583	0.32356302
DMTF1	-0.240869	0.12003	-2.006751	0.04477621	0.32416082
PPARA	-0.439956	0.219466	-2.004669	0.04499847	0.325567255
RGS19	-0.209592	0.104571	-2.004311	0.0450368	0.325642118

PCP2	-1.099172	0.548702	-2.003223	0.0451534	0.326282385
NRSN2.AS1	-0.748102	0.37354	-2.002734	0.0452058	0.326458288
NSUN6	-0.30726	0.153535	-2.001233	0.04536725	0.327257709
ERMARD	0.481285	0.240502	2.001167	0.0453744	0.327257709
ENSG00000260979	0.735893	0.367777	2.000921	0.04540089	0.327257709
DCBLD2	-0.927033	0.463393	-2.000532	0.04544288	0.327357567
ENSG00000272843	-1.711458	0.855544	-2.000432	0.04545364	NA
FOXRED2	0.676423	0.338183	2.000171	0.0454818	0.327435177
VSIG10L	1.366172	0.68319	1.999697	0.04553295	NA
TMEM208	0.149648	0.074848	1.99936	0.04556937	0.327862701
PSMA3	0.186423	0.09328	1.998536	0.04565863	0.328301941
KIF20B	-0.235018	0.117645	-1.997683	0.04575107	0.328499232
JMJD8	0.293168	0.146763	1.997567	0.04576361	0.328499232
PFDN4	0.170172	0.085193	1.997501	0.04577078	0.328499232
UBE3D	0.336388	0.168464	1.996798	0.04584714	0.32884441
STRBP	-0.171188	0.085758	-1.996168	0.04591559	0.328927788
CNR1	0.724619	0.363045	1.99595	0.04593933	0.328927788
YARS	0.210092	0.105261	1.995911	0.04594358	0.328927788
SF3A3	0.188622	0.09453	1.995358	0.04600382	0.329156454
MSTO1	0.360871	0.18091	1.994759	0.0460692	0.329421666
UPF3B	-0.182692	0.091616	-1.994115	0.0461395	0.329721733
HSPH1	0.220146	0.110502	1.992233	0.04634552	0.330774724
KLF3	0.339185	0.170279	1.991929	0.04637884	0.330774724
TFB2M	0.320477	0.160896	1.991831	0.04638966	0.330774724
CISD2	0.172477	0.086597	1.991731	0.04640058	0.330774724
RPGR	-0.450263	0.226102	-1.991412	0.04643566	0.330822113
NFE2L1	0.251859	0.12656	1.990029	0.04658774	0.331702419
TOB2	-0.199162	0.100122	-1.989195	0.04667971	0.332153972
APPBP2	-0.31515	0.158481	-1.988561	0.04674968	0.332257442
AGPAT2	-0.238397	0.119904	-1.988231	0.04678614	0.332257442
PELO	0.360678	0.181429	1.987984	0.04681343	0.332257442
SASH3	-0.241936	0.121702	-1.987929	0.04681955	0.332257442
CARS2	0.320978	0.161542	1.986968	0.04692594	0.332257442
UNC45A	-0.236137	0.118853	-1.98679	0.04694568	0.332257442
TRAP1	0.27768	0.139765	1.986763	0.04694871	0.332257442
PHF11	-0.20197	0.101662	-1.986688	0.04695702	0.332257442
INKA1	-0.510893	0.2572	-1.986363	0.04699304	0.332257442
DTD2	0.303642	0.15287	1.98628	0.04700223	0.332257442
PGK1	0.260144	0.130974	1.986224	0.0470084	0.332257442
RAB11FIP1	-0.23847	0.120112	-1.985399	0.04710011	0.332703539
METRN	0.492511	0.24821	1.984253	0.0472276	0.333165886
BRMS1	0.139578	0.070349	1.984088	0.04724602	0.333165886
POLR2K	0.167935	0.084643	1.984039	0.04725148	0.333165886
ENSG00000225489	0.613169	0.309171	1.983268	0.04733749	0.333502345
PDK3	-0.421913	0.212755	-1.983098	0.04735653	0.333502345
CCDC57	-0.23867	0.120435	-1.981734	0.04750901	0.334045277
SYNC	-0.321062	0.162015	-1.981676	0.04751548	0.334045277

RBM28	0.254226	0.128291	1.981638	0.04751976	0.334045277
HERC1	-0.348193	0.175878	-1.979741	0.04773269	0.335339465
TBXAS1	0.859633	0.434726	1.977413	0.04799497	0.33691414
TSSC4	0.185185	0.093658	1.977238	0.04801475	0.33691414
ENSG00000274536	-0.46241	0.233969	-1.976374	0.04811248	0.337287041
ACSM3	-0.499009	0.252573	-1.975705	0.04818823	0.337287041
NMRK1	-0.731328	0.370196	-1.975517	0.04820955	0.337287041
BRWD3	-0.460698	0.233215	-1.975423	0.04822013	0.337287041
PDPK1	-0.24661	0.12484	-1.975416	0.04822101	0.337287041
SPN	0.490584	0.248391	1.975043	0.04826324	0.337287041
PHF20L1	-0.222503	0.112674	-1.974751	0.04829636	0.337287041
FAH	0.586778	0.297157	1.974639	0.04830908	0.337287041
BRCA2	-0.40212	0.203668	-1.974386	0.04833789	0.337287041
PLXNB2	0.762668	0.386316	1.974211	0.04835781	0.337287041
OGA	-0.148608	0.075296	-1.97364	0.04842271	0.337537306
USP3	-0.185763	0.09415	-1.973065	0.04848812	0.337537649
ZNF431	-0.202435	0.102599	-1.973062	0.04848854	0.337537649
CRLF3	-0.199035	0.100939	-1.971834	0.04862856	0.337537649
CLK4	-0.301907	0.15314	-1.971447	0.04867282	0.337537649
BEND5	-0.434497	0.220414	-1.971279	0.04869195	0.337537649
PLEKHG2	-0.714852	0.362665	-1.97111	0.04871133	0.337537649
CRYM.AS1	-1.3473	0.683535	-1.971076	0.04871513	0.337537649
LAPTM4B	0.713477	0.361978	1.97105	0.04871817	0.337537649
NUDCD3	-0.23053	0.116963	-1.970963	0.04872811	0.337537649
ZNF322	-0.322267	0.163529	-1.970706	0.04875749	0.337537649
INTS6	-0.181002	0.09185	-1.970613	0.04876814	0.337537649
LINC01572	0.693094	0.351741	1.970466	0.04878504	0.337537649
FAM118B	0.419666	0.212992	1.970336	0.04879993	0.337537649
MBD4	-0.172939	0.087809	-1.969498	0.0488959	0.338000505
ENSG00000274184	-0.901064	0.457606	-1.969082	0.04894368	0.338129929
SEMA4A	0.887266	0.450734	1.968492	0.04901141	0.338185077
ENSG00000160602	-0.248527	0.126254	-1.96847	0.04901395	0.338185077
ERP29	-0.118496	0.060204	-1.968254	0.04903887	0.338185077
TEP1	-0.476538	0.242145	-1.967989	0.04906925	0.338194099
TTC39C	-0.491645	0.250037	-1.96629	0.04926507	0.339048645
LTA	-0.370686	0.188526	-1.966228	0.04927228	0.339048645
VPS13C	-0.201181	0.102323	-1.966131	0.0492835	0.339048645
CCND3	0.159973	0.081374	1.965903	0.04930981	0.339048645
NDUFV1	0.170792	0.0869	1.96539	0.04936914	0.339256104
DOT1L	0.30988	0.157709	1.964884	0.04942769	0.339457948
